# Transcript Length Mediates Developmental Timing of Gene Expression Across *Drosophila*

**DOI:** 10.1093/molbev/msu226

**Published:** 2014-07-28

**Authors:** Carlo G. Artieri, Hunter B. Fraser

**Affiliations:** ^1^Department of Biology, Stanford University

**Keywords:** intron delay, syncytium, embryonic development, transcript length, *Drosophila*, gene structure evolution, genome evolution

## Abstract

The time required to transcribe genes with long primary transcripts may limit their ability to be expressed in cells with short mitotic cycles, a phenomenon termed intron delay. As such short cycles are a hallmark of the earliest stages of insect development, we tested the impact of intron delay on the *Drosophila* developmental transcriptome. We find that long zygotically expressed genes show substantial delay in expression relative to their shorter counterparts, which is not observed for maternally deposited transcripts. Patterns of RNA-seq coverage along transcripts show that this delay is consistent with their inability to completely transcribe long transcripts, but not with transcriptional initiation-based regulatory control. We further show that highly expressed zygotic genes maintain compact transcribed regions across the *Drosophila* phylogeny, allowing conservation of embryonic expression patterns. We propose that the physical constraints of intron delay affect patterns of expression and the evolution of gene structure of a substantial portion of the *Drosophila* transcriptome*.*

## Introduction

Although the variability in the lengths of most introns in metazoa appears to be consistent with neutral processes (Lynch 2002, 2005; Lynch and Richardson 2002), the expression of genes with long transcripts is nevertheless likely to impose significant organismal costs (Swinburne and Silver 2008). The time required to transcribe long introns is nontrivial (Gubb 1986): as an extreme example, the largest known gene, human dystrophin, requires approximately 16 h for the transcription of its approximately 2.3 Mb primary transcript. Consequently, rapid production of dystrophin is primarily limited by the time required to produce its mRNA (Tennyson et al. 1995). At the level of the transcriptome, the burden of transcriptional time has manifested itself in the observation that genes with expression patterns that change rapidly in response to stress have low intron densities (Jeffares et al. 2008).

A variety of studies have shown that transcription from all three RNA polymerases ceases once cells leave interphase and enter mitotic division (Gottesfeld and Forbes 1997). Though the precise mechanisms of this repression remain poorly understood, an important component involves lack of access of the polymerase to condensing chromatin. As the mitotic cycle begins, incomplete transcripts are released from the condensing chromosomes and are subsequently degraded by an unidentified nuclear mechanism. These transcripts remain undetectable until the completion of mitosis (Shermoen and O’Farrell 1991). In addition to increasing transcriptional time, introns cannot be cotranscriptionally spliced during mitosis, imposing an additional temporal cost to their presence (Shin and Manley 2002; Guilgur et al. 2014). Accordingly, strong selection is thought to act against the expansion of existing introns (or the introduction of new introns) in genes that must be expressed in cells undergoing frequent mitoses. This is supported by the observation that single-celled eukaryotes with rapid reproductive rates, such as yeasts and *Guillardia*, have very intron-poor genomes despite having descended from more intron-rich ancestors (Mourier and Jeffares 2003; Jeffares et al. 2006).

In multicellular eukaryotes mitotic cycles can be rapid enough to limit the expression of long genes. For example, in *Drosophila melanogaster* reared at 25 °C, the earliest stages of development are characterized by rapid mitotic cycles as short as 8.6 min per division (Foe et al. 1993). It is during this period of development that transcription of the zygotic genome begins—a process known as zygotic genome activation. Most insects achieve these rapid mitotic cycles by avoiding cytokinesis altogether and generating nuclei within a common embryonic cytoplasm known as a syncytial blastoderm (Grbić et al. 1996). In *D. melanogaster*, the zygotic nucleus undergoes 13 synchronous mitotic divisions, the first nine requiring approximately 9 min each, while cycles 10–13 progressively lengthen to a maximum of 17 min per division (Foe et al. 1993). Subsequently, the nuclei undergo an approximately 60 min extended 14th mitotic cycle during which cellularization takes place (Foe and Alberts 1983; Frescas et al. 2006).

Long zygotically expressed transcripts may be prevented from reaching appreciable levels of expression during short mitotic cycles due to the time required for full transcription and splicing. In the case of very long transcripts, transcription may not complete at all, delaying their appearance entirely until cell cycles lengthen. As metazoan transcript length is primarily determined by the length of intronic rather than exonic sequence, this phenomenon has been termed “intron delay” (Gubb 1986). Indeed, one gene with a primary transcript length exceeding 20 kb produced aborted transcripts that were degraded within the nucleus during the syncytial cycles of *Drosophila* embryogenesis (Shermoen and O’Farrell 1991; Rothe et al. 1992), and a lack of introns has been noted among zygotic genes expressed before full zygotic genome activation (De Renzis et al. 2007; Heyn et al. 2014).

Delayed production of functional transcripts by a nontranscription initiation-based mechanism may also play a functional regulatory role during early development (Gubb 1986; Swinburne and Silver 2008). For instance, the early stages of anterior–posterior pattern formation in *Drosophila* involve sequential activation of very short pair-rule genes followed by significantly longer homeodomain box genes (Gubb 1986). In addition, proper oscillation of gene expression in the developing mouse somite segmentation clock requires delayed transcription via long primary transcripts (Takashima et al. 2011). A regulatory mechanism based solely on physical constraint is appealing as it allows for a simple sequential process of activation during early development as cell cycles lengthen, without the need to invoke more complex temporal regulatory networks (Gubb 1998). Furthermore, it could also regulate spatial patterning of gene expression during later periods of embryogenesis, when the embryo is partitioned into discrete mitotic domains, the cells of which may replicate at increased rates via endocycling (Foe 1989; Edgar and Orr-Weaver 2001).

The rapid rate of *Drosophila* development makes it an ideal system in which to look for factors affecting the timing of transcription of important developmental effectors (Chippindale et al. 1997). Here, we show that early developmental intron delay of zygotically expressed genes is a general feature of the fruit fly transcriptome, limiting the expression levels of long transcripts well into embryogenesis. We also extend our observations across the *Drosophila* phylogeny and show that, in contrast to neutral expectations, intron delay may impose significant selective pressure to maintain compact primary transcripts among highly expressed zygotic genes.

## Results

### Long Zygotic Transcripts Show Delayed Activation during *D. melanogaster* Embryogenesis

**Fig. 1.**
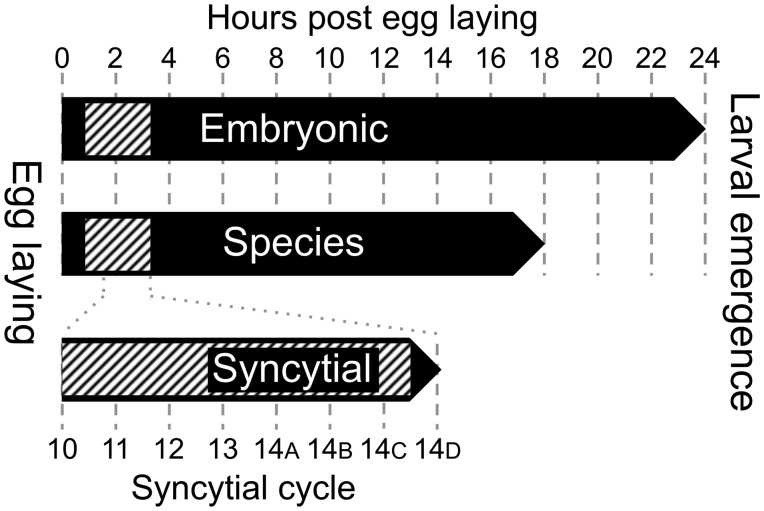
Correspondence between the three expression timecourses analyzed in this study: Embryonic (Gravely et al. 2011), species (Kalinka et al. 2010), and syncytial (Lott et al. 2011). Both the embryonic and species timecourses consist of pools of embryos collected at 2-h intervals, spanning either 24 or 18 h of *Drosophila* embryogenesis. The syncytial timecourse spans syncytial cycles 10–13, followed by four collections during the extended 14th cycle corresponding roughly to 25% increments of cell wall extension to completion of cellularization (indicated by *A–D*). The correspondence between the syncytial timecourse and the other timecourses is indicated by the gray dotted line. The hashed area indicates the period during which the rapid syncytial divisions take place (timing is taken from Campos-Ortega and Hartenstein 1985). The embryonic and syncytial timecourses were generated by RNA-seq, whereas the species timecourse was generated using microarrays.

In order to explore the relationship between transcript length and patterns of expression over the course of embryonic development of *D. melanogaster*, we obtained data from two RNA-seq timecourses generated from poly-A selected RNA: 1) the model organism ENCyclopedia Of DNA Elements (modENCODE) *D. melanogaster* developmental timecourse (Graveley et al. 2011), which consists of 12 sequential 2-h time-synchronized developmental time points spanning the approximately 24 h of fly embryogenesis (hereafter the “embryonic” timecourse), and 2) the data set of Lott et al. (2011), which consists of single embryo samples spanning syncytial cycles 10–13 and four time points spanning the extended 14th mitotic cycle (labeled *A*–*D*) (hereafter the “syncytial” timecourse). As the embryonic timecourse was generated from embryos reared at 25 °C (Graveley et al. 2011), the entire syncytial timecourse, corresponding to Bownes stages 4–5 (Campos-Ortega and Hartenstein 1985), takes place during the first and second time points of the embryonic timecourse (fig. 1). We note that as syncytial timecourse embryos were reared at room temperature (∼22 °C), this may have lengthened cell cycles by a factor of 1.2–1.3-fold (Kuntz and Eisen 2014), but this is not sufficient to have changed its placement within the embryonic timecourse. Expression at the gene level was calculated in Reads Per Kilobase per Million mapped reads (RPKM) (see Materials and Methods) (supplementary table S1, Supplementary Material online, contains all analyzed data).

Complete zygotic genome activation does not begin until approximately 80 min post egg laying (hereafter, all times are indicated as post egg laying), and thus most mRNA present in the embryo before this time is maternally deposited and not subject to intron delay. Many maternal transcripts are eliminated by the beginning of gastrulation (∼180 min at 25 °C or Bownes stage 6), before which time the zygote contains both maternal and zygotic transcripts (Walser and Lipshitz 2011). In order to analyze genes whose initial origin is maternal deposition versus those whose presence is solely derived from zygotic transcription separately, we used the classifications provided by Tadros et al. (2007), resulting in classifications of either “maternal” or “zygotic” for 7,452 genes expressed during the embryonic timecourse and 5,644 genes during the syncytial timecourse. Note that maternal indicates only that these transcripts are initially deposited in the egg; as embryogenesis progresses, the zygotic copies may also be transcribed (see below).

The intron delay hypothesis predicts that the rapid mitotic cycles occurring during early fly embryogenesis will limit production of long zygotic transcripts or prevent their expression altogether. Therefore, we investigated the relationship between primary transcript length and expression by binning genes in both timecourses into two categories: Those with “short” transcripts <5 kb in length and “long” transcripts ≥ 5 kb (table 1; see Materials and Methods). We found that zygotic transcripts are significantly shorter than those maternally deposited (embryonic timecourse median lengths with bootstrapped 95% confidence intervals were 2,287 ± 100 and 3,175 ± 109 bp, respectively; Kruskal–Wallis *P* < 10^−^^15^; patterns are similar for the syncytial timecourse). Consistent with this, there is a significant overrepresentation of intronless zygotic genes detectably expressed during the syncytial timecourse (11.1% vs. 7.8% for zygotic and maternal genes, respectively; χ^2 ^= 11.4, 1 degree of freedom [df], *P* = 0.0008), but not among all zygotic genes expressed over the course of the embryonic timecourse (8.5 vs. 7.5% for zygotic and maternal genes, respectively; χ^2 ^= 1.8, 1 df, *P* = 0.18), suggesting that introns are underrepresented only in zygotic genes expressed during the earliest stages of development.

**Table 1.** Summary Statistics for the Embryonic, Syncytial, and Species Timecourses.

Timecourse	Expression Origin	Length Category	Number of Genes	Median Primary Transcript Length (bp)
Embryonic	Zygotic	Short (<5 kb)	2,100	1,699 ± 61
		Long (≥5 kb)	777	11,300 ± 1,121
	Maternal	Short (<5 kb)	3,058	2,222 ± 64
		Long (≥5 kb)	1,517	10,303 ± 485
Syncytial	Zygotic	Short (<5 kb)	1,184	1,690 ± 78
		Long (≥5 kb)	309	10,365 ± 1,615
	Maternal	Short (<5 kb)	2,843	2,242 ± 66
		Long (≥5 kb)	1,308	9,702 ± 587
Species	Zygotic	Short (<5 kb)	684	191 ± 28
		Long (≥5 kb)	93	10,933 ± 2,092
	Maternal	Short (<5 kb)	1,142	242 ± 20
		Long (≥5 kb)	148	10,244 ± 2,001

Note.—Median primary transcript length is shown with bootstrapped 95% confidence intervals. Note that the species timecourse classifications and median length were calculated using mean orthologous intron lengths across the four species analyzed.

**Fig. 2.**
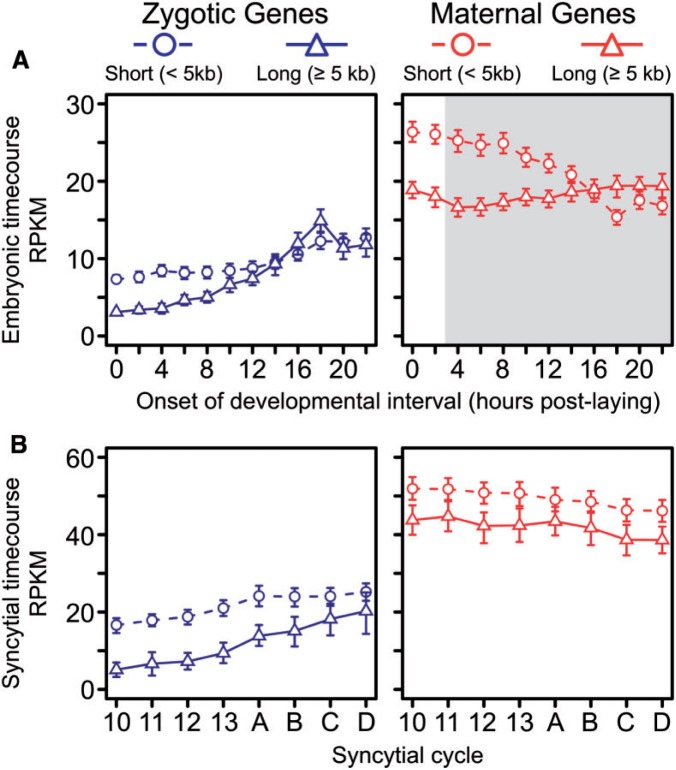
Long, zygotically expressed transcripts show delayed expression in both the embryonic and syncytial timecourses. Median expression levels (with 95% confidence intervals) for zygotic (blue) and maternal (red) genes over the embryonic (*A*) and syncytial (*B*) timecourses. The area shaded in gray indicates postgastrulation time points during which expression levels of maternally deposited genes may include significant contribution from zygotic transcription. Short (<5 kb) and long genes (≥5 kb) are indicated as circles and triangles, respectively. Letters *A–D* in the syncytial timecourse indicate the four consecutive time points collected during the extended 14th mitotic cycle. Median expression levels of both zygotic gene length classes increase over both the embryonic and syncytial timecourses; however, the difference in expression level between two length categories becomes smaller over subsequent stages of development, as predicted by the intron delay hypothesis. Neither length category increases significantly among maternal genes.

A further prediction of the intron delay hypothesis is that the difference in expression level between short and long zygotic genes should be largest during the earliest stages of development and decrease as cell cycle intervals lengthen. To test this, we performed linear regressions on the median expression levels of the two length categories of zygotic genes expressed over the embryonic timecourse (which is not affected by the general tendency for higher expression of short genes) (Duret and Mouchiroud 1999; Castillo-Davis et al. 2002). Although the expression of both short and long zygotic genes increases during embryogenesis (fig. 2*A*), the slope is twice as large for long genes (slope = 0.50 and 1.04, *R*^2 ^= 0.87 and 0.86, *P* = 2.4 × 10^−^^5^ and 3.8 × 10^−^^5^ for short and long genes, respectively). Consistent with this, analysis of covariance (ANCOVA) revealed that the difference in expression between long and short transcripts decreases over development (*F*_3,8_ = 30.3, *P* = 1.0 × 10^−^^4^) (these conclusions remain robust to the removal of any single time point). Reflecting their origin in the embryo, both short and long maternally deposited genes are expressed at high levels during early development (fig. 2*A*). After gastrulation (∼2–4 h time point), the level of expression of genes of maternal origin increasingly begins to reflect the specific regulatory dynamics of zygotic transcription. For instance, we observed a weak pattern of increasing expression among long transcripts of maternal origin beginning approximately 6 h, which is consistent with the prolonged delay of the zygotic contribution to expression of these transcripts into mid-embryogenesis (fig. 2*A*; see below).

We observed the same general patterns in the syncytial timecourse (fig. 2*B*): Median expression levels of both zygotic size classes increased (slope = 1.31 and 2.28, *R*^2^ = 0.91 and 0.97, *P* = 6.9 × 10^−^^4^ and = 2.6 × 10^−^^5^, for short and long genes, respectively), and ANCOVA again revealed that the difference in median expression levels between short and long genes decreased over the timecourse (*F*_3,4_ = 196.9, *P* = 8.5 × 10^−^^5^). Zygotic expression does not contribute substantially to the abundance of maternally deposited transcripts, and accordingly both short and long maternal transcripts showed a significant decrease in median expression level (slope = −1.0 and −0.79, *R*^2^ = 0.90 and 0.71, *P* = 5.6 × 10^−^^6^ and 0.021, for short and long genes, respectively). These patterns remained consistent when using maternal/zygotic classifications from De Renzis et al. (2007) (supplementary fig. S1, Supplementary Material online). Therefore, the patterns observed among zygotic genes are not a general pattern related to transcript length, but rather reflect the expression dynamics of transcripts expressed from the zygotic genome, and both data sets support the predictions of the intron delay hypothesis.

### Patterns of RNA-Seq Coverage Are Consistent with Intron Delay

The decreasing difference in median expression levels of short and long zygotically expressed genes during development is consistent with intron delay. However, it could also be explained by differences in transcription initiation. Therefore, we analyzed additional data sets in order to distinguish between these possibilities (we discuss and reject a third mechanism, a kinetic model explaining the delay of long genes, in the supplementary material S1, Supplementary Material online).

**Fig. 3.**
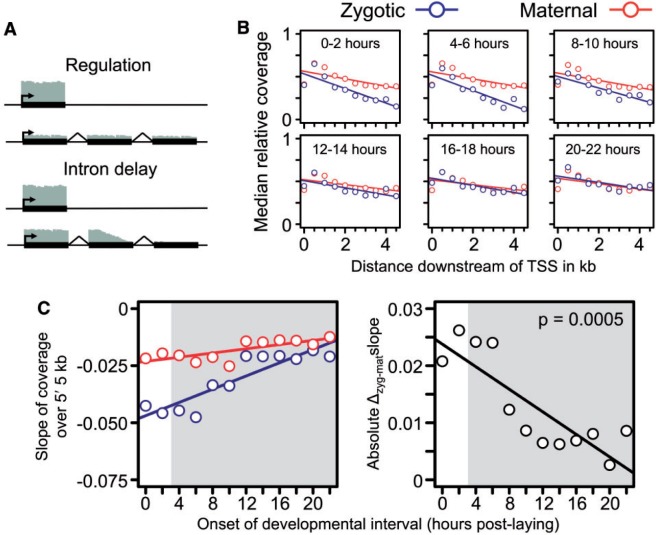
Patterns of transcript coverage are consistent with intron delay. (*A*) Illustration of the predicted read coverage (indicated as gray bars) along short and long transcripts under transcriptional initiation versus intron delay models explaining the lower expression of long zygotic transcript during early development. Under a regulatory model, all transcripts should have relatively similar read coverage along their lengths. Under the intron delay model, relative coverage should decline along the length of the transcript; however, the slope of this decline should become less negative as cell cycles lengthen. (*B*) As predicted by the intron delay model, read coverage declines more rapidly over the first 5 kb of transcripts for zygotic (blue) as compared with maternal (red) transcripts. (*C*) The difference in measured slopes decreases significantly over the 12 2-h developmental windows. Slopes are derived from linear regressions of median coverage over the embryonic time points, and the *P* value of the regression of slope differences over the timecourse is indicated in the panel. The area shaded in gray indicates postgastrulation time points during which the expression levels of maternally deposited genes may include significant contribution from zygotic transcription. Panel *B* with all 12 time points is presented in supplementary figure S2, Supplementary Material online.

Under a model whereby expression of long transcripts is delayed solely by regulatory mechanisms, we would expect to observe relatively consistent levels of coverage across the lengths of genes, with coverage increasing over development (fig. 3*A*). In contrast, a key prediction unique to intron delay is the presence of incomplete long transcripts, which should manifest during early development as a decreasing level of coverage from the 5′- to 3′-ends (fig. 3*A*). However, the slope of this decrease should become less pronounced over development as cell cycles lengthen, allowing complete transcription of progressively longer zygotic transcripts (Rothe et al. 1992).

The poly-A selection used to generate the embryonic timecourse leads to a correlation between gene length and the degree of 3′-bias in read coverage (Zheng et al. 2011). Therefore, in order to measure 5′- to 3′-coverage over development in an unbiased manner, we obtained another modENCODE RNA-seq data set consisting of non-poly-A selected RNA extracted from the same 12 time points as the embryonic timecourse (Graveley et al. 2011). We plotted median base-level exonic coverage (normalized as a fraction of maximum coverage) in nonoverlapping 500-bp windows over the first 5 kb of zygotic and maternal transcripts with measurable expression (fig. 3*B*, supplementary material S1 and fig. S2, Supplementary Material online). Consistent with the intron delay model, the negative relationship between coverage and length is stronger for zygotic as compared with maternal transcripts during early development. When extended to the entire embryonic timecourse, the excess of 5′ versus 3′ coverage of transcripts of entirely zygotic origin decreased as development progressed (fig. 3*C*). In contrast, coverage across the first 5 kb of transcripts of maternal origin remained relatively consistent—likely reflecting a balance between initial maternal deposition and later zygotic transcription.

To further examine coverage levels across genes, we calculated RPKMs for the 5′- and 3′-most 1 kb of exonic transcript and plotted the medians of the 5′:3′ ratios at each time point over the first 12 h of development (supplementary material S1 and fig. S3, Supplementary Material online). Only long zygotic genes show a significant change, with the 5′:3′ ratio decreasing over time, supporting the results of the coverage analysis above (*P* = 0.0091; *P* >0.05 for short zygotic, and short or long maternal categories). We also note that we observed no evidence of differential histone modifications (which are associated with transcription) in the promoters of short versus long zygotic genes within the first half of embryogenesis (supplementary material S1 and fig. S4, Supplementary Material online).

An alternative to both intron delay and transcriptional initiation-based models of delayed activation of long zygotic genes could involve these transcripts being more rapidly degraded than short zygotic transcripts by some yet unidentified mechanism. Thomsen et al. (2010) identified transcripts with patterns of stable expression versus those undergoing degradation during a period coinciding with the syncytial timecourse. We found no significant difference in the proportion of short versus long zygotic genes with stable versus degrading expression (χ^2 ^= 1.4, *P* = 0.25).

### Intron Delay Is Observed Across the *Drosophila* Phylogeny

Having identified a widespread role for intron delay in *D. melanogaster*, we sought to determine if these patterns were shared in other species of flies, and whether intron delay has impacted the evolution of gene structure or expression. Using the maternal and zygotic gene designations from *D. melanogaster*, we analyzed a microarray timecourse spanning embryos reared at 25 °C and collected at 2-h intervals over the first 18 h of embryonic development in six *Drosophila* species (hereafter the “species timecourse”) (Kalinka et al. 2010) (fig. 1). We focused our analysis on four species with high-quality annotations: *D. melanogaster*, *D. ananassae* (∼12 My divergence time from *D. melanogaster*), *D. pseudoobscura* (∼45 My), and *D. virilis* (∼63 My). Among the transcripts represented in the data set, 2,067 genes were represented in the other timecourses and had identifiable 1:1 orthologs among all four species (see Materials and Methods) (McQuilton et al. 2012). Because significant changes in transcript lengths between species are likely to occur via changes in intron lengths—and due to the difficulty in annotating untranslated regions in these other species—we classified genes based on the length of orthologous introns within orthologous genes (see Materials and Methods): Genes were separately binned in each species into short (<5 kb) and long (≥5 kb) categories based on total orthologous intron length.

**Fig. 4.**
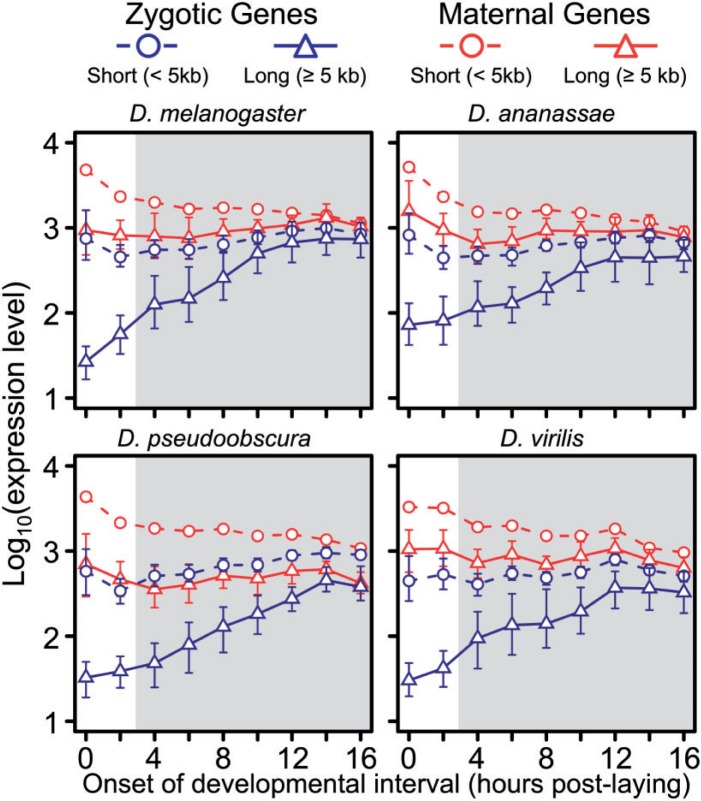
Patterns of delayed activation of long zygotic transcripts are consistent across *Drosophila* species. Median expression levels (with 95% confidence intervals) for *Drosophila melanogaster* zygotic (blue) and maternal (red) genes among the species of the four species microarray-based timecourse. Maternal and zygotic classifications are taken from *D. melanogaster* (Tadros et al. 2007). The area shaded in gray indicates postgastrulation time points during which the expression levels of maternally deposited genes may include significant contribution from zygotic transcription. Short genes (<5 kb) and long genes (≥5 kb) are indicated as circles and triangles, respectively. All three non-*melanogaster* species show patterns consistent with the RNA-seq based embryonic timecourse.

Analysis of the microarray data using the same methods as above showed parallel results in all four species: Long zygotic genes increase significantly in expression level over the timecourse (*P* < 0.001 in all cases; fig. 4). Furthermore, the rate of increase in expression level was significantly greater for long as compared with short zygotic genes in all four species (ANCOVA: *F*_3,5_ *P* < 0.05). Conversely, no significant trend among the median expression levels across time points was observed for short zygotic transcripts in any species after correction for multiple tests. As expected, maternally deposited genes were generally expressed at high levels during early embryogenesis and showed either decreasing or stable expression past the stage where most expression is supplied via zygotic transcription (fig. 4).

### Conservation of Short Introns in Highly Expressed Zygotic Genes

**Fig. 5.**
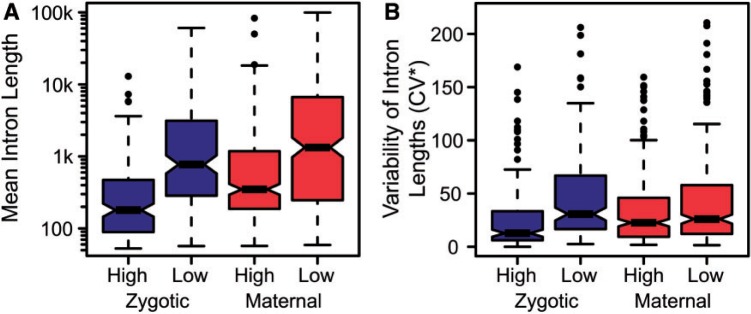
Highly expressed zygotic genes show conservation of short intronic length. (*A*) Highly expressed zygotic genes during the 0–2 h time point of the species timecourse have significantly shorter introns than all other categories. The distributions of mean orthologous intron length are significantly different among all categories (Kruskal–Wallis rank sum test, *P* < 0.001) with the exception of the comparison between low-expression zygotic and high-expression maternal gene categories (*P* = 0.40). (*B*) Highly expressed zygotic genes have significantly lower variability in length than other categories as measured by the corrected CV* in orthologous intron length among the four species (*P* < 0.01).

Our observation that longer transcripts have delayed embryonic expression led us to predict that zygotic genes that are highly expressed during early embryogenesis across species should be subject to selection against intron expansion. Consequently, highly expressed zygotic genes should be more conserved for short transcript lengths than other gene categories. We tested this prediction by dividing genes based on their expression levels in the first time point (0–2 h) of the species timecourse: Zygotic genes in the highest- and lowest-expressed quartiles in *D. melanogaster* (“high-” and “low-expression zygotic”; 100 and 73 genes, respectively), as well as maternal genes in these same quartiles (“high-” and “low-expression maternal”; 142 and 189 genes, respectively). As expected, both zygotic and maternal high-expression genes have shorter mean intron lengths than their corresponding low-expression genes (Kruskal–Wallis rank sum test, *P* < 10^−^^15^) (fig. 5*A*). Furthermore, the highly expressed zygotic genes have the shortest mean intron lengths overall (*P* < 10^−^^15^). We then measured the variability of orthologous intron lengths in each category across the *Drosophila* phylogeny by calculating the corrected coefficient of variation (CV*) of intron lengths for the four species (fig. 5*B*) (see Materials and Methods). The CV* values, as well as intron lengths, of highly expressed zygotic genes are significantly lower than all other categories (*P* < 0.01), suggesting that there exists significant constraint on the expansion of intron lengths among highly expressed zygotic genes during early fly development.

## Discussion

### Genome-Wide Intron Delay in *Drosophila*

The results of our analysis suggest that intron delay plays a significant role in determining patterns of expression in the early development of *Drosophila*. Although a negative relationship between transcript length and expression level across a wide variety of organisms has been noted for some time (Duret and Mouchiroud 1999; Castillo-Davis et al. 2002), this cannot explain our observation that in all three timecourses, the magnitude of the difference in expression level between the two length categories of genes declines across development. Furthermore, our observations are not consistent with reduced transcriptional initiation limiting the transcription of long zygotic transcripts, as evidenced by the declining 5′-bias in coverage among zygotic genes over the course of development (fig. 3*B* and *C*, supplementary material S1 and figs. S2 and S3, Supplementary Material online), as well as the lack of explanatory patterns in well-studied activating or repressive chromatin marks (supplementary material S1 and fig. S4, Supplementary Material online).

Though intron delay clearly places an upper limit on the expression of long zygotic genes, the inability to complete transcription cannot be the sole factor limiting their early expression as the earliest zygotic transcripts are not detected until syncytial cycles 7–8 (Bownes stage 2) (ten Bosch et al. 2006; Ali-Murthy et al. 2013). Furthermore, experimental-forced arrest of embryos in nonmitotic portions of the cell cycle does not lead to full zygotic activation prior to syncytial cycle 10 (Edgar and Schubiger 1986). Therefore, it appears that the earliest steps of zygotic activation require the action of maternally deposited genes (Tadros and Lipshitz 2009).

The higher proportion of entirely intronless zygotic genes expressed during the syncytial timecourse, and not among those zygotically transcribed during other periods of embryogenesis, suggests that purifying selection against long transcripts is strongest during this stage. Nevertheless, in both the RNA-seq data (fig. 2*A*) and four-species microarray data (fig. 4), the expression of long zygotic genes continues to increase more rapidly than short genes well into embryogenesis (∼12–18 h). Although it is unlikely that cell cycle durations continue to lengthen over this entire time, our results suggest that production of incomplete transcripts may continue throughout this extended period. Delay could persist after gastrulation due to the formation of mitotic domains: Regions of the embryo that begin amplifying their genomic content via endocycling (replication of all or parts of the genome via a modified cell cycle that bypasses mitosis as well as large portions of the gap phases to produce polyploid nuclei) (Foe 1989). This modified cell cycle may be shortened, and therefore physically limit long zygotic transcripts from achieving maximal expression until mid-embryogenesis within specific sections of the embryo.

Finally, a recent study of the syncytial divisions in *Drosophila* found evidence that intron splicing may be less efficient during syncytial stages, contributing to the lack of production of long intron-containing transcripts (Guilgur et al. 2014). If splicing efficiency varies both temporally and spatially across the *Drosophila* embryo, it could contribute to the persistence of delay, though future studies are required to determine if such a pattern exists.

### Embryonic Expression Across *Drosophila*

At present, we only have information on the maternally deposited transcriptome for *D. melanogaster*. However, when maternal and zygotic gene classifications from *D. melanogaster* are applied to species up to 63 My diverged, patterns of embryonic expression remain qualitatively similar, indicating that delayed expression of long transcripts during early embryogenesis is a common phenomenon across *Drosophila* (fig. 4). The consistent, significant differences observed in expression patterns among short and long zygotic transcripts as well as maternal genes across the phylogeny suggest that the origin of these transcripts within the developing embryo may be largely conserved (see below).

Results of simulation studies have suggested that the size and distribution of most metazoan introns are consistent with neutral evolutionary processes, reflecting the balance between insertion and deletion (Lynch 2002, 2005; Lynch and Richardson 2002; Lynch and Kewalramani 2003). Our results suggest that while these neutral dynamics may apply to lowly expressed zygotic genes, many highly expressed early zygotic genes are likely subject to purifying selection to maintain short transcript lengths (fig. 5).

### Maternal Deposition versus Zygotic Transcription

Wieschaus (1996) hypothesized that “[i]n organisms where embryonic development is rapid and occurs with no increase in size before hatching from the egg, it will be advantageous to maximize maternal contributions, because the duration of oogenesis is often much longer than embryogenesis and the ovary provides a more sophisticated and efficient synthetic machinery.” Nevertheless, a significant fraction of embryonic transcripts in rapidly developing species originate zygotically: 30–35% in *D. melanogaster* (Tadros et al. 2007) and approximately 30% in the nematode *Caenorhabditis elegans* (Baugh et al. 2003). It is likely that maternal deposition of some fraction of these transcripts is deleterious—especially if they require precise spatial regulation (Wieschaus 1996; Lécuyer et al. 2007). Alternatively, mothers may seek to minimize unnecessary resource investment in producing mRNAs that offer no advantage over zygotic transcription, such as in the case of short genes that are not restricted by the constraints of intron delay. Supporting this possibility, genes expressed during the embryonic timecourse whose processed mRNAs are ≥5 kb are significantly overrepresented among maternal as compared with zygotic genes (567 vs. 239, χ^2 ^= 126, 1 df, *P* < 0.0001). Determining which transcripts are supplied maternally versus expressed zygotically in closely related species would allow us to establish whether transitions to maternal deposition are common and whether such events favor particular types of genes (e.g., those with long preprocessed transcripts).

## Conclusion

Intron delay appears to play a significant role in determining patterns of expression beginning from the earliest moments of *Drosophila* zygotic genome activation, leading to clear expectations that zygotic mRNAs derived from long primary transcripts may take several hours after zygotic genome activation to reach full expression levels. Interestingly, this phenomenon does not appear to be restricted to invertebrates, as recent studies have suggested that delay of long transcripts has a significant effect in early mammalian development as well (Graf et al. 2014). Although evidence exists that intron delay may play an active regulatory role in preventing expression of transcripts until they are required by the developing embryo (Takashima et al. 2011), additional studies are required to determine to what extent such a mechanism can be generalized (Gubb 1986). At present, it is difficult to discern whether delayed expression of some long genes may be under direct transcriptional control in addition to being subject to intron delay. As we continue to decipher the regulatory logic underlying transcription, we should be able to identify candidate genes whose long introns could be experimentally deleted and assessed for elimination of delay (Rothe et al. 1992). Information gleaned from a sufficiently large sample of such genes will allow us to determine to what degree intron delay is used as an active mechanism of temporal regulation.

## Materials and Methods

### RNA and ChIP-seq Data

Mapped data from Graveley et al.’s (2011) timecourse for each of the 12 time points spanning embryogenesis were obtained from the ModENCODE Data Coordination Center (http://www.modencode.org/, last accessed March 1, 2012; data sets modENCODE_2884 to modENCODE_2895). Counts of all 15,233 annotated loci (excluding pseudogenes and microRNA precursors) with FlyBase gene identification numbers (FBgns) in the FlyBase *D. melanogaster* genome annotation release 5.43 (FBr5.43) (McQuilton et al. 2012) were calculated using HTseq-count at the gene level with the “union” option (http://www-huber.embl.de/users/anders/HTSeq/doc/index.html, last accessed March 1, 2012). Data were normalized by conditional quantile normalization using the “cqn” Bioconductor package in R version 2.14 (Hansen et al. 2012) and expression levels were output as RPKM. The raw RNA-seq reads from Lott et al. (2011) were obtained from the National Center for Biotechnology Information’s (NCBI) Gene Expression Omnibus (GEO) (accession GSE25180) and mapped to the FlyBase *D. melanogaster* genome release 5 using Tophat 1.0.13 (Trapnell et al. 2009) with default settings with the exception of a minimum intron length of 42 and retaining only uniquely mapping reads. Sexed data for each stage were collapsed and counting, normalization, and RPKM calculation were performed as for the Graveley et al. (2011) data set. In both data sets, we required that a gene be expressed at RPKM >5 during at least one stage in order to be considered for analysis, leaving 10,454 loci in the Graveley et al. (2011) data set and 7,223 loci in the Lott et al. (2011) data set (lowering the threshold of expression to RPKM >2 had no effect on our conclusions).

We obtained the maternal and zygotic gene classifications of Tadros et al. (2007) as tabulated in NCBI GEO entry GSE8910. All loci represented on the microarray platform used in the study were converted to current FBgns using the FlyBase batch download tool. Those loci that were no longer part of the current annotation were excluded, while instances where multiple loci had been collapsed into a single locus in the current annotation were inspected to determine whether all collapsed loci were originally classified into the same category (i.e., maternal or zygotic). All cases where collapsed loci disagreed in terms of classification were rejected, providing 9,078 loci in the FBr5.43 annotation classified as maternally deposited or zygotically expressed, of which 7,452 (4,575 [61%] maternal/2,877 [39%] zygotic) were expressed in the Graveley et al. (2011) data set and 5,644 (4,151 [74%] maternal/1,493 [26%] zygotic) were expressed in the Lott et al. (2011) data set.

For the comparison of degraded versus stable transcripts among short and long zygotic gene expressed during the syncytial timecourse, we obtained the classifications from Thomsen et al. (2010) (ArrayExpress accession no. E-MEXP-2580). In order to assign zygotically expressed genes during the syncytial timecourse to either stable or degraded categories, we identified the corresponding FlyBase identifier among the microarray probes and only used genes where all corresponding probes were classified into the same class. We then binned all of the degradation categories together to compare against the stable category.

For the analysis of the distribution of read coverage along transcripts, we obtained the non-poly-A selected embryonic timecourse RNA-seq reads generated by a SOLiD instrument (Life Technologies, Carlsbad, California) from the NCBI Short-read archive (SRA Accession numbers: SRX015641–SRX015652) (Graveley et al. 2011). Reads were mapped to the *D. melanogaster* genome using the same methods as those applied to the syncytial timecourse of Lott et al. (2011). Using a custom PERL script combined with HTseq-count at the locus level with the union option, we counted the number of reads mapping to nonoverlapping 500-bp windows over the first 5 kb of each transcript excluding any intronic sequence. Because non-poly-A selected RNA contains a mixture of both processed and unprocessed pre-mRNAs we chose to look only at those sequence segments that would be consistent between these two categories. Coverage within each window was normalized to the length of exonic sequence within the window and then scaled as a fraction of the window with maximal coverage within each transcript. Windows within transcripts that contained no exonic sequence were ignored. Windows were then aggregated among all transcripts within a class (zygotic or maternal) and median fractional coverages across transcripts were determined for each of the ten windows. Slopes were determined by linear regression of median fractional coverage versus window number. Only genes with at least 100 mapping reads at any individual time point were included in the analysis.

### Choice of Short and Long Locus Categories

In order to determine appropriate transcript length cutoffs to detect the potential effect of intron delay, we first began by binning all loci in the FlyBase 5.43 annotation into increments of 5 kb (i.e., 5, 10, 15, 20 kb, etc.). Visual inspection indicated that progressively longer bins showed a more pronounced reduction in expression during early stages of development. We then performed pairwise comparisons of the distributions of expression levels of the individual bins during each of the time points and found that there were no significant differences among those bins with loci >5 kb in length (*P*>0.05), whereas these same bins were significantly different from those loci < 5 kb in length. Therefore, we defined two length categories, short (<5 kb) and long (≥5 kb), whose expression patterns were significantly different from one another.

### Four-Species Microarray Data

We obtained the processed microarray data as described in Kalinka et al. (2010) from http://publications.mpi-cbg.de/getDocument.html?id=ff8080812c477bb6012c5fa1feaf0047 (last accessed May 21, 2012). All locus names were associated with *D. melanogaster* FlyBase FBgns. Loci from the Kalinka et al. (2010) data set, which was based on FlyBase annotation 5.14, that were not associated with unique FBgns (either due to a locus having been split into multiple loci or multiple loci having collapsed into a single locus in the FBr5.43 annotation used in this study) were removed from further analysis. As two species, *D. simulans* and *D. persimilis*, were originally noted to have poor genome sequencing coverage (*Drosophila* 12 Genomes Consortium 2007), we used the remaining FBgns to search for orthologs in *D. ananassae* (FlyBase genome release 1.3, annotation release FB2011_07), *D. pseudoobscura* (FlyBase genome release 2.27, annotation release FB2012_02), and *D. virilis* (FlyBase genome release 1.2, annotation release FB2012_01) using the FlyBase batch download tool. Of the 3,146 loci mapping to a single ortholog in all three non-*melanogaster* species, 2,067 were represented among the *D. melanogaster* zygotic and maternal loci annotated by Tadros et al. (2007). These loci were retained for further analysis and were called maternal or zygotic based on the *D. melanogaster* data. We used the average normalized, processed expression level among all probes represented over time points 1–9 for each locus within a given species for analysis as data was not available for all species for any subsequent time points.

### Orthologous Intron Analysis

As the genome annotations of non-*melanogaster* species of *Drosophila* largely lack untranslated regions as well as alternatively spliced isoforms that could lead to changes in primary transcript length, we sought to compare only orthologous intronic segments. These segments were identified using the software Common Introns Within Orthologous Genes (Wilkerson et al. 2009) on the genome releases indicated above, retaining only those segments that were common among all four species analyzed. In order to compare variability in intron lengths among all nonsingle-exon genes, we used the corrected CV*: CV* = (1 + 1/4n) × CV, where *n* is the number of observations (Sokal and Rohlf 1995). It should be noted that CV* is biased toward low values when mean intron lengths among species are <150 bp as is the case with most introns in the highly expressed zygotic category (fig. 5). Upon reanalyzing the data after removing all loci with mean intron length among species <150 bp, the only significant difference in CV* is observed among highly expressed zygotic and low expressed maternal transcripts (*P* < 0.01). However, this serves to indicate that the transcript length range tolerated by highly expressed zygotic loci during early development is short and narrow relative to other locus categories.

### General Statistics

All statistics were performed using R version 2.14.0 (R Development Core Team 2008). Confidence intervals were obtained by producing a normal approximation of 10,000 resampled subsets of the data using the “boot” package in R (Davison and Hinkley 1997). Comparisons between distributions were performed using the permuted Kruskal–Wallis rank sum test, with 10,000 permutations, as implemented in the “coin” package in R (Hothorn et al. 2008). The *P* values of all comparisons were Bonferroni corrected for multiple tests where appropriate

## Supplementary Material

Supplementary material S1 is available at *Molecular Biology and Evolution* online (http://www.mbe.oxfordjournals.org/).

Supplementary Data
