# Supplementary material for: Transcript Length Mediates Developmental Timing of Gene Expression Across Drosophila
Source: Mol Biol Evol. 2014 Jul 28;31(11):2879–89. doi: 10.1093/molbev/msu226 (PMC4209130; doi:10.1093/molbev/msu226)
Supplement: Supplementary Data [file supp_31_11_2879__index.html]

Transcript Length Mediates Developmental Timing of Gene Expression Across Drosophila — Transcript Length Mediates Developmental Timing of Gene Expression Across Drosophila — Supplementary Data 

# Transcript Length Mediates Developmental Timing of Gene Expression Across *Drosophila*

## Supplementary Data

file

**Files in this Data Supplement:**

- Supplementary Data - pdf file
- Supplementary Data - pdf file
