# Supplementary material for: Transcript Length Mediates Developmental Timing of Gene Expression Across Drosophila
Source: Mol Biol Evol. 2014 Jul 28;31(11):2879–89. doi: 10.1093/molbev/msu226 (PMC4209130; doi:10.1093/molbev/msu226)
Supplement: Supplementary Data [file supp_msu226_Artieri_and_Fraser-Supplementary_Material.pdf]

SUPPLEMENTARY MATERIAL

Rejection of a kinetic mechanism restricting early expression of long zygotic transcripts

A third potential explanation for the low expression level of long transcripts followed by a progressive reduction in the ratio of short/long gene expression is transcriptional kinetics. Consider a simple model under which a single RNA Pol II complex transcribes a locus at any one time. Assuming that expression level eventually reaches saturation, multiple short transcripts could be produced in the same amount of time required to transcribe a long transcript, allowing short genes to reach saturation while delaying maximal expression of longer genes. However, there are several lines of evidence that argue against this possibility. First, while it is not known whether co-transcription of multiple RNA Pol II complexes is a common feature of long genes in general, it has been observed *in situ* at a number of specific loci, suggesting that such a simple model does not capture biological reality (e.g., Beyer and Osheim 1988; Osheim and Beyer 1989). Second, a purely kinetic model cannot explain the decreasing ratio of 5′ - 3′ coverage observed among long zygotic transcripts (fig. 3B, supplementary figures S2, and S3) without the addition of degradation of incomplete transcripts upon mitosis as required by the intron delay model. Thus while it impossible to exclude the possibility that transcriptional kinetics are playing some role in the time required for long loci to reach stable levels of expression, it cannot be the primary determinant of expression delay of genes with long transcripts.

Analysis of 5′:3′ ratios over embryogenesis

We calculated the 5′:3′ ratio for each transcript that a) was included as part of the embryonic timecourse (see Methods), b) had a transcript of at least 2 kb in length, c) had only a single TSS according to the FlyBase 5.43 annotation, and d) did not overlap another transcript, leaving 3,396 loci with 5′:3′ ratios to analyze. Only long zygotic genes show a significant change, with the 5′:3′ ratio decreasing over time (slope = -0.162, R^2 = 0.811, p = 0.00905; p > 0.05 for all other categories; supplementary figure S3). We note that extension of the regressions to all 12 time points results in a significant negative slope among all four gene categories (p < 0.05); however long zygotic genes show a significantly steeper negative slope than the other gene categories (ANCOVA, p < 0.001), as expected by predictions of the intron delay hypothesis.

No evidence for delayed initiation of transcription in long genes based on histone marks

As a further confirmation of a lack of evidence for postponement of transcriptional initiation of long zygotic genes, we explored chromatin profiles in a window 1 kb upstream of the TSS using ChIP-Seq data from the modENCODE Consortium (2010) generated using embryos collected during four-hour windows over the first 12 hours of embryogenesis. It is important to note that these windows are too broad to draw conclusions regarding chromatin occupancy during the syncytial timecourse (though a recent study found that there is no evidence for substantial chromatin organization in promoters prior to mitotic cycle 14, as revealed by the lack of H3K4me3 marks; Chen et al. 2013). However, these data may be informative with regard to explaining the continued delay of full expression of long zygotic transcripts during

time points of the embryonic timecourse after full zygotic activation (i.e., ≥ four hours post egg laying).

Expression of long transcripts delayed solely by postponement of transcriptional initiation over this period could manifest itself via two non-mutually exclusive patterns of chromatin coverage (Yin et al. 2011): 1) long zygotic genes could show a paucity of chromatin marks indicating active transcription (H3K4me3) relative to short zygotic genes during early development, with such marks increasing over time, reflecting increased expression, and/or 2) long zygotic genes could show an excess of repressive or heterochromatic chromatin marks (H3K27me3) relative to short zygotic genes during early development, with this excess decreasing over time.

We first confirmed that H3K4me3 occupancy upstream of the TSS was correlated with expression level within the embryonic timecourse (where expression level was calculated as the average RPKM among the two embryonic time points within each 4 hour chromatin timepoint; supplementary figure 4A). Indeed, while expression levels of zygotic genes were positively correlated with H3K4me3 occupancy, the correlation was stronger for short, as compared to long genes, as expected if length is limiting expression. Notably, occupancy differences of active chromatin over the first 12 hours were inconsistent with an initiation-based model as occupancy of long zygotic genes did not increase over the time period (supplementary figure 4A). While upstream occupancy of the repressive chromatin mark H3K27me3 was negatively correlated with expression level for short zygotic genes, we observed no significant correlation in the case of long zygotic genes (supplementary figure 4B). Furthermore, there was no significant decrease

in repressive chromatin mark occupancy over the period, again providing no support to a delayed initiation-based model during this period of embryogenesis.

Transcript length and zygotic origin are not associated with particular functional classes

In order to better characterize potential functional differences among transcripts, we analyzed the representation of the different size categories of maternal and zygotic genes among Gene Ontology (GO) biological process terms using FatiGO (Al-Shahrour et al. 2007). Consistent with previous analyses (Tadros et al. 2007; Thomsen et al. 2010), maternally deposited genes are over-represented as compared to the genome as a whole in a number of functional classes (supplementary table S3). In contrast, we found that zygotic genes did not show significant over-representation among any GO biological process categories ($p > 0.05$). Furthermore, when zygotic and maternal genes were compared to one another, neither long nor short transcripts were significantly over-represented in any GO category. Consequently, there is not a clear set of functions associated with whether zygotic genes are subject to or escape intron delay.

Supplemental Methods

Analysis of 5′:3′ ratios over embryogenesis

Using a custom script, combined with HTseq-count at the locus level with the ‘union’ option, we counted the number of reads spanning the 5′ and 3′ 1 kb of each transcript excluding any intronic sequence during the first 12 hours of development, during which zygotic activation takes place. Read counts were quantile normalized using the R aroma.light package (Bengtsson and Hössjer 2006) and RPKMs calculated.

Gene ontology analysis

All maternal or zygotic loci considered significantly expressed in either the embryonic or syncytial timecourses were analyzed for functional over- and under-representation using FatiGO (Al-Shahrour et al. 2007) on the Babelomics version 4.3 webserver at (http://babelomics.bioinfo.cipf.es/functional.html). Gene lists were compared either to one another or the whole FlyBase 5.43 annotation among GO biological process levels from three to nine using two-tailed tests and retaining only p values < 0.05 when adjusted for multiple tests by the software.

Analysis of histone marks

Raw developmental timecourse ChIP-seq reads derived from antibodies to histone modifications H3K4me3 and H3K27me3 were obtained from GEO (accession numbers to all datasets are found in supplementary table S2) (modENCODE Consortium 2012). All reads were mapped uniquely to the FlyBase *D. melanogaster* genome release 5 using Bowtie version 0.12.8 and allowing 2 mismatches (Langmead et al. 2009). Base-level coverage was assessed in a 1 kb window upstream of the TSS of genes with a single annotated TSS. Coverage was normalized between time points and chromatin marks by dividing by the total number of mapped reads by 10^6.

Supplemental References

Al-Shahrour F, Minguez P, Tárraga J, Medina I, Alloza E, Montaner D, Dopazo J. 2007. FatiGO +: a functional profiling tool for genomic data. Integration of functional annotation, regulatory motifs and interaction data with microarray experiments. Nucleic Acids Res. 35(Web Server issue):W91–96.

Bengtsson H, Hössjer O. 2006. Methodological study of affine transformations of gene expression data with proposed robust non-parametric multi-dimensional normalization method. BMC Bioinformatics. 7:100.

Beyer AL, Osheim YN. 1988. Splice site selection, rate of splicing, and alternative splicing on nascent transcripts. Genes Dev. 2:754–765.

Chen K, Johnston J, Shao Wanqing, Meier S, Staber C, Zeitlinger J. 2013. A global change in RNA polymerase II pausing during the Drosophila midblastula transition. eLIFE. 2:e00861.

Langmead B, Trapnell C, Pop M, Salzberg SL. 2009. Ultrafast and memory-efficient alignment of short DNA sequences to the human genome. Genome Biol. 10:R25.

modENCODE Consortium, Roy S, Ernst J, Kharchenko PV, Kheradpour P, Negre N, Eaton ML, Landolin JM, Bristow CA, Ma L, et al. 2010. Identification of functional elements and regulatory circuits by Drosophila modENCODE. Science. 330:1787–1797.

Osheim Y, Beyer A. 1989. Electron Microscopy of Ribonucleoprotein Complexes on Nascent RNA using Miller Chromatin Spreading Method. Method enzymol. 180:481–509.

Yin H, Sweeney S, Raha D, Snyder M, Lin H. 2011. A high-resolution whole-genome map of key chromatin modifications in the adult Drosophila melanogaster. PLoS Genet. 7:e1002380.

Supplementary Table S2. GEO datasets used in the analysis of ChIP-Seq chromatin marks.

Time point	H3K4me3	H3K27me3
0-4 h	GSM400657	GSM439448
4-8 h	GSM400674	GSM439447
8-12 h	GSM439446	GSM439446

Supplementary Table S3. Complete list of all GO Biological Process terms significantly over-represented among maternal loci in comparison to the genome as a whole. The loci determined to be expressed during the embryonic and syncytial timecourses were analyzed separately. p-values are adjusted to reflect a false-discovery rate of 0.05.

Embryonic Timecourse

GO Term ID	GO Term Name	Loci in Dataset	Percent Among Maternal Loci	Percent Among Entire Genome	Adjusted p-value
GO:0007049	Cell Cycle	642	3.96	3.03	4.43E-02
GO:0006508	Proteolysis	920	5.79	4.3	1.83E-03
GO:0006950	Response to stress	1009	6.34	4.72	1.09E-03
GO:0009056	Catabolic process	1244	7.91	5.79	1.78E-04
GO:0009266	Response to temperature stimulus	596	3.83	2.76	9.17E-03
GO:0009409	Response to cold	550	3.61	2.53	5.40E-03
GO:0009628	Response to Abiotic stimulus	710	4.48	3.32	8.17E-03
GO:0030163	Protein catabolic process	936	5.95	4.36	9.31E-04
GO:0042309	homoiothermy	548	3.61	2.51	5.02E-03
GO:0042592	Homeostatic process	695	4.59	3.18	8.69E-04
GO:0050826	Response to freezing	548	3.61	2.51	5.02E-03
GO:0016070	RNA metabolic process	1305	8.13	6.12	3.71E-04
GO:0007166	cell surface receptor signaling pathway	929	5.68	4.39	9.90E-03
GO:0006350	Transcription	1086	6.8	5.09	9.11E-04
GO:0006351	transcription, DNA-dependent	956	5.9	4.5	5.40E-03
GO:0006355	regulation of transcription, DNA-dependent	900	5.68	4.2	1.83E-03
GO:0019222	regulation of metabolic process	1362	8.55	6.37	1.78E-04
GO:0045449	Regulation of transcription	995	6.32	4.63	7.98E-04
GO:0006412	Translation	489	3.1	2.28	4.23E-02
GO:0005975	carbohydrate metabolic process	526	3.3	2.46	4.73E-02

GO:0016192	vesicle-mediated transport	470	3.04	2.17	2.49E-02
GO:0007242	intracellular signal transduction	554	3.61	2.55	7.16E-03
GO:0007399	nervous system development	672	4.28	3.12	6.92E-03
GO:0009653	anatomical structure morphogenesis	1159	7.23	5.44	7.98E-04
GO:0009887	organ morphogenesis	610	3.83	2.86	2.61E-02
GO:0022008	neurogenesis	500	3.19	2.32	3.07E-02
GO:0030154	cell differentiation	1072	6.86	4.98	2.62E-04
GO:0009888	tissue development	563	3.54	2.63	3.25E-02
GO:0006836	neurotransmitter transport	147	1.14	0.62	2.08E-02

Syncytial Timecourse

GO Term ID	GO Term Name	Loci in Dataset	Percent Among Maternal Loci	Percent Among Entire Genome	Adjusted p-value
GO:0007049	Cell Cycle	635	4.19	3.03	7.68E-03
GO:0006508	Proteolysis	891	5.69	4.3	6.41E-03
GO:0006950	Response to stress	982	6.34	4.72	2.22E-03
GO:0009056	Catabolic process	1209	7.88	5.79	2.87E-04
GO:0009266	Response to temperature stimulus	582	3.88	2.76	7.72E-03
GO:0009409	Response to cold	538	3.69	2.53	4.23E-03
GO:0009628	Response to Abiotic stimulus	694	4.55	3.32	6.41E-03
GO:0030163	Protein catabolic process	905	5.81	4.36	5.15E-03
GO:0042309	homoiothermy	536	3.69	2.51	3.74E-03
GO:0042592	Homeostatic process	680	4.7	3.18	6.32E-04
GO:0050826	Response to freezing	536	3.69	2.51	3.74E-03
GO:0006396	RNA processing	289	2.02	1.35	3.46E-02
GO:0016070	RNA metabolic process	1274	8.21	6.12	3.57E-04

GO:0007166	cell surface receptor signaling pathway	904	5.66	4.39	1.71E-02
GO:0006350	Transcription	1059	6.84	5.09	1.35E-03
GO:0006351	transcription, DNA-dependent	933	5.95	4.5	5.53E-03
GO:0006355	regulation of transcription, DNA-dependent	879	5.76	4.2	1.97E-03
GO:0019222	regulation of metabolic process	1330	8.65	6.37	2.87E-04
GO:0045449	Regulation of transcription	971	6.38	4.63	7.69E-04
GO:0042221	response to chemical stimulus	362	2.43	1.71	4.39E-02
GO:0005975	carbohydrate metabolic process	515	3.37	2.46	3.23E-02
GO:0006812	cation transport	394	2.65	1.86	3.46E-02
GO:0016044	cellular membrane organization	372	2.48	1.77	4.85E-02
GO:0016192	vesicle-mediated transport	456	3.01	2.17	3.46E-02
GO:0007242	intracellular signal transduction	541	3.66	2.55	6.41E-03
GO:0007389	pattern specification process	414	2.77	1.96	3.46E-02
GO:0007399	nervous system development	657	4.36	3.12	5.53E-03
GO:0009653	anatomical structure morphogenesis	1127	7.2	5.44	1.72E-03
GO:0009887	organ morphogenesis	594	3.83	2.86	3.23E-02
GO:0022008	neurogenesis	488	3.23	2.32	3.00E-02
GO:0030154	cell differentiation	1046	6.94	4.98	2.87E-04
GO:0030182	neuron differentiation	433	2.89	2.05	3.28E-02
GO:0009888	tissue development	553	3.66	2.63	1.45E-02
GO:0030030	cell projection organization	397	2.63	1.89	4.87E-02
GO:0048666	neuron development	369	2.51	1.74	3.46E-02

GO:0051726	regulation of cell cycle	159	1.2	0.72	4.39E-02
GO:0007610	behavior	350	2.41	1.64	3.16E-02
GO:0000278	mitotic cell cycle	440	2.91	2.09	3.46E-02
GO:0007422	peripheral nervous system development	95	0.79	0.41	4.39E-02
GO:0006163	purine nucleotide metabolic process	187	1.4	0.85	3.46E-02
GO:0006164	purine nucleotide biosynthetic process	182	1.35	0.83	4.39E-02
GO:0009117	nucleotide metabolic process	263	1.95	1.19	9.35E-03
GO:0009165	nucleotide biosynthetic process	221	1.59	1.02	4.39E-02
GO:0006836	neurotransmitter transport	140	1.08	0.62	4.39E-02
GO:0015672	monovalent inorganic cation transport	291	2.02	1.36	3.96E-02

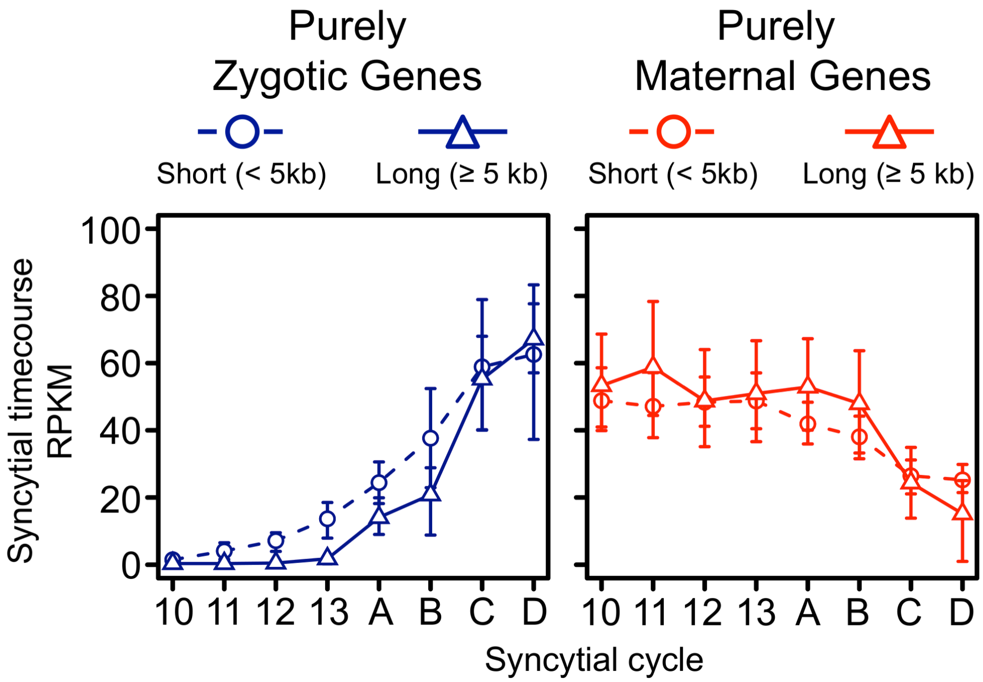

Supplementary Figure S1. Classification of genes as purely zygotic or purely maternal (no zygotic expression during the syncytial timecourse) according to De Renzis et al. (2007) produces results consistent with those observed based on the classifications of Tadros et al. (2007) used in the main manuscript. As expected, purely maternal genes show a sharp decrease in expression level over the timecourse as they are subject to degradation during this period. Gene classifications were obtained from De Renzis et al. (2007) Tables S3 and S4 and associated with the current annotation based on FlyBase gene identifiers (when available), and then FlyBase CG numbers (supplementary table S1).

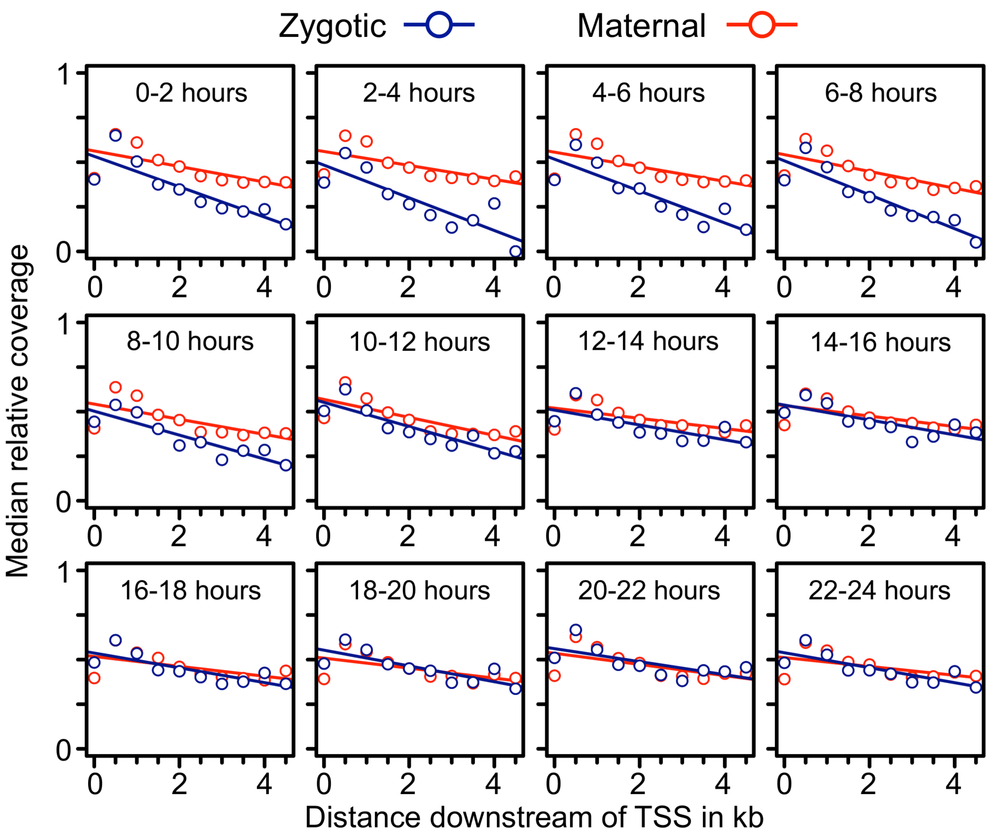

Supplementary Figure S2. Reproduction of fig. 3B showing all 12 developmental time points. The pattern of more rapidly declining read coverage over the first 5 kb of transcripts for zygotic (blue) as compared to maternal (red) transcripts holds during the stages not show in fig. 3B. Furthermore, the difference in measured slopes decreases significantly over the 12 2-hour developmental windows. Note that beginning at the 4h time point, expression levels, and hence coverage patterns of genes of maternal origin are largely determined by zygotic transcription.

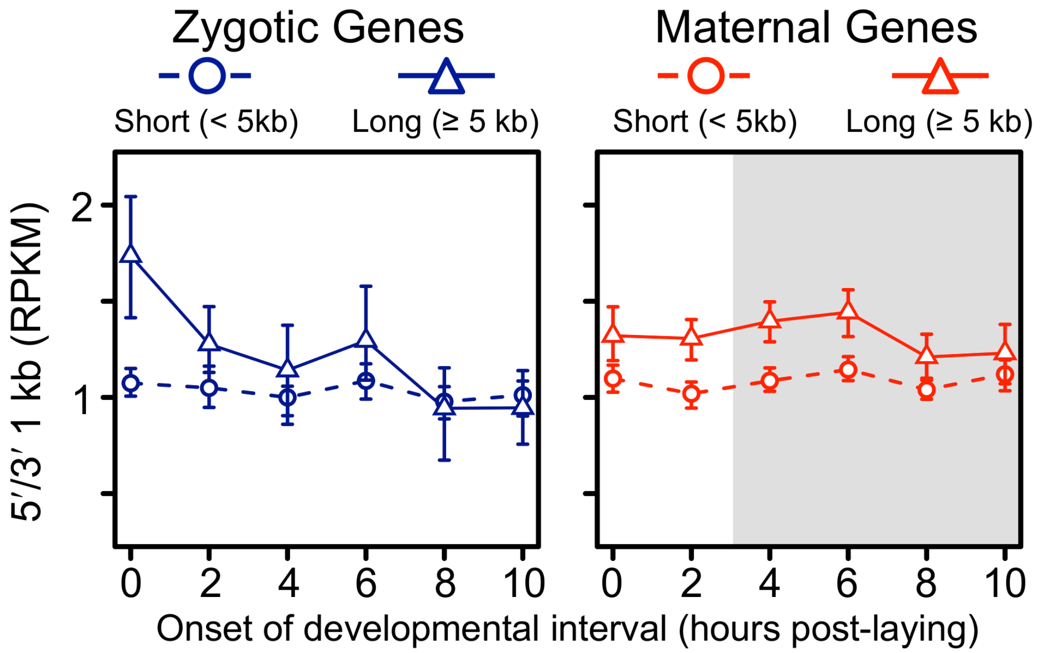

Supplementary Figure S3. 5′:3′ ratios of coverage among different transcript categories support the intron delay model. Under a regulatory model, the 5′ and 3′ ends of all transcripts should have relatively similar read coverage. Under the intron delay model, however, the 5′:3′ ratio of long genes should be > 1 during early development, and decrease as development progresses. Median 5′:3′ ratios over the embryonic timecourse as determined from total RNA data are indicated for zygotic (blue) and maternal (red) genes. Short (< 5 kb) and long (≥ 5 kb) genes are indicated as circles and triangles, respectively. The area shaded in grey indicates time points during which expression levels of genes of maternal origin is largely determined by zygotic transcription. The 5′:3′ ratio decreased over the first six hours of development in long zygotic genes, but not in any other category, as expected under the predictions of the intron delay model.

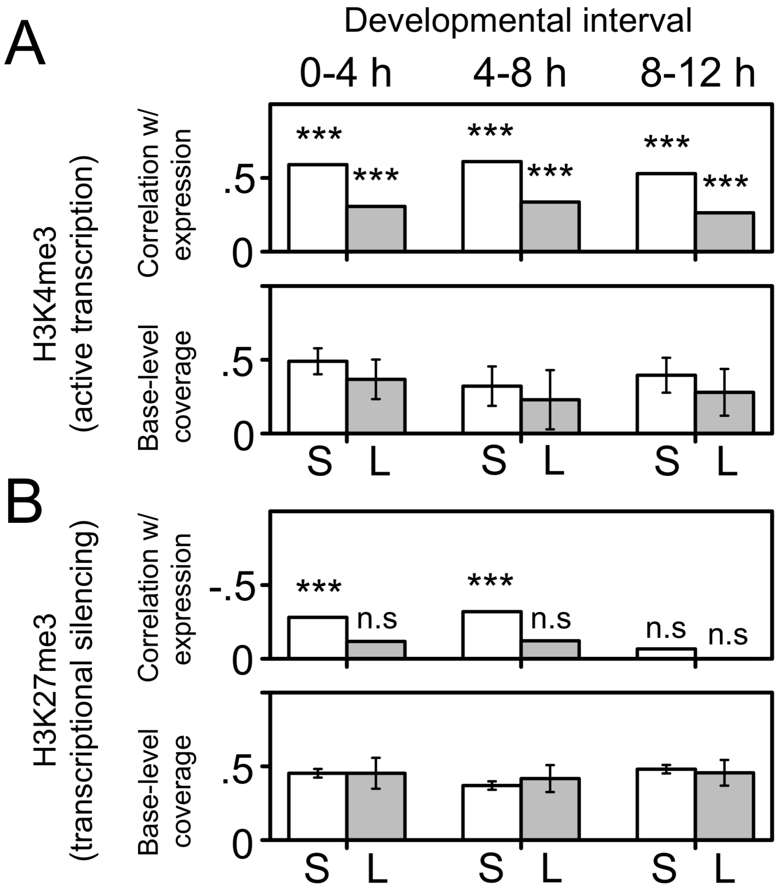

Supplementary Figure S4. The presence of heterochromatic marks measured during four hour windows during the embryonic timecourse are inconsistent with transcriptional initiation-based repression of long zygotic genes (modENCODE Consortium 2012). A) Upstream coverage of ChIP-seq data from the active chromatin mark, H3K4me3, is significantly correlated with expression level across 4-hour developmental windows spanning the first 12 hours of embryogenesis, indicating that it is predictive of active transcription. Spearman correlation coefficients are strongest for short zygotic genes, as expected if long genes are limited from proper transcription by intron delay. Median base-level coverage of H3K4me3 among long zygotic genes does not increase over this interval, as would be predicted from a purely transcription initiation-based model of delay. Error bars indicate the standard error of the mean. B) At no time point is upstream coverage of the repressive chromatin mark, H3K27me3, significantly negatively correlated with expression of long zygotic genes. Furthermore, there is no significant pattern of decreasing upstream coverage as again would be consistent with a purely transcription initiation-based model of delay. ns, non-significant; *, $p < 0.05$; **, $p < 0.01$; ***, $p < 0.001$.
