# Supplementary material for: Transcript Length Mediates Developmental Timing of Gene Expression Across Drosophila
Source: Mol Biol Evol. 2014 Jul 28;31(11):2879–89. doi: 10.1093/molbev/msu226 (PMC4209130; doi:10.1093/molbev/msu226)
Supplement: Supplementary Data [file supp_msu226_Artieri_and_Fraser-Supplementary_Table_S1.pdf]

#Supplementary Table 1. Summary of all data analyzed in the manuscript. A description of column header

#FBR5_43_GENE	Gene name in FlyBase annotation release 5.43 (FBr5.43)
#FBGN	Corresponding unique FlyBase gene name
#KALINKA_GENE	Name given to the Gene in the data used for the syncytial timecourse (Kalinka et al.
#DMEL_LONGEST_	Unique FlyBase D. melanogaster transcript identifier for the longest transcript
#DMEL_LONGEST_	Length in bp of the longest D. melanogaster pre-mRNA transcript
#DMEL_PERC_GC	The percent GC of the mRNA produced from the longest transcript
#DMEL_LENGTH_5F	Number of exonic basepairs in the 5' most 1 kb of transcript.
#DMEL_LENGTH_3F	Number of exonic basepairs in the 3' most 1 kb of transcript.
#DMEL_OVERLAPPI	Does the D. melanogaster gene overlap any other gene in FBr5.43? (0 = no, 1 = ye
#DMEL_SINGLE_TS	Does the D. melanogaster gene have a single transcriptional start site according to
#DMEL_MATERNAL	Gene identified as maternally deposited (maternal) according to Tadros et al. 2007.
#DMEL_ZYGOTIC	Gene identified as zygotically expressed (zygotic) according to Tadros et al. 2007.
DE_RENZIS_MAT_O	Gene identified as purely maternal in origin according to De Renzis et al. 2007.
DE_RENZIS_ZYG_O	Gene identified as purely zygotic in origin according to De Renzis et al. 2007.
#EMBRYONIC_EXP	Gene expressed at > 5 RPKM during at least one time point during the embryonic ti
#SYNCYTIAL_EXP	Gene expressed at > 5 RPKM during at least one time point during the syncytial tim
#EMBRYONIC_[01...	Raw read counts for each of the 12 time points of the embryonic timecourse (Grave
#EMBRYONIC_[01...	Normalized RPKM for each of the 12 time points of the embryonic timecourse (Grav
#SYNCYTIAL_[C10...	Raw read counts for each of the 8 time points of the syncytial timecourse (Lott et al.
#SYNCYTIAL_[C10...	Normalized RPKM for each of the 8 time points of the syncytial timecourse (Lott et a
#SOLID_NONPOLYA	Raw read counts for the 5' end of each of the 12 time points of the non-poly-A selec
#SOLID_NONPOLYA	Normalized RPKM for the 5' end of each of the 12 time points of the non-poly-A sele
#SOLID_NONPOLYA	Raw read counts for the 3' end of each of the 12 time points of the non-poly-A selec
#SOLID_NONPOLYA	Normalized RPKM for the 3' end of each of the 12 time points of the non-poly-A sele
#SOLID_NONPOLYA	The ratio of 5':3' RPKM for each of the 12 time points of the non-poly-A selected SO
#ONE_TO_ONE_OR	Are there 1:1 orthologs with D. melanogaster in D. ananassae, D. pseudoobscura, a
#DANA_ORTHO	The 1:1 ortholog to in D. ananassae.
#DANA_FBGN	Unique FBgn for the D. ananassae ortholog.
#DANA_LONGEST_	The longest trasnscript of the ortholog in D. ananassae.
#DPSE_ORTHO	The 1:1 ortholog to in D. pseudoobscura.
#DPSE_FBGN	Unique FBgn for the D. pseudoobscura ortholog.
#DPSE_LONGEST_T	The longest trasnscript of the ortholog in D. pseudoobscura.
#DVIR_ORTHO	The 1:1 ortholog to in D. virilis.
#DVIR_FBGN	Unique FBgn for the D. virilis ortholog.
#DVIR_LONGEST_T	The longest trasnscript of the ortholog in D. virilis.
#SPECIES_EXPRES	Is the gene represented among the dataset used in the species timecourse (Kalinka
#SPECIES_DMEL_[T	Log10 transformed, normalized D. melanogaster expression levels from each of the
#SPECIES_DANA_[T	Log10 transformed, normalized D. ananassae expression levels from each of the 9
#SPECIES_DPSE_[T	Log10 transformed, normalized D. pseudoobscura expression levels from each of th
#SPECIES_DVIR_[TI	Log10 transformed, normalized D. virilis expression levels from each of the 9 time p
#DMEL_INTRONS	Number of introns according to the D. melanogaster FBr5.43 annotation
#DANA_INTRONS	Number of introns according to the D. ananassae FBr1.3 annotation
#DPSE_INTRONS	Number of introns according to the D. pseudoobscura FBr2.27 annotation
#DVIR_INTRONS	Number of introns according to the D. virilis FBr1.2 annotation
#ALL_COMMON_INT	Do all introns have detected orthologs among the four species analyzed? (0 = no; 1
#DMEL_ORTHO_INT	The total length in bp of the orthologous introns in D. melanogaster
#DANA_ORTHO_INT	The total length in bp of the orthologous introns in D. ananassae
#DPSE_ORTHO_INT	The total length in bp of the orthologous introns in D. pseudoobscura
#DVIR_ORTHO_INT_	The total length in bp of the orthologous introns in D. virilis
#INT_LEN_CCOV	The corrected coefficient of variation among the four species in terms of intron lengt
#MEAN_POLII_TSS_	Mean Pol II Enrichment at the transcriptional start site according to Chen et al. (201
#MEAN_POLII_GENI	Mean Pol II Enrichment in the gene body according to Chen et al. (2013)

FBR5_43_GENE	FBGN	KALINKA_GENE	DMEL_LONGEST_TRANSCRIPT
CG10000	FBgn0039596	NA	FBtr0085315

CG10007	FBgn0260744	NA	FBtr0082508
CG10009	FBgn0026400	Noa36	FBtr0085320
CG10011	FBgn0039590	NA	FBtr0085319
CG10013	FBgn0038012	NA	FBtr0082567
CG10016	FBgn0024244	NA	FBtr0077531
CG10026	FBgn0032785	CG10026	FBtr0081236
CG10032	FBgn0037493	NA	FBtr0081579
CG10035	FBgn0038028	CG10035	FBtr0082606
CG10051	FBgn0034437	NA	FBtr0086521
CG10052	FBgn0020617	Rx	FBtr0071518
CG10059	FBgn0037481	MAGE	FBtr0081606
CG10061	FBgn0011020	NA	FBtr0081569
CG10064	FBgn0035724	CG10064	FBtr0076937
CG10069	FBgn0034611	CG10069	FBtr0071591
CG1007	FBgn0000575	emc	FBtr0072578
CG10072	FBgn0261445	sgl	FBtr0076938
CG10078	FBgn0041194	Prat2	FBtr0076945
CG10079	FBgn0003731	Egfr	FBtr0071654
CG10082	FBgn0034644	CG10082	FBtr0071661
CG10083	FBgn0036372	NA	FBtr0075819
CG10084	FBgn0002044	NA	FBtr0081244
CG10086	FBgn0037517	CG10086	FBtr0081744
CG10089	FBgn0036369	NA	FBtr0075820
CG10090	FBgn0038018	NA	FBtr0082610
CG10091	FBgn0038020	NA	FBtr0082608
CG10093	FBgn0038007	NA	FBtr0082617
CG10095	FBgn0037993	NA	FBtr0082549
CG10096	FBgn0038032	NA	FBtr0082603
CG10097	FBgn0038033	NA	FBtr0100524
CG10101	FBgn0037501	NA	FBtr0081531
CG10103	FBgn0035715	NA	FBtr0076949
CG10104	FBgn0033933	NA	FBtr0087486
CG10106	FBgn0029506	Tsp42Ee	FBtr0086180
CG10107	FBgn0035713	CG10107	FBtr0076952
CG10108	FBgn0013725	phyl	FBtr0087516
CG10112	FBgn0033942	CG10112	FBtr0087514
CG10117	FBgn0020245	NA	FBtr0087495
CG10118	FBgn0005626	ple	FBtr0076957
CG10119	FBgn0010397	LamC	FBtr0087512
CG10121	FBgn0035710	SP1173	FBtr0076959
CG10122	FBgn0019938	NA	FBtr0087511
CG10125	FBgn0024177	zpg	FBtr0076983
CG10126	FBgn0038088	CG10126	FBtr0082686
CG10128	FBgn0003742	tra2	FBtr0089619
CG10129	FBgn0002926	NA	FBtr0076984
CG10131	FBgn0033949	NA	FBtr0087499
CG10137	FBgn0032800	NA	FBtr0081248
CG10138	FBgn0005778	NA	FBtr0071806
CG10139	FBgn0033951	NA	FBtr0087433
CG1014	FBgn0028567	NA	FBtr0072695
CG10143	FBgn0033952	NA	FBtr0087482
CG10145	FBgn0020269	mspo	FBtr0087477
CG10168	FBgn0039087	NA	FBtr0084418
CG10173	FBgn0035696	NA	FBtr0077010
CG10174	FBgn0032680	NA	FBtr0081063
CG10175	FBgn0039084	CG10175	FBtr0084422
CG10181	FBgn0004513	Mdr65	FBtr0077011

CG10182	FBgn0039091	NA	FBtr0084486
CG10185	FBgn0038397	NA	FBtr0083235
CG10192	FBgn0260634	NA	FBtr0089623
CG10202	FBgn0033969	NA	FBtr0087464
CG10203	FBgn0028554	NA	FBtr0079368
CG10205	FBgn0033970	NA	FBtr0087447
CG10207	FBgn0016684	NaPi-T	FBtr0087463
CG10209	FBgn0033971	CG10209	FBtr0087462
CG10210	FBgn0039117	tst	FBtr0084462
CG10211	FBgn0032685	CG10211	FBtr0081069
CG10212	FBgn0027783	SMC2	FBtr0087461
CG10214	FBgn0039115	NA	FBtr0084463
CG10215	FBgn0028434	NA	FBtr0087448
CG10217	FBgn0039113	NA	FBtr0084465
CG10219	FBgn0039112	NA	FBtr0084466
CG10223	FBgn0003732	NA	FBtr0081287
CG10226	FBgn0035695	CG10226	FBtr0077012
CG10234	FBgn0024230	Hs2st	FBtr0081288
CG10236	FBgn0002526	LanA	FBtr0077014
CG10244	FBgn0022800	Cad96Ca	FBtr0084874
CG10252	FBgn0039104	NA	FBtr0084475
CG10254	FBgn0027512	CG10254	FBtr0084477
CG1028	FBgn0260642	Antp	FBtr0081652
CG10286	FBgn0037439	NA	FBtr0081717
CG10289	FBgn0035688	NA	FBtr0077019
CG10296	FBgn0037436	NA	FBtr0081713
CG10302	FBgn0032679	NA	FBtr0081087
CG10303	FBgn0037412	NA	FBtr0078615
CG10305	FBgn0261597	NA	FBtr0081090
CG10306	FBgn0034654	CG10306	FBtr0071670
CG10307	FBgn0034655	NA	FBtr0071671
CG10311	FBgn0038420	NA	FBtr0083324
CG10318	FBgn0034650	NC2alpha	FBtr0071667
CG10321	FBgn0034643	NA	FBtr0071660
CG10324	FBgn0038454	CG10324	FBtr0083356
CG10326	FBgn0038453	NA	FBtr0083336
CG10328	FBgn0015520	NA	FBtr0083335
CG10333	FBgn0032690	NA	FBtr0081072
CG10337	FBgn0032805	NA	FBtr0081276
CG10338	FBgn0032700	CG10338	FBtr0081096
CG10340	FBgn0022344	NA	FBtr0083358
CG10343	FBgn0032703	NA	FBtr0081098
CG10347	FBgn0030342	NA	FBtr0073603
CG10354	FBgn0031868	NA	FBtr0079345
CG10360	FBgn0003231	ref(2)P	FBtr0081253
CG10361	FBgn0036208	CG10361	FBtr0076084
CG10362	FBgn0030358	NA	FBtr0073590
CG10363	FBgn0041180	TepIV	FBtr0081254
CG10365	FBgn0039109	NA	FBtr0084446
CG10371	FBgn0039111	Plip	FBtr0084453
CG10372	FBgn0025608	Faf	FBtr0081126
CG10374	FBgn0039114	Lsd-1	FBtr0084455
CG10376	FBgn0032702	NA	FBtr0081128
CG10382	FBgn0025878	wrapper	FBtr0071821
CG10383	FBgn0032699	NA	FBtr0089628
CG10385	FBgn0005617	NA	FBtr0081130
CG10391	FBgn0032693	Cyp310a1	FBtr0081077

CG10392	FBgn0261403	Ogt	FBtr0086086
CG10393	FBgn0003270	NA	FBtr0081081
CG10396	FBgn0033020	NA	FBtr0086089
CG10413	FBgn0032689	CG10413	FBtr0081080
CG10414	FBgn0032691	CG10414	FBtr0081079
CG10417	FBgn0033021	CG10417	FBtr0086091
CG10422	FBgn0000158	NA	FBtr0084869
CG10426	FBgn0036273	CG10426	FBtr0076007
CG10435	FBgn0037539	NA	FBtr0081820
CG10436	FBgn0011279	NA	FBtr0075955
CG1044	FBgn0016794	NA	FBtr0072927
CG10444	FBgn0034494	NA	FBtr0086305
CG10446	FBgn0032741	Side	FBtr0081141
CG10449	FBgn0002022	Catsup	FBtr0081143
CG10463	FBgn0032819	NA	FBtr0081290
CG10465	FBgn0033017	CG10465	FBtr0086094
CG10466	FBgn0032822	NA	FBtr0081291
CG10470	FBgn0032746	NA	FBtr0081146
CG10472	FBgn0035670	NA	FBtr0077066
CG10473	FBgn0263198	CG10473	FBtr0081145
CG10476	FBgn0034426	NA	FBtr0086562
CG10477	FBgn0035661	NA	FBtr0077068
CG10479	FBgn0035656	CG10479	FBtr0077070
CG10481	FBgn0032827	NA	FBtr0081312
CG10488	FBgn0000625	eyg	FBtr0075979
CG10489	FBgn0035644	NA	FBtr0077081
CG1049	FBgn0041342	Cct1	FBtr0072739
CG10493	FBgn0032749	NA	FBtr0081149
CG10494	FBgn0034634	NA	FBtr0071699
CG10501	FBgn0000075	NA	FBtr0081154
CG10512	FBgn0037057	CG10512	FBtr0078297
CG10516	FBgn0036549	NA	FBtr0075512
CG10522	FBgn0002466	NA	FBtr0075970
CG10527	FBgn0034583	CG10527	FBtr0071541
CG10529	FBgn0020640	NA	FBtr0077056
CG10530	FBgn0020638	NA	FBtr0077058
CG10533	FBgn0020639	NA	FBtr0077057
CG10534	FBgn0020637	NA	FBtr0077059
CG10535	FBgn0037926	CG10535	FBtr0082420
CG10542	FBgn0086694	NA	FBtr0077116
CG10545	FBgn0001105	Gbeta13F	FBtr0074110
CG10561	FBgn0002036	NA	FBtr0081155
CG10563	FBgn0086445	CG10563	FBtr0081156
CG10572	FBgn0015618	Cdk8	FBtr0076369
CG10573	FBgn0020294	ko	FBtr0078324
CG10580	FBgn0011591	fng	FBtr0078267
CG10583	FBgn0035627	Sse	FBtr0077125
CG10589	FBgn0037035	NA	FBtr0078266
CG1059	FBgn0087013	NA	FBtr0078860
CG10591	FBgn0035621	CG10591	FBtr0077128
CG10593	FBgn0016122	Acer	FBtr0079685
CG10596	FBgn0015766	Msr-110	FBtr0077131
CG10601	FBgn0014343	NA	FBtr0075911
CG10604	FBgn0000529	NA	FBtr0089320
CG10605	FBgn0015919	NA	FBtr0075909
CG10610	FBgn0028436	ECSIT	FBtr0078647
CG10612	FBgn0037322	NA	FBtr0078797

CG10617	FBgn0261085	NA	FBtr0073767
CG10619	FBgn0003896	tup	FBtr0081112
CG10620	FBgn0036299	Tsf2	FBtr0075966
CG10626	FBgn0035610	NA	FBtr0077149
CG10627	FBgn0036298	CG10627	FBtr0075967
CG10628	FBgn0032818	CG10628	FBtr0081323
CG1063	FBgn0010051	NA	FBtr0078686
CG10631	FBgn0032817	CG10631	FBtr0081324
CG10633	FBgn0035604	NA	FBtr0077153
CG10639	FBgn0032729	NA	FBtr0081186
CG10641	FBgn0032731	CG10641	FBtr0081185
CG10642	FBgn0004380	NA	FBtr0077157
CG10645	FBgn0016031	lama	FBtr0077160
CG10646	FBgn0036292	CG10646	FBtr0075972
CG10653	FBgn0001202	hk	FBtr0081180
CG10654	FBgn0036294	CG10654	FBtr0075971
CG10655	FBgn0002021	NA	FBtr0081177
CG10657	FBgn0036289	CG10657	FBtr0075975
CG10667	FBgn0022772	NA	FBtr0088959
CG10670	FBgn0263831	Gen	FBtr0077176
CG10671	FBgn0035586	NA	FBtr0077177
CG10683	FBgn0004400	rhi	FBtr0086897
CG10686	FBgn0041775	NA	FBtr0075959
CG10695	FBgn0029878	NA	FBtr0070945
CG10704	FBgn0036285	toe	FBtr0075980
CG10710	FBgn0036377	CG10710	FBtr0075817
CG10712	FBgn0044324	Chro	FBtr0078568
CG1072	FBgn0013751	NA	FBtr0073156
CG10741	FBgn0036373	NA	FBtr0075811
CG10743	FBgn0036376	NA	FBtr0075813
CG10748	FBgn0036327	NA	FBtr0075900
CG10749	FBgn0036328	NA	FBtr0075871
CG10752	FBgn0036325	NA	FBtr0075903
CG10753	FBgn0261933	snRNP69D	FBtr0075952
CG10759	FBgn0030016	NA	FBtr0071186
CG10761	FBgn0030015	NA	FBtr0071187
CG1077	FBgn0037405	NA	FBtr0078587
CG10776	FBgn0024179	NA	FBtr0073279
CG10777	FBgn0029979	NA	FBtr0071132
CG10778	FBgn0029980	CG10778	FBtr0071131
CG1078	FBgn0037255	NA	FBtr0078928
CG10781	FBgn0002933	NA	FBtr0070523
CG10788	FBgn0010295	NA	FBtr0070539
CG10789	FBgn0010296	NA	FBtr0070538
CG10790	FBgn0003086	NA	FBtr0070537
CG10793	FBgn0029656	NA	FBtr0070536
CG10795	FBgn0034626	NA	FBtr0071609
CG10798	FBgn0262656	dm	FBtr0070525
CG10799	FBgn0033821	NA	FBtr0087748
CG10800	FBgn0017551	NA	FBtr0079342
CG10802	FBgn0029664	CG10802	FBtr0070568
CG10803	FBgn0029666	NA	FBtr0070569
CG10805	FBgn0086451	NA	FBtr0079343
CG10806	FBgn0031865	NA	FBtr0079374
CG10808	FBgn0033876	NA	FBtr0087645
CG10814	FBgn0033830	NA	FBtr0087720
CG10837	FBgn0020660	NA	FBtr0113681

CG1084	FBgn0037240	Cont	FBtr0078917
CG10849	FBgn0035471	NA	FBtr0073159
CG10850	FBgn0041147	NA	FBtr0073160
CG10852	FBgn0015585	NA	FBtr0073182
CG10853	FBgn0035478	NA	FBtr0073164
CG10864	FBgn0038621	NA	FBtr0083654
CG10877	FBgn0038804	NA	FBtr0083950
CG1088	FBgn0015324	NA	FBtr0078701
CG10882	FBgn0262126	CG10882	FBtr0077810
CG10887	FBgn0038773	NA	FBtr0083864
CG10888	FBgn0003249	NA	FBtr0083876
CG10890	FBgn0002887	NA	FBtr0005674
CG10907	FBgn0036207	CG10907	FBtr0076029
CG1091	FBgn0037470	CG1091	FBtr0081640
CG10910	FBgn0034289	NA	FBtr0089630
CG10911	FBgn0034295	NA	FBtr0086808
CG10912	FBgn0034296	NA	FBtr0086807
CG10914	FBgn0034307	NA	FBtr0086745
CG10915	FBgn0034308	NA	FBtr0086746
CG10916	FBgn0034312	NA	FBtr0086749
CG10920	FBgn0029963	NA	FBtr0071150
CG10923	FBgn0004379	Klp67A	FBtr0076499
CG10930	FBgn0003140	NA	FBtr0086817
CG10932	FBgn0029969	NA	FBtr0071143
CG10936	FBgn0034253	CG10936	FBtr0086895
CG10939	FBgn0010620	SIP1	FBtr0086909
CG10949	FBgn0032858	CG10949	FBtr0081367
CG10951	FBgn0045980	NA	FBtr0084737
CG10953	FBgn0034204	CG10953	FBtr0086997
CG10954	FBgn0032859	Arc-p34	FBtr0081358
CG10955	FBgn0034722	NA	FBtr0071820
CG10960	FBgn0036316	CG10960	FBtr0075942
CG10962	FBgn0030073	NA	FBtr0071262
CG10970	FBgn0030078	NA	FBtr0071356
CG10971	FBgn0036309	NA	FBtr0075949
CG10972	FBgn0034730	NA	FBtr0071830
CG10973	FBgn0036306	CG10973	FBtr0075950
CG10975	FBgn0014007	Ptp69D	FBtr0075913
CG10979	FBgn0037379	NA	FBtr0078631
CG1098	FBgn0027497	Madm	FBtr0078722
CG10981	FBgn0037384	CG10981	FBtr0078638
CG10984	FBgn0036305	CG10984	FBtr0075921
CG10992	FBgn0030521	CG10992	FBtr0073838
CG10993	FBgn0030524	CG10993	FBtr0073837
CG10997	FBgn0030529	Clic	FBtr0073835
CG1101	FBgn0010774	NA	FBtr0081642
CG11010	FBgn0036319	NA	FBtr0075935
CG11012	FBgn0026756	NA	FBtr0081372
CG11015	FBgn0031830	CG11015	FBtr0079288
CG11025	FBgn0028372	NA	FBtr0086473
CG11027	FBgn0013749	NA	FBtr0089192
CG11029	FBgn0031735	NA	FBtr0079136
CG11044	FBgn0034484	NA	FBtr0086313
CG11048	FBgn0034487	CG11048	FBtr0086316
CG11051	FBgn0040813	NA	FBtr0075839
CG11055	FBgn0034491	NA	FBtr0086272
CG11059	FBgn0039928	NA	FBtr0089207

CG11060	FBgn0033149	NA	FBtr0089045
CG11062	FBgn0024913	NA	FBtr0089209
CG11064	FBgn0087002	NA	FBtr0089188
CG11066	FBgn0033033	scarface	FBtr0086061
CG1107	FBgn0037218	auxillin	FBtr0078965
CG11084	FBgn0003090	NA	FBtr0089044
CG1109	FBgn0046222	CG1109	FBtr0078713
CG11094	FBgn0000504	dsx	FBtr0081759
CG11095	FBgn0030528	NA	FBtr0073827
CG11099	FBgn0034485	NA	FBtr0086315
CG11101	FBgn0003174	NA	FBtr0088962
CG11103	FBgn0030522	CG11103	FBtr0100218
CG11106	FBgn0030280	NA	FBtr0073446
CG11107	FBgn0033160	CG11107	FBtr0088966
CG11109	FBgn0037200	CG11109	FBtr0078553
CG11115	FBgn0037202	Ssl1	FBtr0078554
CG11121	FBgn0003460	so	FBtr0088970
CG11122	FBgn0030266	CG11122	FBtr0073481
CG11128	FBgn0037203	slif	FBtr0078555
CG11131	FBgn0037204	NA	FBtr0078558
CG11133	FBgn0037205	NA	FBtr0078559
CG11145	FBgn0033168	NA	FBtr0089004
CG11153	FBgn0039938	NA	FBtr0100600
CG11155	FBgn0039927	NA	FBtr0089194
CG11160	FBgn0030257	NA	FBtr0073391
CG11162	FBgn0030509	NA	FBtr0073811
CG11163	FBgn0025693	CG11163	FBtr0086054
CG11164	FBgn0030507	CG11164	FBtr0073809
CG11178	FBgn0030499	NA	FBtr0073804
CG11180	FBgn0034528	NA	FBtr0086254
CG11182	FBgn0025334	NA	FBtr0072224
CG11186	FBgn0019650	NA	FBtr0089185
CG11191	FBgn0033249	CG11191	FBtr0088868
CG11194	FBgn0027788	NA	FBtr0088869
CG11196	FBgn0033248	NA	FBtr0088870
CG11199	FBgn0046704	Liprin-alpha	FBtr0079301
CG11202	FBgn0021767	org-1	FBtr0071244
CG11207	FBgn0030241	NA	FBtr0073410
CG11208	FBgn0034488	CG11208	FBtr0086322
CG11209	FBgn0034489	NA	FBtr0086321
CG1121	FBgn0015576	NA	FBtr0081553
CG11218	FBgn0034470	NA	FBtr0086477
CG11221	FBgn0031855	NA	FBtr0079303
CG11231	FBgn0039939	NA	FBtr0089242
CG11237	FBgn0034452	NA	FBtr0086501
CG1124	FBgn0037290	CG1124	FBtr0078841
CG11246	FBgn0037121	Rpb8	FBtr0078432
CG11248	FBgn0037117	NA	FBtr0078431
CG11249	FBgn0037115	NA	FBtr0078429
CG11258	FBgn0036335	NA	FBtr0075897
CG11259	FBgn0036333	NA	FBtr0075898
CG11267	FBgn0036334	CG11267	FBtr0075875
CG11268	FBgn0036336	CG11268	FBtr0075876
CG11269	FBgn0034700	NA	FBtr0071765
CG11276	FBgn0011284	NA	FBtr0075884
CG11278	FBgn0036341	NA	FBtr0075886
CG11279	FBgn0036342	NA	FBtr0075887

CG11281	FBgn0086916	NA	FBtr0075827
CG11284	FBgn0030056	NA	FBtr0071222
CG11290	FBgn0034975	NA	FBtr0072250
CG11291	FBgn0034713	NA	FBtr0071773
CG11298	FBgn0034721	NA	FBtr0071819
CG1130	FBgn0004880	scrt	FBtr0073165
CG11303	FBgn0020372	TM4SF	FBtr0100255
CG11312	FBgn0011674	insc	FBtr0071562
CG11317	FBgn0039816	CG11317	FBtr0085741
CG11318	FBgn0039818	NA	FBtr0085739
CG11319	FBgn0031835	NA	FBtr0089641
CG11323	FBgn0031854	NA	FBtr0079305
CG11329	FBgn0031848	NA	FBtr0079319
CG1133	FBgn0003002	opa	FBtr0078836
CG11330	FBgn0000351	NA	FBtr0079320
CG11331	FBgn0028990	Spn27A	FBtr0079321
CG11348	FBgn0000038	NA	FBtr0073299
CG1135	FBgn0263832	CG1135	FBtr0073202
CG11354	FBgn0026411	NA	FBtr0071279
CG1136	FBgn0035490	CG1136	FBtr0073203
CG11362	FBgn0034737	NA	FBtr0071836
CG1137	FBgn0037454	NA	FBtr0081705
CG11370	FBgn0037181	CG11370	FBtr0078522
CG11373	FBgn0040679	NA	FBtr0078657
CG11375	FBgn0039227	NA	FBtr0084740
CG11376	FBgn0031216	CG11376	FBtr0078103
CG1138	FBgn0037456	NA	FBtr0081703
CG11380	FBgn0040359	CG11380	FBtr0070201
CG11381	FBgn0029568	NA	FBtr0070203
CG11382	FBgn0040367	NA	FBtr0070205
CG11388	FBgn0034959	NA	FBtr0072162
CG11390	FBgn0011695	PebIII	FBtr0072164
CG11395	FBgn0034200	NA	FBtr0086987
CG11396	FBgn0037022	CG11396	FBtr0078276
CG11397	FBgn0015391	NA	FBtr0080935
CG11399	FBgn0037021	NA	FBtr0078277
CG1140	FBgn0035298	CG1140	FBtr0072843
CG11401	FBgn0037170	Trxr-2	FBtr0078514
CG11404	FBgn0037169	NA	FBtr0078513
CG11407	FBgn0038733	CG11407	FBtr0083790
CG11417	FBgn0024364	CG11417	FBtr0070212
CG11420	FBgn0000826	png	FBtr0070234
CG11421	FBgn0011281	NA	FBtr0078656
CG11422	FBgn0010403	NA	FBtr0078655
CG11437	FBgn0037165	CG11437	FBtr0078505
CG11438	FBgn0037164	CG11438	FBtr0078504
CG11440	FBgn0037163	CG11440	FBtr0078508
CG11448	FBgn0024985	CG11448	FBtr0070225
CG11449	FBgn0037162	NA	FBtr0078503
CG11453	FBgn0038734	CG11453	FBtr0083791
CG11458	FBgn0040637	NA	FBtr0078265
CG11459	FBgn0037396	CG11459	FBtr0078623
CG1146	FBgn0035346	CG1146	FBtr0072923
CG11466	FBgn0038037	Cyp9f2	FBtr0082598
CG11482	FBgn0011659	NA	FBtr0088790
CG1149	FBgn0015770	NA	FBtr0081585
CG11495	FBgn0024194	rasp	FBtr0073029

CG11501	FBgn0039666	NA	FBtr0085426
CG1151	FBgn0027527	Osi6	FBtr0078596
CG11522	FBgn0039857	NA	FBtr0085805
CG11525	FBgn0039858	NA	FBtr0085802
CG11527	FBgn0011722	Tig	FBtr0079275
CG1154	FBgn0037419	Osi12	FBtr0078602
CG1155	FBgn0040279	Osi14	FBtr0078606
CG11550	FBgn0039864	NA	FBtr0085867
CG11551	FBgn0025776	NA	FBtr0075668
CG11552	FBgn0036465	NA	FBtr0075669
CG11560	FBgn0036249	NA	FBtr0075993
CG11561	FBgn0003444	smo	FBtr0078129
CG11562	FBgn0031247	CG11562	FBtr0078130
CG11566	FBgn0031159	NA	FBtr0100609
CG11567	FBgn0015623	Cpr	FBtr0079250
CG1157	FBgn0037424	Osi15	FBtr0078607
CG11575	FBgn0039879	NA	FBtr0085846
CG11576	FBgn0039882	CG11576	FBtr0085852
CG11579	FBgn0000117	NA	FBtr0089989
CG11581	FBgn0030540	NA	FBtr0073892
CG11586	FBgn0035520	CG11586	FBtr0073243
CG11588	FBgn0036221	NA	FBtr0076038
CG11590	FBgn0030545	NA	FBtr0073899
CG11591	FBgn0035491	NA	FBtr0073281
CG11592	FBgn0025686	NA	FBtr0078134
CG11601	FBgn0031244	NA	FBtr0078137
CG11607	FBgn0001170	H2.0	FBtr0079227
CG11614	FBgn0002945	NA	FBtr0075039
CG11619	FBgn0036836	NA	FBtr0075046
CG11624	FBgn0003943	NA	FBtr0073178
CG11630	FBgn0032964	NA	FBtr0085909
CG11648	FBgn0000015	NA	FBtr0083384
CG11650	FBgn0002531	NA	FBtr0088763
CG11655	FBgn0030638	NA	FBtr0074031
CG11658	FBgn0036196	CG11658	FBtr0076127
CG11660	FBgn0036187	CG11660	FBtr0076134
CG11665	FBgn0033028	CG11665	FBtr0086072
CG11671	FBgn0037562	NA	FBtr0081829
CG11672	FBgn0037563	NA	FBtr0081865
CG11674	FBgn0030551	NA	FBtr0073926
CG11678	FBgn0011741	Actr13E	FBtr0074203
CG1168	FBgn0041707	NA	FBtr0301640
CG11680	FBgn0002774	NA	FBtr0086030
CG1169	FBgn0037428	Osi18	FBtr0078611
CG11694	FBgn0037571	NA	FBtr0081859
CG11696	FBgn0030314	NA	FBtr0073512
CG11698	FBgn0037572	NA	FBtr0081833
CG11699	FBgn0030311	CG11699	FBtr0073510
CG11709	FBgn0030310	NA	FBtr0073509
CG11711	FBgn0259481	NA	FBtr0089332
CG11715	FBgn0030304	Cyp4g15	FBtr0073503
CG11720	FBgn0003373	NA	FBtr0076096
CG11732	FBgn0037589	NA	FBtr0081849
CG11735	FBgn0037590	NA	FBtr0081900
CG11739	FBgn0037239	NA	FBtr0078914
CG11742	FBgn0037594	NA	FBtr0081872
CG11752	FBgn0030292	NA	FBtr0073452

CG11771	FBgn0039252	CG11771	FBtr0084748
CG11775	FBgn0037630	NA	FBtr0081917
CG11780	FBgn0039258	NA	FBtr0084755
CG11781	FBgn0039259	CG11781	FBtr0084797
CG11783	FBgn0015240	NA	FBtr0084756
CG11785	FBgn0045866	NA	FBtr0084758
CG11788	FBgn0034495	NA	FBtr0086277
CG11790	FBgn0039265	CG11790	FBtr0084761
CG11797	FBgn0034468	Obp56a	FBtr0086476
CG11811	FBgn0036099	CG11811	FBtr0076265
CG11816	FBgn0030495	NA	FBtr0073785
CG11822	FBgn0031261	NA	FBtr0078065
CG11840	FBgn0031260	NA	FBtr0078080
CG11852	FBgn0039297	NA	FBtr0084809
CG11853	FBgn0039298	NA	FBtr0084810
CG11865	FBgn0028947	NA	FBtr0080753
CG11880	FBgn0039637	NA	FBtr0085372
CG11885	FBgn0031253	NA	FBtr0078089
CG11886	FBgn0041186	NA	FBtr0085409
CG11888	FBgn0028692	NA	FBtr0085385
CG11889	FBgn0039308	NA	FBtr0100614
CG11892	FBgn0039313	NA	FBtr0089645
CG11896	FBgn0038488	CG11896	FBtr0083414
CG11897	FBgn0039644	NA	FBtr0085387
CG11899	FBgn0014427	CG11899	FBtr0085390
CG11907	FBgn0031250	Ent1	FBtr0078094
CG11910	FBgn0039332	CG11910	FBtr0084846
CG11921	FBgn0004897	NA	FBtr0084770
CG11922	FBgn0004898	fd96Cb	FBtr0084771
CG11929	FBgn0031620	NA	FBtr0077377
CG11941	FBgn0026175	NA	FBtr0074797
CG11942	FBgn0031074	NA	FBtr0074806
CG11956	FBgn0263236	SP1029	FBtr0085399
CG11970	FBgn0027503	NA	FBtr0081959
CG11971	FBgn0260243	NA	FBtr0082012
CG11975	FBgn0037648	NA	FBtr0081960
CG11979	FBgn0033571	NA	FBtr0088237
CG11980	FBgn0037652	CG11980	FBtr0081962
CG11987	FBgn0015014	NA	FBtr0082005
CG11992	FBgn0014018	Rel	FBtr0089338
CG12000	FBgn0250746	CG12000	FBtr0078805
CG12002	FBgn0011828	Pxn	FBtr0072954
CG12003	FBgn0035234	NA	FBtr0072744
CG12005	FBgn0037301	Mms19	FBtr0078778
CG12007	FBgn0037293	CG12007	FBtr0078842
CG12010	FBgn0035443	NA	FBtr0073074
CG12018	FBgn0027903	NA	FBtr0072775
CG12019	FBgn0011573	NA	FBtr0072777
CG12020	FBgn0035273	NA	FBtr0072778
CG12026	FBgn0262624	CG12026	FBtr0072837
CG12028	FBgn0000449	NA	FBtr0073205
CG12031	FBgn0035145	NA	FBtr0072554
CG12045	FBgn0039805	NA	FBtr0085710
CG12048	FBgn0039675	NA	FBtr0085448
CG12051	FBgn0000043	NA	FBtr0086029
CG12055	FBgn0001091	NA	FBtr0088903
CG12056	FBgn0030099	NA	FBtr0071305

CG12057	FBgn0030098	NA	FBtr0071304
CG12061	FBgn0040031	NA	FBtr0113682
CG12070	FBgn0000416	NA	FBtr0085713
CG12072	FBgn0011739	NA	FBtr0085720
CG12073	FBgn0004573	5-HT7	FBtr0085746
CG12076	FBgn0027616	NA	FBtr0073078
CG12084	FBgn0043458	CG12084	FBtr0072711
CG12090	FBgn0035227	NA	FBtr0072703
CG12091	FBgn0035228	CG12091	FBtr0072831
CG12093	FBgn0035372	NA	FBtr0073015
CG12099	FBgn0035232	CG12099	FBtr0072828
CG12101	FBgn0015245	NA	FBtr0073434
CG12105	FBgn0035241	NA	FBtr0072824
CG12106	FBgn0030100	NA	FBtr0071306
CG12109	FBgn0030054	NA	FBtr0071228
CG12112	FBgn0030048	NA	FBtr0071233
CG12113	FBgn0026679	NA	FBtr0071234
CG12114	FBgn0086362	CG12114	FBtr0085760
CG12115	FBgn0030097	NA	FBtr0071341
CG12117	FBgn0014032	NA	FBtr0071241
CG12118	FBgn0030101	CG12118	FBtr0071339
CG12119	FBgn0030102	NA	FBtr0071336
CG12120	FBgn0086367	NA	FBtr0071335
CG12121	FBgn0030109	NA	FBtr0071334
CG12124	FBgn0260789	NA	FBtr0071332
CG12128	FBgn0033473	NA	FBtr0088394
CG12131	FBgn0027619	Adam	FBtr0088387
CG12132	FBgn0040236	NA	FBtr0071326
CG12133	FBgn0033469	NA	FBtr0088386
CG12134	FBgn0033471	NA	FBtr0088388
CG12135	FBgn0040235	NA	FBtr0071324
CG12147	FBgn0037325	NA	FBtr0078793
CG12149	FBgn0040234	NA	FBtr0071321
CG12153	FBgn0022786	NA	FBtr0071070
CG12157	FBgn0016041	Tom40	FBtr0071074
CG12158	FBgn0040775	NA	FBtr0088557
CG12164	FBgn0033158	NA	FBtr0088963
CG12165	FBgn0260991	NA	FBtr0088961
CG12172	FBgn0024294	Spn43Aa	FBtr0089041
CG12177	FBgn0030510	CG12177	FBtr0073846
CG12181	FBgn0003374	NA	FBtr0070524
CG12189	FBgn0035150	NA	FBtr0072557
CG12191	FBgn0035170	dpr20	FBtr0072594
CG12192	FBgn0034827	NA	FBtr0071968
CG12193	FBgn0026398	NA	FBtr0077873
CG12203	FBgn0031021	CG12203	FBtr0074712
CG12205	FBgn0000227	Bsg25A	FBtr0077423
CG12206	FBgn0029662	CG12206	FBtr0070528
CG12212	FBgn0003053	peb	FBtr0070680
CG12217	FBgn0003139	NA	FBtr0070921
CG12218	FBgn0026206	mei-P26	FBtr0071303
CG12219	FBgn0043796	NA	FBtr0070925
CG12220	FBgn0039835	mRpL32	FBtr0085759
CG12225	FBgn0028982	NA	FBtr0070896
CG12227	FBgn0034863	NA	FBtr0072022
CG12232	FBgn0010223	NA	FBtr0075354
CG12234	FBgn0031051	NA	FBtr0074759

CG12235	FBgn0031050	NA	FBtr0074760
CG12236	FBgn0029822	CG12236	FBtr0070826
CG12237	FBgn0031048	NA	FBtr0074761
CG12238	FBgn0087008	l(1)G0084	FBtr0074762
CG12241	FBgn0038304	NA	FBtr0083051
CG12242	FBgn0010041	NA	FBtr0082572
CG12244	FBgn0261524	NA	FBtr0073720
CG12245	FBgn0014179	gcm	FBtr0079855
CG12249	FBgn0021776	mira	FBtr0083847
CG12252	FBgn0035026	NA	FBtr0072342
CG12255	FBgn0036618	NA	FBtr0075383
CG12276	FBgn0029512	Aos1	FBtr0082593
CG12283	FBgn0015399	NA	FBtr0080401
CG12284	FBgn0260635	th	FBtr0075500
CG12287	FBgn0004394	pdm2	FBtr0080392
CG12296	FBgn0013469	klu	FBtr0076247
CG12297	FBgn0038928	NA	FBtr0084176
CG12298	FBgn0003545	NA	FBtr0086881
CG12303	FBgn0036083	NA	FBtr0076294
CG12306	FBgn0003124	NA	FBtr0074839
CG12309	FBgn0033542	NA	FBtr0088215
CG12311	FBgn0086368	Pomt2	FBtr0070139
CG12313	FBgn0035124	NA	FBtr0072507
CG12316	FBgn0036483	CG12316	FBtr0075651
CG1232	FBgn0003710	tipE	FBtr0073219
CG12323	FBgn0029134	NA	FBtr0088225
CG12324	FBgn0033555	NA	FBtr0088226
CG12325	FBgn0033557	NA	FBtr0088227
CG12330	FBgn0035686	NA	FBtr0076996
CG12333	FBgn0038617	NA	FBtr0083655
CG12334	FBgn0038539	NA	FBtr0083533
CG12338	FBgn0033543	CG12338	FBtr0088256
CG12340	FBgn0027499	NA	FBtr0088253
CG12341	FBgn0033550	CG12341	FBtr0088251
CG12342	FBgn0086898	NA	FBtr0088250
CG12344	FBgn0033558	NA	FBtr0088246
CG12346	FBgn0017414	NA	FBtr0088239
CG12351	FBgn0010358	NA	FBtr0088124
CG12358	FBgn0038100	NA	FBtr0082693
CG12359	FBgn0027603	NA	FBtr0074693
CG1236	FBgn0037370	CG1236	FBtr0078721
CG12362	FBgn0036082	NA	FBtr0076281
CG12363	FBgn0024432	NA	FBtr0083575
CG12366	FBgn0033901	O-fut1	FBtr0087520
CG12367	FBgn0033686	NA	FBtr0087984
CG12369	FBgn0010238	Lac	FBtr0087933
CG12372	FBgn0028683	NA	FBtr0087840
CG12374	FBgn0033774	CG12374	FBtr0087854
CG12391	FBgn0033581	CG12391	FBtr0088205
CG12396	FBgn0022069	NA	FBtr0080473
CG12398	FBgn0030596	NA	FBtr0073976
CG12399	FBgn0011648	Mad	FBtr0077616
CG12400	FBgn0031505	CG12400	FBtr0077617
CG12403	FBgn0020368	Vha68-1	FBtr0080429
CG12404	FBgn0032465	NA	FBtr0080430
CG12405	FBgn0033520	NA	FBtr0088261
CG1241	FBgn0044452	NA	FBtr0072970

CG12423	FBgn0040038	NA	FBtr0113686
CG12424	FBgn0033987	CG12424	FBtr0087409
CG12439	FBgn0032094	NA	FBtr0079767
CG12446	FBgn0031201	NA	FBtr0077343
CG12470	FBgn0040371	NA	FBtr0070069
CG12483	FBgn0040688	NA	FBtr0072540
CG12486	FBgn0036541	NA	FBtr0075533
CG12487	FBgn0040487	NA	FBtr0075673
CG12489	FBgn0260866	Dnr1	FBtr0071837
CG12496	FBgn0040385	NA	FBtr0070462
CG12499	FBgn0038968	NA	FBtr0084295
CG12501	FBgn0034473	NA	FBtr0086344
CG12506	FBgn0031276	NA	FBtr0078003
CG12519	FBgn0036872	NA	FBtr0074930
CG1252	FBgn0004782	NA	FBtr0081692
CG12521	FBgn0040816	NA	FBtr0076074
CG12522	FBgn0036131	NA	FBtr0076209
CG12526	FBgn0036009	NA	FBtr0076375
CG12529	FBgn0004057	NA	FBtr0074748
CG12532	FBgn0010380	NA	FBtr0074789
CG12540	FBgn0030570	NA	FBtr0073929
CG12558	FBgn0039599	NA	FBtr0085311
CG12559	FBgn0003256	NA	FBtr0113702
CG1258	FBgn0011692	pav	FBtr0073227
CG12581	FBgn0037213	CG12581	FBtr0078962
CG12590	FBgn0037294	NA	FBtr0078773
CG12592	FBgn0037811	NA	FBtr0082233
CG12602	FBgn0032373	NA	FBtr0080255
CG12605	FBgn0035481	CG12605	FBtr0073166
CG12609	FBgn0030952	NA	FBtr0074605
CG12611	FBgn0030898	NA	FBtr0074591
CG12620	FBgn0032626	NA	FBtr0080953
CG12622	FBgn0030297	NA	FBtr0073550
CG1263	FBgn0261602	RpL8	FBtr0072925
CG12630	FBgn0028979	NA	FBtr0089377
CG12640	FBgn0030202	NA	FBtr0071464
CG12643	FBgn0040942	NA	FBtr0071481
CG12644	FBgn0040940	NA	FBtr0071434
CG12653	FBgn0000233	NA	FBtr0071379
CG12659	FBgn0040929	NA	FBtr0071229
CG12661	FBgn0030071	NA	FBtr0071265
CG12662	FBgn0030074	NA	FBtr0071264
CG12663	FBgn0029961	NA	FBtr0071075
CG12664	FBgn0030090	NA	FBtr0071349
CG12665	FBgn0030103	NA	FBtr0071307
CG12672	FBgn0030886	NA	FBtr0074539
CG12676	FBgn0000547	ed	FBtr0077465
CG12697	FBgn0030715	NA	FBtr0074133
CG12698	FBgn0030721	NA	FBtr0074139
CG12700	FBgn0026174	NA	FBtr0074796
CG12702	FBgn0031070	NA	FBtr0074809
CG12703	FBgn0031069	NA	FBtr0074795
CG1271	FBgn0035392	NA	FBtr0072989
CG12713	FBgn0036536	NA	FBtr0075506
CG12729	FBgn0029816	NA	FBtr0070819
CG1273	FBgn0035522	CG1273	FBtr0073231
CG12730	FBgn0029771	NA	FBtr0070780

CG12736	FBgn0033184	NA	FBtr0088981
CG12743	FBgn0003023	NA	FBtr0071238
CG12750	FBgn0086707	NA	FBtr0081088
CG12752	FBgn0028411	Nxt1	FBtr0072241
CG12753	FBgn0038427	CG12753	FBtr0083315
CG12754	FBgn0033043	NA	FBtr0086051
CG12755	FBgn0002440	NA	FBtr0077062
CG12765	FBgn0033813	NA	FBtr0087742
CG12769	FBgn0033252	NA	FBtr0088828
CG12772	FBgn0030055	NA	FBtr0071221
CG12773	FBgn0024365	NA	FBtr0070235
CG12775	FBgn0032987	NA	FBtr0085961
CG12782	FBgn0034838	NA	FBtr0072048
CG12783	FBgn0038448	NA	FBtr0089666
CG12784	FBgn0038356	NA	FBtr0083169
CG12788	FBgn0031041	NA	FBtr0100291
CG12789	FBgn0025697	CG12789	FBtr0079464
CG12792	FBgn0022288	l(2)09851	FBtr0086043
CG12794	FBgn0028583	NA	FBtr0077220
CG12806	FBgn0037766	NA	FBtr0082151
CG12819	FBgn0037810	NA	FBtr0082231
CG12832	FBgn0033138	NA	FBtr0089060
CG12835	FBgn0033142	NA	FBtr0089040
CG12836	FBgn0033140	NA	FBtr0089037
CG12838	FBgn0033136	NA	FBtr0089035
CG12839	FBgn0033135	NA	FBtr0089034
CG12840	FBgn0033134	Tsp42El	FBtr0089032
CG12846	FBgn0029507	Tsp42Ed	FBtr0086181
CG12849	FBgn0035068	NA	FBtr0072376
CG12857	FBgn0033963	NA	FBtr0087469
CG12861	FBgn0033953	NA	FBtr0087481
CG12869	FBgn0033943	CG12869	FBtr0087496
CG12876	FBgn0086346	NA	FBtr0085241
CG12878	FBgn0045862	NA	FBtr0085220
CG1288	FBgn0250845	NA	FBtr0081604
CG12880	FBgn0046258	NA	FBtr0085217
CG12898	FBgn0033516	NA	FBtr0088321
CG12900	FBgn0033515	NA	FBtr0088323
CG12905	FBgn0033508	NA	FBtr0088359
CG12908	FBgn0026403	Ndg	FBtr0088350
CG12931	FBgn0033422	NA	FBtr0088519
CG12935	FBgn0033547	NA	FBtr0088221
CG12936	FBgn0033549	CG12936	FBtr0088222
CG12942	FBgn0033569	NA	FBtr0088236
CG12944	FBgn0033573	NA	FBtr0088199
CG12948	FBgn0037739	NA	FBtr0082167
CG12950	FBgn0037736	CG12950	FBtr0082170
CG12951	FBgn0037677	NA	FBtr0082058
CG12952	FBgn0037672	sage	FBtr0081982
CG1298	FBgn0033032	CG1298	FBtr0086037
CG1299	FBgn0035501	NA	FBtr0073215
CG12990	FBgn0030859	NA	FBtr0074479
CG12991	FBgn0030847	NA	FBtr0074448
CG12993	FBgn0030840	NA	FBtr0074437
CG12998	FBgn0030829	NA	FBtr0074394
CG13021	FBgn0029669	NA	FBtr0070572
CG13031	FBgn0036655	NA	FBtr0075319

CG13035	FBgn0036628	NA	FBtr0075415
CG13036	FBgn0036626	NA	FBtr0075416
CG13037	FBgn0260460	mRpS34	FBtr0075379
CG1304	FBgn0031141	NA	FBtr0077271
CG13048	FBgn0036593	NA	FBtr0075475
CG13049	FBgn0036592	CG13049	FBtr0075476
CG13050	FBgn0036591	NA	FBtr0075478
CG13055	FBgn0036583	NA	FBtr0075482
CG13064	FBgn0040796	NA	FBtr0089378
CG13065	FBgn0036590	NA	FBtr0075468
CG13076	FBgn0044028	NA	FBtr0075436
CG13079	FBgn0032808	NA	FBtr0081255
CG13081	FBgn0032804	NA	FBtr0081252
CG13082	FBgn0032803	NA	FBtr0081251
CG13089	FBgn0032052	CG13089	FBtr0079682
CG1309	FBgn0035519	CG1309	FBtr0073229
CG13090	FBgn0032054	NA	FBtr0079683
CG13091	FBgn0032055	NA	FBtr0079684
CG13097	FBgn0032051	NA	FBtr0079697
CG13098	FBgn0032053	NA	FBtr0079696
CG13101	FBgn0032084	NA	FBtr0079784
CG13106	FBgn0032096	NA	FBtr0079770
CG1311	FBgn0035523	NA	FBtr0073232
CG13113	FBgn0032126	NA	FBtr0079832
CG13121	FBgn0032145	NA	FBtr0079922
CG13127	FBgn0032176	NA	FBtr0079933
CG13138	FBgn0032211	NA	FBtr0080066
CG13140	FBgn0032233	NA	FBtr0080004
CG13151	FBgn0033750	CG13151	FBtr0087939
CG13157	FBgn0033732	NA	FBtr0087925
CG13158	FBgn0033727	NA	FBtr0087918
CG13160	FBgn0033720	CG13160	FBtr0087911
CG13162	FBgn0033718	NA	FBtr0087904
CG13164	FBgn0061197	SIP2	FBtr0089670
CG13170	FBgn0033703	NA	FBtr0087996
CG13175	FBgn0033693	NA	FBtr0100143
CG13183	FBgn0033667	CG13183	FBtr0088054
CG13186	FBgn0033680	CG13186	FBtr0087981
CG13188	FBgn0033668	CG13188	FBtr0088039
CG13193	FBgn0033650	NA	FBtr0088029
CG13195	FBgn0033648	NA	FBtr0088080
CG13196	FBgn0033645	CG13196	FBtr0088084
CG13197	FBgn0062449	NA	FBtr0088078
CG13198	FBgn0033640	NA	FBtr0088072
CG13202	FBgn0033623	NA	FBtr0088157
CG13204	FBgn0033627	CG13204	FBtr0088128
CG13206	FBgn0026385	NA	FBtr0088166
CG13213	FBgn0033609	NA	FBtr0088180
CG13218	FBgn0033587	NA	FBtr0088195
CG13220	FBgn0033608	CG13220	FBtr0088116
CG13221	FBgn0041174	Vhl	FBtr0088115
CG13225	FBgn0026386	NA	FBtr0088111
CG13226	FBgn0033594	NA	FBtr0088108
CG13228	FBgn0033588	NA	FBtr0088106
CG13229	FBgn0033579	NA	FBtr0088206
CG13230	FBgn0040764	NA	FBtr0088204
CG13231	FBgn0033580	NA	FBtr0088203

CG13232	FBgn0033578	NA	FBtr0088202
CG1324	FBgn0031129	NA	FBtr0077293
CG13248	FBgn0036984	CG13248	FBtr0078234
CG13250	FBgn0037013	NA	FBtr0078211
CG13251	FBgn0037014	NA	FBtr0078243
CG13252	FBgn0037016	CG13252	FBtr0078245
CG13255	FBgn0040636	NA	FBtr0078263
CG13280	FBgn0032609	NA	FBtr0080931
CG13281	FBgn0022213	Cas	FBtr0080930
CG13284	FBgn0032614	CG13284	FBtr0080927
CG13287	FBgn0035643	NA	FBtr0077027
CG13293	FBgn0035677	NA	FBtr0077050
CG13295	FBgn0035674	CG13295	FBtr0077065
CG13296	FBgn0035687	NA	FBtr0076997
CG13297	FBgn0035685	CG13297	FBtr0077020
CG13298	FBgn0035692	CG13298	FBtr0077015
CG13310	FBgn0035928	NA	FBtr0076590
CG13313	FBgn0035941	CG13313	FBtr0076512
CG13315	FBgn0040827	CG13315	FBtr0076530
CG13324	FBgn0033789	NA	FBtr0087796
CG13325	FBgn0033792	NA	FBtr0087722
CG13337	FBgn0033863	NA	FBtr0087626
CG13338	FBgn0033867	NA	FBtr0087629
CG13339	FBgn0033871	CG13339	FBtr0087632
CG13340	FBgn0033868	NA	FBtr0087649
CG13360	FBgn0025620	CG13360	FBtr0070147
CG13364	FBgn0026879	NA	FBtr0070159
CG13365	FBgn0029529	CG13365	FBtr0070160
CG13377	FBgn0261446	CG13377	FBtr0070106
CG13380	FBgn0036799	NA	FBtr0100555
CG13386	FBgn0032040	NA	FBtr0079661
CG13387	FBgn0020497	emb	FBtr0079663
CG13388	FBgn0027932	Akap200	FBtr0079667
CG1339	FBgn0033202	NA	FBtr0088945
CG13390	FBgn0032031	CG13390	FBtr0079723
CG13396	FBgn0001084	fy	FBtr0079712
CG13397	FBgn0014417	NA	FBtr0079711
CG13398	FBgn0032042	NA	FBtr0079710
CG13399	FBgn0043002	NA	FBtr0100267
CG13407	FBgn0038931	NA	FBtr0084181
CG13417	FBgn0041229	NA	FBtr0084133
CG13418	FBgn0038903	NA	FBtr0084166
CG13419	FBgn0038901	NA	FBtr0084131
CG1342	FBgn0039795	CG1342	FBtr0085698
CG13422	FBgn0034511	NA	FBtr0086214
CG13424	FBgn0034520	CG13424	FBtr0086218
CG13430	FBgn0034518	NA	FBtr0100496
CG13432	FBgn0028622	l(2)05510	FBtr0086258
CG13437	FBgn0034541	NA	FBtr0086235
CG13438	FBgn0034545	NA	FBtr0086237
CG1344	FBgn0027507	CG1344	FBtr0086034
CG13441	FBgn0041240	NA	FBtr0086241
CG13442	FBgn0034546	NA	FBtr0086240
CG13445	FBgn0036532	NA	FBtr0075503
CG13458	FBgn0036479	NA	FBtr0075637
CG13465	FBgn0040809	NA	FBtr0075674
CG13474	FBgn0036439	NA	FBtr0075711

CG13476	FBgn0036441	NA	FBtr0075687
CG13477	FBgn0036438	NA	FBtr0075685
CG13491	FBgn0041237	NA	FBtr0071727
CG13493	FBgn0034667	NA	FBtr0071735
CG13500	FBgn0034683	NA	FBtr0071805
CG13506	FBgn0034723	CG13506	FBtr0071822
CG13521	FBgn0005631	NA	FBtr0071923
CG13524	FBgn0034769	NA	FBtr0071920
CG13526	FBgn0034774	NA	FBtr0071878
CG13531	FBgn0034786	NA	FBtr0071910
CG13533	FBgn0034793	asrij	FBtr0071902
CG13538	FBgn0034820	NA	FBtr0071966
CG13540	FBgn0034839	NA	FBtr0072000
CG13544	FBgn0034826	NA	FBtr0071973
CG13550	FBgn0034853	CG13550	FBtr0072010
CG13551	FBgn0040660	CG13551	FBtr0072035
CG13558	FBgn0034869	NA	FBtr0072056
CG13559	FBgn0034870	NA	FBtr0072057
CG13561	FBgn0034906	NA	FBtr0072243
CG13564	FBgn0034973	NA	FBtr0072246
CG13570	FBgn0015544	NA	FBtr0072256
CG13576	FBgn0034994	NA	FBtr0072274
CG13581	FBgn0035014	NA	FBtr0072300
CG13587	FBgn0035031	NA	FBtr0072314
CG13589	FBgn0035011	NA	FBtr0072354
CG1359	FBgn0260860	NA	FBtr0085570
CG13590	FBgn0035012	NA	FBtr0072353
CG13594	FBgn0035041	NA	FBtr0072336
CG13608	FBgn0039159	NA	FBtr0084577
CG1361	FBgn0000094	NA	FBtr0085611
CG13610	FBgn0086365	NA	FBtr0084598
CG13611	FBgn0039177	CG13611	FBtr0084600
CG13617	FBgn0039201	NA	FBtr0084641
CG13618	FBgn0039203	CG13618	FBtr0084643
CG13627	FBgn0039217	CG13627	FBtr0084686
CG13628	FBgn0039218	NA	FBtr0084688
CG13630	FBgn0039219	NA	FBtr0084745
CG13633	FBgn0015591	Ast	FBtr0084744
CG13646	FBgn0039255	NA	FBtr0084751
CG1365	FBgn0000276	NA	FBtr0085612
CG13653	FBgn0039288	CG13653	FBtr0084804
CG13667	FBgn0035890	NA	FBtr0076610
CG1367	FBgn0000277	NA	FBtr0085614
CG13671	FBgn0035867	NA	FBtr0076706
CG13676	FBgn0035844	CG13676	FBtr0076732
CG13679	FBgn0035856	NA	FBtr0076696
CG13691	FBgn0031255	NA	FBtr0078058
CG13692	FBgn0031254	NA	FBtr0078057
CG13693	FBgn0031235	NA	FBtr0078126
CG13695	FBgn0020300	gk	FBtr0075126
CG13698	FBgn0036773	CG13698	FBtr0075137
CG13699	FBgn0036781	CG13699	FBtr0075134
CG13701	FBgn0036786	skl	FBtr0075119
CG13702	FBgn0036789	NA	FBtr0089387
CG13704	FBgn0035583	CG13704	FBtr0073353
CG13705	FBgn0035582	NA	FBtr0073352
CG1371	FBgn0033482	CG1371	FBtr0088428

CG13711	FBgn0035572	NA	FBtr0073347
CG13712	FBgn0035570	NA	FBtr0073364
CG13713	FBgn0042199	NA	FBtr0073346
CG13716	FBgn0035563	NA	FBtr0073319
CG1372	FBgn0004649	NA	FBtr0073897
CG13721	FBgn0035554	NA	FBtr0073328
CG13722	FBgn0035553	NA	FBtr0073329
CG13724	FBgn0036707	NA	FBtr0075214
CG13726	FBgn0036709	NA	FBtr0075247
CG13727	FBgn0036711	NA	FBtr0075243
CG1373	FBgn0000279	NA	FBtr0085616
CG13737	FBgn0036382	NA	FBtr0075755
CG13738	FBgn0036390	NA	FBtr0075790
CG13739	FBgn0033403	NA	FBtr0088558
CG1374	FBgn0003866	tsh	FBtr0085906
CG13741	FBgn0033374	CG13741	FBtr0088632
CG13744	FBgn0033363	NA	FBtr0088666
CG13761	FBgn0011566	NA	FBtr0070447
CG13771	FBgn0031844	NA	FBtr0079323
CG13775	FBgn0031874	NA	FBtr0079363
CG1378	FBgn0003720	NA	FBtr0085709
CG13780	FBgn0031888	Pvf2	FBtr0079423
CG13786	FBgn0031900	NA	FBtr0079409
CG13787	FBgn0041247	NA	FBtr0079463
CG13791	FBgn0031923	NA	FBtr0079521
CG13792	FBgn0031927	NA	FBtr0079520
CG13809	FBgn0035317	NA	FBtr0072873
CG13814	FBgn0036960	NA	FBtr0074831
CG13822	FBgn0039098	CG13822	FBtr0084434
CG13837	FBgn0039042	NA	FBtr0084345
CG13838	FBgn0039041	NA	FBtr0084344
CG13842	FBgn0039009	NA	FBtr0084331
CG13848	FBgn0038966	CG13848	FBtr0084297
CG13849	FBgn0038964	Nop56	FBtr0084230
CG13852	FBgn0038965	NA	FBtr0084229
CG13855	FBgn0038960	NA	FBtr0084225
CG1386	FBgn0026268	NA	FBtr0073468
CG13865	FBgn0039965	NA	FBtr0111192
CG13867	FBgn0034503	MED8	FBtr0086297
CG1387	FBgn0030033	NA	FBtr0071173
CG13870	FBgn0034506	NA	FBtr0086288
CG13872	FBgn0034477	NA	FBtr0086341
CG13875	FBgn0035104	CG13875	FBtr0072476
CG13888	FBgn0035167	NA	FBtr0072585
CG13889	FBgn0035168	NA	FBtr0072586
CG13890	FBgn0035169	CG13890	FBtr0072587
CG13894	FBgn0035157	CG13894	FBtr0072606
CG13897	FBgn0035160	NA	FBtr0072601
CG13900	FBgn0035162	CG13900	FBtr0072597
CG13907	FBgn0035173	CG13907	FBtr0072593
CG13920	FBgn0025712	CG13920	FBtr0072761
CG13922	FBgn0035272	mRpL46	FBtr0072776
CG13930	FBgn0035256	NA	FBtr0072803
CG13933	FBgn0035270	CG13933	FBtr0072792
CG13939	FBgn0033931	NA	FBtr0087556
CG1394	FBgn0030277	NA	FBtr0073467
CG13946	FBgn0040725	NA	FBtr0078004

CG1395	FBgn0003525	NA	FBtr0085397
CG13955	FBgn0033412	NA	FBtr0088551
CG13956	FBgn0040207	NA	FBtr0074247
CG13957	FBgn0030739	NA	FBtr0074224
CG13965	FBgn0032834	NA	FBtr0081308
CG13969	FBgn0045064	NA	FBtr0081331
CG13970	FBgn0032851	NA	FBtr0089388
CG13972	FBgn0039522	NA	FBtr0085212
CG13977	FBgn0039519	NA	FBtr0085183
CG13978	FBgn0039518	NA	FBtr0085182
CG13989	FBgn0031786	NA	FBtr0079204
CG13990	FBgn0040950	CG13990	FBtr0079203
CG13991	FBgn0031785	NA	FBtr0079202
CG13994	FBgn0031772	NA	FBtr0079188
CG13995	FBgn0031770	NA	FBtr0079187
CG13996	FBgn0031763	NA	FBtr0079181
CG13998	FBgn0040949	NA	FBtr0079241
CG14000	FBgn0031749	NA	FBtr0079153
CG14001	FBgn0043362	NA	FBtr0079144
CG1401	FBgn0039632	NA	FBtr0085417
CG14026	FBgn0003716	tkv	FBtr0079089
CG14031	FBgn0031695	NA	FBtr0079068
CG14032	FBgn0031693	NA	FBtr0079066
CG14052	FBgn0029606	NA	FBtr0070394
CG14053	FBgn0029603	NA	FBtr0070424
CG14064	FBgn0039584	NA	FBtr0085289
CG14069	FBgn0032315	NA	FBtr0080175
CG14073	FBgn0036814	NA	FBtr0075084
CG14080	FBgn0036844	Mkp3	FBtr0075035
CG14089	FBgn0036861	NA	FBtr0075015
CG1410	FBgn0024182	NA	FBtr0100536
CG14101	FBgn0036905	NA	FBtr0074950
CG14111	FBgn0036350	NA	FBtr0075841
CG14112	FBgn0036349	SNCF	FBtr0075840
CG14113	FBgn0040814	NA	FBtr0075837
CG14115	FBgn0036343	NA	FBtr0075893
CG14119	FBgn0036322	NA	FBtr0075869
CG14120	FBgn0036321	NA	FBtr0075868
CG14122	FBgn0036282	CG14122	FBtr0075985
CG14126	FBgn0036223	NA	FBtr0076068
CG14127	FBgn0036219	NA	FBtr0076037
CG1414	FBgn0024251	NA	FBtr0100598
CG14147	FBgn0036112	NA	FBtr0076194
CG14149	FBgn0036105	NA	FBtr0076269
CG14153	FBgn0036094	NA	FBtr0076259
CG14154	FBgn0036093	NA	FBtr0076257
CG14156	FBgn0036078	NA	FBtr0076300
CG14157	FBgn0036080	NA	FBtr0076297
CG14162	FBgn0040823	dpr6	FBtr0076353
CG14173	FBgn0044051	NA	FBtr0076328
CG14177	FBgn0036013	NA	FBtr0076378
CG14186	FBgn0036935	CG14186	FBtr0074870
CG14190	FBgn0030979	NA	FBtr0074663
CG14194	FBgn0030996	CG14194	FBtr0074671
CG14205	FBgn0031034	NA	FBtr0074730
CG1421	FBgn0032966	NA	FBtr0085928
CG14210	FBgn0031040	NA	FBtr0074740

CG14211	FBgn0031044	NA	FBtr0074741
CG14212	FBgn0031045	NA	FBtr0074742
CG14213	FBgn0031047	CG14213	FBtr0074744
CG14214	FBgn0031049	NA	FBtr0074745
CG14215	FBgn0031052	NA	FBtr0074746
CG14216	FBgn0031054	NA	FBtr0074747
CG14219	FBgn0031033	NA	FBtr0074769
CG14222	FBgn0031043	NA	FBtr0074763
CG14223	FBgn0031053	NA	FBtr0074758
CG14226	FBgn0043903	NA	FBtr0074756
CG14234	FBgn0031065	NA	FBtr0074790
CG14238	FBgn0039429	NA	FBtr0085014
CG14242	FBgn0039443	NA	FBtr0085034
CG14243	FBgn0039444	NA	FBtr0085035
CG14247	FBgn0039454	NA	FBtr0085043
CG14250	FBgn0039448	NA	FBtr0085049
CG14253	FBgn0039467	CG14253	FBtr0085073
CG14262	FBgn0039503	NA	FBtr0085177
CG14265	FBgn0040393	NA	FBtr0070541
CG14266	FBgn0010294	NA	FBtr0070540
CG14270	FBgn0029665	CG14270	FBtr0070590
CG14271	FBgn0029667	NA	FBtr0070589
CG14275	FBgn0032022	CG14275	FBtr0079602
CG1428	FBgn0032967	NA	FBtr0085929
CG14280	FBgn0038695	NA	FBtr0083738
CG14292	FBgn0038658	NA	FBtr0083725
CG14303	FBgn0038633	NA	FBtr0083659
CG14314	FBgn0038581	CG14314	FBtr0083560
CG14316	FBgn0038567	NA	FBtr0083546
CG14317	FBgn0038566	CG14317	FBtr0083541
CG14323	FBgn0038529	NA	FBtr0083498
CG1433	FBgn0019637	Atu	FBtr0078704
CG14334	FBgn0038498	beat-IIa	FBtr0083453
CG14339	FBgn0031301	NA	FBtr0077954
CG1434	FBgn0030554	CG1434	FBtr0073903
CG14346	FBgn0031337	NA	FBtr0077926
CG14356	FBgn0038207	CG14356	FBtr0082908
CG14360	FBgn0038203	NA	FBtr0082890
CG14367	FBgn0038170	NA	FBtr0082819
CG14369	FBgn0038159	NA	FBtr0082789
CG14376	FBgn0038153	NA	FBtr0082784
CG14396	FBgn0011829	NA	FBtr0081492
CG14400	FBgn0032896	NA	FBtr0081449
CG14402	FBgn0032894	NA	FBtr0081425
CG14410	FBgn0030583	NA	FBtr0073948
CG14416	FBgn0040352	NA	FBtr0070567
CG14421	FBgn0029644	NA	FBtr0070554
CG14424	FBgn0025644	NA	FBtr0070563
CG1443	FBgn0039620	NA	FBtr0085345
CG14447	FBgn0029830	Grip	FBtr0070865
CG14452	FBgn0037180	NA	FBtr0078520
CG14453	FBgn0037179	NA	FBtr0078519
CG14454	FBgn0037177	NA	FBtr0078542
CG14457	FBgn0037174	NA	FBtr0078517
CG14472	FBgn0011230	NA	FBtr0079545
CG14478	FBgn0028953	NA	FBtr0086921
CG1448	FBgn0028373	inx3	FBtr0085350

CG14482	FBgn0034245	CG14482	FBtr0086905
CG14485	FBgn0034262	NA	FBtr0086889
CG14488	FBgn0034278	NA	FBtr0089558
CG14489	FBgn0015522	olf186-M	FBtr0086799
CG14490	FBgn0034281	NA	FBtr0086801
CG14491	FBgn0034284	NA	FBtr0086803
CG14492	FBgn0034283	NA	FBtr0086818
CG14500	FBgn0034318	NA	FBtr0086752
CG14503	FBgn0040737	NA	FBtr0086763
CG14508	FBgn0039651	NA	FBtr0085406
CG1451	FBgn0015589	NA	FBtr0085351
CG14512	FBgn0039639	NA	FBtr0085413
CG14513	FBgn0005596	NA	FBtr0085415
CG14515	FBgn0039648	NA	FBtr0085389
CG14516	FBgn0039640	CG14516	FBtr0085383
CG14521	FBgn0039617	CG14521	FBtr0085347
CG14523	FBgn0039612	NA	FBtr0085349
CG14527	FBgn0039613	NA	FBtr0085330
CG14528	FBgn0039611	CG14528	FBtr0085329
CG14531	FBgn0261289	NA	FBtr0085312
CG14534	FBgn0031957	CG14534	FBtr0079563
CG14537	FBgn0031954	NA	FBtr0079526
CG14538	FBgn0031943	NA	FBtr0079484
CG14540	FBgn0039398	NA	FBtr0084948
CG14545	FBgn0040602	CG14545	FBtr0084913
CG14562	FBgn0037142	NA	FBtr0078472
CG14575	FBgn0037100	NA	FBtr0078379
CG14584	FBgn0031181	NA	FBtr0077213
CG14598	FBgn0037503	CG14598	FBtr0081534
CG14605	FBgn0037486	NA	FBtr0081601
CG1461	FBgn0030558	NA	FBtr0073908
CG14615	FBgn0031184	NA	FBtr0077369
CG1462	FBgn0016123	NA	FBtr0085733
CG14621	FBgn0031183	CG14621	FBtr0077344
CG14624	FBgn0040357	NA	FBtr0070204
CG14625	FBgn0040358	NA	FBtr0070202
CG14626	FBgn0040360	CG14626	FBtr0070200
CG14636	FBgn0037217	CG14636	FBtr0111206
CG14639	FBgn0037224	CG14639	FBtr0078975
CG1464	FBgn0005558	NA	FBtr0100396
CG14640	FBgn0037227	NA	FBtr0078976
CG14643	FBgn0037225	CG14643	FBtr0078982
CG14644	FBgn0250821	NA	FBtr0078981
CG14654	FBgn0037273	NA	FBtr0078883
CG14657	FBgn0037282	CG14657	FBtr0078875
CG14660	FBgn0020280	NA	FBtr0078852
CG14667	FBgn0037317	CG14667	FBtr0078788
CG14668	FBgn0037320	NA	FBtr0078801
CG14670	FBgn0037332	CG14670	FBtr0078683
CG14675	FBgn0037385	NA	FBtr0078640
CG1468	FBgn0030157	NA	FBtr0071388
CG14687	FBgn0037835	CG14687	FBtr0082280
CG14689	FBgn0037826	NA	FBtr0082317
CG14690	FBgn0037828	NA	FBtr0082316
CG14691	FBgn0037829	NA	FBtr0082314
CG1471	FBgn0039774	CDase	FBtr0085649
CG14712	FBgn0037924	NA	FBtr0082418

CG14716	FBgn0037933	Ho	FBtr0082424
CG14717	FBgn0037938	NA	FBtr0082425
CG14718	FBgn0037939	NA	FBtr0082426
CG14719	FBgn0025821	NA	FBtr0082427
CG14720	FBgn0037940	NA	FBtr0082428
CG14721	FBgn0037942	NA	FBtr0082430
CG14728	FBgn0003312	sad	FBtr0082475
CG14731	FBgn0037964	NA	FBtr0082478
CG14734	FBgn0037976	NA	FBtr0082488
CG14739	FBgn0037987	NA	FBtr0082553
CG14745	FBgn0043575	NA	FBtr0088709
CG14746	FBgn0043576	NA	FBtr0088707
CG1475	FBgn0037351	NA	FBtr0078705
CG14756	FBgn0033275	CG14756	FBtr0088804
CG14757	FBgn0033274	NA	FBtr0088805
CG14760	FBgn0033277	NA	FBtr0088794
CG14779	FBgn0013720	NA	FBtr0070255
CG1478	FBgn0000359	NA	FBtr0071203
CG14782	FBgn0025381	NA	FBtr0070259
CG1480	FBgn0004389	NA	FBtr0085754
CG14803	FBgn0023513	CG14803	FBtr0070310
CG14804	FBgn0014411	NA	FBtr0070311
CG14805	FBgn0023514	CG14805	FBtr0070312
CG14806	FBgn0029594	CG14806	FBtr0070313
CG14816	FBgn0023517	CG14816	FBtr0070366
CG14817	FBgn0026089	NA	FBtr0070365
CG14818	FBgn0026088	NA	FBtr0070364
CG14820	FBgn0035718	NA	FBtr0076894
CG14827	FBgn0016036	NA	FBtr0076836
CG1483	FBgn0002645	Map205	FBtr0085876
CG14839	FBgn0038219	NA	FBtr0082920
CG1484	FBgn0000709	NA	FBtr0077192
CG14840	FBgn0038217	NA	FBtr0082952
CG14841	FBgn0038218	NA	FBtr0082951
CG14851	FBgn0038240	NA	FBtr0082956
CG14852	FBgn0038242	NA	FBtr0082957
CG14853	FBgn0038246	NA	FBtr0082960
CG14856	FBgn0038261	NA	FBtr0082992
CG1486	FBgn0031174	CG1486	FBtr0077191
CG14861	FBgn0038280	NA	FBtr0083023
CG14869	FBgn0038341	NA	FBtr0083159
CG14872	FBgn0038346	NA	FBtr0083156
CG14877	FBgn0038380	NA	FBtr0083249
CG1488	FBgn0030367	NA	FBtr0073639
CG14880	FBgn0038422	NA	FBtr0083321
CG1489	FBgn0020369	Pros45	FBtr0077189
CG1490	FBgn0030366	NA	FBtr0073641
CG14901	FBgn0038440	NA	FBtr0089695
CG14905	FBgn0038452	NA	FBtr0083357
CG14916	FBgn0041246	NA	FBtr0080198
CG1492	FBgn0030361	CG1492	FBtr0073579
CG14921	FBgn0032345	CG14921	FBtr0080227
CG14926	FBgn0032360	NA	FBtr0080251
CG14936	FBgn0032376	NA	FBtr0080265
CG1494	FBgn0031169	NA	FBtr0077187
CG14945	FBgn0032402	CG14945	FBtr0080294
CG14947	FBgn0032406	NA	FBtr0080297

CG14948	FBgn0030723	dpr18	FBtr0074206
CG1495	FBgn0016126	NA	FBtr0089065
CG14968	FBgn0035431	NA	FBtr0073089
CG14982	FBgn0035477	NA	FBtr0073163
CG14994	FBgn0004516	Gad1	FBtr0073275
CG14997	FBgn0035515	NA	FBtr0073225
CG15002	FBgn0011653	mas	FBtr0073260
CG15009	FBgn0001257	ImpL2	FBtr0073249
CG1501	FBgn0003950	NA	FBtr0077259
CG15010	FBgn0041171	NA	FBtr0073245
CG15013	FBgn0066365	NA	FBtr0073341
CG15021	FBgn0035544	NA	FBtr0073301
CG15022	FBgn0035547	NA	FBtr0073333
CG15025	FBgn0038709	NA	FBtr0083768
CG15027	FBgn0030611	NA	FBtr0073965
CG15029	FBgn0030619	NA	FBtr0073998
CG15031	FBgn0030623	NA	FBtr0074023
CG15043	FBgn0030929	CG15043	FBtr0074626
CG15044	FBgn0030928	CG15044	FBtr0074627
CG15046	FBgn0030927	NA	FBtr0074628
CG15056	FBgn0030918	NA	FBtr0074560
CG15059	FBgn0030905	NA	FBtr0074585
CG15065	FBgn0040734	NA	FBtr0086667
CG15068	FBgn0040733	NA	FBtr0086727
CG15071	FBgn0034377	NA	FBtr0086605
CG15073	FBgn0034379	NA	FBtr0086607
CG15080	FBgn0034391	NA	FBtr0086613
CG15085	FBgn0023214	edl	FBtr0086652
CG15087	FBgn0034380	CG15087	FBtr0086647
CG15094	FBgn0034392	CG15094	FBtr0086637
CG15095	FBgn0010651	l(2)08717	FBtr0086634
CG15096	FBgn0034394	NA	FBtr0086632
CG15107	FBgn0041702	NA	FBtr0086600
CG15110	FBgn0027535	NA	FBtr0086579
CG15116	FBgn0034415	NA	FBtr0086596
CG15117	FBgn0034417	CG15117	FBtr0086593
CG1512	FBgn0032956	cul-2	FBtr0085899
CG15121	FBgn0034456	NA	FBtr0086465
CG15122	FBgn0034457	NA	FBtr0086466
CG15126	FBgn0040729	NA	FBtr0086475
CG15128	FBgn0034467	NA	FBtr0086481
CG15138	FBgn0032629	beat-IIIc	FBtr0080971
CG1514	FBgn0029976	NA	FBtr0071092
CG1515	FBgn0260858	NA	FBtr0071093
CG15151	FBgn0243486	PFE	FBtr0081029
CG15153	FBgn0032663	NA	FBtr0081045
CG15154	FBgn0041184	NA	FBtr0081040
CG15160	FBgn0032688	NA	FBtr0081070
CG15161	FBgn0032692	CG15161	FBtr0081078
CG15162	FBgn0032694	MESR3	FBtr0081073
CG15173	FBgn0032744	CG15173	FBtr0081178
CG15185	FBgn0037449	NA	FBtr0081675
CG1520	FBgn0024273	WASp	FBtr0085352
CG15200	FBgn0030278	NA	FBtr0073445
CG15203	FBgn0030261	CG15203	FBtr0073392
CG15207	FBgn0030248	NA	FBtr0073384
CG15208	FBgn0030247	NA	FBtr0073383

CG15225	FBgn0034551	NA	FBtr0071513
CG15227	FBgn0034554	NA	FBtr0071514
CG15231	FBgn0040653	NA	FBtr0071559
CG15236	FBgn0033108	NA	FBtr0086160
CG15239	FBgn0029681	NA	FBtr0070612
CG1524	FBgn0004403	NA	FBtr0071095
CG15253	FBgn0028948	NA	FBtr0080782
CG15263	FBgn0028853	NA	FBtr0080729
CG15269	FBgn0028878	NA	FBtr0080704
CG1527	FBgn0004404	NA	FBtr0071096
CG15284	FBgn0032546	NA	FBtr0080634
CG15286	FBgn0028531	NA	FBtr0080625
CG15287	FBgn0261529	NA	FBtr0080623
CG15302	FBgn0030204	NA	FBtr0071462
CG15306	FBgn0030191	NA	FBtr0071471
CG15311	FBgn0030182	NA	FBtr0071436
CG1532	FBgn0031143	NA	FBtr0077268
CG15326	FBgn0029965	NA	FBtr0071149
CG15329	FBgn0029977	NA	FBtr0071133
CG15335	FBgn0030001	CG15335	FBtr0071193
CG15337	FBgn0030014	NA	FBtr0071160
CG15345	FBgn0040928	NA	FBtr0071176
CG15357	FBgn0040719	NA	FBtr0077871
CG15358	FBgn0031373	NA	FBtr0077892
CG15366	FBgn0030080	NA	FBtr0071355
CG15367	FBgn0040467	NA	FBtr0071302
CG15368	FBgn0030104	NA	FBtr0071308
CG1537	FBgn0030260	CG1537	FBtr0073394
CG15370	FBgn0030107	NA	FBtr0071310
CG15375	FBgn0029694	NA	FBtr0070637
CG15377	FBgn0026396	NA	FBtr0077826
CG15382	FBgn0031393	CG15382	FBtr0077832
CG15393	FBgn0031429	NA	FBtr0077784
CG15403	FBgn0031504	NA	FBtr0077661
CG15406	FBgn0031517	NA	FBtr0077580
CG15429	FBgn0031596	NA	FBtr0077428
CG1543	FBgn0010329	NA	FBtr0089999
CG15438	FBgn0025684	NA	FBtr0077456
CG15439	FBgn0031606	CG15439	FBtr0077455
CG15440	FBgn0031607	NA	FBtr0077454
CG15452	FBgn0031130	NA	FBtr0077292
CG1546	FBgn0039779	NA	FBtr0085654
CG15468	FBgn0061196	SIP3	FBtr0070727
CG15473	FBgn0029719	NA	FBtr0070689
CG15475	FBgn0032867	NA	FBtr0081381
CG1548	FBgn0029093	cathD	FBtr0088898
CG15480	FBgn0032489	NA	FBtr0080482
CG15481	FBgn0032487	NA	FBtr0080483
CG15482	FBgn0032483	NA	FBtr0080487
CG15497	FBgn0038894	NA	FBtr0084113
CG15498	FBgn0038892	NA	FBtr0084122
CG15503	FBgn0038888	NA	FBtr0084089
CG15505	FBgn0039684	Obp99d	FBtr0085455
CG15506	FBgn0039686	NA	FBtr0085456
CG15515	FBgn0039719	NA	FBtr0085500
CG15517	FBgn0039718	NA	FBtr0085515
CG15520	FBgn0039722	NA	FBtr0085514

CG15524	FBgn0039731	NA	FBtr0085546
CG15527	FBgn0039739	NA	FBtr0085601
CG1553	FBgn0033224	NA	FBtr0088901
CG15530	FBgn0039752	NA	FBtr0085569
CG15532	FBgn0010113	NA	FBtr0085619
CG15533	FBgn0039768	CG15533	FBtr0085626
CG15538	FBgn0039771	NA	FBtr0085696
CG1554	FBgn0003277	NA	FBtr0073542
CG15545	FBgn0039806	NA	FBtr0085719
CG15548	FBgn0039812	NA	FBtr0085731
CG15549	FBgn0039810	NA	FBtr0085752
CG1555	FBgn0000337	cn	FBtr0088931
CG15551	FBgn0062411	NA	FBtr0085749
CG15552	FBgn0024288	NA	FBtr0085735
CG15554	FBgn0039820	NA	FBtr0085736
CG15555	FBgn0039839	NA	FBtr0085764
CG15561	FBgn0039829	CG15561	FBtr0085776
CG15564	FBgn0039833	NA	FBtr0085770
CG1558	FBgn0027259	NA	FBtr0073541
CG15581	FBgn0037399	NA	FBtr0078620
CG15582	FBgn0046878	Obp83cd	FBtr0078584
CG15585	FBgn0037406	NA	FBtr0078588
CG15589	FBgn0037409	CG15589	FBtr0078591
CG1559	FBgn0030354	NA	FBtr0073596
CG15591	FBgn0037415	NA	FBtr0078598
CG15592	FBgn0037416	Osi9	FBtr0078599
CG15593	FBgn0037417	NA	FBtr0078600
CG15594	FBgn0037421	NA	FBtr0078603
CG15595	FBgn0037422	NA	FBtr0078605
CG15596	FBgn0037418	NA	FBtr0078614
CG15598	FBgn0037427	Osi17	FBtr0078610
CG1560	FBgn0004657	mys	FBtr0071105
CG15605	FBgn0034196	NA	FBtr0087002
CG15611	FBgn0034194	NA	FBtr0086982
CG15625	FBgn0031644	NA	FBtr0077390
CG15628	FBgn0031632	NA	FBtr0077384
CG15629	FBgn0031630	NA	FBtr0077383
CG15632	FBgn0031623	NA	FBtr0077420
CG15638	FBgn0028943	NA	FBtr0080516
CG15643	FBgn0030654	NA	FBtr0074072
CG15646	FBgn0030665	NA	FBtr0074062
CG15650	FBgn0034565	NA	FBtr0071554
CG15651	FBgn0034567	CG15651	FBtr0071553
CG15667	FBgn0026369	NA	FBtr0071641
CG15669	FBgn0043070	NA	FBtr0089709
CG1567	FBgn0021742	NA	FBtr0073400
CG15674	FBgn0034642	NA	FBtr0071659
CG15676	FBgn0034651	NA	FBtr0071668
CG15678	FBgn0034647	NA	FBtr0071682
CG1569	FBgn0003268	NA	FBtr0085795
CG15695	FBgn0038832	CG15695	FBtr0083967
CG15696	FBgn0038833	NA	FBtr0083968
CG15697	FBgn0038834	NA	FBtr0083969
CG15701	FBgn0034095	NA	FBtr0087131
CG15706	FBgn0034093	CG15706	FBtr0087191
CG15717	FBgn0030451	CG15717	FBtr0073723
CG15725	FBgn0030417	NA	FBtr0073685

CG15729	FBgn0030399	NA	FBtr0073664
CG15730	FBgn0030395	NA	FBtr0073667
CG15731	FBgn0030390	NA	FBtr0073670
CG15732	FBgn0030385	NA	FBtr0073673
CG15735	FBgn0030364	CG15735	FBtr0073621
CG15736	FBgn0043001	Chrac-16	FBtr0073642
CG15737	FBgn0260780	NA	FBtr0073573
CG15738	FBgn0030352	CG15738	FBtr0073572
CG15741	FBgn0030338	NA	FBtr0073606
CG15744	FBgn0030466	NA	FBtr0073756
CG15747	FBgn0030474	NA	FBtr0073766
CG1575	FBgn0029999	CG1575	FBtr0071151
CG15756	FBgn0030493	NA	FBtr0073783
CG15757	FBgn0030494	CG15757	FBtr0073784
CG15760	FBgn0030508	NA	FBtr0073810
CG15765	FBgn0029814	CG15765	FBtr0070817
CG1578	FBgn0030336	NA	FBtr0073607
CG15784	FBgn0029766	NA	FBtr0070777
CG15807	FBgn0038005	NA	FBtr0082619
CG15812	FBgn0035405	pfk	FBtr0073027
CG1582	FBgn0030246	NA	FBtr0073403
CG15824	FBgn0031292	NA	FBtr0078029
CG15845	FBgn0000054	Adf1	FBtr0086112
CG15860	FBgn0060296	NA	FBtr0072417
CG15861	FBgn0035084	CG15861	FBtr0072410
CG15865	FBgn0015336	NA	FBtr0074375
CG15871	FBgn0030552	NA	FBtr0073902
CG15878	FBgn0035316	NA	FBtr0072911
CG15884	FBgn0039481	CG15884	FBtr0085135
CG15887	FBgn0038132	CG15887	FBtr0082776
CG15888	FBgn0038131	NA	FBtr0100257
CG15889	FBgn0038128	NA	FBtr0082779
CG15890	FBgn0030576	NA	FBtr0073939
CG15891	FBgn0029860	NA	FBtr0100319
CG15892	FBgn0029859	NA	FBtr0070927
CG15904	FBgn0034458	NA	FBtr0086497
CG15919	FBgn0040743	NA	FBtr0087032
CG15922	FBgn0040575	NA	FBtr0083951
CG15925	FBgn0034129	NA	FBtr0087159
CG15927	FBgn0030389	NA	FBtr0073647
CG1594	FBgn0004864	NA	FBtr0073457
CG1600	FBgn0033188	CG1600	FBtr0088957
CG1603	FBgn0033185	CG1603	FBtr0088979
CG1605	FBgn0025185	NA	FBtr0088980
CG1607	FBgn0039844	NA	FBtr0085785
CG1619	FBgn0001206	NA	FBtr0071511
CG1631	FBgn0031101	NA	FBtr0077297
CG1633	FBgn0040309	Jafrac1	FBtr0073764
CG1635	FBgn0039854	NA	FBtr0085796
CG1639	FBgn0001491	NA	FBtr0073460
CG1640	FBgn0030478	NA	FBtr0073774
CG1641	FBgn0003411	NA	FBtr0073461
CG1644	FBgn0031182	NA	FBtr0077212
CG1647	FBgn0039602	CG1647	FBtr0085326
CG1648	FBgn0033446	CG1648	FBtr0088493
CG1650	FBgn0015561	unpg	FBtr0088586
CG1655	FBgn0030242	NA	FBtr0073409

CG1668	FBgn0011280	NA	FBtr0077303
CG1670	FBgn0031110	NA	FBtr0077302
CG16700	FBgn0030816	NA	FBtr0074383
CG16708	FBgn0037315	CG16708	FBtr0078803
CG16711	FBgn0036032	NA	FBtr0076396
CG16720	FBgn0004168	NA	FBtr0086577
CG16721	FBgn0029820	CG16721	FBtr0070821
CG1673	FBgn0030482	NA	FBtr0073777
CG16733	FBgn0037665	NA	FBtr0081974
CG16738	FBgn0003430	slp1	FBtr0077499
CG16739	FBgn0034505	NA	FBtr0086286
CG16741	FBgn0042198	NA	FBtr0086289
CG16747	FBgn0014184	Oda	FBtr0088014
CG16752	FBgn0029768	NA	FBtr0070778
CG16753	FBgn0035393	NA	FBtr0072990
CG16755	FBgn0037685	NA	FBtr0082028
CG16756	FBgn0029765	NA	FBtr0070776
CG16779	FBgn0037698	NA	FBtr0082125
CG1678	FBgn0031176	NA	FBtr0077219
CG16783	FBgn0034937	NA	FBtr0072146
CG16787	FBgn0034940	CG16787	FBtr0072207
CG16793	FBgn0036712	CG16793	FBtr0075242
CG16798	FBgn0032856	CG16798	FBtr0081371
CG16807	FBgn0036621	CG16807	FBtr0075423
CG1681	FBgn0030484	NA	FBtr0073778
CG16812	FBgn0032488	CG16812	FBtr0080445
CG16813	FBgn0032490	NA	FBtr0080446
CG16820	FBgn0032495	CG16820	FBtr0080452
CG1683	FBgn0025111	NA	FBtr0073425
CG16836	FBgn0040735	NA	FBtr0086666
CG16837	FBgn0035009	NA	FBtr0072297
CG16840	FBgn0032329	NA	FBtr0080162
CG16848	FBgn0032522	NA	FBtr0080501
CG16852	FBgn0028942	NA	FBtr0080507
CG16853	FBgn0028941	NA	FBtr0080508
CG16857	FBgn0028482	CG16857	FBtr0077425
CG16858	FBgn0016075	vkg	FBtr0079036
CG16868	FBgn0034498	CG16868	FBtr0086302
CG16869	FBgn0032535	NA	FBtr0080554
CG16879	FBgn0028904	NA	FBtr0080596
CG1688	FBgn0027589	NA	FBtr0088495
CG16889	FBgn0028658	NA	FBtr0080573
CG16892	FBgn0030122	NA	FBtr0071319
CG16894	FBgn0034483	NA	FBtr0086324
CG16898	FBgn0034480	NA	FBtr0086325
CG16903	FBgn0040394	CG16903	FBtr0070434
CG16908	FBgn0037741	NA	FBtr0082166
CG16912	FBgn0035064	NA	FBtr0072429
CG16914	FBgn0025578	NA	FBtr0072430
CG16916	FBgn0028686	NA	FBtr0073436
CG1692	FBgn0002641	NA	FBtr0077306
CG16941	FBgn0038464	NA	FBtr0083374
CG16944	FBgn0003360	NA	FBtr0073422
CG16947	FBgn0031816	NA	FBtr0079271
CG16954	FBgn0032525	NA	FBtr0080518
CG16956	FBgn0032523	NA	FBtr0080520
CG1696	FBgn0029067	NA	FBtr0077226

CG16960	FBgn0026392	NA	FBtr0080278
CG16961	FBgn0026391	NA	FBtr0080279
CG16972	FBgn0032481	NA	FBtr0080488
CG16973	FBgn0010909	NA	FBtr0072956
CG1698	FBgn0033443	CG1698	FBtr0088474
CG16984	FBgn0062517	CG16984	FBtr0072928
CG16987	FBgn0031461	Alp23B	FBtr0077721
CG16989	FBgn0025621	NA	FBtr0070140
CG16992	FBgn0035789	NA	FBtr0076789
CG16998	FBgn0035795	NA	FBtr0076829
CG17003	FBgn0031082	NA	FBtr0070035
CG17023	FBgn0024804	NA	FBtr0113715
CG17030	FBgn0035584	NA	FBtr0073374
CG17059	FBgn0040754	NA	FBtr0087739
CG17060	FBgn0015789	NA	FBtr0077337
CG17063	FBgn0027107	NA	FBtr0077339
CG17068	FBgn0031098	CG17068	FBtr0077341
CG1707	FBgn0033162	NA	FBtr0089013
CG17077	FBgn0003118	NA	FBtr0089717
CG1708	FBgn0000352	NA	FBtr0089015
CG17104	FBgn0040496	CG17104	FBtr0080105
CG17105	FBgn0032280	NA	FBtr0080106
CG17122	FBgn0036962	NA	FBtr0074849
CG17124	FBgn0032297	CG17124	FBtr0080118
CG17131	FBgn0029128	NA	FBtr0100135
CG17134	FBgn0032304	NA	FBtr0080140
CG17137	FBgn0069354	NA	FBtr0080184
CG17139	FBgn0260454	NA	FBtr0100836
CG17140	FBgn0260453	NA	FBtr0100837
CG17146	FBgn0022709	Adk1	FBtr0076018
CG17149	FBgn0260397	CG17149	FBtr0074825
CG1715	FBgn0010808	l(3)03670	FBtr0085745
CG17152	FBgn0036250	NA	FBtr0076026
CG17153	FBgn0036248	NA	FBtr0075992
CG17158	FBgn0011570	cpb	FBtr0077881
CG17161	FBgn0261278	NA	FBtr0080886
CG17177	FBgn0036440	NA	FBtr0075710
CG17191	FBgn0039473	NA	FBtr0085152
CG17195	FBgn0039369	NA	FBtr0084921
CG17207	FBgn0038051	NA	FBtr0082682
CG17218	FBgn0032421	CG17218	FBtr0080342
CG17226	FBgn0034866	NA	FBtr0072020
CG17234	FBgn0042187	NA	FBtr0077753
CG17241	FBgn0039033	NA	FBtr0084341
CG17265	FBgn0031488	CG17265	FBtr0077646
CG17269	FBgn0038827	NA	FBtr0083995
CG17278	FBgn0046763	NA	FBtr0084047
CG17280	FBgn0034877	CG17280	FBtr0072061
CG17284	FBgn0038859	NA	FBtr0084030
CG17285	FBgn0000639	NA	FBtr0075750
CG17290	FBgn0034201	NA	FBtr0086989
CG17293	FBgn0032030	NA	FBtr0079650
CG17301	FBgn0031442	NA	FBtr0077697
CG17302	FBgn0031443	NA	FBtr0077699
CG17304	FBgn0038267	NA	FBtr0083012
CG17321	FBgn0032719	CG17321	FBtr0081110
CG17327	FBgn0038107	NA	FBtr0082721

CG17330	FBgn0028841	NA	FBtr0080859
CG17332	FBgn0027779	VhaSFD	FBtr0080941
CG17334	FBgn0035626	lin-28	FBtr0077090
CG17342	FBgn0017581	NA	FBtr0082534
CG17348	FBgn0015380	drl	FBtr0081195
CG17359	FBgn0036396	NA	FBtr0075787
CG1736	FBgn0261395	NA	FBtr0085738
CG17360	FBgn0037949	NA	FBtr0082531
CG17361	FBgn0036395	NA	FBtr0075767
CG17364	FBgn0036391	NA	FBtr0075789
CG17367	FBgn0028717	NA	FBtr0084935
CG1737	FBgn0030293	NA	FBtr0073453
CG17378	FBgn0031858	NA	FBtr0079336
CG17397	FBgn0040020	NA	FBtr0113739
CG1743	FBgn0001145	Gs2	FBtr0073494
CG17436	FBgn0260987	NA	FBtr0113746
CG1744	FBgn0000313	chp	FBtr0085778
CG1745	FBgn0030301	NA	FBtr0073499
CG17453	FBgn0033982	NA	FBtr0087405
CG17461	FBgn0039925	NA	FBtr0089196
CG1747	FBgn0030300	Sk1	FBtr0073497
CG17471	FBgn0039924	NA	FBtr0100543
CG17484	FBgn0260799	NA	FBtr0111113
CG1749	FBgn0030305	CG1749	FBtr0073505
CG17492	FBgn0086442	NA	FBtr0081142
CG1750	FBgn0039836	NA	FBtr0085767
CG1751	FBgn0030306	NA	FBtr0073506
CG17520	FBgn0000258	NA	FBtr0070043
CG17528	FBgn0261387	NA	FBtr0111277
CG1753	FBgn0031148	CG1753	FBtr0077245
CG17534	FBgn0063491	NA	FBtr0086677
CG17556	FBgn0038462	NA	FBtr0083375
CG17559	FBgn0024245	NA	FBtr0081224
CG17562	FBgn0038449	CG17562	FBtr0083329
CG17567	FBgn0040994	NA	FBtr0081231
CG17568	FBgn0032763	NA	FBtr0081233
CG17569	FBgn0035416	NA	FBtr0073041
CG17570	FBgn0260000	NA	FBtr0081375
CG17579	FBgn0003326	NA	FBtr0087856
CG17580	FBgn0040755	NA	FBtr0087857
CG17593	FBgn0031544	CG17593	FBtr0077525
CG17594	FBgn0028993	NA	FBtr0113753
CG17599	FBgn0031196	NA	FBtr0077363
CG17611	FBgn0034915	NA	FBtr0072235
CG17612	FBgn0031597	NA	FBtr0077463
CG1762	FBgn0010395	NA	FBtr0081461
CG17629	FBgn0001314	NA	FBtr0113756
CG1763	FBgn0002948	NA	FBtr0073516
CG17637	FBgn0037004	NA	FBtr0078207
CG1764	FBgn0030467	CG1764	FBtr0073796
CG17642	FBgn0031357	mRpL48	FBtr0077882
CG17646	FBgn0031362	CG17646	FBtr0077886
CG17648	FBgn0031364	CG17648	FBtr0077887
CG17650	FBgn0031365	NA	FBtr0077901
CG17657	FBgn0086698	NA	FBtr0077911
CG17660	FBgn0031356	NA	FBtr0077912
CG17665	FBgn0262117	NA	FBtr0111268

CG17673	FBgn0003034	NA	FBtr0075836
CG17678	FBgn0000384	NA	FBtr0111126
CG17683	FBgn0262115	NA	FBtr0111300
CG17687	FBgn0036348	NA	FBtr0075838
CG17697	FBgn0001085	NA	FBtr0075743
CG17698	FBgn0040056	NA	FBtr0111167
CG17712	FBgn0027597	NA	FBtr0077903
CG17723	FBgn0035432	CG17723	FBtr0073057
CG17725	FBgn0003067	Pepck	FBtr0086700
CG17736	FBgn0036925	schuy	FBtr0074899
CG17739	FBgn0033710	CG17739	FBtr0087893
CG1774	FBgn0039856	NA	FBtr0085798
CG17743	FBgn0002521	NA	FBtr0089204
CG17752	FBgn0038718	CG17752	FBtr0083779
CG17754	FBgn0030114	CG17754	FBtr0071315
CG17767	FBgn0027358	NA	FBtr0074767
CG17778	FBgn0023534	NA	FBtr0070080
CG17784	FBgn0039192	NA	FBtr0084626
CG17786	FBgn0039167	CG17786	FBtr0084572
CG17797	FBgn0015583	NA	FBtr0079716
CG17801	FBgn0038550	NA	FBtr0083525
CG17802	FBgn0038549	NA	FBtr0083526
CG17803	FBgn0038547	NA	FBtr0083528
CG17806	FBgn0038548	NA	FBtr0083527
CG17807	FBgn0034748	NA	FBtr0071938
CG17814	FBgn0040959	NA	FBtr0079713
CG17826	FBgn0036227	NA	FBtr0076042
CG17828	FBgn0016038	mod(r)	FBtr0089726
CG17834	FBgn0028394	CG17834	FBtr0079686
CG17835	FBgn0001269	NA	FBtr0088068
CG17841	FBgn0028480	CG17841	FBtr0071444
CG1785	FBgn0030061	NA	FBtr0071250
CG1786	FBgn0030369	NA	FBtr0073623
CG17867	FBgn0030298	NA	FBtr0073548
CG17868	FBgn0028946	NA	FBtr0080781
CG1787	FBgn0041629	Hexo2	FBtr0071249
CG17870	FBgn0004907	NA	FBtr0088412
CG17876	FBgn0000078	NA	FBtr0086983
CG17883	FBgn0040005	NA	FBtr0111292
CG17885	FBgn0029521	NA	FBtr0070070
CG17896	FBgn0023537	CG17896	FBtr0070093
CG17903	FBgn0000409	Cyt-c-p	FBtr0080889
CG17906	FBgn0032086	NA	FBtr0079762
CG17911	FBgn0037591	NA	FBtr0081899
CG17916	FBgn0038798	NA	FBtr0083887
CG17921	FBgn0010228	NA	FBtr0071674
CG17922	FBgn0034656	NA	FBtr0071675
CG1793	FBgn0039923	NA	FBtr0089254
CG17932	FBgn0040260	Ugt36Bc	FBtr0080912
CG17934	FBgn0004173	NA	FBtr0081586
CG17935	FBgn0004175	NA	FBtr0081588
CG17941	FBgn0000497	NA	FBtr0078045
CG17944	FBgn0037500	NA	FBtr0081590
CG17946	FBgn0004172	NA	FBtr0081584
CG17947	FBgn0010215	NA	FBtr0070038
CG17949	FBgn0061209	NA	FBtr0085927
CG17957	FBgn0003510	Sry-alpha	FBtr0085557

CG17958	FBgn0003512	NA	FBtr0085558
CG17959	FBgn0029647	NA	FBtr0070557
CG1796	FBgn0030365	NA	FBtr0073622
CG17975	FBgn0028562	NA	FBtr0088778
CG17976	FBgn0028561	NA	FBtr0088779
CG17977	FBgn0040778	NA	FBtr0088826
CG17988	FBgn0032536	NA	FBtr0100327
CG17998	FBgn0261988	NA	FBtr0085788
CG17999	FBgn0034552	CG17999	FBtr0071561
CG1800	FBgn0039861	NA	FBtr0085820
CG18000	FBgn0003654	NA	FBtr0089401
CG18001	FBgn0040007	NA	FBtr0111120
CG18004	FBgn0033566	CG18004	FBtr0089729
CG18005	FBgn0037660	CG18005	FBtr0081995
CG18013	FBgn0261976	NA	FBtr0077461
CG18028	FBgn0002566	NA	FBtr0111123
CG1803	FBgn0030362	NA	FBtr0073577
CG18030	FBgn0039778	NA	FBtr0085652
CG18039	FBgn0028422	NA	FBtr0083938
CG1804	FBgn0039862	NA	FBtr0085821
CG1806	FBgn0030360	NA	FBtr0073574
CG18065	FBgn0034519	NA	FBtr0100677
CG18085	FBgn0003366	NA	FBtr0073393
CG18087	FBgn0003377	NA	FBtr0076095
CG1809	FBgn0033423	CG1809	FBtr0088520
CG18096	FBgn0041183	NA	FBtr0080811
CG1810	FBgn0030556	mRNA-capping-enzyme	FBtr0073922
CG18112	FBgn0039702	NA	FBtr0085533
CG1812	FBgn0031119	NA	FBtr0077312
CG18128	FBgn0034898	NA	FBtr0072094
CG18135	FBgn0036837	CG18135	FBtr0075045
CG18139	FBgn0038522	NA	FBtr0083459
CG18140	FBgn0250907	NA	FBtr0111127
CG18143	FBgn0261436	CG18143	FBtr0078986
CG18144	FBgn0032209	NA	FBtr0080073
CG18145	FBgn0032189	NA	FBtr0079962
CG1815	FBgn0039863	NA	FBtr0085822
CG18156	FBgn0035725	CG18156	FBtr0076936
CG18173	FBgn0035265	CG18173	FBtr0072796
CG1821	FBgn0025286	NA	FBtr0088526
CG18212	FBgn0038535	CG18212	FBtr0089414
CG1822	FBgn0014133	NA	FBtr0073527
CG18241	FBgn0032095	NA	FBtr0079768
CG1826	FBgn0030228	NA	FBtr0071497
CG18265	FBgn0036725	NA	FBtr0075219
CG18268	FBgn0037520	NA	FBtr0081772
CG18269	FBgn0031719	NA	FBtr0079103
CG18278	FBgn0033836	NA	FBtr0087716
CG18279	FBgn0033835	NA	FBtr0087655
CG18281	FBgn0037003	NA	FBtr0078206
CG18287	FBgn0039679	NA	FBtr0085451
CG18292	FBgn0030269	NA	FBtr0073437
CG18294	FBgn0036873	NA	FBtr0074984
CG18316	FBgn0033261	CG18316	FBtr0088782
CG18330	FBgn0035231	NA	FBtr0072742
CG18331	FBgn0036181	NA	FBtr0076140
CG18332	FBgn0027055	NA	FBtr0078269

CG18343	FBgn0033683	NA	FBtr0087982
CG18347	FBgn0260743	NA	FBtr0082484
CG1836	FBgn0026777	NA	FBtr0089121
CG18368	FBgn0033864	NA	FBtr0087627
CG18369	FBgn0033860	NA	FBtr0087624
CG18371	FBgn0033893	NA	FBtr0087599
CG18375	FBgn0034606	CG18375	FBtr0071622
CG1838	FBgn0026199	NA	FBtr0089094
CG1839	FBgn0030555	NA	FBtr0073923
CG18397	FBgn0032723	NA	FBtr0081193
CG1840	FBgn0030351	NA	FBtr0073571
CG18410	FBgn0039226	CG18410	FBtr0084741
CG18417	FBgn0035780	CG18417	FBtr0076855
CG18420	FBgn0028866	NA	FBtr0080687
CG18426	FBgn0021895	NA	FBtr0072234
CG1844	FBgn0030350	NA	FBtr0073570
CG18444	FBgn0003863	NA	FBtr0088161
CG18445	FBgn0033476	NA	FBtr0088434
CG18446	FBgn0033458	NA	FBtr0088376
CG1845	FBgn0033155	CG1845	FBtr0089021
CG18455	FBgn0025360	Optix	FBtr0088865
CG18466	FBgn0010222	Nmdmc	FBtr0081996
CG18467	FBgn0034218	NA	FBtr0086918
CG18468	FBgn0034217	NA	FBtr0086917
CG18471	FBgn0024232	NA	FBtr0087109
CG18473	FBgn0037683	CG18473	FBtr0082025
CG18478	FBgn0028517	NA	FBtr0080757
CG18492	FBgn0026323	NA	FBtr0077319
CG18493	FBgn0038701	CG18493	FBtr0083756
CG18505	FBgn0038363	NA	FBtr0083174
CG18507	FBgn0028527	CG18507	FBtr0080528
CG18516	FBgn0038350	NA	FBtr0083150
CG18519	FBgn0038348	NA	FBtr0083153
CG18522	FBgn0038347	CG18522	FBtr0083154
CG1854	FBgn0026389	NA	FBtr0089051
CG18543	FBgn0010431	mtrm	FBtr0076613
CG18545	FBgn0037812	NA	FBtr0082234
CG18549	FBgn0038053	CG18549	FBtr0082632
CG18559	FBgn0041337	NA	FBtr0077748
CG1857	FBgn0002930	NA	FBtr0089052
CG18572	FBgn0003189	NA	FBtr0089734
CG18578	FBgn0040259	NA	FBtr0082338
CG1859	FBgn0044011	NA	FBtr0089053
CG18591	FBgn0261790	NA	FBtr0079560
CG18596	FBgn0038953	CG18596	FBtr0084216
CG18599	FBgn0038592	CG18599	FBtr0083571
CG18600	FBgn0038601	NA	FBtr0083594
CG18607	FBgn0034429	NA	FBtr0086514
CG18608	FBgn0014269	NA	FBtr0086601
CG18617	FBgn0028670	NA	FBtr0083657
CG18619	FBgn0032202	CG18619	FBtr0089735
CG1862	FBgn0040324	NA	FBtr0089075
CG18622	FBgn0038460	NA	FBtr0083377
CG18635	FBgn0034279	NA	FBtr0089557
CG18636	FBgn0032551	NA	FBtr0080688
CG18642	FBgn0025109	NA	FBtr0077643
CG18647	FBgn0045759	NA	FBtr0076900

CG1865	FBgn0024293	NA	FBtr0089054
CG18662	FBgn0040963	NA	FBtr0079760
CG1867	FBgn0039582	NA	FBtr0085288
CG18675	FBgn0040696	CG18675	FBtr0073218
CG1868	FBgn0033427	NA	FBtr0088542
CG18681	FBgn0010425	NA	FBtr0088160
CG18729	FBgn0061476	NA	FBtr0085777
CG1873	FBgn0000557	NA	FBtr0085832
CG18730	FBgn0000079	NA	FBtr0087004
CG18735	FBgn0042098	NA	FBtr0071744
CG18743	FBgn0013276	NA	FBtr0082482
CG18746	FBgn0042103	NA	FBtr0081775
CG18766	FBgn0042111	NA	FBtr0085123
CG18767	FBgn0042112	NA	FBtr0076735
CG18778	FBgn0042119	NA	FBtr0077060
CG18779	FBgn0086611	NA	FBtr0077061
CG1878	FBgn0000278	NA	FBtr0085640
CG18780	FBgn0013531	MED20	FBtr0079544
CG18801	FBgn0041627	NA	FBtr0080763
CG18802	FBgn0011740	alpha-Man-II	FBtr0082031
CG18808	FBgn0042131	NA	FBtr0073361
CG18809	FBgn0042132	NA	FBtr0074810
CG18811	FBgn0042134	NA	FBtr0075098
CG18815	FBgn0042138	CG18815	FBtr0076103
CG18817	FBgn0029508	NA	FBtr0086172
CG18823	FBgn0042146	NA	FBtr0070171
CG1883	FBgn0039757	NA	FBtr0089423
CG18858	FBgn0042175	NA	FBtr0081364
CG18859	FBgn0041626	NA	FBtr0070029
CG18869	FBgn0042179	NA	FBtr0073204
CG1893	FBgn0035390	CG1893	FBtr0073005
CG1897	FBgn0000492	Dr	FBtr0085441
CG1900	FBgn0030391	NA	FBtr0073669
CG1909	FBgn0039911	NA	FBtr0089079
CG1910	FBgn0022349	CG1910	FBtr0085839
CG1913	FBgn0003884	NA	FBtr0081639
CG1919	FBgn0035281	CG1919	FBtr0072869
CG1921	FBgn0014388	sty	FBtr0073145
CG1925	FBgn0002891	NA	FBtr0088831
CG1938	FBgn0030276	NA	FBtr0073442
CG1939	FBgn0037469	CG1939	FBtr0081638
CG1941	FBgn0033214	CG1941	FBtr0088927
CG1942	FBgn0033215	CG1942	FBtr0088928
CG1943	FBgn0037468	NA	FBtr0081635
CG1950	FBgn0030370	NA	FBtr0073638
CG1958	FBgn0029940	NA	FBtr0071046
CG1966	FBgn0027620	Acf1	FBtr0085845
CG1967	FBgn0030341	NA	FBtr0073604
CG1973	FBgn0260990	NA	FBtr0085477
CG1977	FBgn0250789	NA	FBtr0072789
CG1978	FBgn0033404	NA	FBtr0088503
CG1981	FBgn0026869	NA	FBtr0089106
CG1986	FBgn0030162	CG1986	FBtr0071395
CG1994	FBgn0027330	NA	FBtr0071280
CG2004	FBgn0030060	NA	FBtr0071281
CG2009	FBgn0026262	NA	FBtr0089110
CG2010	FBgn0039667	CG2010	FBtr0085427

CG2019	FBgn0029088	NA	FBtr0078665
CG2021	FBgn0035271	NA	FBtr0072791
CG2025	FBgn0030344	NA	FBtr0073601
CG2034	FBgn0015359	NA	FBtr0072877
CG2038	FBgn0028836	NA	FBtr0088742
CG2040	FBgn0010114	hig	FBtr0100119
CG2043	FBgn0002534	NA	FBtr0088743
CG2044	FBgn0002535	NA	FBtr0088744
CG2045	FBgn0019929	NA	FBtr0071387
CG2047	FBgn0001077	NA	FBtr0081625
CG2048	FBgn0002413	dco	FBtr0085744
CG2053	FBgn0039887	NA	FBtr0085873
CG2060	FBgn0014469	Cyp4e2	FBtr0088745
CG2061	FBgn0027498	NA	FBtr0073430
CG2062	FBgn0015034	NA	FBtr0088746
CG2071	FBgn0011834	NA	FBtr0077272
CG2075	FBgn0004372	NA	FBtr0073006
CG2076	FBgn0030263	CG2076	FBtr0073428
CG2078	FBgn0033402	Myd88	FBtr0088598
CG2081	FBgn0030262	NA	FBtr0100644
CG2087	FBgn0037327	PEK	FBtr0078770
CG2092	FBgn0261385	scra	FBtr0088912
CG2096	FBgn0000711	flw	FBtr0071446
CG2097	FBgn0037371	CG2097	FBtr0078723
CG2099	FBgn0037328	RpL35A	FBtr0078769
CG2101	FBgn0035374	NA	FBtr0301626
CG2102	FBgn0004878	cas	FBtr0078725
CG2103	FBgn0035375	pgant6	FBtr0072973
CG2107	FBgn0035383	CG2107	FBtr0072978
CG2110	FBgn0033292	Cyp4ad1	FBtr0088760
CG2113	FBgn0035384	NA	FBtr0072979
CG2114	FBgn0035385	NA	FBtr0072980
CG2124	FBgn0030217	NA	FBtr0071491
CG2144	FBgn0033187	NA	FBtr0088904
CG2145	FBgn0030251	NA	FBtr0073387
CG2151	FBgn0020653	NA	FBtr0071168
CG2155	FBgn0003965	v	FBtr0073386
CG2161	FBgn0017550	Rga	FBtr0078741
CG2171	FBgn0086355	Tpi	FBtr0085582
CG2173	FBgn0021995	NA	FBtr0088788
CG2182	FBgn0037360	NA	FBtr0078730
CG2183	FBgn0033273	NA	FBtr0088789
CG2184	FBgn0002773	Mlc2	FBtr0089747
CG2186	FBgn0030243	NA	FBtr0073381
CG2189	FBgn0000439	NA	FBtr0081621
CG2194	FBgn0086450	NA	FBtr0071338
CG2199	FBgn0035213	CG2199	FBtr0072722
CG2200	FBgn0030447	NA	FBtr0073721
CG2204	FBgn0001122	NA	FBtr0088266
CG2206	FBgn0027280	NA	FBtr0071129
CG2211	FBgn0035211	NA	FBtr0072723
CG2213	FBgn0035210	CG2213	FBtr0072724
CG2221	FBgn0028331	l(1)G0289	FBtr0071470
CG2222	FBgn0030196	NA	FBtr0071465
CG2229	FBgn0039777	NA	FBtr0085690
CG2233	FBgn0029990	CG2233	FBtr0071123
CG2238	FBgn0000559	NA	FBtr0085911

CG2239	FBgn0027654	jdp	FBtr0085681
CG2248	FBgn0010333	Rac1	FBtr0072730
CG2254	FBgn0029994	CG2254	FBtr0071116
CG2257	FBgn0029996	NA	FBtr0071112
CG2258	FBgn0029997	CG2258	FBtr0071109
CG2259	FBgn0040319	NA	FBtr0071107
CG2260	FBgn0030000	CG2260	FBtr0071194
CG2267	FBgn0039792	NA	FBtr0085672
CG2277	FBgn0035204	CG2277	FBtr0072729
CG2304	FBgn0039668	NA	FBtr0085432
CG2310	FBgn0039665	CG2310	FBtr0085434
CG2328	FBgn0000606	NA	FBtr0088390
CG2330	FBgn0037447	CG2330	FBtr0081729
CG2345	FBgn0000552	NA	FBtr0081688
CG2360	FBgn0004783	NA	FBtr0081691
CG2371	FBgn0030323	CG2371	FBtr0073534
CG2374	FBgn0016032	lbm	FBtr0089033
CG2397	FBgn0033304	NA	FBtr0088690
CG2411	FBgn0003892	NA	FBtr0089427
CG2469	FBgn0035205	CG2469	FBtr0072668
CG2493	FBgn0032864	CG2493	FBtr0081363
CG2503	FBgn0010750	atms	FBtr0078868
CG2508	FBgn0032863	NA	FBtr0081362
CG2519	FBgn0037336	NA	FBtr0078761
CG2522	FBgn0010391	NA	FBtr0073480
CG2543	FBgn0030407	NA	FBtr0073655
CG2555	FBgn0030398	CG2555	FBtr0073653
CG2556	FBgn0030396	NA	FBtr0073652
CG2559	FBgn0002562	NA	FBtr0073651
CG2560	FBgn0030394	CG2560	FBtr0073650
CG2577	FBgn0030384	NA	FBtr0073643
CG2615	FBgn0086657	NA	FBtr0081390
CG2616	FBgn0037512	NA	FBtr0081738
CG2641	FBgn0037518	CG2641	FBtr0081745
CG2647	FBgn0003068	NA	FBtr0070477
CG2650	FBgn0000092	NA	FBtr0070504
CG2652	FBgn0025838	NA	FBtr0070505
CG2656	FBgn0037478	CG2656	FBtr0081568
CG2658	FBgn0024992	NA	FBtr0070503
CG2662	FBgn0024993	CG2662	FBtr0070478
CG2668	FBgn0004181	NA	FBtr0072454
CG2681	FBgn0024997	NA	FBtr0070480
CG2682	FBgn0033015	NA	FBtr0086082
CG2684	FBgn0002542	NA	FBtr0081758
CG2691	FBgn0030504	NA	FBtr0073848
CG2692	FBgn0001147	gsb-n	FBtr0072461
CG2699	FBgn0020622	Pi3K21B	FBtr0078132
CG2706	FBgn0000928	NA	FBtr0070484
CG2707	FBgn0000927	NA	FBtr0070485
CG2709	FBgn0024977	NA	FBtr0070486
CG2712	FBgn0024975	NA	FBtr0070488
CG2713	FBgn0250874	CG2713	FBtr0070497
CG2715	FBgn0024980	NA	FBtr0070494
CG2720	FBgn0024352	NA	FBtr0078131
CG2746	FBgn0002607	RpL19	FBtr0072405
CG2759	FBgn0003996	w	FBtr0070490
CG2772	FBgn0031533	NA	FBtr0077516

CG2774	FBgn0031534	CG2774	FBtr0077517
CG2781	FBgn0037534	CG2781	FBtr0081823
CG2791	FBgn0037533	CG2791	FBtr0081824
CG2812	FBgn0034931	CG2812	FBtr0072140
CG2865	FBgn0023526	CG2865	FBtr0070408
CG2871	FBgn0029725	NA	FBtr0070734
CG2890	FBgn0030208	NA	FBtr0071451
CG2899	FBgn0015402	NA	FBtr0078766
CG2902	FBgn0010399	Nmdar1	FBtr0078763
CG2910	FBgn0027548	NA	FBtr0088857
CG2913	FBgn0015565	yin	FBtr0070631
CG2919	FBgn0261004	NA	FBtr0078743
CG2922	FBgn0250753	NA	FBtr0078744
CG2932	FBgn0025679	NA	FBtr0070735
CG2939	FBgn0004567	slp2	FBtr0077500
CG2943	FBgn0037530	NA	FBtr0081765
CG2955	FBgn0031585	NA	FBtr0077468
CG2956	FBgn0003900	twi	FBtr0071953
CG2957	FBgn0037529	mRpS9	FBtr0081766
CG2958	FBgn0040102	NA	FBtr0077469
CG2960	FBgn0003941	NA	FBtr0077470
CG2961	FBgn0030187	lpod	FBtr0071484
CG2970	FBgn0034936	NA	FBtr0072145
CG2980	FBgn0034939	thoc5	FBtr0072147
CG2984	FBgn0022768	NA	FBtr0070685
CG2988	FBgn0000576	NA	FBtr0082863
CG2991	FBgn0031474	NA	FBtr0077625
CG2993	FBgn0037521	NA	FBtr0081771
CG2995	FBgn0040372	NA	FBtr0070063
CG3000	FBgn0262699	rap	FBtr0070686
CG30020	FBgn0050020	NA	FBtr0088235
CG30023	FBgn0082585	sprt	FBtr0088146
CG30025	FBgn0050025	NA	FBtr0088158
CG30030	FBgn0041241	NA	FBtr0088127
CG30039	FBgn0050039	NA	FBtr0088023
CG30040	FBgn0086677	NA	FBtr0088021
CG30043	FBgn0050043	NA	FBtr0087907
CG30046	FBgn0050046	NA	FBtr0087895
CG30048	FBgn0050048	NA	FBtr0087919
CG30074	FBgn0050074	NA	FBtr0087554
CG30076	FBgn0050076	NA	FBtr0087513
CG30081	FBgn0050081	NA	FBtr0087365
CG30085	FBgn0050085	NA	FBtr0087306
CG30087	FBgn0050087	NA	FBtr0100573
CG30090	FBgn0050090	NA	FBtr0087279
CG30094	FBgn0050094	NA	FBtr0087233
CG30103	FBgn0050103	NA	FBtr0086929
CG30104	FBgn0050104	NA	FBtr0086928
CG30110	FBgn0050110	NA	FBtr0086820
CG30116	FBgn0028496	CG30116	FBtr0086720
CG30125	FBgn0050125	NA	FBtr0086595
CG30129	FBgn0046880	NA	FBtr0086480
CG30145	FBgn0050145	NA	FBtr0086265
CG30147	FBgn0050147	NA	FBtr0086255
CG30148	FBgn0050148	NA	FBtr0086266
CG30149	FBgn0250850	NA	FBtr0086252
CG30151	FBgn0050151	NA	FBtr0086269

CG30154	FBgn0050154	NA	FBtr0086215
CG30158	FBgn0050158	NA	FBtr0086165
CG3016	FBgn0029819	NA	FBtr0070828
CG30163	FBgn0050163	NA	FBtr0072334
CG30170	FBgn0004581	bgcn	FBtr0072232
CG30172	FBgn0050172	NA	FBtr0072193
CG30181	FBgn0050181	NA	FBtr0072116
CG30185	FBgn0050185	CG30185	FBtr0072060
CG30186	FBgn0041235	NA	FBtr0071977
CG30189	FBgn0045483	NA	FBtr0071964
CG30191	FBgn0045482	NA	FBtr0071965
CG30192	FBgn0050192	NA	FBtr0071967
CG30197	FBgn0050197	NA	FBtr0087494
CG3024	FBgn0025615	torp4a	FBtr0070690
CG3025	FBgn0014340	NA	FBtr0070829
CG3027	FBgn0037513	pyd3	FBtr0081780
CG30272	FBgn0050272	NA	FBtr0071890
CG30274	FBgn0050274	NA	FBtr0071888
CG30279	FBgn0050279	NA	FBtr0071768
CG3028	FBgn0016672	Ipp	FBtr0082865
CG30284	FBgn0050284	NA	FBtr0071684
CG30285	FBgn0050285	NA	FBtr0071678
CG30289	FBgn0050289	NA	FBtr0071697
CG30325	FBgn0050325	NA	FBtr0086821
CG30329	FBgn0028669	NA	FBtr0086602
CG30330	FBgn0041236	NA	FBtr0071976
CG30349	FBgn0050349	CG30349	FBtr0088650
CG30350	FBgn0050350	NA	FBtr0088636
CG30355	FBgn0050355	NA	FBtr0088726
CG30356	FBgn0050356	NA	FBtr0088717
CG3036	FBgn0031645	CG3036	FBtr0077391
CG30364	FBgn0050364	NA	FBtr0088772
CG3038	FBgn0040373	CG3038	FBtr0070108
CG30384	FBgn0050384	NA	FBtr0089048
CG30385	FBgn0050385	NA	FBtr0089047
CG30388	FBgn0034590	NA	FBtr0071636
CG30396	FBgn0041239	NA	FBtr0071728
CG3041	FBgn0015270	Orc2	FBtr0082866
CG30411	FBgn0050411	NA	FBtr0072016
CG30421	FBgn0050421	NA	FBtr0072369
CG30430	FBgn0050430	NA	FBtr0072448
CG30440	FBgn0050440	NA	FBtr0086076
CG30457	FBgn0050457	NA	FBtr0086998
CG30468	FBgn0050468	NA	FBtr0087329
CG3047	FBgn0003372	NA	FBtr0077393
CG30480	FBgn0050480	NA	FBtr0087478
CG30481	FBgn0050481	NA	FBtr0100285
CG30484	FBgn0050484	NA	FBtr0087639
CG30489	FBgn0050489	NA	FBtr0089419
CG3051	FBgn0023169	NA	FBtr0070217
CG3052	FBgn0011277	NA	FBtr0070691
CG3057	FBgn0019830	NA	FBtr0077731
CG3060	FBgn0002791	NA	FBtr0072156
CG3061	FBgn0038195	CG3061	FBtr0082869
CG3062	FBgn0025612	NA	FBtr0070692
CG3068	FBgn0000147	NA	FBtr0082483
CG3073	FBgn0027296	l(1)G0144	FBtr0070414

CG3074	FBgn0034709	CG3074	FBtr0071784
CG3077	FBgn0031457	CG3077	FBtr0077736
CG3078	FBgn0023524	NA	FBtr0070415
CG3081	FBgn0025613	NA	FBtr0070693
CG3082	FBgn0021979	l(2)k09913	FBtr0071960
CG3090	FBgn0005612	NA	FBtr0072157
CG3092	FBgn0034835	NA	FBtr0071999
CG31005	FBgn0051005	CG31005	FBtr0085769
CG31007	FBgn0051007	NA	FBtr0085747
CG31008	FBgn0051008	NA	FBtr0085748
CG31010	FBgn0051010	NA	FBtr0085725
CG31019	FBgn0051019	NA	FBtr0085665
CG31022	FBgn0039776	PH4alphaEFB	FBtr0085650
CG31029	FBgn0051029	NA	FBtr0085585
CG31037	FBgn0000247	ca	FBtr0085484
CG31038	FBgn0051038	CG31038	FBtr0085496
CG3104	FBgn0031473	CG3104	FBtr0077686
CG3105	FBgn0034950	CG3105	FBtr0072160
CG31055	FBgn0051055	NA	FBtr0085243
CG31057	FBgn0051057	NA	FBtr0085197
CG31058	FBgn0051058	NA	FBtr0085198
CG31059	FBgn0046887	NA	FBtr0085186
CG3106	FBgn0030148	NA	FBtr0071403
CG31060	FBgn0046886	NA	FBtr0085187
CG31061	FBgn0046885	NA	FBtr0085188
CG3107	FBgn0033005	NA	FBtr0086078
CG31080	FBgn0051080	NA	FBtr0085032
CG31088	FBgn0051088	NA	FBtr0083241
CG3109	FBgn0023519	mRpL16	FBtr0070314
CG31096	FBgn0039354	NA	FBtr0084899
CG31100	FBgn0051100	CG31100	FBtr0082011
CG31108	FBgn0051108	CG31108	FBtr0084802
CG31121	FBgn0051121	CG31121	FBtr0084723
CG31127	FBgn0046685	NA	FBtr0084669
CG31128	FBgn0051128	NA	FBtr0084638
CG31137	FBgn0011725	NA	FBtr0084568
CG31148	FBgn0051148	NA	FBtr0084441
CG31150	FBgn0051150	CG31150	FBtr0083189
CG31164	FBgn0051164	NA	FBtr0084193
CG31173	FBgn0045469	NA	FBtr0084159
CG31176	FBgn0051176	NA	FBtr0084114
CG31183	FBgn0051183	NA	FBtr0083187
CG31193	FBgn0044810	NA	FBtr0083985
CG31198	FBgn0051198	NA	FBtr0084128
CG31208	FBgn0045471	NA	FBtr0083865
CG3121	FBgn0034957	NA	FBtr0072161
CG31211	FBgn0051211	NA	FBtr0082480
CG31212	FBgn0086613	NA	FBtr0083771
CG31213	FBgn0051213	NA	FBtr0083824
CG31216	FBgn0051216	CG31216	FBtr0083786
CG31221	FBgn0051221	NA	FBtr0083762
CG31224	FBgn0051224	NA	FBtr0083691
CG31226	FBgn0051226	NA	FBtr0083675
CG31233	FBgn0051233	CG31233	FBtr0084127
CG31235	FBgn0051235	CG31235	FBtr0083588
CG31245	FBgn0051245	NA	FBtr0083773
CG3127	FBgn0250906	Pgk	FBtr0077739

CG31274	FBgn0051274	NA	FBtr0083368
CG31275	FBgn0063261	CG31275	FBtr0083345
CG31280	FBgn0041225	NA	FBtr0084346
CG31284	FBgn0260005	CG31284	FBtr0081755
CG31287	FBgn0051287	NA	FBtr0083256
CG31289	FBgn0024558	Dph5	FBtr0084280
CG31293	FBgn0003227	NA	FBtr0083193
CG31294	FBgn0051294	NA	FBtr0083198
CG31298	FBgn0038092	NA	FBtr0082684
CG31300	FBgn0051300	NA	FBtr0084824
CG31313	FBgn0051313	NA	FBtr0082982
CG3132	FBgn0260746	Ect3	FBtr0082487
CG31320	FBgn0051320	CG31320	FBtr0082943
CG31322	FBgn0027083	Aats-met	FBtr0082895
CG31335	FBgn0045468	NA	FBtr0084158
CG31344	FBgn0051344	CG31344	FBtr0083136
CG31359	FBgn0013278	NA	FBtr0082637
CG3136	FBgn0033010	NA	FBtr0086080
CG31362	FBgn0003357	NA	FBtr0085502
CG31365	FBgn0051365	CG31365	FBtr0084406
CG31366	FBgn0013275	NA	FBtr0082512
CG3139	FBgn0004242	NA	FBtr0077726
CG3140	FBgn0022708	Adk2	FBtr0072163
CG31404	FBgn0051404	NA	FBtr0083774
CG31405	FBgn0045473	NA	FBtr0082216
CG31406	FBgn0051406	NA	FBtr0082236
CG31423	FBgn0051423	NA	FBtr0084248
CG31424	FBgn0051424	NA	FBtr0084249
CG31438	FBgn0051438	NA	FBtr0084090
CG31446	FBgn0051446	NA	FBtr0083163
CG31447	FBgn0043069	NA	FBtr0083367
CG31449	FBgn0013277	NA	FBtr0082679
CG31450	FBgn0051450	NA	FBtr0081897
CG31453	FBgn0051453	CG31453	FBtr0081898
CG31457	FBgn0051457	NA	FBtr0084405
CG31459	FBgn0051459	NA	FBtr0083812
CG31460	FBgn0051460	NA	FBtr0081873
CG31462	FBgn0051462	NA	FBtr0081851
CG31463	FBgn0051463	CG31463	FBtr0081831
CG31468	FBgn0047351	NA	FBtr0084473
CG31475	FBgn0051475	NA	FBtr0083735
CG31478	FBgn0038319	mRpL9	FBtr0083084
CG31481	FBgn0051481	NA	FBtr0089276
CG31482	FBgn0051482	NA	FBtr0081679
CG31483	FBgn0046873	NA	FBtr0081676
CG31495	FBgn0051495	NA	FBtr0082882
CG31507	FBgn0044809	NA	FBtr0083974
CG31508	FBgn0044812	NA	FBtr0083972
CG31509	FBgn0028396	NA	FBtr0083971
CG31510	FBgn0051510	NA	FBtr0084783
CG31516	FBgn0051516	NA	FBtr0078987
CG31519	FBgn0041621	NA	FBtr0078977
CG31523	FBgn0051523	NA	FBtr0078937
CG31528	FBgn0051528	NA	FBtr0078923
CG31530	FBgn0051530	NA	FBtr0078882
CG31543	FBgn0086689	Hph	FBtr0078780
CG31544	FBgn0051544	NA	FBtr0081532

CG31547	FBgn0051547	CG31547	FBtr0078764
CG31551	FBgn0051551	NA	FBtr0078706
CG31556	FBgn0051556	NA	FBtr0078612
CG31558	FBgn0046875	NA	FBtr0078617
CG3156	FBgn0023536	NA	FBtr0070079
CG31560	FBgn0051560	NA	FBtr0078609
CG31561	FBgn0051561	NA	FBtr0078608
CG31562	FBgn0051562	NA	FBtr0078589
CG3157	FBgn0260639	gammaTub23C	FBtr0077641
CG3158	FBgn0003483	NA	FBtr0083183
CG3160	FBgn0029789	NA	FBtr0070847
CG31611	FBgn0051611	NA	FBtr0085926
CG31613	FBgn0051613	NA	FBtr0085894
CG31617	FBgn0051617	NA	FBtr0085892
CG31618	FBgn0051618	NA	FBtr0085893
CG3162	FBgn0034834	NA	FBtr0072052
CG31620	FBgn0041245	NA	FBtr0081522
CG31622	FBgn0041244	NA	FBtr0081509
CG31623	FBgn0023090	NA	FBtr0081516
CG31626	FBgn0051626	NA	FBtr0081485
CG31628	FBgn0000053	NA	FBtr0079431
CG3163	FBgn0034961	CG3163	FBtr0072165
CG31632	FBgn0051632	CG31632	FBtr0079341
CG31634	FBgn0051634	Oatp26F	FBtr0079327
CG31637	FBgn0051637	NA	FBtr0079287
CG31639	FBgn0051639	NA	FBtr0079208
CG31643	FBgn0051643	NA	FBtr0079182
CG3166	FBgn0000097	aop	FBtr0077850
CG31661	FBgn0051661	NA	FBtr0077932
CG31662	FBgn0045501	NA	FBtr0077897
CG3167	FBgn0034962	NA	FBtr0072166
CG31670	FBgn0031375	CG31670	FBtr0077870
CG31676	FBgn0051676	CG31676	FBtr0081424
CG31683	FBgn0051683	NA	FBtr0081365
CG31689	FBgn0031449	CG31689	FBtr0077706
CG31691	FBgn0044811	NA	FBtr0081310
CG31692	FBgn0032820	fbp	FBtr0081322
CG31693	FBgn0051693	NA	FBtr0085931
CG31697	FBgn0051697	NA	FBtr0081250
CG31709	FBgn0051709	NA	FBtr0079856
CG31718	FBgn0051718	NA	FBtr0080015
CG31719	FBgn0051719	NA	FBtr0080040
CG31739	FBgn0051739	NA	FBtr0080919
CG31741	FBgn0051741	NA	FBtr0081032
CG31744	FBgn0045486	NA	FBtr0080963
CG31747	FBgn0045487	NA	FBtr0080962
CG31748	FBgn0045485	NA	FBtr0080964
CG31750	FBgn0046888	NA	FBtr0080965
CG31751	FBgn0086909	NA	FBtr0081092
CG31752	FBgn0051752	NA	FBtr0081119
CG31777	FBgn0051777	NA	FBtr0077542
CG31789	FBgn0051789	NA	FBtr0081071
CG3180	FBgn0262955	RpII140	FBtr0082892
CG31800	FBgn0051800	NA	FBtr0081179
CG31810	FBgn0051810	NA	FBtr0080925
CG31811	FBgn0028509	cenG1A	FBtr0080546
CG31813	FBgn0051813	NA	FBtr0080531

CG31814	FBgn0051814	NA	FBtr0080580
CG31819	FBgn0028893	NA	FBtr0080821
CG3182	FBgn0003353	NA	FBtr0072170
CG31823	FBgn0051823	NA	FBtr0080810
CG31826	FBgn0051826	NA	FBtr0080762
CG31827	FBgn0051827	NA	FBtr0080759
CG31832	FBgn0051832	NA	FBtr0080726
CG31849	FBgn0051849	NA	FBtr0080490
CG3186	FBgn0034967	eIF-5A	FBtr0072173
CG31862	FBgn0051862	NA	FBtr0080350
CG3187	FBgn0029783	Sirt4	FBtr0070851
CG31870	FBgn0051870	NA	FBtr0080138
CG31871	FBgn0051871	CG31871	FBtr0080104
CG31876	FBgn0051876	NA	FBtr0079897
CG3189	FBgn0033083	NA	FBtr0086146
CG31898	FBgn0051898	NA	FBtr0079662
CG31904	FBgn0260479	NA	FBtr0079501
CG31908	FBgn0051908	NA	FBtr0079429
CG31910	FBgn0051910	NA	FBtr0079304
CG3192	FBgn0029888	NA	FBtr0070990
CG31920	FBgn0051920	NA	FBtr0078097
CG31921	FBgn0051921	NA	FBtr0078096
CG31926	FBgn0051926	NA	FBtr0077931
CG31929	FBgn0045499	NA	FBtr0077899
CG31931	FBgn0045500	NA	FBtr0100188
CG31932	FBgn0041249	NA	FBtr0077902
CG31936	FBgn0045497	NA	FBtr0077888
CG3195	FBgn0034968	RpL12	FBtr0072174
CG31958	FBgn0051958	NA	FBtr0077495
CG31961	FBgn0051961	NA	FBtr0077485
CG31962	FBgn0020376	NA	FBtr0077466
CG3198	FBgn0029887	NA	FBtr0070991
CG31991	FBgn0004797	mdy	FBtr0080917
CG32006	FBgn0052006	NA	FBtr0089170
CG32022	FBgn0052022	NA	FBtr0076597
CG3203	FBgn0029897	NA	FBtr0070982
CG32037	FBgn0052037	CG32037	FBtr0076469
CG32050	FBgn0052050	NA	FBtr0076338
CG32055	FBgn0052055	NA	FBtr0076304
CG32056	FBgn0052056	CG32056	FBtr0076301
CG32057	FBgn0052057	NA	FBtr0076310
CG32058	FBgn0052058	NA	FBtr0076293
CG3206	FBgn0023523	NA	FBtr0070397
CG32066	FBgn0052066	CG32066	FBtr0076278
CG32067	FBgn0010762	simj	FBtr0076263
CG32071	FBgn0052071	NA	FBtr0076231
CG32077	FBgn0014368	NA	FBtr0076214
CG32085	FBgn0052085	NA	FBtr0076133
CG32088	FBgn0052088	NA	FBtr0076116
CG32091	FBgn0052091	NA	FBtr0076106
CG3210	FBgn0026479	NA	FBtr0077744
CG32103	FBgn0052103	NA	FBtr0075984
CG32104	FBgn0052104	NA	FBtr0075986
CG32105	FBgn0052105	CG32105	FBtr0075953
CG3212	FBgn0031547	NA	FBtr0077560
CG32120	FBgn0002573	NA	FBtr0075862
CG32132	FBgn0052132	NA	FBtr0075800

CG32133	FBgn0052133	NA	FBtr0075784
CG32134	FBgn0005592	NA	FBtr0075778
CG32135	FBgn0263232	NA	FBtr0075760
CG32137	FBgn0052137	CG32137	FBtr0075758
CG32139	FBgn0042630	Sox21b	FBtr0075747
CG32146	FBgn0041604	dlp	FBtr0075680
CG32147	FBgn0047178	NA	FBtr0075647
CG32148	FBgn0047338	NA	FBtr0075630
CG3215	FBgn0034825	NA	FBtr0071974
CG32152	FBgn0052152	NA	FBtr0075489
CG32154	FBgn0263608	NA	FBtr0075460
CG32164	FBgn0042177	NA	FBtr0075313
CG32165	FBgn0042178	NA	FBtr0075312
CG3217	FBgn0025676	NA	FBtr0072603
CG32176	FBgn0052176	CG32176	FBtr0075218
CG32177	FBgn0052177	NA	FBtr0075210
CG32179	FBgn0052179	Krn	FBtr0075197
CG3218	FBgn0000810	NA	FBtr0070396
CG32182	FBgn0052182	NA	FBtr0075188
CG32183	FBgn0052183	NA	FBtr0075186
CG32188	FBgn0052188	NA	FBtr0075164
CG32189	FBgn0052189	NA	FBtr0075163
CG3219	FBgn0034824	NA	FBtr0071975
CG32198	FBgn0052198	NA	FBtr0075099
CG32199	FBgn0052199	NA	FBtr0075106
CG32202	FBgn0052202	NA	FBtr0075051
CG32209	FBgn0260653	CG32209	FBtr0074953
CG32210	FBgn0262517	NA	FBtr0074974
CG32212	FBgn0052212	NA	FBtr0074932
CG32226	FBgn0052226	NA	FBtr0074846
CG32227	FBgn0052227	NA	FBtr0074848
CG32228	FBgn0052228	NA	FBtr0074836
CG3223	FBgn0037538	CG3223	FBtr0081785
CG32230	FBgn0052230	NA	FBtr0070040
CG32232	FBgn0052232	NA	FBtr0073373
CG32235	FBgn0047330	NA	FBtr0073355
CG32237	FBgn0052237	CG32237	FBtr0073356
CG3225	FBgn0031631	CG3225	FBtr0077416
CG32251	FBgn0052251	NA	FBtr0073286
CG32253	FBgn0035524	CG11583	FBtr0073242
CG3227	FBgn0031434	insv	FBtr0077746
CG32274	FBgn0052274	NA	FBtr0073081
CG32278	FBgn0052278	CG32278	FBtr0073033
CG3228	FBgn0001330	NA	FBtr0070419
CG32280	FBgn0052280	NA	FBtr0073032
CG32281	FBgn0052281	NA	FBtr0073034
CG32313	FBgn0052313	NA	FBtr0072830
CG32315	FBgn0024510	dlt	FBtr0072790
CG32344	FBgn0052344	CG32344	FBtr0072576
CG32350	FBgn0052350	NA	FBtr0070046
CG32354	FBgn0052354	CG32354	FBtr0076662
CG32356	FBgn0001253	ImpE1	FBtr0076606
CG32365	FBgn0052365	NA	FBtr0076746
CG32371	FBgn0052371	NA	FBtr0076788
CG32372	FBgn0052372	CG32372	FBtr0076793
CG32374	FBgn0052374	NA	FBtr0076828
CG32376	FBgn0052376	NA	FBtr0076827

CG3238	FBgn0031540	CG3238	FBtr0077569
CG32380	FBgn0052380	NA	FBtr0076870
CG3239	FBgn0029769	NA	FBtr0070793
CG32392	FBgn0052392	CG32392	FBtr0076948
CG32401	FBgn0041625	NA	FBtr0077004
CG32402	FBgn0041624	NA	FBtr0077005
CG32403	FBgn0041623	NA	FBtr0077006
CG32406	FBgn0052406	NA	FBtr0077037
CG3241	FBgn0005616	NA	FBtr0077571
CG32417	FBgn0040298	NA	FBtr0077118
CG32418	FBgn0052418	CG32418	FBtr0077119
CG3242	FBgn0004892	sob	FBtr0077558
CG32432	FBgn0052432	NA	FBtr0078268
CG32435	FBgn0021760	NA	FBtr0089461
CG3244	FBgn0031629	CG3244	FBtr0077417
CG32440	FBgn0052440	NA	FBtr0078345
CG32443	FBgn0003042	Pc	FBtr0078405
CG32446	FBgn0052446	NA	FBtr0078426
CG32447	FBgn0052447	NA	FBtr0078460
CG3245	FBgn0025573	NA	FBtr0071734
CG32453	FBgn0052453	NA	FBtr0078521
CG3246	FBgn0031538	CG3246	FBtr0077572
CG32475	FBgn0052475	NA	FBtr0072462
CG32479	FBgn0052479	NA	FBtr0072589
CG3248	FBgn0031536	CG3248	FBtr0077573
CG32484	FBgn0052484	NA	FBtr0072984
CG32485	FBgn0052485	NA	FBtr0072985
CG32486	FBgn0052486	CG32486	FBtr0073004
CG32499	FBgn0052499	CG32499	FBtr0077362
CG32505	FBgn0023177	NA	FBtr0077325
CG3252	FBgn0029762	NA	FBtr0070797
CG32528	FBgn0052528	NA	FBtr0074793
CG32532	FBgn0052532	CG32532	FBtr0074778
CG32533	FBgn0052533	NA	FBtr0074786
CG32536	FBgn0052536	NA	FBtr0074686
CG32544	FBgn0052544	NA	FBtr0074644
CG32548	FBgn0052548	CG32548	FBtr0074568
CG32549	FBgn0052549	CG32549	FBtr0074573
CG32555	FBgn0026375	NA	FBtr0074482
CG32556	FBgn0263258	NA	FBtr0074477
CG32563	FBgn0052563	NA	FBtr0074391
CG32572	FBgn0052572	NA	FBtr0074324
CG32579	FBgn0052579	NA	FBtr0074225
CG32580	FBgn0052580	NA	FBtr0074205
CG32588	FBgn0052588	NA	FBtr0074025
CG3260	FBgn0021875	NA	FBtr0072253
CG32600	FBgn0052600	NA	FBtr0073913
CG32602	FBgn0052602	NA	FBtr0073911
CG32604	FBgn0026713	l(1)G0007	FBtr0073900
CG32635	FBgn0052635	NA	FBtr0073780
CG3264	FBgn0034712	NA	FBtr0071781
CG32643	FBgn0052643	NA	FBtr0073716
CG32645	FBgn0052645	NA	FBtr0073725
CG32652	FBgn0052652	NA	FBtr0073671
CG32656	FBgn0052656	CG32656	FBtr0089803
CG32664	FBgn0045502	NA	FBtr0073549
CG32668	FBgn0052668	NA	FBtr0073469

CG32676	FBgn0052676	NA	FBtr0071503
CG32679	FBgn0052679	NA	FBtr0071469
CG3268	FBgn0028579	phtf	FBtr0086125
CG32681	FBgn0052681	NA	FBtr0071466
CG32683	FBgn0052683	NA	FBtr0071463
CG32687	FBgn0052687	CG32687	FBtr0071458
CG3269	FBgn0014009	Rab2	FBtr0086124
CG32693	FBgn0052693	NA	FBtr0071420
CG32695	FBgn0052695	NA	FBtr0071398
CG32700	FBgn0052700	NA	FBtr0071374
CG32701	FBgn0028327	l(1)G0320	FBtr0071372
CG32704	FBgn0052704	NA	FBtr0071333
CG32721	FBgn0027553	NA	FBtr0071077
CG3273	FBgn0013732	sced	FBtr0086110
CG32742	FBgn0028360	NA	FBtr0070950
CG32750	FBgn0052750	NA	FBtr0070881
CG32762	FBgn0052762	NA	FBtr0070791
CG32779	FBgn0052779	NA	FBtr0070601
CG32783	FBgn0052783	NA	FBtr0070615
CG32786	FBgn0052786	NA	FBtr0070614
CG32789	FBgn0260484	NA	FBtr0070600
CG32791	FBgn0052791	NA	FBtr0070588
CG32793	FBgn0052793	NA	FBtr0070550
CG32797	FBgn0052797	NA	FBtr0070461
CG32801	FBgn0052801	NA	FBtr0070355
CG32808	FBgn0052808	NA	FBtr0070295
CG3281	FBgn0260741	NA	FBtr0082511
CG32810	FBgn0025394	NA	FBtr0070260
CG32823	FBgn0052823	NA	FBtr0089465
CG32825	FBgn0062565	NA	FBtr0070003
CG32829	FBgn0052829	NA	FBtr0079370
CG32830	FBgn0052830	NA	FBtr0080205
CG32850	FBgn0052850	NA	FBtr0089123
CG32853	FBgn0045442	NA	FBtr0082746
CG32854	FBgn0044511	NA	FBtr0082764
CG3289	FBgn0016698	Ptpa	FBtr0077589
CG3290	FBgn0034711	NA	FBtr0071782
CG3292	FBgn0034710	NA	FBtr0071783
CG32983	FBgn0052983	NA	FBtr0079737
CG32984	FBgn0052984	NA	FBtr0079758
CG32985	FBgn0052985	NA	FBtr0079757
CG3299	FBgn0004397	Vinc	FBtr0070420
CG33007	FBgn0053007	NA	FBtr0087778
CG3301	FBgn0038878	NA	FBtr0084101
CG33012	FBgn0053012	NA	FBtr0087910
CG33048	FBgn0263241	NA	FBtr0076204
CG33054	FBgn0053054	NA	FBtr0078317
CG33056	FBgn0053056	CG33056	FBtr0078311
CG33080	FBgn0053080	CG33080	FBtr0070844
CG33083	FBgn0041224	NA	FBtr0085081
CG33087	FBgn0053087	NA	FBtr0088791
CG33090	FBgn0028916	NA	FBtr0080624
CG33098	FBgn0053098	NA	FBtr0082579
CG33101	FBgn0013998	NA	FBtr0082883
CG33110	FBgn0053110	CG33110	FBtr0084264
CG33111	FBgn0053111	NA	FBtr0084529
CG33116	FBgn0053116	NA	FBtr0081249

CG33117	FBgn0053117	NA	FBtr0081309
CG33120	FBgn0053120	NA	FBtr0081118
CG33123	FBgn0053123	NA	FBtr0077562
CG33136	FBgn0053136	NA	FBtr0086653
CG33139	FBgn0053139	NA	FBtr0087278
CG33140	FBgn0053140	NA	FBtr0089046
CG33143	FBgn0053143	NA	FBtr0071858
CG33145	FBgn0053145	NA	FBtr0088048
CG33150	FBgn0041234	NA	FBtr0072059
CG33151	FBgn0041233	NA	FBtr0072115
CG33152	FBgn0008636	NA	FBtr0071556
CG33155	FBgn0053155	NA	FBtr0100453
CG33158	FBgn0053158	NA	FBtr0075390
CG33159	FBgn0053159	NA	FBtr0073104
CG33166	FBgn0020248	NA	FBtr0072712
CG33177	FBgn0053177	NA	FBtr0073962
CG33178	FBgn0053178	NA	FBtr0073963
CG3318	FBgn0019643	NA	FBtr0072255
CG33194	FBgn0053194	NA	FBtr0079676
CG33198	FBgn0053198	NA	FBtr0089842
CG33202	FBgn0053202	NA	FBtr0081704
CG33205	FBgn0053205	NA	FBtr0076331
CG33207	FBgn0053207	pxb	FBtr0083162
CG33214	FBgn0053214	NA	FBtr0078388
CG3322	FBgn0002528	NA	FBtr0076382
CG33230	FBgn0053230	NA	FBtr0072757
CG33232	FBgn0035347	NA	FBtr0100539
CG33235	FBgn0030307	NA	FBtr0073545
CG3324	FBgn0000442	Pkg21D	FBtr0078042
CG33257	FBgn0053257	CG33257	FBtr0075378
CG33259	FBgn0036495	NA	FBtr0075615
CG33264	FBgn0041622	NA	FBtr0075901
CG33265	FBgn0053265	NA	FBtr0076063
CG33268	FBgn0036177	NA	FBtr0076145
CG3327	FBgn0020445	NA	FBtr0089979
CG33272	FBgn0053272	NA	FBtr0076159
CG3328	FBgn0034985	CG3328	FBtr0072257
CG33291	FBgn0053291	NA	FBtr0078427
CG33292	FBgn0053292	NA	FBtr0078428
CG3330	FBgn0039511	NA	FBtr0085170
CG33302	FBgn0053302	NA	FBtr0079936
CG33310	FBgn0053310	NA	FBtr0080698
CG3332	FBgn0031514	CG3332	FBtr0077609
CG33320	FBgn0067312	NA	FBtr0081453
CG33349	FBgn0053349	NA	FBtr0086169
CG3335	FBgn0036018	NA	FBtr0076383
CG33351	FBgn0066293	NA	FBtr0086192
CG3338	FBgn0031598	NA	FBtr0077462
CG3340	FBgn0001325	Kr	FBtr0072449
CG33453	FBgn0053453	NA	FBtr0086564
CG33454	FBgn0053454	NA	FBtr0086565
CG33463	FBgn0053463	NA	FBtr0087301
CG33466	FBgn0259878	NA	FBtr0087398
CG33467	FBgn0053467	NA	FBtr0089861
CG33474	FBgn0053474	NA	FBtr0088260
CG33475	FBgn0053475	NA	FBtr0088318
CG33476	FBgn0053476	NA	FBtr0088319

CG33477	FBgn0053477	NA	FBtr0088320
CG3348	FBgn0040609	NA	FBtr0085172
CG33485	FBgn0038282	NA	FBtr0089376
CG33487	FBgn0053487	NA	FBtr0089464
CG33491	FBgn0053491	NA	FBtr0089603
CG33493	FBgn0053493	NA	FBtr0089606
CG33494	FBgn0053494	NA	FBtr0089607
CG33496	FBgn0053496	NA	FBtr0089609
CG33497	FBgn0053497	NA	FBtr0089610
CG33498	FBgn0053498	NA	FBtr0089611
CG33499	FBgn0053499	NA	FBtr0089612
CG3350	FBgn0039509	NA	FBtr0085173
CG33500	FBgn0053500	NA	FBtr0076094
CG33503	FBgn0053503	NA	FBtr0089418
CG33508	FBgn0053508	NA	FBtr0091448
CG3351	FBgn0038234	NA	FBtr0082953
CG33511	FBgn0053511	NA	FBtr0091451
CG33517	FBgn0053517	NA	FBtr0070034
CG3352	FBgn0001075	ft	FBtr0077478
CG33524	FBgn0261293	NA	FBtr0091479
CG33526	FBgn0053526	PNUTS	FBtr0091489
CG3353	FBgn0038869	NA	FBtr0084108
CG33548	FBgn0053548	NA	FBtr0070422
CG33550	FBgn0261292	NA	FBtr0091520
CG33552	FBgn0053552	NA	FBtr0091522
CG33557	FBgn0053557	NA	FBtr0091534
CG3356	FBgn0034989	CG3356	FBtr0072262
CG33627	FBgn0053627	NA	FBtr0091604
CG33630	FBgn0053630	NA	FBtr0091607
CG33631	FBgn0053631	NA	FBtr0091608
CG33640	FBgn0053640	NA	FBtr0091617
CG33643	FBgn0053643	NA	FBtr0091620
CG33644	FBgn0053644	NA	FBtr0091621
CG33645	FBgn0053645	NA	FBtr0091622
CG33647	FBgn0053647	NA	FBtr0091624
CG33648	FBgn0053648	NA	FBtr0091625
CG33654	FBgn0053654	NA	FBtr0091633
CG33658	FBgn0053658	NA	FBtr0091637
CG33670	FBgn0064123	NA	FBtr0091649
CG33671	FBgn0061359	NA	FBtr0091650
CG33672	FBgn0061360	NA	FBtr0091651
CG3368	FBgn0039508	NA	FBtr0085163
CG33687	FBgn0053687	NA	FBtr0091667
CG33688	FBgn0053688	NA	FBtr0091668
CG33689	FBgn0053689	NA	FBtr0091669
CG33690	FBgn0053690	NA	FBtr0091670
CG33692	FBgn0064121	NA	FBtr0091675
CG33694	FBgn0040233	NA	FBtr0091678
CG33695	FBgn0052831	NA	FBtr0091679
CG33698	FBgn0261291	NA	FBtr0091686
CG33702	FBgn0053702	NA	FBtr0091690
CG33703	FBgn0053703	NA	FBtr0091691
CG3371	FBgn0035153	CG3371	FBtr0072610
CG33713	FBgn0064116	NA	FBtr0091702
CG33714	FBgn0064117	NA	FBtr0091703
CG33723	FBgn0053723	NA	FBtr0091721
CG33724	FBgn0262595	NA	FBtr0091722

CG3373	FBgn0015737	Hmu	FBtr0085164
CG33752	FBgn0053752	NA	FBtr0091753
CG33757	FBgn0053757	NA	FBtr0091758
CG33758	FBgn0053758	NA	FBtr0091759
CG33764	FBgn0053764	NA	FBtr0091765
CG33766	FBgn0053766	NA	FBtr0091767
CG33767	FBgn0053767	NA	FBtr0091768
CG33768	FBgn0053768	NA	FBtr0091769
CG33769	FBgn0053769	NA	FBtr0091770
CG33777	FBgn0053777	NA	FBtr0091778
CG33783	FBgn0053783	NA	FBtr0091784
CG33784	FBgn0053784	NA	FBtr0091785
CG33792	FBgn0053792	NA	FBtr0091796
CG33795	FBgn0053795	NA	FBtr0091799
CG33796	FBgn0053796	NA	FBtr0091800
CG33797	FBgn0053797	NA	FBtr0091801
CG33798	FBgn0053798	NA	FBtr0091802
CG33800	FBgn0262594	NA	FBtr0091804
CG33801	FBgn0053801	NA	FBtr0091805
CG33803	FBgn0053803	NA	FBtr0091807
CG33804	FBgn0053804	NA	FBtr0091808
CG33806	FBgn0053806	NA	FBtr0091810
CG33807	FBgn0053807	NA	FBtr0091811
CG33808	FBgn0053808	NA	FBtr0091812
CG33809	FBgn0053809	NA	FBtr0091813
CG33810	FBgn0053810	NA	FBtr0091814
CG33812	FBgn0053812	NA	FBtr0091816
CG33813	FBgn0053813	NA	FBtr0091817
CG33814	FBgn0053814	NA	FBtr0091818
CG33815	FBgn0053815	NA	FBtr0091819
CG33816	FBgn0053816	NA	FBtr0091820
CG33817	FBgn0053817	NA	FBtr0091821
CG33818	FBgn0053818	NA	FBtr0091822
CG33819	FBgn0053819	NA	FBtr0091823
CG33820	FBgn0053820	NA	FBtr0091824
CG33821	FBgn0053821	NA	FBtr0091825
CG33822	FBgn0053822	NA	FBtr0091826
CG33823	FBgn0053823	NA	FBtr0091827
CG33824	FBgn0053824	NA	FBtr0091828
CG33825	FBgn0053825	NA	FBtr0091829
CG33826	FBgn0053826	NA	FBtr0091830
CG33827	FBgn0053827	NA	FBtr0091831
CG33828	FBgn0053828	NA	FBtr0091832
CG33829	FBgn0053829	NA	FBtr0091833
CG33830	FBgn0053830	NA	FBtr0091834
CG33831	FBgn0053831	NA	FBtr0091835
CG33832	FBgn0053832	NA	FBtr0091836
CG33833	FBgn0053833	NA	FBtr0091837
CG33834	FBgn0053834	NA	FBtr0091838
CG33835	FBgn0053835	NA	FBtr0091839
CG33836	FBgn0053836	NA	FBtr0091840
CG33837	FBgn0053837	NA	FBtr0091841
CG33838	FBgn0053838	NA	FBtr0091842
CG33839	FBgn0053839	NA	FBtr0091843
CG33840	FBgn0053840	NA	FBtr0091844
CG33841	FBgn0053841	NA	FBtr0091845
CG33842	FBgn0053842	NA	FBtr0091846

CG33843	FBgn0053843	NA	FBtr0091847
CG33844	FBgn0053844	NA	FBtr0091848
CG33845	FBgn0053845	NA	FBtr0091849
CG33846	FBgn0053846	NA	FBtr0091850
CG33847	FBgn0053847	NA	FBtr0091851
CG33848	FBgn0053848	NA	FBtr0091852
CG33849	FBgn0053849	NA	FBtr0091853
CG3385	FBgn0005636	nvy	FBtr0072267
CG33850	FBgn0053850	NA	FBtr0091854
CG33851	FBgn0053851	NA	FBtr0091855
CG33852	FBgn0053852	NA	FBtr0091856
CG33853	FBgn0053853	NA	FBtr0091857
CG33854	FBgn0053854	NA	FBtr0091858
CG33855	FBgn0053855	NA	FBtr0091859
CG33856	FBgn0053856	NA	FBtr0091860
CG33857	FBgn0053857	NA	FBtr0091861
CG33858	FBgn0053858	NA	FBtr0091862
CG33859	FBgn0053859	NA	FBtr0091863
CG3386	FBgn0035152	NA	FBtr0072611
CG33860	FBgn0053860	NA	FBtr0091864
CG33861	FBgn0053861	NA	FBtr0091865
CG33862	FBgn0053862	NA	FBtr0091866
CG33863	FBgn0053863	NA	FBtr0091867
CG33864	FBgn0053864	NA	FBtr0091868
CG33865	FBgn0053865	NA	FBtr0091869
CG33866	FBgn0053866	NA	FBtr0091870
CG33868	FBgn0053868	NA	FBtr0091872
CG33869	FBgn0053869	NA	FBtr0091873
CG33870	FBgn0053870	NA	FBtr0091874
CG33871	FBgn0053871	NA	FBtr0091875
CG33872	FBgn0053872	NA	FBtr0091876
CG33873	FBgn0053873	NA	FBtr0091877
CG33874	FBgn0053874	NA	FBtr0091878
CG33875	FBgn0053875	NA	FBtr0091879
CG33876	FBgn0053876	NA	FBtr0091880
CG33877	FBgn0053877	NA	FBtr0091881
CG33878	FBgn0053878	NA	FBtr0091882
CG33879	FBgn0053879	NA	FBtr0091883
CG33880	FBgn0053880	NA	FBtr0091884
CG33881	FBgn0053881	NA	FBtr0091885
CG33882	FBgn0053882	NA	FBtr0091886
CG33883	FBgn0053883	NA	FBtr0091887
CG33884	FBgn0053884	NA	FBtr0091888
CG33885	FBgn0053885	NA	FBtr0091889
CG33886	FBgn0053886	NA	FBtr0091890
CG33887	FBgn0053887	NA	FBtr0091891
CG33888	FBgn0053888	NA	FBtr0091892
CG33889	FBgn0053889	NA	FBtr0091893
CG3389	FBgn0038247	NA	FBtr0082965
CG33890	FBgn0053890	NA	FBtr0091894
CG33891	FBgn0053891	NA	FBtr0091895
CG33892	FBgn0053892	NA	FBtr0091896
CG33893	FBgn0053893	NA	FBtr0091897
CG33894	FBgn0053894	NA	FBtr0091898
CG33895	FBgn0053895	NA	FBtr0091899
CG33896	FBgn0053896	NA	FBtr0091900
CG33897	FBgn0053897	NA	FBtr0091901

CG33898	FBgn0053898	NA	FBtr0091902
CG33899	FBgn0053899	NA	FBtr0091903
CG33900	FBgn0053900	NA	FBtr0091904
CG33901	FBgn0053901	NA	FBtr0091905
CG33902	FBgn0053902	NA	FBtr0091906
CG33903	FBgn0053903	NA	FBtr0091907
CG33904	FBgn0053904	NA	FBtr0091908
CG33905	FBgn0053905	NA	FBtr0091909
CG33906	FBgn0053906	NA	FBtr0091910
CG33907	FBgn0053907	NA	FBtr0091911
CG33908	FBgn0053908	NA	FBtr0091912
CG33909	FBgn0053909	NA	FBtr0091913
CG33910	FBgn0053910	NA	FBtr0091914
CG33912	FBgn0053912	NA	FBtr0091916
CG33919	FBgn0053919	NA	FBtr0091923
CG33920	FBgn0053920	NA	FBtr0091924
CG33922	FBgn0053922	NA	FBtr0091926
CG33923	FBgn0053923	NA	FBtr0091927
CG33924	FBgn0261294	NA	FBtr0091928
CG33927	FBgn0053927	NA	FBtr0091931
CG33928	FBgn0053928	NA	FBtr0091932
CG33931	FBgn0066304	NA	FBtr0091935
CG33932	FBgn0066303	NA	FBtr0091936
CG33937	FBgn0053937	NA	FBtr0091947
CG3394	FBgn0034999	CG3394	FBtr0072268
CG33964	FBgn0053964	NA	FBtr0100005
CG33965	FBgn0053965	NA	FBtr0100006
CG33967	FBgn0262127	NA	FBtr0100008
CG33968	FBgn0260006	NA	FBtr0100009
CG33970	FBgn0053970	NA	FBtr0100012
CG33971	FBgn0053971	NA	FBtr0100014
CG33979	FBgn0261458	capt	FBtr0100023
CG33980	FBgn0263512	NA	FBtr0100024
CG33981	FBgn0250851	NA	FBtr0100026
CG33988	FBgn0053988	NA	FBtr0100033
CG33990	FBgn0067905	NA	FBtr0100038
CG33992	FBgn0045202	NA	FBtr0100045
CG3401	FBgn0003888	NA	FBtr0072270
CG34023	FBgn0054023	NA	FBtr0100078
CG34028	FBgn0054028	NA	FBtr0100083
CG34030	FBgn0054030	NA	FBtr0100085
CG34042	FBgn0065110	NA	FBtr0100097
CG34050	FBgn0054050	NA	FBtr0100105
CG34058	FBgn0065109	NA	FBtr0100831
CG34059	FBgn0065108	NA	FBtr0100832
CG3407	FBgn0031573	NA	FBtr0077515
CG3412	FBgn0023423	NA	FBtr0084032
CG3413	FBgn0034718	NA	FBtr0071849
CG3415	FBgn0030731	NA	FBtr0074209
CG3416	FBgn0002787	Mov34	FBtr0072290
CG3419	FBgn0035002	NA	FBtr0072292
CG3421	FBgn0038853	NA	FBtr0084036
CG3423	FBgn0020616	SA	FBtr0079349
CG3424	FBgn0036007	path	FBtr0076427
CG3425	FBgn0017482	NA	FBtr0071848
CG3428	FBgn0036005	NA	FBtr0076471
CG3440	FBgn0003046	NA	FBtr0079388

CG3443	FBgn0003048	NA	FBtr0070395
CG3446	FBgn0029868	NA	FBtr0070924
CG3448	FBgn0035996	NA	FBtr0076476
CG3454	FBgn0005619	NA	FBtr0088333
CG3456	FBgn0023549	NA	FBtr0070393
CG3457	FBgn0024984	NA	FBtr0070392
CG3469	FBgn0015000	NA	FBtr0078991
CG3473	FBgn0028913	NA	FBtr0080661
CG3474	FBgn0028871	NA	FBtr0080633
CG3476	FBgn0031881	NA	FBtr0079389
CG3481	FBgn0000055	NA	FBtr0100591
CG3483	FBgn0035005	NA	FBtr0072293
CG3484	FBgn0000056	NA	FBtr0006151
CG3491	FBgn0028887	NA	FBtr0080675
CG3492	FBgn0035007	NA	FBtr0072294
CG3493	FBgn0034854	NA	FBtr0072036
CG3494	FBgn0035008	NA	FBtr0072295
CG3495	FBgn0034794	Gmer	FBtr0071905
CG3496	FBgn0003977	NA	FBtr0072037
CG3497	FBgn0004837	Su(H)	FBtr0080700
CG3511	FBgn0035027	CG3511	FBtr0072309
CG3515	FBgn0031431	NA	FBtr0077750
CG3517	FBgn0038706	NA	FBtr0083770
CG3520	FBgn0034859	CG3520	FBtr0072027
CG3524	FBgn0042627	NA	FBtr0077657
CG3529	FBgn0035995	CG3529	FBtr0076461
CG3533	FBgn0004055	uzip	FBtr0072397
CG3534	FBgn0038463	NA	FBtr0083370
CG3544	FBgn0031279	CG3544	FBtr0078007
CG3546	FBgn0029716	NA	FBtr0070679
CG3557	FBgn0031423	NA	FBtr0077790
CG3565	FBgn0035034	NA	FBtr0072316
CG3569	FBgn0034865	NA	FBtr0072021
CG3570	FBgn0035035	NA	FBtr0072317
CG3571	FBgn0037978	NA	FBtr0082557
CG3572	FBgn0022960	vimar	FBtr0086182
CG3573	FBgn0023508	CG3573	FBtr0070317
CG3581	FBgn0038697	NA	FBtr0083775
CG3582	FBgn0017457	U2af38	FBtr0078133
CG3592	FBgn0029642	NA	FBtr0070551
CG3593	FBgn0003257	r-l	FBtr0084038
CG3594	FBgn0035063	NA	FBtr0072374
CG3608	FBgn0035039	CG3608	FBtr0072320
CG3616	FBgn0015040	NA	FBtr0072321
CG3624	FBgn0034724	CG3624	FBtr0071845
CG3647	FBgn0001978	stc	FBtr0080705
CG3650	FBgn0035070	NA	FBtr0072380
CG3651	FBgn0032974	NA	FBtr0085937
CG3654	FBgn0036004	CG3654	FBtr0076468
CG3656	FBgn0005670	NA	FBtr0070428
CG3663	FBgn0035044	NA	FBtr0072323
CG3664	FBgn0014010	Rab5	FBtr0077796
CG3665	FBgn0000635	NA	FBtr0070665
CG3666	FBgn0034094	NA	FBtr0087130
CG3668	FBgn0004896	NA	FBtr0071898
CG3678	FBgn0038461	NA	FBtr0083376
CG3687	FBgn0034097	NA	FBtr0087132

CG3690	FBgn0040350	NA	FBtr0070185
CG3692	FBgn0260450	NA	FBtr0074226
CG3699	FBgn0040349	CG3699	FBtr0070186
CG3702	FBgn0031590	NA	FBtr0077477
CG3703	FBgn0040348	CG3703	FBtr0070187
CG3705	FBgn0023129	aay	FBtr0076457
CG3707	FBgn0004655	NA	FBtr0070429
CG3715	FBgn0015296	Shc	FBtr0076458
CG3717	FBgn0013432	NA	FBtr0070431
CG3719	FBgn0024986	NA	FBtr0070233
CG3722	FBgn0003391	shg	FBtr0071546
CG3724	FBgn0004654	NA	FBtr0070384
CG3725	FBgn0263006	Ca-P60A	FBtr0072216
CG3726	FBgn0029824	CG3726	FBtr0070858
CG3727	FBgn0010583	NA	FBtr0078013
CG3732	FBgn0034750	CG3732	FBtr0071936
CG3735	FBgn0034933	NA	FBtr0072210
CG3744	FBgn0039240	NA	FBtr0084725
CG3753	FBgn0031655	NA	FBtr0078992
CG3757	FBgn0004034	y	FBtr0070071
CG3758	FBgn0001981	NA	FBtr0080734
CG3772	FBgn0025680	cry	FBtr0083736
CG3776	FBgn0035088	CG3776	FBtr0072395
CG3777	FBgn0024989	NA	FBtr0070068
CG3779	FBgn0002973	NA	FBtr0079822
CG3797	FBgn0036842	NA	FBtr0075037
CG3803	FBgn0034938	NA	FBtr0072208
CG3812	FBgn0030421	NA	FBtr0073689
CG3815	FBgn0029861	NA	FBtr0070899
CG3820	FBgn0010660	NA	FBtr0071996
CG3822	FBgn0038837	NA	FBtr0083987
CG3823	FBgn0029863	NA	FBtr0070900
CG3825	FBgn0034948	CG3825	FBtr0072204
CG3832	FBgn0019948	Phm	FBtr0072203
CG3835	FBgn0023507	CG3835	FBtr0070383
CG3837	FBgn0038279	NA	FBtr0083004
CG3838	FBgn0032130	CG3838	FBtr0079835
CG3843	FBgn0038281	NA	FBtr0083024
CG3845	FBgn0010488	NA	FBtr0087786
CG3847	FBgn0029867	NA	FBtr0070905
CG3848	FBgn0023518	NA	FBtr0070363
CG3850	FBgn0033782	sug	FBtr0087789
CG3851	FBgn0002985	odd	FBtr0077557
CG3853	FBgn0015230	NA	FBtr0083340
CG3860	FBgn0034951	CG3860	FBtr0072201
CG3861	FBgn0261955	l(1)G0030	FBtr0070906
CG3869	FBgn0029870	NA	FBtr0070908
CG3871	FBgn0027364	NA	FBtr0078274
CG3886	FBgn0005624	NA	FBtr0087798
CG3889	FBgn0027057	CSN1b	FBtr0075074
CG3891	FBgn0035993	CG3891	FBtr0076504
CG3893	FBgn0036826	NA	FBtr0075075
CG3897	FBgn0027660	NA	FBtr0075233
CG3902	FBgn0036824	CG3902	FBtr0075076
CG3903	FBgn0001987	Gli	FBtr0080767
CG3906	FBgn0034871	NA	FBtr0072121
CG3907	FBgn0034958	NA	FBtr0072196

CG3911	FBgn0260859	NA	FBtr0089879
CG3916	FBgn0038003	NA	FBtr0082560
CG3920	FBgn0021800	l(2)k16918	FBtr0077498
CG3922	FBgn0005533	NA	FBtr0076479
CG3923	FBgn0001337	NA	FBtr0070098
CG3924	FBgn0013764	Chi	FBtr0072191
CG3927	FBgn0034739	NA	FBtr0071853
CG3929	FBgn0000524	NA	FBtr0070914
CG3931	FBgn0034879	NA	FBtr0072111
CG3934	FBgn0037783	NA	FBtr0082176
CG3935	FBgn0000061	al	FBtr0078053
CG3940	FBgn0037788	NA	FBtr0082179
CG3941	FBgn0034878	NA	FBtr0072112
CG3954	FBgn0000382	NA	FBtr0070379
CG3955	FBgn0033793	NA	FBtr0087780
CG3956	FBgn0003448	sna	FBtr0080739
CG3964	FBgn0031574	CG3964	FBtr0077502
CG3966	FBgn0002936	ninaA	FBtr0078023
CG3967	FBgn0035989	CG3967	FBtr0076481
CG3969	FBgn0013955	PR2	FBtr0087777
CG3971	FBgn0260960	NA	FBtr0075356
CG3978	FBgn0003117	NA	FBtr0083220
CG3981	FBgn0040395	NA	FBtr0070376
CG3982	FBgn0035988	NA	FBtr0076485
CG3984	FBgn0038291	NA	FBtr0083025
CG3986	FBgn0022700	NA	FBtr0071543
CG3987	FBgn0038292	CG3987	FBtr0083026
CG3991	FBgn0020370	TppII	FBtr0087774
CG40002	FBgn0058002	NA	FBtr0113773
CG40006	FBgn0058006	NA	FBtr0111230
CG4004	FBgn0030418	NA	FBtr0073686
CG40053	FBgn0058053	NA	FBtr0111154
CG4006	FBgn0010379	NA	FBtr0083227
CG4012	FBgn0023081	NA	FBtr0072287
CG40127	FBgn0262116	NA	FBtr0111266
CG4013	FBgn0263865	NA	FBtr0073708
CG4025	FBgn0025624	NA	FBtr0070375
CG4027	FBgn0000042	Act5C	FBtr0100662
CG4029	FBgn0015396	jumu	FBtr0082237
CG40298	FBgn0058298	NA	FBtr0111169
CG4030	FBgn0034585	CG4030	FBtr0071640
CG4035	FBgn0015218	eIF-4E	FBtr0076489
CG4038	FBgn0011824	CG4038	FBtr0071639
CG4039	FBgn0025815	NA	FBtr0070952
CG4043	FBgn0037815	NA	FBtr0082248
CG40439	FBgn0058439	NA	FBtr0111232
CG40440	FBgn0046697	NA	FBtr0113889
CG40444	FBgn0001315	NA	FBtr0113891
CG40470	FBgn0058470	NA	FBtr0111152
CG40478	FBgn0027101	NA	FBtr0100406
CG4049	FBgn0034976	NA	FBtr0072285
CG4050	FBgn0020312	NA	FBtr0071638
CG4057	FBgn0041582	NA	FBtr0072281
CG4058	FBgn0038818	Nep4	FBtr0083937
CG4060	FBgn0038487	CG4060	FBtr0083415
CG4062	FBgn0027079	NA	FBtr0087734
CG4065	FBgn0034982	CG4065	FBtr0072280

CG4066	FBgn0038011	NA	FBtr0082566
CG4070	FBgn0011837	NA	FBtr0073683
CG4073	FBgn0037827	NA	FBtr0082269
CG4078	FBgn0029798	NA	FBtr0070807
CG40801	FBgn0085520	NA	FBtr0113933
CG40813	FBgn0085521	NA	FBtr0113934
CG4083	FBgn0017572	Mo25	FBtr0075358
CG4087	FBgn0002593	NA	FBtr0078056
CG4090	FBgn0038492	NA	FBtr0083413
CG4091	FBgn0034894	NA	FBtr0072097
CG4094	FBgn0028336	NA	FBtr0070953
CG4097	FBgn0002284	Pros26	FBtr0075360
CG4098	FBgn0036648	CG4098	FBtr0075361
CG4099	FBgn0014033	NA	FBtr0077467
CG4104	FBgn0027560	Tps1	FBtr0077431
CG4108	FBgn0036805	NA	FBtr0075092
CG41099	FBgn0039955	NA	FBtr0113991
CG41106	FBgn0069938	NA	FBtr0111194
CG4115	FBgn0038017	NA	FBtr0082568
CG4116	FBgn0029649	NA	FBtr0070561
CG4119	FBgn0028474	CG4119	FBtr0070799
CG4120	FBgn0036806	NA	FBtr0075091
CG4125	FBgn0003285	rst	FBtr0070562
CG41257	FBgn0085582	NA	FBtr0114025
CG41265	FBgn0262123	NA	FBtr0114030
CG41320	FBgn0085612	NA	FBtr0114056
CG4136	FBgn0263511	CG4136	FBtr0070781
CG4141	FBgn0015279	NA	FBtr0083940
CG41423	FBgn0085644	NA	FBtr0114088
CG41434	FBgn0084001	NA	FBtr0111262
CG4144	FBgn0040322	NA	FBtr0075094
CG4145	FBgn0000299	NA	FBtr0079003
CG41454	FBgn0084017	NA	FBtr0111173
CG4147	FBgn0001218	NA	FBtr0073608
CG4148	FBgn0001990	wek	FBtr0080764
CG41497	FBgn0085658	NA	FBtr0114103
CG4151	FBgn0029770	NA	FBtr0070779
CG4152	FBgn0001986	l(2)35Df	FBtr0080760
CG41562	FBgn0085693	NA	FBtr0114142
CG4158	FBgn0001983	wor	FBtr0080740
CG4161	FBgn0028892	NA	FBtr0080735
CG4162	FBgn0002524	NA	FBtr0080738
CG4165	FBgn0029763	NA	FBtr0070773
CG4170	FBgn0024183	vig	FBtr0080718
CG4174	FBgn0036793	NA	FBtr0100566
CG4178	FBgn0002563	NA	FBtr0078025
CG4180	FBgn0001977	NA	FBtr0080725
CG4182	FBgn0041713	NA	FBtr0080699
CG4183	FBgn0001225	Hsp26	FBtr0076496
CG4193	FBgn0011761	NA	FBtr0070749
CG4198	FBgn0029753	NA	FBtr0070750
CG4200	FBgn0003416	NA	FBtr0074230
CG4207	FBgn0026261	NA	FBtr0071860
CG4211	FBgn0004227	NA	FBtr0100586
CG4214	FBgn0011708	NA	FBtr0080832
CG4216	FBgn0003683	NA	FBtr0075112
CG4217	FBgn0038805	TFAM	FBtr0083949

CG4218	FBgn0250844	NA	FBtr0080662
CG4221	FBgn0038385	CG4221	FBtr0083201
CG4225	FBgn0038376	NA	FBtr0083188
CG4231	FBgn0026397	NA	FBtr0077874
CG4237	FBgn0020655	NA	FBtr0075981
CG4239	FBgn0030745	CG4239	FBtr0074268
CG4244	FBgn0003557	NA	FBtr0077822
CG4249	FBgn0028525	NA	FBtr0080830
CG4252	FBgn0004367	NA	FBtr0074271
CG4254	FBgn0011726	NA	FBtr0072188
CG4257	FBgn0016917	NA	FBtr0089485
CG4258	FBgn0020305	NA	FBtr0078030
CG4259	FBgn0031389	NA	FBtr0077854
CG4272	FBgn0261647	NA	FBtr0077762
CG4274	FBgn0001086	fzy	FBtr0080833
CG4276	FBgn0029095	NA	FBtr0078031
CG4278	FBgn0014092	CG4278	FBtr0080834
CG4288	FBgn0038799	NA	FBtr0083889
CG4300	FBgn0036272	CG4300	FBtr0076009
CG4311	FBgn0010611	Hmgs	FBtr0087145
CG4312	FBgn0002869	NA	FBtr0083890
CG4316	FBgn0003319	NA	FBtr0083250
CG4317	FBgn0026060	NA	FBtr0070884
CG4319	FBgn0011706	NA	FBtr0075120
CG4320	FBgn0029840	NA	FBtr0070885
CG4321	FBgn0015033	NA	FBtr0076832
CG4323	FBgn0038797	NA	FBtr0083891
CG4329	FBgn0034745	NA	FBtr0071940
CG4330	FBgn0030452	NA	FBtr0073724
CG4337	FBgn0010438	NA	FBtr0100152
CG4338	FBgn0038313	NA	FBtr0083066
CG4345	FBgn0015946	grim	FBtr0075130
CG4353	FBgn0010303	NA	FBtr0073747
CG4354	FBgn0005638	slbo	FBtr0072272
CG4356	FBgn0000037	NA	FBtr0072368
CG4357	FBgn0036279	NA	FBtr0075988
CG4367	FBgn0038783	NA	FBtr0083901
CG4372	FBgn0034665	NA	FBtr0071738
CG4385	FBgn0003310	S	FBtr0077993
CG4389	FBgn0028479	CG4389	FBtr0079857
CG4400	FBgn0030434	NA	FBtr0073693
CG4402	FBgn0034660	NA	FBtr0071745
CG4408	FBgn0039073	CG4408	FBtr0084431
CG4413	FBgn0038767	NA	FBtr0083840
CG4420	FBgn0030753	CG4420	FBtr0074278
CG4424	FBgn0038765	NA	FBtr0083841
CG4426	FBgn0015905	ast	FBtr0077952
CG4440	FBgn0040984	NA	FBtr0080835
CG4443	FBgn0015374	NA	FBtr0074283
CG4445	FBgn0027558	NA	FBtr0089061
CG4447	FBgn0035980	NA	FBtr0076444
CG4451	FBgn0038755	Hs6st	FBtr0083846
CG4471	FBgn0033137	NA	FBtr0089062
CG4476	FBgn0035969	CG4476	FBtr0076429
CG4477	FBgn0035971	NA	FBtr0076430
CG4478	FBgn0013301	NA	FBtr0080681
CG4479	FBgn0013300	NA	FBtr0080682

CG4483	FBgn0035970	NA	FBtr0076502
CG4488	FBgn0011737	wee	FBtr0079371
CG4491	FBgn0005771	noc	FBtr0080643
CG4494	FBgn0026170	NA	FBtr0079356
CG4496	FBgn0031894	NA	FBtr0079419
CG4502	FBgn0031896	CG4502	FBtr0079417
CG4510	FBgn0038746	NA	FBtr0083854
CG4520	FBgn0038355	NA	FBtr0083167
CG4521	FBgn0030766	NA	FBtr0074314
CG4525	FBgn0038358	NA	FBtr0083170
CG4531	FBgn0004569	argos	FBtr0075408
CG4536	FBgn0086693	NA	FBtr0070969
CG4538	FBgn0038745	NA	FBtr0083855
CG4545	FBgn0010414	SerT	FBtr0072362
CG4546	FBgn0038373	NA	FBtr0083184
CG4550	FBgn0002940	NA	FBtr0083857
CG4551	FBgn0016930	smi35A	FBtr0089290
CG4552	FBgn0031304	CG4552	FBtr0077956
CG4554	FBgn0034734	NA	FBtr0071833
CG4563	FBgn0035006	NA	FBtr0072355
CG4567	FBgn0263133	CG4567	FBtr0079413
CG4570	FBgn0037844	CG4570	FBtr0082287
CG4574	FBgn0004611	NA	FBtr0078047
CG4576	FBgn0038366	CG4576	FBtr0083180
CG4577	FBgn0031306	NA	FBtr0077957
CG4579	FBgn0021761	NA	FBtr0089881
CG4580	FBgn0032585	NA	FBtr0080865
CG4581	FBgn0025352	Thiolase	FBtr0072226
CG4582	FBgn0039344	NA	FBtr0084896
CG4584	FBgn0250837	NA	FBtr0080157
CG4589	FBgn0019886	CG4589	FBtr0072349
CG4597	FBgn0035594	NA	FBtr0077162
CG4599	FBgn0032586	NA	FBtr0080866
CG4605	FBgn0023415	NA	FBtr0080158
CG4606	FBgn0026616	NA	FBtr0083179
CG4607	FBgn0029932	NA	FBtr0071013
CG4609	FBgn0014163	NA	FBtr0075412
CG4610	FBgn0034735	NA	FBtr0071834
CG4613	FBgn0036427	NA	FBtr0075725
CG4617	FBgn0029936	CG4617	FBtr0071019
CG4626	FBgn0027342	NA	FBtr0071017
CG4630	FBgn0033809	NA	FBtr0087765
CG4633	FBgn0028962	Aats-ala-m	FBtr0077168
CG4637	FBgn0004644	NA	FBtr0084404
CG4643	FBgn0043010	CG4643	FBtr0087762
CG4644	FBgn0261938	NA	FBtr0077989
CG4645	FBgn0030435	NA	FBtr0073692
CG4646	FBgn0033810	NA	FBtr0087764
CG4647	FBgn0030433	NA	FBtr0073691
CG4654	FBgn0011763	Dp	FBtr0087740
CG4660	FBgn0029839	CG4660	FBtr0070886
CG4663	FBgn0033812	NA	FBtr0087761
CG4666	FBgn0029838	CG4666	FBtr0070887
CG4680	FBgn0036627	CG4680	FBtr0075388
CG4696	FBgn0002789	Mp20	FBtr0087745
CG4698	FBgn0010453	Wnt4	FBtr0089291
CG4700	FBgn0011260	Sema-2a	FBtr0087090

CG4703	FBgn0038742	NA	FBtr0083817
CG4704	FBgn0039029	NA	FBtr0084314
CG4707	FBgn0035036	NA	FBtr0072338
CG4710	FBgn0016926	smi21F	FBtr0077988
CG4712	FBgn0033818	NA	FBtr0087757
CG4714	FBgn0033819	NA	FBtr0087756
CG4715	FBgn0031305	NA	FBtr0077986
CG4716	FBgn0033820	NA	FBtr0087747
CG4717	FBgn0001320	NA	FBtr0078283
CG4722	FBgn0000180	bib	FBtr0079929
CG4726	FBgn0031307	CG4726	FBtr0077958
CG4738	FBgn0262647	NA	FBtr0080199
CG4739	FBgn0040257	NA	FBtr0082337
CG4749	FBgn0031308	NA	FBtr0077959
CG4751	FBgn0032348	NA	FBtr0080200
CG4752	FBgn0034733	CG4752	FBtr0071832
CG4753	FBgn0036622	CG4753	FBtr0075421
CG4757	FBgn0027584	NA	FBtr0082339
CG4759	FBgn0039359	RpL27	FBtr0084932
CG4761	FBgn0001323	knrl	FBtr0078212
CG4763	FBgn0034910	NA	FBtr0072238
CG4764	FBgn0031310	CG4764	FBtr0077960
CG4766	FBgn0027546	NA	FBtr0070891
CG4774	FBgn0039360	NA	FBtr0084929
CG4775	FBgn0031312	NA	FBtr0077961
CG4778	FBgn0027600	CG4778	FBtr0079934
CG4783	FBgn0038756	NA	FBtr0083823
CG4784	FBgn0036619	NA	FBtr0075424
CG4785	FBgn0031314	CG4785	FBtr0077962
CG4786	FBgn0037012	NA	FBtr0078213
CG4792	FBgn0039016	NA	FBtr0084324
CG4795	FBgn0261714	NA	FBtr0082548
CG4797	FBgn0034909	NA	FBtr0072239
CG4798	FBgn0022029	l(2)k01209	FBtr0086913
CG4802	FBgn0034215	NA	FBtr0086916
CG4817	FBgn0010278	NA	FBtr0072242
CG4818	FBgn0036617	NA	FBtr0075382
CG4827	FBgn0034225	CG4827	FBtr0086926
CG4830	FBgn0037996	NA	FBtr0082625
CG4845	FBgn0243511	NA	FBtr0083829
CG4846	FBgn0013433	NA	FBtr0080806
CG4847	FBgn0034229	CG4847	FBtr0086935
CG4848	FBgn0037998	CG4848	FBtr0082624
CG4851	FBgn0032358	CG4851	FBtr0080212
CG4854	FBgn0038766	CG4854	FBtr0083830
CG4865	FBgn0026085	NA	FBtr0070642
CG4871	FBgn0035050	NA	FBtr0072441
CG4872	FBgn0030799	CG4872	FBtr0074339
CG4877	FBgn0036624	NA	FBtr0075387
CG4882	FBgn0025336	CG4882	FBtr0072225
CG4889	FBgn0004009	wg	FBtr0079432
CG4891	FBgn0028520	NA	FBtr0080815
CG4892	FBgn0028884	NA	FBtr0080816
CG4894	FBgn0001991	NA	FBtr0090004
CG4900	FBgn0024958	NA	FBtr0084327
CG4908	FBgn0032195	CG4908	FBtr0079950
CG4909	FBgn0040294	POSH	FBtr0086828

CG4913	FBgn0026441	NA	FBtr0083082
CG4914	FBgn0036436	CG4914	FBtr0075712
CG4920	FBgn0000533	ea	FBtr0083083
CG4925	FBgn0036614	NA	FBtr0075428
CG4927	FBgn0034139	NA	FBtr0100547
CG4928	FBgn0027556	NA	FBtr0074344
CG4930	FBgn0028515	NA	FBtr0080824
CG4931	FBgn0038320	NA	FBtr0083085
CG4935	FBgn0028897	NA	FBtr0080825
CG4938	FBgn0021750	Aats-ser	FBtr0083086
CG4943	FBgn0029006	NA	FBtr0086833
CG4954	FBgn0034258	NA	FBtr0086836
CG4955	FBgn0030814	NA	FBtr0074382
CG4956	FBgn0039370	NA	FBtr0084920
CG4962	FBgn0036597	NA	FBtr0075471
CG4963	FBgn0039561	NA	FBtr0085266
CG4965	FBgn0002673	NA	FBtr0080855
CG4968	FBgn0032214	CG4968	FBtr0079987
CG4976	FBgn0039559	Mes-4	FBtr0085267
CG4988	FBgn0032372	NA	FBtr0080254
CG4993	FBgn0024734	NA	FBtr0080856
CG4996	FBgn0034271	NA	FBtr0086864
CG4999	FBgn0035936	Tsp66E	FBtr0076508
CG5002	FBgn0034275	CG5002	FBtr0089546
CG5005	FBgn0022740	NA	FBtr0089547
CG5006	FBgn0026390	NA	FBtr0080280
CG5009	FBgn0027572	NA	FBtr0089548
CG5010	FBgn0260747	CG5010	FBtr0074387
CG5014	FBgn0029687	NA	FBtr0070616
CG5018	FBgn0263605	NA	FBtr0075484
CG5023	FBgn0038774	CG5023	FBtr0083866
CG5032	FBgn0026309	NA	FBtr0089549
CG5037	FBgn0032222	CG5037	FBtr0079992
CG5041	FBgn0031309	Tfb4	FBtr0077985
CG5044	FBgn0038326	CG5044	FBtr0083091
CG5047	FBgn0037008	CG5047	FBtr0078215
CG5048	FBgn0036437	CG5048	FBtr0075684
CG5053	FBgn0261986	NA	FBtr0084898
CG5059	FBgn0037007	CG5059	FBtr0078217
CG5062	FBgn0029747	NA	FBtr0070759
CG5064	FBgn0035947	NA	FBtr0076517
CG5068	FBgn0035951	CG5068	FBtr0076519
CG5069	FBgn0014143	NA	FBtr0078395
CG5075	FBgn0032464	NA	FBtr0080421
CG5076	FBgn0011589	NA	FBtr0086802
CG5079	FBgn0039346	NA	FBtr0084883
CG5080	FBgn0031313	CG5080	FBtr0077982
CG5081	FBgn0086377	NA	FBtr0078352
CG5087	FBgn0035953	CG5087	FBtr0076520
CG5091	FBgn0032234	NA	FBtr0080005
CG5092	FBgn0021796	Tor	FBtr0080422
CG5093	FBgn0035954	Doc3	FBtr0076521
CG5102	FBgn0000413	da	FBtr0080008
CG5104	FBgn0037009	NA	FBtr0078209
CG5108	FBgn0032236	NA	FBtr0080007
CG5112	FBgn0039341	CG5112	FBtr0084878
CG5123	FBgn0003997	W	FBtr0075133

CG5125	FBgn0002938	NA	FBtr0079436
CG5130	FBgn0037000	CG5130	FBtr0078221
CG5133	FBgn0028789	Doc1	FBtr0076522
CG5144	FBgn0035957	NA	FBtr0076523
CG5147	FBgn0036775	NA	FBtr0075136
CG5150	FBgn0035620	CG5150	FBtr0077086
CG5161	FBgn0263604	NA	FBtr0075491
CG5162	FBgn0030828	CG5162	FBtr0074393
CG5165	FBgn0003076	NA	FBtr0075492
CG5167	FBgn0038038	NA	FBtr0082583
CG5170	FBgn0027835	Dp1	FBtr0086681
CG5178	FBgn0000047	NA	FBtr0083143
CG5179	FBgn0019949	Cdk9	FBtr0071937
CG5185	FBgn0026320	Tom	FBtr0075620
CG5191	FBgn0038803	NA	FBtr0083908
CG5192	FBgn0019940	NA	FBtr0083146
CG5193	FBgn0004915	TfIIB	FBtr0080025
CG5196	FBgn0038039	NA	FBtr0082585
CG5198	FBgn0032250	NA	FBtr0080026
CG5201	FBgn0020493	NA	FBtr0083393
CG5203	FBgn0027052	CHIP	FBtr0080027
CG5204	FBgn0032473	NA	FBtr0080433
CG5205	FBgn0038344	CG5205	FBtr0083147
CG5208	FBgn0028470	NA	FBtr0083392
CG5210	FBgn0013763	Chit	FBtr0087111
CG5213	FBgn0038345	NA	FBtr0083148
CG5214	FBgn0037891	CG5214	FBtr0082358
CG5216	FBgn0024291	NA	FBtr0080434
CG5219	FBgn0036990	NA	FBtr0078227
CG5224	FBgn0034354	NA	FBtr0086697
CG5232	FBgn0038045	NA	FBtr0082588
CG5249	FBgn0035625	Blimp-1	FBtr0077089
CG5258	FBgn0029148	NHP2	FBtr0075698
CG5261	FBgn0031912	NA	FBtr0079444
CG5265	FBgn0038486	CG5265	FBtr0083407
CG5266	FBgn0086134	Pros25	FBtr0082590
CG5271	FBgn0003942	NA	FBtr0080016
CG5272	FBgn0001120	NA	FBtr0075697
CG5277	FBgn0025366	NA	FBtr0080017
CG5278	FBgn0038986	CG5278	FBtr0084281
CG5279	FBgn0014019	NA	FBtr0080291
CG5280	FBgn0035952	NA	FBtr0076552
CG5287	FBgn0032477	CG5287	FBtr0080436
CG5304	FBgn0010497	NA	FBtr0080292
CG5308	FBgn0037908	NA	FBtr0082394
CG5327	FBgn0034363	NA	FBtr0086707
CG5334	FBgn0030577	NA	FBtr0073935
CG5335	FBgn0034365	NA	FBtr0086708
CG5336	FBgn0032409	Ced-12	FBtr0080330
CG5337	FBgn0032249	NA	FBtr0080043
CG5341	FBgn0034367	sec6	FBtr0086709
CG5346	FBgn0038981	NA	FBtr0084289
CG5347	FBgn0030578	NA	FBtr0073936
CG5357	FBgn0034862	NA	FBtr0072017
CG5360	FBgn0261786	NA	FBtr0072058
CG5362	FBgn0262782	Mdh	FBtr0080050
CG5363	FBgn0004106	cdc2	FBtr0080051

CG5366	FBgn0027568	NA	FBtr0080054
CG5370	FBgn0010501	Dcp-1	FBtr0072062
CG5371	FBgn0011703	NA	FBtr0080059
CG5372	FBgn0034880	NA	FBtr0072063
CG5374	FBgn0003676	T-cp1	FBtr0084291
CG5376	FBgn0038977	NA	FBtr0084292
CG5384	FBgn0032216	NA	FBtr0080061
CG5385	FBgn0032215	NA	FBtr0080062
CG5386	FBgn0038945	NA	FBtr0084240
CG5387	FBgn0027491	NA	FBtr0080063
CG5390	FBgn0032213	NA	FBtr0080064
CG5392	FBgn0036463	NA	FBtr0075671
CG5394	FBgn0005674	NA	FBtr0084511
CG5395	FBgn0005322	NA	FBtr0080065
CG5397	FBgn0031327	CG5397	FBtr0077920
CG5399	FBgn0038353	NA	FBtr0083164
CG5400	FBgn0000564	NA	FBtr0083444
CG5403	FBgn0004795	retn	FBtr0072073
CG5404	FBgn0038354	NA	FBtr0083165
CG5408	FBgn0028978	trbl	FBtr0078235
CG5422	FBgn0005649	Rox8	FBtr0084591
CG5429	FBgn0010709	NA	FBtr0084590
CG5430	FBgn0011294	NA	FBtr0077922
CG5436	FBgn0001230	NA	FBtr0084589
CG5439	FBgn0032476	CG5439	FBtr0080435
CG5441	FBgn0263118	dei	FBtr0085009
CG5445	FBgn0030838	CG5445	FBtr0074428
CG5455	FBgn0039430	CG5455	FBtr0085015
CG5458	FBgn0032478	NA	FBtr0080437
CG5460	FBgn0001169	H	FBtr0083917
CG5466	FBgn0038815	NA	FBtr0083913
CG5468	FBgn0039434	CG5468	FBtr0085029
CG5474	FBgn0011016	NA	FBtr0075453
CG5476	FBgn0039441	NA	FBtr0085031
CG5479	FBgn0034893	mRpL43	FBtr0072084
CG5484	FBgn0039450	NA	FBtr0085039
CG5488	FBgn0004854	NA	FBtr0074429
CG5489	FBgn0034366	NA	FBtr0086712
CG5490	FBgn0262473	NA	FBtr0085060
CG5491	FBgn0039459	CG5491	FBtr0085061
CG5494	FBgn0038819	NA	FBtr0083919
CG5499	FBgn0001197	His2Av	FBtr0085062
CG5502	FBgn0003279	NA	FBtr0085248
CG5507	FBgn0004359	NA	FBtr0085065
CG5510	FBgn0039160	NA	FBtr0084576
CG5515	FBgn0039163	NA	FBtr0084574
CG5519	FBgn0261119	NA	FBtr0086723
CG5520	FBgn0039562	Gp93	FBtr0085254
CG5522	FBgn0034158	CG5522	FBtr0087030
CG5529	FBgn0011758	B-H1	FBtr0074430
CG5538	FBgn0038052	NA	FBtr0082631
CG5540	FBgn0039551	NA	FBtr0085226
CG5541	FBgn0030603	CG5541	FBtr0073959
CG5543	FBgn0034908	CG5543	FBtr0072124
CG5546	FBgn0036761	NA	FBtr0075160
CG5547	FBgn0032482	NA	FBtr0080439
CG5548	FBgn0030605	NA	FBtr0073960

CG5557	FBgn0010768	NA	FBtr0083733
CG5560	FBgn0030607	NA	FBtr0073961
CG5562	FBgn0024234	gbb	FBtr0072127
CG5567	FBgn0036760	CG5567	FBtr0075161
CG5575	FBgn0011236	ken	FBtr0072129
CG5577	FBgn0036759	NA	FBtr0075162
CG5582	FBgn0036756	cln3	FBtr0075167
CG5590	FBgn0039537	CG5590	FBtr0085215
CG5591	FBgn0263667	NA	FBtr0072134
CG5594	FBgn0261794	CG5594	FBtr0072137
CG5595	FBgn0003330	Sce	FBtr0085245
CG5596	FBgn0002772	Mlc1	FBtr0085196
CG5597	FBgn0034920	NA	FBtr0072131
CG5599	FBgn0030612	CG5599	FBtr0073964
CG5603	FBgn0032210	NA	FBtr0080070
CG5604	FBgn0032208	NA	FBtr0080074
CG5605	FBgn0036974	eRF1	FBtr0078177
CG5608	FBgn0038058	NA	FBtr0082680
CG5609	FBgn0038838	NA	FBtr0083973
CG5610	FBgn0000036	NA	FBtr0084619
CG5613	FBgn0030839	NA	FBtr0074431
CG5619	FBgn0003751	NA	FBtr0080078
CG5623	FBgn0038357	NA	FBtr0083197
CG5626	FBgn0036266	NA	FBtr0076016
CG5638	FBgn0036260	NA	FBtr0076020
CG5639	FBgn0039527	NA	FBtr0085202
CG5644	FBgn0035948	NA	FBtr0076556
CG5646	FBgn0039525	NA	FBtr0085210
CG5648	FBgn0032492	NA	FBtr0080448
CG5651	FBgn0086706	NA	FBtr0076557
CG5653	FBgn0035943	NA	FBtr0076559
CG5655	FBgn0011305	Rsf1	FBtr0080079
CG5656	FBgn0037083	CG5656	FBtr0078396
CG5660	FBgn0035942	CG5660	FBtr0076560
CG5662	FBgn0030620	NA	FBtr0073999
CG5664	FBgn0037082	NA	FBtr0078397
CG5682	FBgn0032480	NA	FBtr0080438
CG5683	FBgn0005694	NA	FBtr0078348
CG5687	FBgn0035293	NA	FBtr0072858
CG5688	FBgn0026432	Grip163	FBtr0076061
CG5694	FBgn0032197	CG5694	FBtr0079954
CG5700	FBgn0028573	prc	FBtr0076064
CG5701	FBgn0036980	RhoBTB	FBtr0078185
CG5703	FBgn0030853	NA	FBtr0074452
CG5705	FBgn0032486	NA	FBtr0080444
CG5708	FBgn0032196	NA	FBtr0079955
CG5709	FBgn0025186	NA	FBtr0071774
CG5718	FBgn0036222	NA	FBtr0076069
CG5726	FBgn0034313	CG5726	FBtr0086772
CG5729	FBgn0027836	NA	FBtr0086773
CG5730	FBgn0000083	NA	FBtr0089519
CG5733	FBgn0034310	NA	FBtr0086775
CG5737	FBgn0038851	NA	FBtr0084007
CG5744	FBgn0030897	NA	FBtr0100239
CG5745	FBgn0038855	CG5745	FBtr0084008
CG5755	FBgn0032664	NA	FBtr0081033
CG5756	FBgn0034301	CG5756	FBtr0086786

CG5757	FBgn0034299	CG5757	FBtr0086787
CG5760	FBgn0028468	rtet	FBtr0084009
CG5765	FBgn0034294	CG5765	FBtr0086809
CG5770	FBgn0034291	NA	FBtr0086812
CG5771	FBgn0015790	NA	FBtr0084010
CG5773	FBgn0034290	CG5773	FBtr0086813
CG5776	FBgn0032450	CG5776	FBtr0080407
CG5785	FBgn0003701	NA	FBtr0086825
CG5788	FBgn0026316	NA	FBtr0089553
CG5791	FBgn0040582	NA	FBtr0084180
CG5800	FBgn0030855	NA	FBtr0074453
CG5807	FBgn0027539	NA	FBtr0084739
CG5810	FBgn0038866	NA	FBtr0084026
CG5811	FBgn0004842	NA	FBtr0085100
CG5812	FBgn0029170	GCR(ich)	FBtr0085101
CG5814	FBgn0015625	CycB3	FBtr0084728
CG5815	FBgn0027574	NA	FBtr0085107
CG5818	FBgn0001995	NA	FBtr0080843
CG5820	FBgn0013272	Gp150	FBtr0071815
CG5821	FBgn0022985	NA	FBtr0071828
CG5826	FBgn0038519	NA	FBtr0083503
CG5830	FBgn0036556	NA	FBtr0075521
CG5834	FBgn0051354	NA	FBtr0082636
CG5836	FBgn0025571	SF1	FBtr0083456
CG5841	FBgn0263601	NA	FBtr0075520
CG5844	FBgn0038049	CG5844	FBtr0082591
CG5848	FBgn0000250	NA	FBtr0080847
CG5853	FBgn0032167	CG5853	FBtr0079894
CG5855	FBgn0000339	NA	FBtr0080849
CG5859	FBgn0025830	NA	FBtr0087036
CG5861	FBgn0015338	CG5861	FBtr0080850
CG5863	FBgn0038507	NA	FBtr0083439
CG5867	FBgn0027586	NA	FBtr0080449
CG5869	FBgn0028894	NA	FBtr0080851
CG5870	FBgn0250788	NA	FBtr0074454
CG5872	FBgn0036991	CG5872	FBtr0078194
CG5873	FBgn0038511	CG5873	FBtr0083442
CG5874	FBgn0038872	NA	FBtr0084051
CG5877	FBgn0030625	NA	FBtr0074001
CG5883	FBgn0036225	NA	FBtr0076067
CG5886	FBgn0039379	CG5886	FBtr0084907
CG5887	FBgn0086687	desat1	FBtr0082627
CG5888	FBgn0028523	CG5888	FBtr0080880
CG5892	FBgn0038873	NA	FBtr0084052
CG5893	FBgn0000411	D	FBtr0075746
CG5903	FBgn0038400	NA	FBtr0083281
CG5904	FBgn0036557	mRpS31	FBtr0075519
CG5905	FBgn0029843	Nep1	FBtr0070872
CG5906	FBgn0036217	NA	FBtr0076071
CG5911	FBgn0038874	NA	FBtr0084054
CG5916	FBgn0038401	NA	FBtr0083251
CG5930	FBgn0011289	NA	FBtr0085155
CG5931	FBgn0263599	CG5931	FBtr0075527
CG5935	FBgn0026533	Dek	FBtr0100437
CG5942	FBgn0000212	NA	FBtr0075526
CG5945	FBgn0032494	NA	FBtr0080450
CG5949	FBgn0263600	DNApol-delta	FBtr0075522

CG5954	FBgn0002441	NA	FBtr0085175
CG5958	FBgn0031913	CG5958	FBtr0079466
CG5962	FBgn0000121	NA	FBtr0076599
CG5966	FBgn0029831	CG5966	FBtr0070866
CG5974	FBgn0010441	NA	FBtr0085179
CG5984	FBgn0039500	NA	FBtr0085181
CG5987	FBgn0039501	NA	FBtr0085180
CG5991	FBgn0026576	CG5991	FBtr0084536
CG5992	FBgn0036752	Adgf-A	FBtr0075190
CG5998	FBgn0036751	NA	FBtr0075193
CG6000	FBgn0039145	CG6000	FBtr0084540
CG6004	FBgn0036203	NA	FBtr0076119
CG6012	FBgn0032615	NA	FBtr0080923
CG6017	FBgn0259824	CG6017	FBtr0075518
CG6020	FBgn0037001	CG6020	FBtr0078201
CG6023	FBgn0030912	NA	FBtr0074579
CG6033	FBgn0004638	NA	FBtr0087698
CG6045	FBgn0038349	NA	FBtr0083151
CG6046	FBgn0024491	Bin1	FBtr0083279
CG6049	FBgn0037081	CG6049	FBtr0078398
CG6057	FBgn0040283	NA	FBtr0084541
CG6066	FBgn0039488	NA	FBtr0085131
CG6067	FBgn0029828	NA	FBtr0070864
CG6070	FBgn0039487	CG6070	FBtr0085132
CG6071	FBgn0036186	NA	FBtr0076136
CG6072	FBgn0086370	NA	FBtr0083255
CG6083	FBgn0036183	NA	FBtr0076137
CG6084	FBgn0086254	CG6084	FBtr0076138
CG6098	FBgn0010398	Lrr47	FBtr0080092
CG6106	FBgn0030914	CG6106	FBtr0074553
CG6108	FBgn0032498	NA	FBtr0080459
CG6116	FBgn0032499	CG6116	FBtr0080460
CG6117	FBgn0000489	Pka-C3	FBtr0075529
CG6120	FBgn0027865	Tsp96F	FBtr0084912
CG6122	FBgn0004872	piwi	FBtr0080166
CG6125	FBgn0038337	CG6125	FBtr0083105
CG6126	FBgn0038407	CG6126	FBtr0083278
CG6127	FBgn0004197	NA	FBtr0085128
CG6130	FBgn0038334	NA	FBtr0083107
CG6131	FBgn0039480	CG6131	FBtr0085136
CG6140	FBgn0036162	NA	FBtr0076177
CG6141	FBgn0015756	NA	FBtr0080164
CG6163	FBgn0036155	NA	FBtr0076181
CG6175	FBgn0036152	NA	FBtr0076183
CG6177	FBgn0026634	ldlCp	FBtr0083114
CG6182	FBgn0039158	NA	FBtr0084551
CG6186	FBgn0022355	NA	FBtr0074559
CG6189	FBgn0001341	NA	FBtr0070119
CG6193	FBgn0026598	NA	FBtr0084552
CG6194	FBgn0038325	CG6194	FBtr0083115
CG6197	FBgn0033859	NA	FBtr0087623
CG6198	FBgn0029503	CHORD	FBtr0084554
CG6201	FBgn0032343	NA	FBtr0080229
CG6206	FBgn0027611	CG6206	FBtr0080030
CG6209	FBgn0033862	NA	FBtr0087651
CG6214	FBgn0032456	MRP	FBtr0090027
CG6218	FBgn0038321	CG6218	FBtr0083119

CG6222	FBgn0003575	NA	FBtr0070120
CG6223	FBgn0008635	betaCop	FBtr0074576
CG6224	FBgn0040230	dbo	FBtr0075505
CG6226	FBgn0013269	FK506-bp1	FBtr0083120
CG6227	FBgn0030631	NA	FBtr0074011
CG6230	FBgn0027582	NA	FBtr0080228
CG6234	FBgn0038071	NA	FBtr0082648
CG6235	FBgn0004889	NA	FBtr0082186
CG6236	FBgn0038318	NA	FBtr0083122
CG6240	FBgn0038714	NA	FBtr0083811
CG6244	FBgn0036531	NA	FBtr0075502
CG6249	FBgn0032346	NA	FBtr0080226
CG6255	FBgn0038708	NA	FBtr0083769
CG6258	FBgn0028700	NA	FBtr0080225
CG6269	FBgn0024184	NA	FBtr0074460
CG6271	FBgn0039476	NA	FBtr0085149
CG6276	FBgn0038316	CG6276	FBtr0083123
CG6277	FBgn0039475	NA	FBtr0085150
CG6280	FBgn0033866	NA	FBtr0087628
CG6283	FBgn0039474	NA	FBtr0085151
CG6287	FBgn0032350	NA	FBtr0080222
CG6290	FBgn0030921	NA	FBtr0074563
CG6292	FBgn0025455	CycT	FBtr0075205
CG6293	FBgn0037807	NA	FBtr0082240
CG6297	FBgn0020412	NA	FBtr0076236
CG6302	FBgn0010741	NA	FBtr0076238
CG6303	FBgn0037808	Bruce	FBtr0082239
CG6304	FBgn0032624	NA	FBtr0080955
CG6305	FBgn0033869	CG6305	FBtr0087630
CG6310	FBgn0036121	NA	FBtr0076239
CG6329	FBgn0033872	CG6329	FBtr0087633
CG6331	FBgn0019952	NA	FBtr0084599
CG6335	FBgn0027087	NA	FBtr0074594
CG6337	FBgn0033873	CG6337	FBtr0087646
CG6341	FBgn0028737	NA	FBtr0087041
CG6342	FBgn0024957	NA	FBtr0082259
CG6345	FBgn0037816	NA	FBtr0082258
CG6347	FBgn0033874	CG6347	FBtr0087637
CG6348	FBgn0003267	NA	FBtr0085091
CG6349	FBgn0259113	NA	FBtr0084115
CG6352	FBgn0026058	NA	FBtr0074461
CG6353	FBgn0038893	NA	FBtr0084116
CG6355	FBgn0028741	NA	FBtr0089554
CG6356	FBgn0039178	NA	FBtr0084601
CG6357	FBgn0033875	NA	FBtr0087638
CG6358	FBgn0004832	NA	FBtr0070670
CG6362	FBgn0028740	NA	FBtr0089556
CG6363	FBgn0027378	MRG15	FBtr0083125
CG6367	FBgn0030926	NA	FBtr0074629
CG6369	FBgn0039260	NA	FBtr0084796
CG6370	FBgn0034277	CG6370	FBtr0089559
CG6376	FBgn0011766	E2f	FBtr0084119
CG6382	FBgn0020443	Elf	FBtr0080369
CG6384	FBgn0000283	NA	FBtr0083127
CG6385	FBgn0034276	NA	FBtr0089560
CG6386	FBgn0027889	NA	FBtr0085096
CG6394	FBgn0030930	GalNAc-T2	FBtr0074625

CG6398	FBgn0030870	CG6398	FBtr0074462
CG6401	FBgn0034270	NA	FBtr0086882
CG6403	FBgn0039453	NA	FBtr0085044
CG6404	FBgn0027615	CG6404	FBtr0076272
CG6405	FBgn0032428	NA	FBtr0080366
CG6409	FBgn0036106	NA	FBtr0076273
CG6413	FBgn0039183	NA	FBtr0084605
CG6414	FBgn0029690	NA	FBtr0070624
CG6415	FBgn0032287	CG6415	FBtr0080108
CG6417	FBgn0032435	Oatp33Eb	FBtr0080370
CG6420	FBgn0039451	NA	FBtr0085046
CG6421	FBgn0025827	NA	FBtr0087043
CG6425	FBgn0039449	NA	FBtr0085047
CG6426	FBgn0034162	NA	FBtr0087042
CG6428	FBgn0029689	CG6428	FBtr0070625
CG6429	FBgn0046999	CG6429	FBtr0087044
CG6432	FBgn0039184	NA	FBtr0084606
CG6434	FBgn0036972	NA	FBtr0074844
CG6435	FBgn0034165	NA	FBtr0087045
CG6438	FBgn0023179	amon	FBtr0085048
CG6440	FBgn0011581	Dms	FBtr0084607
CG6447	FBgn0039437	NA	FBtr0085055
CG6450	FBgn0029688	NA	FBtr0070626
CG6451	FBgn0041161	NA	FBtr0075781
CG6452	FBgn0039438	NA	FBtr0085054
CG6456	FBgn0036713	Mip	FBtr0075241
CG6457	FBgn0040060	yip7	FBtr0077038
CG6460	FBgn0039439	NA	FBtr0085053
CG6461	FBgn0030932	CG6461	FBtr0074622
CG6462	FBgn0035663	NA	FBtr0077039
CG6463	FBgn0047038	NA	FBtr0076276
CG6464	FBgn0261648	NA	FBtr0089913
CG6465	FBgn0037818	CG6465	FBtr0082323
CG6467	FBgn0250815	Jon65Aiv	FBtr0077040
CG6470	FBgn0030933	NA	FBtr0074621
CG6472	FBgn0034166	NA	FBtr0087046
CG6478	FBgn0039436	CG6478	FBtr0085057
CG6479	FBgn0036710	NA	FBtr0075245
CG6481	FBgn0030936	NA	FBtr0074620
CG6488	FBgn0032361	NA	FBtr0080248
CG6493	FBgn0034246	NA	FBtr0086904
CG6494	FBgn0001168	h	FBtr0100153
CG6495	FBgn0027550	CG6495	FBtr0080114
CG6508	FBgn0032303	NA	FBtr0080139
CG6509	FBgn0032363	NA	FBtr0080246
CG6510	FBgn0010409	RpL18A	FBtr0086947
CG6518	FBgn0004784	inaC	FBtr0087047
CG6521	FBgn0027363	Stam	FBtr0080143
CG6523	FBgn0032509	CG6523	FBtr0080465
CG6530	FBgn0028956	NA	FBtr0086955
CG6531	FBgn0030941	wgn	FBtr0074617
CG6534	FBgn0002941	NA	FBtr0084123
CG6542	FBgn0027506	EDTP	FBtr0086958
CG6545	FBgn0011278	NA	FBtr0084091
CG6546	FBgn0025716	Bap55	FBtr0086960
CG6550	FBgn0034214	CG6550	FBtr0086961
CG6551	FBgn0001079	NA	FBtr0074602

CG6556	FBgn0021818	NA	FBtr0086962
CG6565	FBgn0032513	NA	FBtr0080468
CG6571	FBgn0004366	rdgC	FBtr0074835
CG6575	FBgn0015229	NA	FBtr0084157
CG6577	FBgn0011569	can	FBtr0076299
CG6578	FBgn0004959	phm	FBtr0074603
CG6592	FBgn0035669	NA	FBtr0077044
CG6593	FBgn0003134	NA	FBtr0084642
CG6604	FBgn0016660	NA	FBtr0079076
CG6614	FBgn0032369	NA	FBtr0080267
CG6619	FBgn0035676	CG6619	FBtr0077049
CG6620	FBgn0024227	NA	FBtr0080180
CG6627	FBgn0027453	NA	FBtr0080181
CG6629	FBgn0037860	NA	FBtr0082380
CG6632	FBgn0030945	NA	FBtr0074614
CG6642	FBgn0011293	NA	FBtr0075290
CG6649	FBgn0026314	NA	FBtr0082375
CG6652	FBgn0036687	NA	FBtr0075291
CG6657	FBgn0015562	NA	FBtr0087052
CG6659	FBgn0030946	NA	FBtr0074613
CG6662	FBgn0035907	CG6662	FBtr0076652
CG6663	FBgn0036969	NA	FBtr0074843
CG6664	FBgn0036685	NA	FBtr0100439
CG6665	FBgn0034172	CG6665	FBtr0087053
CG6668	FBgn0039213	atl	FBtr0084657
CG6669	FBgn0017590	NA	FBtr0084337
CG6673	FBgn0035906	CG6673	FBtr0076654
CG6678	FBgn0038917	CG6678	FBtr0084148
CG6679	FBgn0039034	NA	FBtr0084342
CG6683	FBgn0035902	NA	FBtr0076655
CG6687	FBgn0038299	NA	FBtr0083140
CG6691	FBgn0032971	NA	FBtr0085935
CG6695	FBgn0039215	CG6695	FBtr0089916
CG6696	FBgn0030947	NA	FBtr0074611
CG6702	FBgn0004580	NA	FBtr0087056
CG6705	FBgn0003867	tsl	FBtr0084164
CG6706	FBgn0027575	NA	FBtr0084168
CG6707	FBgn0036058	NA	FBtr0076357
CG6713	FBgn0011676	Nos	FBtr0100491
CG6716	FBgn0003145	prd	FBtr0080298
CG6720	FBgn0015320	UbcD2	FBtr0080115
CG6721	FBgn0004390	NA	FBtr0076368
CG6727	FBgn0029084	NA	FBtr0071766
CG6729	FBgn0032296	NA	FBtr0080119
CG6730	FBgn0031925	NA	FBtr0079471
CG6734	FBgn0032395	NA	FBtr0080312
CG6736	FBgn0044049	Ilp4	FBtr0076372
CG6739	FBgn0031926	CG6739	FBtr0079472
CG6745	FBgn0035901	CG6745	FBtr0076620
CG6756	FBgn0032397	Tom70	FBtr0080310
CG6759	FBgn0025781	NA	FBtr0084377
CG6760	FBgn0013563	l(3)70Da	FBtr0075777
CG6761	FBgn0036031	NA	FBtr0076414
CG6765	FBgn0035903	CG6765	FBtr0076621
CG6766	FBgn0032398	CG6766	FBtr0080308
CG6768	FBgn0020756	NA	FBtr0084408
CG6770	FBgn0032400	NA	FBtr0080306

CG6776	FBgn0035904	CG6776	FBtr0076622
CG6778	FBgn0027088	Aats-gly	FBtr0075633
CG6781	FBgn0086348	NA	FBtr0076623
CG6782	FBgn0037912	NA	FBtr0100464
CG6783	FBgn0037913	NA	FBtr0100323
CG6785	FBgn0032399	NA	FBtr0080307
CG6789	FBgn0029745	NA	FBtr0070741
CG6791	FBgn0037918	CG6791	FBtr0082458
CG6792	FBgn0032401	NA	FBtr0080305
CG6796	FBgn0034177	NA	FBtr0087057
CG6800	FBgn0038902	NA	FBtr0084132
CG6801	FBgn0011335	NA	FBtr0075991
CG6812	FBgn0036843	CG6812	FBtr0075025
CG6816	FBgn0010383	Cyp18a1	FBtr0074610
CG6817	FBgn0024236	NA	FBtr0076624
CG6818	FBgn0036846	MESR6	FBtr0075026
CG6822	FBgn0035909	ergic53	FBtr0076626
CG6830	FBgn0037934	CG6830	FBtr0082439
CG6834	FBgn0037935	NA	FBtr0082438
CG6836	FBgn0036834	NA	FBtr0075023
CG6844	FBgn0000039	nAcRalpha-96Ab	FBtr0084639
CG6846	FBgn0036825	NA	FBtr0075066
CG6847	FBgn0030884	CG6847	FBtr0074505
CG6850	FBgn0014075	NA	FBtr0075064
CG6854	FBgn0262707	CG6854	FBtr0075636
CG6859	FBgn0036484	CG6859	FBtr0075640
CG6860	FBgn0032633	CG6860	FBtr0080969
CG6863	FBgn0004885	tok	FBtr0084690
CG6869	FBgn0036485	FucTA	FBtr0075641
CG6871	FBgn0000261	Cat	FBtr0075058
CG6874	FBgn0036815	NA	FBtr0075056
CG6875	FBgn0000140	NA	FBtr0084692
CG6876	FBgn0036487	CG6876	FBtr0075642
CG6878	FBgn0036488	NA	FBtr0075643
CG6884	FBgn0036811	NA	FBtr0075054
CG6885	FBgn0036810	CG6885	FBtr0075053
CG6888	FBgn0036490	NA	FBtr0075644
CG6889	FBgn0040071	tara	FBtr0083257
CG6890	FBgn0029114	NA	FBtr0075607
CG6891	FBgn0030955	NA	FBtr0074631
CG6892	FBgn0039225	NA	FBtr0084697
CG6895	FBgn0040323	NA	FBtr0075050
CG6896	FBgn0036801	NA	FBtr0075049
CG6897	FBgn0259791	CG6897	FBtr0075108
CG6898	FBgn0038412	NA	FBtr0083266
CG6903	FBgn0029737	NA	FBtr0070723
CG6904	FBgn0038293	NA	FBtr0083036
CG6906	FBgn0027843	NA	FBtr0076001
CG6908	FBgn0037936	NA	FBtr0082437
CG6913	FBgn0037937	NA	FBtr0082435
CG6914	FBgn0037172	NA	FBtr0078515
CG6920	FBgn0002906	NA	FBtr0082434
CG6921	FBgn0260942	CG6921	FBtr0084261
CG6928	FBgn0036240	CG6928	FBtr0076050
CG6932	FBgn0028837	CSN6	FBtr0084265
CG6939	FBgn0025802	NA	FBtr0082529
CG6953	FBgn0026721	fat-spondin	FBtr0086976

CG6971	FBgn0037962	NA	FBtr0082514
CG6972	FBgn0039008	CG6972	FBtr0084305
CG6974	FBgn0038285	NA	FBtr0083042
CG6975	FBgn0005198	NA	FBtr0074819
CG6978	FBgn0029727	NA	FBtr0070715
CG6980	FBgn0039228	CG6980	FBtr0084698
CG6984	FBgn0034191	CG6984	FBtr0086979
CG6985	FBgn0039017	CG6985	FBtr0084308
CG6986	FBgn0029723	NA	FBtr0070713
CG6995	FBgn0039229	NA	FBtr0084699
CG7002	FBgn0029167	NA	FBtr0075753
CG7003	FBgn0036486	NA	FBtr0075646
CG7004	FBgn0004373	NA	FBtr0072516
CG7008	FBgn0035121	Tudor-SN	FBtr0072519
CG7010	FBgn0028325	NA	FBtr0070710
CG7011	FBgn0036489	CG7011	FBtr0075645
CG7021	FBgn0013949	NA	FBtr0084708
CG7024	FBgn0029722	NA	FBtr0070712
CG7031	FBgn0039027	NA	FBtr0084313
CG7034	FBgn0038856	NA	FBtr0084034
CG7035	FBgn0022942	NA	FBtr0070684
CG7037	FBgn0020224	NA	FBtr0076615
CG7038	FBgn0029718	NA	FBtr0070682
CG7044	FBgn0038854	NA	FBtr0084035
CG7045	FBgn0038978	NA	FBtr0084258
CG7052	FBgn0041182	TepII	FBtr0079510
CG7055	FBgn0030093	NA	FBtr0071300
CG7059	FBgn0038957	NA	FBtr0084219
CG7062	FBgn0015793	Rab-RP3	FBtr0076705
CG7065	FBgn0030091	NA	FBtr0071299
CG7066	FBgn0087039	NA	FBtr0076707
CG7068	FBgn0041181	TepIII	FBtr0079477
CG7069	FBgn0038952	NA	FBtr0084215
CG7070	FBgn0003178	PyK	FBtr0084214
CG7074	FBgn0031399	NA	FBtr0077835
CG7082	FBgn0031401	CG7082	FBtr0077837
CG7085	FBgn0010704	l(2)s5379	FBtr0077840
CG7094	FBgn0032650	NA	FBtr0080990
CG7097	FBgn0263395	NA	FBtr0086504
CG7099	FBgn0032517	NA	FBtr0080499
CG7102	FBgn0031961	NA	FBtr0079559
CG7104	FBgn0031959	Spz3	FBtr0079561
CG7105	FBgn0045038	Proct	FBtr0079562
CG7109	FBgn0004177	mts	FBtr0079525
CG7111	FBgn0020618	NA	FBtr0079565
CG7118	FBgn0035886	NA	FBtr0076608
CG7121	FBgn0026760	NA	FBtr0080502
CG7126	FBgn0038602	NA	FBtr0083593
CG7127	FBgn0035892	exo70	FBtr0076612
CG7129	FBgn0010877	l(3)05822	FBtr0083595
CG7143	FBgn0037141	NA	FBtr0078489
CG7144	FBgn0025687	NA	FBtr0079495
CG7146	FBgn0038593	NA	FBtr0083601
CG7149	FBgn0031948	CG7149	FBtr0079496
CG7154	FBgn0031947	CG7154	FBtr0079488
CG7158	FBgn0037116	NA	FBtr0078462
CG7160	FBgn0037114	NA	FBtr0078463

CG7161	FBgn0035891	NA	FBtr0076663
CG7164	FBgn0031946	NA	FBtr0079487
CG7169	FBgn0037105	NA	FBtr0078466
CG7170	FBgn0035887	NA	FBtr0076666
CG7183	FBgn0038583	NA	FBtr0083610
CG7191	FBgn0031945	NA	FBtr0079485
CG7196	FBgn0031944	NA	FBtr0079482
CG7197	FBgn0035866	CG7197	FBtr0076715
CG7201	FBgn0035865	CG7201	FBtr0076716
CG7207	FBgn0027569	NA	FBtr0076717
CG7210	FBgn0001301	kel	FBtr0081042
CG7211	FBgn0031941	NA	FBtr0079481
CG7213	FBgn0035861	NA	FBtr0076718
CG7214	FBgn0031940	NA	FBtr0079499
CG7216	FBgn0014454	NA	FBtr0079500
CG7222	FBgn0033551	CG7222	FBtr0088223
CG7223	FBgn0010389	htl	FBtr0083548
CG7225	FBgn0004003	NA	FBtr0086510
CG7229	FBgn0034423	NA	FBtr0086511
CG7230	FBgn0003254	rib	FBtr0086512
CG7241	FBgn0038095	Cyp304a1	FBtr0082691
CG7242	FBgn0087021	NA	FBtr0082690
CG7254	FBgn0004507	GlyP	FBtr0077828
CG7257	FBgn0036224	CG7257	FBtr0076040
CG7260	FBgn0011723	NA	FBtr0076039
CG7261	FBgn0027509	NA	FBtr0077829
CG7263	FBgn0031392	NA	FBtr0077831
CG7267	FBgn0030079	NA	FBtr0071354
CG7275	FBgn0036500	CG7275	FBtr0075547
CG7285	FBgn0036790	NA	FBtr0089578
CG7293	FBgn0004381	Klp68D	FBtr0076030
CG7303	FBgn0041231	NA	FBtr0076118
CG7313	FBgn0036783	NA	FBtr0075129
CG7314	FBgn0036199	Bmcp	FBtr0076115
CG7324	FBgn0037074	NA	FBtr0078404
CG7326	FBgn0030970	NA	FBtr0074653
CG7328	FBgn0036942	CG7328	FBtr0074864
CG7330	FBgn0036780	NA	FBtr0075127
CG7332	FBgn0030973	CG7332	FBtr0074647
CG7339	FBgn0036188	NA	FBtr0076107
CG7341	FBgn0036777	CG7341	FBtr0075124
CG7349	FBgn0030975	CG7349	FBtr0074648
CG7351	FBgn0036184	NA	FBtr0076102
CG7354	FBgn0036774	mRpS26	FBtr0075121
CG7356	FBgn0031975	CG7356	FBtr0079533
CG7358	FBgn0030974	NA	FBtr0074652
CG7362	FBgn0038258	NA	FBtr0083015
CG7365	FBgn0036939	NA	FBtr0074867
CG7366	FBgn0035855	NA	FBtr0076721
CG7369	FBgn0037188	CG7369	FBtr0078530
CG7372	FBgn0036522	NA	FBtr0075570
CG7375	FBgn0035853	CG7375	FBtr0076722
CG7376	FBgn0035689	NA	FBtr0076998
CG7377	FBgn0036176	NA	FBtr0076100
CG7383	FBgn0000560	NA	FBtr0078438
CG7395	FBgn0036934	NPFR76F	FBtr0074871
CG7398	FBgn0024921	NA	FBtr0077000

CG7399	FBgn0001208	Hn	FBtr0100225
CG7405	FBgn0022936	NA	FBtr0078439
CG7406	FBgn0030980	NA	FBtr0074664
CG7411	FBgn0003011	NA	FBtr0083784
CG7413	FBgn0015799	Rbf	FBtr0070146
CG7430	FBgn0036762	CG7430	FBtr0075140
CG7436	FBgn0020392	NA	FBtr0076728
CG7441	FBgn0036763	NA	FBtr0075141
CG7442	FBgn0037140	NA	FBtr0078471
CG7446	FBgn0001134	NA	FBtr0075139
CG7448	FBgn0037143	NA	FBtr0078474
CG7449	FBgn0029082	NA	FBtr0087414
CG7454	FBgn0037576	NA	FBtr0081836
CG7458	FBgn0037144	NA	FBtr0078475
CG7465	FBgn0035551	NA	FBtr0073306
CG7466	FBgn0031981	NA	FBtr0079541
CG7476	FBgn0035847	NA	FBtr0076726
CG7478	FBgn0000045	NA	FBtr0078478
CG7479	FBgn0027085	Aats-leu	FBtr0073348
CG7483	FBgn0037573	NA	FBtr0081834
CG7484	FBgn0036745	NA	FBtr0075184
CG7487	FBgn0040290	RecQ4	FBtr0076729
CG7508	FBgn0010433	NA	FBtr0081828
CG7509	FBgn0035575	NA	FBtr0073362
CG7510	FBgn0036741	CG7510	FBtr0075182
CG7528	FBgn0029113	NA	FBtr0076734
CG7529	FBgn0037090	NA	FBtr0078354
CG7530	FBgn0038256	NA	FBtr0083019
CG7536	FBgn0030890	CG7536	FBtr0074531
CG7537	FBgn0030989	NA	FBtr0074698
CG7538	FBgn0014861	Mcm2	FBtr0081827
CG7542	FBgn0036738	NA	FBtr0075181
CG7550	FBgn0035839	NA	FBtr0076736
CG7554	FBgn0041160	comm2	FBtr0075580
CG7557	FBgn0036159	NA	FBtr0076174
CG7558	FBgn0262716	NA	FBtr0076737
CG7560	FBgn0036157	NA	FBtr0076173
CG7562	FBgn0010287	Trf	FBtr0079542
CG7570	FBgn0036924	NA	FBtr0074912
CG7576	FBgn0005586	Rab3	FBtr0088257
CG7577	FBgn0039676	NA	FBtr0085466
CG7578	FBgn0028538	NA	FBtr0080537
CG7579	FBgn0036528	NA	FBtr0075581
CG7583	FBgn0020496	NA	FBtr0100161
CG7586	FBgn0020240	NA	FBtr0079543
CG7588	FBgn0014850	NA	FBtr0075582
CG7597	FBgn0037093	CG7597	FBtr0078357
CG7602	FBgn0037554	NA	FBtr0081798
CG7620	FBgn0002354	l(3)87Df	FBtr0082703
CG7622	FBgn0002579	NA	FBtr0070158
CG7637	FBgn0033548	NA	FBtr0088252
CG7642	FBgn0003308	ry	FBtr0082704
CG7655	FBgn0038536	CG7655	FBtr0083479
CG7659	FBgn0015550	tap	FBtr0075216
CG7664	FBgn0001994	NA	FBtr0080854
CG7665	FBgn0016650	Fsh	FBtr0083510
CG7668	FBgn0036929	NA	FBtr0074904

CG7673	FBgn0000551	NA	FBtr0078408
CG7675	FBgn0038610	CG7675	FBtr0083582
CG7678	FBgn0038613	NA	FBtr0083658
CG7692	FBgn0036714	NA	FBtr0075215
CG7697	FBgn0027841	CstF-64	FBtr0083660
CG7702	FBgn0038638	CG7702	FBtr0083665
CG7708	FBgn0038641	CG7708	FBtr0083669
CG7709	FBgn0038642	NA	FBtr0083687
CG7712	FBgn0033570	NA	FBtr0088238
CG7714	FBgn0038645	NA	FBtr0083686
CG7715	FBgn0038646	CG7715	FBtr0083685
CG7724	FBgn0036698	CG7724	FBtr0075284
CG7725	FBgn0036697	rogdi	FBtr0075282
CG7728	FBgn0036686	NA	FBtr0075274
CG7729	FBgn0036688	NA	FBtr0075275
CG7730	FBgn0036689	NA	FBtr0089584
CG7734	FBgn0003396	NA	FBtr0088101
CG7736	FBgn0037084	Syx6	FBtr0088102
CG7737	FBgn0033584	CG7737	FBtr0088105
CG7748	FBgn0011336	OstStt3	FBtr0084786
CG7749	FBgn0261574	NA	FBtr0100509
CG7752	FBgn0259785	Z4	FBtr0078336
CG7753	FBgn0002716	NA	FBtr0086461
CG7754	FBgn0015001	NA	FBtr0088125
CG7760	FBgn0024249	NA	FBtr0087183
CG7762	FBgn0028695	NA	FBtr0074893
CG7769	FBgn0260962	NA	FBtr0082709
CG7770	FBgn0036918	NA	FBtr0074894
CG7771	FBgn0004666	sim	FBtr0082711
CG7776	FBgn0000581	NA	FBtr0088139
CG7778	FBgn0032025	CG7778	FBtr0079606
CG7781	FBgn0032021	NA	FBtr0079609
CG7786	FBgn0034096	NA	FBtr0087190
CG7788	FBgn0019972	Ice	FBtr0085482
CG7789	FBgn0039698	NA	FBtr0085483
CG7791	FBgn0033038	NA	FBtr0086057
CG7793	FBgn0001965	NA	FBtr0080541
CG7794	FBgn0038565	NA	FBtr0083540
CG7798	FBgn0034092	NA	FBtr0087192
CG7802	FBgn0039704	NA	FBtr0085486
CG7804	FBgn0036496	NA	FBtr0075617
CG7806	FBgn0032018	NA	FBtr0079600
CG7807	FBgn0261953	AP-2	FBtr0078418
CG7809	FBgn0036919	NA	FBtr0074895
CG7810	FBgn0032017	NA	FBtr0079612
CG7813	FBgn0034133	NA	FBtr0087126
CG7815	FBgn0036497	NA	FBtr0075616
CG7818	FBgn0032016	CG7818	FBtr0079599
CG7823	FBgn0036921	RhoGDI	FBtr0074896
CG7829	FBgn0039703	NA	FBtr0085531
CG7830	FBgn0032015	CG7830	FBtr0079613
CG7831	FBgn0002924	ncd	FBtr0085534
CG7834	FBgn0039697	CG7834	FBtr0085535
CG7837	FBgn0039696	NA	FBtr0085537
CG7838	FBgn0263855	NA	FBtr0086032
CG7839	FBgn0036124	CG7839	FBtr0076207
CG7840	FBgn0032014	CG7840	FBtr0079598

CG7848	FBgn0034127	NA	FBtr0087160
CG7850	FBgn0243512	NA	FBtr0081792
CG7851	FBgn0032013	Scgalpha	FBtr0079597
CG7852	FBgn0035229	NA	FBtr0072737
CG7855	FBgn0038118	timeout	FBtr0082712
CG7856	FBgn0033056	NA	FBtr0086024
CG7860	FBgn0030653	CG7860	FBtr0074045
CG7863	FBgn0033051	dream	FBtr0086028
CG7864	FBgn0035233	NA	FBtr0072743
CG7870	FBgn0261020	CG7870	FBtr0079614
CG7873	FBgn0004603	Src42A	FBtr0085968
CG7875	FBgn0003861	NA	FBtr0085513
CG7876	FBgn0031000	CG7876	FBtr0074673
CG7879	FBgn0035235	NA	FBtr0072826
CG7881	FBgn0033048	CG7881	FBtr0086033
CG7884	FBgn0031001	NA	FBtr0074674
CG7889	FBgn0031003	CG7889	FBtr0074675
CG7890	FBgn0031005	NA	FBtr0074676
CG7891	FBgn0037551	CG7891	FBtr0081794
CG7895	FBgn0004110	tin	FBtr0084086
CG7896	FBgn0039728	NA	FBtr0085545
CG7897	FBgn0033039	NA	FBtr0086044
CG7899	FBgn0000032	NA	FBtr0085609
CG7902	FBgn0004862	bap	FBtr0084087
CG7903	FBgn0039730	NA	FBtr0085608
CG7910	FBgn0037547	NA	FBtr0081790
CG7914	FBgn0030995	NA	FBtr0074696
CG7917	FBgn0016685	NA	FBtr0085552
CG7918	FBgn0037546	NA	FBtr0081789
CG7921	FBgn0039738	Mgat2	FBtr0085602
CG7927	FBgn0027549	CG7927	FBtr0076684
CG7928	FBgn0039740	NA	FBtr0085600
CG7929	FBgn0041102	NA	FBtr0085599
CG7931	FBgn0001281	NA	FBtr0085598
CG7935	FBgn0026252	msk	FBtr0076685
CG7937	FBgn0004863	NA	FBtr0084111
CG7938	FBgn0003511	Sry-beta	FBtr0085556
CG7942	FBgn0035838	NA	FBtr0076686
CG7949	FBgn0036107	NA	FBtr0076270
CG7952	FBgn0001150	NA	FBtr0070460
CG7955	FBgn0035244	CG7955	FBtr0072756
CG7960	FBgn0013755	NA	FBtr0072760
CG7962	FBgn0010350	CdsA	FBtr0076688
CG7966	FBgn0038115	NA	FBtr0082715
CG7968	FBgn0028532	NA	FBtr0080551
CG7975	FBgn0034658	NA	FBtr0071714
CG7985	FBgn0028499	NA	FBtr0083559
CG7987	FBgn0038244	CG7987	FBtr0082979
CG7992	FBgn0031004	CG7992	FBtr0074690
CG8002	FBgn0031006	NA	FBtr0074689
CG8003	FBgn0036096	CG8003	FBtr0076260
CG8004	FBgn0036920	NA	FBtr0074915
CG8005	FBgn0035854	NA	FBtr0076695
CG8013	FBgn0020887	NA	FBtr0074916
CG8014	FBgn0015477	Rme-8	FBtr0088618
CG8023	FBgn0035860	NA	FBtr0076701
CG8024	FBgn0002567	NA	FBtr0089931

CG8027	FBgn0033392	CG8027	FBtr0088609
CG8032	FBgn0037606	CG8032	FBtr0081880
CG8036	FBgn0037607	CG8036	FBtr0081881
CG8042	FBgn0027554	NA	FBtr0076766
CG8050	FBgn0004629	NA	FBtr0082980
CG8051	FBgn0031012	NA	FBtr0074683
CG8053	FBgn0026250	NA	FBtr0083573
CG8062	FBgn0259834	CG8062	FBtr0074682
CG8063	FBgn0038105	NA	FBtr0082722
CG8066	FBgn0038243	NA	FBtr0082981
CG8072	FBgn0036070	NA	FBtr0076295
CG8073	FBgn0033377	NA	FBtr0088630
CG8078	FBgn0033375	CG8078	FBtr0088631
CG8079	FBgn0034002	NA	FBtr0087368
CG8087	FBgn0038241	NA	FBtr0082983
CG8092	FBgn0033998	CG8092	FBtr0087402
CG8095	FBgn0003328	NA	FBtr0087369
CG8098	FBgn0024315	Picot	FBtr0087114
CG8102	FBgn0034007	NA	FBtr0087394
CG8105	FBgn0030661	NA	FBtr0074048
CG8118	FBgn0002643	NA	FBtr0087596
CG8119	FBgn0030664	NA	FBtr0074051
CG8121	FBgn0037680	NA	FBtr0082019
CG8129	FBgn0037684	CG8129	FBtr0082026
CG8132	FBgn0037687	CG8132	FBtr0082029
CG8152	FBgn0034008	CG8152	FBtr0087392
CG8155	FBgn0034009	NA	FBtr0087391
CG8156	FBgn0013750	Arf51F	FBtr0087377
CG8157	FBgn0034010	NA	FBtr0087390
CG8160	FBgn0034011	NA	FBtr0087389
CG8165	FBgn0037703	NA	FBtr0082063
CG8166	FBgn0034013	NA	FBtr0087382
CG8169	FBgn0011660	NA	FBtr0087388
CG8175	FBgn0014865	NA	FBtr0087386
CG8178	FBgn0024319	NA	FBtr0089588
CG8180	FBgn0034021	CG8180	FBtr0087359
CG8182	FBgn0034025	GalNAc-T1	FBtr0087332
CG8183	FBgn0019968	NA	FBtr0087356
CG8184	FBgn0030674	NA	FBtr0074082
CG8188	FBgn0030863	NA	FBtr0100463
CG8190	FBgn0034029	NA	FBtr0087336
CG8191	FBgn0030675	CG8191	FBtr0074083
CG8192	FBgn0034030	NA	FBtr0087354
CG8194	FBgn0010406	NA	FBtr0076752
CG8196	FBgn0033366	Ance-4	FBtr0088664
CG8198	FBgn0026666	NA	FBtr0074084
CG8199	FBgn0037709	CG8199	FBtr0082067
CG8200	FBgn0024754	Flo	FBtr0087352
CG8202	FBgn0037622	NA	FBtr0081914
CG8203	FBgn0013762	NA	FBtr0087350
CG8204	FBgn0034033	NA	FBtr0087349
CG8208	FBgn0027950	NA	FBtr0082069
CG8211	FBgn0030858	NA	FBtr0074480
CG8219	FBgn0035693	NA	FBtr0077003
CG8221	FBgn0020506	NA	FBtr0087117
CG8228	FBgn0261049	CG8228	FBtr0082070
CG8238	FBgn0040491	NA	FBtr0088077

CG8239	FBgn0030683	NA	FBtr0074087
CG8245	FBgn0033031	CG8245	FBtr0086036
CG8246	FBgn0003130	NA	FBtr0087311
CG8250	FBgn0040505	NA	FBtr0087118
CG8251	FBgn0003074	NA	FBtr0088679
CG8252	FBgn0033344	NA	FBtr0088682
CG8254	FBgn0041156	exex	FBtr0076751
CG8258	FBgn0033342	NA	FBtr0088683
CG8260	FBgn0030684	NA	FBtr0074089
CG8261	FBgn0004921	NA	FBtr0088645
CG8262	FBgn0027513	NA	FBtr0088685
CG8271	FBgn0033657	NA	FBtr0088032
CG8274	FBgn0013756	NA	FBtr0088033
CG8276	FBgn0263144	NA	FBtr0085993
CG8285	FBgn0000206	NA	FBtr0084949
CG8289	FBgn0030854	CG8289	FBtr0074484
CG8290	FBgn0026573	NA	FBtr0088042
CG8291	FBgn0034049	NA	FBtr0087299
CG8299	FBgn0034052	NA	FBtr0087291
CG8300	FBgn0029937	NA	FBtr0071015
CG8312	FBgn0037720	CG8312	FBtr0082075
CG8316	FBgn0030852	NA	FBtr0074485
CG8321	FBgn0033677	CG8321	FBtr0087975
CG8326	FBgn0030851	CG8326	FBtr0074486
CG8334	FBgn0036913	NA	FBtr0074890
CG8344	FBgn0004463	NA	FBtr0087977
CG8351	FBgn0037632	NA	FBtr0081918
CG8357	FBgn0024732	NA	FBtr0087978
CG8364	FBgn0028407	NA	FBtr0087979
CG8366	FBgn0034054	CG8366	FBtr0087201
CG8368	FBgn0035707	NA	FBtr0076977
CG8370	FBgn0034060	NA	FBtr0087203
CG8378	FBgn0027495	NA	FBtr0087983
CG8386	FBgn0034061	CG8386	FBtr0087264
CG8388	FBgn0034062	CG8388	FBtr0087263
CG8389	FBgn0034063	NA	FBtr0087205
CG8392	FBgn0010590	NA	FBtr0087262
CG8395	FBgn0034065	NA	FBtr0087206
CG8397	FBgn0034066	NA	FBtr0087261
CG8399	FBgn0034067	NA	FBtr0087207
CG8402	FBgn0005777	PpD3	FBtr0082128
CG8404	FBgn0005613	NA	FBtr0087576
CG8405	FBgn0034071	CG8405	FBtr0087210
CG8411	FBgn0005695	gcl	FBtr0088710
CG8415	FBgn0033912	NA	FBtr0087575
CG8416	FBgn0014020	Rho1	FBtr0087212
CG8420	FBgn0037664	NA	FBtr0081973
CG8426	FBgn0033029	NA	FBtr0086062
CG8428	FBgn0086676	NA	FBtr0089590
CG8430	FBgn0001124	Got1	FBtr0087232
CG8431	FBgn0027091	Aats-cys	FBtr0087234
CG8432	FBgn0026378	Rep	FBtr0086462
CG8442	FBgn0004619	Glu-RI	FBtr0076982
CG8443	FBgn0034087	CG8443	FBtr0087242
CG8444	FBgn0037671	CG8444	FBtr0081978
CG8445	FBgn0262166	CG8445	FBtr0087245
CG8449	FBgn0038129	NA	FBtr0082778

CG8451	FBgn0031998	NA	FBtr0079637
CG8454	FBgn0261241	Vps16A	FBtr0081979
CG8458	FBgn0038134	NA	FBtr0082775
CG8464	FBgn0038233	NA	FBtr0082987
CG8468	FBgn0033913	CG8468	FBtr0087534
CG8474	FBgn0025874	NA	FBtr0075791
CG8476	FBgn0038127	NA	FBtr0082781
CG8479	FBgn0261276	CG8479	FBtr0087574
CG8483	FBgn0038126	CG8483	FBtr0082782
CG8485	FBgn0033915	NA	FBtr0087570
CG8489	FBgn0038225	NA	FBtr0082940
CG8494	FBgn0033916	NA	FBtr0087535
CG8497	FBgn0026374	NA	FBtr0074096
CG8500	FBgn0037754	NA	FBtr0082139
CG8503	FBgn0033917	CG8503	FBtr0087536
CG8505	FBgn0033728	CG8505	FBtr0087920
CG8507	FBgn0037756	CG8507	FBtr0082140
CG8510	FBgn0033729	NA	FBtr0087921
CG8511	FBgn0033730	NA	FBtr0087922
CG8522	FBgn0261283	HLH106	FBtr0074874
CG8523	FBgn0010241	NA	FBtr0087537
CG8539	FBgn0035791	NA	FBtr0076831
CG8540	FBgn0035790	NA	FBtr0076790
CG8542	FBgn0001220	Hsc70-5	FBtr0087568
CG8543	FBgn0035787	NA	FBtr0076863
CG8545	FBgn0033741	CG8545	FBtr0087934
CG8548	FBgn0024889	NA	FBtr0074881
CG8549	FBgn0035714	NA	FBtr0076943
CG8550	FBgn0033742	NA	FBtr0087935
CG8553	FBgn0261270	NA	FBtr0087564
CG8560	FBgn0035781	CG8560	FBtr0076868
CG8562	FBgn0035779	NA	FBtr0076854
CG8563	FBgn0035777	NA	FBtr0076852
CG8565	FBgn0030697	NA	FBtr0074103
CG8568	FBgn0030841	CG8568	FBtr0074436
CG8569	FBgn0033752	CG8569	FBtr0087940
CG8577	FBgn0033327	NA	FBtr0088708
CG8578	FBgn0030699	NA	FBtr0074104
CG8579	FBgn0001285	NA	FBtr0088704
CG8581	FBgn0011592	fra	FBtr0087822
CG8584	FBgn0033322	NA	FBtr0088724
CG8586	FBgn0033320	NA	FBtr0088725
CG8587	FBgn0033753	Cyp301a1	FBtr0087824
CG8590	FBgn0011606	NA	FBtr0070448
CG8596	FBgn0035767	CG8596	FBtr0076881
CG8597	FBgn0011640	NA	FBtr0076845
CG8598	FBgn0035766	eco	FBtr0076882
CG8602	FBgn0035763	CG8602	FBtr0076884
CG8604	FBgn0027356	Amph	FBtr0087828
CG8605	FBgn0035762	NA	FBtr0076838
CG8606	FBgn0035761	RhoGEF4	FBtr0076885
CG8607	FBgn0035760	NA	FBtr0076837
CG8611	FBgn0027602	NA	FBtr0074440
CG8612	FBgn0028648	mRpL50	FBtr0076891
CG8613	FBgn0033924	NA	FBtr0087546
CG8614	FBgn0024542	Neos	FBtr0076835
CG8615	FBgn0035753	NA	FBtr0076892

CG8616	FBgn0261111	CG8616	FBtr0076834
CG8625	FBgn0011604	Iswi	FBtr0087841
CG8630	FBgn0038130	CG8630	FBtr0082737
CG8631	FBgn0002775	msl-3	FBtr0076926
CG8634	FBgn0035737	NA	FBtr0076909
CG8635	FBgn0033317	CG8635	FBtr0088727
CG8636	FBgn0029629	NA	FBtr0070446
CG8638	FBgn0035736	CG8638	FBtr0076928
CG8644	FBgn0038133	NA	FBtr0082738
CG8648	FBgn0025832	NA	FBtr0087075
CG8649	FBgn0024238	NA	FBtr0074441
CG8651	FBgn0003862	NA	FBtr0082947
CG8652	FBgn0026754	NA	FBtr0087076
CG8657	FBgn0020930	NA	FBtr0087853
CG8661	FBgn0030837	NA	FBtr0074444
CG8663	FBgn0032946	nrv3	FBtr0081520
CG8665	FBgn0032945	CG8665	FBtr0081517
CG8669	FBgn0000370	crc	FBtr0081513
CG8674	FBgn0021856	l(2)k14505	FBtr0081482
CG8676	FBgn0261239	Hr39	FBtr0081479
CG8677	FBgn0026577	NA	FBtr0081477
CG8694	FBgn0002569	NA	FBtr0088747
CG8695	FBgn0002571	NA	FBtr0088758
CG8696	FBgn0002570	NA	FBtr0088759
CG8697	FBgn0002533	NA	FBtr0088761
CG8701	FBgn0033287	NA	FBtr0088740
CG8712	FBgn0033258	CG8712	FBtr0088823
CG8713	FBgn0033257	CG8713	FBtr0088780
CG8722	FBgn0033247	NA	FBtr0088862
CG8728	FBgn0033235	NA	FBtr0088882
CG8730	FBgn0026722	NA	FBtr0088850
CG8732	FBgn0263120	NA	FBtr0088699
CG8738	FBgn0033321	CG8738	FBtr0088705
CG8739	FBgn0086784	cmp44E	FBtr0301639
CG8745	FBgn0036381	CG8745	FBtr0075801
CG8746	FBgn0033330	NA	FBtr0088714
CG8750	FBgn0040812	NA	FBtr0075802
CG8759	FBgn0086904	Nacalpha	FBtr0087858
CG8765	FBgn0036900	NA	FBtr0089597
CG8770	FBgn0004623	Gbeta76C	FBtr0074949
CG8782	FBgn0022774	Oat	FBtr0074961
CG8783	FBgn0036397	NA	FBtr0075786
CG8785	FBgn0033760	CG8785	FBtr0087881
CG8787	FBgn0261823	NA	FBtr0087493
CG8795	FBgn0038139	NA	FBtr0082773
CG8811	FBgn0033757	muskelin	FBtr0087885
CG8818	FBgn0033751	NA	FBtr0087941
CG8819	FBgn0033749	NA	FBtr0087942
CG8821	FBgn0033748	NA	FBtr0087945
CG8824	FBgn0045063	NA	FBtr0087946
CG8827	FBgn0012037	Ance	FBtr0080552
CG8828	FBgn0033740	CG8828	FBtr0087948
CG8830	FBgn0033738	NA	FBtr0087950
CG8833	FBgn0036386	CG8833	FBtr0075792
CG8836	FBgn0033726	NA	FBtr0087955
CG8838	FBgn0031526	NA	FBtr0077588
CG8839	FBgn0033717	CG8839	FBtr0087960

CG8843	FBgn0031537	sec5	FBtr0077519
CG8844	FBgn0021967	Pdsw	FBtr0077521
CG8849	FBgn0031651	mRpL24	FBtr0079058
CG8853	FBgn0031550	NA	FBtr0077529
CG8858	FBgn0033698	NA	FBtr0088008
CG8863	FBgn0038145	NA	FBtr0082752
CG8865	FBgn0026376	Rgl	FBtr0075793
CG8866	FBgn0037679	CG8866	FBtr0082056
CG8867	FBgn0020906	NA	FBtr0079054
CG8869	FBgn0031654	NA	FBtr0079055
CG8870	FBgn0038144	NA	FBtr0082750
CG8871	FBgn0031653	NA	FBtr0079056
CG8874	FBgn0000723	NA	FBtr0082052
CG8878	FBgn0027504	NA	FBtr0088018
CG8888	FBgn0033679	CG8888	FBtr0088022
CG8896	FBgn0004364	NA	FBtr0086309
CG8908	FBgn0034493	NA	FBtr0086275
CG8910	FBgn0025833	NA	FBtr0087071
CG8912	FBgn0014870	NA	FBtr0087073
CG8913	FBgn0038465	NA	FBtr0083371
CG8914	FBgn0026136	NA	FBtr0086278
CG8922	FBgn0002590	NA	FBtr0074406
CG8927	FBgn0038405	CG8927	FBtr0100110
CG8933	FBgn0000611	NA	FBtr0074136
CG8937	FBgn0001216	NA	FBtr0075761
CG8938	FBgn0010226	GstS1	FBtr0087005
CG8939	FBgn0030720	NA	FBtr0074138
CG8945	FBgn0030815	NA	FBtr0074411
CG8946	FBgn0010591	Sply	FBtr0087007
CG8947	FBgn0250848	26-29-p	FBtr0075766
CG8948	FBgn0030685	NA	FBtr0074197
CG8954	FBgn0019890	Smg5	FBtr0080561
CG8956	FBgn0015011	Ahcy89E	FBtr0083379
CG8960	FBgn0035315	NA	FBtr0072871
CG8961	FBgn0086350	NA	FBtr0087014
CG8965	FBgn0031745	CG8965	FBtr0079158
CG8967	FBgn0004839	NA	FBtr0088028
CG8971	FBgn0030092	fh	FBtr0071348
CG8974	FBgn0030693	NA	FBtr0074185
CG8982	FBgn0002855	NA	FBtr0079155
CG8985	FBgn0035331	NA	FBtr0072887
CG8989	FBgn0004828	NA	FBtr0071346
CG8991	FBgn0033654	NA	FBtr0088064
CG8996	FBgn0010516	NA	FBtr0088082
CG9000	FBgn0034176	CG9000	FBtr0087059
CG9001	FBgn0034175	NA	FBtr0087060
CG9007	FBgn0036398	NA	FBtr0075768
CG9008	FBgn0028540	CG9008	FBtr0080577
CG9010	FBgn0034173	CG9010	FBtr0087067
CG9014	FBgn0028847	NA	FBtr0080579
CG9015	FBgn0000577	en	FBtr0088095
CG9016	FBgn0031751	NA	FBtr0079151
CG9018	FBgn0035318	CG9018	FBtr0072910
CG9019	FBgn0015381	NA	FBtr0079145
CG9020	FBgn0027093	Aats-arg	FBtr0074178
CG9021	FBgn0031747	NA	FBtr0079154
CG9022	FBgn0014868	Ost48	FBtr0071344

CG9023	FBgn0015872	NA	FBtr0088141
CG9024	FBgn0002856	NA	FBtr0079157
CG9028	FBgn0036389	CG9028	FBtr0075759
CG9034	FBgn0040931	NA	FBtr0071343
CG9036	FBgn0034499	CG9036	FBtr0086301
CG9045	FBgn0002914	NA	FBtr0074173
CG9053	FBgn0030606	CG9053	FBtr0073968
CG9060	FBgn0030096	Zpr1	FBtr0071342
CG9062	FBgn0033607	CG9062	FBtr0088181
CG9063	FBgn0028500	NA	FBtr0078512
CG9065	FBgn0030610	NA	FBtr0073966
CG9067	FBgn0033605	NA	FBtr0088182
CG9068	FBgn0034106	NA	FBtr0087138
CG9076	FBgn0033601	NA	FBtr0088188
CG9077	FBgn0033600	NA	FBtr0088189
CG9084	FBgn0033582	CG9084	FBtr0088197
CG9089	FBgn0030805	wus	FBtr0074418
CG9090	FBgn0034497	CG9090	FBtr0086303
CG9091	FBgn0030616	NA	FBtr0074027
CG9097	FBgn0004870	NA	FBtr0072642
CG9098	FBgn0031762	CG9098	FBtr0079228
CG9102	FBgn0025525	NA	FBtr0072639
CG9104	FBgn0030800	CG9104	FBtr0074348
CG9106	FBgn0030624	NA	FBtr0074021
CG9111	FBgn0004426	NA	FBtr0072629
CG9127	FBgn0000052	ade2	FBtr0079219
CG9134	FBgn0035199	CG9134	FBtr0072659
CG9135	FBgn0031769	CG9135	FBtr0079186
CG9140	FBgn0031771	CG9140	FBtr0079216
CG9143	FBgn0034496	NA	FBtr0086304
CG9144	FBgn0031773	NA	FBtr0079215
CG9148	FBgn0025682	scf	FBtr0089942
CG9149	FBgn0035203	CG9149	FBtr0072667
CG9155	FBgn0010246	Myo61F	FBtr0072672
CG9160	FBgn0011361	mtacp1	FBtr0072676
CG9165	FBgn0010786	NA	FBtr0072679
CG9177	FBgn0030719	eIF5	FBtr0074146
CG9196	FBgn0035056	NA	FBtr0072370
CG9198	FBgn0004391	shtd	FBtr0074077
CG9204	FBgn0025720	NA	FBtr0086319
CG9206	FBgn0001108	Gl	FBtr0075756
CG9207	FBgn0031873	NA	FBtr0079364
CG9209	FBgn0003969	NA	FBtr0074142
CG9210	FBgn0022710	NA	FBtr0100142
CG9211	FBgn0031872	iHog	FBtr0079365
CG9220	FBgn0030662	NA	FBtr0074064
CG9224	FBgn0003463	sog	FBtr0074063
CG9231	FBgn0036887	CG9231	FBtr0074972
CG9232	FBgn0263200	CG9232	FBtr0079322
CG9235	FBgn0034560	NA	FBtr0071517
CG9238	FBgn0036428	CG9238	FBtr0075724
CG9239	FBgn0023407	NA	FBtr0080513
CG9250	FBgn0032921	Mpp6	FBtr0081469
CG9252	FBgn0086251	NA	FBtr0081502
CG9257	FBgn0032916	NA	FBtr0081465
CG9261	FBgn0015777	nrv2	FBtr0079381
CG9263	FBgn0032530	NA	FBtr0080515

CG9264	FBgn0032911	NA	FBtr0081462
CG9267	FBgn0032524	CG9267	FBtr0080519
CG9269	FBgn0038161	NA	FBtr0082794
CG9271	FBgn0003983	NA	FBtr0080521
CG9277	FBgn0003887	betaTub56D	FBtr0086537
CG9281	FBgn0030672	NA	FBtr0074056
CG9283	FBgn0036878	NA	FBtr0074977
CG9285	FBgn0000454	NA	FBtr0082832
CG9286	FBgn0038183	NA	FBtr0082830
CG9287	FBgn0032057	NA	FBtr0079753
CG9289	FBgn0032058	NA	FBtr0079751
CG9290	FBgn0036879	NA	FBtr0074933
CG9296	FBgn0032059	CG9296	FBtr0079750
CG9297	FBgn0038181	CG9297	FBtr0082828
CG9300	FBgn0036886	CG9300	FBtr0074938
CG9302	FBgn0032514	CG9302	FBtr0080470
CG9305	FBgn0032512	NA	FBtr0080471
CG9306	FBgn0032511	CG9306	FBtr0080472
CG9307	FBgn0038180	CG9307	FBtr0082826
CG9312	FBgn0038179	NA	FBtr0082825
CG9313	FBgn0034566	NA	FBtr0071521
CG9320	FBgn0032882	CG9320	FBtr0081409
CG9324	FBgn0032884	NA	FBtr0081414
CG9326	FBgn0250785	CG9326	FBtr0089947
CG9335	FBgn0032895	CG9335	FBtr0081426
CG9346	FBgn0034572	CG9346	FBtr0071526
CG9347	FBgn0002937	ninaB	FBtr0082820
CG9354	FBgn0037686	NA	FBtr0082050
CG9355	FBgn0004511	NA	FBtr0073612
CG9356	FBgn0037688	NA	FBtr0082047
CG9358	FBgn0035089	Phk-3	FBtr0072404
CG9362	FBgn0037696	NA	FBtr0082044
CG9363	FBgn0037697	NA	FBtr0082041
CG9364	FBgn0003748	Treh	FBtr0071535
CG9377	FBgn0032507	NA	FBtr0080474
CG9378	FBgn0014023	NA	FBtr0082121
CG9384	FBgn0036446	NA	FBtr0075690
CG9388	FBgn0024833	AP-47	FBtr0082114
CG9393	FBgn0037710	CG9393	FBtr0082113
CG9394	FBgn0034588	CG9394	FBtr0071567
CG9396	FBgn0037714	NA	FBtr0082111
CG9398	FBgn0015721	king-tubby	FBtr0089600
CG9401	FBgn0002736	NA	FBtr0071569
CG9411	FBgn0030569	CG9411	FBtr0073916
CG9412	FBgn0015778	NA	FBtr0082798
CG9416	FBgn0034438	CG9416	FBtr0086555
CG9425	FBgn0036451	NA	FBtr0075693
CG9426	FBgn0032485	CG9426	FBtr0080484
CG9427	FBgn0037721	CG9427	FBtr0082104
CG9431	FBgn0032484	NA	FBtr0080485
CG9445	FBgn0040674	NA	FBtr0086210
CG9446	FBgn0033109	NA	FBtr0086207
CG9448	FBgn0037734	NA	FBtr0082091
CG9450	FBgn0003891	NA	FBtr0071582
CG9461	FBgn0037760	NA	FBtr0082155
CG9470	FBgn0002868	NA	FBtr0082158
CG9481	FBgn0026755	NA	FBtr0079242

CG9493	FBgn0031799	NA	FBtr0079284
CG9497	FBgn0031800	NA	FBtr0079283
CG9498	FBgn0031801	NA	FBtr0079282
CG9499	FBgn0031802	NA	FBtr0079281
CG9500	FBgn0031804	NA	FBtr0079280
CG9501	FBgn0031803	NA	FBtr0079252
CG9504	FBgn0030597	NA	FBtr0073975
CG9505	FBgn0031805	NA	FBtr0079253
CG9507	FBgn0031808	NA	FBtr0079255
CG9515	FBgn0032077	NA	FBtr0079791
CG9520	FBgn0032078	CG9520	FBtr0079788
CG9521	FBgn0030588	NA	FBtr0073985
CG9523	FBgn0263278	CG9523	FBtr0079257
CG9525	FBgn0032080	NA	FBtr0079786
CG9526	FBgn0031815	CG9526	FBtr0079259
CG9528	FBgn0031814	NA	FBtr0079272
CG9531	FBgn0031817	NA	FBtr0079270
CG9535	FBgn0259749	CG9535	FBtr0079268
CG9537	FBgn0031820	DLP	FBtr0079260
CG9540	FBgn0020508	NA	FBtr0073938
CG9542	FBgn0031821	NA	FBtr0079265
CG9543	FBgn0027496	NA	FBtr0079261
CG9547	FBgn0031824	NA	FBtr0079263
CG9548	FBgn0031822	CG9548	FBtr0079264
CG9550	FBgn0031826	NA	FBtr0079286
CG9552	FBgn0011705	NA	FBtr0079782
CG9559	FBgn0000719	fog	FBtr0077342
CG9570	FBgn0031085	NA	FBtr0070002
CG9571	FBgn0031086	NA	FBtr0070032
CG9576	FBgn0031091	NA	FBtr0070027
CG9582	FBgn0032090	NA	FBtr0079778
CG9589	FBgn0038361	NA	FBtr0083173
CG9590	FBgn0038360	CG9590	FBtr0083172
CG9591	FBgn0038168	NA	FBtr0082848
CG9595	FBgn0031829	NA	FBtr0079333
CG9609	FBgn0030787	NA	FBtr0074356
CG9613	FBgn0037574	NA	FBtr0081858
CG9619	FBgn0036862	NA	FBtr0075014
CG9621	FBgn0038172	NA	FBtr0082845
CG9630	FBgn0037561	CG9630	FBtr0081866
CG9632	FBgn0038377	NA	FBtr0083185
CG9634	FBgn0027528	NA	FBtr0074359
CG9638	FBgn0037555	NA	FBtr0081807
CG9642	FBgn0034183	NA	FBtr0086973
CG9646	FBgn0034184	NA	FBtr0086974
CG9655	FBgn0026630	NA	FBtr0075019
CG9659	FBgn0001404	NA	FBtr0070439
CG9663	FBgn0031516	CG9663	FBtr0077604
CG9667	FBgn0037550	CG9667	FBtr0081815
CG9668	FBgn0003250	NA	FBtr0075338
CG9686	FBgn0030158	NA	FBtr0071389
CG9689	FBgn0030159	NA	FBtr0071390
CG9691	FBgn0030160	NA	FBtr0071392
CG9699	FBgn0259923	CG9699	FBtr0074368
CG9701	FBgn0036659	CG9701	FBtr0075321
CG9707	FBgn0034628	NA	FBtr0071606
CG9716	FBgn0037601	Cyp313b1	FBtr0081893

CG9717	FBgn0039789	CG9717	FBtr0085679
CG9730	FBgn0036853	NA	FBtr0075022
CG9733	FBgn0039759	NA	FBtr0085638
CG9735	FBgn0010803	NA	FBtr0081980
CG9737	FBgn0039758	NA	FBtr0085639
CG9750	FBgn0040075	rept	FBtr0074988
CG9774	FBgn0026181	NA	FBtr0074286
CG9776	FBgn0027866	NA	FBtr0078955
CG9778	FBgn0261086	NA	FBtr0078957
CG9786	FBgn0001180	hb	FBtr0081950
CG9792	FBgn0041711	yellow-e	FBtr0082757
CG9795	FBgn0037234	CG9795	FBtr0100617
CG9802	FBgn0015615	Cap	FBtr0074291
CG9813	FBgn0038143	NA	FBtr0082765
CG9820	FBgn0026384	NA	FBtr0072023
CG9826	FBgn0034784	NA	FBtr0071895
CG9828	FBgn0032474	NA	FBtr0080493
CG9841	FBgn0034627	NA	FBtr0071608
CG9856	FBgn0016641	PTP-ER	FBtr0071686
CG9862	FBgn0034646	Rae1	FBtr0071683
CG9866	FBgn0031420	NA	FBtr0077765
CG9867	FBgn0031421	CG9867	FBtr0077767
CG9870	FBgn0031422	NA	FBtr0077768
CG9874	FBgn0003687	NA	FBtr0071679
CG9876	FBgn0034821	CG9876	FBtr0071979
CG9877	FBgn0034819	NA	FBtr0071980
CG9879	FBgn0031444	NA	FBtr0077700
CG9880	FBgn0026395	NA	FBtr0077698
CG9881	FBgn0031437	p16-ARC	FBtr0077694
CG9883	FBgn0031435	CG9883	FBtr0077693
CG9884	FBgn0011818	oaf	FBtr0077777
CG9886	FBgn0031428	NA	FBtr0077770
CG9889	FBgn0041712	NA	FBtr0072011
CG9896	FBgn0034808	NA	FBtr0071988
CG9900	FBgn0004643	mit(1)15	FBtr0100272
CG9906	FBgn0030755	NA	FBtr0074299
CG9916	FBgn0004432	NA	FBtr0074238
CG9917	FBgn0030740	NA	FBtr0074237
CG9919	FBgn0030742	NA	FBtr0074236
CG9921	FBgn0030743	CG9921	FBtr0074235
CG9922	FBgn0038196	NA	FBtr0082870
CG9925	FBgn0038191	CG9925	FBtr0082876
CG9926	FBgn0038190	NA	FBtr0082877
CG9938	FBgn0030500	NA	FBtr0073850
CG9945	FBgn0034527	CG9945	FBtr0086225
CG9947	FBgn0030752	NA	FBtr0074301
CG9949	FBgn0003410	NA	FBtr0075332
CG9952	FBgn0020257	ppa	FBtr0071924
CG9953	FBgn0035726	CG9953	FBtr0076898
CG9961	FBgn0031451	NA	FBtr0077740
CG9967	FBgn0031413	NA	FBtr0100648
CG9968	FBgn0030749	Anxb11	FBtr0074303
CG9972	FBgn0035379	CG9972	FBtr0073011
CG9977	FBgn0035371	CG9977	FBtr0073016
CG9981	FBgn0030746	NA	FBtr0074304
CG9983	FBgn0001215	Hrb98DE	FBtr0085298
CG9984	FBgn0010416	NA	FBtr0074305

CG9985	FBgn0016984	sktl	FBtr0071563
CG9986	FBgn0039589	CG9986	FBtr0085304
CG9992	FBgn0030744	NA	FBtr0074308
CG9993	FBgn0034553	CG9993	FBtr0071560
CG9995	FBgn0027655	NA	FBtr0085310
CG9999	FBgn0003346	NA	FBtr0081234
CR12628	FBgn0042701	NA	FBtr0085952
CR14753	FBgn0033303	NA	FBtr0088689
CR15280	FBgn0028867	NA	FBtr0100604
CR15821	FBgn0035311	NA	FBtr0072914
CR17875	FBgn0038034	NA	FBtr0082602
CR18775	FBgn0020646	NA	FBtr0076993
CR30003	FBgn0011921	NA	FBtr0088465
CR30024	FBgn0011987	NA	FBtr0088156
CR30138	FBgn0011870	NA	FBtr0086314
CR30155	FBgn0050155	NA	FBtr0086234
CR30198	FBgn0050198	NA	FBtr0072445
CR30199	FBgn0050199	NA	FBtr0072446
CR30200	FBgn0050200	NA	FBtr0072447
CR30201	FBgn0050201	NA	FBtr0071983
CR30202	FBgn0050202	NA	FBtr0071962
CR30206	FBgn0050206	NA	FBtr0071631
CR30207	FBgn0011871	NA	FBtr0071630
CR30208	FBgn0050208	NA	FBtr0071574
CR30209	FBgn0050209	NA	FBtr0071626
CR30210	FBgn0011872	NA	FBtr0071581
CR30211	FBgn0050211	NA	FBtr0086247
CR30212	FBgn0050212	NA	FBtr0086326
CR30214	FBgn0011869	NA	FBtr0086312
CR30215	FBgn0050215	NA	FBtr0086330
CR30218	FBgn0050218	NA	FBtr0086331
CR30220	FBgn0050220	NA	FBtr0086332
CR30223	FBgn0050223	NA	FBtr0086498
CR30224	FBgn0028981	NA	FBtr0086526
CR30225	FBgn0050225	NA	FBtr0086527
CR30227	FBgn0050227	NA	FBtr0086603
CR30228	FBgn0011868	NA	FBtr0086659
CR30229	FBgn0050229	NA	FBtr0086611
CR30231	FBgn0050231	NA	FBtr0086835
CR30232	FBgn0050232	NA	FBtr0086965
CR30233	FBgn0011879	NA	FBtr0086908
CR30234	FBgn0050234	NA	FBtr0087001
CR30235	FBgn0050235	NA	FBtr0086988
CR30236	FBgn0011867	NA	FBtr0087055
CR30237	FBgn0050237	NA	FBtr0087198
CR30238	FBgn0050238	NA	FBtr0087197
CR30239	FBgn0050239	NA	FBtr0087128
CR30240	FBgn0050240	NA	FBtr0087129
CR30241	FBgn0050241	NA	FBtr0087425
CR30244	FBgn0011882	NA	FBtr0087658
CR30246	FBgn0011884	NA	FBtr0087660
CR30247	FBgn0011885	NA	FBtr0087661
CR30249	FBgn0050249	NA	FBtr0087963
CR30250	FBgn0050250	NA	FBtr0087962
CR30251	FBgn0050251	NA	FBtr0087961
CR30252	FBgn0011878	NA	FBtr0087900
CR30253	FBgn0011877	NA	FBtr0087901

CR30254	FBgn0050254	NA	FBtr0088087
CR30255	FBgn0011915	NA	FBtr0088071
CR30257	FBgn0050257	NA	FBtr0088131
CR30260	FBgn0050260	NA	FBtr0100840
CR30292	FBgn0011906	NA	FBtr0088703
CR30297	FBgn0050297	NA	FBtr0088787
CR30298	FBgn0050298	NA	FBtr0089059
CR30299	FBgn0050299	NA	FBtr0089058
CR30300	FBgn0011892	NA	FBtr0086190
CR30301	FBgn0011893	NA	FBtr0086189
CR30302	FBgn0011894	NA	FBtr0086188
CR30303	FBgn0011895	NA	FBtr0086187
CR30304	FBgn0011958	NA	FBtr0085979
CR30312	FBgn0011887	NA	FBtr0085983
CR30313	FBgn0011880	NA	FBtr0085984
CR30314	FBgn0011956	NA	FBtr0085985
CR30316	FBgn0050316	NA	FBtr0085986
CR30319	FBgn0011934	NA	FBtr0085989
CR30326	FBgn0050326	NA	FBtr0086528
CR30333	FBgn0050333	NA	FBtr0086898
CR30367	FBgn0011812	NA	FBtr0088762
CR30406	FBgn0050406	NA	FBtr0071736
CR30407	FBgn0050407	NA	FBtr0071737
CR30449	FBgn0050449	NA	FBtr0086333
CR30451	FBgn0011850	NA	FBtr0086448
CR30452	FBgn0050452	NA	FBtr0086449
CR30453	FBgn0011848	NA	FBtr0086446
CR30454	FBgn0050454	NA	FBtr0086445
CR30455	FBgn0011849	NA	FBtr0086447
CR30505	FBgn0050505	NA	FBtr0100841
CR30506	FBgn0050506	NA	FBtr0088145
CR30507	FBgn0011896	NA	FBtr0087688
CR30508	FBgn0011910	NA	FBtr0089302
CR30509	FBgn0050509	NA	FBtr0087665
CR30510	FBgn0011931	NA	FBtr0085982
CR30511	FBgn0011932	NA	FBtr0085981
CR30512	FBgn0011957	NA	FBtr0085980
CR30513	FBgn0011938	NA	FBtr0086016
CR30514	FBgn0011955	NA	FBtr0086006
CR30515	FBgn0011890	NA	FBtr0086007
CR30516	FBgn0011888	NA	FBtr0086013
CR30517	FBgn0011889	NA	FBtr0086014
CR30518	FBgn0011933	NA	FBtr0086015
CR30519	FBgn0011935	NA	FBtr0086004
CR30520	FBgn0011897	NA	FBtr0086334
CR30521	FBgn0011883	NA	FBtr0087659
CR31023	FBgn0051023	NA	FBtr0085636
CR31070	FBgn0051070	NA	FBtr0085086
CR31071	FBgn0051071	NA	FBtr0085087
CR31130	FBgn0051130	NA	FBtr0084597
CR31143	FBgn0051143	NA	FBtr0084482
CR31162	FBgn0051162	NA	FBtr0084233
CR31165	FBgn0051165	NA	FBtr0084222
CR31166	FBgn0051166	NA	FBtr0084223
CR31167	FBgn0051167	NA	FBtr0084232
CR31185	FBgn0004187	NA	FBtr0084487
CR31197	FBgn0011905	NA	FBtr0082544

CR31214	FBgn0012003	NA	FBtr0083799
CR31215	FBgn0051215	NA	FBtr0083793
CR31228	FBgn0051228	NA	FBtr0083693
CR31242	FBgn0051242	NA	FBtr0083544
CR31271	FBgn0020546	NA	FBtr0083362
CR31273	FBgn0020556	NA	FBtr0083344
CR31282	FBgn0051282	NA	FBtr0083285
CR31310	FBgn0011919	NA	FBtr0081706
CR31331	FBgn0051331	NA	FBtr0082836
CR31333	FBgn0051333	NA	FBtr0083977
CR31334	FBgn0051334	NA	FBtr0083976
CR31341	FBgn0004185	NA	FBtr0084528
CR31356	FBgn0051356	NA	FBtr0082558
CR31379	FBgn0004188	NA	FBtr0084650
CR31382	FBgn0051382	NA	FBtr0084714
CR31383	FBgn0051383	NA	FBtr0085637
CR31387	FBgn0012028	NA	FBtr0081895
CR31396	FBgn0011970	NA	FBtr0082241
CR31416	FBgn0051416	NA	FBtr0084721
CR31430	FBgn0012004	NA	FBtr0083792
CR31432	FBgn0051432	NA	FBtr0082616
CR31433	FBgn0012025	NA	FBtr0081885
CR31434	FBgn0012026	NA	FBtr0081886
CR31435	FBgn0012024	NA	FBtr0081884
CR31443	FBgn0011966	NA	FBtr0081923
CR31471	FBgn0051471	NA	FBtr0083794
CR31476	FBgn0011971	NA	FBtr0082919
CR31480	FBgn0051480	NA	FBtr0083975
CR31485	FBgn0051485	NA	FBtr0081662
CR31486	FBgn0011904	NA	FBtr0081660
CR31487	FBgn0026163	NA	FBtr0081661
CR31489	FBgn0026164	NA	FBtr0081623
CR31490	FBgn0026162	NA	FBtr0081622
CR31491	FBgn0051491	NA	FBtr0081610
CR31494	FBgn0051494	NA	FBtr0081565
CR31497	FBgn0051497	NA	FBtr0083317
CR31500	FBgn0012000	NA	FBtr0081561
CR31502	FBgn0012001	NA	FBtr0081558
CR31506	FBgn0051506	NA	FBtr0083943
CR31512	FBgn0011999	NA	FBtr0081535
CR31518	FBgn0051518	NA	FBtr0083545
CR31539	FBgn0004190	NA	FBtr0084652
CR31540	FBgn0011845	NA	FBtr0084649
CR31552	FBgn0012027	NA	FBtr0081894
CR31566	FBgn0011960	NA	FBtr0081810
CR31567	FBgn0011944	NA	FBtr0083501
CR31568	FBgn0051568	NA	FBtr0083493
CR31569	FBgn0051569	NA	FBtr0083494
CR31570	FBgn0051570	NA	FBtr0083468
CR31571	FBgn0011945	NA	FBtr0083469
CR31572	FBgn0012002	NA	FBtr0083466
CR31573	FBgn0051573	NA	FBtr0083472
CR31574	FBgn0011840	NA	FBtr0083499
CR31575	FBgn0051575	NA	FBtr0083491
CR31576	FBgn0051576	NA	FBtr0083477
CR31577	FBgn0051577	NA	FBtr0083475
CR31578	FBgn0051578	NA	FBtr0083473

CR31579	FBgn0051579	NA	FBtr0083474
CR31580	FBgn0051580	NA	FBtr0083492
CR31581	FBgn0011959	NA	FBtr0081809
CR31582	FBgn0011961	NA	FBtr0081811
CR31583	FBgn0051583	NA	FBtr0081814
CR31584	FBgn0011963	NA	FBtr0081813
CR31585	FBgn0011962	NA	FBtr0081812
CR31586	FBgn0011858	NA	FBtr0083267
CR31587	FBgn0012011	NA	FBtr0083268
CR31588	FBgn0051588	NA	FBtr0082834
CR31589	FBgn0011993	NA	FBtr0083497
CR31590	FBgn0011994	NA	FBtr0083496
CR31591	FBgn0051591	NA	FBtr0083495
CR31592	FBgn0011998	NA	FBtr0081536
CR31593	FBgn0011965	NA	FBtr0078790
CR31602	FBgn0051602	NA	FBtr0079693
CR31603	FBgn0051603	NA	FBtr0079690
CR31604	FBgn0051604	NA	FBtr0079694
CR31614	FBgn0051614	NA	FBtr0085895
CR31615	FBgn0051615	NA	FBtr0085890
CR31616	FBgn0051616	NA	FBtr0085889
CR31621	FBgn0041336	NA	FBtr0081529
CR31625	FBgn0003930	NA	FBtr0081489
CR31631	FBgn0051631	NA	FBtr0079338
CR31667	FBgn0011859	NA	FBtr0077872
CR31669	FBgn0051669	NA	FBtr0077860
CR31696	FBgn0047133	NA	FBtr0080920
CR31734	FBgn0011985	NA	FBtr0077577
CR31754	FBgn0051754	NA	FBtr0085891
CR31831	FBgn0028889	NA	FBtr0080717
CR31838	FBgn0028909	NA	FBtr0080609
CR31850	FBgn0004191	NA	FBtr0080486
CR31853	FBgn0003935	NA	FBtr0080451
CR31854	FBgn0004192	NA	FBtr0080443
CR31877	FBgn0025881	NA	FBtr0079907
CR31885	FBgn0051885	NA	FBtr0079820
CR31888	FBgn0051888	NA	FBtr0079752
CR31889	FBgn0051889	NA	FBtr0079728
CR31890	FBgn0051890	NA	FBtr0079729
CR31892	FBgn0051892	NA	FBtr0079702
CR31895	FBgn0051895	NA	FBtr0079692
CR31896	FBgn0051896	NA	FBtr0079677
CR31899	FBgn0011902	NA	FBtr0079596
CR31903	FBgn0011861	NA	FBtr0079528
CR31905	FBgn0012018	NA	FBtr0089297
CR31914	FBgn0051914	NA	FBtr0079090
CR31927	FBgn0003926	NA	FBtr0077928
CR31939	FBgn0051939	NA	FBtr0077862
CR31940	FBgn0051940	NA	FBtr0077861
CR31942	FBgn0051942	NA	FBtr0077812
CR31943	FBgn0051943	NA	FBtr0077818
CR31944	FBgn0051944	NA	FBtr0077819
CR31945	FBgn0011860	NA	FBtr0077821
CR31946	FBgn0051946	NA	FBtr0077834
CR31951	FBgn0011984	NA	FBtr0077613
CR31963	FBgn0051963	NA	FBtr0077458
CR31971	FBgn0051971	NA	FBtr0079064

CR31977	FBgn0011862	NA	FBtr0089613
CR31978	FBgn0011863	NA	FBtr0080666
CR31979	FBgn0028911	NA	FBtr0080660
CR31980	FBgn0011866	NA	FBtr0080663
CR31981	FBgn0011865	NA	FBtr0080664
CR31982	FBgn0011864	NA	FBtr0080665
CR31983	FBgn0028912	NA	FBtr0080646
CR31984	FBgn0028910	NA	FBtr0080647
CR31985	FBgn0028882	NA	FBtr0080644
CR31986	FBgn0012017	NA	FBtr0089299
CR31987	FBgn0012016	NA	FBtr0089298
CR32034	FBgn0052034	NA	FBtr0076505
CR32093	FBgn0052093	NA	FBtr0076083
CR32122	FBgn0012009	NA	FBtr0075856
CR32123	FBgn0052123	NA	FBtr0075835
CR32124	FBgn0012010	NA	FBtr0075847
CR32125	FBgn0011844	NA	FBtr0075834
CR32126	FBgn0052126	NA	FBtr0075830
CR32127	FBgn0052127	NA	FBtr0075831
CR32128	FBgn0052128	NA	FBtr0075829
CR32129	FBgn0052129	NA	FBtr0075828
CR32142	FBgn0011927	NA	FBtr0075713
CR32144	FBgn0011928	NA	FBtr0075681
CR32153	FBgn0052153	NA	FBtr0075456
CR32162	FBgn0041721	NA	FBtr0075315
CR32173	FBgn0052173	NA	FBtr0075244
CR32200	FBgn0052200	NA	FBtr0075107
CR32272	FBgn0052272	NA	FBtr0073125
CR32273	FBgn0052273	NA	FBtr0073121
CR32285	FBgn0052285	NA	FBtr0073021
CR32286	FBgn0052286	NA	FBtr0073022
CR32287	FBgn0052287	NA	FBtr0073020
CR32288	FBgn0052288	NA	FBtr0073018
CR32289	FBgn0052289	NA	FBtr0073019
CR32303	FBgn0052303	NA	FBtr0072882
CR32312	FBgn0052312	NA	FBtr0072840
CR32322	FBgn0011855	NA	FBtr0072691
CR32323	FBgn0011854	NA	FBtr0072690
CR32324	FBgn0052324	NA	FBtr0072688
CR32325	FBgn0011853	NA	FBtr0072687
CR32326	FBgn0011852	NA	FBtr0072686
CR32327	FBgn0011851	NA	FBtr0072685
CR32328	FBgn0052328	NA	FBtr0072683
CR32329	FBgn0052329	NA	FBtr0072684
CR32330	FBgn0052330	NA	FBtr0072682
CR32357	FBgn0052357	NA	FBtr0075380
CR32358	FBgn0052358	NA	FBtr0076744
CR32359	FBgn0052359	NA	FBtr0076743
CR32360	FBgn0052360	NA	FBtr0076742
CR32361	FBgn0052361	NA	FBtr0076741
CR32362	FBgn0052362	NA	FBtr0076740
CR32363	FBgn0052363	NA	FBtr0076739
CR32370	FBgn0052370	NA	FBtr0076762
CR32419	FBgn0011986	NA	FBtr0077142
CR32420	FBgn0052420	NA	FBtr0077143
CR32421	FBgn0052421	NA	FBtr0077158
CR32449	FBgn0052449	NA	FBtr0078490

CR32456	FBgn0052456	NA	FBtr0078547
CR32460	FBgn0052460	NA	FBtr0078580
CR32480	FBgn0052480	NA	FBtr0072579
CR32481	FBgn0052481	NA	FBtr0072580
CR32482	FBgn0052482	NA	FBtr0072600
CR32493	FBgn0052493	NA	FBtr0070533
CR32518	FBgn0052518	NA	FBtr0077238
CR32519	FBgn0025882	NA	FBtr0077222
CR32520	FBgn0052520	NA	FBtr0089301
CR32525	FBgn0052525	NA	FBtr0089300
CR32526	FBgn0011954	NA	FBtr0077184
CR32546	FBgn0052546	NA	FBtr0074600
CR32607	FBgn0011982	NA	FBtr0073885
CR32608	FBgn0011977	NA	FBtr0073872
CR32609	FBgn0011983	NA	FBtr0073870
CR32610	FBgn0011953	NA	FBtr0073871
CR32612	FBgn0011980	NA	FBtr0073886
CR32615	FBgn0011974	NA	FBtr0073865
CR32617	FBgn0011951	NA	FBtr0073863
CR32618	FBgn0011981	NA	FBtr0073866
CR32619	FBgn0011950	NA	FBtr0073862
CR32620	FBgn0011949	NA	FBtr0073861
CR32621	FBgn0011975	NA	FBtr0073858
CR32622	FBgn0011948	NA	FBtr0073860
CR32623	FBgn0011976	NA	FBtr0073859
CR32624	FBgn0011952	NA	FBtr0073857
CR32660	FBgn0052660	NA	FBtr0073592
CR32661	FBgn0030356	NA	FBtr0073593
CR32740	FBgn0052740	NA	FBtr0070979
CR32748	FBgn0052748	NA	FBtr0070911
CR32761	FBgn0052761	NA	FBtr0070818
CR32777	FBgn0019661	NA	FBtr0070634
CR32784	FBgn0025118	NA	FBtr0070604
CR32785	FBgn0052785	NA	FBtr0070603
CR32807	FBgn0044508	NA	FBtr0070292
CR32826	FBgn0052826	NA	FBtr0070001
CR32837	FBgn0011891	NA	FBtr0086017
CR32841	FBgn0011909	NA	FBtr0089303
CR32842	FBgn0011881	NA	FBtr0087713
CR32844	FBgn0011916	NA	FBtr0088086
CR32863	FBgn0043853	NA	FBtr0081560
CR32864	FBgn0000003	NA	FBtr0081624
CR32865	FBgn0052865	NA	FBtr0082678
CR32866	FBgn0004186	NA	FBtr0084488
CR32867	FBgn0004189	NA	FBtr0084651
CR32868	FBgn0046696	NA	FBtr0085775
CR32873	FBgn0063449	NA	FBtr0079906
CR32874	FBgn0063389	NA	FBtr0079908
CR32875	FBgn0063392	NA	FBtr0079909
CR32876	FBgn0063391	NA	FBtr0079910
CR32877	FBgn0003936	NA	FBtr0080770
CR32878	FBgn0003923	NA	FBtr0081293
CR32879	FBgn0003931	NA	FBtr0081292
CR32880	FBgn0043021	NA	FBtr0081294
CR32881	FBgn0043022	NA	FBtr0081315
CR32882	FBgn0003922	NA	FBtr0081313
CR32884	FBgn0026169	NA	FBtr0072259

CR32886	FBgn0045800	NA	FBtr0086842
CR32887	FBgn0063381	NA	FBtr0086843
CR32888	FBgn0063385	NA	FBtr0086844
CR32889	FBgn0063377	NA	FBtr0086845
CR32890	FBgn0063384	NA	FBtr0086846
CR32891	FBgn0063376	NA	FBtr0086848
CR32892	FBgn0063388	NA	FBtr0086850
CR32893	FBgn0063375	NA	FBtr0086851
CR32894	FBgn0063383	NA	FBtr0086847
CR32895	FBgn0063382	NA	FBtr0086849
CR32896	FBgn0063374	NA	FBtr0086852
CR32897	FBgn0063380	NA	FBtr0086853
CR32898	FBgn0063379	NA	FBtr0086854
CR32899	FBgn0063378	NA	FBtr0086855
CR32900	FBgn0063387	NA	FBtr0086856
CR32901	FBgn0063373	NA	FBtr0086857
CR32902	FBgn0063386	NA	FBtr0086858
CR32905	FBgn0015543	NA	FBtr0088037
CR32907	FBgn0020518	NA	FBtr0078576
CR32908	FBgn0003938	NA	FBtr0073017
CR32910	FBgn0060291	NA	FBtr0076635
CR32911	FBgn0060292	NA	FBtr0076634
CR32913	FBgn0003920	NA	FBtr0074208
CR32914	FBgn0003933	NA	FBtr0074249
CR32926	FBgn0000280	NA	FBtr0085613
CR32934	FBgn0000281	NA	FBtr0089614
CR32989	FBgn0041717	NA	FBtr0079659
CR32998	FBgn0041719	NA	FBtr0079108
CR32999	FBgn0003934	NA	FBtr0077658
CR33315	FBgn0053315	NA	FBtr0081172
CR33318	FBgn0053318	NA	FBtr0089857
CR33319	FBgn0053319	NA	FBtr0089858
CR33345	FBgn0067407	NA	FBtr0085225
CR33353	FBgn0053353	NA	FBtr0086345
CR33354	FBgn0053354	NA	FBtr0086346
CR33355	FBgn0053355	NA	FBtr0086347
CR33356	FBgn0053356	NA	FBtr0086348
CR33357	FBgn0053357	NA	FBtr0086349
CR33358	FBgn0053358	NA	FBtr0086350
CR33359	FBgn0053359	NA	FBtr0086351
CR33360	FBgn0053360	NA	FBtr0086352
CR33361	FBgn0053361	NA	FBtr0086353
CR33362	FBgn0053362	NA	FBtr0086354
CR33363	FBgn0053363	NA	FBtr0086355
CR33364	FBgn0053364	NA	FBtr0086356
CR33365	FBgn0053365	NA	FBtr0086357
CR33366	FBgn0053366	NA	FBtr0086358
CR33367	FBgn0053367	NA	FBtr0086359
CR33368	FBgn0053368	NA	FBtr0086360
CR33369	FBgn0053369	NA	FBtr0086361
CR33370	FBgn0053370	NA	FBtr0086362
CR33371	FBgn0053371	NA	FBtr0086363
CR33372	FBgn0053372	NA	FBtr0086364
CR33373	FBgn0053373	NA	FBtr0086365
CR33374	FBgn0053374	NA	FBtr0086366
CR33375	FBgn0053375	NA	FBtr0086367
CR33376	FBgn0053376	NA	FBtr0086368

CR33377	FBgn0053377	NA	FBtr0086369
CR33378	FBgn0053378	NA	FBtr0086370
CR33379	FBgn0053379	NA	FBtr0086371
CR33380	FBgn0053380	NA	FBtr0086372
CR33381	FBgn0053381	NA	FBtr0086373
CR33382	FBgn0053382	NA	FBtr0086374
CR33383	FBgn0053383	NA	FBtr0086375
CR33384	FBgn0053384	NA	FBtr0086376
CR33385	FBgn0053385	NA	FBtr0086377
CR33386	FBgn0053386	NA	FBtr0086378
CR33387	FBgn0053387	NA	FBtr0086379
CR33388	FBgn0053388	NA	FBtr0086380
CR33389	FBgn0053389	NA	FBtr0086381
CR33390	FBgn0053390	NA	FBtr0086382
CR33391	FBgn0053391	NA	FBtr0086383
CR33392	FBgn0053392	NA	FBtr0086384
CR33393	FBgn0053393	NA	FBtr0086385
CR33394	FBgn0053394	NA	FBtr0086386
CR33395	FBgn0053395	NA	FBtr0086387
CR33396	FBgn0053396	NA	FBtr0086388
CR33397	FBgn0053397	NA	FBtr0086389
CR33398	FBgn0053398	NA	FBtr0086390
CR33399	FBgn0053399	NA	FBtr0086391
CR33400	FBgn0053400	NA	FBtr0086392
CR33401	FBgn0053401	NA	FBtr0086393
CR33402	FBgn0053402	NA	FBtr0086394
CR33403	FBgn0053403	NA	FBtr0086395
CR33404	FBgn0053404	NA	FBtr0086396
CR33405	FBgn0053405	NA	FBtr0086397
CR33406	FBgn0053406	NA	FBtr0086398
CR33407	FBgn0053407	NA	FBtr0086399
CR33408	FBgn0053408	NA	FBtr0086400
CR33409	FBgn0053409	NA	FBtr0086401
CR33410	FBgn0053410	NA	FBtr0086402
CR33411	FBgn0053411	NA	FBtr0086403
CR33412	FBgn0053412	NA	FBtr0086404
CR33413	FBgn0053413	NA	FBtr0086405
CR33414	FBgn0053414	NA	FBtr0086406
CR33415	FBgn0053415	NA	FBtr0086407
CR33416	FBgn0053416	NA	FBtr0086408
CR33417	FBgn0053417	NA	FBtr0086409
CR33418	FBgn0053418	NA	FBtr0086410
CR33419	FBgn0053419	NA	FBtr0086411
CR33420	FBgn0053420	NA	FBtr0086412
CR33421	FBgn0053421	NA	FBtr0086413
CR33422	FBgn0053422	NA	FBtr0086414
CR33423	FBgn0053423	NA	FBtr0086415
CR33424	FBgn0053424	NA	FBtr0086416
CR33425	FBgn0053425	NA	FBtr0086417
CR33426	FBgn0053426	NA	FBtr0086418
CR33427	FBgn0053427	NA	FBtr0086419
CR33428	FBgn0053428	NA	FBtr0086420
CR33429	FBgn0053429	NA	FBtr0086421
CR33430	FBgn0053430	NA	FBtr0086422
CR33431	FBgn0053431	NA	FBtr0086423
CR33432	FBgn0053432	NA	FBtr0086424
CR33433	FBgn0053433	NA	FBtr0086425

CR33434	FBgn0053434	NA	FBtr0086426
CR33435	FBgn0053435	NA	FBtr0086427
CR33436	FBgn0053436	NA	FBtr0086428
CR33437	FBgn0053437	NA	FBtr0086429
CR33438	FBgn0053438	NA	FBtr0086430
CR33439	FBgn0053439	NA	FBtr0086431
CR33440	FBgn0053440	NA	FBtr0086432
CR33441	FBgn0053441	NA	FBtr0086433
CR33442	FBgn0053442	NA	FBtr0086434
CR33443	FBgn0053443	NA	FBtr0086435
CR33444	FBgn0053444	NA	FBtr0086436
CR33445	FBgn0053445	NA	FBtr0086437
CR33446	FBgn0053446	NA	FBtr0086438
CR33447	FBgn0053447	NA	FBtr0086439
CR33448	FBgn0053448	NA	FBtr0086440
CR33449	FBgn0053449	NA	FBtr0086441
CR33450	FBgn0053450	NA	FBtr0086442
CR33451	FBgn0053451	NA	FBtr0086443
CR33452	FBgn0053452	NA	FBtr0086444
CR33471	FBgn0053471	NA	FBtr0088081
CR33504	FBgn0053504	NA	FBtr0100848
CR33534	FBgn0053534	NA	FBtr0100842
CR33535	FBgn0053535	NA	FBtr0100843
CR33536	FBgn0053536	NA	FBtr0100845
CR33537	FBgn0053537	NA	FBtr0100849
CR33538	FBgn0053538	NA	FBtr0100846
CR33539	FBgn0053539	NA	FBtr0100847
CR33551	FBgn0053551	NA	FBtr0091521
CR33625	FBgn0086663	NA	FBtr0091602
CR33628	FBgn0065047	NA	FBtr0091605
CR33629	FBgn0053629	NA	FBtr0091606
CR33633	FBgn0086600	NA	FBtr0091610
CR33634	FBgn0065080	NA	FBtr0091611
CR33636	FBgn0086603	NA	FBtr0091613
CR33637	FBgn0086672	NA	FBtr0091614
CR33638	FBgn0065070	NA	FBtr0091615
CR33646	FBgn0086671	NA	FBtr0091623
CR33652	FBgn0086660	NA	FBtr0091629
CR33656	FBgn0086670	NA	FBtr0091635
CR33657	FBgn0065101	NA	FBtr0091636
CR33659	FBgn0065097	NA	FBtr0091638
CR33660	FBgn0065092	NA	FBtr0091639
CR33661	FBgn0086658	NA	FBtr0091640
CR33662	FBgn0065046	NA	FBtr0091641
CR33663	FBgn0065071	NA	FBtr0091642
CR33674	FBgn0065053	NA	FBtr0091653
CR33683	FBgn0065088	NA	FBtr0091663
CR33684	FBgn0065073	NA	FBtr0091664
CR33685	FBgn0065087	NA	FBtr0091665
CR33686	FBgn0086661	NA	FBtr0091666
CR33693	FBgn0065055	NA	FBtr0091677
CR33699	FBgn0065095	NA	FBtr0091687
CR33701	FBgn0053701	NA	FBtr0091689
CR33705	FBgn0065100	NA	FBtr0091693
CR33707	FBgn0065105	NA	FBtr0091695
CR33709	FBgn0086662	NA	FBtr0091697
CR33711	FBgn0065084	NA	FBtr0091699

CR33712	FBgn0065096	NA	FBtr0091700
CR33716	FBgn0065051	NA	FBtr0091708
CR33726	FBgn0065057	NA	FBtr0091724
CR33727	FBgn0053727	NA	FBtr0091725
CR33728	FBgn0053728	NA	FBtr0091726
CR33729	FBgn0053729	NA	FBtr0091727
CR33730	FBgn0053730	NA	FBtr0091728
CR33731	FBgn0053731	NA	FBtr0091729
CR33732	FBgn0053732	NA	FBtr0091730
CR33733	FBgn0053733	NA	FBtr0091731
CR33734	FBgn0053734	NA	FBtr0091732
CR33735	FBgn0053735	NA	FBtr0091733
CR33736	FBgn0053736	NA	FBtr0091734
CR33737	FBgn0053737	NA	FBtr0091735
CR33738	FBgn0053738	NA	FBtr0091736
CR33739	FBgn0053739	NA	FBtr0091737
CR33740	FBgn0053740	NA	FBtr0091738
CR33741	FBgn0053741	NA	FBtr0091739
CR33742	FBgn0053742	NA	FBtr0091740
CR33743	FBgn0053743	NA	FBtr0091741
CR33744	FBgn0053744	NA	FBtr0091742
CR33745	FBgn0053745	NA	FBtr0091743
CR33746	FBgn0053746	NA	FBtr0091744
CR33749	FBgn0065104	NA	FBtr0091750
CR33750	FBgn0086665	NA	FBtr0091751
CR33751	FBgn0086668	NA	FBtr0091752
CR33753	FBgn0086601	NA	FBtr0091754
CR33754	FBgn0065064	NA	FBtr0091755
CR33765	FBgn0086669	NA	FBtr0091766
CR33776	FBgn0086664	NA	FBtr0091777
CR33778	FBgn0065066	NA	FBtr0091779
CR33780	FBgn0065059	NA	FBtr0091781
CR33787	FBgn0086667	NA	FBtr0091788
CR33788	FBgn0041720	NA	FBtr0091789
CR33789	FBgn0065083	NA	FBtr0091790
CR33793	FBgn0065081	NA	FBtr0091797
CR33794	FBgn0065058	NA	FBtr0091798
CR33799	FBgn0086602	NA	FBtr0091803
CR33802	FBgn0053802	NA	FBtr0091806
CR33805	FBgn0053805	NA	FBtr0091809
CR33811	FBgn0053811	NA	FBtr0091815
CR33867	FBgn0053867	NA	FBtr0091871
CR33913	FBgn0086659	NA	FBtr0091917
CR33915	FBgn0065107	NA	FBtr0091919
CR33916	FBgn0065102	NA	FBtr0091920
CR33917	FBgn0065094	NA	FBtr0091921
CR33918	FBgn0086666	NA	FBtr0091922
CR33921	FBgn0065048	NA	FBtr0091925
CR33930	FBgn0065076	NA	FBtr0091934
CR33938	FBgn0063127	NA	FBtr0091948
CR33939	FBgn0047095	NA	FBtr0091949
CR33940	FBgn0062928	NA	FBtr0091951
CR33942	FBgn0062961	NA	FBtr0091953
CR33946	FBgn0047000	NA	FBtr0091957
CR33947	FBgn0083068	NA	FBtr0091958
CR33948	FBgn0063083	NA	FBtr0091959
CR33972	FBgn0011937	NA	FBtr0100015

CR33973	FBgn0011936	NA	FBtr0100016
CR33987	FBgn0053987	NA	FBtr0100032
CR40450	FBgn0085734	NA	FBtr0114183
CR40454	FBgn0250731	NA	FBtr0114184
CR40465	FBgn0250817	NA	FBtr0100850
CR40502	FBgn0085737	NA	FBtr0114187
CR40503	FBgn0085738	NA	FBtr0114188
CR40507	FBgn0085739	NA	FBtr0114189
CR40508	FBgn0085740	NA	FBtr0114190
CR40528	FBgn0085741	NA	FBtr0114191
CR40546	FBgn0085742	NA	FBtr0114192
CR40560	FBgn0085743	NA	FBtr0114193
CR40561	FBgn0085744	NA	FBtr0114194
CR40565	FBgn0085745	NA	FBtr0114195
CR40571	FBgn0085746	NA	FBtr0114196
CR40572	FBgn0085747	NA	FBtr0114197
CR40573	FBgn0085748	NA	FBtr0114198
CR40574	FBgn0085749	NA	FBtr0114199
CR40581	FBgn0085750	NA	FBtr0114200
CR40582	FBgn0085751	NA	FBtr0114201
CR40594	FBgn0085752	NA	FBtr0114202
CR40596	FBgn0085753	NA	FBtr0114203
CR40597	FBgn0085754	NA	FBtr0114204
CR40611	FBgn0085755	NA	FBtr0114205
CR40613	FBgn0085756	NA	FBtr0114206
CR40621	FBgn0085757	NA	FBtr0114207
CR40639	FBgn0085758	NA	FBtr0114208
CR40640	FBgn0085759	NA	FBtr0114209
CR40641	FBgn0085760	NA	FBtr0114210
CR40642	FBgn0085761	NA	FBtr0114211
CR40668	FBgn0085764	NA	FBtr0114214
CR40677	FBgn0085765	NA	FBtr0114215
CR40679	FBgn0085766	NA	FBtr0114216
CR40685	FBgn0085767	NA	FBtr0114217
CR40712	FBgn0085768	NA	FBtr0114218
CR40728	FBgn0085769	NA	FBtr0114219
CR40734	FBgn0085770	NA	FBtr0114220
CR40741	FBgn0085771	NA	FBtr0114221
CR40743	FBgn0085772	NA	FBtr0114222
CR40766	FBgn0085773	NA	FBtr0114223
CR40779	FBgn0085774	NA	FBtr0114224
CR40959	FBgn0085777	NA	FBtr0114228
CR40962	FBgn0085778	NA	FBtr0114229
CR40963	FBgn0085779	NA	FBtr0114230
CR40976	FBgn0085780	NA	FBtr0114231
CR41510	FBgn0085793	NA	FBtr0114246
CR41535	FBgn0085795	NA	FBtr0114249
CR41539	FBgn0085796	NA	FBtr0114250
CR41540	FBgn0085797	NA	FBtr0114251
CR41544	FBgn0085799	NA	FBtr0114253
CR41548	FBgn0085802	NA	FBtr0114256
CR41550	FBgn0085803	NA	FBtr0114257
CR41571	FBgn0085804	NA	FBtr0114258
CR41583	FBgn0085805	NA	FBtr0114259
CR41590	FBgn0085807	NA	FBtr0114261
CR41591	FBgn0085808	NA	FBtr0114262
CR41601	FBgn0085812	NA	FBtr0114267

CR41602	FBgn0085813	NA	FBtr0114268
CR41604	FBgn0085814	NA	FBtr0114271
CR41605	FBgn0085815	NA	FBtr0114273
CR41606	FBgn0085816	NA	FBtr0114274
CR41607	FBgn0085817	NA	FBtr0114275
CR41608	FBgn0085818	NA	FBtr0114276
CR41609	FBgn0085819	NA	FBtr0114277
CR41613	FBgn0085822	NA	FBtr0114280
CR41617	FBgn0085823	NA	FBtr0114281
CR41618	FBgn0085824	NA	FBtr0114282
CR41619	FBgn0085825	NA	FBtr0114283
CR41620	FBgn0085826	NA	FBtr0114284
CR41621	FBgn0085827	NA	FBtr0114285
CR42195	FBgn0085828	NA	FBtr0114286
CR7249	FBgn0031726	NA	FBtr0100853
CR9337	FBgn0032898	NA	FBtr0081448
FBgn0000008	FBgn0000008	a	FBtr0071764
FBgn0000014	FBgn0000014	abd-A	FBtr0306337
FBgn0000017	FBgn0000017	NA	FBtr0112790
FBgn0000018	FBgn0000018	NA	FBtr0080168
FBgn0000022	FBgn0000022	ac	FBtr0070072
FBgn0000024	FBgn0000024	Ace	FBtr0082780
FBgn0000028	FBgn0000028	NA	FBtr0100328
FBgn0000044	FBgn0000044	Act57B	FBtr0300946
FBgn0000046	FBgn0000046	Act87E	FBtr0082786
FBgn0000057	FBgn0000057	adp	FBtr0086744
FBgn0000063	FBgn0000063	NA	FBtr0083478
FBgn0000064	FBgn0000064	NA	FBtr0084994
FBgn0000071	FBgn0000071	Ama	FBtr0081618
FBgn0000077	FBgn0000077	NA	FBtr0071331
FBgn0000084	FBgn0000084	AnnX	FBtr0070025
FBgn0000099	FBgn0000099	ap	FBtr0086058
FBgn0000100	FBgn0000100	NA	FBtr0078481
FBgn0000108	FBgn0000108	Appl	FBtr0307294
FBgn0000109	FBgn0000109	NA	FBtr0089420
FBgn0000114	FBgn0000114	NA	FBtr0080344
FBgn0000115	FBgn0000115	NA	FBtr0075517
FBgn0000116	FBgn0000116	Argk	FBtr0076543
FBgn0000119	FBgn0000119	arr	FBtr0087702
FBgn0000120	FBgn0000120	Arr1	FBtr0307350
FBgn0000137	FBgn0000137	ase	FBtr0070075
FBgn0000139	FBgn0000139	NA	FBtr0084660
FBgn0000146	FBgn0000146	NA	FBtr0080165
FBgn0000150	FBgn0000150	awd	FBtr0085864
FBgn0000152	FBgn0000152	NA	FBtr0100417
FBgn0000153	FBgn0000153	b	FBtr0309960
FBgn0000157	FBgn0000157	NA	FBtr0072379
FBgn0000163	FBgn0000163	NA	FBtr0074388
FBgn0000166	FBgn0000166	bcd	FBtr0081666
FBgn0000171	FBgn0000171	NA	FBtr0081888
FBgn0000173	FBgn0000173	NA	FBtr0307296
FBgn0000179	FBgn0000179	NA	FBtr0308915
FBgn0000181	FBgn0000181	bic	FBtr0087782
FBgn0000182	FBgn0000182	NA	FBtr0080803
FBgn0000183	FBgn0000183	BicD	FBtr0081002
FBgn0000210	FBgn0000210	br	FBtr0303562
FBgn0000216	FBgn0000216	NA	FBtr0075621

FBgn0000221	FBgn0000221	NA	FBtr0070638
FBgn0000228	FBgn0000228	NA	FBtr0079083
FBgn0000229	FBgn0000229	bsk	FBtr0302378
FBgn0000239	FBgn0000239	NA	FBtr0301342
FBgn0000241	FBgn0000241	bw	FBtr0072117
FBgn0000242	FBgn0000242	Bx	FBtr0074597
FBgn0000244	FBgn0000244	NA	FBtr0301093
FBgn0000246	FBgn0000246	NA	FBtr0083194
FBgn0000251	FBgn0000251	cad	FBtr0081412
FBgn0000253	FBgn0000253	Cam	FBtr0304963
FBgn0000256	FBgn0000256	capu	FBtr0290078
FBgn0000257	FBgn0000257	NA	FBtr0074727
FBgn0000259	FBgn0000259	NA	FBtr0073558
FBgn0000273	FBgn0000273	Pka-C1	FBtr0079852
FBgn0000274	FBgn0000274	NA	FBtr0085727
FBgn0000286	FBgn0000286	NA	FBtr0304887
FBgn0000287	FBgn0000287	salr	FBtr0307093
FBgn0000289	FBgn0000289	NA	FBtr0290035
FBgn0000307	FBgn0000307	chif	FBtr0080838
FBgn0000308	FBgn0000308	NA	FBtr0079236
FBgn0000316	FBgn0000316	cin	FBtr0070064
FBgn0000317	FBgn0000317	NA	FBtr0080723
FBgn0000318	FBgn0000318	cl	FBtr0079095
FBgn0000319	FBgn0000319	NA	FBtr0074182
FBgn0000320	FBgn0000320	eya	FBtr0079334
FBgn0000326	FBgn0000326	NA	FBtr0071687
FBgn0000330	FBgn0000330	NA	FBtr0071033
FBgn0000346	FBgn0000346	comt	FBtr0073754
FBgn0000355	FBgn0000355	NA	FBtr0076572
FBgn0000356	FBgn0000356	NA	FBtr0076574
FBgn0000357	FBgn0000357	NA	FBtr0076571
FBgn0000358	FBgn0000358	NA	FBtr0076573
FBgn0000360	FBgn0000360	NA	FBtr0300448
FBgn0000363	FBgn0000363	cpo	FBtr0308290
FBgn0000377	FBgn0000377	NA	FBtr0070418
FBgn0000392	FBgn0000392	cup	FBtr0290322
FBgn0000394	FBgn0000394	cv	FBtr0070804
FBgn0000395	FBgn0000395	NA	FBtr0071610
FBgn0000404	FBgn0000404	CycA	FBtr0304650
FBgn0000405	FBgn0000405	NA	FBtr0071911
FBgn0000406	FBgn0000406	Cyt-b5-r	FBtr0080932
FBgn0000412	FBgn0000412	NA	FBtr0081987
FBgn0000414	FBgn0000414	Dab	FBtr0305085
FBgn0000422	FBgn0000422	Ddc	FBtr0290291
FBgn0000426	FBgn0000426	NA	FBtr0088010
FBgn0000427	FBgn0000427	NA	FBtr0071145
FBgn0000447	FBgn0000447	NA	FBtr0300442
FBgn0000448	FBgn0000448	Hr46	FBtr0306345
FBgn0000451	FBgn0000451	ect	FBtr0301385
FBgn0000455	FBgn0000455	Dip-C	FBtr0082597
FBgn0000459	FBgn0000459	disco	FBtr0074250
FBgn0000463	FBgn0000463	Dl	FBtr0083739
FBgn0000464	FBgn0000464	NA	FBtr0305949
FBgn0000473	FBgn0000473	NA	FBtr0086133
FBgn0000477	FBgn0000477	DNaseII	FBtr0083538
FBgn0000479	FBgn0000479	dnc	FBtr0070509
FBgn0000482	FBgn0000482	NA	FBtr0070269

FBgn0000490	FBgn0000490	dpp	FBtr0077773
FBgn0000499	FBgn0000499	NA	FBtr0073454
FBgn0000500	FBgn0000500	Dsk	FBtr0078989
FBgn0000527	FBgn0000527	e	FBtr0084106
FBgn0000535	FBgn0000535	NA	FBtr0111009
FBgn0000536	FBgn0000536	NA	FBtr0308617
FBgn0000541	FBgn0000541	E(bx)	FBtr0072521
FBgn0000542	FBgn0000542	NA	FBtr0112801
FBgn0000543	FBgn0000543	ecd	FBtr0072903
FBgn0000546	FBgn0000546	NA	FBtr0302439
FBgn0000556	FBgn0000556	NA	FBtr0088035
FBgn0000562	FBgn0000562	egl	FBtr0114586
FBgn0000565	FBgn0000565	Eip71CD	FBtr0075552
FBgn0000566	FBgn0000566	Eip55E	FBtr0086710
FBgn0000567	FBgn0000567	NA	FBtr0075202
FBgn0000568	FBgn0000568	Eip75B	FBtr0306814
FBgn0000578	FBgn0000578	NA	FBtr0114623
FBgn0000579	FBgn0000579	NA	FBtr0302115
FBgn0000588	FBgn0000588	NA	FBtr0080325
FBgn0000591	FBgn0000591	E(spl)	FBtr0084961
FBgn0000592	FBgn0000592	Est-6	FBtr0076003
FBgn0000594	FBgn0000594	NA	FBtr0076004
FBgn0000615	FBgn0000615	NA	FBtr0302285
FBgn0000617	FBgn0000617	e(y)1	FBtr0074463
FBgn0000618	FBgn0000618	NA	FBtr0073540
FBgn0000629	FBgn0000629	E(z)	FBtr0076279
FBgn0000633	FBgn0000633	fas	FBtr0087625
FBgn0000634	FBgn0000634	NA	FBtr0300037
FBgn0000636	FBgn0000636	Fas3	FBtr0081051
FBgn0000640	FBgn0000640	NA	FBtr0079808
FBgn0000644	FBgn0000644	NA	FBtr0070542
FBgn0000658	FBgn0000658	NA	FBtr0086754
FBgn0000659	FBgn0000659	fkh	FBtr0300259
FBgn0000662	FBgn0000662	fl(2)d	FBtr0087647
FBgn0000667	FBgn0000667	Actn	FBtr0310657
FBgn0000715	FBgn0000715	Fmrf	FBtr0088383
FBgn0000721	FBgn0000721	for	FBtr0089304
FBgn0000808	FBgn0000808	NA	FBtr0309804
FBgn0000964	FBgn0000964	NA	FBtr0301669
FBgn0000986	FBgn0000986	NA	FBtr0305586
FBgn0000996	FBgn0000996	NA	FBtr0087387
FBgn0001078	FBgn0001078	ftz-f1	FBtr0304627
FBgn0001083	FBgn0001083	NA	FBtr0073580
FBgn0001087	FBgn0001087	g	FBtr0308643
FBgn0001089	FBgn0001089	NA	FBtr0079230
FBgn0001090	FBgn0001090	NA	FBtr0074656
FBgn0001092	FBgn0001092	NA	FBtr0074112
FBgn0001098	FBgn0001098	Gdh	FBtr0089498
FBgn0001099	FBgn0001099	gdl	FBtr0091757
FBgn0001104	FBgn0001104	NA	FBtr0076934
FBgn0001112	FBgn0001112	Gld	FBtr0081596
FBgn0001114	FBgn0001114	Glt	FBtr0079726
FBgn0001123	FBgn0001123	G-salpha60A	FBtr0072143
FBgn0001125	FBgn0001125	Got2	FBtr0100359
FBgn0001128	FBgn0001128	NA	FBtr0079147
FBgn0001133	FBgn0001133	NA	FBtr0071548
FBgn0001137	FBgn0001137	NA	FBtr0079708

FBgn0001138	FBgn0001138	NA	FBtr0300040
FBgn0001139	FBgn0001139	NA	FBtr0305047
FBgn0001142	FBgn0001142	Gs1	FBtr0078115
FBgn0001148	FBgn0001148	gsb	FBtr0072444
FBgn0001149	FBgn0001149	GstD1	FBtr0100410
FBgn0001174	FBgn0001174	halo	FBtr0077918
FBgn0001179	FBgn0001179	NA	FBtr0076253
FBgn0001185	FBgn0001185	NA	FBtr0080883
FBgn0001186	FBgn0001186	Hex-A	FBtr0071361
FBgn0001187	FBgn0001187	NA	FBtr0087399
FBgn0001189	FBgn0001189	hfw	FBtr0070270
FBgn0001205	FBgn0001205	NA	FBtr0084450
FBgn0001217	FBgn0001217	NA	FBtr0082707
FBgn0001219	FBgn0001219	NA	FBtr0083057
FBgn0001222	FBgn0001222	Hsf	FBtr0110966
FBgn0001223	FBgn0001223	NA	FBtr0100558
FBgn0001224	FBgn0001224	NA	FBtr0309504
FBgn0001226	FBgn0001226	Hsp27	FBtr0076454
FBgn0001227	FBgn0001227	NA	FBtr0076495
FBgn0001228	FBgn0001228	NA	FBtr0100655
FBgn0001229	FBgn0001229	NA	FBtr0076497
FBgn0001233	FBgn0001233	Hsp83	FBtr0073040
FBgn0001234	FBgn0001234	NA	FBtr0084058
FBgn0001235	FBgn0001235	NA	FBtr0082255
FBgn0001247	FBgn0001247	NA	FBtr0273273
FBgn0001248	FBgn0001248	Idh	FBtr0076669
FBgn0001250	FBgn0001250	if	FBtr0301354
FBgn0001254	FBgn0001254	ImpE2	FBtr0073141
FBgn0001255	FBgn0001255	NA	FBtr0081819
FBgn0001256	FBgn0001256	ImpL1	FBtr0075843
FBgn0001258	FBgn0001258	ImpL3	FBtr0077008
FBgn0001259	FBgn0001259	in	FBtr0078183
FBgn0001263	FBgn0001263	inaD	FBtr0305132
FBgn0001276	FBgn0001276	NA	FBtr0088154
FBgn0001280	FBgn0001280	NA	FBtr0085595
FBgn0001291	FBgn0001291	NA	FBtr0088410
FBgn0001296	FBgn0001296	kar	FBtr0302563
FBgn0001297	FBgn0001297	kay	FBtr0290076
FBgn0001308	FBgn0001308	NA	FBtr0087184
FBgn0001311	FBgn0001311	kkv	FBtr0078800
FBgn0001313	FBgn0001313	NA	FBtr0300022
FBgn0001316	FBgn0001316	NA	FBtr0072565
FBgn0001319	FBgn0001319	NA	FBtr0112809
FBgn0001321	FBgn0001321	knk	FBtr0082194
FBgn0001324	FBgn0001324	NA	FBtr0074884
FBgn0001332	FBgn0001332	L	FBtr0087492
FBgn0001565	FBgn0001565	NA	FBtr0077221
FBgn0001612	FBgn0001612	l(1)dd4	FBtr0073841
FBgn0001624	FBgn0001624	NA	FBtr0112813
FBgn0001941	FBgn0001941	ifc	FBtr0079179
FBgn0001942	FBgn0001942	eIF-4a	FBtr0079177
FBgn0001961	FBgn0001961	Sop2	FBtr0080570
FBgn0001967	FBgn0001967	NA	FBtr0290243
FBgn0001970	FBgn0001970	NA	FBtr0080629
FBgn0001974	FBgn0001974	NA	FBtr0080696
FBgn0001989	FBgn0001989	l(2)35Di	FBtr0080772
FBgn0001992	FBgn0001992	NA	FBtr0080828

FBgn0002023	FBgn0002023	Lim3	FBtr0309835
FBgn0002031	FBgn0002031	l(2)37Cc	FBtr0081164
FBgn0002069	FBgn0002069	Aats-asp	FBtr0087784
FBgn0002121	FBgn0002121	NA	FBtr0078171
FBgn0002174	FBgn0002174	l(2)tid	FBtr0089505
FBgn0002183	FBgn0002183	NA	FBtr0072864
FBgn0002283	FBgn0002283	l(3)73Ah	FBtr0075311
FBgn0002306	FBgn0002306	NA	FBtr0081570
FBgn0002431	FBgn0002431	hyd	FBtr0082090
FBgn0002478	FBgn0002478	NA	FBtr0082181
FBgn0002522	FBgn0002522	lab	FBtr0081696
FBgn0002525	FBgn0002525	NA	FBtr0307059
FBgn0002543	FBgn0002543	NA	FBtr0077941
FBgn0002552	FBgn0002552	lin	FBtr0088651
FBgn0002561	FBgn0002561	l(1)sc	FBtr0070074
FBgn0002564	FBgn0002564	NA	FBtr0072463
FBgn0002565	FBgn0002565	NA	FBtr0089324
FBgn0002576	FBgn0002576	NA	FBtr0071320
FBgn0002577	FBgn0002577	NA	FBtr0073555
FBgn0002578	FBgn0002578	m1	FBtr0310083
FBgn0002592	FBgn0002592	m2	FBtr0084981
FBgn0002609	FBgn0002609	NA	FBtr0084958
FBgn0002622	FBgn0002622	NA	FBtr0084410
FBgn0002626	FBgn0002626	NA	FBtr0085593
FBgn0002629	FBgn0002629	NA	FBtr0084980
FBgn0002631	FBgn0002631	HLHm5	FBtr0084979
FBgn0002632	FBgn0002632	m6	FBtr0084959
FBgn0002633	FBgn0002633	NA	FBtr0084960
FBgn0002638	FBgn0002638	NA	FBtr0077074
FBgn0002652	FBgn0002652	NA	FBtr0080887
FBgn0002707	FBgn0002707	NA	FBtr0070656
FBgn0002709	FBgn0002709	NA	FBtr0299823
FBgn0002715	FBgn0002715	NA	FBtr0071761
FBgn0002719	FBgn0002719	Men	FBtr0082660
FBgn0002723	FBgn0002723	NA	FBtr0073521
FBgn0002732	FBgn0002732	NA	FBtr0084956
FBgn0002733	FBgn0002733	HLHmbeta	FBtr0084982
FBgn0002734	FBgn0002734	NA	FBtr0084954
FBgn0002735	FBgn0002735	NA	FBtr0084955
FBgn0002778	FBgn0002778	mnd	FBtr0075627
FBgn0002780	FBgn0002780	mod	FBtr0085874
FBgn0002781	FBgn0002781	NA	FBtr0084064
FBgn0002783	FBgn0002783	NA	FBtr0083238
FBgn0002838	FBgn0002838	NA	FBtr0085063
FBgn0002842	FBgn0002842	sa	FBtr0078401
FBgn0002862	FBgn0002862	NA	FBtr0082884
FBgn0002863	FBgn0002863	NA	FBtr0084561
FBgn0002865	FBgn0002865	NA	FBtr0085277
FBgn0002872	FBgn0002872	NA	FBtr0072856
FBgn0002873	FBgn0002873	NA	FBtr0308328
FBgn0002878	FBgn0002878	NA	FBtr0073817
FBgn0002899	FBgn0002899	NA	FBtr0076689
FBgn0002901	FBgn0002901	mus304	FBtr0075122
FBgn0002905	FBgn0002905	mus308	FBtr0082662
FBgn0002909	FBgn0002909	NA	FBtr0299690
FBgn0002917	FBgn0002917	NA	FBtr0112815
FBgn0002921	FBgn0002921	NA	FBtr0089512

FBgn0002922	FBgn0002922	NA	FBtr0084472
FBgn0002931	FBgn0002931	net	FBtr0290323
FBgn0002932	FBgn0002932	neur	FBtr0082003
FBgn0002939	FBgn0002939	NA	FBtr0081031
FBgn0002962	FBgn0002962	nos	FBtr0083732
FBgn0002968	FBgn0002968	NA	FBtr0305914
FBgn0002989	FBgn0002989	NA	FBtr0077673
FBgn0003008	FBgn0003008	or	FBtr0072152
FBgn0003013	FBgn0003013	NA	FBtr0089581
FBgn0003015	FBgn0003015	NA	FBtr0081956
FBgn0003016	FBgn0003016	osp	FBtr0301496
FBgn0003022	FBgn0003022	Ote	FBtr0086768
FBgn0003028	FBgn0003028	NA	FBtr0301914
FBgn0003031	FBgn0003031	NA	FBtr0086740
FBgn0003041	FBgn0003041	NA	FBtr0076772
FBgn0003044	FBgn0003044	Pcl	FBtr0086735
FBgn0003057	FBgn0003057	NA	FBtr0074400
FBgn0003060	FBgn0003060	NA	FBtr0082860
FBgn0003062	FBgn0003062	NA	FBtr0071982
FBgn0003065	FBgn0003065	CG2150	FBtr0085859
FBgn0003071	FBgn0003071	Pfk	FBtr0088422
FBgn0003079	FBgn0003079	phl	FBtr0111025
FBgn0003082	FBgn0003082	NA	FBtr0088839
FBgn0003087	FBgn0003087	NA	FBtr0080001
FBgn0003089	FBgn0003089	pip	FBtr0300340
FBgn0003091	FBgn0003091	NA	FBtr0087049
FBgn0003093	FBgn0003093	NA	FBtr0085359
FBgn0003114	FBgn0003114	NA	FBtr0086306
FBgn0003116	FBgn0003116	pn	FBtr0070389
FBgn0003129	FBgn0003129	NA	FBtr0111026
FBgn0003132	FBgn0003132	NA	FBtr0074017
FBgn0003137	FBgn0003137	NA	FBtr0301837
FBgn0003138	FBgn0003138	Ptp61F	FBtr0310161
FBgn0003141	FBgn0003141	pr	FBtr0089395
FBgn0003149	FBgn0003149	NA	FBtr0076593
FBgn0003159	FBgn0003159	ptr	FBtr0307284
FBgn0003162	FBgn0003162	NA	FBtr0308706
FBgn0003165	FBgn0003165	NA	FBtr0081993
FBgn0003169	FBgn0003169	NA	FBtr0082977
FBgn0003175	FBgn0003175	NA	FBtr0071838
FBgn0003187	FBgn0003187	qua	FBtr0306165
FBgn0003204	FBgn0003204	ras	FBtr0071495
FBgn0003205	FBgn0003205	Ras85D	FBtr0082122
FBgn0003206	FBgn0003206	NA	FBtr0073212
FBgn0003209	FBgn0003209	NA	FBtr0303868
FBgn0003210	FBgn0003210	rb	FBtr0070702
FBgn0003218	FBgn0003218	NA	FBtr0301532
FBgn0003248	FBgn0003248	NA	FBtr0083697
FBgn0003255	FBgn0003255	NA	FBtr0080610
FBgn0003261	FBgn0003261	NA	FBtr0309319
FBgn0003274	FBgn0003274	NA	FBtr0087106
FBgn0003275	FBgn0003275	NA	FBtr0078787
FBgn0003278	FBgn0003278	RpI135	FBtr0078054
FBgn0003292	FBgn0003292	NA	FBtr0076146
FBgn0003295	FBgn0003295	ru	FBtr0072681
FBgn0003300	FBgn0003300	run	FBtr0077279
FBgn0003301	FBgn0003301	NA	FBtr0305581

FBgn0003302	FBgn0003302	NA	FBtr0070889
FBgn0003313	FBgn0003313	NA	FBtr0308136
FBgn0003317	FBgn0003317	sax	FBtr0088833
FBgn0003321	FBgn0003321	NA	FBtr0073411
FBgn0003334	FBgn0003334	NA	FBtr0082102
FBgn0003339	FBgn0003339	NA	FBtr0081657
FBgn0003345	FBgn0003345	NA	FBtr0308131
FBgn0003356	FBgn0003356	NA	FBtr0085512
FBgn0003358	FBgn0003358	NA	FBtr0085511
FBgn0003371	FBgn0003371	NA	FBtr0070471
FBgn0003375	FBgn0003375	NA	FBtr0083461
FBgn0003378	FBgn0003378	NA	FBtr0076160
FBgn0003380	FBgn0003380	NA	FBtr0302902
FBgn0003382	FBgn0003382	sha	FBtr0273343
FBgn0003386	FBgn0003386	NA	FBtr0089275
FBgn0003388	FBgn0003388	shd	FBtr0075676
FBgn0003392	FBgn0003392	NA	FBtr0111036
FBgn0003401	FBgn0003401	NA	FBtr0072237
FBgn0003415	FBgn0003415	NA	FBtr0112830
FBgn0003423	FBgn0003423	NA	FBtr0077210
FBgn0003425	FBgn0003425	NA	FBtr0087303
FBgn0003429	FBgn0003429	NA	FBtr0301582
FBgn0003435	FBgn0003435	sm	FBtr0308797
FBgn0003447	FBgn0003447	sn	FBtr0071102
FBgn0003449	FBgn0003449	NA	FBtr0070748
FBgn0003450	FBgn0003450	NA	FBtr0112833
FBgn0003459	FBgn0003459	stwl	FBtr0075728
FBgn0003462	FBgn0003462	Sod	FBtr0076229
FBgn0003464	FBgn0003464	sol	FBtr0089678
FBgn0003475	FBgn0003475	spir	FBtr0081352
FBgn0003479	FBgn0003479	NA	FBtr0085590
FBgn0003480	FBgn0003480	spn-B	FBtr0082933
FBgn0003482	FBgn0003482	NA	FBtr0085116
FBgn0003486	FBgn0003486	NA	FBtr0077130
FBgn0003495	FBgn0003495	spz	FBtr0085137
FBgn0003499	FBgn0003499	NA	FBtr0083542
FBgn0003501	FBgn0003501	NA	FBtr0304990
FBgn0003502	FBgn0003502	Btk29A	FBtr0112835
FBgn0003507	FBgn0003507	srp	FBtr0083215
FBgn0003513	FBgn0003513	ss	FBtr0083318
FBgn0003514	FBgn0003514	NA	FBtr0070877
FBgn0003515	FBgn0003515	st	FBtr0075391
FBgn0003517	FBgn0003517	sta	FBtr0070289
FBgn0003520	FBgn0003520	stau	FBtr0086783
FBgn0003527	FBgn0003527	NA	FBtr0087825
FBgn0003559	FBgn0003559	NA	FBtr0306544
FBgn0003567	FBgn0003567	NA	FBtr0310002
FBgn0003598	FBgn0003598	NA	FBtr0082736
FBgn0003607	FBgn0003607	Su(var)205	FBtr0079635
FBgn0003612	FBgn0003612	Su(var)2-10	FBtr0088574
FBgn0003638	FBgn0003638	NA	FBtr0070178
FBgn0003651	FBgn0003651	NA	FBtr0082564
FBgn0003655	FBgn0003655	NA	FBtr0070922
FBgn0003656	FBgn0003656	NA	FBtr0071125
FBgn0003659	FBgn0003659	NA	FBtr0100208
FBgn0003660	FBgn0003660	NA	FBtr0088363
FBgn0003714	FBgn0003714	NA	FBtr0308831

FBgn0003715	FBgn0003715	NA	FBtr0100126
FBgn0003719	FBgn0003719	tld	FBtr0084691
FBgn0003721	FBgn0003721	Tm1	FBtr0089962
FBgn0003733	FBgn0003733	tor	FBtr0112838
FBgn0003741	FBgn0003741	NA	FBtr0100244
FBgn0003744	FBgn0003744	trc	FBtr0074922
FBgn0003747	FBgn0003747	NA	FBtr0070802
FBgn0003865	FBgn0003865	NA	FBtr0073582
FBgn0003870	FBgn0003870	ttk	FBtr0085826
FBgn0003882	FBgn0003882	tub	FBtr0273017
FBgn0003885	FBgn0003885	NA	FBtr0081538
FBgn0003886	FBgn0003886	NA	FBtr0082087
FBgn0003889	FBgn0003889	NA	FBtr0082046
FBgn0003890	FBgn0003890	NA	FBtr0085261
FBgn0003916	FBgn0003916	NA	FBtr0078028
FBgn0003944	FBgn0003944	Ubx	FBtr0083347
FBgn0003961	FBgn0003961	NA	FBtr0079486
FBgn0003963	FBgn0003963	ush	FBtr0078063
FBgn0003964	FBgn0003964	NA	FBtr0070346
FBgn0003975	FBgn0003975	vg	FBtr0087785
FBgn0003978	FBgn0003978	NA	FBtr0081347
FBgn0003979	FBgn0003979	NA	FBtr0079239
FBgn0003980	FBgn0003980	NA	FBtr0079171
FBgn0003984	FBgn0003984	NA	FBtr0077082
FBgn0003986	FBgn0003986	NA	FBtr0070110
FBgn0004028	FBgn0004028	wupA	FBtr0074524
FBgn0004045	FBgn0004045	Yp1	FBtr0071419
FBgn0004047	FBgn0004047	Yp3	FBtr0073821
FBgn0004049	FBgn0004049	NA	FBtr0114376
FBgn0004050	FBgn0004050	NA	FBtr0070435
FBgn0004052	FBgn0004052	NA	FBtr0075549
FBgn0004053	FBgn0004053	zen	FBtr0081670
FBgn0004054	FBgn0004054	zen2	FBtr0290296
FBgn0004066	FBgn0004066	Pros28.1	FBtr0074210
FBgn0004087	FBgn0004087	NA	FBtr0110910
FBgn0004101	FBgn0004101	NA	FBtr0290089
FBgn0004102	FBgn0004102	NA	FBtr0300106
FBgn0004103	FBgn0004103	NA	FBtr0082595
FBgn0004107	FBgn0004107	cdc2c	FBtr0300447
FBgn0004108	FBgn0004108	Nrt	FBtr0075322
FBgn0004117	FBgn0004117	Tm2	FBtr0083080
FBgn0004118	FBgn0004118	NA	FBtr0084682
FBgn0004132	FBgn0004132	NA	FBtr0088953
FBgn0004133	FBgn0004133	blow	FBtr0300591
FBgn0004143	FBgn0004143	NA	FBtr0299565
FBgn0004145	FBgn0004145	NA	FBtr0086152
FBgn0004167	FBgn0004167	kst	FBtr0073072
FBgn0004169	FBgn0004169	up	FBtr0073853
FBgn0004170	FBgn0004170	NA	FBtr0070073
FBgn0004171	FBgn0004171	NA	FBtr0085278
FBgn0004174	FBgn0004174	NA	FBtr0081587
FBgn0004179	FBgn0004179	NA	FBtr0078493
FBgn0004183	FBgn0004183	NA	FBtr0078851
FBgn0004198	FBgn0004198	ct	FBtr0114613
FBgn0004228	FBgn0004228	mex1	FBtr0303786
FBgn0004237	FBgn0004237	NA	FBtr0082851
FBgn0004240	FBgn0004240	NA	FBtr0086620

FBgn0004244	FBgn0004244	NA	FBtr0076536
FBgn0004360	FBgn0004360	NA	FBtr0088512
FBgn0004362	FBgn0004362	NA	FBtr0071709
FBgn0004363	FBgn0004363	NA	FBtr0100584
FBgn0004368	FBgn0004368	Ptp4E	FBtr0301793
FBgn0004369	FBgn0004369	NA	FBtr0303148
FBgn0004370	FBgn0004370	NA	FBtr0073524
FBgn0004374	FBgn0004374	NA	FBtr0081332
FBgn0004378	FBgn0004378	Klp61F	FBtr0072733
FBgn0004387	FBgn0004387	NA	FBtr0085211
FBgn0004395	FBgn0004395	NA	FBtr0305573
FBgn0004396	FBgn0004396	CrebA	FBtr0075558
FBgn0004399	FBgn0004399	NA	FBtr0100605
FBgn0004401	FBgn0004401	Pep	FBtr0075199
FBgn0004406	FBgn0004406	tam	FBtr0080571
FBgn0004407	FBgn0004407	NA	FBtr0091627
FBgn0004414	FBgn0004414	NA	FBtr0078482
FBgn0004419	FBgn0004419	me31B	FBtr0079975
FBgn0004425	FBgn0004425	NA	FBtr0072628
FBgn0004427	FBgn0004427	NA	FBtr0072634
FBgn0004428	FBgn0004428	NA	FBtr0072630
FBgn0004429	FBgn0004429	NA	FBtr0072633
FBgn0004430	FBgn0004430	NA	FBtr0072631
FBgn0004431	FBgn0004431	NA	FBtr0072637
FBgn0004435	FBgn0004435	NA	FBtr0304955
FBgn0004436	FBgn0004436	NA	FBtr0078849
FBgn0004449	FBgn0004449	NA	FBtr0306107
FBgn0004456	FBgn0004456	mew	FBtr0073732
FBgn0004462	FBgn0004462	NA	FBtr0074634
FBgn0004465	FBgn0004465	Su(P)	FBtr0075359
FBgn0004509	FBgn0004509	NA	FBtr0089983
FBgn0004510	FBgn0004510	Ets97D	FBtr0085088
FBgn0004512	FBgn0004512	Mdr49	FBtr0087801
FBgn0004514	FBgn0004514	NA	FBtr0078479
FBgn0004552	FBgn0004552	NA	FBtr0073213
FBgn0004554	FBgn0004554	NA	FBtr0083502
FBgn0004556	FBgn0004556	NA	FBtr0075347
FBgn0004572	FBgn0004572	NA	FBtr0086599
FBgn0004574	FBgn0004574	NA	FBtr0310632
FBgn0004575	FBgn0004575	Syn	FBtr0100472
FBgn0004577	FBgn0004577	NA	FBtr0089287
FBgn0004583	FBgn0004583	NA	FBtr0078059
FBgn0004584	FBgn0004584	Rrp1	FBtr0290241
FBgn0004587	FBgn0004587	NA	FBtr0300588
FBgn0004588	FBgn0004588	NA	FBtr0075587
FBgn0004589	FBgn0004589	NA	FBtr0075571
FBgn0004590	FBgn0004590	NA	FBtr0075586
FBgn0004591	FBgn0004591	NA	FBtr0075572
FBgn0004592	FBgn0004592	NA	FBtr0075585
FBgn0004593	FBgn0004593	NA	FBtr0075584
FBgn0004594	FBgn0004594	NA	FBtr0075573
FBgn0004595	FBgn0004595	pros	FBtr0304604
FBgn0004597	FBgn0004597	CycC	FBtr0083010
FBgn0004598	FBgn0004598	NA	FBtr0074267
FBgn0004606	FBgn0004606	zfh1	FBtr0085701
FBgn0004607	FBgn0004607	NA	FBtr0307167
FBgn0004618	FBgn0004618	gl	FBtr0083585

FBgn0004620	FBgn0004620	GluRIIA	FBtr0079106
FBgn0004622	FBgn0004622	NA	FBtr0085507
FBgn0004624	FBgn0004624	NA	FBtr0100146
FBgn0004625	FBgn0004625	NA	FBtr0100670
FBgn0004635	FBgn0004635	rho	FBtr0072694
FBgn0004636	FBgn0004636	NA	FBtr0303154
FBgn0004646	FBgn0004646	ogre	FBtr0309874
FBgn0004647	FBgn0004647	NA	FBtr0070507
FBgn0004648	FBgn0004648	NA	FBtr0070081
FBgn0004650	FBgn0004650	NA	FBtr0070207
FBgn0004652	FBgn0004652	NA	FBtr0083642
FBgn0004656	FBgn0004656	NA	FBtr0071119
FBgn0004687	FBgn0004687	NA	FBtr0070783
FBgn0004698	FBgn0004698	mus210	FBtr0087373
FBgn0004777	FBgn0004777	Ccp84Ag	FBtr0081689
FBgn0004778	FBgn0004778	NA	FBtr0081695
FBgn0004779	FBgn0004779	NA	FBtr0081694
FBgn0004780	FBgn0004780	NA	FBtr0081690
FBgn0004781	FBgn0004781	NA	FBtr0081693
FBgn0004811	FBgn0004811	fs(2)ltoPP43	FBtr0081317
FBgn0004838	FBgn0004838	NA	FBtr0307030
FBgn0004841	FBgn0004841	NA	FBtr0112789
FBgn0004852	FBgn0004852	NA	FBtr0304746
FBgn0004855	FBgn0004855	NA	FBtr0082925
FBgn0004856	FBgn0004856	NA	FBtr0071350
FBgn0004858	FBgn0004858	NA	FBtr0080637
FBgn0004859	FBgn0004859	NA	FBtr0306168
FBgn0004860	FBgn0004860	NA	FBtr0112843
FBgn0004861	FBgn0004861	NA	FBtr0070432
FBgn0004865	FBgn0004865	NA	FBtr0078306
FBgn0004867	FBgn0004867	NA	FBtr0079905
FBgn0004868	FBgn0004868	NA	FBtr0306169
FBgn0004875	FBgn0004875	NA	FBtr0112845
FBgn0004876	FBgn0004876	NA	FBtr0305321
FBgn0004882	FBgn0004882	NA	FBtr0084384
FBgn0004888	FBgn0004888	NA	FBtr0073151
FBgn0004893	FBgn0004893	bowl	FBtr0077492
FBgn0004895	FBgn0004895	NA	FBtr0309789
FBgn0004901	FBgn0004901	Prat	FBtr0304705
FBgn0004903	FBgn0004903	NA	FBtr0085093
FBgn0004908	FBgn0004908	NA	FBtr0081838
FBgn0004910	FBgn0004910	NA	FBtr0073174
FBgn0004913	FBgn0004913	Gnf1	FBtr0100159
FBgn0004914	FBgn0004914	Hnf4	FBtr0079747
FBgn0004919	FBgn0004919	gol	FBtr0300178
FBgn0004922	FBgn0004922	NA	FBtr0071136
FBgn0004924	FBgn0004924	NA	FBtr0074005
FBgn0004925	FBgn0004925	NA	FBtr0074300
FBgn0004926	FBgn0004926	eIF-2beta	FBtr0075968
FBgn0004956	FBgn0004956	NA	FBtr0074581
FBgn0004957	FBgn0004957	por	FBtr0074577
FBgn0005278	FBgn0005278	M(2)21AB	FBtr0089432
FBgn0005355	FBgn0005355	NA	FBtr0082665
FBgn0005386	FBgn0005386	NA	FBtr0306010
FBgn0005390	FBgn0005390	NA	FBtr0070892
FBgn0005391	FBgn0005391	NA	FBtr0071424
FBgn0005410	FBgn0005410	NA	FBtr0112849

FBgn0005411	FBgn0005411	NA	FBtr0074234
FBgn0005427	FBgn0005427	NA	FBtr0089442
FBgn0005536	FBgn0005536	Mbs	FBtr0308214
FBgn0005561	FBgn0005561	NA	FBtr0100343
FBgn0005564	FBgn0005564	Shal	FBtr0074973
FBgn0005585	FBgn0005585	Crc	FBtr0082103
FBgn0005590	FBgn0005590	NA	FBtr0081261
FBgn0005593	FBgn0005593	NA	FBtr0079946
FBgn0005614	FBgn0005614	NA	FBtr0088473
FBgn0005630	FBgn0005630	lola	FBtr0089350
FBgn0005632	FBgn0005632	faf	FBtr0307550
FBgn0005633	FBgn0005633	NA	FBtr0074910
FBgn0005634	FBgn0005634	zip	FBtr0072398
FBgn0005640	FBgn0005640	Eip63E	FBtr0302598
FBgn0005642	FBgn0005642	wdn	FBtr0085356
FBgn0005648	FBgn0005648	Pabp2	FBtr0088785
FBgn0005654	FBgn0005654	NA	FBtr0087768
FBgn0005655	FBgn0005655	mus209	FBtr0086307
FBgn0005658	FBgn0005658	Ets65A	FBtr0077054
FBgn0005659	FBgn0005659	NA	FBtr0085224
FBgn0005660	FBgn0005660	Ets21C	FBtr0078079
FBgn0005664	FBgn0005664	Cry	FBtr0080281
FBgn0005666	FBgn0005666	NA	FBtr0301340
FBgn0005671	FBgn0005671	Vha55	FBtr0082670
FBgn0005672	FBgn0005672	spi	FBtr0081268
FBgn0005677	FBgn0005677	dac	FBtr0080879
FBgn0005683	FBgn0005683	NA	FBtr0080046
FBgn0005696	FBgn0005696	NA	FBtr0114543
FBgn0005775	FBgn0005775	NA	FBtr0309972
FBgn0005779	FBgn0005779	NA	FBtr0077660
FBgn0008646	FBgn0008646	NA	FBtr0082880
FBgn0008651	FBgn0008651	lbl	FBtr0084092
FBgn0008654	FBgn0008654	NA	FBtr0300387
FBgn0010014	FBgn0010014	CanB	FBtr0070764
FBgn0010015	FBgn0010015	NA	FBtr0085732
FBgn0010019	FBgn0010019	Cyp4g1	FBtr0070099
FBgn0010038	FBgn0010038	NA	FBtr0082569
FBgn0010039	FBgn0010039	NA	FBtr0082570
FBgn0010040	FBgn0010040	NA	FBtr0082571
FBgn0010042	FBgn0010042	NA	FBtr0082573
FBgn0010043	FBgn0010043	NA	FBtr0082574
FBgn0010044	FBgn0010044	NA	FBtr0082575
FBgn0010052	FBgn0010052	NA	FBtr0087223
FBgn0010053	FBgn0010053	Jheh1	FBtr0086625
FBgn0010078	FBgn0010078	NA	FBtr0071897
FBgn0010083	FBgn0010083	NA	FBtr0080045
FBgn0010097	FBgn0010097	gammaTub37C	FBtr0081228
FBgn0010100	FBgn0010100	NA	FBtr0300425
FBgn0010105	FBgn0010105	comm	FBtr0075579
FBgn0010173	FBgn0010173	RpA-70	FBtr0081867
FBgn0010194	FBgn0010194	NA	FBtr0074623
FBgn0010197	FBgn0010197	NA	FBtr0112856
FBgn0010198	FBgn0010198	NA	FBtr0073794
FBgn0010213	FBgn0010213	Sod2	FBtr0087078
FBgn0010217	FBgn0010217	NA	FBtr0089186
FBgn0010220	FBgn0010220	Dbp45A	FBtr0088564
FBgn0010225	FBgn0010225	Gel	FBtr0078969

FBgn0010235	FBgn0010235	Klc	FBtr0075951
FBgn0010240	FBgn0010240	Lcch3	FBtr0074129
FBgn0010247	FBgn0010247	NA	FBtr0113885
FBgn0010256	FBgn0010256	NA	FBtr0074279
FBgn0010258	FBgn0010258	NA	FBtr0082853
FBgn0010263	FBgn0010263	NA	FBtr0100468
FBgn0010265	FBgn0010265	NA	FBtr0079724
FBgn0010269	FBgn0010269	Dsor1	FBtr0071313
FBgn0010280	FBgn0010280	NA	FBtr0114589
FBgn0010282	FBgn0010282	NA	FBtr0081719
FBgn0010288	FBgn0010288	Uch	FBtr0077844
FBgn0010292	FBgn0010292	bys	FBtr0071090
FBgn0010300	FBgn0010300	brat	FBtr0081159
FBgn0010309	FBgn0010309	pigeon	FBtr0302561
FBgn0010313	FBgn0010313	corto	FBtr0308119
FBgn0010314	FBgn0010314	NA	FBtr0079799
FBgn0010315	FBgn0010315	NA	FBtr0300393
FBgn0010316	FBgn0010316	dap	FBtr0088469
FBgn0010317	FBgn0010317	NA	FBtr0073127
FBgn0010323	FBgn0010323	NA	FBtr0078072
FBgn0010328	FBgn0010328	NA	FBtr0112857
FBgn0010339	FBgn0010339	NA	FBtr0087976
FBgn0010340	FBgn0010340	140up	FBtr0082909
FBgn0010341	FBgn0010341	Cdc42	FBtr0309216
FBgn0010342	FBgn0010342	Map60	FBtr0088530
FBgn0010348	FBgn0010348	NA	FBtr0078573
FBgn0010349	FBgn0010349	NA	FBtr0073359
FBgn0010352	FBgn0010352	Nc73EF	FBtr0112860
FBgn0010355	FBgn0010355	NA	FBtr0304903
FBgn0010356	FBgn0010356	Taf5	FBtr0088240
FBgn0010357	FBgn0010357	NA	FBtr0088122
FBgn0010359	FBgn0010359	NA	FBtr0088159
FBgn0010381	FBgn0010381	NA	FBtr0073073
FBgn0010382	FBgn0010382	CycE	FBtr0080776
FBgn0010385	FBgn0010385	NA	FBtr0088432
FBgn0010387	FBgn0010387	Dbi	FBtr0076915
FBgn0010388	FBgn0010388	NA	FBtr0309055
FBgn0010401	FBgn0010401	NA	FBtr0300321
FBgn0010407	FBgn0010407	NA	FBtr0079978
FBgn0010408	FBgn0010408	NA	FBtr0076424
FBgn0010411	FBgn0010411	NA	FBtr0086274
FBgn0010412	FBgn0010412	NA	FBtr0074313
FBgn0010415	FBgn0010415	Sdc	FBtr0273207
FBgn0010417	FBgn0010417	Taf6	FBtr0074941
FBgn0010421	FBgn0010421	NA	FBtr0082310
FBgn0010422	FBgn0010422	NA	FBtr0080722
FBgn0010423	FBgn0010423	NA	FBtr0088184
FBgn0010424	FBgn0010424	TpnC73F	FBtr0089585
FBgn0010426	FBgn0010426	NA	FBtr0087341
FBgn0010434	FBgn0010434	cora	FBtr0086509
FBgn0010435	FBgn0010435	emp	FBtr0072400
FBgn0010452	FBgn0010452	NA	FBtr0075890
FBgn0010470	FBgn0010470	Fkbp13	FBtr0306013
FBgn0010473	FBgn0010473	tutl	FBtr0301863
FBgn0010482	FBgn0010482	NA	FBtr0301423
FBgn0010504	FBgn0010504	NA	FBtr0088811
FBgn0010520	FBgn0010520	Bka	FBtr0079874

FBgn0010531	FBgn0010531	NA	FBtr0300944
FBgn0010548	FBgn0010548	Aldh-III	FBtr0300481
FBgn0010549	FBgn0010549	NA	FBtr0302504
FBgn0010551	FBgn0010551	NA	FBtr0112863
FBgn0010575	FBgn0010575	NA	FBtr0301547
FBgn0010602	FBgn0010602	NA	FBtr0310028
FBgn0010607	FBgn0010607	NA	FBtr0079051
FBgn0010612	FBgn0010612	NA	FBtr0305994
FBgn0010621	FBgn0010621	Cct5	FBtr0087987
FBgn0010622	FBgn0010622	l(2)06496	FBtr0071985
FBgn0010638	FBgn0010638	Sec61beta	FBtr0087498
FBgn0010715	FBgn0010715	NA	FBtr0075945
FBgn0010747	FBgn0010747	NA	FBtr0077169
FBgn0010770	FBgn0010770	NA	FBtr0084012
FBgn0010772	FBgn0010772	NA	FBtr0078728
FBgn0010812	FBgn0010812	Tom34	FBtr0081788
FBgn0010825	FBgn0010825	NA	FBtr0305697
FBgn0010830	FBgn0010830	l(3)04053	FBtr0078535
FBgn0010894	FBgn0010894	NA	FBtr0077136
FBgn0010905	FBgn0010905	NA	FBtr0304614
FBgn0010926	FBgn0010926	l(3)07882	FBtr0083504
FBgn0011013	FBgn0011013	NA	FBtr0085863
FBgn0011202	FBgn0011202	NA	FBtr0081410
FBgn0011204	FBgn0011204	NA	FBtr0072706
FBgn0011205	FBgn0011205	NA	FBtr0078187
FBgn0011206	FBgn0011206	NA	FBtr0076538
FBgn0011207	FBgn0011207	NA	FBtr0079847
FBgn0011211	FBgn0011211	NA	FBtr0071883
FBgn0011217	FBgn0011217	NA	FBtr0083018
FBgn0011219	FBgn0011219	NA	FBtr0079571
FBgn0011224	FBgn0011224	heph	FBtr0306693
FBgn0011225	FBgn0011225	NA	FBtr0301368
FBgn0011227	FBgn0011227	NA	FBtr0087861
FBgn0011232	FBgn0011232	scat	FBtr0079809
FBgn0011239	FBgn0011239	NA	FBtr0080731
FBgn0011241	FBgn0011241	cbx	FBtr0088452
FBgn0011244	FBgn0011244	NA	FBtr0078000
FBgn0011259	FBgn0011259	NA	FBtr0303877
FBgn0011270	FBgn0011270	NA	FBtr0082620
FBgn0011272	FBgn0011272	NA	FBtr0310482
FBgn0011273	FBgn0011273	NA	FBtr0301963
FBgn0011274	FBgn0011274	Dif	FBtr0301577
FBgn0011276	FBgn0011276	NA	FBtr0070506
FBgn0011282	FBgn0011282	NA	FBtr0300016
FBgn0011283	FBgn0011283	NA	FBtr0079455
FBgn0011285	FBgn0011285	NA	FBtr0077360
FBgn0011286	FBgn0011286	Rya-r44F	FBtr0304572
FBgn0011288	FBgn0011288	NA	FBtr0111143
FBgn0011290	FBgn0011290	NA	FBtr0082442
FBgn0011291	FBgn0011291	NA	FBtr0079924
FBgn0011296	FBgn0011296	l(2)efl	FBtr0072101
FBgn0011297	FBgn0011297	NA	FBtr0072095
FBgn0011300	FBgn0011300	NA	FBtr0088658
FBgn0011327	FBgn0011327	Uch-L3	FBtr0076472
FBgn0011455	FBgn0011455	l(3)neo18	FBtr0076023
FBgn0011474	FBgn0011474	NA	FBtr0082930
FBgn0011476	FBgn0011476	NA	FBtr0301089

FBgn0011481	FBgn0011481	Ssdp	FBtr0083612
FBgn0011509	FBgn0011509	NA	FBtr0076598
FBgn0011554	FBgn0011554	NA	FBtr0088163
FBgn0011555	FBgn0011555	NA	FBtr0088162
FBgn0011556	FBgn0011556	NA	FBtr0088164
FBgn0011559	FBgn0011559	NA	FBtr0081054
FBgn0011571	FBgn0011571	caz	FBtr0074217
FBgn0011576	FBgn0011576	NA	FBtr0070387
FBgn0011577	FBgn0011577	NA	FBtr0305901
FBgn0011582	FBgn0011582	NA	FBtr0308191
FBgn0011584	FBgn0011584	Trp1	FBtr0079932
FBgn0011586	FBgn0011586	NA	FBtr0071267
FBgn0011596	FBgn0011596	NA	FBtr0084399
FBgn0011603	FBgn0011603	ine	FBtr0077446
FBgn0011638	FBgn0011638	La	FBtr0081374
FBgn0011642	FBgn0011642	NA	FBtr0089212
FBgn0011655	FBgn0011655	NA	FBtr0085815
FBgn0011656	FBgn0011656	NA	FBtr0088447
FBgn0011661	FBgn0011661	Moe	FBtr0308193
FBgn0011666	FBgn0011666	NA	FBtr0084882
FBgn0011668	FBgn0011668	NA	FBtr0084914
FBgn0011669	FBgn0011669	NA	FBtr0084915
FBgn0011670	FBgn0011670	NA	FBtr0084916
FBgn0011672	FBgn0011672	Mvl	FBtr0301477
FBgn0011693	FBgn0011693	Pdh	FBtr0075426
FBgn0011694	FBgn0011694	NA	FBtr0072455
FBgn0011701	FBgn0011701	repo	FBtr0083564
FBgn0011704	FBgn0011704	RnrS	FBtr0088046
FBgn0011710	FBgn0011710	Sep1	FBtr0077198
FBgn0011715	FBgn0011715	Snr1	FBtr0078682
FBgn0011742	FBgn0011742	NA	FBtr0112868
FBgn0011743	FBgn0011743	NA	FBtr0087023
FBgn0011745	FBgn0011745	Arp87C	FBtr0082652
FBgn0011746	FBgn0011746	NA	FBtr0300501
FBgn0011747	FBgn0011747	NA	FBtr0089173
FBgn0011754	FBgn0011754	NA	FBtr0301501
FBgn0011760	FBgn0011760	NA	FBtr0307065
FBgn0011762	FBgn0011762	NA	FBtr0076603
FBgn0011764	FBgn0011764	NA	FBtr0089261
FBgn0011768	FBgn0011768	NA	FBtr0082290
FBgn0011769	FBgn0011769	NA	FBtr0303475
FBgn0011770	FBgn0011770	NA	FBtr0071472
FBgn0011771	FBgn0011771	Hem	FBtr0078510
FBgn0011774	FBgn0011774	Irbp	FBtr0082385
FBgn0011785	FBgn0011785	NA	FBtr0084604
FBgn0011787	FBgn0011787	mRpL12	FBtr0308224
FBgn0011802	FBgn0011802	NA	FBtr0076292
FBgn0011817	FBgn0011817	nmo	FBtr0076755
FBgn0011822	FBgn0011822	pcl	FBtr0070100
FBgn0011823	FBgn0011823	NA	FBtr0079937
FBgn0011826	FBgn0011826	NA	FBtr0309793
FBgn0011832	FBgn0011832	NA	FBtr0077806
FBgn0011836	FBgn0011836	NA	FBtr0076361
FBgn0012034	FBgn0012034	NA	FBtr0078413
FBgn0012036	FBgn0012036	Aldh	FBtr0079806
FBgn0012042	FBgn0012042	NA	FBtr0087437
FBgn0012051	FBgn0012051	CalpA	FBtr0112869

FBgn0012058	FBgn0012058	Cdc27	FBtr0076890
FBgn0012344	FBgn0012344	NA	FBtr0112870
FBgn0013263	FBgn0013263	Trl	FBtr0075707
FBgn0013279	FBgn0013279	NA	FBtr0082638
FBgn0013303	FBgn0013303	NA	FBtr0302048
FBgn0013305	FBgn0013305	Nmda1	FBtr0089878
FBgn0013307	FBgn0013307	Odc1	FBtr0088863
FBgn0013308	FBgn0013308	NA	FBtr0088864
FBgn0013323	FBgn0013323	NA	FBtr0301522
FBgn0013325	FBgn0013325	NA	FBtr0086533
FBgn0013334	FBgn0013334	NA	FBtr0083205
FBgn0013342	FBgn0013342	NA	FBtr0306578
FBgn0013343	FBgn0013343	Syx1A	FBtr0303161
FBgn0013347	FBgn0013347	TfIIA-S	FBtr0084526
FBgn0013348	FBgn0013348	NA	FBtr0310003
FBgn0013435	FBgn0013435	NA	FBtr0088429
FBgn0013442	FBgn0013442	NA	FBtr0273229
FBgn0013467	FBgn0013467	igl	FBtr0087361
FBgn0013548	FBgn0013548	l(2)dtl	FBtr0072085
FBgn0013576	FBgn0013576	l(3)82Fd	FBtr0306694
FBgn0013591	FBgn0013591	NA	FBtr0302046
FBgn0013672	FBgn0013672	NA	FBtr0100867
FBgn0013673	FBgn0013673	NA	FBtr0100866
FBgn0013674	FBgn0013674	NA	FBtr0100861
FBgn0013675	FBgn0013675	NA	FBtr0100863
FBgn0013676	FBgn0013676	NA	FBtr0100868
FBgn0013678	FBgn0013678	NA	FBtr0100884
FBgn0013679	FBgn0013679	NA	FBtr0100886
FBgn0013680	FBgn0013680	NA	FBtr0100857
FBgn0013681	FBgn0013681	NA	FBtr0100870
FBgn0013682	FBgn0013682	NA	FBtr0100879
FBgn0013683	FBgn0013683	NA	FBtr0100880
FBgn0013684	FBgn0013684	NA	FBtr0100877
FBgn0013685	FBgn0013685	NA	FBtr0100883
FBgn0013686	FBgn0013686	NA	FBtr0100888
FBgn0013688	FBgn0013688	NA	FBtr0100890
FBgn0013689	FBgn0013689	NA	FBtr0100871
FBgn0013690	FBgn0013690	NA	FBtr0100859
FBgn0013691	FBgn0013691	NA	FBtr0100865
FBgn0013692	FBgn0013692	NA	FBtr0100875
FBgn0013693	FBgn0013693	NA	FBtr0100876
FBgn0013694	FBgn0013694	NA	FBtr0100869
FBgn0013695	FBgn0013695	NA	FBtr0100878
FBgn0013696	FBgn0013696	NA	FBtr0100854
FBgn0013697	FBgn0013697	NA	FBtr0100864
FBgn0013698	FBgn0013698	NA	FBtr0100887
FBgn0013699	FBgn0013699	NA	FBtr0100862
FBgn0013700	FBgn0013700	NA	FBtr0100856
FBgn0013701	FBgn0013701	NA	FBtr0100873
FBgn0013702	FBgn0013702	NA	FBtr0100882
FBgn0013703	FBgn0013703	NA	FBtr0100855
FBgn0013704	FBgn0013704	NA	FBtr0100872
FBgn0013705	FBgn0013705	NA	FBtr0100874
FBgn0013706	FBgn0013706	NA	FBtr0100885
FBgn0013707	FBgn0013707	NA	FBtr0100881
FBgn0013708	FBgn0013708	NA	FBtr0100889
FBgn0013709	FBgn0013709	NA	FBtr0100858

FBgn0013710	FBgn0013710	NA	FBtr0100860
FBgn0013717	FBgn0013717	NA	FBtr0290259
FBgn0013718	FBgn0013718	nuf	FBtr0306619
FBgn0013733	FBgn0013733	NA	FBtr0087621
FBgn0013746	FBgn0013746	alien	FBtr0079781
FBgn0013753	FBgn0013753	NA	FBtr0072758
FBgn0013759	FBgn0013759	Caki	FBtr0301327
FBgn0013765	FBgn0013765	NA	FBtr0309891
FBgn0013767	FBgn0013767	NA	FBtr0082927
FBgn0013770	FBgn0013770	NA	FBtr0087592
FBgn0013771	FBgn0013771	NA	FBtr0300128
FBgn0013772	FBgn0013772	NA	FBtr0087455
FBgn0013773	FBgn0013773	Cyp6a22	FBtr0087450
FBgn0013799	FBgn0013799	Deaf1	FBtr0074882
FBgn0013809	FBgn0013809	NA	FBtr0074509
FBgn0013810	FBgn0013810	NA	FBtr0304742
FBgn0013811	FBgn0013811	NA	FBtr0303106
FBgn0013812	FBgn0013812	NA	FBtr0273236
FBgn0013813	FBgn0013813	NA	FBtr0301302
FBgn0013948	FBgn0013948	NA	FBtr0084169
FBgn0013953	FBgn0013953	Esp	FBtr0304054
FBgn0013954	FBgn0013954	NA	FBtr0086515
FBgn0013972	FBgn0013972	NA	FBtr0085453
FBgn0013973	FBgn0013973	NA	FBtr0085753
FBgn0013981	FBgn0013981	NA	FBtr0082963
FBgn0013983	FBgn0013983	imd	FBtr0086725
FBgn0013984	FBgn0013984	NA	FBtr0084121
FBgn0013987	FBgn0013987	NA	FBtr0302940
FBgn0013988	FBgn0013988	NA	FBtr0302791
FBgn0013995	FBgn0013995	Calx	FBtr0309205
FBgn0013997	FBgn0013997	NA	FBtr0075998
FBgn0014000	FBgn0014000	NA	FBtr0081306
FBgn0014001	FBgn0014001	NA	FBtr0114607
FBgn0014002	FBgn0014002	Pdi	FBtr0075648
FBgn0014006	FBgn0014006	NA	FBtr0083819
FBgn0014011	FBgn0014011	Rac2	FBtr0076867
FBgn0014022	FBgn0014022	Rlb1	FBtr0082062
FBgn0014024	FBgn0014024	NA	FBtr0301880
FBgn0014026	FBgn0014026	NA	FBtr0070916
FBgn0014028	FBgn0014028	SdhB	FBtr0086156
FBgn0014029	FBgn0014029	NA	FBtr0083945
FBgn0014031	FBgn0014031	NA	FBtr0070913
FBgn0014037	FBgn0014037	NA	FBtr0074920
FBgn0014073	FBgn0014073	NA	FBtr0073323
FBgn0014076	FBgn0014076	NA	FBtr0080216
FBgn0014127	FBgn0014127	NA	FBtr0081349
FBgn0014135	FBgn0014135	NA	FBtr0300123
FBgn0014141	FBgn0014141	NA	FBtr0290014
FBgn0014189	FBgn0014189	NA	FBtr0079123
FBgn0014342	FBgn0014342	NA	FBtr0089313
FBgn0014362	FBgn0014362	NA	FBtr0304980
FBgn0014366	FBgn0014366	noi	FBtr0078751
FBgn0014380	FBgn0014380	RhoL	FBtr0305340
FBgn0014391	FBgn0014391	sun	FBtr0074177
FBgn0014395	FBgn0014395	NA	FBtr0077345
FBgn0014396	FBgn0014396	tim	FBtr0077567
FBgn0014455	FBgn0014455	NA	FBtr0074030

FBgn0014464	FBgn0014464	NA	FBtr0300066
FBgn0014465	FBgn0014465	NA	FBtr0071201
FBgn0014466	FBgn0014466	NA	FBtr0071202
FBgn0014467	FBgn0014467	NA	FBtr0112878
FBgn0014848	FBgn0014848	NA	FBtr0305334
FBgn0014849	FBgn0014849	NA	FBtr0075574
FBgn0014851	FBgn0014851	NA	FBtr0075575
FBgn0014857	FBgn0014857	NA	FBtr0079010
FBgn0014859	FBgn0014859	NA	FBtr0081397
FBgn0014863	FBgn0014863	Mlp84B	FBtr0081616
FBgn0014869	FBgn0014869	Pglym78	FBtr0085384
FBgn0014877	FBgn0014877	Roe1	FBtr0087675
FBgn0014879	FBgn0014879	Set	FBtr0083062
FBgn0014903	FBgn0014903	NA	FBtr0070175
FBgn0014906	FBgn0014906	CG3488	FBtr0308315
FBgn0014930	FBgn0014930	NA	FBtr0081763
FBgn0014931	FBgn0014931	NA	FBtr0304703
FBgn0014949	FBgn0014949	NA	FBtr0084278
FBgn0015008	FBgn0015008	NA	FBtr0083380
FBgn0015010	FBgn0015010	NA	FBtr0305582
FBgn0015019	FBgn0015019	Cctgamma	FBtr0302370
FBgn0015024	FBgn0015024	NA	FBtr0073680
FBgn0015025	FBgn0015025	CkIIalpha-i1	FBtr0075504
FBgn0015031	FBgn0015031	NA	FBtr0307035
FBgn0015032	FBgn0015032	NA	FBtr0085712
FBgn0015035	FBgn0015035	NA	FBtr0079925
FBgn0015036	FBgn0015036	NA	FBtr0070388
FBgn0015037	FBgn0015037	NA	FBtr0088592
FBgn0015038	FBgn0015038	NA	FBtr0089056
FBgn0015039	FBgn0015039	NA	FBtr0089055
FBgn0015075	FBgn0015075	NA	FBtr0078492
FBgn0015129	FBgn0015129	NA	FBtr0085467
FBgn0015221	FBgn0015221	Fer2LCH	FBtr0085623
FBgn0015222	FBgn0015222	Fer1HCH	FBtr0085631
FBgn0015239	FBgn0015239	Hr78	FBtr0078392
FBgn0015247	FBgn0015247	NA	FBtr0087296
FBgn0015268	FBgn0015268	NA	FBtr0305989
FBgn0015269	FBgn0015269	NA	FBtr0100113
FBgn0015271	FBgn0015271	Orc5	FBtr0080543
FBgn0015277	FBgn0015277	Pi3K59F	FBtr0072064
FBgn0015278	FBgn0015278	NA	FBtr0076088
FBgn0015282	FBgn0015282	Pros26.4	FBtr0084520
FBgn0015283	FBgn0015283	NA	FBtr0078368
FBgn0015286	FBgn0015286	NA	FBtr0070583
FBgn0015288	FBgn0015288	NA	FBtr0070148
FBgn0015295	FBgn0015295	NA	FBtr0087244
FBgn0015298	FBgn0015298	Srp19	FBtr0076879
FBgn0015299	FBgn0015299	NA	FBtr0079590
FBgn0015300	FBgn0015300	NA	FBtr0072351
FBgn0015316	FBgn0015316	NA	FBtr0301665
FBgn0015321	FBgn0015321	UbcD4	FBtr0076395
FBgn0015331	FBgn0015331	abs	FBtr0078967
FBgn0015351	FBgn0015351	CG14906	FBtr0112880
FBgn0015360	FBgn0015360	oxt	FBtr0072904
FBgn0015371	FBgn0015371	chn	FBtr0305217
FBgn0015376	FBgn0015376	NA	FBtr0077541
FBgn0015379	FBgn0015379	NA	FBtr0077193

FBgn0015393	FBgn0015393	NA	FBtr0079846
FBgn0015400	FBgn0015400	NA	FBtr0080252
FBgn0015509	FBgn0015509	lin19	FBtr0088848
FBgn0015513	FBgn0015513	mbc	FBtr0304729
FBgn0015519	FBgn0015519	NA	FBtr0289988
FBgn0015521	FBgn0015521	NA	FBtr0077623
FBgn0015524	FBgn0015524	otp	FBtr0290289
FBgn0015527	FBgn0015527	pen	FBtr0077223
FBgn0015541	FBgn0015541	NA	FBtr0112882
FBgn0015542	FBgn0015542	NA	FBtr0085565
FBgn0015546	FBgn0015546	NA	FBtr0100661
FBgn0015553	FBgn0015553	NA	FBtr0081131
FBgn0015558	FBgn0015558	NA	FBtr0077224
FBgn0015567	FBgn0015567	NA	FBtr0089488
FBgn0015568	FBgn0015568	NA	FBtr0081544
FBgn0015569	FBgn0015569	NA	FBtr0289953
FBgn0015570	FBgn0015570	NA	FBtr0310421
FBgn0015571	FBgn0015571	NA	FBtr0310527
FBgn0015572	FBgn0015572	NA	FBtr0081546
FBgn0015574	FBgn0015574	NA	FBtr0081549
FBgn0015575	FBgn0015575	alpha-Est7	FBtr0081550
FBgn0015577	FBgn0015577	NA	FBtr0302355
FBgn0015582	FBgn0015582	dare	FBtr0088176
FBgn0015584	FBgn0015584	NA	FBtr0087080
FBgn0015586	FBgn0015586	NA	FBtr0075038
FBgn0015600	FBgn0015600	toc	FBtr0077662
FBgn0015602	FBgn0015602	BEAF-32	FBtr0305588
FBgn0015609	FBgn0015609	NA	FBtr0100313
FBgn0015610	FBgn0015610	NA	FBtr0083052
FBgn0015614	FBgn0015614	NA	FBtr0309866
FBgn0015617	FBgn0015617	NA	FBtr0070756
FBgn0015621	FBgn0015621	NA	FBtr0078011
FBgn0015622	FBgn0015622	NA	FBtr0110908
FBgn0015657	FBgn0015657	NA	FBtr0077124
FBgn0015663	FBgn0015663	NA	FBtr0077532
FBgn0015664	FBgn0015664	NA	FBtr0079889
FBgn0015714	FBgn0015714	Cyp6a17	FBtr0087451
FBgn0015754	FBgn0015754	Lis-1	FBtr0087241
FBgn0015764	FBgn0015764	NA	FBtr0110977
FBgn0015765	FBgn0015765	Mpk2	FBtr0084581
FBgn0015772	FBgn0015772	Nak	FBtr0081187
FBgn0015773	FBgn0015773	NetA	FBtr0308337
FBgn0015774	FBgn0015774	NA	FBtr0302016
FBgn0015776	FBgn0015776	nrv1	FBtr0079384
FBgn0015781	FBgn0015781	P5cr	FBtr0083713
FBgn0015791	FBgn0015791	NA	FBtr0080627
FBgn0015794	FBgn0015794	Rab-RP4	FBtr0070849
FBgn0015795	FBgn0015795	NA	FBtr0084503
FBgn0015797	FBgn0015797	Rab6	FBtr0080361
FBgn0015801	FBgn0015801	Reg-5	FBtr0305671
FBgn0015803	FBgn0015803	NA	FBtr0112884
FBgn0015805	FBgn0015805	NA	FBtr0073317
FBgn0015806	FBgn0015806	NA	FBtr0077114
FBgn0015808	FBgn0015808	NA	FBtr0081109
FBgn0015816	FBgn0015816	Slh	FBtr0077783
FBgn0015818	FBgn0015818	NA	FBtr0070894
FBgn0015828	FBgn0015828	TfIIEalpha	FBtr0076108

FBgn0015829	FBgn0015829	NA	FBtr0300449
FBgn0015831	FBgn0015831	NA	FBtr0307276
FBgn0015834	FBgn0015834	NA	FBtr0079053
FBgn0015838	FBgn0015838	NA	FBtr0088566
FBgn0015844	FBgn0015844	NA	FBtr0071576
FBgn0015903	FBgn0015903	apt	FBtr0072070
FBgn0015904	FBgn0015904	ara	FBtr0075908
FBgn0015907	FBgn0015907	NA	FBtr0086221
FBgn0015924	FBgn0015924	crq	FBtr0078087
FBgn0015925	FBgn0015925	NA	FBtr0089871
FBgn0015926	FBgn0015926	NA	FBtr0074007
FBgn0015929	FBgn0015929	dpa	FBtr0088982
FBgn0015933	FBgn0015933	NA	FBtr0089743
FBgn0015949	FBgn0015949	hrg	FBtr0086483
FBgn0016013	FBgn0016013	Faa	FBtr0073278
FBgn0016034	FBgn0016034	mael	FBtr0089631
FBgn0016047	FBgn0016047	nompA	FBtr0089381
FBgn0016053	FBgn0016053	NA	FBtr0112520
FBgn0016054	FBgn0016054	phr6-4	FBtr0081405
FBgn0016059	FBgn0016059	NA	FBtr0086890
FBgn0016061	FBgn0016061	side	FBtr0309180
FBgn0016070	FBgn0016070	NA	FBtr0076550
FBgn0016076	FBgn0016076	NA	FBtr0079071
FBgn0016078	FBgn0016078	wun	FBtr0088552
FBgn0016080	FBgn0016080	NA	FBtr0074405
FBgn0016081	FBgn0016081	NA	FBtr0306240
FBgn0016119	FBgn0016119	NA	FBtr0305982
FBgn0016120	FBgn0016120	ATPsyn-d	FBtr0083728
FBgn0016131	FBgn0016131	NA	FBtr0087104
FBgn0016675	FBgn0016675	NA	FBtr0081201
FBgn0016687	FBgn0016687	Nurf-38	FBtr0305985
FBgn0016691	FBgn0016691	Oscp	FBtr0083064
FBgn0016693	FBgn0016693	Past1	FBtr0082655
FBgn0016694	FBgn0016694	NA	FBtr0306535
FBgn0016696	FBgn0016696	Pitslre	FBtr0304935
FBgn0016697	FBgn0016697	ProsMA5	FBtr0086910
FBgn0016700	FBgn0016700	Rab1	FBtr0084104
FBgn0016701	FBgn0016701	NA	FBtr0086831
FBgn0016715	FBgn0016715	NA	FBtr0072561
FBgn0016726	FBgn0016726	RpL29	FBtr0071593
FBgn0016754	FBgn0016754	NA	FBtr0112888
FBgn0016756	FBgn0016756	Ubp64E	FBtr0077095
FBgn0016762	FBgn0016762	angel	FBtr0072083
FBgn0016792	FBgn0016792	NA	FBtr0082086
FBgn0016797	FBgn0016797	NA	FBtr0112890
FBgn0016919	FBgn0016919	NA	FBtr0085925
FBgn0016920	FBgn0016920	NA	FBtr0300012
FBgn0016970	FBgn0016970	NA	FBtr0088505
FBgn0016974	FBgn0016974	NA	FBtr0070923
FBgn0016975	FBgn0016975	NA	FBtr0302020
FBgn0016976	FBgn0016976	NA	FBtr0301619
FBgn0016977	FBgn0016977	NA	FBtr0078121
FBgn0016978	FBgn0016978	snRNP70K	FBtr0079355
FBgn0016983	FBgn0016983	smid	FBtr0308701
FBgn0017397	FBgn0017397	NA	FBtr0084177
FBgn0017418	FBgn0017418	NA	FBtr0306057
FBgn0017429	FBgn0017429	CG5989	FBtr0076649

FBgn0017430	FBgn0017430	Nelf-E	FBtr0076650
FBgn0017448	FBgn0017448	NA	FBtr0085861
FBgn0017456	FBgn0017456	NA	FBtr0081552
FBgn0017545	FBgn0017545	NA	FBtr0308296
FBgn0017549	FBgn0017549	NA	FBtr0087254
FBgn0017556	FBgn0017556	NA	FBtr0072350
FBgn0017557	FBgn0017557	NA	FBtr0083928
FBgn0017558	FBgn0017558	Pdk	FBtr0306807
FBgn0017561	FBgn0017561	NA	FBtr0073404
FBgn0017566	FBgn0017566	ND75	FBtr0071180
FBgn0017567	FBgn0017567	ND23	FBtr0083191
FBgn0017577	FBgn0017577	Mcm5	FBtr0082279
FBgn0017578	FBgn0017578	Max	FBtr0075018
FBgn0017579	FBgn0017579	NA	FBtr0076633
FBgn0019624	FBgn0019624	CoVa	FBtr0082473
FBgn0019644	FBgn0019644	NA	FBtr0076406
FBgn0019660	FBgn0019660	NA	FBtr0073515
FBgn0019662	FBgn0019662	NA	FBtr0302045
FBgn0019686	FBgn0019686	lok	FBtr0306234
FBgn0019809	FBgn0019809	NA	FBtr0079838
FBgn0019828	FBgn0019828	NA	FBtr0305962
FBgn0019925	FBgn0019925	Surf4	FBtr0083117
FBgn0019928	FBgn0019928	NA	FBtr0087664
FBgn0019932	FBgn0019932	NA	FBtr0079994
FBgn0019936	FBgn0019936	RpS20	FBtr0083964
FBgn0019947	FBgn0019947	NA	FBtr0078192
FBgn0019957	FBgn0019957	ND42	FBtr0084173
FBgn0019960	FBgn0019960	CG6455	FBtr0290271
FBgn0019982	FBgn0019982	NA	FBtr0077453
FBgn0019985	FBgn0019985	NA	FBtr0089184
FBgn0019990	FBgn0019990	NA	FBtr0085787
FBgn0020018	FBgn0020018	Ppox	FBtr0084664
FBgn0020235	FBgn0020235	ATPsyn-gamma	FBtr0085540
FBgn0020236	FBgn0020236	NA	FBtr0300597
FBgn0020238	FBgn0020238	NA	FBtr0083566
FBgn0020249	FBgn0020249	stck	FBtr0306260
FBgn0020251	FBgn0020251	NA	FBtr0076968
FBgn0020255	FBgn0020255	NA	FBtr0073472
FBgn0020258	FBgn0020258	ppk	FBtr0080638
FBgn0020261	FBgn0020261	NA	FBtr0302082
FBgn0020270	FBgn0020270	NA	FBtr0308750
FBgn0020272	FBgn0020272	NA	FBtr0077211
FBgn0020277	FBgn0020277	NA	FBtr0074969
FBgn0020278	FBgn0020278	loco	FBtr0084273
FBgn0020279	FBgn0020279	NA	FBtr0088776
FBgn0020299	FBgn0020299	stumps	FBtr0112893
FBgn0020304	FBgn0020304	drongo	FBtr0302120
FBgn0020306	FBgn0020306	dom	FBtr0071603
FBgn0020307	FBgn0020307	dve	FBtr0071775
FBgn0020309	FBgn0020309	NA	FBtr0080261
FBgn0020367	FBgn0020367	NA	FBtr0080418
FBgn0020371	FBgn0020371	NA	FBtr0089699
FBgn0020377	FBgn0020377	NA	FBtr0088004
FBgn0020378	FBgn0020378	NA	FBtr0310290
FBgn0020379	FBgn0020379	NA	FBtr0305997
FBgn0020381	FBgn0020381	NA	FBtr0114561
FBgn0020385	FBgn0020385	NA	FBtr0082264

FBgn0020386	FBgn0020386	Pk61C	FBtr0072467
FBgn0020388	FBgn0020388	Pcaf	FBtr0075969
FBgn0020389	FBgn0020389	Paps	FBtr0111108
FBgn0020391	FBgn0020391	NA	FBtr0087728
FBgn0020399	FBgn0020399	NA	FBtr0083260
FBgn0020407	FBgn0020407	NA	FBtr0083259
FBgn0020414	FBgn0020414	Idgf3	FBtr0080864
FBgn0020415	FBgn0020415	Idgf2	FBtr0305984
FBgn0020416	FBgn0020416	NA	FBtr0080860
FBgn0020429	FBgn0020429	NA	FBtr0089577
FBgn0020439	FBgn0020439	fau	FBtr0307389
FBgn0020440	FBgn0020440	Fak56D	FBtr0114605
FBgn0020503	FBgn0020503	NA	FBtr0080976
FBgn0020509	FBgn0020509	NA	FBtr0072943
FBgn0020510	FBgn0020510	Abi	FBtr0307077
FBgn0020513	FBgn0020513	ade5	FBtr0073705
FBgn0020521	FBgn0020521	pio	FBtr0301502
FBgn0020545	FBgn0020545	kraken	FBtr0078016
FBgn0020611	FBgn0020611	NA	FBtr0290031
FBgn0020620	FBgn0020620	RN-tre	FBtr0087604
FBgn0020621	FBgn0020621	Pkn	FBtr0112897
FBgn0020623	FBgn0020623	NA	FBtr0089500
FBgn0020626	FBgn0020626	Osbp	FBtr0084801
FBgn0020633	FBgn0020633	Mcm7	FBtr0076585
FBgn0020641	FBgn0020641	NA	FBtr0076990
FBgn0020642	FBgn0020642	Lcp65Ac	FBtr0076991
FBgn0020643	FBgn0020643	NA	FBtr0076992
FBgn0020644	FBgn0020644	NA	FBtr0076994
FBgn0020645	FBgn0020645	NA	FBtr0076995
FBgn0020647	FBgn0020647	KrT95D	FBtr0084507
FBgn0020762	FBgn0020762	Atet	FBtr0112899
FBgn0020764	FBgn0020764	Alas	FBtr0072151
FBgn0020765	FBgn0020765	Acp65Aa	FBtr0077021
FBgn0020766	FBgn0020766	Aats-phe	FBtr0087594
FBgn0020767	FBgn0020767	NA	FBtr0087468
FBgn0020880	FBgn0020880	CG7179	FBtr0301151
FBgn0020907	FBgn0020907	NA	FBtr0083361
FBgn0020908	FBgn0020908	NA	FBtr0113713
FBgn0020909	FBgn0020909	NA	FBtr0073820
FBgn0020910	FBgn0020910	RpL3	FBtr0302586
FBgn0020912	FBgn0020912	Ptx1	FBtr0089391
FBgn0021738	FBgn0021738	NA	FBtr0070599
FBgn0021764	FBgn0021764	NA	FBtr0302259
FBgn0021765	FBgn0021765	NA	FBtr0308351
FBgn0021768	FBgn0021768	nudC	FBtr0305912
FBgn0021795	FBgn0021795	Tapdelta	FBtr0088153
FBgn0021814	FBgn0021814	Vps28	FBtr0310370
FBgn0021825	FBgn0021825	Dmn	FBtr0088641
FBgn0021847	FBgn0021847	NA	FBtr0086000
FBgn0021872	FBgn0021872	Xbp1	FBtr0310044
FBgn0021873	FBgn0021873	NA	FBtr0300038
FBgn0021874	FBgn0021874	Nle	FBtr0078075
FBgn0021906	FBgn0021906	RFeSP	FBtr0077914
FBgn0021944	FBgn0021944	Coprox	FBtr0079352
FBgn0021953	FBgn0021953	Fatp	FBtr0080134
FBgn0022023	FBgn0022023	eIF-3p40	FBtr0079030
FBgn0022027	FBgn0022027	NA	FBtr0088694

FBgn0022063	FBgn0022063	NA	FBtr0302294
FBgn0022070	FBgn0022070	NA	FBtr0087170
FBgn0022073	FBgn0022073	NA	FBtr0077524
FBgn0022085	FBgn0022085	NA	FBtr0273446
FBgn0022097	FBgn0022097	Vha36	FBtr0087335
FBgn0022153	FBgn0022153	l(2)k05819	FBtr0077432
FBgn0022160	FBgn0022160	l(2)k05713	FBtr0087309
FBgn0022224	FBgn0022224	ubl	FBtr0086211
FBgn0022238	FBgn0022238	NA	FBtr0300483
FBgn0022246	FBgn0022246	NA	FBtr0078143
FBgn0022268	FBgn0022268	KdelR	FBtr0080023
FBgn0022338	FBgn0022338	dnk	FBtr0321263
FBgn0022343	FBgn0022343	CG3760	FBtr0072391
FBgn0022359	FBgn0022359	Sodh-2	FBtr0082324
FBgn0022361	FBgn0022361	NA	FBtr0310638
FBgn0022382	FBgn0022382	NA	FBtr0088438
FBgn0022699	FBgn0022699	NA	FBtr0077017
FBgn0022702	FBgn0022702	Cht2	FBtr0305966
FBgn0022720	FBgn0022720	zf30C	FBtr0079865
FBgn0022724	FBgn0022724	NA	FBtr0074514
FBgn0022764	FBgn0022764	NA	FBtr0087888
FBgn0022770	FBgn0022770	NA	FBtr0308243
FBgn0022787	FBgn0022787	Hel89B	FBtr0301715
FBgn0022893	FBgn0022893	NA	FBtr0100293
FBgn0022935	FBgn0022935	NA	FBtr0077016
FBgn0022943	FBgn0022943	NA	FBtr0083551
FBgn0022959	FBgn0022959	NA	FBtr0305992
FBgn0022981	FBgn0022981	NA	FBtr0078859
FBgn0022984	FBgn0022984	NA	FBtr0307214
FBgn0022986	FBgn0022986	qkr58E-1	FBtr0071843
FBgn0022987	FBgn0022987	NA	FBtr0306248
FBgn0023000	FBgn0023000	NA	FBtr0306850
FBgn0023001	FBgn0023001	NA	FBtr0302044
FBgn0023023	FBgn0023023	CRMP	FBtr0089684
FBgn0023076	FBgn0023076	NA	FBtr0100132
FBgn0023083	FBgn0023083	NA	FBtr0083637
FBgn0023091	FBgn0023091	NA	FBtr0081514
FBgn0023094	FBgn0023094	NA	FBtr0074924
FBgn0023095	FBgn0023095	NA	FBtr0075832
FBgn0023096	FBgn0023096	NA	FBtr0273418
FBgn0023097	FBgn0023097	NA	FBtr0083912
FBgn0023130	FBgn0023130	NA	FBtr0070279
FBgn0023143	FBgn0023143	Uba1	FBtr0088499
FBgn0023167	FBgn0023167	NA	FBtr0087986
FBgn0023170	FBgn0023170	NA	FBtr0072185
FBgn0023171	FBgn0023171	NA	FBtr0088883
FBgn0023172	FBgn0023172	NA	FBtr0086970
FBgn0023174	FBgn0023174	Prosbeta2	FBtr0075629
FBgn0023175	FBgn0023175	NA	FBtr0089998
FBgn0023178	FBgn0023178	Pdf	FBtr0085024
FBgn0023180	FBgn0023180	Orc6	FBtr0088482
FBgn0023181	FBgn0023181	Orc4	FBtr0306317
FBgn0023197	FBgn0023197	NA	FBtr0075207
FBgn0023211	FBgn0023211	Elongin-C	FBtr0086541
FBgn0023212	FBgn0023212	NA	FBtr0083947
FBgn0023213	FBgn0023213	NA	FBtr0112904
FBgn0023215	FBgn0023215	Mnt	FBtr0307277

FBgn0023216	FBgn0023216	NA	FBtr0307280
FBgn0023388	FBgn0023388	Dap160	FBtr0306019
FBgn0023395	FBgn0023395	NA	FBtr0074998
FBgn0023416	FBgn0023416	NA	FBtr0085897
FBgn0023441	FBgn0023441	fus	FBtr0087346
FBgn0023444	FBgn0023444	NA	FBtr0078055
FBgn0023458	FBgn0023458	NA	FBtr0303341
FBgn0023477	FBgn0023477	Tal	FBtr0072141
FBgn0023479	FBgn0023479	NA	FBtr0076526
FBgn0023489	FBgn0023489	Pph13	FBtr0078070
FBgn0023495	FBgn0023495	NA	FBtr0082771
FBgn0023496	FBgn0023496	Lip1	FBtr0080124
FBgn0023506	FBgn0023506	NA	FBtr0071198
FBgn0023509	FBgn0023509	NA	FBtr0070315
FBgn0023510	FBgn0023510	NA	FBtr0070316
FBgn0023511	FBgn0023511	NA	FBtr0070352
FBgn0023512	FBgn0023512	NA	FBtr0070351
FBgn0023515	FBgn0023515	NA	FBtr0070369
FBgn0023516	FBgn0023516	NA	FBtr0070367
FBgn0023520	FBgn0023520	NA	FBtr0070349
FBgn0023521	FBgn0023521	CG3587	FBtr0070321
FBgn0023522	FBgn0023522	CG11596	FBtr0070318
FBgn0023525	FBgn0023525	CG3191	FBtr0305504
FBgn0023527	FBgn0023527	NA	FBtr0070398
FBgn0023528	FBgn0023528	CG2924	FBtr0070412
FBgn0023529	FBgn0023529	CG2918	FBtr0070411
FBgn0023530	FBgn0023530	NA	FBtr0070283
FBgn0023531	FBgn0023531	NA	FBtr0308324
FBgn0023535	FBgn0023535	arg	FBtr0070088
FBgn0023540	FBgn0023540	CG3630	FBtr0070385
FBgn0023541	FBgn0023541	NA	FBtr0310077
FBgn0023542	FBgn0023542	NA	FBtr0070425
FBgn0023545	FBgn0023545	CG4406	FBtr0070347
FBgn0023550	FBgn0023550	CG18031	FBtr0070423
FBgn0024150	FBgn0024150	Ac78C	FBtr0301701
FBgn0024187	FBgn0024187	syd	FBtr0300412
FBgn0024188	FBgn0024188	NA	FBtr0088126
FBgn0024189	FBgn0024189	NA	FBtr0111043
FBgn0024191	FBgn0024191	sip1	FBtr0079134
FBgn0024196	FBgn0024196	NA	FBtr0086920
FBgn0024222	FBgn0024222	NA	FBtr0304804
FBgn0024242	FBgn0024242	NA	FBtr0110919
FBgn0024248	FBgn0024248	NA	FBtr0080088
FBgn0024250	FBgn0024250	NA	FBtr0071048
FBgn0024252	FBgn0024252	NA	FBtr0086968
FBgn0024277	FBgn0024277	NA	FBtr0072645
FBgn0024285	FBgn0024285	Srp54	FBtr0079875
FBgn0024289	FBgn0024289	NA	FBtr0081627
FBgn0024290	FBgn0024290	NA	FBtr0079517
FBgn0024314	FBgn0024314	NA	FBtr0077980
FBgn0024320	FBgn0024320	NA	FBtr0079957
FBgn0024321	FBgn0024321	NK7.1	FBtr0306742
FBgn0024326	FBgn0024326	NA	FBtr0081892
FBgn0024329	FBgn0024329	NA	FBtr0301775
FBgn0024330	FBgn0024330	NA	FBtr0301873
FBgn0024332	FBgn0024332	Mcm3	FBtr0070762
FBgn0024360	FBgn0024360	NA	FBtr0070236

FBgn0024361	FBgn0024361	Tsp2A	FBtr0070211
FBgn0024362	FBgn0024362	NA	FBtr0070210
FBgn0024366	FBgn0024366	NA	FBtr0070238
FBgn0024371	FBgn0024371	E2f2	FBtr0081501
FBgn0024509	FBgn0024509	sec13	FBtr0084409
FBgn0024555	FBgn0024555	NA	FBtr0082817
FBgn0024556	FBgn0024556	NA	FBtr0301389
FBgn0024689	FBgn0024689	fws	FBtr0305686
FBgn0024698	FBgn0024698	NA	FBtr0089258
FBgn0024728	FBgn0024728	NA	FBtr0089090
FBgn0024733	FBgn0024733	NA	FBtr0299870
FBgn0024740	FBgn0024740	NA	FBtr0080123
FBgn0024753	FBgn0024753	NA	FBtr0073951
FBgn0024806	FBgn0024806	NA	FBtr0072520
FBgn0024807	FBgn0024807	NA	FBtr0300373
FBgn0024811	FBgn0024811	NA	FBtr0304871
FBgn0024814	FBgn0024814	NA	FBtr0301126
FBgn0024836	FBgn0024836	stan	FBtr0304899
FBgn0024841	FBgn0024841	Pcd	FBtr0085458
FBgn0024846	FBgn0024846	p38b	FBtr0080534
FBgn0024887	FBgn0024887	NA	FBtr0078181
FBgn0024891	FBgn0024891	ferrochelatase	FBtr0085850
FBgn0024897	FBgn0024897	NA	FBtr0070272
FBgn0024909	FBgn0024909	NA	FBtr0081756
FBgn0024912	FBgn0024912	agt	FBtr0081697
FBgn0024920	FBgn0024920	Ts	FBtr0077679
FBgn0024923	FBgn0024923	NA	FBtr0112906
FBgn0024941	FBgn0024941	NA	FBtr0307538
FBgn0024943	FBgn0024943	NA	FBtr0071235
FBgn0024944	FBgn0024944	NA	FBtr0083932
FBgn0024945	FBgn0024945	NitFhit	FBtr0072483
FBgn0024947	FBgn0024947	NTPase	FBtr0077682
FBgn0024956	FBgn0024956	Mat1	FBtr0088255
FBgn0024963	FBgn0024963	NA	FBtr0083787
FBgn0024973	FBgn0024973	NA	FBtr0070498
FBgn0024983	FBgn0024983	NA	FBtr0100476
FBgn0024987	FBgn0024987	NA	FBtr0070215
FBgn0024991	FBgn0024991	NA	FBtr0070482
FBgn0024994	FBgn0024994	NA	FBtr0070501
FBgn0024995	FBgn0024995	NA	FBtr0273279
FBgn0024996	FBgn0024996	NA	FBtr0070479
FBgn0024998	FBgn0024998	NA	FBtr0070499
FBgn0025115	FBgn0025115	NA	FBtr0080555
FBgn0025117	FBgn0025117	NA	FBtr0079866
FBgn0025140	FBgn0025140	NA	FBtr0084174
FBgn0025335	FBgn0025335	NA	FBtr0072227
FBgn0025355	FBgn0025355	NA	FBtr0076203
FBgn0025373	FBgn0025373	NA	FBtr0088170
FBgn0025378	FBgn0025378	NA	FBtr0070278
FBgn0025382	FBgn0025382	NA	FBtr0070294
FBgn0025383	FBgn0025383	NA	FBtr0070257
FBgn0025387	FBgn0025387	NA	FBtr0070696
FBgn0025388	FBgn0025388	NA	FBtr0300755
FBgn0025390	FBgn0025390	NA	FBtr0070284
FBgn0025391	FBgn0025391	NA	FBtr0302175
FBgn0025393	FBgn0025393	NA	FBtr0070285
FBgn0025454	FBgn0025454	NA	FBtr0087992

FBgn0025456	FBgn0025456	NA	FBtr0089897
FBgn0025457	FBgn0025457	Bub3	FBtr0085465
FBgn0025463	FBgn0025463	Bap60	FBtr0073741
FBgn0025469	FBgn0025469	slv	FBtr0310369
FBgn0025519	FBgn0025519	NA	FBtr0087185
FBgn0025549	FBgn0025549	NA	FBtr0071066
FBgn0025558	FBgn0025558	NA	FBtr0075314
FBgn0025574	FBgn0025574	NA	FBtr0112908
FBgn0025582	FBgn0025582	Int6	FBtr0075345
FBgn0025583	FBgn0025583	NA	FBtr0086664
FBgn0025592	FBgn0025592	Gyk	FBtr0072529
FBgn0025595	FBgn0025595	NA	FBtr0079309
FBgn0025616	FBgn0025616	NA	FBtr0070145
FBgn0025625	FBgn0025625	NA	FBtr0070340
FBgn0025626	FBgn0025626	NA	FBtr0070339
FBgn0025627	FBgn0025627	CG4194	FBtr0070328
FBgn0025628	FBgn0025628	CG4199	FBtr0300255
FBgn0025629	FBgn0025629	NA	FBtr0070331
FBgn0025630	FBgn0025630	CG4061	FBtr0070330
FBgn0025631	FBgn0025631	CG4322	FBtr0303043
FBgn0025632	FBgn0025632	CG4313	FBtr0070327
FBgn0025633	FBgn0025633	NA	FBtr0110791
FBgn0025634	FBgn0025634	NA	FBtr0070121
FBgn0025635	FBgn0025635	CG17829	FBtr0070116
FBgn0025637	FBgn0025637	NA	FBtr0070124
FBgn0025638	FBgn0025638	NA	FBtr0308651
FBgn0025639	FBgn0025639	NA	FBtr0070151
FBgn0025640	FBgn0025640	NA	FBtr0070115
FBgn0025641	FBgn0025641	NA	FBtr0310282
FBgn0025642	FBgn0025642	NA	FBtr0070206
FBgn0025643	FBgn0025643	NA	FBtr0301058
FBgn0025645	FBgn0025645	NA	FBtr0070552
FBgn0025674	FBgn0025674	NA	FBtr0085941
FBgn0025678	FBgn0025678	CaBP1	FBtr0080837
FBgn0025681	FBgn0025681	NA	FBtr0273230
FBgn0025683	FBgn0025683	CG3164	FBtr0100379
FBgn0025692	FBgn0025692	CG3814	FBtr0087814
FBgn0025700	FBgn0025700	NA	FBtr0079900
FBgn0025701	FBgn0025701	NA	FBtr0302027
FBgn0025702	FBgn0025702	NA	FBtr0306595
FBgn0025709	FBgn0025709	CG8083	FBtr0088560
FBgn0025724	FBgn0025724	NA	FBtr0080469
FBgn0025725	FBgn0025725	alphaCop	FBtr0072812
FBgn0025726	FBgn0025726	NA	FBtr0308805
FBgn0025739	FBgn0025739	pon	FBtr0070681
FBgn0025740	FBgn0025740	NA	FBtr0308297
FBgn0025741	FBgn0025741	NA	FBtr0309865
FBgn0025742	FBgn0025742	NA	FBtr0079223
FBgn0025743	FBgn0025743	NA	FBtr0074285
FBgn0025777	FBgn0025777	homer	FBtr0079306
FBgn0025790	FBgn0025790	TBPH	FBtr0089625
FBgn0025800	FBgn0025800	NA	FBtr0071192
FBgn0025803	FBgn0025803	NA	FBtr0084020
FBgn0025806	FBgn0025806	NA	FBtr0072177
FBgn0025807	FBgn0025807	NA	FBtr0075080
FBgn0025808	FBgn0025808	NA	FBtr0082974
FBgn0025809	FBgn0025809	NA	FBtr0074190

FBgn0025814	FBgn0025814	NA	FBtr0077243
FBgn0025820	FBgn0025820	NA	FBtr0072788
FBgn0025825	FBgn0025825	Hdac3	FBtr0078767
FBgn0025835	FBgn0025835	NA	FBtr0300673
FBgn0025836	FBgn0025836	NA	FBtr0112919
FBgn0025837	FBgn0025837	NA	FBtr0307095
FBgn0025839	FBgn0025839	NA	FBtr0070386
FBgn0025864	FBgn0025864	NA	FBtr0071219
FBgn0025865	FBgn0025865	Cortactin	FBtr0084039
FBgn0025866	FBgn0025866	CalpB	FBtr0076343
FBgn0025879	FBgn0025879	Timp	FBtr0082245
FBgn0025885	FBgn0025885	NA	FBtr0089329
FBgn0025936	FBgn0025936	NA	FBtr0089084
FBgn0026015	FBgn0026015	NA	FBtr0070930
FBgn0026056	FBgn0026056	NA	FBtr0084002
FBgn0026059	FBgn0026059	Mhcl	FBtr0083231
FBgn0026061	FBgn0026061	Mipp1	FBtr0075399
FBgn0026063	FBgn0026063	NA	FBtr0301427
FBgn0026064	FBgn0026064	NA	FBtr0301426
FBgn0026076	FBgn0026076	NA	FBtr0074174
FBgn0026077	FBgn0026077	Gasp	FBtr0078619
FBgn0026079	FBgn0026079	NA	FBtr0070669
FBgn0026080	FBgn0026080	NA	FBtr0070668
FBgn0026083	FBgn0026083	NA	FBtr0305180
FBgn0026084	FBgn0026084	cib	FBtr0307206
FBgn0026086	FBgn0026086	NA	FBtr0305499
FBgn0026090	FBgn0026090	NA	FBtr0070372
FBgn0026141	FBgn0026141	NA	FBtr0077924
FBgn0026143	FBgn0026143	NA	FBtr0070182
FBgn0026144	FBgn0026144	NA	FBtr0071052
FBgn0026147	FBgn0026147	NA	FBtr0304953
FBgn0026148	FBgn0026148	NA	FBtr0080149
FBgn0026149	FBgn0026149	NA	FBtr0071358
FBgn0026150	FBgn0026150	ApepP	FBtr0304931
FBgn0026160	FBgn0026160	NA	FBtr0076267
FBgn0026176	FBgn0026176	NA	FBtr0088020
FBgn0026179	FBgn0026179	siz	FBtr0078290
FBgn0026188	FBgn0026188	NA	FBtr0111030
FBgn0026189	FBgn0026189	NA	FBtr0073118
FBgn0026190	FBgn0026190	NA	FBtr0085689
FBgn0026192	FBgn0026192	NA	FBtr0074455
FBgn0026196	FBgn0026196	nop5	FBtr0079369
FBgn0026197	FBgn0026197	NA	FBtr0110845
FBgn0026207	FBgn0026207	NA	FBtr0082668
FBgn0026208	FBgn0026208	NA	FBtr0075394
FBgn0026238	FBgn0026238	NA	FBtr0309848
FBgn0026239	FBgn0026239	gukh	FBtr0112924
FBgn0026255	FBgn0026255	NA	FBtr0081476
FBgn0026257	FBgn0026257	cav	FBtr0084556
FBgn0026259	FBgn0026259	NA	FBtr0309839
FBgn0026263	FBgn0026263	NA	FBtr0076745
FBgn0026313	FBgn0026313	NA	FBtr0301696
FBgn0026315	FBgn0026315	Ugt35a	FBtr0082374
FBgn0026317	FBgn0026317	Tsc1	FBtr0084546
FBgn0026318	FBgn0026318	NA	FBtr0071188
FBgn0026319	FBgn0026319	Traf1	FBtr0077429
FBgn0026324	FBgn0026324	NA	FBtr0077735

FBgn0026326	FBgn0026326	TXBP181-like	FBtr0088599
FBgn0026361	FBgn0026361	Sep5	FBtr0088877
FBgn0026370	FBgn0026370	NA	FBtr0087384
FBgn0026371	FBgn0026371	NA	FBtr0078387
FBgn0026372	FBgn0026372	NA	FBtr0072805
FBgn0026373	FBgn0026373	RpII33	FBtr0080544
FBgn0026379	FBgn0026379	Pten	FBtr0300562
FBgn0026380	FBgn0026380	NA	FBtr0082010
FBgn0026388	FBgn0026388	NA	FBtr0088330
FBgn0026393	FBgn0026393	NA	FBtr0088887
FBgn0026394	FBgn0026394	NA	FBtr0289965
FBgn0026399	FBgn0026399	NA	FBtr0310300
FBgn0026401	FBgn0026401	NA	FBtr0111119
FBgn0026402	FBgn0026402	NiPp1	FBtr0087058
FBgn0026404	FBgn0026404	NA	FBtr0076347
FBgn0026409	FBgn0026409	Mpcp	FBtr0308595
FBgn0026415	FBgn0026415	Idgf4	FBtr0071394
FBgn0026417	FBgn0026417	Hus1-like	FBtr0078876
FBgn0026418	FBgn0026418	Hsc70Cb	FBtr0304036
FBgn0026427	FBgn0026427	NA	FBtr0087501
FBgn0026428	FBgn0026428	NA	FBtr0074008
FBgn0026430	FBgn0026430	Grip84	FBtr0301307
FBgn0026431	FBgn0026431	Grip75	FBtr0080033
FBgn0026433	FBgn0026433	NA	FBtr0100271
FBgn0026438	FBgn0026438	NA	FBtr0301316
FBgn0026439	FBgn0026439	NA	FBtr0079801
FBgn0026562	FBgn0026562	NA	FBtr0085094
FBgn0026563	FBgn0026563	NA	FBtr0081628
FBgn0026565	FBgn0026565	NA	FBtr0081699
FBgn0026566	FBgn0026566	CG1307	FBtr0301623
FBgn0026567	FBgn0026567	NA	FBtr0081687
FBgn0026570	FBgn0026570	NA	FBtr0072915
FBgn0026575	FBgn0026575	NA	FBtr0290069
FBgn0026582	FBgn0026582	CG9418	FBtr0071573
FBgn0026585	FBgn0026585	NA	FBtr0079290
FBgn0026592	FBgn0026592	NA	FBtr0073157
FBgn0026593	FBgn0026593	CG5707	FBtr0308231
FBgn0026597	FBgn0026597	NA	FBtr0085553
FBgn0026602	FBgn0026602	NA	FBtr0089050
FBgn0026611	FBgn0026611	NA	FBtr0305592
FBgn0026619	FBgn0026619	tafazzin	FBtr0087862
FBgn0026620	FBgn0026620	NA	FBtr0302616
FBgn0026702	FBgn0026702	NA	FBtr0070782
FBgn0026718	FBgn0026718	fu12	FBtr0302160
FBgn0026737	FBgn0026737	NA	FBtr0083113
FBgn0026738	FBgn0026738	NA	FBtr0075605
FBgn0026741	FBgn0026741	NA	FBtr0087852
FBgn0026749	FBgn0026749	NA	FBtr0100168
FBgn0026751	FBgn0026751	NA	FBtr0070763
FBgn0026753	FBgn0026753	NA	FBtr0083804
FBgn0026758	FBgn0026758	NA	FBtr0112927
FBgn0026761	FBgn0026761	NA	FBtr0086149
FBgn0026778	FBgn0026778	NA	FBtr0077747
FBgn0026781	FBgn0026781	NA	FBtr0088895
FBgn0026787	FBgn0026787	NA	FBtr0301452
FBgn0026871	FBgn0026871	NA	FBtr0070258
FBgn0026872	FBgn0026872	NA	FBtr0070253

FBgn0026873	FBgn0026873	NA	FBtr0114454
FBgn0026874	FBgn0026874	NA	FBtr0305568
FBgn0026876	FBgn0026876	CG11403	FBtr0070245
FBgn0026878	FBgn0026878	NA	FBtr0310443
FBgn0027053	FBgn0027053	NA	FBtr0083292
FBgn0027054	FBgn0027054	NA	FBtr0088859
FBgn0027066	FBgn0027066	Eb1	FBtr0300285
FBgn0027070	FBgn0027070	NA	FBtr0081106
FBgn0027073	FBgn0027073	NA	FBtr0071625
FBgn0027074	FBgn0027074	CG17324	FBtr0300818
FBgn0027080	FBgn0027080	Aats-tyr	FBtr0075410
FBgn0027081	FBgn0027081	Aats-thr	FBtr0080332
FBgn0027082	FBgn0027082	NA	FBtr0072948
FBgn0027084	FBgn0027084	Aats-lys	FBtr0303560
FBgn0027086	FBgn0027086	Aats-ile	FBtr0078498
FBgn0027090	FBgn0027090	NA	FBtr0084866
FBgn0027094	FBgn0027094	Aats-ala	FBtr0079722
FBgn0027095	FBgn0027095	ARP-like	FBtr0083283
FBgn0027103	FBgn0027103	NA	FBtr0084547
FBgn0027106	FBgn0027106	inx7	FBtr0071034
FBgn0027108	FBgn0027108	NA	FBtr0071005
FBgn0027109	FBgn0027109	NA	FBtr0083328
FBgn0027111	FBgn0027111	miple	FBtr0072504
FBgn0027279	FBgn0027279	l(1)G0196	FBtr0299526
FBgn0027287	FBgn0027287	NA	FBtr0074042
FBgn0027291	FBgn0027291	l(1)G0156	FBtr0307904
FBgn0027329	FBgn0027329	l(1)G0022	FBtr0074086
FBgn0027334	FBgn0027334	l(1)G0004	FBtr0077298
FBgn0027335	FBgn0027335	NA	FBtr0074616
FBgn0027338	FBgn0027338	Kap-alpha3	FBtr0082106
FBgn0027339	FBgn0027339	NA	FBtr0078582
FBgn0027341	FBgn0027341	NA	FBtr0302847
FBgn0027343	FBgn0027343	NA	FBtr0070138
FBgn0027348	FBgn0027348	bgm	FBtr0080590
FBgn0027359	FBgn0027359	NA	FBtr0073482
FBgn0027360	FBgn0027360	Tim10	FBtr0071676
FBgn0027375	FBgn0027375	NA	FBtr0075714
FBgn0027376	FBgn0027376	NA	FBtr0084832
FBgn0027490	FBgn0027490	NA	FBtr0079707
FBgn0027492	FBgn0027492	NA	FBtr0085207
FBgn0027493	FBgn0027493	NA	FBtr0310504
FBgn0027494	FBgn0027494	NA	FBtr0085194
FBgn0027500	FBgn0027500	NA	FBtr0075363
FBgn0027505	FBgn0027505	NA	FBtr0080301
FBgn0027508	FBgn0027508	NA	FBtr0084890
FBgn0027514	FBgn0027514	NA	FBtr0290212
FBgn0027515	FBgn0027515	CG7115	FBtr0079523
FBgn0027518	FBgn0027518	NA	FBtr0085543
FBgn0027521	FBgn0027521	NA	FBtr0074222
FBgn0027524	FBgn0027524	CG3909	FBtr0082172
FBgn0027525	FBgn0027525	CG7686	FBtr0088245
FBgn0027526	FBgn0027526	NA	FBtr0084347
FBgn0027529	FBgn0027529	CG8920	FBtr0086280
FBgn0027532	FBgn0027532	NA	FBtr0078486
FBgn0027534	FBgn0027534	NA	FBtr0075929
FBgn0027537	FBgn0027537	NA	FBtr0073828
FBgn0027538	FBgn0027538	NA	FBtr0087539

FBgn0027544	FBgn0027544	NA	FBtr0303200
FBgn0027547	FBgn0027547	CG1927	FBtr0072853
FBgn0027552	FBgn0027552	CG10863	FBtr0073171
FBgn0027559	FBgn0027559	NA	FBtr0303164
FBgn0027561	FBgn0027561	CG18659	FBtr0308824
FBgn0027562	FBgn0027562	NA	FBtr0083360
FBgn0027563	FBgn0027563	NA	FBtr0082898
FBgn0027564	FBgn0027564	NA	FBtr0070846
FBgn0027565	FBgn0027565	NA	FBtr0078238
FBgn0027567	FBgn0027567	NA	FBtr0076339
FBgn0027570	FBgn0027570	Nep2	FBtr0305133
FBgn0027571	FBgn0027571	NA	FBtr0290093
FBgn0027578	FBgn0027578	NA	FBtr0301945
FBgn0027579	FBgn0027579	CG5508	FBtr0308501
FBgn0027580	FBgn0027580	NA	FBtr0088458
FBgn0027581	FBgn0027581	NA	FBtr0087678
FBgn0027583	FBgn0027583	NA	FBtr0085544
FBgn0027585	FBgn0027585	CG8740	FBtr0290115
FBgn0027587	FBgn0027587	CG7028	FBtr0072528
FBgn0027588	FBgn0027588	NA	FBtr0070061
FBgn0027590	FBgn0027590	NA	FBtr0305310
FBgn0027592	FBgn0027592	NA	FBtr0078062
FBgn0027594	FBgn0027594	drpr	FBtr0072799
FBgn0027596	FBgn0027596	CG10249	FBtr0303757
FBgn0027598	FBgn0027598	NA	FBtr0310424
FBgn0027599	FBgn0027599	NA	FBtr0072409
FBgn0027601	FBgn0027601	CG9009	FBtr0303294
FBgn0027605	FBgn0027605	NA	FBtr0074504
FBgn0027607	FBgn0027607	NA	FBtr0088674
FBgn0027608	FBgn0027608	NA	FBtr0300910
FBgn0027609	FBgn0027609	NA	FBtr0077457
FBgn0027610	FBgn0027610	NA	FBtr0082747
FBgn0027617	FBgn0027617	NA	FBtr0084738
FBgn0027621	FBgn0027621	NA	FBtr0307194
FBgn0027657	FBgn0027657	NA	FBtr0083247
FBgn0027780	FBgn0027780	NA	FBtr0089673
FBgn0027784	FBgn0027784	Prp18	FBtr0083717
FBgn0027785	FBgn0027785	NA	FBtr0083712
FBgn0027786	FBgn0027786	Mtch	FBtr0305543
FBgn0027790	FBgn0027790	GV1	FBtr0072784
FBgn0027791	FBgn0027791	NA	FBtr0070296
FBgn0027793	FBgn0027793	CG14787	FBtr0070298
FBgn0027794	FBgn0027794	CG14786	FBtr0070230
FBgn0027795	FBgn0027795	NA	FBtr0070231
FBgn0027796	FBgn0027796	NA	FBtr0300224
FBgn0027842	FBgn0027842	NA	FBtr0301282
FBgn0027844	FBgn0027844	NA	FBtr0080539
FBgn0027864	FBgn0027864	NA	FBtr0071223
FBgn0027868	FBgn0027868	NA	FBtr0080121
FBgn0027872	FBgn0027872	NA	FBtr0086888
FBgn0027873	FBgn0027873	CG1957	FBtr0085357
FBgn0027885	FBgn0027885	Aac11	FBtr0081001
FBgn0027929	FBgn0027929	CG33119	FBtr0310074
FBgn0027930	FBgn0027930	NA	FBtr0300936
FBgn0027934	FBgn0027934	NA	FBtr0090038
FBgn0027936	FBgn0027936	NA	FBtr0075958
FBgn0027945	FBgn0027945	ppl	FBtr0078412

FBgn0027948	FBgn0027948	NA	FBtr0304801
FBgn0027949	FBgn0027949	msb1l	FBtr0081258
FBgn0027951	FBgn0027951	NA	FBtr0290206
FBgn0028274	FBgn0028274	NA	FBtr0070297
FBgn0028292	FBgn0028292	ric8a	FBtr0071317
FBgn0028341	FBgn0028341	NA	FBtr0303558
FBgn0028342	FBgn0028342	NA	FBtr0071438
FBgn0028343	FBgn0028343	NA	FBtr0301414
FBgn0028369	FBgn0028369	NA	FBtr0070559
FBgn0028370	FBgn0028370	NA	FBtr0080752
FBgn0028371	FBgn0028371	jbug	FBtr0301374
FBgn0028374	FBgn0028374	hug	FBtr0082639
FBgn0028375	FBgn0028375	NA	FBtr0080852
FBgn0028377	FBgn0028377	NA	FBtr0091756
FBgn0028379	FBgn0028379	NA	FBtr0076683
FBgn0028380	FBgn0028380	fal	FBtr0321324
FBgn0028381	FBgn0028381	decay	FBtr0083304
FBgn0028382	FBgn0028382	cyp33	FBtr0086945
FBgn0028387	FBgn0028387	chm	FBtr0079443
FBgn0028397	FBgn0028397	NA	FBtr0301652
FBgn0028398	FBgn0028398	Taf10	FBtr0077717
FBgn0028399	FBgn0028399	TMS1	FBtr0075389
FBgn0028400	FBgn0028400	SytIV	FBtr0081578
FBgn0028401	FBgn0028401	NA	FBtr0082163
FBgn0028402	FBgn0028402	NA	FBtr0304820
FBgn0028406	FBgn0028406	NA	FBtr0080478
FBgn0028408	FBgn0028408	Rep2	FBtr0088597
FBgn0028410	FBgn0028410	NA	FBtr0080432
FBgn0028412	FBgn0028412	NA	FBtr0080271
FBgn0028415	FBgn0028415	NA	FBtr0075116
FBgn0028416	FBgn0028416	NA	FBtr0075115
FBgn0028418	FBgn0028418	NA	FBtr0075722
FBgn0028419	FBgn0028419	NA	FBtr0079192
FBgn0028420	FBgn0028420	Kr-h1	FBtr0079194
FBgn0028421	FBgn0028421	NA	FBtr0289956
FBgn0028424	FBgn0028424	NA	FBtr0087139
FBgn0028425	FBgn0028425	JhI-21	FBtr0089656
FBgn0028426	FBgn0028426	NA	FBtr0088360
FBgn0028427	FBgn0028427	Ilk	FBtr0078305
FBgn0028428	FBgn0028428	NA	FBtr0100372
FBgn0028429	FBgn0028429	NA	FBtr0112935
FBgn0028430	FBgn0028430	NA	FBtr0080616
FBgn0028431	FBgn0028431	NA	FBtr0076431
FBgn0028433	FBgn0028433	NA	FBtr0079797
FBgn0028467	FBgn0028467	NA	FBtr0079297
FBgn0028471	FBgn0028471	NA	FBtr0084633
FBgn0028473	FBgn0028473	NA	FBtr0088554
FBgn0028475	FBgn0028475	CG10221	FBtr0321255
FBgn0028476	FBgn0028476	NA	FBtr0300833
FBgn0028481	FBgn0028481	NA	FBtr0077949
FBgn0028484	FBgn0028484	Ack	FBtr0073211
FBgn0028487	FBgn0028487	NA	FBtr0112937
FBgn0028490	FBgn0028490	CG31705	FBtr0080243
FBgn0028491	FBgn0028491	NA	FBtr0070610
FBgn0028494	FBgn0028494	NA	FBtr0086886
FBgn0028497	FBgn0028497	CG3530	FBtr0112940
FBgn0028504	FBgn0028504	NA	FBtr0072955

FBgn0028506	FBgn0028506	CG4455	FBtr0080836
FBgn0028507	FBgn0028507	CG3793	FBtr0305560
FBgn0028513	FBgn0028513	NA	FBtr0080512
FBgn0028514	FBgn0028514	NA	FBtr0300612
FBgn0028516	FBgn0028516	CG3994	FBtr0080714
FBgn0028518	FBgn0028518	NA	FBtr0080755
FBgn0028519	FBgn0028519	NA	FBtr0080591
FBgn0028526	FBgn0028526	NA	FBtr0080603
FBgn0028528	FBgn0028528	NA	FBtr0111015
FBgn0028529	FBgn0028529	NA	FBtr0080639
FBgn0028530	FBgn0028530	NA	FBtr0080641
FBgn0028533	FBgn0028533	NA	FBtr0080550
FBgn0028534	FBgn0028534	NA	FBtr0080549
FBgn0028537	FBgn0028537	NA	FBtr0080691
FBgn0028539	FBgn0028539	CG31731	FBtr0110941
FBgn0028541	FBgn0028541	NA	FBtr0080532
FBgn0028542	FBgn0028542	NA	FBtr0080617
FBgn0028543	FBgn0028543	NA	FBtr0080618
FBgn0028544	FBgn0028544	NA	FBtr0080557
FBgn0028546	FBgn0028546	Rsu-1	FBtr0080511
FBgn0028550	FBgn0028550	NA	FBtr0070244
FBgn0028552	FBgn0028552	NA	FBtr0072187
FBgn0028560	FBgn0028560	NA	FBtr0089674
FBgn0028563	FBgn0028563	sut1	FBtr0088777
FBgn0028569	FBgn0028569	NA	FBtr0081139
FBgn0028570	FBgn0028570	NA	FBtr0077800
FBgn0028572	FBgn0028572	qtc	FBtr0079012
FBgn0028577	FBgn0028577	pUf68	FBtr0300416
FBgn0028581	FBgn0028581	NA	FBtr0303848
FBgn0028582	FBgn0028582	NA	FBtr0076798
FBgn0028642	FBgn0028642	NA	FBtr0089038
FBgn0028644	FBgn0028644	NA	FBtr0080801
FBgn0028645	FBgn0028645	NA	FBtr0300615
FBgn0028646	FBgn0028646	NA	FBtr0085693
FBgn0028647	FBgn0028647	CG11902	FBtr0300735
FBgn0028662	FBgn0028662	NA	FBtr0083005
FBgn0028663	FBgn0028663	NA	FBtr0078369
FBgn0028664	FBgn0028664	NA	FBtr0073256
FBgn0028665	FBgn0028665	VhaAC39	FBtr0070611
FBgn0028667	FBgn0028667	NA	FBtr0302359
FBgn0028668	FBgn0028668	NA	FBtr0076091
FBgn0028671	FBgn0028671	Vha100-1	FBtr0085374
FBgn0028675	FBgn0028675	Sur	FBtr0290268
FBgn0028684	FBgn0028684	Tbp-1	FBtr0084452
FBgn0028685	FBgn0028685	NA	FBtr0305495
FBgn0028687	FBgn0028687	Rpt1	FBtr0088947
FBgn0028688	FBgn0028688	Rpn7	FBtr0084294
FBgn0028689	FBgn0028689	Rpn6	FBtr0087475
FBgn0028690	FBgn0028690	NA	FBtr0078629
FBgn0028691	FBgn0028691	Rpn9	FBtr0084469
FBgn0028693	FBgn0028693	Rpn12	FBtr0075308
FBgn0028694	FBgn0028694	Rpn11	FBtr0079027
FBgn0028696	FBgn0028696	NA	FBtr0302570
FBgn0028697	FBgn0028697	NA	FBtr0113743
FBgn0028699	FBgn0028699	NA	FBtr0073354
FBgn0028703	FBgn0028703	Nhe3	FBtr0273247
FBgn0028704	FBgn0028704	NA	FBtr0303908

FBgn0028707	FBgn0028707	NA	FBtr0080329
FBgn0028708	FBgn0028708	NA	FBtr0081969
FBgn0028734	FBgn0028734	NA	FBtr0082198
FBgn0028736	FBgn0028736	NA	FBtr0073217
FBgn0028738	FBgn0028738	NA	FBtr0072426
FBgn0028743	FBgn0028743	NA	FBtr0301142
FBgn0028744	FBgn0028744	CG5033	FBtr0089551
FBgn0028746	FBgn0028746	NA	FBtr0070543
FBgn0028833	FBgn0028833	Dak1	FBtr0084976
FBgn0028838	FBgn0028838	NA	FBtr0080671
FBgn0028844	FBgn0028844	NA	FBtr0300837
FBgn0028848	FBgn0028848	NA	FBtr0305567
FBgn0028850	FBgn0028850	NA	FBtr0300846
FBgn0028852	FBgn0028852	NA	FBtr0080733
FBgn0028855	FBgn0028855	NA	FBtr0080656
FBgn0028856	FBgn0028856	NA	FBtr0300064
FBgn0028857	FBgn0028857	NA	FBtr0300613
FBgn0028858	FBgn0028858	NA	FBtr0305563
FBgn0028862	FBgn0028862	CG15267	FBtr0080707
FBgn0028863	FBgn0028863	NA	FBtr0302502
FBgn0028864	FBgn0028864	NA	FBtr0080756
FBgn0028868	FBgn0028868	NA	FBtr0080672
FBgn0028870	FBgn0028870	NA	FBtr0080670
FBgn0028872	FBgn0028872	NA	FBtr0080606
FBgn0028875	FBgn0028875	NA	FBtr0309867
FBgn0028879	FBgn0028879	CG15270	FBtr0080702
FBgn0028880	FBgn0028880	NA	FBtr0305661
FBgn0028886	FBgn0028886	NA	FBtr0080685
FBgn0028888	FBgn0028888	NA	FBtr0305559
FBgn0028895	FBgn0028895	NA	FBtr0080829
FBgn0028896	FBgn0028896	NA	FBtr0080827
FBgn0028899	FBgn0028899	NA	FBtr0307508
FBgn0028900	FBgn0028900	NA	FBtr0080814
FBgn0028901	FBgn0028901	NA	FBtr0080786
FBgn0028902	FBgn0028902	Tektin-A	FBtr0080788
FBgn0028903	FBgn0028903	NA	FBtr0080787
FBgn0028906	FBgn0028906	NA	FBtr0305557
FBgn0028915	FBgn0028915	NA	FBtr0080640
FBgn0028917	FBgn0028917	NA	FBtr0080642
FBgn0028919	FBgn0028919	CG16865	FBtr0080540
FBgn0028920	FBgn0028920	NA	FBtr0080563
FBgn0028926	FBgn0028926	NA	FBtr0080695
FBgn0028931	FBgn0028931	NA	FBtr0080536
FBgn0028932	FBgn0028932	NA	FBtr0329825
FBgn0028935	FBgn0028935	NA	FBtr0306681
FBgn0028936	FBgn0028936	NA	FBtr0302501
FBgn0028938	FBgn0028938	NA	FBtr0080560
FBgn0028939	FBgn0028939	NA	FBtr0080589
FBgn0028940	FBgn0028940	Cyp28a5	FBtr0080587
FBgn0028944	FBgn0028944	NA	FBtr0080784
FBgn0028945	FBgn0028945	NA	FBtr0080754
FBgn0028949	FBgn0028949	NA	FBtr0080783
FBgn0028950	FBgn0028950	NA	FBtr0080785
FBgn0028952	FBgn0028952	NA	FBtr0310547
FBgn0028954	FBgn0028954	CG3287	FBtr0086158
FBgn0028955	FBgn0028955	CG8788	FBtr0088581
FBgn0028961	FBgn0028961	NA	FBtr0070575

FBgn0028963	FBgn0028963	NA		FBtr0087781
FBgn0028965	FBgn0028965	NA		FBtr0080427
FBgn0028968	FBgn0028968	NA		FBtr0085794
FBgn0028969	FBgn0028969	deltaCOP		FBtr0070371
FBgn0028970	FBgn0028970	betaggt-II		FBtr0077631
FBgn0028974	FBgn0028974	NA		FBtr0074404
FBgn0028980	FBgn0028980	NA		FBtr0077043
FBgn0028983	FBgn0028983	Spn6		FBtr0086733
FBgn0028984	FBgn0028984	Spn5		FBtr0083141
FBgn0028985	FBgn0028985	Spn4		FBtr0300336
FBgn0028986	FBgn0028986	NA		FBtr0081450
FBgn0028987	FBgn0028987	NA		FBtr0079624
FBgn0028988	FBgn0028988	NA		FBtr0300357
FBgn0028991	FBgn0028991	NA		FBtr0100501
FBgn0028992	FBgn0028992	NA		FBtr0083436
FBgn0028996	FBgn0028996	NA		FBtr0089104
FBgn0028997	FBgn0028997	NA		FBtr0082083
FBgn0028999	FBgn0028999	nerfin-1		FBtr0072592
FBgn0029002	FBgn0029002	miple2		FBtr0310417
FBgn0029003	FBgn0029003	mab-2		FBtr0070890
FBgn0029079	FBgn0029079	NA		FBtr0086832
FBgn0029091	FBgn0029091	NA		FBtr0308081
FBgn0029092	FBgn0029092	ced-6		FBtr0088548
FBgn0029094	FBgn0029094	NA		FBtr0074947
FBgn0029105	FBgn0029105	NA		FBtr0072148
FBgn0029117	FBgn0029117	NA		FBtr0076897
FBgn0029118	FBgn0029118	Sucb		FBtr0077134
FBgn0029121	FBgn0029121	NA		FBtr0077083
FBgn0029123	FBgn0029123	SoxN		FBtr0079733
FBgn0029131	FBgn0029131	debcl		FBtr0086108
FBgn0029133	FBgn0029133	REG		FBtr0310646
FBgn0029137	FBgn0029137	NA		FBtr0080362
FBgn0029147	FBgn0029147	NtR		FBtr0071762
FBgn0029152	FBgn0029152	Mkrn1		FBtr0078383
FBgn0029153	FBgn0029153	NA		FBtr0301868
FBgn0029154	FBgn0029154	NA		FBtr0301869
FBgn0029155	FBgn0029155	NA		FBtr0085110
FBgn0029157	FBgn0029157	NA		FBtr0084799
FBgn0029158	FBgn0029158	Las		FBtr0290266
FBgn0029161	FBgn0029161	slmo		FBtr0299810
FBgn0029172	FBgn0029172	NA		FBtr0076196
FBgn0029173	FBgn0029173	fu2		FBtr0079674
FBgn0029174	FBgn0029174	FKBP59		FBtr0079872
FBgn0029175	FBgn0029175	NA		FBtr0100512
FBgn0029176	FBgn0029176	NA		FBtr0085393
FBgn0029501	FBgn0029501	NA		FBtr0072299
FBgn0029502	FBgn0029502	NA		FBtr0070963
FBgn0029504	FBgn0029504	NA		FBtr0310287
FBgn0029514	FBgn0029514		312	FBtr0072680
FBgn0029518	FBgn0029518	NA		FBtr0308207
FBgn0029522	FBgn0029522	NA		FBtr0112947
FBgn0029523	FBgn0029523	NA		FBtr0070097
FBgn0029524	FBgn0029524	NA		FBtr0070078
FBgn0029525	FBgn0029525	CG18273		FBtr0070094
FBgn0029526	FBgn0029526	NA		FBtr0070095
FBgn0029531	FBgn0029531	CG13362		FBtr0070150
FBgn0029532	FBgn0029532	NA		FBtr0070149

FBgn0029535	FBgn0029535	NA	FBtr0070142
FBgn0029538	FBgn0029538	NA	FBtr0070193
FBgn0029539	FBgn0029539	NA	FBtr0310165
FBgn0029552	FBgn0029552	NA	FBtr0070172
FBgn0029573	FBgn0029573	NA	FBtr0070213
FBgn0029580	FBgn0029580	NA	FBtr0070254
FBgn0029584	FBgn0029584	NA	FBtr0305287
FBgn0029587	FBgn0029587	NA	FBtr0308320
FBgn0029588	FBgn0029588	NA	FBtr0089694
FBgn0029589	FBgn0029589	NA	FBtr0070374
FBgn0029590	FBgn0029590	NA	FBtr0309970
FBgn0029596	FBgn0029596	NA	FBtr0070338
FBgn0029608	FBgn0029608	NA	FBtr0100226
FBgn0029639	FBgn0029639	CG14419	FBtr0070564
FBgn0029643	FBgn0029643	NA	FBtr0070553
FBgn0029645	FBgn0029645	NA	FBtr0070555
FBgn0029646	FBgn0029646	NA	FBtr0070556
FBgn0029648	FBgn0029648	CG3603	FBtr0070558
FBgn0029657	FBgn0029657	NA	FBtr0070534
FBgn0029658	FBgn0029658	NA	FBtr0070532
FBgn0029659	FBgn0029659	NA	FBtr0070531
FBgn0029660	FBgn0029660	NA	FBtr0070526
FBgn0029661	FBgn0029661	NA	FBtr0310466
FBgn0029663	FBgn0029663	CG10804	FBtr0070591
FBgn0029672	FBgn0029672	NA	FBtr0070586
FBgn0029676	FBgn0029676	NA	FBtr0070598
FBgn0029679	FBgn0029679	NA	FBtr0070605
FBgn0029685	FBgn0029685	CG2938	FBtr0070627
FBgn0029686	FBgn0029686	NA	FBtr0070613
FBgn0029693	FBgn0029693	NA	FBtr0070671
FBgn0029696	FBgn0029696	NA	FBtr0070664
FBgn0029697	FBgn0029697	NA	FBtr0070645
FBgn0029700	FBgn0029700	NA	FBtr0070661
FBgn0029701	FBgn0029701	NA	FBtr0070646
FBgn0029702	FBgn0029702	NA	FBtr0070647
FBgn0029703	FBgn0029703	NA	FBtr0070658
FBgn0029704	FBgn0029704	NA	FBtr0070648
FBgn0029705	FBgn0029705	NA	FBtr0070654
FBgn0029706	FBgn0029706	NA	FBtr0070652
FBgn0029707	FBgn0029707	NA	FBtr0308918
FBgn0029708	FBgn0029708	NA	FBtr0308916
FBgn0029709	FBgn0029709	NA	FBtr0070675
FBgn0029710	FBgn0029710	NA	FBtr0070676
FBgn0029711	FBgn0029711	Usf	FBtr0308917
FBgn0029712	FBgn0029712	NA	FBtr0070677
FBgn0029713	FBgn0029713	CG11436	FBtr0070699
FBgn0029714	FBgn0029714	NA	FBtr0070678
FBgn0029715	FBgn0029715	NA	FBtr0070698
FBgn0029717	FBgn0029717	NA	FBtr0070697
FBgn0029720	FBgn0029720	NA	FBtr0070688
FBgn0029724	FBgn0029724	NA	FBtr0070736
FBgn0029726	FBgn0029726	NA	FBtr0299602
FBgn0029728	FBgn0029728	NA	FBtr0070731
FBgn0029729	FBgn0029729	NA	FBtr0070716
FBgn0029730	FBgn0029730	NA	FBtr0070717
FBgn0029733	FBgn0029733	NA	FBtr0070721
FBgn0029736	FBgn0029736	CG4041	FBtr0300998

FBgn0029738	FBgn0029738	NA	FBtr0321276
FBgn0029740	FBgn0029740	NA	FBtr0300018
FBgn0029746	FBgn0029746	NA	FBtr0070742
FBgn0029748	FBgn0029748	NA	FBtr0299590
FBgn0029750	FBgn0029750	NA	FBtr0070758
FBgn0029751	FBgn0029751	NA	FBtr0070757
FBgn0029752	FBgn0029752	NA	FBtr0070755
FBgn0029754	FBgn0029754	NA	FBtr0301881
FBgn0029755	FBgn0029755	NA	FBtr0070751
FBgn0029756	FBgn0029756	NA	FBtr0070798
FBgn0029761	FBgn0029761	NA	FBtr0301951
FBgn0029764	FBgn0029764	NA	FBtr0070795
FBgn0029778	FBgn0029778	NA	FBtr0070785
FBgn0029785	FBgn0029785	RpL35	FBtr0070801
FBgn0029791	FBgn0029791	NA	FBtr0301083
FBgn0029795	FBgn0029795	NA	FBtr0070806
FBgn0029799	FBgn0029799	CG15772	FBtr0070808
FBgn0029800	FBgn0029800	NA	FBtr0070840
FBgn0029801	FBgn0029801	NA	FBtr0070809
FBgn0029804	FBgn0029804	CG3097	FBtr0070836
FBgn0029807	FBgn0029807	CG3108	FBtr0070833
FBgn0029809	FBgn0029809	NA	FBtr0070813
FBgn0029810	FBgn0029810	NA	FBtr0070814
FBgn0029811	FBgn0029811	NA	FBtr0070815
FBgn0029812	FBgn0029812	NA	FBtr0070816
FBgn0029813	FBgn0029813	NA	FBtr0070832
FBgn0029817	FBgn0029817	NA	FBtr0070820
FBgn0029818	FBgn0029818	CG3033	FBtr0070830
FBgn0029821	FBgn0029821	NA	FBtr0070824
FBgn0029823	FBgn0029823	CG3011	FBtr0070827
FBgn0029825	FBgn0029825	NA	FBtr0070859
FBgn0029826	FBgn0029826	NA	FBtr0070861
FBgn0029827	FBgn0029827	NA	FBtr0070863
FBgn0029833	FBgn0029833	CG5941	FBtr0070867
FBgn0029834	FBgn0029834	NA	FBtr0305346
FBgn0029835	FBgn0029835	NA	FBtr0305343
FBgn0029836	FBgn0029836	CG5928	FBtr0308190
FBgn0029837	FBgn0029837	NA	FBtr0070888
FBgn0029846	FBgn0029846	NA	FBtr0112953
FBgn0029848	FBgn0029848	NA	FBtr0070878
FBgn0029849	FBgn0029849	NA	FBtr0070875
FBgn0029851	FBgn0029851	NA	FBtr0070893
FBgn0029853	FBgn0029853	NA	FBtr0070895
FBgn0029854	FBgn0029854	NA	FBtr0089869
FBgn0029856	FBgn0029856	NA	FBtr0070931
FBgn0029857	FBgn0029857	NA	FBtr0070897
FBgn0029858	FBgn0029858	CG15896	FBtr0305494
FBgn0029864	FBgn0029864	NA	FBtr0112955
FBgn0029866	FBgn0029866	NA	FBtr0070904
FBgn0029873	FBgn0029873	CG3918	FBtr0070912
FBgn0029874	FBgn0029874	NA	FBtr0070918
FBgn0029877	FBgn0029877	NA	FBtr0070944
FBgn0029879	FBgn0029879	NA	FBtr0070946
FBgn0029880	FBgn0029880	NA	FBtr0308654
FBgn0029881	FBgn0029881	NA	FBtr0070948
FBgn0029882	FBgn0029882	NA	FBtr0070993
FBgn0029885	FBgn0029885	CG3224	FBtr0070992

FBgn0029890	FBgn0029890	NA	FBtr0070955
FBgn0029891	FBgn0029891	NA	FBtr0100419
FBgn0029892	FBgn0029892	CG3184	FBtr0070989
FBgn0029893	FBgn0029893	NA	FBtr0070988
FBgn0029894	FBgn0029894	NA	FBtr0070987
FBgn0029895	FBgn0029895	NA	FBtr0070959
FBgn0029896	FBgn0029896	CG3168	FBtr0070984
FBgn0029898	FBgn0029898	NA	FBtr0302356
FBgn0029899	FBgn0029899	CG14438	FBtr0070961
FBgn0029903	FBgn0029903	NA	FBtr0308228
FBgn0029905	FBgn0029905	NA	FBtr0070974
FBgn0029906	FBgn0029906	NA	FBtr0070970
FBgn0029907	FBgn0029907	NA	FBtr0299814
FBgn0029909	FBgn0029909	NA	FBtr0070973
FBgn0029911	FBgn0029911	NA	FBtr0300880
FBgn0029912	FBgn0029912	NA	FBtr0070994
FBgn0029913	FBgn0029913	NA	FBtr0071041
FBgn0029914	FBgn0029914	NA	FBtr0070995
FBgn0029915	FBgn0029915	NA	FBtr0071040
FBgn0029920	FBgn0029920	NA	FBtr0307391
FBgn0029922	FBgn0029922	NA	FBtr0304108
FBgn0029924	FBgn0029924	CG4586	FBtr0071003
FBgn0029925	FBgn0029925	CG3040	FBtr0071038
FBgn0029928	FBgn0029928	NA	FBtr0071032
FBgn0029929	FBgn0029929	NA	FBtr0071009
FBgn0029930	FBgn0029930	NA	FBtr0300759
FBgn0029931	FBgn0029931	CG14427	FBtr0071010
FBgn0029933	FBgn0029933	NA	FBtr0100572
FBgn0029935	FBgn0029935	CG4615	FBtr0071020
FBgn0029939	FBgn0029939	NA	FBtr0309030
FBgn0029941	FBgn0029941	NA	FBtr0303415
FBgn0029942	FBgn0029942	NA	FBtr0303414
FBgn0029943	FBgn0029943	Atg5	FBtr0071065
FBgn0029944	FBgn0029944	NA	FBtr0071049
FBgn0029945	FBgn0029945	NA	FBtr0071051
FBgn0029946	FBgn0029946	NA	FBtr0071064
FBgn0029947	FBgn0029947	NA	FBtr0071054
FBgn0029948	FBgn0029948	NA	FBtr0071055
FBgn0029949	FBgn0029949	NA	FBtr0071063
FBgn0029950	FBgn0029950	CG9657	FBtr0071057
FBgn0029951	FBgn0029951	NA	FBtr0071067
FBgn0029952	FBgn0029952	NA	FBtr0071081
FBgn0029955	FBgn0029955	CG15478	FBtr0300836
FBgn0029957	FBgn0029957	CG12155	FBtr0071071
FBgn0029958	FBgn0029958	NA	FBtr0307273
FBgn0029959	FBgn0029959	NA	FBtr0307272
FBgn0029962	FBgn0029962	NA	FBtr0300019
FBgn0029964	FBgn0029964	NA	FBtr0273372
FBgn0029966	FBgn0029966	NA	FBtr0309827
FBgn0029968	FBgn0029968	NA	FBtr0071088
FBgn0029970	FBgn0029970	Nek2	FBtr0071142
FBgn0029971	FBgn0029971	NA	FBtr0300900
FBgn0029973	FBgn0029973	NA	FBtr0299927
FBgn0029974	FBgn0029974	NA	FBtr0307270
FBgn0029975	FBgn0029975	CG1444	FBtr0071091
FBgn0029986	FBgn0029986	NA	FBtr0071128
FBgn0029987	FBgn0029987	NA	FBtr0071099

FBgn0029989	FBgn0029989	NA	FBtr0071124
FBgn0029992	FBgn0029992	NA	FBtr0071117
FBgn0029993	FBgn0029993	NA	FBtr0071106
FBgn0029995	FBgn0029995	NA	FBtr0301736
FBgn0030003	FBgn0030003	CG2116	FBtr0071152
FBgn0030004	FBgn0030004	CG10958	FBtr0071153
FBgn0030005	FBgn0030005	NA	FBtr0300914
FBgn0030006	FBgn0030006	NA	FBtr0071191
FBgn0030007	FBgn0030007	NA	FBtr0071190
FBgn0030008	FBgn0030008	NA	FBtr0071155
FBgn0030009	FBgn0030009	NA	FBtr0301688
FBgn0030010	FBgn0030010	NA	FBtr0071157
FBgn0030011	FBgn0030011	NA	FBtr0071189
FBgn0030012	FBgn0030012	NA	FBtr0071158
FBgn0030013	FBgn0030013	NA	FBtr0303457
FBgn0030017	FBgn0030017	NA	FBtr0071185
FBgn0030018	FBgn0030018	NA	FBtr0303843
FBgn0030025	FBgn0030025	NA	FBtr0071166
FBgn0030026	FBgn0030026	sni	FBtr0071183
FBgn0030027	FBgn0030027	NA	FBtr0071170
FBgn0030028	FBgn0030028	NA	FBtr0307283
FBgn0030029	FBgn0030029	NA	FBtr0071178
FBgn0030030	FBgn0030030	NA	FBtr0071171
FBgn0030034	FBgn0030034	NA	FBtr0071174
FBgn0030035	FBgn0030035	NA	FBtr0071175
FBgn0030037	FBgn0030037	NA	FBtr0071246
FBgn0030038	FBgn0030038	CG1440	FBtr0112962
FBgn0030039	FBgn0030039	NA	FBtr0071245
FBgn0030040	FBgn0030040	CG15347	FBtr0302500
FBgn0030041	FBgn0030041	NA	FBtr0071240
FBgn0030049	FBgn0030049	NA	FBtr0071213
FBgn0030050	FBgn0030050	CG12111	FBtr0071232
FBgn0030051	FBgn0030051	NA	FBtr0071231
FBgn0030052	FBgn0030052	NA	FBtr0071214
FBgn0030053	FBgn0030053	NA	FBtr0071218
FBgn0030057	FBgn0030057	NA	FBtr0071225
FBgn0030058	FBgn0030058	NA	FBtr0071224
FBgn0030063	FBgn0030063	CG1789	FBtr0071251
FBgn0030065	FBgn0030065	CG12075	FBtr0307271
FBgn0030066	FBgn0030066	CG1885	FBtr0071268
FBgn0030067	FBgn0030067	NA	FBtr0071255
FBgn0030077	FBgn0030077	NA	FBtr0071357
FBgn0030081	FBgn0030081	NA	FBtr0071353
FBgn0030082	FBgn0030082	HP1b	FBtr0309871
FBgn0030085	FBgn0030085	NA	FBtr0071288
FBgn0030086	FBgn0030086	NA	FBtr0071289
FBgn0030087	FBgn0030087	NA	FBtr0309251
FBgn0030088	FBgn0030088	NA	FBtr0071293
FBgn0030089	FBgn0030089	NA	FBtr0309255
FBgn0030105	FBgn0030105	NA	FBtr0071309
FBgn0030108	FBgn0030108	NA	FBtr0071311
FBgn0030116	FBgn0030116	NA	FBtr0304143
FBgn0030117	FBgn0030117	NA	FBtr0071318
FBgn0030120	FBgn0030120	NA	FBtr0071328
FBgn0030121	FBgn0030121	NA	FBtr0071327
FBgn0030136	FBgn0030136	NA	FBtr0071360
FBgn0030137	FBgn0030137	NA	FBtr0071371

FBgn0030141	FBgn0030141	NA	FBtr0071363
FBgn0030142	FBgn0030142	NA	FBtr0071368
FBgn0030151	FBgn0030151	NA	FBtr0089384
FBgn0030156	FBgn0030156	NA	FBtr0071397
FBgn0030161	FBgn0030161	NA	FBtr0112967
FBgn0030163	FBgn0030163	NA	FBtr0112968
FBgn0030164	FBgn0030164	NA	FBtr0071408
FBgn0030167	FBgn0030167	CG15252	FBtr0307590
FBgn0030170	FBgn0030170	CG2990	FBtr0071415
FBgn0030174	FBgn0030174	CG15312	FBtr0071423
FBgn0030177	FBgn0030177	NA	FBtr0071430
FBgn0030178	FBgn0030178	NA	FBtr0290234
FBgn0030181	FBgn0030181	NA	FBtr0112973
FBgn0030183	FBgn0030183	NA	FBtr0112974
FBgn0030186	FBgn0030186	NA	FBtr0071486
FBgn0030189	FBgn0030189	NA	FBtr0071441
FBgn0030192	FBgn0030192	NA	FBtr0071442
FBgn0030193	FBgn0030193	NA	FBtr0071443
FBgn0030195	FBgn0030195	NA	FBtr0071445
FBgn0030200	FBgn0030200	NA	FBtr0071448
FBgn0030206	FBgn0030206	NA	FBtr0071450
FBgn0030207	FBgn0030207	NA	FBtr0071459
FBgn0030215	FBgn0030215	NA	FBtr0071512
FBgn0030218	FBgn0030218	NA	FBtr0071510
FBgn0030221	FBgn0030221	NA	FBtr0071507
FBgn0030222	FBgn0030222	NA	FBtr0071506
FBgn0030223	FBgn0030223	NA	FBtr0071492
FBgn0030224	FBgn0030224	NA	FBtr0071504
FBgn0030230	FBgn0030230	NA	FBtr0301558
FBgn0030234	FBgn0030234	CG15211	FBtr0112976
FBgn0030237	FBgn0030237	CG15209	FBtr0073378
FBgn0030239	FBgn0030239	NA	FBtr0073379
FBgn0030240	FBgn0030240	NA	FBtr0073380
FBgn0030244	FBgn0030244	CG2157	FBtr0073382
FBgn0030245	FBgn0030245	CG1637	FBtr0073406
FBgn0030249	FBgn0030249	NA	FBtr0112978
FBgn0030258	FBgn0030258	NA	FBtr0073396
FBgn0030259	FBgn0030259	CG1545	FBtr0299600
FBgn0030270	FBgn0030270	NA	FBtr0073473
FBgn0030271	FBgn0030271	NA	FBtr0307187
FBgn0030272	FBgn0030272	NA	FBtr0073439
FBgn0030274	FBgn0030274	NA	FBtr0307188
FBgn0030283	FBgn0030283	NA	FBtr0073447
FBgn0030286	FBgn0030286	NA	FBtr0073448
FBgn0030289	FBgn0030289	NA	FBtr0290227
FBgn0030290	FBgn0030290	NA	FBtr0073450
FBgn0030291	FBgn0030291	CG1738	FBtr0073451
FBgn0030294	FBgn0030294	NA	FBtr0073456
FBgn0030296	FBgn0030296	NA	FBtr0300849
FBgn0030299	FBgn0030299	NA	FBtr0073547
FBgn0030309	FBgn0030309	CG1572	FBtr0073543
FBgn0030313	FBgn0030313	NA	FBtr0073511
FBgn0030316	FBgn0030316	NA	FBtr0300872
FBgn0030317	FBgn0030317	CG1561	FBtr0073539
FBgn0030318	FBgn0030318	NA	FBtr0073519
FBgn0030319	FBgn0030319	NA	FBtr0073537
FBgn0030320	FBgn0030320	NA	FBtr0073536

FBgn0030321	FBgn0030321	NA	FBtr0073520
FBgn0030322	FBgn0030322	NA	FBtr0073535
FBgn0030326	FBgn0030326	NA	FBtr0073531
FBgn0030327	FBgn0030327	FucT6	FBtr0073620
FBgn0030328	FBgn0030328	NA	FBtr0073615
FBgn0030329	FBgn0030329	CG1837	FBtr0073551
FBgn0030330	FBgn0030330	NA	FBtr0073552
FBgn0030331	FBgn0030331	NA	FBtr0112979
FBgn0030332	FBgn0030332	CG9360	FBtr0073613
FBgn0030334	FBgn0030334	NA	FBtr0073557
FBgn0030339	FBgn0030339	NA	FBtr0073565
FBgn0030340	FBgn0030340	NA	FBtr0303268
FBgn0030343	FBgn0030343	NA	FBtr0273257
FBgn0030345	FBgn0030345	CG1847	FBtr0073567
FBgn0030346	FBgn0030346	NA	FBtr0073600
FBgn0030347	FBgn0030347	CG15739	FBtr0073569
FBgn0030348	FBgn0030348	NA	FBtr0073599
FBgn0030349	FBgn0030349	NA	FBtr0114452
FBgn0030357	FBgn0030357	NA	FBtr0073591
FBgn0030359	FBgn0030359	NA	FBtr0073581
FBgn0030373	FBgn0030373	NA	FBtr0073637
FBgn0030374	FBgn0030374	NA	FBtr0300835
FBgn0030375	FBgn0030375	NA	FBtr0073635
FBgn0030376	FBgn0030376	NA	FBtr0300918
FBgn0030377	FBgn0030377	NA	FBtr0073634
FBgn0030386	FBgn0030386	NA	FBtr0073644
FBgn0030400	FBgn0030400	NA	FBtr0273245
FBgn0030403	FBgn0030403	NA	FBtr0073658
FBgn0030406	FBgn0030406	NA	FBtr0073657
FBgn0030408	FBgn0030408	NA	FBtr0073656
FBgn0030409	FBgn0030409	NA	FBtr0307897
FBgn0030410	FBgn0030410	CG15727	FBtr0073709
FBgn0030411	FBgn0030411	NA	FBtr0073675
FBgn0030412	FBgn0030412	NA	FBtr0073678
FBgn0030420	FBgn0030420	NA	FBtr0073704
FBgn0030425	FBgn0030425	NA	FBtr0073690
FBgn0030429	FBgn0030429	NA	FBtr0073699
FBgn0030431	FBgn0030431	NA	FBtr0073695
FBgn0030432	FBgn0030432	NA	FBtr0073694
FBgn0030437	FBgn0030437	NA	FBtr0073753
FBgn0030438	FBgn0030438	NA	FBtr0305600
FBgn0030439	FBgn0030439	NA	FBtr0073752
FBgn0030440	FBgn0030440	NA	FBtr0310532
FBgn0030441	FBgn0030441	NA	FBtr0073717
FBgn0030443	FBgn0030443	NA	FBtr0073750
FBgn0030449	FBgn0030449	NA	FBtr0073745
FBgn0030455	FBgn0030455	NA	FBtr0301690
FBgn0030456	FBgn0030456	CG4332	FBtr0073726
FBgn0030457	FBgn0030457	CG12096	FBtr0073727
FBgn0030459	FBgn0030459	CG12723	FBtr0073738
FBgn0030460	FBgn0030460	NA	FBtr0073737
FBgn0030462	FBgn0030462	NA	FBtr0290202
FBgn0030465	FBgn0030465	CG15743	FBtr0073755
FBgn0030468	FBgn0030468	NA	FBtr0310465
FBgn0030469	FBgn0030469	NA	FBtr0073758
FBgn0030477	FBgn0030477	NA	FBtr0073768
FBgn0030479	FBgn0030479	NA	FBtr0304001

FBgn0030480	FBgn0030480	NA	FBtr0073775
FBgn0030481	FBgn0030481	CG1662	FBtr0073776
FBgn0030483	FBgn0030483	NA	FBtr0073788
FBgn0030485	FBgn0030485	NA	FBtr0073787
FBgn0030486	FBgn0030486	CG1716	FBtr0073779
FBgn0030491	FBgn0030491	NA	FBtr0273441
FBgn0030492	FBgn0030492	NA	FBtr0073782
FBgn0030501	FBgn0030501	NA	FBtr0073806
FBgn0030502	FBgn0030502	NA	FBtr0073849
FBgn0030503	FBgn0030503	NA	FBtr0308644
FBgn0030505	FBgn0030505	NFAT	FBtr0073808
FBgn0030506	FBgn0030506	NA	FBtr0073847
FBgn0030511	FBgn0030511	NA	FBtr0073812
FBgn0030512	FBgn0030512	NA	FBtr0073845
FBgn0030514	FBgn0030514	NA	FBtr0073843
FBgn0030518	FBgn0030518	CG11134	FBtr0073818
FBgn0030519	FBgn0030519	CG11151	FBtr0073819
FBgn0030520	FBgn0030520	NA	FBtr0112981
FBgn0030525	FBgn0030525	NA	FBtr0073836
FBgn0030530	FBgn0030530	NA	FBtr0073834
FBgn0030532	FBgn0030532	NA	FBtr0073829
FBgn0030536	FBgn0030536	NA	FBtr0073854
FBgn0030537	FBgn0030537	NA	FBtr0073868
FBgn0030538	FBgn0030538	NA	FBtr0089267
FBgn0030539	FBgn0030539	NA	FBtr0073891
FBgn0030541	FBgn0030541	NA	FBtr0073893
FBgn0030542	FBgn0030542	NA	FBtr0100281
FBgn0030543	FBgn0030543	NA	FBtr0114508
FBgn0030544	FBgn0030544	NA	FBtr0073928
FBgn0030559	FBgn0030559	NA	FBtr0299543
FBgn0030562	FBgn0030562	CG9400	FBtr0112982
FBgn0030563	FBgn0030563	NA	FBtr0073919
FBgn0030566	FBgn0030566	NA	FBtr0073915
FBgn0030571	FBgn0030571	CG14414	FBtr0303288
FBgn0030572	FBgn0030572	NA	FBtr0073945
FBgn0030573	FBgn0030573	NA	FBtr0073933
FBgn0030574	FBgn0030574	NA	FBtr0073944
FBgn0030575	FBgn0030575	NA	FBtr0290021
FBgn0030581	FBgn0030581	NA	FBtr0100150
FBgn0030582	FBgn0030582	NA	FBtr0073946
FBgn0030584	FBgn0030584	CG14407	FBtr0073989
FBgn0030586	FBgn0030586	NA	FBtr0073987
FBgn0030587	FBgn0030587	CG9522	FBtr0073986
FBgn0030589	FBgn0030589	NA	FBtr0073984
FBgn0030590	FBgn0030590	NA	FBtr0073983
FBgn0030591	FBgn0030591	CG9517	FBtr0073981
FBgn0030592	FBgn0030592	CG9514	FBtr0073980
FBgn0030593	FBgn0030593	NA	FBtr0073979
FBgn0030594	FBgn0030594	NA	FBtr0073978
FBgn0030595	FBgn0030595	NA	FBtr0073977
FBgn0030598	FBgn0030598	CG9503	FBtr0073974
FBgn0030599	FBgn0030599	NA	FBtr0303295
FBgn0030600	FBgn0030600	NA	FBtr0073970
FBgn0030608	FBgn0030608	Lsd-2	FBtr0110969
FBgn0030613	FBgn0030613	NA	FBtr0307208
FBgn0030614	FBgn0030614	NA	FBtr0074029
FBgn0030615	FBgn0030615	NA	FBtr0074028

FBgn0030617	FBgn0030617	NA	FBtr0112984
FBgn0030618	FBgn0030618	NA	FBtr0307025
FBgn0030622	FBgn0030622	NA	FBtr0074024
FBgn0030628	FBgn0030628	CG9114	FBtr0074020
FBgn0030629	FBgn0030629	NA	FBtr0074019
FBgn0030630	FBgn0030630	NA	FBtr0074018
FBgn0030634	FBgn0030634	NA	FBtr0074012
FBgn0030640	FBgn0030640	NA	FBtr0074034
FBgn0030641	FBgn0030641	NA	FBtr0074032
FBgn0030642	FBgn0030642	NA	FBtr0074076
FBgn0030643	FBgn0030643	NA	FBtr0074035
FBgn0030645	FBgn0030645	NA	FBtr0074036
FBgn0030646	FBgn0030646	NA	FBtr0074074
FBgn0030647	FBgn0030647	NA	FBtr0074037
FBgn0030648	FBgn0030648	CG6340	FBtr0310061
FBgn0030655	FBgn0030655	NA	FBtr0074071
FBgn0030657	FBgn0030657	NA	FBtr0089702
FBgn0030658	FBgn0030658	CG7872	FBtr0074046
FBgn0030659	FBgn0030659	CG9215	FBtr0074066
FBgn0030660	FBgn0030660	NA	FBtr0074047
FBgn0030663	FBgn0030663	NA	FBtr0074050
FBgn0030666	FBgn0030666	CG12708	FBtr0289961
FBgn0030667	FBgn0030667	NA	FBtr0307503
FBgn0030668	FBgn0030668	NA	FBtr0074053
FBgn0030669	FBgn0030669	CG9240	FBtr0074060
FBgn0030670	FBgn0030670	CG9245	FBtr0074059
FBgn0030671	FBgn0030671	NA	FBtr0074054
FBgn0030673	FBgn0030673	NA	FBtr0074055
FBgn0030676	FBgn0030676	NA	FBtr0273278
FBgn0030678	FBgn0030678	CG11679	FBtr0074202
FBgn0030679	FBgn0030679	CG8206	FBtr0074085
FBgn0030680	FBgn0030680	NA	FBtr0074200
FBgn0030686	FBgn0030686	NA	FBtr0074090
FBgn0030687	FBgn0030687	NA	FBtr0300811
FBgn0030688	FBgn0030688	NA	FBtr0074193
FBgn0030691	FBgn0030691	NA	FBtr0074192
FBgn0030692	FBgn0030692	mRpS30	FBtr0074094
FBgn0030694	FBgn0030694	NA	FBtr0074097
FBgn0030695	FBgn0030695	NA	FBtr0307978
FBgn0030696	FBgn0030696	NA	FBtr0074098
FBgn0030700	FBgn0030700	NA	FBtr0074105
FBgn0030701	FBgn0030701	NA	FBtr0074116
FBgn0030703	FBgn0030703	NA	FBtr0074166
FBgn0030704	FBgn0030704	NA	FBtr0074117
FBgn0030706	FBgn0030706	NA	FBtr0301666
FBgn0030707	FBgn0030707	NA	FBtr0301232
FBgn0030710	FBgn0030710	CG8924	FBtr0074130
FBgn0030711	FBgn0030711	NA	FBtr0074132
FBgn0030716	FBgn0030716	NA	FBtr0074153
FBgn0030717	FBgn0030717	CG8931	FBtr0074134
FBgn0030718	FBgn0030718	CG9172	FBtr0074151
FBgn0030722	FBgn0030722	NA	FBtr0074260
FBgn0030724	FBgn0030724	Nipsnap	FBtr0074258
FBgn0030725	FBgn0030725	NA	FBtr0074207
FBgn0030729	FBgn0030729	NA	FBtr0290087
FBgn0030733	FBgn0030733	NA	FBtr0074216
FBgn0030734	FBgn0030734	NA	FBtr0074243

FBgn0030735	FBgn0030735	CG3632	FBtr0112991
FBgn0030737	FBgn0030737	NA	FBtr0074240
FBgn0030738	FBgn0030738	NA	FBtr0112992
FBgn0030747	FBgn0030747	NA	FBtr0308604
FBgn0030748	FBgn0030748	NA	FBtr0074273
FBgn0030756	FBgn0030756	NA	FBtr0074297
FBgn0030757	FBgn0030757	NA	FBtr0074296
FBgn0030758	FBgn0030758	NA	FBtr0074293
FBgn0030759	FBgn0030759	NA	FBtr0074282
FBgn0030761	FBgn0030761	NA	FBtr0074289
FBgn0030763	FBgn0030763	NA	FBtr0074288
FBgn0030764	FBgn0030764	NA	FBtr0074287
FBgn0030765	FBgn0030765	NA	FBtr0074377
FBgn0030768	FBgn0030768	NA	FBtr0074376
FBgn0030769	FBgn0030769	NA	FBtr0290216
FBgn0030770	FBgn0030770	NA	FBtr0074373
FBgn0030773	FBgn0030773	NA	FBtr0074364
FBgn0030774	FBgn0030774	CG9675	FBtr0074363
FBgn0030775	FBgn0030775	CG9673	FBtr0074362
FBgn0030776	FBgn0030776	NA	FBtr0074321
FBgn0030777	FBgn0030777	NA	FBtr0074361
FBgn0030778	FBgn0030778	CG4678	FBtr0300033
FBgn0030780	FBgn0030780	NA	FBtr0074322
FBgn0030786	FBgn0030786	mRpL22	FBtr0074330
FBgn0030788	FBgn0030788	NA	FBtr0074331
FBgn0030789	FBgn0030789	Rrp45	FBtr0074355
FBgn0030790	FBgn0030790	NA	FBtr0305899
FBgn0030791	FBgn0030791	NA	FBtr0074353
FBgn0030792	FBgn0030792	CG4789	FBtr0074333
FBgn0030793	FBgn0030793	NA	FBtr0074352
FBgn0030794	FBgn0030794	CG13005	FBtr0074334
FBgn0030795	FBgn0030795	NA	FBtr0074335
FBgn0030796	FBgn0030796	CG4829	FBtr0074336
FBgn0030797	FBgn0030797	CG13004	FBtr0112996
FBgn0030798	FBgn0030798	CG13003	FBtr0074349
FBgn0030801	FBgn0030801	NA	FBtr0074341
FBgn0030802	FBgn0030802	NA	FBtr0074347
FBgn0030803	FBgn0030803	NA	FBtr0074342
FBgn0030804	FBgn0030804	NA	FBtr0074343
FBgn0030806	FBgn0030806	NA	FBtr0074417
FBgn0030807	FBgn0030807	NA	FBtr0074416
FBgn0030808	FBgn0030808	NA	FBtr0290096
FBgn0030809	FBgn0030809	NA	FBtr0074415
FBgn0030810	FBgn0030810	NA	FBtr0299585
FBgn0030811	FBgn0030811	NA	FBtr0299578
FBgn0030812	FBgn0030812	NA	FBtr0304840
FBgn0030813	FBgn0030813	CG4949	FBtr0074381
FBgn0030817	FBgn0030817	NA	FBtr0074384
FBgn0030823	FBgn0030823	NA	FBtr0074403
FBgn0030824	FBgn0030824	NA	FBtr0074389
FBgn0030826	FBgn0030826	NA	FBtr0074402
FBgn0030827	FBgn0030827	NA	FBtr0074392
FBgn0030830	FBgn0030830	NA	FBtr0114540
FBgn0030832	FBgn0030832	NA	FBtr0074398
FBgn0030833	FBgn0030833	CG8915	FBtr0074447
FBgn0030834	FBgn0030834	NA	FBtr0074446
FBgn0030836	FBgn0030836	NA	FBtr0074445

FBgn0030844	FBgn0030844	NA	FBtr0329827
FBgn0030846	FBgn0030846	NA	FBtr0300778
FBgn0030850	FBgn0030850	CG8408	FBtr0074487
FBgn0030864	FBgn0030864	NA	FBtr0074475
FBgn0030868	FBgn0030868	NA	FBtr0074474
FBgn0030869	FBgn0030869	NA	FBtr0310310
FBgn0030871	FBgn0030871	CG8142	FBtr0074472
FBgn0030872	FBgn0030872	NA	FBtr0074464
FBgn0030873	FBgn0030873	CG15814	FBtr0074469
FBgn0030874	FBgn0030874	NA	FBtr0074465
FBgn0030876	FBgn0030876	NA	FBtr0303769
FBgn0030877	FBgn0030877	CG7846	FBtr0074544
FBgn0030878	FBgn0030878	CG6769	FBtr0074493
FBgn0030880	FBgn0030880	NA	FBtr0074495
FBgn0030881	FBgn0030881	NA	FBtr0074494
FBgn0030882	FBgn0030882	NA	FBtr0300973
FBgn0030883	FBgn0030883	NA	FBtr0074540
FBgn0030887	FBgn0030887	NA	FBtr0089574
FBgn0030889	FBgn0030889	NA	FBtr0112999
FBgn0030891	FBgn0030891	NA	FBtr0074510
FBgn0030892	FBgn0030892	NA	FBtr0308352
FBgn0030893	FBgn0030893	NA	FBtr0074513
FBgn0030894	FBgn0030894	NA	FBtr0074527
FBgn0030895	FBgn0030895	NA	FBtr0074515
FBgn0030899	FBgn0030899	NA	FBtr0074590
FBgn0030900	FBgn0030900	NA	FBtr0074549
FBgn0030904	FBgn0030904	NA	FBtr0300182
FBgn0030913	FBgn0030913	NA	FBtr0307553
FBgn0030915	FBgn0030915	CG6179	FBtr0074558
FBgn0030925	FBgn0030925	CG6361	FBtr0113000
FBgn0030931	FBgn0030931	NA	FBtr0074592
FBgn0030935	FBgn0030935	NA	FBtr0299567
FBgn0030937	FBgn0030937	NA	FBtr0074619
FBgn0030938	FBgn0030938	NA	FBtr0074596
FBgn0030940	FBgn0030940	NA	FBtr0074618
FBgn0030943	FBgn0030943	NA	FBtr0074601
FBgn0030944	FBgn0030944	NA	FBtr0074615
FBgn0030949	FBgn0030949	NA	FBtr0074604
FBgn0030951	FBgn0030951	NA	FBtr0074607
FBgn0030954	FBgn0030954	NA	FBtr0113003
FBgn0030956	FBgn0030956	NA	FBtr0074661
FBgn0030958	FBgn0030958	NA	FBtr0302363
FBgn0030959	FBgn0030959	NA	FBtr0074660
FBgn0030960	FBgn0030960	NA	FBtr0074635
FBgn0030961	FBgn0030961	NA	FBtr0301153
FBgn0030963	FBgn0030963	NA	FBtr0074639
FBgn0030964	FBgn0030964	Pvf1	FBtr0074640
FBgn0030966	FBgn0030966	CG7280	FBtr0074655
FBgn0030968	FBgn0030968	CG7322	FBtr0074654
FBgn0030969	FBgn0030969	NA	FBtr0074643
FBgn0030976	FBgn0030976	NA	FBtr0113004
FBgn0030981	FBgn0030981	NA	FBtr0074665
FBgn0030982	FBgn0030982	NA	FBtr0074704
FBgn0030983	FBgn0030983	NA	FBtr0074703
FBgn0030984	FBgn0030984	tgy	FBtr0290276
FBgn0030985	FBgn0030985	NA	FBtr0074701
FBgn0030990	FBgn0030990	CG7556	FBtr0074697

FBgn0030991	FBgn0030991	NA	FBtr0074666
FBgn0030992	FBgn0030992	NA	FBtr0074668
FBgn0030993	FBgn0030993	NA	FBtr0074669
FBgn0030994	FBgn0030994	NA	FBtr0074670
FBgn0030997	FBgn0030997	NA	FBtr0308082
FBgn0030998	FBgn0030998	NA	FBtr0074692
FBgn0030999	FBgn0030999	CG7874	FBtr0074672
FBgn0031002	FBgn0031002	NA	FBtr0074691
FBgn0031008	FBgn0031008	NA	FBtr0074688
FBgn0031010	FBgn0031010	NA	FBtr0074685
FBgn0031011	FBgn0031011	CG8034	FBtr0074684
FBgn0031016	FBgn0031016	NA	FBtr0309224
FBgn0031018	FBgn0031018	NA	FBtr0074710
FBgn0031020	FBgn0031020	NA	FBtr0074711
FBgn0031022	FBgn0031022	NA	FBtr0074783
FBgn0031023	FBgn0031023	NA	FBtr0074721
FBgn0031026	FBgn0031026	NA	FBtr0074779
FBgn0031030	FBgn0031030	Tao-1	FBtr0303999
FBgn0031031	FBgn0031031	NA	FBtr0308568
FBgn0031032	FBgn0031032	NA	FBtr0074729
FBgn0031036	FBgn0031036	NA	FBtr0307162
FBgn0031037	FBgn0031037	CG14207	FBtr0074734
FBgn0031038	FBgn0031038	NA	FBtr0074736
FBgn0031039	FBgn0031039	NA	FBtr0074738
FBgn0031042	FBgn0031042	NA	FBtr0290086
FBgn0031055	FBgn0031055	NA	FBtr0308570
FBgn0031057	FBgn0031057	NA	FBtr0074750
FBgn0031058	FBgn0031058	NA	FBtr0300350
FBgn0031059	FBgn0031059	NA	FBtr0074753
FBgn0031060	FBgn0031060	NA	FBtr0309217
FBgn0031061	FBgn0031061	NA	FBtr0309218
FBgn0031062	FBgn0031062	NA	FBtr0074787
FBgn0031064	FBgn0031064	NA	FBtr0074788
FBgn0031066	FBgn0031066	NA	FBtr0074815
FBgn0031068	FBgn0031068	CG12534	FBtr0074794
FBgn0031077	FBgn0031077	NA	FBtr0308650
FBgn0031078	FBgn0031078	NA	FBtr0074798
FBgn0031080	FBgn0031080	NA	FBtr0070036
FBgn0031081	FBgn0031081	NA	FBtr0307555
FBgn0031088	FBgn0031088	NA	FBtr0301569
FBgn0031089	FBgn0031089	NA	FBtr0070006
FBgn0031090	FBgn0031090	NA	FBtr0070028
FBgn0031092	FBgn0031092	NA	FBtr0070007
FBgn0031093	FBgn0031093	NA	FBtr0070026
FBgn0031094	FBgn0031094	NA	FBtr0070008
FBgn0031097	FBgn0031097	CG17052	FBtr0077334
FBgn0031099	FBgn0031099	CG17065	FBtr0301568
FBgn0031100	FBgn0031100	NA	FBtr0306678
FBgn0031103	FBgn0031103	NA	FBtr0077332
FBgn0031104	FBgn0031104	NA	FBtr0302241
FBgn0031106	FBgn0031106	NA	FBtr0077330
FBgn0031107	FBgn0031107	NA	FBtr0301344
FBgn0031108	FBgn0031108	NA	FBtr0077329
FBgn0031109	FBgn0031109	NA	FBtr0307166
FBgn0031111	FBgn0031111	NA	FBtr0077326
FBgn0031114	FBgn0031114	NA	FBtr0077305
FBgn0031115	FBgn0031115	NA	FBtr0089642

FBgn0031116	FBgn0031116	NA	FBtr0077308
FBgn0031117	FBgn0031117	CG1702	FBtr0300210
FBgn0031118	FBgn0031118	RhoGAP19D	FBtr0077320
FBgn0031126	FBgn0031126	NA	FBtr0077276
FBgn0031127	FBgn0031127	NA	FBtr0077277
FBgn0031128	FBgn0031128	NA	FBtr0077294
FBgn0031132	FBgn0031132	NA	FBtr0077280
FBgn0031134	FBgn0031134	NA	FBtr0077286
FBgn0031135	FBgn0031135	NA	FBtr0303307
FBgn0031139	FBgn0031139	NA	FBtr0077274
FBgn0031140	FBgn0031140	NA	FBtr0077239
FBgn0031142	FBgn0031142	NA	FBtr0077269
FBgn0031144	FBgn0031144	NA	FBtr0077267
FBgn0031145	FBgn0031145	NA	FBtr0077241
FBgn0031146	FBgn0031146	NA	FBtr0077266
FBgn0031149	FBgn0031149	NA	FBtr0077264
FBgn0031150	FBgn0031150	NA	FBtr0077246
FBgn0031155	FBgn0031155	NA	FBtr0300353
FBgn0031157	FBgn0031157	NA	FBtr0077260
FBgn0031161	FBgn0031161	NA	FBtr0077250
FBgn0031163	FBgn0031163	NA	FBtr0077256
FBgn0031164	FBgn0031164	NA	FBtr0077257
FBgn0031168	FBgn0031168	NA	FBtr0077237
FBgn0031170	FBgn0031170	NA	FBtr0306679
FBgn0031171	FBgn0031171	NA	FBtr0300938
FBgn0031178	FBgn0031178	NA	FBtr0077218
FBgn0031186	FBgn0031186	NA	FBtr0077368
FBgn0031187	FBgn0031187	NA	FBtr0309239
FBgn0031188	FBgn0031188	NA	FBtr0077367
FBgn0031189	FBgn0031189	CG14618	FBtr0077351
FBgn0031190	FBgn0031190	CG12576	FBtr0077365
FBgn0031191	FBgn0031191	CG14617	FBtr0100652
FBgn0031194	FBgn0031194	CG17598	FBtr0077364
FBgn0031195	FBgn0031195	CG17600	FBtr0077358
FBgn0031197	FBgn0031197	NA	FBtr0077361
FBgn0031208	FBgn0031208	NA	FBtr0300689
FBgn0031209	FBgn0031209	NA	FBtr0113008
FBgn0031213	FBgn0031213	galectin	FBtr0078101
FBgn0031214	FBgn0031214	NA	FBtr0310526
FBgn0031217	FBgn0031217	NA	FBtr0078104
FBgn0031219	FBgn0031219	NA	FBtr0078160
FBgn0031220	FBgn0031220	NA	FBtr0078159
FBgn0031224	FBgn0031224	NA	FBtr0078116
FBgn0031227	FBgn0031227	CG3709	FBtr0078147
FBgn0031228	FBgn0031228	CG11455	FBtr0306542
FBgn0031229	FBgn0031229	CG3436	FBtr0078119
FBgn0031231	FBgn0031231	NA	FBtr0078124
FBgn0031232	FBgn0031232	CG11617	FBtr0308246
FBgn0031233	FBgn0031233	NA	FBtr0078125
FBgn0031238	FBgn0031238	NA	FBtr0078141
FBgn0031239	FBgn0031239	NA	FBtr0308252
FBgn0031240	FBgn0031240	NA	FBtr0078127
FBgn0031245	FBgn0031245	CG3625	FBtr0308693
FBgn0031248	FBgn0031248	NA	FBtr0078099
FBgn0031249	FBgn0031249	NA	FBtr0078098
FBgn0031251	FBgn0031251	CG4213	FBtr0078093
FBgn0031252	FBgn0031252	NA	FBtr0078090

FBgn0031256	FBgn0031256	CG4164	FBtr0078060
FBgn0031257	FBgn0031257	CG4133	FBtr0078061
FBgn0031258	FBgn0031258	CG4297	FBtr0310034
FBgn0031263	FBgn0031263	CG2789	FBtr0078066
FBgn0031264	FBgn0031264	NA	FBtr0078076
FBgn0031265	FBgn0031265	NA	FBtr0078067
FBgn0031266	FBgn0031266	NA	FBtr0078068
FBgn0031267	FBgn0031267	NA	FBtr0078074
FBgn0031268	FBgn0031268	NA	FBtr0078069
FBgn0031270	FBgn0031270	NA	FBtr0310023
FBgn0031273	FBgn0031273	NA	FBtr0077999
FBgn0031275	FBgn0031275	NA	FBtr0308257
FBgn0031277	FBgn0031277	NA	FBtr0078005
FBgn0031281	FBgn0031281	CG3883	FBtr0078041
FBgn0031282	FBgn0031282	NA	FBtr0078010
FBgn0031283	FBgn0031283	NA	FBtr0078040
FBgn0031284	FBgn0031284	CG3876	FBtr0078039
FBgn0031285	FBgn0031285	NA	FBtr0100469
FBgn0031286	FBgn0031286	NA	FBtr0078038
FBgn0031287	FBgn0031287	NA	FBtr0078034
FBgn0031288	FBgn0031288	CG13949	FBtr0078017
FBgn0031289	FBgn0031289	NA	FBtr0078018
FBgn0031294	FBgn0031294	NA	FBtr0301417
FBgn0031295	FBgn0031295	NA	FBtr0077997
FBgn0031296	FBgn0031296	CG4415	FBtr0077951
FBgn0031298	FBgn0031298	Atg4	FBtr0077953
FBgn0031299	FBgn0031299	CG4629	FBtr0077991
FBgn0031302	FBgn0031302	NA	FBtr0077955
FBgn0031315	FBgn0031315	CG14341	FBtr0077964
FBgn0031317	FBgn0031317	NA	FBtr0077979
FBgn0031318	FBgn0031318	NA	FBtr0077966
FBgn0031319	FBgn0031319	NA	FBtr0308697
FBgn0031320	FBgn0031320	CG5126	FBtr0077978
FBgn0031321	FBgn0031321	Tgt	FBtr0077971
FBgn0031322	FBgn0031322	NA	FBtr0307056
FBgn0031323	FBgn0031323	NA	FBtr0077977
FBgn0031324	FBgn0031324	NA	FBtr0114498
FBgn0031331	FBgn0031331	NA	FBtr0305552
FBgn0031332	FBgn0031332	NA	FBtr0077948
FBgn0031333	FBgn0031333	NA	FBtr0077947
FBgn0031335	FBgn0031335	NA	FBtr0077944
FBgn0031343	FBgn0031343	NA	FBtr0077934
FBgn0031344	FBgn0031344	NA	FBtr0077933
FBgn0031345	FBgn0031345	NA	FBtr0077919
FBgn0031347	FBgn0031347	NA	FBtr0077917
FBgn0031351	FBgn0031351	CG14352	FBtr0077876
FBgn0031359	FBgn0031359	NA	FBtr0305618
FBgn0031360	FBgn0031360	NA	FBtr0077884
FBgn0031361	FBgn0031361	NA	FBtr0077904
FBgn0031367	FBgn0031367	NA	FBtr0077894
FBgn0031372	FBgn0031372	NA	FBtr0077891
FBgn0031374	FBgn0031374	CG7337	FBtr0110883
FBgn0031376	FBgn0031376	NA	FBtr0077813
FBgn0031377	FBgn0031377	NA	FBtr0077869
FBgn0031378	FBgn0031378	NA	FBtr0077814
FBgn0031379	FBgn0031379	NA	FBtr0077815
FBgn0031381	FBgn0031381	NA	FBtr0077816

FBgn0031384	FBgn0031384	NA	FBtr0301067
FBgn0031388	FBgn0031388	NA	FBtr0077827
FBgn0031390	FBgn0031390	NA	FBtr0301660
FBgn0031391	FBgn0031391	CG11723	FBtr0077852
FBgn0031395	FBgn0031395	CG10874	FBtr0077848
FBgn0031397	FBgn0031397	NA	FBtr0077845
FBgn0031398	FBgn0031398	CG10880	FBtr0077843
FBgn0031403	FBgn0031403	NA	FBtr0077841
FBgn0031405	FBgn0031405	CG4267	FBtr0077751
FBgn0031406	FBgn0031406	NA	FBtr0077804
FBgn0031407	FBgn0031407	NA	FBtr0077754
FBgn0031409	FBgn0031409	NA	FBtr0077756
FBgn0031410	FBgn0031410	NA	FBtr0077801
FBgn0031412	FBgn0031412	NA	FBtr0077758
FBgn0031414	FBgn0031414	eys	FBtr0114370
FBgn0031417	FBgn0031417	CG3597	FBtr0077792
FBgn0031418	FBgn0031418	NA	FBtr0077791
FBgn0031419	FBgn0031419	CG15390	FBtr0077764
FBgn0031424	FBgn0031424	NA	FBtr0307078
FBgn0031426	FBgn0031426	NA	FBtr0307079
FBgn0031430	FBgn0031430	NA	FBtr0077781
FBgn0031432	FBgn0031432	Cyp309a1	FBtr0077749
FBgn0031436	FBgn0031436	NA	FBtr0077745
FBgn0031439	FBgn0031439	NA	FBtr0113016
FBgn0031440	FBgn0031440	NA	FBtr0114513
FBgn0031441	FBgn0031441	NA	FBtr0077743
FBgn0031446	FBgn0031446	NA	FBtr0300986
FBgn0031450	FBgn0031450	NA	FBtr0089750
FBgn0031452	FBgn0031452	NA	FBtr0077712
FBgn0031453	FBgn0031453	CG9894	FBtr0307080
FBgn0031454	FBgn0031454	NA	FBtr0100383
FBgn0031455	FBgn0031455	NA	FBtr0077737
FBgn0031456	FBgn0031456	NA	FBtr0077715
FBgn0031458	FBgn0031458	NA	FBtr0077716
FBgn0031459	FBgn0031459	NA	FBtr0077718
FBgn0031460	FBgn0031460	NA	FBtr0077730
FBgn0031462	FBgn0031462	NA	FBtr0077722
FBgn0031463	FBgn0031463	NA	FBtr0113017
FBgn0031464	FBgn0031464	NA	FBtr0300382
FBgn0031465	FBgn0031465	NA	FBtr0077723
FBgn0031466	FBgn0031466	NA	FBtr0077691
FBgn0031467	FBgn0031467	CG2973	FBtr0077618
FBgn0031468	FBgn0031468	NA	FBtr0077619
FBgn0031469	FBgn0031469	NA	FBtr0273251
FBgn0031470	FBgn0031470	NA	FBtr0077689
FBgn0031471	FBgn0031471	NA	FBtr0300356
FBgn0031472	FBgn0031472	NA	FBtr0077620
FBgn0031476	FBgn0031476	NA	FBtr0077628
FBgn0031478	FBgn0031478	CG8814	FBtr0077629
FBgn0031479	FBgn0031479	NA	FBtr0077684
FBgn0031483	FBgn0031483	NA	FBtr0077676
FBgn0031484	FBgn0031484	NA	FBtr0077642
FBgn0031485	FBgn0031485	CG9643	FBtr0077675
FBgn0031489	FBgn0031489	NA	FBtr0077671
FBgn0031490	FBgn0031490	NA	FBtr0077647
FBgn0031491	FBgn0031491	NA	FBtr0077670
FBgn0031492	FBgn0031492	NA	FBtr0077648

FBgn0031493	FBgn0031493	CG3605	FBtr0077669
FBgn0031494	FBgn0031494	CG17219	FBtr0077650
FBgn0031495	FBgn0031495	NA	FBtr0077652
FBgn0031496	FBgn0031496	NA	FBtr0300814
FBgn0031497	FBgn0031497	NA	FBtr0077667
FBgn0031498	FBgn0031498	CG17260	FBtr0077653
FBgn0031500	FBgn0031500	CG17221	FBtr0077655
FBgn0031501	FBgn0031501	NA	FBtr0077656
FBgn0031512	FBgn0031512	NA	FBtr0077578
FBgn0031513	FBgn0031513	NA	FBtr0301033
FBgn0031515	FBgn0031515	NA	FBtr0077606
FBgn0031518	FBgn0031518	NA	FBtr0114460
FBgn0031519	FBgn0031519	NA	FBtr0077603
FBgn0031520	FBgn0031520	NA	FBtr0077583
FBgn0031522	FBgn0031522	CG3285	FBtr0077586
FBgn0031523	FBgn0031523	NA	FBtr0077587
FBgn0031528	FBgn0031528	NA	FBtr0113018
FBgn0031529	FBgn0031529	CG9662	FBtr0077594
FBgn0031530	FBgn0031530	pgant2	FBtr0077592
FBgn0031531	FBgn0031531	NA	FBtr0077590
FBgn0031535	FBgn0031535	CG12795	FBtr0113019
FBgn0031542	FBgn0031542	NA	FBtr0077568
FBgn0031545	FBgn0031545	NA	FBtr0077561
FBgn0031546	FBgn0031546	NA	FBtr0077526
FBgn0031548	FBgn0031548	NA	FBtr0089937
FBgn0031549	FBgn0031549	NA	FBtr0301590
FBgn0031554	FBgn0031554	NA	FBtr0077556
FBgn0031558	FBgn0031558	NA	FBtr0077550
FBgn0031559	FBgn0031559	NA	FBtr0077549
FBgn0031560	FBgn0031560	NA	FBtr0077547
FBgn0031561	FBgn0031561	CG16712	FBtr0077546
FBgn0031562	FBgn0031562	CG3604	FBtr0077545
FBgn0031563	FBgn0031563	NA	FBtr0077544
FBgn0031564	FBgn0031564	NA	FBtr0300299
FBgn0031566	FBgn0031566	CG2818	FBtr0077537
FBgn0031568	FBgn0031568	NA	FBtr0273188
FBgn0031571	FBgn0031571	NA	FBtr0307051
FBgn0031575	FBgn0031575	NA	FBtr0290255
FBgn0031579	FBgn0031579	NA	FBtr0077505
FBgn0031580	FBgn0031580	NA	FBtr0077506
FBgn0031581	FBgn0031581	NA	FBtr0077507
FBgn0031589	FBgn0031589	CG3714	FBtr0077483
FBgn0031591	FBgn0031591	NA	FBtr0077471
FBgn0031592	FBgn0031592	NA	FBtr0077476
FBgn0031600	FBgn0031600	NA	FBtr0077460
FBgn0031601	FBgn0031601	CG3058	FBtr0077435
FBgn0031602	FBgn0031602	NA	FBtr0077459
FBgn0031603	FBgn0031603	NA	FBtr0077438
FBgn0031604	FBgn0031604	CG15433	FBtr0077439
FBgn0031608	FBgn0031608	NA	FBtr0077441
FBgn0031609	FBgn0031609	NA	FBtr0077450
FBgn0031610	FBgn0031610	NA	FBtr0077442
FBgn0031611	FBgn0031611	CG17840	FBtr0077443
FBgn0031613	FBgn0031613	NA	FBtr0077444
FBgn0031617	FBgn0031617	NA	FBtr0300354
FBgn0031619	FBgn0031619	NA	FBtr0100560
FBgn0031621	FBgn0031621	CG15634	FBtr0077378

FBgn0031622	FBgn0031622	NA	FBtr0077422
FBgn0031626	FBgn0031626	NA	FBtr0077419
FBgn0031627	FBgn0031627	NA	FBtr0077418
FBgn0031628	FBgn0031628	NA	FBtr0077381
FBgn0031633	FBgn0031633	NA	FBtr0077415
FBgn0031634	FBgn0031634	NA	FBtr0289979
FBgn0031635	FBgn0031635	CG15626	FBtr0077412
FBgn0031636	FBgn0031636	NA	FBtr0077385
FBgn0031637	FBgn0031637	NA	FBtr0300941
FBgn0031638	FBgn0031638	NA	FBtr0077386
FBgn0031639	FBgn0031639	mRpS2	FBtr0077408
FBgn0031640	FBgn0031640	NA	FBtr0077387
FBgn0031643	FBgn0031643	CG3008	FBtr0077389
FBgn0031646	FBgn0031646	CG2837	FBtr0077402
FBgn0031649	FBgn0031649	NA	FBtr0077394
FBgn0031650	FBgn0031650	NA	FBtr0078990
FBgn0031652	FBgn0031652	NA	FBtr0079057
FBgn0031656	FBgn0031656	NA	FBtr0079052
FBgn0031657	FBgn0031657	NA	FBtr0078994
FBgn0031659	FBgn0031659	CG14043	FBtr0078995
FBgn0031660	FBgn0031660	NA	FBtr0078996
FBgn0031661	FBgn0031661	NA	FBtr0079049
FBgn0031662	FBgn0031662	CG3792	FBtr0078997
FBgn0031663	FBgn0031663	CG8891	FBtr0079048
FBgn0031664	FBgn0031664	CG8892	FBtr0079045
FBgn0031668	FBgn0031668	CG31917	FBtr0079037
FBgn0031670	FBgn0031670	NA	FBtr0079000
FBgn0031671	FBgn0031671	NA	FBtr0110984
FBgn0031673	FBgn0031673	CG31650	FBtr0079033
FBgn0031674	FBgn0031674	NA	FBtr0079007
FBgn0031675	FBgn0031675	NA	FBtr0079032
FBgn0031676	FBgn0031676	NA	FBtr0079009
FBgn0031677	FBgn0031677	NA	FBtr0079029
FBgn0031678	FBgn0031678	NA	FBtr0079028
FBgn0031681	FBgn0031681	pgant5	FBtr0111024
FBgn0031682	FBgn0031682	CG5828	FBtr0079018
FBgn0031683	FBgn0031683	CG4230	FBtr0079019
FBgn0031684	FBgn0031684	NA	FBtr0079025
FBgn0031688	FBgn0031688	NA	FBtr0079061
FBgn0031689	FBgn0031689	NA	FBtr0079062
FBgn0031690	FBgn0031690	NA	FBtr0079092
FBgn0031691	FBgn0031691	NA	FBtr0110802
FBgn0031692	FBgn0031692	NA	FBtr0079063
FBgn0031694	FBgn0031694	NA	FBtr0300456
FBgn0031696	FBgn0031696	CG14030	FBtr0079069
FBgn0031697	FBgn0031697	NA	FBtr0079082
FBgn0031698	FBgn0031698	NA	FBtr0307041
FBgn0031700	FBgn0031700	NA	FBtr0079080
FBgn0031701	FBgn0031701	NA	FBtr0079073
FBgn0031702	FBgn0031702	CG14021	FBtr0079079
FBgn0031703	FBgn0031703	NA	FBtr0079077
FBgn0031707	FBgn0031707	NA	FBtr0079094
FBgn0031708	FBgn0031708	CG7382	FBtr0079130
FBgn0031710	FBgn0031710	CG7371	FBtr0079129
FBgn0031711	FBgn0031711	CG6907	FBtr0079096
FBgn0031713	FBgn0031713	CG7277	FBtr0079128
FBgn0031715	FBgn0031715	NA	FBtr0079127

FBgn0031716	FBgn0031716	NA	FBtr0079126
FBgn0031717	FBgn0031717	Oscillin	FBtr0307062
FBgn0031718	FBgn0031718	CG14014	FBtr0114511
FBgn0031720	FBgn0031720	NA	FBtr0079121
FBgn0031721	FBgn0031721	NA	FBtr0079104
FBgn0031722	FBgn0031722	NA	FBtr0079118
FBgn0031723	FBgn0031723	NA	FBtr0079117
FBgn0031724	FBgn0031724	NA	FBtr0079109
FBgn0031725	FBgn0031725	NA	FBtr0302933
FBgn0031728	FBgn0031728	NA	FBtr0079110
FBgn0031729	FBgn0031729	NA	FBtr0305128
FBgn0031730	FBgn0031730	NA	FBtr0079133
FBgn0031731	FBgn0031731	NA	FBtr0113023
FBgn0031732	FBgn0031732	NA	FBtr0079169
FBgn0031733	FBgn0031733	NA	FBtr0079167
FBgn0031734	FBgn0031734	CG11147	FBtr0079166
FBgn0031736	FBgn0031736	NA	FBtr0079135
FBgn0031737	FBgn0031737	NA	FBtr0079164
FBgn0031738	FBgn0031738	CG9171	FBtr0301235
FBgn0031739	FBgn0031739	NA	FBtr0079160
FBgn0031740	FBgn0031740	CG7239	FBtr0079139
FBgn0031741	FBgn0031741	NA	FBtr0306841
FBgn0031746	FBgn0031746	NA	FBtr0079143
FBgn0031752	FBgn0031752	NA	FBtr0273412
FBgn0031753	FBgn0031753	NA	FBtr0079150
FBgn0031756	FBgn0031756	NA	FBtr0079237
FBgn0031757	FBgn0031757	NA	FBtr0079172
FBgn0031758	FBgn0031758	NA	FBtr0079173
FBgn0031759	FBgn0031759	lid	FBtr0307071
FBgn0031760	FBgn0031760	NA	FBtr0113024
FBgn0031764	FBgn0031764	CG9107	FBtr0079225
FBgn0031765	FBgn0031765	NA	FBtr0100665
FBgn0031766	FBgn0031766	NA	FBtr0079222
FBgn0031768	FBgn0031768	NA	FBtr0273281
FBgn0031774	FBgn0031774	NA	FBtr0079190
FBgn0031775	FBgn0031775	NA	FBtr0302213
FBgn0031776	FBgn0031776	CG13993	FBtr0079191
FBgn0031777	FBgn0031777	NA	FBtr0079213
FBgn0031778	FBgn0031778	NA	FBtr0308942
FBgn0031779	FBgn0031779	CG9175	FBtr0079211
FBgn0031781	FBgn0031781	Arc-p20	FBtr0079200
FBgn0031782	FBgn0031782	NA	FBtr0079210
FBgn0031784	FBgn0031784	NA	FBtr0079201
FBgn0031791	FBgn0031791	NA	FBtr0079243
FBgn0031792	FBgn0031792	CG13983	FBtr0079285
FBgn0031811	FBgn0031811	NA	FBtr0079256
FBgn0031813	FBgn0031813	CG9527	FBtr0114603
FBgn0031818	FBgn0031818	CG9536	FBtr0079267
FBgn0031831	FBgn0031831	NA	FBtr0079289
FBgn0031832	FBgn0031832	NA	FBtr0079332
FBgn0031834	FBgn0031834	NA	FBtr0079330
FBgn0031836	FBgn0031836	NA	FBtr0079291
FBgn0031837	FBgn0031837	NA	FBtr0113026
FBgn0031842	FBgn0031842	NA	FBtr0079295
FBgn0031849	FBgn0031849	NA	FBtr0079316
FBgn0031850	FBgn0031850	Tsp	FBtr0079312
FBgn0031851	FBgn0031851	CG11188	FBtr0079299

FBgn0031853	FBgn0031853	NA	FBtr0300056
FBgn0031856	FBgn0031856	NA	FBtr0290242
FBgn0031857	FBgn0031857	NA	FBtr0079386
FBgn0031859	FBgn0031859	NA	FBtr0309815
FBgn0031860	FBgn0031860	CG11236	FBtr0079339
FBgn0031861	FBgn0031861	NA	FBtr0309813
FBgn0031866	FBgn0031866	neuroligin	FBtr0307033
FBgn0031869	FBgn0031869	NA	FBtr0079367
FBgn0031871	FBgn0031871	CG10158	FBtr0079366
FBgn0031875	FBgn0031875	CG3430	FBtr0079350
FBgn0031876	FBgn0031876	NA	FBtr0079362
FBgn0031877	FBgn0031877	NA	FBtr0079351
FBgn0031878	FBgn0031878	NA	FBtr0079358
FBgn0031879	FBgn0031879	NA	FBtr0302162
FBgn0031882	FBgn0031882	NA	FBtr0079428
FBgn0031883	FBgn0031883	NA	FBtr0089637
FBgn0031885	FBgn0031885	NA	FBtr0110978
FBgn0031886	FBgn0031886	NA	FBtr0079424
FBgn0031887	FBgn0031887	NA	FBtr0289952
FBgn0031893	FBgn0031893	NA	FBtr0113028
FBgn0031895	FBgn0031895	CG4497	FBtr0302948
FBgn0031897	FBgn0031897	CG13784	FBtr0110799
FBgn0031902	FBgn0031902	NA	FBtr0303251
FBgn0031903	FBgn0031903	NA	FBtr0303207
FBgn0031904	FBgn0031904	CG5149	FBtr0114587
FBgn0031905	FBgn0031905	NA	FBtr0079438
FBgn0031906	FBgn0031906	CG5160	FBtr0079468
FBgn0031907	FBgn0031907	CG5171	FBtr0079439
FBgn0031908	FBgn0031908	CG5177	FBtr0079441
FBgn0031909	FBgn0031909	NA	FBtr0079467
FBgn0031910	FBgn0031910	NA	FBtr0079442
FBgn0031914	FBgn0031914	CG5973	FBtr0301152
FBgn0031918	FBgn0031918	CG6055	FBtr0079449
FBgn0031920	FBgn0031920	NA	FBtr0079451
FBgn0031929	FBgn0031929	NA	FBtr0079516
FBgn0031930	FBgn0031930	NA	FBtr0079515
FBgn0031934	FBgn0031934	NA	FBtr0079478
FBgn0031935	FBgn0031935	NA	FBtr0302518
FBgn0031936	FBgn0031936	NA	FBtr0079507
FBgn0031937	FBgn0031937	CG13795	FBtr0300709
FBgn0031939	FBgn0031939	NA	FBtr0079503
FBgn0031942	FBgn0031942	NA	FBtr0290272
FBgn0031951	FBgn0031951	NA	FBtr0079489
FBgn0031952	FBgn0031952	NA	FBtr0301331
FBgn0031955	FBgn0031955	NA	FBtr0079564
FBgn0031968	FBgn0031968	NA	FBtr0300184
FBgn0031969	FBgn0031969	NA	FBtr0114550
FBgn0031970	FBgn0031970	CG7227	FBtr0079551
FBgn0031971	FBgn0031971	CG7224	FBtr0100151
FBgn0031972	FBgn0031972	NA	FBtr0079550
FBgn0031973	FBgn0031973	NA	FBtr0079549
FBgn0031974	FBgn0031974	NA	FBtr0300761
FBgn0031976	FBgn0031976	NA	FBtr0301128
FBgn0031977	FBgn0031977	NA	FBtr0079535
FBgn0031979	FBgn0031979	CG7429	FBtr0079540
FBgn0031980	FBgn0031980	NA	FBtr0079546
FBgn0031985	FBgn0031985	NA	FBtr0301229

FBgn0031986	FBgn0031986	NA	FBtr0079575
FBgn0031987	FBgn0031987	CG12375	FBtr0079642
FBgn0031988	FBgn0031988	CG8668	FBtr0079576
FBgn0031990	FBgn0031990	CG8552	FBtr0079641
FBgn0031992	FBgn0031992	NA	FBtr0079640
FBgn0031993	FBgn0031993	CG8486	FBtr0300332
FBgn0031995	FBgn0031995	NA	FBtr0079639
FBgn0031996	FBgn0031996	NA	FBtr0079586
FBgn0031997	FBgn0031997	NA	FBtr0079587
FBgn0031999	FBgn0031999	NA	FBtr0079589
FBgn0032000	FBgn0032000	CG8372	FBtr0079634
FBgn0032001	FBgn0032001	NA	FBtr0089935
FBgn0032002	FBgn0032002	CG8353	FBtr0079593
FBgn0032003	FBgn0032003	NA	FBtr0079594
FBgn0032004	FBgn0032004	NA	FBtr0079632
FBgn0032005	FBgn0032005	Snx6	FBtr0306170
FBgn0032006	FBgn0032006	NA	FBtr0079628
FBgn0032008	FBgn0032008	NA	FBtr0079595
FBgn0032010	FBgn0032010	NA	FBtr0273380
FBgn0032019	FBgn0032019	NA	FBtr0301169
FBgn0032020	FBgn0032020	NA	FBtr0079601
FBgn0032023	FBgn0032023	NA	FBtr0079604
FBgn0032024	FBgn0032024	NA	FBtr0079605
FBgn0032026	FBgn0032026	NA	FBtr0307092
FBgn0032029	FBgn0032029	CG17292	FBtr0079649
FBgn0032032	FBgn0032032	NA	FBtr0079651
FBgn0032033	FBgn0032033	NA	FBtr0079720
FBgn0032034	FBgn0032034	NA	FBtr0079652
FBgn0032035	FBgn0032035	NA	FBtr0079719
FBgn0032036	FBgn0032036	NA	FBtr0079654
FBgn0032039	FBgn0032039	NA	FBtr0079660
FBgn0032047	FBgn0032047	NA	FBtr0079681
FBgn0032048	FBgn0032048	NA	FBtr0079700
FBgn0032049	FBgn0032049	NA	FBtr0079701
FBgn0032050	FBgn0032050	NA	FBtr0079698
FBgn0032060	FBgn0032060	NA	FBtr0079730
FBgn0032061	FBgn0032061	NA	FBtr0079731
FBgn0032065	FBgn0032065	NA	FBtr0079732
FBgn0032066	FBgn0032066	CG9463	FBtr0079744
FBgn0032067	FBgn0032067	NA	FBtr0302992
FBgn0032068	FBgn0032068	CG9466	FBtr0079742
FBgn0032069	FBgn0032069	CG9468	FBtr0079741
FBgn0032072	FBgn0032072	NA	FBtr0079738
FBgn0032074	FBgn0032074	Tsp29Fa	FBtr0300289
FBgn0032075	FBgn0032075	NA	FBtr0273420
FBgn0032076	FBgn0032076	NA	FBtr0299780
FBgn0032079	FBgn0032079	CG31886	FBtr0079787
FBgn0032082	FBgn0032082	CG18088	FBtr0079759
FBgn0032083	FBgn0032083	NA	FBtr0306803
FBgn0032085	FBgn0032085	NA	FBtr0290045
FBgn0032087	FBgn0032087	CG9568	FBtr0079765
FBgn0032088	FBgn0032088	NA	FBtr0309842
FBgn0032089	FBgn0032089	NA	FBtr0079779
FBgn0032100	FBgn0032100	NA	FBtr0079773
FBgn0032101	FBgn0032101	CG9586	FBtr0079774
FBgn0032104	FBgn0032104	NA	FBtr0079819
FBgn0032105	FBgn0032105	NA	FBtr0079818

FBgn0032109	FBgn0032109	NA	FBtr0273186
FBgn0032110	FBgn0032110	NA	FBtr0079803
FBgn0032111	FBgn0032111	NA	FBtr0079805
FBgn0032115	FBgn0032115	NA	FBtr0079813
FBgn0032116	FBgn0032116	NA	FBtr0303896
FBgn0032117	FBgn0032117	NA	FBtr0079811
FBgn0032119	FBgn0032119	NA	FBtr0079810
FBgn0032120	FBgn0032120	NA	FBtr0089853
FBgn0032122	FBgn0032122	NA	FBtr0079829
FBgn0032123	FBgn0032123	NA	FBtr0301909
FBgn0032124	FBgn0032124	NA	FBtr0079830
FBgn0032125	FBgn0032125	NA	FBtr0079831
FBgn0032127	FBgn0032127	NA	FBtr0273329
FBgn0032129	FBgn0032129	jp	FBtr0079861
FBgn0032131	FBgn0032131	NA	FBtr0303250
FBgn0032132	FBgn0032132	NA	FBtr0114535
FBgn0032135	FBgn0032135	GlcAT-S	FBtr0079842
FBgn0032136	FBgn0032136	CG15828	FBtr0303874
FBgn0032138	FBgn0032138	CG4364	FBtr0079848
FBgn0032139	FBgn0032139	NA	FBtr0079863
FBgn0032140	FBgn0032140	NA	FBtr0079864
FBgn0032142	FBgn0032142	NA	FBtr0079923
FBgn0032143	FBgn0032143	CG4017	FBtr0079867
FBgn0032144	FBgn0032144	CG17633	FBtr0079868
FBgn0032147	FBgn0032147	NA	FBtr0079869
FBgn0032149	FBgn0032149	NA	FBtr0079870
FBgn0032150	FBgn0032150	CG13123	FBtr0079917
FBgn0032151	FBgn0032151	NA	FBtr0303898
FBgn0032153	FBgn0032153	NA	FBtr0113031
FBgn0032154	FBgn0032154	NA	FBtr0079911
FBgn0032156	FBgn0032156	CG13124	FBtr0300868
FBgn0032157	FBgn0032157	NA	FBtr0100279
FBgn0032160	FBgn0032160	NA	FBtr0079877
FBgn0032161	FBgn0032161	CG4594	FBtr0079878
FBgn0032162	FBgn0032162	NA	FBtr0079879
FBgn0032163	FBgn0032163	NA	FBtr0079898
FBgn0032166	FBgn0032166	CG4619	FBtr0079883
FBgn0032168	FBgn0032168	CG13126	FBtr0079892
FBgn0032169	FBgn0032169	NA	FBtr0079884
FBgn0032170	FBgn0032170	NA	FBtr0079885
FBgn0032171	FBgn0032171	NA	FBtr0079891
FBgn0032172	FBgn0032172	CG5850	FBtr0309209
FBgn0032175	FBgn0032175	NA	FBtr0079971
FBgn0032178	FBgn0032178	NA	FBtr0079938
FBgn0032180	FBgn0032180	NA	FBtr0113032
FBgn0032181	FBgn0032181	NA	FBtr0079969
FBgn0032184	FBgn0032184	NA	FBtr0079966
FBgn0032187	FBgn0032187	CG4839	FBtr0079943
FBgn0032188	FBgn0032188	NA	FBtr0301684
FBgn0032191	FBgn0032191	NA	FBtr0079960
FBgn0032192	FBgn0032192	NA	FBtr0079959
FBgn0032193	FBgn0032193	NA	FBtr0079958
FBgn0032194	FBgn0032194	NA	FBtr0079947
FBgn0032198	FBgn0032198	eEF1delta	FBtr0079952
FBgn0032200	FBgn0032200	NA	FBtr0080086
FBgn0032204	FBgn0032204	NA	FBtr0079984
FBgn0032205	FBgn0032205	NA	FBtr0079985

FBgn0032217	FBgn0032217	CG4972	FBtr0079988
FBgn0032218	FBgn0032218	CG5381	FBtr0080060
FBgn0032219	FBgn0032219	NA	FBtr0079989
FBgn0032221	FBgn0032221	NA	FBtr0080058
FBgn0032223	FBgn0032223	NA	FBtr0079993
FBgn0032224	FBgn0032224	NA	FBtr0079998
FBgn0032225	FBgn0032225	NA	FBtr0079999
FBgn0032228	FBgn0032228	NA	FBtr0080055
FBgn0032229	FBgn0032229	NA	FBtr0080000
FBgn0032230	FBgn0032230	NA	FBtr0302490
FBgn0032231	FBgn0032231	CG5056	FBtr0080003
FBgn0032235	FBgn0032235	NA	FBtr0080006
FBgn0032240	FBgn0032240	NA	FBtr0100253
FBgn0032242	FBgn0032242	NA	FBtr0080047
FBgn0032243	FBgn0032243	Klp31E	FBtr0302040
FBgn0032244	FBgn0032244	NA	FBtr0080019
FBgn0032246	FBgn0032246	CG5168	FBtr0080020
FBgn0032247	FBgn0032247	NA	FBtr0080024
FBgn0032248	FBgn0032248	CG5343	FBtr0080044
FBgn0032251	FBgn0032251	NA	FBtr0273339
FBgn0032252	FBgn0032252	NA	FBtr0301168
FBgn0032253	FBgn0032253	NA	FBtr0321259
FBgn0032256	FBgn0032256	NA	FBtr0080032
FBgn0032258	FBgn0032258	CG7456	FBtr0080038
FBgn0032259	FBgn0032259	NA	FBtr0100565
FBgn0032260	FBgn0032260	NA	FBtr0080037
FBgn0032261	FBgn0032261	CG6094	FBtr0080091
FBgn0032262	FBgn0032262	CG7384	FBtr0080135
FBgn0032264	FBgn0032264	CG6113	FBtr0080093
FBgn0032265	FBgn0032265	NA	FBtr0080094
FBgn0032266	FBgn0032266	NA	FBtr0080095
FBgn0032268	FBgn0032268	NA	FBtr0080098
FBgn0032269	FBgn0032269	NA	FBtr0080132
FBgn0032271	FBgn0032271	NA	FBtr0080131
FBgn0032275	FBgn0032275	NA	FBtr0080101
FBgn0032276	FBgn0032276	NA	FBtr0080103
FBgn0032281	FBgn0032281	CG17107	FBtr0080107
FBgn0032282	FBgn0032282	NA	FBtr0080130
FBgn0032283	FBgn0032283	CG7296	FBtr0080129
FBgn0032284	FBgn0032284	NA	FBtr0080128
FBgn0032285	FBgn0032285	NA	FBtr0080127
FBgn0032286	FBgn0032286	NA	FBtr0080125
FBgn0032289	FBgn0032289	NA	FBtr0273319
FBgn0032290	FBgn0032290	NA	FBtr0080110
FBgn0032291	FBgn0032291	NA	FBtr0080111
FBgn0032292	FBgn0032292	NA	FBtr0080122
FBgn0032293	FBgn0032293	NA	FBtr0080112
FBgn0032294	FBgn0032294	NA	FBtr0080120
FBgn0032295	FBgn0032295	NA	FBtr0080113
FBgn0032298	FBgn0032298	CG6724	FBtr0080191
FBgn0032299	FBgn0032299	NA	FBtr0290229
FBgn0032305	FBgn0032305	NA	FBtr0080187
FBgn0032311	FBgn0032311	NA	FBtr0080144
FBgn0032312	FBgn0032312	NA	FBtr0305259
FBgn0032313	FBgn0032313	NA	FBtr0080177
FBgn0032314	FBgn0032314	NA	FBtr0301184
FBgn0032318	FBgn0032318	NA	FBtr0306114

FBgn0032321	FBgn0032321	NA	FBtr0080146
FBgn0032322	FBgn0032322	CG16743	FBtr0080147
FBgn0032330	FBgn0032330	NA	FBtr0080161
FBgn0032331	FBgn0032331	NA	FBtr0113035
FBgn0032335	FBgn0032335	NA	FBtr0080193
FBgn0032336	FBgn0032336	Ast-C	FBtr0080234
FBgn0032337	FBgn0032337	NA	FBtr0114455
FBgn0032338	FBgn0032338	NA	FBtr0304586
FBgn0032339	FBgn0032339	NA	FBtr0080196
FBgn0032340	FBgn0032340	CG6181	FBtr0080232
FBgn0032341	FBgn0032341	NA	FBtr0080230
FBgn0032354	FBgn0032354	NA	FBtr0080202
FBgn0032359	FBgn0032359	NA	FBtr0080214
FBgn0032362	FBgn0032362	NA	FBtr0305261
FBgn0032364	FBgn0032364	NA	FBtr0080239
FBgn0032365	FBgn0032365	NA	FBtr0100456
FBgn0032366	FBgn0032366	NA	FBtr0080245
FBgn0032367	FBgn0032367	NA	FBtr0080270
FBgn0032368	FBgn0032368	NA	FBtr0080268
FBgn0032370	FBgn0032370	NA	FBtr0080266
FBgn0032371	FBgn0032371	NA	FBtr0080253
FBgn0032374	FBgn0032374	NA	FBtr0300370
FBgn0032375	FBgn0032375	NA	FBtr0300384
FBgn0032377	FBgn0032377	CG14937	FBtr0080264
FBgn0032378	FBgn0032378	NA	FBtr0080259
FBgn0032381	FBgn0032381	NA	FBtr0080275
FBgn0032382	FBgn0032382	CG14935	FBtr0303220
FBgn0032385	FBgn0032385	NA	FBtr0080282
FBgn0032387	FBgn0032387	NA	FBtr0310149
FBgn0032388	FBgn0032388	NA	FBtr0301171
FBgn0032389	FBgn0032389	NA	FBtr0080316
FBgn0032390	FBgn0032390	CG16969	FBtr0080285
FBgn0032391	FBgn0032391	NA	FBtr0080286
FBgn0032393	FBgn0032393	CG12264	FBtr0080290
FBgn0032394	FBgn0032394	NA	FBtr0080313
FBgn0032404	FBgn0032404	CG5317	FBtr0080296
FBgn0032405	FBgn0032405	NA	FBtr0302029
FBgn0032407	FBgn0032407	NA	FBtr0080327
FBgn0032408	FBgn0032408	CG6712	FBtr0080364
FBgn0032412	FBgn0032412	NA	FBtr0080334
FBgn0032414	FBgn0032414	NA	FBtr0080336
FBgn0032415	FBgn0032415	NA	FBtr0080360
FBgn0032416	FBgn0032416	NA	FBtr0080337
FBgn0032419	FBgn0032419	NA	FBtr0080341
FBgn0032420	FBgn0032420	NA	FBtr0080358
FBgn0032422	FBgn0032422	NA	FBtr0273342
FBgn0032424	FBgn0032424	NA	FBtr0080354
FBgn0032429	FBgn0032429	CG5446	FBtr0080388
FBgn0032430	FBgn0032430	CG6388	FBtr0080367
FBgn0032431	FBgn0032431	NA	FBtr0080387
FBgn0032433	FBgn0032433	NA	FBtr0080384
FBgn0032434	FBgn0032434	NA	FBtr0080383
FBgn0032436	FBgn0032436	NA	FBtr0080382
FBgn0032437	FBgn0032437	NA	FBtr0329828
FBgn0032439	FBgn0032439	NA	FBtr0308801
FBgn0032442	FBgn0032442	NA	FBtr0080412
FBgn0032444	FBgn0032444	NA	FBtr0080411

FBgn0032445	FBgn0032445	NA	FBtr0300275
FBgn0032446	FBgn0032446	NA	FBtr0300273
FBgn0032447	FBgn0032447	NA	FBtr0305550
FBgn0032448	FBgn0032448	NA	FBtr0080409
FBgn0032449	FBgn0032449	NA	FBtr0080408
FBgn0032451	FBgn0032451	NA	FBtr0080397
FBgn0032452	FBgn0032452	NA	FBtr0080406
FBgn0032453	FBgn0032453	NA	FBtr0080398
FBgn0032454	FBgn0032454	NA	FBtr0080405
FBgn0032455	FBgn0032455	CG5792	FBtr0301163
FBgn0032457	FBgn0032457	NA	FBtr0080402
FBgn0032462	FBgn0032462	NA	FBtr0273445
FBgn0032467	FBgn0032467	CG9934	FBtr0080428
FBgn0032470	FBgn0032470	NA	FBtr0301366
FBgn0032471	FBgn0032471	NA	FBtr0080431
FBgn0032472	FBgn0032472	NA	FBtr0080496
FBgn0032475	FBgn0032475	CG16975	FBtr0289981
FBgn0032479	FBgn0032479	NA	FBtr0305555
FBgn0032491	FBgn0032491	NA	FBtr0273448
FBgn0032493	FBgn0032493	NA	FBtr0080481
FBgn0032497	FBgn0032497	NA	FBtr0113039
FBgn0032502	FBgn0032502	NA	FBtr0080477
FBgn0032503	FBgn0032503	NA	FBtr0329824
FBgn0032504	FBgn0032504	NA	FBtr0302309
FBgn0032505	FBgn0032505	NA	FBtr0080464
FBgn0032506	FBgn0032506	NA	FBtr0302042
FBgn0032515	FBgn0032515	NA	FBtr0100220
FBgn0032516	FBgn0032516	NA	FBtr0080525
FBgn0032518	FBgn0032518	RpL24	FBtr0080524
FBgn0032519	FBgn0032519	NA	FBtr0080523
FBgn0032520	FBgn0032520	NA	FBtr0080522
FBgn0032521	FBgn0032521	NA	FBtr0080500
FBgn0032533	FBgn0032533	NA	FBtr0080572
FBgn0032538	FBgn0032538	CG16885	FBtr0080558
FBgn0032549	FBgn0032549	NA	FBtr0301695
FBgn0032553	FBgn0032553	NA	FBtr0304003
FBgn0032554	FBgn0032554	NA	FBtr0305558
FBgn0032563	FBgn0032563	NA	FBtr0080743
FBgn0032577	FBgn0032577	NA	FBtr0080792
FBgn0032582	FBgn0032582	NA	FBtr0305564
FBgn0032587	FBgn0032587	CG5953	FBtr0080874
FBgn0032588	FBgn0032588	NA	FBtr0080872
FBgn0032590	FBgn0032590	NA	FBtr0080871
FBgn0032593	FBgn0032593	NA	FBtr0080945
FBgn0032595	FBgn0032595	NA	FBtr0080937
FBgn0032596	FBgn0032596	NA	FBtr0080936
FBgn0032597	FBgn0032597	NA	FBtr0080891
FBgn0032598	FBgn0032598	CG17905	FBtr0080892
FBgn0032600	FBgn0032600	NA	FBtr0308803
FBgn0032601	FBgn0032601	yellow-b	FBtr0080894
FBgn0032602	FBgn0032602	NA	FBtr0080933
FBgn0032603	FBgn0032603	NA	FBtr0080908
FBgn0032607	FBgn0032607	NA	FBtr0080914
FBgn0032612	FBgn0032612	NA	FBtr0080929
FBgn0032613	FBgn0032613	NA	FBtr0080928
FBgn0032618	FBgn0032618	NA	FBtr0080947
FBgn0032620	FBgn0032620	CG12288	FBtr0080957

FBgn0032625	FBgn0032625	NA	FBtr0080954
FBgn0032627	FBgn0032627	NA	FBtr0303301
FBgn0032631	FBgn0032631	NA	FBtr0302408
FBgn0032632	FBgn0032632	NA	FBtr0080970
FBgn0032634	FBgn0032634	NA	FBtr0081011
FBgn0032635	FBgn0032635	CG15141	FBtr0080979
FBgn0032636	FBgn0032636	NA	FBtr0080980
FBgn0032637	FBgn0032637	NA	FBtr0080981
FBgn0032638	FBgn0032638	NA	FBtr0081004
FBgn0032639	FBgn0032639	NA	FBtr0300902
FBgn0032640	FBgn0032640	Sgt	FBtr0305683
FBgn0032642	FBgn0032642	NA	FBtr0306162
FBgn0032643	FBgn0032643	NA	FBtr0080998
FBgn0032644	FBgn0032644	NA	FBtr0080984
FBgn0032645	FBgn0032645	NA	FBtr0080995
FBgn0032646	FBgn0032646	CG6412	FBtr0080994
FBgn0032647	FBgn0032647	NA	FBtr0080993
FBgn0032648	FBgn0032648	NA	FBtr0303652
FBgn0032649	FBgn0032649	NA	FBtr0080991
FBgn0032651	FBgn0032651	Oli	FBtr0080986
FBgn0032652	FBgn0032652	CG6870	FBtr0080989
FBgn0032654	FBgn0032654	NA	FBtr0308799
FBgn0032656	FBgn0032656	NA	FBtr0081022
FBgn0032658	FBgn0032658	NA	FBtr0081024
FBgn0032660	FBgn0032660	NA	FBtr0302030
FBgn0032665	FBgn0032665	NA	FBtr0081034
FBgn0032666	FBgn0032666	NA	FBtr0081037
FBgn0032668	FBgn0032668	NA	FBtr0081038
FBgn0032669	FBgn0032669	NA	FBtr0081048
FBgn0032670	FBgn0032670	NA	FBtr0081049
FBgn0032671	FBgn0032671	CG7200	FBtr0081062
FBgn0032673	FBgn0032673	NA	FBtr0081061
FBgn0032677	FBgn0032677	NA	FBtr0081050
FBgn0032681	FBgn0032681	CG10283	FBtr0300675
FBgn0032682	FBgn0032682	CG10176	FBtr0300674
FBgn0032683	FBgn0032683	CG10275	FBtr0300341
FBgn0032684	FBgn0032684	NA	FBtr0081068
FBgn0032698	FBgn0032698	CG10336	FBtr0273194
FBgn0032701	FBgn0032701	CG10341	FBtr0081097
FBgn0032704	FBgn0032704	NA	FBtr0081127
FBgn0032705	FBgn0032705	Grip71	FBtr0081099
FBgn0032706	FBgn0032706	NA	FBtr0114486
FBgn0032707	FBgn0032707	NA	FBtr0301647
FBgn0032709	FBgn0032709	NA	FBtr0081101
FBgn0032713	FBgn0032713	CG17323	FBtr0308573
FBgn0032715	FBgn0032715	NA	FBtr0081121
FBgn0032717	FBgn0032717	NA	FBtr0273228
FBgn0032720	FBgn0032720	NA	FBtr0089318
FBgn0032721	FBgn0032721	CG10602	FBtr0308069
FBgn0032724	FBgn0032724	CG10428	FBtr0081132
FBgn0032725	FBgn0032725	NA	FBtr0081192
FBgn0032726	FBgn0032726	CG10621	FBtr0081191
FBgn0032727	FBgn0032727	CG10623	FBtr0081190
FBgn0032728	FBgn0032728	NA	FBtr0081133
FBgn0032730	FBgn0032730	NA	FBtr0081134
FBgn0032732	FBgn0032732	CG15168	FBtr0081135
FBgn0032733	FBgn0032733	NA	FBtr0081184

FBgn0032734	FBgn0032734	NA	FBtr0081136
FBgn0032740	FBgn0032740	NA	FBtr0321270
FBgn0032748	FBgn0032748	NA	FBtr0081148
FBgn0032750	FBgn0032750	CG10495	FBtr0081150
FBgn0032751	FBgn0032751	NA	FBtr0081151
FBgn0032752	FBgn0032752	CG10702	FBtr0081174
FBgn0032753	FBgn0032753	CG17572	FBtr0081173
FBgn0032754	FBgn0032754	NA	FBtr0081171
FBgn0032755	FBgn0032755	NA	FBtr0081152
FBgn0032768	FBgn0032768	NA	FBtr0081225
FBgn0032769	FBgn0032769	NA	FBtr0081196
FBgn0032770	FBgn0032770	NA	FBtr0081197
FBgn0032771	FBgn0032771	NA	FBtr0081198
FBgn0032772	FBgn0032772	NA	FBtr0081199
FBgn0032773	FBgn0032773	CG15825	FBtr0081222
FBgn0032774	FBgn0032774	CG17549	FBtr0300823
FBgn0032775	FBgn0032775	NA	FBtr0081217
FBgn0032779	FBgn0032779	NA	FBtr0302938
FBgn0032780	FBgn0032780	NA	FBtr0081203
FBgn0032781	FBgn0032781	NA	FBtr0081205
FBgn0032782	FBgn0032782	Rab9	FBtr0081206
FBgn0032783	FBgn0032783	CG10237	FBtr0081208
FBgn0032787	FBgn0032787	NA	FBtr0302930
FBgn0032788	FBgn0032788	NA	FBtr0081240
FBgn0032789	FBgn0032789	NA	FBtr0081242
FBgn0032790	FBgn0032790	NA	FBtr0081285
FBgn0032791	FBgn0032791	NA	FBtr0081243
FBgn0032793	FBgn0032793	NA	FBtr0081284
FBgn0032796	FBgn0032796	NA	FBtr0081283
FBgn0032797	FBgn0032797	NA	FBtr0081281
FBgn0032798	FBgn0032798	CG10132	FBtr0081247
FBgn0032799	FBgn0032799	NA	FBtr0081279
FBgn0032801	FBgn0032801	NA	FBtr0303780
FBgn0032809	FBgn0032809	NA	FBtr0081256
FBgn0032810	FBgn0032810	NA	FBtr0081257
FBgn0032811	FBgn0032811	NA	FBtr0081266
FBgn0032812	FBgn0032812	CG10263	FBtr0081264
FBgn0032813	FBgn0032813	NA	FBtr0305305
FBgn0032814	FBgn0032814	CG10366	FBtr0081259
FBgn0032815	FBgn0032815	CG10462	FBtr0100351
FBgn0032816	FBgn0032816	NA	FBtr0081289
FBgn0032821	FBgn0032821	NA	FBtr0081318
FBgn0032824	FBgn0032824	NA	FBtr0081314
FBgn0032833	FBgn0032833	CG10664	FBtr0081301
FBgn0032835	FBgn0032835	NA	FBtr0081302
FBgn0032836	FBgn0032836	NA	FBtr0081303
FBgn0032838	FBgn0032838	NA	FBtr0302173
FBgn0032839	FBgn0032839	NA	FBtr0081350
FBgn0032840	FBgn0032840	NA	FBtr0081327
FBgn0032843	FBgn0032843	NA	FBtr0310522
FBgn0032845	FBgn0032845	NA	FBtr0081341
FBgn0032846	FBgn0032846	NA	FBtr0081333
FBgn0032847	FBgn0032847	NA	FBtr0081340
FBgn0032848	FBgn0032848	CG10722	FBtr0081335
FBgn0032849	FBgn0032849	mRpS18B	FBtr0081339
FBgn0032850	FBgn0032850	NA	FBtr0081336
FBgn0032853	FBgn0032853	NA	FBtr0081378

FBgn0032857	FBgn0032857	NA	FBtr0081369
FBgn0032860	FBgn0032860	NA	FBtr0307020
FBgn0032868	FBgn0032868	NA	FBtr0081382
FBgn0032869	FBgn0032869	NA	FBtr0081406
FBgn0032870	FBgn0032870	CG2608	FBtr0081384
FBgn0032871	FBgn0032871	NA	FBtr0081387
FBgn0032873	FBgn0032873	NA	FBtr0081388
FBgn0032876	FBgn0032876	NA	FBtr0081399
FBgn0032877	FBgn0032877	NA	FBtr0081391
FBgn0032878	FBgn0032878	NA	FBtr0081396
FBgn0032879	FBgn0032879	NA	FBtr0081395
FBgn0032880	FBgn0032880	CG9318	FBtr0081393
FBgn0032881	FBgn0032881	NA	FBtr0081460
FBgn0032883	FBgn0032883	CG9323	FBtr0081459
FBgn0032886	FBgn0032886	NA	FBtr0081415
FBgn0032888	FBgn0032888	NA	FBtr0081452
FBgn0032889	FBgn0032889	CG9331	FBtr0302945
FBgn0032891	FBgn0032891	NA	FBtr0290042
FBgn0032897	FBgn0032897	CG9336	FBtr0081427
FBgn0032899	FBgn0032899	CG9338	FBtr0081428
FBgn0032900	FBgn0032900	NA	FBtr0307163
FBgn0032901	FBgn0032901	NA	FBtr0081442
FBgn0032904	FBgn0032904	NA	FBtr0081439
FBgn0032906	FBgn0032906	NA	FBtr0081438
FBgn0032907	FBgn0032907	CG9272	FBtr0089944
FBgn0032908	FBgn0032908	NA	FBtr0306031
FBgn0032910	FBgn0032910	CG9265	FBtr0081507
FBgn0032913	FBgn0032913	NA	FBtr0081464
FBgn0032914	FBgn0032914	NA	FBtr0300444
FBgn0032915	FBgn0032915	NA	FBtr0114615
FBgn0032919	FBgn0032919	CG9253	FBtr0081468
FBgn0032922	FBgn0032922	NA	FBtr0081500
FBgn0032923	FBgn0032923	CG9248	FBtr0081471
FBgn0032924	FBgn0032924	NA	FBtr0081499
FBgn0032925	FBgn0032925	CG9246	FBtr0081472
FBgn0032929	FBgn0032929	NA	FBtr0081475
FBgn0032934	FBgn0032934	NA	FBtr0081488
FBgn0032935	FBgn0032935	NA	FBtr0081487
FBgn0032938	FBgn0032938	CG8671	FBtr0081483
FBgn0032940	FBgn0032940	Mio	FBtr0301457
FBgn0032943	FBgn0032943	NA	FBtr0273404
FBgn0032947	FBgn0032947	NA	FBtr0081376
FBgn0032949	FBgn0032949	NA	FBtr0304734
FBgn0032955	FBgn0032955	NA	FBtr0100511
FBgn0032957	FBgn0032957	CG2225	FBtr0304885
FBgn0032961	FBgn0032961	CG1416	FBtr0085903
FBgn0032965	FBgn0032965	NA	FBtr0085950
FBgn0032968	FBgn0032968	NA	FBtr0085930
FBgn0032969	FBgn0032969	NA	FBtr0300121
FBgn0032972	FBgn0032972	NA	FBtr0085943
FBgn0032973	FBgn0032973	NA	FBtr0299905
FBgn0032979	FBgn0032979	NA	FBtr0100407
FBgn0032981	FBgn0032981	NA	FBtr0299879
FBgn0032986	FBgn0032986	NA	FBtr0299875
FBgn0032988	FBgn0032988	NA	FBtr0085955
FBgn0032997	FBgn0032997	NA	FBtr0299924
FBgn0033000	FBgn0033000	NA	FBtr0299923

FBgn0033019	FBgn0033019	CG10395	FBtr0086087
FBgn0033027	FBgn0033027	NA	FBtr0086075
FBgn0033041	FBgn0033041	NA	FBtr0086052
FBgn0033042	FBgn0033042	NA	FBtr0086045
FBgn0033047	FBgn0033047	NA	FBtr0085966
FBgn0033049	FBgn0033049	NA	FBtr0085970
FBgn0033050	FBgn0033050	PNGase	FBtr0085971
FBgn0033052	FBgn0033052	NA	FBtr0086027
FBgn0033054	FBgn0033054	CG14591	FBtr0086026
FBgn0033055	FBgn0033055	CG7861	FBtr0085974
FBgn0033058	FBgn0033058	NA	FBtr0301687
FBgn0033059	FBgn0033059	NA	FBtr0302508
FBgn0033060	FBgn0033060	NA	FBtr0085977
FBgn0033061	FBgn0033061	NA	FBtr0086022
FBgn0033062	FBgn0033062	NA	FBtr0086020
FBgn0033063	FBgn0033063	NA	FBtr0085978
FBgn0033065	FBgn0033065	NA	FBtr0085987
FBgn0033067	FBgn0033067	NA	FBtr0086005
FBgn0033069	FBgn0033069	NA	FBtr0085990
FBgn0033072	FBgn0033072	NA	FBtr0086001
FBgn0033074	FBgn0033074	NA	FBtr0085994
FBgn0033075	FBgn0033075	Pld	FBtr0085995
FBgn0033076	FBgn0033076	NA	FBtr0305958
FBgn0033079	FBgn0033079	Fmo-2	FBtr0086148
FBgn0033081	FBgn0033081	geminin	FBtr0086147
FBgn0033085	FBgn0033085	NA	FBtr0299799
FBgn0033086	FBgn0033086	NA	FBtr0299800
FBgn0033087	FBgn0033087	NA	FBtr0086145
FBgn0033088	FBgn0033088	NA	FBtr0086118
FBgn0033089	FBgn0033089	NA	FBtr0086144
FBgn0033090	FBgn0033090	NA	FBtr0086122
FBgn0033092	FBgn0033092	NA	FBtr0086121
FBgn0033093	FBgn0033093	NA	FBtr0086123
FBgn0033095	FBgn0033095	CG3409	FBtr0302166
FBgn0033096	FBgn0033096	NA	FBtr0086139
FBgn0033097	FBgn0033097	NA	FBtr0306802
FBgn0033100	FBgn0033100	CG3420	FBtr0086135
FBgn0033101	FBgn0033101	CG9436	FBtr0086134
FBgn0033104	FBgn0033104	NA	FBtr0086212
FBgn0033107	FBgn0033107	NA	FBtr0303340
FBgn0033110	FBgn0033110	NA	FBtr0300359
FBgn0033112	FBgn0033112	NA	FBtr0310521
FBgn0033113	FBgn0033113	NA	FBtr0086162
FBgn0033115	FBgn0033115	CG9460	FBtr0309371
FBgn0033117	FBgn0033117	NA	FBtr0306613
FBgn0033121	FBgn0033121	NA	FBtr0086185
FBgn0033122	FBgn0033122	CG17002	FBtr0086170
FBgn0033124	FBgn0033124	Tsp42Ec	FBtr0086179
FBgn0033127	FBgn0033127	Tsp42Ef	FBtr0089023
FBgn0033128	FBgn0033128	Tsp42Eg	FBtr0089024
FBgn0033129	FBgn0033129	Tsp42Eh	FBtr0089026
FBgn0033130	FBgn0033130	Tsp42Ei	FBtr0089028
FBgn0033131	FBgn0033131	NA	FBtr0306743
FBgn0033132	FBgn0033132	Tsp42Ej	FBtr0089030
FBgn0033133	FBgn0033133	NA	FBtr0305678
FBgn0033139	FBgn0033139	NA	FBtr0089036
FBgn0033141	FBgn0033141	NA	FBtr0300063

FBgn0033145	FBgn0033145	NA	FBtr0112770
FBgn0033153	FBgn0033153	NA	FBtr0089049
FBgn0033154	FBgn0033154	NA	FBtr0089022
FBgn0033159	FBgn0033159	Dscam	FBtr0306767
FBgn0033164	FBgn0033164	NA	FBtr0113047
FBgn0033165	FBgn0033165	NA	FBtr0113048
FBgn0033166	FBgn0033166	CG11166	FBtr0089006
FBgn0033167	FBgn0033167	NA	FBtr0113049
FBgn0033169	FBgn0033169	CG11123	FBtr0088971
FBgn0033170	FBgn0033170	NA	FBtr0100138
FBgn0033174	FBgn0033174	NA	FBtr0088974
FBgn0033177	FBgn0033177	NA	FBtr0088999
FBgn0033178	FBgn0033178	CG11127	FBtr0088975
FBgn0033179	FBgn0033179	p47	FBtr0088997
FBgn0033182	FBgn0033182	NA	FBtr0088984
FBgn0033183	FBgn0033183	NA	FBtr0088983
FBgn0033186	FBgn0033186	NA	FBtr0088960
FBgn0033189	FBgn0033189	Cyt-b5	FBtr0088905
FBgn0033190	FBgn0033190	NA	FBtr0088907
FBgn0033191	FBgn0033191	CG1598	FBtr0088955
FBgn0033192	FBgn0033192	NA	FBtr0088909
FBgn0033194	FBgn0033194	NA	FBtr0310024
FBgn0033195	FBgn0033195	CG1360	FBtr0088951
FBgn0033196	FBgn0033196	NA	FBtr0088948
FBgn0033199	FBgn0033199	NA	FBtr0088946
FBgn0033203	FBgn0033203	NA	FBtr0088921
FBgn0033204	FBgn0033204	NA	FBtr0088922
FBgn0033205	FBgn0033205	NA	FBtr0088923
FBgn0033206	FBgn0033206	CG12042	FBtr0088944
FBgn0033207	FBgn0033207	NA	FBtr0088943
FBgn0033208	FBgn0033208	NA	FBtr0088942
FBgn0033209	FBgn0033209	CG12107	FBtr0088924
FBgn0033210	FBgn0033210	U2A	FBtr0088941
FBgn0033212	FBgn0033212	NA	FBtr0300779
FBgn0033216	FBgn0033216	NA	FBtr0088929
FBgn0033221	FBgn0033221	NA	FBtr0088829
FBgn0033222	FBgn0033222	NA	FBtr0088830
FBgn0033225	FBgn0033225	CG1550	FBtr0088899
FBgn0033226	FBgn0033226	CG1882	FBtr0088834
FBgn0033229	FBgn0033229	NA	FBtr0088841
FBgn0033232	FBgn0033232	NA	FBtr0088888
FBgn0033233	FBgn0033233	CG15835	FBtr0088886
FBgn0033234	FBgn0033234	CG8791	FBtr0304751
FBgn0033236	FBgn0033236	NA	FBtr0088880
FBgn0033238	FBgn0033238	NA	FBtr0088853
FBgn0033240	FBgn0033240	NA	FBtr0305126
FBgn0033241	FBgn0033241	CG2915	FBtr0088878
FBgn0033243	FBgn0033243	NA	FBtr0088875
FBgn0033244	FBgn0033244	NA	FBtr0301231
FBgn0033246	FBgn0033246	NA	FBtr0113054
FBgn0033250	FBgn0033250	CG14762	FBtr0304750
FBgn0033259	FBgn0033259	CG11210	FBtr0088781
FBgn0033260	FBgn0033260	NA	FBtr0301355
FBgn0033264	FBgn0033264	CG2158	FBtr0088783
FBgn0033265	FBgn0033265	NA	FBtr0100667
FBgn0033266	FBgn0033266	Socs44A	FBtr0088784
FBgn0033268	FBgn0033268	Obp44a	FBtr0088816

FBgn0033269	FBgn0033269	NA	FBtr0303583
FBgn0033271	FBgn0033271	CG8708	FBtr0307067
FBgn0033272	FBgn0033272	CG8707	FBtr0088807
FBgn0033278	FBgn0033278	NA	FBtr0088796
FBgn0033279	FBgn0033279	NA	FBtr0088802
FBgn0033280	FBgn0033280	NA	FBtr0088801
FBgn0033283	FBgn0033283	NA	FBtr0088739
FBgn0033285	FBgn0033285	NA	FBtr0089732
FBgn0033286	FBgn0033286	NA	FBtr0088765
FBgn0033289	FBgn0033289	NA	FBtr0290124
FBgn0033294	FBgn0033294	NA	FBtr0088748
FBgn0033296	FBgn0033296	NA	FBtr0088757
FBgn0033297	FBgn0033297	NA	FBtr0088752
FBgn0033301	FBgn0033301	NA	FBtr0088736
FBgn0033302	FBgn0033302	NA	FBtr0329847
FBgn0033307	FBgn0033307	NA	FBtr0088691
FBgn0033308	FBgn0033308	CG8736	FBtr0290280
FBgn0033309	FBgn0033309	NA	FBtr0088730
FBgn0033310	FBgn0033310	NA	FBtr0301542
FBgn0033312	FBgn0033312	NA	FBtr0310502
FBgn0033313	FBgn0033313	NA	FBtr0305082
FBgn0033315	FBgn0033315	NA	FBtr0088728
FBgn0033316	FBgn0033316	NA	FBtr0088695
FBgn0033323	FBgn0033323	NA	FBtr0088706
FBgn0033324	FBgn0033324	NA	FBtr0307024
FBgn0033326	FBgn0033326	NA	FBtr0307023
FBgn0033337	FBgn0033337	CG8272	FBtr0088686
FBgn0033339	FBgn0033339	NA	FBtr0088643
FBgn0033340	FBgn0033340	NA	FBtr0088644
FBgn0033341	FBgn0033341	NA	FBtr0088684
FBgn0033347	FBgn0033347	NA	FBtr0088678
FBgn0033348	FBgn0033348	CG8247	FBtr0290033
FBgn0033349	FBgn0033349	CG8243	FBtr0088652
FBgn0033350	FBgn0033350	CG8237	FBtr0088653
FBgn0033351	FBgn0033351	CG8235	FBtr0088676
FBgn0033352	FBgn0033352	NA	FBtr0088654
FBgn0033353	FBgn0033353	NA	FBtr0300706
FBgn0033354	FBgn0033354	NA	FBtr0088675
FBgn0033355	FBgn0033355	NA	FBtr0088656
FBgn0033356	FBgn0033356	CG8229	FBtr0290279
FBgn0033357	FBgn0033357	NA	FBtr0088657
FBgn0033358	FBgn0033358	NA	FBtr0088671
FBgn0033359	FBgn0033359	NA	FBtr0273385
FBgn0033362	FBgn0033362	NA	FBtr0301193
FBgn0033364	FBgn0033364	NA	FBtr0088660
FBgn0033365	FBgn0033365	NA	FBtr0088665
FBgn0033367	FBgn0033367	CG8193	FBtr0088663
FBgn0033368	FBgn0033368	NA	FBtr0088662
FBgn0033369	FBgn0033369	NA	FBtr0088661
FBgn0033371	FBgn0033371	NA	FBtr0300173
FBgn0033372	FBgn0033372	NA	FBtr0310147
FBgn0033373	FBgn0033373	NA	FBtr0088633
FBgn0033376	FBgn0033376	NA	FBtr0088565
FBgn0033378	FBgn0033378	tsu	FBtr0088567
FBgn0033379	FBgn0033379	Mys45A	FBtr0088628
FBgn0033380	FBgn0033380	Phax	FBtr0088568
FBgn0033381	FBgn0033381	NA	FBtr0302495

FBgn0033382	FBgn0033382	CG8058	FBtr0302496
FBgn0033383	FBgn0033383	NA	FBtr0088624
FBgn0033387	FBgn0033387	NA	FBtr0088622
FBgn0033388	FBgn0033388	NA	FBtr0088621
FBgn0033389	FBgn0033389	NA	FBtr0301527
FBgn0033391	FBgn0033391	CG8026	FBtr0088611
FBgn0033393	FBgn0033393	NA	FBtr0088588
FBgn0033395	FBgn0033395	NA	FBtr0088591
FBgn0033397	FBgn0033397	Cyp4p3	FBtr0088593
FBgn0033400	FBgn0033400	NA	FBtr0088600
FBgn0033401	FBgn0033401	CG1968	FBtr0088595
FBgn0033405	FBgn0033405	NA	FBtr0113059
FBgn0033408	FBgn0033408	NA	FBtr0088555
FBgn0033413	FBgn0033413	NA	FBtr0300293
FBgn0033421	FBgn0033421	NA	FBtr0310032
FBgn0033426	FBgn0033426	CG1814	FBtr0088522
FBgn0033428	FBgn0033428	NA	FBtr0088524
FBgn0033429	FBgn0033429	NA	FBtr0113060
FBgn0033431	FBgn0033431	CG1827	FBtr0088529
FBgn0033434	FBgn0033434	NA	FBtr0088532
FBgn0033437	FBgn0033437	CG12926	FBtr0088502
FBgn0033438	FBgn0033438	NA	FBtr0088501
FBgn0033439	FBgn0033439	NA	FBtr0088467
FBgn0033440	FBgn0033440	NA	FBtr0088468
FBgn0033442	FBgn0033442	NA	FBtr0088496
FBgn0033447	FBgn0033447	NA	FBtr0309880
FBgn0033448	FBgn0033448	NA	FBtr0088487
FBgn0033449	FBgn0033449	NA	FBtr0088477
FBgn0033450	FBgn0033450	NA	FBtr0088485
FBgn0033451	FBgn0033451	CG1665	FBtr0088478
FBgn0033452	FBgn0033452	CG1599	FBtr0089394
FBgn0033453	FBgn0033453	NA	FBtr0088479
FBgn0033454	FBgn0033454	CG1671	FBtr0088373
FBgn0033457	FBgn0033457	CG1675	FBtr0088375
FBgn0033459	FBgn0033459	NA	FBtr0088377
FBgn0033460	FBgn0033460	NA	FBtr0088451
FBgn0033461	FBgn0033461	NA	FBtr0088379
FBgn0033463	FBgn0033463	NA	FBtr0088450
FBgn0033464	FBgn0033464	CG1441	FBtr0088448
FBgn0033465	FBgn0033465	NA	FBtr0088384
FBgn0033466	FBgn0033466	CG12130	FBtr0273269
FBgn0033467	FBgn0033467	NA	FBtr0088441
FBgn0033468	FBgn0033468	CG1418	FBtr0088440
FBgn0033474	FBgn0033474	CG1407	FBtr0088436
FBgn0033475	FBgn0033475	CG12129	FBtr0088395
FBgn0033477	FBgn0033477	NA	FBtr0088433
FBgn0033478	FBgn0033478	NA	FBtr0088400
FBgn0033479	FBgn0033479	NA	FBtr0088431
FBgn0033480	FBgn0033480	mRpL42	FBtr0088403
FBgn0033481	FBgn0033481	NA	FBtr0088404
FBgn0033483	FBgn0033483	egr	FBtr0088406
FBgn0033484	FBgn0033484	NA	FBtr0088409
FBgn0033485	FBgn0033485	NA	FBtr0088425
FBgn0033486	FBgn0033486	NA	FBtr0304926
FBgn0033490	FBgn0033490	NA	FBtr0290287
FBgn0033491	FBgn0033491	NA	FBtr0088332
FBgn0033494	FBgn0033494	NA	FBtr0301444

FBgn0033495	FBgn0033495	CG12214	FBtr0088369
FBgn0033497	FBgn0033497	NA	FBtr0088365
FBgn0033498	FBgn0033498	NA	FBtr0088364
FBgn0033499	FBgn0033499	NA	FBtr0088334
FBgn0033500	FBgn0033500	NA	FBtr0088335
FBgn0033501	FBgn0033501	CG12911	FBtr0289962
FBgn0033502	FBgn0033502	NA	FBtr0310142
FBgn0033504	FBgn0033504	CAP	FBtr0306689
FBgn0033507	FBgn0033507	CG12909	FBtr0088348
FBgn0033512	FBgn0033512	NA	FBtr0088356
FBgn0033518	FBgn0033518	NA	FBtr0088259
FBgn0033519	FBgn0033519	NA	FBtr0305945
FBgn0033521	FBgn0033521	NA	FBtr0088315
FBgn0033523	FBgn0033523	CG12895	FBtr0088314
FBgn0033524	FBgn0033524	Cyp49a1	FBtr0304592
FBgn0033526	FBgn0033526	NA	FBtr0088310
FBgn0033527	FBgn0033527	CG11777	FBtr0088273
FBgn0033528	FBgn0033528	translin	FBtr0088306
FBgn0033529	FBgn0033529	CG17765	FBtr0088274
FBgn0033538	FBgn0033538	CG11883	FBtr0113063
FBgn0033539	FBgn0033539	NA	FBtr0088258
FBgn0033540	FBgn0033540	NA	FBtr0088212
FBgn0033541	FBgn0033541	NA	FBtr0088213
FBgn0033544	FBgn0033544	NA	FBtr0088218
FBgn0033554	FBgn0033554	NA	FBtr0088248
FBgn0033556	FBgn0033556	NA	FBtr0088247
FBgn0033562	FBgn0033562	NA	FBtr0088228
FBgn0033564	FBgn0033564	NA	FBtr0100498
FBgn0033572	FBgn0033572	NA	FBtr0088198
FBgn0033574	FBgn0033574	NA	FBtr0088211
FBgn0033589	FBgn0033589	NA	FBtr0088107
FBgn0033590	FBgn0033590	NA	FBtr0088194
FBgn0033591	FBgn0033591	CG13216	FBtr0088193
FBgn0033592	FBgn0033592	NA	FBtr0088192
FBgn0033593	FBgn0033593	NA	FBtr0088191
FBgn0033597	FBgn0033597	NA	FBtr0088190
FBgn0033598	FBgn0033598	NA	FBtr0088112
FBgn0033599	FBgn0033599	NA	FBtr0088113
FBgn0033602	FBgn0033602	CG13222	FBtr0088114
FBgn0033603	FBgn0033603	NA	FBtr0302718
FBgn0033604	FBgn0033604	NA	FBtr0088183
FBgn0033610	FBgn0033610	NA	FBtr0088177
FBgn0033614	FBgn0033614	NA	FBtr0088171
FBgn0033615	FBgn0033615	NA	FBtr0088118
FBgn0033616	FBgn0033616	NA	FBtr0088119
FBgn0033624	FBgn0033624	CG12384	FBtr0304698
FBgn0033628	FBgn0033628	CG13203	FBtr0304901
FBgn0033629	FBgn0033629	NA	FBtr0088151
FBgn0033631	FBgn0033631	NA	FBtr0100658
FBgn0033633	FBgn0033633	NA	FBtr0088133
FBgn0033635	FBgn0033635	CG7777	FBtr0305074
FBgn0033636	FBgn0033636	tou	FBtr0113066
FBgn0033638	FBgn0033638	CG9005	FBtr0306029
FBgn0033639	FBgn0033639	NA	FBtr0113069
FBgn0033644	FBgn0033644	NA	FBtr0088075
FBgn0033649	FBgn0033649	NA	FBtr0113070
FBgn0033651	FBgn0033651	NA	FBtr0088065

FBgn0033652	FBgn0033652	NA	FBtr0304623
FBgn0033653	FBgn0033653	NA	FBtr0088031
FBgn0033656	FBgn0033656	NA	FBtr0088063
FBgn0033658	FBgn0033658	NA	FBtr0088062
FBgn0033659	FBgn0033659	NA	FBtr0088061
FBgn0033661	FBgn0033661	NA	FBtr0310323
FBgn0033662	FBgn0033662	NA	FBtr0088034
FBgn0033663	FBgn0033663	ERp60	FBtr0088058
FBgn0033665	FBgn0033665	CG13189	FBtr0088038
FBgn0033669	FBgn0033669	CG8979	FBtr0088052
FBgn0033672	FBgn0033672	rho-7	FBtr0088045
FBgn0033673	FBgn0033673	CG8298	FBtr0306003
FBgn0033674	FBgn0033674	NA	FBtr0088027
FBgn0033685	FBgn0033685	NA	FBtr0088019
FBgn0033687	FBgn0033687	NA	FBtr0301127
FBgn0033688	FBgn0033688	NA	FBtr0088016
FBgn0033690	FBgn0033690	NA	FBtr0088012
FBgn0033691	FBgn0033691	NA	FBtr0088011
FBgn0033692	FBgn0033692	NA	FBtr0087989
FBgn0033696	FBgn0033696	NA	FBtr0088009
FBgn0033697	FBgn0033697	NA	FBtr0087993
FBgn0033699	FBgn0033699	NA	FBtr0088007
FBgn0033701	FBgn0033701	NA	FBtr0087995
FBgn0033702	FBgn0033702	NA	FBtr0114562
FBgn0033705	FBgn0033705	NA	FBtr0087998
FBgn0033706	FBgn0033706	NA	FBtr0088000
FBgn0033708	FBgn0033708	CG8850	FBtr0087972
FBgn0033712	FBgn0033712	NA	FBtr0087896
FBgn0033713	FBgn0033713	CG8841	FBtr0087964
FBgn0033714	FBgn0033714	NA	FBtr0087897
FBgn0033715	FBgn0033715	NA	FBtr0087899
FBgn0033716	FBgn0033716	NA	FBtr0087902
FBgn0033721	FBgn0033721	CG13159	FBtr0087912
FBgn0033723	FBgn0033723	NA	FBtr0087956
FBgn0033724	FBgn0033724	NA	FBtr0087915
FBgn0033725	FBgn0033725	CG8502	FBtr0087916
FBgn0033731	FBgn0033731	NA	FBtr0087924
FBgn0033733	FBgn0033733	NA	FBtr0087954
FBgn0033734	FBgn0033734	CG8520	FBtr0087926
FBgn0033735	FBgn0033735	CG8525	FBtr0087927
FBgn0033736	FBgn0033736	NA	FBtr0087953
FBgn0033737	FBgn0033737	CG8831	FBtr0087952
FBgn0033739	FBgn0033739	NA	FBtr0100298
FBgn0033744	FBgn0033744	NA	FBtr0113075
FBgn0033754	FBgn0033754	NA	FBtr0087891
FBgn0033755	FBgn0033755	NA	FBtr0087826
FBgn0033756	FBgn0033756	NA	FBtr0110824
FBgn0033761	FBgn0033761	NA	FBtr0087880
FBgn0033762	FBgn0033762	CG8632	FBtr0087844
FBgn0033763	FBgn0033763	CG8646	FBtr0301237
FBgn0033764	FBgn0033764	NA	FBtr0087868
FBgn0033766	FBgn0033766	NA	FBtr0306684
FBgn0033767	FBgn0033767	NA	FBtr0087848
FBgn0033769	FBgn0033769	NA	FBtr0087871
FBgn0033770	FBgn0033770	NA	FBtr0113076
FBgn0033773	FBgn0033773	NA	FBtr0087865
FBgn0033775	FBgn0033775	NA	FBtr0087820

FBgn0033777	FBgn0033777	NA	FBtr0087816
FBgn0033778	FBgn0033778	NA	FBtr0087815
FBgn0033781	FBgn0033781	NA	FBtr0087788
FBgn0033783	FBgn0033783	NA	FBtr0087806
FBgn0033784	FBgn0033784	NA	FBtr0087803
FBgn0033785	FBgn0033785	NA	FBtr0087790
FBgn0033786	FBgn0033786	CG3884	FBtr0087800
FBgn0033787	FBgn0033787	CG13321	FBtr0087792
FBgn0033788	FBgn0033788	NA	FBtr0087797
FBgn0033791	FBgn0033791	NA	FBtr0087794
FBgn0033794	FBgn0033794	NA	FBtr0087779
FBgn0033798	FBgn0033798	NA	FBtr0300670
FBgn0033799	FBgn0033799	NA	FBtr0310152
FBgn0033802	FBgn0033802	CG17724	FBtr0310499
FBgn0033806	FBgn0033806	NA	FBtr0087737
FBgn0033807	FBgn0033807	NA	FBtr0087766
FBgn0033808	FBgn0033808	NA	FBtr0087738
FBgn0033814	FBgn0033814	CG4670	FBtr0087760
FBgn0033815	FBgn0033815	NA	FBtr0087743
FBgn0033816	FBgn0033816	CG4679	FBtr0087759
FBgn0033817	FBgn0033817	NA	FBtr0087744
FBgn0033826	FBgn0033826	NA	FBtr0087754
FBgn0033827	FBgn0033827	NA	FBtr0087753
FBgn0033828	FBgn0033828	NA	FBtr0302450
FBgn0033834	FBgn0033834	NA	FBtr0087717
FBgn0033842	FBgn0033842	CG5970	FBtr0087701
FBgn0033844	FBgn0033844	NA	FBtr0087699
FBgn0033845	FBgn0033845	NA	FBtr0301387
FBgn0033846	FBgn0033846	NA	FBtr0087669
FBgn0033848	FBgn0033848	NA	FBtr0087671
FBgn0033850	FBgn0033850	NA	FBtr0087673
FBgn0033851	FBgn0033851	NA	FBtr0087674
FBgn0033853	FBgn0033853	NA	FBtr0087686
FBgn0033855	FBgn0033855	CG13333	FBtr0087676
FBgn0033856	FBgn0033856	NA	FBtr0305955
FBgn0033861	FBgn0033861	NA	FBtr0087652
FBgn0033865	FBgn0033865	NA	FBtr0087650
FBgn0033879	FBgn0033879	NA	FBtr0087644
FBgn0033880	FBgn0033880	NA	FBtr0087642
FBgn0033882	FBgn0033882	NA	FBtr0087585
FBgn0033883	FBgn0033883	NA	FBtr0087622
FBgn0033884	FBgn0033884	NA	FBtr0087586
FBgn0033885	FBgn0033885	NA	FBtr0087615
FBgn0033886	FBgn0033886	CG13349	FBtr0114493
FBgn0033887	FBgn0033887	CG6704	FBtr0087609
FBgn0033888	FBgn0033888	NA	FBtr0087608
FBgn0033889	FBgn0033889	NA	FBtr0303990
FBgn0033890	FBgn0033890	NA	FBtr0087606
FBgn0033891	FBgn0033891	NA	FBtr0087595
FBgn0033897	FBgn0033897	CG8233	FBtr0310669
FBgn0033899	FBgn0033899	CG13016	FBtr0087519
FBgn0033900	FBgn0033900	NA	FBtr0087580
FBgn0033902	FBgn0033902	NA	FBtr0087579
FBgn0033903	FBgn0033903	NA	FBtr0087521
FBgn0033904	FBgn0033904	NA	FBtr0087522
FBgn0033905	FBgn0033905	NA	FBtr0087524
FBgn0033906	FBgn0033906	NA	FBtr0302451

FBgn0033907	FBgn0033907	NA	FBtr0087578
FBgn0033911	FBgn0033911	NA	FBtr0087577
FBgn0033918	FBgn0033918	CG8531	FBtr0087538
FBgn0033919	FBgn0033919	CG8547	FBtr0301204
FBgn0033920	FBgn0033920	NA	FBtr0087540
FBgn0033921	FBgn0033921	NA	FBtr0087562
FBgn0033925	FBgn0033925	NA	FBtr0087561
FBgn0033926	FBgn0033926	NA	FBtr0087560
FBgn0033927	FBgn0033927	NA	FBtr0305617
FBgn0033928	FBgn0033928	NA	FBtr0087547
FBgn0033929	FBgn0033929	NA	FBtr0087550
FBgn0033932	FBgn0033932	NA	FBtr0087485
FBgn0033934	FBgn0033934	NA	FBtr0087517
FBgn0033935	FBgn0033935	CG10105	FBtr0087487
FBgn0033936	FBgn0033936	NA	FBtr0087488
FBgn0033945	FBgn0033945	NA	FBtr0301735
FBgn0033948	FBgn0033948	CG12863	FBtr0087500
FBgn0033950	FBgn0033950	NA	FBtr0087484
FBgn0033954	FBgn0033954	NA	FBtr0087480
FBgn0033955	FBgn0033955	NA	FBtr0300765
FBgn0033958	FBgn0033958	CG12858	FBtr0309056
FBgn0033960	FBgn0033960	CG10151	FBtr0087473
FBgn0033961	FBgn0033961	NA	FBtr0087440
FBgn0033962	FBgn0033962	CG10153	FBtr0087470
FBgn0033968	FBgn0033968	NA	FBtr0087445
FBgn0033972	FBgn0033972	NA	FBtr0087460
FBgn0033973	FBgn0033973	NA	FBtr0087449
FBgn0033978	FBgn0033978	NA	FBtr0087452
FBgn0033979	FBgn0033979	NA	FBtr0087453
FBgn0033980	FBgn0033980	NA	FBtr0300127
FBgn0033981	FBgn0033981	NA	FBtr0087456
FBgn0033983	FBgn0033983	CG10253	FBtr0303761
FBgn0033984	FBgn0033984	NA	FBtr0087407
FBgn0033985	FBgn0033985	NA	FBtr0087408
FBgn0033988	FBgn0033988	NA	FBtr0301507
FBgn0033989	FBgn0033989	NA	FBtr0087424
FBgn0033990	FBgn0033990	CG10265	FBtr0087421
FBgn0033993	FBgn0033993	NA	FBtr0087418
FBgn0033994	FBgn0033994	CG7544	FBtr0087417
FBgn0033995	FBgn0033995	NA	FBtr0087404
FBgn0033996	FBgn0033996	CG11807	FBtr0310139
FBgn0033997	FBgn0033997	NA	FBtr0087364
FBgn0033999	FBgn0033999	NA	FBtr0087401
FBgn0034000	FBgn0034000	CG11808	FBtr0087366
FBgn0034001	FBgn0034001	mRpL41	FBtr0087400
FBgn0034005	FBgn0034005	NA	FBtr0087371
FBgn0034012	FBgn0034012	NA	FBtr0114456
FBgn0034020	FBgn0034020	NA	FBtr0087325
FBgn0034022	FBgn0034022	NA	FBtr0273307
FBgn0034023	FBgn0034023	NA	FBtr0273302
FBgn0034027	FBgn0034027	NA	FBtr0087355
FBgn0034031	FBgn0034031	NA	FBtr0306568
FBgn0034032	FBgn0034032	CG8195	FBtr0087353
FBgn0034035	FBgn0034035	CG8207	FBtr0087342
FBgn0034037	FBgn0034037	NA	FBtr0087320
FBgn0034045	FBgn0034045	CG8249	FBtr0308818
FBgn0034046	FBgn0034046	NA	FBtr0308814

FBgn0034047	FBgn0034047	NA	FBtr0308816
FBgn0034050	FBgn0034050	NA	FBtr0087290
FBgn0034051	FBgn0034051	Mlf	FBtr0302149
FBgn0034053	FBgn0034053	NA	FBtr0114558
FBgn0034056	FBgn0034056	NA	FBtr0307361
FBgn0034057	FBgn0034057	NA	FBtr0087268
FBgn0034058	FBgn0034058	CG8315	FBtr0087267
FBgn0034059	FBgn0034059	CG8320	FBtr0087202
FBgn0034068	FBgn0034068	CG8400	FBtr0087260
FBgn0034069	FBgn0034069	NA	FBtr0301196
FBgn0034070	FBgn0034070	NA	FBtr0087209
FBgn0034072	FBgn0034072	NA	FBtr0306238
FBgn0034073	FBgn0034073	CG8414	FBtr0087255
FBgn0034075	FBgn0034075	NA	FBtr0305091
FBgn0034076	FBgn0034076	NA	FBtr0087222
FBgn0034081	FBgn0034081	NA	FBtr0100497
FBgn0034082	FBgn0034082	NA	FBtr0087250
FBgn0034083	FBgn0034083	NA	FBtr0302084
FBgn0034084	FBgn0034084	CG8435	FBtr0087249
FBgn0034085	FBgn0034085	NA	FBtr0289985
FBgn0034086	FBgn0034086	CG8441	FBtr0304748
FBgn0034089	FBgn0034089	CG8446	FBtr0303859
FBgn0034091	FBgn0034091	NA	FBtr0306653
FBgn0034098	FBgn0034098	NA	FBtr0087189
FBgn0034099	FBgn0034099	NA	FBtr0087188
FBgn0034103	FBgn0034103	NA	FBtr0087136
FBgn0034104	FBgn0034104	NA	FBtr0087137
FBgn0034105	FBgn0034105	NA	FBtr0087182
FBgn0034109	FBgn0034109	NA	FBtr0087179
FBgn0034110	FBgn0034110	Atg9	FBtr0087141
FBgn0034113	FBgn0034113	NA	FBtr0087178
FBgn0034114	FBgn0034114	CG4282	FBtr0087144
FBgn0034117	FBgn0034117	CG7997	FBtr0087172
FBgn0034118	FBgn0034118	Nup62	FBtr0087150
FBgn0034120	FBgn0034120	NA	FBtr0087169
FBgn0034121	FBgn0034121	NA	FBtr0087151
FBgn0034122	FBgn0034122	NA	FBtr0087167
FBgn0034126	FBgn0034126	NA	FBtr0087157
FBgn0034128	FBgn0034128	NA	FBtr0113085
FBgn0034131	FBgn0034131	NA	FBtr0087127
FBgn0034132	FBgn0034132	NA	FBtr0087087
FBgn0034134	FBgn0034134	NA	FBtr0087092
FBgn0034135	FBgn0034135	NA	FBtr0087093
FBgn0034136	FBgn0034136	DAT	FBtr0087125
FBgn0034137	FBgn0034137	CG4945	FBtr0087100
FBgn0034138	FBgn0034138	RpS15	FBtr0087124
FBgn0034140	FBgn0034140	NA	FBtr0306006
FBgn0034141	FBgn0034141	CG8311	FBtr0087121
FBgn0034142	FBgn0034142	CG8306	FBtr0087120
FBgn0034143	FBgn0034143	CG8303	FBtr0273388
FBgn0034144	FBgn0034144	NA	FBtr0087107
FBgn0034145	FBgn0034145	NA	FBtr0302206
FBgn0034151	FBgn0034151	NA	FBtr0087083
FBgn0034152	FBgn0034152	NA	FBtr0087082
FBgn0034153	FBgn0034153	NA	FBtr0087081
FBgn0034154	FBgn0034154	NA	FBtr0087021
FBgn0034155	FBgn0034155	NA	FBtr0087079

FBgn0034156	FBgn0034156	NA	FBtr0305319
FBgn0034157	FBgn0034157	NA	FBtr0087024
FBgn0034159	FBgn0034159	NA	FBtr0300552
FBgn0034160	FBgn0034160	NA	FBtr0308764
FBgn0034168	FBgn0034168	NA	FBtr0087050
FBgn0034169	FBgn0034169	NA	FBtr0087070
FBgn0034179	FBgn0034179	CG6805	FBtr0086966
FBgn0034180	FBgn0034180	NA	FBtr0114627
FBgn0034181	FBgn0034181	CG8963	FBtr0087017
FBgn0034182	FBgn0034182	NA	FBtr0086972
FBgn0034186	FBgn0034186	CG8950	FBtr0306810
FBgn0034187	FBgn0034187	CG6967	FBtr0300277
FBgn0034195	FBgn0034195	NA	FBtr0110777
FBgn0034197	FBgn0034197	NA	FBtr0086984
FBgn0034198	FBgn0034198	NA	FBtr0086985
FBgn0034199	FBgn0034199	NA	FBtr0086986
FBgn0034202	FBgn0034202	NA	FBtr0087000
FBgn0034205	FBgn0034205	NA	FBtr0086990
FBgn0034206	FBgn0034206	NA	FBtr0086996
FBgn0034210	FBgn0034210	NA	FBtr0086964
FBgn0034219	FBgn0034219	NA	FBtr0086957
FBgn0034221	FBgn0034221	NA	FBtr0086919
FBgn0034223	FBgn0034223	NA	FBtr0086954
FBgn0034224	FBgn0034224	NA	FBtr0086953
FBgn0034230	FBgn0034230	NA	FBtr0086939
FBgn0034231	FBgn0034231	CG11419	FBtr0086951
FBgn0034232	FBgn0034232	CG4866	FBtr0300602
FBgn0034237	FBgn0034237	eIF3-S9	FBtr0086942
FBgn0034240	FBgn0034240	MESR4	FBtr0113090
FBgn0034242	FBgn0034242	CG14480	FBtr0086827
FBgn0034243	FBgn0034243	Ngp	FBtr0086906
FBgn0034247	FBgn0034247	CG6484	FBtr0086903
FBgn0034248	FBgn0034248	NA	FBtr0086902
FBgn0034249	FBgn0034249	NA	FBtr0086900
FBgn0034251	FBgn0034251	NA	FBtr0086834
FBgn0034255	FBgn0034255	NA	FBtr0086896
FBgn0034259	FBgn0034259	CG6459	FBtr0086892
FBgn0034261	FBgn0034261	CG4966	FBtr0086840
FBgn0034263	FBgn0034263	NA	FBtr0086859
FBgn0034264	FBgn0034264	NA	FBtr0086860
FBgn0034265	FBgn0034265	CG6410	FBtr0086885
FBgn0034266	FBgn0034266	NA	FBtr0299815
FBgn0034267	FBgn0034267	CG4984	FBtr0301183
FBgn0034269	FBgn0034269	NA	FBtr0086883
FBgn0034272	FBgn0034272	NA	FBtr0086865
FBgn0034274	FBgn0034274	NA	FBtr0089545
FBgn0034282	FBgn0034282	NA	FBtr0300410
FBgn0034286	FBgn0034286	NA	FBtr0300215
FBgn0034288	FBgn0034288	NA	FBtr0086804
FBgn0034292	FBgn0034292	NA	FBtr0086811
FBgn0034293	FBgn0034293	NA	FBtr0086810
FBgn0034300	FBgn0034300	NA	FBtr0086732
FBgn0034304	FBgn0034304	NA	FBtr0086780
FBgn0034314	FBgn0034314	NA	FBtr0086750
FBgn0034315	FBgn0034315	CG5721	FBtr0086771
FBgn0034317	FBgn0034317	NA	FBtr0310320
FBgn0034321	FBgn0034321	NA	FBtr0086757

FBgn0034322	FBgn0034322	NA	FBtr0086758
FBgn0034323	FBgn0034323	NA	FBtr0086759
FBgn0034324	FBgn0034324	NA	FBtr0086760
FBgn0034325	FBgn0034325	NA	FBtr0307184
FBgn0034326	FBgn0034326	NA	FBtr0307185
FBgn0034327	FBgn0034327	NA	FBtr0307186
FBgn0034328	FBgn0034328	NA	FBtr0086729
FBgn0034329	FBgn0034329	NA	FBtr0086662
FBgn0034330	FBgn0034330	NA	FBtr0086663
FBgn0034331	FBgn0034331	NA	FBtr0086728
FBgn0034335	FBgn0034335	NA	FBtr0086669
FBgn0034345	FBgn0034345	NA	FBtr0306637
FBgn0034346	FBgn0034346	NA	FBtr0086691
FBgn0034350	FBgn0034350	NA	FBtr0299805
FBgn0034351	FBgn0034351	CG5190	FBtr0086696
FBgn0034352	FBgn0034352	NA	FBtr0086722
FBgn0034356	FBgn0034356	CG10924	FBtr0086699
FBgn0034360	FBgn0034360	NA	FBtr0086705
FBgn0034361	FBgn0034361	NA	FBtr0086714
FBgn0034362	FBgn0034362	NA	FBtr0086706
FBgn0034364	FBgn0034364	CG5493	FBtr0086713
FBgn0034368	FBgn0034368	CG5482	FBtr0086661
FBgn0034371	FBgn0034371	NA	FBtr0301862
FBgn0034372	FBgn0034372	Gint3	FBtr0086654
FBgn0034374	FBgn0034374	CG15086	FBtr0299528
FBgn0034381	FBgn0034381	NA	FBtr0300864
FBgn0034382	FBgn0034382	CG18609	FBtr0086643
FBgn0034383	FBgn0034383	NA	FBtr0086642
FBgn0034387	FBgn0034387	NA	FBtr0086609
FBgn0034389	FBgn0034389	NA	FBtr0110970
FBgn0034390	FBgn0034390	NA	FBtr0086640
FBgn0034396	FBgn0034396	CG15097	FBtr0303237
FBgn0034397	FBgn0034397	NA	FBtr0086617
FBgn0034398	FBgn0034398	NA	FBtr0086629
FBgn0034399	FBgn0034399	NA	FBtr0086618
FBgn0034400	FBgn0034400	NA	FBtr0086628
FBgn0034401	FBgn0034401	NA	FBtr0086627
FBgn0034402	FBgn0034402	CG15084	FBtr0086619
FBgn0034403	FBgn0034403	NA	FBtr0273238
FBgn0034405	FBgn0034405	Jheh2	FBtr0089970
FBgn0034406	FBgn0034406	NA	FBtr0086622
FBgn0034407	FBgn0034407	NA	FBtr0086621
FBgn0034408	FBgn0034408	sano	FBtr0086566
FBgn0034410	FBgn0034410	NA	FBtr0086570
FBgn0034411	FBgn0034411	NA	FBtr0302494
FBgn0034412	FBgn0034412	CG15105	FBtr0114622
FBgn0034413	FBgn0034413	NA	FBtr0086597
FBgn0034416	FBgn0034416	NA	FBtr0086574
FBgn0034418	FBgn0034418	CG15118	FBtr0113096
FBgn0034419	FBgn0034419	NA	FBtr0086580
FBgn0034420	FBgn0034420	CG10737	FBtr0290317
FBgn0034422	FBgn0034422	NA	FBtr0086505
FBgn0034425	FBgn0034425	NA	FBtr0086563
FBgn0034427	FBgn0034427	NA	FBtr0086561
FBgn0034428	FBgn0034428	NA	FBtr0300592
FBgn0034430	FBgn0034430	NA	FBtr0086560
FBgn0034432	FBgn0034432	CG7461	FBtr0300283

FBgn0034433	FBgn0034433	NA	FBtr0086558
FBgn0034434	FBgn0034434	Rgk1	FBtr0301531
FBgn0034435	FBgn0034435	NA	FBtr0086518
FBgn0034436	FBgn0034436	CG11961	FBtr0308495
FBgn0034439	FBgn0034439	NA	FBtr0086522
FBgn0034440	FBgn0034440	NA	FBtr0086523
FBgn0034441	FBgn0034441	NA	FBtr0086524
FBgn0034442	FBgn0034442	NA	FBtr0086525
FBgn0034443	FBgn0034443	NA	FBtr0086551
FBgn0034446	FBgn0034446	NA	FBtr0086534
FBgn0034447	FBgn0034447	NA	FBtr0086535
FBgn0034451	FBgn0034451	NA	FBtr0086502
FBgn0034454	FBgn0034454	NA	FBtr0086463
FBgn0034455	FBgn0034455	NA	FBtr0086464
FBgn0034459	FBgn0034459	NA	FBtr0308783
FBgn0034460	FBgn0034460	NA	FBtr0086469
FBgn0034461	FBgn0034461	NA	FBtr0086470
FBgn0034462	FBgn0034462	NA	FBtr0303023
FBgn0034463	FBgn0034463	NA	FBtr0086471
FBgn0034464	FBgn0034464	NA	FBtr0086472
FBgn0034471	FBgn0034471	NA	FBtr0086327
FBgn0034472	FBgn0034472	NA	FBtr0086329
FBgn0034474	FBgn0034474	NA	FBtr0086343
FBgn0034475	FBgn0034475	NA	FBtr0086335
FBgn0034476	FBgn0034476	Toll-7	FBtr0086336
FBgn0034478	FBgn0034478	NA	FBtr0086339
FBgn0034479	FBgn0034479	CG8654	FBtr0086340
FBgn0034481	FBgn0034481	NA	FBtr0300692
FBgn0034486	FBgn0034486	NA	FBtr0086323
FBgn0034490	FBgn0034490	NA	FBtr0086320
FBgn0034500	FBgn0034500	CG11200	FBtr0086300
FBgn0034501	FBgn0034501	CG13868	FBtr0086298
FBgn0034502	FBgn0034502	NA	FBtr0086282
FBgn0034504	FBgn0034504	CG8929	FBtr0086285
FBgn0034507	FBgn0034507	NA	FBtr0290122
FBgn0034509	FBgn0034509	NA	FBtr0113099
FBgn0034510	FBgn0034510	NA	FBtr0086295
FBgn0034512	FBgn0034512	NA	FBtr0086216
FBgn0034513	FBgn0034513	NA	FBtr0086217
FBgn0034514	FBgn0034514	NA	FBtr0086268
FBgn0034515	FBgn0034515	NA	FBtr0086267
FBgn0034517	FBgn0034517	NA	FBtr0299803
FBgn0034521	FBgn0034521	NA	FBtr0086259
FBgn0034523	FBgn0034523	CG13434	FBtr0086257
FBgn0034529	FBgn0034529	NA	FBtr0086227
FBgn0034530	FBgn0034530	CG11175	FBtr0300698
FBgn0034532	FBgn0034532	NA	FBtr0086229
FBgn0034534	FBgn0034534	NA	FBtr0086230
FBgn0034535	FBgn0034535	NA	FBtr0086231
FBgn0034537	FBgn0034537	NA	FBtr0086232
FBgn0034538	FBgn0034538	NA	FBtr0086249
FBgn0034539	FBgn0034539	NA	FBtr0086248
FBgn0034540	FBgn0034540	CG11136	FBtr0302284
FBgn0034542	FBgn0034542	NA	FBtr0086245
FBgn0034543	FBgn0034543	NA	FBtr0086236
FBgn0034548	FBgn0034548	NA	FBtr0086239
FBgn0034558	FBgn0034558	NA	FBtr0304717

FBgn0034563	FBgn0034563	NA	FBtr0300563
FBgn0034564	FBgn0034564	CG9344	FBtr0071520
FBgn0034568	FBgn0034568	NA	FBtr0273396
FBgn0034569	FBgn0034569	NA	FBtr0273414
FBgn0034570	FBgn0034570	CG10543	FBtr0071524
FBgn0034573	FBgn0034573	CG3295	FBtr0071547
FBgn0034576	FBgn0034576	NA	FBtr0071530
FBgn0034577	FBgn0034577	NA	FBtr0071532
FBgn0034578	FBgn0034578	NA	FBtr0071545
FBgn0034579	FBgn0034579	NA	FBtr0071533
FBgn0034580	FBgn0034580	NA	FBtr0071534
FBgn0034582	FBgn0034582	NA	FBtr0071542
FBgn0034592	FBgn0034592	NA	FBtr0071570
FBgn0034595	FBgn0034595	NA	FBtr0071635
FBgn0034598	FBgn0034598	NA	FBtr0113100
FBgn0034599	FBgn0034599	CG9437	FBtr0071575
FBgn0034600	FBgn0034600	NA	FBtr0071628
FBgn0034601	FBgn0034601	NA	FBtr0071627
FBgn0034602	FBgn0034602	CG15658	FBtr0071583
FBgn0034603	FBgn0034603	Glycogenin	FBtr0308066
FBgn0034605	FBgn0034605	NA	FBtr0110814
FBgn0034612	FBgn0034612	NA	FBtr0071619
FBgn0034614	FBgn0034614	NA	FBtr0071616
FBgn0034617	FBgn0034617	CG9754	FBtr0071615
FBgn0034618	FBgn0034618	NA	FBtr0071600
FBgn0034622	FBgn0034622	NA	FBtr0300455
FBgn0034623	FBgn0034623	NA	FBtr0071612
FBgn0034624	FBgn0034624	NA	FBtr0071611
FBgn0034629	FBgn0034629	Acox57D-d	FBtr0071607
FBgn0034631	FBgn0034631	NA	FBtr0071701
FBgn0034636	FBgn0034636	CG10440	FBtr0308642
FBgn0034638	FBgn0034638	CG10433	FBtr0071691
FBgn0034639	FBgn0034639	NA	FBtr0300355
FBgn0034641	FBgn0034641	NA	FBtr0304728
FBgn0034645	FBgn0034645	NA	FBtr0300679
FBgn0034649	FBgn0034649	NA	FBtr0071680
FBgn0034659	FBgn0034659	NA	FBtr0071716
FBgn0034661	FBgn0034661	CG4386	FBtr0071743
FBgn0034662	FBgn0034662	NA	FBtr0302446
FBgn0034663	FBgn0034663	NA	FBtr0071740
FBgn0034664	FBgn0034664	NA	FBtr0071739
FBgn0034666	FBgn0034666	NA	FBtr0071720
FBgn0034670	FBgn0034670	NA	FBtr0071723
FBgn0034671	FBgn0034671	NA	FBtr0071733
FBgn0034674	FBgn0034674	NA	FBtr0071726
FBgn0034681	FBgn0034681	NA	FBtr0071749
FBgn0034684	FBgn0034684	NA	FBtr0071804
FBgn0034687	FBgn0034687	NA	FBtr0071803
FBgn0034688	FBgn0034688	CG11474	FBtr0303766
FBgn0034689	FBgn0034689	CG2921	FBtr0071800
FBgn0034691	FBgn0034691	NA	FBtr0071757
FBgn0034692	FBgn0034692	NA	FBtr0071799
FBgn0034693	FBgn0034693	NA	FBtr0071797
FBgn0034694	FBgn0034694	NA	FBtr0071758
FBgn0034697	FBgn0034697	NA	FBtr0071790
FBgn0034703	FBgn0034703	NA	FBtr0071787
FBgn0034704	FBgn0034704	NA	FBtr0071767

FBgn0034705	FBgn0034705	NA	FBtr0071786
FBgn0034706	FBgn0034706	CG11275	FBtr0071769
FBgn0034707	FBgn0034707	MED16	FBtr0071770
FBgn0034708	FBgn0034708	NA	FBtr0071771
FBgn0034715	FBgn0034715	NA	FBtr0071778
FBgn0034716	FBgn0034716	Oatp58Dc	FBtr0301476
FBgn0034717	FBgn0034717	CG5819	FBtr0071812
FBgn0034720	FBgn0034720	CG11206	FBtr0071846
FBgn0034725	FBgn0034725	CG6044	FBtr0301188
FBgn0034726	FBgn0034726	NA	FBtr0301243
FBgn0034727	FBgn0034727	mRpS29	FBtr0071842
FBgn0034728	FBgn0034728	NA	FBtr0301668
FBgn0034729	FBgn0034729	CG10344	FBtr0071841
FBgn0034731	FBgn0034731	NA	FBtr0300682
FBgn0034732	FBgn0034732	NA	FBtr0071839
FBgn0034736	FBgn0034736	NA	FBtr0071835
FBgn0034740	FBgn0034740	NA	FBtr0071861
FBgn0034741	FBgn0034741	NA	FBtr0071854
FBgn0034742	FBgn0034742	CG4294	FBtr0300409
FBgn0034743	FBgn0034743	RpS16	FBtr0071855
FBgn0034744	FBgn0034744	NA	FBtr0308602
FBgn0034753	FBgn0034753	NA	FBtr0071933
FBgn0034755	FBgn0034755	NA	FBtr0071929
FBgn0034756	FBgn0034756	NA	FBtr0071928
FBgn0034758	FBgn0034758	CG13510	FBtr0309799
FBgn0034759	FBgn0034759	NA	FBtr0309801
FBgn0034761	FBgn0034761	NA	FBtr0309802
FBgn0034763	FBgn0034763	NA	FBtr0071868
FBgn0034766	FBgn0034766	NA	FBtr0071870
FBgn0034768	FBgn0034768	NA	FBtr0071871
FBgn0034770	FBgn0034770	NA	FBtr0071872
FBgn0034776	FBgn0034776	NA	FBtr0071880
FBgn0034782	FBgn0034782	NA	FBtr0302717
FBgn0034783	FBgn0034783	NA	FBtr0071893
FBgn0034785	FBgn0034785	NA	FBtr0303785
FBgn0034788	FBgn0034788	CG13532	FBtr0071899
FBgn0034789	FBgn0034789	NA	FBtr0304922
FBgn0034791	FBgn0034791	NA	FBtr0071907
FBgn0034792	FBgn0034792	CG3499	FBtr0304924
FBgn0034795	FBgn0034795	NA	FBtr0299811
FBgn0034796	FBgn0034796	NA	FBtr0299812
FBgn0034797	FBgn0034797	nahoda	FBtr0304795
FBgn0034800	FBgn0034800	NA	FBtr0303465
FBgn0034802	FBgn0034802	CG3800	FBtr0071994
FBgn0034803	FBgn0034803	CG9849	FBtr0071951
FBgn0034804	FBgn0034804	CG3831	FBtr0071993
FBgn0034807	FBgn0034807	NA	FBtr0071989
FBgn0034814	FBgn0034814	NA	FBtr0071984
FBgn0034816	FBgn0034816	CG3085	FBtr0310344
FBgn0034817	FBgn0034817	NA	FBtr0114564
FBgn0034822	FBgn0034822	NA	FBtr0071978
FBgn0034828	FBgn0034828	NA	FBtr0306080
FBgn0034829	FBgn0034829	NA	FBtr0071969
FBgn0034833	FBgn0034833	NA	FBtr0071998
FBgn0034837	FBgn0034837	NA	FBtr0072049
FBgn0034840	FBgn0034840	NA	FBtr0072046
FBgn0034841	FBgn0034841	NA	FBtr0072001

FBgn0034842	FBgn0034842	NA	FBtr0072045
FBgn0034844	FBgn0034844	NA	FBtr0072002
FBgn0034846	FBgn0034846	NA	FBtr0290285
FBgn0034849	FBgn0034849	CG3500	FBtr0072038
FBgn0034850	FBgn0034850	NA	FBtr0072006
FBgn0034856	FBgn0034856	NA	FBtr0072012
FBgn0034858	FBgn0034858	eIF2B-delta	FBtr0302315
FBgn0034860	FBgn0034860	CG9812	FBtr0306150
FBgn0034861	FBgn0034861	NA	FBtr0113111
FBgn0034867	FBgn0034867	NA	FBtr0072019
FBgn0034876	FBgn0034876	CG3957	FBtr0113112
FBgn0034882	FBgn0034882	NA	FBtr0072071
FBgn0034883	FBgn0034883	CG17664	FBtr0072107
FBgn0034884	FBgn0034884	NA	FBtr0306160
FBgn0034885	FBgn0034885	CG4019	FBtr0306384
FBgn0034886	FBgn0034886	Pde8	FBtr0302129
FBgn0034887	FBgn0034887	CG5428	FBtr0114541
FBgn0034888	FBgn0034888	NA	FBtr0072080
FBgn0034889	FBgn0034889	NA	FBtr0301508
FBgn0034897	FBgn0034897	CG11299	FBtr0072087
FBgn0034899	FBgn0034899	NA	FBtr0072088
FBgn0034900	FBgn0034900	NA	FBtr0072089
FBgn0034901	FBgn0034901	NA	FBtr0072090
FBgn0034902	FBgn0034902	CG5532	FBtr0072091
FBgn0034903	FBgn0034903	NA	FBtr0290120
FBgn0034904	FBgn0034904	NA	FBtr0072245
FBgn0034907	FBgn0034907	NA	FBtr0072123
FBgn0034911	FBgn0034911	NA	FBtr0301162
FBgn0034913	FBgn0034913	usnp	FBtr0072236
FBgn0034914	FBgn0034914	NA	FBtr0072126
FBgn0034918	FBgn0034918	NA	FBtr0072233
FBgn0034919	FBgn0034919	CG5569	FBtr0072128
FBgn0034921	FBgn0034921	Dcp1	FBtr0072223
FBgn0034922	FBgn0034922	NA	FBtr0072132
FBgn0034923	FBgn0034923	NA	FBtr0072221
FBgn0034924	FBgn0034924	CG17658	FBtr0072133
FBgn0034925	FBgn0034925	CG5339	FBtr0072220
FBgn0034928	FBgn0034928	NA	FBtr0305988
FBgn0034935	FBgn0034935	CG13565	FBtr0072209
FBgn0034942	FBgn0034942	NA	FBtr0306797
FBgn0034943	FBgn0034943	Fmo-1	FBtr0072150
FBgn0034945	FBgn0034945	CG10904	FBtr0072205
FBgn0034946	FBgn0034946	NA	FBtr0072155
FBgn0034956	FBgn0034956	NA	FBtr0072197
FBgn0034963	FBgn0034963	NA	FBtr0072190
FBgn0034964	FBgn0034964	NA	FBtr0072168
FBgn0034965	FBgn0034965	NA	FBtr0289968
FBgn0034966	FBgn0034966	NA	FBtr0113117
FBgn0034970	FBgn0034970	NA	FBtr0290258
FBgn0034971	FBgn0034971	NA	FBtr0302165
FBgn0034972	FBgn0034972	NA	FBtr0072288
FBgn0034974	FBgn0034974	CG16786	FBtr0072247
FBgn0034978	FBgn0034978	CG3257	FBtr0100568
FBgn0034979	FBgn0034979	NA	FBtr0072284
FBgn0034986	FBgn0034986	mRpS17	FBtr0072277
FBgn0034987	FBgn0034987	NA	FBtr0072260
FBgn0034988	FBgn0034988	NA	FBtr0072261

FBgn0034990	FBgn0034990	NA	FBtr0300874
FBgn0034996	FBgn0034996	NA	FBtr0072264
FBgn0034997	FBgn0034997	CG3376	FBtr0072265
FBgn0034998	FBgn0034998	NA	FBtr0300366
FBgn0035000	FBgn0035000	NA	FBtr0072289
FBgn0035001	FBgn0035001	NA	FBtr0072366
FBgn0035003	FBgn0035003	NA	FBtr0072357
FBgn0035004	FBgn0035004	NA	FBtr0072356
FBgn0035010	FBgn0035010	NA	FBtr0072298
FBgn0035016	FBgn0035016	CG4612	FBtr0306137
FBgn0035019	FBgn0035019	NA	FBtr0273359
FBgn0035020	FBgn0035020	CG13585	FBtr0072304
FBgn0035021	FBgn0035021	CG4622	FBtr0300490
FBgn0035022	FBgn0035022	NA	FBtr0072305
FBgn0035023	FBgn0035023	NA	FBtr0290219
FBgn0035024	FBgn0035024	NA	FBtr0072307
FBgn0035025	FBgn0035025	NA	FBtr0072308
FBgn0035028	FBgn0035028	Start1	FBtr0089867
FBgn0035032	FBgn0035032	NA	FBtr0072340
FBgn0035033	FBgn0035033	NA	FBtr0072315
FBgn0035040	FBgn0035040	NA	FBtr0304961
FBgn0035042	FBgn0035042	NA	FBtr0072322
FBgn0035043	FBgn0035043	CG4781	FBtr0072335
FBgn0035046	FBgn0035046	CG3683	FBtr0072324
FBgn0035047	FBgn0035047	NA	FBtr0072327
FBgn0035049	FBgn0035049	Mmp1	FBtr0304005
FBgn0035057	FBgn0035057	NA	FBtr0072371
FBgn0035059	FBgn0035059	NA	FBtr0300130
FBgn0035060	FBgn0035060	NA	FBtr0072435
FBgn0035065	FBgn0035065	CG3589	FBtr0072375
FBgn0035069	FBgn0035069	NA	FBtr0072377
FBgn0035073	FBgn0035073	CG16896	FBtr0072423
FBgn0035076	FBgn0035076	NA	FBtr0072420
FBgn0035077	FBgn0035077	CG9083	FBtr0114563
FBgn0035078	FBgn0035078	NA	FBtr0304800
FBgn0035082	FBgn0035082	CG2811	FBtr0072412
FBgn0035083	FBgn0035083	NA	FBtr0100389
FBgn0035085	FBgn0035085	NA	FBtr0072393
FBgn0035086	FBgn0035086	NA	FBtr0300763
FBgn0035087	FBgn0035087	NA	FBtr0308686
FBgn0035090	FBgn0035090	CG2736	FBtr0072403
FBgn0035091	FBgn0035091	CG3829	FBtr0072396
FBgn0035092	FBgn0035092	Nplp1	FBtr0110861
FBgn0035094	FBgn0035094	NA	FBtr0072453
FBgn0035097	FBgn0035097	NA	FBtr0305595
FBgn0035099	FBgn0035099	NA	FBtr0273353
FBgn0035100	FBgn0035100	NA	FBtr0072538
FBgn0035101	FBgn0035101	p130CAS	FBtr0301889
FBgn0035102	FBgn0035102	NA	FBtr0072537
FBgn0035103	FBgn0035103	CG7047	FBtr0301890
FBgn0035106	FBgn0035106	rno	FBtr0072532
FBgn0035107	FBgn0035107	NA	FBtr0072481
FBgn0035109	FBgn0035109	NA	FBtr0072484
FBgn0035110	FBgn0035110	NA	FBtr0072526
FBgn0035111	FBgn0035111	CG16940	FBtr0072487
FBgn0035112	FBgn0035112	NA	FBtr0304058
FBgn0035113	FBgn0035113	NA	FBtr0072524

FBgn0035120	FBgn0035120	NA	FBtr0289972
FBgn0035122	FBgn0035122	mRpL17	FBtr0310415
FBgn0035131	FBgn0035131	mthl9	FBtr0072574
FBgn0035132	FBgn0035132	NA	FBtr0306564
FBgn0035134	FBgn0035134	NA	FBtr0072546
FBgn0035136	FBgn0035136	NA	FBtr0072569
FBgn0035137	FBgn0035137	CG1233	FBtr0072549
FBgn0035138	FBgn0035138	NA	FBtr0072568
FBgn0035139	FBgn0035139	NA	FBtr0072566
FBgn0035140	FBgn0035140	NA	FBtr0072550
FBgn0035141	FBgn0035141	Cypl	FBtr0072564
FBgn0035142	FBgn0035142	NA	FBtr0072551
FBgn0035143	FBgn0035143	NA	FBtr0072553
FBgn0035144	FBgn0035144	NA	FBtr0072563
FBgn0035146	FBgn0035146	CG13893	FBtr0072562
FBgn0035147	FBgn0035147	CG12030	FBtr0072556
FBgn0035148	FBgn0035148	CG3402	FBtr0072559
FBgn0035149	FBgn0035149	NA	FBtr0072558
FBgn0035151	FBgn0035151	NA	FBtr0072613
FBgn0035154	FBgn0035154	CG3344	FBtr0072609
FBgn0035155	FBgn0035155	RabX6	FBtr0072577
FBgn0035158	FBgn0035158	NA	FBtr0072605
FBgn0035159	FBgn0035159	NA	FBtr0072602
FBgn0035161	FBgn0035161	NA	FBtr0072599
FBgn0035164	FBgn0035164	NA	FBtr0072596
FBgn0035165	FBgn0035165	CG13887	FBtr0304797
FBgn0035166	FBgn0035166	NA	FBtr0300712
FBgn0035171	FBgn0035171	NA	FBtr0300345
FBgn0035176	FBgn0035176	NA	FBtr0289973
FBgn0035178	FBgn0035178	NA	FBtr0072618
FBgn0035179	FBgn0035179	CG12038	FBtr0072619
FBgn0035181	FBgn0035181	NA	FBtr0072621
FBgn0035186	FBgn0035186	CG13912	FBtr0072625
FBgn0035187	FBgn0035187	NA	FBtr0072638
FBgn0035189	FBgn0035189	CG9119	FBtr0072635
FBgn0035192	FBgn0035192	CG9194	FBtr0072651
FBgn0035193	FBgn0035193	CG9192	FBtr0072734
FBgn0035194	FBgn0035194	NA	FBtr0072731
FBgn0035195	FBgn0035195	Sac1	FBtr0072652
FBgn0035196	FBgn0035196	NA	FBtr0072653
FBgn0035197	FBgn0035197	NA	FBtr0072655
FBgn0035198	FBgn0035198	NA	FBtr0100647
FBgn0035202	FBgn0035202	NA	FBtr0072663
FBgn0035206	FBgn0035206	CG9186	FBtr0072727
FBgn0035207	FBgn0035207	NA	FBtr0072671
FBgn0035208	FBgn0035208	CG9184	FBtr0072725
FBgn0035209	FBgn0035209	NA	FBtr0072678
FBgn0035216	FBgn0035216	NA	FBtr0072692
FBgn0035217	FBgn0035217	NA	FBtr0072693
FBgn0035218	FBgn0035218	NA	FBtr0072720
FBgn0035236	FBgn0035236	CG12004	FBtr0072745
FBgn0035237	FBgn0035237	NA	FBtr0303993
FBgn0035238	FBgn0035238	CG12104	FBtr0072825
FBgn0035239	FBgn0035239	NA	FBtr0100190
FBgn0035240	FBgn0035240	NA	FBtr0091793
FBgn0035243	FBgn0035243	NA	FBtr0072823
FBgn0035245	FBgn0035245	NA	FBtr0072821

FBgn0035246	FBgn0035246	CG13928	FBtr0072822
FBgn0035247	FBgn0035247	NA	FBtr0072814
FBgn0035248	FBgn0035248	NA	FBtr0072759
FBgn0035249	FBgn0035249	CG17249	FBtr0072810
FBgn0035251	FBgn0035251	CG7967	FBtr0072809
FBgn0035252	FBgn0035252	CG7970	FBtr0072762
FBgn0035253	FBgn0035253	NA	FBtr0303107
FBgn0035254	FBgn0035254	CG7974	FBtr0072763
FBgn0035255	FBgn0035255	NA	FBtr0301528
FBgn0035257	FBgn0035257	CG12011	FBtr0072764
FBgn0035258	FBgn0035258	NA	FBtr0072802
FBgn0035260	FBgn0035260	NA	FBtr0303103
FBgn0035262	FBgn0035262	NA	FBtr0072800
FBgn0035263	FBgn0035263	NA	FBtr0072766
FBgn0035264	FBgn0035264	Oseg4	FBtr0072797
FBgn0035266	FBgn0035266	NA	FBtr0072767
FBgn0035267	FBgn0035267	NA	FBtr0072773
FBgn0035268	FBgn0035268	CG8001	FBtr0072774
FBgn0035279	FBgn0035279	NA	FBtr0299561
FBgn0035280	FBgn0035280	CG13935	FBtr0299562
FBgn0035282	FBgn0035282	NA	FBtr0303155
FBgn0035283	FBgn0035283	CG12024	FBtr0072832
FBgn0035285	FBgn0035285	CG12025	FBtr0072834
FBgn0035286	FBgn0035286	NA	FBtr0303153
FBgn0035287	FBgn0035287	CG13937	FBtr0300717
FBgn0035290	FBgn0035290	NA	FBtr0273261
FBgn0035294	FBgn0035294	CG1017	FBtr0072841
FBgn0035295	FBgn0035295	CG5690	FBtr0072842
FBgn0035299	FBgn0035299	NA	FBtr0072852
FBgn0035300	FBgn0035300	NA	FBtr0072845
FBgn0035308	FBgn0035308	NA	FBtr0114630
FBgn0035309	FBgn0035309	NA	FBtr0072916
FBgn0035312	FBgn0035312	NA	FBtr0072913
FBgn0035313	FBgn0035313	NA	FBtr0308229
FBgn0035321	FBgn0035321	NA	FBtr0072905
FBgn0035323	FBgn0035323	NA	FBtr0072878
FBgn0035325	FBgn0035325	CG13806	FBtr0072879
FBgn0035328	FBgn0035328	NA	FBtr0072883
FBgn0035329	FBgn0035329	NA	FBtr0113126
FBgn0035330	FBgn0035330	NA	FBtr0113127
FBgn0035332	FBgn0035332	NA	FBtr0072888
FBgn0035333	FBgn0035333	NA	FBtr0072901
FBgn0035334	FBgn0035334	CG8993	FBtr0072889
FBgn0035335	FBgn0035335	mRpL23	FBtr0289966
FBgn0035336	FBgn0035336	NA	FBtr0072890
FBgn0035337	FBgn0035337	NA	FBtr0072899
FBgn0035338	FBgn0035338	NA	FBtr0072891
FBgn0035343	FBgn0035343	NA	FBtr0072968
FBgn0035344	FBgn0035344	NA	FBtr0072967
FBgn0035348	FBgn0035348	NA	FBtr0306577
FBgn0035355	FBgn0035355	CG16985	FBtr0072929
FBgn0035356	FBgn0035356	NA	FBtr0072930
FBgn0035357	FBgn0035357	NA	FBtr0290340
FBgn0035358	FBgn0035358	NA	FBtr0072949
FBgn0035359	FBgn0035359	NA	FBtr0072937
FBgn0035360	FBgn0035360	CG1246	FBtr0303022
FBgn0035364	FBgn0035364	NA	FBtr0273408

FBgn0035370	FBgn0035370	CG1240	FBtr0072969
FBgn0035380	FBgn0035380	NA	FBtr0073010
FBgn0035382	FBgn0035382	NA	FBtr0301497
FBgn0035388	FBgn0035388	CG2162	FBtr0072981
FBgn0035397	FBgn0035397	CG11486	FBtr0072991
FBgn0035398	FBgn0035398	NA	FBtr0073124
FBgn0035399	FBgn0035399	NA	FBtr0307168
FBgn0035400	FBgn0035400	CG11537	FBtr0113132
FBgn0035401	FBgn0035401	CG1291	FBtr0073023
FBgn0035402	FBgn0035402	CG12082	FBtr0073114
FBgn0035404	FBgn0035404	NA	FBtr0073113
FBgn0035407	FBgn0035407	NA	FBtr0073112
FBgn0035409	FBgn0035409	NA	FBtr0073111
FBgn0035410	FBgn0035410	NA	FBtr0307018
FBgn0035411	FBgn0035411	NA	FBtr0073035
FBgn0035412	FBgn0035412	NA	FBtr0073038
FBgn0035413	FBgn0035413	NA	FBtr0073039
FBgn0035414	FBgn0035414	CG14965	FBtr0073109
FBgn0035415	FBgn0035415	NA	FBtr0073108
FBgn0035420	FBgn0035420	NA	FBtr0302302
FBgn0035421	FBgn0035421	CG12034	FBtr0073043
FBgn0035422	FBgn0035422	NA	FBtr0073095
FBgn0035423	FBgn0035423	CG17737	FBtr0073044
FBgn0035424	FBgn0035424	NA	FBtr0073046
FBgn0035425	FBgn0035425	CG17746	FBtr0073094
FBgn0035426	FBgn0035426	NA	FBtr0073092
FBgn0035427	FBgn0035427	NA	FBtr0073050
FBgn0035428	FBgn0035428	CG14960	FBtr0073051
FBgn0035429	FBgn0035429	CG12017	FBtr0308311
FBgn0035430	FBgn0035430	CG12009	FBtr0073053
FBgn0035434	FBgn0035434	NA	FBtr0073062
FBgn0035435	FBgn0035435	NA	FBtr0300753
FBgn0035436	FBgn0035436	CG12016	FBtr0073063
FBgn0035437	FBgn0035437	NA	FBtr0073083
FBgn0035438	FBgn0035438	PHGPx	FBtr0073066
FBgn0035439	FBgn0035439	NA	FBtr0073069
FBgn0035440	FBgn0035440	NA	FBtr0073082
FBgn0035444	FBgn0035444	NA	FBtr0300749
FBgn0035445	FBgn0035445	NA	FBtr0073077
FBgn0035449	FBgn0035449	NA	FBtr0073143
FBgn0035452	FBgn0035452	CG10359	FBtr0273204
FBgn0035453	FBgn0035453	NA	FBtr0300062
FBgn0035454	FBgn0035454	NA	FBtr0113134
FBgn0035455	FBgn0035455	NA	FBtr0073139
FBgn0035458	FBgn0035458	NA	FBtr0303094
FBgn0035461	FBgn0035461	NA	FBtr0073185
FBgn0035462	FBgn0035462	CG1120	FBtr0073148
FBgn0035464	FBgn0035464	CG12006	FBtr0073149
FBgn0035468	FBgn0035468	NA	FBtr0114512
FBgn0035469	FBgn0035469	NA	FBtr0073158
FBgn0035470	FBgn0035470	NA	FBtr0073180
FBgn0035473	FBgn0035473	NA	FBtr0073176
FBgn0035475	FBgn0035475	NA	FBtr0073173
FBgn0035476	FBgn0035476	NA	FBtr0073172
FBgn0035479	FBgn0035479	NA	FBtr0073170
FBgn0035480	FBgn0035480	NA	FBtr0290224
FBgn0035482	FBgn0035482	NA	FBtr0289977

FBgn0035483	FBgn0035483	NA	FBtr0073193
FBgn0035484	FBgn0035484	NA	FBtr0073284
FBgn0035486	FBgn0035486	NA	FBtr0073197
FBgn0035488	FBgn0035488	NA	FBtr0114509
FBgn0035495	FBgn0035495	NA	FBtr0308363
FBgn0035496	FBgn0035496	NA	FBtr0073208
FBgn0035497	FBgn0035497	CG14995	FBtr0073271
FBgn0035498	FBgn0035498	NA	FBtr0073210
FBgn0035499	FBgn0035499	Chd64	FBtr0073270
FBgn0035500	FBgn0035500	NA	FBtr0113135
FBgn0035504	FBgn0035504	NA	FBtr0301645
FBgn0035505	FBgn0035505	NA	FBtr0300621
FBgn0035508	FBgn0035508	CG15005	FBtr0073255
FBgn0035510	FBgn0035510	CG15006	FBtr0073254
FBgn0035511	FBgn0035511	NA	FBtr0073253
FBgn0035512	FBgn0035512	NA	FBtr0073252
FBgn0035513	FBgn0035513	CG1259	FBtr0073223
FBgn0035517	FBgn0035517	NA	FBtr0073228
FBgn0035518	FBgn0035518	CG15011	FBtr0073244
FBgn0035521	FBgn0035521	NA	FBtr0113137
FBgn0035525	FBgn0035525	NA	FBtr0300579
FBgn0035526	FBgn0035526	CG1316	FBtr0073233
FBgn0035528	FBgn0035528	NA	FBtr0073240
FBgn0035529	FBgn0035529	CG1319	FBtr0300775
FBgn0035532	FBgn0035532	NA	FBtr0073340
FBgn0035533	FBgn0035533	NA	FBtr0308201
FBgn0035534	FBgn0035534	mRpS6	FBtr0306093
FBgn0035537	FBgn0035537	NA	FBtr0073338
FBgn0035538	FBgn0035538	NA	FBtr0100185
FBgn0035539	FBgn0035539	CG7447	FBtr0073291
FBgn0035540	FBgn0035540	NA	FBtr0073293
FBgn0035541	FBgn0035541	NA	FBtr0113139
FBgn0035542	FBgn0035542	CG11347	FBtr0308033
FBgn0035543	FBgn0035543	NA	FBtr0073298
FBgn0035545	FBgn0035545	NA	FBtr0073302
FBgn0035546	FBgn0035546	NA	FBtr0073334
FBgn0035548	FBgn0035548	NA	FBtr0290225
FBgn0035550	FBgn0035550	NA	FBtr0303005
FBgn0035552	FBgn0035552	NA	FBtr0073307
FBgn0035555	FBgn0035555	NA	FBtr0073327
FBgn0035557	FBgn0035557	CG11353	FBtr0073309
FBgn0035558	FBgn0035558	NA	FBtr0113143
FBgn0035567	FBgn0035567	NA	FBtr0073367
FBgn0035568	FBgn0035568	NA	FBtr0073366
FBgn0035569	FBgn0035569	NA	FBtr0073365
FBgn0035571	FBgn0035571	NA	FBtr0073363
FBgn0035574	FBgn0035574	Gef64C	FBtr0073345
FBgn0035577	FBgn0035577	NA	FBtr0073358
FBgn0035578	FBgn0035578	NA	FBtr0308822
FBgn0035581	FBgn0035581	NA	FBtr0289982
FBgn0035585	FBgn0035585	NA	FBtr0073372
FBgn0035587	FBgn0035587	CG4623	FBtr0077167
FBgn0035588	FBgn0035588	CG10672	FBtr0077179
FBgn0035589	FBgn0035589	NA	FBtr0077166
FBgn0035590	FBgn0035590	NA	FBtr0077180
FBgn0035591	FBgn0035591	NA	FBtr0077165
FBgn0035592	FBgn0035592	NA	FBtr0077181

FBgn0035593	FBgn0035593	NA	FBtr0077164
FBgn0035598	FBgn0035598	NA	FBtr0077171
FBgn0035600	FBgn0035600	NA	FBtr0077144
FBgn0035601	FBgn0035601	Uev1A	FBtr0077155
FBgn0035603	FBgn0035603	NA	FBtr0077154
FBgn0035607	FBgn0035607	NA	FBtr0077148
FBgn0035608	FBgn0035608	NA	FBtr0077152
FBgn0035611	FBgn0035611	NA	FBtr0306593
FBgn0035612	FBgn0035612	CG10625	FBtr0273231
FBgn0035617	FBgn0035617	NA	FBtr0077085
FBgn0035619	FBgn0035619	NA	FBtr0077129
FBgn0035622	FBgn0035622	CG10590	FBtr0077127
FBgn0035623	FBgn0035623	NA	FBtr0077088
FBgn0035624	FBgn0035624	NA	FBtr0077126
FBgn0035630	FBgn0035630	NA	FBtr0077121
FBgn0035631	FBgn0035631	NA	FBtr0077098
FBgn0035632	FBgn0035632	NA	FBtr0077120
FBgn0035635	FBgn0035635	NA	FBtr0091709
FBgn0035636	FBgn0035636	NA	FBtr0077117
FBgn0035638	FBgn0035638	Tektin-C	FBtr0113147
FBgn0035639	FBgn0035639	CG5537	FBtr0310534
FBgn0035640	FBgn0035640	NA	FBtr0077113
FBgn0035641	FBgn0035641	NA	FBtr0077025
FBgn0035642	FBgn0035642	NA	FBtr0300903
FBgn0035645	FBgn0035645	NA	FBtr0077029
FBgn0035647	FBgn0035647	NA	FBtr0305273
FBgn0035648	FBgn0035648	NA	FBtr0113148
FBgn0035649	FBgn0035649	NA	FBtr0077077
FBgn0035657	FBgn0035657	NA	FBtr0077069
FBgn0035665	FBgn0035665	NA	FBtr0077041
FBgn0035666	FBgn0035666	NA	FBtr0077042
FBgn0035667	FBgn0035667	NA	FBtr0077067
FBgn0035673	FBgn0035673	NA	FBtr0077047
FBgn0035675	FBgn0035675	CG6610	FBtr0077048
FBgn0035678	FBgn0035678	NA	FBtr0077064
FBgn0035679	FBgn0035679	CG10467	FBtr0077063
FBgn0035690	FBgn0035690	NA	FBtr0077018
FBgn0035691	FBgn0035691	NA	FBtr0076999
FBgn0035694	FBgn0035694	NA	FBtr0077013
FBgn0035697	FBgn0035697	NA	FBtr0077009
FBgn0035699	FBgn0035699	NA	FBtr0077007
FBgn0035702	FBgn0035702	NA	FBtr0076987
FBgn0035703	FBgn0035703	NA	FBtr0303036
FBgn0035704	FBgn0035704	NA	FBtr0076986
FBgn0035708	FBgn0035708	CG8398	FBtr0076981
FBgn0035709	FBgn0035709	NA	FBtr0076963
FBgn0035711	FBgn0035711	NA	FBtr0076942
FBgn0035719	FBgn0035719	CG14821	FBtr0076895
FBgn0035720	FBgn0035720	NA	FBtr0300937
FBgn0035721	FBgn0035721	CG9948	FBtr0076896
FBgn0035722	FBgn0035722	CG10075	FBtr0076939
FBgn0035727	FBgn0035727	NA	FBtr0076935
FBgn0035730	FBgn0035730	NA	FBtr0076932
FBgn0035733	FBgn0035733	NA	FBtr0076931
FBgn0035734	FBgn0035734	NA	FBtr0076908
FBgn0035735	FBgn0035735	NA	FBtr0076929
FBgn0035741	FBgn0035741	NA	FBtr0076912

FBgn0035742	FBgn0035742	CG8629	FBtr0076925
FBgn0035743	FBgn0035743	NA	FBtr0076913
FBgn0035750	FBgn0035750	NA	FBtr0113151
FBgn0035751	FBgn0035751	NA	FBtr0273403
FBgn0035754	FBgn0035754	NA	FBtr0076889
FBgn0035755	FBgn0035755	CG14830	FBtr0309298
FBgn0035756	FBgn0035756	NA	FBtr0290001
FBgn0035765	FBgn0035765	CG8600	FBtr0300197
FBgn0035768	FBgn0035768	CG14834	FBtr0076880
FBgn0035769	FBgn0035769	CTCF	FBtr0076878
FBgn0035770	FBgn0035770	NA	FBtr0273399
FBgn0035771	FBgn0035771	NA	FBtr0076847
FBgn0035772	FBgn0035772	NA	FBtr0302548
FBgn0035776	FBgn0035776	NA	FBtr0076869
FBgn0035782	FBgn0035782	NA	FBtr0076866
FBgn0035785	FBgn0035785	NA	FBtr0273394
FBgn0035786	FBgn0035786	NA	FBtr0302557
FBgn0035788	FBgn0035788	NA	FBtr0076862
FBgn0035792	FBgn0035792	NA	FBtr0076830
FBgn0035793	FBgn0035793	NA	FBtr0290026
FBgn0035797	FBgn0035797	NA	FBtr0290223
FBgn0035798	FBgn0035798	CG7526	FBtr0301222
FBgn0035799	FBgn0035799	CG14838	FBtr0076801
FBgn0035800	FBgn0035800	NA	FBtr0076824
FBgn0035802	FBgn0035802	CG33275	FBtr0089850
FBgn0035805	FBgn0035805	CG7506	FBtr0076822
FBgn0035806	FBgn0035806	NA	FBtr0076807
FBgn0035807	FBgn0035807	NA	FBtr0076821
FBgn0035811	FBgn0035811	CG12262	FBtr0076808
FBgn0035812	FBgn0035812	CG7457	FBtr0076814
FBgn0035813	FBgn0035813	NA	FBtr0306682
FBgn0035815	FBgn0035815	NA	FBtr0301205
FBgn0035816	FBgn0035816	NA	FBtr0076809
FBgn0035817	FBgn0035817	NA	FBtr0076810
FBgn0035823	FBgn0035823	NA	FBtr0076747
FBgn0035824	FBgn0035824	NA	FBtr0076748
FBgn0035825	FBgn0035825	NA	FBtr0076770
FBgn0035827	FBgn0035827	NA	FBtr0076750
FBgn0035829	FBgn0035829	NA	FBtr0076765
FBgn0035830	FBgn0035830	CG8209	FBtr0076753
FBgn0035831	FBgn0035831	NA	FBtr0076763
FBgn0035833	FBgn0035833	CG7565	FBtr0076738
FBgn0035842	FBgn0035842	NA	FBtr0273367
FBgn0035845	FBgn0035845	NA	FBtr0300119
FBgn0035848	FBgn0035848	CG7979	FBtr0076690
FBgn0035849	FBgn0035849	ERR	FBtr0076724
FBgn0035850	FBgn0035850	Atg18	FBtr0076692
FBgn0035851	FBgn0035851	MED24	FBtr0076694
FBgn0035852	FBgn0035852	NA	FBtr0076723
FBgn0035857	FBgn0035857	NA	FBtr0076698
FBgn0035858	FBgn0035858	NA	FBtr0076719
FBgn0035859	FBgn0035859	NA	FBtr0076700
FBgn0035868	FBgn0035868	NA	FBtr0076714
FBgn0035870	FBgn0035870	NA	FBtr0300280
FBgn0035871	FBgn0035871	CG7188	FBtr0076712
FBgn0035872	FBgn0035872	CG7185	FBtr0076710
FBgn0035873	FBgn0035873	NA	FBtr0076708

FBgn0035875	FBgn0035875	NA	FBtr0076601
FBgn0035876	FBgn0035876	NA	FBtr0076602
FBgn0035877	FBgn0035877	NA	FBtr0076604
FBgn0035878	FBgn0035878	CG7182	FBtr0076677
FBgn0035879	FBgn0035879	NA	FBtr0113153
FBgn0035880	FBgn0035880	NA	FBtr0100664
FBgn0035888	FBgn0035888	NA	FBtr0307378
FBgn0035889	FBgn0035889	NA	FBtr0076664
FBgn0035895	FBgn0035895	NA	FBtr0308954
FBgn0035896	FBgn0035896	NA	FBtr0076659
FBgn0035900	FBgn0035900	NA	FBtr0076656
FBgn0035911	FBgn0035911	NA	FBtr0076651
FBgn0035914	FBgn0035914	NA	FBtr0076637
FBgn0035915	FBgn0035915	NA	FBtr0076638
FBgn0035916	FBgn0035916	NA	FBtr0076648
FBgn0035917	FBgn0035917	NA	FBtr0089561
FBgn0035918	FBgn0035918	CG5971	FBtr0076647
FBgn0035921	FBgn0035921	NA	FBtr0273348
FBgn0035922	FBgn0035922	NA	FBtr0076568
FBgn0035923	FBgn0035923	NA	FBtr0076570
FBgn0035924	FBgn0035924	NA	FBtr0076576
FBgn0035926	FBgn0035926	CG5804	FBtr0076591
FBgn0035929	FBgn0035929	NA	FBtr0076589
FBgn0035931	FBgn0035931	NA	FBtr0076587
FBgn0035932	FBgn0035932	NA	FBtr0303232
FBgn0035933	FBgn0035933	NA	FBtr0076582
FBgn0035934	FBgn0035934	NA	FBtr0300208
FBgn0035935	FBgn0035935	NA	FBtr0273288
FBgn0035944	FBgn0035944	CG5021	FBtr0301139
FBgn0035945	FBgn0035945	NA	FBtr0076516
FBgn0035949	FBgn0035949	NA	FBtr0076518
FBgn0035950	FBgn0035950	NA	FBtr0076554
FBgn0035955	FBgn0035955	NA	FBtr0076548
FBgn0035956	FBgn0035956	Doc2	FBtr0076547
FBgn0035959	FBgn0035959	NA	FBtr0076542
FBgn0035960	FBgn0035960	NA	FBtr0076524
FBgn0035964	FBgn0035964	Dhpr	FBtr0076531
FBgn0035965	FBgn0035965	NA	FBtr0076537
FBgn0035968	FBgn0035968	NA	FBtr0076503
FBgn0035975	FBgn0035975	NA	FBtr0076435
FBgn0035976	FBgn0035976	PGRP-LC	FBtr0089492
FBgn0035977	FBgn0035977	NA	FBtr0076439
FBgn0035978	FBgn0035978	UGP	FBtr0301699
FBgn0035981	FBgn0035981	NA	FBtr0089493
FBgn0035982	FBgn0035982	NA	FBtr0076452
FBgn0035983	FBgn0035983	NA	FBtr0309506
FBgn0035985	FBgn0035985	NA	FBtr0076455
FBgn0035986	FBgn0035986	CG4022	FBtr0309089
FBgn0035987	FBgn0035987	CG3689	FBtr0076456
FBgn0035997	FBgn0035997	NA	FBtr0076475
FBgn0035998	FBgn0035998	NA	FBtr0301032
FBgn0035999	FBgn0035999	CG3552	FBtr0114371
FBgn0036000	FBgn0036000	NA	FBtr0076473
FBgn0036003	FBgn0036003	NA	FBtr0310529
FBgn0036008	FBgn0036008	NA	FBtr0076426
FBgn0036010	FBgn0036010	NA	FBtr0076422
FBgn0036014	FBgn0036014	NA	FBtr0076379

FBgn0036015	FBgn0036015	NA	FBtr0076421
FBgn0036016	FBgn0036016	NA	FBtr0076380
FBgn0036017	FBgn0036017	NA	FBtr0304601
FBgn0036019	FBgn0036019	NA	FBtr0076420
FBgn0036020	FBgn0036020	CG8336	FBtr0076384
FBgn0036022	FBgn0036022	NA	FBtr0076387
FBgn0036023	FBgn0036023	NA	FBtr0076388
FBgn0036024	FBgn0036024	NA	FBtr0076389
FBgn0036028	FBgn0036028	NA	FBtr0076392
FBgn0036029	FBgn0036029	NA	FBtr0076394
FBgn0036030	FBgn0036030	CG6767	FBtr0076416
FBgn0036035	FBgn0036035	CG18178	FBtr0076412
FBgn0036036	FBgn0036036	NA	FBtr0076402
FBgn0036038	FBgn0036038	NA	FBtr0076403
FBgn0036039	FBgn0036039	NA	FBtr0089411
FBgn0036040	FBgn0036040	NA	FBtr0076410
FBgn0036043	FBgn0036043	NA	FBtr0076319
FBgn0036044	FBgn0036044	NA	FBtr0310628
FBgn0036046	FBgn0036046	NA	FBtr0076329
FBgn0036052	FBgn0036052	NA	FBtr0300688
FBgn0036053	FBgn0036053	CG6718	FBtr0076364
FBgn0036056	FBgn0036056	NA	FBtr0113156
FBgn0036057	FBgn0036057	NA	FBtr0113157
FBgn0036059	FBgn0036059	NA	FBtr0113158
FBgn0036062	FBgn0036062	NA	FBtr0076355
FBgn0036063	FBgn0036063	NA	FBtr0076354
FBgn0036064	FBgn0036064	NA	FBtr0076351
FBgn0036066	FBgn0036066	NA	FBtr0076316
FBgn0036072	FBgn0036072	NA	FBtr0076311
FBgn0036075	FBgn0036075	NA	FBtr0301227
FBgn0036085	FBgn0036085	NA	FBtr0076291
FBgn0036089	FBgn0036089	NA	FBtr0076280
FBgn0036090	FBgn0036090	NA	FBtr0076255
FBgn0036091	FBgn0036091	NA	FBtr0076256
FBgn0036101	FBgn0036101	NA	FBtr0089569
FBgn0036102	FBgn0036102	NA	FBtr0076266
FBgn0036104	FBgn0036104	CG6418	FBtr0076274
FBgn0036108	FBgn0036108	NA	FBtr0076191
FBgn0036109	FBgn0036109	NA	FBtr0076192
FBgn0036110	FBgn0036110	NA	FBtr0076193
FBgn0036111	FBgn0036111	NA	FBtr0076252
FBgn0036116	FBgn0036116	NA	FBtr0076199
FBgn0036117	FBgn0036117	CG6321	FBtr0076242
FBgn0036118	FBgn0036118	NA	FBtr0076202
FBgn0036125	FBgn0036125	NA	FBtr0076235
FBgn0036126	FBgn0036126	NA	FBtr0076234
FBgn0036128	FBgn0036128	NA	FBtr0113161
FBgn0036133	FBgn0036133	CG7638	FBtr0076210
FBgn0036134	FBgn0036134	Mnf	FBtr0300784
FBgn0036135	FBgn0036135	mRpL2	FBtr0076211
FBgn0036136	FBgn0036136	Ufd1-like	FBtr0076223
FBgn0036138	FBgn0036138	NA	FBtr0302875
FBgn0036139	FBgn0036139	NA	FBtr0076221
FBgn0036141	FBgn0036141	NA	FBtr0076218
FBgn0036142	FBgn0036142	NA	FBtr0076161
FBgn0036143	FBgn0036143	NA	FBtr0300348
FBgn0036144	FBgn0036144	GlcAT-P	FBtr0076189

FBgn0036145	FBgn0036145	NA	FBtr0076163
FBgn0036146	FBgn0036146	NA	FBtr0076164
FBgn0036147	FBgn0036147	CG6199	FBtr0076187
FBgn0036150	FBgn0036150	NA	FBtr0076185
FBgn0036153	FBgn0036153	NA	FBtr0076171
FBgn0036154	FBgn0036154	NA	FBtr0076182
FBgn0036156	FBgn0036156	NA	FBtr0076172
FBgn0036158	FBgn0036158	NA	FBtr0300274
FBgn0036160	FBgn0036160	NA	FBtr0076178
FBgn0036161	FBgn0036161	NA	FBtr0076176
FBgn0036168	FBgn0036168	NA	FBtr0113163
FBgn0036169	FBgn0036169	NA	FBtr0100588
FBgn0036170	FBgn0036170	NA	FBtr0076156
FBgn0036173	FBgn0036173	CG7394	FBtr0305648
FBgn0036178	FBgn0036178	NA	FBtr0076144
FBgn0036179	FBgn0036179	NA	FBtr0110884
FBgn0036180	FBgn0036180	CG6091	FBtr0273305
FBgn0036191	FBgn0036191	Sug	FBtr0300100
FBgn0036192	FBgn0036192	NA	FBtr0300726
FBgn0036193	FBgn0036193	NA	FBtr0290309
FBgn0036194	FBgn0036194	CG11652	FBtr0076130
FBgn0036195	FBgn0036195	NA	FBtr0301326
FBgn0036198	FBgn0036198	CG6038	FBtr0076123
FBgn0036202	FBgn0036202	CG6024	FBtr0113165
FBgn0036204	FBgn0036204	NA	FBtr0076089
FBgn0036205	FBgn0036205	NA	FBtr0076028
FBgn0036206	FBgn0036206	NA	FBtr0076085
FBgn0036210	FBgn0036210	NA	FBtr0076031
FBgn0036211	FBgn0036211	CG5946	FBtr0301135
FBgn0036212	FBgn0036212	NA	FBtr0302137
FBgn0036213	FBgn0036213	RpL10Ab	FBtr0302135
FBgn0036214	FBgn0036214	CG7264	FBtr0076035
FBgn0036218	FBgn0036218	NA	FBtr0300721
FBgn0036220	FBgn0036220	NA	FBtr0076070
FBgn0036226	FBgn0036226	NA	FBtr0076041
FBgn0036228	FBgn0036228	NA	FBtr0076066
FBgn0036229	FBgn0036229	NA	FBtr0076043
FBgn0036230	FBgn0036230	NA	FBtr0306793
FBgn0036232	FBgn0036232	NA	FBtr0310535
FBgn0036233	FBgn0036233	NA	FBtr0076045
FBgn0036234	FBgn0036234	NA	FBtr0076046
FBgn0036235	FBgn0036235	NA	FBtr0290131
FBgn0036236	FBgn0036236	NA	FBtr0076048
FBgn0036237	FBgn0036237	viaf	FBtr0076062
FBgn0036239	FBgn0036239	NA	FBtr0076059
FBgn0036242	FBgn0036242	NA	FBtr0076051
FBgn0036246	FBgn0036246	NA	FBtr0075990
FBgn0036254	FBgn0036254	CG5645	FBtr0076024
FBgn0036255	FBgn0036255	NA	FBtr0300011
FBgn0036257	FBgn0036257	RhoGAP68F	FBtr0310580
FBgn0036258	FBgn0036258	NA	FBtr0076022
FBgn0036259	FBgn0036259	NA	FBtr0273438
FBgn0036262	FBgn0036262	CG6910	FBtr0290130
FBgn0036263	FBgn0036263	NA	FBtr0076019
FBgn0036264	FBgn0036264	NA	FBtr0076005
FBgn0036271	FBgn0036271	Pbgs	FBtr0076011
FBgn0036274	FBgn0036274	NA	FBtr0301071

FBgn0036277	FBgn0036277	NA	FBtr0075954
FBgn0036278	FBgn0036278	GRHRII	FBtr0075989
FBgn0036286	FBgn0036286	NA	FBtr0308896
FBgn0036287	FBgn0036287	NA	FBtr0113167
FBgn0036288	FBgn0036288	CG10660	FBtr0075976
FBgn0036290	FBgn0036290	CG10638	FBtr0300686
FBgn0036291	FBgn0036291	NA	FBtr0075957
FBgn0036300	FBgn0036300	NA	FBtr0075961
FBgn0036301	FBgn0036301	CG4069	FBtr0075965
FBgn0036302	FBgn0036302	NA	FBtr0300888
FBgn0036310	FBgn0036310	NA	FBtr0075946
FBgn0036311	FBgn0036311	NA	FBtr0075923
FBgn0036314	FBgn0036314	CG10754	FBtr0075944
FBgn0036317	FBgn0036317	NA	FBtr0075939
FBgn0036318	FBgn0036318	NA	FBtr0273240
FBgn0036320	FBgn0036320	NA	FBtr0075936
FBgn0036323	FBgn0036323	NA	FBtr0075870
FBgn0036324	FBgn0036324	NA	FBtr0113169
FBgn0036329	FBgn0036329	NA	FBtr0075872
FBgn0036330	FBgn0036330	CG11263	FBtr0075873
FBgn0036331	FBgn0036331	NA	FBtr0306091
FBgn0036332	FBgn0036332	NA	FBtr0075899
FBgn0036337	FBgn0036337	CG11255	FBtr0075895
FBgn0036338	FBgn0036338	NA	FBtr0075894
FBgn0036340	FBgn0036340	NA	FBtr0075883
FBgn0036345	FBgn0036345	NA	FBtr0075892
FBgn0036346	FBgn0036346	NA	FBtr0075891
FBgn0036351	FBgn0036351	CG14107	FBtr0075866
FBgn0036352	FBgn0036352	CG14110	FBtr0075844
FBgn0036353	FBgn0036353	CG10171	FBtr0075865
FBgn0036354	FBgn0036354	NA	FBtr0075863
FBgn0036356	FBgn0036356	NA	FBtr0075861
FBgn0036359	FBgn0036359	NA	FBtr0075857
FBgn0036360	FBgn0036360	NA	FBtr0075855
FBgn0036361	FBgn0036361	NA	FBtr0075854
FBgn0036362	FBgn0036362	NA	FBtr0089323
FBgn0036363	FBgn0036363	NA	FBtr0075853
FBgn0036364	FBgn0036364	CG14109	FBtr0075850
FBgn0036365	FBgn0036365	CG10732	FBtr0075851
FBgn0036366	FBgn0036366	CG10133	FBtr0075825
FBgn0036367	FBgn0036367	NA	FBtr0075824
FBgn0036368	FBgn0036368	NA	FBtr0302488
FBgn0036374	FBgn0036374	NA	FBtr0304904
FBgn0036380	FBgn0036380	NA	FBtr0306683
FBgn0036393	FBgn0036393	NA	FBtr0075764
FBgn0036394	FBgn0036394	NA	FBtr0075765
FBgn0036402	FBgn0036402	NA	FBtr0075769
FBgn0036403	FBgn0036403	NA	FBtr0075770
FBgn0036405	FBgn0036405	CG6833	FBtr0075774
FBgn0036406	FBgn0036406	NA	FBtr0075780
FBgn0036410	FBgn0036410	NA	FBtr0075779
FBgn0036411	FBgn0036411	NA	FBtr0075748
FBgn0036414	FBgn0036414	NA	FBtr0321258
FBgn0036415	FBgn0036415	CG7768	FBtr0075737
FBgn0036416	FBgn0036416	NA	FBtr0075740
FBgn0036417	FBgn0036417	NA	FBtr0321257
FBgn0036419	FBgn0036419	NA	FBtr0075744

FBgn0036421	FBgn0036421	NA	FBtr0113170
FBgn0036422	FBgn0036422	NA	FBtr0075729
FBgn0036423	FBgn0036423	NA	FBtr0273239
FBgn0036426	FBgn0036426	NA	FBtr0290282
FBgn0036433	FBgn0036433	CG9628	FBtr0075677
FBgn0036442	FBgn0036442	NA	FBtr0075689
FBgn0036443	FBgn0036443	NA	FBtr0075709
FBgn0036447	FBgn0036447	NA	FBtr0075701
FBgn0036448	FBgn0036448	NA	FBtr0075700
FBgn0036449	FBgn0036449	CG5295	FBtr0075699
FBgn0036450	FBgn0036450	CG13472	FBtr0075691
FBgn0036454	FBgn0036454	NA	FBtr0075695
FBgn0036459	FBgn0036459	NA	FBtr0309063
FBgn0036460	FBgn0036460	CG5114	FBtr0309064
FBgn0036461	FBgn0036461	NA	FBtr0113171
FBgn0036462	FBgn0036462	mRpL39	FBtr0075672
FBgn0036466	FBgn0036466	NA	FBtr0075666
FBgn0036467	FBgn0036467	NA	FBtr0075665
FBgn0036468	FBgn0036468	NA	FBtr0075664
FBgn0036469	FBgn0036469	NA	FBtr0075663
FBgn0036470	FBgn0036470	CG13463	FBtr0075631
FBgn0036471	FBgn0036471	NA	FBtr0114510
FBgn0036474	FBgn0036474	NA	FBtr0075659
FBgn0036476	FBgn0036476	NA	FBtr0075657
FBgn0036480	FBgn0036480	CG17081	FBtr0303375
FBgn0036481	FBgn0036481	CG16959	FBtr0075655
FBgn0036482	FBgn0036482	NA	FBtr0075654
FBgn0036491	FBgn0036491	NA	FBtr0113172
FBgn0036492	FBgn0036492	NA	FBtr0075618
FBgn0036493	FBgn0036493	NA	FBtr0100360
FBgn0036494	FBgn0036494	Toll-6	FBtr0306308
FBgn0036498	FBgn0036498	NA	FBtr0075544
FBgn0036499	FBgn0036499	NA	FBtr0075546
FBgn0036501	FBgn0036501	CG7272	FBtr0075548
FBgn0036502	FBgn0036502	CG7841	FBtr0075604
FBgn0036503	FBgn0036503	NA	FBtr0075603
FBgn0036504	FBgn0036504	NA	FBtr0075556
FBgn0036505	FBgn0036505	CG7945	FBtr0075600
FBgn0036509	FBgn0036509	CG7739	FBtr0075596
FBgn0036510	FBgn0036510	NA	FBtr0306156
FBgn0036511	FBgn0036511	NA	FBtr0075562
FBgn0036512	FBgn0036512	CG16979	FBtr0075563
FBgn0036514	FBgn0036514	CG12301	FBtr0075564
FBgn0036515	FBgn0036515	NA	FBtr0075565
FBgn0036516	FBgn0036516	NA	FBtr0075592
FBgn0036518	FBgn0036518	RhoGAP71E	FBtr0290065
FBgn0036519	FBgn0036519	NA	FBtr0075591
FBgn0036520	FBgn0036520	NA	FBtr0075590
FBgn0036527	FBgn0036527	NA	FBtr0075577
FBgn0036529	FBgn0036529	NA	FBtr0075578
FBgn0036534	FBgn0036534	CG6169	FBtr0304975
FBgn0036537	FBgn0036537	NA	FBtr0306855
FBgn0036538	FBgn0036538	NA	FBtr0075536
FBgn0036542	FBgn0036542	NA	FBtr0301510
FBgn0036544	FBgn0036544	NA	FBtr0089527
FBgn0036545	FBgn0036545	GXIVsPLA2	FBtr0273189
FBgn0036546	FBgn0036546	CG17033	FBtr0075510

FBgn0036547	FBgn0036547	CG17032	FBtr0075511
FBgn0036550	FBgn0036550	NA	FBtr0075513
FBgn0036551	FBgn0036551	CG17029	FBtr0075514
FBgn0036552	FBgn0036552	NA	FBtr0075515
FBgn0036553	FBgn0036553	CG17027	FBtr0075516
FBgn0036560	FBgn0036560	NA	FBtr0075437
FBgn0036563	FBgn0036563	NA	FBtr0301031
FBgn0036564	FBgn0036564	NA	FBtr0075498
FBgn0036565	FBgn0036565	NA	FBtr0075497
FBgn0036566	FBgn0036566	NA	FBtr0302503
FBgn0036567	FBgn0036567	NA	FBtr0075496
FBgn0036568	FBgn0036568	CG5389	FBtr0075446
FBgn0036569	FBgn0036569	NA	FBtr0302328
FBgn0036570	FBgn0036570	NA	FBtr0075495
FBgn0036571	FBgn0036571	NA	FBtr0301912
FBgn0036574	FBgn0036574	NA	FBtr0089713
FBgn0036575	FBgn0036575	NA	FBtr0075490
FBgn0036576	FBgn0036576	NA	FBtr0307521
FBgn0036577	FBgn0036577	NA	FBtr0075485
FBgn0036579	FBgn0036579	CG5027	FBtr0075457
FBgn0036580	FBgn0036580	PDCD-5	FBtr0075483
FBgn0036581	FBgn0036581	NA	FBtr0301481
FBgn0036584	FBgn0036584	NA	FBtr0075481
FBgn0036585	FBgn0036585	NA	FBtr0303379
FBgn0036586	FBgn0036586	NA	FBtr0303380
FBgn0036587	FBgn0036587	NA	FBtr0290260
FBgn0036588	FBgn0036588	NA	FBtr0075464
FBgn0036589	FBgn0036589	NA	FBtr0075465
FBgn0036594	FBgn0036594	NA	FBtr0289964
FBgn0036595	FBgn0036595	NA	FBtr0289963
FBgn0036596	FBgn0036596	NA	FBtr0306796
FBgn0036598	FBgn0036598	NA	FBtr0075469
FBgn0036599	FBgn0036599	NA	FBtr0075470
FBgn0036600	FBgn0036600	CG13043	FBtr0075434
FBgn0036601	FBgn0036601	NA	FBtr0075366
FBgn0036602	FBgn0036602	NA	FBtr0075433
FBgn0036603	FBgn0036603	NA	FBtr0303000
FBgn0036605	FBgn0036605	NA	FBtr0075432
FBgn0036606	FBgn0036606	NA	FBtr0075370
FBgn0036607	FBgn0036607	NA	FBtr0075371
FBgn0036608	FBgn0036608	NA	FBtr0310461
FBgn0036609	FBgn0036609	NA	FBtr0075430
FBgn0036610	FBgn0036610	NA	FBtr0075372
FBgn0036612	FBgn0036612	NA	FBtr0110866
FBgn0036615	FBgn0036615	NA	FBtr0075381
FBgn0036616	FBgn0036616	CG4893	FBtr0075425
FBgn0036620	FBgn0036620	NA	FBtr0075384
FBgn0036623	FBgn0036623	CG4729	FBtr0308819
FBgn0036629	FBgn0036629	NA	FBtr0075411
FBgn0036638	FBgn0036638	NA	FBtr0075400
FBgn0036639	FBgn0036639	NA	FBtr0075392
FBgn0036640	FBgn0036640	NA	FBtr0089478
FBgn0036641	FBgn0036641	Smn	FBtr0075395
FBgn0036643	FBgn0036643	NA	FBtr0075365
FBgn0036646	FBgn0036646	NA	FBtr0301925
FBgn0036652	FBgn0036652	NA	FBtr0075316
FBgn0036654	FBgn0036654	NA	FBtr0075317

FBgn0036656	FBgn0036656	NA	FBtr0299542
FBgn0036660	FBgn0036660	NA	FBtr0075346
FBgn0036661	FBgn0036661	CG9705	FBtr0075325
FBgn0036662	FBgn0036662	CG9706	FBtr0075326
FBgn0036663	FBgn0036663	NA	FBtr0075344
FBgn0036665	FBgn0036665	NA	FBtr0305913
FBgn0036666	FBgn0036666	TSG101	FBtr0075329
FBgn0036667	FBgn0036667	NA	FBtr0075339
FBgn0036668	FBgn0036668	NA	FBtr0075330
FBgn0036670	FBgn0036670	NA	FBtr0075336
FBgn0036671	FBgn0036671	NA	FBtr0075337
FBgn0036676	FBgn0036676	NA	FBtr0075261
FBgn0036677	FBgn0036677	NA	FBtr0075307
FBgn0036678	FBgn0036678	CG11905	FBtr0075306
FBgn0036679	FBgn0036679	NA	FBtr0273313
FBgn0036680	FBgn0036680	CG9665	FBtr0113176
FBgn0036681	FBgn0036681	NA	FBtr0113177
FBgn0036684	FBgn0036684	CG3764	FBtr0321285
FBgn0036690	FBgn0036690	NA	FBtr0290221
FBgn0036691	FBgn0036691	NA	FBtr0075278
FBgn0036695	FBgn0036695	NA	FBtr0075279
FBgn0036696	FBgn0036696	NA	FBtr0075286
FBgn0036702	FBgn0036702	NA	FBtr0075251
FBgn0036703	FBgn0036703	NA	FBtr0075212
FBgn0036704	FBgn0036704	NA	FBtr0075250
FBgn0036705	FBgn0036705	NA	FBtr0075213
FBgn0036706	FBgn0036706	NA	FBtr0075249
FBgn0036708	FBgn0036708	NA	FBtr0075248
FBgn0036715	FBgn0036715	Cad74A	FBtr0309231
FBgn0036716	FBgn0036716	NA	FBtr0075238
FBgn0036717	FBgn0036717	NA	FBtr0306800
FBgn0036723	FBgn0036723	NA	FBtr0075226
FBgn0036726	FBgn0036726	NA	FBtr0075220
FBgn0036727	FBgn0036727	NA	FBtr0075221
FBgn0036728	FBgn0036728	NA	FBtr0302509
FBgn0036729	FBgn0036729	NA	FBtr0075225
FBgn0036730	FBgn0036730	NA	FBtr0075223
FBgn0036731	FBgn0036731	NA	FBtr0302208
FBgn0036732	FBgn0036732	Oatp74D	FBtr0302041
FBgn0036733	FBgn0036733	CG6322	FBtr0075209
FBgn0036734	FBgn0036734	NA	FBtr0075175
FBgn0036735	FBgn0036735	NA	FBtr0075208
FBgn0036740	FBgn0036740	CG6259	FBtr0075201
FBgn0036742	FBgn0036742	NA	FBtr0300284
FBgn0036746	FBgn0036746	NA	FBtr0075196
FBgn0036747	FBgn0036747	NA	FBtr0075195
FBgn0036749	FBgn0036749	NA	FBtr0075187
FBgn0036750	FBgn0036750	NA	FBtr0075194
FBgn0036754	FBgn0036754	NA	FBtr0075170
FBgn0036757	FBgn0036757	NA	FBtr0273392
FBgn0036764	FBgn0036764	NA	FBtr0075159
FBgn0036765	FBgn0036765	NA	FBtr0075142
FBgn0036766	FBgn0036766	NA	FBtr0075157
FBgn0036767	FBgn0036767	NA	FBtr0290228
FBgn0036768	FBgn0036768	NA	FBtr0075143
FBgn0036769	FBgn0036769	Tsp74F	FBtr0075155
FBgn0036770	FBgn0036770	NA	FBtr0075153

FBgn0036771	FBgn0036771	NA	FBtr0075144
FBgn0036772	FBgn0036772	NA	FBtr0075152
FBgn0036778	FBgn0036778	NA	FBtr0075135
FBgn0036782	FBgn0036782	NA	FBtr0310156
FBgn0036784	FBgn0036784	NA	FBtr0075132
FBgn0036785	FBgn0036785	NA	FBtr0299605
FBgn0036787	FBgn0036787	CG4306	FBtr0075117
FBgn0036791	FBgn0036791	NA	FBtr0075101
FBgn0036794	FBgn0036794	NA	FBtr0300049
FBgn0036795	FBgn0036795	NA	FBtr0304702
FBgn0036796	FBgn0036796	NA	FBtr0300052
FBgn0036804	FBgn0036804	NA	FBtr0113179
FBgn0036807	FBgn0036807	NA	FBtr0114373
FBgn0036808	FBgn0036808	NA	FBtr0075090
FBgn0036809	FBgn0036809	NA	FBtr0075089
FBgn0036812	FBgn0036812	NA	FBtr0075088
FBgn0036813	FBgn0036813	Aut1	FBtr0075055
FBgn0036816	FBgn0036816	Indy	FBtr0075082
FBgn0036817	FBgn0036817	NA	FBtr0308811
FBgn0036818	FBgn0036818	NA	FBtr0075061
FBgn0036819	FBgn0036819	CG6856	FBtr0075062
FBgn0036820	FBgn0036820	NA	FBtr0308813
FBgn0036821	FBgn0036821	CG3961	FBtr0075077
FBgn0036822	FBgn0036822	NA	FBtr0075065
FBgn0036827	FBgn0036827	NA	FBtr0075067
FBgn0036828	FBgn0036828	CG6841	FBtr0075068
FBgn0036829	FBgn0036829	NA	FBtr0309798
FBgn0036830	FBgn0036830	NA	FBtr0299821
FBgn0036831	FBgn0036831	NA	FBtr0075069
FBgn0036832	FBgn0036832	NA	FBtr0075071
FBgn0036833	FBgn0036833	CG3819	FBtr0075048
FBgn0036835	FBgn0036835	NA	FBtr0075024
FBgn0036838	FBgn0036838	CG3808	FBtr0075041
FBgn0036839	FBgn0036839	CG18136	FBtr0075040
FBgn0036847	FBgn0036847	CG11577	FBtr0075034
FBgn0036848	FBgn0036848	CG10424	FBtr0075033
FBgn0036849	FBgn0036849	NA	FBtr0075027
FBgn0036850	FBgn0036850	NA	FBtr0075032
FBgn0036851	FBgn0036851	NA	FBtr0114620
FBgn0036856	FBgn0036856	CG9666	FBtr0074991
FBgn0036857	FBgn0036857	CG9629	FBtr0075017
FBgn0036858	FBgn0036858	NA	FBtr0309116
FBgn0036859	FBgn0036859	NA	FBtr0074992
FBgn0036860	FBgn0036860	NA	FBtr0300720
FBgn0036868	FBgn0036868	NA	FBtr0307012
FBgn0036870	FBgn0036870	NA	FBtr0074987
FBgn0036871	FBgn0036871	NA	FBtr0074986
FBgn0036874	FBgn0036874	NA	FBtr0074982
FBgn0036875	FBgn0036875	CG9449	FBtr0273417
FBgn0036876	FBgn0036876	CG9451	FBtr0074979
FBgn0036877	FBgn0036877	NA	FBtr0074978
FBgn0036880	FBgn0036880	NA	FBtr0113180
FBgn0036881	FBgn0036881	NA	FBtr0113181
FBgn0036882	FBgn0036882	CG9279	FBtr0074976
FBgn0036888	FBgn0036888	CG9330	FBtr0074940
FBgn0036889	FBgn0036889	NA	FBtr0074971
FBgn0036890	FBgn0036890	NA	FBtr0074943

FBgn0036891	FBgn0036891	CG9372	FBtr0074945
FBgn0036892	FBgn0036892	NA	FBtr0273410
FBgn0036893	FBgn0036893	CG9376	FBtr0074946
FBgn0036895	FBgn0036895	NA	FBtr0074948
FBgn0036896	FBgn0036896	NA	FBtr0074965
FBgn0036897	FBgn0036897	NA	FBtr0074962
FBgn0036899	FBgn0036899	NA	FBtr0273407
FBgn0036906	FBgn0036906	CG14102	FBtr0074928
FBgn0036908	FBgn0036908	NA	FBtr0074927
FBgn0036909	FBgn0036909	NA	FBtr0074879
FBgn0036910	FBgn0036910	Cyp305a1	FBtr0074925
FBgn0036911	FBgn0036911	NA	FBtr0089596
FBgn0036915	FBgn0036915	NA	FBtr0074891
FBgn0036916	FBgn0036916	NA	FBtr0074918
FBgn0036922	FBgn0036922	NA	FBtr0074913
FBgn0036923	FBgn0036923	NA	FBtr0303781
FBgn0036926	FBgn0036926	NA	FBtr0074900
FBgn0036927	FBgn0036927	NA	FBtr0302049
FBgn0036928	FBgn0036928	Tom20	FBtr0074903
FBgn0036931	FBgn0036931	NA	FBtr0074907
FBgn0036932	FBgn0036932	NA	FBtr0074872
FBgn0036936	FBgn0036936	NA	FBtr0074818
FBgn0036937	FBgn0036937	NA	FBtr0074869
FBgn0036938	FBgn0036938	NA	FBtr0074868
FBgn0036940	FBgn0036940	NA	FBtr0074866
FBgn0036941	FBgn0036941	NA	FBtr0074865
FBgn0036945	FBgn0036945	CG6981	FBtr0074820
FBgn0036947	FBgn0036947	NA	FBtr0074860
FBgn0036948	FBgn0036948	CG7298	FBtr0074859
FBgn0036949	FBgn0036949	NA	FBtr0074858
FBgn0036950	FBgn0036950	NA	FBtr0302721
FBgn0036951	FBgn0036951	CG7017	FBtr0074856
FBgn0036952	FBgn0036952	CG6933	FBtr0310468
FBgn0036953	FBgn0036953	NA	FBtr0074823
FBgn0036956	FBgn0036956	NA	FBtr0074827
FBgn0036958	FBgn0036958	NA	FBtr0074850
FBgn0036959	FBgn0036959	NA	FBtr0074828
FBgn0036964	FBgn0036964	NA	FBtr0074847
FBgn0036967	FBgn0036967	CG6597	FBtr0074838
FBgn0036968	FBgn0036968	NA	FBtr0074841
FBgn0036970	FBgn0036970	NA	FBtr0078172
FBgn0036975	FBgn0036975	CG5618	FBtr0078179
FBgn0036977	FBgn0036977	NA	FBtr0308655
FBgn0036978	FBgn0036978	NA	FBtr0078240
FBgn0036979	FBgn0036979	NA	FBtr0078184
FBgn0036985	FBgn0036985	NA	FBtr0078191
FBgn0036986	FBgn0036986	CG5282	FBtr0078233
FBgn0036987	FBgn0036987	NA	FBtr0078232
FBgn0036988	FBgn0036988	CG5262	FBtr0310615
FBgn0036992	FBgn0036992	CG11796	FBtr0078226
FBgn0036993	FBgn0036993	NA	FBtr0300411
FBgn0036994	FBgn0036994	CG5199	FBtr0078224
FBgn0036995	FBgn0036995	NA	FBtr0078223
FBgn0036996	FBgn0036996	NA	FBtr0078196
FBgn0036997	FBgn0036997	NA	FBtr0078197
FBgn0036998	FBgn0036998	NA	FBtr0078198
FBgn0036999	FBgn0036999	NA	FBtr0078200

FBgn0037005	FBgn0037005	NA	FBtr0078208
FBgn0037010	FBgn0037010	NA	FBtr0078214
FBgn0037011	FBgn0037011	CG4858	FBtr0078210
FBgn0037015	FBgn0037015	CG13253	FBtr0113182
FBgn0037017	FBgn0037017	NA	FBtr0078280
FBgn0037018	FBgn0037018	CG4042	FBtr0321286
FBgn0037019	FBgn0037019	CG3947	FBtr0078278
FBgn0037020	FBgn0037020	NA	FBtr0078251
FBgn0037023	FBgn0037023	NA	FBtr0078273
FBgn0037024	FBgn0037024	CG4365	FBtr0078254
FBgn0037025	FBgn0037025	NA	FBtr0321284
FBgn0037026	FBgn0037026	NA	FBtr0078272
FBgn0037027	FBgn0037027	NA	FBtr0078256
FBgn0037028	FBgn0037028	CG3618	FBtr0078271
FBgn0037030	FBgn0037030	NA	FBtr0078262
FBgn0037031	FBgn0037031	CG11456	FBtr0300873
FBgn0037036	FBgn0037036	NA	FBtr0078335
FBgn0037037	FBgn0037037	NA	FBtr0078284
FBgn0037038	FBgn0037038	NA	FBtr0078334
FBgn0037039	FBgn0037039	NA	FBtr0300055
FBgn0037040	FBgn0037040	CG12983	FBtr0114617
FBgn0037042	FBgn0037042	NA	FBtr0078286
FBgn0037044	FBgn0037044	CG10585	FBtr0078287
FBgn0037045	FBgn0037045	NA	FBtr0078327
FBgn0037046	FBgn0037046	NA	FBtr0078326
FBgn0037050	FBgn0037050	CG10566	FBtr0078292
FBgn0037051	FBgn0037051	CG10565	FBtr0305502
FBgn0037059	FBgn0037059	NA	FBtr0078310
FBgn0037060	FBgn0037060	NA	FBtr0307387
FBgn0037061	FBgn0037061	NA	FBtr0078309
FBgn0037063	FBgn0037063	CG9391	FBtr0305708
FBgn0037064	FBgn0037064	NA	FBtr0078415
FBgn0037065	FBgn0037065	NA	FBtr0078410
FBgn0037067	FBgn0037067	NA	FBtr0078409
FBgn0037068	FBgn0037068	CG7663	FBtr0078337
FBgn0037069	FBgn0037069	NA	FBtr0078338
FBgn0037070	FBgn0037070	NA	FBtr0078407
FBgn0037071	FBgn0037071	CG7632	FBtr0078339
FBgn0037073	FBgn0037073	NA	FBtr0078342
FBgn0037076	FBgn0037076	NA	FBtr0305306
FBgn0037078	FBgn0037078	NA	FBtr0306795
FBgn0037085	FBgn0037085	NA	FBtr0078394
FBgn0037086	FBgn0037086	NA	FBtr0078393
FBgn0037087	FBgn0037087	CG7519	FBtr0078353
FBgn0037092	FBgn0037092	M6	FBtr0078355
FBgn0037094	FBgn0037094	CG7611	FBtr0301197
FBgn0037097	FBgn0037097	NA	FBtr0078382
FBgn0037098	FBgn0037098	NA	FBtr0305328
FBgn0037099	FBgn0037099	CG7173	FBtr0078380
FBgn0037101	FBgn0037101	NA	FBtr0078371
FBgn0037102	FBgn0037102	NA	FBtr0078377
FBgn0037106	FBgn0037106	NA	FBtr0078421
FBgn0037107	FBgn0037107	NA	FBtr0301161
FBgn0037108	FBgn0037108	CG11306	FBtr0078422
FBgn0037109	FBgn0037109	NA	FBtr0078464
FBgn0037110	FBgn0037110	NA	FBtr0078423
FBgn0037120	FBgn0037120	CG11247	FBtr0078458

FBgn0037122	FBgn0037122	NA	FBtr0078457
FBgn0037123	FBgn0037123	CG14569	FBtr0078456
FBgn0037124	FBgn0037124	NA	FBtr0078455
FBgn0037125	FBgn0037125	NA	FBtr0078433
FBgn0037126	FBgn0037126	NA	FBtr0078454
FBgn0037127	FBgn0037127	NA	FBtr0078453
FBgn0037128	FBgn0037128	NA	FBtr0078434
FBgn0037129	FBgn0037129	NA	FBtr0078452
FBgn0037130	FBgn0037130	NA	FBtr0310451
FBgn0037131	FBgn0037131	NA	FBtr0078435
FBgn0037133	FBgn0037133	NA	FBtr0078436
FBgn0037134	FBgn0037134	CG7407	FBtr0078440
FBgn0037135	FBgn0037135	CG7414	FBtr0303245
FBgn0037137	FBgn0037137	NA	FBtr0089580
FBgn0037138	FBgn0037138	NA	FBtr0078445
FBgn0037139	FBgn0037139	NA	FBtr0078444
FBgn0037146	FBgn0037146	CG7470	FBtr0309869
FBgn0037147	FBgn0037147	NA	FBtr0114546
FBgn0037149	FBgn0037149	NA	FBtr0078480
FBgn0037150	FBgn0037150	NA	FBtr0078485
FBgn0037151	FBgn0037151	NA	FBtr0078484
FBgn0037153	FBgn0037153	olf413	FBtr0301492
FBgn0037156	FBgn0037156	CG11523	FBtr0078511
FBgn0037166	FBgn0037166	CG11426	FBtr0078506
FBgn0037167	FBgn0037167	NA	FBtr0078507
FBgn0037171	FBgn0037171	NA	FBtr0113185
FBgn0037175	FBgn0037175	NA	FBtr0078543
FBgn0037176	FBgn0037176	NA	FBtr0289975
FBgn0037178	FBgn0037178	NA	FBtr0078541
FBgn0037182	FBgn0037182	CG6838	FBtr0078539
FBgn0037183	FBgn0037183	NA	FBtr0078538
FBgn0037184	FBgn0037184	NA	FBtr0078537
FBgn0037185	FBgn0037185	CG11367	FBtr0078528
FBgn0037186	FBgn0037186	CG11241	FBtr0113188
FBgn0037191	FBgn0037191	NA	FBtr0078534
FBgn0037195	FBgn0037195	NA	FBtr0078546
FBgn0037197	FBgn0037197	NA	FBtr0078548
FBgn0037199	FBgn0037199	CG11137	FBtr0078577
FBgn0037206	FBgn0037206	NA	FBtr0078560
FBgn0037207	FBgn0037207	Mes2	FBtr0078561
FBgn0037212	FBgn0037212	NA	FBtr0300118
FBgn0037215	FBgn0037215	CG12582	FBtr0308680
FBgn0037220	FBgn0037220	CG14641	FBtr0078985
FBgn0037222	FBgn0037222	NA	FBtr0089688
FBgn0037223	FBgn0037223	NA	FBtr0113190
FBgn0037228	FBgn0037228	CG1092	FBtr0078978
FBgn0037229	FBgn0037229	NA	FBtr0113191
FBgn0037230	FBgn0037230	NA	FBtr0078959
FBgn0037231	FBgn0037231	CG9779	FBtr0078958
FBgn0037232	FBgn0037232	NA	FBtr0273009
FBgn0037235	FBgn0037235	CG1103	FBtr0078909
FBgn0037236	FBgn0037236	NA	FBtr0078953
FBgn0037238	FBgn0037238	NA	FBtr0078912
FBgn0037241	FBgn0037241	NA	FBtr0078919
FBgn0037242	FBgn0037242	NA	FBtr0078950
FBgn0037244	FBgn0037244	CG14647	FBtr0290222
FBgn0037245	FBgn0037245	NA	FBtr0078921

FBgn0037248	FBgn0037248	CG9809	FBtr0078946
FBgn0037249	FBgn0037249	NA	FBtr0078944
FBgn0037250	FBgn0037250	CG1074	FBtr0078925
FBgn0037251	FBgn0037251	NA	FBtr0078943
FBgn0037252	FBgn0037252	NA	FBtr0078926
FBgn0037254	FBgn0037254	NA	FBtr0299947
FBgn0037260	FBgn0037260	NA	FBtr0303866
FBgn0037261	FBgn0037261	NA	FBtr0301297
FBgn0037262	FBgn0037262	MED31	FBtr0078856
FBgn0037263	FBgn0037263	NA	FBtr0301296
FBgn0037265	FBgn0037265	CG12001	FBtr0078861
FBgn0037270	FBgn0037270	CG9769	FBtr0078892
FBgn0037275	FBgn0037275	CG14655	FBtr0078881
FBgn0037276	FBgn0037276	NA	FBtr0078880
FBgn0037279	FBgn0037279	CG1129	FBtr0303910
FBgn0037280	FBgn0037280	NA	FBtr0078872
FBgn0037283	FBgn0037283	NA	FBtr0078874
FBgn0037284	FBgn0037284	NA	FBtr0078873
FBgn0037288	FBgn0037288	NA	FBtr0078848
FBgn0037291	FBgn0037291	NA	FBtr0078846
FBgn0037292	FBgn0037292	NA	FBtr0301312
FBgn0037295	FBgn0037295	NA	FBtr0078772
FBgn0037296	FBgn0037296	NA	FBtr0078831
FBgn0037297	FBgn0037297	CG1116	FBtr0078774
FBgn0037298	FBgn0037298	CG2604	FBtr0078829
FBgn0037299	FBgn0037299	CG1115	FBtr0305002
FBgn0037304	FBgn0037304	NA	FBtr0078779
FBgn0037305	FBgn0037305	NA	FBtr0300669
FBgn0037307	FBgn0037307	NA	FBtr0078820
FBgn0037309	FBgn0037309	NA	FBtr0078783
FBgn0037310	FBgn0037310	NA	FBtr0078784
FBgn0037312	FBgn0037312	NA	FBtr0078807
FBgn0037313	FBgn0037313	CG1161	FBtr0100434
FBgn0037321	FBgn0037321	NA	FBtr0309312
FBgn0037323	FBgn0037323	CG2663	FBtr0078796
FBgn0037324	FBgn0037324	NA	FBtr0078794
FBgn0037326	FBgn0037326	NA	FBtr0078679
FBgn0037329	FBgn0037329	CG12162	FBtr0078681
FBgn0037330	FBgn0037330	mRpL44	FBtr0078768
FBgn0037338	FBgn0037338	Snm1	FBtr0078688
FBgn0037339	FBgn0037339	NA	FBtr0078756
FBgn0037340	FBgn0037340	NA	FBtr0078754
FBgn0037341	FBgn0037341	CG12746	FBtr0290215
FBgn0037342	FBgn0037342	CG2931	FBtr0078753
FBgn0037344	FBgn0037344	NA	FBtr0078752
FBgn0037345	FBgn0037345	NA	FBtr0078699
FBgn0037347	FBgn0037347	NA	FBtr0273377
FBgn0037350	FBgn0037350	NA	FBtr0078740
FBgn0037354	FBgn0037354	NA	FBtr0078708
FBgn0037356	FBgn0037356	NA	FBtr0078709
FBgn0037358	FBgn0037358	NA	FBtr0078732
FBgn0037359	FBgn0037359	MED27	FBtr0078712
FBgn0037363	FBgn0037363	NA	FBtr0078716
FBgn0037364	FBgn0037364	Rab23	FBtr0078727
FBgn0037365	FBgn0037365	NA	FBtr0078726
FBgn0037368	FBgn0037368	CG1239	FBtr0078720
FBgn0037369	FBgn0037369	CG2100	FBtr0301574

FBgn0037372	FBgn0037372	CG2091	FBtr0078678
FBgn0037374	FBgn0037374	jagn	FBtr0078624
FBgn0037375	FBgn0037375	CG1193	FBtr0300740
FBgn0037376	FBgn0037376	CG2051	FBtr0078670
FBgn0037377	FBgn0037377	CG1218	FBtr0078630
FBgn0037378	FBgn0037378	CG2046	FBtr0078668
FBgn0037380	FBgn0037380	NA	FBtr0308953
FBgn0037382	FBgn0037382	Hpr1	FBtr0078667
FBgn0037383	FBgn0037383	NA	FBtr0078666
FBgn0037386	FBgn0037386	NA	FBtr0113196
FBgn0037387	FBgn0037387	NA	FBtr0078644
FBgn0037388	FBgn0037388	NA	FBtr0078645
FBgn0037389	FBgn0037389	NA	FBtr0306741
FBgn0037391	FBgn0037391	NA	FBtr0078664
FBgn0037395	FBgn0037395	NA	FBtr0309318
FBgn0037397	FBgn0037397	NA	FBtr0307114
FBgn0037398	FBgn0037398	NA	FBtr0078621
FBgn0037402	FBgn0037402	NA	FBtr0309512
FBgn0037408	FBgn0037408	NPFR1	FBtr0078590
FBgn0037410	FBgn0037410	Osi2	FBtr0078593
FBgn0037411	FBgn0037411	Osi3	FBtr0310414
FBgn0037413	FBgn0037413	NA	FBtr0078595
FBgn0037414	FBgn0037414	Osi7	FBtr0078597
FBgn0037420	FBgn0037420	NA	FBtr0302305
FBgn0037429	FBgn0037429	NA	FBtr0081707
FBgn0037430	FBgn0037430	Osi20	FBtr0081708
FBgn0037431	FBgn0037431	NA	FBtr0081709
FBgn0037432	FBgn0037432	NA	FBtr0081710
FBgn0037433	FBgn0037433	CG17919	FBtr0081711
FBgn0037434	FBgn0037434	NA	FBtr0081737
FBgn0037435	FBgn0037435	CG18048	FBtr0081712
FBgn0037440	FBgn0037440	NA	FBtr0081734
FBgn0037442	FBgn0037442	NA	FBtr0081721
FBgn0037443	FBgn0037443	NA	FBtr0289939
FBgn0037445	FBgn0037445	NA	FBtr0081730
FBgn0037446	FBgn0037446	NA	FBtr0081728
FBgn0037448	FBgn0037448	NA	FBtr0308038
FBgn0037455	FBgn0037455	NA	FBtr0089748
FBgn0037460	FBgn0037460	NA	FBtr0081701
FBgn0037461	FBgn0037461	NA	FBtr0081700
FBgn0037462	FBgn0037462	NA	FBtr0081681
FBgn0037463	FBgn0037463	NA	FBtr0081631
FBgn0037464	FBgn0037464	NA	FBtr0081633
FBgn0037465	FBgn0037465	NA	FBtr0081645
FBgn0037466	FBgn0037466	NA	FBtr0305315
FBgn0037467	FBgn0037467	CG1104	FBtr0081643
FBgn0037471	FBgn0037471	NA	FBtr0300494
FBgn0037472	FBgn0037472	CG10098	FBtr0081563
FBgn0037473	FBgn0037473	CG10068	FBtr0081611
FBgn0037475	FBgn0037475	Fer1	FBtr0081567
FBgn0037477	FBgn0037477	NA	FBtr0303197
FBgn0037482	FBgn0037482	NA	FBtr0081605
FBgn0037483	FBgn0037483	NA	FBtr0081573
FBgn0037485	FBgn0037485	NA	FBtr0081603
FBgn0037487	FBgn0037487	NA	FBtr0303219
FBgn0037488	FBgn0037488	NA	FBtr0303218
FBgn0037489	FBgn0037489	CG1234	FBtr0081599

FBgn0037490	FBgn0037490	NA	FBtr0081576
FBgn0037491	FBgn0037491	CG1227	FBtr0081598
FBgn0037492	FBgn0037492	CG10050	FBtr0081577
FBgn0037498	FBgn0037498	NA	FBtr0081591
FBgn0037502	FBgn0037502	NA	FBtr0301649
FBgn0037504	FBgn0037504	NA	FBtr0081559
FBgn0037506	FBgn0037506	NA	FBtr0081554
FBgn0037514	FBgn0037514	NA	FBtr0081739
FBgn0037515	FBgn0037515	NA	FBtr0081742
FBgn0037516	FBgn0037516	NA	FBtr0306042
FBgn0037519	FBgn0037519	NA	FBtr0299818
FBgn0037525	FBgn0037525	NA	FBtr0113203
FBgn0037526	FBgn0037526	CG10092	FBtr0081752
FBgn0037531	FBgn0037531	NA	FBtr0304704
FBgn0037535	FBgn0037535	NA	FBtr0081783
FBgn0037536	FBgn0037536	NA	FBtr0308075
FBgn0037537	FBgn0037537	CG2767	FBtr0290325
FBgn0037540	FBgn0037540	NA	FBtr0081786
FBgn0037541	FBgn0037541	NA	FBtr0081817
FBgn0037543	FBgn0037543	CG10903	FBtr0310411
FBgn0037544	FBgn0037544	NA	FBtr0081816
FBgn0037548	FBgn0037548	NA	FBtr0113204
FBgn0037549	FBgn0037549	NA	FBtr0113205
FBgn0037552	FBgn0037552	NA	FBtr0081795
FBgn0037553	FBgn0037553	NA	FBtr0302178
FBgn0037556	FBgn0037556	NA	FBtr0081806
FBgn0037560	FBgn0037560	NA	FBtr0300631
FBgn0037565	FBgn0037565	NA	FBtr0081864
FBgn0037566	FBgn0037566	mRpL1	FBtr0081832
FBgn0037567	FBgn0037567	NA	FBtr0081863
FBgn0037568	FBgn0037568	NA	FBtr0081862
FBgn0037569	FBgn0037569	NA	FBtr0081861
FBgn0037570	FBgn0037570	NA	FBtr0081860
FBgn0037577	FBgn0037577	NA	FBtr0081837
FBgn0037578	FBgn0037578	NA	FBtr0081854
FBgn0037579	FBgn0037579	NA	FBtr0081839
FBgn0037580	FBgn0037580	DppIII	FBtr0081841
FBgn0037581	FBgn0037581	NA	FBtr0081842
FBgn0037583	FBgn0037583	NA	FBtr0081853
FBgn0037584	FBgn0037584	NA	FBtr0301759
FBgn0037592	FBgn0037592	CG11737	FBtr0310049
FBgn0037602	FBgn0037602	NA	FBtr0081878
FBgn0037603	FBgn0037603	NA	FBtr0081879
FBgn0037608	FBgn0037608	mRpL19	FBtr0081901
FBgn0037609	FBgn0037609	CG9773	FBtr0081953
FBgn0037610	FBgn0037610	NA	FBtr0081902
FBgn0037611	FBgn0037611	NA	FBtr0081903
FBgn0037612	FBgn0037612	CG8112	FBtr0081904
FBgn0037613	FBgn0037613	Cks85A	FBtr0305339
FBgn0037614	FBgn0037614	NA	FBtr0300543
FBgn0037615	FBgn0037615	CG11760	FBtr0081907
FBgn0037616	FBgn0037616	NA	FBtr0081909
FBgn0037617	FBgn0037617	NA	FBtr0081911
FBgn0037618	FBgn0037618	NA	FBtr0081912
FBgn0037619	FBgn0037619	NA	FBtr0301194
FBgn0037620	FBgn0037620	NA	FBtr0081948
FBgn0037621	FBgn0037621	CG9797	FBtr0081947

FBgn0037623	FBgn0037623	NA	FBtr0081944
FBgn0037624	FBgn0037624	CG8223	FBtr0308889
FBgn0037625	FBgn0037625	NA	FBtr0308890
FBgn0037626	FBgn0037626	NA	FBtr0081916
FBgn0037627	FBgn0037627	CG13318	FBtr0081942
FBgn0037633	FBgn0037633	NA	FBtr0081934
FBgn0037634	FBgn0037634	CG8359	FBtr0081919
FBgn0037635	FBgn0037635	CG9837	FBtr0309098
FBgn0037636	FBgn0037636	NA	FBtr0081931
FBgn0037637	FBgn0037637	CG9836	FBtr0081930
FBgn0037638	FBgn0037638	CG8379	FBtr0081922
FBgn0037643	FBgn0037643	NA	FBtr0303526
FBgn0037644	FBgn0037644	CG11964	FBtr0082015
FBgn0037645	FBgn0037645	NA	FBtr0082014
FBgn0037646	FBgn0037646	CG11967	FBtr0081958
FBgn0037647	FBgn0037647	NA	FBtr0082013
FBgn0037650	FBgn0037650	NA	FBtr0300742
FBgn0037653	FBgn0037653	CG11982	FBtr0081965
FBgn0037654	FBgn0037654	NA	FBtr0081966
FBgn0037655	FBgn0037655	NA	FBtr0300748
FBgn0037656	FBgn0037656	NA	FBtr0081967
FBgn0037657	FBgn0037657	NA	FBtr0300415
FBgn0037659	FBgn0037659	NA	FBtr0300691
FBgn0037661	FBgn0037661	NA	FBtr0081971
FBgn0037662	FBgn0037662	NA	FBtr0081972
FBgn0037663	FBgn0037663	NA	FBtr0081986
FBgn0037666	FBgn0037666	NA	FBtr0303309
FBgn0037667	FBgn0037667	NA	FBtr0302133
FBgn0037668	FBgn0037668	NA	FBtr0081985
FBgn0037669	FBgn0037669	NA	FBtr0081984
FBgn0037670	FBgn0037670	NA	FBtr0081977
FBgn0037675	FBgn0037675	NA	FBtr0082016
FBgn0037676	FBgn0037676	CG8861	FBtr0301210
FBgn0037678	FBgn0037678	NA	FBtr0082057
FBgn0037689	FBgn0037689	NA	FBtr0082030
FBgn0037690	FBgn0037690	NA	FBtr0082045
FBgn0037700	FBgn0037700	NA	FBtr0082061
FBgn0037702	FBgn0037702	NA	FBtr0113208
FBgn0037705	FBgn0037705	NA	FBtr0082118
FBgn0037707	FBgn0037707	RnpS1	FBtr0082066
FBgn0037708	FBgn0037708	CG9386	FBtr0082115
FBgn0037712	FBgn0037712	CG16789	FBtr0082071
FBgn0037713	FBgn0037713	NA	FBtr0082112
FBgn0037715	FBgn0037715	CG9399	FBtr0302332
FBgn0037716	FBgn0037716	NA	FBtr0082072
FBgn0037717	FBgn0037717	CG8301	FBtr0082073
FBgn0037718	FBgn0037718	CG8286	FBtr0082074
FBgn0037719	FBgn0037719	NA	FBtr0100537
FBgn0037722	FBgn0037722	NA	FBtr0082077
FBgn0037723	FBgn0037723	SpdS	FBtr0082078
FBgn0037724	FBgn0037724	NA	FBtr0082101
FBgn0037726	FBgn0037726	NA	FBtr0309027
FBgn0037727	FBgn0037727	NA	FBtr0082081
FBgn0037728	FBgn0037728	NA	FBtr0082082
FBgn0037730	FBgn0037730	CG9444	FBtr0082099
FBgn0037731	FBgn0037731	NA	FBtr0082084
FBgn0037737	FBgn0037737	NA	FBtr0113210

FBgn0037742	FBgn0037742	NA	FBtr0301536
FBgn0037743	FBgn0037743	NA	FBtr0082130
FBgn0037744	FBgn0037744	CG8417	FBtr0082131
FBgn0037746	FBgn0037746	NA	FBtr0301871
FBgn0037747	FBgn0037747	NA	FBtr0100583
FBgn0037749	FBgn0037749	NA	FBtr0082160
FBgn0037750	FBgn0037750	NA	FBtr0110789
FBgn0037751	FBgn0037751	NA	FBtr0082135
FBgn0037752	FBgn0037752	NA	FBtr0082136
FBgn0037753	FBgn0037753	NA	FBtr0113212
FBgn0037755	FBgn0037755	NA	FBtr0113214
FBgn0037757	FBgn0037757	NA	FBtr0082141
FBgn0037758	FBgn0037758	NA	FBtr0082156
FBgn0037759	FBgn0037759	NA	FBtr0082142
FBgn0037761	FBgn0037761	NA	FBtr0082143
FBgn0037762	FBgn0037762	NA	FBtr0082144
FBgn0037763	FBgn0037763	NA	FBtr0082154
FBgn0037764	FBgn0037764	NA	FBtr0082153
FBgn0037765	FBgn0037765	NA	FBtr0082152
FBgn0037769	FBgn0037769	NA	FBtr0082148
FBgn0037770	FBgn0037770	Art4	FBtr0082150
FBgn0037772	FBgn0037772	NA	FBtr0082171
FBgn0037773	FBgn0037773	CG5359	FBtr0082215
FBgn0037777	FBgn0037777	CG11722	FBtr0082205
FBgn0037778	FBgn0037778	mtTFB2	FBtr0082173
FBgn0037779	FBgn0037779	NA	FBtr0082204
FBgn0037780	FBgn0037780	CG3925	FBtr0082174
FBgn0037781	FBgn0037781	NA	FBtr0082203
FBgn0037782	FBgn0037782	NA	FBtr0082202
FBgn0037786	FBgn0037786	NA	FBtr0309853
FBgn0037789	FBgn0037789	NA	FBtr0082195
FBgn0037792	FBgn0037792	NA	FBtr0082185
FBgn0037794	FBgn0037794	NA	FBtr0082184
FBgn0037796	FBgn0037796	NA	FBtr0110786
FBgn0037797	FBgn0037797	CG12420	FBtr0082222
FBgn0037798	FBgn0037798	CG12817	FBtr0110952
FBgn0037801	FBgn0037801	CG3999	FBtr0082225
FBgn0037802	FBgn0037802	NA	FBtr0305358
FBgn0037809	FBgn0037809	NA	FBtr0082230
FBgn0037814	FBgn0037814	NA	FBtr0082260
FBgn0037817	FBgn0037817	NA	FBtr0301283
FBgn0037819	FBgn0037819	NA	FBtr0113215
FBgn0037822	FBgn0037822	NA	FBtr0082267
FBgn0037824	FBgn0037824	NA	FBtr0082268
FBgn0037831	FBgn0037831	NA	FBtr0301310
FBgn0037832	FBgn0037832	NA	FBtr0082274
FBgn0037834	FBgn0037834	NA	FBtr0082304
FBgn0037836	FBgn0037836	NA	FBtr0302798
FBgn0037837	FBgn0037837	CG14693	FBtr0300078
FBgn0037838	FBgn0037838	CG4089	FBtr0082281
FBgn0037841	FBgn0037841	NA	FBtr0113216
FBgn0037842	FBgn0037842	NA	FBtr0113217
FBgn0037843	FBgn0037843	CG4511	FBtr0082286
FBgn0037845	FBgn0037845	NA	FBtr0082298
FBgn0037846	FBgn0037846	NA	FBtr0082297
FBgn0037847	FBgn0037847	SelR	FBtr0306641
FBgn0037848	FBgn0037848	NA	FBtr0082288

FBgn0037849	FBgn0037849	CG4596	FBtr0082289
FBgn0037850	FBgn0037850	NA	FBtr0082335
FBgn0037852	FBgn0037852	CG6608	FBtr0082333
FBgn0037853	FBgn0037853	NA	FBtr0082332
FBgn0037855	FBgn0037855	NA	FBtr0082329
FBgn0037856	FBgn0037856	NA	FBtr0082325
FBgn0037857	FBgn0037857	NA	FBtr0082326
FBgn0037862	FBgn0037862	CG4706	FBtr0082336
FBgn0037870	FBgn0037870	NA	FBtr0082341
FBgn0037872	FBgn0037872	NA	FBtr0113219
FBgn0037873	FBgn0037873	CG6666	FBtr0082372
FBgn0037874	FBgn0037874	NA	FBtr0082344
FBgn0037875	FBgn0037875	NA	FBtr0082371
FBgn0037876	FBgn0037876	NA	FBtr0113221
FBgn0037877	FBgn0037877	NA	FBtr0082368
FBgn0037878	FBgn0037878	CG6693	FBtr0082367
FBgn0037879	FBgn0037879	NA	FBtr0082351
FBgn0037880	FBgn0037880	CG17726	FBtr0082352
FBgn0037881	FBgn0037881	NA	FBtr0082366
FBgn0037882	FBgn0037882	NA	FBtr0082365
FBgn0037883	FBgn0037883	NA	FBtr0082353
FBgn0037884	FBgn0037884	NA	FBtr0082363
FBgn0037885	FBgn0037885	NA	FBtr0082354
FBgn0037888	FBgn0037888	NA	FBtr0082362
FBgn0037889	FBgn0037889	NA	FBtr0082357
FBgn0037890	FBgn0037890	NA	FBtr0308073
FBgn0037892	FBgn0037892	mRpL40	FBtr0082384
FBgn0037893	FBgn0037893	NA	FBtr0082406
FBgn0037894	FBgn0037894	Ranbp9	FBtr0082386
FBgn0037895	FBgn0037895	CG6723	FBtr0082405
FBgn0037896	FBgn0037896	NA	FBtr0082404
FBgn0037897	FBgn0037897	CG5270	FBtr0082387
FBgn0037898	FBgn0037898	NA	FBtr0300901
FBgn0037899	FBgn0037899	NA	FBtr0082403
FBgn0037900	FBgn0037900	NA	FBtr0082390
FBgn0037901	FBgn0037901	CG6744	FBtr0082402
FBgn0037902	FBgn0037902	NA	FBtr0082391
FBgn0037906	FBgn0037906	NA	FBtr0306099
FBgn0037910	FBgn0037910	NA	FBtr0082409
FBgn0037911	FBgn0037911	NA	FBtr0082410
FBgn0037915	FBgn0037915	NA	FBtr0082460
FBgn0037916	FBgn0037916	NA	FBtr0301159
FBgn0037917	FBgn0037917	wkd	FBtr0082414
FBgn0037920	FBgn0037920	NA	FBtr0299566
FBgn0037921	FBgn0037921	NA	FBtr0082451
FBgn0037922	FBgn0037922	NA	FBtr0082416
FBgn0037923	FBgn0037923	NA	FBtr0300580
FBgn0037930	FBgn0037930	CG14715	FBtr0082423
FBgn0037931	FBgn0037931	NA	FBtr0082443
FBgn0037941	FBgn0037941	NA	FBtr0082429
FBgn0037943	FBgn0037943	NA	FBtr0082431
FBgn0037944	FBgn0037944	NA	FBtr0307892
FBgn0037950	FBgn0037950	NA	FBtr0082470
FBgn0037955	FBgn0037955	NA	FBtr0082520
FBgn0037956	FBgn0037956	NA	FBtr0299594
FBgn0037958	FBgn0037958	CG6962	FBtr0082516
FBgn0037960	FBgn0037960	mthl5	FBtr0082515

FBgn0037963	FBgn0037963	NA	FBtr0082513
FBgn0037968	FBgn0037968	NA	FBtr0113223
FBgn0037970	FBgn0037970	NA	FBtr0273272
FBgn0037972	FBgn0037972	NA	FBtr0082507
FBgn0037973	FBgn0037973	NA	FBtr0082506
FBgn0037974	FBgn0037974	NA	FBtr0306697
FBgn0037975	FBgn0037975	NA	FBtr0082504
FBgn0037979	FBgn0037979	NA	FBtr0113224
FBgn0037980	FBgn0037980	CG3313	FBtr0082555
FBgn0037981	FBgn0037981	NA	FBtr0082537
FBgn0037985	FBgn0037985	NA	FBtr0082538
FBgn0037986	FBgn0037986	NA	FBtr0301723
FBgn0037988	FBgn0037988	NA	FBtr0082552
FBgn0037989	FBgn0037989	NA	FBtr0300351
FBgn0037992	FBgn0037992	CG4702	FBtr0308292
FBgn0037994	FBgn0037994	NA	FBtr0082546
FBgn0037995	FBgn0037995	NA	FBtr0082545
FBgn0037997	FBgn0037997	NA	FBtr0306051
FBgn0037999	FBgn0037999	CG4860	FBtr0082623
FBgn0038000	FBgn0038000	CG10014	FBtr0082559
FBgn0038001	FBgn0038001	NA	FBtr0302311
FBgn0038002	FBgn0038002	NA	FBtr0082621
FBgn0038006	FBgn0038006	NA	FBtr0308561
FBgn0038008	FBgn0038008	NA	FBtr0082561
FBgn0038009	FBgn0038009	NA	FBtr0082565
FBgn0038013	FBgn0038013	NA	FBtr0082615
FBgn0038014	FBgn0038014	NA	FBtr0082613
FBgn0038016	FBgn0038016	NA	FBtr0082611
FBgn0038029	FBgn0038029	NA	FBtr0082576
FBgn0038035	FBgn0038035	NA	FBtr0082600
FBgn0038042	FBgn0038042	Scgbeta	FBtr0082596
FBgn0038043	FBgn0038043	NA	FBtr0082587
FBgn0038046	FBgn0038046	CG5641	FBtr0082594
FBgn0038047	FBgn0038047	NA	FBtr0082589
FBgn0038054	FBgn0038054	NA	FBtr0082633
FBgn0038055	FBgn0038055	NA	FBtr0082634
FBgn0038056	FBgn0038056	CG5961	FBtr0082681
FBgn0038057	FBgn0038057	CG12267	FBtr0303028
FBgn0038063	FBgn0038063	NA	FBtr0304846
FBgn0038065	FBgn0038065	NA	FBtr0082641
FBgn0038067	FBgn0038067	NA	FBtr0110781
FBgn0038068	FBgn0038068	NA	FBtr0303840
FBgn0038069	FBgn0038069	NA	FBtr0303841
FBgn0038070	FBgn0038070	NA	FBtr0301150
FBgn0038072	FBgn0038072	CG6225	FBtr0113225
FBgn0038073	FBgn0038073	CG14395	FBtr0310613
FBgn0038074	FBgn0038074	CG6188	FBtr0082651
FBgn0038076	FBgn0038076	NA	FBtr0082667
FBgn0038078	FBgn0038078	NA	FBtr0082663
FBgn0038079	FBgn0038079	NA	FBtr0113226
FBgn0038080	FBgn0038080	NA	FBtr0082656
FBgn0038082	FBgn0038082	NA	FBtr0082657
FBgn0038083	FBgn0038083	NA	FBtr0302379
FBgn0038084	FBgn0038084	NA	FBtr0082658
FBgn0038087	FBgn0038087	NA	FBtr0082688
FBgn0038089	FBgn0038089	NA	FBtr0082683
FBgn0038090	FBgn0038090	NA	FBtr0082685

FBgn0038097	FBgn0038097	NA	FBtr0082731
FBgn0038098	FBgn0038098	CG7381	FBtr0301219
FBgn0038099	FBgn0038099	NA	FBtr0300278
FBgn0038102	FBgn0038102	NA	FBtr0082724
FBgn0038106	FBgn0038106	NA	FBtr0082695
FBgn0038108	FBgn0038108	NA	FBtr0301224
FBgn0038109	FBgn0038109	NA	FBtr0082698
FBgn0038110	FBgn0038110	NA	FBtr0082719
FBgn0038111	FBgn0038111	CG12360	FBtr0082717
FBgn0038113	FBgn0038113	NA	FBtr0082705
FBgn0038114	FBgn0038114	CG11670	FBtr0300072
FBgn0038122	FBgn0038122	NA	FBtr0082732
FBgn0038123	FBgn0038123	NA	FBtr0082783
FBgn0038124	FBgn0038124	NA	FBtr0300768
FBgn0038125	FBgn0038125	NA	FBtr0082734
FBgn0038135	FBgn0038135	NA	FBtr0290037
FBgn0038136	FBgn0038136	NA	FBtr0082740
FBgn0038140	FBgn0038140	NA	FBtr0082745
FBgn0038142	FBgn0038142	NA	FBtr0082749
FBgn0038146	FBgn0038146	NA	FBtr0082763
FBgn0038147	FBgn0038147	CG14375	FBtr0303263
FBgn0038148	FBgn0038148	NA	FBtr0082756
FBgn0038149	FBgn0038149	CG9796	FBtr0082760
FBgn0038150	FBgn0038150	yellow-e3	FBtr0082759
FBgn0038151	FBgn0038151	NA	FBtr0302576
FBgn0038156	FBgn0038156	NA	FBtr0309090
FBgn0038157	FBgn0038157	NA	FBtr0082791
FBgn0038158	FBgn0038158	NA	FBtr0082788
FBgn0038160	FBgn0038160	NA	FBtr0082861
FBgn0038163	FBgn0038163	NA	FBtr0082795
FBgn0038165	FBgn0038165	NA	FBtr0301654
FBgn0038166	FBgn0038166	NA	FBtr0082849
FBgn0038167	FBgn0038167	lkb1	FBtr0301488
FBgn0038173	FBgn0038173	NA	FBtr0082821
FBgn0038175	FBgn0038175	NA	FBtr0082838
FBgn0038182	FBgn0038182	NA	FBtr0082829
FBgn0038186	FBgn0038186	NA	FBtr0082881
FBgn0038188	FBgn0038188	NA	FBtr0082879
FBgn0038189	FBgn0038189	NA	FBtr0082878
FBgn0038194	FBgn0038194	Cyp6d5	FBtr0089766
FBgn0038197	FBgn0038197	foxo	FBtr0082886
FBgn0038198	FBgn0038198	CG3153	FBtr0082889
FBgn0038199	FBgn0038199	NA	FBtr0113232
FBgn0038200	FBgn0038200	NA	FBtr0082917
FBgn0038201	FBgn0038201	NA	FBtr0305681
FBgn0038202	FBgn0038202	NA	FBtr0082914
FBgn0038204	FBgn0038204	NA	FBtr0301280
FBgn0038205	FBgn0038205	NA	FBtr0082912
FBgn0038206	FBgn0038206	twf	FBtr0082891
FBgn0038208	FBgn0038208	CG14355	FBtr0082907
FBgn0038209	FBgn0038209	NA	FBtr0082906
FBgn0038210	FBgn0038210	NA	FBtr0082894
FBgn0038211	FBgn0038211	NA	FBtr0082902
FBgn0038213	FBgn0038213	NA	FBtr0082897
FBgn0038214	FBgn0038214	NA	FBtr0300059
FBgn0038220	FBgn0038220	CG12207	FBtr0082921
FBgn0038221	FBgn0038221	NA	FBtr0082924

FBgn0038223	FBgn0038223	NA	FBtr0310001
FBgn0038224	FBgn0038224	NA	FBtr0303795
FBgn0038235	FBgn0038235	NA	FBtr0082986
FBgn0038236	FBgn0038236	NA	FBtr0082954
FBgn0038237	FBgn0038237	NA	FBtr0300330
FBgn0038238	FBgn0038238	NA	FBtr0113233
FBgn0038239	FBgn0038239	NA	FBtr0082955
FBgn0038248	FBgn0038248	NA	FBtr0082976
FBgn0038250	FBgn0038250	CG3505	FBtr0082966
FBgn0038251	FBgn0038251	NA	FBtr0082968
FBgn0038252	FBgn0038252	NA	FBtr0082969
FBgn0038257	FBgn0038257	smp-30	FBtr0083017
FBgn0038260	FBgn0038260	NA	FBtr0082991
FBgn0038262	FBgn0038262	NA	FBtr0082993
FBgn0038266	FBgn0038266	NA	FBtr0082994
FBgn0038268	FBgn0038268	NA	FBtr0082997
FBgn0038269	FBgn0038269	NA	FBtr0113235
FBgn0038271	FBgn0038271	NA	FBtr0083000
FBgn0038272	FBgn0038272	NA	FBtr0083009
FBgn0038273	FBgn0038273	NA	FBtr0289976
FBgn0038274	FBgn0038274	NA	FBtr0083008
FBgn0038275	FBgn0038275	NA	FBtr0083003
FBgn0038276	FBgn0038276	NA	FBtr0290022
FBgn0038277	FBgn0038277	RpS5b	FBtr0308031
FBgn0038286	FBgn0038286	NA	FBtr0113236
FBgn0038290	FBgn0038290	NA	FBtr0083038
FBgn0038294	FBgn0038294	Zeelin1	FBtr0306642
FBgn0038295	FBgn0038295	NA	FBtr0110961
FBgn0038296	FBgn0038296	CG6752	FBtr0083029
FBgn0038300	FBgn0038300	NA	FBtr0083048
FBgn0038301	FBgn0038301	NA	FBtr0083139
FBgn0038302	FBgn0038302	NA	FBtr0309248
FBgn0038303	FBgn0038303	NA	FBtr0083138
FBgn0038306	FBgn0038306	NA	FBtr0083135
FBgn0038307	FBgn0038307	mRpS10	FBtr0083054
FBgn0038309	FBgn0038309	NA	FBtr0113238
FBgn0038311	FBgn0038311	NA	FBtr0083128
FBgn0038312	FBgn0038312	NA	FBtr0290018
FBgn0038315	FBgn0038315	CG14866	FBtr0083081
FBgn0038323	FBgn0038323	NA	FBtr0083116
FBgn0038324	FBgn0038324	NA	FBtr0083089
FBgn0038327	FBgn0038327	NA	FBtr0083092
FBgn0038330	FBgn0038330	CG14868	FBtr0083109
FBgn0038331	FBgn0038331	CG5073	FBtr0083094
FBgn0038332	FBgn0038332	CG6136	FBtr0303236
FBgn0038339	FBgn0038339	NA	FBtr0113239
FBgn0038342	FBgn0038342	NA	FBtr0083145
FBgn0038343	FBgn0038343	NA	FBtr0300174
FBgn0038359	FBgn0038359	NA	FBtr0083171
FBgn0038365	FBgn0038365	NA	FBtr0307203
FBgn0038369	FBgn0038369	NA	FBtr0300571
FBgn0038371	FBgn0038371	NA	FBtr0083190
FBgn0038381	FBgn0038381	NA	FBtr0083200
FBgn0038386	FBgn0038386	NA	FBtr0083242
FBgn0038387	FBgn0038387	blp	FBtr0083203
FBgn0038388	FBgn0038388	CG4287	FBtr0083240
FBgn0038389	FBgn0038389	CG5516	FBtr0083212

FBgn0038390	FBgn0038390	Rbf2	FBtr0083213
FBgn0038391	FBgn0038391	GATAe	FBtr0083218
FBgn0038394	FBgn0038394	NA	FBtr0083222
FBgn0038395	FBgn0038395	NA	FBtr0083237
FBgn0038396	FBgn0038396	NA	FBtr0083223
FBgn0038398	FBgn0038398	NA	FBtr0083225
FBgn0038402	FBgn0038402	NA	FBtr0301709
FBgn0038404	FBgn0038404	NA	FBtr0083253
FBgn0038410	FBgn0038410	NA	FBtr0083275
FBgn0038414	FBgn0038414	NA	FBtr0083269
FBgn0038415	FBgn0038415	NA	FBtr0273219
FBgn0038416	FBgn0038416	NA	FBtr0083271
FBgn0038418	FBgn0038418	NA	FBtr0301514
FBgn0038419	FBgn0038419	CG14879	FBtr0083325
FBgn0038421	FBgn0038421	NA	FBtr0083323
FBgn0038423	FBgn0038423	NA	FBtr0300677
FBgn0038424	FBgn0038424	CG17565	FBtr0083286
FBgn0038425	FBgn0038425	CG14881	FBtr0083288
FBgn0038426	FBgn0038426	NA	FBtr0083316
FBgn0038428	FBgn0038428	NA	FBtr0083314
FBgn0038429	FBgn0038429	NA	FBtr0083289
FBgn0038431	FBgn0038431	NA	FBtr0110773
FBgn0038432	FBgn0038432	CG14883	FBtr0083291
FBgn0038435	FBgn0038435	NA	FBtr0110963
FBgn0038436	FBgn0038436	NA	FBtr0083294
FBgn0038437	FBgn0038437	CG14898	FBtr0083308
FBgn0038438	FBgn0038438	NA	FBtr0083307
FBgn0038439	FBgn0038439	NA	FBtr0301702
FBgn0038445	FBgn0038445	NA	FBtr0083301
FBgn0038446	FBgn0038446	NA	FBtr0083303
FBgn0038447	FBgn0038447	NA	FBtr0083327
FBgn0038450	FBgn0038450	NA	FBtr0083330
FBgn0038451	FBgn0038451	NA	FBtr0300865
FBgn0038455	FBgn0038455	NA	FBtr0113242
FBgn0038458	FBgn0038458	NA	FBtr0083366
FBgn0038466	FBgn0038466	CG8907	FBtr0083372
FBgn0038467	FBgn0038467	NA	FBtr0083373
FBgn0038468	FBgn0038468	NA	FBtr0083389
FBgn0038469	FBgn0038469	NA	FBtr0305130
FBgn0038470	FBgn0038470	NA	FBtr0305129
FBgn0038471	FBgn0038471	CG5220	FBtr0083391
FBgn0038472	FBgn0038472	NA	FBtr0083428
FBgn0038473	FBgn0038473	NA	FBtr0083426
FBgn0038474	FBgn0038474	mRpS11	FBtr0089894
FBgn0038475	FBgn0038475	Keap1	FBtr0083422
FBgn0038476	FBgn0038476	NA	FBtr0083397
FBgn0038478	FBgn0038478	NA	FBtr0083400
FBgn0038479	FBgn0038479	NA	FBtr0083418
FBgn0038481	FBgn0038481	NA	FBtr0083417
FBgn0038482	FBgn0038482	NA	FBtr0303413
FBgn0038484	FBgn0038484	NA	FBtr0083405
FBgn0038485	FBgn0038485	NA	FBtr0083406
FBgn0038490	FBgn0038490	NA	FBtr0083409
FBgn0038491	FBgn0038491	CG5292	FBtr0083410
FBgn0038494	FBgn0038494	NA	FBtr0300610
FBgn0038499	FBgn0038499	NA	FBtr0083452
FBgn0038504	FBgn0038504	NA	FBtr0083447

FBgn0038505	FBgn0038505	NA	FBtr0083437
FBgn0038506	FBgn0038506	NA	FBtr0083438
FBgn0038508	FBgn0038508	NA	FBtr0303755
FBgn0038509	FBgn0038509	NA	FBtr0083446
FBgn0038510	FBgn0038510	NA	FBtr0113244
FBgn0038512	FBgn0038512	NA	FBtr0083443
FBgn0038515	FBgn0038515	CG5823	FBtr0083505
FBgn0038516	FBgn0038516	CG5840	FBtr0083455
FBgn0038523	FBgn0038523	NA	FBtr0083460
FBgn0038524	FBgn0038524	NA	FBtr0305308
FBgn0038525	FBgn0038525	NA	FBtr0083463
FBgn0038526	FBgn0038526	NA	FBtr0083465
FBgn0038527	FBgn0038527	CG14324	FBtr0083500
FBgn0038528	FBgn0038528	CG14326	FBtr0083467
FBgn0038530	FBgn0038530	NA	FBtr0083470
FBgn0038531	FBgn0038531	NA	FBtr0289974
FBgn0038532	FBgn0038532	NA	FBtr0083476
FBgn0038533	FBgn0038533	NA	FBtr0083490
FBgn0038538	FBgn0038538	NA	FBtr0083508
FBgn0038540	FBgn0038540	NA	FBtr0083511
FBgn0038541	FBgn0038541	NA	FBtr0083512
FBgn0038542	FBgn0038542	NA	FBtr0083532
FBgn0038545	FBgn0038545	CG7713	FBtr0305007
FBgn0038546	FBgn0038546	CG7379	FBtr0083529
FBgn0038551	FBgn0038551	CG7357	FBtr0083524
FBgn0038552	FBgn0038552	NA	FBtr0083523
FBgn0038558	FBgn0038558	NA	FBtr0308497
FBgn0038564	FBgn0038564	NA	FBtr0083539
FBgn0038568	FBgn0038568	NA	FBtr0083547
FBgn0038569	FBgn0038569	CG7218	FBtr0083623
FBgn0038570	FBgn0038570	NA	FBtr0100619
FBgn0038571	FBgn0038571	NA	FBtr0100283
FBgn0038574	FBgn0038574	NA	FBtr0083619
FBgn0038575	FBgn0038575	NA	FBtr0083617
FBgn0038576	FBgn0038576	CG7940	FBtr0083557
FBgn0038577	FBgn0038577	CG12321	FBtr0083616
FBgn0038578	FBgn0038578	MED17	FBtr0083558
FBgn0038579	FBgn0038579	NA	FBtr0083615
FBgn0038582	FBgn0038582	NA	FBtr0083561
FBgn0038584	FBgn0038584	CG7175	FBtr0083609
FBgn0038585	FBgn0038585	CG7993	FBtr0083562
FBgn0038586	FBgn0038586	CG7168	FBtr0083608
FBgn0038587	FBgn0038587	NA	FBtr0083563
FBgn0038588	FBgn0038588	CG7156	FBtr0083606
FBgn0038589	FBgn0038589	NA	FBtr0083569
FBgn0038590	FBgn0038590	NA	FBtr0083605
FBgn0038595	FBgn0038595	NA	FBtr0083600
FBgn0038596	FBgn0038596	NA	FBtr0083599
FBgn0038597	FBgn0038597	CG8064	FBtr0083574
FBgn0038598	FBgn0038598	NA	FBtr0083597
FBgn0038603	FBgn0038603	NA	FBtr0083589
FBgn0038606	FBgn0038606	NA	FBtr0302308
FBgn0038607	FBgn0038607	NA	FBtr0083576
FBgn0038608	FBgn0038608	NA	FBtr0302307
FBgn0038609	FBgn0038609	CG7671	FBtr0083577
FBgn0038611	FBgn0038611	CG14309	FBtr0083580
FBgn0038612	FBgn0038612	NA	FBtr0083624

FBgn0038619	FBgn0038619	NA	FBtr0083630
FBgn0038626	FBgn0038626	NA	FBtr0083631
FBgn0038627	FBgn0038627	NA	FBtr0083632
FBgn0038628	FBgn0038628	NA	FBtr0113245
FBgn0038629	FBgn0038629	NA	FBtr0083639
FBgn0038630	FBgn0038630	NA	FBtr0083636
FBgn0038631	FBgn0038631	NA	FBtr0083694
FBgn0038632	FBgn0038632	NA	FBtr0290199
FBgn0038636	FBgn0038636	NA	FBtr0083690
FBgn0038639	FBgn0038639	NA	FBtr0083689
FBgn0038640	FBgn0038640	NA	FBtr0083667
FBgn0038643	FBgn0038643	NA	FBtr0100288
FBgn0038647	FBgn0038647	NA	FBtr0083673
FBgn0038649	FBgn0038649	CG7718	FBtr0083682
FBgn0038651	FBgn0038651	CG14299	FBtr0273382
FBgn0038652	FBgn0038652	CG7720	FBtr0301200
FBgn0038653	FBgn0038653	NA	FBtr0083677
FBgn0038654	FBgn0038654	NA	FBtr0083695
FBgn0038655	FBgn0038655	NA	FBtr0083696
FBgn0038656	FBgn0038656	NA	FBtr0083726
FBgn0038659	FBgn0038659	endoA	FBtr0083699
FBgn0038660	FBgn0038660	CG14291	FBtr0083724
FBgn0038661	FBgn0038661	NA	FBtr0083700
FBgn0038662	FBgn0038662	NA	FBtr0083723
FBgn0038665	FBgn0038665	NA	FBtr0304691
FBgn0038666	FBgn0038666	CG5451	FBtr0304690
FBgn0038672	FBgn0038672	NA	FBtr0083719
FBgn0038673	FBgn0038673	NA	FBtr0083710
FBgn0038674	FBgn0038674	NA	FBtr0083711
FBgn0038675	FBgn0038675	CG6013	FBtr0083714
FBgn0038676	FBgn0038676	NA	FBtr0273301
FBgn0038678	FBgn0038678	mRpL55	FBtr0083754
FBgn0038679	FBgn0038679	CG6040	FBtr0083729
FBgn0038680	FBgn0038680	NA	FBtr0083730
FBgn0038681	FBgn0038681	NA	FBtr0083731
FBgn0038682	FBgn0038682	NA	FBtr0083751
FBgn0038683	FBgn0038683	NA	FBtr0083750
FBgn0038685	FBgn0038685	NA	FBtr0083734
FBgn0038686	FBgn0038686	CG5555	FBtr0083746
FBgn0038690	FBgn0038690	NA	FBtr0083744
FBgn0038691	FBgn0038691	NA	FBtr0083743
FBgn0038692	FBgn0038692	NA	FBtr0083737
FBgn0038693	FBgn0038693	NA	FBtr0301693
FBgn0038694	FBgn0038694	NA	FBtr0083741
FBgn0038700	FBgn0038700	CG3734	FBtr0083755
FBgn0038702	FBgn0038702	NA	FBtr0083757
FBgn0038704	FBgn0038704	NA	FBtr0083759
FBgn0038705	FBgn0038705	NA	FBtr0113247
FBgn0038715	FBgn0038715	NA	FBtr0114553
FBgn0038716	FBgn0038716	NA	FBtr0304745
FBgn0038717	FBgn0038717	NA	FBtr0273217
FBgn0038719	FBgn0038719	NA	FBtr0083780
FBgn0038720	FBgn0038720	NA	FBtr0083810
FBgn0038721	FBgn0038721	NA	FBtr0302492
FBgn0038722	FBgn0038722	Nup58	FBtr0083781
FBgn0038723	FBgn0038723	CG6195	FBtr0083803
FBgn0038725	FBgn0038725	NA	FBtr0083802

FBgn0038727	FBgn0038727	NA	FBtr0301213
FBgn0038730	FBgn0038730	NA	FBtr0083798
FBgn0038731	FBgn0038731	NA	FBtr0083797
FBgn0038732	FBgn0038732	NA	FBtr0083789
FBgn0038735	FBgn0038735	NA	FBtr0083814
FBgn0038736	FBgn0038736	NA	FBtr0113249
FBgn0038737	FBgn0038737	NA	FBtr0083815
FBgn0038738	FBgn0038738	CG4572	FBtr0083861
FBgn0038739	FBgn0038739	NA	FBtr0083816
FBgn0038740	FBgn0038740	NA	FBtr0113250
FBgn0038741	FBgn0038741	NA	FBtr0083858
FBgn0038744	FBgn0038744	NA	FBtr0308829
FBgn0038747	FBgn0038747	RhoGAP92B	FBtr0083821
FBgn0038749	FBgn0038749	CG4468	FBtr0083852
FBgn0038750	FBgn0038750	NA	FBtr0083851
FBgn0038751	FBgn0038751	NA	FBtr0083822
FBgn0038752	FBgn0038752	NA	FBtr0083850
FBgn0038753	FBgn0038753	NA	FBtr0083849
FBgn0038760	FBgn0038760	NA	FBtr0083825
FBgn0038761	FBgn0038761	NA	FBtr0307346
FBgn0038762	FBgn0038762	NA	FBtr0083828
FBgn0038763	FBgn0038763	NA	FBtr0083842
FBgn0038768	FBgn0038768	NA	FBtr0083831
FBgn0038769	FBgn0038769	NA	FBtr0083839
FBgn0038771	FBgn0038771	CG4390	FBtr0083836
FBgn0038772	FBgn0038772	CG4973	FBtr0083835
FBgn0038775	FBgn0038775	NA	FBtr0083874
FBgn0038784	FBgn0038784	NA	FBtr0083900
FBgn0038787	FBgn0038787	NA	FBtr0113252
FBgn0038788	FBgn0038788	Sirt2	FBtr0083882
FBgn0038789	FBgn0038789	NA	FBtr0113253
FBgn0038790	FBgn0038790	NA	FBtr0083884
FBgn0038795	FBgn0038795	NA	FBtr0083894
FBgn0038796	FBgn0038796	NA	FBtr0083885
FBgn0038806	FBgn0038806	NA	FBtr0083909
FBgn0038808	FBgn0038808	NA	FBtr0300802
FBgn0038809	FBgn0038809	CG16953	FBtr0300803
FBgn0038810	FBgn0038810	NA	FBtr0083911
FBgn0038811	FBgn0038811	NA	FBtr0302323
FBgn0038814	FBgn0038814	NA	FBtr0113254
FBgn0038816	FBgn0038816	NA	FBtr0112788
FBgn0038820	FBgn0038820	NA	FBtr0083935
FBgn0038821	FBgn0038821	NA	FBtr0083931
FBgn0038826	FBgn0038826	CG17838	FBtr0083959
FBgn0038828	FBgn0038828	NA	FBtr0083994
FBgn0038829	FBgn0038829	CG17271	FBtr0083993
FBgn0038830	FBgn0038830	CG17272	FBtr0083992
FBgn0038839	FBgn0038839	CG10830	FBtr0083986
FBgn0038840	FBgn0038840	NA	FBtr0083978
FBgn0038842	FBgn0038842	NA	FBtr0113257
FBgn0038845	FBgn0038845	NA	FBtr0084048
FBgn0038846	FBgn0038846	NA	FBtr0084001
FBgn0038847	FBgn0038847	NA	FBtr0084003
FBgn0038849	FBgn0038849	NA	FBtr0084043
FBgn0038850	FBgn0038850	NA	FBtr0300816
FBgn0038852	FBgn0038852	NA	FBtr0084037
FBgn0038857	FBgn0038857	NA	FBtr0084033

FBgn0038858	FBgn0038858	NA	FBtr0304806
FBgn0038860	FBgn0038860	NA	FBtr0084015
FBgn0038861	FBgn0038861	NA	FBtr0084029
FBgn0038862	FBgn0038862	NA	FBtr0084016
FBgn0038865	FBgn0038865	NA	FBtr0084028
FBgn0038868	FBgn0038868	NA	FBtr0084049
FBgn0038870	FBgn0038870	NA	FBtr0084050
FBgn0038871	FBgn0038871	NA	FBtr0084107
FBgn0038876	FBgn0038876	CG5919	FBtr0084055
FBgn0038877	FBgn0038877	NA	FBtr0084102
FBgn0038879	FBgn0038879	NA	FBtr0084056
FBgn0038880	FBgn0038880	NA	FBtr0301815
FBgn0038881	FBgn0038881	NA	FBtr0084095
FBgn0038886	FBgn0038886	NA	FBtr0306680
FBgn0038887	FBgn0038887	NA	FBtr0113261
FBgn0038889	FBgn0038889	NA	FBtr0084109
FBgn0038890	FBgn0038890	NA	FBtr0100124
FBgn0038897	FBgn0038897	CG5849	FBtr0301164
FBgn0038908	FBgn0038908	NA	FBtr0084135
FBgn0038909	FBgn0038909	CG6569	FBtr0084155
FBgn0038912	FBgn0038912	NA	FBtr0084139
FBgn0038914	FBgn0038914	NA	FBtr0084153
FBgn0038915	FBgn0038915	NA	FBtr0084152
FBgn0038916	FBgn0038916	CG6560	FBtr0084151
FBgn0038918	FBgn0038918	NA	FBtr0084149
FBgn0038919	FBgn0038919	NA	FBtr0084150
FBgn0038921	FBgn0038921	NA	FBtr0084170
FBgn0038922	FBgn0038922	CG6439	FBtr0084190
FBgn0038923	FBgn0038923	NA	FBtr0084171
FBgn0038924	FBgn0038924	CG6028	FBtr0084186
FBgn0038925	FBgn0038925	CG6022	FBtr0084185
FBgn0038926	FBgn0038926	NA	FBtr0113264
FBgn0038927	FBgn0038927	NA	FBtr0084183
FBgn0038929	FBgn0038929	NA	FBtr0084179
FBgn0038930	FBgn0038930	NA	FBtr0084182
FBgn0038934	FBgn0038934	NA	FBtr0310204
FBgn0038941	FBgn0038941	CG7080	FBtr0084203
FBgn0038942	FBgn0038942	NA	FBtr0084204
FBgn0038943	FBgn0038943	NA	FBtr0084242
FBgn0038944	FBgn0038944	NA	FBtr0084241
FBgn0038946	FBgn0038946	NA	FBtr0310473
FBgn0038947	FBgn0038947	sar1	FBtr0084207
FBgn0038948	FBgn0038948	NA	FBtr0084239
FBgn0038949	FBgn0038949	NA	FBtr0084211
FBgn0038950	FBgn0038950	CG5382	FBtr0084237
FBgn0038951	FBgn0038951	NA	FBtr0305320
FBgn0038956	FBgn0038956	NA	FBtr0084235
FBgn0038958	FBgn0038958	NA	FBtr0084234
FBgn0038959	FBgn0038959	NA	FBtr0084224
FBgn0038961	FBgn0038961	CG13850	FBtr0084231
FBgn0038967	FBgn0038967	CG13847	FBtr0301711
FBgn0038972	FBgn0038972	CG7054	FBtr0084254
FBgn0038973	FBgn0038973	CG18594	FBtr0084255
FBgn0038974	FBgn0038974	NA	FBtr0084293
FBgn0038975	FBgn0038975	NA	FBtr0301485
FBgn0038976	FBgn0038976	CG7048	FBtr0301486
FBgn0038979	FBgn0038979	NA	FBtr0084259

FBgn0038980	FBgn0038980	NA	FBtr0100322
FBgn0038983	FBgn0038983	NA	FBtr0084285
FBgn0038984	FBgn0038984	CG5315	FBtr0084282
FBgn0038989	FBgn0038989	CG6937	FBtr0084266
FBgn0038993	FBgn0038993	NA	FBtr0084269
FBgn0038996	FBgn0038996	NA	FBtr0084272
FBgn0039000	FBgn0039000	NA	FBtr0084300
FBgn0039002	FBgn0039002	NA	FBtr0084301
FBgn0039003	FBgn0039003	wfs1	FBtr0084335
FBgn0039004	FBgn0039004	NA	FBtr0084302
FBgn0039005	FBgn0039005	NA	FBtr0084303
FBgn0039006	FBgn0039006	NA	FBtr0084304
FBgn0039007	FBgn0039007	NA	FBtr0084333
FBgn0039010	FBgn0039010	NA	FBtr0084330
FBgn0039013	FBgn0039013	CG4813	FBtr0084326
FBgn0039014	FBgn0039014	NA	FBtr0113268
FBgn0039015	FBgn0039015	NA	FBtr0302204
FBgn0039019	FBgn0039019	HP1c	FBtr0084309
FBgn0039020	FBgn0039020	NA	FBtr0084322
FBgn0039022	FBgn0039022	NA	FBtr0084319
FBgn0039023	FBgn0039023	CG4723	FBtr0084318
FBgn0039024	FBgn0039024	CG4721	FBtr0084317
FBgn0039025	FBgn0039025	CG7023	FBtr0084311
FBgn0039026	FBgn0039026	NA	FBtr0084312
FBgn0039028	FBgn0039028	NA	FBtr0301686
FBgn0039030	FBgn0039030	NA	FBtr0084338
FBgn0039031	FBgn0039031	NA	FBtr0084340
FBgn0039038	FBgn0039038	NA	FBtr0084343
FBgn0039039	FBgn0039039	lmd	FBtr0084364
FBgn0039040	FBgn0039040	NA	FBtr0084362
FBgn0039043	FBgn0039043	CG17121	FBtr0084361
FBgn0039044	FBgn0039044	p53	FBtr0301765
FBgn0039045	FBgn0039045	CG17119	FBtr0084358
FBgn0039048	FBgn0039048	NA	FBtr0303508
FBgn0039049	FBgn0039049	CG6726	FBtr0084349
FBgn0039050	FBgn0039050	CG17110	FBtr0084350
FBgn0039051	FBgn0039051	NA	FBtr0084351
FBgn0039052	FBgn0039052	NA	FBtr0084352
FBgn0039053	FBgn0039053	CG6738	FBtr0084353
FBgn0039054	FBgn0039054	NA	FBtr0302023
FBgn0039055	FBgn0039055	NA	FBtr0084407
FBgn0039056	FBgn0039056	cenB1A	FBtr0084368
FBgn0039058	FBgn0039058	NA	FBtr0084401
FBgn0039059	FBgn0039059	NA	FBtr0084400
FBgn0039060	FBgn0039060	NA	FBtr0084370
FBgn0039061	FBgn0039061	NA	FBtr0303041
FBgn0039064	FBgn0039064	NA	FBtr0110801
FBgn0039065	FBgn0039065	NA	FBtr0084388
FBgn0039066	FBgn0039066	NA	FBtr0084374
FBgn0039067	FBgn0039067	NA	FBtr0084387
FBgn0039068	FBgn0039068	CG13827	FBtr0084376
FBgn0039069	FBgn0039069	CG6763	FBtr0084378
FBgn0039070	FBgn0039070	CG17083	FBtr0084383
FBgn0039071	FBgn0039071	NA	FBtr0084382
FBgn0039075	FBgn0039075	NA	FBtr0301085
FBgn0039077	FBgn0039077	NA	FBtr0299867
FBgn0039078	FBgn0039078	NA	FBtr0310575

FBgn0039079	FBgn0039079	NA	FBtr0273202
FBgn0039080	FBgn0039080	NA	FBtr0273205
FBgn0039081	FBgn0039081	Irk2	FBtr0084425
FBgn0039083	FBgn0039083	NA	FBtr0084424
FBgn0039085	FBgn0039085	NA	FBtr0084420
FBgn0039086	FBgn0039086	NA	FBtr0084419
FBgn0039088	FBgn0039088	NA	FBtr0300585
FBgn0039089	FBgn0039089	NA	FBtr0084433
FBgn0039092	FBgn0039092	NA	FBtr0304611
FBgn0039093	FBgn0039093	NA	FBtr0273191
FBgn0039094	FBgn0039094	NA	FBtr0084481
FBgn0039099	FBgn0039099	NA	FBtr0084435
FBgn0039101	FBgn0039101	NA	FBtr0303150
FBgn0039102	FBgn0039102	NA	FBtr0084440
FBgn0039106	FBgn0039106	NA	FBtr0114504
FBgn0039107	FBgn0039107	CG10300	FBtr0084445
FBgn0039108	FBgn0039108	NA	FBtr0306706
FBgn0039110	FBgn0039110	CG10225	FBtr0084468
FBgn0039116	FBgn0039116	NA	FBtr0084458
FBgn0039118	FBgn0039118	CG10208	FBtr0084461
FBgn0039120	FBgn0039120	NA	FBtr0084460
FBgn0039124	FBgn0039124	NA	FBtr0084525
FBgn0039125	FBgn0039125	CG5857	FBtr0084491
FBgn0039126	FBgn0039126	CG13601	FBtr0084522
FBgn0039127	FBgn0039127	NA	FBtr0084521
FBgn0039128	FBgn0039128	CG13599	FBtr0084493
FBgn0039129	FBgn0039129	NA	FBtr0084519
FBgn0039130	FBgn0039130	CG5854	FBtr0084495
FBgn0039131	FBgn0039131	CG12268	FBtr0308071
FBgn0039132	FBgn0039132	NA	FBtr0305039
FBgn0039135	FBgn0039135	NA	FBtr0084510
FBgn0039136	FBgn0039136	NA	FBtr0084502
FBgn0039137	FBgn0039137	CG13604	FBtr0084509
FBgn0039139	FBgn0039139	NA	FBtr0084532
FBgn0039140	FBgn0039140	Miro	FBtr0321262
FBgn0039141	FBgn0039141	NA	FBtr0084533
FBgn0039147	FBgn0039147	NA	FBtr0084542
FBgn0039149	FBgn0039149	NA	FBtr0305314
FBgn0039150	FBgn0039150	NA	FBtr0300705
FBgn0039151	FBgn0039151	CG13607	FBtr0304156
FBgn0039152	FBgn0039152	NA	FBtr0304161
FBgn0039153	FBgn0039153	NA	FBtr0084584
FBgn0039154	FBgn0039154	NA	FBtr0084548
FBgn0039155	FBgn0039155	NA	FBtr0084549
FBgn0039156	FBgn0039156	CG6178	FBtr0084550
FBgn0039157	FBgn0039157	NA	FBtr0100516
FBgn0039161	FBgn0039161	CG13606	FBtr0084553
FBgn0039164	FBgn0039164	NA	FBtr0084573
FBgn0039165	FBgn0039165	CG6204	FBtr0084555
FBgn0039169	FBgn0039169	NA	FBtr0084565
FBgn0039170	FBgn0039170	NA	FBtr0084560
FBgn0039172	FBgn0039172	NA	FBtr0305044
FBgn0039175	FBgn0039175	CG5706	FBtr0084637
FBgn0039179	FBgn0039179	NA	FBtr0309096
FBgn0039180	FBgn0039180	NA	FBtr0084634
FBgn0039182	FBgn0039182	NA	FBtr0084632
FBgn0039186	FBgn0039186	NA	FBtr0273282

FBgn0039187	FBgn0039187	NA	FBtr0273323
FBgn0039188	FBgn0039188	Golgin84	FBtr0084629
FBgn0039189	FBgn0039189	CG18528	FBtr0084628
FBgn0039190	FBgn0039190	NA	FBtr0084627
FBgn0039193	FBgn0039193	NA	FBtr0303582
FBgn0039194	FBgn0039194	NA	FBtr0084614
FBgn0039195	FBgn0039195	NA	FBtr0084624
FBgn0039196	FBgn0039196	NA	FBtr0113279
FBgn0039197	FBgn0039197	CG17780	FBtr0113280
FBgn0039198	FBgn0039198	NA	FBtr0084620
FBgn0039199	FBgn0039199	NA	FBtr0084617
FBgn0039200	FBgn0039200	CG13616	FBtr0084618
FBgn0039202	FBgn0039202	NA	FBtr0289971
FBgn0039204	FBgn0039204	CG6607	FBtr0084644
FBgn0039205	FBgn0039205	NA	FBtr0084679
FBgn0039206	FBgn0039206	NA	FBtr0084645
FBgn0039207	FBgn0039207	NA	FBtr0089906
FBgn0039208	FBgn0039208	NA	FBtr0084647
FBgn0039209	FBgn0039209	CG13624	FBtr0084675
FBgn0039210	FBgn0039210	CG13625	FBtr0084671
FBgn0039212	FBgn0039212	Syx18	FBtr0084670
FBgn0039214	FBgn0039214	NA	FBtr0084668
FBgn0039223	FBgn0039223	CG5805	FBtr0084743
FBgn0039224	FBgn0039224	NA	FBtr0084742
FBgn0039232	FBgn0039232	CG13636	FBtr0084702
FBgn0039233	FBgn0039233	CG7006	FBtr0084704
FBgn0039234	FBgn0039234	nct	FBtr0301865
FBgn0039235	FBgn0039235	NA	FBtr0308616
FBgn0039236	FBgn0039236	NA	FBtr0306105
FBgn0039237	FBgn0039237	CG13640	FBtr0084730
FBgn0039238	FBgn0039238	NA	FBtr0084707
FBgn0039239	FBgn0039239	NA	FBtr0084729
FBgn0039241	FBgn0039241	NA	FBtr0303478
FBgn0039244	FBgn0039244	CG11069	FBtr0084712
FBgn0039246	FBgn0039246	NA	FBtr0084713
FBgn0039249	FBgn0039249	NA	FBtr0306089
FBgn0039250	FBgn0039250	CG11120	FBtr0084716
FBgn0039251	FBgn0039251	CG17462	FBtr0084718
FBgn0039254	FBgn0039254	NA	FBtr0084749
FBgn0039257	FBgn0039257	tnc	FBtr0305667
FBgn0039261	FBgn0039261	CG6422	FBtr0084795
FBgn0039264	FBgn0039264	CG11786	FBtr0113282
FBgn0039266	FBgn0039266	CG11791	FBtr0084763
FBgn0039269	FBgn0039269	veli	FBtr0301664
FBgn0039270	FBgn0039270	NA	FBtr0084765
FBgn0039271	FBgn0039271	NA	FBtr0304718
FBgn0039272	FBgn0039272	CG11836	FBtr0084767
FBgn0039273	FBgn0039273	NA	FBtr0113285
FBgn0039274	FBgn0039274	NA	FBtr0084769
FBgn0039277	FBgn0039277	NA	FBtr0113286
FBgn0039280	FBgn0039280	NA	FBtr0084782
FBgn0039282	FBgn0039282	NA	FBtr0302172
FBgn0039283	FBgn0039283	NA	FBtr0084780
FBgn0039286	FBgn0039286	dan	FBtr0084803
FBgn0039290	FBgn0039290	NA	FBtr0084805
FBgn0039291	FBgn0039291	NA	FBtr0084873
FBgn0039293	FBgn0039293	CG11851	FBtr0084808

FBgn0039294	FBgn0039294	NA	FBtr0301090
FBgn0039296	FBgn0039296	NA	FBtr0084870
FBgn0039299	FBgn0039299	NA	FBtr0084811
FBgn0039300	FBgn0039300	NA	FBtr0084868
FBgn0039301	FBgn0039301	CG11875	FBtr0084812
FBgn0039302	FBgn0039302	NA	FBtr0304692
FBgn0039303	FBgn0039303	CG11857	FBtr0084814
FBgn0039304	FBgn0039304	CG10425	FBtr0084867
FBgn0039305	FBgn0039305	CG11858	FBtr0084815
FBgn0039306	FBgn0039306	NA	FBtr0084816
FBgn0039307	FBgn0039307	NA	FBtr0084817
FBgn0039309	FBgn0039309	NA	FBtr0300131
FBgn0039310	FBgn0039310	NA	FBtr0084821
FBgn0039311	FBgn0039311	NA	FBtr0114505
FBgn0039312	FBgn0039312	NA	FBtr0084864
FBgn0039315	FBgn0039315	NA	FBtr0273361
FBgn0039316	FBgn0039316	NA	FBtr0084827
FBgn0039319	FBgn0039319	NA	FBtr0084828
FBgn0039321	FBgn0039321	NA	FBtr0084858
FBgn0039323	FBgn0039323	NA	FBtr0289942
FBgn0039324	FBgn0039324	NA	FBtr0084853
FBgn0039325	FBgn0039325	NA	FBtr0084852
FBgn0039326	FBgn0039326	NA	FBtr0084851
FBgn0039328	FBgn0039328	NA	FBtr0084849
FBgn0039329	FBgn0039329	NA	FBtr0300687
FBgn0039330	FBgn0039330	NA	FBtr0084847
FBgn0039331	FBgn0039331	NA	FBtr0273265
FBgn0039335	FBgn0039335	NA	FBtr0084835
FBgn0039336	FBgn0039336	NA	FBtr0084839
FBgn0039337	FBgn0039337	NA	FBtr0084836
FBgn0039338	FBgn0039338	XNP	FBtr0084838
FBgn0039339	FBgn0039339	NA	FBtr0084877
FBgn0039342	FBgn0039342	NA	FBtr0084879
FBgn0039343	FBgn0039343	NA	FBtr0084880
FBgn0039345	FBgn0039345	NA	FBtr0084894
FBgn0039347	FBgn0039347	NA	FBtr0084884
FBgn0039348	FBgn0039348	NA	FBtr0084893
FBgn0039349	FBgn0039349	NA	FBtr0084891
FBgn0039350	FBgn0039350	NA	FBtr0084887
FBgn0039355	FBgn0039355	NA	FBtr0084934
FBgn0039356	FBgn0039356	NA	FBtr0084900
FBgn0039357	FBgn0039357	NA	FBtr0301166
FBgn0039358	FBgn0039358	NA	FBtr0113289
FBgn0039366	FBgn0039366	NA	FBtr0084924
FBgn0039367	FBgn0039367	NA	FBtr0290204
FBgn0039368	FBgn0039368	NA	FBtr0084922
FBgn0039371	FBgn0039371	NA	FBtr0084919
FBgn0039373	FBgn0039373	NA	FBtr0084902
FBgn0039374	FBgn0039374	NA	FBtr0084903
FBgn0039376	FBgn0039376	NA	FBtr0084905
FBgn0039378	FBgn0039378	NA	FBtr0300129
FBgn0039380	FBgn0039380	NA	FBtr0301147
FBgn0039381	FBgn0039381	CG17370	FBtr0300819
FBgn0039385	FBgn0039385	CG5913	FBtr0084938
FBgn0039386	FBgn0039386	NA	FBtr0084939
FBgn0039387	FBgn0039387	NA	FBtr0084940
FBgn0039395	FBgn0039395	NA	FBtr0084984

FBgn0039396	FBgn0039396	NA	FBtr0304043
FBgn0039402	FBgn0039402	NA	FBtr0084967
FBgn0039403	FBgn0039403	NA	FBtr0084975
FBgn0039404	FBgn0039404	CG14543	FBtr0084968
FBgn0039405	FBgn0039405	NA	FBtr0084974
FBgn0039406	FBgn0039406	NA	FBtr0084973
FBgn0039407	FBgn0039407	NA	FBtr0084969
FBgn0039408	FBgn0039408	NA	FBtr0084971
FBgn0039411	FBgn0039411	dys	FBtr0113294
FBgn0039413	FBgn0039413	NA	FBtr0273389
FBgn0039415	FBgn0039415	NA	FBtr0084987
FBgn0039417	FBgn0039417	CG6073	FBtr0085003
FBgn0039419	FBgn0039419	NA	FBtr0084990
FBgn0039420	FBgn0039420	NA	FBtr0300399
FBgn0039421	FBgn0039421	NA	FBtr0085027
FBgn0039424	FBgn0039424	NA	FBtr0113295
FBgn0039425	FBgn0039425	NA	FBtr0085006
FBgn0039427	FBgn0039427	CG5447	FBtr0085012
FBgn0039428	FBgn0039428	NA	FBtr0085013
FBgn0039431	FBgn0039431	NA	FBtr0305049
FBgn0039440	FBgn0039440	NA	FBtr0114538
FBgn0039452	FBgn0039452	NA	FBtr0100553
FBgn0039461	FBgn0039461	NA	FBtr0085064
FBgn0039462	FBgn0039462	NA	FBtr0085067
FBgn0039463	FBgn0039463	NA	FBtr0085068
FBgn0039464	FBgn0039464	NA	FBtr0085084
FBgn0039465	FBgn0039465	Tsp97E	FBtr0085082
FBgn0039466	FBgn0039466	NA	FBtr0301134
FBgn0039469	FBgn0039469	CG14254	FBtr0085075
FBgn0039470	FBgn0039470	NA	FBtr0085078
FBgn0039471	FBgn0039471	NA	FBtr0085077
FBgn0039472	FBgn0039472	NA	FBtr0085153
FBgn0039478	FBgn0039478	NA	FBtr0113296
FBgn0039479	FBgn0039479	NA	FBtr0299564
FBgn0039482	FBgn0039482	NA	FBtr0085103
FBgn0039483	FBgn0039483	NA	FBtr0085104
FBgn0039485	FBgn0039485	NA	FBtr0085105
FBgn0039486	FBgn0039486	NA	FBtr0085133
FBgn0039489	FBgn0039489	NA	FBtr0085108
FBgn0039490	FBgn0039490	NA	FBtr0085109
FBgn0039491	FBgn0039491	NA	FBtr0085130
FBgn0039492	FBgn0039492	CG6051	FBtr0305050
FBgn0039494	FBgn0039494	CG5896	FBtr0085111
FBgn0039495	FBgn0039495	NA	FBtr0085114
FBgn0039498	FBgn0039498	NA	FBtr0085121
FBgn0039504	FBgn0039504	NA	FBtr0085157
FBgn0039505	FBgn0039505	NA	FBtr0085158
FBgn0039507	FBgn0039507	NA	FBtr0305973
FBgn0039510	FBgn0039510	NA	FBtr0273192
FBgn0039520	FBgn0039520	NA	FBtr0085184
FBgn0039521	FBgn0039521	NA	FBtr0085213
FBgn0039523	FBgn0039523	NA	FBtr0321256
FBgn0039528	FBgn0039528	NA	FBtr0301390
FBgn0039529	FBgn0039529	NA	FBtr0085200
FBgn0039530	FBgn0039530	NA	FBtr0302590
FBgn0039531	FBgn0039531	NA	FBtr0085199
FBgn0039532	FBgn0039532	Mtl	FBtr0085192

FBgn0039536	FBgn0039536	NA	FBtr0273246
FBgn0039538	FBgn0039538	NA	FBtr0085216
FBgn0039543	FBgn0039543	CG12428	FBtr0085237
FBgn0039544	FBgn0039544	NA	FBtr0100446
FBgn0039552	FBgn0039552	NA	FBtr0300050
FBgn0039553	FBgn0039553	NA	FBtr0085246
FBgn0039554	FBgn0039554	NA	FBtr0085272
FBgn0039555	FBgn0039555	NA	FBtr0085247
FBgn0039557	FBgn0039557	CG12259	FBtr0085270
FBgn0039558	FBgn0039558	CG4980	FBtr0085269
FBgn0039560	FBgn0039560	CG5514	FBtr0301143
FBgn0039563	FBgn0039563	CG4951	FBtr0113298
FBgn0039564	FBgn0039564	NA	FBtr0085255
FBgn0039565	FBgn0039565	NA	FBtr0085264
FBgn0039566	FBgn0039566	NA	FBtr0085257
FBgn0039568	FBgn0039568	NA	FBtr0085260
FBgn0039576	FBgn0039576	NA	FBtr0085279
FBgn0039577	FBgn0039577	NA	FBtr0301672
FBgn0039580	FBgn0039580	Gfat2	FBtr0085297
FBgn0039581	FBgn0039581	NA	FBtr0300346
FBgn0039585	FBgn0039585	NA	FBtr0085290
FBgn0039588	FBgn0039588	NA	FBtr0085293
FBgn0039591	FBgn0039591	NA	FBtr0085305
FBgn0039592	FBgn0039592	NA	FBtr0100373
FBgn0039593	FBgn0039593	NA	FBtr0085307
FBgn0039594	FBgn0039594	CG9990	FBtr0085309
FBgn0039595	FBgn0039595	NA	FBtr0308641
FBgn0039597	FBgn0039597	NA	FBtr0085314
FBgn0039598	FBgn0039598	NA	FBtr0085313
FBgn0039600	FBgn0039600	NA	FBtr0113301
FBgn0039601	FBgn0039601	CG1523	FBtr0085355
FBgn0039609	FBgn0039609	NA	FBtr0085328
FBgn0039616	FBgn0039616	NA	FBtr0308601
FBgn0039621	FBgn0039621	NA	FBtr0085341
FBgn0039622	FBgn0039622	NA	FBtr0302515
FBgn0039623	FBgn0039623	NA	FBtr0085342
FBgn0039625	FBgn0039625	NA	FBtr0085419
FBgn0039626	FBgn0039626	CG1420	FBtr0085418
FBgn0039627	FBgn0039627	CG11837	FBtr0085360
FBgn0039628	FBgn0039628	NA	FBtr0085361
FBgn0039629	FBgn0039629	NA	FBtr0085362
FBgn0039630	FBgn0039630	NA	FBtr0085363
FBgn0039631	FBgn0039631	NA	FBtr0085364
FBgn0039633	FBgn0039633	NA	FBtr0085365
FBgn0039634	FBgn0039634	CG11874	FBtr0085416
FBgn0039635	FBgn0039635	NA	FBtr0085369
FBgn0039636	FBgn0039636	CG11877	FBtr0085370
FBgn0039638	FBgn0039638	CG11881	FBtr0085414
FBgn0039641	FBgn0039641	NA	FBtr0085412
FBgn0039642	FBgn0039642	NA	FBtr0085411
FBgn0039645	FBgn0039645	NA	FBtr0273260
FBgn0039647	FBgn0039647	CG14509	FBtr0113304
FBgn0039650	FBgn0039650	NA	FBtr0085407
FBgn0039654	FBgn0039654	NA	FBtr0085394
FBgn0039655	FBgn0039655	CG14507	FBtr0113305
FBgn0039656	FBgn0039656	NA	FBtr0306020
FBgn0039659	FBgn0039659	NA	FBtr0085439

FBgn0039663	FBgn0039663	CG2321	FBtr0085436
FBgn0039664	FBgn0039664	NA	FBtr0085425
FBgn0039669	FBgn0039669	NA	FBtr0085440
FBgn0039670	FBgn0039670	CG7567	FBtr0085472
FBgn0039671	FBgn0039671	NA	FBtr0085471
FBgn0039673	FBgn0039673	NA	FBtr0304600
FBgn0039674	FBgn0039674	CG1907	FBtr0085447
FBgn0039677	FBgn0039677	NA	FBtr0085449
FBgn0039678	FBgn0039678	Obp99a	FBtr0085450
FBgn0039680	FBgn0039680	CAP-D2	FBtr0085452
FBgn0039681	FBgn0039681	NA	FBtr0085464
FBgn0039682	FBgn0039682	Obp99c	FBtr0085463
FBgn0039683	FBgn0039683	NA	FBtr0085454
FBgn0039685	FBgn0039685	Obp99b	FBtr0085462
FBgn0039687	FBgn0039687	CG7593	FBtr0085461
FBgn0039688	FBgn0039688	NA	FBtr0085460
FBgn0039689	FBgn0039689	CG7598	FBtr0085473
FBgn0039690	FBgn0039690	CG1969	FBtr0085474
FBgn0039691	FBgn0039691	CG1972	FBtr0085476
FBgn0039694	FBgn0039694	NA	FBtr0085538
FBgn0039705	FBgn0039705	CG31033	FBtr0085528
FBgn0039707	FBgn0039707	NA	FBtr0301745
FBgn0039709	FBgn0039709	NA	FBtr0085488
FBgn0039710	FBgn0039710	CG18041	FBtr0085527
FBgn0039711	FBgn0039711	NA	FBtr0089927
FBgn0039712	FBgn0039712	CG15514	FBtr0085523
FBgn0039713	FBgn0039713	NA	FBtr0085489
FBgn0039714	FBgn0039714	CG7816	FBtr0089925
FBgn0039723	FBgn0039723	CG15522	FBtr0085503
FBgn0039726	FBgn0039726	eIF2B-alpha	FBtr0085508
FBgn0039727	FBgn0039727	NA	FBtr0085505
FBgn0039732	FBgn0039732	NA	FBtr0085607
FBgn0039733	FBgn0039733	CG11504	FBtr0085547
FBgn0039734	FBgn0039734	NA	FBtr0085551
FBgn0039735	FBgn0039735	CG7911	FBtr0085606
FBgn0039736	FBgn0039736	NA	FBtr0085605
FBgn0039737	FBgn0039737	CG7920	FBtr0085603
FBgn0039741	FBgn0039741	CG7943	FBtr0085559
FBgn0039742	FBgn0039742	NA	FBtr0303240
FBgn0039743	FBgn0039743	CG7946	FBtr0085561
FBgn0039744	FBgn0039744	NA	FBtr0085562
FBgn0039747	FBgn0039747	NA	FBtr0085581
FBgn0039748	FBgn0039748	CG15529	FBtr0085567
FBgn0039749	FBgn0039749	NA	FBtr0302562
FBgn0039751	FBgn0039751	NA	FBtr0085574
FBgn0039754	FBgn0039754	CG9747	FBtr0085573
FBgn0039755	FBgn0039755	NA	FBtr0085572
FBgn0039756	FBgn0039756	CG9743	FBtr0085571
FBgn0039760	FBgn0039760	NA	FBtr0085617
FBgn0039761	FBgn0039761	NA	FBtr0085618
FBgn0039764	FBgn0039764	NA	FBtr0085629
FBgn0039765	FBgn0039765	NA	FBtr0085625
FBgn0039766	FBgn0039766	NA	FBtr0085628
FBgn0039767	FBgn0039767	NA	FBtr0085627
FBgn0039769	FBgn0039769	NA	FBtr0085644
FBgn0039770	FBgn0039770	NA	FBtr0113310
FBgn0039773	FBgn0039773	NA	FBtr0085691

FBgn0039780	FBgn0039780	NA	FBtr0085688
FBgn0039782	FBgn0039782	NA	FBtr0085655
FBgn0039783	FBgn0039783	NA	FBtr0085685
FBgn0039784	FBgn0039784	NA	FBtr0085657
FBgn0039787	FBgn0039787	NA	FBtr0085664
FBgn0039788	FBgn0039788	NA	FBtr0085680
FBgn0039790	FBgn0039790	CG2246	FBtr0085677
FBgn0039796	FBgn0039796	NA	FBtr0085728
FBgn0039797	FBgn0039797	NA	FBtr0085699
FBgn0039798	FBgn0039798	NA	FBtr0300700
FBgn0039799	FBgn0039799	NA	FBtr0085700
FBgn0039800	FBgn0039800	NA	FBtr0085723
FBgn0039801	FBgn0039801	NA	FBtr0085722
FBgn0039802	FBgn0039802	NA	FBtr0085703
FBgn0039804	FBgn0039804	CG15544	FBtr0085708
FBgn0039807	FBgn0039807	NA	FBtr0085718
FBgn0039808	FBgn0039808	NA	FBtr0085717
FBgn0039809	FBgn0039809	NA	FBtr0085715
FBgn0039811	FBgn0039811	NA	FBtr0085751
FBgn0039817	FBgn0039817	NA	FBtr0290201
FBgn0039821	FBgn0039821	NA	FBtr0085737
FBgn0039827	FBgn0039827	NA	FBtr0113311
FBgn0039828	FBgn0039828	CG1542	FBtr0085757
FBgn0039830	FBgn0039830	NA	FBtr0085773
FBgn0039831	FBgn0039831	CG12054	FBtr0305118
FBgn0039832	FBgn0039832	NA	FBtr0085771
FBgn0039838	FBgn0039838	NA	FBtr0113313
FBgn0039840	FBgn0039840	NA	FBtr0085766
FBgn0039846	FBgn0039846	CG11337	FBtr0300881
FBgn0039848	FBgn0039848	NA	FBtr0085810
FBgn0039849	FBgn0039849	CG11334	FBtr0085808
FBgn0039850	FBgn0039850	NA	FBtr0085789
FBgn0039851	FBgn0039851	CG12063	FBtr0085790
FBgn0039852	FBgn0039852	CG1499	FBtr0300875
FBgn0039855	FBgn0039855	NA	FBtr0113315
FBgn0039859	FBgn0039859	NA	FBtr0085868
FBgn0039860	FBgn0039860	CG1792	FBtr0085819
FBgn0039867	FBgn0039867	CstF-50	FBtr0085865
FBgn0039868	FBgn0039868	NA	FBtr0085831
FBgn0039869	FBgn0039869	NA	FBtr0085836
FBgn0039870	FBgn0039870	CG1896	FBtr0085837
FBgn0039872	FBgn0039872	NA	FBtr0300915
FBgn0039873	FBgn0039873	CG2191	FBtr0085860
FBgn0039874	FBgn0039874	NA	FBtr0085858
FBgn0039875	FBgn0039875	sip3	FBtr0085842
FBgn0039876	FBgn0039876	CG2126	FBtr0085857
FBgn0039877	FBgn0039877	CG2118	FBtr0307551
FBgn0039881	FBgn0039881	NA	FBtr0085847
FBgn0039883	FBgn0039883	NA	FBtr0302181
FBgn0039886	FBgn0039886	CG2003	FBtr0085871
FBgn0039889	FBgn0039889	NA	FBtr0308248
FBgn0039890	FBgn0039890	NA	FBtr0089148
FBgn0039896	FBgn0039896	NA	FBtr0089115
FBgn0039897	FBgn0039897	NA	FBtr0308087
FBgn0039900	FBgn0039900	NA	FBtr0089139
FBgn0039902	FBgn0039902	NA	FBtr0089133
FBgn0039904	FBgn0039904	NA	FBtr0089127

FBgn0039907	FBgn0039907	NA	FBtr0089111
FBgn0039908	FBgn0039908	NA	FBtr0300344
FBgn0039909	FBgn0039909	NA	FBtr0089105
FBgn0039914	FBgn0039914	NA	FBtr0089103
FBgn0039915	FBgn0039915	NA	FBtr0089972
FBgn0039916	FBgn0039916	NA	FBtr0089102
FBgn0039920	FBgn0039920	NA	FBtr0310541
FBgn0039929	FBgn0039929	NA	FBtr0089221
FBgn0039930	FBgn0039930	NA	FBtr0089222
FBgn0039932	FBgn0039932	NA	FBtr0301336
FBgn0039936	FBgn0039936	NA	FBtr0300885
FBgn0039937	FBgn0039937	NA	FBtr0089251
FBgn0039941	FBgn0039941	NA	FBtr0300807
FBgn0039942	FBgn0039942	NA	FBtr0113727
FBgn0039943	FBgn0039943	NA	FBtr0113730
FBgn0039944	FBgn0039944	NA	FBtr0113723
FBgn0039945	FBgn0039945	NA	FBtr0113717
FBgn0039946	FBgn0039946	NA	FBtr0301978
FBgn0039958	FBgn0039958	NA	FBtr0300703
FBgn0039959	FBgn0039959	NA	FBtr0113748
FBgn0039966	FBgn0039966	NA	FBtr0111134
FBgn0039969	FBgn0039969	NA	FBtr0273211
FBgn0039970	FBgn0039970	NA	FBtr0086071
FBgn0039972	FBgn0039972	NA	FBtr0306236
FBgn0039977	FBgn0039977	NA	FBtr0299868
FBgn0039979	FBgn0039979	NA	FBtr0307119
FBgn0039987	FBgn0039987	NA	FBtr0302440
FBgn0039993	FBgn0039993	NA	FBtr0113761
FBgn0039994	FBgn0039994	NA	FBtr0111288
FBgn0040001	FBgn0040001	NA	FBtr0305959
FBgn0040009	FBgn0040009	NA	FBtr0302386
FBgn0040010	FBgn0040010	NA	FBtr0111255
FBgn0040011	FBgn0040011	NA	FBtr0111252
FBgn0040017	FBgn0040017	NA	FBtr0300103
FBgn0040022	FBgn0040022	NA	FBtr0113870
FBgn0040028	FBgn0040028	NA	FBtr0070057
FBgn0040030	FBgn0040030	NA	FBtr0113768
FBgn0040034	FBgn0040034	NA	FBtr0302250
FBgn0040037	FBgn0040037	NA	FBtr0299529
FBgn0040045	FBgn0040045	NA	FBtr0307117
FBgn0040063	FBgn0040063	NA	FBtr0072050
FBgn0040064	FBgn0040064	yip2	FBtr0079876
FBgn0040066	FBgn0040066	wds	FBtr0308566
FBgn0040068	FBgn0040068	vav	FBtr0074678
FBgn0040069	FBgn0040069	NA	FBtr0070879
FBgn0040070	FBgn0040070	NA	FBtr0079839
FBgn0040074	FBgn0040074	NA	FBtr0075375
FBgn0040076	FBgn0040076	NA	FBtr0091746
FBgn0040077	FBgn0040077	NA	FBtr0091747
FBgn0040078	FBgn0040078	pont	FBtr0082226
FBgn0040079	FBgn0040079	pkaap	FBtr0080826
FBgn0040080	FBgn0040080	NA	FBtr0085189
FBgn0040087	FBgn0040087	p115	FBtr0071089
FBgn0040089	FBgn0040089	NA	FBtr0301703
FBgn0040091	FBgn0040091	Ugt58Fa	FBtr0071934
FBgn0040092	FBgn0040092	lectin-46Cb	FBtr0088475
FBgn0040093	FBgn0040093	NA	FBtr0088476

FBgn0040096	FBgn0040096	NA	FBtr0113317
FBgn0040097	FBgn0040097	NA	FBtr0079816
FBgn0040098	FBgn0040098	NA	FBtr0079715
FBgn0040099	FBgn0040099	NA	FBtr0308065
FBgn0040104	FBgn0040104	NA	FBtr0077539
FBgn0040106	FBgn0040106	NA	FBtr0308305
FBgn0040107	FBgn0040107	NA	FBtr0077998
FBgn0040153	FBgn0040153	NA	FBtr0073927
FBgn0040206	FBgn0040206	krz	FBtr0085877
FBgn0040208	FBgn0040208	katanin-60	FBtr0078827
FBgn0040211	FBgn0040211	hgo	FBtr0080201
FBgn0040212	FBgn0040212	NA	FBtr0084897
FBgn0040227	FBgn0040227	eIF-3p66	FBtr0089622
FBgn0040228	FBgn0040228	NA	FBtr0080693
FBgn0040232	FBgn0040232	NA	FBtr0080215
FBgn0040237	FBgn0040237	bor	FBtr0083276
FBgn0040238	FBgn0040238	NA	FBtr0306023
FBgn0040239	FBgn0040239	NA	FBtr0077084
FBgn0040250	FBgn0040250	NA	FBtr0082373
FBgn0040251	FBgn0040251	NA	FBtr0082378
FBgn0040252	FBgn0040252	NA	FBtr0082340
FBgn0040253	FBgn0040253	NA	FBtr0308027
FBgn0040255	FBgn0040255	NA	FBtr0082376
FBgn0040256	FBgn0040256	NA	FBtr0113321
FBgn0040261	FBgn0040261	NA	FBtr0080910
FBgn0040262	FBgn0040262	NA	FBtr0080909
FBgn0040268	FBgn0040268	NA	FBtr0081246
FBgn0040271	FBgn0040271	Sulf1	FBtr0083273
FBgn0040273	FBgn0040273	Spt5	FBtr0086531
FBgn0040280	FBgn0040280	NA	FBtr0079005
FBgn0040281	FBgn0040281	NA	FBtr0072627
FBgn0040284	FBgn0040284	SF2	FBtr0304727
FBgn0040285	FBgn0040285	NA	FBtr0074079
FBgn0040286	FBgn0040286	SC35	FBtr0306252
FBgn0040291	FBgn0040291	NA	FBtr0072547
FBgn0040296	FBgn0040296	Ocho	FBtr0299779
FBgn0040297	FBgn0040297	Nhe2	FBtr0306016
FBgn0040299	FBgn0040299	NA	FBtr0079475
FBgn0040305	FBgn0040305	MTF-1	FBtr0113322
FBgn0040308	FBgn0040308	Jafrac2	FBtr0072983
FBgn0040318	FBgn0040318	HGTX	FBtr0075720
FBgn0040321	FBgn0040321	NA	FBtr0076510
FBgn0040333	FBgn0040333	NA	FBtr0300708
FBgn0040334	FBgn0040334	NA	FBtr0307159
FBgn0040336	FBgn0040336	NA	FBtr0070442
FBgn0040337	FBgn0040337	CG3021	FBtr0070173
FBgn0040338	FBgn0040338	NA	FBtr0070184
FBgn0040339	FBgn0040339	NA	FBtr0070165
FBgn0040340	FBgn0040340	NA	FBtr0070167
FBgn0040341	FBgn0040341	NA	FBtr0070163
FBgn0040342	FBgn0040342	NA	FBtr0070189
FBgn0040343	FBgn0040343	NA	FBtr0070195
FBgn0040344	FBgn0040344	NA	FBtr0070191
FBgn0040345	FBgn0040345	NA	FBtr0070190
FBgn0040346	FBgn0040346	NA	FBtr0070188
FBgn0040347	FBgn0040347	mus81	FBtr0070170
FBgn0040351	FBgn0040351	NA	FBtr0070183

FBgn0040353	FBgn0040353	NA	FBtr0070566
FBgn0040354	FBgn0040354	NA	FBtr0070565
FBgn0040355	FBgn0040355	NA	FBtr0070546
FBgn0040356	FBgn0040356	NA	FBtr0070489
FBgn0040361	FBgn0040361	NA	FBtr0310612
FBgn0040362	FBgn0040362	NA	FBtr0070198
FBgn0040363	FBgn0040363	NA	FBtr0070251
FBgn0040364	FBgn0040364	NA	FBtr0310611
FBgn0040365	FBgn0040365	NA	FBtr0070180
FBgn0040366	FBgn0040366	NA	FBtr0070250
FBgn0040368	FBgn0040368	NA	FBtr0070252
FBgn0040370	FBgn0040370	NA	FBtr0300326
FBgn0040375	FBgn0040375	NA	FBtr0300707
FBgn0040376	FBgn0040376	NA	FBtr0070437
FBgn0040377	FBgn0040377	NA	FBtr0070450
FBgn0040382	FBgn0040382	NA	FBtr0070137
FBgn0040383	FBgn0040383	CG5254	FBtr0070134
FBgn0040384	FBgn0040384	NA	FBtr0309881
FBgn0040387	FBgn0040387	NA	FBtr0070463
FBgn0040388	FBgn0040388	CG32796	FBtr0114528
FBgn0040389	FBgn0040389	mRpL14	FBtr0070405
FBgn0040390	FBgn0040390	NA	FBtr0301752
FBgn0040391	FBgn0040391	NA	FBtr0070399
FBgn0040392	FBgn0040392	NA	FBtr0308937
FBgn0040396	FBgn0040396	CG3939	FBtr0070508
FBgn0040397	FBgn0040397	NA	FBtr0070181
FBgn0040398	FBgn0040398	CG14629	FBtr0070179
FBgn0040465	FBgn0040465	Dip3	FBtr0086734
FBgn0040466	FBgn0040466	NA	FBtr0078893
FBgn0040475	FBgn0040475	SH3PX1	FBtr0076413
FBgn0040477	FBgn0040477	cid	FBtr0087667
FBgn0040493	FBgn0040493	NA	FBtr0290273
FBgn0040502	FBgn0040502	NA	FBtr0085988
FBgn0040503	FBgn0040503	NA	FBtr0088135
FBgn0040506	FBgn0040506	NA	FBtr0310556
FBgn0040507	FBgn0040507	ACXD	FBtr0072874
FBgn0040508	FBgn0040508	NA	FBtr0100494
FBgn0040509	FBgn0040509	NA	FBtr0080414
FBgn0040510	FBgn0040510	NA	FBtr0305492
FBgn0040512	FBgn0040512	NA	FBtr0075353
FBgn0040513	FBgn0040513	NA	FBtr0089002
FBgn0040514	FBgn0040514	NA	FBtr0302055
FBgn0040519	FBgn0040519	NA	FBtr0085944
FBgn0040524	FBgn0040524	CG11052	FBtr0081821
FBgn0040528	FBgn0040528	NA	FBtr0300372
FBgn0040529	FBgn0040529	NA	FBtr0081855
FBgn0040531	FBgn0040531	NA	FBtr0112769
FBgn0040532	FBgn0040532	NA	FBtr0305188
FBgn0040534	FBgn0040534	NA	FBtr0082006
FBgn0040551	FBgn0040551	CG11686	FBtr0082735
FBgn0040553	FBgn0040553	NA	FBtr0082761
FBgn0040554	FBgn0040554	NA	FBtr0309273
FBgn0040565	FBgn0040565	NA	FBtr0300628
FBgn0040571	FBgn0040571	NA	FBtr0083838
FBgn0040587	FBgn0040587	NA	FBtr0113328
FBgn0040588	FBgn0040588	NA	FBtr0084316
FBgn0040600	FBgn0040600	CG13631	FBtr0084693

FBgn0040601	FBgn0040601	CG13643	FBtr0306798
FBgn0040606	FBgn0040606	NA	FBtr0085025
FBgn0040607	FBgn0040607	NA	FBtr0303466
FBgn0040608	FBgn0040608	NA	FBtr0100441
FBgn0040623	FBgn0040623	NA	FBtr0085435
FBgn0040625	FBgn0040625	NA	FBtr0085588
FBgn0040628	FBgn0040628	NA	FBtr0305335
FBgn0040629	FBgn0040629	NA	FBtr0305336
FBgn0040634	FBgn0040634	NA	FBtr0078246
FBgn0040648	FBgn0040648	NA	FBtr0077240
FBgn0040649	FBgn0040649	NA	FBtr0077311
FBgn0040650	FBgn0040650	NA	FBtr0077323
FBgn0040651	FBgn0040651	NA	FBtr0077328
FBgn0040658	FBgn0040658	NA	FBtr0100554
FBgn0040666	FBgn0040666	NA	FBtr0310371
FBgn0040670	FBgn0040670	NA	FBtr0081562
FBgn0040682	FBgn0040682	NA	FBtr0305000
FBgn0040684	FBgn0040684	NA	FBtr0078843
FBgn0040687	FBgn0040687	NA	FBtr0078908
FBgn0040694	FBgn0040694	NA	FBtr0073140
FBgn0040697	FBgn0040697	NA	FBtr0073257
FBgn0040699	FBgn0040699	NA	FBtr0290214
FBgn0040705	FBgn0040705	NA	FBtr0077440
FBgn0040715	FBgn0040715	NA	FBtr0077842
FBgn0040717	FBgn0040717	NA	FBtr0077817
FBgn0040718	FBgn0040718	NA	FBtr0077863
FBgn0040723	FBgn0040723	NA	FBtr0077973
FBgn0040726	FBgn0040726	NA	FBtr0086238
FBgn0040730	FBgn0040730	NA	FBtr0086482
FBgn0040732	FBgn0040732	NA	FBtr0086499
FBgn0040736	FBgn0040736	NA	FBtr0086665
FBgn0040747	FBgn0040747	NA	FBtr0087406
FBgn0040751	FBgn0040751	NA	FBtr0087581
FBgn0040752	FBgn0040752	NA	FBtr0304956
FBgn0040759	FBgn0040759	NA	FBtr0112772
FBgn0040763	FBgn0040763	NA	FBtr0088175
FBgn0040765	FBgn0040765	NA	FBtr0088210
FBgn0040773	FBgn0040773	NA	FBtr0088396
FBgn0040777	FBgn0040777	NA	FBtr0088720
FBgn0040780	FBgn0040780	NA	FBtr0113332
FBgn0040784	FBgn0040784	NA	FBtr0301572
FBgn0040786	FBgn0040786	NA	FBtr0299563
FBgn0040793	FBgn0040793	CG7630	FBtr0075217
FBgn0040794	FBgn0040794	NA	FBtr0075376
FBgn0040795	FBgn0040795	NA	FBtr0075429
FBgn0040797	FBgn0040797	NA	FBtr0302928
FBgn0040798	FBgn0040798	NA	FBtr0075463
FBgn0040799	FBgn0040799	NA	FBtr0112771
FBgn0040801	FBgn0040801	NA	FBtr0075480
FBgn0040805	FBgn0040805	NA	FBtr0300757
FBgn0040817	FBgn0040817	NA	FBtr0113334
FBgn0040828	FBgn0040828	NA	FBtr0076592
FBgn0040832	FBgn0040832	NA	FBtr0307388
FBgn0040837	FBgn0040837	NA	FBtr0273402
FBgn0040842	FBgn0040842	CG15212	FBtr0077163
FBgn0040843	FBgn0040843	NA	FBtr0077182
FBgn0040849	FBgn0040849	NA	FBtr0299925

FBgn0040850	FBgn0040850	NA	FBtr0073377
FBgn0040859	FBgn0040859	NA	FBtr0073626
FBgn0040871	FBgn0040871	NA	FBtr0073869
FBgn0040877	FBgn0040877	NA	FBtr0074438
FBgn0040890	FBgn0040890	NA	FBtr0300214
FBgn0040892	FBgn0040892	NA	FBtr0310164
FBgn0040899	FBgn0040899	NA	FBtr0070390
FBgn0040900	FBgn0040900	NA	FBtr0290085
FBgn0040904	FBgn0040904	NA	FBtr0070660
FBgn0040905	FBgn0040905	NA	FBtr0070659
FBgn0040906	FBgn0040906	NA	FBtr0070657
FBgn0040907	FBgn0040907	NA	FBtr0070655
FBgn0040918	FBgn0040918	NA	FBtr0070938
FBgn0040922	FBgn0040922	NA	FBtr0071060
FBgn0040923	FBgn0040923	NA	FBtr0071058
FBgn0040941	FBgn0040941	NA	FBtr0300073
FBgn0040954	FBgn0040954	CG13779	FBtr0079403
FBgn0040958	FBgn0040958	NA	FBtr0079714
FBgn0040964	FBgn0040964	NA	FBtr0079763
FBgn0040965	FBgn0040965	NA	FBtr0079974
FBgn0040967	FBgn0040967	NA	FBtr0080160
FBgn0040968	FBgn0040968	NA	FBtr0080274
FBgn0040972	FBgn0040972	NA	FBtr0307212
FBgn0040973	FBgn0040973	NA	FBtr0113336
FBgn0040985	FBgn0040985	CG6115	FBtr0080959
FBgn0040987	FBgn0040987	NA	FBtr0304947
FBgn0040989	FBgn0040989	NA	FBtr0081026
FBgn0040992	FBgn0040992	NA	FBtr0081122
FBgn0040993	FBgn0040993	CG17325	FBtr0081102
FBgn0040995	FBgn0040995	NA	FBtr0081305
FBgn0040996	FBgn0040996	NA	FBtr0081351
FBgn0041004	FBgn0041004	NA	FBtr0111249
FBgn0041005	FBgn0041005	NA	FBtr0303019
FBgn0041087	FBgn0041087	NA	FBtr0088506
FBgn0041092	FBgn0041092	NA	FBtr0304974
FBgn0041094	FBgn0041094	NA	FBtr0076168
FBgn0041096	FBgn0041096	rols	FBtr0076053
FBgn0041097	FBgn0041097	NA	FBtr0077921
FBgn0041100	FBgn0041100	NA	FBtr0078318
FBgn0041103	FBgn0041103	NA	FBtr0300021
FBgn0041105	FBgn0041105	NA	FBtr0082177
FBgn0041111	FBgn0041111	lilli	FBtr0077633
FBgn0041150	FBgn0041150	hoe1	FBtr0077399
FBgn0041164	FBgn0041164	armi	FBtr0309841
FBgn0041188	FBgn0041188	NA	FBtr0083100
FBgn0041191	FBgn0041191	Rheb	FBtr0078693
FBgn0041195	FBgn0041195	NA	FBtr0080365
FBgn0041203	FBgn0041203	NA	FBtr0073660
FBgn0041205	FBgn0041205	NA	FBtr0072428
FBgn0041210	FBgn0041210	NA	FBtr0301298
FBgn0041232	FBgn0041232	NA	FBtr0300026
FBgn0041238	FBgn0041238	NA	FBtr0071732
FBgn0041242	FBgn0041242	NA	FBtr0303149
FBgn0041243	FBgn0041243	NA	FBtr0110820
FBgn0041248	FBgn0041248	NA	FBtr0077702
FBgn0041250	FBgn0041250	NA	FBtr0078006
FBgn0041252	FBgn0041252	NA	FBtr0070663

FBgn0041579	FBgn0041579	NA	FBtr0305795
FBgn0041581	FBgn0041581	NA	FBtr0300942
FBgn0041585	FBgn0041585	olf186-F	FBtr0114614
FBgn0041588	FBgn0041588	NA	FBtr0085410
FBgn0041605	FBgn0041605	NA	FBtr0301768
FBgn0041607	FBgn0041607	NA	FBtr0089463
FBgn0041630	FBgn0041630	Hexo1	FBtr0073237
FBgn0041706	FBgn0041706	NA	FBtr0305505
FBgn0041709	FBgn0041709	NA	FBtr0072902
FBgn0041710	FBgn0041710	NA	FBtr0082723
FBgn0041723	FBgn0041723	NA	FBtr0089855
FBgn0041780	FBgn0041780	NA	FBtr0085343
FBgn0041781	FBgn0041781	SCAR	FBtr0305255
FBgn0041789	FBgn0041789	Pax	FBtr0100449
FBgn0042083	FBgn0042083	CG3267	FBtr0086126
FBgn0042085	FBgn0042085	NA	FBtr0086107
FBgn0042086	FBgn0042086	NA	FBtr0086177
FBgn0042092	FBgn0042092	NA	FBtr0079372
FBgn0042094	FBgn0042094	NA	FBtr0082331
FBgn0042101	FBgn0042101	NA	FBtr0114516
FBgn0042102	FBgn0042102	NA	FBtr0081777
FBgn0042104	FBgn0042104	NA	FBtr0273255
FBgn0042105	FBgn0042105	NA	FBtr0113345
FBgn0042106	FBgn0042106	NA	FBtr0303151
FBgn0042110	FBgn0042110	NA	FBtr0113346
FBgn0042118	FBgn0042118	NA	FBtr0077022
FBgn0042125	FBgn0042125	NA	FBtr0321267
FBgn0042126	FBgn0042126	NA	FBtr0289986
FBgn0042127	FBgn0042127	NA	FBtr0080315
FBgn0042129	FBgn0042129	NA	FBtr0081307
FBgn0042133	FBgn0042133	NA	FBtr0081386
FBgn0042135	FBgn0042135	NA	FBtr0088935
FBgn0042137	FBgn0042137	NA	FBtr0307504
FBgn0042173	FBgn0042173	NA	FBtr0089426
FBgn0042174	FBgn0042174	CG18854	FBtr0302441
FBgn0042180	FBgn0042180	NA	FBtr0071688
FBgn0042182	FBgn0042182	NA	FBtr0100545
FBgn0042185	FBgn0042185	NA	FBtr0076972
FBgn0042186	FBgn0042186	NA	FBtr0077805
FBgn0042189	FBgn0042189	NA	FBtr0079380
FBgn0042201	FBgn0042201	NA	FBtr0075369
FBgn0042205	FBgn0042205	NA	FBtr0082417
FBgn0042206	FBgn0042206	NA	FBtr0082609
FBgn0042207	FBgn0042207	NA	FBtr0082644
FBgn0042213	FBgn0042213	NA	FBtr0085459
FBgn0042641	FBgn0042641	frc	FBtr0299820
FBgn0042650	FBgn0042650	NA	FBtr0310302
FBgn0042693	FBgn0042693	NA	FBtr0306646
FBgn0042696	FBgn0042696	NA	FBtr0089141
FBgn0042710	FBgn0042710	NA	FBtr0085011
FBgn0042711	FBgn0042711	NA	FBtr0085010
FBgn0042712	FBgn0042712	HBS1	FBtr0072866
FBgn0043005	FBgn0043005	NA	FBtr0113347
FBgn0043012	FBgn0043012	AP-2sigma	FBtr0084103
FBgn0043025	FBgn0043025	NA	FBtr0075192
FBgn0043043	FBgn0043043	NA	FBtr0082592
FBgn0043364	FBgn0043364	cbt	FBtr0078084

FBgn0043455	FBgn0043455	CG5986	FBtr0084535
FBgn0043456	FBgn0043456	CG4747	FBtr0303902
FBgn0043457	FBgn0043457	NA	FBtr0273268
FBgn0043470	FBgn0043470	NA	FBtr0088121
FBgn0043471	FBgn0043471	NA	FBtr0301671
FBgn0043530	FBgn0043530	NA	FBtr0087420
FBgn0043532	FBgn0043532	NA	FBtr0303458
FBgn0043533	FBgn0043533	NA	FBtr0086328
FBgn0043534	FBgn0043534	NA	FBtr0086293
FBgn0043535	FBgn0043535	NA	FBtr0086294
FBgn0043536	FBgn0043536	NA	FBtr0299804
FBgn0043539	FBgn0043539	NA	FBtr0100503
FBgn0043550	FBgn0043550	NA	FBtr0075751
FBgn0043577	FBgn0043577	NA	FBtr0075320
FBgn0043578	FBgn0043578	NA	FBtr0075348
FBgn0043783	FBgn0043783	NA	FBtr0078424
FBgn0043791	FBgn0043791	CG8147	FBtr0082060
FBgn0043792	FBgn0043792	CG30427	FBtr0300956
FBgn0043799	FBgn0043799	NA	FBtr0303481
FBgn0043806	FBgn0043806	NA	FBtr0076529
FBgn0043825	FBgn0043825	NA	FBtr0300805
FBgn0043841	FBgn0043841	NA	FBtr0080389
FBgn0043854	FBgn0043854	NA	FBtr0290281
FBgn0043865	FBgn0043865	NA	FBtr0301788
FBgn0043884	FBgn0043884	mask	FBtr0305043
FBgn0043900	FBgn0043900	NA	FBtr0085806
FBgn0044020	FBgn0044020	NA	FBtr0302211
FBgn0044030	FBgn0044030	NA	FBtr0074785
FBgn0044046	FBgn0044046	NA	FBtr0070577
FBgn0044047	FBgn0044047	NA	FBtr0070406
FBgn0044048	FBgn0044048	NA	FBtr0076371
FBgn0044050	FBgn0044050	NA	FBtr0076373
FBgn0044323	FBgn0044323	NA	FBtr0306001
FBgn0044328	FBgn0044328	NA	FBtr0273387
FBgn0044329	FBgn0044329	NA	FBtr0100361
FBgn0044419	FBgn0044419	NA	FBtr0299940
FBgn0044510	FBgn0044510	NA	FBtr0111147
FBgn0044817	FBgn0044817	NA	FBtr0307297
FBgn0044823	FBgn0044823	Spec2	FBtr0078739
FBgn0044826	FBgn0044826	NA	FBtr0305003
FBgn0044871	FBgn0044871	NA	FBtr0083661
FBgn0044872	FBgn0044872	NA	FBtr0299926
FBgn0045035	FBgn0045035	NA	FBtr0110874
FBgn0045073	FBgn0045073	Stim	FBtr0308705
FBgn0045443	FBgn0045443	NA	FBtr0082247
FBgn0045470	FBgn0045470	NA	FBtr0290236
FBgn0045474	FBgn0045474	NA	FBtr0078281
FBgn0045476	FBgn0045476	NA	FBtr0073199
FBgn0045477	FBgn0045477	NA	FBtr0309790
FBgn0045478	FBgn0045478	NA	FBtr0073195
FBgn0045479	FBgn0045479	NA	FBtr0073194
FBgn0045495	FBgn0045495	NA	FBtr0079460
FBgn0045498	FBgn0045498	NA	FBtr0310114
FBgn0045761	FBgn0045761	NA	FBtr0113352
FBgn0045770	FBgn0045770	NA	FBtr0076286
FBgn0045823	FBgn0045823	vsg	FBtr0076400
FBgn0045827	FBgn0045827	NA	FBtr0080802

FBgn0045842	FBgn0045842	yuri	FBtr0080709
FBgn0045843	FBgn0045843	NA	FBtr0081580
FBgn0045852	FBgn0045852	NA	FBtr0305621
FBgn0046113	FBgn0046113	NA	FBtr0273244
FBgn0046114	FBgn0046114	Gclm	FBtr0084336
FBgn0046212	FBgn0046212	NA	FBtr0100526
FBgn0046214	FBgn0046214	NA	FBtr0084776
FBgn0046225	FBgn0046225	NA	FBtr0082392
FBgn0046247	FBgn0046247	NA	FBtr0085160
FBgn0046253	FBgn0046253	NA	FBtr0273227
FBgn0046294	FBgn0046294	NA	FBtr0300762
FBgn0046296	FBgn0046296	CG11534	FBtr0075995
FBgn0046297	FBgn0046297	NA	FBtr0308943
FBgn0046301	FBgn0046301	CG7148	FBtr0078448
FBgn0046302	FBgn0046302	CG10650	FBtr0081183
FBgn0046322	FBgn0046322	NA	FBtr0084582
FBgn0046323	FBgn0046323	NA	FBtr0302286
FBgn0046332	FBgn0046332	NA	FBtr0085784
FBgn0046687	FBgn0046687	Tre1	FBtr0070848
FBgn0046689	FBgn0046689	NA	FBtr0083996
FBgn0046692	FBgn0046692	NA	FBtr0111111
FBgn0046698	FBgn0046698	NA	FBtr0310442
FBgn0046706	FBgn0046706	NA	FBtr0113784
FBgn0046756	FBgn0046756	NA	FBtr0091956
FBgn0046776	FBgn0046776	NA	FBtr0303308
FBgn0046793	FBgn0046793	CG32236	FBtr0073369
FBgn0046874	FBgn0046874	NA	FBtr0091716
FBgn0046876	FBgn0046876	NA	FBtr0078618
FBgn0046879	FBgn0046879	NA	FBtr0086479
FBgn0047092	FBgn0047092	NA	FBtr0071016
FBgn0047114	FBgn0047114	CG31142	FBtr0084492
FBgn0047135	FBgn0047135	NA	FBtr0073106
FBgn0047199	FBgn0047199	NA	FBtr0082864
FBgn0047205	FBgn0047205	NA	FBtr0076899
FBgn0047311	FBgn0047311	NA	FBtr0300249
FBgn0047334	FBgn0047334	NA	FBtr0091954
FBgn0050000	FBgn0050000	NA	FBtr0088463
FBgn0050001	FBgn0050001	NA	FBtr0088492
FBgn0050005	FBgn0050005	NA	FBtr0088464
FBgn0050007	FBgn0050007	NA	FBtr0300921
FBgn0050008	FBgn0050008	NA	FBtr0088380
FBgn0050009	FBgn0050009	NA	FBtr0088361
FBgn0050010	FBgn0050010	NA	FBtr0088374
FBgn0050011	FBgn0050011	gem	FBtr0300943
FBgn0050015	FBgn0050015	NA	FBtr0113356
FBgn0050016	FBgn0050016	NA	FBtr0088241
FBgn0050018	FBgn0050018	NA	FBtr0304706
FBgn0050021	FBgn0050021	NA	FBtr0088196
FBgn0050022	FBgn0050022	NA	FBtr0289990
FBgn0050026	FBgn0050026	NA	FBtr0300922
FBgn0050029	FBgn0050029	NA	FBtr0088109
FBgn0050031	FBgn0050031	NA	FBtr0088123
FBgn0050033	FBgn0050033	NA	FBtr0088117
FBgn0050035	FBgn0050035	CG30035	FBtr0088073
FBgn0050036	FBgn0050036	NA	FBtr0088050
FBgn0050037	FBgn0050037	NA	FBtr0088049
FBgn0050042	FBgn0050042	NA	FBtr0087914

FBgn0050044	FBgn0050044	NA	FBtr0087938
FBgn0050045	FBgn0050045	NA	FBtr0113359
FBgn0050047	FBgn0050047	NA	FBtr0087905
FBgn0050049	FBgn0050049	NA	FBtr0087906
FBgn0050050	FBgn0050050	NA	FBtr0289993
FBgn0050051	FBgn0050051	NA	FBtr0114521
FBgn0050052	FBgn0050052	NA	FBtr0087872
FBgn0050053	FBgn0050053	NA	FBtr0087873
FBgn0050054	FBgn0050054	NA	FBtr0302389
FBgn0050055	FBgn0050055	NA	FBtr0303919
FBgn0050056	FBgn0050056	NA	FBtr0087823
FBgn0050058	FBgn0050058	NA	FBtr0087767
FBgn0050060	FBgn0050060	NA	FBtr0087714
FBgn0050062	FBgn0050062	NA	FBtr0303158
FBgn0050065	FBgn0050065	NA	FBtr0087663
FBgn0050067	FBgn0050067	NA	FBtr0087558
FBgn0050069	FBgn0050069	NA	FBtr0087530
FBgn0050072	FBgn0050072	NA	FBtr0303244
FBgn0050073	FBgn0050073	NA	FBtr0303243
FBgn0050075	FBgn0050075	NA	FBtr0087555
FBgn0050077	FBgn0050077	NA	FBtr0301734
FBgn0050080	FBgn0050080	NA	FBtr0087319
FBgn0050082	FBgn0050082	NA	FBtr0302917
FBgn0050083	FBgn0050083	NA	FBtr0300381
FBgn0050088	FBgn0050088	NA	FBtr0273296
FBgn0050089	FBgn0050089	NA	FBtr0087285
FBgn0050091	FBgn0050091	NA	FBtr0087277
FBgn0050093	FBgn0050093	NA	FBtr0087199
FBgn0050095	FBgn0050095	NA	FBtr0087224
FBgn0050096	FBgn0050096	NA	FBtr0289994
FBgn0050098	FBgn0050098	NA	FBtr0087168
FBgn0050099	FBgn0050099	NA	FBtr0087180
FBgn0050100	FBgn0050100	NA	FBtr0087140
FBgn0050101	FBgn0050101	CG30101	FBtr0086963
FBgn0050105	FBgn0050105	NA	FBtr0086899
FBgn0050106	FBgn0050106	NA	FBtr0086893
FBgn0050108	FBgn0050108	NA	FBtr0273298
FBgn0050109	FBgn0050109	NA	FBtr0086838
FBgn0050114	FBgn0050114	NA	FBtr0086753
FBgn0050115	FBgn0050115	CG30115	FBtr0310055
FBgn0050118	FBgn0050118	CG30118	FBtr0113361
FBgn0050120	FBgn0050120	NA	FBtr0299806
FBgn0050121	FBgn0050121	NA	FBtr0308920
FBgn0050122	FBgn0050122	NA	FBtr0300923
FBgn0050127	FBgn0050127	NA	FBtr0308496
FBgn0050156	FBgn0050156	NA	FBtr0086184
FBgn0050157	FBgn0050157	NA	FBtr0086186
FBgn0050159	FBgn0050159	CG30159	FBtr0301713
FBgn0050160	FBgn0050160	NA	FBtr0086178
FBgn0050161	FBgn0050161	NA	FBtr0072333
FBgn0050169	FBgn0050169	NA	FBtr0072302
FBgn0050177	FBgn0050177	NA	FBtr0072122
FBgn0050178	FBgn0050178	NA	FBtr0072159
FBgn0050183	FBgn0050183	NA	FBtr0072099
FBgn0050184	FBgn0050184	NA	FBtr0072065
FBgn0050187	FBgn0050187	NA	FBtr0304796
FBgn0050194	FBgn0050194	CG30194	FBtr0071954

FBgn0050195	FBgn0050195	NA	FBtr0071931
FBgn0050196	FBgn0050196	NA	FBtr0071927
FBgn0050203	FBgn0050203	NA	FBtr0273303
FBgn0050222	FBgn0050222	NA	FBtr0300458
FBgn0050259	FBgn0050259	NA	FBtr0071921
FBgn0050263	FBgn0050263	NA	FBtr0273308
FBgn0050265	FBgn0050265	NA	FBtr0071891
FBgn0050267	FBgn0050267	NA	FBtr0309275
FBgn0050268	FBgn0050268	NA	FBtr0273311
FBgn0050269	FBgn0050269	NA	FBtr0071867
FBgn0050271	FBgn0050271	NA	FBtr0303513
FBgn0050273	FBgn0050273	NA	FBtr0071866
FBgn0050275	FBgn0050275	NA	FBtr0273316
FBgn0050277	FBgn0050277	NA	FBtr0071779
FBgn0050278	FBgn0050278	NA	FBtr0071780
FBgn0050280	FBgn0050280	NA	FBtr0300928
FBgn0050281	FBgn0050281	NA	FBtr0071760
FBgn0050286	FBgn0050286	NA	FBtr0300951
FBgn0050287	FBgn0050287	NA	FBtr0071695
FBgn0050288	FBgn0050288	NA	FBtr0113367
FBgn0050290	FBgn0050290	NA	FBtr0071672
FBgn0050291	FBgn0050291	CG30291	FBtr0071549
FBgn0050293	FBgn0050293	NA	FBtr0071544
FBgn0050295	FBgn0050295	Ipk1	FBtr0113368
FBgn0050296	FBgn0050296	NA	FBtr0310057
FBgn0050323	FBgn0050323	NA	FBtr0273321
FBgn0050324	FBgn0050324	NA	FBtr0087181
FBgn0050334	FBgn0050334	NA	FBtr0087949
FBgn0050338	FBgn0050338	NA	FBtr0088533
FBgn0050339	FBgn0050339	NA	FBtr0088534
FBgn0050340	FBgn0050340	NA	FBtr0088535
FBgn0050342	FBgn0050342	NA	FBtr0088582
FBgn0050343	FBgn0050343	NA	FBtr0088594
FBgn0050344	FBgn0050344	CG30344	FBtr0088583
FBgn0050345	FBgn0050345	NA	FBtr0305317
FBgn0050354	FBgn0050354	NA	FBtr0088702
FBgn0050357	FBgn0050357	NA	FBtr0088721
FBgn0050359	FBgn0050359	NA	FBtr0300953
FBgn0050360	FBgn0050360	NA	FBtr0273322
FBgn0050361	FBgn0050361	NA	FBtr0273325
FBgn0050362	FBgn0050362	NA	FBtr0088769
FBgn0050363	FBgn0050363	NA	FBtr0088770
FBgn0050365	FBgn0050365	NA	FBtr0088773
FBgn0050366	FBgn0050366	NA	FBtr0113373
FBgn0050369	FBgn0050369	NA	FBtr0088795
FBgn0050371	FBgn0050371	NA	FBtr0088798
FBgn0050372	FBgn0050372	NA	FBtr0300954
FBgn0050373	FBgn0050373	NA	FBtr0088806
FBgn0050374	FBgn0050374	NA	FBtr0303310
FBgn0050375	FBgn0050375	NA	FBtr0088799
FBgn0050376	FBgn0050376	NA	FBtr0088800
FBgn0050377	FBgn0050377	CG30377	FBtr0088844
FBgn0050378	FBgn0050378	NA	FBtr0088854
FBgn0050379	FBgn0050379	NA	FBtr0305125
FBgn0050380	FBgn0050380	NA	FBtr0088881
FBgn0050381	FBgn0050381	NA	FBtr0113374
FBgn0050382	FBgn0050382	NA	FBtr0088894

FBgn0050383	FBgn0050383	NA	FBtr0088897
FBgn0050389	FBgn0050389	NA	FBtr0310423
FBgn0050390	FBgn0050390	NA	FBtr0071617
FBgn0050391	FBgn0050391	NA	FBtr0071621
FBgn0050392	FBgn0050392	NA	FBtr0071618
FBgn0050393	FBgn0050393	NA	FBtr0071620
FBgn0050394	FBgn0050394	CG30394	FBtr0071614
FBgn0050395	FBgn0050395	NA	FBtr0300226
FBgn0050398	FBgn0050398	NA	FBtr0071710
FBgn0050401	FBgn0050401	NA	FBtr0071731
FBgn0050403	FBgn0050403	NA	FBtr0114523
FBgn0050404	FBgn0050404	NA	FBtr0071747
FBgn0050409	FBgn0050409	NA	FBtr0072004
FBgn0050410	FBgn0050410	NA	FBtr0305675
FBgn0050412	FBgn0050412	NA	FBtr0072043
FBgn0050413	FBgn0050413	NA	FBtr0072040
FBgn0050414	FBgn0050414	NA	FBtr0072032
FBgn0050416	FBgn0050416	NA	FBtr0300955
FBgn0050417	FBgn0050417	NA	FBtr0072041
FBgn0050418	FBgn0050418	nord	FBtr0072263
FBgn0050419	FBgn0050419	NA	FBtr0072275
FBgn0050420	FBgn0050420	NA	FBtr0072382
FBgn0050423	FBgn0050423	NA	FBtr0300122
FBgn0050424	FBgn0050424	NA	FBtr0113377
FBgn0050428	FBgn0050428	NA	FBtr0072451
FBgn0050429	FBgn0050429	NA	FBtr0072450
FBgn0050431	FBgn0050431	NA	FBtr0085991
FBgn0050432	FBgn0050432	NA	FBtr0307499
FBgn0050438	FBgn0050438	NA	FBtr0086069
FBgn0050441	FBgn0050441	NA	FBtr0299872
FBgn0050443	FBgn0050443	NA	FBtr0086109
FBgn0050446	FBgn0050446	NA	FBtr0086142
FBgn0050447	FBgn0050447	NA	FBtr0086338
FBgn0050456	FBgn0050456	NA	FBtr0273336
FBgn0050458	FBgn0050458	NA	FBtr0086999
FBgn0050460	FBgn0050460	NA	FBtr0113378
FBgn0050461	FBgn0050461	NA	FBtr0100252
FBgn0050462	FBgn0050462	NA	FBtr0100120
FBgn0050463	FBgn0050463	NA	FBtr0087112
FBgn0050464	FBgn0050464	NA	FBtr0273344
FBgn0050466	FBgn0050466	NA	FBtr0087339
FBgn0050467	FBgn0050467	NA	FBtr0087334
FBgn0050469	FBgn0050469	NA	FBtr0273350
FBgn0050471	FBgn0050471	NA	FBtr0087333
FBgn0050472	FBgn0050472	NA	FBtr0087360
FBgn0050476	FBgn0050476	NA	FBtr0087439
FBgn0050479	FBgn0050479	NA	FBtr0087479
FBgn0050485	FBgn0050485	NA	FBtr0087640
FBgn0050486	FBgn0050486	NA	FBtr0087817
FBgn0050487	FBgn0050487	NA	FBtr0087802
FBgn0050488	FBgn0050488	NA	FBtr0306584
FBgn0050491	FBgn0050491	CG30491	FBtr0088919
FBgn0050493	FBgn0050493	NA	FBtr0088925
FBgn0050495	FBgn0050495	NA	FBtr0088920
FBgn0050496	FBgn0050496	NA	FBtr0088926
FBgn0050497	FBgn0050497	CG30497	FBtr0088932
FBgn0050499	FBgn0050499	CG30499	FBtr0310026

FBgn0050502	FBgn0050502	NA	FBtr0300959
FBgn0050503	FBgn0050503	NA	FBtr0299802
FBgn0051002	FBgn0051002	NA	FBtr0085813
FBgn0051004	FBgn0051004	CG31004	FBtr0300961
FBgn0051013	FBgn0051013	NA	FBtr0085671
FBgn0051014	FBgn0051014	NA	FBtr0085686
FBgn0051015	FBgn0051015	NA	FBtr0085670
FBgn0051016	FBgn0051016	NA	FBtr0300962
FBgn0051017	FBgn0051017	NA	FBtr0085667
FBgn0051021	FBgn0051021	NA	FBtr0300983
FBgn0051025	FBgn0051025	NA	FBtr0085586
FBgn0051028	FBgn0051028	NA	FBtr0290051
FBgn0051030	FBgn0051030	NA	FBtr0085579
FBgn0051031	FBgn0051031	NA	FBtr0085566
FBgn0051032	FBgn0051032	NA	FBtr0085524
FBgn0051036	FBgn0051036	CG31036	FBtr0114571
FBgn0051040	FBgn0051040	NA	FBtr0085509
FBgn0051041	FBgn0051041	NA	FBtr0085470
FBgn0051044	FBgn0051044	NA	FBtr0085391
FBgn0051048	FBgn0051048	NA	FBtr0289996
FBgn0051050	FBgn0051050	NA	FBtr0085317
FBgn0051051	FBgn0051051	NA	FBtr0085296
FBgn0051053	FBgn0051053	NA	FBtr0085268
FBgn0051054	FBgn0051054	NA	FBtr0307118
FBgn0051064	FBgn0051064	NA	FBtr0085126
FBgn0051065	FBgn0051065	NA	FBtr0113383
FBgn0051068	FBgn0051068	NA	FBtr0303570
FBgn0051072	FBgn0051072	NA	FBtr0300939
FBgn0051075	FBgn0051075	NA	FBtr0307506
FBgn0051076	FBgn0051076	NA	FBtr0307505
FBgn0051077	FBgn0051077	NA	FBtr0301977
FBgn0051084	FBgn0051084	NA	FBtr0085007
FBgn0051086	FBgn0051086	NA	FBtr0307341
FBgn0051087	FBgn0051087	NA	FBtr0084855
FBgn0051089	FBgn0051089	NA	FBtr0084942
FBgn0051091	FBgn0051091	NA	FBtr0084941
FBgn0051092	FBgn0051092	NA	FBtr0113386
FBgn0051093	FBgn0051093	NA	FBtr0084917
FBgn0051097	FBgn0051097	NA	FBtr0290052
FBgn0051098	FBgn0051098	CG31098	FBtr0084863
FBgn0051099	FBgn0051099	NA	FBtr0084856
FBgn0051102	FBgn0051102	NA	FBtr0084862
FBgn0051103	FBgn0051103	NA	FBtr0113389
FBgn0051104	FBgn0051104	NA	FBtr0084825
FBgn0051105	FBgn0051105	NA	FBtr0273357
FBgn0051106	FBgn0051106	NA	FBtr0301022
FBgn0051109	FBgn0051109	CG31109	FBtr0084791
FBgn0051111	FBgn0051111	NA	FBtr0084790
FBgn0051115	FBgn0051115	NA	FBtr0290053
FBgn0051116	FBgn0051116	CG31116	FBtr0301023
FBgn0051118	FBgn0051118	NA	FBtr0084736
FBgn0051119	FBgn0051119	NA	FBtr0305045
FBgn0051120	FBgn0051120	CG31120	FBtr0089439
FBgn0051122	FBgn0051122	NA	FBtr0301432
FBgn0051125	FBgn0051125	NA	FBtr0084665
FBgn0051126	FBgn0051126	NA	FBtr0084663
FBgn0051131	FBgn0051131	NA	FBtr0300438

FBgn0051133	FBgn0051133	NA	FBtr0084583
FBgn0051139	FBgn0051139	NA	FBtr0084321
FBgn0051140	FBgn0051140	NA	FBtr0084497
FBgn0051141	FBgn0051141	NA	FBtr0084490
FBgn0051144	FBgn0051144	NA	FBtr0303864
FBgn0051145	FBgn0051145	CG31145	FBtr0290056
FBgn0051146	FBgn0051146	NA	FBtr0081541
FBgn0051151	FBgn0051151	NA	FBtr0310426
FBgn0051155	FBgn0051155	NA	FBtr0083137
FBgn0051156	FBgn0051156	NA	FBtr0084277
FBgn0051157	FBgn0051157	NA	FBtr0113394
FBgn0051158	FBgn0051158	NA	FBtr0302187
FBgn0051159	FBgn0051159	NA	FBtr0084270
FBgn0051161	FBgn0051161	NA	FBtr0304668
FBgn0051163	FBgn0051163	NA	FBtr0305036
FBgn0051174	FBgn0051174	NA	FBtr0084154
FBgn0051184	FBgn0051184	NA	FBtr0084505
FBgn0051189	FBgn0051189	NA	FBtr0300817
FBgn0051190	FBgn0051190	NA	FBtr0113397
FBgn0051191	FBgn0051191	NA	FBtr0083984
FBgn0051195	FBgn0051195	NA	FBtr0302188
FBgn0051199	FBgn0051199	NA	FBtr0083930
FBgn0051200	FBgn0051200	NA	FBtr0083929
FBgn0051201	FBgn0051201	NA	FBtr0110837
FBgn0051202	FBgn0051202	NA	FBtr0085510
FBgn0051204	FBgn0051204	NA	FBtr0085814
FBgn0051205	FBgn0051205	NA	FBtr0300451
FBgn0051206	FBgn0051206	NA	FBtr0083875
FBgn0051207	FBgn0051207	NA	FBtr0084044
FBgn0051217	FBgn0051217	NA	FBtr0083353
FBgn0051219	FBgn0051219	NA	FBtr0083783
FBgn0051220	FBgn0051220	NA	FBtr0302358
FBgn0051223	FBgn0051223	NA	FBtr0083965
FBgn0051225	FBgn0051225	NA	FBtr0290293
FBgn0051229	FBgn0051229	NA	FBtr0083663
FBgn0051230	FBgn0051230	NA	FBtr0083666
FBgn0051231	FBgn0051231	NA	FBtr0083662
FBgn0051232	FBgn0051232	CG31232	FBtr0083625
FBgn0051244	FBgn0051244	NA	FBtr0083772
FBgn0051248	FBgn0051248	NA	FBtr0081564
FBgn0051249	FBgn0051249	NA	FBtr0083483
FBgn0051251	FBgn0051251	NA	FBtr0083484
FBgn0051253	FBgn0051253	NA	FBtr0085263
FBgn0051258	FBgn0051258	NA	FBtr0089973
FBgn0051259	FBgn0051259	CG31259	FBtr0081871
FBgn0051262	FBgn0051262	NA	FBtr0300053
FBgn0051265	FBgn0051265	NA	FBtr0083402
FBgn0051266	FBgn0051266	NA	FBtr0300060
FBgn0051267	FBgn0051267	NA	FBtr0083404
FBgn0051268	FBgn0051268	NA	FBtr0307190
FBgn0051269	FBgn0051269	NA	FBtr0113401
FBgn0051272	FBgn0051272	CG31272	FBtr0082312
FBgn0051278	FBgn0051278	NA	FBtr0082319
FBgn0051279	FBgn0051279	NA	FBtr0310428
FBgn0051281	FBgn0051281	NA	FBtr0084339
FBgn0051286	FBgn0051286	NA	FBtr0081682
FBgn0051288	FBgn0051288	NA	FBtr0084860

FBgn0051291	FBgn0051291	CG31291	FBtr0083244
FBgn0051292	FBgn0051292	NA	FBtr0304144
FBgn0051296	FBgn0051296	NA	FBtr0083155
FBgn0051301	FBgn0051301	CG31301	FBtr0083087
FBgn0051314	FBgn0051314	Neu3	FBtr0301498
FBgn0051321	FBgn0051321	NA	FBtr0082926
FBgn0051323	FBgn0051323	NA	FBtr0084988
FBgn0051324	FBgn0051324	CG31324	FBtr0084985
FBgn0051326	FBgn0051326	NA	FBtr0082901
FBgn0051327	FBgn0051327	NA	FBtr0082904
FBgn0051337	FBgn0051337	NA	FBtr0082790
FBgn0051342	FBgn0051342	NA	FBtr0113404
FBgn0051343	FBgn0051343	NA	FBtr0084126
FBgn0051345	FBgn0051345	NA	FBtr0082689
FBgn0051347	FBgn0051347	NA	FBtr0082664
FBgn0051352	FBgn0051352	NA	FBtr0082092
FBgn0051357	FBgn0051357	NA	FBtr0084735
FBgn0051358	FBgn0051358	NA	FBtr0082554
FBgn0051360	FBgn0051360	NA	FBtr0083480
FBgn0051361	FBgn0051361	dpr17	FBtr0082542
FBgn0051363	FBgn0051363	Jupiter	FBtr0082454
FBgn0051368	FBgn0051368	CG31368	FBtr0301042
FBgn0051370	FBgn0051370	NA	FBtr0290055
FBgn0051371	FBgn0051371	NA	FBtr0302522
FBgn0051373	FBgn0051373	NA	FBtr0082318
FBgn0051374	FBgn0051374	NA	FBtr0082399
FBgn0051380	FBgn0051380	NA	FBtr0084918
FBgn0051386	FBgn0051386	NA	FBtr0308921
FBgn0051388	FBgn0051388	NA	FBtr0304709
FBgn0051390	FBgn0051390	NA	FBtr0082311
FBgn0051391	FBgn0051391	NA	FBtr0082321
FBgn0051407	FBgn0051407	NA	FBtr0082214
FBgn0051410	FBgn0051410	NA	FBtr0273369
FBgn0051413	FBgn0051413	NA	FBtr0084442
FBgn0051414	FBgn0051414	NA	FBtr0113405
FBgn0051415	FBgn0051415	NA	FBtr0082127
FBgn0051418	FBgn0051418	NA	FBtr0083432
FBgn0051419	FBgn0051419	NA	FBtr0309516
FBgn0051427	FBgn0051427	NA	FBtr0113407
FBgn0051431	FBgn0051431	NA	FBtr0308826
FBgn0051436	FBgn0051436	NA	FBtr0084830
FBgn0051437	FBgn0051437	NA	FBtr0084834
FBgn0051439	FBgn0051439	NA	FBtr0084845
FBgn0051441	FBgn0051441	NA	FBtr0082355
FBgn0051445	FBgn0051445	NA	FBtr0085401
FBgn0051451	FBgn0051451	NA	FBtr0308923
FBgn0051454	FBgn0051454	NA	FBtr0081870
FBgn0051464	FBgn0051464	NA	FBtr0306804
FBgn0051465	FBgn0051465	NA	FBtr0084147
FBgn0051467	FBgn0051467	NA	FBtr0082320
FBgn0051469	FBgn0051469	NA	FBtr0082844
FBgn0051472	FBgn0051472	NA	FBtr0081748
FBgn0051473	FBgn0051473	NA	FBtr0081749
FBgn0051477	FBgn0051477	NA	FBtr0113408
FBgn0051493	FBgn0051493	NA	FBtr0081566
FBgn0051496	FBgn0051496	NA	FBtr0081582
FBgn0051501	FBgn0051501	NA	FBtr0081583

FBgn0051514	FBgn0051514	NA	FBtr0304109
FBgn0051515	FBgn0051515	NA	FBtr0304565
FBgn0051522	FBgn0051522	CG31522	FBtr0306343
FBgn0051524	FBgn0051524	NA	FBtr0085684
FBgn0051525	FBgn0051525	NA	FBtr0078924
FBgn0051526	FBgn0051526	NA	FBtr0078929
FBgn0051533	FBgn0051533	NA	FBtr0082905
FBgn0051536	FBgn0051536	Cdep	FBtr0110958
FBgn0051538	FBgn0051538	NA	FBtr0078819
FBgn0051542	FBgn0051542	NA	FBtr0302421
FBgn0051546	FBgn0051546	NA	FBtr0078737
FBgn0051548	FBgn0051548	NA	FBtr0078736
FBgn0051549	FBgn0051549	CG31549	FBtr0078707
FBgn0051550	FBgn0051550	NA	FBtr0078684
FBgn0051559	FBgn0051559	CG31559	FBtr0078616
FBgn0051600	FBgn0051600	NA	FBtr0085949
FBgn0051601	FBgn0051601	NA	FBtr0307507
FBgn0051606	FBgn0051606	NA	FBtr0079577
FBgn0051609	FBgn0051609	NA	FBtr0299873
FBgn0051612	FBgn0051612	NA	FBtr0300999
FBgn0051619	FBgn0051619	CG31619	FBtr0307497
FBgn0051624	FBgn0051624	NA	FBtr0301928
FBgn0051627	FBgn0051627	NA	FBtr0300424
FBgn0051633	FBgn0051633	NA	FBtr0079325
FBgn0051635	FBgn0051635	NA	FBtr0113410
FBgn0051636	FBgn0051636	NA	FBtr0079324
FBgn0051638	FBgn0051638	NA	FBtr0079262
FBgn0051641	FBgn0051641	NA	FBtr0304908
FBgn0051642	FBgn0051642	NA	FBtr0079199
FBgn0051644	FBgn0051644	NA	FBtr0079159
FBgn0051646	FBgn0051646	NA	FBtr0079114
FBgn0051647	FBgn0051647	NA	FBtr0308924
FBgn0051648	FBgn0051648	NA	FBtr0079098
FBgn0051658	FBgn0051658	NA	FBtr0078008
FBgn0051659	FBgn0051659	NA	FBtr0301010
FBgn0051660	FBgn0051660	CG31660	FBtr0304910
FBgn0051663	FBgn0051663	CG31663	FBtr0077893
FBgn0051664	FBgn0051664	NA	FBtr0303377
FBgn0051665	FBgn0051665	CG31665	FBtr0089787
FBgn0051668	FBgn0051668	NA	FBtr0307523
FBgn0051673	FBgn0051673	CG31673	FBtr0081422
FBgn0051674	FBgn0051674	NA	FBtr0081423
FBgn0051675	FBgn0051675	NA	FBtr0081429
FBgn0051677	FBgn0051677	NA	FBtr0081407
FBgn0051678	FBgn0051678	NA	FBtr0290059
FBgn0051679	FBgn0051679	NA	FBtr0077809
FBgn0051680	FBgn0051680	NA	FBtr0081383
FBgn0051681	FBgn0051681	NA	FBtr0077802
FBgn0051682	FBgn0051682	NA	FBtr0077807
FBgn0051686	FBgn0051686	NA	FBtr0077752
FBgn0051687	FBgn0051687	NA	FBtr0081361
FBgn0051688	FBgn0051688	NA	FBtr0308895
FBgn0051690	FBgn0051690	NA	FBtr0301681
FBgn0051694	FBgn0051694	NA	FBtr0077685
FBgn0051698	FBgn0051698	NA	FBtr0077612
FBgn0051700	FBgn0051700	NA	FBtr0301969
FBgn0051702	FBgn0051702	NA	FBtr0085934

FBgn0051703	FBgn0051703	NA	FBtr0085946
FBgn0051704	FBgn0051704	NA	FBtr0080273
FBgn0051706	FBgn0051706	NA	FBtr0080241
FBgn0051708	FBgn0051708	NA	FBtr0079776
FBgn0051710	FBgn0051710	NA	FBtr0079849
FBgn0051712	FBgn0051712	NA	FBtr0079896
FBgn0051713	FBgn0051713	NA	FBtr0079895
FBgn0051715	FBgn0051715	NA	FBtr0079996
FBgn0051716	FBgn0051716	CG31716	FBtr0304112
FBgn0051717	FBgn0051717	NA	FBtr0079977
FBgn0051720	FBgn0051720	NA	FBtr0301974
FBgn0051721	FBgn0051721	Trim9	FBtr0304114
FBgn0051728	FBgn0051728	NA	FBtr0305556
FBgn0051729	FBgn0051729	NA	FBtr0080461
FBgn0051730	FBgn0051730	NA	FBtr0080480
FBgn0051735	FBgn0051735	NA	FBtr0080790
FBgn0051740	FBgn0051740	NA	FBtr0081028
FBgn0051742	FBgn0051742	NA	FBtr0081025
FBgn0051755	FBgn0051755	NA	FBtr0303882
FBgn0051759	FBgn0051759	NA	FBtr0290240
FBgn0051760	FBgn0051760	NA	FBtr0301019
FBgn0051769	FBgn0051769	NA	FBtr0080602
FBgn0051773	FBgn0051773	NA	FBtr0077509
FBgn0051774	FBgn0051774	NA	FBtr0308125
FBgn0051776	FBgn0051776	NA	FBtr0077522
FBgn0051778	FBgn0051778	NA	FBtr0077543
FBgn0051779	FBgn0051779	NA	FBtr0305673
FBgn0051780	FBgn0051780	NA	FBtr0080758
FBgn0051781	FBgn0051781	CG31781	FBtr0308927
FBgn0051784	FBgn0051784	NA	FBtr0302499
FBgn0051785	FBgn0051785	NA	FBtr0300025
FBgn0051787	FBgn0051787	NA	FBtr0081055
FBgn0051788	FBgn0051788	NA	FBtr0081056
FBgn0051792	FBgn0051792	NA	FBtr0321312
FBgn0051793	FBgn0051793	NA	FBtr0310616
FBgn0051797	FBgn0051797	NA	FBtr0081227
FBgn0051798	FBgn0051798	NA	FBtr0081200
FBgn0051802	FBgn0051802	NA	FBtr0081058
FBgn0051803	FBgn0051803	NA	FBtr0081010
FBgn0051804	FBgn0051804	NA	FBtr0080967
FBgn0051805	FBgn0051805	NA	FBtr0304932
FBgn0051806	FBgn0051806	NA	FBtr0080952
FBgn0051807	FBgn0051807	NA	FBtr0080944
FBgn0051809	FBgn0051809	NA	FBtr0113414
FBgn0051812	FBgn0051812	NA	FBtr0290062
FBgn0051815	FBgn0051815	NA	FBtr0080870
FBgn0051816	FBgn0051816	NA	FBtr0080868
FBgn0051820	FBgn0051820	NA	FBtr0080807
FBgn0051821	FBgn0051821	NA	FBtr0080809
FBgn0051822	FBgn0051822	NA	FBtr0304145
FBgn0051824	FBgn0051824	NA	FBtr0303311
FBgn0051835	FBgn0051835	NA	FBtr0080667
FBgn0051845	FBgn0051845	NA	FBtr0307578
FBgn0051848	FBgn0051848	NA	FBtr0303784
FBgn0051851	FBgn0051851	NA	FBtr0080454
FBgn0051852	FBgn0051852	NA	FBtr0080466
FBgn0051855	FBgn0051855	NA	FBtr0290000

FBgn0051858	FBgn0051858	NA	FBtr0080381
FBgn0051860	FBgn0051860	NA	FBtr0302984
FBgn0051861	FBgn0051861	NA	FBtr0302986
FBgn0051864	FBgn0051864	NA	FBtr0321269
FBgn0051865	FBgn0051865	NA	FBtr0080289
FBgn0051866	FBgn0051866	NA	FBtr0080287
FBgn0051867	FBgn0051867	NA	FBtr0080224
FBgn0051869	FBgn0051869	NA	FBtr0290245
FBgn0051872	FBgn0051872	NA	FBtr0080099
FBgn0051874	FBgn0051874	NA	FBtr0079964
FBgn0051875	FBgn0051875	NA	FBtr0303883
FBgn0051882	FBgn0051882	NA	FBtr0079853
FBgn0051897	FBgn0051897	NA	FBtr0079673
FBgn0051901	FBgn0051901	NA	FBtr0079607
FBgn0051902	FBgn0051902	NA	FBtr0110841
FBgn0051907	FBgn0051907	NA	FBtr0079425
FBgn0051909	FBgn0051909	NA	FBtr0300460
FBgn0051913	FBgn0051913	NA	FBtr0079138
FBgn0051915	FBgn0051915	NA	FBtr0079099
FBgn0051922	FBgn0051922	NA	FBtr0077965
FBgn0051924	FBgn0051924	NA	FBtr0077946
FBgn0051928	FBgn0051928	NA	FBtr0077929
FBgn0051933	FBgn0051933	NA	FBtr0077896
FBgn0051935	FBgn0051935	NA	FBtr0077916
FBgn0051948	FBgn0051948	NA	FBtr0077787
FBgn0051949	FBgn0051949	NA	FBtr0303208
FBgn0051950	FBgn0051950	NA	FBtr0077630
FBgn0051952	FBgn0051952	NA	FBtr0077591
FBgn0051953	FBgn0051953	NA	FBtr0077575
FBgn0051954	FBgn0051954	NA	FBtr0077563
FBgn0051955	FBgn0051955	NA	FBtr0077540
FBgn0051956	FBgn0051956	NA	FBtr0077523
FBgn0051957	FBgn0051957	NA	FBtr0077514
FBgn0051960	FBgn0051960	NA	FBtr0077494
FBgn0051973	FBgn0051973	NA	FBtr0078164
FBgn0051974	FBgn0051974	NA	FBtr0300987
FBgn0051975	FBgn0051975	NA	FBtr0302196
FBgn0051976	FBgn0051976	NA	FBtr0302194
FBgn0051988	FBgn0051988	NA	FBtr0081490
FBgn0051989	FBgn0051989	NA	FBtr0079097
FBgn0051992	FBgn0051992	NA	FBtr0310542
FBgn0051997	FBgn0051997	NA	FBtr0089154
FBgn0051998	FBgn0051998	NA	FBtr0308249
FBgn0051999	FBgn0051999	NA	FBtr0089144
FBgn0052000	FBgn0052000	NA	FBtr0113417
FBgn0052009	FBgn0052009	NA	FBtr0089180
FBgn0052010	FBgn0052010	NA	FBtr0089181
FBgn0052011	FBgn0052011	NA	FBtr0089182
FBgn0052016	FBgn0052016	NA	FBtr0089233
FBgn0052017	FBgn0052017	NA	FBtr0300992
FBgn0052023	FBgn0052023	NA	FBtr0076588
FBgn0052024	FBgn0052024	NA	FBtr0076580
FBgn0052026	FBgn0052026	CG32026	FBtr0076584
FBgn0052027	FBgn0052027	NA	FBtr0307116
FBgn0052029	FBgn0052029	CG32029	FBtr0076567
FBgn0052036	FBgn0052036	NA	FBtr0114525
FBgn0052038	FBgn0052038	NA	FBtr0076462

FBgn0052039	FBgn0052039	NA	FBtr0076441
FBgn0052040	FBgn0052040	NA	FBtr0076440
FBgn0052043	FBgn0052043	NA	FBtr0076466
FBgn0052053	FBgn0052053	NA	FBtr0076315
FBgn0052054	FBgn0052054	NA	FBtr0076314
FBgn0052061	FBgn0052061	NA	FBtr0076285
FBgn0052062	FBgn0052062	NA	FBtr0305093
FBgn0052064	FBgn0052064	NA	FBtr0076288
FBgn0052068	FBgn0052068	NA	FBtr0113421
FBgn0052069	FBgn0052069	NA	FBtr0076241
FBgn0052072	FBgn0052072	NA	FBtr0076232
FBgn0052073	FBgn0052073	NA	FBtr0076230
FBgn0052074	FBgn0052074	NA	FBtr0076222
FBgn0052075	FBgn0052075	NA	FBtr0076240
FBgn0052076	FBgn0052076	Alg10	FBtr0076217
FBgn0052079	FBgn0052079	NA	FBtr0300188
FBgn0052081	FBgn0052081	NA	FBtr0076198
FBgn0052082	FBgn0052082	NA	FBtr0076216
FBgn0052086	FBgn0052086	NA	FBtr0076147
FBgn0052087	FBgn0052087	NA	FBtr0076117
FBgn0052095	FBgn0052095	NA	FBtr0076077
FBgn0052099	FBgn0052099	NA	FBtr0076000
FBgn0052100	FBgn0052100	NA	FBtr0076006
FBgn0052102	FBgn0052102	NA	FBtr0075978
FBgn0052106	FBgn0052106	NA	FBtr0307888
FBgn0052107	FBgn0052107	NA	FBtr0306792
FBgn0052109	FBgn0052109	NA	FBtr0075918
FBgn0052110	FBgn0052110	NA	FBtr0075931
FBgn0052111	FBgn0052111	NA	FBtr0075910
FBgn0052112	FBgn0052112	NA	FBtr0301047
FBgn0052113	FBgn0052113	NA	FBtr0273390
FBgn0052115	FBgn0052115	NA	FBtr0075904
FBgn0052117	FBgn0052117	NA	FBtr0075905
FBgn0052119	FBgn0052119	NA	FBtr0075867
FBgn0052121	FBgn0052121	NA	FBtr0113424
FBgn0052130	FBgn0052130	NA	FBtr0113425
FBgn0052138	FBgn0052138	NA	FBtr0075775
FBgn0052141	FBgn0052141	NA	FBtr0075735
FBgn0052150	FBgn0052150	NA	FBtr0075534
FBgn0052155	FBgn0052155	NA	FBtr0075459
FBgn0052159	FBgn0052159	NA	FBtr0075409
FBgn0052160	FBgn0052160	NA	FBtr0308930
FBgn0052161	FBgn0052161	NA	FBtr0075331
FBgn0052163	FBgn0052163	NA	FBtr0075310
FBgn0052170	FBgn0052170	NA	FBtr0075258
FBgn0052174	FBgn0052174	NA	FBtr0299819
FBgn0052181	FBgn0052181	NA	FBtr0301018
FBgn0052185	FBgn0052185	NA	FBtr0075174
FBgn0052187	FBgn0052187	NA	FBtr0075169
FBgn0052190	FBgn0052190	NUCB1	FBtr0075171
FBgn0052191	FBgn0052191	NA	FBtr0307509
FBgn0052192	FBgn0052192	NA	FBtr0075147
FBgn0052194	FBgn0052194	NA	FBtr0306733
FBgn0052195	FBgn0052195	NA	FBtr0075125
FBgn0052196	FBgn0052196	NA	FBtr0273401
FBgn0052201	FBgn0052201	NA	FBtr0300051
FBgn0052203	FBgn0052203	NA	FBtr0110771

FBgn0052204	FBgn0052204	NA	FBtr0113429
FBgn0052205	FBgn0052205	NA	FBtr0075000
FBgn0052206	FBgn0052206	NA	FBtr0089796
FBgn0052207	FBgn0052207	NA	FBtr0303927
FBgn0052208	FBgn0052208	NA	FBtr0273406
FBgn0052213	FBgn0052213	NA	FBtr0273409
FBgn0052214	FBgn0052214	NA	FBtr0273411
FBgn0052218	FBgn0052218	NA	FBtr0304110
FBgn0052219	FBgn0052219	NA	FBtr0074914
FBgn0052220	FBgn0052220	NA	FBtr0074911
FBgn0052221	FBgn0052221	NA	FBtr0301008
FBgn0052225	FBgn0052225	NA	FBtr0074845
FBgn0052238	FBgn0052238	NA	FBtr0073344
FBgn0052240	FBgn0052240	NA	FBtr0073336
FBgn0052241	FBgn0052241	NA	FBtr0303006
FBgn0052243	FBgn0052243	NA	FBtr0073322
FBgn0052246	FBgn0052246	NA	FBtr0302941
FBgn0052248	FBgn0052248	NA	FBtr0303004
FBgn0052249	FBgn0052249	NA	FBtr0303003
FBgn0052250	FBgn0052250	NA	FBtr0073287
FBgn0052252	FBgn0052252	NA	FBtr0307363
FBgn0052255	FBgn0052255	NA	FBtr0073201
FBgn0052259	FBgn0052259	NA	FBtr0300989
FBgn0052260	FBgn0052260	NA	FBtr0073214
FBgn0052262	FBgn0052262	NA	FBtr0073184
FBgn0052263	FBgn0052263	NA	FBtr0073183
FBgn0052264	FBgn0052264	CG32264	FBtr0308639
FBgn0052265	FBgn0052265	NA	FBtr0073162
FBgn0052266	FBgn0052266	NA	FBtr0073147
FBgn0052267	FBgn0052267	NA	FBtr0073142
FBgn0052268	FBgn0052268	NA	FBtr0073080
FBgn0052269	FBgn0052269	NA	FBtr0100678
FBgn0052270	FBgn0052270	NA	FBtr0100421
FBgn0052271	FBgn0052271	NA	FBtr0073105
FBgn0052277	FBgn0052277	NA	FBtr0073103
FBgn0052279	FBgn0052279	NA	FBtr0073059
FBgn0052282	FBgn0052282	NA	FBtr0073061
FBgn0052283	FBgn0052283	NA	FBtr0073060
FBgn0052284	FBgn0052284	NA	FBtr0073037
FBgn0052296	FBgn0052296	NA	FBtr0306331
FBgn0052298	FBgn0052298	NA	FBtr0299807
FBgn0052299	FBgn0052299	NA	FBtr0299808
FBgn0052301	FBgn0052301	NA	FBtr0301320
FBgn0052302	FBgn0052302	NA	FBtr0072881
FBgn0052304	FBgn0052304	NA	FBtr0072880
FBgn0052305	FBgn0052305	NA	FBtr0301322
FBgn0052311	FBgn0052311	NA	FBtr0110858
FBgn0052318	FBgn0052318	NA	FBtr0072732
FBgn0052319	FBgn0052319	NA	FBtr0072714
FBgn0052320	FBgn0052320	NA	FBtr0300067
FBgn0052333	FBgn0052333	NA	FBtr0072662
FBgn0052343	FBgn0052343	NA	FBtr0309236
FBgn0052351	FBgn0052351	NA	FBtr0076639
FBgn0052364	FBgn0052364	NA	FBtr0076720
FBgn0052368	FBgn0052368	NA	FBtr0076749
FBgn0052369	FBgn0052369	CG32369	FBtr0304992
FBgn0052373	FBgn0052373	NA	FBtr0076806

FBgn0052379	FBgn0052379	NA	FBtr0273419
FBgn0052382	FBgn0052382	NA	FBtr0300362
FBgn0052383	FBgn0052383	NA	FBtr0300363
FBgn0052391	FBgn0052391	NA	FBtr0076930
FBgn0052396	FBgn0052396	NA	FBtr0076969
FBgn0052397	FBgn0052397	NA	FBtr0307513
FBgn0052404	FBgn0052404	NA	FBtr0307048
FBgn0052405	FBgn0052405	NA	FBtr0077024
FBgn0052407	FBgn0052407	NA	FBtr0077032
FBgn0052409	FBgn0052409	NA	FBtr0077030
FBgn0052412	FBgn0052412	CG32412	FBtr0077080
FBgn0052413	FBgn0052413	NA	FBtr0305274
FBgn0052423	FBgn0052423	NA	FBtr0077174
FBgn0052425	FBgn0052425	CG32425	FBtr0078205
FBgn0052428	FBgn0052428	CG32428	FBtr0303381
FBgn0052436	FBgn0052436	NA	FBtr0078343
FBgn0052437	FBgn0052437	NA	FBtr0078344
FBgn0052438	FBgn0052438	NA	FBtr0078372
FBgn0052441	FBgn0052441	NA	FBtr0300058
FBgn0052442	FBgn0052442	NA	FBtr0089797
FBgn0052445	FBgn0052445	NA	FBtr0078425
FBgn0052448	FBgn0052448	NA	FBtr0303248
FBgn0052450	FBgn0052450	NA	FBtr0078501
FBgn0052451	FBgn0052451	NA	FBtr0078533
FBgn0052452	FBgn0052452	NA	FBtr0078523
FBgn0052457	FBgn0052457	NA	FBtr0300619
FBgn0052459	FBgn0052459	NA	FBtr0078578
FBgn0052461	FBgn0052461	NA	FBtr0078567
FBgn0052462	FBgn0052462	NA	FBtr0078579
FBgn0052463	FBgn0052463	NA	FBtr0077808
FBgn0052473	FBgn0052473	CG32473	FBtr0082743
FBgn0052476	FBgn0052476	NA	FBtr0072501
FBgn0052483	FBgn0052483	NA	FBtr0072608
FBgn0052487	FBgn0052487	NA	FBtr0073007
FBgn0052488	FBgn0052488	NA	FBtr0073008
FBgn0052495	FBgn0052495	NA	FBtr0300975
FBgn0052496	FBgn0052496	NA	FBtr0074499
FBgn0052500	FBgn0052500	NA	FBtr0077374
FBgn0052506	FBgn0052506	NA	FBtr0307539
FBgn0052507	FBgn0052507	NA	FBtr0302987
FBgn0052511	FBgn0052511	NA	FBtr0307579
FBgn0052512	FBgn0052512	NA	FBtr0077262
FBgn0052521	FBgn0052521	NA	FBtr0310654
FBgn0052523	FBgn0052523	NA	FBtr0077183
FBgn0052529	FBgn0052529	NA	FBtr0306520
FBgn0052537	FBgn0052537	NA	FBtr0074687
FBgn0052541	FBgn0052541	NA	FBtr0074650
FBgn0052547	FBgn0052547	NA	FBtr0290002
FBgn0052551	FBgn0052551	NA	FBtr0290003
FBgn0052553	FBgn0052553	NA	FBtr0302988
FBgn0052554	FBgn0052554	NA	FBtr0074467
FBgn0052557	FBgn0052557	NA	FBtr0074466
FBgn0052564	FBgn0052564	CG32564	FBtr0074390
FBgn0052568	FBgn0052568	NA	FBtr0301043
FBgn0052569	FBgn0052569	NA	FBtr0290248
FBgn0052570	FBgn0052570	NA	FBtr0074326
FBgn0052571	FBgn0052571	NA	FBtr0074325

FBgn0052573	FBgn0052573	NA	FBtr0074315
FBgn0052574	FBgn0052574	NA	FBtr0074329
FBgn0052576	FBgn0052576	NA	FBtr0100556
FBgn0052581	FBgn0052581	NA	FBtr0074184
FBgn0052582	FBgn0052582	NA	FBtr0074102
FBgn0052590	FBgn0052590	NA	FBtr0073988
FBgn0052591	FBgn0052591	NA	FBtr0073973
FBgn0052594	FBgn0052594	NA	FBtr0073956
FBgn0052595	FBgn0052595	NA	FBtr0073941
FBgn0052598	FBgn0052598	NA	FBtr0073918
FBgn0052601	FBgn0052601	NA	FBtr0073914
FBgn0052603	FBgn0052603	NA	FBtr0073894
FBgn0052625	FBgn0052625	NA	FBtr0073839
FBgn0052626	FBgn0052626	CG32626	FBtr0309245
FBgn0052627	FBgn0052627	NA	FBtr0073813
FBgn0052628	FBgn0052628	NA	FBtr0113442
FBgn0052631	FBgn0052631	NA	FBtr0073799
FBgn0052633	FBgn0052633	NA	FBtr0073800
FBgn0052638	FBgn0052638	NA	FBtr0300453
FBgn0052639	FBgn0052639	NA	FBtr0073734
FBgn0052640	FBgn0052640	NA	FBtr0073728
FBgn0052641	FBgn0052641	NA	FBtr0073729
FBgn0052642	FBgn0052642	NA	FBtr0305598
FBgn0052644	FBgn0052644	NA	FBtr0305599
FBgn0052647	FBgn0052647	NA	FBtr0073703
FBgn0052649	FBgn0052649	NA	FBtr0073697
FBgn0052650	FBgn0052650	NA	FBtr0073698
FBgn0052651	FBgn0052651	NA	FBtr0073672
FBgn0052654	FBgn0052654	NA	FBtr0310305
FBgn0052655	FBgn0052655	NA	FBtr0073633
FBgn0052657	FBgn0052657	NA	FBtr0073625
FBgn0052666	FBgn0052666	NA	FBtr0301020
FBgn0052667	FBgn0052667	NA	FBtr0073474
FBgn0052669	FBgn0052669	NA	FBtr0073412
FBgn0052670	FBgn0052670	NA	FBtr0073401
FBgn0052671	FBgn0052671	NA	FBtr0073402
FBgn0052672	FBgn0052672	NA	FBtr0071498
FBgn0052673	FBgn0052673	NA	FBtr0071505
FBgn0052675	FBgn0052675	NA	FBtr0071501
FBgn0052677	FBgn0052677	NA	FBtr0071493
FBgn0052685	FBgn0052685	CG32685	FBtr0071489
FBgn0052686	FBgn0052686	NA	FBtr0071468
FBgn0052690	FBgn0052690	NA	FBtr0303456
FBgn0052694	FBgn0052694	NA	FBtr0071412
FBgn0052698	FBgn0052698	NA	FBtr0307489
FBgn0052699	FBgn0052699	NA	FBtr0110850
FBgn0052702	FBgn0052702	NA	FBtr0273437
FBgn0052703	FBgn0052703	NA	FBtr0071329
FBgn0052706	FBgn0052706	NA	FBtr0071287
FBgn0052707	FBgn0052707	NA	FBtr0071285
FBgn0052708	FBgn0052708	NA	FBtr0071286
FBgn0052709	FBgn0052709	NA	FBtr0305288
FBgn0052712	FBgn0052712	NA	FBtr0300386
FBgn0052713	FBgn0052713	NA	FBtr0071243
FBgn0052718	FBgn0052718	NA	FBtr0300461
FBgn0052719	FBgn0052719	NA	FBtr0071083
FBgn0052720	FBgn0052720	NA	FBtr0304870

FBgn0052726	FBgn0052726	NA	FBtr0071059
FBgn0052727	FBgn0052727	NA	FBtr0071056
FBgn0052732	FBgn0052732	NA	FBtr0071039
FBgn0052733	FBgn0052733	NA	FBtr0071001
FBgn0052736	FBgn0052736	CG32736	FBtr0071008
FBgn0052743	FBgn0052743	NA	FBtr0070972
FBgn0052745	FBgn0052745	NA	FBtr0070932
FBgn0052751	FBgn0052751	NA	FBtr0070880
FBgn0052755	FBgn0052755	NA	FBtr0070862
FBgn0052756	FBgn0052756	NA	FBtr0070860
FBgn0052758	FBgn0052758	NA	FBtr0070843
FBgn0052767	FBgn0052767	NA	FBtr0302118
FBgn0052772	FBgn0052772	NA	FBtr0300997
FBgn0052773	FBgn0052773	NA	FBtr0070703
FBgn0052774	FBgn0052774	NA	FBtr0307050
FBgn0052792	FBgn0052792	NA	FBtr0307180
FBgn0052803	FBgn0052803	NA	FBtr0070356
FBgn0052806	FBgn0052806	NA	FBtr0070301
FBgn0052811	FBgn0052811	NA	FBtr0302877
FBgn0052813	FBgn0052813	NA	FBtr0070220
FBgn0052814	FBgn0052814	NA	FBtr0302882
FBgn0052815	FBgn0052815	NA	FBtr0307181
FBgn0052816	FBgn0052816	NA	FBtr0070102
FBgn0052817	FBgn0052817	NA	FBtr0300328
FBgn0052819	FBgn0052819	NA	FBtr0070050
FBgn0052820	FBgn0052820	NA	FBtr0070053
FBgn0052821	FBgn0052821	NA	FBtr0307588
FBgn0052822	FBgn0052822	NA	FBtr0305289
FBgn0052832	FBgn0052832	NA	FBtr0080960
FBgn0052833	FBgn0052833	NA	FBtr0071990
FBgn0052834	FBgn0052834	NA	FBtr0303210
FBgn0052835	FBgn0052835	NA	FBtr0308940
FBgn0052843	FBgn0052843	NA	FBtr0087755
FBgn0052845	FBgn0052845	NA	FBtr0072518
FBgn0052846	FBgn0052846	NA	FBtr0073009
FBgn0052847	FBgn0052847	NA	FBtr0075639
FBgn0052855	FBgn0052855	NA	FBtr0083234
FBgn0052856	FBgn0052856	NA	FBtr0302551
FBgn0052857	FBgn0052857	NA	FBtr0070052
FBgn0052939	FBgn0052939	NA	FBtr0082085
FBgn0052944	FBgn0052944	NA	FBtr0290250
FBgn0052945	FBgn0052945	NA	FBtr0078896
FBgn0052971	FBgn0052971	NA	FBtr0114462
FBgn0052982	FBgn0052982	CG32982	FBtr0301040
FBgn0052986	FBgn0052986	NA	FBtr0079734
FBgn0052987	FBgn0052987	NA	FBtr0079735
FBgn0052988	FBgn0052988	NA	FBtr0079736
FBgn0053002	FBgn0053002	mRpL27	FBtr0077451
FBgn0053003	FBgn0053003	NA	FBtr0290071
FBgn0053013	FBgn0053013	NA	FBtr0290072
FBgn0053017	FBgn0053017	NA	FBtr0087135
FBgn0053051	FBgn0053051	NA	FBtr0075230
FBgn0053052	FBgn0053052	NA	FBtr0075229
FBgn0053057	FBgn0053057	NA	FBtr0076678
FBgn0053060	FBgn0053060	NA	FBtr0305307
FBgn0053061	FBgn0053061	NA	FBtr0310460
FBgn0053062	FBgn0053062	NA	FBtr0302886

FBgn0053082	FBgn0053082	NA	FBtr0070174
FBgn0053092	FBgn0053092	NA	FBtr0084140
FBgn0053093	FBgn0053093	NA	FBtr0084287
FBgn0053094	FBgn0053094	Synd	FBtr0083966
FBgn0053095	FBgn0053095	NA	FBtr0100204
FBgn0053096	FBgn0053096	NA	FBtr0301976
FBgn0053099	FBgn0053099	CG33099	FBtr0084288
FBgn0053100	FBgn0053100	NA	FBtr0300475
FBgn0053105	FBgn0053105	NA	FBtr0303038
FBgn0053107	FBgn0053107	NA	FBtr0084279
FBgn0053108	FBgn0053108	NA	FBtr0084504
FBgn0053109	FBgn0053109	NA	FBtr0082900
FBgn0053113	FBgn0053113	Rtnl1	FBtr0079038
FBgn0053121	FBgn0053121	NA	FBtr0079557
FBgn0053124	FBgn0053124	NA	FBtr0307522
FBgn0053125	FBgn0053125	NA	FBtr0302456
FBgn0053126	FBgn0053126	NA	FBtr0077942
FBgn0053127	FBgn0053127	NA	FBtr0078052
FBgn0053128	FBgn0053128	NA	FBtr0077930
FBgn0053129	FBgn0053129	CG33129	FBtr0080169
FBgn0053137	FBgn0053137	NA	FBtr0310497
FBgn0053138	FBgn0053138	CG33138	FBtr0087732
FBgn0053144	FBgn0053144	CG33144	FBtr0088254
FBgn0053147	FBgn0053147	CG33147	FBtr0086608
FBgn0053156	FBgn0053156	NA	FBtr0087683
FBgn0053160	FBgn0053160	NA	FBtr0073102
FBgn0053169	FBgn0053169	NA	FBtr0078549
FBgn0053170	FBgn0053170	NA	FBtr0089836
FBgn0053172	FBgn0053172	NA	FBtr0308092
FBgn0053174	FBgn0053174	NA	FBtr0073825
FBgn0053179	FBgn0053179	NA	FBtr0273450
FBgn0053180	FBgn0053180	NA	FBtr0303646
FBgn0053181	FBgn0053181	NA	FBtr0113455
FBgn0053182	FBgn0053182	NA	FBtr0310498
FBgn0053189	FBgn0053189	NA	FBtr0081940
FBgn0053191	FBgn0053191	NA	FBtr0081941
FBgn0053192	FBgn0053192	NA	FBtr0308064
FBgn0053193	FBgn0053193	sav	FBtr0084356
FBgn0053196	FBgn0053196	dp	FBtr0290006
FBgn0053199	FBgn0053199	NA	FBtr0307344
FBgn0053200	FBgn0053200	NA	FBtr0307213
FBgn0053203	FBgn0053203	NA	FBtr0085348
FBgn0053208	FBgn0053208	MICAL	FBtr0306610
FBgn0053213	FBgn0053213	NA	FBtr0085271
FBgn0053217	FBgn0053217	NA	FBtr0070045
FBgn0053218	FBgn0053218	NA	FBtr0308931
FBgn0053221	FBgn0053221	NA	FBtr0305348
FBgn0053222	FBgn0053222	NA	FBtr0300652
FBgn0053223	FBgn0053223	NA	FBtr0071239
FBgn0053225	FBgn0053225	NA	FBtr0300405
FBgn0053228	FBgn0053228	NA	FBtr0301037
FBgn0053229	FBgn0053229	NA	FBtr0072499
FBgn0053233	FBgn0053233	NA	FBtr0273190
FBgn0053234	FBgn0053234	NA	FBtr0113456
FBgn0053236	FBgn0053236	NA	FBtr0300085
FBgn0053237	FBgn0053237	NA	FBtr0300086
FBgn0053238	FBgn0053238	NA	FBtr0300087

FBgn0053239	FBgn0053239	NA	FBtr0300088
FBgn0053240	FBgn0053240	NA	FBtr0300089
FBgn0053241	FBgn0053241	NA	FBtr0300090
FBgn0053242	FBgn0053242	NA	FBtr0300091
FBgn0053243	FBgn0053243	NA	FBtr0300092
FBgn0053244	FBgn0053244	NA	FBtr0300093
FBgn0053245	FBgn0053245	NA	FBtr0300094
FBgn0053246	FBgn0053246	NA	FBtr0300095
FBgn0053247	FBgn0053247	NA	FBtr0300096
FBgn0053252	FBgn0053252	NA	FBtr0074346
FBgn0053255	FBgn0053255	NA	FBtr0303862
FBgn0053258	FBgn0053258	NA	FBtr0301030
FBgn0053260	FBgn0053260	NA	FBtr0302436
FBgn0053262	FBgn0053262	NA	FBtr0302393
FBgn0053263	FBgn0053263	NA	FBtr0075859
FBgn0053267	FBgn0053267	NA	FBtr0305649
FBgn0053269	FBgn0053269	NA	FBtr0308057
FBgn0053270	FBgn0053270	NA	FBtr0076149
FBgn0053271	FBgn0053271	NA	FBtr0076150
FBgn0053276	FBgn0053276	NA	FBtr0114530
FBgn0053278	FBgn0053278	NA	FBtr0076857
FBgn0053281	FBgn0053281	NA	FBtr0077584
FBgn0053282	FBgn0053282	NA	FBtr0113458
FBgn0053283	FBgn0053283	NA	FBtr0302444
FBgn0053284	FBgn0053284	NA	FBtr0078291
FBgn0053285	FBgn0053285	NA	FBtr0078325
FBgn0053286	FBgn0053286	NA	FBtr0113459
FBgn0053287	FBgn0053287	NA	FBtr0290073
FBgn0053288	FBgn0053288	NA	FBtr0301013
FBgn0053289	FBgn0053289	NA	FBtr0305326
FBgn0053290	FBgn0053290	NA	FBtr0078370
FBgn0053293	FBgn0053293	NA	FBtr0078824
FBgn0053294	FBgn0053294	NA	FBtr0308945
FBgn0053296	FBgn0053296	NA	FBtr0302519
FBgn0053299	FBgn0053299	NA	FBtr0300213
FBgn0053300	FBgn0053300	NA	FBtr0305657
FBgn0053301	FBgn0053301	NA	FBtr0079935
FBgn0053303	FBgn0053303	NA	FBtr0080052
FBgn0053306	FBgn0053306	NA	FBtr0080564
FBgn0053307	FBgn0053307	NA	FBtr0080565
FBgn0053308	FBgn0053308	NA	FBtr0080673
FBgn0053309	FBgn0053309	NA	FBtr0080674
FBgn0053317	FBgn0053317	NA	FBtr0089856
FBgn0053322	FBgn0053322	NA	FBtr0081455
FBgn0053325	FBgn0053325	NA	FBtr0307515
FBgn0053329	FBgn0053329	NA	FBtr0300406
FBgn0053333	FBgn0053333	NA	FBtr0083441
FBgn0053337	FBgn0053337	NA	FBtr0304612
FBgn0053339	FBgn0053339	NA	FBtr0303581
FBgn0053340	FBgn0053340	NA	FBtr0084611
FBgn0053341	FBgn0053341	NA	FBtr0084613
FBgn0053346	FBgn0053346	NA	FBtr0302527
FBgn0053348	FBgn0053348	NA	FBtr0086168
FBgn0053458	FBgn0053458	NA	FBtr0087085
FBgn0053459	FBgn0053459	NA	FBtr0089859
FBgn0053460	FBgn0053460	NA	FBtr0100642
FBgn0053461	FBgn0053461	NA	FBtr0302528

FBgn0053462	FBgn0053462	NA	FBtr0087284
FBgn0053464	FBgn0053464	NA	FBtr0087305
FBgn0053465	FBgn0053465	NA	FBtr0301048
FBgn0053468	FBgn0053468	NA	FBtr0087503
FBgn0053469	FBgn0053469	NA	FBtr0087504
FBgn0053470	FBgn0053470	NA	FBtr0087656
FBgn0053481	FBgn0053481	NA	FBtr0113463
FBgn0053483	FBgn0053483	NA	FBtr0303425
FBgn0053489	FBgn0053489	NA	FBtr0089525
FBgn0053490	FBgn0053490	NA	FBtr0089526
FBgn0053502	FBgn0053502	NA	FBtr0070055
FBgn0053505	FBgn0053505	CG33505	FBtr0091445
FBgn0053506	FBgn0053506	NA	FBtr0091446
FBgn0053509	FBgn0053509	NA	FBtr0091449
FBgn0053510	FBgn0053510	NA	FBtr0300360
FBgn0053512	FBgn0053512	NA	FBtr0300491
FBgn0053513	FBgn0053513	NA	FBtr0301844
FBgn0053514	FBgn0053514	NA	FBtr0091456
FBgn0053516	FBgn0053516	dpr3	FBtr0300671
FBgn0053519	FBgn0053519	NA	FBtr0113464
FBgn0053523	FBgn0053523	NA	FBtr0306383
FBgn0053527	FBgn0053527	NA	FBtr0091490
FBgn0053530	FBgn0053530	NA	FBtr0091496
FBgn0053531	FBgn0053531	NA	FBtr0091497
FBgn0053532	FBgn0053532	NA	FBtr0091498
FBgn0053533	FBgn0053533	NA	FBtr0091499
FBgn0053542	FBgn0053542	NA	FBtr0113465
FBgn0053543	FBgn0053543	NA	FBtr0309285
FBgn0053544	FBgn0053544	NA	FBtr0091510
FBgn0053546	FBgn0053546	NA	FBtr0091512
FBgn0053547	FBgn0053547	NA	FBtr0305015
FBgn0053554	FBgn0053554	NA	FBtr0091528
FBgn0053555	FBgn0053555	NA	FBtr0306612
FBgn0053556	FBgn0053556	NA	FBtr0113469
FBgn0053558	FBgn0053558	NA	FBtr0302034
FBgn0053626	FBgn0053626	NA	FBtr0290008
FBgn0053632	FBgn0053632	NA	FBtr0091609
FBgn0053635	FBgn0053635	NA	FBtr0091612
FBgn0053639	FBgn0053639	NA	FBtr0302909
FBgn0053641	FBgn0053641	NA	FBtr0091618
FBgn0053642	FBgn0053642	NA	FBtr0091619
FBgn0053653	FBgn0053653	NA	FBtr0091632
FBgn0053664	FBgn0053664	NA	FBtr0302417
FBgn0053665	FBgn0053665	NA	FBtr0302414
FBgn0053666	FBgn0053666	NA	FBtr0302415
FBgn0053667	FBgn0053667	NA	FBtr0302416
FBgn0053668	FBgn0053668	NA	FBtr0302418
FBgn0053669	FBgn0053669	NA	FBtr0302419
FBgn0053673	FBgn0053673	NA	FBtr0307516
FBgn0053679	FBgn0053679	NA	FBtr0091659
FBgn0053680	FBgn0053680	NA	FBtr0091660
FBgn0053696	FBgn0053696	NA	FBtr0091684
FBgn0053700	FBgn0053700	NA	FBtr0301069
FBgn0053704	FBgn0053704	NA	FBtr0091692
FBgn0053710	FBgn0053710	NA	FBtr0091698
FBgn0053721	FBgn0053721	NA	FBtr0091718
FBgn0053725	FBgn0053725	NA	FBtr0091723

FBgn0053770	FBgn0053770	NA	FBtr0290125
FBgn0053771	FBgn0053771	NA	FBtr0303713
FBgn0053772	FBgn0053772	NA	FBtr0303714
FBgn0053773	FBgn0053773	NA	FBtr0091774
FBgn0053774	FBgn0053774	NA	FBtr0091775
FBgn0053775	FBgn0053775	NA	FBtr0091776
FBgn0053914	FBgn0053914	NA	FBtr0307216
FBgn0053926	FBgn0053926	NA	FBtr0091930
FBgn0053929	FBgn0053929	NA	FBtr0303313
FBgn0053958	FBgn0053958	NA	FBtr0099994
FBgn0053960	FBgn0053960	NA	FBtr0290009
FBgn0053963	FBgn0053963	NA	FBtr0100004
FBgn0053966	FBgn0053966	NA	FBtr0100007
FBgn0053969	FBgn0053969	NA	FBtr0100010
FBgn0053977	FBgn0053977	NA	FBtr0100020
FBgn0053978	FBgn0053978	NA	FBtr0308251
FBgn0053983	FBgn0053983	NA	FBtr0100028
FBgn0053985	FBgn0053985	NA	FBtr0100030
FBgn0053986	FBgn0053986	NA	FBtr0305542
FBgn0053993	FBgn0053993	NA	FBtr0100046
FBgn0053995	FBgn0053995	NA	FBtr0100049
FBgn0053998	FBgn0053998	NA	FBtr0100053
FBgn0054001	FBgn0054001	NA	FBtr0300947
FBgn0054002	FBgn0054002	NA	FBtr0302155
FBgn0054003	FBgn0054003	NA	FBtr0100058
FBgn0054005	FBgn0054005	NA	FBtr0300216
FBgn0054006	FBgn0054006	NA	FBtr0303711
FBgn0054007	FBgn0054007	NA	FBtr0100062
FBgn0054008	FBgn0054008	NA	FBtr0100063
FBgn0054010	FBgn0054010	NA	FBtr0100065
FBgn0054011	FBgn0054011	NA	FBtr0304044
FBgn0054012	FBgn0054012	NA	FBtr0100067
FBgn0054015	FBgn0054015	NA	FBtr0100070
FBgn0054021	FBgn0054021	NA	FBtr0100076
FBgn0054022	FBgn0054022	NA	FBtr0308645
FBgn0054024	FBgn0054024	NA	FBtr0308933
FBgn0054025	FBgn0054025	NA	FBtr0290083
FBgn0054026	FBgn0054026	NA	FBtr0100081
FBgn0054027	FBgn0054027	NA	FBtr0100082
FBgn0054029	FBgn0054029	NA	FBtr0100084
FBgn0054031	FBgn0054031	NA	FBtr0308948
FBgn0054032	FBgn0054032	NA	FBtr0100087
FBgn0054033	FBgn0054033	NA	FBtr0100088
FBgn0054034	FBgn0054034	NA	FBtr0300407
FBgn0054038	FBgn0054038	NA	FBtr0299878
FBgn0054039	FBgn0054039	NA	FBtr0301014
FBgn0054040	FBgn0054040	NA	FBtr0100095
FBgn0054041	FBgn0054041	NA	FBtr0304736
FBgn0054043	FBgn0054043	NA	FBtr0300408
FBgn0054044	FBgn0054044	NA	FBtr0308934
FBgn0054045	FBgn0054045	NA	FBtr0302390
FBgn0054046	FBgn0054046	NA	FBtr0308935
FBgn0054047	FBgn0054047	NA	FBtr0308936
FBgn0054049	FBgn0054049	NA	FBtr0302372
FBgn0054051	FBgn0054051	NA	FBtr0100106
FBgn0054052	FBgn0054052	NA	FBtr0308939
FBgn0054054	FBgn0054054	NA	FBtr0100109

FBgn0054056	FBgn0054056	NA	FBtr0100833
FBgn0054057	FBgn0054057	NA	FBtr0100834
FBgn0058005	FBgn0058005	NA	FBtr0308291
FBgn0058042	FBgn0058042	NA	FBtr0300365
FBgn0058045	FBgn0058045	NA	FBtr0304740
FBgn0058064	FBgn0058064	NA	FBtr0301808
FBgn0058068	FBgn0058068	NA	FBtr0302912
FBgn0058100	FBgn0058100	NA	FBtr0302913
FBgn0058155	FBgn0058155	NA	FBtr0302251
FBgn0058160	FBgn0058160	NA	FBtr0301802
FBgn0058172	FBgn0058172	NA	FBtr0299948
FBgn0058178	FBgn0058178	NA	FBtr0113817
FBgn0058182	FBgn0058182	NA	FBtr0302347
FBgn0058188	FBgn0058188	NA	FBtr0302217
FBgn0058191	FBgn0058191	NA	FBtr0113827
FBgn0058196	FBgn0058196	NA	FBtr0113831
FBgn0058198	FBgn0058198	NA	FBtr0301809
FBgn0058204	FBgn0058204	NA	FBtr0302255
FBgn0058245	FBgn0058245	NA	FBtr0302520
FBgn0058249	FBgn0058249	NA	FBtr0302216
FBgn0058263	FBgn0058263	NA	FBtr0301780
FBgn0058354	FBgn0058354	NA	FBtr0302346
FBgn0058378	FBgn0058378	NA	FBtr0302337
FBgn0058441	FBgn0058441	NA	FBtr0302914
FBgn0058461	FBgn0058461	NA	FBtr0302353
FBgn0058469	FBgn0058469	NA	FBtr0307364
FBgn0061173	FBgn0061173	NA	FBtr0070602
FBgn0061188	FBgn0061188	NA	FBtr0302365
FBgn0061198	FBgn0061198	NA	FBtr0072194
FBgn0061200	FBgn0061200	NA	FBtr0113475
FBgn0061356	FBgn0061356	NA	FBtr0100229
FBgn0061361	FBgn0061361	NA	FBtr0091787
FBgn0061362	FBgn0061362	NA	FBtr0091786
FBgn0061435	FBgn0061435	NA	FBtr0300124
FBgn0061469	FBgn0061469	NA	FBtr0076186
FBgn0061492	FBgn0061492	NA	FBtr0301448
FBgn0061515	FBgn0061515	NA	FBtr0304673
FBgn0062412	FBgn0062412	Ctr1B	FBtr0081835
FBgn0062413	FBgn0062413	NA	FBtr0070951
FBgn0062440	FBgn0062440	NA	FBtr0086781
FBgn0062442	FBgn0062442	CG1458	FBtr0085405
FBgn0062978	FBgn0062978	NA	FBtr0303445
FBgn0063298	FBgn0063298	NA	FBtr0309817
FBgn0063368	FBgn0063368	NA	FBtr0113777
FBgn0063485	FBgn0063485	NA	FBtr0100145
FBgn0063492	FBgn0063492	NA	FBtr0086676
FBgn0063493	FBgn0063493	NA	FBtr0086675
FBgn0063494	FBgn0063494	NA	FBtr0086674
FBgn0063495	FBgn0063495	NA	FBtr0086673
FBgn0063496	FBgn0063496	NA	FBtr0086672
FBgn0063497	FBgn0063497	NA	FBtr0086671
FBgn0063498	FBgn0063498	NA	FBtr0086670
FBgn0063499	FBgn0063499	NA	FBtr0086726
FBgn0063649	FBgn0063649	NA	FBtr0309302
FBgn0063667	FBgn0063667	NA	FBtr0273416
FBgn0063670	FBgn0063670	NA	FBtr0113841
FBgn0063923	FBgn0063923	NA	FBtr0072493

FBgn0063924	FBgn0063924	NA	FBtr0304146
FBgn0064115	FBgn0064115	NA	FBtr0091626
FBgn0064119	FBgn0064119	NA	FBtr0301700
FBgn0064122	FBgn0064122	NA	FBtr0091672
FBgn0064126	FBgn0064126	NA	FBtr0091720
FBgn0064225	FBgn0064225	NA	FBtr0111132
FBgn0064227	FBgn0064227	NA	FBtr0073154
FBgn0064237	FBgn0064237	NA	FBtr0086668
FBgn0064766	FBgn0064766	NA	FBtr0076165
FBgn0064912	FBgn0064912	NA	FBtr0300075
FBgn0065032	FBgn0065032	NA	FBtr0113478
FBgn0065035	FBgn0065035	NA	FBtr0074168
FBgn0065098	FBgn0065098	NA	FBtr0091662
FBgn0065099	FBgn0065099	NA	FBtr0091929
FBgn0066084	FBgn0066084	NA	FBtr0100231
FBgn0066101	FBgn0066101	LpR1	FBtr0301470
FBgn0066114	FBgn0066114	NA	FBtr0070644
FBgn0066292	FBgn0066292	NA	FBtr0086191
FBgn0067052	FBgn0067052	NA	FBtr0071490
FBgn0067102	FBgn0067102	NA	FBtr0071755
FBgn0067311	FBgn0067311	NA	FBtr0081454
FBgn0067317	FBgn0067317	NA	FBtr0290217
FBgn0067318	FBgn0067318	NA	FBtr0113895
FBgn0067622	FBgn0067622	NA	FBtr0091657
FBgn0067628	FBgn0067628	NA	FBtr0082998
FBgn0067629	FBgn0067629	CG33332	FBtr0082999
FBgn0067779	FBgn0067779	NA	FBtr0078100
FBgn0067782	FBgn0067782	NA	FBtr0090034
FBgn0067783	FBgn0067783	NA	FBtr0089480
FBgn0067861	FBgn0067861	NA	FBtr0089604
FBgn0067864	FBgn0067864	Patj	FBtr0072779
FBgn0067903	FBgn0067903	NA	FBtr0091694
FBgn0069056	FBgn0069056	NA	FBtr0308585
FBgn0069242	FBgn0069242	eca	FBtr0082098
FBgn0069913	FBgn0069913	NA	FBtr0301624
FBgn0069923	FBgn0069923	NA	FBtr0301812
FBgn0069947	FBgn0069947	NA	FBtr0301119
FBgn0069969	FBgn0069969	NA	FBtr0302340
FBgn0069972	FBgn0069972	NA	FBtr0111187
FBgn0069973	FBgn0069973	NA	FBtr0111189
FBgn0069977	FBgn0069977	NA	FBtr0301118
FBgn0082582	FBgn0082582	tmod	FBtr0306687
FBgn0082598	FBgn0082598	NA	FBtr0302544
FBgn0082831	FBgn0082831	NA	FBtr0082586
FBgn0082919	FBgn0082919	NA	FBtr0113511
FBgn0082922	FBgn0082922	NA	FBtr0113512
FBgn0082923	FBgn0082923	NA	FBtr0113586
FBgn0082924	FBgn0082924	NA	FBtr0113562
FBgn0082925	FBgn0082925	NA	FBtr0113530
FBgn0082926	FBgn0082926	NA	FBtr0113504
FBgn0082927	FBgn0082927	NA	FBtr0113498
FBgn0082928	FBgn0082928	NA	FBtr0113495
FBgn0082929	FBgn0082929	NA	FBtr0113510
FBgn0082930	FBgn0082930	NA	FBtr0113509
FBgn0082931	FBgn0082931	NA	FBtr0113508
FBgn0082932	FBgn0082932	NA	FBtr0113534
FBgn0082933	FBgn0082933	NA	FBtr0113533

FBgn0082934	FBgn0082934	NA	FBtr0113532
FBgn0082935	FBgn0082935	NA	FBtr0113531
FBgn0082936	FBgn0082936	NA	FBtr0113558
FBgn0082937	FBgn0082937	NA	FBtr0113557
FBgn0082938	FBgn0082938	NA	FBtr0113556
FBgn0082939	FBgn0082939	NA	FBtr0113503
FBgn0082940	FBgn0082940	NA	FBtr0113502
FBgn0082941	FBgn0082941	NA	FBtr0113507
FBgn0082942	FBgn0082942	NA	FBtr0113497
FBgn0082943	FBgn0082943	NA	FBtr0113496
FBgn0082944	FBgn0082944	NA	FBtr0113603
FBgn0082945	FBgn0082945	NA	FBtr0113604
FBgn0082946	FBgn0082946	NA	FBtr0113605
FBgn0082947	FBgn0082947	NA	FBtr0113615
FBgn0082948	FBgn0082948	NA	FBtr0113614
FBgn0082949	FBgn0082949	NA	FBtr0113613
FBgn0082950	FBgn0082950	NA	FBtr0113505
FBgn0082951	FBgn0082951	NA	FBtr0113501
FBgn0082952	FBgn0082952	NA	FBtr0113506
FBgn0082953	FBgn0082953	NA	FBtr0113609
FBgn0082954	FBgn0082954	NA	FBtr0113580
FBgn0082957	FBgn0082957	NA	FBtr0113592
FBgn0082958	FBgn0082958	NA	FBtr0113590
FBgn0082959	FBgn0082959	NA	FBtr0113589
FBgn0082960	FBgn0082960	NA	FBtr0113587
FBgn0082961	FBgn0082961	NA	FBtr0113576
FBgn0082962	FBgn0082962	NA	FBtr0113577
FBgn0082963	FBgn0082963	NA	FBtr0113518
FBgn0082966	FBgn0082966	NA	FBtr0113612
FBgn0082967	FBgn0082967	NA	FBtr0113611
FBgn0082968	FBgn0082968	NA	FBtr0113523
FBgn0082969	FBgn0082969	NA	FBtr0113521
FBgn0082970	FBgn0082970	NA	FBtr0113519
FBgn0082971	FBgn0082971	NA	FBtr0113517
FBgn0082972	FBgn0082972	NA	FBtr0113516
FBgn0082973	FBgn0082973	NA	FBtr0113601
FBgn0082974	FBgn0082974	NA	FBtr0113593
FBgn0082975	FBgn0082975	NA	FBtr0113591
FBgn0082976	FBgn0082976	NA	FBtr0113588
FBgn0082978	FBgn0082978	NA	FBtr0113578
FBgn0082979	FBgn0082979	NA	FBtr0113579
FBgn0082980	FBgn0082980	NA	FBtr0309749
FBgn0082981	FBgn0082981	NA	FBtr0113494
FBgn0082983	FBgn0082983	NA	FBtr0113598
FBgn0082986	FBgn0082986	NA	FBtr0113585
FBgn0082987	FBgn0082987	NA	FBtr0113515
FBgn0082988	FBgn0082988	NA	FBtr0113581
FBgn0082989	FBgn0082989	NA	FBtr0113582
FBgn0082990	FBgn0082990	NA	FBtr0113537
FBgn0082993	FBgn0082993	NA	FBtr0113499
FBgn0082994	FBgn0082994	NA	FBtr0113607
FBgn0082995	FBgn0082995	NA	FBtr0113608
FBgn0082997	FBgn0082997	NA	FBtr0113561
FBgn0082998	FBgn0082998	NA	FBtr0113563
FBgn0082999	FBgn0082999	NA	FBtr0113564
FBgn0083000	FBgn0083000	NA	FBtr0113565
FBgn0083001	FBgn0083001	NA	FBtr0113566

FBgn0083003	FBgn0083003	NA	FBtr0113529
FBgn0083004	FBgn0083004	NA	FBtr0113528
FBgn0083005	FBgn0083005	NA	FBtr0113527
FBgn0083006	FBgn0083006	NA	FBtr0113500
FBgn0083007	FBgn0083007	NA	FBtr0113568
FBgn0083008	FBgn0083008	NA	FBtr0113570
FBgn0083009	FBgn0083009	NA	FBtr0113572
FBgn0083010	FBgn0083010	NA	FBtr0113573
FBgn0083011	FBgn0083011	NA	FBtr0113574
FBgn0083012	FBgn0083012	NA	FBtr0113575
FBgn0083013	FBgn0083013	NA	FBtr0113560
FBgn0083014	FBgn0083014	NA	FBtr0113599
FBgn0083015	FBgn0083015	NA	FBtr0113610
FBgn0083016	FBgn0083016	NA	FBtr0113524
FBgn0083017	FBgn0083017	NA	FBtr0113522
FBgn0083018	FBgn0083018	NA	FBtr0113520
FBgn0083020	FBgn0083020	NA	FBtr0113554
FBgn0083021	FBgn0083021	NA	FBtr0113552
FBgn0083022	FBgn0083022	NA	FBtr0113550
FBgn0083023	FBgn0083023	NA	FBtr0113547
FBgn0083024	FBgn0083024	NA	FBtr0113545
FBgn0083025	FBgn0083025	NA	FBtr0113543
FBgn0083026	FBgn0083026	NA	FBtr0113540
FBgn0083027	FBgn0083027	NA	FBtr0113559
FBgn0083028	FBgn0083028	NA	FBtr0113538
FBgn0083029	FBgn0083029	NA	FBtr0113555
FBgn0083030	FBgn0083030	NA	FBtr0113553
FBgn0083031	FBgn0083031	NA	FBtr0113551
FBgn0083032	FBgn0083032	NA	FBtr0113549
FBgn0083033	FBgn0083033	NA	FBtr0113548
FBgn0083034	FBgn0083034	NA	FBtr0113546
FBgn0083035	FBgn0083035	NA	FBtr0113544
FBgn0083036	FBgn0083036	NA	FBtr0113542
FBgn0083037	FBgn0083037	NA	FBtr0113541
FBgn0083038	FBgn0083038	NA	FBtr0113539
FBgn0083039	FBgn0083039	NA	FBtr0113583
FBgn0083040	FBgn0083040	NA	FBtr0113567
FBgn0083041	FBgn0083041	NA	FBtr0113569
FBgn0083042	FBgn0083042	NA	FBtr0113571
FBgn0083044	FBgn0083044	NA	FBtr0113606
FBgn0083045	FBgn0083045	NA	FBtr0113526
FBgn0083046	FBgn0083046	NA	FBtr0113525
FBgn0083047	FBgn0083047	NA	FBtr0113594
FBgn0083048	FBgn0083048	NA	FBtr0113595
FBgn0083049	FBgn0083049	NA	FBtr0113596
FBgn0083050	FBgn0083050	NA	FBtr0113597
FBgn0083053	FBgn0083053	NA	FBtr0113514
FBgn0083054	FBgn0083054	NA	FBtr0113513
FBgn0083055	FBgn0083055	NA	FBtr0113584
FBgn0083057	FBgn0083057	NA	FBtr0113600
FBgn0083058	FBgn0083058	NA	FBtr0113602
FBgn0083077	FBgn0083077	NA	FBtr0304913
FBgn0083141	FBgn0083141	NA	FBtr0079470
FBgn0083167	FBgn0083167	NA	FBtr0071449
FBgn0083228	FBgn0083228	NA	FBtr0301628
FBgn0083919	FBgn0083919	NA	FBtr0100388
FBgn0083936	FBgn0083936	NA	FBtr0110768

FBgn0083938	FBgn0083938	NA	FBtr0110769
FBgn0083940	FBgn0083940	NA	FBtr0110783
FBgn0083941	FBgn0083941	NA	FBtr0110784
FBgn0083942	FBgn0083942	NA	FBtr0110785
FBgn0083943	FBgn0083943	NA	FBtr0110787
FBgn0083945	FBgn0083945	NA	FBtr0300423
FBgn0083946	FBgn0083946	NA	FBtr0110797
FBgn0083949	FBgn0083949	NA	FBtr0110806
FBgn0083950	FBgn0083950	NA	FBtr0110808
FBgn0083951	FBgn0083951	NA	FBtr0110813
FBgn0083952	FBgn0083952	NA	FBtr0110816
FBgn0083953	FBgn0083953	NA	FBtr0110817
FBgn0083956	FBgn0083956	NA	FBtr0110827
FBgn0083959	FBgn0083959	NA	FBtr0114579
FBgn0083960	FBgn0083960	NA	FBtr0110838
FBgn0083961	FBgn0083961	NA	FBtr0110839
FBgn0083962	FBgn0083962	NA	FBtr0290010
FBgn0083963	FBgn0083963	NA	FBtr0301072
FBgn0083965	FBgn0083965	NA	FBtr0110870
FBgn0083966	FBgn0083966	NA	FBtr0110871
FBgn0083967	FBgn0083967	NA	FBtr0110878
FBgn0083968	FBgn0083968	NA	FBtr0110879
FBgn0083969	FBgn0083969	NA	FBtr0301073
FBgn0083970	FBgn0083970	NA	FBtr0110894
FBgn0083971	FBgn0083971	NA	FBtr0308891
FBgn0083972	FBgn0083972	NA	FBtr0110944
FBgn0083973	FBgn0083973	NA	FBtr0110947
FBgn0083974	FBgn0083974	NA	FBtr0110954
FBgn0083975	FBgn0083975	NA	FBtr0301680
FBgn0083976	FBgn0083976	NA	FBtr0110975
FBgn0083977	FBgn0083977	NA	FBtr0301077
FBgn0083978	FBgn0083978	CG17672	FBtr0110982
FBgn0083979	FBgn0083979	NA	FBtr0113487
FBgn0083980	FBgn0083980	NA	FBtr0110988
FBgn0083981	FBgn0083981	NA	FBtr0308028
FBgn0083983	FBgn0083983	NA	FBtr0111011
FBgn0083984	FBgn0083984	NA	FBtr0301007
FBgn0083985	FBgn0083985	NA	FBtr0111020
FBgn0083986	FBgn0083986	NA	FBtr0301004
FBgn0083987	FBgn0083987	NA	FBtr0111039
FBgn0083988	FBgn0083988	NA	FBtr0111041
FBgn0083989	FBgn0083989	NA	FBtr0111042
FBgn0083990	FBgn0083990	NA	FBtr0111045
FBgn0083991	FBgn0083991	NA	FBtr0111047
FBgn0084008	FBgn0084008	NA	FBtr0308117
FBgn0084049	FBgn0084049	NA	FBtr0111304
FBgn0084245	FBgn0084245	NA	FBtr0113490
FBgn0084688	FBgn0084688	NA	FBtr0113491
FBgn0085056	FBgn0085056	NA	FBtr0113492
FBgn0085188	FBgn0085188	NA	FBtr0112350
FBgn0085190	FBgn0085190	NA	FBtr0301006
FBgn0085191	FBgn0085191	NA	FBtr0302420
FBgn0085192	FBgn0085192	NA	FBtr0112354
FBgn0085193	FBgn0085193	NA	FBtr0112355
FBgn0085194	FBgn0085194	NA	FBtr0112356
FBgn0085195	FBgn0085195	NA	FBtr0112357
FBgn0085196	FBgn0085196	NA	FBtr0112358

FBgn0085197	FBgn0085197	NA	FBtr0112359
FBgn0085198	FBgn0085198	NA	FBtr0112360
FBgn0085199	FBgn0085199	NA	FBtr0112361
FBgn0085200	FBgn0085200	NA	FBtr0305066
FBgn0085201	FBgn0085201	NA	FBtr0112363
FBgn0085202	FBgn0085202	NA	FBtr0112364
FBgn0085203	FBgn0085203	NA	FBtr0112365
FBgn0085204	FBgn0085204	NA	FBtr0112366
FBgn0085205	FBgn0085205	NA	FBtr0112367
FBgn0085206	FBgn0085206	NA	FBtr0112368
FBgn0085207	FBgn0085207	NA	FBtr0112370
FBgn0085208	FBgn0085208	NA	FBtr0299809
FBgn0085209	FBgn0085209	NA	FBtr0300048
FBgn0085210	FBgn0085210	NA	FBtr0112374
FBgn0085211	FBgn0085211	NA	FBtr0112375
FBgn0085212	FBgn0085212	NA	FBtr0112376
FBgn0085213	FBgn0085213	NA	FBtr0112377
FBgn0085214	FBgn0085214	NA	FBtr0112378
FBgn0085215	FBgn0085215	NA	FBtr0112379
FBgn0085216	FBgn0085216	NA	FBtr0112380
FBgn0085217	FBgn0085217	NA	FBtr0112381
FBgn0085218	FBgn0085218	NA	FBtr0112382
FBgn0085219	FBgn0085219	NA	FBtr0112383
FBgn0085220	FBgn0085220	NA	FBtr0112384
FBgn0085221	FBgn0085221	NA	FBtr0112385
FBgn0085222	FBgn0085222	NA	FBtr0112386
FBgn0085223	FBgn0085223	NA	FBtr0112387
FBgn0085224	FBgn0085224	NA	FBtr0112388
FBgn0085225	FBgn0085225	NA	FBtr0112389
FBgn0085227	FBgn0085227	NA	FBtr0112391
FBgn0085228	FBgn0085228	NA	FBtr0112392
FBgn0085229	FBgn0085229	NA	FBtr0112393
FBgn0085230	FBgn0085230	NA	FBtr0112394
FBgn0085231	FBgn0085231	NA	FBtr0112395
FBgn0085232	FBgn0085232	NA	FBtr0112396
FBgn0085233	FBgn0085233	NA	FBtr0112397
FBgn0085234	FBgn0085234	NA	FBtr0112398
FBgn0085235	FBgn0085235	NA	FBtr0112399
FBgn0085236	FBgn0085236	NA	FBtr0112400
FBgn0085237	FBgn0085237	NA	FBtr0112401
FBgn0085239	FBgn0085239	NA	FBtr0112403
FBgn0085240	FBgn0085240	NA	FBtr0112404
FBgn0085241	FBgn0085241	NA	FBtr0302826
FBgn0085242	FBgn0085242	NA	FBtr0112406
FBgn0085243	FBgn0085243	NA	FBtr0112407
FBgn0085244	FBgn0085244	NA	FBtr0112408
FBgn0085245	FBgn0085245	NA	FBtr0112409
FBgn0085246	FBgn0085246	NA	FBtr0112410
FBgn0085248	FBgn0085248	NA	FBtr0112412
FBgn0085249	FBgn0085249	NA	FBtr0112413
FBgn0085250	FBgn0085250	NA	FBtr0299834
FBgn0085252	FBgn0085252	NA	FBtr0112416
FBgn0085253	FBgn0085253	NA	FBtr0112417
FBgn0085254	FBgn0085254	NA	FBtr0112418
FBgn0085256	FBgn0085256	NA	FBtr0112420
FBgn0085257	FBgn0085257	NA	FBtr0112421
FBgn0085259	FBgn0085259	NA	FBtr0112423

FBgn0085260	FBgn0085260	NA	FBtr0112424
FBgn0085261	FBgn0085261	NA	FBtr0112426
FBgn0085263	FBgn0085263	NA	FBtr0112428
FBgn0085264	FBgn0085264	NA	FBtr0300636
FBgn0085265	FBgn0085265	NA	FBtr0301011
FBgn0085267	FBgn0085267	NA	FBtr0112432
FBgn0085271	FBgn0085271	NA	FBtr0112436
FBgn0085272	FBgn0085272	NA	FBtr0112437
FBgn0085273	FBgn0085273	NA	FBtr0112438
FBgn0085274	FBgn0085274	NA	FBtr0302580
FBgn0085275	FBgn0085275	NA	FBtr0112440
FBgn0085276	FBgn0085276	NA	FBtr0112441
FBgn0085277	FBgn0085277	NA	FBtr0302929
FBgn0085278	FBgn0085278	NA	FBtr0112443
FBgn0085279	FBgn0085279	NA	FBtr0112444
FBgn0085280	FBgn0085280	NA	FBtr0301012
FBgn0085281	FBgn0085281	NA	FBtr0112446
FBgn0085282	FBgn0085282	NA	FBtr0112447
FBgn0085283	FBgn0085283	NA	FBtr0112448
FBgn0085284	FBgn0085284	NA	FBtr0112449
FBgn0085285	FBgn0085285	NA	FBtr0302318
FBgn0085287	FBgn0085287	NA	FBtr0112452
FBgn0085288	FBgn0085288	NA	FBtr0300030
FBgn0085289	FBgn0085289	NA	FBtr0305578
FBgn0085290	FBgn0085290	NA	FBtr0112456
FBgn0085291	FBgn0085291	NA	FBtr0112457
FBgn0085292	FBgn0085292	NA	FBtr0112458
FBgn0085293	FBgn0085293	NA	FBtr0299835
FBgn0085294	FBgn0085294	NA	FBtr0112460
FBgn0085295	FBgn0085295	NA	FBtr0112461
FBgn0085296	FBgn0085296	NA	FBtr0112462
FBgn0085297	FBgn0085297	NA	FBtr0112463
FBgn0085298	FBgn0085298	NA	FBtr0112464
FBgn0085300	FBgn0085300	NA	FBtr0112466
FBgn0085302	FBgn0085302	NA	FBtr0112468
FBgn0085303	FBgn0085303	NA	FBtr0112469
FBgn0085304	FBgn0085304	NA	FBtr0112470
FBgn0085305	FBgn0085305	NA	FBtr0112471
FBgn0085306	FBgn0085306	NA	FBtr0302422
FBgn0085307	FBgn0085307	NA	FBtr0112473
FBgn0085308	FBgn0085308	NA	FBtr0112474
FBgn0085309	FBgn0085309	NA	FBtr0112476
FBgn0085310	FBgn0085310	NA	FBtr0112477
FBgn0085311	FBgn0085311	NA	FBtr0112478
FBgn0085312	FBgn0085312	NA	FBtr0112479
FBgn0085313	FBgn0085313	NA	FBtr0112480
FBgn0085314	FBgn0085314	NA	FBtr0112481
FBgn0085315	FBgn0085315	NA	FBtr0112482
FBgn0085316	FBgn0085316	NA	FBtr0112483
FBgn0085317	FBgn0085317	NA	FBtr0300623
FBgn0085318	FBgn0085318	NA	FBtr0307053
FBgn0085319	FBgn0085319	NA	FBtr0299531
FBgn0085320	FBgn0085320	NA	FBtr0112487
FBgn0085321	FBgn0085321	NA	FBtr0112488
FBgn0085322	FBgn0085322	NA	FBtr0112489
FBgn0085323	FBgn0085323	NA	FBtr0112490
FBgn0085324	FBgn0085324	NA	FBtr0112491

FBgn0085325	FBgn0085325	NA	FBtr0112492
FBgn0085326	FBgn0085326	NA	FBtr0112493
FBgn0085327	FBgn0085327	NA	FBtr0112494
FBgn0085328	FBgn0085328	NA	FBtr0112495
FBgn0085329	FBgn0085329	NA	FBtr0305269
FBgn0085330	FBgn0085330	NA	FBtr0112497
FBgn0085331	FBgn0085331	NA	FBtr0301078
FBgn0085332	FBgn0085332	NA	FBtr0112499
FBgn0085333	FBgn0085333	NA	FBtr0112500
FBgn0085334	FBgn0085334	NA	FBtr0112501
FBgn0085335	FBgn0085335	NA	FBtr0112502
FBgn0085336	FBgn0085336	NA	FBtr0301079
FBgn0085337	FBgn0085337	NA	FBtr0112504
FBgn0085339	FBgn0085339	NA	FBtr0112506
FBgn0085340	FBgn0085340	NA	FBtr0112509
FBgn0085341	FBgn0085341	NA	FBtr0112510
FBgn0085342	FBgn0085342	NA	FBtr0112511
FBgn0085343	FBgn0085343	NA	FBtr0112512
FBgn0085344	FBgn0085344	NA	FBtr0300287
FBgn0085345	FBgn0085345	NA	FBtr0112514
FBgn0085346	FBgn0085346	NA	FBtr0112516
FBgn0085347	FBgn0085347	NA	FBtr0306214
FBgn0085349	FBgn0085349	NA	FBtr0112522
FBgn0085350	FBgn0085350	NA	FBtr0112523
FBgn0085351	FBgn0085351	NA	FBtr0112524
FBgn0085352	FBgn0085352	NA	FBtr0112525
FBgn0085353	FBgn0085353	NA	FBtr0112526
FBgn0085354	FBgn0085354	NA	FBtr0112527
FBgn0085355	FBgn0085355	NA	FBtr0112528
FBgn0085356	FBgn0085356	NA	FBtr0112529
FBgn0085357	FBgn0085357	NA	FBtr0112530
FBgn0085358	FBgn0085358	NA	FBtr0112531
FBgn0085359	FBgn0085359	NA	FBtr0112532
FBgn0085360	FBgn0085360	NA	FBtr0301576
FBgn0085361	FBgn0085361	NA	FBtr0112534
FBgn0085362	FBgn0085362	NA	FBtr0112535
FBgn0085363	FBgn0085363	NA	FBtr0112536
FBgn0085364	FBgn0085364	NA	FBtr0307366
FBgn0085365	FBgn0085365	NA	FBtr0112538
FBgn0085366	FBgn0085366	NA	FBtr0112539
FBgn0085367	FBgn0085367	NA	FBtr0303204
FBgn0085369	FBgn0085369	CG2808	FBtr0302391
FBgn0085370	FBgn0085370	Pde11	FBtr0112544
FBgn0085371	FBgn0085371	NA	FBtr0300031
FBgn0085374	FBgn0085374	NA	FBtr0112553
FBgn0085375	FBgn0085375	NA	FBtr0307299
FBgn0085376	FBgn0085376	NA	FBtr0290046
FBgn0085377	FBgn0085377	NA	FBtr0112557
FBgn0085378	FBgn0085378	NA	FBtr0303025
FBgn0085379	FBgn0085379	NA	FBtr0112560
FBgn0085380	FBgn0085380	NA	FBtr0112561
FBgn0085382	FBgn0085382	NA	FBtr0305292
FBgn0085383	FBgn0085383	NA	FBtr0304917
FBgn0085384	FBgn0085384	NA	FBtr0306735
FBgn0085385	FBgn0085385	NA	FBtr0112566
FBgn0085386	FBgn0085386	NA	FBtr0303867
FBgn0085387	FBgn0085387	NA	FBtr0309370

FBgn0085388	FBgn0085388	IP3K2	FBtr0112575
FBgn0085390	FBgn0085390	NA	FBtr0308589
FBgn0085391	FBgn0085391	NA	FBtr0301397
FBgn0085395	FBgn0085395	NA	FBtr0308029
FBgn0085396	FBgn0085396	NA	FBtr0112592
FBgn0085397	FBgn0085397	NA	FBtr0112593
FBgn0085398	FBgn0085398	NA	FBtr0112594
FBgn0085399	FBgn0085399	CG33227	FBtr0299544
FBgn0085400	FBgn0085400	NA	FBtr0112596
FBgn0085401	FBgn0085401	NA	FBtr0273218
FBgn0085403	FBgn0085403	Rapgap1	FBtr0112603
FBgn0085404	FBgn0085404	NA	FBtr0302319
FBgn0085405	FBgn0085405	NA	FBtr0112608
FBgn0085407	FBgn0085407	Pvf3	FBtr0112611
FBgn0085408	FBgn0085408	CG8603	FBtr0112614
FBgn0085409	FBgn0085409	CG31640	FBtr0112617
FBgn0085410	FBgn0085410	NA	FBtr0112618
FBgn0085411	FBgn0085411	NA	FBtr0112620
FBgn0085412	FBgn0085412	CG9322	FBtr0112621
FBgn0085413	FBgn0085413	NA	FBtr0304918
FBgn0085414	FBgn0085414	NA	FBtr0300553
FBgn0085415	FBgn0085415	NA	FBtr0112627
FBgn0085417	FBgn0085417	NA	FBtr0112629
FBgn0085418	FBgn0085418	NA	FBtr0112630
FBgn0085419	FBgn0085419	NA	FBtr0112633
FBgn0085420	FBgn0085420	NA	FBtr0112634
FBgn0085421	FBgn0085421	NA	FBtr0329849
FBgn0085422	FBgn0085422	NA	FBtr0112637
FBgn0085423	FBgn0085423	NA	FBtr0112641
FBgn0085424	FBgn0085424	nub	FBtr0112642
FBgn0085425	FBgn0085425	NA	FBtr0112645
FBgn0085426	FBgn0085426	NA	FBtr0305983
FBgn0085427	FBgn0085427	NA	FBtr0303892
FBgn0085428	FBgn0085428	NA	FBtr0112650
FBgn0085429	FBgn0085429	NA	FBtr0290126
FBgn0085430	FBgn0085430	NA	FBtr0112653
FBgn0085431	FBgn0085431	NA	FBtr0112654
FBgn0085432	FBgn0085432	NA	FBtr0309803
FBgn0085433	FBgn0085433	NA	FBtr0112667
FBgn0085434	FBgn0085434	NA	FBtr0308342
FBgn0085435	FBgn0085435	NA	FBtr0112671
FBgn0085436	FBgn0085436	NA	FBtr0304957
FBgn0085437	FBgn0085437	NA	FBtr0112675
FBgn0085438	FBgn0085438	NA	FBtr0112676
FBgn0085440	FBgn0085440	NA	FBtr0300364
FBgn0085442	FBgn0085442	NA	FBtr0305056
FBgn0085443	FBgn0085443	NA	FBtr0310439
FBgn0085444	FBgn0085444	NA	FBtr0112691
FBgn0085446	FBgn0085446	CG3960	FBtr0112707
FBgn0085447	FBgn0085447	NA	FBtr0112711
FBgn0085448	FBgn0085448	NA	FBtr0273220
FBgn0085449	FBgn0085449	NA	FBtr0112716
FBgn0085450	FBgn0085450	NA	FBtr0112717
FBgn0085451	FBgn0085451	NA	FBtr0310440
FBgn0085452	FBgn0085452	NA	FBtr0301854
FBgn0085453	FBgn0085453	NA	FBtr0112725
FBgn0085454	FBgn0085454	NA	FBtr0112726

FBgn0085455	FBgn0085455	NA	FBtr0112727
FBgn0085456	FBgn0085456	NA	FBtr0303231
FBgn0085457	FBgn0085457	NA	FBtr0112729
FBgn0085458	FBgn0085458	NA	FBtr0112730
FBgn0085459	FBgn0085459	NA	FBtr0112731
FBgn0085460	FBgn0085460	NA	FBtr0112732
FBgn0085461	FBgn0085461	NA	FBtr0112733
FBgn0085462	FBgn0085462	NA	FBtr0112734
FBgn0085464	FBgn0085464	NA	FBtr0112736
FBgn0085465	FBgn0085465	NA	FBtr0112737
FBgn0085466	FBgn0085466	NA	FBtr0112738
FBgn0085468	FBgn0085468	NA	FBtr0112741
FBgn0085469	FBgn0085469	NA	FBtr0079695
FBgn0085470	FBgn0085470	NA	FBtr0112744
FBgn0085471	FBgn0085471	NA	FBtr0112746
FBgn0085472	FBgn0085472	NA	FBtr0112747
FBgn0085473	FBgn0085473	NA	FBtr0112748
FBgn0085474	FBgn0085474	NA	FBtr0112749
FBgn0085475	FBgn0085475	NA	FBtr0112750
FBgn0085476	FBgn0085476	NA	FBtr0112751
FBgn0085477	FBgn0085477	NA	FBtr0112752
FBgn0085478	FBgn0085478	NA	FBtr0308591
FBgn0085479	FBgn0085479	NA	FBtr0112756
FBgn0085480	FBgn0085480	NA	FBtr0308640
FBgn0085481	FBgn0085481	NA	FBtr0299517
FBgn0085482	FBgn0085482	NA	FBtr0299518
FBgn0085483	FBgn0085483	NA	FBtr0112760
FBgn0085484	FBgn0085484	NA	FBtr0112761
FBgn0085485	FBgn0085485	NA	FBtr0112762
FBgn0085486	FBgn0085486	NA	FBtr0112763
FBgn0085487	FBgn0085487	NA	FBtr0112764
FBgn0085488	FBgn0085488	NA	FBtr0112765
FBgn0085489	FBgn0085489	NA	FBtr0112766
FBgn0085491	FBgn0085491	NA	FBtr0112768
FBgn0085494	FBgn0085494	NA	FBtr0308947
FBgn0085496	FBgn0085496	NA	FBtr0302523
FBgn0085498	FBgn0085498	NA	FBtr0302524
FBgn0085503	FBgn0085503	NA	FBtr0302234
FBgn0085505	FBgn0085505	NA	FBtr0302349
FBgn0085506	FBgn0085506	NA	FBtr0302344
FBgn0085511	FBgn0085511	NA	FBtr0304147
FBgn0085512	FBgn0085512	NA	FBtr0310523
FBgn0085523	FBgn0085523	NA	FBtr0302336
FBgn0085538	FBgn0085538	NA	FBtr0302237
FBgn0085539	FBgn0085539	NA	FBtr0302345
FBgn0085540	FBgn0085540	NA	FBtr0302352
FBgn0085541	FBgn0085541	NA	FBtr0302240
FBgn0085546	FBgn0085546	NA	FBtr0302238
FBgn0085550	FBgn0085550	NA	FBtr0302343
FBgn0085556	FBgn0085556	NA	FBtr0302525
FBgn0085569	FBgn0085569	NA	FBtr0301782
FBgn0085570	FBgn0085570	NA	FBtr0302915
FBgn0085597	FBgn0085597	NA	FBtr0302257
FBgn0085599	FBgn0085599	NA	FBtr0301798
FBgn0085625	FBgn0085625	NA	FBtr0301120
FBgn0085633	FBgn0085633	NA	FBtr0302235
FBgn0085638	FBgn0085638	NA	FBtr0301790

FBgn0085663	FBgn0085663	NA	FBtr0302253
FBgn0085664	FBgn0085664	NA	FBtr0304148
FBgn0085670	FBgn0085670	NA	FBtr0302258
FBgn0085673	FBgn0085673	NA	FBtr0302348
FBgn0085692	FBgn0085692	NA	FBtr0114141
FBgn0085732	FBgn0085732	NA	FBtr0302397
FBgn0085736	FBgn0085736	NA	FBtr0302233
FBgn0085781	FBgn0085781	NA	FBtr0302220
FBgn0085784	FBgn0085784	NA	FBtr0114236
FBgn0085786	FBgn0085786	NA	FBtr0302232
FBgn0085787	FBgn0085787	NA	FBtr0302230
FBgn0085788	FBgn0085788	NA	FBtr0114241
FBgn0085789	FBgn0085789	NA	FBtr0114242
FBgn0085790	FBgn0085790	NA	FBtr0114243
FBgn0085791	FBgn0085791	NA	FBtr0114244
FBgn0085792	FBgn0085792	NA	FBtr0114245
FBgn0085810	FBgn0085810	NA	FBtr0114264
FBgn0085831	FBgn0085831	NA	FBtr0114289
FBgn0086021	FBgn0086021	NA	FBtr0114297
FBgn0086022	FBgn0086022	NA	FBtr0114298
FBgn0086023	FBgn0086023	NA	FBtr0114299
FBgn0086024	FBgn0086024	NA	FBtr0114300
FBgn0086025	FBgn0086025	NA	FBtr0114301
FBgn0086026	FBgn0086026	NA	FBtr0114302
FBgn0086027	FBgn0086027	NA	FBtr0114303
FBgn0086028	FBgn0086028	NA	FBtr0114304
FBgn0086029	FBgn0086029	NA	FBtr0114305
FBgn0086030	FBgn0086030	NA	FBtr0114306
FBgn0086031	FBgn0086031	NA	FBtr0114307
FBgn0086032	FBgn0086032	NA	FBtr0114308
FBgn0086033	FBgn0086033	NA	FBtr0114309
FBgn0086034	FBgn0086034	NA	FBtr0114310
FBgn0086035	FBgn0086035	NA	FBtr0114311
FBgn0086036	FBgn0086036	NA	FBtr0114312
FBgn0086037	FBgn0086037	NA	FBtr0114313
FBgn0086038	FBgn0086038	NA	FBtr0114314
FBgn0086039	FBgn0086039	NA	FBtr0114315
FBgn0086040	FBgn0086040	NA	FBtr0114316
FBgn0086041	FBgn0086041	NA	FBtr0114317
FBgn0086042	FBgn0086042	NA	FBtr0114318
FBgn0086043	FBgn0086043	NA	FBtr0114319
FBgn0086044	FBgn0086044	NA	FBtr0114320
FBgn0086045	FBgn0086045	NA	FBtr0114321
FBgn0086046	FBgn0086046	NA	FBtr0114322
FBgn0086047	FBgn0086047	NA	FBtr0114323
FBgn0086048	FBgn0086048	NA	FBtr0114324
FBgn0086049	FBgn0086049	NA	FBtr0114325
FBgn0086050	FBgn0086050	NA	FBtr0114326
FBgn0086051	FBgn0086051	NA	FBtr0114327
FBgn0086052	FBgn0086052	NA	FBtr0114328
FBgn0086053	FBgn0086053	NA	FBtr0114329
FBgn0086054	FBgn0086054	NA	FBtr0114330
FBgn0086055	FBgn0086055	NA	FBtr0114331
FBgn0086056	FBgn0086056	NA	FBtr0114332
FBgn0086057	FBgn0086057	NA	FBtr0114333
FBgn0086058	FBgn0086058	NA	FBtr0114334
FBgn0086059	FBgn0086059	NA	FBtr0114335

FBgn0086060	FBgn0086060	NA	FBtr0114336
FBgn0086061	FBgn0086061	NA	FBtr0114337
FBgn0086062	FBgn0086062	NA	FBtr0114338
FBgn0086063	FBgn0086063	NA	FBtr0114339
FBgn0086064	FBgn0086064	NA	FBtr0114340
FBgn0086065	FBgn0086065	NA	FBtr0114341
FBgn0086066	FBgn0086066	NA	FBtr0114342
FBgn0086067	FBgn0086067	NA	FBtr0114343
FBgn0086068	FBgn0086068	NA	FBtr0114344
FBgn0086069	FBgn0086069	NA	FBtr0114345
FBgn0086070	FBgn0086070	NA	FBtr0114346
FBgn0086071	FBgn0086071	NA	FBtr0114347
FBgn0086072	FBgn0086072	NA	FBtr0114348
FBgn0086073	FBgn0086073	NA	FBtr0114349
FBgn0086074	FBgn0086074	NA	FBtr0114350
FBgn0086075	FBgn0086075	NA	FBtr0114351
FBgn0086076	FBgn0086076	NA	FBtr0114352
FBgn0086077	FBgn0086077	NA	FBtr0114353
FBgn0086078	FBgn0086078	NA	FBtr0114354
FBgn0086079	FBgn0086079	NA	FBtr0114355
FBgn0086080	FBgn0086080	NA	FBtr0114356
FBgn0086081	FBgn0086081	NA	FBtr0114357
FBgn0086082	FBgn0086082	NA	FBtr0114358
FBgn0086129	FBgn0086129	NA	FBtr0072249
FBgn0086130	FBgn0086130	CG3561	FBtr0078009
FBgn0086200	FBgn0086200	NA	FBtr0306300
FBgn0086253	FBgn0086253	CG31152	FBtr0084320
FBgn0086265	FBgn0086265	NA	FBtr0079238
FBgn0086266	FBgn0086266	NA	FBtr0079240
FBgn0086347	FBgn0086347	Myo31DF	FBtr0080036
FBgn0086356	FBgn0086356	NA	FBtr0087641
FBgn0086357	FBgn0086357	Sec61alpha	FBtr0079266
FBgn0086358	FBgn0086358	NA	FBtr0305333
FBgn0086360	FBgn0086360	NA	FBtr0083718
FBgn0086361	FBgn0086361	NA	FBtr0085443
FBgn0086371	FBgn0086371	NA	FBtr0303265
FBgn0086372	FBgn0086372	NA	FBtr0081572
FBgn0086378	FBgn0086378	NA	FBtr0301811
FBgn0086384	FBgn0086384	NA	FBtr0074754
FBgn0086408	FBgn0086408	NA	FBtr0309857
FBgn0086443	FBgn0086443	NA	FBtr0081162
FBgn0086444	FBgn0086444	CG10689	FBtr0081163
FBgn0086446	FBgn0086446	NA	FBtr0081157
FBgn0086447	FBgn0086447	NA	FBtr0081161
FBgn0086448	FBgn0086448	NA	FBtr0307175
FBgn0086472	FBgn0086472	RpS25	FBtr0082369
FBgn0086475	FBgn0086475	NA	FBtr0075231
FBgn0086532	FBgn0086532	Spt-I	FBtr0087729
FBgn0086533	FBgn0086533	NA	FBtr0309750
FBgn0086558	FBgn0086558	NA	FBtr0070933
FBgn0086604	FBgn0086604	NA	FBtr0112775
FBgn0086605	FBgn0086605	CG9853	FBtr0113192
FBgn0086608	FBgn0086608	NA	FBtr0308809
FBgn0086610	FBgn0086610	NA	FBtr0305970
FBgn0086655	FBgn0086655	jing	FBtr0114365
FBgn0086656	FBgn0086656	NA	FBtr0088623
FBgn0086674	FBgn0086674	NA	FBtr0310433

FBgn0086675	FBgn0086675	NA	FBtr0306738
FBgn0086679	FBgn0086679	NA	FBtr0081887
FBgn0086680	FBgn0086680	NA	FBtr0076944
FBgn0086681	FBgn0086681	NA	FBtr0305341
FBgn0086683	FBgn0086683	NA	FBtr0111239
FBgn0086686	FBgn0086686	CG7832	FBtr0082975
FBgn0086690	FBgn0086690	NA	FBtr0305979
FBgn0086691	FBgn0086691	CG15261	FBtr0080746
FBgn0086695	FBgn0086695	CG2669	FBtr0078802
FBgn0086699	FBgn0086699	NA	FBtr0075960
FBgn0086704	FBgn0086704	NA	FBtr0301346
FBgn0086710	FBgn0086710	NA	FBtr0310617
FBgn0086712	FBgn0086712	l(2)k14708	FBtr0088090
FBgn0086736	FBgn0086736	NA	FBtr0083399
FBgn0086757	FBgn0086757	NA	FBtr0087666
FBgn0086758	FBgn0086758	CG31666	FBtr0307047
FBgn0086768	FBgn0086768	Pcmt	FBtr0310546
FBgn0086778	FBgn0086778	NA	FBtr0074680
FBgn0086779	FBgn0086779	Grp1	FBtr0301617
FBgn0086782	FBgn0086782	NA	FBtr0074802
FBgn0086783	FBgn0086783	NA	FBtr0080905
FBgn0086785	FBgn0086785	NA	FBtr0075818
FBgn0086855	FBgn0086855	NA	FBtr0300804
FBgn0086856	FBgn0086856	CG11555	FBtr0078128
FBgn0086895	FBgn0086895	NA	FBtr0087518
FBgn0086897	FBgn0086897	NA	FBtr0082854
FBgn0086899	FBgn0086899	NA	FBtr0299581
FBgn0086900	FBgn0086900	NA	FBtr0112465
FBgn0086902	FBgn0086902	NA	FBtr0299837
FBgn0086906	FBgn0086906	sls	FBtr0309043
FBgn0086907	FBgn0086907	NA	FBtr0080888
FBgn0086908	FBgn0086908	CG30426	FBtr0112777
FBgn0086910	FBgn0086910	NA	FBtr0110876
FBgn0086911	FBgn0086911	rg	FBtr0290088
FBgn0086912	FBgn0086912	NA	FBtr0078139
FBgn0086913	FBgn0086913	NA	FBtr0112677
FBgn0086915	FBgn0086915	NA	FBtr0078270
FBgn0086917	FBgn0086917	NA	FBtr0300207
FBgn0087005	FBgn0087005	NA	FBtr0078825
FBgn0087007	FBgn0087007	CG9598	FBtr0308596
FBgn0087011	FBgn0087011	NA	FBtr0114111
FBgn0087012	FBgn0087012	5-HT2	FBtr0078854
FBgn0087035	FBgn0087035	NA	FBtr0075560
FBgn0087040	FBgn0087040	alphaTub67C	FBtr0076393
FBgn0087041	FBgn0087041	NA	FBtr0273183
FBgn0243513	FBgn0243513	NA	FBtr0077666
FBgn0243514	FBgn0243514	NA	FBtr0310079
FBgn0243516	FBgn0243516	CG13503	FBtr0071793
FBgn0243586	FBgn0243586	NA	FBtr0085036
FBgn0250754	FBgn0250754	CG14897	FBtr0290134
FBgn0250755	FBgn0250755	NA	FBtr0290135
FBgn0250756	FBgn0250756	NA	FBtr0290136
FBgn0250757	FBgn0250757	NA	FBtr0290141
FBgn0250786	FBgn0250786	NA	FBtr0077674
FBgn0250791	FBgn0250791	Snap	FBtr0074840
FBgn0250814	FBgn0250814	NA	FBtr0075309
FBgn0250816	FBgn0250816	NA	FBtr0299882

FBgn0250818	FBgn0250818	NA	FBtr0305157
FBgn0250819	FBgn0250819	NA	FBtr0091473
FBgn0250820	FBgn0250820	CG5802	FBtr0113259
FBgn0250822	FBgn0250822	NA	FBtr0112415
FBgn0250823	FBgn0250823	NA	FBtr0305071
FBgn0250824	FBgn0250824	NA	FBtr0300632
FBgn0250825	FBgn0250825	NA	FBtr0112435
FBgn0250826	FBgn0250826	NA	FBtr0112351
FBgn0250827	FBgn0250827	NA	FBtr0112411
FBgn0250830	FBgn0250830	NA	FBtr0329829
FBgn0250831	FBgn0250831	NA	FBtr0110770
FBgn0250832	FBgn0250832	NA	FBtr0305053
FBgn0250833	FBgn0250833	NA	FBtr0112767
FBgn0250836	FBgn0250836	NA	FBtr0076914
FBgn0250838	FBgn0250838	NA	FBtr0072031
FBgn0250839	FBgn0250839	CG2016	FBtr0305964
FBgn0250840	FBgn0250840	NA	FBtr0305904
FBgn0250841	FBgn0250841	NA	FBtr0077803
FBgn0250842	FBgn0250842	NA	FBtr0302939
FBgn0250843	FBgn0250843	NA	FBtr0079948
FBgn0250846	FBgn0250846	NA	FBtr0301286
FBgn0250849	FBgn0250849	NA	FBtr0076933
FBgn0250867	FBgn0250867	NA	FBtr0290305
FBgn0250868	FBgn0250868	NA	FBtr0290307
FBgn0250869	FBgn0250869	NA	FBtr0290308
FBgn0250870	FBgn0250870	NA	FBtr0290304
FBgn0250871	FBgn0250871	NA	FBtr0305609
FBgn0250876	FBgn0250876	Sema-5c	FBtr0076052
FBgn0250904	FBgn0250904	NA	FBtr0112735
FBgn0250908	FBgn0250908	NA	FBtr0290328
FBgn0250910	FBgn0250910	NA	FBtr0290334
FBgn0250911	FBgn0250911	NA	FBtr0290341
FBgn0259098	FBgn0259098	NA	FBtr0299508
FBgn0259099	FBgn0259099	CG13466	FBtr0299509
FBgn0259100	FBgn0259100	NA	FBtr0299511
FBgn0259101	FBgn0259101	NA	FBtr0299514
FBgn0259107	FBgn0259107	NA	FBtr0299516
FBgn0259108	FBgn0259108	NA	FBtr0307598
FBgn0259109	FBgn0259109	NA	FBtr0299532
FBgn0259110	FBgn0259110	mmd	FBtr0307540
FBgn0259111	FBgn0259111	Ndae1	FBtr0299539
FBgn0259112	FBgn0259112	NA	FBtr0305590
FBgn0259139	FBgn0259139	NA	FBtr0089919
FBgn0259140	FBgn0259140	NA	FBtr0299545
FBgn0259141	FBgn0259141	NA	FBtr0299551
FBgn0259142	FBgn0259142	NA	FBtr0299553
FBgn0259143	FBgn0259143	NA	FBtr0299558
FBgn0259144	FBgn0259144	NA	FBtr0299571
FBgn0259145	FBgn0259145	NA	FBtr0299572
FBgn0259146	FBgn0259146	NA	FBtr0299575
FBgn0259147	FBgn0259147	NA	FBtr0299576
FBgn0259148	FBgn0259148	NA	FBtr0302430
FBgn0259149	FBgn0259149	NA	FBtr0299596
FBgn0259150	FBgn0259150	NA	FBtr0299597
FBgn0259151	FBgn0259151	NA	FBtr0321314
FBgn0259152	FBgn0259152	NA	FBtr0301277
FBgn0259162	FBgn0259162	NA	FBtr0299603

FBgn0259163	FBgn0259163	CG32046	FBtr0299606
FBgn0259164	FBgn0259164	NA	FBtr0299610
FBgn0259166	FBgn0259166	NA	FBtr0299615
FBgn0259167	FBgn0259167	NA	FBtr0299617
FBgn0259168	FBgn0259168	NA	FBtr0300508
FBgn0259170	FBgn0259170	NA	FBtr0299633
FBgn0259171	FBgn0259171	NA	FBtr0299635
FBgn0259172	FBgn0259172	NA	FBtr0300518
FBgn0259173	FBgn0259173	corn	FBtr0299641
FBgn0259174	FBgn0259174	NA	FBtr0300519
FBgn0259175	FBgn0259175	ome	FBtr0299649
FBgn0259176	FBgn0259176	bun	FBtr0300523
FBgn0259178	FBgn0259178	NA	FBtr0299661
FBgn0259179	FBgn0259179	NA	FBtr0299663
FBgn0259183	FBgn0259183	NA	FBtr0299667
FBgn0259184	FBgn0259184	NA	FBtr0299668
FBgn0259185	FBgn0259185	NA	FBtr0299669
FBgn0259186	FBgn0259186	NA	FBtr0302916
FBgn0259187	FBgn0259187	NA	FBtr0302288
FBgn0259188	FBgn0259188	NA	FBtr0299672
FBgn0259189	FBgn0259189	NA	FBtr0299673
FBgn0259190	FBgn0259190	NA	FBtr0299674
FBgn0259191	FBgn0259191	NA	FBtr0299675
FBgn0259192	FBgn0259192	NA	FBtr0299676
FBgn0259193	FBgn0259193	NA	FBtr0299677
FBgn0259194	FBgn0259194	NA	FBtr0299678
FBgn0259195	FBgn0259195	NA	FBtr0299679
FBgn0259196	FBgn0259196	NA	FBtr0299680
FBgn0259197	FBgn0259197	NA	FBtr0299681
FBgn0259198	FBgn0259198	NA	FBtr0299682
FBgn0259199	FBgn0259199	NA	FBtr0299683
FBgn0259200	FBgn0259200	NA	FBtr0299684
FBgn0259201	FBgn0259201	NA	FBtr0299687
FBgn0259202	FBgn0259202	NA	FBtr0299688
FBgn0259203	FBgn0259203	NA	FBtr0299691
FBgn0259204	FBgn0259204	NA	FBtr0299694
FBgn0259209	FBgn0259209	NA	FBtr0299697
FBgn0259210	FBgn0259210	NA	FBtr0299700
FBgn0259211	FBgn0259211	grh	FBtr0300539
FBgn0259212	FBgn0259212	NA	FBtr0299709
FBgn0259213	FBgn0259213	NA	FBtr0299711
FBgn0259214	FBgn0259214	NA	FBtr0304050
FBgn0259215	FBgn0259215	CG17274	FBtr0299718
FBgn0259216	FBgn0259216	NA	FBtr0308691
FBgn0259217	FBgn0259217	NA	FBtr0299729
FBgn0259219	FBgn0259219	CG17041	FBtr0299740
FBgn0259220	FBgn0259220	NA	FBtr0299751
FBgn0259221	FBgn0259221	CG17034	FBtr0299764
FBgn0259222	FBgn0259222	NA	FBtr0299771
FBgn0259223	FBgn0259223	NA	FBtr0299776
FBgn0259224	FBgn0259224	NA	FBtr0299778
FBgn0259225	FBgn0259225	CG31757	FBtr0299783
FBgn0259226	FBgn0259226	NA	FBtr0299788
FBgn0259227	FBgn0259227	NA	FBtr0299789
FBgn0259228	FBgn0259228	C3G	FBtr0300526
FBgn0259229	FBgn0259229	NA	FBtr0299796
FBgn0259232	FBgn0259232	NA	FBtr0299841

FBgn0259233	FBgn0259233	CG6879	FBtr0304106
FBgn0259234	FBgn0259234	Camta	FBtr0300527
FBgn0259235	FBgn0259235	NA	FBtr0299849
FBgn0259236	FBgn0259236	NA	FBtr0299851
FBgn0259237	FBgn0259237	NA	FBtr0299852
FBgn0259238	FBgn0259238	CG13211	FBtr0303157
FBgn0259239	FBgn0259239	NA	FBtr0305981
FBgn0259240	FBgn0259240	NA	FBtr0299860
FBgn0259241	FBgn0259241	NA	FBtr0299862
FBgn0259242	FBgn0259242	NA	FBtr0299863
FBgn0259243	FBgn0259243	NA	FBtr0302641
FBgn0259244	FBgn0259244	NA	FBtr0299896
FBgn0259245	FBgn0259245	NA	FBtr0299898
FBgn0259246	FBgn0259246	NA	FBtr0305060
FBgn0259247	FBgn0259247	NA	FBtr0299918
FBgn0259482	FBgn0259482	CG4946	FBtr0079983
FBgn0259483	FBgn0259483	CG3403	FBtr0307075
FBgn0259676	FBgn0259676	NA	FBtr0078241
FBgn0259677	FBgn0259677	NA	FBtr0299928
FBgn0259678	FBgn0259678	CG1776	FBtr0310326
FBgn0259680	FBgn0259680	NA	FBtr0299935
FBgn0259681	FBgn0259681	NA	FBtr0299938
FBgn0259682	FBgn0259682	CG15092	FBtr0299943
FBgn0259683	FBgn0259683	NA	FBtr0301086
FBgn0259685	FBgn0259685	NA	FBtr0304726
FBgn0259697	FBgn0259697	NA	FBtr0113473
FBgn0259699	FBgn0259699	NA	FBtr0299953
FBgn0259700	FBgn0259700	NA	FBtr0299951
FBgn0259701	FBgn0259701	NA	FBtr0309792
FBgn0259702	FBgn0259702	NA	FBtr0299955
FBgn0259703	FBgn0259703	NA	FBtr0299956
FBgn0259704	FBgn0259704	NA	FBtr0299957
FBgn0259705	FBgn0259705	CG5558	FBtr0299958
FBgn0259707	FBgn0259707	NA	FBtr0299960
FBgn0259708	FBgn0259708	NA	FBtr0299961
FBgn0259709	FBgn0259709	NA	FBtr0299962
FBgn0259710	FBgn0259710	NA	FBtr0302526
FBgn0259711	FBgn0259711	NA	FBtr0299964
FBgn0259712	FBgn0259712	NA	FBtr0299965
FBgn0259713	FBgn0259713	NA	FBtr0299966
FBgn0259714	FBgn0259714	NA	FBtr0299967
FBgn0259715	FBgn0259715	NA	FBtr0299968
FBgn0259716	FBgn0259716	NA	FBtr0299969
FBgn0259717	FBgn0259717	NA	FBtr0299970
FBgn0259718	FBgn0259718	NA	FBtr0299971
FBgn0259719	FBgn0259719	NA	FBtr0301130
FBgn0259720	FBgn0259720	NA	FBtr0299975
FBgn0259721	FBgn0259721	NA	FBtr0299976
FBgn0259722	FBgn0259722	NA	FBtr0299979
FBgn0259723	FBgn0259723	NA	FBtr0299980
FBgn0259725	FBgn0259725	NA	FBtr0299990
FBgn0259726	FBgn0259726	NA	FBtr0299992
FBgn0259727	FBgn0259727	NA	FBtr0299989
FBgn0259728	FBgn0259728	NA	FBtr0299994
FBgn0259729	FBgn0259729	NA	FBtr0299995
FBgn0259730	FBgn0259730	NA	FBtr0299997
FBgn0259731	FBgn0259731	NA	FBtr0299998

FBgn0259732	FBgn0259732	NA	FBtr0299999
FBgn0259733	FBgn0259733	NA	FBtr0299996
FBgn0259734	FBgn0259734	NA	FBtr0300000
FBgn0259735	FBgn0259735	NA	FBtr0300003
FBgn0259736	FBgn0259736	NA	FBtr0300006
FBgn0259737	FBgn0259737	NA	FBtr0300042
FBgn0259738	FBgn0259738	NA	FBtr0300043
FBgn0259739	FBgn0259739	NA	FBtr0300044
FBgn0259740	FBgn0259740	NA	FBtr0304667
FBgn0259741	FBgn0259741	NA	FBtr0300046
FBgn0259742	FBgn0259742	NA	FBtr0299959
FBgn0259743	FBgn0259743	RhoGEF3	FBtr0299981
FBgn0259745	FBgn0259745	NA	FBtr0300081
FBgn0259748	FBgn0259748	NA	FBtr0300506
FBgn0259750	FBgn0259750	NA	FBtr0080204
FBgn0259784	FBgn0259784	NA	FBtr0083408
FBgn0259789	FBgn0259789	NA	FBtr0307537
FBgn0259794	FBgn0259794	NA	FBtr0301683
FBgn0259817	FBgn0259817	NA	FBtr0300105
FBgn0259818	FBgn0259818	CG4648	FBtr0301929
FBgn0259821	FBgn0259821	NA	FBtr0301942
FBgn0259822	FBgn0259822	NA	FBtr0306385
FBgn0259823	FBgn0259823	NA	FBtr0305614
FBgn0259831	FBgn0259831	NA	FBtr0112505
FBgn0259832	FBgn0259832	NA	FBtr0112422
FBgn0259836	FBgn0259836	NA	FBtr0300133
FBgn0259837	FBgn0259837	NA	FBtr0300134
FBgn0259838	FBgn0259838	NA	FBtr0300135
FBgn0259839	FBgn0259839	NA	FBtr0300139
FBgn0259840	FBgn0259840	NA	FBtr0300138
FBgn0259841	FBgn0259841	NA	FBtr0300140
FBgn0259842	FBgn0259842	NA	FBtr0300141
FBgn0259843	FBgn0259843	NA	FBtr0300137
FBgn0259844	FBgn0259844	NA	FBtr0300142
FBgn0259845	FBgn0259845	NA	FBtr0300136
FBgn0259846	FBgn0259846	NA	FBtr0300143
FBgn0259847	FBgn0259847	NA	FBtr0300144
FBgn0259848	FBgn0259848	NA	FBtr0300145
FBgn0259849	FBgn0259849	NA	FBtr0300146
FBgn0259850	FBgn0259850	NA	FBtr0300147
FBgn0259851	FBgn0259851	NA	FBtr0300149
FBgn0259852	FBgn0259852	NA	FBtr0300148
FBgn0259853	FBgn0259853	NA	FBtr0300151
FBgn0259854	FBgn0259854	NA	FBtr0300150
FBgn0259855	FBgn0259855	NA	FBtr0300152
FBgn0259856	FBgn0259856	NA	FBtr0300153
FBgn0259857	FBgn0259857	NA	FBtr0300154
FBgn0259858	FBgn0259858	NA	FBtr0300155
FBgn0259859	FBgn0259859	NA	FBtr0300156
FBgn0259860	FBgn0259860	NA	FBtr0300157
FBgn0259861	FBgn0259861	NA	FBtr0300158
FBgn0259862	FBgn0259862	NA	FBtr0300159
FBgn0259863	FBgn0259863	NA	FBtr0300160
FBgn0259864	FBgn0259864	NA	FBtr0300161
FBgn0259865	FBgn0259865	NA	FBtr0300162
FBgn0259866	FBgn0259866	NA	FBtr0300163
FBgn0259867	FBgn0259867	NA	FBtr0300164

FBgn0259868	FBgn0259868	NA	FBtr0300165
FBgn0259869	FBgn0259869	NA	FBtr0300166
FBgn0259870	FBgn0259870	NA	FBtr0300167
FBgn0259871	FBgn0259871	NA	FBtr0300169
FBgn0259872	FBgn0259872	NA	FBtr0300168
FBgn0259873	FBgn0259873	NA	FBtr0300170
FBgn0259874	FBgn0259874	NA	FBtr0300171
FBgn0259875	FBgn0259875	NA	FBtr0300172
FBgn0259876	FBgn0259876	Cap-G	FBtr0299507
FBgn0259896	FBgn0259896	NA	FBtr0080615
FBgn0259916	FBgn0259916	NA	FBtr0300199
FBgn0259917	FBgn0259917	NA	FBtr0300198
FBgn0259918	FBgn0259918	NA	FBtr0300200
FBgn0259922	FBgn0259922	NA	FBtr0300217
FBgn0259926	FBgn0259926	NA	FBtr0300222
FBgn0259927	FBgn0259927	NA	FBtr0310343
FBgn0259928	FBgn0259928	NA	FBtr0300230
FBgn0259929	FBgn0259929	NA	FBtr0300251
FBgn0259930	FBgn0259930	NA	FBtr0300232
FBgn0259931	FBgn0259931	NA	FBtr0300233
FBgn0259932	FBgn0259932	NA	FBtr0300239
FBgn0259933	FBgn0259933	NA	FBtr0300240
FBgn0259934	FBgn0259934	NA	FBtr0306758
FBgn0259935	FBgn0259935	NA	FBtr0300248
FBgn0259937	FBgn0259937	NA	FBtr0300861
FBgn0259938	FBgn0259938	NA	FBtr0305152
FBgn0259949	FBgn0259949	NA	FBtr0300295
FBgn0259950	FBgn0259950	NA	FBtr0300294
FBgn0259951	FBgn0259951	NA	FBtr0300296
FBgn0259952	FBgn0259952	NA	FBtr0300297
FBgn0259953	FBgn0259953	NA	FBtr0302203
FBgn0259954	FBgn0259954	NA	FBtr0300300
FBgn0259955	FBgn0259955	NA	FBtr0300301
FBgn0259956	FBgn0259956	NA	FBtr0300302
FBgn0259957	FBgn0259957	NA	FBtr0300303
FBgn0259958	FBgn0259958	NA	FBtr0300304
FBgn0259959	FBgn0259959	NA	FBtr0300305
FBgn0259960	FBgn0259960	NA	FBtr0300308
FBgn0259961	FBgn0259961	NA	FBtr0300307
FBgn0259962	FBgn0259962	NA	FBtr0300306
FBgn0259963	FBgn0259963	NA	FBtr0300309
FBgn0259964	FBgn0259964	NA	FBtr0300310
FBgn0259965	FBgn0259965	NA	FBtr0300311
FBgn0259966	FBgn0259966	NA	FBtr0300323
FBgn0259967	FBgn0259967	NA	FBtr0300312
FBgn0259968	FBgn0259968	NA	FBtr0300313
FBgn0259969	FBgn0259969	NA	FBtr0300314
FBgn0259970	FBgn0259970	NA	FBtr0300316
FBgn0259971	FBgn0259971	NA	FBtr0300315
FBgn0259972	FBgn0259972	NA	FBtr0300317
FBgn0259973	FBgn0259973	NA	FBtr0300318
FBgn0259974	FBgn0259974	NA	FBtr0300322
FBgn0259975	FBgn0259975	NA	FBtr0300319
FBgn0259977	FBgn0259977	NA	FBtr0086143
FBgn0259978	FBgn0259978	NA	FBtr0300502
FBgn0259979	FBgn0259979	NA	FBtr0086038
FBgn0259982	FBgn0259982	NA	FBtr0080708

FBgn0259984	FBgn0259984	NA	FBtr0080509
FBgn0259985	FBgn0259985	CG8889	FBtr0088026
FBgn0259986	FBgn0259986	NA	FBtr0302032
FBgn0259989	FBgn0259989	NA	FBtr0300437
FBgn0259990	FBgn0259990	NA	FBtr0300439
FBgn0259991	FBgn0259991	NA	FBtr0300440
FBgn0259992	FBgn0259992	NA	FBtr0304002
FBgn0259993	FBgn0259993	NA	FBtr0300446
FBgn0259994	FBgn0259994	NA	FBtr0310286
FBgn0259996	FBgn0259996	NA	FBtr0302056
FBgn0260004	FBgn0260004	NA	FBtr0084027
FBgn0260007	FBgn0260007	NA	FBtr0300507
FBgn0260008	FBgn0260008	NA	FBtr0301785
FBgn0260010	FBgn0260010	NA	FBtr0082123
FBgn0260011	FBgn0260011	CG16876	FBtr0080592
FBgn0260012	FBgn0260012	NA	FBtr0306062
FBgn0260026	FBgn0260026	NA	FBtr0300584
FBgn0260027	FBgn0260027	NA	FBtr0300582
FBgn0260049	FBgn0260049	CG10724	FBtr0075845
FBgn0260222	FBgn0260222	NA	FBtr0300625
FBgn0260223	FBgn0260223	NA	FBtr0300626
FBgn0260224	FBgn0260224	NA	FBtr0300624
FBgn0260225	FBgn0260225	NA	FBtr0300629
FBgn0260226	FBgn0260226	NA	FBtr0300627
FBgn0260228	FBgn0260228	NA	FBtr0300635
FBgn0260229	FBgn0260229	NA	FBtr0300637
FBgn0260230	FBgn0260230	NA	FBtr0300639
FBgn0260231	FBgn0260231	NA	FBtr0300638
FBgn0260232	FBgn0260232	NA	FBtr0300640
FBgn0260233	FBgn0260233	NA	FBtr0300641
FBgn0260234	FBgn0260234	NA	FBtr0300643
FBgn0260235	FBgn0260235	NA	FBtr0300644
FBgn0260237	FBgn0260237	NA	FBtr0300646
FBgn0260238	FBgn0260238	NA	FBtr0300651
FBgn0260241	FBgn0260241	NA	FBtr0299939
FBgn0260386	FBgn0260386	CG7549	FBtr0300601
FBgn0260388	FBgn0260388	NA	FBtr0300655
FBgn0260389	FBgn0260389	NA	FBtr0300658
FBgn0260390	FBgn0260390	NA	FBtr0300660
FBgn0260391	FBgn0260391	NA	FBtr0300664
FBgn0260392	FBgn0260392	NA	FBtr0300662
FBgn0260393	FBgn0260393	NA	FBtr0310467
FBgn0260396	FBgn0260396	NA	FBtr0300668
FBgn0260399	FBgn0260399	gwl	FBtr0083674
FBgn0260400	FBgn0260400	NA	FBtr0307173
FBgn0260407	FBgn0260407	NA	FBtr0080566
FBgn0260428	FBgn0260428	NA	FBtr0300851
FBgn0260429	FBgn0260429	NA	FBtr0300853
FBgn0260430	FBgn0260430	NA	FBtr0300854
FBgn0260431	FBgn0260431	NA	FBtr0300855
FBgn0260432	FBgn0260432	NA	FBtr0300856
FBgn0260434	FBgn0260434	NA	FBtr0300858
FBgn0260435	FBgn0260435	NA	FBtr0302459
FBgn0260436	FBgn0260436	NA	FBtr0300859
FBgn0260437	FBgn0260437	NA	FBtr0300860
FBgn0260439	FBgn0260439	NA	FBtr0306102
FBgn0260440	FBgn0260440	spdo	FBtr0085651

FBgn0260441	FBgn0260441	RpS12	FBtr0075878
FBgn0260442	FBgn0260442	rhea	FBtr0305210
FBgn0260444	FBgn0260444	NA	FBtr0082643
FBgn0260446	FBgn0260446	NA	FBtr0304732
FBgn0260452	FBgn0260452	NA	FBtr0079247
FBgn0260455	FBgn0260455	NA	FBtr0072109
FBgn0260456	FBgn0260456	NA	FBtr0072332
FBgn0260459	FBgn0260459	NA	FBtr0075858
FBgn0260461	FBgn0260461	NA	FBtr0074936
FBgn0260462	FBgn0260462	NA	FBtr0078823
FBgn0260463	FBgn0260463	NA	FBtr0303039
FBgn0260466	FBgn0260466	NA	FBtr0091937
FBgn0260468	FBgn0260468	NA	FBtr0085564
FBgn0260469	FBgn0260469	NA	FBtr0301970
FBgn0260474	FBgn0260474	NA	FBtr0088466
FBgn0260475	FBgn0260475	NA	FBtr0087715
FBgn0260477	FBgn0260477	NA	FBtr0300950
FBgn0260480	FBgn0260480	NA	FBtr0072962
FBgn0260481	FBgn0260481	NA	FBtr0078529
FBgn0260482	FBgn0260482	NA	FBtr0073904
FBgn0260486	FBgn0260486	NA	FBtr0300897
FBgn0260487	FBgn0260487	NA	FBtr0309307
FBgn0260499	FBgn0260499	NA	FBtr0114575
FBgn0260632	FBgn0260632	dl	FBtr0081007
FBgn0260643	FBgn0260643	NA	FBtr0300948
FBgn0260644	FBgn0260644	NA	FBtr0300949
FBgn0260645	FBgn0260645	NA	FBtr0301001
FBgn0260646	FBgn0260646	NA	FBtr0301002
FBgn0260648	FBgn0260648	NA	FBtr0077883
FBgn0260655	FBgn0260655	NA	FBtr0074966
FBgn0260656	FBgn0260656	NA	FBtr0301215
FBgn0260657	FBgn0260657	NA	FBtr0301097
FBgn0260658	FBgn0260658	NA	FBtr0301099
FBgn0260659	FBgn0260659	NA	FBtr0301104
FBgn0260660	FBgn0260660	NA	FBtr0301958
FBgn0260713	FBgn0260713	NA	FBtr0301214
FBgn0260718	FBgn0260718	NA	FBtr0301174
FBgn0260719	FBgn0260719	NA	FBtr0301172
FBgn0260720	FBgn0260720	NA	FBtr0301177
FBgn0260721	FBgn0260721	NA	FBtr0301175
FBgn0260722	FBgn0260722	NA	FBtr0301250
FBgn0260723	FBgn0260723	NA	FBtr0301216
FBgn0260745	FBgn0260745	mfas	FBtr0082494
FBgn0260748	FBgn0260748	NA	FBtr0074386
FBgn0260749	FBgn0260749	CG5640	FBtr0080075
FBgn0260750	FBgn0260750	NA	FBtr0079986
FBgn0260753	FBgn0260753	NA	FBtr0308565
FBgn0260754	FBgn0260754	NA	FBtr0301251
FBgn0260755	FBgn0260755	NA	FBtr0301252
FBgn0260756	FBgn0260756	NA	FBtr0301253
FBgn0260758	FBgn0260758	NA	FBtr0301255
FBgn0260759	FBgn0260759	NA	FBtr0301256
FBgn0260760	FBgn0260760	NA	FBtr0301257
FBgn0260761	FBgn0260761	NA	FBtr0301260
FBgn0260762	FBgn0260762	NA	FBtr0301262
FBgn0260763	FBgn0260763	NA	FBtr0301265
FBgn0260764	FBgn0260764	NA	FBtr0301266

FBgn0260765	FBgn0260765	NA	FBtr0301267
FBgn0260766	FBgn0260766	NA	FBtr0301268
FBgn0260767	FBgn0260767	NA	FBtr0301269
FBgn0260768	FBgn0260768	NA	FBtr0301270
FBgn0260769	FBgn0260769	NA	FBtr0301272
FBgn0260770	FBgn0260770	NA	FBtr0301274
FBgn0260771	FBgn0260771	NA	FBtr0301275
FBgn0260776	FBgn0260776	NA	FBtr0301333
FBgn0260777	FBgn0260777	NA	FBtr0301332
FBgn0260792	FBgn0260792	NA	FBtr0301431
FBgn0260793	FBgn0260793	NA	FBtr0301436
FBgn0260794	FBgn0260794	CG17735	FBtr0303916
FBgn0260795	FBgn0260795	CG7628	FBtr0301438
FBgn0260798	FBgn0260798	NA	FBtr0111121
FBgn0260813	FBgn0260813	NA	FBtr0301608
FBgn0260817	FBgn0260817	NA	FBtr0077574
FBgn0260855	FBgn0260855	CG7359	FBtr0310162
FBgn0260856	FBgn0260856	NA	FBtr0077145
FBgn0260857	FBgn0260857	NA	FBtr0074990
FBgn0260862	FBgn0260862	CG3279	FBtr0072607
FBgn0260867	FBgn0260867	NA	FBtr0301567
FBgn0260868	FBgn0260868	NA	FBtr0301566
FBgn0260869	FBgn0260869	NA	FBtr0301565
FBgn0260870	FBgn0260870	NA	FBtr0301564
FBgn0260871	FBgn0260871	NA	FBtr0301573
FBgn0260872	FBgn0260872	NA	FBtr0301571
FBgn0260873	FBgn0260873	NA	FBtr0301570
FBgn0260874	FBgn0260874	NA	FBtr0301607
FBgn0260933	FBgn0260933	NA	FBtr0303763
FBgn0260934	FBgn0260934	NA	FBtr0304962
FBgn0260936	FBgn0260936	mule	FBtr0307975
FBgn0260938	FBgn0260938	NA	FBtr0074167
FBgn0260941	FBgn0260941	NA	FBtr0301054
FBgn0260943	FBgn0260943	CG32169	FBtr0310005
FBgn0260944	FBgn0260944	NA	FBtr0082277
FBgn0260946	FBgn0260946	NA	FBtr0084978
FBgn0260953	FBgn0260953	NA	FBtr0301738
FBgn0260954	FBgn0260954	NA	FBtr0301741
FBgn0260955	FBgn0260955	NA	FBtr0301742
FBgn0260963	FBgn0260963	CG13913	FBtr0072632
FBgn0260964	FBgn0260964	NA	FBtr0091491
FBgn0260965	FBgn0260965	NA	FBtr0301717
FBgn0260967	FBgn0260967	NA	FBtr0301744
FBgn0260968	FBgn0260968	NA	FBtr0301743
FBgn0260969	FBgn0260969	NA	FBtr0301747
FBgn0260970	FBgn0260970	NA	FBtr0301748
FBgn0260971	FBgn0260971	NA	FBtr0301750
FBgn0260985	FBgn0260985	RfC40	FBtr0073264
FBgn0260993	FBgn0260993	NA	FBtr0303687
FBgn0260994	FBgn0260994	NA	FBtr0302226
FBgn0260995	FBgn0260995	NA	FBtr0301776
FBgn0260996	FBgn0260996	NA	FBtr0301799
FBgn0260997	FBgn0260997	NA	FBtr0301800
FBgn0261006	FBgn0261006	NA	FBtr0309752
FBgn0261015	FBgn0261015	NA	FBtr0301876
FBgn0261016	FBgn0261016	NA	FBtr0305178
FBgn0261041	FBgn0261041	NA	FBtr0304694

FBgn0261053	FBgn0261053	NA	FBtr0301933
FBgn0261054	FBgn0261054	NA	FBtr0301934
FBgn0261055	FBgn0261055	NA	FBtr0301935
FBgn0261056	FBgn0261056	NA	FBtr0301936
FBgn0261057	FBgn0261057	NA	FBtr0301937
FBgn0261058	FBgn0261058	NA	FBtr0301938
FBgn0261059	FBgn0261059	NA	FBtr0301939
FBgn0261060	FBgn0261060	NA	FBtr0301940
FBgn0261061	FBgn0261061	NA	FBtr0301941
FBgn0261064	FBgn0261064	CG8506	FBtr0079581
FBgn0261068	FBgn0261068	CG13277	FBtr0080934
FBgn0261089	FBgn0261089	NA	FBtr0303255
FBgn0261108	FBgn0261108	NA	FBtr0081843
FBgn0261109	FBgn0261109	Tfb2	FBtr0075595
FBgn0261112	FBgn0261112	NA	FBtr0076208
FBgn0261243	FBgn0261243	Psa	FBtr0072700
FBgn0261245	FBgn0261245	CG13011	FBtr0074318
FBgn0261258	FBgn0261258	NA	FBtr0078346
FBgn0261259	FBgn0261259	CG32030	FBtr0302087
FBgn0261260	FBgn0261260	CG12139	FBtr0302153
FBgn0261261	FBgn0261261	NA	FBtr0302091
FBgn0261262	FBgn0261262	NA	FBtr0304685
FBgn0261267	FBgn0261267	NA	FBtr0302222
FBgn0261268	FBgn0261268	gft	FBtr0302156
FBgn0261277	FBgn0261277	NA	FBtr0073470
FBgn0261280	FBgn0261280	NA	FBtr0302215
FBgn0261281	FBgn0261281	NA	FBtr0302219
FBgn0261284	FBgn0261284	CG14430	FBtr0071004
FBgn0261285	FBgn0261285	NA	FBtr0083752
FBgn0261290	FBgn0261290	NA	FBtr0091780
FBgn0261334	FBgn0261334	NA	FBtr0302236
FBgn0261335	FBgn0261335	NA	FBtr0302239
FBgn0261336	FBgn0261336	NA	FBtr0302249
FBgn0261337	FBgn0261337	NA	FBtr0302252
FBgn0261338	FBgn0261338	NA	FBtr0302254
FBgn0261339	FBgn0261339	NA	FBtr0302256
FBgn0261341	FBgn0261341	NA	FBtr0302334
FBgn0261349	FBgn0261349	NA	FBtr0302267
FBgn0261352	FBgn0261352	NA	FBtr0302270
FBgn0261353	FBgn0261353	NA	FBtr0302271
FBgn0261354	FBgn0261354	NA	FBtr0302272
FBgn0261356	FBgn0261356	NA	FBtr0302274
FBgn0261357	FBgn0261357	NA	FBtr0302275
FBgn0261358	FBgn0261358	NA	FBtr0302276
FBgn0261359	FBgn0261359	NA	FBtr0302282
FBgn0261360	FBgn0261360	NA	FBtr0302279
FBgn0261361	FBgn0261361	NA	FBtr0302283
FBgn0261362	FBgn0261362	NA	FBtr0302291
FBgn0261363	FBgn0261363	Dox-A3	FBtr0302290
FBgn0261364	FBgn0261364	NA	FBtr0302289
FBgn0261365	FBgn0261365	NA	FBtr0302292
FBgn0261366	FBgn0261366	NA	FBtr0302293
FBgn0261380	FBgn0261380	NA	FBtr0302273
FBgn0261383	FBgn0261383	NA	FBtr0070841
FBgn0261386	FBgn0261386	NA	FBtr0304153
FBgn0261393	FBgn0261393	NA	FBtr0307274
FBgn0261394	FBgn0261394	NA	FBtr0071522

FBgn0261399	FBgn0261399	NA	FBtr0302342
FBgn0261404	FBgn0261404	NA	FBtr0310454
FBgn0261429	FBgn0261429	NA	FBtr0302350
FBgn0261434	FBgn0261434	hkb	FBtr0078951
FBgn0261437	FBgn0261437	NA	FBtr0079646
FBgn0261439	FBgn0261439	Scs-fp	FBtr0086553
FBgn0261444	FBgn0261444	NA	FBtr0070249
FBgn0261451	FBgn0261451	NA	FBtr0305161
FBgn0261452	FBgn0261452	NA	FBtr0309753
FBgn0261453	FBgn0261453	NA	FBtr0309754
FBgn0261454	FBgn0261454	NA	FBtr0309755
FBgn0261455	FBgn0261455	NA	FBtr0309756
FBgn0261456	FBgn0261456	hpo	FBtr0086500
FBgn0261461	FBgn0261461	NA	FBtr0299623
FBgn0261477	FBgn0261477	slim	FBtr0086693
FBgn0261501	FBgn0261501	NA	FBtr0302392
FBgn0261502	FBgn0261502	NA	FBtr0302394
FBgn0261503	FBgn0261503	NA	FBtr0302395
FBgn0261504	FBgn0261504	NA	FBtr0302398
FBgn0261505	FBgn0261505	NA	FBtr0302396
FBgn0261506	FBgn0261506	NA	FBtr0302431
FBgn0261507	FBgn0261507	NA	FBtr0307886
FBgn0261508	FBgn0261508	NA	FBtr0302437
FBgn0261509	FBgn0261509	NA	FBtr0310020
FBgn0261514	FBgn0261514	NA	FBtr0304719
FBgn0261522	FBgn0261522	NA	FBtr0302457
FBgn0261523	FBgn0261523	NA	FBtr0302458
FBgn0261530	FBgn0261530	NA	FBtr0306040
FBgn0261531	FBgn0261531	NA	FBtr0303712
FBgn0261536	FBgn0261536	NA	FBtr0302558
FBgn0261537	FBgn0261537	NA	FBtr0302559
FBgn0261538	FBgn0261538	NA	FBtr0302555
FBgn0261545	FBgn0261545	NA	FBtr0302645
FBgn0261547	FBgn0261547	NA	FBtr0302650
FBgn0261548	FBgn0261548	CG14801	FBtr0309969
FBgn0261549	FBgn0261549	NA	FBtr0302660
FBgn0261550	FBgn0261550	CG5077	FBtr0302673
FBgn0261551	FBgn0261551	CG32306	FBtr0306701
FBgn0261553	FBgn0261553	NA	FBtr0304933
FBgn0261554	FBgn0261554	NA	FBtr0302702
FBgn0261555	FBgn0261555	CG32048	FBtr0302710
FBgn0261556	FBgn0261556	NA	FBtr0302714
FBgn0261559	FBgn0261559	NA	FBtr0308355
FBgn0261561	FBgn0261561	NA	FBtr0302725
FBgn0261562	FBgn0261562	CG12022	FBtr0302728
FBgn0261563	FBgn0261563	NA	FBtr0302731
FBgn0261564	FBgn0261564	NA	FBtr0302770
FBgn0261565	FBgn0261565	NA	FBtr0302786
FBgn0261566	FBgn0261566	NA	FBtr0302802
FBgn0261567	FBgn0261567	NA	FBtr0302803
FBgn0261568	FBgn0261568	NA	FBtr0302804
FBgn0261569	FBgn0261569	NA	FBtr0302838
FBgn0261570	FBgn0261570	NA	FBtr0302833
FBgn0261571	FBgn0261571	NA	FBtr0302857
FBgn0261572	FBgn0261572	NA	FBtr0302858
FBgn0261573	FBgn0261573	NA	FBtr0302863
FBgn0261578	FBgn0261578	NA	FBtr0302890

FBgn0261579	FBgn0261579	NA	FBtr0302891
FBgn0261580	FBgn0261580	NA	FBtr0302889
FBgn0261581	FBgn0261581	NA	FBtr0302888
FBgn0261582	FBgn0261582	NA	FBtr0302893
FBgn0261583	FBgn0261583	NA	FBtr0302894
FBgn0261584	FBgn0261584	NA	FBtr0302896
FBgn0261586	FBgn0261586	NA	FBtr0302900
FBgn0261587	FBgn0261587	NA	FBtr0302904
FBgn0261588	FBgn0261588	NA	FBtr0302919
FBgn0261593	FBgn0261593	NA	FBtr0308192
FBgn0261596	FBgn0261596	NA	FBtr0071935
FBgn0261606	FBgn0261606	RpL27A	FBtr0303765
FBgn0261610	FBgn0261610	NA	FBtr0302921
FBgn0261611	FBgn0261611	NA	FBtr0302922
FBgn0261612	FBgn0261612	NA	FBtr0302925
FBgn0261613	FBgn0261613	CG17390	FBtr0302927
FBgn0261614	FBgn0261614	NA	FBtr0302953
FBgn0261615	FBgn0261615	NA	FBtr0302954
FBgn0261616	FBgn0261616	NA	FBtr0302955
FBgn0261617	FBgn0261617	NA	FBtr0302723
FBgn0261618	FBgn0261618	NA	FBtr0301180
FBgn0261624	FBgn0261624	NA	FBtr0302971
FBgn0261625	FBgn0261625	NA	FBtr0302974
FBgn0261626	FBgn0261626	NA	FBtr0302981
FBgn0261627	FBgn0261627	NA	FBtr0302991
FBgn0261628	FBgn0261628	NA	FBtr0302989
FBgn0261629	FBgn0261629	NA	FBtr0302994
FBgn0261630	FBgn0261630	NA	FBtr0302995
FBgn0261631	FBgn0261631	NA	FBtr0302996
FBgn0261632	FBgn0261632	NA	FBtr0302997
FBgn0261633	FBgn0261633	NA	FBtr0302998
FBgn0261634	FBgn0261634	NA	FBtr0302999
FBgn0261635	FBgn0261635	NA	FBtr0303001
FBgn0261636	FBgn0261636	NA	FBtr0303002
FBgn0261637	FBgn0261637	NA	FBtr0303007
FBgn0261638	FBgn0261638	NA	FBtr0309069
FBgn0261639	FBgn0261639	NA	FBtr0303009
FBgn0261640	FBgn0261640	NA	FBtr0303010
FBgn0261641	FBgn0261641	CG31367	FBtr0303013
FBgn0261642	FBgn0261642	NA	FBtr0306602
FBgn0261649	FBgn0261649	NA	FBtr0089771
FBgn0261671	FBgn0261671	NA	FBtr0304929
FBgn0261679	FBgn0261679	NA	FBtr0303074
FBgn0261680	FBgn0261680	NA	FBtr0303075
FBgn0261681	FBgn0261681	NA	FBtr0303076
FBgn0261682	FBgn0261682	NA	FBtr0303077
FBgn0261683	FBgn0261683	NA	FBtr0303078
FBgn0261684	FBgn0261684	NA	FBtr0303079
FBgn0261686	FBgn0261686	NA	FBtr0303084
FBgn0261687	FBgn0261687	NA	FBtr0303125
FBgn0261698	FBgn0261698	NA	FBtr0303083
FBgn0261699	FBgn0261699	NA	FBtr0303192
FBgn0261700	FBgn0261700	NA	FBtr0303193
FBgn0261702	FBgn0261702	NA	FBtr0303196
FBgn0261703	FBgn0261703	NA	FBtr0303198
FBgn0261704	FBgn0261704	NA	FBtr0303202
FBgn0261705	FBgn0261705	NA	FBtr0303203

FBgn0261706	FBgn0261706	NA	FBtr0303209
FBgn0261707	FBgn0261707	NA	FBtr0303212
FBgn0261708	FBgn0261708	NA	FBtr0303214
FBgn0261709	FBgn0261709	NA	FBtr0303215
FBgn0261710	FBgn0261710	NA	FBtr0071461
FBgn0261722	FBgn0261722	CG6151	FBtr0075541
FBgn0261787	FBgn0261787	CG2478	FBtr0081403
FBgn0261792	FBgn0261792	CG5454	FBtr0083720
FBgn0261800	FBgn0261800	NA	FBtr0079490
FBgn0261801	FBgn0261801	NA	FBtr0303270
FBgn0261802	FBgn0261802	NA	FBtr0303277
FBgn0261803	FBgn0261803	CG15786	FBtr0303278
FBgn0261804	FBgn0261804	NA	FBtr0303280
FBgn0261805	FBgn0261805	NA	FBtr0303281
FBgn0261806	FBgn0261806	NA	FBtr0303282
FBgn0261807	FBgn0261807	NA	FBtr0303283
FBgn0261811	FBgn0261811	NA	FBtr0074804
FBgn0261812	FBgn0261812	NA	FBtr0303298
FBgn0261813	FBgn0261813	NA	FBtr0303299
FBgn0261814	FBgn0261814	NA	FBtr0303300
FBgn0261815	FBgn0261815	NA	FBtr0303302
FBgn0261816	FBgn0261816	NA	FBtr0303303
FBgn0261817	FBgn0261817	NA	FBtr0303304
FBgn0261818	FBgn0261818	NA	FBtr0303306
FBgn0261819	FBgn0261819	NA	FBtr0303305
FBgn0261830	FBgn0261830	NA	FBtr0303390
FBgn0261831	FBgn0261831	NA	FBtr0303391
FBgn0261832	FBgn0261832	NA	FBtr0303393
FBgn0261833	FBgn0261833	NA	FBtr0303394
FBgn0261834	FBgn0261834	NA	FBtr0303395
FBgn0261835	FBgn0261835	NA	FBtr0303397
FBgn0261836	FBgn0261836	NA	FBtr0303387
FBgn0261837	FBgn0261837	NA	FBtr0303403
FBgn0261838	FBgn0261838	NA	FBtr0303402
FBgn0261839	FBgn0261839	NA	FBtr0303405
FBgn0261840	FBgn0261840	NA	FBtr0303404
FBgn0261841	FBgn0261841	NA	FBtr0303407
FBgn0261842	FBgn0261842	NA	FBtr0303406
FBgn0261843	FBgn0261843	NA	FBtr0303408
FBgn0261844	FBgn0261844	NA	FBtr0303409
FBgn0261845	FBgn0261845	NA	FBtr0303410
FBgn0261846	FBgn0261846	NA	FBtr0307889
FBgn0261847	FBgn0261847	NA	FBtr0303412
FBgn0261848	FBgn0261848	NA	FBtr0303416
FBgn0261849	FBgn0261849	NA	FBtr0303417
FBgn0261853	FBgn0261853	NA	FBtr0303428
FBgn0261854	FBgn0261854	NA	FBtr0303435
FBgn0261855	FBgn0261855	NA	FBtr0308950
FBgn0261856	FBgn0261856	NA	FBtr0303442
FBgn0261857	FBgn0261857	NA	FBtr0303443
FBgn0261858	FBgn0261858	NA	FBtr0303463
FBgn0261859	FBgn0261859	CG6934	FBtr0303486
FBgn0261860	FBgn0261860	NA	FBtr0310472
FBgn0261861	FBgn0261861	NA	FBtr0310469
FBgn0261871	FBgn0261871	dpr2	FBtr0306117
FBgn0261872	FBgn0261872	scaf6	FBtr0091474
FBgn0261873	FBgn0261873	NA	FBtr0100376

FBgn0261881	FBgn0261881	NA	FBtr0080728
FBgn0261882	FBgn0261882	NA	FBtr0080727
FBgn0261914	FBgn0261914	NA	FBtr0306037
FBgn0261922	FBgn0261922	NA	FBtr0307158
FBgn0261924	FBgn0261924	NA	FBtr0303588
FBgn0261925	FBgn0261925	NA	FBtr0303589
FBgn0261927	FBgn0261927	NA	FBtr0303591
FBgn0261928	FBgn0261928	NA	FBtr0303602
FBgn0261929	FBgn0261929	NA	FBtr0303603
FBgn0261931	FBgn0261931	NA	FBtr0303710
FBgn0261932	FBgn0261932	NA	FBtr0303731
FBgn0261934	FBgn0261934	NA	FBtr0303750
FBgn0261954	FBgn0261954	NA	FBtr0307290
FBgn0261963	FBgn0261963	NA	FBtr0079093
FBgn0261972	FBgn0261972	NA	FBtr0303790
FBgn0261973	FBgn0261973	NA	FBtr0303791
FBgn0261974	FBgn0261974	NA	FBtr0303792
FBgn0261975	FBgn0261975	NA	FBtr0304022
FBgn0261983	FBgn0261983	CG4713	FBtr0306138
FBgn0261985	FBgn0261985	Ptpmeg	FBtr0306560
FBgn0261989	FBgn0261989	NA	FBtr0303819
FBgn0261990	FBgn0261990	NA	FBtr0303818
FBgn0261991	FBgn0261991	NA	FBtr0303820
FBgn0261992	FBgn0261992	NA	FBtr0303821
FBgn0261993	FBgn0261993	NA	FBtr0303823
FBgn0261994	FBgn0261994	NA	FBtr0303822
FBgn0261995	FBgn0261995	NA	FBtr0303825
FBgn0261996	FBgn0261996	NA	FBtr0303824
FBgn0261997	FBgn0261997	NA	FBtr0303827
FBgn0261998	FBgn0261998	NA	FBtr0307890
FBgn0261999	FBgn0261999	NA	FBtr0303797
FBgn0262000	FBgn0262000	NA	FBtr0303798
FBgn0262001	FBgn0262001	NA	FBtr0303800
FBgn0262002	FBgn0262002	NA	FBtr0303799
FBgn0262003	FBgn0262003	NA	FBtr0303828
FBgn0262004	FBgn0262004	NA	FBtr0303829
FBgn0262005	FBgn0262005	NA	FBtr0303831
FBgn0262006	FBgn0262006	NA	FBtr0303830
FBgn0262007	FBgn0262007	NA	FBtr0303833
FBgn0262008	FBgn0262008	CG6640	FBtr0303832
FBgn0262009	FBgn0262009	NA	FBtr0303834
FBgn0262010	FBgn0262010	NA	FBtr0303835
FBgn0262017	FBgn0262017	NA	FBtr0303837
FBgn0262018	FBgn0262018	NA	FBtr0308770
FBgn0262020	FBgn0262020	NA	FBtr0303850
FBgn0262021	FBgn0262021	NA	FBtr0303851
FBgn0262022	FBgn0262022	NA	FBtr0303852
FBgn0262023	FBgn0262023	NA	FBtr0303853
FBgn0262024	FBgn0262024	NA	FBtr0303854
FBgn0262025	FBgn0262025	NA	FBtr0303856
FBgn0262026	FBgn0262026	NA	FBtr0303860
FBgn0262027	FBgn0262027	NA	FBtr0303863
FBgn0262028	FBgn0262028	NA	FBtr0309032
FBgn0262029	FBgn0262029	d	FBtr0303923
FBgn0262030	FBgn0262030	NA	FBtr0303925
FBgn0262031	FBgn0262031	NA	FBtr0303926
FBgn0262032	FBgn0262032	NA	FBtr0303928

FBgn0262033	FBgn0262033	NA	FBtr0303929
FBgn0262035	FBgn0262035	NA	FBtr0303931
FBgn0262036	FBgn0262036	NA	FBtr0303932
FBgn0262095	FBgn0262095	NA	FBtr0304012
FBgn0262097	FBgn0262097	NA	FBtr0310549
FBgn0262098	FBgn0262098	NA	FBtr0304016
FBgn0262099	FBgn0262099	NA	FBtr0304017
FBgn0262100	FBgn0262100	NA	FBtr0304019
FBgn0262101	FBgn0262101	NA	FBtr0304020
FBgn0262102	FBgn0262102	NA	FBtr0304023
FBgn0262103	FBgn0262103	NA	FBtr0304026
FBgn0262104	FBgn0262104	NA	FBtr0304028
FBgn0262105	FBgn0262105	NA	FBtr0304027
FBgn0262106	FBgn0262106	NA	FBtr0309033
FBgn0262107	FBgn0262107	NA	FBtr0304033
FBgn0262108	FBgn0262108	NA	FBtr0304031
FBgn0262109	FBgn0262109	NA	FBtr0304059
FBgn0262110	FBgn0262110	CG11814	FBtr0304060
FBgn0262111	FBgn0262111	f	FBtr0304061
FBgn0262112	FBgn0262112	NA	FBtr0085481
FBgn0262114	FBgn0262114	NA	FBtr0100460
FBgn0262125	FBgn0262125	NA	FBtr0304794
FBgn0262139	FBgn0262139	NA	FBtr0304120
FBgn0262140	FBgn0262140	NA	FBtr0304123
FBgn0262141	FBgn0262141	NA	FBtr0304124
FBgn0262142	FBgn0262142	NA	FBtr0304125
FBgn0262143	FBgn0262143	NA	FBtr0309855
FBgn0262144	FBgn0262144	NA	FBtr0309854
FBgn0262145	FBgn0262145	NA	FBtr0304128
FBgn0262146	FBgn0262146	NA	FBtr0304129
FBgn0262148	FBgn0262148	NA	FBtr0304139
FBgn0262149	FBgn0262149	NA	FBtr0304141
FBgn0262150	FBgn0262150	NA	FBtr0304142
FBgn0262160	FBgn0262160	NA	FBtr0089956
FBgn0262163	FBgn0262163	NA	FBtr0304150
FBgn0262170	FBgn0262170	NA	FBtr0304652
FBgn0262350	FBgn0262350	bru-3	FBtr0303519
FBgn0262351	FBgn0262351	NA	FBtr0304634
FBgn0262352	FBgn0262352	NA	FBtr0304630
FBgn0262353	FBgn0262353	NA	FBtr0304629
FBgn0262354	FBgn0262354	NA	FBtr0304632
FBgn0262355	FBgn0262355	NA	FBtr0304633
FBgn0262356	FBgn0262356	NA	FBtr0304636
FBgn0262357	FBgn0262357	NA	FBtr0304637
FBgn0262358	FBgn0262358	NA	FBtr0304638
FBgn0262359	FBgn0262359	NA	FBtr0304640
FBgn0262360	FBgn0262360	NA	FBtr0304641
FBgn0262361	FBgn0262361	NA	FBtr0304642
FBgn0262362	FBgn0262362	NA	FBtr0304643
FBgn0262363	FBgn0262363	NA	FBtr0304645
FBgn0262364	FBgn0262364	NA	FBtr0304646
FBgn0262365	FBgn0262365	NA	FBtr0304647
FBgn0262366	FBgn0262366	NA	FBtr0304649
FBgn0262468	FBgn0262468	NA	FBtr0310064
FBgn0262475	FBgn0262475	NA	FBtr0304856
FBgn0262476	FBgn0262476	CG5226	FBtr0304867
FBgn0262477	FBgn0262477	NA	FBtr0304869

FBgn0262478	FBgn0262478	NA	FBtr0304816
FBgn0262479	FBgn0262479	NA	FBtr0304817
FBgn0262480	FBgn0262480	NA	FBtr0304818
FBgn0262481	FBgn0262481	NA	FBtr0307055
FBgn0262482	FBgn0262482	NA	FBtr0304821
FBgn0262483	FBgn0262483	NA	FBtr0304826
FBgn0262484	FBgn0262484	NA	FBtr0304829
FBgn0262485	FBgn0262485	NA	FBtr0304838
FBgn0262486	FBgn0262486	NA	FBtr0304837
FBgn0262487	FBgn0262487	NA	FBtr0304841
FBgn0262508	FBgn0262508	CG32355	FBtr0304815
FBgn0262509	FBgn0262509	NA	FBtr0304833
FBgn0262510	FBgn0262510	NA	FBtr0305350
FBgn0262516	FBgn0262516	NA	FBtr0074952
FBgn0262518	FBgn0262518	Rab8	FBtr0074921
FBgn0262526	FBgn0262526	vas	FBtr0304855
FBgn0262527	FBgn0262527	NA	FBtr0301732
FBgn0262528	FBgn0262528	NA	FBtr0304968
FBgn0262529	FBgn0262529	NA	FBtr0304969
FBgn0262530	FBgn0262530	NA	FBtr0304970
FBgn0262531	FBgn0262531	NA	FBtr0304976
FBgn0262532	FBgn0262532	NA	FBtr0304978
FBgn0262533	FBgn0262533	NA	FBtr0304979
FBgn0262534	FBgn0262534	NA	FBtr0304873
FBgn0262535	FBgn0262535	NA	FBtr0308636
FBgn0262536	FBgn0262536	NA	FBtr0304876
FBgn0262537	FBgn0262537	NA	FBtr0304881
FBgn0262538	FBgn0262538	NA	FBtr0304880
FBgn0262539	FBgn0262539	NA	FBtr0304879
FBgn0262540	FBgn0262540	NA	FBtr0304878
FBgn0262541	FBgn0262541	NA	FBtr0304877
FBgn0262542	FBgn0262542	NA	FBtr0304882
FBgn0262543	FBgn0262543	NA	FBtr0304883
FBgn0262544	FBgn0262544	NA	FBtr0304986
FBgn0262545	FBgn0262545	NA	FBtr0304884
FBgn0262546	FBgn0262546	NA	FBtr0304886
FBgn0262547	FBgn0262547	NA	FBtr0304888
FBgn0262562	FBgn0262562	NA	FBtr0305010
FBgn0262563	FBgn0262563	NA	FBtr0304891
FBgn0262564	FBgn0262564	NA	FBtr0304892
FBgn0262565	FBgn0262565	NA	FBtr0306724
FBgn0262566	FBgn0262566	NA	FBtr0304889
FBgn0262567	FBgn0262567	NA	FBtr0304890
FBgn0262568	FBgn0262568	NA	FBtr0304895
FBgn0262569	FBgn0262569	NA	FBtr0304896
FBgn0262570	FBgn0262570	NA	FBtr0304897
FBgn0262571	FBgn0262571	NA	FBtr0304898
FBgn0262572	FBgn0262572	NA	FBtr0304907
FBgn0262573	FBgn0262573	NA	FBtr0304944
FBgn0262574	FBgn0262574	NA	FBtr0304945
FBgn0262575	FBgn0262575	NA	FBtr0304946
FBgn0262576	FBgn0262576	NA	FBtr0305046
FBgn0262577	FBgn0262577	NA	FBtr0305048
FBgn0262579	FBgn0262579	Ect4	FBtr0304998
FBgn0262580	FBgn0262580	NA	FBtr0304959
FBgn0262581	FBgn0262581	NA	FBtr0304960
FBgn0262582	FBgn0262582	NA	FBtr0305029

FBgn0262583	FBgn0262583	NA	FBtr0305124
FBgn0262587	FBgn0262587	NA	FBtr0305116
FBgn0262588	FBgn0262588	NA	FBtr0305117
FBgn0262592	FBgn0262592	NA	FBtr0305199
FBgn0262593	FBgn0262593	NA	FBtr0305204
FBgn0262599	FBgn0262599	CG17086	FBtr0305258
FBgn0262603	FBgn0262603	NA	FBtr0078677
FBgn0262604	FBgn0262604	NA	FBtr0305278
FBgn0262605	FBgn0262605	NA	FBtr0305279
FBgn0262606	FBgn0262606	NA	FBtr0305280
FBgn0262607	FBgn0262607	NA	FBtr0305281
FBgn0262608	FBgn0262608	NA	FBtr0305282
FBgn0262609	FBgn0262609	NA	FBtr0305283
FBgn0262610	FBgn0262610	NA	FBtr0305284
FBgn0262611	FBgn0262611	NA	FBtr0305285
FBgn0262612	FBgn0262612	NA	FBtr0305286
FBgn0262613	FBgn0262613	NA	FBtr0305291
FBgn0262614	FBgn0262614	pyd	FBtr0310205
FBgn0262616	FBgn0262616	NA	FBtr0305347
FBgn0262617	FBgn0262617	NA	FBtr0305355
FBgn0262620	FBgn0262620	NA	FBtr0305481
FBgn0262621	FBgn0262621	NA	FBtr0305483
FBgn0262622	FBgn0262622	NA	FBtr0305484
FBgn0262623	FBgn0262623	NA	FBtr0305486
FBgn0262631	FBgn0262631	NA	FBtr0309039
FBgn0262636	FBgn0262636	NA	FBtr0307045
FBgn0262679	FBgn0262679	NA	FBtr0305596
FBgn0262680	FBgn0262680	NA	FBtr0305594
FBgn0262681	FBgn0262681	NA	FBtr0305597
FBgn0262682	FBgn0262682	NA	FBtr0309992
FBgn0262683	FBgn0262683	NA	FBtr0305602
FBgn0262684	FBgn0262684	NA	FBtr0305606
FBgn0262685	FBgn0262685	NA	FBtr0305607
FBgn0262686	FBgn0262686	NA	FBtr0305610
FBgn0262687	FBgn0262687	NA	FBtr0305608
FBgn0262688	FBgn0262688	NA	FBtr0305612
FBgn0262689	FBgn0262689	NA	FBtr0305611
FBgn0262690	FBgn0262690	NA	FBtr0305615
FBgn0262717	FBgn0262717	NA	FBtr0305665
FBgn0262718	FBgn0262718	NA	FBtr0305694
FBgn0262719	FBgn0262719	NA	FBtr0308608
FBgn0262720	FBgn0262720	NA	FBtr0305701
FBgn0262721	FBgn0262721	NA	FBtr0305706
FBgn0262722	FBgn0262722	NA	FBtr0305707
FBgn0262730	FBgn0262730	NA	FBtr0307164
FBgn0262735	FBgn0262735	Imp	FBtr0305150
FBgn0262741	FBgn0262741	NA	FBtr0309075
FBgn0262786	FBgn0262786	NA	FBtr0305896
FBgn0262787	FBgn0262787	NA	FBtr0305900
FBgn0262788	FBgn0262788	NA	FBtr0305902
FBgn0262789	FBgn0262789	NA	FBtr0305903
FBgn0262790	FBgn0262790	NA	FBtr0305906
FBgn0262791	FBgn0262791	NA	FBtr0305907
FBgn0262792	FBgn0262792	NA	FBtr0305908
FBgn0262793	FBgn0262793	NA	FBtr0305909
FBgn0262794	FBgn0262794	NA	FBtr0305917
FBgn0262795	FBgn0262795	NA	FBtr0308919

FBgn0262806	FBgn0262806	NA	FBtr0305947
FBgn0262807	FBgn0262807	NA	FBtr0305948
FBgn0262808	FBgn0262808	NA	FBtr0306052
FBgn0262810	FBgn0262810	NA	FBtr0306054
FBgn0262811	FBgn0262811	NA	FBtr0306055
FBgn0262812	FBgn0262812	NA	FBtr0306056
FBgn0262813	FBgn0262813	NA	FBtr0306058
FBgn0262814	FBgn0262814	NA	FBtr0306046
FBgn0262815	FBgn0262815	NA	FBtr0306723
FBgn0262816	FBgn0262816	NA	FBtr0306048
FBgn0262817	FBgn0262817	NA	FBtr0306049
FBgn0262818	FBgn0262818	NA	FBtr0306050
FBgn0262819	FBgn0262819	NA	FBtr0306061
FBgn0262820	FBgn0262820	NA	FBtr0306063
FBgn0262821	FBgn0262821	NA	FBtr0306064
FBgn0262822	FBgn0262822	NA	FBtr0306065
FBgn0262823	FBgn0262823	NA	FBtr0306140
FBgn0262824	FBgn0262824	NA	FBtr0306044
FBgn0262825	FBgn0262825	NA	FBtr0306045
FBgn0262827	FBgn0262827	NA	FBtr0306067
FBgn0262828	FBgn0262828	NA	FBtr0306066
FBgn0262836	FBgn0262836	NA	FBtr0306077
FBgn0262837	FBgn0262837	NA	FBtr0306076
FBgn0262838	FBgn0262838	NA	FBtr0306078
FBgn0262839	FBgn0262839	NA	FBtr0306081
FBgn0262840	FBgn0262840	NA	FBtr0306082
FBgn0262842	FBgn0262842	NA	FBtr0306084
FBgn0262843	FBgn0262843	NA	FBtr0306079
FBgn0262844	FBgn0262844	NA	FBtr0306120
FBgn0262845	FBgn0262845	NA	FBtr0306121
FBgn0262846	FBgn0262846	NA	FBtr0306142
FBgn0262847	FBgn0262847	NA	FBtr0306149
FBgn0262848	FBgn0262848	NA	FBtr0306148
FBgn0262849	FBgn0262849	NA	FBtr0306147
FBgn0262850	FBgn0262850	NA	FBtr0306146
FBgn0262851	FBgn0262851	NA	FBtr0306145
FBgn0262852	FBgn0262852	NA	FBtr0306144
FBgn0262853	FBgn0262853	NA	FBtr0306143
FBgn0262854	FBgn0262854	NA	FBtr0306159
FBgn0262855	FBgn0262855	NA	FBtr0306161
FBgn0262856	FBgn0262856	NA	FBtr0310202
FBgn0262857	FBgn0262857	NA	FBtr0306163
FBgn0262858	FBgn0262858	NA	FBtr0306166
FBgn0262867	FBgn0262867	Ptr	FBtr0307501
FBgn0262868	FBgn0262868	NA	FBtr0306213
FBgn0262869	FBgn0262869	NA	FBtr0306201
FBgn0262870	FBgn0262870	NA	FBtr0302646
FBgn0262871	FBgn0262871	NA	FBtr0306210
FBgn0262872	FBgn0262872	milt	FBtr0306231
FBgn0262873	FBgn0262873	NA	FBtr0306279
FBgn0262874	FBgn0262874	NA	FBtr0306280
FBgn0262875	FBgn0262875	NA	FBtr0306282
FBgn0262876	FBgn0262876	NA	FBtr0306283
FBgn0262877	FBgn0262877	NA	FBtr0306284
FBgn0262878	FBgn0262878	NA	FBtr0306287
FBgn0262879	FBgn0262879	NA	FBtr0306286
FBgn0262880	FBgn0262880	NA	FBtr0306288

FBgn0262881	FBgn0262881	NA	FBtr0306289
FBgn0262882	FBgn0262882	NA	FBtr0306290
FBgn0262883	FBgn0262883	NA	FBtr0306291
FBgn0262884	FBgn0262884	NA	FBtr0306294
FBgn0262885	FBgn0262885	NA	FBtr0306298
FBgn0262886	FBgn0262886	NA	FBtr0306297
FBgn0262887	FBgn0262887	NA	FBtr0306299
FBgn0262888	FBgn0262888	NA	FBtr0306302
FBgn0262889	FBgn0262889	NA	FBtr0306303
FBgn0262890	FBgn0262890	NA	FBtr0306304
FBgn0262891	FBgn0262891	NA	FBtr0306305
FBgn0262892	FBgn0262892	NA	FBtr0306306
FBgn0262893	FBgn0262893	NA	FBtr0306309
FBgn0262894	FBgn0262894	NA	FBtr0306310
FBgn0262895	FBgn0262895	NA	FBtr0306311
FBgn0262896	FBgn0262896	NA	FBtr0306312
FBgn0262897	FBgn0262897	NA	FBtr0306313
FBgn0262898	FBgn0262898	NA	FBtr0306318
FBgn0262899	FBgn0262899	NA	FBtr0310579
FBgn0262900	FBgn0262900	NA	FBtr0306324
FBgn0262901	FBgn0262901	NA	FBtr0306314
FBgn0262902	FBgn0262902	NA	FBtr0306316
FBgn0262903	FBgn0262903	NA	FBtr0306319
FBgn0262904	FBgn0262904	NA	FBtr0306322
FBgn0262905	FBgn0262905	NA	FBtr0306325
FBgn0262906	FBgn0262906	NA	FBtr0309897
FBgn0262943	FBgn0262943	NA	FBtr0306547
FBgn0262944	FBgn0262944	NA	FBtr0306546
FBgn0262945	FBgn0262945	NA	FBtr0306548
FBgn0262946	FBgn0262946	NA	FBtr0306553
FBgn0262947	FBgn0262947	NA	FBtr0306552
FBgn0262948	FBgn0262948	NA	FBtr0306554
FBgn0262950	FBgn0262950	NA	FBtr0306791
FBgn0262951	FBgn0262951	NA	FBtr0306851
FBgn0262960	FBgn0262960	NA	FBtr0306713
FBgn0262961	FBgn0262961	NA	FBtr0306715
FBgn0262962	FBgn0262962	NA	FBtr0306717
FBgn0262963	FBgn0262963	NA	FBtr0306710
FBgn0262964	FBgn0262964	NA	FBtr0306709
FBgn0262965	FBgn0262965	NA	FBtr0306716
FBgn0262966	FBgn0262966	NA	FBtr0306718
FBgn0262967	FBgn0262967	NA	FBtr0306720
FBgn0262968	FBgn0262968	NA	FBtr0306719
FBgn0262969	FBgn0262969	NA	FBtr0306721
FBgn0262970	FBgn0262970	NA	FBtr0306722
FBgn0262971	FBgn0262971	NA	FBtr0308067
FBgn0262972	FBgn0262972	NA	FBtr0306729
FBgn0262974	FBgn0262974	NA	FBtr0306736
FBgn0262975	FBgn0262975	cnc	FBtr0306754
FBgn0262976	FBgn0262976	NA	FBtr0307286
FBgn0262979	FBgn0262979	NA	FBtr0306815
FBgn0262980	FBgn0262980	NA	FBtr0306816
FBgn0262981	FBgn0262981	NA	FBtr0306817
FBgn0262982	FBgn0262982	NA	FBtr0306818
FBgn0262983	FBgn0262983	NA	FBtr0306819
FBgn0262984	FBgn0262984	NA	FBtr0306820
FBgn0262985	FBgn0262985	NA	FBtr0306821

FBgn0262986	FBgn0262986	NA	FBtr0306824
FBgn0262987	FBgn0262987	NA	FBtr0306825
FBgn0262988	FBgn0262988	NA	FBtr0306826
FBgn0262989	FBgn0262989	NA	FBtr0306827
FBgn0262990	FBgn0262990	NA	FBtr0306828
FBgn0262991	FBgn0262991	NA	FBtr0306830
FBgn0262992	FBgn0262992	NA	FBtr0306832
FBgn0262993	FBgn0262993	NA	FBtr0306833
FBgn0262994	FBgn0262994	NA	FBtr0306837
FBgn0262995	FBgn0262995	NA	FBtr0306835
FBgn0262996	FBgn0262996	NA	FBtr0306838
FBgn0262998	FBgn0262998	NA	FBtr0306840
FBgn0262999	FBgn0262999	NA	FBtr0306842
FBgn0263000	FBgn0263000	NA	FBtr0306843
FBgn0263001	FBgn0263001	NA	FBtr0306844
FBgn0263002	FBgn0263002	NA	FBtr0306845
FBgn0263003	FBgn0263003	NA	FBtr0306846
FBgn0263004	FBgn0263004	NA	FBtr0306848
FBgn0263005	FBgn0263005	NA	FBtr0306847
FBgn0263019	FBgn0263019	NA	FBtr0306899
FBgn0263020	FBgn0263020	NA	FBtr0306900
FBgn0263021	FBgn0263021	NA	FBtr0306902
FBgn0263022	FBgn0263022	NA	FBtr0306903
FBgn0263023	FBgn0263023	NA	FBtr0306904
FBgn0263024	FBgn0263024	NA	FBtr0306923
FBgn0263025	FBgn0263025	NA	FBtr0306924
FBgn0263026	FBgn0263026	NA	FBtr0306925
FBgn0263027	FBgn0263027	NA	FBtr0306926
FBgn0263028	FBgn0263028	NA	FBtr0306906
FBgn0263029	FBgn0263029	NA	FBtr0306907
FBgn0263030	FBgn0263030	NA	FBtr0306908
FBgn0263031	FBgn0263031	NA	FBtr0306911
FBgn0263032	FBgn0263032	NA	FBtr0306912
FBgn0263033	FBgn0263033	NA	FBtr0306913
FBgn0263034	FBgn0263034	NA	FBtr0306914
FBgn0263035	FBgn0263035	NA	FBtr0306915
FBgn0263036	FBgn0263036	NA	FBtr0306916
FBgn0263037	FBgn0263037	NA	FBtr0306928
FBgn0263038	FBgn0263038	CG32972	FBtr0306927
FBgn0263039	FBgn0263039	NA	FBtr0306918
FBgn0263040	FBgn0263040	NA	FBtr0306929
FBgn0263041	FBgn0263041	NA	FBtr0306930
FBgn0263042	FBgn0263042	NA	FBtr0306919
FBgn0263043	FBgn0263043	NA	FBtr0306922
FBgn0263044	FBgn0263044	NA	FBtr0306920
FBgn0263045	FBgn0263045	NA	FBtr0306941
FBgn0263046	FBgn0263046	NA	FBtr0306944
FBgn0263047	FBgn0263047	NA	FBtr0306948
FBgn0263048	FBgn0263048	NA	FBtr0306946
FBgn0263049	FBgn0263049	NA	FBtr0306973
FBgn0263050	FBgn0263050	NA	FBtr0306974
FBgn0263051	FBgn0263051	CG9243	FBtr0306975
FBgn0263072	FBgn0263072	NA	FBtr0307022
FBgn0263080	FBgn0263080	NA	FBtr0307096
FBgn0263081	FBgn0263081	NA	FBtr0307097
FBgn0263082	FBgn0263082	NA	FBtr0307098
FBgn0263083	FBgn0263083	NA	FBtr0307099

FBgn0263084	FBgn0263084	NA	FBtr0307100
FBgn0263085	FBgn0263085	NA	FBtr0307102
FBgn0263086	FBgn0263086	NA	FBtr0307101
FBgn0263087	FBgn0263087	NA	FBtr0308135
FBgn0263088	FBgn0263088	NA	FBtr0307106
FBgn0263089	FBgn0263089	NA	FBtr0307107
FBgn0263090	FBgn0263090	NA	FBtr0307108
FBgn0263091	FBgn0263091	NA	FBtr0307110
FBgn0263092	FBgn0263092	NA	FBtr0307111
FBgn0263093	FBgn0263093	NA	FBtr0307112
FBgn0263094	FBgn0263094	NA	FBtr0307109
FBgn0263095	FBgn0263095	NA	FBtr0307113
FBgn0263096	FBgn0263096	NA	FBtr0307120
FBgn0263108	FBgn0263108	NA	FBtr0307304
FBgn0263109	FBgn0263109	CG14470	FBtr0307307
FBgn0263110	FBgn0263110	CG1308	FBtr0307311
FBgn0263111	FBgn0263111	cac	FBtr0307325
FBgn0263112	FBgn0263112	NA	FBtr0307326
FBgn0263113	FBgn0263113	NA	FBtr0307337
FBgn0263114	FBgn0263114	NA	FBtr0307338
FBgn0263117	FBgn0263117	NA	FBtr0112610
FBgn0263130	FBgn0263130	NA	FBtr0307362
FBgn0263131	FBgn0263131	CG32158	FBtr0307367
FBgn0263132	FBgn0263132	NA	FBtr0307374
FBgn0263143	FBgn0263143	NA	FBtr0084323
FBgn0263177	FBgn0263177	NA	FBtr0307533
FBgn0263201	FBgn0263201	NA	FBtr0309751
FBgn0263206	FBgn0263206	NA	FBtr0307577
FBgn0263207	FBgn0263207	NA	FBtr0307587
FBgn0263208	FBgn0263208	NA	FBtr0307586
FBgn0263209	FBgn0263209	NA	FBtr0307585
FBgn0263210	FBgn0263210	NA	FBtr0307584
FBgn0263211	FBgn0263211	NA	FBtr0307583
FBgn0263212	FBgn0263212	NA	FBtr0307582
FBgn0263213	FBgn0263213	NA	FBtr0307581
FBgn0263214	FBgn0263214	NA	FBtr0307580
FBgn0263215	FBgn0263215	NA	FBtr0307589
FBgn0263216	FBgn0263216	CG15251	FBtr0307593
FBgn0263217	FBgn0263217	NA	FBtr0307594
FBgn0263220	FBgn0263220	Hk	FBtr0307876
FBgn0263235	FBgn0263235	NA	FBtr0302854
FBgn0263246	FBgn0263246	NA	FBtr0308062
FBgn0263247	FBgn0263247	NA	FBtr0308059
FBgn0263248	FBgn0263248	NA	FBtr0308058
FBgn0263249	FBgn0263249	NA	FBtr0308060
FBgn0263250	FBgn0263250	NA	FBtr0308240
FBgn0263256	FBgn0263256	NA	FBtr0308299
FBgn0263257	FBgn0263257	NA	FBtr0308309
FBgn0263270	FBgn0263270	NA	FBtr0308492
FBgn0263271	FBgn0263271	NA	FBtr0308493
FBgn0263289	FBgn0263289	CG5467	FBtr0308630
FBgn0263290	FBgn0263290	NA	FBtr0308635
FBgn0263314	FBgn0263314	NA	FBtr0308708
FBgn0263320	FBgn0263320	NA	FBtr0308744
FBgn0263321	FBgn0263321	NA	FBtr0308745
FBgn0263322	FBgn0263322	NA	FBtr0308746
FBgn0263323	FBgn0263323	NA	FBtr0308747

FBgn0263324	FBgn0263324	NA	FBtr0308749
FBgn0263325	FBgn0263325	NA	FBtr0308757
FBgn0263326	FBgn0263326	NA	FBtr0308782
FBgn0263327	FBgn0263327	NA	FBtr0308758
FBgn0263328	FBgn0263328	NA	FBtr0308751
FBgn0263329	FBgn0263329	NA	FBtr0308752
FBgn0263330	FBgn0263330	NA	FBtr0308753
FBgn0263331	FBgn0263331	NA	FBtr0308754
FBgn0263332	FBgn0263332	NA	FBtr0308755
FBgn0263333	FBgn0263333	NA	FBtr0308759
FBgn0263334	FBgn0263334	NA	FBtr0308761
FBgn0263335	FBgn0263335	NA	FBtr0308763
FBgn0263336	FBgn0263336	NA	FBtr0308766
FBgn0263337	FBgn0263337	NA	FBtr0308767
FBgn0263338	FBgn0263338	NA	FBtr0308768
FBgn0263339	FBgn0263339	NA	FBtr0308769
FBgn0263340	FBgn0263340	NA	FBtr0308772
FBgn0263341	FBgn0263341	NA	FBtr0308773
FBgn0263342	FBgn0263342	NA	FBtr0308775
FBgn0263343	FBgn0263343	NA	FBtr0308777
FBgn0263344	FBgn0263344	NA	FBtr0308778
FBgn0263345	FBgn0263345	NA	FBtr0308779
FBgn0263346	FBgn0263346	CG31534	FBtr0308858
FBgn0263351	FBgn0263351	AP-50	FBtr0084253
FBgn0263376	FBgn0263376	NA	FBtr0309057
FBgn0263377	FBgn0263377	NA	FBtr0309058
FBgn0263378	FBgn0263378	NA	FBtr0309059
FBgn0263379	FBgn0263379	NA	FBtr0309061
FBgn0263380	FBgn0263380	NA	FBtr0309066
FBgn0263381	FBgn0263381	NA	FBtr0309067
FBgn0263382	FBgn0263382	NA	FBtr0309068
FBgn0263383	FBgn0263383	NA	FBtr0309070
FBgn0263384	FBgn0263384	NA	FBtr0309071
FBgn0263385	FBgn0263385	NA	FBtr0309072
FBgn0263386	FBgn0263386	NA	FBtr0309073
FBgn0263387	FBgn0263387	NA	FBtr0309074
FBgn0263388	FBgn0263388	NA	FBtr0309076
FBgn0263389	FBgn0263389	NA	FBtr0309077
FBgn0263390	FBgn0263390	NA	FBtr0309101
FBgn0263391	FBgn0263391	NA	FBtr0309113
FBgn0263392	FBgn0263392	CG2083	FBtr0309117
FBgn0263399	FBgn0263399	NA	FBtr0309175
FBgn0263400	FBgn0263400	NA	FBtr0309177
FBgn0263401	FBgn0263401	NA	FBtr0309178
FBgn0263402	FBgn0263402	NA	FBtr0309181
FBgn0263403	FBgn0263403	NA	FBtr0309183
FBgn0263404	FBgn0263404	NA	FBtr0309189
FBgn0263405	FBgn0263405	NA	FBtr0309184
FBgn0263406	FBgn0263406	NA	FBtr0309185
FBgn0263407	FBgn0263407	NA	FBtr0309186
FBgn0263408	FBgn0263408	NA	FBtr0309187
FBgn0263409	FBgn0263409	NA	FBtr0309188
FBgn0263410	FBgn0263410	NA	FBtr0309191
FBgn0263411	FBgn0263411	NA	FBtr0309190
FBgn0263412	FBgn0263412	NA	FBtr0309193
FBgn0263413	FBgn0263413	NA	FBtr0309197
FBgn0263414	FBgn0263414	NA	FBtr0309199

FBgn0263415	FBgn0263415	NA	FBtr0309201
FBgn0263416	FBgn0263416	NA	FBtr0309233
FBgn0263440	FBgn0263440	NA	FBtr0309283
FBgn0263441	FBgn0263441	NA	FBtr0309284
FBgn0263442	FBgn0263442	NA	FBtr0309287
FBgn0263443	FBgn0263443	NA	FBtr0309289
FBgn0263444	FBgn0263444	NA	FBtr0309291
FBgn0263445	FBgn0263445	NA	FBtr0309293
FBgn0263446	FBgn0263446	NA	FBtr0309292
FBgn0263447	FBgn0263447	NA	FBtr0309297
FBgn0263448	FBgn0263448	NA	FBtr0309300
FBgn0263449	FBgn0263449	NA	FBtr0309304
FBgn0263450	FBgn0263450	NA	FBtr0309313
FBgn0263451	FBgn0263451	NA	FBtr0309306
FBgn0263452	FBgn0263452	NA	FBtr0309305
FBgn0263453	FBgn0263453	NA	FBtr0309309
FBgn0263454	FBgn0263454	NA	FBtr0309310
FBgn0263455	FBgn0263455	NA	FBtr0309311
FBgn0263456	FBgn0263456	nwk	FBtr0309280
FBgn0263457	FBgn0263457	NA	FBtr0309315
FBgn0263458	FBgn0263458	NA	FBtr0309757
FBgn0263459	FBgn0263459	NA	FBtr0309758
FBgn0263460	FBgn0263460	NA	FBtr0309759
FBgn0263461	FBgn0263461	NA	FBtr0309760
FBgn0263462	FBgn0263462	NA	FBtr0309761
FBgn0263463	FBgn0263463	NA	FBtr0309762
FBgn0263464	FBgn0263464	NA	FBtr0309763
FBgn0263465	FBgn0263465	NA	FBtr0309764
FBgn0263466	FBgn0263466	NA	FBtr0309765
FBgn0263467	FBgn0263467	NA	FBtr0309766
FBgn0263468	FBgn0263468	NA	FBtr0309767
FBgn0263469	FBgn0263469	NA	FBtr0309768
FBgn0263470	FBgn0263470	NA	FBtr0309769
FBgn0263471	FBgn0263471	NA	FBtr0309770
FBgn0263472	FBgn0263472	NA	FBtr0309771
FBgn0263473	FBgn0263473	NA	FBtr0309772
FBgn0263474	FBgn0263474	NA	FBtr0309773
FBgn0263475	FBgn0263475	NA	FBtr0309774
FBgn0263476	FBgn0263476	NA	FBtr0309775
FBgn0263477	FBgn0263477	NA	FBtr0309776
FBgn0263478	FBgn0263478	NA	FBtr0309777
FBgn0263479	FBgn0263479	NA	FBtr0309778
FBgn0263480	FBgn0263480	NA	FBtr0309779
FBgn0263481	FBgn0263481	NA	FBtr0309780
FBgn0263482	FBgn0263482	NA	FBtr0309781
FBgn0263483	FBgn0263483	NA	FBtr0309782
FBgn0263484	FBgn0263484	NA	FBtr0309783
FBgn0263485	FBgn0263485	NA	FBtr0309784
FBgn0263486	FBgn0263486	NA	FBtr0309785
FBgn0263487	FBgn0263487	NA	FBtr0309786
FBgn0263488	FBgn0263488	NA	FBtr0309787
FBgn0263489	FBgn0263489	NA	FBtr0309788
FBgn0263492	FBgn0263492	NA	FBtr0309503
FBgn0263493	FBgn0263493	NA	FBtr0309505
FBgn0263494	FBgn0263494	NA	FBtr0309507
FBgn0263495	FBgn0263495	NA	FBtr0309508
FBgn0263496	FBgn0263496	NA	FBtr0309510

FBgn0263497	FBgn0263497	NA	FBtr0309515
FBgn0263498	FBgn0263498	NA	FBtr0309514
FBgn0263499	FBgn0263499	NA	FBtr0309521
FBgn0263500	FBgn0263500	NA	FBtr0309518
FBgn0263501	FBgn0263501	NA	FBtr0309517
FBgn0263502	FBgn0263502	NA	FBtr0309522
FBgn0263503	FBgn0263503	NA	FBtr0309523
FBgn0263504	FBgn0263504	NA	FBtr0309524
FBgn0263505	FBgn0263505	NA	FBtr0309525
FBgn0263506	FBgn0263506	NA	FBtr0309526
FBgn0263507	FBgn0263507	NA	FBtr0309527
FBgn0263508	FBgn0263508	NA	FBtr0309529
FBgn0263509	FBgn0263509	NA	FBtr0309530
FBgn0263580	FBgn0263580	NA	FBtr0309816
FBgn0263581	FBgn0263581	NA	FBtr0309806
FBgn0263582	FBgn0263582	NA	FBtr0309807
FBgn0263583	FBgn0263583	NA	FBtr0309809
FBgn0263584	FBgn0263584	NA	FBtr0309810
FBgn0263585	FBgn0263585	NA	FBtr0309811
FBgn0263586	FBgn0263586	NA	FBtr0309820
FBgn0263587	FBgn0263587	NA	FBtr0309823
FBgn0263588	FBgn0263588	NA	FBtr0309824
FBgn0263589	FBgn0263589	NA	FBtr0309825
FBgn0263590	FBgn0263590	NA	FBtr0309826
FBgn0263595	FBgn0263595	NA	FBtr0309896
FBgn0263596	FBgn0263596	NA	FBtr0309894
FBgn0263597	FBgn0263597	NA	FBtr0310089
FBgn0263603	FBgn0263603	Zn72D	FBtr0075493
FBgn0263611	FBgn0263611	NA	FBtr0309974
FBgn0263612	FBgn0263612	NA	FBtr0309976
FBgn0263613	FBgn0263613	NA	FBtr0309977
FBgn0263615	FBgn0263615	NA	FBtr0309979
FBgn0263616	FBgn0263616	NA	FBtr0309981
FBgn0263617	FBgn0263617	NA	FBtr0309982
FBgn0263618	FBgn0263618	NA	FBtr0309984
FBgn0263619	FBgn0263619	NA	FBtr0309985
FBgn0263620	FBgn0263620	NA	FBtr0309986
FBgn0263621	FBgn0263621	NA	FBtr0309980
FBgn0263622	FBgn0263622	NA	FBtr0309990
FBgn0263623	FBgn0263623	NA	FBtr0309989
FBgn0263624	FBgn0263624	NA	FBtr0309988
FBgn0263625	FBgn0263625	NA	FBtr0309987
FBgn0263626	FBgn0263626	NA	FBtr0309991
FBgn0263627	FBgn0263627	NA	FBtr0309993
FBgn0263628	FBgn0263628	NA	FBtr0309994
FBgn0263647	FBgn0263647	NA	FBtr0310008
FBgn0263648	FBgn0263648	NA	FBtr0310012
FBgn0263649	FBgn0263649	NA	FBtr0310013
FBgn0263650	FBgn0263650	NA	FBtr0310042
FBgn0263651	FBgn0263651	NA	FBtr0310043
FBgn0263652	FBgn0263652	NA	FBtr0310085
FBgn0263653	FBgn0263653	NA	FBtr0310084
FBgn0263654	FBgn0263654	NA	FBtr0310090
FBgn0263655	FBgn0263655	NA	FBtr0310091
FBgn0263656	FBgn0263656	NA	FBtr0310092
FBgn0263657	FBgn0263657	NA	FBtr0310099
FBgn0263658	FBgn0263658	NA	FBtr0310101

FBgn0263659	FBgn0263659	NA	FBtr0310102
FBgn0263660	FBgn0263660	NA	FBtr0310105
FBgn0263661	FBgn0263661	NA	FBtr0310106
FBgn0263662	FBgn0263662	NA	FBtr0310107
FBgn0263663	FBgn0263663	NA	FBtr0310108
FBgn0263664	FBgn0263664	NA	FBtr0310111
FBgn0263665	FBgn0263665	NA	FBtr0310112
FBgn0263705	FBgn0263705	NA	FBtr0310334
FBgn0263706	FBgn0263706	NA	FBtr0310335
FBgn0263707	FBgn0263707	NA	FBtr0310345
FBgn0263708	FBgn0263708	NA	FBtr0310349
FBgn0263709	FBgn0263709	NA	FBtr0310348
FBgn0263737	FBgn0263737	NA	FBtr0310379
FBgn0263738	FBgn0263738	NA	FBtr0310381
FBgn0263739	FBgn0263739	NA	FBtr0310385
FBgn0263740	FBgn0263740	NA	FBtr0310386
FBgn0263741	FBgn0263741	NA	FBtr0310390
FBgn0263742	FBgn0263742	NA	FBtr0310389
FBgn0263743	FBgn0263743	NA	FBtr0310388
FBgn0263744	FBgn0263744	NA	FBtr0310393
FBgn0263745	FBgn0263745	NA	FBtr0310392
FBgn0263746	FBgn0263746	NA	FBtr0310391
FBgn0263747	FBgn0263747	NA	FBtr0310394
FBgn0263748	FBgn0263748	NA	FBtr0310395
FBgn0263749	FBgn0263749	NA	FBtr0310397
FBgn0263750	FBgn0263750	NA	FBtr0310402
FBgn0263759	FBgn0263759	NA	FBtr0310453
FBgn0263760	FBgn0263760	NA	FBtr0310452
FBgn0263761	FBgn0263761	NA	FBtr0310458
FBgn0263762	FBgn0263762	NA	FBtr0310456
FBgn0263763	FBgn0263763	NA	FBtr0310455
FBgn0263764	FBgn0263764	NA	FBtr0310478
FBgn0263765	FBgn0263765	NA	FBtr0310480
FBgn0263766	FBgn0263766	NA	FBtr0310481
FBgn0263767	FBgn0263767	NA	FBtr0310484
FBgn0263768	FBgn0263768	NA	FBtr0310485
FBgn0263769	FBgn0263769	NA	FBtr0310488
FBgn0263770	FBgn0263770	NA	FBtr0310489
FBgn0263771	FBgn0263771	NA	FBtr0310490
FBgn0263772	FBgn0263772	NA	FBtr0310495
FBgn0263773	FBgn0263773	fok	FBtr0310559
FBgn0263774	FBgn0263774	NA	FBtr0310562
FBgn0263775	FBgn0263775	NA	FBtr0310574
FBgn0263776	FBgn0263776	CG6327	FBtr0310566
FBgn0263777	FBgn0263777	NA	FBtr0310577
FBgn0263778	FBgn0263778	NA	FBtr0310578
FBgn0263836	FBgn0263836	NA	FBtr0321279
FBgn0263837	FBgn0263837	NA	FBtr0321278
FBgn0263838	FBgn0263838	NA	FBtr0321273
FBgn0263839	FBgn0263839	NA	FBtr0321280
FBgn0263841	FBgn0263841	NA	FBtr0321303
FBgn0263842	FBgn0263842	NA	FBtr0321298
FBgn0263843	FBgn0263843	NA	FBtr0321299
FBgn0263844	FBgn0263844	NA	FBtr0321301
FBgn0263845	FBgn0263845	NA	FBtr0321300
FBgn0263846	FBgn0263846	NA	FBtr0321304
FBgn0263847	FBgn0263847	NA	FBtr0321322

FBgn0263848	FBgn0263848	NA	FBtr0321317
FBgn0263849	FBgn0263849	NA	FBtr0321316
FBgn0263850	FBgn0263850	NA	FBtr0321315
FBgn0263851	FBgn0263851	NA	FBtr0321323
FBgn0263866	FBgn0263866	NA	FBtr0329835
FBgn0263867	FBgn0263867	NA	FBtr0329836
FBgn0263868	FBgn0263868	NA	FBtr0329837
FBgn0263869	FBgn0263869	NA	FBtr0329834
FBgn0263870	FBgn0263870	NA	FBtr0329833
FBgn0263871	FBgn0263871	NA	FBtr0329831
FBgn0263872	FBgn0263872	NA	FBtr0329830
FBgn0263873	FBgn0263873	CG33316	FBtr0329846
FBgn0263874	FBgn0263874	NA	FBtr0329848

rs preceeds the data themselves.

. 2010).

s)
FBr5.43? (0 = no, 1 = yes)

mecourse.
ecourse.
ly et al. 2010).
vely et al. 2010).
. 2010).
al. 2010).
cted SOLiD data from Gravely et al. 2010.
ected SOLiD data from Gravely et al. 2010.
cted SOLiD data from Gravely et al. 2010.
ected SOLiD data from Gravely et al. 2010.
OLiD data from Gravely et al. 2010.
and D. virilis? (0 = no; 1 = yes)

a et al. 2010)? (0 = no; 1 = yes)
9 time points in the species timecourse (Kalinka et al. 2010)
time points in the species timecourse (Kalinka et al. 2010)
ne 9 time points in the species timecourse (Kalinka et al. 2010)
points in the species timecourse (Kalinka et al. 2010)

= yes)

th.
3)

DMEL_LONGEST_TRANS_I	DMEL_PERC_G	DMEL_LENGTH	DMEL_LENGTH	DMEL_SINGLE_
3209	0.525316456	826	1000	1

1780	0.483537766	NA	NA		1
1222	0.532743363	NA	NA		1
27133	0.501848856	NA	NA		1
1692	0.466312057	NA	NA		1
8836	0.447195443	384		1000	0
3471	0.437009691	558		775	0
918	0.521525215	NA	NA		1
1040	0.559217302	NA	NA		1
3650	0.489507959	NA	NA		1
20253	0.581739667	638		996	1
939	0.464323749	NA	NA		1
2811	0.536231884	1000		949	1
2702	0.538272616	938		929	1
3370	0.482831708	757		944	0
4100	0.421206226	491		1000	1
5256	0.514187446	NA	NA		1
2866	0.512967513	822		1000	0
36423	0.544566279	NA	NA		0
11262	0.533914422	NA	NA		0
2766	0.501985703	938		937	1
4730	0.52623942	737		930	0
2859	0.514423077	NA	NA		1
7717	0.534983253	247		741	0
1014	0.467455621	NA	NA		1
1385	0.473614776	NA	NA		1
1831	0.446247465	NA	NA		1
19763	0.46991404	62		1000	1
5298	0.456249078	NA	NA		0
5298	0.45398773	NA	NA		1
2603	0.502881844	863		943	1
2504	0.504914005	NA	NA		1
1541	0.499026606	NA	NA		1
6109	0.413199426	NA	NA		1
7861	0.557542605	243		938	0
5000	0.517147523	NA	NA		1
2667	0.458646617	NA	NA		1
58751	0.47211803	NA	NA		1
6170	0.435869712	520		1000	1
4999	0.551580699	NA	NA		1
14195	0.466206692	657		1000	0
5591	0.479952597	NA	NA		1
1675	0.440853264	NA	NA		1
3680	0.455778302	NA	NA		0
2349	0.476426548	NA	NA		0
9429	0.487484812	522		820	1
1109	0.555555556	NA	NA		1
6307	0.548237477	NA	NA		1
1216	0.506578947	NA	NA		1
901	0.475118483	NA	NA		1
751	0.450066578	NA	NA		1
1958	0.518146287	NA	NA		1
41687	0.527209572	NA	NA		1
1683	0.480591497	NA	NA		1
10735	0.484080023	120		1000	1
611	0.466448445	NA	NA		1
8282	0.489814454	41		878	0
5237	0.478545455	427		940	1

2609	0.474776049	824	861	1
6826	0.528178496	639	928	1
8815	0.511516854	NA	NA	1
2128	0.508754864	1000	1000	1
5975	0.508639309	NA	NA	1
1123	0.532423208	NA	NA	1
3164	0.468714874	753	936	1
2303	0.522727273	721	1000	1
4409	0.509345794	NA	NA	1
14290	0.526020817	726	871	1
4062	0.487227749	879	1000	1
934	0.43254818	NA	NA	1
984	0.501079914	NA	NA	1
2519	0.491679964	793	942	0
1112	0.510135135	NA	NA	1
6146	0.488715798	257	919	1
5273	0.477694719	509	868	1
1932	0.461584634	NA	NA	1
14154	0.512100927	538	1000	1
10715	0.497789867	NA	NA	1
1065	0.491408935	NA	NA	1
5979	0.494874949	663	946	0
102976	0.420975371	NA	NA	0
2162	0.495374653	NA	NA	1
4316	0.486163705	NA	NA	1
1270	0.609318996	NA	NA	1
5074	0.515470852	571	937	1
3464	0.569261214	NA	NA	1
740	0.561250641	NA	NA	0
935	0.517836594	NA	NA	1
1342	0.506950123	NA	NA	1
6107	0.464174455	NA	NA	1
1426	0.531214094	NA	NA	0
3734	0.534632684	601	1000	1
2114	0.467871486	NA	NA	1
1561	0.439440994	NA	NA	1
2613	0.471871412	1000	1000	1
2726	0.508073601	937	1000	1
1539	0.439297125	NA	NA	1
2839	0.469135802	641	931	1
976	0.52826087	NA	NA	1
991	0.475352113	NA	NA	1
2189	0.498998999	938	941	0
3346	0.486252241	NA	NA	1
3103	0.490732759	NA	NA	0
1913	0.523076923	NA	NA	1
4441	0.526912929	817	863	1
6663	0.513919095	136	931	1
8142	0.509427828	NA	NA	0
1270	0.496918768	NA	NA	0
2428	0.523382145	746	943	1
2810	0.513773839	NA	NA	0
2514	0.453858393	NA	NA	1
3175	0.499290109	468	878	1
4521	0.4986053	NA	NA	1
5077	0.459039266	1000	1000	1
1928	0.442743417	NA	NA	1

22231	0.383978939	402	472	0
1154	0.487868284	NA	NA	1
733	0.46119403	NA	NA	1
5273	0.473864836	530	1000	1
2671	0.479785479	819	934	1
2778	0.390089723	866	947	0
2124	0.456228114	NA	NA	1
3784	0.520516963	441	939	1
2290	0.521188773	NA	NA	1
787	0.397435897	NA	NA	1
6189	0.503902316	394	1000	1
2875	0.486160397	351	1000	1
3076	0.558139535	1000	1000	1
1901	0.485373781	NA	NA	1
2108	0.52368558	NA	NA	1
1290	0.356970185	NA	NA	1
657	0.434056761	NA	NA	1
808	0.461428571	NA	NA	1
1530	0.563106796	NA	NA	1
3273	0.526805659	1000	851	0
669	0.520179372	NA	NA	1
877	0.513939394	NA	NA	1
8465	0.500895522	NA	NA	1
2283	0.470770096	NA	NA	1
4990	0.577209391	536	1000	1
1695	0.531746032	NA	NA	1
7155	0.541165587	607	1000	0
3403	0.533333333	226	1000	1
3011	0.465973202	NA	NA	1
3952	0.540900085	427	1000	0
5175	0.505828476	403	659	0
1648	0.527386541	NA	NA	1
6800	0.488602577	688	1000	1
2723	0.496235455	NA	NA	1
360	0.52	NA	NA	1
574	0.46588694	NA	NA	1
468	0.497560976	NA	NA	1
612	0.484573503	NA	NA	1
4353	0.502797558	939	775	1
4018	0.525623458	797	1000	1
2761	0.485863028	578	1000	1
2171	0.510472479	NA	NA	1
2065	0.496426608	NA	NA	1
1566	0.509308511	NA	NA	1
55593	0.511642676	NA	NA	1
23627	0.49389002	NA	NA	1
2548	0.463941203	828	887	1
1519	0.462804477	NA	NA	1
5416	0.514199954	NA	NA	1
1133	0.531716418	NA	NA	1
3093	0.507450665	NA	NA	1
10448	0.527072234	NA	NA	0
16396	0.548015753	606	1000	1
3292	0.535037594	581	853	0
11683	0.521659708	694	1000	1
1330	0.527819549	NA	NA	1
1554	0.486049927	NA	NA	1

8932	0.45179349	188	1000	1
21757	0.467195606	600	1000	1
3286	0.505380076	783	1000	1
16925	0.432856169	391	1000	1
2991	0.521466905	314	931	1
1371	0.540669856	NA	NA	1
21723	0.53002359	NA	NA	0
14054	0.521696008	NA	NA	1
4036	0.508914729	NA	NA	1
2458	0.50385208	NA	NA	1
9529	0.462814997	NA	NA	1
2034	0.52114061	1000	1000	1
12309	0.489911728	596	1000	0
1444	0.506101938	NA	NA	1
2658	0.491810345	945	770	1
1942	0.539933444	NA	NA	1
1764	0.507050529	NA	NA	1
5374	0.526803441	NA	NA	1
3274	0.536236491	NA	NA	1
2503	0.496441281	801	1000	1
2070	0.532374101	NA	NA	0
5025	0.454429573	NA	NA	1
5088	0.519270989	546	971	1
5857	0.480848556	546	1000	1
10161	0.582014988	593	818	1
3329	0.557538929	NA	NA	1
5065	0.491912215	NA	NA	0
15545	0.454285032	595	871	0
15601	0.574349442	372	1000	0
3023	0.491882248	NA	NA	0
1150	0.464347826	NA	NA	1
1181	0.473327688	NA	NA	1
1880	0.419621749	NA	NA	1
1250	0.452804378	NA	NA	1
1443	0.557971014	NA	NA	1
1258	0.540540541	NA	NA	1
2793	0.521604938	482	1000	1
7568	0.490400569	NA	NA	1
7238	0.442772029	700	1000	1
1552	0.51221374	NA	NA	1
2423	0.567019807	1000	748	1
523	0.47418738	NA	NA	1
594	0.513468013	NA	NA	1
238	0.382352941	NA	NA	1
695	0.437410072	NA	NA	1
1659	0.471971067	NA	NA	1
1115	0.523809524	NA	NA	1
12834	0.447330447	541	1000	1
607	0.438220758	NA	NA	1
1553	0.516419833	NA	NA	1
1592	0.50305499	NA	NA	1
3256	0.458124553	709	948	1
6695	0.509521598	1000	1000	1
9485	0.550958436	254	1000	1
4503	0.444812362	587	1000	1
1744	0.552941176	NA	NA	1
15567	0.37842339	940	68	0

5710	0.476682844	651	854	1
1523	0.536900369	NA	NA	1
2565	0.489391513	NA	NA	1
432	0.365930599	NA	NA	1
774	0.569911504	NA	NA	1
1170	0.562393162	NA	NA	1
1582	0.529709229	NA	NA	1
2977	0.430800089	886	1000	0
5050	0.546882552	471	1000	1
2145	0.447562777	592	932	1
1401	0.524625268	NA	NA	1
4942	0.468669872	NA	NA	0
1878	0.466835123	NA	NA	1
2867	0.506503849	NA	NA	0
2158	0.521393035	NA	NA	1
1518	0.476268412	NA	NA	1
997	0.486187845	NA	NA	1
2044	0.513209393	NA	NA	1
3498	0.539032144	NA	NA	1
1020	0.501960784	NA	NA	1
1712	0.480724299	NA	NA	1
4310	0.499481507	NA	NA	1
1176	0.424319728	NA	NA	1
1486	0.534993271	NA	NA	1
34184	0.513171367	NA	NA	1
10578	0.465617716	NA	NA	1
1748	0.512123004	NA	NA	1
2983	0.541567696	874	778	1
1041	0.595876289	NA	NA	1
5974	0.445624684	NA	NA	1
2756	0.52260111	889	943	1
18224	0.46190777	326	1000	0
76081	0.475030953	NA	NA	0
1810	0.387292818	NA	NA	1
5138	0.397682475	NA	NA	0
2106	0.499415205	NA	NA	1
1526	0.423244552	NA	NA	0
6620	0.416658596	301	877	1
3923	0.522016398	810	1000	1
3576	0.572320117	1000	1000	1
1972	0.419246093	NA	NA	1
3284	0.473545747	NA	NA	0
2164	0.525104603	NA	NA	1
771	0.476123596	NA	NA	1
8883	0.470758123	NA	NA	1
2509	0.525498891	NA	NA	1
3266	0.504567814	812	1000	1
1495	0.476784477	NA	NA	1
1509	0.462277092	NA	NA	1
2859	0.462044756	922	937	0
1008	0.377406932	NA	NA	1
1344	0.583333333	NA	NA	1
3103	0.48606357	NA	NA	1
2896	0.519762846	NA	NA	1
696	0.423580786	NA	NA	1
4085	0.50760474	895	940	0
8221	0.353220697	306	841	0

2403	0.522510823	NA	NA		1
7473	0.376019576	179		944	1
11293	0.380702266	241		887	1
14947	0.485094428	463		1000	0
15740	0.489964445	NA	NA		0
72679	0.590660559	NA	NA		0
4810	0.524376325	NA	NA		1
43086	0.473215106	NA	NA		0
1180	0.570915619	NA	NA		1
2967	0.4875	NA	NA		1
5095	0.598878695	494		581	1
874	0.506864989	NA	NA		1
539	0.452690167	NA	NA		1
3811	0.514516722	NA	NA		1
1518	0.514848066	NA	NA		0
1879	0.510192196	NA	NA		1
15841	0.53318299	NA	NA		1
11204	0.604369809	1000		1000	1
12784	0.508438287	353		1000	0
1089	0.574288725	NA	NA		1
3537	0.473282443	1000		1000	1
690	0.513043478	NA	NA		1
33584	0.457928197	635		1000	0
9315	0.358147229	693		946	1
4788	0.513239188	378		945	1
907	0.534050179	NA	NA		1
8082	0.440543735	557		582	1
1273	0.512905912	NA	NA		1
3850	0.526123551	773		1000	0
2390	0.43916024	NA	NA		1
1128	0.466666667	NA	NA		1
16576	0.447585907	775		441	1
1472	0.556153846	NA	NA		1
3138	0.579637097	NA	NA		1
1179	0.529236868	NA	NA		1
7440	0.542222708	474		885	1
6062	0.571428571	163		928	1
2430	0.562962963	1000		1000	1
2649	0.549098196	NA	NA		1
1803	0.522712732	NA	NA		1
6327	0.484310019	508		936	1
666	0.458053691	NA	NA		1
13179	0.507271926	NA	NA		1
495	0.537373737	NA	NA		1
4693	0.507356431	887		888	1
3058	0.461606355	NA	NA		1
758	0.475982533	NA	NA		1
3146	0.458139935	NA	NA		0
2380	0.514691943	NA	NA		1
689	0.487762238	NA	NA		1
3333	0.442244224	1000		1000	1
851	0.485617597	NA	NA		1
1438	0.494770716	NA	NA		1
324	0.558641975	NA	NA		1
2157	0.557377049	NA	NA		0
2466	0.482920295	NA	NA		1
500	0.485125858	NA	NA		1

3016	0.468065268		201	928	1
7179	0.465386559	NA	NA		1
7154	0.517572802		941	1000	1
1264	0.443801653	NA	NA		1
576	0.527777778	NA	NA		1
4674	0.509750069		1000	1000	1
2402	0.492249116	NA	NA		1
14799	0.520540954	NA	NA		1
6612	0.504173623	NA	NA		1
2823	0.487486398		1000	1000	1
32677	0.517553306		456	476	1
3728	0.57972474		1000	1000	1
878	0.503416856	NA	NA		1
17175	0.556498134		1000	1000	1
1999	0.505882353	NA	NA		1
1567	0.559668156	NA	NA		1
6630	0.461348176		797	915	0
2064	0.538619979	NA	NA		1
48541	0.508287293		812	1000	1
13055	0.477728285	NA	NA		1
494	0.375576037	NA	NA		1
1737	0.449487555	NA	NA		1
1507	0.598942598	NA	NA		1
663	0.445121951	NA	NA		1
5287	0.516917661		1000	1000	1
7335	0.417298468		791	690	1
959	0.382690302	NA	NA		1
5262	0.499261993		303	1000	1
1657	0.520274485	NA	NA		1
1617	0.567099567	NA	NA		1
1612	0.519230769	NA	NA		1
985	0.52006689	NA	NA		1
1592	0.502310231	NA	NA		1
1796	0.48905566	NA	NA		1
4756	0.49900111	NA	NA		1
5863	0.50035137	NA	NA		1
3666	0.531777957	NA	NA		0
1797	0.574846967	NA	NA		1
871	0.469520104	NA	NA		1
1895	0.477017937	NA	NA		1
2438	0.568498769		1000	1000	1
1126	0.58259325	NA	NA		1
3098	0.539007092	NA	NA		1
643	0.458490566	NA	NA		1
997	0.562688064	NA	NA		1
1247	0.595829992	NA	NA		1
2368	0.547719595		1000	1000	1
4274	0.509803922		1000	1000	1
1617	0.594310451	NA	NA		1
2008	0.515480717	NA	NA		1
553	0.518987342	NA	NA		1
1131	0.505747126	NA	NA		1
3795	0.506286982	NA	NA		0
2753	0.466868298	NA	NA		1
2367	0.518181818	NA	NA		1
3367	0.400980993	NA	NA		1
1673	0.499103407	NA	NA		1

414	0.548309179	NA	NA	1
2173	0.522977346	NA	NA	1
1459	0.561198578	NA	NA	1
14215	0.518095238	NA	NA	0
8052	0.560824445	NA	NA	1
3965	0.535612536	NA	NA	1
1619	0.575068244	NA	NA	1
1654	0.505102041	NA	NA	1
1284	0.515576324	NA	NA	1
945	0.461375661	NA	NA	1
1126	0.506603774	NA	NA	1
4580	0.449775112	NA	NA	1
757	0.479315264	NA	NA	1
17574	0.521327014	NA	NA	1
7511	0.521976909	354	937	0
1848	0.515447154	NA	NA	1
1812	0.479028698	NA	NA	1
3320	0.515755329	560	1000	1
9240	0.540627769	277	719	0
1005	0.59800995	NA	NA	1
1164	0.504977376	NA	NA	1
886	0.431151242	NA	NA	1
704	0.525568182	NA	NA	1
674	0.479553903	NA	NA	1
1668	0.502997602	NA	NA	1
1312	0.485395189	NA	NA	1
6342	0.536746988	NA	NA	1
41600	0.502832861	1000	1000	1
2100	0.495238095	1000	1000	1
3880	0.501132931	382	1000	0
1896	0.484177215	NA	NA	1
45027	0.464405134	478	1000	0
599	0.571962617	NA	NA	1
2071	0.550877193	NA	NA	1
8476	0.514918671	332	1000	0
2433	0.476517755	941	939	0
11129	0.417016406	285	1000	1
680	0.446227929	NA	NA	1
1160	0.474499089	NA	NA	1
1242	0.555461473	NA	NA	1
1304	0.532208589	NA	NA	1
1226	0.544639043	NA	NA	1
6035	0.478389534	875	1000	0
1565	0.560879811	NA	NA	1
883	0.554926387	NA	NA	1
2350	0.565880721	NA	NA	1
875	0.533714286	NA	NA	1
789	0.558685446	NA	NA	1
1252	0.502206531	NA	NA	1
39477	0.447546243	340	1000	0
6025	0.556113482	711	808	0
1183	0.54954955	NA	NA	1
776	0.49132948	NA	NA	1
1282	0.434782609	NA	NA	1
2547	0.509624033	NA	NA	0
1361	0.478611784	NA	NA	1
496	0.554435484	NA	NA	1

2899	0.524278677	NA	NA	1
1962	0.49559956	NA	NA	1
1211	0.531855956	NA	NA	1
535	0.467640919	NA	NA	1
4115	0.51730226	972	873	1
1076	0.487084871	NA	NA	1
1458	0.543624161	NA	NA	1
1756	0.488306829	NA	NA	1
789	0.492727273	NA	NA	1
1585	0.452996845	NA	NA	1
1203	0.53358562	NA	NA	1
1949	0.579599619	NA	NA	1
1852	0.522093023	NA	NA	1
1271	0.537717602	NA	NA	1
1274	0.453757225	NA	NA	1
793	0.43153527	NA	NA	1
4869	0.500509165	723	1000	0
521	0.483685221	NA	NA	1
1177	0.539426523	NA	NA	1
3797	0.542182891	913	958	1
3037	0.465434634	NA	NA	1
1695	0.470303422	NA	NA	0
3956	0.517081851	770	874	1
6848	0.493946731	566	937	0
1319	0.532258065	NA	NA	1
2310	0.450834403	NA	NA	1
1738	0.460299194	NA	NA	1
1119	0.588918677	NA	NA	1
825	0.530909091	NA	NA	1
4559	0.421468554	NA	NA	1
477	0.545073375	NA	NA	1
504	0.537698413	NA	NA	1
5488	0.540790312	143	882	0
5718	0.513628778	1000	936	1
2286	0.466225791	951	962	1
1583	0.502949853	NA	NA	1
817	0.506731946	NA	NA	1
1314	0.473254505	NA	NA	0
3248	0.497746297	NA	NA	1
3800	0.501783026	NA	NA	0
1353	0.517195176	NA	NA	0
28623	0.563820215	NA	NA	1
2411	0.539568345	NA	NA	1
3744	0.497678737	NA	NA	1
1856	0.511853448	NA	NA	1
2342	0.492358804	1000	944	1
1392	0.551005747	NA	NA	1
1882	0.521876982	NA	NA	1
1306	0.548309179	NA	NA	1
5812	0.472222222	NA	NA	0
1999	0.491831972	NA	NA	1
5997	0.532398784	NA	NA	0
1664	0.486937591	NA	NA	1
2005	0.490623109	NA	NA	1
1776	0.503699137	NA	NA	1
1483	0.541907514	NA	NA	0
959	0.554744526	NA	NA	1

877	0.517673888	NA	NA		1
11561	0.455345241	NA	NA		1
5610	0.505441176	NA	NA		0
16963	0.515335297	332		1000	1
47706	0.503450259	NA	NA		1
3066	0.562385991	851		1000	1
5298	0.530580076	350		935	1
10623	0.534053978	NA	NA		1
1903	0.545980032	NA	NA		1
1442	0.515503876	NA	NA		1
3092	0.505502307	NA	NA		0
3836	0.574137196	161		937	0
5908	0.51591063	NA	NA		1
1408	0.510416667	NA	NA		1
3942	0.547812418	1000		1000	1
1316	0.411974978	NA	NA		1
3519	0.56101104	1000		1000	1
1525	0.504461222	NA	NA		1
1259	0.515330189	NA	NA		1
1693	0.437093916	NA	NA		1
1720	0.499433321	NA	NA		1
4428	0.538945331	1000		952	1
5603	0.439493671	NA	NA		1
3587	0.494252874	NA	NA		1
6404	0.518580516	706		926	1
3828	0.454855196	NA	NA		1
1946	0.468656716	NA	NA		1
7450	0.532798087	307		877	1
1205	0.497237569	NA	NA		1
1642	0.509931507	NA	NA		0
1331	0.501577287	NA	NA		1
1889	0.536791953	NA	NA		1
4868	0.579908676	600		890	1
3776	0.514866058	900		932	1
2701	0.493999442	NA	NA		0
336	0.510638298	NA	NA		1
1943	0.533667622	NA	NA		1
3095	0.569508449	872		844	1
1555	0.549160671	NA	NA		1
2498	0.512479201	NA	NA		1
1540	0.435064935	NA	NA		1
3615	0.490153846	942		936	1
4527	0.504972376	897		873	1
2501	0.524590164	1000		1000	1
1539	0.438948995	NA	NA		1
918	0.583875163	NA	NA		1
1410	0.514053254	NA	NA		1
8784	0.496963153	380		771	0
8655	0.556966927	495		1000	1
1783	0.485376478	NA	NA		1
8411	0.508661717	24		967	1
2220	0.555333998	934		852	1
785	0.477744807	NA	NA		1
7173	0.546740367	NA	NA		1
697	0.50071736	NA	NA		1
5358	0.495290859	NA	NA		1
5342	0.549172532	915		1000	1

1519	0.563520227	NA	NA	1
4955	0.516999261	NA	NA	1
1677	0.567680382	NA	NA	1
10404	0.586768521	735	861	1
3110	0.51409396	NA	NA	1
651	0.52688172	NA	NA	1
2871	0.500505051	NA	NA	1
2294	0.48490311	NA	NA	1
3322	0.541653952	NA	NA	1
3597	0.529133858	NA	NA	1
654	0.547400612	NA	NA	1
1372	0.495596477	NA	NA	1
3858	0.515438108	1000	737	1
12251	0.515369752	198	1000	0
28485	0.513955892	NA	NA	0
27099	0.514329977	NA	NA	1
1127	0.461401952	NA	NA	1
2617	0.488390713	NA	NA	1
2295	0.377777778	1000	1000	1
3462	0.484084084	404	933	1
2009	0.492284719	NA	NA	1
3254	0.520198171	NA	NA	1
1498	0.474632844	NA	NA	1
5422	0.49454016	NA	NA	0
5259	0.466834171	NA	NA	1
1217	0.492825112	NA	NA	0
739	0.460081191	NA	NA	1
4045	0.470606532	NA	NA	1
776	0.581944444	NA	NA	1
2345	0.55396679	880	1000	1
621	0.518518519	NA	NA	1
1340	0.531555556	NA	NA	1
8507	0.467667918	186	938	1
1215	0.465977606	NA	NA	1
3848	0.503748126	746	1000	1
6808	0.454289733	NA	NA	1
1338	0.430913349	NA	NA	1
796	0.595477387	NA	NA	1
3578	0.422624434	NA	NA	1
5546	0.480943739	759	921	1
1531	0.489347079	NA	NA	1
1962	0.435751295	NA	NA	0
838	0.472553699	NA	NA	1
1387	0.497747748	NA	NA	1
7029	0.420782553	NA	NA	1
12459	0.485473057	NA	NA	1
2425	0.493472585	NA	NA	1
1714	0.510580205	NA	NA	1
2460	0.499367356	911	1000	1
2889	0.48044306	1000	1000	1
1955	0.515247108	NA	NA	1
13147	0.475812547	137	1000	1
595	0.522776573	NA	NA	1
3378	0.501886792	412	945	1
6884	0.498349835	NA	NA	1
1036	0.521226415	NA	NA	1
6310	0.517739605	919	1000	1

32088	0.458523245		101	1000	0
17271	0.516134316		721	1000	0
868	0.463078849	NA	NA		1
2649	0.541713854		1000	1000	1
1259	0.434086629	NA	NA		1
753	0.502446982	NA	NA		1
696	0.567528736	NA	NA		1
614	0.429967427	NA	NA		1
29462	0.538725796		645	933	1
1543	0.530136099	NA	NA		1
6232	0.477831715		948	1000	1
1773	0.450793651	NA	NA		1
446	0.522421525	NA	NA		1
560	0.534653465	NA	NA		1
911	0.585628743	NA	NA		1
469	0.420042644	NA	NA		1
414	0.61352657	NA	NA		1
1416	0.433931485	NA	NA		1
5120	0.565175795	NA	NA		0
3585	0.554250386		715	1000	1
1106	0.519430052	NA	NA		1
940	0.482978723	NA	NA		1
49634	0.350230415		594	471	0
3501	0.507169576		1000	762	1
9821	0.538043478		912	939	1
1294	0.500772798	NA	NA		1
2470	0.465473146		940	936	1
5210	0.511172089		85	834	1
7931	0.478166304		281	1000	0
1938	0.463693228	NA	NA		1
947	0.505807814	NA	NA		1
1184	0.447635135	NA	NA		1
1279	0.500891266	NA	NA		1
1548	0.545759866	NA	NA		0
4279	0.536127168	NA	NA		1
348	0.554597701	NA	NA		1
1034	0.404255319	NA	NA		1
282	0.535460993	NA	NA		1
3386	0.513742789		1000	1000	1
1775	0.425208808	NA	NA		1
396	0.527777778	NA	NA		1
516	0.513565891	NA	NA		1
1845	0.498102981	NA	NA		1
9887	0.515055468	NA	NA		1
807	0.546551724	NA	NA		1
1596	0.543859649	NA	NA		1
83681	0.483292297	NA	NA		1
2282	0.446300716	NA	NA		1
2579	0.447643979	NA	NA		1
660	0.48030303	NA	NA		1
3156	0.562284928	NA	NA		1
8491	0.535025381	NA	NA		1
2394	0.535978836		787	934	0
1484	0.450777202	NA	NA		1
827	0.489721886	NA	NA		1
14062	0.487228455	NA	NA		1
1510	0.47092084	NA	NA		1

1928	0.512168142	NA	NA		1
4528	0.479464939	NA	NA		1
4490	0.492974239	844		1000	1
653	0.498098859	NA	NA		1
4781	0.513986516	827		546	1
1318	0.513333333	NA	NA		1
4734	0.481852024	NA	NA		1
1083	0.516634051	NA	NA		1
11726	0.531213365	312		1000	0
3656	0.523994812	574		854	1
4090	0.540077391	946		1000	1
723	0.446841294	NA	NA		1
1266	0.526446281	NA	NA		1
1772	0.505851756	NA	NA		1
450	0.393333333	NA	NA		1
1867	0.521191294	NA	NA		1
5821	0.474029126	217		1000	0
1526	0.530799476	NA	NA		1
573	0.556719023	NA	NA		1
40263	0.479000724	453		938	1
5103	0.470513229	850		936	0
1460	0.469767442	NA	NA		1
987	0.614994934	NA	NA		1
1571	0.598742138	NA	NA		1
1870	0.529477197	NA	NA		1
1270	0.542948038	NA	NA		1
2773	0.453521127	NA	NA		1
1995	0.483516484	NA	NA		1
689	0.520231214	NA	NA		1
1543	0.473794549	NA	NA		1
1106	0.491862568	NA	NA		1
4419	0.51031108	NA	NA		1
3781	0.53005284	719		883	1
3189	0.569335938	NA	NA		0
1043	0.449392713	NA	NA		1
6831	0.503691983	NA	NA		1
471	0.430997877	NA	NA		1
2982	0.402749832	1000		1000	1
852	0.50921273	NA	NA		1
7983	0.548970748	NA	NA		1
1471	0.518891688	NA	NA		1
1263	0.447674419	NA	NA		1
1131	0.472148541	NA	NA		1
2712	0.495265541	NA	NA		1
563	0.48216106	NA	NA		1
1366	0.508798776	NA	NA		1
5092	0.459048691	697		837	1
1050	0.507518797	NA	NA		1
1082	0.478448276	NA	NA		0
1026	0.426900585	NA	NA		1
3781	0.534456355	NA	NA		1
2681	0.498712446	NA	NA		1
19105	0.498920553	NA	NA		0
2427	0.533297816	NA	NA		1
613	0.508972268	NA	NA		1
1745	0.42832938	NA	NA		0
424	0.555555556	NA	NA		1

3496	0.501371742	693	733	1
560	0.580729167	NA	NA	1
680	0.5	NA	NA	1
896	0.518973214	NA	NA	1
680	0.582930757	NA	NA	1
864	0.59375	NA	NA	0
552	0.574074074	NA	NA	1
930	0.607526882	NA	NA	1
327	0.526515152	NA	NA	1
751	0.513196481	NA	NA	1
9282	0.456703182	440	1000	1
5003	0.437079731	145	766	1
651	0.501872659	NA	NA	1
1482	0.556005398	NA	NA	1
1659	0.47731889	NA	NA	1
2231	0.512793177	NA	NA	1
1735	0.508166969	NA	NA	1
2044	0.469186047	NA	NA	1
2696	0.502179945	NA	NA	1
624	0.516025641	NA	NA	1
6989	0.410554265	NA	NA	1
1472	0.403880071	NA	NA	1
3655	0.478554408	NA	NA	1
618	0.487055016	NA	NA	1
602	0.524691358	NA	NA	1
2560	0.465625	1000	1000	1
1734	0.443455325	NA	NA	0
3417	0.424883623	534	986	1
1906	0.538824764	NA	NA	1
1688	0.560541311	NA	NA	1
1360	0.43240974	NA	NA	1
3497	0.503646973	808	855	1
5993	0.485338726	1000	1000	1
3733	0.47837658	821	892	1
354	0.553672316	NA	NA	1
2868	0.49532294	NA	NA	1
8453	0.48436792	363	895	1
782	0.480818414	NA	NA	1
27954	0.51490995	NA	NA	1
1109	0.533333333	NA	NA	1
1803	0.438713256	NA	NA	1
2284	0.491939424	NA	NA	1
4158	0.436220472	NA	NA	1
865	0.46961326	NA	NA	1
380	0.451410658	NA	NA	1
3574	0.553345389	391	1000	0
1603	0.496368039	NA	NA	1
4601	0.479453411	NA	NA	1
441	0.58276644	NA	NA	1
685	0.518248175	NA	NA	1
1308	0.455657492	NA	NA	1
1548	0.465099192	NA	NA	1
714	0.567226891	NA	NA	1
408	0.590686275	NA	NA	1
3803	0.478484265	NA	NA	1
299	0.589041096	NA	NA	1
436	0.600660066	NA	NA	1

3334	0.514715948	NA	NA		1
1217	0.517331023	NA	NA		1
3469	0.47465921		392	1000	1
1627	0.507772021	NA	NA		1
3566	0.519436792		942	1000	1
13938	0.443573668	NA	NA		1
930	0.48172043	NA	NA		1
19073	0.521792511	NA	NA		1
4057	0.471930907		518	927	1
5522	0.501375246	NA	NA		0
1386	0.562049062	NA	NA		1
2766	0.578166402	NA	NA		1
1752	0.485312118	NA	NA		1
1526	0.572246066	NA	NA		1
779	0.472789116	NA	NA		1
1069	0.471596999	NA	NA		1
1629	0.528192371	NA	NA		1
2520	0.518117902	NA	NA		1
621	0.425120773	NA	NA		1
477	0.48427673	NA	NA		1
9476	0.472373806		196	888	1
2566	0.431800468		1000	1000	1
17891	0.483673469	NA	NA		1
686	0.439178515	NA	NA		1
2122	0.531102733	NA	NA		1
2241	0.526413922	NA	NA		1
444	0.515625	NA	NA		1
494	0.538461538	NA	NA		1
5663	0.45040549	NA	NA		1
17784	0.389824305	NA	NA		1
929	0.560233918	NA	NA		1
5279	0.461915367		656	1000	1
15295	0.489710736		197	704	0
1363	0.505800464	NA	NA		1
1277	0.53014038	NA	NA		1
1997	0.444148936	NA	NA		1
2818	0.500547645		921	1000	1
2835	0.470503076		928	1000	1
4942	0.46928006	NA	NA		1
2177	0.564794007	NA	NA		1
1511	0.46	NA	NA		1
953	0.490453461	NA	NA		1
644	0.563218391	NA	NA		1
2125	0.532416503		1000	916	1
500	0.498	NA	NA		1
3095	0.509765625	NA	NA		1
1754	0.473001508	NA	NA		1
19094	0.502207506	NA	NA		1
679	0.535555556	NA	NA		1
740	0.542986425	NA	NA		1
2310	0.426926484		948	1000	1
1306	0.507593925	NA	NA		1
2787	0.527472527	NA	NA		1
386	0.50617284	NA	NA		1
2817	0.466179159		885	823	1
237	0.481012658	NA	NA		1
556	0.456834532	NA	NA		1

675	0.377723971	NA	NA		1
503	0.4055666	NA	NA		1
1296	0.441485069	NA	NA		1
1993	0.468138485	NA	NA		1
1215	0.526748971	NA	NA		1
10887	0.476190476		59	1000	1
8439	0.488138475		415	1000	0
716	0.498333333	NA	NA		1
657	0.436834094	NA	NA		1
5866	0.495392954	NA	NA		1
1185	0.544007491	NA	NA		1
904	0.442477876	NA	NA		1
484	0.483471074	NA	NA		1
1509	0.472113119	NA	NA		1
4573	0.508414526	NA	NA		1
5562	0.50750268	NA	NA		0
588	0.600340136	NA	NA		1
345	0.576811594	NA	NA		1
1935	0.511363636	NA	NA		1
468	0.493589744	NA	NA		1
2134	0.497553018	NA	NA		1
2249	0.523012552	NA	NA		1
839	0.497020262	NA	NA		1
1097	0.541538462	NA	NA		1
642	0.472380952	NA	NA		1
740	0.481343284	NA	NA		1
693	0.487972509	NA	NA		1
23167	0.528477736	NA	NA		1
718	0.505823627	NA	NA		1
342	0.375	NA	NA		1
2261	0.536930562		1000	1000	1
2006	0.535829567	NA	NA		1
2443	0.536133695		884	887	1
2740	0.485798237	NA	NA		1
4640	0.552845528		314	939	0
447	0.428571429	NA	NA		1
1428	0.533435819	NA	NA		1
4529	0.477869986	NA	NA		1
1908	0.556818182	NA	NA		1
414	0.407932011	NA	NA		1
776	0.516949153	NA	NA		1
1924	0.488199417	NA	NA		0
414	0.407303371	NA	NA		1
2656	0.482680723		1000	1000	1
9943	0.519316909	NA	NA		1
707	0.589484328	NA	NA		0
1840	0.566060606	NA	NA		1
636	0.508591065	NA	NA		1
1452	0.452517986	NA	NA		1
6017	0.471927162	NA	NA		1
17022	0.48857645	NA	NA		1
7623	0.49466951		209	1000	1
1382	0.373371925	NA	NA		1
16056	0.499155049		343	829	1
1970	0.508797654	NA	NA		1
1613	0.575226978	NA	NA		1
4672	0.506835182	NA	NA		1

384	0.484375	NA	NA		1
354	0.553672316	NA	NA		1
300	0.556666667	NA	NA		1
354	0.449152542	NA	NA		1
6745	0.572458601	519		1000	1
451	0.542056075	NA	NA		1
2605	0.563088512	739		1000	1
1653	0.506957048	NA	NA		1
1437	0.513580247	NA	NA		1
1038	0.558855886	NA	NA		1
454	0.451948052	NA	NA		1
4041	0.513368984	NA	NA		0
354	0.471751412	NA	NA		1
34094	0.534106412	NA	NA		1
8419	0.497894863	1000		825	0
2166	0.464028777	NA	NA		1
2174	0.591452991	NA	NA		1
1989	0.539253539	NA	NA		1
1555	0.523076923	NA	NA		1
2288	0.470043573	NA	NA		1
2059	0.531475748	NA	NA		1
15057	0.445361826	247		891	1
2054	0.462814339	NA	NA		1
1645	0.4623813	NA	NA		1
327	0.525993884	NA	NA		1
2471	0.554694229	1000		1000	1
5608	0.555555556	768		1000	1
315	0.647619048	NA	NA		1
1066	0.498064516	NA	NA		1
672	0.508928571	NA	NA		1
430	0.495348837	NA	NA		1
2840	0.509450172	839		854	1
1421	0.475609756	NA	NA		1
1974	0.504009164	NA	NA		1
1692	0.509478673	NA	NA		1
2679	0.488331893	NA	NA		1
1044	0.541968912	NA	NA		1
1660	0.474616293	NA	NA		0
966	0.49689441	NA	NA		1
3724	0.523934618	925		1000	1
2035	0.535336672	NA	NA		1
2093	0.517839445	926		901	1
2242	0.458692347	NA	NA		1
1694	0.522501907	NA	NA		1
6813	0.560583702	890		872	1
1355	0.478577203	NA	NA		1
6056	0.516689529	282		1000	1
1444	0.550042052	NA	NA		1
6693	0.513509501	NA	NA		0
4593	0.493794905	NA	NA		1
2556	0.498449612	NA	NA		1
907	0.52370452	NA	NA		1
2234	0.592322643	938		886	1
3335	0.495412844	1000		1000	1
760	0.45177665	NA	NA		1
827	0.506650544	NA	NA		1
357	0.540616246	NA	NA		1

3963	0.484600262	883	876	1
1205	0.542303772	NA	NA	1
9176	0.480793061	NA	NA	0
3609	0.495605775	908	1000	1
462	0.527363184	NA	NA	1
7356	0.467334495	NA	NA	1
618	0.508090615	NA	NA	1
1347	0.532293987	NA	NA	1
1792	0.457587998	NA	NA	1
2815	0.42676311	950	1000	1
639	0.558219178	NA	NA	1
1731	0.501199041	NA	NA	1
5551	0.565663844	1000	1000	1
659	0.50247117	NA	NA	1
5863	0.510669828	407	1000	1
823	0.586251621	NA	NA	1
294	0.530612245	NA	NA	1
1384	0.488439306	NA	NA	1
14063	0.522525919	NA	NA	1
3890	0.519510111	924	937	1
52358	0.460968616	NA	NA	0
1813	0.430371771	NA	NA	1
1899	0.448379352	NA	NA	1
815	0.568627451	NA	NA	1
1115	0.49058296	NA	NA	1
55006	0.529654461	NA	NA	1
306	0.532679739	NA	NA	1
7151	0.495175569	1000	935	0
18691	0.475402603	1000	1000	0
675	0.545185185	NA	NA	1
10121	0.509811113	NA	NA	1
1299	0.541185527	NA	NA	1
1089	0.492264416	NA	NA	1
615	0.495934959	NA	NA	1
488	0.424311927	NA	NA	1
873	0.546969697	NA	NA	1
420	0.473809524	NA	NA	1
4116	0.490767736	1000	1000	1
2379	0.550069865	942	886	1
1891	0.580340265	NA	NA	1
2163	0.518699187	NA	NA	1
10121	0.499773294	NA	NA	0
829	0.477003942	NA	NA	1
1106	0.384030418	NA	NA	1
1083	0.428711898	NA	NA	1
999	0.469600463	NA	NA	1
1387	0.410699588	NA	NA	1
1421	0.427721088	NA	NA	1
66690	0.497480202	1000	453	1
465	0.574193548	NA	NA	1
342	0.590643275	NA	NA	1
5727	0.52125535	1000	1000	1
2007	0.531141006	1000	1000	1
2481	0.521155831	NA	NA	1
2937	0.513490364	795	843	1
678	0.448377581	NA	NA	1
619	0.526978417	NA	NA	1

2616	0.497879559	807	1000	1
872	0.582568807 NA	NA		1
1872	0.533858268 NA	NA		0
1164	0.434554974 NA	NA		1
7105	0.557359796	888	1000	1
2309	0.506624029	880	1000	1
2690	0.513103448	801	880	1
1627	0.47815534 NA	NA		1
1811	0.619129439 NA	NA		1
7460	0.57678901	1000	1000	1
4883	0.499724821	502	1000	1
2395	0.536895674 NA	NA		1
747	0.497816594 NA	NA		1
1032	0.544984488 NA	NA		1
968	0.48553719 NA	NA		1
890	0.578590786 NA	NA		1
7166	0.436714884 NA	NA		0
1104	0.513796384 NA	NA		1
559	0.570661896 NA	NA		1
547	0.469835466 NA	NA		1
775	0.48516129 NA	NA		1
13843	0.482253771	313	1000	1
5454	0.45481394 NA	NA		0
2044	0.482387476	836	772	1
2617	0.513757116	1000	1000	1
723	0.561728395 NA	NA		1
5683	0.461787072 NA	NA		1
1886	0.514515133 NA	NA		1
1523	0.407482993 NA	NA		1
1347	0.484780995 NA	NA		1
633	0.526066351 NA	NA		1
2579	0.568143101	1000	879	1
46119	0.463006361	869	1000	1
4164	0.534961832	352	1000	1
2167	0.519862678	939	933	1
946	0.501210654 NA	NA		1
1392	0.48985115 NA	NA		1
1379	0.492537313 NA	NA		1
1710	0.517549897 NA	NA		1
339	0.530973451 NA	NA		1
2868	0.465097067	916	947	1
16352	0.456541329 NA	NA		0
905	0.467403315 NA	NA		1
1632	0.475490196 NA	NA		1
606	0.419141914 NA	NA		1
832	0.548262548 NA	NA		1
789	0.651324965 NA	NA		1
1121	0.613636364 NA	NA		1
27430	0.487247142	384	881	1
20416	0.551210428	457	878	1
485	0.540206186 NA	NA		1
560	0.567857143 NA	NA		1
856	0.519859813 NA	NA		1
10079	0.493169877 NA	NA		1
17270	0.493480813 NA	NA		1
7900	0.499260256	192	1000	0
5079	0.494396015 NA	NA		1

423	0.46779661	NA	NA		1
5078	0.433552886	NA	NA		1
594	0.406654344	NA	NA		1
1744	0.47706422	NA	NA		1
715	0.537037037	NA	NA		1
501	0.467065868	NA	NA		1
661	0.473333333	NA	NA		1
676	0.48816568	NA	NA		1
177	0.536723164	NA	NA		1
1469	0.528726708	NA	NA		1
12351	0.52852349		91	1000	1
604	0.435430464	NA	NA		1
4550	0.539749941	NA	NA		1
1629	0.576797386	NA	NA		1
7128	0.504680974	NA	NA		0
45360	0.51744186	NA	NA		1
2228	0.518850987		1000	1000	1
2103	0.490252021		1000	1000	1
2185	0.509382151		1000	1000	1
708	0.458762887	NA	NA		1
2882	0.495167286	NA	NA		1
782	0.524296675	NA	NA		1
618	0.582524272	NA	NA		1
1966	0.513029316	NA	NA		1
889	0.419512195	NA	NA		1
5707	0.507519593		942	796	1
3390	0.436626747		716	435	1
1692	0.525413712	NA	NA		1
2227	0.443548387	NA	NA		1
2257	0.439940577	NA	NA		1
3624	0.515182186	NA	NA		1
1748	0.504065041	NA	NA		1
3615	0.5357399		54	1000	0
2428	0.503569111	NA	NA		1
693	0.54978355	NA	NA		1
3304	0.545134302		511	940	1
3809	0.559405941	NA	NA		1
10827	0.543100511	NA	NA		0
1297	0.532310178	NA	NA		1
23473	0.499310345		195	840	0
815	0.613756614	NA	NA		1
2080	0.485854859	NA	NA		1
1152	0.502895753	NA	NA		1
336	0.556547619	NA	NA		1
3131	0.486618922		1000	1000	1
2529	0.468138276		949	923	1
1392	0.476688103	NA	NA		1
1047	0.505253104	NA	NA		1
10492	0.491939547	NA	NA		1
1724	0.46332737	NA	NA		1
858	0.47755102	NA	NA		1
743	0.475100942	NA	NA		1
1664	0.497596154	NA	NA		1
612	0.473856209	NA	NA		1
2305	0.498817268	NA	NA		1
5948	0.521810089		1000	1000	0
4944	0.491150442	NA	NA		1

1218	0.453477868	NA	NA		1
1154	0.497400347	NA	NA		1
2107	0.513051732	411		940	1
1317	0.412300683	NA	NA		1
576	0.560763889	NA	NA		1
1397	0.511354738	NA	NA		1
2520	0.564579808	NA	NA		1
2382	0.440677966	684		1000	1
11298	0.503759398	NA	NA		1
961	0.506651885	NA	NA		1
555	0.598198198	NA	NA		1
558	0.641577061	NA	NA		1
1494	0.571428571	NA	NA		1
819	0.567765568	NA	NA		1
1150	0.516431925	NA	NA		1
2481	0.579874214	NA	NA		1
1652	0.513406157	NA	NA		0
944	0.612865497	NA	NA		1
2871	0.55134357	1000		415	1
1572	0.501908397	NA	NA		1
3564	0.542392567	NA	NA		1
2619	0.499606299	NA	NA		1
1763	0.533411489	NA	NA		1
1138	0.475149105	NA	NA		1
1136	0.557943925	NA	NA		1
494	0.517162471	NA	NA		1
572	0.485322896	NA	NA		1
1910	0.439972241	NA	NA		1
996	0.493975904	NA	NA		1
11849	0.447222813	1000		1000	1
986	0.517241379	NA	NA		1
5295	0.521575199	837		1000	1
1006	0.577534791	NA	NA		1
1028	0.487447699	NA	NA		1
459	0.605664488	NA	NA		1
525	0.582857143	NA	NA		1
9339	0.446983363	459		1000	0
2176	0.496244367	NA	NA		1
4105	0.529941345	1000		1000	0
1268	0.422712934	NA	NA		1
33951	0.526799168	862		1000	1
1582	0.509925558	NA	NA		0
922	0.508496732	NA	NA		1
2182	0.484796044	445		1000	1
7976	0.537794061	923		1000	1
1604	0.517191977	NA	NA		1
6201	0.464848592	616		1000	1
1143	0.477502296	NA	NA		1
1745	0.516877637	NA	NA		1
1573	0.483405483	NA	NA		1
2501	0.477937964	NA	NA		1
1043	0.536294691	NA	NA		1
951	0.441475827	NA	NA		1
1341	0.534653465	NA	NA		1
6777	0.513685551	307		877	1
2627	0.49532294	NA	NA		0
813	0.531365314	NA	NA		1

6564	0.427103159	935	929	1
8447	0.362643235	1000	969	0
3773	0.5146282	NA	NA	0
3608	0.555555556	948	943	1
14676	0.472841444	NA	NA	0
2431	0.486585366	NA	NA	0
5808	0.538188762	132	941	1
11449	0.534401261	NA	NA	0
5094	0.561068702	NA	NA	1
7984	0.522420822	NA	NA	0
13717	0.544293219	NA	NA	0
1600	0.609061489	NA	NA	1
1122	0.533962264	NA	NA	1
1546	0.456475584	NA	NA	1
647	0.515517241	NA	NA	1
3853	0.555729167	NA	NA	1
1062	0.456685499	NA	NA	1
764	0.519536903	NA	NA	1
815	0.511656442	NA	NA	1
1839	0.61010101	NA	NA	1
1400	0.452147702	NA	NA	1
623	0.529163145	NA	NA	1
290	0.383928571	NA	NA	1
268	0.396825397	NA	NA	1
621	0.458937198	NA	NA	1
2242	0.475073314	NA	NA	1
11300	0.518198198	378	1000	1
6012	0.440247253	NA	NA	1
2714	0.483043838	NA	NA	1
2059	0.504096122	NA	NA	0
6464	0.514778814	629	940	0
6674	0.512787393	NA	NA	0
1130	0.468718967	NA	NA	1
4125	0.494605809	NA	NA	1
694	0.489859594	NA	NA	1
7012	0.5075953	245	782	0
4467	0.43731525	840	812	1
1278	0.531377343	NA	NA	1
1704	0.476525822	NA	NA	1
162	0.617283951	NA	NA	1
2473	0.464091134	388	878	1
70923	0.496309418	NA	NA	1
5357	0.516349094	929	940	1
1513	0.490870787	NA	NA	1
57843	0.475635593	626	1000	1
1298	0.578082192	NA	NA	1
13743	0.527965492	371	1000	1
4535	0.458083832	827	1000	1
1729	0.524314214	NA	NA	1
44531	0.469416785	NA	NA	1
1612	0.455493741	NA	NA	1
1029	0.484936832	NA	NA	1
7530	0.499437289	1000	1000	0
557	0.468581688	NA	NA	1
1502	0.513937282	NA	NA	1
3114	0.530507386	313	1000	1
1379	0.44960116	NA	NA	1

720	0.577777778	NA	NA		1
876	0.485159817	NA	NA		1
486	0.37470726	NA	NA		1
10003	0.485501066	NA	NA		0
6316	0.558676028	NA	NA		1
935	0.525083612	NA	NA		0
815	0.43832021	NA	NA		1
879	0.47440273	NA	NA		1
3330	0.53968254	NA	NA		1
896	0.573109244	NA	NA		1
484	0.542253521	NA	NA		1
1841	0.531776209	NA	NA		1
5439	0.474653424		886	822	1
1491	0.547921968	NA	NA		1
1626	0.453909727	NA	NA		1
6469	0.51979346		288	1000	1
2059	0.50094518	NA	NA		1
2328	0.518962076		947	729	1
1615	0.529774127	NA	NA		1
14438	0.534201954	NA	NA		1
1441	0.556851312	NA	NA		1
1106	0.367016206	NA	NA		1
485	0.455670103	NA	NA		1
1642	0.547619048	NA	NA		1
354	0.516949153	NA	NA		1
722	0.503793627	NA	NA		1
489	0.562372188	NA	NA		1
1065	0.470886076	NA	NA		1
1113	0.583108715	NA	NA		1
1148	0.49200492	NA	NA		1
3379	0.578990902	NA	NA		1
1032	0.420542636	NA	NA		1
366	0.601092896	NA	NA		1
745	0.422818792	NA	NA		1
2463	0.535194175	NA	NA		1
1056	0.463258786	NA	NA		1
30838	0.513392857	NA	NA		1
1976	0.500538793	NA	NA		1
3716	0.550347747	NA	NA		1
1291	0.520325203	NA	NA		1
879	0.499192246	NA	NA		1
1927	0.482288037	NA	NA		1
1075	0.490232558	NA	NA		1
2396	0.556643357	NA	NA		1
942	0.456475584	NA	NA		1
1459	0.52722063	NA	NA		1
501	0.558882236	NA	NA		1
922	0.495661605	NA	NA		1
1042	0.524952015	NA	NA		1
949	0.551724138	NA	NA		1
1631	0.52238806	NA	NA		1
732	0.414593698	NA	NA		1
414	0.538647343	NA	NA		1
3711	0.54562246	NA	NA		0
984	0.475728155	NA	NA		1
538	0.517819706	NA	NA		1
1344	0.508052709	NA	NA		1

1682	0.49106552	NA	NA		1
195	0.605128205	NA	NA		1
5619	0.501288185		1000	864	0
2305	0.51684032		341	788	1
84236	0.520695293	NA	NA		0
2544	0.549455535		881	885	1
1161	0.486381323	NA	NA		1
7550	0.525373576	NA	NA		1
1223	0.533890436	NA	NA		1
2445	0.480163599	NA	NA		1
1112	0.521582734	NA	NA		1
2410	0.496630378	NA	NA		1
1032	0.504573171	NA	NA		1
18051	0.53470437	NA	NA		1
1186	0.545	NA	NA		1
2782	0.544183445	NA	NA		1
1099	0.518653321	NA	NA		1
2662	0.598307292	NA	NA		1
1076	0.437131631	NA	NA		1
1480	0.452261307	NA	NA		1
1602	0.46281909	NA	NA		1
1189	0.566343042	NA	NA		1
8869	0.515969582	NA	NA		1
4904	0.548462354		1000	656	1
1118	0.574545455	NA	NA		1
1898	0.522377109	NA	NA		1
3537	0.51225676		880	931	0
3263	0.4540732		631	936	0
868	0.515007899	NA	NA		1
909	0.567656766	NA	NA		1
12014	0.492459016		1000	1000	1
8591	0.477475248		79	1000	1
617	0.465153971	NA	NA		1
5515	0.497327791	NA	NA		0
1384	0.516250945	NA	NA		1
6777	0.446423523		1000	1000	1
2867	0.499293785	NA	NA		1
648	0.447278912	NA	NA		1
488	0.536885246	NA	NA		1
1726	0.471480583	NA	NA		1
1233	0.644768856	NA	NA		1
510	0.582191781	NA	NA		1
2662	0.495679497	NA	NA		1
5195	0.508126977		889	853	0
15157	0.473737872	NA	NA		0
2926	0.569547796	NA	NA		1
1885	0.53064275	NA	NA		1
646	0.458204334	NA	NA		1
1278	0.478873239	NA	NA		1
7468	0.497519916	NA	NA		1
2846	0.477447745	NA	NA		1
540	0.583333333	NA	NA		1
886	0.535447761	NA	NA		0
3870	0.53719749	NA	NA		1
7471	0.466774716	NA	NA		1
1272	0.514285714	NA	NA		1
2373	0.527703307	NA	NA		1

2409	0.593567251	NA	NA	1
2204	0.495009074	1000	1000	1
1357	0.572758037	NA	NA	1
2160	0.556565657	NA	NA	1
2088	0.491660048	NA	NA	1
683	0.497803807	NA	NA	1
5966	0.521000195	739	936	1
1740	0.538253825	NA	NA	1
408	0.519607843	NA	NA	1
6074	0.534106183	878	1000	1
2666	0.465769231	934	1000	1
2071	0.52293578	NA	NA	1
1331	0.557413601	NA	NA	1
1277	0.477011494	NA	NA	1
4780	0.555939525	564	1000	1
21010	0.534631344	NA	NA	1
7178	0.552357456	118	1000	1
1782	0.563973064	NA	NA	1
1832	0.398801598	NA	NA	1
2030	0.515088986	993	1000	0
4367	0.543660582	940	872	1
6878	0.53294702	NA	NA	1
5819	0.475301439	NA	NA	1
6767	0.500164636	27	933	1
1207	0.507042254	NA	NA	1
3563	0.542807222	NA	NA	1
1660	0.56223176	NA	NA	1
1631	0.524831392	NA	NA	1
1429	0.514204545	NA	NA	1
1162	0.476905312	NA	NA	1
1581	0.515952598	NA	NA	1
1302	0.512882448	NA	NA	1
8261	0.489479513	385	939	1
2012	0.51874679	NA	NA	1
2012	0.525304292	NA	NA	0
1899	0.477093207	NA	NA	1
424	0.54954955	NA	NA	1
405	0.465116279	NA	NA	1
1529	0.4970569	NA	NA	1
570	0.480701754	NA	NA	1
7199	0.507177974	NA	NA	1
6865	0.535636057	230	955	0
2301	0.480696798	879	1000	1
2114	0.493975904	NA	NA	1
9791	0.452561983	NA	NA	0
4898	0.513525118	934	943	1
1188	0.459698849	NA	NA	1
2150	0.520698254	NA	NA	0
1722	0.516774194	NA	NA	1
1002	0.400651466	NA	NA	1
9555	0.496635966	728	938	0
768	0.528645833	NA	NA	1
1736	0.519585253	NA	NA	1
4235	0.47308642	815	1000	1
8667	0.587646077	NA	NA	0
4659	0.591206179	NA	NA	1
2242	0.521409456	1000	1000	1

1370	0.541883304	NA	NA		0
1088	0.555895865	NA	NA		1
6634	0.451069079		278	1000	1
9443	0.537022901		385	628	0
5040	0.547821682		1000	1000	1
54800	0.49442701	NA	NA		0
3543	0.513639906		275	937	1
4337	0.489884966		408	1000	1
1113	0.511867905	NA	NA		1
1458	0.518518519	NA	NA		1
747	0.459854015	NA	NA		1
561	0.436720143	NA	NA		1
7691	0.440334572	NA	NA		0
46897	0.449919485		315	1000	1
743	0.471063257	NA	NA		1
1362	0.482612383	NA	NA		1
699	0.479768786	NA	NA		1
9294	0.56099613		848	142	1
366	0.516393443	NA	NA		1
1594	0.555207026	NA	NA		1
1069	0.52666043	NA	NA		1
2646	0.51183432		859	918	1
5373	0.546831407		643	790	1
5598	0.469623915		219	1000	1
4461	0.50994575	NA	NA		1
1990	0.490296804	NA	NA		1
555	0.583783784	NA	NA		1
3963	0.459784674	NA	NA		1
7991	0.517165814	NA	NA		0
364	0.355704698	NA	NA		1
596	0.508571429	NA	NA		1
1265	0.487179487	NA	NA		1
1200	0.451666667	NA	NA		1
897	0.471291866	NA	NA		1
1293	0.487238979	NA	NA		1
7265	0.566593083		557	932	1
15269	0.597666719		246	1000	1
5451	0.512502423		881	1000	1
2001	0.493464052	NA	NA		1
2031	0.572649573	NA	NA		1
40504	0.559883155	NA	NA		1
3113	0.468843719	NA	NA		1
1938	0.538801262	NA	NA		1
1016	0.493110236	NA	NA		1
1369	0.418320611	NA	NA		1
2046	0.554455446	NA	NA		1
3466	0.496145544		883	943	1
1775	0.528571429	NA	NA		1
335	0.541218638	NA	NA		1
1620	0.543682796	NA	NA		1
3530	0.48204808		797	1000	1
2977	0.525555989	NA	NA		1
5960	0.477438137	NA	NA		0
4765	0.503712164	NA	NA		1
1960	0.516632017	NA	NA		0
656	0.506666667	NA	NA		1
4255	0.406300747		903	1000	1

1212	0.395778364	NA	NA	1
1321	0.429824561	NA	NA	1
6178	0.512785544	NA	NA	1
30766	0.53760507	272	955	1
4665	0.492909535	NA	NA	1
1189	0.518093557	NA	NA	1
6630	0.503041825	NA	NA	0
3204	0.561485643	NA	NA	1
1774	0.41995842	NA	NA	1
777	0.462033462	NA	NA	1
1130	0.520353982	NA	NA	1
134336	0.38084507	NA	NA	0
789	0.482889734	NA	NA	1
695	0.423376623	NA	NA	1
3267	0.471765229	NA	NA	1
1520	0.594052559	NA	NA	1
3140	0.530254777	NA	NA	1
2403	0.559782609	NA	NA	1
55059	0.51078307	765	870	0
4949	0.502724225	942	1000	1
2156	0.512387387	NA	NA	1
312	0.580128205	NA	NA	1
1952	0.524077869	NA	NA	1
12242	0.425652667	413	1000	1
23599	0.459546348	334	853	1
1259	0.516282764	NA	NA	1
1262	0.52603707	NA	NA	1
2651	0.482485405	NA	NA	1
2651	0.482381863	NA	NA	0
3687	0.52	NA	NA	1
3835	0.483347211	1000	1000	0
1302	0.537154989	NA	NA	1
1908	0.547169811	NA	NA	1
1550	0.452064381	NA	NA	1
2059	0.477452086	NA	NA	1
19885	0.493628809	NA	NA	0
4366	0.347826087	1000	1000	1
1144	0.488990826	NA	NA	1
923	0.417118093	NA	NA	1
1326	0.465309201	NA	NA	1
1108	0.447419669	NA	NA	1
1291	0.522653722	NA	NA	1
756	0.464285714	NA	NA	1
1223	0.482817869	NA	NA	1
2569	0.539884868	1000	1000	1
5399	0.474233529	934	943	1
3130	0.45473251	NA	NA	1
743	0.545608108	NA	NA	1
768	0.45294635	NA	NA	0
3471	0.532722513	1000	1000	0
612	0.498069498	NA	NA	1
1069	0.507015903	NA	NA	1
820	0.44603381	NA	NA	1
5437	0.452801228	NA	NA	1
534	0.61423221	NA	NA	1
9935	0.49011095	179	937	1
815	0.525412541	NA	NA	1

1059	0.44120603	NA	NA		1
4688	0.479166667		675	944	1
2702	0.493043478	NA	NA		1
11674	0.514571191		974	1000	0
18270	0.495475113		489	990	1
1356	0.460176991	NA	NA		1
1385	0.435379061	NA	NA		1
5886	0.463325653		806	938	1
868	0.460829493	NA	NA		1
6472	0.542211653		388	464	1
4735	0.513017176		523	829	0
4665	0.512935089		849	834	1
4418	0.516775935		644	1000	0
1049	0.325541619	NA	NA		0
7341	0.506804573		642	190	0
21793	0.373535792		218	957	1
6549	0.507597536		470	875	1
3522	0.502538071		910	535	0
1557	0.495825305	NA	NA		1
21774	0.335009141		153	1000	1
3472	0.499213527	NA	NA		0
4425	0.35645933	NA	NA		1
14198	0.36071682		170	1000	1
1558	0.538355218	NA	NA		1
7098	0.517857143	NA	NA		1
1250	0.455280445	NA	NA		0
1018	0.514041514	NA	NA		1
3883	0.375212585	NA	NA		1
7148	0.414625612		412	858	0
3963	0.558199028		212	934	0
825	0.506666667	NA	NA		1
1553	0.463687151	NA	NA		1
24549	0.48003043		566	898	1
1844	0.502990431	NA	NA		1
523	0.451632406	NA	NA		1
1954	0.478520935	NA	NA		1
12327	0.5090657	NA	NA		1
405	0.535802469	NA	NA		1
21467	0.534053405		849	1000	1
1135	0.409579668	NA	NA		1
2375	0.508269019	NA	NA		1
44963	0.384099766		270	881	0
1774	0.562460766	NA	NA		1
1054	0.546489564	NA	NA		1
2212	0.433341062	NA	NA		0
5213	0.42360915	NA	NA		1
31550	0.379801885	NA	NA		1
4937	0.505193187	NA	NA		1
1515	0.443564356	NA	NA		1
2183	0.496107573	NA	NA		1
1001	0.542566709	NA	NA		1
18088	0.514490495		129	935	0
503	0.495029821	NA	NA		1
793	0.416184971	NA	NA		1
4563	0.544548976	NA	NA		1
3284	0.506592939		550	938	1
7177	0.380805956		790	694	1

288	0.448430493	NA	NA	1
10547	0.383908046	792	906	1
2027	0.408293111	NA	NA	0
5624	0.507741251	835	619	1
94302	0.444370949	NA	NA	0
11101	0.350281602	246	816	0
1430	0.516083916	NA	NA	1
9759	0.530619972	616	901	0
2653	0.535200311	1000	1000	1
1996	0.499224004	NA	NA	1
3889	0.54199012	217	1000	1
1984	0.526206539	NA	NA	1
9178	0.339784473	512	683	0
2213	0.473193473	NA	NA	1
5394	0.575929344	603	422	0
1867	0.520282187	NA	NA	1
1470	0.457551385	NA	NA	1
900	0.509803922	NA	NA	1
4094	0.488031265	NA	NA	1
774	0.410852713	NA	NA	1
1181	0.444971537	NA	NA	1
2018	0.459093584	NA	NA	1
2417	0.466072313	725	877	1
1690	0.491161616	NA	NA	1
1912	0.5041841	NA	NA	1
424	0.501416431	NA	NA	1
2385	0.497636442	942	1000	1
1513	0.500201613	NA	NA	0
12320	0.482057796	415	1000	0
32719	0.538393095	900	581	0
15181	0.523831776	NA	NA	1
2115	0.504812834	NA	NA	1
2652	0.511405836	NA	NA	1
1515	0.496314496	NA	NA	1
1431	0.429268293	NA	NA	1
2829	0.574696545	NA	NA	1
10009	0.398821056	NA	NA	0
1621	0.604565083	NA	NA	1
1784	0.351318581	NA	NA	1
1435	0.452078032	NA	NA	1
2708	0.518909627	522	1000	0
2786	0.461538462	NA	NA	1
1084	0.47826087	NA	NA	1
1289	0.441880342	NA	NA	1
1399	0.461102107	NA	NA	1
6938	0.417238422	NA	NA	0
4467	0.519190717	NA	NA	1
10525	0.360577402	84	1000	0
2557	0.494075219	NA	NA	1
470	0.501199041	NA	NA	1
479	0.475409836	NA	NA	1
74948	0.49806514	694	1000	1
744	0.490620491	NA	NA	1
414	0.512534819	NA	NA	1
19432	0.396372187	231	687	1
621	0.468599034	NA	NA	1
1872	0.512820513	NA	NA	1

1546	0.554333765	NA	NA		1
447	0.516778523	NA	NA		1
1685	0.576260763	NA	NA		1
1882	0.517632242	NA	NA		1
1822	0.507509387	NA	NA		1
1044	0.401273885	NA	NA		1
41033	0.487145242	NA	NA		1
52529	0.483660131		708	1000	1
1940	0.523040096	NA	NA		1
3499	0.50386236		737	1000	1
8423	0.529721968		793	901	1
460	0.348258706	NA	NA		1
1357	0.545118571	NA	NA		0
1966	0.531299316	NA	NA		1
871	0.457564576	NA	NA		1
15974	0.391949629		827	889	1
4625	0.548674589	NA	NA		0
939	0.571884984	NA	NA		1
3862	0.464031108	NA	NA		1
2692	0.565909996		819	1000	1
2730	0.497271073	NA	NA		1
1754	0.474815725	NA	NA		1
14859	0.559260105		691	920	1
387	0.46728972	NA	NA		1
2225	0.552176506	NA	NA		1
5173	0.449763033		519	1000	1
2554	0.541964286		936	812	1
1531	0.522534291	NA	NA		1
4286	0.461886413		541	1000	0
1140	0.578947368	NA	NA		1
8270	0.499753977	NA	NA		0
351	0.641025641	NA	NA		1
26358	0.459505062		116	1000	1
1866	0.524314214	NA	NA		1
2519	0.384761905	NA	NA		1
1577	0.409667195	NA	NA		1
8747	0.497572945		1000	933	0
1008	0.424969988	NA	NA		1
1953	0.502896261	NA	NA		1
818	0.513951979	NA	NA		0
8332	0.502095599	NA	NA		0
15747	0.529347065		295	1000	1
5334	0.401420959		124	1000	1
3167	0.523793566		503	1000	1
15873	0.490616203		946	1000	1
1247	0.464252553	NA	NA		1
603	0.497512438	NA	NA		1
1912	0.512335055	NA	NA		1
1098	0.521321962	NA	NA		1
1665	0.436036036	NA	NA		1
1768	0.483072917	NA	NA		1
1812	0.520971302	NA	NA		1
558	0.532803181	NA	NA		1
948	0.428270042	NA	NA		1
1833	0.513181019	NA	NA		1
9408	0.494047619		1000	1000	1
2482	0.497619048	NA	NA		1

475	0.475789474	NA	NA	1
3750	0.510846746	NA	NA	1
2212	0.413596785	NA	NA	0
3584	0.400669643	1000	1000	1
2074	0.539537126	1000	1000	1
489	0.482617587	NA	NA	1
33098	0.541469993	411	1000	0
4786	0.345860343	836	1000	1
3518	0.533264392	682	891	1
27424	0.472937724	946	785	1
832	0.441340782	NA	NA	1
2212	0.48106766	NA	NA	1
1598	0.464092141	NA	NA	1
1170	0.442222222	NA	NA	1
3927	0.489473684	NA	NA	1
965	0.504191617	NA	NA	1
865	0.576878613	NA	NA	1
12856	0.458310326	NA	NA	1
2175	0.47515381	NA	NA	1
5544	0.574052813	612	1000	1
10904	0.566550148	1000	1000	0
8046	0.51552795	NA	NA	0
1886	0.471145564	NA	NA	1
1173	0.449275362	NA	NA	1
7540	0.566297011	230	1000	1
1529	0.49412686	NA	NA	1
985	0.440677966	NA	NA	1
9022	0.521521226	1000	885	1
1861	0.489130435	NA	NA	1
656	0.431578947	NA	NA	1
5327	0.552651611	595	417	1
4145	0.50649695	851	940	1
4549	0.543365653	873	1000	0
5152	0.553267045	521	940	1
2075	0.531331593	NA	NA	1
1089	0.432506887	NA	NA	1
720	0.43030303	NA	NA	1
7067	0.480855296	NA	NA	1
8166	0.526910657	NA	NA	1
1753	0.566317088	NA	NA	1
13699	0.549237638	NA	NA	1
2648	0.473182957	NA	NA	1
1686	0.571628232	NA	NA	1
494	0.487854251	NA	NA	1
4015	0.448313682	NA	NA	1
3386	0.548404543	NA	NA	1
1541	0.472906404	NA	NA	1
675	0.548148148	NA	NA	1
1747	0.517967782	NA	NA	1
8838	0.52120812	NA	NA	0
1099	0.372297838	NA	NA	1
5777	0.390200953	210	555	1
4429	0.496644295	NA	NA	1
2099	0.525628141	NA	NA	1
1245	0.467619048	NA	NA	1
1960	0.479532164	NA	NA	1
3823	0.552096306	1000	498	1

2019	0.549606889	NA	NA	0
758	0.423809524	NA	NA	1
1261	0.488734835	NA	NA	1
19116	0.494813911	NA	NA	1
2790	0.489605431	NA	NA	0
834	0.574340528	NA	NA	1
2388	0.49298325	1000	878	1
4839	0.50894747	62	1000	0
1598	0.608260325	NA	NA	1
1095	0.536986301	NA	NA	1
2399	0.525635682	NA	NA	1
1904	0.478114478	NA	NA	1
2896	0.502744592	NA	NA	1
530	0.514705882	NA	NA	1
797	0.457142857	NA	NA	1
602	0.478743068	NA	NA	1
464	0.428571429	NA	NA	1
1036	0.474903475	NA	NA	1
2769	0.480141565	NA	NA	1
4716	0.511755878	786	904	1
1269	0.513790386	NA	NA	1
2559	0.595066804	NA	NA	1
4871	0.526594337	559	1000	1
1930	0.511049724	NA	NA	0
11586	0.469922573	NA	NA	1
321	0.523364486	NA	NA	1
2503	0.546388637	NA	NA	0
2026	0.499452355	NA	NA	1
1370	0.56185567	NA	NA	1
2118	0.477835052	NA	NA	1
2706	0.488065327	NA	NA	1
8899	0.550925926	1000	1000	1
3319	0.465109034	NA	NA	1
6240	0.451361047	300	779	0
4980	0.515835364	NA	NA	0
2323	0.555070883	NA	NA	1
1803	0.52603707	NA	NA	1
23799	0.476468249	511	1000	0
7793	0.498666292	NA	NA	1
8812	0.520447795	NA	NA	0
1063	0.504961411	NA	NA	1
2116	0.48463357	NA	NA	1
1568	0.488170347	NA	NA	1
2951	0.47985348	NA	NA	0
1298	0.468412943	NA	NA	1
978	0.442740286	NA	NA	1
5723	0.480433474	NA	NA	1
1185	0.514492754	NA	NA	1
4382	0.543393276	943	902	1
16209	0.562615344	NA	NA	1
1191	0.519788918	NA	NA	1
12552	0.429639259	372	1000	1
1735	0.561891516	NA	NA	1
3530	0.536281532	NA	NA	1
2169	0.531173594	NA	NA	1
6943	0.390697674	324	882	1
4126	0.543394407	NA	NA	0

6618	0.487824841	NA	NA		1
356	0.519662921	NA	NA		1
4236	0.480108723	943		1000	1
1254	0.537845058	NA	NA		1
7597	0.510500808	NA	NA		1
19467	0.548340374	NA	NA		1
575	0.504816956	NA	NA		1
747	0.495652174	NA	NA		1
1853	0.535714286	NA	NA		1
1905	0.560864619	NA	NA		1
6027	0.477007772	539		1000	0
1232	0.443316413	NA	NA		1
2460	0.474007826	NA	NA		1
2172	0.541371158	990		1000	0
2277	0.451539339	NA	NA		1
899	0.500556174	NA	NA		1
2393	0.478164976	874		1000	1
1714	0.564589666	NA	NA		1
4999	0.532079646	1000		658	1
816	0.484615385	NA	NA		1
6188	0.509275237	536		1000	1
5136	0.54806538	831		998	1
22154	0.467578947	631		875	1
4269	0.48281092	NA	NA		1
1531	0.542647059	NA	NA		1
1185	0.542780749	NA	NA		1
4359	0.520521881	1000		1000	1
3739	0.523309379	818		1000	0
2529	0.518502765	1000		883	1
3387	0.521219366	NA	NA		1
979	0.484256243	NA	NA		1
9308	0.451056911	139		1000	1
3091	0.507610619	NA	NA		1
3267	0.50377488	NA	NA		1
2314	0.558924205	933		798	1
5556	0.578500481	NA	NA		0
1649	0.520865533	NA	NA		1
5766	0.536157391	NA	NA		1
1624	0.577213823	NA	NA		0
2885	0.514670896	NA	NA		1
3680	0.536286732	NA	NA		0
2145	0.450026164	NA	NA		1
3114	0.425149701	311		1000	1
5924	0.562373567	583		1000	1
10596	0.484682713	1000		807	1
5932	0.566829124	253		868	0
2947	0.450976778	932		1000	0
1224	0.526627219	NA	NA		1
25832	0.418141314	NA	NA		0
5530	0.492927308	NA	NA		0
2067	0.478537361	NA	NA		1
853	0.512309496	NA	NA		1
9144	0.535420744	190		728	1
1519	0.460431655	NA	NA		1
914	0.571115974	NA	NA		1
1769	0.44701087	NA	NA		1
4054	0.518408551	NA	NA		0

9988	0.446659365	189	1000	1
1805	0.454847645	NA	NA	1
3254	0.492154066	NA	NA	1
6063	0.408174851	290	1000	0
5478	0.530343277	968	750	0
13565	0.506847231	317	1000	0
2043	0.549722362	NA	NA	1
1789	0.463400576	NA	NA	1
2143	0.498833411	NA	NA	1
4958	0.549241008	NA	NA	0
1990	0.561456103	NA	NA	0
1539	0.619209809	NA	NA	1
2606	0.534535687	1000	1000	1
684	0.528089888	NA	NA	1
822	0.590612777	NA	NA	1
1209	0.452996475	NA	NA	0
2170	0.501463415	NA	NA	1
1644	0.512773723	NA	NA	1
15589	0.537669377	916	1000	1
4589	0.530544045	NA	NA	0
1865	0.505093834	NA	NA	1
1891	0.554678692	NA	NA	1
2335	0.541271989	NA	NA	1
8266	0.47424054	162	1000	1
2229	0.56316297	885	1000	1
3617	0.487475915	455	934	1
1112	0.515892421	NA	NA	1
19387	0.499351492	NA	NA	1
2518	0.507955936	933	1000	1
2479	0.472850679	NA	NA	1
1241	0.489927478	NA	NA	1
3075	0.468540345	NA	NA	1
2002	0.5554826	NA	NA	1
2391	0.50729927	NA	NA	1
7201	0.59929313	NA	NA	1
1156	0.525641026	NA	NA	1
946	0.542283298	NA	NA	1
6537	0.505964215	NA	NA	1
3494	0.554647933	886	833	0
2215	0.534911961	NA	NA	1
1375	0.474885845	NA	NA	1
2985	0.578186597	1000	931	1
35992	0.434466019	NA	NA	0
4056	0.48728095	NA	NA	1
5067	0.520875195	867	861	1
11946	0.506714414	673	886	1
6074	0.462852007	522	1000	1
3692	0.53182986	941	904	1
2296	0.598034837	943	1000	1
783	0.602225313	NA	NA	1
2032	0.537329127	NA	NA	1
1967	0.563044741	NA	NA	1
4980	0.458107294	NA	NA	1
2305	0.5	NA	NA	1
1103	0.546632124	NA	NA	0
5868	0.548044218	230	877	1
1595	0.537827715	NA	NA	1

2421	0.49382716	724	880	1
14030	0.460365854	NA	NA	1
3361	0.511079679	384	729	1
1672	0.555639667	NA	NA	1
11235	0.567226891	NA	NA	1
1418	0.465444288	NA	NA	1
3112	0.511175497	488	1000	0
3737	0.539737758	NA	NA	1
14503	0.521261347	NA	NA	1
7330	0.536767819	NA	NA	1
5364	0.513616213	388	1000	0
3296	0.508031496	944	1000	1
5806	0.442542423	NA	NA	0
962	0.540540541	NA	NA	1
2360	0.466101695	1000	1000	1
4114	0.519856771	NA	NA	1
1925	0.392207792	NA	NA	1
2219	0.566365008	1000	879	0
13388	0.527906977	NA	NA	1
1163	0.465176268	NA	NA	1
922	0.496969697	NA	NA	1
1402	0.564141035	NA	NA	1
3261	0.513274336	NA	NA	1
2315	0.5093412	NA	NA	1
6472	0.536226343	1000	1000	1
2766	0.540791599	1000	1000	1
11395	0.478995332	418	1000	0
3994	0.472913118	950	859	1
8019	0.45376476	1000	957	1
12305	0.468491039	322	1000	0
6377	0.492910053	NA	NA	1
12698	0.49447021	NA	NA	1
762	0.611548556	NA	NA	1
1370	0.497991968	NA	NA	1
781	0.38957476	NA	NA	1
28251	0.450691656	1000	1000	1
3713	0.486133768	829	1000	1
3113	0.532551098	700	1000	1
4165	0.416619559	890	1000	0
636	0.469348659	NA	NA	1
1323	0.547241119	NA	NA	1
1794	0.42419175	NA	NA	1
4842	0.531032986	915	1000	1
1304	0.494004796	NA	NA	1
1172	0.484725051	NA	NA	1
707	0.459876543	NA	NA	1
2238	0.462167689	NA	NA	1
8179	0.486304781	135	939	0
1908	0.387840671	NA	NA	1
41050	0.541308055	256	637	0
1908	0.421633554	NA	NA	1
476	0.47826087	NA	NA	1
476	0.440389294	NA	NA	1
11334	0.520958084	148	866	0
652	0.481595092	NA	NA	1
6811	0.501937984	NA	NA	1
961	0.44640999	NA	NA	1

563	0.414979757	NA	NA		1
30851	0.478927203	208		1000	1
2838	0.520112631	946		752	1
556	0.475903614	NA	NA		1
5514	0.529718975	NA	NA		1
560	0.428571429	NA	NA		1
2143	0.590174129	NA	NA		1
859	0.5375	NA	NA		1
1257	0.476549414	NA	NA		1
1165	0.460144928	NA	NA		1
1164	0.460490463	NA	NA		1
510	0.409326425	NA	NA		1
962	0.4875	NA	NA		1
1243	0.492827004	NA	NA		1
3017	0.526019224	1000		1000	1
2164	0.487481591	NA	NA		1
2029	0.510709505	NA	NA		1
1638	0.512437811	NA	NA		1
348	0.488505747	NA	NA		1
1724	0.54375	NA	NA		1
1175	0.54460719	NA	NA		0
619	0.404718693	NA	NA		1
1379	0.399277326	NA	NA		1
2253	0.39325344	NA	NA		1
2715	0.497605893	NA	NA		1
1229	0.477408355	NA	NA		1
3366	0.52584493	NA	NA		1
1373	0.532410779	NA	NA		1
810	0.440903054	NA	NA		1
543	0.453038674	NA	NA		1
11775	0.483504399	NA	NA		1
934	0.539614561	NA	NA		1
1793	0.422295278	NA	NA		0
2307	0.513220633	NA	NA		1
1712	0.473714953	NA	NA		1
13100	0.506410256	NA	NA		1
1240	0.405723906	NA	NA		1
2165	0.513458608	NA	NA		1
1294	0.467912266	NA	NA		1
13167	0.503679318	1000		941	1
481	0.423529412	NA	NA		1
27523	0.392638037	1000		130	1
672	0.637704918	NA	NA		1
1800	0.412222222	NA	NA		1
4016	0.477347507	935		1000	1
2963	0.463719204	NA	NA		1
1471	0.508433735	NA	NA		1
2637	0.422449754	1000		1000	1
1744	0.481481481	NA	NA		1
3311	0.561851264	NA	NA		1
2288	0.469101124	NA	NA		1
1477	0.525273224	NA	NA		1
2980	0.472819711	883		828	1
2676	0.460725076	NA	NA		1
3602	0.523305085	NA	NA		1
1699	0.499046408	NA	NA		1
2325	0.501606241	904		1000	1

12853	0.548970989	NA	NA		0
1620	0.528490028	NA	NA		1
5465	0.520210896		143	1000	1
1752	0.607305936	NA	NA		1
4680	0.532563774		822	924	0
5957	0.534727144		1000	1000	0
1303	0.521872602	NA	NA		1
16576	0.465838509		923	1000	1
723	0.539419087	NA	NA		1
530	0.469811321	NA	NA		1
1335	0.445669291	NA	NA		1
2293	0.541919192	NA	NA		1
19748	0.521371327	NA	NA		1
3107	0.528074866		938	1000	1
7813	0.532565889	NA	NA		1
26149	0.537782486		1000	1000	0
6018	0.573816156	NA	NA		0
3334	0.515283843		932	940	1
2505	0.492677308	NA	NA		0
16021	0.460325048	NA	NA		1
1338	0.585201794	NA	NA		1
1392	0.479372937	NA	NA		1
3661	0.518230234		394	936	1
1394	0.41401793	NA	NA		1
1411	0.446327684	NA	NA		1
4504	0.367644022		156	1000	0
982	0.556039173	NA	NA		1
743	0.4646098	NA	NA		1
982	0.548736462	NA	NA		1
11408	0.505154639	NA	NA		1
6659	0.499030632	NA	NA		1
10045	0.431962942	NA	NA		1
13148	0.565911911	NA	NA		0
3015	0.529655172	NA	NA		1
923	0.452243959	NA	NA		1
24378	0.516369048	NA	NA		0
1965	0.492316448	NA	NA		1
9114	0.523618726	NA	NA		1
1788	0.377516779	NA	NA		1
1252	0.469011725	NA	NA		1
47363	0.481717011		1000	761	1
11974	0.55370617	NA	NA		1
603	0.368159204	NA	NA		1
3445	0.522614724		779	942	1
1218	0.389319552	NA	NA		1
2475	0.535698642		941	781	1
4479	0.541077739		861	947	1
34154	0.50897289	NA	NA		1
5500	0.43357729		928	1000	1
27090	0.470653378	NA	NA		1
47618	0.34802624	NA	NA		1
9121	0.511120555	NA	NA		1
502	0.430925222	NA	NA		1
3385	0.528277215		832	1000	1
11934	0.575	NA	NA		1
1031	0.460103627	NA	NA		1
1643	0.566074951	NA	NA		1

1614	0.449541284	NA	NA	0
658	0.391891892	NA	NA	0
1215	0.50781893	NA	NA	1
9814	0.50691327	NA	NA	1
888	0.387387387	NA	NA	1
1202	0.55565371	NA	NA	1
9646	0.470333894	NA	NA	1
1090	0.38261851	NA	NA	1
18692	0.451807229	NA	NA	1
1613	0.423751687	NA	NA	1
847	0.478605388	NA	NA	1
4211	0.528532609	NA	NA	1
3384	0.423167849	NA	NA	1
8497	0.546186896	NA	NA	1
1206	0.404013962	NA	NA	1
2054	0.512242268	NA	NA	1
2591	0.512157468	NA	NA	1
10065	0.348161267	1000	1000	0
893	0.600223964	NA	NA	1
2464	0.50641574	NA	NA	1
2630	0.521292776	162	1000	1
19230	0.411139006	62	1000	0
1680	0.50744879	NA	NA	1
883	0.506228766	NA	NA	1
1246	0.460636516	NA	NA	1
1174	0.391054313	NA	NA	1
2546	0.428104575	NA	NA	1
1656	0.381038647	NA	NA	1
734	0.392156863	NA	NA	1
1110	0.425287356	NA	NA	1
1490	0.461370262	NA	NA	1
2475	0.52	1000	1000	1
678	0.465635739	NA	NA	1
1572	0.514089347	NA	NA	1
1481	0.492264416	NA	NA	1
1201	0.440628067	NA	NA	1
597	0.493530499	NA	NA	1
3159	0.562519785	NA	NA	1
1259	0.507545671	NA	NA	1
534	0.424050633	NA	NA	1
20438	0.488467262	456	929	1
853	0.545226131	NA	NA	1
34268	0.504977132	896	860	1
824	0.419207317	NA	NA	1
948	0.546786922	NA	NA	1
1693	0.438275251	NA	NA	1
707	0.386503067	NA	NA	1
601	0.434944238	NA	NA	1
670	0.381849315	NA	NA	1
5485	0.421763694	NA	NA	1
1674	0.510752688	NA	NA	1
1473	0.450777202	NA	NA	1
13690	0.435310603	474	1000	0
1237	0.540824576	NA	NA	1
22679	0.48470255	NA	NA	1
11435	0.477131135	NA	NA	0
1135	0.481060606	NA	NA	1

13198	0.560517529		666		669	0
4182	0.492826399		1000		1000	1
2052	0.477721376	NA		NA		1
628	0.559292035	NA		NA		1
2796	0.511556912		696		927	1
895	0.447619048	NA		NA		1
896	0.53046595	NA		NA		1
984	0.46969697	NA		NA		1
1971	0.488936859	NA		NA		1
6980	0.468568977	NA		NA		1
4035	0.519495413		937		872	1
576	0.479166667	NA		NA		1
468	0.536324786	NA		NA		1
898	0.443207127	NA		NA		1
569	0.451669596	NA		NA		1
1415	0.533568905	NA		NA		1
1799	0.417117117	NA		NA		1
6953	0.424563729	NA		NA		0
6082	0.397199236		831		1000	1
4250	0.538337369	NA		NA		0
9080	0.549166939	NA		NA		1
1235	0.530364372	NA		NA		1
16689	0.540492435		743		1000	1
7277	0.535670103	NA		NA		1
28355	0.484871124		1000		1000	1
1249	0.452484743	NA		NA		1
3123	0.48341396	NA		NA		1
22266	0.477066229	NA		NA		0
1182	0.503384095	NA		NA		1
1250	0.443037975	NA		NA		1
2500	0.495980348		206		1000	1
4285	0.563661873		745		1000	1
8383	0.503311258	NA		NA		1
2240	0.476470588		933		935	1
9728	0.48879525		419		837	0
515	0.484716157	NA		NA		1
2556	0.525170648	NA		NA		1
2104	0.479768786		1000		1000	1
660	0.471212121	NA		NA		1
1103	0.40040858	NA		NA		1
1937	0.4	NA		NA		1
11012	0.461809636		198		886	1
4608	0.482340809		515		1000	1
1889	0.514227642	NA		NA		1
1293	0.4092827	NA		NA		1
1304	0.409863946	NA		NA		1
1295	0.430520034	NA		NA		1
1717	0.407137064	NA		NA		1
6515	0.449464612	NA		NA		0
1240	0.432029795	NA		NA		1
518	0.505154639	NA		NA		1
582	0.413461538	NA		NA		1
4034	0.498012192		873		941	1
800	0.437400951	NA		NA		1
3349	0.441021127	NA		NA		1
63149	0.489519615	NA		NA		0
654	0.600917431	NA		NA		1

40451	0.438788828	NA	NA	1
1849	0.430005907	NA	NA	1
3835	0.46747076	NA	NA	1
1568	0.513207547	NA	NA	1
1861	0.476581758	NA	NA	1
985	0.440677966	NA	NA	1
565	0.412389381	NA	NA	1
2197	0.505689577	NA	NA	1
1656	0.416774194	NA	NA	1
549	0.444444444	NA	NA	1
1342	0.537688442	NA	NA	1
535	0.390446521	NA	NA	1
9403	0.5395189	659	970	1
506	0.623582766	NA	NA	1
1469	0.543909348	NA	NA	1
1021	0.431929481	NA	NA	1
4385	0.474977252	1000	887	1
2471	0.524539877	912	994	1
954	0.456062291	NA	NA	1
1858	0.507048872	NA	NA	0
4242	0.492694331	NA	NA	1
2345	0.466098081	NA	NA	1
1233	0.469586375	NA	NA	1
1207	0.407986111	NA	NA	1
1212	0.393626184	NA	NA	1
1199	0.44591029	NA	NA	1
1225	0.481196581	NA	NA	1
1117	0.524762381	NA	NA	0
474	0.419831224	NA	NA	1
1545	0.475817923	NA	NA	1
1080	0.468328141	NA	NA	1
2773	0.510282776	927	983	1
10327	0.42878137	NA	NA	0
2783	0.323877069	NA	NA	1
993	0.463752665	NA	NA	1
2721	0.535733622	NA	NA	0
2397	0.467412772	NA	NA	1
2205	0.543310658	NA	NA	1
1661	0.438629283	NA	NA	1
4756	0.47751967	381	739	0
38653	0.452049497	NA	NA	1
1722	0.380371661	NA	NA	1
1346	0.526645768	NA	NA	1
20621	0.452665441	NA	NA	0
24638	0.538468652	628	774	1
453	0.59602649	NA	NA	1
572	0.527972028	NA	NA	1
29841	0.546828787	389	768	1
3525	0.441358025	718	867	1
11510	0.476736345	196	938	1
4012	0.541391941	1000	926	1
11338	0.523208469	279	843	0
5489	0.479133226	NA	NA	1
6671	0.537065053	586	928	1
1367	0.473382473	NA	NA	1
4897	0.497959184	719	839	1
696	0.521551724	NA	NA	1

7624	0.520551924		621	877	1
7114	0.516929699	NA	NA		0
1680	0.458333333	NA	NA		1
23398	0.494677198		207	1000	0
18942	0.53258427		187	1000	1
38997	0.477930175		763	1000	1
965	0.424345848	NA	NA		1
633	0.364273205	NA	NA		1
1587	0.498347107	NA	NA		1
1584	0.462752525	NA	NA		1
1640	0.502890173	NA	NA		1
5068	0.499317872	NA	NA		1
4749	0.510422871		454	796	1
1803	0.430693069	NA	NA		0
4102	0.479231568	NA	NA		1
6838	0.454371715	NA	NA		1
4713	0.471068249	NA	NA		1
3943	0.470397929		1000	1000	1
1260	0.51984127	NA	NA		1
58829	0.478186646	NA	NA		0
318	0.591194969	NA	NA		1
431	0.52900232	NA	NA		1
2049	0.53147877		1000	1000	1
411	0.571776156	NA	NA		1
2104	0.445890006	NA	NA		1
622	0.450980392	NA	NA		1
7033	0.543195266		590	1000	1
7940	0.468136347	NA	NA		1
395	0.577380952	NA	NA		1
4974	0.522508399	NA	NA		1
26273	0.511084952	NA	NA		1
2748	0.38209607		1000	1000	1
1648	0.521423863	NA	NA		1
2281	0.351674641	NA	NA		1
2928	0.431693989	NA	NA		1
662	0.484949833	NA	NA		1
3021	0.569779287	NA	NA		1
2598	0.504676871	NA	NA		1
4839	0.472840606	NA	NA		1
1295	0.482982172	NA	NA		1
1731	0.514097181	NA	NA		1
210	0.504761905	NA	NA		1
2481	0.43957346	NA	NA		1
4116	0.513842173	NA	NA		1
3899	0.444444444	NA	NA		0
1535	0.48534202	NA	NA		1
917	0.43792633	NA	NA		1
5694	0.511511512	NA	NA		1
3486	0.511144131		892	449	1
2837	0.321818823		1000	1000	1
5428	0.555364807		440	883	1
18773	0.528001149		175	919	1
25771	0.494727592		521	1000	1
1105	0.39638009	NA	NA		1
8529	0.489779006		490	1000	1
900	0.478888889	NA	NA		1
876	0.457762557	NA	NA		1

2592	0.484251969		897	1000	1
7476	0.489046249		1000	1000	0
2010	0.488972566	NA	NA		0
6624	0.55769694		324	936	0
1488	0.417065391	NA	NA		1
1438	0.431613432	NA	NA		1
1462	0.432278994	NA	NA		1
28997	0.476835353	NA	NA		1
3899	0.494246793		1000	1000	1
2116	0.519072165	NA	NA		1
973	0.503282276	NA	NA		1
2757	0.524703179		1000	854	1
40864	0.517578994	NA	NA		1
10426	0.50265861	NA	NA		0
5340	0.498069498	NA	NA		1
1051	0.467614534	NA	NA		1
11841	0.437190083		686	63	1
1558	0.436915888	NA	NA		1
13546	0.544767537		1000	920	1
1182	0.4678511	NA	NA		1
434	0.578341014	NA	NA		1
1661	0.544451655	NA	NA		1
3370	0.434694566		1000	864	1
21632	0.522026432	NA	NA		1
3362	0.524435457		678	934	1
3206	0.532453245		887	942	1
1437	0.505309735	NA	NA		1
14936	0.504237288		1000	1000	1
49080	0.510583376	NA	NA		1
2932	0.505684402	NA	NA		1
5614	0.498592682		360	814	1
2402	0.51754386	NA	NA		1
15471	0.547489824		259	1000	1
5168	0.58428246	NA	NA		1
1869	0.602787456	NA	NA		1
717	0.618618619	NA	NA		1
4867	0.565794066	NA	NA		0
19648	0.520624377	NA	NA		0
13410	0.485291464		462	1000	0
26077	0.545969319		124	1000	0
574	0.512670565	NA	NA		1
5773	0.549097314	NA	NA		1
1833	0.465224111	NA	NA		1
52548	0.363412229		434	390	1
826	0.389301634	NA	NA		1
1401	0.534883721	NA	NA		1
140146	0.434310968	NA	NA		1
10212	0.50733945	NA	NA		1
23357	0.578512397	NA	NA		0
73725	0.482902171	NA	NA		1
1836	0.535547786	NA	NA		1
649	0.43605547	NA	NA		1
14258	0.504106485	NA	NA		1
1122	0.40070922	NA	NA		1
5347	0.582381239		515	1000	1
2070	0.509372453	NA	NA		1
3001	0.561342593	NA	NA		1

31788	0.523122457	NA	NA		1
952	0.551633987	NA	NA		1
4278	0.50489469	917		1000	1
4521	0.523556735	NA	NA		1
44509	0.475014583	NA	NA		1
4600	0.515977844	374		942	1
3215	0.39571949	NA	NA		1
1159	0.486354776	NA	NA		1
451	0.507731959	NA	NA		1
44519	0.488948787	NA	NA		1
2066	0.533619456	NA	NA		1
3860	0.564923515	603		1000	1
2329	0.520328917	922		938	1
3297	0.48892933	1000		1000	1
5094	0.518163804	768		1000	1
1630	0.505725191	NA	NA		1
922	0.615384615	NA	NA		1
1197	0.568922306	NA	NA		1
2848	0.485957132	947		911	1
2848	0.486326681	947		911	1
2427	0.477626459	703		1000	1
19342	0.443856227	NA	NA		1
2747	0.580099885	856		1000	1
609	0.556650246	NA	NA		1
636	0.572327044	NA	NA		1
917	0.615204678	NA	NA		1
1919	0.483386924	NA	NA		1
4562	0.483486239	NA	NA		1
4731	0.520668058	285		1000	1
1370	0.56185567	NA	NA		1
705	0.523404255	NA	NA		1
2004	0.589928058	NA	NA		1
6442	0.360262009	234		1000	1
1741	0.401499659	NA	NA		1
464	0.409876543	NA	NA		1
1579	0.51626898	NA	NA		1
1983	0.526106697	NA	NA		1
1883	0.548349057	NA	NA		1
810	0.482716049	NA	NA		1
1895	0.436373874	NA	NA		1
2012	0.426299046	NA	NA		1
8174	0.56805485	228		868	1
2068	0.534816248	NA	NA		1
2009	0.523922312	NA	NA		1
3436	0.490351873	889		819	1
2766	0.495952345	506		941	1
5468	0.480626546	NA	NA		0
4977	0.490603233	NA	NA		0
9218	0.555276382	NA	NA		0
1329	0.464622642	NA	NA		1
19221	0.523469459	223		808	1
11643	0.509537572	NA	NA		1
1233	0.48057041	NA	NA		0
3514	0.524409449	509		1000	1
10210	0.485572139	474		964	1
17533	0.484387727	NA	NA		0
3893	0.464285714	NA	NA		1

670	0.389885808	NA	NA		1
3159	0.453070175	231	1000		1
4186	0.509891849	848	939		1
784	0.405925926	NA	NA		1
4705	0.563914193	NA	NA		1
2271	0.531483928	1000	1000		1
19756	0.488471294	NA	NA		0
2372	0.444273467	936	1000		1
1376	0.472563473	NA	NA		1
1326	0.5175	NA	NA		1
6246	0.532741398	NA	NA		1
1471	0.502017756	NA	NA		0
76777	0.507848569	NA	NA		1
774	0.534883721	NA	NA		1
14119	0.457698617	NA	NA		0
645	0.575396825	NA	NA		1
953	0.502555366	NA	NA		1
18178	0.524229075	NA	NA		0
740	0.39212828	NA	NA		1
628	0.406185567	NA	NA		1
63032	0.492473729	NA	NA		1
18278	0.482287082	NA	NA		0
21751	0.491049126	401	1000		1
9995	0.515813424	NA	NA		1
9062	0.537432691	54	939		1
1878	0.490291262	NA	NA		1
32326	0.514301645	NA	NA		0
4755	0.447318612	1000	1000		1
4643	0.551550388	NA	NA		1
1298	0.580060423	NA	NA		1
383	0.412532637	NA	NA		1
3374	0.426818951	975	802		0
5400	0.455555556	1000	1000		1
285	0.585964912	NA	NA		1
20130	0.49349906	527	982		0
267	0.505617978	NA	NA		1
11138	0.595896147	212	743		1
21346	0.579169175	NA	NA		1
900	0.566666667	NA	NA		1
1198	0.46327212	NA	NA		1
1721	0.581092095	NA	NA		1
2605	0.392706334	NA	NA		1
4740	0.517269737	226	940		0
717	0.454545455	NA	NA		1
1774	0.48825257	NA	NA		1
3043	0.489944521	NA	NA		1
817	0.465313029	NA	NA		1
3143	0.491062395	884	1000		1
2920	0.433843738	627	1000		1
641	0.371212121	NA	NA		1
640	0.377394636	NA	NA		1
599	0.352320675	NA	NA		1
18024	0.504922644	NA	NA		1
739	0.43315508	NA	NA		1
606	0.447194719	NA	NA		1
444	0.396396396	NA	NA		1
586	0.453815261	NA	NA		1

597	0.370184255	NA	NA		1
659	0.487666034	NA	NA		1
20733	0.5	NA	NA		1
843	0.617886179	NA	NA		1
847	0.620596206	NA	NA		1
321	0.570093458	NA	NA		1
1603	0.351491569	NA	NA		1
855	0.617886179	NA	NA		1
4450	0.547560375	275		933	1
843	0.620218579	NA	NA		1
4948	0.509695291	314		1000	1
1131	0.534632035	NA	NA		1
267	0.498127341	NA	NA		1
1961	0.463264162	NA	NA		1
1831	0.476500362	NA	NA		1
826	0.487252125	NA	NA		1
1368	0.382051282	NA	NA		1
23512	0.533929647	NA	NA		0
19451	0.517051773	1000		1000	1
726	0.416260163	NA	NA		1
6587	0.497096849	1000		1000	0
1524	0.519911504	NA	NA		1
7926	0.566422913	909		614	0
814	0.430249633	NA	NA		1
474	0.426160338	NA	NA		1
619	0.51214128	NA	NA		1
4306	0.524628014	NA	NA		1
668	0.43115942	NA	NA		1
704	0.430359937	NA	NA		1
639	0.43707483	NA	NA		1
697	0.447674419	NA	NA		1
589	0.389199255	NA	NA		1
552	0.392033543	NA	NA		1
583	0.367424242	NA	NA		1
698	0.378708551	NA	NA		1
700	0.359698682	NA	NA		1
708	0.368836292	NA	NA		1
655	0.373626374	NA	NA		1
17574	0.521327014	NA	NA		1
1999	0.498749375	NA	NA		1
1999	0.498749375	NA	NA		1
3673	0.528916339	NA	NA		1
703	0.353293413	NA	NA		1
707	0.340909091	NA	NA		1
584	0.373165618	NA	NA		1
705	0.378167641	NA	NA		1
12012	0.527540319	NA	NA		0
10121	0.473519469	NA	NA		1
10121	0.506952751	NA	NA		0
634	0.39122807	NA	NA		1
613	0.398148148	NA	NA		1
653	0.401098901	NA	NA		1
2667	0.547404547	664		1000	1
1339	0.522664564	NA	NA		0
1339	0.522664564	NA	NA		0
730	0.348066298	NA	NA		1
609	0.4106814	NA	NA		1

2820	0.532258065	NA	NA	1
613	0.369175627	NA	NA	1
652	0.426443203	NA	NA	1
647	0.389199255	NA	NA	1
715	0.372007366	NA	NA	1
610	0.370577281	NA	NA	1
653	0.447971781	NA	NA	1
562	0.525793651	NA	NA	1
593	0.475925926	NA	NA	1
700	0.371868979	NA	NA	1
798	0.397979798	NA	NA	1
668	0.413043478	NA	NA	1
781	0.349206349	NA	NA	1
652	0.337037037	NA	NA	1
652	0.4	NA	NA	1
258	0.403100775	NA	NA	1
726	0.366852886	NA	NA	1
590	0.438202247	NA	NA	1
898	0.442093541	NA	NA	1
468	0.536324786	NA	NA	1
898	0.444320713	NA	NA	1
468	0.536324786	NA	NA	1
898	0.444320713	NA	NA	1
569	0.451669596	NA	NA	1
468	0.536324786	NA	NA	1
898	0.444320713	NA	NA	1
468	0.536324786	NA	NA	1
898	0.443207127	NA	NA	1
569	0.451669596	NA	NA	1
468	0.536324786	NA	NA	1
898	0.444320713	NA	NA	1
569	0.451669596	NA	NA	1
468	0.534188034	NA	NA	1
898	0.444320713	NA	NA	1
569	0.451669596	NA	NA	1
468	0.534188034	NA	NA	1
898	0.444320713	NA	NA	1
569	0.451669596	NA	NA	1
468	0.534188034	NA	NA	1
898	0.444320713	NA	NA	1
569	0.451669596	NA	NA	1
468	0.534188034	NA	NA	1
898	0.444320713	NA	NA	1
569	0.451669596	NA	NA	1
468	0.534188034	NA	NA	1
898	0.444320713	NA	NA	1
569	0.451669596	NA	NA	1
468	0.536324786	NA	NA	1
898	0.443207127	NA	NA	1
569	0.451669596	NA	NA	1
468	0.536324786	NA	NA	1
898	0.443207127	NA	NA	1
569	0.451669596	NA	NA	1
468	0.536324786	NA	NA	1
898	0.443207127	NA	NA	1
569	0.451669596	NA	NA	1
468	0.536324786	NA	NA	1

898	0.443207127	NA	NA	1
569	0.451669596	NA	NA	1
468	0.536324786	NA	NA	1
898	0.443207127	NA	NA	1
569	0.451669596	NA	NA	1
468	0.536324786	NA	NA	1
898	0.443207127	NA	NA	1
15352	0.56463379	395	1000	1
569	0.451669596	NA	NA	1
468	0.536324786	NA	NA	1
898	0.443207127	NA	NA	1
569	0.453427065	NA	NA	1
468	0.536324786	NA	NA	1
898	0.444320713	NA	NA	1
569	0.453427065	NA	NA	1
468	0.536324786	NA	NA	1
898	0.444320713	NA	NA	1
569	0.453427065	NA	NA	1
1546	0.485148515	NA	NA	1
468	0.536324786	NA	NA	1
898	0.444320713	NA	NA	1
569	0.451669596	NA	NA	1
468	0.536324786	NA	NA	1
898	0.443207127	NA	NA	1
569	0.451669596	NA	NA	1
468	0.536324786	NA	NA	1
555	0.475675676	NA	NA	1
526	0.486692015	NA	NA	1
555	0.475675676	NA	NA	1
528	0.484848485	NA	NA	1
555	0.472072072	NA	NA	1
528	0.484848485	NA	NA	1
555	0.472072072	NA	NA	1
528	0.484848485	NA	NA	1
555	0.472072072	NA	NA	1
526	0.486692015	NA	NA	1
555	0.475675676	NA	NA	1
526	0.486692015	NA	NA	1
555	0.475675676	NA	NA	1
526	0.486692015	NA	NA	1
555	0.475675676	NA	NA	1
526	0.482889734	NA	NA	1
555	0.475675676	NA	NA	1
526	0.484790875	NA	NA	1
555	0.475675676	NA	NA	1
526	0.484790875	NA	NA	1
555	0.475675676	NA	NA	1
526	0.482889734	NA	NA	1
6860	0.483774338	NA	NA	1
555	0.472072072	NA	NA	1
524	0.480916031	NA	NA	1
555	0.472072072	NA	NA	1
524	0.480916031	NA	NA	1
555	0.472072072	NA	NA	1
524	0.480916031	NA	NA	1
555	0.472072072	NA	NA	1
524	0.480916031	NA	NA	1

555	0.472072072	NA	NA		1
524	0.480916031	NA	NA		1
555	0.472072072	NA	NA		1
528	0.484848485	NA	NA		1
555	0.472072072	NA	NA		1
528	0.484848485	NA	NA		1
555	0.472072072	NA	NA		1
526	0.482889734	NA	NA		1
555	0.472072072	NA	NA		1
530	0.501886792	NA	NA		1
415	0.474698795	NA	NA		1
574	0.479094077	NA	NA		1
585	0.47008547	NA	NA		1
710	0.34939759	NA	NA		1
746	0.4375	NA	NA		1
715	0.388581952	NA	NA		1
729	0.368715084	NA	NA		1
731	0.384094755	NA	NA		1
662	0.397372742	NA	NA		1
658	0.387978142	NA	NA		1
710	0.377532228	NA	NA		1
1315	0.54589372	NA	NA		1
1315	0.54589372	NA	NA		1
2198	0.492205638	NA	NA		1
4026	0.550886408		124	702	0
2868	0.49532294	NA	NA		1
1062	0.511530398	NA	NA		1
25727	0.534665579		946	940	1
11557	0.512812691		654	899	1
43632	0.490576497	NA	NA		0
1821	0.514003295	NA	NA		1
6587	0.565080613	NA	NA		0
31257	0.572943172		774	917	1
7949	0.499694049	NA	NA		1
37709	0.573846805		40	1000	1
205	0.503546099	NA	NA		1
1090	0.476146789	NA	NA		1
7222	0.544625149		257	1000	1
431	0.402203857	NA	NA		1
639	0.445614035	NA	NA		1
1772	0.441605839	NA	NA		1
2068	0.388888889	NA	NA		1
645	0.522108844	NA	NA		1
1923	0.427466151	NA	NA		1
1890	0.482478098	NA	NA		1
2609	0.496483244		873	935	1
8230	0.465309633		543	1000	1
13708	0.524324324		448	1000	0
2852	0.55244123	NA	NA		1
1557	0.526845638	NA	NA		1
3407	0.533032773		942	917	1
14770	0.506556763		555	1000	1
4151	0.476374689	NA	NA		1
10796	0.432457269		198	1000	0
2332	0.471193416	NA	NA		1
1320	0.46830093	NA	NA		1
792	0.546463245	NA	NA		1

12346	0.529116722	NA	NA	1
1077	0.508368201	NA	NA	1
1017	0.394296952	NA	NA	1
5215	0.535409035	NA	NA	1
10791	0.555706062	NA	NA	1
894	0.531319911	NA	NA	1
2724	0.493757094	NA	NA	1
655	0.427480916	NA	NA	1
719	0.564687976	NA	NA	1
1366	0.490229397	NA	NA	1
3348	0.50437357	NA	NA	0
1340	0.565671642	NA	NA	1
3348	0.491583313	NA	NA	0
5842	0.475026196	NA	NA	1
1522	0.549277267	NA	NA	1
5662	0.516996047	NA	NA	1
883	0.524758454	NA	NA	0
1280	0.503597122	NA	NA	1
5992	0.576821773	NA	NA	1
3840	0.464152226	825	1000	1
2836	0.508098103	775	681	1
1701	0.519268452	NA	NA	1
1443	0.516285516	NA	NA	1
5544	0.538557837	477	945	1
11629	0.558893281	NA	NA	1
3912	0.523222749	NA	NA	1
18037	0.427070707	202	1000	1
2999	0.495440729	NA	NA	1
1826	0.54381161	NA	NA	1
1931	0.544277576	NA	NA	1
1032	0.507676561	NA	NA	1
1017	0.464961068	NA	NA	1
1309	0.557226399	NA	NA	1
1060	0.559433962	NA	NA	1
2321	0.524703088	1000	927	0
10428	0.468213925	NA	NA	1
4518	0.545057346	861	879	1
993	0.475327291	NA	NA	1
1343	0.520784314	NA	NA	1
750	0.508	NA	NA	1
2765	0.538951494	NA	NA	1
840	0.523809524	NA	NA	1
2203	0.531988473	NA	NA	1
1858	0.527586207	NA	NA	1
7995	0.484739677	NA	NA	1
4589	0.541550744	935	869	1
786	0.632315522	NA	NA	1
3307	0.494122257	930	435	1
9483	0.513782089	NA	NA	1
2986	0.569380909	547	940	0
894	0.482398957	NA	NA	1
5817	0.450960931	NA	NA	0
71719	0.518619042	191	532	1
3349	0.525243954	NA	NA	1
4186	0.486549405	1000	1000	1
1406	0.491179201	NA	NA	1
710	0.488514548	NA	NA	1

2593	0.522289488	NA	NA		1
2759	0.529981378	NA	NA		1
832	0.567307692	NA	NA		1
2448	0.512030393	921		1000	1
3307	0.551338394	NA	NA		1
1552	0.460051546	NA	NA		1
8882	0.543784239	691		1000	0
1680	0.517923362	NA	NA		1
1154	0.463040446	NA	NA		1
1769	0.506972112	NA	NA		1
6558	0.519394163	1000		1000	1
3431	0.562532982	NA	NA		1
8930	0.536683283	178		702	0
24494	0.541522491	451		577	1
7282	0.490285506	544		1000	1
1359	0.529363785	NA	NA		1
2366	0.475598935	943		1000	1
4864	0.52937135	1000		693	0
2774	0.480216576	942		813	1
4737	0.456329735	NA	NA		1
2287	0.474420638	1000		1000	1
3252	0.538461538	NA	NA		1
1088	0.506122449	NA	NA		1
70502	0.491508689	NA	NA		0
25781	0.488645024	NA	NA		0
3034	0.474918802	933		1000	1
1499	0.521105142	NA	NA		1
5716	0.475392392	NA	NA		1
3583	0.569130838	781		929	1
5797	0.519248826	885		907	1
6048	0.49833795	470		1000	1
2756	0.503551697	NA	NA		1
1678	0.480840544	NA	NA		1
3241	0.507778881	NA	NA		1
3452	0.562698133	242		934	0
3626	0.478421702	922		938	1
4681	0.474051708	NA	NA		0
785	0.426751592	NA	NA		1
7244	0.532525095	68		932	0
2138	0.519176801	1000		1000	1
8469	0.5460652	NA	NA		1
1784	0.567064083	NA	NA		1
2527	0.50106792	814		1000	1
1713	0.506713368	NA	NA		1
2913	0.471455756	656		810	1
10565	0.515244526	326		1000	0
4979	0.508945211	600		931	1
3883	0.508879363	NA	NA		0
14680	0.522447622	942		1000	1
2065	0.485198194	NA	NA		1
2198	0.489955904	NA	NA		1
907	0.445768772	NA	NA		1
25489	0.515324828	NA	NA		0
5171	0.539190898	NA	NA		1
6755	0.480917935	400		937	0
939	0.547169811	NA	NA		1
2359	0.469237563	NA	NA		0

3335	0.547477745	NA	NA		1
864	0.562189055	NA	NA		1
5484	0.476961635	150		1000	0
1129	0.496075353	NA	NA		1
5541	0.500727802	723		860	1
2398	0.555243446	840		1000	0
1666	0.485527544	NA	NA		1
4573	0.504445607	892		1000	1
1184	0.560214095	NA	NA		1
840	0.500754148	NA	NA		1
9324	0.561043285	NA	NA		1
6875	0.480796586	NA	NA		1
5754	0.532451679	NA	NA		0
19430	0.485484591	NA	NA		0
1143	0.53493014	NA	NA		1
1677	0.523553965	NA	NA		1
4827	0.539771907	271		1000	1
978	0.538636364	NA	NA		1
13082	0.490585009	NA	NA		0
9825	0.526243568	NA	NA		0
12808	0.451797386	NA	NA		1
14821	0.504778157	742		1000	0
12485	0.500342936	1000		1000	1
1634	0.514089347	NA	NA		1
1743	0.511771995	NA	NA		1
1708	0.533967391	NA	NA		1
1738	0.468215995	NA	NA		1
6203	0.537170534	NA	NA		0
873	0.342629482	NA	NA		1
133933	0.423938224	493		287	1
5320	0.40632806	592		1000	0
1433	0.450404115	NA	NA		1
5302	0.422923828	445		1000	1
6558	0.495105717	137		731	1
671	0.345724907	NA	NA		1
56446	0.55952473	NA	NA		0
1474	0.513888889	NA	NA		1
3927	0.510239828	150		1000	0
11438	0.452547194	404		1000	1
1080	0.325925926	NA	NA		1
2620	0.530603448	885		862	1
2850	0.484562951	651		639	0
1362	0.524201854	NA	NA		1
2784	0.537356322	1000		1000	1
946	0.5018226	NA	NA		1
717	0.31411863	NA	NA		0
49505	0.348583878	NA	NA		1
33103	0.402976692	771		199	1
146532	0.34870414	127		322	1
5594	0.367346939	787		1000	0
6365	0.508532423	266		951	1
4763	0.505280141	159		1000	0
6914	0.527327177	555		1000	0
7040	0.543600057	89		632	0
1201	0.561946903	NA	NA		1
3862	0.556085919	786		936	0
2866	0.543731491	NA	NA		1

1767	0.421618563	NA	NA	1
20342	0.522874192	NA	NA	1
1102	0.499092559	NA	NA	1
4034	0.524198427	1000	781	1
714	0.56501182	NA	NA	1
715	0.512461059	NA	NA	1
2255	0.470509978	1000	1000	1
776	0.54199683	NA	NA	1
6429	0.508263812	1000	924	1
3456	0.4432656	NA	NA	0
3389	0.55637111	NA	NA	0
1025	0.521052632	NA	NA	1
1083	0.533592989	NA	NA	1
2282	0.473917323	809	941	1
4468	0.553474114	NA	NA	1
917	0.502364066	NA	NA	1
20963	0.389801783	259	927	0
1021	0.438998957	NA	NA	1
3243	0.510810811	NA	NA	1
795	0.554716981	NA	NA	1
4460	0.546017223	617	895	1
1795	0.503708282	NA	NA	1
21626	0.475118217	432	1000	1
632	0.379069767	NA	NA	1
94547	0.341535263	107	949	0
13461	0.353754941	NA	NA	1
17961	0.607777405	818	1000	1
5737	0.491226058	168	937	1
1258	0.46277205	NA	NA	1
939	0.420660277	NA	NA	1
1955	0.499214248	NA	NA	0
7500	0.597170345	295	732	0
2429	0.384946237	NA	NA	1
5484	0.495290234	493	1000	0
1843	0.497287523	NA	NA	1
420	0.464285714	NA	NA	1
744	0.576612903	NA	NA	1
3653	0.517777778	1000	1000	1
715	0.510903427	NA	NA	1
2293	0.493052443	938	1000	1
1889	0.464796188	NA	NA	1
5909	0.457446809	315	1000	1
5379	0.54790692	460	1000	0
6119	0.522856014	NA	NA	0
17784	0.376489138	NA	NA	0
2674	0.546815042	932	1000	1
2565	0.477909941	NA	NA	1
2372	0.502624672	NA	NA	1
1010	0.5	NA	NA	1
750	0.468	NA	NA	1
1970	0.547268908	NA	NA	1
6179	0.450694322	1000	1000	1
1097	0.490296221	NA	NA	1
10015	0.429131403	522	234	1
1915	0.494150418	NA	NA	0
1463	0.551606288	NA	NA	1
1669	0.553644183	NA	NA	1

1556	0.487603306	NA	NA		1
11980	0.560012634	NA	NA		1
3605	0.504350736		569	1000	1
1569	0.439509954	NA	NA		1
2678	0.532155797		935	904	1
3389	0.473644999		407	1000	0
6572	0.503518374		412	1000	0
2682	0.386413763	NA	NA		1
7808	0.518136087		941	936	1
2389	0.447470817	NA	NA		1
16630	0.460739575		232	863	0
1237	0.529506871	NA	NA		1
997	0.444919786	NA	NA		1
4780	0.501643905	NA	NA		0
2169	0.500741473	NA	NA		1
6504	0.562662448	NA	NA		0
1061	0.464655985	NA	NA		1
5058	0.529101923		111	789	0
5614	0.499624906		377	1000	1
5166	0.530373142	NA	NA		0
362	0.448504983	NA	NA		1
16556	0.520645844		152	807	1
8733	0.478583196	NA	NA		1
851	0.3960047	NA	NA		1
4933	0.586461538	NA	NA		1
2631	0.496521189	NA	NA		1
1885	0.454959692	NA	NA		1
4044	0.523468169	NA	NA		0
3028	0.560444874		434	1000	1
1062	0.496953873	NA	NA		0
939	0.535676251	NA	NA		1
1696	0.352004717	NA	NA		1
11227	0.52768883		1000	1000	0
2785	0.531418312		1000	1000	1
11077	0.510452418	NA	NA		1
22794	0.533880126		117	548	0
877	0.458333333	NA	NA		1
1194	0.538525963	NA	NA		1
26935	0.502994012		977	1000	1
3206	0.551774607	NA	NA		0
1615	0.551114527	NA	NA		1
2040	0.515737162	NA	NA		1
2150	0.47627907		1000	1000	1
1744	0.502962475	NA	NA		1
3935	0.504280822		588	978	1
1540	0.475884244	NA	NA		1
2830	0.504597278		944	1000	1
443	0.421875	NA	NA		1
1742	0.448851775	NA	NA		1
4069	0.517397356		598	943	1
882	0.489795918	NA	NA		1
78809	0.485222596	NA	NA		1
1139	0.528601695	NA	NA		1
3386	0.471980075		416	804	1
1114	0.425228891	NA	NA		1
1615	0.417533432	NA	NA		1
2608	0.412790698	NA	NA		1

1499	0.449633089	NA	NA	1
2800	0.501872659	NA	NA	1
3158	0.49894958	698	1000	1
818	0.443965517	NA	NA	1
2608	0.45308311	NA	NA	1
9352	0.456367636	321	938	1
1206	0.504340278	NA	NA	1
1370	0.502134927	NA	NA	1
8793	0.481252757	1000	1000	1
3268	0.507796258	NA	NA	1
13323	0.463888889	NA	NA	1
4039	0.576512456	742	885	1
4864	0.498185878	NA	NA	1
5953	0.554402516	479	1000	1
1929	0.505280712	NA	NA	1
1961	0.504696306	NA	NA	1
49631	0.44522999	530	1000	1
3179	0.537715517	NA	NA	1
8693	0.493859545	935	939	1
1956	0.570538244	NA	NA	1
2808	0.483833718	907	883	1
1284	0.505451713	NA	NA	1
40609	0.496620194	NA	NA	0
4574	0.506167763	NA	NA	1
6455	0.489795918	380	1000	1
5056	0.485635486	NA	NA	1
1913	0.456989247	NA	NA	1
2016	0.578342408	NA	NA	1
1498	0.548113934	NA	NA	1
807	0.531270573	NA	NA	0
5529	0.524821412	482	943	0
567	0.430335097	NA	NA	1
15811	0.477880184	510	924	0
932	0.565819861	NA	NA	1
19867	0.473605948	NA	NA	1
3277	0.510143071	811	1000	0
8553	0.422618101	838	1000	0
2082	0.532180596	1000	1000	1
1875	0.550970874	NA	NA	1
1949	0.528135991	NA	NA	1
4021	0.485124555	NA	NA	0
3594	0.531514581	NA	NA	1
3750	0.52850062	NA	NA	1
14457	0.504854369	NA	NA	0
1521	0.508966043	NA	NA	0
4887	0.528205128	877	942	1
1963	0.518518519	NA	NA	1
736	0.490398818	NA	NA	1
739	0.525925926	NA	NA	1
5813	0.478963111	716	1000	0
2657	0.557221784	NA	NA	1
1962	0.534726143	NA	NA	1
1416	0.5	NA	NA	1
1714	0.508168028	NA	NA	1
1122	0.52742616	NA	NA	0
21709	0.482395426	NA	NA	1
33234	0.450426789	334	1000	0

1565	0.540425532	NA	NA	1
1121	0.462910798	NA	NA	1
2425	0.536541889	NA	NA	1
14302	0.472308799	367	1000	0
2816	0.442951492	1000	535	0
2492	0.480728505	1000	869	1
1791	0.484645449	NA	NA	1
864	0.506995336	NA	NA	1
3034	0.524220624	289	1000	1
10899	0.524230418	NA	NA	1
4193	0.55550074	208	867	1
5557	0.481348788	NA	NA	1
1859	0.456577267	NA	NA	1
1622	0.541307028	NA	NA	1
5263	0.528182005	887	1000	1
4668	0.546777025	802	942	1
2601	0.513319672	NA	NA	0
1945	0.465448315	NA	NA	1
1354	0.457446809	NA	NA	1
23396	0.454305335	795	1000	1
1512	0.556521739	NA	NA	1
982	0.488798371	NA	NA	1
3294	0.497198356	804	1000	1
2137	0.497818711	NA	NA	0
1543	0.578486056	NA	NA	1
6261	0.583406114	NA	NA	1
440	0.412987013	NA	NA	1
1436	0.539693593	NA	NA	1
1963	0.550131234	NA	NA	1
12447	0.413624221	NA	NA	1
7026	0.529034141	1000	937	1
3298	0.531227821	553	1000	1
6146	0.538480513	276	1000	1
5255	0.485337824	NA	NA	1
1592	0.479152427	NA	NA	1
2635	0.538831065	923	940	1
1078	0.551020408	NA	NA	1
3005	0.517039922	NA	NA	1
2201	0.52311828	NA	NA	1
4783	0.524622829	637	877	1
31694	0.441364606	716	1000	1
2337	0.548420584	NA	NA	0
4036	0.515037594	903	299	1
1616	0.467060811	NA	NA	1
1513	0.467046379	NA	NA	1
712	0.515449438	NA	NA	1
2134	0.571118686	NA	NA	1
1429	0.511041009	NA	NA	1
4485	0.492875728	881	825	0
1615	0.530812325	NA	NA	1
9095	0.47546288	547	1000	0
936	0.507478632	NA	NA	1
905	0.447513812	NA	NA	1
19430	0.496953518	392	945	1
3284	0.528295376	803	940	1
1785	0.479842675	NA	NA	1
3585	0.500474233	765	880	1

3714	0.54562618		464	1000	1
3602	0.483323226	NA	NA		1
1684	0.559972106	NA	NA		1
3154	0.489633174	NA	NA		1
2056	0.490223464	NA	NA		1
24214	0.494705493		1000	908	0
1441	0.500728863	NA	NA		1
5103	0.501187648	NA	NA		1
1361	0.49137148	NA	NA		1
1533	0.528375734	NA	NA		1
11171	0.47934224	NA	NA		1
3567	0.526139818		723	1000	1
1855	0.520471116	NA	NA		1
1038	0.410404624	NA	NA		1
832	0.516254876	NA	NA		1
5221	0.511543536		816	1000	1
2460	0.429473684	NA	NA		1
1148	0.471287129	NA	NA		1
4868	0.53217012		907	878	1
1217	0.50397878	NA	NA		1
11778	0.347187025		528	1000	0
4083	0.466590844		792	933	1
19567	0.429168533	NA	NA		0
2917	0.509282489	NA	NA		1
3101	0.491557223	NA	NA		1
1234	0.496103896	NA	NA		1
3628	0.499210111		457	813	1
1610	0.541666667	NA	NA		1
5130	0.40535733		683	1000	0
2568	0.473127753		930	772	1
5440	0.512010114	NA	NA		1
2431	0.479121825	NA	NA		1
1672	0.485868102	NA	NA		1
1229	0.544723618	NA	NA		1
2016	0.49539102	NA	NA		1
1156	0.458523245	NA	NA		1
1118	0.433962264	NA	NA		1
3845	0.427362894		853	1000	1
4837	0.40588596		465	940	0
2189	0.518117647		936	1000	1
2363	0.524076671	NA	NA		1
2169	0.52581864	NA	NA		1
2522	0.531324346		1000	1000	1
2516	0.52996331		1000	1000	1
51646	0.529801325	NA	NA		1
514	0.552529183	NA	NA		1
3593	0.589535741		461	930	0
2314	0.479043775	NA	NA		1
4381	0.491503931		914	939	1
2748	0.396382226	NA	NA		1
8416	0.514579537		802	1000	1
2054	0.530819868	NA	NA		1
4709	0.524780427		450	1000	1
1750	0.398739874	NA	NA		1
861	0.461245235	NA	NA		1
1774	0.536076663	NA	NA		1
17896	0.413243714		846	1000	1

6627	0.499319574	452		897	0
10501	0.474774775	NA	NA		0
3668	0.517693316	NA	NA		1
1294	0.499227202	NA	NA		1
1728	0.539158576	NA	NA		1
1807	0.531267294	NA	NA		1
612	0.385585586	NA	NA		1
1915	0.547619048	NA	NA		1
2542	0.538214858	NA	NA		1
1571	0.542966264	NA	NA		1
9119	0.487127059	134		1000	0
2186	0.554071247	NA	NA		1
1661	0.510869565	NA	NA		1
971	0.486096807	NA	NA		1
12055	0.532483778	NA	NA		0
1542	0.535278155	NA	NA		1
1656	0.435516543	NA	NA		1
3337	0.535673188	719		946	0
1327	0.433083957	NA	NA		1
16407	0.47460074	466		1000	0
1694	0.410015649	NA	NA		1
2627	0.510575296	794		943	1
7169	0.521227101	946		939	1
4155	0.47934115	830		944	1
2058	0.529715762	NA	NA		1
1025	0.501463415	NA	NA		1
2581	0.534022863	NA	NA		1
4001	0.481423339	NA	NA		1
1067	0.507028112	NA	NA		1
1235	0.539473684	NA	NA		1
1300	0.463149079	NA	NA		1
17967	0.547346752	499		935	1
800	0.477027027	NA	NA		1
3464	0.51259916	542		876	0
2028	0.463510848	1000		1000	1
1233	0.519083969	NA	NA		1
2117	0.478476821	NA	NA		1
978	0.477173913	NA	NA		1
1252	0.484444444	NA	NA		1
3711	0.525790349	NA	NA		1
1471	0.510638298	NA	NA		1
1161	0.504559271	NA	NA		1
4254	0.504481434	NA	NA		1
6684	0.507628866	382		939	1
5972	0.542231813	NA	NA		0
2331	0.480112307	NA	NA		1
760	0.476315789	NA	NA		1
1426	0.483639266	NA	NA		1
2997	0.486245641	NA	NA		1
3003	0.478661659	NA	NA		1
2478	0.509738914	1000		1000	1
8580	0.525987526	NA	NA		1
1210	0.453719008	NA	NA		1
2636	0.551972686	NA	NA		1
4408	0.508835759	NA	NA		1
2453	0.550295858	NA	NA		1
1525	0.438596491	NA	NA		1

4542	0.516878669		877	874	1
2242	0.508542141	NA	NA		1
2969	0.48630137		659	1000	1
3388	0.538128538		902	884	1
2347	0.539030612	NA	NA		1
925	0.484029484	NA	NA		1
2081	0.480708412	NA	NA		1
901	0.416772554	NA	NA		1
925	0.576470588	NA	NA		1
2613	0.478379858	NA	NA		1
2532	0.493248045	NA	NA		1
12791	0.481577704	NA	NA		1
6266	0.514349043		821	1000	0
1354	0.498459168	NA	NA		1
2168	0.537356322		933	1000	1
1295	0.478168264	NA	NA		1
2887	0.409024745	NA	NA		1
22209	0.527406028		653	1000	1
3740	0.470288006		534	1000	1
5321	0.527625474		1000	1000	1
5064	0.499174781	NA	NA		0
1843	0.48932487	NA	NA		1
744	0.475982533	NA	NA		1
2228	0.522890485	NA	NA		1
1807	0.510237963	NA	NA		1
7668	0.483646113	NA	NA		1
3003	0.479955917	NA	NA		1
8482	0.490552752		118	727	0
1433	0.516154452	NA	NA		1
6973	0.497056182		1000	1000	1
3359	0.51476207		832	1000	1
1109	0.580426357	NA	NA		1
1347	0.511827957	NA	NA		1
1086	0.598526703	NA	NA		1
907	0.494871795	NA	NA		1
1960	0.52154047	NA	NA		1
9582	0.515700874		1000	1000	1
4593	0.449953445	NA	NA		1
43361	0.465238632	NA	NA		1
1170	0.533333333	NA	NA		1
3246	0.557474366	NA	NA		1
1978	0.486813187	NA	NA		1
2130	0.584815321	NA	NA		1
29264	0.518987342	NA	NA		1
1889	0.495964784	NA	NA		1
1220	0.47117567	NA	NA		0
3686	0.544267877	NA	NA		1
3503	0.552531864	NA	NA		1
8786	0.454690137		192	1000	0
5779	0.510196515		1000	1000	1
1394	0.468373494	NA	NA		1
1311	0.409547739	NA	NA		1
7393	0.470704175	NA	NA		0
2140	0.544392523	NA	NA		1
2695	0.530773574	NA	NA		1
7081	0.445315416	NA	NA		0
653	0.568027211	NA	NA		1

6354	0.512939002	696	1000	1
2089	0.511565358	NA	NA	1
1667	0.566886623	NA	NA	1
2008	0.484120837	NA	NA	1
7168	0.516949153	606	1000	1
1623	0.57000993	NA	NA	1
4882	0.487063389	NA	NA	1
1442	0.564493759	NA	NA	1
5086	0.53407359	877	942	1
16969	0.483306836	504	553	0
1621	0.552429668	NA	NA	1
2637	0.430727763	NA	NA	1
1804	0.549510337	NA	NA	1
2495	0.504833512	NA	NA	1
3972	0.411581892	420	861	0
10336	0.494966443	69	935	1
4800	0.470505864	877	323	0
2737	0.477272727	802	818	1
588	0.419659735	NA	NA	1
55704	0.545978668	NA	NA	1
5144	0.551522533	NA	NA	0
781	0.467349552	NA	NA	1
1193	0.512866016	NA	NA	1
1334	0.420327304	NA	NA	1
11282	0.443753111	464	1000	1
7166	0.561352856	NA	NA	1
2486	0.517523364	NA	NA	1
1928	0.523015873	NA	NA	1
1021	0.510284035	NA	NA	1
2695	0.502962625	699	943	0
1464	0.525273224	NA	NA	1
1157	0.493517718	NA	NA	1
2592	0.510089686	NA	NA	1
3389	0.503336511	932	881	1
983	0.554425229	NA	NA	1
2700	0.445647969	942	1000	1
5076	0.488970588	NA	NA	1
4446	0.465586146	438	1000	0
5110	0.51416122	300	943	1
4811	0.455022981	1000	938	1
3930	0.370624723	407	1000	1
7259	0.573545356	NA	NA	1
6189	0.509273335	390	784	1
1418	0.526861451	NA	NA	1
1414	0.520509194	NA	NA	1
2145	0.382572227	NA	NA	1
4424	0.509660227	679	1000	1
2155	0.5225058	1000	1000	1
4892	0.4875	699	1000	1
4200	0.512057736	170	1000	0
5689	0.531955607	NA	NA	1
2442	0.51424568	943	817	1
2792	0.591002045	NA	NA	1
15155	0.418010753	NA	NA	1
2900	0.53525819	NA	NA	1
1499	0.436290861	NA	NA	1
7337	0.505203687	NA	NA	1

980	0.466975666	NA	NA		1
2385	0.516880093	NA	NA		1
1880	0.528562461	NA	NA		1
1015	0.464882943	NA	NA		1
4749	0.452912199	NA	NA		0
841	0.44459279	NA	NA		1
2998	0.49982681	921		1000	1
4400	0.471425239	NA	NA		1
1003	0.500498504	NA	NA		1
529	0.496277916	NA	NA		1
2723	0.53305629	924		1000	1
8364	0.463441654	NA	NA		1
1287	0.432012432	NA	NA		1
30022	0.514302059	NA	NA		1
1796	0.546353523	NA	NA		1
2543	0.524970507	1000		1000	1
2570	0.483864074	860		938	1
1076	0.517751479	NA	NA		1
11767	0.486884573	NA	NA		0
2892	0.515813253	939		1000	1
1282	0.494438827	NA	NA		1
12083	0.38743308	563		1000	1
2591	0.512157468	1000		1000	1
4729	0.570197487	828		1000	1
5261	0.537009439	NA	NA		1
1542	0.538860104	NA	NA		1
13073	0.515820456	NA	NA		0
6674	0.5125981	NA	NA		1
1143	0.421338156	NA	NA		0
3644	0.517589477	NA	NA		1
1490	0.442754919	NA	NA		1
1188	0.515993266	NA	NA		1
2804	0.482758621	NA	NA		1
1297	0.393313668	NA	NA		1
11817	0.555154915	NA	NA		1
2111	0.446727961	NA	NA		1
20722	0.488114621	NA	NA		1
5809	0.544965577	376		928	1
3771	0.45578575	861		1000	0
1199	0.474381625	NA	NA		1
2352	0.498940678	NA	NA		1
8017	0.46746645	112		994	0
48297	0.431757833	NA	NA		1
2790	0.485763589	831		879	1
3020	0.497282609	924		1000	1
1181	0.533989267	NA	NA		1
1405	0.50745098	NA	NA		1
12204	0.553764236	310		946	0
862	0.454756381	NA	NA		1
17831	0.551992754	129		1000	1
2375	0.47295423	848		959	1
5353	0.53633218	NA	NA		0
7049	0.500818574	847		942	1
4136	0.492849059	NA	NA		0
12788	0.533924674	NA	NA		0
1631	0.446486486	NA	NA		1
3496	0.497385241	NA	NA		1

7044	0.533801498	NA	NA		0
2952	0.528533802	NA	NA		1
1670	0.524983777	NA	NA		1
3833	0.482321291	546		1000	1
2050	0.529289787	NA	NA		1
959	0.625	NA	NA		1
2781	0.524405002	897		876	1
2804	0.522835719	NA	NA		0
12436	0.482459522	NA	NA		1
1756	0.513577332	NA	NA		1
683	0.454303461	NA	NA		0
4846	0.435163917	943		1000	1
1347	0.507011866	NA	NA		1
3404	0.529983793	949		1000	1
5007	0.556656347	NA	NA		1
7043	0.494934877	NA	NA		0
7616	0.383973695	NA	NA		0
4984	0.502405002	463		941	1
736	0.467571644	NA	NA		1
12785	0.486645606	NA	NA		1
4515	0.493094629	829		839	1
1446	0.526970954	NA	NA		1
2655	0.455965909	NA	NA		1
3782	0.480290853	985		786	1
3091	0.476289373	932		935	1
10660	0.494038362	NA	NA		1
1602	0.454268293	NA	NA		1
3849	0.470325203	NA	NA		0
1655	0.484316186	NA	NA		1
3080	0.561468866	NA	NA		1
4242	0.500471476	NA	NA		1
2772	0.51433121	940		863	1
28994	0.450219912	210		533	0
7907	0.394195445	425		1000	1
5212	0.441837732	NA	NA		1
4264	0.515276821	167		883	0
5079	0.532767562	290		1000	1
21899	0.532420749	51		1000	1
1545	0.498381877	NA	NA		1
3488	0.563492063	NA	NA		1
1743	0.53960396	NA	NA		1
1357	0.502594034	NA	NA		0
2900	0.502475248	310		1000	1
6866	0.479144385	1000		1000	1
3207	0.470279146	NA	NA		1
1428	0.467987805	NA	NA		1
2413	0.589730808	718		875	1
3851	0.502179837	NA	NA		1
4352	0.510752688	NA	NA		1
3209	0.498313975	NA	NA		1
2915	0.5182534	942		937	1
1429	0.485116653	NA	NA		1
2507	0.516450216	NA	NA		1
4820	0.485730594	515		942	1
2104	0.425380228	NA	NA		1
25739	0.506773692	NA	NA		0
5349	0.475370636	NA	NA		1

7874	0.522838499	393	1000	1
3649	0.567764408	1000	769	1
5125	0.472389284	217	1000	1
1559	0.517584098	NA	NA	1
4903	0.560799814	601	1000	1
4679	0.486461467	NA	NA	1
4854	0.486815416	423	1000	1
12970	0.429197599	444	1000	0
2962	0.522008679	661	867	1
1448	0.570915619	NA	NA	1
302	0.421686747	NA	NA	1
1229	0.44573991	NA	NA	1
1188	0.537037037	NA	NA	1
1716	0.454545455	NA	NA	1
11501	0.473829201	233	1000	1
1140	0.552336449	NA	NA	1
1845	0.528026906	NA	NA	1
1144	0.553604436	NA	NA	1
3514	0.557586837	824	1000	1
1147	0.564738292	NA	NA	1
4211	0.532447224	NA	NA	1
733	0.575716235	NA	NA	1
5276	0.53648863	1000	1000	0
3334	0.503526093	134	936	1
14391	0.469244	868	1000	0
925	0.487210719	NA	NA	1
18706	0.497329818	382	924	1
1508	0.432619212	NA	NA	1
10434	0.574468085	NA	NA	1
811	0.478645066	NA	NA	1
6540	0.415484923	521	1000	0
2534	0.531570639	1000	1000	1
5049	0.556628834	284	947	1
1235	0.547085202	NA	NA	1
984	0.53220339	NA	NA	1
4302	0.53812267	201	937	1
1827	0.513957307	NA	NA	1
2944	0.538520214	NA	NA	1
4401	0.548559671	NA	NA	1
5198	0.493632635	889	671	1
22939	0.53588907	NA	NA	1
1773	0.44205298	NA	NA	1
7949	0.499544419	NA	NA	1
4488	0.490044248	NA	NA	1
1668	0.402679831	NA	NA	1
1184	0.556747096	NA	NA	1
2148	0.595733333	NA	NA	1
1670	0.512700535	NA	NA	1
2440	0.518787879	NA	NA	1
4033	0.491833031	872	703	1
2636	0.485897436	NA	NA	1
40102	0.468078803	190	1000	0
6174	0.48932487	NA	NA	1
4782	0.493833132	NA	NA	0
2872	0.545751634	1000	993	1
2203	0.534592738	878	1000	0
4694	0.538461538	638	1000	1

10758	0.494060475	NA	NA		1
1806	0.502478315	NA	NA		1
743	0.550748752	NA	NA		1
2211	0.508604207	NA	NA		0
1537	0.4826546	NA	NA		1
1358	0.564250412	NA	NA		1
3827	0.503473187	NA	NA		1
2351	0.544676297	NA	NA		1
1315	0.545671168	NA	NA		1
3568	0.520931926	NA	NA		1
4746	0.57448606	1000		929	1
659	0.46179402	NA	NA		1
2224	0.484285714	941		935	1
2463	0.476584022	NA	NA		1
3139	0.507833397	603		1000	1
745	0.457100592	NA	NA		1
3276	0.547860609	NA	NA		1
833	0.470409712	NA	NA		1
1170	0.434036939	NA	NA		1
16464	0.487975952	649		1000	1
2133	0.437703142	NA	NA		1
1073	0.545629203	NA	NA		1
9210	0.532410611	805		778	1
6207	0.536125654	884		1000	1
690	0.607246377	NA	NA		1
4716	0.505747126	NA	NA		1
950	0.55704698	NA	NA		1
744	0.584677419	NA	NA		1
6479	0.563059034	316		714	1
960	0.504166667	NA	NA		1
930	0.504983389	NA	NA		1
11293	0.520058949	576		1000	1
1612	0.468774094	NA	NA		1
992	0.587978142	NA	NA		1
1990	0.54349292	NA	NA		1
4228	0.574683544	NA	NA		1
1098	0.557281553	NA	NA		1
2089	0.501917808	NA	NA		0
1374	0.475982533	NA	NA		1
2326	0.523091881	872		942	1
6528	0.456487202	NA	NA		1
3481	0.49640126	590		1000	0
3826	0.56370051	824		944	1
1272	0.482704403	NA	NA		1
8659	0.508411867	NA	NA		0
1105	0.547584187	NA	NA		1
2996	0.469210755	696		823	1
3488	0.52281435	508		1000	1
945	0.486552567	NA	NA		1
4062	0.453650958	NA	NA		1
10855	0.517270079	495		1000	1
8630	0.569474442	1000		781	1
12829	0.492245026	NA	NA		1
4729	0.578973105	1000		809	1
1587	0.514837819	NA	NA		1
1817	0.500284576	NA	NA		1
3477	0.531726663	NA	NA		1

5629	0.517477344	778		1000	1
2004	0.505912596	NA	NA		1
5688	0.425660835	626		1000	0
5358	0.427247956	NA	NA		1
3205	0.408460237	750		1000	1
2456	0.598694124	NA	NA		1
1433	0.526955201	NA	NA		1
3087	0.462192394	800		1000	1
11587	0.52446184	851		575	1
3707	0.452733313	1000		833	1
4815	0.521998743	624		1000	1
1295	0.541525424	NA	NA		1
2237	0.48582996	NA	NA		1
1036	0.426246185	NA	NA		1
2374	0.628807659	924		1000	1
620	0.508802817	NA	NA		1
1737	0.461261261	NA	NA		1
2517	0.541173794	855		933	1
1858	0.491520788	NA	NA		1
3895	0.559421999	895		895	0
1120	0.463645943	NA	NA		1
1304	0.471698113	NA	NA		1
3295	0.510832055	1000		1000	0
1430	0.476173021	NA	NA		1
9040	0.49564646	NA	NA		0
62433	0.467178598	NA	NA		1
2062	0.487431694	NA	NA		1
20142	0.503002473	NA	NA		1
1274	0.472222222	NA	NA		1
1023	0.422797927	NA	NA		1
2067	0.495132128	NA	NA		1
1071	0.450046685	NA	NA		1
4218	0.565528197	909		857	1
1665	0.607142857	NA	NA		1
22102	0.40284006	NA	NA		1
4421	0.49599013	NA	NA		1
5508	0.521700843	431		939	1
3779	0.445816664	579		1000	0
34180	0.512121682	NA	NA		0
2796	0.555789474	862		1000	0
2444	0.477310924	NA	NA		0
16185	0.488743787	615		1000	1
1403	0.513219285	NA	NA		1
3149	0.546854083	949		1000	1
2104	0.42402633	NA	NA		1
6635	0.516129032	1000		937	1
888	0.516324063	NA	NA		1
8139	0.507572959	88		804	1
2967	0.514275256	NA	NA		1
2549	0.521430727	NA	NA		0
2891	0.464963221	NA	NA		1
4233	0.485347985	NA	NA		1
3079	0.538892782	949		886	1
5843	0.49397953	NA	NA		1
2070	0.469096672	NA	NA		1
6978	0.516713297	877		936	1
755	0.414569536	NA	NA		1

1423	0.494960806	NA	NA	1
2535	0.517554479	1000	1000	0
945	0.53125	NA	NA	1
7160	0.518877728	NA	NA	0
7160	0.501609599	NA	NA	1
4152	0.446675031	1000	925	1
1092	0.484432234	NA	NA	1
4789	0.521604938	1000	769	1
2065	0.460428074	944	1000	1
1589	0.46894198	NA	NA	1
1076	0.513751375	NA	NA	1
1921	0.443542934	NA	NA	1
2978	0.464198734	545	934	1
4209	0.541046278	918	1000	1
8741	0.478188846	461	1000	1
2089	0.499713795	NA	NA	1
3067	0.488290654	813	937	1
3372	0.466570812	NA	NA	1
3462	0.450796951	711	875	1
4778	0.482047364	NA	NA	1
5712	0.512295974	759	336	0
851	0.553284672	NA	NA	1
12704	0.549162418	241	1000	1
5506	0.483374037	NA	NA	1
14843	0.489848097	380	1000	0
1706	0.517482517	NA	NA	1
16639	0.482376636	678	880	1
29437	0.536681716	NA	NA	0
7258	0.446998723	NA	NA	1
5589	0.521281923	NA	NA	1
1182	0.464559819	NA	NA	0
6611	0.499522597	797	876	1
2056	0.520489622	NA	NA	1
657	0.498127341	NA	NA	1
717	0.49244713	NA	NA	1
1210	0.449586777	NA	NA	1
749	0.477970628	NA	NA	1
34654	0.54016354	791	1000	0
7192	0.46092881	1000	1000	1
6116	0.502314815	NA	NA	0
6214	0.535048802	NA	NA	0
2071	0.502529511	NA	NA	1
41217	0.507173421	212	1000	1
2062	0.526946108	942	1000	1
2940	0.518924303	1000	372	1
2919	0.527453524	751	946	1
5768	0.510255187	832	566	0
3477	0.501362398	NA	NA	1
1647	0.454416961	NA	NA	1
868	0.492518703	NA	NA	1
762	0.509186352	NA	NA	1
5453	0.476064333	NA	NA	1
5818	0.482222695	NA	NA	0
4881	0.485720467	119	731	0
1264	0.532338308	NA	NA	1
7145	0.489069379	NA	NA	1
5303	0.54204893	NA	NA	1

989	0.469017094	NA	NA		1
2333	0.483154671	NA	NA		1
756	0.501322751	NA	NA		1
10169	0.519563091	NA	NA		1
1440	0.540277778	NA	NA		1
1071	0.445270988	NA	NA		1
1061	0.460159363	NA	NA		1
1859	0.460213777	NA	NA		1
21577	0.455645161	389		1000	1
4905	0.520967591	785		932	0
13960	0.497195785	NA	NA		1
4350	0.483658537	NA	NA		1
13674	0.49341142	NA	NA		0
5093	0.512634239	NA	NA		1
2852	0.523966488	621		859	0
2756	0.502971768	NA	NA		1
1353	0.575692042	NA	NA		1
2196	0.515974441	NA	NA		1
2199	0.520601336	NA	NA		1
2744	0.496941896	936		1000	1
8239	0.508679726	NA	NA		1
6280	0.495749848	918		1000	1
773	0.494949495	NA	NA		1
3545	0.497520149	870		1000	1
558	0.433691756	NA	NA		1
7786	0.482129311	NA	NA		1
2654	0.603617182	1000		1000	1
2583	0.472351421	NA	NA		0
1334	0.457725948	NA	NA		1
5179	0.535113749	925		1000	1
1174	0.456603774	NA	NA		1
7891	0.48874924	426		871	1
2745	0.480505013	948		1000	1
5231	0.56	157		856	0
3263	0.53798127	797		879	1
4789	0.49034536	NA	NA		0
4574	0.452088452	759		1000	1
1413	0.455060156	NA	NA		1
48447	0.516025297	374		1000	1
6327	0.524576413	888		1000	1
4022	0.461538462	NA	NA		1
6056	0.509411765	1000		930	1
6794	0.433333333	NA	NA		1
5003	0.470615243	411		1000	1
1627	0.578475336	NA	NA		1
838	0.594272076	NA	NA		1
5319	0.459078979	NA	NA		1
680	0.461788618	NA	NA		1
4059	0.479194043	NA	NA		1
10028	0.507920458	761		942	0
3160	0.476377953	NA	NA		1
4553	0.512718357	328		879	1
3261	0.503084304	886		900	1
4401	0.439676113	536		937	1
3207	0.505274262	750		940	1
5338	0.522921215	NA	NA		1
527	0.570498915	NA	NA		1

4307	0.51082837	NA	NA		1
1125	0.507981221	NA	NA		1
3572	0.421035806	764		892	1
792	0.601010101	NA	NA		1
2572	0.503352892	NA	NA		1
7385	0.476572133	NA	NA		1
11526	0.503616948	NA	NA		0
1303	0.458173446	NA	NA		1
5037	0.487714988	NA	NA		1
7337	0.50372403	459		938	1
8881	0.483189865	NA	NA		1
532	0.467640919	NA	NA		1
8788	0.498152709	NA	NA		1
1165	0.522900763	NA	NA		1
991	0.53722795	NA	NA		1
2232	0.416557162	NA	NA		1
7771	0.468281536	483		1000	0
1154	0.498947368	NA	NA		1
2326	0.472271127	946		1000	1
5671	0.516910651	427		1000	1
2134	0.529411765	NA	NA		1
1502	0.432124352	NA	NA		1
6482	0.544356173	440		914	1
1467	0.507526882	NA	NA		1
4351	0.599821747	491		689	1
4146	0.495523152	NA	NA		1
3727	0.501590491	NA	NA		1
7048	0.505546751	NA	NA		1
1598	0.518791946	NA	NA		1
16069	0.419642857	447		1000	1
3271	0.498170934	841		1000	1
1170	0.47008547	NA	NA		1
759	0.420600858	NA	NA		1
7199	0.472547255	NA	NA		1
6037	0.486224709	NA	NA		1
2174	0.482520699	1000		1000	1
1300	0.450769231	NA	NA		1
4700	0.532110092	NA	NA		1
3121	0.534958602	673		764	1
1084	0.494834711	NA	NA		1
8043	0.449948043	NA	NA		1
2077	0.517989418	NA	NA		1
1281	0.4980484	NA	NA		1
922	0.527845036	NA	NA		1
15495	0.519816514	477		936	0
4624	0.602757212	927		864	1
13676	0.446422684	64		205	1
3070	0.539855072	NA	NA		1
1324	0.485649547	NA	NA		1
6553	0.483669412	488		1000	1
4178	0.464805825	NA	NA		1
1324	0.496710526	NA	NA		1
4530	0.468319559	NA	NA		1
285	0.585964912	NA	NA		1
9279	0.471340713	501		1000	0
28987	0.525030902	485		726	1
5522	0.460474951	613		1000	0

2692	0.491930478	NA	NA	0
1409	0.473729543	NA	NA	1
870	0.553770087	NA	NA	1
3932	0.462355658	331	897	1
4370	0.530425118	921	931	1
2468	0.537364798	692	937	1
2277	0.505016722	NA	NA	1
2760	0.509163347	832	1000	1
3933	0.53381295	NA	NA	1
9936	0.535131029	NA	NA	1
1726	0.473839662	NA	NA	1
29999	0.526705447	NA	NA	1
1308	0.464824121	NA	NA	1
2931	0.523787529	686	880	1
1206	0.594498669	NA	NA	1
10614	0.494236745	NA	NA	1
1840	0.44184446	NA	NA	1
1960	0.567674113	NA	NA	1
3182	0.50397878	948	944	1
1654	0.5	NA	NA	1
1020	0.469135802	NA	NA	1
5080	0.506691761	947	818	1
1483	0.53202967	NA	NA	1
2198	0.460674157	NA	NA	1
8664	0.478067885	NA	NA	1
2497	0.501713796	837	1000	1
2540	0.487964989	NA	NA	1
6760	0.480820223	999	933	0
9536	0.489632107	NA	NA	0
1885	0.542713568	NA	NA	1
3089	0.520118734	1000	943	1
833	0.553421369	NA	NA	1
921	0.523344191	NA	NA	1
2259	0.4743083	NA	NA	1
1411	0.471286371	NA	NA	0
2413	0.491052632	NA	NA	1
1246	0.511210762	NA	NA	1
1053	0.448065173	NA	NA	1
1449	0.558273381	NA	NA	1
7863	0.434817382	NA	NA	1
2064	0.50170068	NA	NA	1
6135	0.514340511	1000	931	0
1629	0.445557782	NA	NA	1
14518	0.529632906	194	795	0
8861	0.474328406	159	1000	1
467	0.397022333	NA	NA	1
6372	0.489922638	467	1000	0
3300	0.517846694	396	1000	0
654	0.477842004	NA	NA	1
1146	0.511427339	NA	NA	0
339	0.437956204	NA	NA	1
5490	0.538532533	271	935	1
1084	0.473170732	NA	NA	1
2172	0.482965009	1000	1000	1
23668	0.468292683	631	1000	1
7705	0.499277665	457	862	1
1849	0.399452804	NA	NA	1

1041	0.43123061	NA	NA		1
3056	0.523372527	NA	NA		1
2971	0.514630578	NA	NA		1
5067	0.475285171	846		1000	1
1737	0.529868578	NA	NA		1
5288	0.510853835	NA	NA		1
4702	0.507387083	1000		1000	0
5134	0.591366439	211		1000	1
748	0.487055016	NA	NA		1
1041	0.536534447	NA	NA		1
898	0.579393939	NA	NA		1
2916	0.537154989	NA	NA		1
5941	0.440110142	225		1000	0
3717	0.52173913	1000		1000	1
2790	0.516129032	1000		1000	1
2298	0.479019331	NA	NA		1
43594	0.500691938	NA	NA		0
2942	0.454137931	777		846	0
3876	0.482088908	NA	NA		1
2939	0.515832106	NA	NA		1
15773	0.439618122	1000		423	1
4248	0.519738435	1000		941	1
3330	0.432776801	NA	NA		1
815	0.542331288	NA	NA		1
570	0.563157895	NA	NA		1
3110	0.542584676	1000		983	1
4477	0.494970338	645		882	1
803	0.477848101	NA	NA		1
20470	0.498734578	619		1000	0
11814	0.505324557	261		1000	0
3577	0.489482201	NA	NA		1
4024	0.409066667	NA	NA		1
579	0.585492228	NA	NA		1
1933	0.487842732	NA	NA		1
1189	0.552	NA	NA		1
2337	0.527477477	NA	NA		1
5999	0.546844444	1000		935	1
1672	0.519044545	NA	NA		1
447	0.561521253	NA	NA		1
6689	0.527906977	168		857	1
1914	0.457607433	NA	NA		1
5599	0.476603469	NA	NA		1
14496	0.491111638	481		834	0
1819	0.611713666	NA	NA		1
1276	0.450626959	NA	NA		1
2541	0.481263158	834		1000	1
1010	0.391089109	NA	NA		1
1524	0.489255014	NA	NA		1
4126	0.401894452	NA	NA		1
1037	0.499386503	NA	NA		1
1505	0.48603352	NA	NA		1
2841	0.551230261	NA	NA		1
1228	0.564938272	NA	NA		0
3637	0.515779407	NA	NA		1
5523	0.468325792	937		841	1
3966	0.479795259	934		929	1
1154	0.493934142	NA	NA		1

2107	0.507356431	1000	1000	1
16967	0.45706466	761	1000	1
1944	0.46400484	NA	NA	1
7678	0.535805939	762	1000	0
75226	0.524786719	NA	NA	1
2202	0.539963669	NA	NA	1
1449	0.540372671	NA	NA	1
2340	0.480682839	1000	941	1
1629	0.487837838	NA	NA	1
1639	0.489872468	NA	NA	1
31255	0.451147981	NA	NA	0
5770	0.541646489	756	1000	1
1074	0.554003724	NA	NA	1
8809	0.502858662	NA	NA	1
5839	0.494670543	146	747	1
15290	0.55718253	445	914	1
1590	0.509562842	NA	NA	1
2972	0.527916251	585	1000	1
2233	0.452934662	NA	NA	1
2992	0.559566787	NA	NA	1
7341	0.53808752	499	1000	1
11705	0.462353136	NA	NA	1
2270	0.46482543	886	870	0
1567	0.562240664	NA	NA	1
1736	0.524096386	NA	NA	1
2317	0.497026759	861	840	1
948	0.577004219	NA	NA	1
5568	0.485185185	NA	NA	1
27762	0.494915958	NA	NA	1
6754	0.525345622	609	1000	1
3065	0.5108	854	648	1
1807	0.513558384	NA	NA	1
736	0.421052632	NA	NA	1
759	0.392795883	NA	NA	1
5404	0.502009041	NA	NA	1
12078	0.619004525	NA	NA	1
1300	0.566158781	NA	NA	1
2125	0.477911647	NA	NA	0
526	0.537154989	NA	NA	1
1857	0.533670034	NA	NA	1
5022	0.522299775	NA	NA	1
745	0.534228188	NA	NA	1
3624	0.43543669	NA	NA	1
2053	0.48970399	NA	NA	1
916	0.478311841	NA	NA	1
552	0.498188406	NA	NA	1
14703	0.560229446	136	933	1
5377	0.526806527	289	1000	1
2069	0.549430797	NA	NA	1
9116	0.535919976	240	1000	1
1499	0.538293217	NA	NA	1
1304	0.469536962	NA	NA	1
1283	0.516734694	NA	NA	1
5164	0.517322219	705	1000	0
9425	0.492659478	480	836	1
1336	0.452958293	NA	NA	1
8498	0.517558128	1000	859	0

2714	0.497303544	939	987	1
2992	0.504225352	387	880	1
5728	0.54603917	NA	NA	0
2560	0.535960591	NA	NA	1
611	0.464737794	NA	NA	1
6604	0.50019478	NA	NA	1
7820	0.452694103	NA	NA	1
11038	0.49860042	410	937	1
1755	0.533469945	NA	NA	1
1201	0.408091469	NA	NA	1
793	0.467741935	NA	NA	1
2582	0.524911403	875	1000	1
1537	0.492652204	NA	NA	1
5444	0.466466235	NA	NA	1
461	0.577006508	NA	NA	1
7110	0.491940299	NA	NA	1
9714	0.484397718	699	936	0
12364	0.496261314	127	937	0
1566	0.492357724	NA	NA	1
1634	0.579617834	NA	NA	1
69176	0.512587772	NA	NA	0
735	0.53877551	NA	NA	1
2091	0.436047933	NA	NA	0
2347	0.501345895	NA	NA	0
1099	0.489463602	NA	NA	1
1199	0.461217681	NA	NA	1
4326	0.478718317	913	1000	1
2421	0.398715703	NA	NA	0
551	0.49694501	NA	NA	1
1133	0.457249071	NA	NA	1
4930	0.488896722	NA	NA	1
18149	0.514359793	NA	NA	1
3445	0.465437788	NA	NA	1
271	0.457564576	NA	NA	1
1842	0.424393279	NA	NA	1
10369	0.57790493	600	757	1
3885	0.513140967	990	1000	0
16461	0.489658579	274	1000	1
17374	0.560005069	596	801	1
3190	0.441671739	761	1000	1
1748	0.480284421	NA	NA	1
1482	0.512145749	NA	NA	1
5666	0.560502283	NA	NA	1
1857	0.431845597	NA	NA	1
2423	0.531697342	NA	NA	1
1502	0.501945525	NA	NA	1
1993	0.487721302	NA	NA	1
3136	0.507393209	NA	NA	1
3739	0.478274968	761	879	1
1884	0.455569462	NA	NA	1
856	0.464333782	NA	NA	1
1769	0.549880668	NA	NA	1
4051	0.559683147	1000	988	1
3304	0.488057041	854	758	1
1608	0.540592784	NA	NA	1
2112	0.474974975	NA	NA	1
9287	0.470214456	NA	NA	1

2046	0.465663833	NA	NA		1
1594	0.427258806	NA	NA		1
8083	0.525711662	93	1000		1
11690	0.452484949	127	1000		1
3225	0.535591353	755	945		1
920	0.511956522	NA	NA		1
3377	0.514514093	1000	1000		1
2160	0.545644236	NA	NA		1
1400	0.459265176	NA	NA		0
3389	0.421405658	386	1000		1
1479	0.533613445	NA	NA		1
6999	0.536772217	785	978		1
8241	0.509482179	559	691		1
25244	0.5483669	NA	NA		0
4955	0.500829738	50	939		1
2352	0.459841629	NA	NA		1
7058	0.490214797	NA	NA		1
8890	0.517553306	406	908		0
783	0.58109834	NA	NA		1
1446	0.539712919	NA	NA		1
33766	0.522499191	875	1000		0
2236	0.434538971	NA	NA		1
2313	0.458507963	NA	NA		1
1313	0.485530547	NA	NA		1
7176	0.549030896	669	876		1
3544	0.50959061	949	1000		1
4684	0.538694992	NA	NA		1
5560	0.471189591	295	1000		1
9926	0.471907216	400	1000		0
1597	0.491847826	NA	NA		1
4624	0.494762288	NA	NA		0
5786	0.501145374	NA	NA		1
2725	0.508408291	NA	NA		1
765	0.504643963	NA	NA		1
1945	0.520316027	NA	NA		1
5453	0.493062967	NA	NA		0
977	0.529550827	NA	NA		1
1077	0.544262295	NA	NA		1
1134	0.511677282	NA	NA		1
6896	0.555603448	NA	NA		1
3226	0.445556691	622	898		0
11027	0.528735632	1000	1000		1
11796	0.533375715	753	1000		1
4745	0.483177949	NA	NA		1
1393	0.487603306	NA	NA		1
4544	0.421852326	767	1000		0
3988	0.534114053	NA	NA		1
6303	0.447870778	850	877		1
13256	0.50999632	800	1000		1
3583	0.503550296	NA	NA		0
2761	0.510187449	NA	NA		1
2035	0.475247525	NA	NA		1
11033	0.484927916	663	833		1
7665	0.495994546	NA	NA		1
1953	0.505449591	NA	NA		1
2246	0.484567901	NA	NA		1
2890	0.526789541	NA	NA		1

4664	0.450301205	NA	NA	1
3205	0.46459854	829	883	1
1147	0.514385353	NA	NA	1
1799	0.500910747	NA	NA	1
10707	0.521820216	NA	NA	0
2040	0.474935401	NA	NA	1
939	0.474973376	NA	NA	1
4829	0.517230427	NA	NA	1
5718	0.490666667	NA	NA	1
3918	0.486724973	933	995	0
1317	0.415337889	NA	NA	1
3726	0.511532847	846	912	1
4855	0.493307468	NA	NA	1
4652	0.428194607	NA	NA	1
2380	0.537265416	NA	NA	1
2901	0.467336683	NA	NA	1
1649	0.454481299	NA	NA	1
439	0.530183727	NA	NA	1
698	0.499189627	NA	NA	1
5043	0.530730109	NA	NA	1
5632	0.495535714	220	876	1
1403	0.464393939	NA	NA	1
1621	0.429366736	NA	NA	1
3595	0.50752909	621	877	1
660	0.61969697	NA	NA	1
3083	0.500337838	NA	NA	1
3152	0.490680537	345	936	1
1125	0.444019139	NA	NA	1
1902	0.486330179	NA	NA	1
1885	0.52792475	NA	NA	1
1852	0.53380102	NA	NA	1
1625	0.502027027	NA	NA	1
4938	0.514269726	NA	NA	1
3231	0.474862529	623	780	1
3559	0.4826546	501	1000	1
2478	0.493412665	935	1000	1
639	0.599374022	NA	NA	1
3131	0.479623824	NA	NA	1
902	0.562084257	NA	NA	1
35131	0.48321707	NA	NA	1
1103	0.54306437	NA	NA	1
2058	0.542838457	NA	NA	1
2875	0.50877193	NA	NA	1
4896	0.514575972	919	940	1
3299	0.536819637	NA	NA	1
2631	0.557114883	NA	NA	0
3654	0.51208139	1000	938	1
2586	0.484532096	1000	1000	1
18491	0.442056075	486	1000	1
2630	0.511939673	945	924	1
2312	0.495940959	NA	NA	0
1079	0.504772004	NA	NA	1
8359	0.53511798	941	846	1
859	0.498655914	NA	NA	1
3437	0.483708431	660	937	1
1448	0.490974729	NA	NA	1
1156	0.570451436	NA	NA	1

1712	0.531629393	NA	NA		1
4011	0.489296636	846	880		0
4872	0.497319035	370	943		1
2314	0.495319604	NA	NA		0
635	0.541740675	NA	NA		1
1769	0.523659306	NA	NA		1
1120	0.550614948	NA	NA		1
1079	0.525793651	NA	NA		1
1131	0.523364486	NA	NA		1
1362	0.524615385	NA	NA		1
16276	0.454892889	619	1000		0
23544	0.47738371	NA	NA		1
1458	0.46090535	NA	NA		1
2176	0.523667165	NA	NA		1
723	0.520423601	NA	NA		1
10348	0.408769022	NA	NA		0
6764	0.465874551	125	1000		1
8683	0.416312057	280	1000		0
1129	0.507446809	NA	NA		1
22439	0.452924756	184	1000		1
12700	0.513318191	NA	NA		1
2155	0.497297297	1000	880		1
1977	0.548098434	NA	NA		1
2239	0.526864035	NA	NA		1
609	0.564899452	NA	NA		1
1079	0.509731233	NA	NA		1
1518	0.480013436	NA	NA		1
5248	0.526609225	NA	NA		1
2823	0.528004784	939	844		0
2313	0.537768884	NA	NA		1
4430	0.508820799	1000	936		1
16937	0.439698965	NA	NA		0
2158	0.568362968	NA	NA		1
4807	0.521162559	NA	NA		0
6884	0.479046836	NA	NA		1
1145	0.524017467	NA	NA		1
430	0.423255814	NA	NA		1
1180	0.542597501	NA	NA		0
3421	0.481505728	794	1000		0
2365	0.497627119	NA	NA		1
2574	0.564204923	NA	NA		1
2501	0.479786096	941	1000		0
7524	0.502415459	338	1000		0
8438	0.49125829	361	1000		0
5099	0.502783371	274	891		1
3412	0.495212285	856	831		0
1664	0.54516129	NA	NA		1
3522	0.469785575	418	869		1
2916	0.455299539	942	799		1
19589	0.49103402	NA	NA		0
3528	0.525644233	212	812		0
6104	0.488291414	NA	NA		1
4676	0.52536181	NA	NA		0
3113	0.486982644	941	1000		1
622	0.516966068	NA	NA		1
935	0.471657754	NA	NA		1
5004	0.53327836	154	939		1

3043	0.553808526	NA	NA		1
1165	0.530007794	NA	NA		0
1088	0.47957993	NA	NA		1
1624	0.489326765	NA	NA		1
6472	0.507728894	NA	NA		1
3215	0.502479688	437		910	0
18358	0.4589475	250		1000	0
2654	0.490928215	578		938	1
1283	0.54723127	NA	NA		0
885	0.621468927	NA	NA		1
1204	0.452847806	NA	NA		1
817	0.58629131	NA	NA		1
26945	0.486734845	NA	NA		0
5039	0.508800399	NA	NA		1
14979	0.552693208	NA	NA		1
5422	0.526005164	1000		1000	1
5854	0.516302147	884		418	1
20928	0.498678414	NA	NA		1
4749	0.496805649	1000		1000	0
4332	0.481574539	275		643	1
925	0.474127557	NA	NA		1
4668	0.538011696	NA	NA		1
5217	0.540250447	NA	NA		0
3521	0.430762736	409		1000	0
3603	0.544193062	577		1000	0
4178	0.495163241	NA	NA		0
2663	0.522674865	1000		939	1
7190	0.521779641	NA	NA		1
3524	0.525656468	446		883	0
2344	0.521066667	NA	NA		1
9035	0.435253965	NA	NA		0
5307	0.518180741	624		870	0
3745	0.508353222	NA	NA		0
1095	0.485992692	NA	NA		0
2282	0.396759259	NA	NA		1
19720	0.526530612	494		1000	1
18347	0.510551249	947		831	1
966	0.494983278	NA	NA		1
2331	0.45290499	NA	NA		1
1014	0.399791232	NA	NA		1
22827	0.458026961	252		1000	1
3451	0.492052832	NA	NA		0
12368	0.561434659	259		1000	1
1949	0.531755915	NA	NA		0
1687	0.481770833	NA	NA		1
1517	0.489754098	NA	NA		1
18003	0.492642405	153		1000	1
5824	0.467637927	NA	NA		1
1205	0.489626556	NA	NA		1
1473	0.512877116	NA	NA		1
4207	0.545143509	1000		844	0
743	0.500364166	NA	NA		1
1789	0.54620586	NA	NA		0
12831	0.569832402	265		1000	1
2374	0.466697936	821		1000	1
1316	0.512647555	NA	NA		1
1590	0.558707124	NA	NA		1

17700	0.473189087	NA	NA	0
565	0.378151261	NA	NA	1
6816	0.507977952	NA	NA	1
478	0.516666667	NA	NA	1
3589	0.509819324	NA	NA	1
2990	0.477798139	NA	NA	0
945	0.545764639	NA	NA	0
1925	0.5146328	NA	NA	1
3480	0.490584737	NA	NA	1
5149	0.528087567	943	1000	1
635	0.540206186	NA	NA	1
709	0.480686695	NA	NA	1
4016	0.481541802	884	907	1
478	0.513020833	NA	NA	1
523	0.550505051	NA	NA	1
1801	0.481359649	NA	NA	1
2330	0.510451045	NA	NA	1
2069	0.568965517	NA	NA	1
1066	0.493055556	NA	NA	1
64721	0.541150092	1000	1000	1
4975	0.530314777	611	942	0
36826	0.522527794	294	1000	1
1814	0.530048077	NA	NA	1
1370	0.470532319	NA	NA	0
487	0.583162218	NA	NA	1
4793	0.553571429	NA	NA	0
18047	0.505157056	909	545	0
2670	0.51864319	451	940	1
1923	0.541014402	NA	NA	1
3027	0.525429799	NA	NA	1
2388	0.519070583	NA	NA	1
1772	0.512532411	NA	NA	0
1773	0.536161335	NA	NA	1
11142	0.532574229	NA	NA	0
1828	0.442340792	NA	NA	1
4531	0.538671875	NA	NA	1
4830	0.44152731	540	922	0
3087	0.56656535	NA	NA	1
6797	0.542804654	NA	NA	0
3590	0.489812821	NA	NA	1
5268	0.523746702	827	1000	1
826	0.454427083	NA	NA	1
5711	0.460236542	525	853	0
11288	0.510749564	204	1000	1
3321	0.527060271	931	1000	1
4935	0.57296467	NA	NA	1
21971	0.527931111	1000	878	1
1136	0.395522388	NA	NA	1
1768	0.539136302	NA	NA	1
1105	0.517647059	NA	NA	1
9439	0.445496536	597	941	1
46668	0.47804083	NA	NA	0
812	0.464285714	NA	NA	1
3413	0.427617602	NA	NA	1
1591	0.495285984	NA	NA	1
9104	0.441026574	475	982	0
902	0.483193277	NA	NA	1

10045	0.518567892	308	935	0
1553	0.489917849	NA	NA	1
441	0.551020408	NA	NA	1
455	0.615384615	NA	NA	1
4299	0.559127805	NA	NA	0
4793	0.485628077	293	1000	0
615	0.578861789	NA	NA	1
3390	0.533532537	364	1000	0
1095	0.500512821	NA	NA	1
2935	0.5314164	NA	NA	1
2948	0.557037037	NA	NA	1
597	0.58961474	NA	NA	1
1140	0.447368421	NA	NA	1
7718	0.493448702	291	1000	1
2351	0.496989347	808	1000	1
2098	0.471299094	NA	NA	1
2427	0.518358531	1000	946	1
865	0.498626374	NA	NA	1
3182	0.511792453	591	641	1
1333	0.541666667	NA	NA	1
4700	0.517136329	NA	NA	1
2258	0.517900932	NA	NA	1
1452	0.423251589	NA	NA	1
8382	0.496032246	NA	NA	0
4533	0.481255449	NA	NA	1
3657	0.508344459	NA	NA	1
2537	0.51737268	NA	NA	1
946	0.558804418	NA	NA	0
8904	0.493565481	645	1000	1
1706	0.514276444	NA	NA	0
1000	0.482504604	NA	NA	1
1031	0.43654267	NA	NA	1
2994	0.504930394	NA	NA	0
14205	0.490349504	88	553	0
1627	0.526452732	NA	NA	1
1292	0.491186279	NA	NA	1
5668	0.423736462	NA	NA	1
1809	0.519428571	NA	NA	1
2091	0.482758621	NA	NA	1
2316	0.572970639	NA	NA	1
1213	0.5	NA	NA	1
21786	0.49908341	NA	NA	0
1162	0.411231884	NA	NA	1
7677	0.579847909	307	1000	1
6075	0.504513392	582	1000	0
6002	0.474367294	NA	NA	1
7236	0.557601881	800	885	1
3644	0.479649343	940	1000	1
1313	0.536904762	NA	NA	1
2818	0.428914698	744	935	1
854	0.447306792	NA	NA	1
10769	0.488350527	236	1000	0
5243	0.501030928	552	1000	1
8967	0.48031688	731	1000	1
4592	0.536922015	1000	919	1
607	0.486880466	NA	NA	1
1793	0.485040276	NA	NA	1

5618	0.525955379	436		884	1
1610	0.53030303	NA	NA		1
1636	0.524535809	NA	NA		1
2008	0.56271777	NA	NA		1
985	0.47008547	NA	NA		1
1543	0.534482759	NA	NA		1
1974	0.513171226	NA	NA		1
2312	0.557958478	1000		1000	1
2156	0.576530612	NA	NA		1
3485	0.482869379	NA	NA		1
7747	0.478735539	431		935	0
1866	0.524651661	NA	NA		1
2037	0.507556019	NA	NA		1
2037	0.378988709	1000		1000	1
2903	0.488255034	798		838	0
7134	0.479809976	400		1000	1
1484	0.510576222	NA	NA		1
5382	0.524271845	289		1000	0
6630	0.495789474	NA	NA		1
1091	0.548881037	NA	NA		1
1054	0.533932136	NA	NA		1
1104	0.531702899	NA	NA		1
1556	0.540333797	NA	NA		1
678	0.463983051	NA	NA		1
1403	0.502719503	NA	NA		1
3356	0.412239408	NA	NA		1
29023	0.437873988	1000		1000	0
1229	0.528885273	NA	NA		1
783	0.551724138	NA	NA		1
2378	0.51530153	844		1000	1
955	0.489005236	NA	NA		1
972	0.474279835	NA	NA		1
1641	0.515802781	NA	NA		1
7782	0.533485323	NA	NA		1
1549	0.532846715	NA	NA		1
1614	0.531531532	NA	NA		1
1712	0.518495298	NA	NA		1
7206	0.463824604	178		1000	1
2375	0.524865189	NA	NA		1
1948	0.523613963	NA	NA		1
1283	0.470391993	NA	NA		1
24608	0.498088476	NA	NA		1
3397	0.505921364	297		933	0
1663	0.501670007	NA	NA		1
4981	0.508588064	553		810	1
3269	0.445804989	263		954	1
10105	0.476609105	1000		1000	0
8204	0.498856583	84		1000	1
1047	0.527842227	NA	NA		1
10504	0.476369495	NA	NA		1
667	0.508245877	NA	NA		1
977	0.423413567	NA	NA		1
605	0.534618755	NA	NA		1
10963	0.473388621	806		1000	0
5798	0.512096774	NA	NA		1
2930	0.489260143	845		863	1
2398	0.502174964	828		945	1

3416	0.506756757	1000	1000	1
824	0.513368984	NA	NA	1
2728	0.528250511	NA	NA	1
3132	0.47826087	876	1000	1
2048	0.495115995	NA	NA	1
1769	0.534200113	NA	NA	1
12311	0.533111005	NA	NA	1
6201	0.500959079	938	967	0
3482	0.522279036	869	1000	1
6503	0.516215365	493	1000	0
5743	0.528486164	NA	NA	1
6685	0.470370722	201	793	1
4428	0.505089374	698	926	1
6674	0.484848485	NA	NA	0
1198	0.533333333	NA	NA	1
1631	0.559044815	NA	NA	1
2782	0.506925208	NA	NA	0
1661	0.472568579	NA	NA	1
5059	0.523590564	1000	1000	1
1248	0.558798999	NA	NA	1
4621	0.53606769	327	1000	0
1938	0.535519126	NA	NA	1
1433	0.457246377	NA	NA	1
1441	0.53231663	NA	NA	1
4160	0.498910675	NA	NA	1
383	0.553524804	NA	NA	1
1086	0.476190476	NA	NA	1
1337	0.477449456	NA	NA	1
824	0.538057743	NA	NA	1
1510	0.537748344	NA	NA	1
5892	0.476508201	342	1000	1
2108	0.5082846	1000	944	1
1906	0.548297041	NA	NA	1
5221	0.453125	181	1000	1
2637	0.535008632	NA	NA	1
1920	0.530208333	NA	NA	1
2740	0.453900709	NA	NA	1
1827	0.464148878	NA	NA	1
2304	0.511056511	NA	NA	1
1028	0.389303483	NA	NA	1
1150	0.418064516	NA	NA	1
3460	0.479937304	916	879	1
915	0.473224044	NA	NA	1
2312	0.536792453	808	1000	1
2579	0.47678703	NA	NA	1
2134	0.555377207	NA	NA	1
6206	0.45115304	NA	NA	0
3449	0.512032473	1000	1000	1
2056	0.511552929	NA	NA	1
1569	0.489302968	NA	NA	1
48575	0.516686183	NA	NA	0
4981	0.489648033	NA	NA	0
8527	0.48422634	NA	NA	1
2976	0.560861423	1000	936	1
3583	0.506440465	868	931	1
5971	0.485859081	NA	NA	0
2591	0.457029528	832	932	1

5004	0.526840066	NA	NA		1
2238	0.467263307	NA	NA		1
2954	0.43904119	NA	NA		0
1976	0.536707453	NA	NA		1
38110	0.537352103		639	1000	1
5282	0.493975904	NA	NA		1
621	0.528180354	NA	NA		1
779	0.473684211	NA	NA		1
1396	0.461538462	NA	NA		1
997	0.513513514	NA	NA		1
2915	0.417035943		253	1000	1
645	0.522435897	NA	NA		1
72	0.583333333	NA	NA		1
87	0.609195402	NA	NA		1
71	0.676056338	NA	NA		1
72	0.583333333	NA	NA		1
72	0.625	NA	NA		1
72	0.625	NA	NA		1
72	0.625	NA	NA		1
82	0.56097561	NA	NA		1
82	0.56097561	NA	NA		1
72	0.611111111	NA	NA		1
71	0.676056338	NA	NA		1
72	0.611111111	NA	NA		1
72	0.611111111	NA	NA		1
71	0.676056338	NA	NA		1
72	0.583333333	NA	NA		1
73	0.602739726	NA	NA		1
71	0.676056338	NA	NA		1
74	0.581081081	NA	NA		1
72	0.597222222	NA	NA		1
72	0.569444444	NA	NA		1
72	0.597222222	NA	NA		1
82	0.585365854	NA	NA		1
73	0.589041096	NA	NA		1
72	0.611111111	NA	NA		1
71	0.676056338	NA	NA		1
82	0.646341463	NA	NA		1
73	0.534246575	NA	NA		1
72	0.625	NA	NA		1
72	0.611111111	NA	NA		1
82	0.646341463	NA	NA		1
82	0.646341463	NA	NA		1
71	0.676056338	NA	NA		1
72	0.5	NA	NA		1
72	0.541666667	NA	NA		1
72	0.555555556	NA	NA		1
72	0.541666667	NA	NA		1
82	0.646341463	NA	NA		1
74	0.581081081	NA	NA		1
74	0.581081081	NA	NA		1
74	0.581081081	NA	NA		1
72	0.597222222	NA	NA		1
72	0.597222222	NA	NA		1
72	0.597222222	NA	NA		1
72	0.597222222	NA	NA		1
72	0.583333333	NA	NA		1

73	0.589041096	NA	NA	1
73	0.602739726	NA	NA	1
72	0.541666667	NA	NA	1
71	0.352112676	NA	NA	1
83	0.578313253	NA	NA	1
74	0.581081081	NA	NA	1
74	0.554054054	NA	NA	1
74	0.567567568	NA	NA	1
73	0.616438356	NA	NA	1
73	0.616438356	NA	NA	1
73	0.616438356	NA	NA	1
73	0.616438356	NA	NA	1
73	0.561643836	NA	NA	1
73	0.616438356	NA	NA	1
74	0.581081081	NA	NA	1
73	0.561643836	NA	NA	1
73	0.561643836	NA	NA	1
74	0.635135135	NA	NA	1
73	0.589041096	NA	NA	1
73	0.534246575	NA	NA	1
499	0.502192982	NA	NA	1
72	0.583333333	NA	NA	1
72	0.583333333	NA	NA	1
72	0.555555556	NA	NA	1
72	0.541666667	NA	NA	1
72	0.597222222	NA	NA	1
72	0.541666667	NA	NA	1
72	0.569444444	NA	NA	1
72	0.541666667	NA	NA	1
71	0.366197183	NA	NA	1
72	0.541666667	NA	NA	1
73	0.616438356	NA	NA	1
127	0.578313253	NA	NA	1
74	0.581081081	NA	NA	1
74	0.635135135	NA	NA	1
74	0.635135135	NA	NA	1
73	0.561643836	NA	NA	1
74	0.635135135	NA	NA	1
73	0.561643836	NA	NA	1
73	0.616438356	NA	NA	1
73	0.616438356	NA	NA	1
73	0.616438356	NA	NA	1
74	0.635135135	NA	NA	1
74	0.635135135	NA	NA	1
73	0.616438356	NA	NA	1
74	0.581081081	NA	NA	1
80	0.6375	NA	NA	1
72	0.458333333	NA	NA	1
72	0.458333333	NA	NA	1
84	0.595238095	NA	NA	1
123	0.590361446	NA	NA	1
82	0.585365854	NA	NA	1
82	0.585365854	NA	NA	1
82	0.585365854	NA	NA	1
82	0.585365854	NA	NA	1
164	0.542682927	NA	NA	1
72	0.555555556	NA	NA	1

73	0.589041096	NA	NA	1
73	0.589041096	NA	NA	1
72	0.583333333	NA	NA	1
72	0.583333333	NA	NA	1
6494	0.45255102	NA	NA	1
31065	0.414318222	NA	NA	0
74	0.567567568	NA	NA	1
73	0.602739726	NA	NA	1
73	0.534246575	NA	NA	1
72	0.541666667	NA	NA	1
72	0.541666667	NA	NA	1
164	0.536585366	NA	NA	1
72	0.527777778	NA	NA	1
107	0.439252336	NA	NA	1
72	0.597222222	NA	NA	1
80	0.6375	NA	NA	1
93	0.534246575	NA	NA	1
82	0.585365854	NA	NA	1
72	0.583333333	NA	NA	1
73	0.589041096	NA	NA	1
72	0.541666667	NA	NA	1
94	0.534246575	NA	NA	1
93	0.534246575	NA	NA	1
94	0.534246575	NA	NA	1
73	0.575342466	NA	NA	1
74	0.567567568	NA	NA	1
82	0.585365854	NA	NA	1
72	0.541666667	NA	NA	1
73	0.397260274	NA	NA	1
73	0.561643836	NA	NA	1
73	0.561643836	NA	NA	1
73	0.561643836	NA	NA	1
73	0.561643836	NA	NA	1
72	0.583333333	NA	NA	1
72	0.583333333	NA	NA	1
74	0.567567568	NA	NA	1
73	0.589041096	NA	NA	1
73	0.589041096	NA	NA	1
84	0.607142857	NA	NA	1
73	0.589041096	NA	NA	1
72	0.583333333	NA	NA	1
107	0.439252336	NA	NA	1
72	0.597222222	NA	NA	1
93	0.534246575	NA	NA	1
73	0.561643836	NA	NA	1
72	0.583333333	NA	NA	1
73	0.589041096	NA	NA	1
73	0.589041096	NA	NA	1
73	0.589041096	NA	NA	1
72	0.583333333	NA	NA	1
73	0.534246575	NA	NA	1
72	0.583333333	NA	NA	1
73	0.589041096	NA	NA	1
73	0.589041096	NA	NA	1
73	0.589041096	NA	NA	1
73	0.575342466	NA	NA	1
72	0.583333333	NA	NA	1

73	0.589041096	NA	NA	1
73	0.589041096	NA	NA	1
73	0.561643836	NA	NA	1
73	0.561643836	NA	NA	1
74	0.635135135	NA	NA	1
73	0.561643836	NA	NA	1
73	0.561643836	NA	NA	1
73	0.534246575	NA	NA	1
73	0.589041096	NA	NA	1
73	0.534246575	NA	NA	1
74	0.567567568	NA	NA	1
74	0.567567568	NA	NA	1
73	0.589041096	NA	NA	1
73	0.589041096	NA	NA	1
73	0.561643836	NA	NA	1
72	0.597222222	NA	NA	1
72	0.597222222	NA	NA	1
72	0.597222222	NA	NA	1
718	0.456824513	NA	NA	1
399	0.49122807	NA	NA	1
465	0.468817204	NA	NA	1
1463	0.473643411	NA	NA	1
143	0.48951049	NA	NA	1
73	0.602739726	NA	NA	1
71	0.676056338	NA	NA	1
72	0.5	NA	NA	1
3295	0.429967427	NA	NA	0
82	0.573170732	NA	NA	1
314	0.547770701	NA	NA	1
84	0.607142857	NA	NA	1
72	0.458333333	NA	NA	1
192	0.489583333	NA	NA	1
127	0.417322835	NA	NA	1
192	0.484375	NA	NA	1
113	0.389380531	NA	NA	1
74	0.567567568	NA	NA	1
72	0.597222222	NA	NA	1
72	0.597222222	NA	NA	1
72	0.597222222	NA	NA	1
72	0.597222222	NA	NA	1
73	0.534246575	NA	NA	1
72	0.597222222	NA	NA	1
73	0.589041096	NA	NA	1
71	0.676056338	NA	NA	1
186	0.534246575	NA	NA	1
72	0.597222222	NA	NA	1
211	0.488151659	NA	NA	1
72	0.5	NA	NA	1
72	0.472222222	NA	NA	1
73	0.547945205	NA	NA	1
72	0.5	NA	NA	1
72	0.5	NA	NA	1
71	0.676056338	NA	NA	1
73	0.534246575	NA	NA	1
82	0.573170732	NA	NA	1
72	0.611111111	NA	NA	1
72	0.583333333	NA	NA	1

71	0.676056338	NA	NA	1
71	0.676056338	NA	NA	1
72	0.625	NA	NA	1
71	0.661971831	NA	NA	1
71	0.676056338	NA	NA	1
71	0.676056338	NA	NA	1
72	0.625	NA	NA	1
72	0.625	NA	NA	1
72	0.625	NA	NA	1
121	0.534246575	NA	NA	1
94	0.534246575	NA	NA	1
74	0.540540541	NA	NA	1
72	0.458333333	NA	NA	1
73	0.589041096	NA	NA	1
53	0.622641509	NA	NA	1
73	0.589041096	NA	NA	1
72	0.597222222	NA	NA	1
72	0.625	NA	NA	1
125	0.590361446	NA	NA	1
72	0.625	NA	NA	1
72	0.625	NA	NA	1
72	0.597222222	NA	NA	1
72	0.597222222	NA	NA	1
73	0.602739726	NA	NA	1
238	0.605042017	NA	NA	1
72	0.611111111	NA	NA	1
72	0.611111111	NA	NA	1
73	0.616438356	NA	NA	1
73	0.575342466	NA	NA	1
73	0.616438356	NA	NA	1
72	0.583333333	NA	NA	1
72	0.583333333	NA	NA	1
72	0.583333333	NA	NA	1
72	0.583333333	NA	NA	1
72	0.611111111	NA	NA	1
72	0.625	NA	NA	1
72	0.541666667	NA	NA	1
72	0.541666667	NA	NA	1
72	0.541666667	NA	NA	1
72	0.541666667	NA	NA	1
72	0.541666667	NA	NA	1
72	0.541666667	NA	NA	1
72	0.541666667	NA	NA	1
72	0.541666667	NA	NA	1
72	0.541666667	NA	NA	1
73	0.602739726	NA	NA	1
83	0.578313253	NA	NA	1
83	0.578313253	NA	NA	1
83	0.578313253	NA	NA	1
83	0.578313253	NA	NA	1
83	0.578313253	NA	NA	1
83	0.578313253	NA	NA	1
73	0.589041096	NA	NA	1
82	0.573170732	NA	NA	1
73	0.534246575	NA	NA	1
73	0.534246575	NA	NA	1
72	0.625	NA	NA	1

83	0.578313253	NA	NA	1
72	0.569444444	NA	NA	1
98	0.567567568	NA	NA	1
100	0.567567568	NA	NA	1
72	0.597222222	NA	NA	1
72	0.472222222	NA	NA	1
75	0.52	NA	NA	1
91	0.527472527	NA	NA	1
95	0.534246575	NA	NA	1
108	0.547945205	NA	NA	1
73	0.575342466	NA	NA	1
72	0.611111111	NA	NA	1
82	0.573170732	NA	NA	1
82	0.585365854	NA	NA	1
82	0.573170732	NA	NA	1
73	0.561643836	NA	NA	1
82	0.585365854	NA	NA	1
82	0.573170732	NA	NA	1
73	0.589041096	NA	NA	1
82	0.585365854	NA	NA	1
73	0.589041096	NA	NA	1
73	0.589041096	NA	NA	1
82	0.585365854	NA	NA	1
73	0.589041096	NA	NA	1
82	0.585365854	NA	NA	1
73	0.575342466	NA	NA	1
782	0.348797251	NA	NA	1
873	0.402140673	NA	NA	1
73	0.534246575	NA	NA	1
73	0.534246575	NA	NA	1
71	0.478873239	NA	NA	1
3792	0.404134295	1000	1000	0
72	0.611111111	NA	NA	1
72	0.5	NA	NA	1
99	0.464646465	NA	NA	1
72	0.625	NA	NA	1
73	0.616438356	NA	NA	1
121	0.590361446	NA	NA	1
74	0.581081081	NA	NA	1
73	0.602739726	NA	NA	1
316	0.46835443	NA	NA	1
299	0.558528428	NA	NA	1
2932	0.371407517	NA	NA	1
164	0.536585366	NA	NA	1
107	0.439252336	NA	NA	1
299	0.531772575	NA	NA	1
1199	0.413612565	NA	NA	1
68	0.382352941	NA	NA	1
81	0.419753086	NA	NA	1
81	0.432098765	NA	NA	1
126	0.396825397	NA	NA	1
191	0.486910995	NA	NA	1
142	0.478873239	NA	NA	1
127	0.377952756	NA	NA	1
127	0.362204724	NA	NA	1
192	0.484375	NA	NA	1
140	0.535714286	NA	NA	1

4226	0.350910834	NA	NA	1
69	0.376811594	NA	NA	1
87	0.367816092	NA	NA	1
73	0.287671233	NA	NA	1
86	0.360465116	NA	NA	1
73	0.287671233	NA	NA	1
69	0.391304348	NA	NA	1
77	0.324675325	NA	NA	1
88	0.340909091	NA	NA	1
87	0.344827586	NA	NA	1
76	0.342105263	NA	NA	1
67	0.358208955	NA	NA	1
67	0.373134328	NA	NA	1
67	0.373134328	NA	NA	1
72	0.347222222	NA	NA	1
77	0.324675325	NA	NA	1
67	0.402985075	NA	NA	1
73	0.328767123	NA	NA	1
78	0.294871795	NA	NA	1
123	0.398373984	NA	NA	1
84	0.345238095	NA	NA	1
80	0.375	NA	NA	1
192	0.489583333	NA	NA	1
110	0.363636364	NA	NA	1
227	0.463855422	NA	NA	1
240	0.417910448	NA	NA	1
97	0.474226804	NA	NA	1
148	0.439189189	NA	NA	1
131	0.41221374	NA	NA	1
421	0.48456057	NA	NA	1
580	0.447418738	NA	NA	1
702	0.413153457	NA	NA	1
326	0.438095238	NA	NA	1
135	0.540740741	NA	NA	1
135	0.540740741	NA	NA	1
135	0.540740741	NA	NA	1
143	0.496503497	NA	NA	1
135	0.548148148	NA	NA	1
135	0.548148148	NA	NA	1
135	0.548148148	NA	NA	1
135	0.548148148	NA	NA	1
135	0.540740741	NA	NA	1
135	0.548148148	NA	NA	1
144	0.548611111	NA	NA	1
135	0.533333333	NA	NA	1
135	0.533333333	NA	NA	1
135	0.548148148	NA	NA	1
135	0.533333333	NA	NA	1
135	0.548148148	NA	NA	1
135	0.540740741	NA	NA	1
135	0.548148148	NA	NA	1
137	0.459854015	NA	NA	1
135	0.540740741	NA	NA	1
135	0.548148148	NA	NA	1
135	0.548148148	NA	NA	1
135	0.548148148	NA	NA	1
135	0.548148148	NA	NA	1

135	0.548148148	NA	NA	1
135	0.533333333	NA	NA	1
135	0.548148148	NA	NA	1
135	0.548148148	NA	NA	1
135	0.548148148	NA	NA	1
135	0.548148148	NA	NA	1
135	0.548148148	NA	NA	1
135	0.548148148	NA	NA	1
135	0.548148148	NA	NA	1
135	0.548148148	NA	NA	1
135	0.548148148	NA	NA	1
135	0.548148148	NA	NA	1
135	0.540740741	NA	NA	1
135	0.540740741	NA	NA	1
134	0.544776119	NA	NA	1
135	0.548148148	NA	NA	1
135	0.548148148	NA	NA	1
135	0.548148148	NA	NA	1
135	0.548148148	NA	NA	1
135	0.548148148	NA	NA	1
135	0.548148148	NA	NA	1
135	0.533333333	NA	NA	1
135	0.540740741	NA	NA	1
135	0.548148148	NA	NA	1
135	0.548148148	NA	NA	1
135	0.548148148	NA	NA	1
135	0.548148148	NA	NA	1
135	0.548148148	NA	NA	1
134	0.544776119	NA	NA	1
135	0.540740741	NA	NA	1
135	0.540740741	NA	NA	1
135	0.540740741	NA	NA	1
135	0.540740741	NA	NA	1
135	0.540740741	NA	NA	1
135	0.540740741	NA	NA	1
135	0.540740741	NA	NA	1
135	0.548148148	NA	NA	1
135	0.548148148	NA	NA	1
135	0.548148148	NA	NA	1
143	0.566433566	NA	NA	1
135	0.548148148	NA	NA	1
135	0.533333333	NA	NA	1
135	0.548148148	NA	NA	1
135	0.548148148	NA	NA	1
135	0.548148148	NA	NA	1
135	0.540740741	NA	NA	1
135	0.540740741	NA	NA	1
135	0.548148148	NA	NA	1
135	0.548148148	NA	NA	1
135	0.548148148	NA	NA	1
135	0.540740741	NA	NA	1
135	0.540740741	NA	NA	1
135	0.548148148	NA	NA	1
135	0.548148148	NA	NA	1
135	0.548148148	NA	NA	1
135	0.533333333	NA	NA	1
135	0.548148148	NA	NA	1

135	0.548148148	NA	NA	1
135	0.548148148	NA	NA	1
135	0.548148148	NA	NA	1
135	0.548148148	NA	NA	1
135	0.540740741	NA	NA	1
135	0.548148148	NA	NA	1
135	0.540740741	NA	NA	1
135	0.540740741	NA	NA	1
135	0.548148148	NA	NA	1
135	0.540740741	NA	NA	1
135	0.533333333	NA	NA	1
135	0.548148148	NA	NA	1
135	0.548148148	NA	NA	1
135	0.540740741	NA	NA	1
135	0.540740741	NA	NA	1
135	0.533333333	NA	NA	1
135	0.540740741	NA	NA	1
135	0.548148148	NA	NA	1
135	0.533333333	NA	NA	1
1226	0.402120718	NA	NA	1
71	0.366197183	NA	NA	1
73	0.589041096	NA	NA	1
72	0.541666667	NA	NA	1
84	0.607142857	NA	NA	1
74	0.635135135	NA	NA	1
73	0.602739726	NA	NA	1
72	0.611111111	NA	NA	1
73	0.575342466	NA	NA	1
140	0.485714286	NA	NA	1
173	0.514450867	NA	NA	1
629	0.382022472	NA	NA	1
146	0.445205479	NA	NA	1
68	0.588235294	NA	NA	1
140	0.4	NA	NA	1
148	0.412162162	NA	NA	1
74	0.554054054	NA	NA	1
140	0.428571429	NA	NA	1
137	0.510948905	NA	NA	1
140	0.45	NA	NA	1
35	0.485714286	NA	NA	1
77	0.649350649	NA	NA	1
64	0.5	NA	NA	1
145	0.427586207	NA	NA	1
168	0.553571429	NA	NA	1
67	0.582089552	NA	NA	1
72	0.541666667	NA	NA	1
143	0.482517483	NA	NA	1
140	0.557142857	NA	NA	1
59	0.508474576	NA	NA	1
268	0.537313433	NA	NA	1
162	0.518518519	NA	NA	1
52	0.596153846	NA	NA	1
315	0.415873016	NA	NA	1
31	0.612903226	NA	NA	1
43	0.558139535	NA	NA	1
143	0.454545455	NA	NA	1
171	0.589473684	NA	NA	1

34	0.558823529	NA	NA	1
111	0.576576577	NA	NA	1
49	0.489795918	NA	NA	1
36	0.527777778	NA	NA	1
36	0.527777778	NA	NA	1
36	0.527777778	NA	NA	1
36	0.527777778	NA	NA	1
36	0.527777778	NA	NA	1
36	0.527777778	NA	NA	1
36	0.527777778	NA	NA	1
36	0.527777778	NA	NA	1
36	0.527777778	NA	NA	1
36	0.527777778	NA	NA	1
36	0.527777778	NA	NA	1
36	0.527777778	NA	NA	1
36	0.527777778	NA	NA	1
36	0.527777778	NA	NA	1
36	0.527777778	NA	NA	1
36	0.527777778	NA	NA	1
36	0.527777778	NA	NA	1
36	0.527777778	NA	NA	1
36	0.527777778	NA	NA	1
36	0.527777778	NA	NA	1
157	0.560509554	NA	NA	1
139	0.460431655	NA	NA	1
141	0.524822695	NA	NA	1
140	0.45	NA	NA	1
220	0.572727273	NA	NA	1
144	0.486111111	NA	NA	1
144	0.548611111	NA	NA	1
46	0.47826087	NA	NA	1
96	0.572916667	NA	NA	1
164	0.365853659	NA	NA	1
192	0.484375	NA	NA	1
46	0.565217391	NA	NA	1
73	0.547945205	NA	NA	1
78	0.5	NA	NA	1
143	0.517482517	NA	NA	1
409	0.413202934	NA	NA	1
533	0.452157598	NA	NA	1
409	0.415647922	NA	NA	1
1999	0.482889734	NA	NA	1
154	0.454545455	NA	NA	1
63	0.571428571	NA	NA	1
35	0.6	NA	NA	1
74	0.567567568	NA	NA	1
145	0.510344828	NA	NA	1
173	0.514450867	NA	NA	1
55	0.527272727	NA	NA	1
613	0.461988304	NA	NA	1
3820	0.426229508	NA	NA	1
1643	0.516129032	NA	NA	1
562	0.378968254	NA	NA	1
375	0.450479233	NA	NA	1
1904	0.3798627	NA	NA	1
297	0.393939394	NA	NA	1
74	0.635135135	NA	NA	1

74	0.635135135	NA	NA	1
939	0.478168264	NA	NA	1
2165	0.549054374	NA	NA	1
123	0.512195122	NA	NA	1
590	0.450847458	NA	NA	1
445	0.274157303	NA	NA	1
1462	0.299589603	NA	NA	1
1077	0.26369545	NA	NA	1
1092	0.282051282	NA	NA	1
1033	0.271055179	NA	NA	1
1944	0.357510288	NA	NA	1
2391	0.335006274	1000	1000	1
1697	0.290512669	NA	NA	1
1049	0.28122021	NA	NA	1
967	0.279214064	NA	NA	1
1172	0.307167235	NA	NA	1
1110	0.321621622	NA	NA	1
1045	0.30430622	NA	NA	1
1278	0.283255086	NA	NA	1
824	0.300970874	NA	NA	1
265	0.320754717	NA	NA	1
1001	0.410589411	NA	NA	1
1108	0.306859206	NA	NA	1
1088	0.262867647	NA	NA	1
834	0.29736211	NA	NA	1
1134	0.421516755	NA	NA	1
549	0.406193078	NA	NA	1
569	0.409490334	NA	NA	1
559	0.413237925	NA	NA	1
566	0.408127208	NA	NA	1
740	0.42027027	NA	NA	1
931	0.310418904	NA	NA	1
1012	0.334980237	NA	NA	1
1124	0.282918149	NA	NA	1
506	0.440711462	NA	NA	1
1032	0.390503876	NA	NA	1
533	0.277673546	NA	NA	1
561	0.349376114	NA	NA	1
530	0.290566038	NA	NA	1
838	0.411694511	NA	NA	1
1211	0.426919901	NA	NA	1
765	0.445751634	NA	NA	1
208	0.379807692	NA	NA	1
200	0.385	NA	NA	1
345	0.327536232	NA	NA	1
22711	0.330347734	NA	NA	1
668	0.404191617	NA	NA	1
618	0.411003236	NA	NA	1
1279	0.398749023	NA	NA	1
540	0.398148148	NA	NA	1
1052	0.269961977	NA	NA	1
1030	0.295145631	NA	NA	1
252	0.464285714	NA	NA	1
1975	0.29721519	NA	NA	1
1994	0.387662989	NA	NA	1
1942	0.277548919	NA	NA	1
759	0.36970684	NA	NA	1

6026	0.373713906	1000	1000	1
6256	0.351840491	NA	NA	0
1460	0.428082192	NA	NA	1
2099	0.316817532	1000	1000	1
1266	0.417851501	NA	NA	1
912	0.305921053	NA	NA	1
951	0.400630915	NA	NA	1
575	0.410434783	NA	NA	1
507	0.437869822	NA	NA	1
1015	0.298522167	NA	NA	1
1005	0.336318408	NA	NA	1
524	0.291984733	NA	NA	1
462	0.354978355	NA	NA	1
752	0.409574468	NA	NA	1
2159	0.582733813	NA	NA	1
1229	0.437299035	NA	NA	1
35853	0.533674514	NA	NA	0
22832	0.467858923	1000	1000	0
26214	0.543659044	1000	1000	1
1831	0.490417136	NA	NA	1
917	0.425299891	NA	NA	1
31277	0.496190365	416	1000	0
27173	0.520840123	NA	NA	1
2413	0.498053528	NA	NA	1
2105	0.530860349	NA	NA	0
2357	0.53164557	938	1000	1
2545	0.50047619	718	837	1
7523	0.538858049	427	1000	0
2669	0.529712069	NA	NA	0
1422	0.486430063	NA	NA	0
2075	0.592668024	NA	NA	0
20629	0.443359375	1000	1000	0
1446	0.570182395	NA	NA	1
48575	0.490577578	85	1000	0
994	0.479746835	NA	NA	1
106953	0.516417244	NA	NA	0
1242	0.503990878	NA	NA	1
17166	0.54416511	320	1000	0
29565	0.496916153	NA	NA	1
2919	0.483525535	581	1000	1
2677	0.436309301	1000	1000	1
3289	0.514775161	NA	NA	0
3671	0.469505594	608	1000	0
813	0.558359621	NA	NA	1
3807	0.489431487	NA	NA	1
2777	0.542280285	946	936	0
20334	0.548359364	738	1000	1
35289	0.528825863	1000	1000	0
3636	0.495087901	463	1000	1
5175	0.557181248	799	920	1
3452	0.401962876	NA	NA	0
72535	0.512550289	NA	NA	0
1558	0.512944984	NA	NA	0
6595	0.51953125	609	772	0
12439	0.481346105	NA	NA	1
89536	0.53055755	NA	NA	0
530	0.449056604	NA	NA	1

1448	0.522099448	NA	NA		1
6831	0.507142857	625	1000		0
2978	0.416540899	462	1000		0
5315	0.480368727	NA	NA		1
10689	0.547642929	NA	NA		0
37062	0.509210526	NA	NA		0
4775	0.48601428	1000	1000		1
3141	0.390890637	NA	NA		0
12400	0.524876645	1000	1000		0
17797	0.378829573	200	1000		0
30203	0.538241945	266	867		0
3383	0.518506701	942	1000		0
8129	0.42086804	658	672		0
16979	0.429161067	317	1000		1
1603	0.502962963	NA	NA		1
6452	0.446391125	NA	NA		0
14765	0.545069394	882	867		0
9171	0.507089577	903	1000		0
10171	0.4744552	NA	NA		0
8911	0.43817107	525	1000		0
3561	0.457608696	NA	NA		0
12886	0.485154682	181	863		1
1148	0.5125	NA	NA		1
7489	0.4990591	325	805		0
19526	0.543424049	524	924		0
1999	0.51370614	NA	NA		1
1825	0.517477724	NA	NA		1
3492	0.487945998	768	916		1
586	0.557281553	NA	NA		1
656	0.567049808	NA	NA		1
823	0.585516179	NA	NA		1
740	0.588325653	NA	NA		1
1475	0.608216857	NA	NA		1
98949	0.460442504	NA	NA		0
2389	0.541398541	942	1000		1
10826	0.515317559	NA	NA		1
3089	0.460242233	NA	NA		1
24448	0.510053155	958	1000		1
6878	0.458196046	NA	NA		0
3465	0.498045944	907	1000		0
1853	0.525991792	NA	NA		1
2572	0.500588928	NA	NA		0
12290	0.530289026	809	1000		0
3821	0.496536478	NA	NA		0
430	0.493023256	NA	NA		1
6024	0.525127263	NA	NA		1
1526	0.51774308	NA	NA		0
33081	0.427461914	NA	NA		0
11559	0.516727494	308	931		0
1930	0.513471503	NA	NA		1
6149	0.510699001	496	1000		0
23483	0.472560293	732	1000		1
124993	0.504033759	NA	NA		0
1743	0.550179211	NA	NA		1
1785	0.517128874	NA	NA		1
162886	0.530107726	NA	NA		0
3397	0.555098163	NA	NA		1

31452	0.504533679		679	1000	0
2844	0.51371308		1000	1000	1
569	0.444639719	NA	NA		1
7340	0.541839007		284	817	1
54513	0.459450568		338	1000	1
7292	0.43664979		1000	740	0
12801	0.440436481		915	608	1
45975	0.540794259	NA	NA		0
2231	0.56858202	NA	NA		1
78758	0.450401869	NA	NA		0
3429	0.523252983	NA	NA		0
11922	0.51017179		626	935	1
2150	0.489780469	NA	NA		0
1386	0.52958153	NA	NA		1
59150	0.494279341	NA	NA		0
113214	0.530916971	NA	NA		0
20985	0.525019894	NA	NA		0
4656	0.533733133	NA	NA		0
2174	0.520286396	NA	NA		1
664	0.543674699	NA	NA		1
1871	0.462637363	NA	NA		1
1796	0.442528736	NA	NA		1
3456	0.479242069		701	931	0
1174	0.545945946	NA	NA		1
478	0.491631799	NA	NA		1
3556	0.498924311		873	932	0
126280	0.469160768	NA	NA		1
14546	0.471312342		365	1000	1
73341	0.480449956	NA	NA		1
976	0.498908297	NA	NA		1
1019	0.513023783	NA	NA		1
3549	0.531135531		1000	1000	1
4030	0.514911081		1000	1000	1
4926	0.478958741		758	1000	1
17556	0.540809664	NA	NA		0
4167	0.53006993	NA	NA		1
34881	0.507569131		244	938	0
2190	0.524522457	NA	NA		1
3179	0.507706826		1000	1000	1
16232	0.473044768	NA	NA		0
4342	0.512861736	NA	NA		1
50703	0.447946639		1000	1000	0
10996	0.532900834		164	839	0
9075	0.48724043		337	1000	1
3218	0.527067995	NA	NA		1
3160	0.574580095	NA	NA		0
1517	0.511063526	NA	NA		0
7145	0.547175918	NA	NA		0
1393	0.420725389	NA	NA		1
7304	0.484585742	NA	NA		1
13639	0.490162237		337	1000	1
4769	0.533826248		274	1000	0
4396	0.424860055	NA	NA		0
3277	0.509201472	NA	NA		0
5411	0.484695854		886	907	0
2151	0.541600424	NA	NA		1
2513	0.46798603	NA	NA		1

34093	0.473524363		244	1000	1
8202	0.532252836	NA	NA		0
2413	0.493040177		1000	1000	1
2665	0.523867809	NA	NA		1
1719	0.495180112	NA	NA		0
653	0.466456196	NA	NA		0
2843	0.505032564	NA	NA		0
2174	0.44792176		1000	939	1
2517	0.504568931		1000	1000	1
1576	0.541243655	NA	NA		1
3518	0.52443385		345	934	1
17823	0.526315789	NA	NA		0
2632	0.534466912		544	1000	1
3967	0.550103416		278	1000	0
4329	0.47862946		450	755	1
2034	0.452054795	NA	NA		0
880	0.489275362	NA	NA		1
1218	0.486863711	NA	NA		1
1733	0.522215811	NA	NA		1
2243	0.430177367	NA	NA		0
973	0.473792395	NA	NA		1
4033	0.505168849	NA	NA		1
14580	0.348610335		1000	1000	0
128818	0.41726004		41	1000	0
4224	0.484293194	NA	NA		1
5126	0.52471459		226	1000	0
31246	0.509770417		143	1000	0
1704	0.512910798	NA	NA		1
2236	0.468233977	NA	NA		0
1243	0.507998877	NA	NA		0
3202	0.543165468	NA	NA		1
3060	0.531699346		1000	1000	1
3107	0.533506269	NA	NA		1
727	0.502063274	NA	NA		1
921	0.461048506	NA	NA		0
2077	0.502109705	NA	NA		0
7182	0.568156549		159	908	1
27342	0.496864252	NA	NA		0
5019	0.52966443		640	929	1
14773	0.539570961	NA	NA		1
67041	0.364337349		1000	943	1
106332	0.526385928	NA	NA		0
34415	0.480851964		212	1000	0
3297	0.462373643		636	946	1
8331	0.558068266		939	1000	0
16102	0.517254601		580	867	1
2423	0.505687204	NA	NA		1
8086	0.471634208	NA	NA		1
39651	0.473210594		163	1000	0
2533	0.481767956	NA	NA		1
4035	0.507938295		312	917	0
1808	0.561419201	NA	NA		0
802	0.57605985	NA	NA		1
2194	0.556517776	NA	NA		1
1824	0.471986418	NA	NA		1
1454	0.462801932	NA	NA		1
1791	0.498604132	NA	NA		1

29172	0.523860965	NA	NA		0
1827	0.524602768	NA	NA		0
2850	0.51571355	NA	NA		1
11534	0.395539415	307		1000	0
2165	0.597600872	NA	NA		0
4763	0.52810833	741		461	1
1658	0.479426892	NA	NA		0
20874	0.527436309	212		824	1
10989	0.480546677	NA	NA		1
14438	0.489528065	NA	NA		1
17164	0.51410227	1000		899	0
4099	0.494079872	948		1000	0
40364	0.520638504	546		1000	1
3707	0.544383562	NA	NA		1
1095	0.438356164	NA	NA		1
2563	0.488791033	935		1000	1
2304	0.544704861	1000		1000	1
18989	0.555437262	88		1000	1
15236	0.482232012	NA	NA		1
1764	0.44280287	NA	NA		1
957	0.513061651	NA	NA		1
1413	0.470629866	NA	NA		1
1166	0.544850498	NA	NA		1
930	0.537249754	NA	NA		0
733	0.487039563	NA	NA		1
882	0.5	NA	NA		1
1137	0.419525066	NA	NA		1
982	0.476578411	NA	NA		1
2512	0.529534606	912		1000	0
1600	0.4275	NA	NA		0
4572	0.508596783	390		911	1
7012	0.483231707	NA	NA		0
2466	0.489136817	NA	NA		1
9443	0.519044321	410		1000	0
3823	0.484816196	1000		1000	1
676	0.48964497	NA	NA		1
978	0.538854806	NA	NA		1
1013	0.440276407	NA	NA		1
911	0.503841932	NA	NA		1
6553	0.478860962	40		912	0
2903	0.440310808	679		934	0
25791	0.546160117	NA	NA		1
4781	0.540380658	NA	NA		1
711	0.488045007	NA	NA		1
1351	0.506545821	NA	NA		1
560	0.461702128	NA	NA		1
305	0.382716049	NA	NA		1
1319	0.561031084	NA	NA		1
4780	0.484496124	NA	NA		1
10753	0.480458435	798		1000	1
4825	0.52373057	1000		1000	1
4201	0.481238833	NA	NA		1
3051	0.477585598	921		861	1
6777	0.49953271	NA	NA		1
4341	0.477269897	NA	NA		1
10808	0.537385938	342		1000	1
26587	0.513141283	116		1000	0

5544	0.460353866	NA	NA		1
4927	0.399084668	363	1000		1
19122	0.46731697	NA	NA		0
1979	0.471125612	NA	NA		1
2963	0.455475075	NA	NA		1
34745	0.493859194	NA	NA		0
3168	0.507291667	NA	NA		1
1002	0.457317073	NA	NA		1
30520	0.560270515	382	1000		1
3265	0.452164804	752	1000		1
89550	0.495216889	NA	NA		1
1587	0.5463138	NA	NA		1
22633	0.514284644	1000	1000		0
6158	0.467786892	NA	NA		0
15843	0.476965249	530	894		0
4014	0.522828072	1000	933		1
731	0.566347469	NA	NA		1
564	0.569148936	NA	NA		1
1824	0.59245283	NA	NA		1
1463	0.554982818	NA	NA		1
7718	0.533496482	633	921		0
3746	0.500932702	1000	1000		1
1828	0.528269063	NA	NA		0
1058	0.375536481	NA	NA		1
39070	0.478042448	NA	NA		1
18239	0.457145019	732	820		0
7861	0.537267081	191	972		1
762	0.456221198	NA	NA		1
1823	0.488888889	NA	NA		1
15620	0.494148625	NA	NA		1
1961	0.47410148	NA	NA		1
19667	0.546584026	413	874		1
132757	0.513596716	NA	NA		0
1749	0.456561922	NA	NA		1
12490	0.53244787	385	1000		0
21344	0.502140241	385	1000		0
7298	0.471905365	NA	NA		0
170118	0.513334087	NA	NA		0
5370	0.469176818	500	940		1
65567	0.551552232	NA	NA		0
10398	0.477367089	469	871		0
4301	0.524169867	NA	NA		1
2527	0.447927199	NA	NA		1
2321	0.439431397	NA	NA		1
30781	0.499450751	276	864		0
5002	0.496083271	973	1000		1
13980	0.557809855	119	74		0
1597	0.492307692	NA	NA		1
18527	0.49739746	NA	NA		0
8154	0.521072403	715	935		0
639	0.55088853	NA	NA		0
632	0.528070175	NA	NA		1
3665	0.475451467	938	1000		1
3646	0.503828484	1000	941		1
16810	0.519466073	NA	NA		1
2881	0.552988855	961	1000		1
38446	0.531212048	328	1000		1

2048	0.50391353	NA	NA		0
2243	0.43592233	NA	NA		0
3590	0.494337635		701	938	0
14341	0.459146341	NA	NA		1
4249	0.490163516	NA	NA		1
26862	0.47459087		437	1000	0
23310	0.461637766	NA	NA		0
858	0.607226107	NA	NA		1
960	0.512735327	NA	NA		1
43897	0.478352184		392	746	0
751	0.426100629	NA	NA		1
428	0.512534819	NA	NA		1
134199	0.437187428	NA	NA		0
11814	0.51521835		199	1000	0
9401	0.515239679		299	909	0
7748	0.453357781		473	924	0
14137	0.455397025		738	479	0
1819	0.500889152	NA	NA		1
33297	0.506167345	NA	NA		1
11171	0.475035303		645	1000	0
49448	0.553699361	NA	NA		0
48695	0.485069827	NA	NA		0
111236	0.41289404	NA	NA		0
22062	0.510239362	NA	NA		0
1255	0.506024096	NA	NA		1
2606	0.53718717	NA	NA		0
6414	0.483090459		309	1000	1
1458	0.522678186	NA	NA		0
7583	0.583135665		521	860	0
37168	0.519265692	NA	NA		0
1350	0.52152381	NA	NA		0
1545	0.496717724	NA	NA		1
1405	0.440429688	NA	NA		1
2495	0.414829659	NA	NA		1
4999	0.457736079		925	482	1
42947	0.497028442	NA	NA		0
33526	0.4858924	NA	NA		0
41569	0.454674284	NA	NA		0
17881	0.525841053		587	1000	0
29265	0.518302494		295	1000	0
2103	0.49122807	NA	NA		1
2809	0.530536131		600	807	1
1617	0.590732436	NA	NA		0
7937	0.492328946		373	1000	0
1528	0.505076142	NA	NA		1
11055	0.49010261		111	873	0
4133	0.477551804	NA	NA		0
5684	0.509476631	NA	NA		1
1508	0.47591623	NA	NA		0
5877	0.506515421		865	825	0
33264	0.530496635	NA	NA		0
44039	0.506996453	NA	NA		0
2292	0.514087988		819	912	1
11417	0.518856966	NA	NA		0
22389	0.414722227	NA	NA		0
2975	0.449113924	NA	NA		1
1664	0.502877379	NA	NA		1

13386	0.502841119		439	1000	0
3778	0.504617218		739	1000	1
26705	0.490878175		326	597	0
6363	0.485425532	NA	NA		0
992	0.503550678	NA	NA		1
2910	0.503299604		691	943	1
1872	0.538255034	NA	NA		1
881	0.538024972	NA	NA		1
21524	0.489366948		401	1000	0
2569	0.473793103		836	1000	0
1977	0.539706626	NA	NA		1
2045	0.547936508	NA	NA		1
1900	0.519282998	NA	NA		1
21360	0.561886051	NA	NA		1
164	0.536585366	NA	NA		1
78004	0.435018359		1000	1000	1
1331	0.488906498	NA	NA		1
49705	0.54369139		503	1000	0
2513	0.52884998	NA	NA		1
15194	0.487829874		1000	713	1
1415	0.555130168	NA	NA		1
641	0.5975039	NA	NA		1
625	0.6384	NA	NA		1
32327	0.47625805		1000	784	1
15124	0.546652229		379	1000	0
11270	0.436642661	NA	NA		0
1647	0.568789809	NA	NA		1
1623	0.572867696	NA	NA		1
6116	0.490020728		935	903	1
2782	0.544326596	NA	NA		0
453	0.430463576	NA	NA		1
1331	0.505919495	NA	NA		1
1009	0.417064123	NA	NA		0
1316	0.509369676	NA	NA		1
681	0.484922575	NA	NA		0
33610	0.514669052	NA	NA		0
20519	0.439231547		584	1000	1
2297	0.472991375		1000	906	1
1919	0.484644397	NA	NA		1
11392	0.497235533	NA	NA		0
5006	0.482668804		107	671	0
5887	0.424319296		663	499	1
863	0.524964337	NA	NA		1
6589	0.531619064	NA	NA		0
844	0.523696682	NA	NA		1
7887	0.422467804		422	1000	0
31291	0.521596712		885	682	0
9030	0.517806894		86	656	0
1438	0.431849791	NA	NA		1
1066	0.531894934	NA	NA		1
421	0.461325967	NA	NA		1
6485	0.443874011		527	1000	0
164	0.524390244	NA	NA		1
69112	0.467708513		391	1000	0
2169	0.397648686	NA	NA		0
3674	0.516492045		309	802	1
496	0.461693548	NA	NA		1

36310	0.427177914	101	1000	0
9020	0.537773804	1000	718	1
3395	0.446248004	NA	NA	1
3642	0.476682005	1000	942	0
16294	0.527952066	196	654	0
109440	0.476537492	760	1000	0
55311	0.495325565	330	1000	1
13942	0.561804009	NA	NA	1
4368	0.482032854	NA	NA	1
11366	0.498764528	815	1000	1
7630	0.404934326	302	1000	1
17801	0.438096483	1000	1000	1
58345	0.509622143	630	1000	0
6935	0.546623794	189	1000	1
3814	0.546486486	1000	941	1
1686	0.50245098	NA	NA	1
377	0.527851459	NA	NA	1
2847	0.423657212	NA	NA	0
486	0.574074074	NA	NA	1
480	0.575	NA	NA	1
580	0.543103448	NA	NA	1
615	0.517073171	NA	NA	1
492	0.577235772	NA	NA	1
564	0.487588652	NA	NA	1
10027	0.37639053	318	1000	0
3930	0.413570007	280	1000	0
114856	0.52394247	NA	NA	1
44082	0.505126296	NA	NA	0
17592	0.495846754	NA	NA	0
1536	0.526950355	NA	NA	1
125689	0.507433642	NA	NA	1
2112	0.493541442	NA	NA	1
6023	0.545153905	NA	NA	0
30279	0.495718363	563	1000	1
604	0.486940299	NA	NA	1
656	0.540825285	NA	NA	0
2315	0.472512494	920	1000	1
10299	0.463945756	NA	NA	1
2638	0.501959167	1000	1000	1
31754	0.469929347	NA	NA	1
13421	0.514126624	NA	NA	0
17475	0.484763066	NA	NA	1
2904	0.497479933	1000	938	1
5592	0.513676981	484	711	0
542	0.421052632	NA	NA	1
606	0.385730212	NA	NA	1
776	0.456964006	NA	NA	1
553	0.421568627	NA	NA	1
1539	0.476446837	NA	NA	1
747	0.431554524	NA	NA	1
531	0.417061611	NA	NA	1
77159	0.459888563	NA	NA	0
1190	0.468141593	NA	NA	1
8696	0.52101291	302	957	0
22558	0.542240735	455	1000	0
38540	0.423357664	42	1000	1
4812	0.5131209	353	1000	1

3921	0.490925869	NA	NA		1
18652	0.526416406	465		924	1
17687	0.353932363	206		1000	0
42255	0.460552982	NA	NA		0
4684	0.450196078	339		1000	1
3142	0.411723039	747		1000	1
17704	0.477974398	NA	NA		0
37391	0.519395899	956		1000	1
12610	0.472697595	919		1000	0
6914	0.521540569	942		1000	1
128994	0.555462739	474		708	0
21494	0.524709502	227		1000	0
3423	0.438318128	NA	NA		0
6357	0.488913304	935		883	0
1312	0.510483136	NA	NA		1
638	0.581034483	NA	NA		1
872	0.586206897	NA	NA		1
786	0.609655172	NA	NA		1
1124	0.5	NA	NA		1
2948	0.494634146	504		852	1
7986	0.456498739	469		1000	0
13637	0.459367562	1000		1000	0
38675	0.507763245	NA	NA		1
770	0.503865074	NA	NA		1
2285	0.563236047	NA	NA		1
18923	0.494283995	1000		1000	1
9332	0.391141443	1000		1000	0
6266	0.556423983	150		937	0
10646	0.529300078	839		1000	0
39895	0.485375588	NA	NA		0
1278	0.582710779	NA	NA		1
3680	0.504285714	634		1000	1
22522	0.53760256	NA	NA		0
49473	0.479685983	666		1000	1
16208	0.470238971	419		1000	0
4194	0.54637315	NA	NA		1
12924	0.491292591	470		1000	0
2383	0.502183406	NA	NA		0
2182	0.526741146	923		1000	1
3614	0.475438968	722		1000	1
773	0.460365854	NA	NA		1
25645	0.352805755	NA	NA		0
3456	0.504788384	934		942	1
21758	0.502503021	NA	NA		0
11694	0.449746193	NA	NA		1
1710	0.533460803	NA	NA		0
8538	0.471212385	439		1000	0
1480	0.559695174	NA	NA		1
1645	0.517636684	NA	NA		1
3789	0.532757235	334		1000	0
2331	0.557310361	NA	NA		1
7530	0.440686976	177		999	0
1840	0.511956522	NA	NA		1
7983	0.499046993	824		916	1
5947	0.484989278	NA	NA		1
1621	0.573084353	NA	NA		1
16168	0.473833437	567		1000	1

2344	0.512458031	NA	NA		0
11126	0.468646313	450	896		0
30059	0.484564187	420	1000		0
24500	0.375514139	245	1000		0
16143	0.53759252	270	279		1
1948	0.527322404	NA	NA		1
1409	0.48899929	NA	NA		1
1045	0.546979866	NA	NA		1
11822	0.541465131	NA	NA		0
61083	0.512659362	NA	NA		0
13671	0.512871828	NA	NA		0
1629	0.432624113	NA	NA		1
21396	0.50899405	386	708		0
94837	0.450591785	NA	NA		0
3345	0.54536862	1000	889		1
2942	0.431390688	NA	NA		1
3780	0.505038168	NA	NA		1
1104	0.518373346	NA	NA		0
25809	0.497536946	NA	NA		0
11828	0.50367539	594	1000		1
5041	0.582738702	1000	728		0
2616	0.552294557	NA	NA		1
51678	0.434452418	88	1000		1
4068	0.501480883	381	1000		0
9425	0.478523663	NA	NA		0
19473	0.519533711	1000	1000		0
2071	0.49251497	933	1000		1
2048	0.541015625	1000	1000		1
108685	0.453286795	NA	NA		0
1327	0.474001507	NA	NA		1
7572	0.576343073	657	690		1
24145	0.466201117	859	807		0
11278	0.476834128	377	1000		0
1951	0.447647308	NA	NA		0
7446	0.508619572	NA	NA		1
2277	0.483091787	1000	1000		1
734	0.508174387	NA	NA		1
746	0.463806971	NA	NA		1
723	0.479944675	NA	NA		1
736	0.438858696	NA	NA		1
717	0.513249651	NA	NA		1
793	0.493064313	NA	NA		1
2968	0.514968553	512	763		0
1676	0.50729443	NA	NA		1
1314	0.547794118	NA	NA		1
1309	0.530041642	NA	NA		0
1755	0.527320606	NA	NA		1
4402	0.532708628	189	939		1
6227	0.512208657	NA	NA		1
2330	0.475386779	882	921		1
3848	0.503118503	1000	1000		1
10075	0.481328979	NA	NA		1
1882	0.472807018	NA	NA		0
1065	0.519494204	NA	NA		1
2736	0.474189676	NA	NA		1
1947	0.501213592	NA	NA		1
5913	0.509374819	NA	NA		0

3441	0.48724905	NA		NA	1
3474	0.464139344	NA		NA	1
91611	0.344059406		190	1000	1
6296	0.409637395		53	1000	1
1839	0.524092409	NA		NA	1
13673	0.453035468	NA		NA	0
911	0.517045455	NA		NA	0
2696	0.530850472	NA		NA	1
8146	0.496653815		492	1000	0
2146	0.530832477	NA		NA	1
1442	0.49076639	NA		NA	0
1762	0.507255937	NA		NA	1
44167	0.505331656		471	228	0
3903	0.515072686		864	802	1
8506	0.504283782		1000	1000	1
1215	0.415573053	NA		NA	1
2854	0.436950147		1000	916	0
3779	0.439106616		941	1000	1
1685	0.467248908	NA		NA	1
11271	0.524524076		902	906	0
7444	0.546712803		675	937	0
1546	0.512345679	NA		NA	1
1119	0.474812433	NA		NA	1
2855	0.452601411	NA		NA	0
1919	0.506199461	NA		NA	1
3853	0.410755368	NA		NA	0
18366	0.524879168	NA		NA	1
12576	0.530030772		137	699	0
9160	0.525743314		815	873	1
2359	0.515048749		1000	1000	1
798	0.596491228	NA		NA	1
809	0.593325093	NA		NA	1
374	0.483957219	NA		NA	1
20394	0.478834499		323	1000	0
399	0.488721805	NA		NA	1
1599	0.516169154	NA		NA	0
758	0.435094002	NA		NA	1
866	0.478125	NA		NA	1
2849	0.439367311	NA		NA	1
1653	0.560319462	NA		NA	0
877	0.530546624	NA		NA	0
1042	0.532633501	NA		NA	0
88570	0.437452836		365	1000	0
2296	0.548371415		958	880	1
1325	0.454761905	NA		NA	1
1835	0.481062747	NA		NA	1
1257	0.518151815	NA		NA	1
2229	0.485416667	NA		NA	1
655	0.471755725	NA		NA	1
14687	0.526582779		280	716	1
8343	0.50042505		271	1000	0
3805	0.489146595		558	1000	1
10762	0.474654378	NA		NA	0
38087	0.554847141		106	1000	0
19544	0.457995396		606	874	1
9704	0.509606987	NA		NA	0
816	0.485449735	NA		NA	1

991	0.523321957	NA	NA		1
10284	0.510421286	916		725	0
4743	0.498666667	703		936	1
2784	0.518796125	NA	NA		0
78197	0.552499072	NA	NA		0
1974	0.518908865	NA	NA		1
1319	0.461054669	NA	NA		1
871	0.479930192	NA	NA		0
2741	0.560622318	NA	NA		0
1183	0.478444632	NA	NA		1
952	0.5030012	NA	NA		1
20686	0.514731369	NA	NA		0
2209	0.510828625	1000		929	1
1664	0.525752508	NA	NA		1
4191	0.494537747	NA	NA		0
3348	0.491955446	943		1000	1
22748	0.532927159	576		1000	0
2783	0.543204868	934		802	1
2128	0.425112332	936		1000	1
49048	0.533512752	NA	NA		0
3045	0.498805053	NA	NA		1
1085	0.549308756	NA	NA		1
9390	0.494469255	517		1000	0
14013	0.542268786	NA	NA		1
7483	0.49493838	NA	NA		0
28712	0.456942356	456		1000	0
8088	0.476444876	NA	NA		1
3125	0.560348584	374		888	1
8507	0.43433212	NA	NA		0
27140	0.388852204	215		81	0
142297	0.476231431	NA	NA		0
18406	0.516239778	NA	NA		0
385	0.435736677	NA	NA		1
3939	0.503932584	621		1000	1
1230	0.476190476	NA	NA		1
10563	0.43123061	NA	NA		0
2153	0.531816071	1000		1000	1
129268	0.450744816	NA	NA		0
1085	0.547465438	NA	NA		1
1308	0.557226706	NA	NA		0
1292	0.44809322	NA	NA		0
26676	0.489424315	NA	NA		0
1525	0.570728291	NA	NA		1
1233	0.44567474	NA	NA		0
576	0.550347222	NA	NA		1
4117	0.510361318	NA	NA		1
27734	0.555396425	288		1000	1
224977	0.328702981	82		1000	1
1310	0.496954152	NA	NA		0
881	0.478632479	NA	NA		1
890	0.5345953	NA	NA		1
4350	0.560588235	NA	NA		1
10456	0.448388668	927		1000	1
1446	0.465983906	NA	NA		1
1089	0.481981982	NA	NA		1
4507	0.48083021	NA	NA		0
482	0.448931116	NA	NA		1

7102	0.488067881	894	1000	1
1120	0.49795501	NA	NA	1
911	0.497255763	NA	NA	1
1043	0.486097795	NA	NA	1
943	0.538706257	NA	NA	1
2902	0.434400281	NA	NA	1
4675	0.534969325	NA	NA	1
1838	0.524652339	NA	NA	1
63686	0.464091884	NA	NA	1
49432	0.451164197	821	1000	1
3785	0.45161861	177	771	0
2101	0.427991886	NA	NA	0
2391	0.449602677	1000	1000	1
9025	0.543651768	NA	NA	0
1936	0.522960026	NA	NA	0
6946	0.369798689	66	833	0
4141	0.58176	NA	NA	1
45313	0.487934161	685	1000	0
25299	0.51266736	287	1000	0
89641	0.471855984	NA	NA	0
325	0.489230769	NA	NA	1
193	0.440414508	NA	NA	1
375	0.397333333	NA	NA	1
9413	0.469686831	909	860	0
1071	0.485576923	NA	NA	0
410	0.388732394	NA	NA	1
3409	0.526386106	NA	NA	1
2299	0.49786975	NA	NA	1
3413	0.485265226	118	1000	1
1838	0.555059524	NA	NA	1
5679	0.472338582	625	1000	0
1369	0.493161094	NA	NA	1
2497	0.504065041	NA	NA	1
8454	0.49369183	526	1000	1
13364	0.398718154	431	945	0
9283	0.505851128	NA	NA	0
14243	0.408809818	704	1000	0
4085	0.460652591	NA	NA	1
6832	0.407942907	802	1000	0
1475	0.556886228	NA	NA	1
1044	0.475652788	NA	NA	0
973	0.516957862	NA	NA	1
3846	0.487966147	1000	1000	1
2393	0.502793296	NA	NA	1
9601	0.54366782	NA	NA	1
793	0.559539919	NA	NA	1
3479	0.43262204	942	1000	1
71285	0.513408521	NA	NA	1
1304	0.439417178	NA	NA	1
3190	0.506417736	522	1000	1
5213	0.482464891	1000	1000	1
845	0.455621302	NA	NA	1
4271	0.498236776	NA	NA	1
9035	0.514296464	769	945	0
4179	0.50531401	NA	NA	1
824	0.503947368	NA	NA	1
5491	0.464034547	205	882	1

3584	0.528949665		853	1000	1
12525	0.505863103	NA	NA		0
13121	0.505416683	NA	NA		0
2386	0.521793797		1000	1000	1
5776	0.373383621	NA	NA		1
3099	0.443279989		599	942	0
1870	0.551020408	NA	NA		1
1799	0.5112	NA	NA		1
1186	0.523061825	NA	NA		1
1161	0.550432277	NA	NA		1
27025	0.424228313	NA	NA		0
7420	0.490005039		500	1000	0
4144	0.465690209	NA	NA		1
669	0.497553018	NA	NA		1
4974	0.37398374	NA	NA		1
1485	0.496	NA	NA		1
1110	0.547149123	NA	NA		1
34854	0.399006996	NA	NA		0
2925	0.586948321		726	1000	1
79223	0.509024365	NA	NA		0
28159	0.541761658	NA	NA		1
675	0.234074074	NA	NA		1
162	0.166666667	NA	NA		1
1536	0.291015625	NA	NA		1
685	0.261313869	NA	NA		1
789	0.292775665	NA	NA		1
1137	0.259454705	NA	NA		1
939	0.22257721	NA	NA		1
1026	0.171539961	NA	NA		1
354	0.192090395	NA	NA		1
1339	0.197162061	NA	NA		1
291	0.178694158	NA	NA		1
1723	0.211259431	NA	NA		1
525	0.146666667	NA	NA		1
1325	0.170566038	NA	NA		1
786	0.198473282	NA	NA		1
65	0.246153846	NA	NA		1
62	0.306451613	NA	NA		1
67	0.104477612	NA	NA		1
67	0.104477612	NA	NA		1
65	0.276923077	NA	NA		1
65	0.2	NA	NA		1
66	0.166666667	NA	NA		1
65	0.34375	NA	NA		1
71	0.309859155	NA	NA		1
65	0.2	NA	NA		1
66	0.227272727	NA	NA		1
69	0.275362319	NA	NA		1
65	0.246153846	NA	NA		1
61	0.213114754	NA	NA		1
69	0.188405797	NA	NA		1
64	0.3125	NA	NA		1
68	0.235294118	NA	NA		1
66	0.212121212	NA	NA		1
66	0.166666667	NA	NA		1
73	0.205479452	NA	NA		1
66	0.227272727	NA	NA		1

66	0.272727273	NA	NA		1
3331	0.522244489	760	831		1
42378	0.428058055	NA	NA		0
77874	0.530751979	45	1000		0
2707	0.450405653	NA	NA		1
1266	0.514218009	NA	NA		1
36881	0.504646921	160	1000		0
11163	0.492967119	NA	NA		0
863	0.506116208	NA	NA		1
7323	0.502992988	NA	NA		0
2017	0.435336049	NA	NA		1
1839	0.448314607	NA	NA		1
1793	0.513325608	NA	NA		1
11351	0.53419674	565	926		0
13871	0.534447781	938	905		1
24049	0.501483191	NA	NA		1
12479	0.553914205	NA	NA		1
17401	0.523363792	724	298		1
32446	0.51466271	1000	466		1
63075	0.45878494	522	1000		0
8370	0.514908945	428	938		0
858	0.525902669	NA	NA		1
3256	0.573916737	NA	NA		1
37129	0.542535019	NA	NA		1
876	0.450255827	NA	NA		0
1729	0.5	NA	NA		1
46823	0.472525103	NA	NA		0
4291	0.477096966	208	913		0
39028	0.494524628	NA	NA		0
37429	0.506847629	NA	NA		0
10432	0.457142857	NA	NA		1
957	0.473154362	NA	NA		1
9355	0.491489682	454	929		0
5316	0.481276857	346	1000		0
6318	0.507471853	928	875		1
1288	0.472826087	NA	NA		1
1489	0.473463687	NA	NA		1
3799	0.525197328	NA	NA		0
2270	0.55204461	NA	NA		0
1561	0.578264396	NA	NA		1
2235	0.532186106	NA	NA		1
1779	0.559574468	NA	NA		1
17996	0.522140437	NA	NA		1
21448	0.493124523	800	849		1
437	0.544622426	NA	NA		1
2813	0.494676806	941	928		1
42745	0.477482023	NA	NA		1
31150	0.539079053	NA	NA		0
2985	0.497302697	NA	NA		0
2842	0.467089947	1000	863		1
87532	0.434527169	NA	NA		0
1858	0.520452099	NA	NA		1
5477	0.450490633	NA	NA		0
1820	0.356804734	NA	NA		1
1727	0.497054492	NA	NA		1
13769	0.503108348	568	636		0
2387	0.577861163	NA	NA		1

2200	0.544309701	NA	NA		1
2867	0.457889642	997		1000	1
1204	0.524734982	NA	NA		1
10925	0.524631924	NA	NA		0
517	0.400881057	NA	NA		1
508	0.417302799	NA	NA		1
556	0.366742597	NA	NA		1
938	0.530054645	NA	NA		1
31128	0.465225754	NA	NA		0
4210	0.505860193	NA	NA		0
1654	0.577981651	NA	NA		0
1525	0.506603774	NA	NA		1
1719	0.492411467	NA	NA		1
1661	0.52260934	NA	NA		1
5828	0.454708699	440		1000	1
1261	0.450292398	NA	NA		1
2694	0.431088901	NA	NA		1
477	0.49475891	NA	NA		1
1911	0.427262931	NA	NA		1
1570	0.443446305	NA	NA		1
2390	0.550890279	857		1000	0
4052	0.445868316	429		1000	0
1632	0.442185515	NA	NA		1
729	0.498376623	NA	NA		0
6728	0.516389309	NA	NA		1
2233	0.455955679	NA	NA		1
2115	0.504884319	NA	NA		1
2097	0.456495828	NA	NA		1
1970	0.46659052	NA	NA		1
1859	0.500300661	NA	NA		1
2649	0.538461538	NA	NA		1
29681	0.524616956	602		1000	1
2753	0.513105639	NA	NA		0
2557	0.512680316	NA	NA		0
3467	0.515756148	614		1000	0
2656	0.523298365	543		937	0
2140	0.518409426	NA	NA		0
12725	0.480742679	658		1000	1
1720	0.49970006	NA	NA		1
3598	0.56265586	NA	NA		1
15359	0.486855941	NA	NA		0
1653	0.528266332	NA	NA		1
1583	0.534458509	NA	NA		1
14649	0.381320012	270		1000	1
2580	0.582506763	NA	NA		1
3458	0.528503937	830		941	1
716	0.489130435	NA	NA		1
592	0.434701493	NA	NA		1
989	0.513761468	NA	NA		1
932	0.526824034	NA	NA		1
1540	0.531278332	NA	NA		1
1999	0.522261131	NA	NA		1
2327	0.463257413	NA	NA		1
3823	0.532129109	NA	NA		1
29527	0.509709157	432		1000	0
4493	0.507165109	986		504	1
1511	0.538312318	NA	NA		1

669	0.495881384	NA	NA		1
19611	0.517512077	NA	NA		1
4200	0.497576364	NA	NA		0
19869	0.477656271		623	1000	1
46882	0.557208643	NA	NA		1
972	0.501251564	NA	NA		1
19785	0.491911211	NA	NA		0
3337	0.555226407	NA	NA		1
29544	0.494693436		203	1000	1
63065	0.504112676	NA	NA		1
3632	0.487959579		881	940	1
2525	0.477327935	NA	NA		1
7620	0.553516029		190	1000	1
6746	0.469475494		333	1000	0
3390	0.517208864		345	1000	1
7332	0.457541809	NA	NA		0
3420	0.481942411		248	1000	0
2656	0.524456522	NA	NA		0
1925	0.497994269	NA	NA		1
4211	0.510348584	NA	NA		1
4192	0.504459861		158	883	1
10990	0.463580247	NA	NA		1
2178	0.520858164	NA	NA		1
520	0.410989011	NA	NA		1
1270	0.40475198	NA	NA		1
76275	0.498291572	NA	NA		0
2197	0.465955285	NA	NA		0
105042	0.476988376	NA	NA		1
1998	0.506673931	NA	NA		1
3042	0.425		297	1000	1
1473	0.508559201	NA	NA		1
1246	0.547351525	NA	NA		1
4402	0.53011811		414	868	0
2202	0.475835537		1000	1000	1
2383	0.473064735	NA	NA		1
3309	0.481793198	NA	NA		0
1605	0.481553398	NA	NA		1
6300	0.424775143		189	1000	0
1901	0.483339059	NA	NA		0
1886	0.456820195	NA	NA		0
10822	0.483868423		469	1000	0
49820	0.509401973		1000	882	1
61763	0.455562802	NA	NA		0
8303	0.437243268		575	1000	1
1223	0.529259897	NA	NA		1
3618	0.472357491	NA	NA		0
983	0.493975904	NA	NA		1
2441	0.43878818	NA	NA		0
1838	0.489118607	NA	NA		1
5399	0.539404553	NA	NA		1
14819	0.511672847	NA	NA		0
2751	0.516113965		485	905	1
12010	0.466116173	NA	NA		1
2446	0.531914894	NA	NA		1
2462	0.540788884		869	1000	1
1789	0.49909475	NA	NA		1
1780	0.547735618	NA	NA		1

1312	0.494470046	NA	NA		1
1378	0.416797079	NA	NA		0
1412	0.53220339	NA	NA		1
3553	0.474756168	933		1000	1
3550	0.518142064	886		978	0
34673	0.484164202	NA	NA		0
15345	0.540129781	480		1000	1
19350	0.516955995	240		489	0
4770	0.44847948	NA	NA		0
2285	0.487011349	NA	NA		1
2657	0.529587058	NA	NA		1
3188	0.528596803	877		871	1
8471	0.501200961	NA	NA		1
5905	0.474651861	814		688	0
1918	0.502118644	NA	NA		1
2001	0.482580645	NA	NA		1
8228	0.551663748	NA	NA		0
2663	0.451629863	NA	NA		0
2439	0.477832512	783		1000	1
10657	0.458467482	203		1000	0
61882	0.524253084	1000		1000	1
7453	0.488	668		1000	0
11350	0.518877152	105		1000	0
12930	0.480564688	236		618	0
3362	0.529453015	NA	NA		1
47270	0.530057375	NA	NA		0
808	0.50472103	NA	NA		0
1082	0.561076605	NA	NA		0
5973	0.474392523	803		863	0
828	0.457221081	NA	NA		1
2121	0.516416249	NA	NA		0
1115	0.539011316	NA	NA		0
7554	0.531133113	NA	NA		0
55086	0.378161877	132		1000	0
5395	0.516545877	NA	NA		1
1006	0.519886364	NA	NA		0
3081	0.418780356	NA	NA		1
1906	0.455526992	NA	NA		0
1405	0.537938845	NA	NA		1
636	0.49851338	NA	NA		0
28807	0.522151027	78		1000	1
8583	0.535510421	249		768	0
1235	0.536842105	NA	NA		1
3536	0.454887218	NA	NA		1
92194	0.542712949	287		1000	0
11037	0.451520913	326		886	1
23440	0.521648347	NA	NA		0
811	0.48458693	NA	NA		1
1466	0.481454006	NA	NA		1
23926	0.502741896	NA	NA		1
23926	0.502741896	NA	NA		1
44217	0.483495722	790		1000	0
1912	0.573922531	NA	NA		1
3791	0.491448653	823		864	0
37130	0.449331311	449		1000	1
7239	0.474300042	633		1000	0
1735	0.536788228	NA	NA		1

1433	0.4599018	NA	NA		1
3282	0.496196479	863	938		0
637	0.48822606	NA	NA		1
2182	0.416851889	NA	NA		1
2576	0.461040462	705	1000		1
1371	0.550133097	NA	NA		1
891	0.498316498	NA	NA		1
7984	0.489546794	932	599		1
12871	0.5459822	593	1000		0
3059	0.589369159	716	933		0
1050	0.526908636	NA	NA		1
2623	0.515114379	946	937		1
1666	0.456034954	NA	NA		1
1391	0.540930979	NA	NA		1
941	0.545585068	NA	NA		0
1248	0.549618321	NA	NA		1
1377	0.37483871	NA	NA		0
5308	0.470274036	NA	NA		1
2905	0.482868164	NA	NA		1
4228	0.51997749	430	1000		0
1210	0.367878614	NA	NA		0
2531	0.471064095	NA	NA		0
828	0.549516908	NA	NA		1
2035	0.404164442	NA	NA		1
1178	0.5	NA	NA		1
2689	0.512788852	NA	NA		0
1689	0.536593707	NA	NA		0
3977	0.52	743	877		1
1630	0.55955956	NA	NA		1
10007	0.410762332	111	601		1
6356	0.498693152	NA	NA		1
1655	0.541993958	NA	NA		1
1433	0.550391937	NA	NA		0
8835	0.520693642	NA	NA		0
6778	0.453052694	379	1000		0
2998	0.505077499	NA	NA		0
53554	0.479569892	NA	NA		1
3714	0.468670771	NA	NA		0
2953	0.446341463	759	928		1
6072	0.565606075	941	869		1
2257	0.515249194	948	819		1
2025	0.548927263	NA	NA		1
1693	0.42301629	NA	NA		0
19393	0.536965282	NA	NA		0
10861	0.562137586	471	1000		0
30864	0.531058899	274	1000		0
17513	0.528642881	997	1000		0
18404	0.519362261	NA	NA		0
42507	0.539426523	785	1000		0
15107	0.467622695	NA	NA		0
4294	0.522594899	277	933		0
723	0.495159059	NA	NA		1
2372	0.448495229	840	929		1
26554	0.533314432	1000	1000		1
15558	0.548853648	378	931		1
2208	0.480207433	NA	NA		1
6799	0.553122414	NA	NA		0

15842	0.475506506		437	1000	0
2886	0.537184116	NA	NA		1
15629	0.549220011		334	936	0
3436	0.513388735	NA	NA		1
1422	0.464646465	NA	NA		1
2633	0.513750498		1000	876	1
2270	0.502337359	NA	NA		0
3175	0.492201742	NA	NA		0
1514	0.469161469	NA	NA		1
4161	0.477449983		420	884	1
9203	0.50590281		192	935	1
6611	0.448559819		932	936	0
24960	0.456959666	NA	NA		0
479	0.4217119	NA	NA		1
3159	0.516535934		696	967	0
2859	0.513409962	NA	NA		1
16614	0.512801967		830	1000	0
1773	0.566994701	NA	NA		1
11825	0.473769746	NA	NA		0
5872	0.485985617	NA	NA		0
32788	0.504022724	NA	NA		0
2427	0.533935413	NA	NA		0
3369	0.552558995	NA	NA		1
2721	0.518518519		865	938	1
600	0.524557957	NA	NA		1
570	0.534	NA	NA		1
419	0.529832936	NA	NA		1
413	0.527845036	NA	NA		1
338	0.532544379	NA	NA		1
33371	0.556503314		663	879	0
11851	0.498536702		514	1000	0
1991	0.57282079	NA	NA		1
696	0.530081301	NA	NA		1
1556	0.53597848	NA	NA		1
8267	0.498870482		145	1000	1
2224	0.522931655		1000	1000	1
9118	0.4484375	NA	NA		1
34518	0.345528455	NA	NA		1
1422	0.516500384	NA	NA		1
3051	0.559196617	NA	NA		1
17795	0.555493896		697	1000	0
2473	0.376199616	NA	NA		1
62657	0.495848734	NA	NA		1
1609	0.572275422	NA	NA		1
14539	0.467061694	NA	NA		0
871	0.548029557	NA	NA		1
1188	0.489043825	NA	NA		1
1500	0.55037037	NA	NA		1
4337	0.508823529		742	810	1
2206	0.524994133		936	1000	1
7786	0.534317713		352	769	1
1797	0.542153846	NA	NA		1
1491	0.588739746	NA	NA		1
1423	0.553056922	NA	NA		1
5965	0.519456455		642	1000	0
2017	0.532620321	NA	NA		0
730	0.53539823	NA	NA		1

9915	0.503362517	393	966	1
1661	0.526189043	NA	NA	1
1179	0.500662252	NA	NA	1
16420	0.525154763	NA	NA	0
1082	0.500924214	NA	NA	1
5908	0.520405001	926	855	1
4996	0.567750147	338	821	0
504	0.392105263	NA	NA	1
3348	0.460649819	NA	NA	0
1204	0.48973306	NA	NA	1
1766	0.454782815	NA	NA	1
1788	0.489247312	NA	NA	0
1509	0.551845343	NA	NA	0
1874	0.556463596	NA	NA	1
9276	0.34361949	319	1000	0
30216	0.478505781	207	802	0
2863	0.559757403	1000	1000	1
3336	0.541339492	NA	NA	0
4038	0.523825503	NA	NA	1
1511	0.498879761	NA	NA	1
17653	0.513269457	NA	NA	0
3551	0.529025745	NA	NA	0
10154	0.51257522	527	870	1
3341	0.420454545	NA	NA	1
3302	0.529774761	NA	NA	1
777	0.515050167	NA	NA	1
3883	0.5145303	445	1000	1
2954	0.489745323	NA	NA	1
2253	0.437256177	NA	NA	1
2626	0.499381188	NA	NA	1
5023	0.465581336	176	1000	0
3793	0.433133935	345	566	0
8557	0.515582822	NA	NA	1
8084	0.500957934	124	933	1
11633	0.491601951	NA	NA	1
16359	0.46957285	NA	NA	0
1671	0.494710641	NA	NA	1
2169	0.519384615	NA	NA	1
50954	0.457294504	NA	NA	1
19738	0.489811974	NA	NA	1
19382	0.516068408	NA	NA	1
2275	0.557013575	NA	NA	1
5040	0.521759153	1000	1000	1
1278	0.544726302	NA	NA	1
577	0.451048951	NA	NA	1
1333	0.48502994	NA	NA	1
17413	0.506904259	NA	NA	1
1384	0.500476644	NA	NA	1
1199	0.523613963	NA	NA	1
680	0.479411765	NA	NA	1
1001	0.461375661	NA	NA	1
1915	0.515317919	NA	NA	1
2260	0.525606469	NA	NA	1
1208	0.471332209	NA	NA	0
1650	0.502442997	NA	NA	1
15437	0.405379551	694	741	1
31485	0.457380211	239	1000	0

2666	0.491221225	NA	NA		0
8413	0.507071499	NA	NA		1
3064	0.484007833	1000		1000	1
15369	0.431287186	372		1000	0
19376	0.519861883	465		1000	0
3468	0.494736842	NA	NA		1
14074	0.569531996	87		743	1
1702	0.548068284	NA	NA		1
17487	0.489325018	744		761	0
2062	0.530883919	NA	NA		1
1665	0.515341702	NA	NA		1
2051	0.522884013	NA	NA		1
1874	0.531755196	NA	NA		1
12106	0.53974175	NA	NA		1
7251	0.526645768	NA	NA		1
5124	0.520550692	NA	NA		0
2719	0.538203191	NA	NA		1
3701	0.529842013	1000		875	1
2230	0.553352912	NA	NA		0
2308	0.521663778	1000		1000	1
1301	0.527210884	NA	NA		1
2824	0.500870238	NA	NA		0
1849	0.542245141	NA	NA		0
1986	0.515119917	NA	NA		1
5766	0.462542565	100		905	0
3794	0.513741064	594		1000	0
874	0.52404439	NA	NA		1
19211	0.543024581	NA	NA		0
18611	0.55	NA	NA		1
10086	0.523471236	83		1000	0
1899	0.495703704	NA	NA		1
1838	0.524184477	NA	NA		1
1433	0.509306261	NA	NA		1
2009	0.536822885	NA	NA		1
35482	0.516447976	275		863	0
9939	0.511075601	586		813	0
1164	0.482630273	NA	NA		1
57121	0.545672929	NA	NA		1
2753	0.571428571	949		937	1
656	0.424761905	NA	NA		1
3076	0.445158698	591		833	1
134308	0.506129068	NA	NA		0
4553	0.451546392	165		1000	1
3192	0.495614035	1000		1000	1
6660	0.453588085	198		959	1
38894	0.481719238	NA	NA		0
2138	0.515434986	1000		1000	1
2208	0.535302594	NA	NA		1
28026	0.525555164	NA	NA		0
2908	0.548612422	NA	NA		0
7066	0.450576107	265		931	1
42812	0.504239818	NA	NA		0
3211	0.441272274	839		1000	1
10526	0.527154169	NA	NA		0
2954	0.473279239	NA	NA		0
2891	0.555555556	NA	NA		1
4486	0.555104251	NA	NA		0

1593	0.559286464	NA	NA	0
2686	0.509676062	1000	992	1
8705	0.559120485	NA	NA	1
1953	0.483978638	NA	NA	1
1964	0.510204082	NA	NA	1
9950	0.458478591	NA	NA	1
1775	0.565880322	NA	NA	0
2771	0.506332197	NA	NA	0
4784	0.466849918	949	1000	1
6297	0.390222772	641	755	1
1834	0.452914798	NA	NA	0
3956	0.507845934	NA	NA	1
94206	0.42745463	NA	NA	0
7746	0.457468354	NA	NA	1
5363	0.491549575	1000	1000	1
2909	0.356958984	NA	NA	1
2683	0.503550296	NA	NA	0
47583	0.510303557	414	1000	0
1066	0.517193948	NA	NA	1
1917	0.508539945	NA	NA	1
1523	0.492763611	NA	NA	1
1837	0.526240798	NA	NA	0
3562	0.567618156	NA	NA	1
2391	0.458790567	NA	NA	0
693	0.500721501	NA	NA	1
1328	0.504518072	NA	NA	1
4407	0.525822928	397	868	0
6096	0.481283422	1000	1000	1
4312	0.522377109	NA	NA	1
25524	0.511481315	271	1000	1
1611	0.516046213	NA	NA	1
5147	0.50684285	NA	NA	0
1218	0.514778325	NA	NA	1
44482	0.468356003	NA	NA	1
2688	0.573669005	NA	NA	1
2351	0.534122563	NA	NA	0
4137	0.499676794	353	1000	1
3574	0.515406162	NA	NA	0
2653	0.500129366	441	1000	0
1267	0.524338173	NA	NA	1
1642	0.513313609	NA	NA	1
2202	0.551564905	NA	NA	1
458	0.530567686	NA	NA	1
1994	0.519187359	NA	NA	1
4095	0.499557718	NA	NA	0
2433	0.51734104	NA	NA	1
4029	0.462334218	893	1000	1
2055	0.486684421	NA	NA	1
1226	0.570255474	NA	NA	1
3047	0.615907546	NA	NA	1
1203	0.606116775	NA	NA	1
3557	0.5009113	1000	862	1
4944	0.493085518	1000	860	1
94602	0.511974735	NA	NA	1
8188	0.539573991	348	828	0
573	0.569536424	NA	NA	1
2769	0.50653753	NA	NA	1

1369	0.462506842	NA	NA		0
1287	0.501620746	NA	NA		1
2134	0.545862413	932		940	1
2479	0.492647059	NA	NA		0
2338	0.499242041	NA	NA		1
2625	0.455571919	NA	NA		1
466	0.474248927	NA	NA		1
29914	0.44308254	803		1000	0
1748	0.521045918	NA	NA		1
434	0.411290323	NA	NA		1
3747	0.498354361	NA	NA		0
4898	0.482908546	158		1000	1
1802	0.487791343	NA	NA		1
6606	0.56749522	NA	NA		1
2824	0.536493604	889		945	1
1095	0.464840183	NA	NA		1
7044	0.535060699	NA	NA		0
1106	0.542795233	NA	NA		1
2478	0.538647343	NA	NA		1
5829	0.505750683	925		1000	0
2798	0.507194245	NA	NA		1
9650	0.505690309	351		931	0
1626	0.554193548	NA	NA		1
2465	0.505821866	859		1000	0
1828	0.443705572	NA	NA		0
1311	0.480347116	NA	NA		1
7560	0.52226649	NA	NA		0
1299	0.588435374	NA	NA		1
32682	0.556840002	NA	NA		0
1656	0.533458412	NA	NA		1
4614	0.584114053	461		786	0
946	0.572938689	NA	NA		1
1879	0.488246956	NA	NA		0
1791	0.526713124	NA	NA		1
5651	0.533854803	833		824	1
8168	0.453817405	284		1000	0
1803	0.470560077	NA	NA		0
945	0.5221843	NA	NA		1
6688	0.489208633	NA	NA		1
10327	0.514028151	1000		1000	0
3648	0.492337165	215		808	1
3486	0.530446109	882		1000	1
4170	0.543783917	1000		1000	0
36124	0.392519798	NA	NA		0
2783	0.516415604	806		1000	1
11303	0.374001452	867		831	1
17200	0.373903569	645		1000	1
2142	0.559655007	1000		945	1
4153	0.520615385	1000		1000	1
2961	0.522162958	NA	NA		0
3546	0.514082894	1000		869	0
3878	0.504746835	633		1000	1
71947	0.537553464	NA	NA		0
2353	0.389	NA	NA		1
2500	0.4909375	NA	NA		1
2017	0.46149615	NA	NA		1
1571	0.431152011	NA	NA		1

1619	0.546184739	NA	NA	0
828	0.486714976	NA	NA	1
1789	0.528347407	NA	NA	1
5426	0.450092817	452	487	1
12888	0.471076108	143	940	0
2512	0.484609053	NA	NA	0
610	0.545098039	NA	NA	1
9179	0.55501963	NA	NA	0
2792	0.501767887	749	1000	0
4558	0.489234886	NA	NA	0
9130	0.474811083	NA	NA	1
3947	0.564646465	NA	NA	1
10241	0.377206591	582	1000	1
3524	0.570273003	929	853	1
3053	0.487405038	878	1000	1
30239	0.547320913	NA	NA	0
9702	0.514070772	NA	NA	0
6840	0.512015833	NA	NA	0
6840	0.521843687	NA	NA	1
3238	0.414348981	698	666	0
11659	0.474420317	NA	NA	1
2579	0.551554828	923	942	1
2643	0.502345416	NA	NA	1
11928	0.525002982	932	1000	0
4867	0.436433064	NA	NA	0
14349	0.430713094	NA	NA	0
1026	0.49261745	NA	NA	1
665	0.509594883	NA	NA	1
2291	0.529700759	NA	NA	1
5362	0.455661323	632	1000	0
8396	0.447113916	NA	NA	1
1425	0.445890968	NA	NA	1
1949	0.455205811	NA	NA	1
2203	0.493990385	1000	1000	0
39119	0.572815534	218	826	0
768	0.466197183	NA	NA	1
40906	0.519200999	NA	NA	0
31527	0.480510498	59	1000	0
13167	0.483937705	NA	NA	0
4341	0.498937301	NA	NA	1
3768	0.425204583	397	1000	1
1989	0.517945886	NA	NA	1
1002	0.478740157	NA	NA	1
2558	0.497263487	1000	1000	1
2436	0.456229568	NA	NA	0
11322	0.363420038	NA	NA	0
38414	0.56982973	1000	1000	1
5301	0.46004481	668	895	1
2387	0.426096811	NA	NA	0
32320	0.526561261	927	426	0
4379	0.472964763	NA	NA	0
7269	0.490077741	69	1000	1
1848	0.459292532	NA	NA	1
4345	0.520164398	788	817	1
2768	0.485910405	1000	1000	1
18177	0.519965025	NA	NA	0
886	0.483702738	NA	NA	1

2598	0.504530478	NA	NA	1
1790	0.486603284	NA	NA	0
7134	0.490397024	NA	NA	0
3744	0.415380048	NA	NA	1
1486	0.583253128	NA	NA	1
968	0.545454545	NA	NA	1
4699	0.356527666	764	401	0
914	0.50547046	NA	NA	1
2811	0.483883648	NA	NA	0
1770	0.472379603	NA	NA	1
1446	0.452798663	NA	NA	1
1559	0.527065527	NA	NA	1
37339	0.356384903	637	883	0
1591	0.487804878	NA	NA	1
2560	0.473915163	892	1000	1
2824	0.516061585	NA	NA	0
2714	0.54186718	NA	NA	1
3726	0.473477407	NA	NA	1
6562	0.512206967	635	1000	0
10892	0.463239075	861	936	1
7051	0.585323641	842	734	0
4257	0.517994064	920	1000	0
2573	0.430497443	NA	NA	1
3804	0.519015976	NA	NA	0
7184	0.493187728	544	1000	0
8358	0.439053972	260	1000	0
2779	0.575601375	NA	NA	1
4086	0.439416058	945	394	1
2126	0.533002129	NA	NA	1
1197	0.551431601	NA	NA	1
1561	0.521637427	NA	NA	1
1320	0.468067227	NA	NA	1
14655	0.534669078	784	1000	1
1401	0.534277198	NA	NA	1
2079	0.498316498	NA	NA	1
2260	0.448125	NA	NA	1
3021	0.52339688	NA	NA	0
12468	0.514588859	NA	NA	0
2735	0.505263158	693	1000	1
14703	0.457815167	NA	NA	0
3385	0.504575163	NA	NA	0
14644	0.453840803	410	939	0
2846	0.566030946	1000	1000	1
14300	0.453383909	364	1000	0
1140	0.447718631	NA	NA	1
1295	0.473257699	NA	NA	1
775	0.487741935	NA	NA	1
1481	0.489299203	NA	NA	1
2525	0.492119089	939	820	1
1147	0.497919556	NA	NA	1
20924	0.460567156	NA	NA	1
2616	0.510895884	924	938	1
1657	0.498687664	NA	NA	1
1374	0.518898488	NA	NA	0
2757	0.448167127	NA	NA	1
1389	0.55453149	NA	NA	1
1940	0.569528416	NA	NA	0

979	0.504979496	NA	NA	1
3881	0.511521972	NA	NA	0
3020	0.531400966	NA	NA	1
1089	0.483783784	NA	NA	1
1306	0.5	NA	NA	1
1925	0.505047319	NA	NA	0
6846	0.426282051	251	926	0
2288	0.487049399	NA	NA	1
2101	0.472190692	NA	NA	1
3230	0.469047619	NA	NA	1
1954	0.536984754	NA	NA	1
3161	0.530643801	530	1000	0
1674	0.497655727	NA	NA	1
2522	0.558045792	808	934	0
4149	0.475244103	936	1000	1
2469	0.539489672	NA	NA	1
3495	0.521387463	989	1000	0
1436	0.466666667	NA	NA	1
2706	0.463414634	940	801	1
18258	0.489002712	NA	NA	0
5487	0.442822384	NA	NA	0
1569	0.459649123	NA	NA	1
3038	0.385319486	NA	NA	0
20487	0.496071063	NA	NA	0
23862	0.533735943	647	939	0
3383	0.524799525	155	1000	1
2348	0.550821355	NA	NA	1
951	0.588853838	NA	NA	1
11922	0.533234127	374	706	1
3301	0.466729768	807	1000	0
39218	0.478754152	26	1000	0
11441	0.467098768	317	941	0
13284	0.555037727	420	1000	0
5582	0.5296	171	941	1
753	0.484444444	NA	NA	1
924	0.497458243	NA	NA	0
3914	0.421179302	NA	NA	1
4587	0.445895522	NA	NA	1
3289	0.509591584	1000	1000	1
17198	0.490463537	595	937	0
5949	0.537602383	340	936	1
528	0.543560606	NA	NA	1
3836	0.541464728	918	1000	1
4868	0.501502196	892	752	1
8197	0.540185894	690	1000	1
1933	0.521925134	NA	NA	1
3817	0.478305362	523	824	1
3097	0.512523364	880	871	1
3011	0.483443709	NA	NA	1
1349	0.512567325	NA	NA	1
1718	0.534056661	NA	NA	1
1257	0.49	NA	NA	1
6767	0.503700014	NA	NA	0
4611	0.498765813	904	943	0
7144	0.543264296	NA	NA	0
3108	0.510857957	NA	NA	1
2119	0.454155496	NA	NA	1

4205	0.435240779	NA	NA	1
3351	0.479899497	NA	NA	1
1454	0.494930876	NA	NA	1
1486	0.487213997	NA	NA	1
8678	0.527780145	NA	NA	0
7716	0.504255319	NA	NA	1
1662	0.52835408	NA	NA	1
2262	0.536368135	NA	NA	1
1690	0.462864721	NA	NA	1
6069	0.533155248	538	941	1
12079	0.539513951	331	745	1
11570	0.547090374	310	939	0
2323	0.489883771	1000	1000	1
7781	0.501636024	NA	NA	0
19646	0.562237372	NA	NA	0
12751	0.503492433	443	843	1
1552	0.486792453	NA	NA	1
23772	0.583035959	NA	NA	0
3576	0.485346216	852	946	1
4668	0.534681714	640	931	0
1457	0.524401616	NA	NA	1
3150	0.513157895	NA	NA	1
15500	0.489208633	NA	NA	1
26611	0.538434705	153	872	0
18385	0.518834978	748	1000	0
1815	0.492588369	NA	NA	1
17970	0.529144323	433	1000	0
3031	0.523575639	278	869	1
3064	0.518942102	921	881	1
22397	0.506341069	NA	NA	0
1808	0.54467354	NA	NA	1
4153	0.493554953	NA	NA	0
2105	0.488497308	NA	NA	1
13853	0.539145803	NA	NA	0
5579	0.469808542	NA	NA	0
3711	0.474392742	887	941	1
1284	0.497663551	NA	NA	1
664	0.567771084	NA	NA	1
1711	0.507832639	NA	NA	0
12315	0.534965035	NA	NA	1
2070	0.559903382	1000	1000	1
861	0.596980256	NA	NA	1
3443	0.598166765	1000	1000	1
1840	0.573847052	NA	NA	1
1402	0.596031746	NA	NA	1
9096	0.519263994	NA	NA	0
6367	0.537826685	NA	NA	1
1482	0.40107362	NA	NA	1
2785	0.575891216	NA	NA	1
2586	0.443157133	NA	NA	1
2460	0.513026487	NA	NA	0
2975	0.45276873	740	933	1
1568	0.552807224	NA	NA	1
1928	0.493756328	NA	NA	1
1700	0.475092937	NA	NA	1
1535	0.440435671	NA	NA	1
1011	0.490502793	NA	NA	1

9853	0.485457101	354	946	0
1425	0.519852941	NA	NA	1
10083	0.489772727	NA	NA	0
2233	0.56496063	926	873	1
2494	0.506381227	935	1000	1
30193	0.513703194	234	873	0
1270	0.549019608	NA	NA	1
7376	0.535218736	511	872	0
392408	0.501721803	NA	NA	0
30857	0.530607531	151	1000	1
19725	0.555420977	441	867	0
2889	0.545982575	NA	NA	1
3827	0.426484598	1000	1000	1
1393	0.420725389	NA	NA	1
912	0.463815789	NA	NA	1
4062	0.433558559	NA	NA	1
2615	0.507212926	NA	NA	1
1181	0.504048583	NA	NA	1
9937	0.488407163	927	800	1
15632	0.403255865	NA	NA	0
711	0.509202454	NA	NA	1
2665	0.495098605	NA	NA	1
4749	0.461216608	228	1000	1
1846	0.459913326	NA	NA	1
1765	0.545592245	NA	NA	0
2517	0.420454545	595	1000	1
10354	0.4819183	1000	1000	0
1364	0.478894858	NA	NA	1
1401	0.394301704	NA	NA	1
241	0.481327801	NA	NA	1
242	0.479338843	NA	NA	1
626	0.562300319	NA	NA	1
2972	0.48479613	NA	NA	0
14691	0.506425548	1000	1000	0
5898	0.497355697	NA	NA	0
2009	0.496644295	NA	NA	1
4288	0.492023772	NA	NA	0
2742	0.483606557	1000	1000	1
2347	0.460950081	942	938	0
23541	0.477495981	246	1000	0
2437	0.492194967	NA	NA	0
852	0.338781575	NA	NA	1
34810	0.510365071	NA	NA	1
38762	0.484168724	NA	NA	0
3527	0.506027639	NA	NA	1
3761	0.509882555	853	877	1
2391	0.543468366	806	1000	1
3935	0.50209986	NA	NA	0
5997	0.492344799	412	1000	0
35304	0.556114113	425	931	1
5953	0.529035208	779	1000	1
5582	0.494861841	NA	NA	0
4898	0.549317147	81	1000	0
7876	0.524600171	196	784	1
15993	0.492399683	154	1000	0
7679	0.535220613	NA	NA	0
2575	0.475965665	NA	NA	1

1620	0.420881971	NA	NA		1
1899	0.502842704	NA	NA		1
1684	0.454275534	NA	NA		1
2809	0.435953421	NA	NA		1
19105	0.516797312		214	495	0
2535	0.468503937		817	934	1
2359	0.520710059	NA	NA		1
1248	0.453098827	NA	NA		1
21810	0.449484363		843	867	1
2487	0.518134715	NA	NA		1
1580	0.490075291	NA	NA		1
1226	0.5226642	NA	NA		1
1042	0.52780807	NA	NA		1
670	0.431513903	NA	NA		1
6474	0.467470292		627	934	1
2819	0.494127177		910	846	1
1694	0.576271186	NA	NA		1
2011	0.578179291	NA	NA		1
2721	0.544939844	NA	NA		1
2842	0.467282525	NA	NA		1
31910	0.454180602		224	1000	1
1745	0.45149592	NA	NA		1
1622	0.463625154	NA	NA		1
4172	0.518272425	NA	NA		1
617	0.572123177	NA	NA		1
450	0.508888889	NA	NA		1
9919	0.541144017		159	654	0
5746	0.53073325	NA	NA		1
6413	0.496103896	NA	NA		1
9476	0.463117143		383	1000	0
41557	0.554830918	NA	NA		0
39871	0.44825547	NA	NA		1
57299	0.432603687	NA	NA		1
10233	0.530597342		217	940	0
4778	0.478777216		821	1000	1
1749	0.512801756	NA	NA		1
853	0.441958042	NA	NA		1
799	0.430538173	NA	NA		1
2224	0.48830735	NA	NA		1
682	0.495081967	NA	NA		0
595	0.467226891	NA	NA		1
6931	0.522318345	NA	NA		0
16556	0.487970133	NA	NA		1
1746	0.496	NA	NA		1
1774	0.549555707	NA	NA		1
1509	0.540755467	NA	NA		1
1389	0.524838013	NA	NA		1
2745	0.498439938	NA	NA		0
2081	0.488690163	NA	NA		1
1960	0.508249641	NA	NA		0
1040	0.506730769	NA	NA		1
1413	0.527552674	NA	NA		1
1099	0.485210466	NA	NA		0
1191	0.428675189	NA	NA		0
8653	0.535986914	NA	NA		1
6523	0.463179317		632	922	1
33901	0.484314734	NA	NA		0

1163	0.459815547	NA		NA	1
1408	0.473684211	NA		NA	1
8702	0.482408256		408	1000	0
3524	0.489722967		579	882	1
789	0.50752394	NA		NA	1
5247	0.493533729		1000	798	1
2757	0.510461903		1000	877	0
686	0.449920509	NA		NA	1
1252	0.506389776	NA		NA	1
1301	0.461952344	NA		NA	1
641	0.482059282	NA		NA	1
2369	0.522342733	NA		NA	1
814	0.484029484	NA		NA	1
1560	0.482051282	NA		NA	1
602	0.420131291	NA		NA	1
1574	0.391508205	NA		NA	1
1414	0.423047177	NA		NA	1
1921	0.437170011	NA		NA	1
4723	0.571236106		682	561	1
66403	0.530917613	NA		NA	0
1713	0.472690492	NA		NA	1
1318	0.501517451	NA		NA	1
1158	0.507772021	NA		NA	1
2278	0.40862158	NA		NA	1
49267	0.422595104	NA		NA	1
22140	0.475235849		1000	1000	1
1620	0.437618147	NA		NA	0
6757	0.48217794		468	886	0
16214	0.46214338		277	1000	1
1513	0.458476321	NA		NA	1
665	0.503759398	NA		NA	1
7263	0.390336134		937	1000	1
1090	0.398165138	NA		NA	1
5943	0.370792248		939	1000	1
2183	0.507153429		844	1000	1
1392	0.471641791	NA		NA	1
24159	0.628407461		219	886	1
2397	0.537519142	NA		NA	1
774	0.472868217	NA		NA	1
1157	0.509330406	NA		NA	1
933	0.517836594	NA		NA	1
733	0.544338336	NA		NA	1
2302	0.519147972		931	1000	1
1227	0.494011976	NA		NA	1
3011	0.462681025		889	793	1
1645	0.502875924	NA		NA	1
2838	0.504745834	NA		NA	1
2807	0.514672216	NA		NA	1
2271	0.481062747	NA		NA	1
842	0.433460076	NA		NA	1
869	0.463444857	NA		NA	1
877	0.483556638	NA		NA	1
1238	0.45638126	NA		NA	1
2530	0.448011165		679	1000	1
3793	0.490179691		1000	776	0
3508	0.511231603	NA		NA	1
92189	0.363930886		383	1000	1

2045	0.430045872	NA	NA		1
1733	0.534900808	NA	NA		1
3565	0.534782146	1000		808	1
2652	0.534899793	768		869	1
1585	0.559465165	NA	NA		1
5197	0.50680071	NA	NA		1
1662	0.453168044	NA	NA		1
1906	0.545182972	NA	NA		1
3378	0.522225881	NA	NA		1
3020	0.514962385	1000		812	0
1559	0.420977011	NA	NA		1
1426	0.408146965	NA	NA		1
1593	0.523450586	NA	NA		1
10907	0.514264813	NA	NA		1
1355	0.46723869	NA	NA		1
9171	0.416614223	1000		1000	0
1562	0.488489209	NA	NA		1
3181	0.458975165	1000		1000	1
3819	0.467184801	NA	NA		0
4996	0.511784512	1000		975	1
916	0.478313253	NA	NA		1
7564	0.553484243	489		1000	1
10266	0.466345341	262		880	0
1534	0.460995542	NA	NA		1
3519	0.528143275	828		500	1
1241	0.524170616	NA	NA		1
1986	0.488401254	NA	NA		1
1424	0.467399267	NA	NA		1
4046	0.525210084	1000		1000	1
4188	0.553105049	NA	NA		1
1643	0.473950796	NA	NA		1
2521	0.523925385	NA	NA		1
2376	0.50925181	NA	NA		1
3123	0.440670186	620		968	0
4584	0.48723099	NA	NA		0
4584	0.468135513	NA	NA		1
5963	0.513364394	266		1000	1
5954	0.550674507	539		1000	0
1767	0.503253796	NA	NA		1
2057	0.550274223	NA	NA		0
1312	0.476371951	NA	NA		1
2039	0.508329127	NA	NA		1
2086	0.503209243	NA	NA		1
3711	0.463351211	NA	NA		0
1944	0.559866745	NA	NA		0
2510	0.560618389	948		1000	1
1804	0.524774775	NA	NA		1
27048	0.461167565	NA	NA		0
1745	0.458682635	NA	NA		1
956	0.541317365	NA	NA		1
1204	0.5	NA	NA		0
1460	0.49187935	NA	NA		1
797	0.477234401	NA	NA		1
4778	0.499652375	939		778	1
1946	0.49432497	NA	NA		1
2057	0.453032105	NA	NA		1
1643	0.502875399	NA	NA		1

475	0.503157895	NA	NA	1
17475	0.576241901	NA	NA	1
719	0.549648163	NA	NA	0
590	0.501694915	NA	NA	1
899	0.540090772	NA	NA	1
3122	0.569721116	NA	NA	1
742	0.388140162	NA	NA	1
3326	0.552378407	NA	NA	0
1008	0.510158014	NA	NA	1
847	0.491997216	NA	NA	1
913	0.494699647	NA	NA	1
1087	0.495652174	NA	NA	1
1803	0.499602228	NA	NA	1
725	0.511724138	NA	NA	1
1045	0.628844115	NA	NA	1
1169	0.577928364	NA	NA	1
1187	0.551811289	NA	NA	1
914	0.53964497	NA	NA	1
56458	0.45545977	NA	NA	1
835	0.470658683	NA	NA	1
1225	0.43982684	NA	NA	1
1873	0.441927991	NA	NA	1
1575	0.446057407	NA	NA	1
5647	0.438914398	297	1000	0
1900	0.463000877	NA	NA	1
2509	0.473405535	703	1000	0
3311	0.493719395	802	868	1
4096	0.493274774	619	1000	1
3487	0.452740741	947	941	1
3118	0.512429566	1000	1000	1
522	0.440613027	NA	NA	1
5162	0.514492754	935	864	1
921	0.456026059	NA	NA	1
573	0.511343805	NA	NA	1
2383	0.488879564	1000	1000	1
2195	0.47566719	NA	NA	1
2306	0.503565062	938	1000	1
2224	0.488247146	NA	NA	1
4718	0.513613534	NA	NA	1
1844	0.535741159	NA	NA	0
4884	0.487763996	371	1000	1
1529	0.490018149	NA	NA	1
1868	0.499113999	NA	NA	1
4155	0.439368389	1000	1000	1
888	0.506756757	NA	NA	1
1998	0.468968969	NA	NA	1
1040	0.494877049	NA	NA	1
1077	0.507892293	NA	NA	1
939	0.431792559	NA	NA	1
8540	0.526039016	267	1000	1
979	0.459652707	NA	NA	1
577	0.465402844	NA	NA	0
7502	0.428187449	165	844	0
860	0.48372093	NA	NA	1
1594	0.410288582	NA	NA	1
1910	0.493534483	NA	NA	1
3166	0.557883131	801	812	1

11437	0.492877136		126	1000	0
648	0.479938272	NA	NA		1
11178	0.491011236	NA	NA		1
530	0.520754717	NA	NA		1
2731	0.502824859	NA	NA		1
1157	0.534140017	NA	NA		1
1308	0.427797834	NA	NA		1
2166	0.469463622	NA	NA		1
1643	0.527084601	NA	NA		1
2676	0.518914788	NA	NA		1
60886	0.462228096		141	1000	0
8478	0.440078724		555	1000	0
2230	0.555642633	NA	NA		1
1102	0.508268059	NA	NA		0
10077	0.58680746	NA	NA		0
1645	0.580645161	NA	NA		0
1527	0.534175334	NA	NA		1
856	0.489953632	NA	NA		1
4114	0.493819267		812	1000	0
4925	0.522704339	NA	NA		1
4422	0.541410185		1000	929	1
1433	0.504535939	NA	NA		1
2397	0.536386986		1000	939	1
668	0.547107438	NA	NA		1
1156	0.544747082	NA	NA		1
957	0.524555904	NA	NA		1
1014	0.539447732	NA	NA		1
2524	0.53611691		940	931	1
2109	0.535365152	NA	NA		1
3728	0.572135202	NA	NA		0
2015	0.431766055	NA	NA		1
2019	0.505097312	NA	NA		1
1482	0.535405872	NA	NA		1
1703	0.475204622	NA	NA		1
9373	0.548592999		432	1000	0
13685	0.551245283		318	881	1
1305	0.502991904	NA	NA		0
5727	0.47907344	NA	NA		0
86306	0.547742396		182	1000	1
1848	0.506784661	NA	NA		1
2796	0.484453893	NA	NA		1
3242	0.512373096	NA	NA		1
951	0.516298633	NA	NA		1
1741	0.47423037	NA	NA		1
1298	0.517013233	NA	NA		1
1440	0.58125	NA	NA		1
2129	0.575892857	NA	NA		0
11756	0.602715028		165	869	0
4211	0.515283843	NA	NA		0
1870	0.520289049	NA	NA		1
1756	0.51783591	NA	NA		1
1357	0.499076639	NA	NA		1
2655	0.483340737		840	930	0
2211	0.47987336	NA	NA		1
52034	0.484515949	NA	NA		0
1134	0.50896861	NA	NA		1
896	0.5	NA	NA		1

1942	0.54912598	NA	NA	1
3217	0.534130435	880	936	0
1175	0.536170213	NA	NA	1
5706	0.537706725	1000	1000	1
2705	0.496961512	1000	1000	0
19872	0.459855052	846	1000	1
19512	0.48086785	NA	NA	0
8204	0.502718589	492	940	1
14391	0.503744076	NA	NA	0
10144	0.489134359	361	1000	0
2738	0.563542494	842	938	1
1822	0.562003665	NA	NA	1
1000	0.541573034	NA	NA	1
1288	0.540229885	NA	NA	1
4499	0.475314618	1000	841	1
3505	0.592958616	932	877	1
1611	0.494825356	NA	NA	1
2112	0.50513347	1000	1000	1
730	0.505479452	NA	NA	1
757	0.474240423	NA	NA	1
58322	0.512455516	NA	NA	1
2349	0.562248996	1000	1000	1
1087	0.483900644	NA	NA	1
1424	0.535814607	NA	NA	1
1107	0.497741644	NA	NA	1
43668	0.480170349	NA	NA	1
1162	0.512908778	NA	NA	1
22389	0.392996531	NA	NA	0
3720	0.502538071	NA	NA	1
103795	0.505376344	NA	NA	0
124672	0.538870174	NA	NA	0
1760	0.49906367	NA	NA	0
9144	0.440905874	NA	NA	1
4589	0.449612403	455	1000	1
2096	0.462493533	NA	NA	1
794	0.406801008	NA	NA	1
1263	0.431714024	NA	NA	1
814	0.404993065	NA	NA	1
1477	0.44211239	NA	NA	1
3045	0.457866537	369	938	1
2478	0.462066182	NA	NA	1
829	0.400130548	NA	NA	1
5146	0.528092923	NA	NA	0
2971	0.503725782	713	1000	1
2922	0.532217782	523	932	0
1832	0.458316871	NA	NA	1
14245	0.481368357	NA	NA	1
622	0.508038585	NA	NA	1
2454	0.46783886	1000	1000	1
2317	0.558125741	NA	NA	1
2507	0.520941364	1000	1000	1
744	0.507952286	NA	NA	0
628	0.515151515	NA	NA	1
4134	0.467983244	829	1000	0
3008	0.494880546	NA	NA	1
2139	0.486786861	1000	1000	0
639	0.508607199	NA	NA	1

1745	0.477363897	NA	NA	1
5232	0.511499902	1000	1000	1
2364	0.517166449	1000	937	1
3196	0.54973822	NA	NA	1
2044	0.528375734	NA	NA	1
2562	0.530444965	NA	NA	1
1723	0.506344411	NA	NA	1
1056	0.533143939	NA	NA	1
1698	0.551825677	NA	NA	1
2002	0.536463536	1000	1000	1
548	0.578467153	NA	NA	1
1883	0.512947658	NA	NA	1
1548	0.523901809	NA	NA	1
1586	0.570617907	NA	NA	1
1364	0.517706577	NA	NA	1
1969	0.583925642	NA	NA	1
6780	0.503736112	902	1000	1
800	0.524128686	NA	NA	1
1830	0.528372093	NA	NA	1
19791	0.559913896	NA	NA	1
1508	0.456569184	NA	NA	1
1483	0.485232068	NA	NA	1
2785	0.454931571	475	941	1
5003	0.555916775	1000	1000	1
2752	0.502751572	792	1000	1
2884	0.523479295	939	1000	1
3857	0.511683439	215	871	0
1095	0.467695275	NA	NA	1
2401	0.500796601	NA	NA	1
1565	0.457507987	NA	NA	1
6986	0.527531527	431	1000	0
684	0.529126214	NA	NA	1
2483	0.548317977	NA	NA	0
14225	0.534696951	104	941	1
1887	0.544780074	NA	NA	1
1418	0.471195185	NA	NA	1
1512	0.573852295	NA	NA	1
982	0.491841492	NA	NA	1
34452	0.545042398	162	1000	0
1143	0.523184602	NA	NA	1
1774	0.519261637	NA	NA	1
13286	0.538670695	476	1000	1
2236	0.519569472	879	929	1
1684	0.472817564	NA	NA	0
948	0.475428571	NA	NA	1
3314	0.533239038	111	928	1
10112	0.546961326	656	938	0
1318	0.475728155	NA	NA	1
9052	0.494528907	432	1000	0
476	0.496453901	NA	NA	1
1695	0.506278916	NA	NA	1
1119	0.528150134	NA	NA	1
636	0.537735849	NA	NA	1
1488	0.521538462	NA	NA	1
2289	0.54002809	912	1000	1
588	0.481765835	NA	NA	1
938	0.432727273	NA	NA	1

3247	0.532258065	NA	NA		1
1813	0.47239613	NA	NA		1
3173	0.541616197	NA	NA		0
2181	0.518255097	1000		928	1
871	0.461048159	NA	NA		1
1426	0.488617201	NA	NA		1
1722	0.474433986	NA	NA		1
2356	0.525896414	NA	NA		1
3777	0.474245115	943		1000	1
15238	0.463295161	NA	NA		1
1673	0.539653036	NA	NA		1
1258	0.485	NA	NA		1
1400	0.514001245	NA	NA		1
5754	0.453868195	NA	NA		1
1780	0.606799704	NA	NA		1
2476	0.493286922	NA	NA		1
11153	0.587048832	NA	NA		1
1390	0.505755396	NA	NA		1
1255	0.540239044	NA	NA		1
735	0.491156463	NA	NA		1
3264	0.54712813	NA	NA		0
1234	0.504051864	NA	NA		1
750	0.427325581	NA	NA		1
4569	0.549300156	NA	NA		0
871	0.451205511	NA	NA		1
3388	0.520933425	891		929	1
2992	0.532620321	NA	NA		1
1034	0.478723404	NA	NA		1
8590	0.523269012	272		1000	1
6438	0.447251937	NA	NA		1
841	0.472057075	NA	NA		1
1039	0.543726236	NA	NA		1
3566	0.557176891	1000		937	1
1386	0.553014553	NA	NA		1
7214	0.510292524	NA	NA		1
7963	0.613230461	442		462	1
1181	0.520047876	NA	NA		1
2075	0.471408647	NA	NA		1
1362	0.502568493	NA	NA		0
1215	0.457217505	NA	NA		1
690	0.54601227	NA	NA		1
3600	0.50636436	NA	NA		1
650	0.510769231	NA	NA		1
8613	0.541174427	49		856	1
3495	0.542447798	1000		1000	1
746	0.477211796	NA	NA		1
790	0.460759494	NA	NA		1
1693	0.404425323	NA	NA		1
1721	0.463191153	NA	NA		1
23777	0.476991818	NA	NA		1
3370	0.487692308	NA	NA		0
934	0.438972163	NA	NA		1
1924	0.575098814	NA	NA		1
2219	0.523611111	NA	NA		1
6161	0.506082725	NA	NA		1
952	0.555699482	NA	NA		1
5361	0.479224181	303		1000	0

3112	0.518356349	929	1000	1
612	0.463955638	NA	NA	1
702	0.475961538	NA	NA	1
3376	0.535433071	748	1000	1
4911	0.516651534	739	1000	0
3107	0.512825458	NA	NA	0
3620	0.500718276	829	1000	0
12278	0.493333333	NA	NA	1
1115	0.495643756	NA	NA	1
3408	0.476898353	NA	NA	1
1774	0.561244329	NA	NA	1
3438	0.55467075	827	1000	1
8796	0.55521327	1000	935	1
1981	0.491467577	NA	NA	1
1480	0.543918919	NA	NA	1
2027	0.521596859	NA	NA	1
1651	0.47238806	NA	NA	1
9423	0.507312749	204	1000	0
3016	0.431089744	NA	NA	0
2651	0.55024255	883	863	0
819	0.472527473	NA	NA	1
557	0.440166493	NA	NA	1
867	0.528781794	NA	NA	1
6158	0.45888186	691	1000	1
2187	0.482395976	NA	NA	1
977	0.496696035	NA	NA	1
11973	0.497339901	1000	1000	0
3081	0.56547619	760	942	1
1623	0.469000729	NA	NA	1
4903	0.507761966	NA	NA	1
4154	0.458577406	NA	NA	0
1229	0.522746781	NA	NA	1
1202	0.498336106	NA	NA	1
21725	0.505505336	194	711	0
2728	0.455278592	1000	1000	1
2567	0.494667235	NA	NA	1
2035	0.425061425	1000	1000	1
2165	0.514763015	NA	NA	0
2291	0.50262697	NA	NA	1
6132	0.516540917	NA	NA	1
3138	0.447299814	NA	NA	1
1896	0.476553341	NA	NA	1
925	0.444672131	NA	NA	0
752	0.430851064	NA	NA	1
2213	0.504896627	NA	NA	1
813	0.464713715	NA	NA	1
1464	0.465163934	NA	NA	1
2333	0.48447205	950	1000	1
1994	0.484004128	NA	NA	1
2240	0.600852273	935	937	1
1280	0.520958084	NA	NA	1
1104	0.444839858	NA	NA	0
1712	0.541564792	NA	NA	1
2814	0.529446936	NA	NA	1
2513	0.514240146	1000	1000	0
2282	0.556162246	NA	NA	1
4169	0.477833978	574	339	1

684	0.439922481 NA	NA		1
1246	0.501605136 NA	NA		1
1025	0.407804878 NA	NA		1
3706	0.526751812 NA	NA		0
7747	0.545995852	1000	871	1
3059	0.593434343 NA	NA		1
1268	0.552313883 NA	NA		1
1115	0.502012072 NA	NA		1
3162	0.4889883 NA	NA		1
2218	0.503842299 NA	NA		0
38901	0.492747651	833	932	0
3225	0.500493908	874	1000	1
1709	0.487893462 NA	NA		1
33731	0.462378213 NA	NA		1
3292	0.523568491	1000	935	1
1035	0.520624303 NA	NA		1
1358	0.490780142 NA	NA		1
9812	0.483714234	559	1000	0
1250	0.4688 NA	NA		1
3985	0.576962283	1000	632	1
6277	0.456890459 NA	NA		1
1324	0.485258964 NA	NA		1
1951	0.53622422 NA	NA		0
1138	0.449261993 NA	NA		1
910	0.568270481 NA	NA		1
2286	0.579954442	909	1000	1
589	0.448217317 NA	NA		1
1240	0.564806867 NA	NA		1
772	0.517799353 NA	NA		1
1514	0.435259693 NA	NA		1
13785	0.472233649 NA	NA		0
961	0.465270121 NA	NA		1
1066	0.480237154 NA	NA		1
1567	0.535803497 NA	NA		0
847	0.516380655 NA	NA		1
940	0.428240741 NA	NA		1
17848	0.444625407	119	1000	0
1352	0.555815769 NA	NA		1
5000	0.532709478	926	974	1
3625	0.554511857	817	1000	1
727	0.523238381 NA	NA		1
2224	0.505073801 NA	NA		1
2329	0.504351809 NA	NA		1
2034	0.511307768	1000	1000	1
8186	0.436546841	187	1000	1
7216	0.482534402	412	1000	1
10937	0.493540052	743	1000	1
3590	0.524940618	535	992	1
2731	0.483304795	605	1000	1
973	0.446776612 NA	NA		1
4238	0.474603175	189	1000	1
978	0.408588957 NA	NA		1
52725	0.55771372 NA	NA		1
4748	0.473563218	456	1000	1
24063	0.529509155 NA	NA		1
585	0.511111111 NA	NA		1
3238	0.482275711 NA	NA		1

23294	0.523940887	668	1000	1
5987	0.54244568	209	923	1
1132	0.519230769	NA	NA	1
2218	0.523589269	NA	NA	1
1739	0.513240858	NA	NA	1
1765	0.513027295	NA	NA	1
4768	0.488366211	496	1000	0
4100	0.579930228	NA	NA	0
4101	0.583100168	NA	NA	0
1346	0.562252181	NA	NA	1
413	0.619854722	NA	NA	1
696	0.556034483	NA	NA	1
2597	0.514687101	NA	NA	1
5612	0.445385267	22	773	1
4236	0.534795975	356	1000	1
2510	0.533026756	NA	NA	1
999	0.473572939	NA	NA	1
1688	0.552132701	NA	NA	1
1943	0.563561503	NA	NA	1
1865	0.492546213	NA	NA	1
770	0.461038961	NA	NA	1
2237	0.567696719	NA	NA	0
3715	0.48013468	941	678	1
2530	0.524687294	NA	NA	1
1206	0.481175864	NA	NA	0
2315	0.458761179	NA	NA	1
1607	0.547501759	NA	NA	1
1092	0.497115385	NA	NA	1
725	0.492957746	NA	NA	1
1417	0.537755822	NA	NA	1
1001	0.547452547	NA	NA	1
4865	0.472956531	888	1000	1
1404	0.504467912	NA	NA	1
6515	0.523080439	NA	NA	1
976	0.494456763	NA	NA	1
3313	0.515826495	886	1000	1
1987	0.522216127	NA	NA	1
1366	0.47440273	NA	NA	1
2613	0.466746197	NA	NA	0
1393	0.510409189	NA	NA	1
1550	0.457102672	NA	NA	1
5355	0.51416122	411	938	1
2550	0.407317915	NA	NA	0
949	0.453108535	NA	NA	1
9800	0.482487491	81	1000	1
2604	0.449286641	671	909	0
3371	0.458608628	554	1000	1
877	0.406674907	NA	NA	1
4839	0.518229167	578	1000	1
1632	0.494490358	NA	NA	1
958	0.514412417	NA	NA	0
954	0.405660377	NA	NA	1
3202	0.434220679	NA	NA	0
2520	0.434695912	NA	NA	1
1115	0.518032787	NA	NA	1
701	0.493307839	NA	NA	1
3866	0.487032202	NA	NA	0

6614	0.491597662	NA	NA		0
1472	0.555555556	NA	NA		1
3041	0.540778839		935	861	1
8709	0.520669133		38	870	0
2517	0.461237723	NA	NA		0
1700	0.518427518	NA	NA		1
3951	0.472460559		851	1000	1
25451	0.466318753		575	1000	0
1072	0.532649254	NA	NA		1
2657	0.54473212	NA	NA		0
6559	0.546685674	NA	NA		1
1564	0.416666667	NA	NA		1
616	0.479257642	NA	NA		0
1793	0.528968714	NA	NA		1
4209	0.467496542	NA	NA		1
681	0.528634361	NA	NA		1
1019	0.529045643	NA	NA		1
1500	0.506472492	NA	NA		1
1007	0.564334086	NA	NA		1
1198	0.537430168	NA	NA		1
900	0.60197775	NA	NA		1
15014	0.532885906	NA	NA		1
3070	0.513684913		926	1000	1
1139	0.548319328	NA	NA		1
1889	0.455855856	NA	NA		1
1781	0.51596424	NA	NA		1
4636	0.470728019		614	1000	1
3811	0.436418479		226	1000	0
1094	0.550259067	NA	NA		1
1300	0.536685903	NA	NA		1
2082	0.49500998		922	1000	1
2079	0.552443712	NA	NA		1
14797	0.517159037		362	927	0
8834	0.470097803		903	930	0
13055	0.521619697		421	1000	0
1282	0.464435146	NA	NA		0
1015	0.510954064	NA	NA		0
1900	0.436575386	NA	NA		0
828	0.511737089	NA	NA		1
1777	0.503095104	NA	NA		1
508	0.468503937	NA	NA		1
12277	0.528598307	NA	NA		1
11705	0.523559376		352	1000	1
46002	0.518342936		143	1000	0
1642	0.459140691	NA	NA		1
9181	0.505391304		760	1000	0
2072	0.422752111	NA	NA		1
3287	0.490017212		120	841	0
1873	0.49202454	NA	NA		1
812	0.54679803	NA	NA		1
522	0.603448276	NA	NA		1
1650	0.47696477	NA	NA		1
1208	0.551003344	NA	NA		1
1396	0.567901235	NA	NA		0
3746	0.495674016	NA	NA		1
1171	0.533753375	NA	NA		1
935	0.546628407	NA	NA		1

2677	0.487140062		801	860	1
1429	0.484693878	NA	NA		1
4683	0.446148929		769	992	1
2258	0.479975728	NA	NA		1
531	0.529190207	NA	NA		1
6757	0.501546711		198	1000	1
1325	0.542544229	NA	NA		1
2957	0.550461361	NA	NA		1
2254	0.496138996	NA	NA		0
1406	0.539299611	NA	NA		1
1460	0.501907735	NA	NA		0
2429	0.56358885	NA	NA		1
1773	0.568741894	NA	NA		1
1228	0.517391304	NA	NA		1
1521	0.54532056	NA	NA		1
9057	0.564921513		659	847	0
1254	0.488452656	NA	NA		1
4391	0.535211268		639	762	1
4560	0.551242913		745	811	1
2661	0.526493799	NA	NA		1
1785	0.514869888	NA	NA		1
4253	0.559738134		409	869	0
3766	0.492375081		231	954	0
1713	0.515436242	NA	NA		1
742	0.472312704	NA	NA		1
1311	0.543668122	NA	NA		1
2528	0.536494406	NA	NA		1
32689	0.487709974		168	1000	1
1397	0.527843137	NA	NA		1
3644	0.54630371		305	842	1
1077	0.441039926	NA	NA		1
408	0.6	NA	NA		1
1405	0.487544484	NA	NA		1
581	0.576592083	NA	NA		1
10888	0.500992392		117	1000	1
1303	0.568327974	NA	NA		1
1051	0.547931382	NA	NA		1
1775	0.552911709	NA	NA		1
680	0.527941176	NA	NA		1
9749	0.570254725		1000	920	1
2227	0.484892788	NA	NA		1
406	0.42042042	NA	NA		1
2064	0.505558497	NA	NA		1
1850	0.442162162	NA	NA		1
5309	0.495404717		800	1000	1
1558	0.522650439	NA	NA		1
10199	0.50068362	NA	NA		0
2170	0.569055529	NA	NA		1
1532	0.571266326	NA	NA		0
1866	0.50975754	NA	NA		1
8888	0.515459723	NA	NA		1
900	0.603821656	NA	NA		1
595	0.537815126	NA	NA		1
637	0.459968603	NA	NA		1
2041	0.529351536	NA	NA		1
788	0.408719346	NA	NA		1
2959	0.553521127		956	966	1

1847	0.48890092	NA	NA		1
3856	0.55401502	NA	NA		1
2159	0.567851373	NA	NA		1
816	0.453431373	NA	NA		1
5720	0.462244405	314		1000	1
2816	0.596796985	617		859	1
1683	0.588344699	NA	NA		1
5782	0.556008147	528		809	1
2238	0.562576687	NA	NA		1
3093	0.54379085	1000		643	0
15201	0.540063091	605		771	0
118730	0.548089659	469		1000	1
929	0.484391819	NA	NA		1
4491	0.55905307	661		960	1
1110	0.581081081	NA	NA		1
22784	0.555919887	NA	NA		0
711	0.503516174	NA	NA		1
9424	0.524721181	411		1000	0
1340	0.572609209	NA	NA		1
2949	0.537346221	810		872	1
2048	0.51167253	NA	NA		1
6116	0.474266906	NA	NA		0
4092	0.566801619	NA	NA		1
4092	0.566521911	NA	NA		0
2302	0.569222851	NA	NA		1
3580	0.564005866	NA	NA		1
3493	0.508629111	657		1000	1
1135	0.505894962	NA	NA		1
844	0.494871795	NA	NA		1
1935	0.550042772	NA	NA		1
2272	0.560107817	NA	NA		0
2258	0.522474625	NA	NA		1
7200	0.574246371	228		595	1
3797	0.517850703	NA	NA		0
1253	0.574887892	NA	NA		1
5782	0.597498366	778		1000	1
7164	0.569441206	780		843	1
996	0.533557047	NA	NA		1
7383	0.482356159	461		1000	0
4583	0.578208832	224		908	1
7389	0.619721197	NA	NA		1
3360	0.463515574	943		1000	1
1406	0.567759079	NA	NA		1
1939	0.589798087	NA	NA		1
1312	0.522252498	NA	NA		1
3321	0.471783296	NA	NA		1
2440	0.59258085	NA	NA		1
5399	0.589826143	NA	NA		1
759	0.534914361	NA	NA		1
711	0.463620981	NA	NA		1
1447	0.588863463	NA	NA		1
17770	0.603448276	1000		933	1
1268	0.616014559	NA	NA		1
777	0.551401869	NA	NA		1
1044	0.518404908	NA	NA		1
2423	0.553858853	NA	NA		1
2065	0.541360024	NA	NA		1

4137	0.596406161		812		735	1
2117	0.535951327	NA		NA		0
40740	0.517133956	NA		NA		1
3609	0.511797512		1000		666	1
2508	0.44191344	NA		NA		1
6232	0.451106232	NA		NA		0
3496	0.551677852	NA		NA		1
1409	0.476224273	NA		NA		1
192	0.557291667	NA		NA		1
2588	0.454936199		943		1000	1
4429	0.512616202		1000		811	1
2499	0.525233645		1000		741	1
3304	0.45829486		959		1000	1
3763	0.469444444	NA		NA		1
1092	0.491503268	NA		NA		1
4153	0.516287215	NA		NA		1
24931	0.43798752	NA		NA		0
2856	0.476886606	NA		NA		1
1505	0.536842105	NA		NA		1
3175	0.51541019		457		884	0
1269	0.429065744	NA		NA		1
843	0.507710558	NA		NA		1
1173	0.477408355	NA		NA		1
13952	0.479098168	NA		NA		1
9937	0.514048652	NA		NA		1
643	0.600311042	NA		NA		1
3873	0.424778761	NA		NA		1
12671	0.525383104	NA		NA		0
1755	0.525800131	NA		NA		1
1268	0.522514868	NA		NA		1
7243	0.479314947	NA		NA		0
5058	0.528458987	NA		NA		0
3923	0.522331567		622		739	1
26826	0.519529006	NA		NA		0
960	0.470833333	NA		NA		1
1956	0.467680608	NA		NA		1
3233	0.450771056	NA		NA		1
4455	0.420585487		549		1000	0
1436	0.41436865	NA		NA		1
1761	0.466160658	NA		NA		1
1124	0.418149466	NA		NA		1
4896	0.439244734		1000		938	0
919	0.50054407	NA		NA		1
1783	0.434985423	NA		NA		1
41305	0.46744868	NA		NA		0
1638	0.443802521	NA		NA		1
999	0.486636971	NA		NA		1
2555	0.447807528	NA		NA		0
3118	0.484069887	NA		NA		1
19174	0.52745377	NA		NA		1
7127	0.487149637	NA		NA		1
2539	0.478278858	NA		NA		1
8368	0.453712026	NA		NA		0
893	0.525195969	NA		NA		1
968	0.537190083	NA		NA		1
5587	0.492419099		1000		1000	1
1209	0.473945409	NA		NA		1

1544	0.5003367	NA	NA		1
3203	0.533817348	607	1000		1
16108	0.471498182	NA	NA		0
913	0.545667447	NA	NA		1
2510	0.564595258	936	1000		1
2303	0.527138515	NA	NA		1
4380	0.530784031	789	1000		1
1200	0.5325	NA	NA		1
1444	0.427573358	NA	NA		0
1618	0.419384474	NA	NA		1
2538	0.371158392	1000	1000		1
11534	0.538227334	195	1000		0
455	0.597802198	NA	NA		1
2638	0.523789822	NA	NA		1
1010	0.521782178	NA	NA		1
1400	0.44137416	NA	NA		1
1442	0.467438495	NA	NA		1
2920	0.512363352	NA	NA		1
1533	0.52902805	NA	NA		1
1394	0.535130719	NA	NA		1
900	0.488461538	NA	NA		1
1498	0.5694315	NA	NA		1
36633	0.518768046	NA	NA		0
793	0.436046512	NA	NA		0
1411	0.550038491	NA	NA		1
2466	0.532786885	NA	NA		1
19417	0.534195635	NA	NA		1
1153	0.52893401	NA	NA		1
698	0.509404389	NA	NA		1
1993	0.493968983	NA	NA		1
4486	0.525382998	886	876		1
4749	0.51022864	NA	NA		0
1725	0.521582734	NA	NA		1
1611	0.550226831	NA	NA		1
11051	0.515413737	456	592		0
740	0.45	NA	NA		1
1010	0.520618557	NA	NA		1
688	0.483306836	NA	NA		1
1065	0.504459861	NA	NA		1
1140	0.495875344	NA	NA		1
1014	0.454352442	NA	NA		1
2306	0.492016679	NA	NA		0
2069	0.527248851	NA	NA		1
689	0.40203193	NA	NA		1
2290	0.533453076	NA	NA		1
1156	0.458292444	NA	NA		1
8148	0.506212609	510	986		1
1125	0.557333333	NA	NA		1
999	0.476517755	NA	NA		1
931	0.432494279	NA	NA		1
1090	0.472477064	NA	NA		1
59296	0.498861504	NA	NA		0
1288	0.512104283	NA	NA		1
4520	0.564044306	650	904		1
924	0.483394834	NA	NA		1
2295	0.508227551	NA	NA		1
677	0.548005908	NA	NA		1

20419	0.538468756	NA	NA		0
1310	0.470284238	NA	NA		1
7826	0.498005751		731	664	1
1791	0.470040721	NA	NA		1
2460	0.511639185	NA	NA		0
2998	0.479696395		753	1000	1
1333	0.464788732	NA	NA		1
1292	0.411982705	NA	NA		0
2037	0.531013616	NA	NA		1
875	0.456	NA	NA		1
778	0.492659054	NA	NA		1
753	0.470119522	NA	NA		1
868	0.503456221	NA	NA		1
724	0.463461538	NA	NA		1
46489	0.557819598	NA	NA		1
1373	0.537203598	NA	NA		1
2099	0.560464079	NA	NA		0
1009	0.456453305	NA	NA		1
10368	0.422811576		459	1000	0
4411	0.531291611	NA	NA		1
936	0.466591166	NA	NA		1
1964	0.53750775	NA	NA		1
840	0.49010293	NA	NA		0
3922	0.442519685	NA	NA		1
1138	0.540665434	NA	NA		1
1144	0.513111888	NA	NA		1
1063	0.471022129	NA	NA		1
3392	0.546915623	NA	NA		0
1843	0.506952491	NA	NA		1
4905	0.412554895		468	1000	0
1284	0.528037383	NA	NA		1
1284	0.528037383	NA	NA		1
3696	0.518395657		930	938	1
920	0.50058072	NA	NA		1
769	0.508757298	NA	NA		0
893	0.512135922	NA	NA		1
1993	0.548704663	NA	NA		1
1425	0.453098827	NA	NA		1
14267	0.531367393	NA	NA		1
1961	0.463029067	NA	NA		1
2945	0.522420313	NA	NA		1
1134	0.579492004	NA	NA		1
2515	0.476645435	NA	NA		1
1925	0.494208494	NA	NA		1
1111	0.526666667	NA	NA		1
1114	0.511494253	NA	NA		1
1366	0.448355011	NA	NA		1
998	0.52004008	NA	NA		1
2297	0.518580144	NA	NA		1
1142	0.4970964	NA	NA		1
2001	0.496360582	NA	NA		0
1369	0.467631379	NA	NA		1
885	0.497175141	NA	NA		1
1714	0.475750577	NA	NA		1
2999	0.522677396	NA	NA		1
1861	0.474741676	NA	NA		1
3205	0.517156863		885	872	0

2657	0.521039604	886	881	1
1366	0.395219124	NA	NA	1
1739	0.51869455	NA	NA	0
3139	0.520083828	957	825	1
1911	0.535845107	NA	NA	1
1383	0.504699928	NA	NA	1
1651	0.534090909	NA	NA	0
675	0.493311037	NA	NA	1
454	0.488431877	NA	NA	1
2370	0.411040146	880	954	1
3462	0.491157703	719	939	1
3740	0.529113924	643	775	1
2397	0.495657209	NA	NA	1
2887	0.551232877	NA	NA	1
1947	0.505831799	NA	NA	1
1977	0.490066225	NA	NA	1
2145	0.521785906	NA	NA	1
804	0.462365591	NA	NA	1
22183	0.475903614	NA	NA	0
590	0.452542373	NA	NA	1
1146	0.53987069	NA	NA	1
10294	0.515591008	NA	NA	1
2425	0.530309278	1000	1000	1
2294	0.502908277	1000	1000	1
2456	0.463689482	NA	NA	1
2931	0.477388911	NA	NA	1
592	0.446969697	NA	NA	1
451	0.43452381	NA	NA	1
484	0.470449173	NA	NA	1
375	0.425	NA	NA	1
422	0.450276243	NA	NA	1
631	0.45800317	NA	NA	1
641	0.409722222	NA	NA	1
595	0.420664207	NA	NA	1
3437	0.486125177	NA	NA	0
23895	0.491147478	327	1000	0
15342	0.467856179	151	1000	0
3712	0.491096272	546	933	0
687	0.458515284	NA	NA	1
566	0.475265018	NA	NA	1
1310	0.51372549	NA	NA	1
8562	0.477272727	325	933	0
854	0.441509434	NA	NA	1
1620	0.486768802	NA	NA	1
1082	0.504750594	NA	NA	1
701	0.478405316	NA	NA	1
33419	0.585847195	NA	NA	1
557	0.497307002	NA	NA	1
2034	0.52173913	NA	NA	1
1912	0.52062975	NA	NA	1
1338	0.498435055	NA	NA	1
1434	0.442753623	NA	NA	1
2823	0.524232334	NA	NA	1
483	0.48447205	NA	NA	1
3475	0.522248244	941	1000	1
1475	0.535150079	NA	NA	1
1444	0.474376731	NA	NA	1

1738	0.491394659	NA	NA	1
2046	0.445614035	NA	NA	1
59449	0.450524738	NA	NA	1
2028	0.538997677	NA	NA	1
1306	0.5237691	NA	NA	1
4455	0.500998668	153	945	1
2664	0.53293562	673	912	0
2247	0.505200208	NA	NA	1
5207	0.5396735	178	1000	0
1752	0.523401826	NA	NA	1
980	0.538461538	NA	NA	1
2002	0.502123142	NA	NA	1
2499	0.481589958	NA	NA	1
2991	0.475310034	NA	NA	0
3536	0.50108972	798	948	0
1558	0.546493902	NA	NA	1
1550	0.542109256	NA	NA	1
1105	0.483685221	NA	NA	1
1213	0.496964441	NA	NA	1
1984	0.514616935	NA	NA	1
1307	0.525528169	NA	NA	1
1719	0.502570694	NA	NA	1
1302	0.485483871	NA	NA	1
856	0.504801097	NA	NA	1
2076	0.528956865	1000	1000	0
895	0.512544803	NA	NA	1
1047	0.494335736	NA	NA	1
2764	0.496226415	NA	NA	1
4065	0.482245732	NA	NA	0
5098	0.465522195	NA	NA	0
3060	0.499229245	NA	NA	0
1730	0.464419476	NA	NA	1
426	0.453051643	NA	NA	1
2260	0.454424779	NA	NA	1
21435	0.450068556	170	1000	1
1419	0.539112051	NA	NA	1
2387	0.493538462	NA	NA	1
715	0.494604317	NA	NA	1
2038	0.452205882	NA	NA	1
1985	0.488861386	NA	NA	1
2336	0.498303167	NA	NA	1
1323	0.435920578	NA	NA	1
801	0.49380805	NA	NA	1
2064	0.40842598	NA	NA	1
3952	0.522822665	1000	929	1
2757	0.576621924	NA	NA	1
11258	0.528287735	732	1000	1
905	0.444785276	NA	NA	1
608	0.433678269	NA	NA	1
7604	0.515519117	233	914	0
3218	0.499308437	NA	NA	1
1127	0.499556344	NA	NA	1
997	0.50737798	NA	NA	1
2597	0.472752678	NA	NA	1
1671	0.52363854	NA	NA	1
1576	0.559010152	NA	NA	1
898	0.550239234	NA	NA	1

2320	0.507779349		927	932	1
2516	0.448106811		843	976	0
1127	0.542147294	NA	NA		1
1006	0.495278069	NA	NA		1
1241	0.472434266	NA	NA		1
1537	0.576810286	NA	NA		1
1266	0.53019024	NA	NA		1
1804	0.541019956	NA	NA		1
14790	0.51269217	NA	NA		1
7990	0.541693189		133	1000	1
586	0.514124294	NA	NA		1
5200	0.555915721	NA	NA		0
1160	0.502579979	NA	NA		1
2776	0.462982005		421	1000	0
1591	0.50282841	NA	NA		1
7570	0.48241123		426	870	1
35390	0.488320356	NA	NA		1
3589	0.553768844	NA	NA		1
31955	0.479061609	NA	NA		1
1453	0.520143885	NA	NA		1
1776	0.559697146	NA	NA		1
3464	0.548290598		271	879	1
594	0.525519849	NA	NA		1
5755	0.48300578		650	943	1
1155	0.453410183	NA	NA		1
2362	0.531610855		951	1000	1
1196	0.530936455	NA	NA		1
1155	0.536527353	NA	NA		1
9945	0.536947275		201	1000	0
3537	0.4114623	NA	NA		0
1595	0.526742301	NA	NA		1
8554	0.542486918		796	81	1
906	0.484547461	NA	NA		1
2476	0.553345036	NA	NA		0
1450	0.55317521	NA	NA		0
968	0.522502744	NA	NA		1
760	0.455462185	NA	NA		1
857	0.500669344	NA	NA		1
1284	0.469733656	NA	NA		1
2267	0.548434804		936	873	1
774	0.535410765	NA	NA		1
1874	0.552616108	NA	NA		1
1462	0.479158551	NA	NA		1
857	0.532088681	NA	NA		1
931	0.561784897	NA	NA		1
3030	0.44984917	NA	NA		1
2947	0.544594595		677	815	0
2535	0.541557763	NA	NA		1
703	0.510046368	NA	NA		1
1530	0.516326531	NA	NA		1
2225	0.41752809	NA	NA		1
6095	0.486477573		508	939	0
4567	0.525244913	NA	NA		1
5145	0.516893374		894	980	1
2151	0.464322382	NA	NA		0
22300	0.532304551	NA	NA		0
1763	0.485596708	NA	NA		1

3184	0.527596846	1000	805	1
1765	0.490651558	NA	NA	1
10423	0.532108613	651	931	1
1412	0.537123746	NA	NA	1
1340	0.499350368	NA	NA	1
1174	0.485249237	NA	NA	1
31759	0.510675982	638	1000	0
10130	0.543166614	671	801	1
1545	0.504388926	NA	NA	1
2095	0.501268392	NA	NA	1
1187	0.49031171	NA	NA	1
1127	0.540262172	NA	NA	1
2558	0.499279885	NA	NA	0
37485	0.569072518	55	940	0
2111	0.434580079	941	1000	1
5204	0.533404868	306	1000	0
7101	0.473608746	NA	NA	0
1403	0.479782271	NA	NA	1
1564	0.477197621	NA	NA	1
3295	0.45520644	NA	NA	1
4158	0.468245021	239	1000	0
14780	0.459622495	1000	1000	1
17742	0.51300905	NA	NA	1
12709	0.542126671	NA	NA	1
3763	0.437387964	274	878	1
2366	0.506072874	918	939	1
7420	0.493644936	NA	NA	0
8695	0.434253916	NA	NA	0
3262	0.53115727	NA	NA	1
1247	0.446790541	NA	NA	1
1296	0.420524691	NA	NA	1
7764	0.421059764	283	799	0
5948	0.5	NA	NA	1
2249	0.465216223	NA	NA	1
1682	0.46676514	NA	NA	1
1575	0.465925926	NA	NA	1
3013	0.456510551	NA	NA	0
2516	0.410057024	NA	NA	1
2977	0.417365565	687	691	1
2989	0.419427037	662	864	1
5519	0.458107736	190	869	1
989	0.54985755	NA	NA	1
1842	0.424414716	NA	NA	0
8283	0.508173034	536	1000	1
10351	0.485016054	232	1000	1
2066	0.494949495	NA	NA	0
7070	0.477775778	342	1000	1
3314	0.486123749	485	871	0
810	0.483016304	NA	NA	1
4644	0.4970569	NA	NA	1
2706	0.531880109	NA	NA	1
1157	0.43545082	NA	NA	1
12875	0.502626552	NA	NA	0
687	0.474380165	NA	NA	1
765	0.526315789	NA	NA	1
977	0.502202643	NA	NA	1
7037	0.507503881	686	870	1

1471	0.433048433	NA	NA		1
1209	0.482189401	NA	NA		1
6966	0.477320307		623	1000	1
25329	0.499169952	NA	NA		0
596	0.450431034	NA	NA		1
26747	0.4878811	NA	NA		1
5940	0.464587321		686	891	1
1618	0.492307692	NA	NA		1
1694	0.466098919	NA	NA		1
3434	0.499220455	NA	NA		1
843	0.50248139	NA	NA		1
957	0.517160686	NA	NA		1
935	0.463382157	NA	NA		1
987	0.502155172	NA	NA		1
1167	0.461538462	NA	NA		1
2667	0.503042035	NA	NA		1
18896	0.507138518		937	1000	0
768	0.5	NA	NA		1
13343	0.53997576		425	720	0
3032	0.456733638	NA	NA		0
962	0.424887892	NA	NA		1
3745	0.426405559	NA	NA		1
1615	0.517137097	NA	NA		1
6770	0.48428556		409	930	1
3738	0.496908558	NA	NA		0
1156	0.442771084	NA	NA		1
819	0.49734748	NA	NA		1
843	0.465753425	NA	NA		0
521	0.452975048	NA	NA		1
4375	0.464022309	NA	NA		0
1174	0.497444634	NA	NA		1
1588	0.468834688	NA	NA		1
14978	0.411451815	NA	NA		1
1197	0.557226399	NA	NA		1
2594	0.507123776	NA	NA		1
822	0.48496732	NA	NA		1
1869	0.507198228	NA	NA		1
905	0.443093923	NA	NA		1
3348	0.464607465		880	1000	1
3170	0.457269322		880	1000	1
3310	0.501630789		879	1000	1
3591	0.528331751		693	1000	1
920	0.443478261	NA	NA		1
2133	0.466533467	NA	NA		0
2357	0.525681492	NA	NA		0
3485	0.499773243	NA	NA		0
5497	0.410907052		229	1000	0
2723	0.437555359		771	1000	1
4176	0.508243174	NA	NA		1
1644	0.440596053	NA	NA		1
773	0.432137285	NA	NA		1
1957	0.490212411	NA	NA		1
1186	0.532040472	NA	NA		1
1192	0.466209598	NA	NA		1
1123	0.471502591	NA	NA		1
955	0.472440945	NA	NA		1
1645	0.524253007	NA	NA		0

2324	0.507042254	NA	NA		1
1660	0.492771084	NA	NA		1
527	0.459203036	NA	NA		1
879	0.474233129	NA	NA		1
9750	0.461917203		62	934	1
2259	0.468854655	NA	NA		1
1333	0.501636661	NA	NA		1
25225	0.471961482		784	977	0
1134	0.436507937	NA	NA		1
18847	0.472457706	NA	NA		0
1850	0.452307692	NA	NA		1
601	0.549905838	NA	NA		1
1614	0.525490196	NA	NA		1
19031	0.495689482		318	1000	0
2308	0.463912394		956	875	0
1914	0.484796328	NA	NA		1
6641	0.466182405		676	943	0
21826	0.483543733		128	867	1
2349	0.528746466		835	938	1
1006	0.459244533	NA	NA		1
685	0.538686131	NA	NA		1
1556	0.481740482	NA	NA		1
1669	0.452631579	NA	NA		1
1343	0.568875652	NA	NA		1
6110	0.522792845	NA	NA		0
1292	0.519052523	NA	NA		0
1137	0.495387454	NA	NA		1
89267	0.450380486		489	455	1
452	0.473282443	NA	NA		1
2165	0.518575851	NA	NA		1
5271	0.474976045		548	873	1
3756	0.475040431		647	1000	1
1266	0.506639428	NA	NA		1
1299	0.458105647	NA	NA		1
1128	0.521393035	NA	NA		1
2124	0.494880546	NA	NA		1
2235	0.481201172	NA	NA		1
1753	0.49754902	NA	NA		1
1728	0.518979465	NA	NA		1
2882	0.509469975	NA	NA		0
872	0.517201835	NA	NA		1
4520	0.505187281	NA	NA		1
4193	0.442499616		1000	947	1
3055	0.491624181	NA	NA		1
23563	0.553611854		50	994	1
1212	0.559405941	NA	NA		1
576	0.522569444	NA	NA		1
10661	0.507392473		369	810	0
1389	0.462203024	NA	NA		1
2205	0.520181406		1000	1000	1
2653	0.403648803	NA	NA		1
848	0.383059418	NA	NA		1
2539	0.484997761		885	937	1
1958	0.473100388	NA	NA		1
1275	0.381889764	NA	NA		1
2044	0.443807339	NA	NA		1
1030	0.424242424	NA	NA		1

2775	0.413577275	NA	NA	1
4304	0.435804702	NA	NA	1
2515	0.489267209	NA	NA	0
2770	0.406652914	NA	NA	1
4528	0.428938671	899	463	1
1630	0.429454545	NA	NA	0
3248	0.48622523	285	859	1
1855	0.385053381	NA	NA	1
1134	0.495609756	NA	NA	1
1946	0.422780705	NA	NA	0
1249	0.456365092	NA	NA	1
2478	0.419446193	NA	NA	0
1979	0.438494935	NA	NA	0
3036	0.471945881	844	879	1
4958	0.427991355	442	871	0
1423	0.467372134	NA	NA	1
2192	0.499070056	NA	NA	0
1156	0.486615679	NA	NA	1
1061	0.478606965	NA	NA	1
1447	0.406041759	NA	NA	1
7354	0.443796444	1000	872	0
4565	0.451525747	50	1000	0
5862	0.430601282	NA	NA	1
3086	0.489688789	NA	NA	1
1717	0.471053464	NA	NA	0
1530	0.509150327	NA	NA	1
802	0.539918809	NA	NA	1
2603	0.529308065	NA	NA	1
2143	0.468869123	NA	NA	1
1827	0.458789205	NA	NA	1
2224	0.391531874	941	937	1
723	0.47164592	NA	NA	1
1933	0.405731119	NA	NA	1
1702	0.497783407	NA	NA	1
3718	0.472345836	936	783	1
2522	0.541315346	1000	1000	1
409	0.577017115	NA	NA	1
641	0.515097691	NA	NA	1
598	0.593645485	NA	NA	1
536	0.569029851	NA	NA	1
1164	0.618556701	NA	NA	1
2816	0.524917552	1000	1000	1
4467	0.450071839	NA	NA	0
1328	0.49789385	NA	NA	1
1102	0.538304392	NA	NA	1
1007	0.531151003	NA	NA	1
559	0.56529517	NA	NA	1
1258	0.514553991	NA	NA	1
3418	0.551057402	NA	NA	1
1731	0.528765352	NA	NA	1
504	0.54813137	NA	NA	0
4872	0.456846673	893	1000	1
556	0.496402878	NA	NA	1
1259	0.441273326	NA	NA	0
1118	0.459510358	NA	NA	1
2543	0.464214712	704	815	1
1270	0.441986234	NA	NA	0

1433	0.487075929	NA	NA		1
760	0.511842105	NA	NA		1
53777	0.513172709	NA	NA		1
1568	0.458333333	NA	NA		1
506	0.434782609	NA	NA		1
5238	0.398005816	NA	NA		1
1785	0.487993139	NA	NA		1
3960	0.497355534	432		1000	0
3560	0.509534229	881		873	1
5514	0.495123781	782		925	1
5690	0.487509146	945		1000	1
1390	0.45323741	NA	NA		1
1365	0.570234114	NA	NA		1
6900	0.468311065	NA	NA		1
2380	0.449864499	1000		834	1
1626	0.46074283	NA	NA		1
928	0.486143187	NA	NA		1
340	0.367647059	NA	NA		1
1263	0.457142857	NA	NA		1
680	0.494117647	NA	NA		1
1390	0.434586466	NA	NA		1
1015	0.488738739	NA	NA		1
1412	0.451980467	NA	NA		1
1672	0.49215317	NA	NA		1
3304	0.535055351	76		1000	1
1944	0.479051819	NA	NA		1
2919	0.520347883	742		786	0
670	0.376666667	NA	NA		1
2765	0.47032967	NA	NA		1
3709	0.509018456	NA	NA		1
1127	0.446317657	NA	NA		1
897	0.517857143	NA	NA		1
1922	0.462133776	NA	NA		1
1591	0.549902153	NA	NA		1
1043	0.535953979	NA	NA		1
1093	0.509375	NA	NA		1
3250	0.455184992	1000		864	1
1480	0.529411765	NA	NA		1
1335	0.516853933	NA	NA		1
1066	0.544090056	NA	NA		1
15537	0.489048265	291		1000	1
2852	0.5	NA	NA		1
2328	0.456563094	NA	NA		1
647	0.447457627	NA	NA		1
1366	0.408918406	NA	NA		1
2439	0.477897252	NA	NA		1
1466	0.517338995	NA	NA		1
765	0.454678363	NA	NA		1
1990	0.530646052	NA	NA		1
1066	0.515608181	NA	NA		1
4448	0.515491999	243		938	1
1890	0.504794521	NA	NA		1
1286	0.469594595	NA	NA		1
1288	0.468944099	NA	NA		1
35842	0.403813662	NA	NA		0
2472	0.444174757	NA	NA		1
2602	0.53621678	NA	NA		1

907	0.478769231	NA		NA	1
1111	0.476571429	NA		NA	1
3880	0.505764506	NA		NA	0
898	0.498886414	NA		NA	1
1723	0.50331525	NA		NA	1
1960	0.51504788	NA		NA	1
792	0.464646465	NA		NA	1
1020	0.489215686	NA		NA	1
3677	0.516973533		1000	935	1
8682	0.469512195	NA		NA	1
1495	0.494934877	NA		NA	1
1040	0.546525324	NA		NA	1
6047	0.515337423		645	876	1
2598	0.535521803		843	733	1
4192	0.44868256		940	942	1
409	0.41809291	NA		NA	1
7141	0.500589362		339	1000	1
7008	0.506575186		46	1000	1
1079	0.484708063	NA		NA	1
838	0.447494033	NA		NA	1
23034	0.508417508	NA		NA	0
1891	0.547403133	NA		NA	1
3020	0.525672372	NA		NA	0
23287	0.5695887		302	889	0
1169	0.526090676	NA		NA	1
5251	0.4797593		480	1000	0
3024	0.511545293		892	811	1
1452	0.485643564	NA		NA	1
993	0.520295203	NA		NA	1
825	0.437575758	NA		NA	1
2273	0.497057492		1000	936	1
12164	0.478081321		586	645	1
535	0.514018692	NA		NA	1
2132	0.552967693	NA		NA	1
1172	0.412190083	NA		NA	1
1652	0.42279709	NA		NA	1
1123	0.485970819	NA		NA	1
707	0.480905233	NA		NA	1
720	0.468085106	NA		NA	1
844	0.558213716	NA		NA	1
23758	0.515762798	NA		NA	0
637	0.411359725	NA		NA	1
2615	0.407648184		1000	1000	1
11900	0.522212378		96	739	1
582	0.491493384	NA		NA	1
909	0.483116883	NA		NA	1
1061	0.515551367	NA		NA	1
3759	0.50861688	NA		NA	1
10303	0.459904892		737	1000	1
3948	0.497890295	NA		NA	1
1955	0.436449227	NA		NA	1
2392	0.436749769		927	943	1
8755	0.488462691	NA		NA	1
1934	0.469363474	NA		NA	1
2123	0.442769666		1000	1000	1
6807	0.50156041	NA		NA	1
1655	0.463796477	NA		NA	1

908	0.534136546	NA	NA		1
36902	0.543672014	NA	NA		1
1166	0.50297619	NA	NA		1
1069	0.450888681	NA	NA		1
704	0.430528376	NA	NA		1
2843	0.4985003	NA	NA		1
1939	0.467576792	NA	NA		1
5853	0.451761745	NA	NA		0
1655	0.471853257	NA	NA		1
1356	0.439123377	NA	NA		1
3106	0.530891602	NA	NA		0
827	0.443432043	NA	NA		1
2315	0.526340326	1000		1000	1
1107	0.498065764	NA	NA		1
794	0.550744249	NA	NA		1
1847	0.518252934	NA	NA		1
3658	0.442926274	NA	NA		1
3486	0.459818731	824		1000	1
2924	0.446358091	863		1000	1
1861	0.466284822	NA	NA		0
851	0.444183314	NA	NA		1
1247	0.502705628	NA	NA		1
11533	0.466538585	NA	NA		0
1820	0.357668364	NA	NA		1
1833	0.444701987	NA	NA		1
674	0.46735905	NA	NA		1
9838	0.463659927	NA	NA		0
1394	0.421216849	NA	NA		1
1079	0.456473214	NA	NA		1
1120	0.465897167	NA	NA		1
1577	0.476690742	NA	NA		1
3635	0.466451802	NA	NA		1
2358	0.491942324	1000		1000	1
11475	0.495450644	NA	NA		1
2257	0.40239726	NA	NA		1
16230	0.463137539	163		1000	1
3942	0.467255717	NA	NA		1
1326	0.498787389	NA	NA		0
1927	0.503211991	NA	NA		1
1130	0.478761062	NA	NA		1
2449	0.490541135	NA	NA		1
2693	0.501178936	NA	NA		1
16805	0.511298353	523		1000	1
450	0.515555556	NA	NA		1
2980	0.507259074	444		943	0
1660	0.551020408	NA	NA		1
5170	0.515444232	1000		1000	1
1219	0.478325859	NA	NA		1
4087	0.4986053	NA	NA		0
2077	0.481132075	NA	NA		1
699	0.436325678	NA	NA		1
2220	0.516975309	NA	NA		1
1689	0.485154769	NA	NA		1
2973	0.42732049	NA	NA		1
3481	0.434782609	NA	NA		1
605	0.425847458	NA	NA		1
1966	0.501164144	NA	NA		1

1353	0.453806356	NA	NA		1
1230	0.435740514	NA	NA		0
4743	0.527257933		1000	1000	1
1402	0.50857569	NA	NA		1
4697	0.472275335	NA	NA		1
4579	0.495407541	NA	NA		1
3016	0.560775541	NA	NA		1
1753	0.476896748	NA	NA		1
866	0.447073474	NA	NA		1
1507	0.510220769	NA	NA		1
1295	0.495551601	NA	NA		1
1004	0.546822742	NA	NA		1
2234	0.508976661	NA	NA		1
2342	0.516627997	NA	NA		1
2816	0.543921767		326	882	0
3021	0.526522226	NA	NA		0
8124	0.531555556	NA	NA		0
5135	0.534132694	NA	NA		0
3949	0.480738363	NA	NA		1
1788	0.560763889	NA	NA		1
3249	0.517981073	NA	NA		1
6515	0.531515261	NA	NA		0
1826	0.48707483	NA	NA		1
1603	0.523316062	NA	NA		1
1202	0.481222707	NA	NA		1
1402	0.487437186	NA	NA		1
1194	0.453662842	NA	NA		1
1281	0.505854801	NA	NA		1
6427	0.516267123		1000	1000	1
8249	0.564779874		492	1000	0
6668	0.561719246	NA	NA		1
914	0.481622307	NA	NA		1
3021	0.500315989	NA	NA		0
1229	0.481318681	NA	NA		1
1497	0.486038394	NA	NA		1
1254	0.496090356	NA	NA		1
1890	0.607616849	NA	NA		1
1199	0.475846501	NA	NA		0
2196	0.539961014		923	933	1
3875	0.446582561		665	922	0
1047	0.407526882	NA	NA		1
16791	0.506257391		137	1000	1
792	0.445355191	NA	NA		1
1298	0.553971487	NA	NA		0
1167	0.531021898	NA	NA		1
1321	0.474658869	NA	NA		1
47799	0.54137931	NA	NA		0
837	0.458781362	NA	NA		1
28459	0.410706988		415	1000	1
2168	0.489527521	NA	NA		1
1585	0.451211526	NA	NA		1
1492	0.512064343	NA	NA		1
759	0.4528	NA	NA		1
2290	0.496322657		915	937	1
1635	0.513149022	NA	NA		0
5518	0.439563365	NA	NA		0
1119	0.46983185	NA	NA		1

14102	0.476237235	303	1000	1
1415	0.496420048	NA	NA	1
611	0.392592593	NA	NA	1
939	0.476038339	NA	NA	1
1556	0.463000679	NA	NA	1
705	0.473767886	NA	NA	1
2791	0.468805704	NA	NA	1
2924	0.46740112	NA	NA	0
1260	0.455555556	NA	NA	1
2343	0.471428571	945	882	1
3159	0.473705667	539	925	0
3666	0.48024787	385	940	1
1408	0.520599251	NA	NA	1
3413	0.493771958	NA	NA	1
16314	0.459837878	NA	NA	0
779	0.463175123	NA	NA	1
3289	0.531900881	NA	NA	0
2750	0.530498282	NA	NA	1
2326	0.507213364	NA	NA	0
2085	0.503692762	NA	NA	1
1718	0.444619629	NA	NA	1
47594	0.418117237	NA	NA	0
3757	0.491391185	386	875	1
1170	0.485416667	NA	NA	1
1427	0.479871176	NA	NA	1
4658	0.468259715	NA	NA	1
12190	0.46321394	NA	NA	1
1435	0.451567944	NA	NA	1
3624	0.468208092	NA	NA	1
7432	0.482989241	NA	NA	1
1921	0.489832536	NA	NA	1
1156	0.487064677	NA	NA	1
1906	0.497315626	NA	NA	0
2452	0.494655836	NA	NA	1
2480	0.480793061	NA	NA	1
2872	0.456614509	NA	NA	1
2524	0.510697306	1000	1000	1
7875	0.491986401	NA	NA	1
18615	0.488103255	302	386	1
23955	0.484125575	NA	NA	0
13671	0.341649049	134	1000	1
1048	0.548664122	NA	NA	1
3726	0.388566516	NA	NA	0
9392	0.423874966	258	938	0
13739	0.493800731	208	807	0
2914	0.473568282	NA	NA	1
1624	0.496305419	NA	NA	1
1090	0.449541284	NA	NA	1
11362	0.42555632	1000	165	1
418	0.413875598	NA	NA	1
1486	0.461754386	NA	NA	1
3226	0.44621958	697	1000	1
8577	0.459731544	NA	NA	1
976	0.427015251	NA	NA	1
8383	0.389616464	919	551	1
1805	0.379501385	NA	NA	1
716	0.32428356	NA	NA	1

1480	0.352320675	NA	NA	1
4430	0.366144201	NA	NA	1
1527	0.47109375	NA	NA	1
936	0.495726496	NA	NA	1
1963	0.536614646	NA	NA	1
3630	0.528650138	NA	NA	1
6334	0.478419731	NA	NA	0
10145	0.50749173	1000	931	1
4025	0.479874776	NA	NA	0
2310	0.471634615	882	888	1
17709	0.536047666	NA	NA	1
1907	0.458281445	NA	NA	1
1179	0.511627907	NA	NA	1
1932	0.501331913	NA	NA	1
4796	0.495682649	762	680	0
1118	0.46882494	NA	NA	1
1748	0.493857494	NA	NA	1
701	0.418212479	NA	NA	1
1162	0.46925859	NA	NA	1
2090	0.569912609	NA	NA	1
1258	0.507154213	NA	NA	1
13243	0.495587806	729	422	0
935	0.452554745	NA	NA	1
2678	0.496385542	NA	NA	1
988	0.530364372	NA	NA	1
1927	0.495704901	NA	NA	1
1927	0.489821484	NA	NA	0
3192	0.495039683	944	1000	1
1453	0.485582468	NA	NA	1
933	0.479087452	NA	NA	0
1166	0.464737794	NA	NA	1
17044	0.499314129	NA	NA	0
1758	0.562456867	NA	NA	1
16171	0.499901865	NA	NA	0
1466	0.530935252	NA	NA	1
1254	0.52680067	NA	NA	1
914	0.467132867	NA	NA	1
1299	0.571208622	NA	NA	1
704	0.499136442	NA	NA	1
3140	0.448013245	1000	1000	1
2282	0.454545455	NA	NA	1
1489	0.453747715	NA	NA	0
2412	0.491244528	NA	NA	0
2645	0.526717557	NA	NA	0
1560	0.521433141	NA	NA	0
2898	0.526399155	NA	NA	1
2860	0.60559191	NA	NA	1
2176	0.494117647	NA	NA	1
3071	0.494423792	NA	NA	1
4321	0.463743676	NA	NA	1
1357	0.504960317	NA	NA	1
1823	0.461597542	NA	NA	1
684	0.471246006	NA	NA	1
2624	0.539982031	NA	NA	1
2055	0.456328818	NA	NA	0
1698	0.42599278	NA	NA	1
1072	0.488669951	NA	NA	1

350	0.493197279	NA	NA		1
1493	0.499665104	NA	NA		1
2385	0.579874214	1000		1000	1
62316	0.526660244	399		1000	1
696	0.408227848	NA	NA		1
498	0.4	NA	NA		1
9596	0.524163906	NA	NA		1
516	0.395143488	NA	NA		1
2193	0.515690867	NA	NA		1
979	0.525308379	NA	NA		0
1932	0.521437579	NA	NA		1
2965	0.522418605	NA	NA		0
1617	0.531849103	NA	NA		1
1714	0.570512821	NA	NA		1
1744	0.554166667	NA	NA		1
2661	0.518292683	941		1000	1
2001	0.485169492	NA	NA		1
2429	0.492105263	NA	NA		0
2379	0.562421185	NA	NA		1
1465	0.528196982	NA	NA		1
15593	0.569473248	598		755	1
13845	0.489663462	NA	NA		1
2334	0.490307868	NA	NA		1
24977	0.51798175	214		1000	0
3687	0.504704301	NA	NA		1
1278	0.512217195	NA	NA		1
1483	0.494596841	NA	NA		1
1511	0.475518672	NA	NA		1
1782	0.529741863	NA	NA		1
777	0.50488145	NA	NA		1
683	0.499108734	NA	NA		1
917	0.486005089	NA	NA		1
991	0.553985873	NA	NA		1
22351	0.505368679	781		1000	0
1842	0.471843003	NA	NA		1
787	0.451612903	NA	NA		1
781	0.441588785	NA	NA		1
1968	0.547764228	NA	NA		1
2240	0.540037785	833		1000	0
1675	0.543695797	NA	NA		1
1437	0.512209302	NA	NA		1
2209	0.479332669	864		944	1
3246	0.496533063	NA	NA		0
13833	0.468641115	NA	NA		1
627	0.475279107	NA	NA		1
2108	0.464824121	NA	NA		1
1821	0.517528223	NA	NA		0
1247	0.547140649	NA	NA		1
3921	0.470092099	NA	NA		1
13110	0.54015374	276		871	0
4636	0.50248089	809		1000	1
3630	0.478745645	NA	NA		1
4055	0.455461687	NA	NA		1
2043	0.525687597	NA	NA		1
2262	0.43078056	NA	NA		0
1846	0.506165919	NA	NA		1
714	0.541925466	NA	NA		1

19628	0.552787382	NA	NA		0
2104	0.511388394	NA	NA		0
1835	0.513685551	NA	NA		1
520	0.484615385	NA	NA		1
817	0.479804162	NA	NA		1
605	0.444628099	NA	NA		1
935	0.547593583	NA	NA		1
767	0.417209909	NA	NA		1
1697	0.541348795	NA	NA		0
15331	0.534357106		282	839	0
2061	0.554305008	NA	NA		1
2101	0.534444444	NA	NA		1
1979	0.508683473	NA	NA		1
395	0.437974684	NA	NA		1
1684	0.496004917	NA	NA		1
1452	0.568518519	NA	NA		1
974	0.595827901	NA	NA		1
2404	0.517995766	NA	NA		1
10806	0.529308975		797	1000	1
2472	0.539385848	NA	NA		1
10482	0.51212904		612	1000	0
1431	0.517819706	NA	NA		1
2310	0.513852814		1000	1000	1
2668	0.466405101		667	820	1
2291	0.483628556	NA	NA		1
2516	0.470043573	NA	NA		1
3033	0.48291652		869	1000	1
5175	0.552654162	NA	NA		1
377	0.517241379	NA	NA		1
807	0.574834437	NA	NA		1
1427	0.479562044	NA	NA		1
2482	0.48321513	NA	NA		1
2559	0.53842958		848	1000	1
1202	0.522462562	NA	NA		1
1087	0.592628516	NA	NA		1
4930	0.508897677		780	816	1
1023	0.518394649	NA	NA		1
5222	0.464798058		823	941	1
722	0.527700831	NA	NA		1
2992	0.493905978	NA	NA		1
493	0.434895833	NA	NA		1
5019	0.523730225	NA	NA		1
16223	0.543557982		632	939	1
6275	0.551504809		490	916	0
1180	0.473963868	NA	NA		1
12017	0.57769241	NA	NA		1
2448	0.591487495		1000	944	1
15057	0.482600601		348	1000	0
1362	0.537267081	NA	NA		1
3281	0.478934102	NA	NA		1
3201	0.415322581	NA	NA		1
3196	0.521481654	NA	NA		0
4584	0.483244681		701	822	1
782	0.497835498	NA	NA		1
2449	0.500628404	NA	NA		1
1768	0.53626943	NA	NA		1
1185	0.495905834	NA	NA		0

2967	0.525608098	NA	NA	1
2576	0.527226137	NA	NA	1
3529	0.474751198	729	872	1
2914	0.477621764	819	905	1
1132	0.486646884	NA	NA	1
5426	0.471671388	820	1000	0
2103	0.514401077	NA	NA	0
2302	0.446844798	NA	NA	1
2070	0.445929526	NA	NA	1
1320	0.494117647	NA	NA	1
2191	0.530460624	NA	NA	1
4107	0.595495495	1000	223	1
769	0.503840246	NA	NA	1
2037	0.490083173	NA	NA	1
6017	0.469796147	1000	1000	0
4421	0.524599329	NA	NA	0
1452	0.555779299	NA	NA	0
566	0.533126294	NA	NA	1
1338	0.570608496	NA	NA	0
11542	0.453165282	NA	NA	0
2160	0.488095238	NA	NA	1
72713	0.495782116	NA	NA	1
1297	0.468766133	NA	NA	0
1152	0.516493056	NA	NA	1
1141	0.478098788	NA	NA	1
4407	0.546681058	947	944	0
8542	0.561337288	200	1000	0
1452	0.479913917	NA	NA	1
1067	0.432989691	NA	NA	1
1385	0.518549747	NA	NA	1
1430	0.46684832	NA	NA	1
2826	0.426031746	NA	NA	1
2756	0.517241379	NA	NA	1
1036	0.502895753	NA	NA	1
959	0.443384982	NA	NA	0
6122	0.515503876	NA	NA	1
1234	0.471636953	NA	NA	1
9107	0.475494673	NA	NA	0
4266	0.534054054	NA	NA	1
2596	0.531807176	833	936	1
4305	0.503295048	NA	NA	1
1836	0.437067773	NA	NA	1
1226	0.488849242	NA	NA	1
4446	0.470461334	NA	NA	1
1405	0.441152922	NA	NA	1
1316	0.462818004	NA	NA	1
11171	0.480940057	NA	NA	0
3171	0.469964664	942	1000	1
594	0.512978986	NA	NA	0
1959	0.514570552	NA	NA	1
5846	0.477977162	NA	NA	1
11148	0.489839795	NA	NA	0
1069	0.486228814	NA	NA	1
3759	0.510906544	718	1000	1
1612	0.436241611	NA	NA	1
3867	0.45716862	NA	NA	1
46498	0.532075166	NA	NA	0

2542	0.524513948		1000	1000	0
1111	0.477947795	NA	NA		1
1402	0.466949153	NA	NA		1
1591	0.46528332	NA	NA		1
5718	0.523155216	NA	NA		1
4588	0.560990712	NA	NA		1
4130	0.555082581	NA	NA		0
38002	0.519005774	NA	NA		0
1020	0.512745098	NA	NA		1
1108	0.467724868	NA	NA		1
1042	0.521077283	NA	NA		1
1495	0.433293269	NA	NA		0
1034	0.517730496	NA	NA		1
688	0.452631579	NA	NA		1
9539	0.511055675	NA	NA		0
2477	0.514888337		941	1000	1
1044	0.444909344	NA	NA		1
1097	0.479069767	NA	NA		1
1727	0.47983871	NA	NA		1
23550	0.494033679		173	868	0
3603	0.520032051		817	866	1
2910	0.485478807		949	804	1
863	0.48783314	NA	NA		1
7256	0.408312469		415	1000	0
456	0.480263158	NA	NA		1
1084	0.446494465	NA	NA		1
1543	0.532728451	NA	NA		1
4984	0.501457726	NA	NA		1
1994	0.473718791	NA	NA		1
1514	0.450921399	NA	NA		0
502	0.503984064	NA	NA		1
481	0.513513514	NA	NA		1
887	0.512965051	NA	NA		1
621	0.444444444	NA	NA		1
451	0.545454545	NA	NA		1
1648	0.51145959	NA	NA		1
815	0.564417178	NA	NA		1
2261	0.470887507	NA	NA		1
1731	0.542568543	NA	NA		1
5600	0.62013676		138	498	1
486	0.511961722	NA	NA		1
1349	0.519440124	NA	NA		1
741	0.538461538	NA	NA		1
3687	0.4476014	NA	NA		0
1946	0.495233051	NA	NA		1
1268	0.436603557	NA	NA		1
3503	0.570330515		794	881	0
1716	0.494142705	NA	NA		1
3765	0.447518743		1000	477	0
2394	0.492641737		806	973	0
5970	0.491847165	NA	NA		1
36952	0.529634898	NA	NA		0
21435	0.511611944		768	1000	0
9356	0.483147131		709	1000	0
2055	0.511257036	NA	NA		0
14084	0.43697479		869	1000	1
1686	0.43772242	NA	NA		1

43626	0.440538817 NA	NA		0
1733	0.490551776 NA	NA		1
2136	0.503964321 NA	NA		1
712	0.599719101 NA	NA		1
1208	0.399377593 NA	NA		1
17675	0.467409898	886	945	1
1537	0.447876448 NA	NA		1
3448	0.524145415 NA	NA		1
1876	0.518656716 NA	NA		1
2744	0.469629202 NA	NA		0
2039	0.535080956 NA	NA		1
2834	0.499678042	890	902	1
2125	0.508138352 NA	NA		1
1343	0.501285347 NA	NA		1
1192	0.503840246 NA	NA		0
8734	0.512844654 NA	NA		1
1101	0.532243415 NA	NA		1
405	0.488888889 NA	NA		1
1734	0.566897347 NA	NA		1
2040	0.527903469 NA	NA		1
1755	0.489374262 NA	NA		1
1810	0.477137177 NA	NA		1
392	0.474489796 NA	NA		1
2388	0.527216174 NA	NA		1
1701	0.466383812 NA	NA		1
1371	0.543981481 NA	NA		1
3459	0.495650129	70	938	0
1220	0.473684211 NA	NA		1
3752	0.4871502 NA	NA		0
7146	0.507509461	882	1000	1
889	0.508547009 NA	NA		0
1744	0.499284283 NA	NA		0
390	0.528205128 NA	NA		1
813	0.525965379 NA	NA		1
1476	0.493964717 NA	NA		1
3091	0.50334984 NA	NA		1
1209	0.475757576 NA	NA		1
2725	0.517791062	771	597	1
2475	0.478317659	770	892	0
1493	0.515965167 NA	NA		1
979	0.506535948 NA	NA		1
2647	0.531311155 NA	NA		1
15582	0.525786632 NA	NA		1
13543	0.479150429 NA	NA		1
855	0.413934426 NA	NA		1
3185	0.463846154 NA	NA		1
2787	0.368874172 NA	NA		1
1182	0.501340483 NA	NA		1
2937	0.494187192	946	800	1
3386	0.502555366 NA	NA		1
10721	0.485255854	331	929	0
6199	0.478583268	938	940	1
2178	0.521918679 NA	NA		1
1381	0.471854305 NA	NA		1
771	0.473254086 NA	NA		1
1454	0.473880597 NA	NA		1
1746	0.496598639 NA	NA		1

20060	0.4738055	409	747	0
2226	0.516178737	NA	NA	1
761	0.537735849	NA	NA	1
3134	0.509598604	850	881	1
2294	0.470121457	887	944	1
3518	0.515678524	NA	NA	1
3939	0.534448328	210	1000	1
1615	0.582781457	NA	NA	1
556	0.482014388	NA	NA	1
22963	0.509490296	455	1000	1
2945	0.537077034	1000	945	1
481	0.511434511	NA	NA	1
3977	0.480929487	NA	NA	1
9719	0.469100761	NA	NA	0
3714	0.436573074	NA	NA	1
2169	0.530935808	NA	NA	1
1028	0.470343392	NA	NA	1
3285	0.542265427	1000	793	1
1194	0.487740806	NA	NA	1
2212	0.502780352	946	1000	1
1285	0.466269841	NA	NA	1
1266	0.546983185	NA	NA	1
1939	0.534615385	NA	NA	1
743	0.451473137	NA	NA	1
4754	0.483230079	NA	NA	1
1885	0.498493068	NA	NA	1
8236	0.507212205	619	1000	1
3437	0.541299985	NA	NA	0
6671	0.506134051	NA	NA	0
2194	0.517613903	NA	NA	1
1323	0.616134752	NA	NA	1
1072	0.454291045	NA	NA	1
10190	0.512951254	NA	NA	0
1450	0.504827586	NA	NA	1
2224	0.489828269	1000	1000	1
506	0.416996047	NA	NA	1
1663	0.522775053	NA	NA	1
1428	0.56281407	NA	NA	0
1188	0.48409894	NA	NA	1
1476	0.537940379	NA	NA	1
1654	0.519756839	NA	NA	1
2145	0.504700643	876	1000	1
844	0.496598639	NA	NA	1
1877	0.564038381	NA	NA	0
1248	0.507892293	NA	NA	1
1231	0.528025995	NA	NA	1
7167	0.475898393	894	1000	0
3113	0.529071635	1000	1000	1
1242	0.49911032	NA	NA	1
4857	0.496504065	838	946	0
1664	0.501364256	NA	NA	1
1859	0.486666667	NA	NA	1
1804	0.493880049	NA	NA	1
1937	0.49793956	NA	NA	1
1252	0.49906015	NA	NA	1
1378	0.48372093	NA	NA	0
2533	0.482605946	NA	NA	0

643	0.514024788	NA	NA	0
2717	0.471833648	928	1000	1
2327	0.518937531	878	881	1
6709	0.520621417	497	907	0
4072	0.443804825	814	938	1
2251	0.440267765	NA	NA	1
1516	0.537941397	NA	NA	1
2368	0.535050676	1000	1000	1
1348	0.525964392	NA	NA	1
882	0.475056689	NA	NA	1
2431	0.521654501	865	885	0
7235	0.498569677	541	650	1
1556	0.504194631	NA	NA	1
2041	0.514453699	1000	1000	1
4254	0.495768688	1000	1000	1
789	0.395604396	NA	NA	1
1518	0.474504249	NA	NA	1
413	0.540123457	NA	NA	1
1177	0.523364486	NA	NA	1
3285	0.471059662	824	936	0
5971	0.486645367	132	1000	1
2952	0.533903421	972	940	0
552	0.533898305	NA	NA	1
766	0.480739599	NA	NA	1
1508	0.527631579	NA	NA	0
1206	0.527363184	NA	NA	1
2061	0.519165454	NA	NA	1
1822	0.453044963	NA	NA	1
1965	0.442176871	NA	NA	1
1927	0.430794886	NA	NA	1
1688	0.491131498	NA	NA	1
4779	0.530667534	NA	NA	0
4626	0.453009636	815	1000	1
1859	0.493127148	NA	NA	1
10060	0.485013624	NA	NA	1
4559	0.484632272	NA	NA	1
1500	0.450952717	NA	NA	1
2000	0.43	NA	NA	1
1833	0.444994694	NA	NA	1
2112	0.411731207	NA	NA	1
3171	0.45016835	NA	NA	0
1681	0.461629982	NA	NA	1
1406	0.509745127	NA	NA	1
860	0.498117942	NA	NA	1
590	0.493220339	NA	NA	1
4100	0.445639188	NA	NA	1
7720	0.586620926	NA	NA	1
12178	0.513341298	NA	NA	1
11256	0.52538913	241	928	1
1800	0.452222222	NA	NA	1
1782	0.455688623	NA	NA	1
6639	0.504502341	469	622	0
3091	0.439099735	473	929	1
1722	0.517152104	NA	NA	1
1598	0.549561699	NA	NA	1
4601	0.48714442	370	1000	0
7040	0.453238165	1000	1000	1

1562	0.472295515	NA	NA		1
1071	0.5	NA	NA		1
4181	0.484975388	NA	NA		0
4387	0.494741874	NA	NA		1
1571	0.52	NA	NA		1
1600	0.519592476	NA	NA		1
985	0.497854077	NA	NA		1
1298	0.46578141	NA	NA		1
3534	0.494522692	NA	NA		0
1489	0.55365622	NA	NA		1
14296	0.522793581		475	909	1
17365	0.475105673		413	1000	1
3298	0.444373402	NA	NA		1
6512	0.493491224		187	944	1
2041	0.472127418	NA	NA		1
3711	0.465646651	NA	NA		1
1528	0.359558316	NA	NA		1
19397	0.480929895	NA	NA		1
1261	0.509154316	NA	NA		1
7066	0.51592284		136	828	0
1385	0.481961669	NA	NA		0
2993	0.505013193	NA	NA		0
18475	0.419000331	NA	NA		0
2615	0.452044025		1000	929	1
999	0.517497349	NA	NA		1
457	0.492341357	NA	NA		1
680	0.410752688	NA	NA		1
1317	0.529992407	NA	NA		1
1863	0.483451537	NA	NA		1
2835	0.541093474		1000	1000	1
4406	0.484136897		875	1000	1
2614	0.494353827		901	886	1
2929	0.513657957	NA	NA		0
1452	0.513774105	NA	NA		1
914	0.478118162	NA	NA		1
3795	0.525131972		863	1000	0
757	0.470277411	NA	NA		1
1005	0.446448703	NA	NA		1
1610	0.487964989	NA	NA		1
712	0.476407915	NA	NA		1
2134	0.521167883		921	1000	1
2137	0.523843757		1000	1000	0
6227	0.41415902		362	1000	0
6692	0.450980392		276	1000	1
4074	0.526698351	NA	NA		1
1139	0.516422082	NA	NA		0
2004	0.477953715		1000	1000	1
2137	0.522703273	NA	NA		1
5196	0.501316945		631	881	1
10184	0.489578714	NA	NA		1
1664	0.490985577	NA	NA		1
17049	0.475344266		594	1000	0
1340	0.462138954	NA	NA		1
513	0.436008677	NA	NA		1
542	0.452674897	NA	NA		1
723	0.478561549	NA	NA		1
20995	0.47700063	NA	NA		0

2402	0.418508623	883	808	1
3267	0.574355222	1000	1000	1
3292	0.494413408	NA	NA	1
987	0.448387097	NA	NA	1
1677	0.491469816	NA	NA	1
567	0.486772487	NA	NA	1
2049	0.466493356	NA	NA	1
7981	0.490605963	482	880	1
3207	0.512958435	719	1000	0
1813	0.504398827	NA	NA	1
2927	0.524825237	1000	1000	1
3770	0.432122093	NA	NA	1
1345	0.41588785	NA	NA	1
1502	0.514797508	NA	NA	1
696	0.461206897	NA	NA	1
447	0.48098434	NA	NA	1
1783	0.429613012	NA	NA	1
1957	0.43697479	NA	NA	1
509	0.469548134	NA	NA	1
2122	0.483710657	NA	NA	1
2301	0.427536232	NA	NA	1
2311	0.427380952	NA	NA	1
3298	0.502418575	930	1000	1
848	0.527122642	NA	NA	1
3241	0.481896552	NA	NA	0
923	0.460648148	NA	NA	1
850	0.44716585	NA	NA	1
2930	0.508517228	826	929	1
8316	0.491821884	725	1000	0
1513	0.48240985	NA	NA	0
2667	0.49279928	NA	NA	1
2003	0.526780088	NA	NA	1
520	0.455769231	NA	NA	1
4458	0.48241031	910	912	1
2143	0.498366776	NA	NA	1
379	0.461741425	NA	NA	1
1335	0.48573898	NA	NA	1
7226	0.459723035	NA	NA	0
1043	0.468839885	NA	NA	1
1744	0.460658083	NA	NA	1
1771	0.49031477	NA	NA	1
6050	0.500477251	NA	NA	0
6016	0.514717425	NA	NA	0
2846	0.508235725	NA	NA	1
1761	0.473594549	NA	NA	1
1205	0.499585062	NA	NA	1
5689	0.420426376	439	932	1
32749	0.445093817	832	1000	1
1127	0.522309711	NA	NA	1
1087	0.587885986	NA	NA	1
922	0.417661098	NA	NA	1
6096	0.501242493	NA	NA	0
2924	0.510259918	1000	1000	1
2024	0.468936679	NA	NA	1
1679	0.517857143	NA	NA	1
568	0.528169014	NA	NA	1
5625	0.415701416	NA	NA	0

900	0.388324873	NA		NA		1
729	0.441515651	NA		NA		1
682	0.444444444	NA		NA		1
723	0.449081803	NA		NA		1
756	0.467304625	NA		NA		1
1033	0.492709904	NA		NA		1
529	0.513978495	NA		NA		1
427	0.407821229	NA		NA		1
347	0.35335689	NA		NA		1
677	0.508880995	NA		NA		1
831	0.495788207	NA		NA		1
4818	0.462371681		144		888	0
3904	0.49623431		942		865	1
2851	0.459817208	NA		NA		1
1469	0.510551396	NA		NA		1
1726	0.497682503	NA		NA		1
8327	0.493418066		369		1000	0
1173	0.502983802	NA		NA		1
807	0.497822932	NA		NA		1
918	0.454664915	NA		NA		1
2636	0.494196429	NA		NA		0
1984	0.461726883	NA		NA		1
17231	0.503566796	NA		NA		0
3134	0.498351467	NA		NA		0
8096	0.476586889		1000		1000	1
2436	0.523652365	NA		NA		1
1100	0.423901941	NA		NA		1
1191	0.434296365	NA		NA		1
2089	0.461279461	NA		NA		1
12688	0.476047455	NA		NA		0
1308	0.557692308	NA		NA		0
7488	0.553236898	NA		NA		0
2149	0.564957265	NA		NA		1
1400	0.448893572	NA		NA		1
1578	0.451274363	NA		NA		1
9043	0.512370886	NA		NA		1
3822	0.511915016		835		976	1
1103	0.481414325	NA		NA		1
1105	0.440191388	NA		NA		1
2172	0.493069307	NA		NA		1
1697	0.532186106	NA		NA		1
506	0.5	NA		NA		1
81102	0.514666906	NA		NA		0
4319	0.527950311		940		1000	1
1745	0.538032787	NA		NA		0
21624	0.569178462	NA		NA		0
896	0.444015444	NA		NA		1
1650	0.485945581	NA		NA		1
10425	0.511239014	NA		NA		0
3658	0.501353383	NA		NA		1
14077	0.535396033		29		462	0
1284	0.490654206	NA		NA		1
2554	0.515006525		928		877	1
1336	0.519245876	NA		NA		1
1013	0.447186575	NA		NA		1
1099	0.485896269	NA		NA		1
2022	0.50148368		1000		1000	1

3246	0.437753721	722	1000	1
13097	0.522949974	NA	NA	1
3754	0.500327869	NA	NA	1
4808	0.489067225	291	804	0
4450	0.488521089	190	815	0
4421	0.460363636	71	868	0
3395	0.4669346	837	866	1
1773	0.48661234	NA	NA	1
560	0.394789579	NA	NA	1
877	0.457507082	NA	NA	1
3019	0.472093023	650	1000	1
1342	0.434707904	NA	NA	1
2619	0.537453184	NA	NA	1
1211	0.506193229	NA	NA	1
3786	0.478090152	925	946	1
741	0.473958333	NA	NA	1
752	0.392287234	NA	NA	1
1163	0.505572442	NA	NA	1
1901	0.445502646	NA	NA	1
1303	0.448	NA	NA	1
612	0.473021583	NA	NA	1
793	0.43852459	NA	NA	1
537	0.495217853	NA	NA	1
651	0.452205882	NA	NA	1
6064	0.496042216	1000	1000	1
519	0.430406852	NA	NA	1
5507	0.489562192	66	1000	0
692	0.473180077	NA	NA	1
830	0.49382716	NA	NA	1
5810	0.545905707	NA	NA	1
4149	0.495439835	NA	NA	0
8199	0.474488366	460	1000	0
1717	0.503785673	NA	NA	1
3455	0.548437297	868	940	1
949	0.543730242	NA	NA	1
1236	0.408450704	NA	NA	0
476	0.444180523	NA	NA	1
866	0.49068323	NA	NA	1
1687	0.466174662	NA	NA	1
438	0.5	NA	NA	1
937	0.509561305	NA	NA	1
1726	0.467281106	NA	NA	1
3174	0.503191489	NA	NA	1
1032	0.460545194	NA	NA	1
4742	0.460546448	NA	NA	1
5317	0.501666374	NA	NA	1
2032	0.476264997	NA	NA	1
698	0.479559748	NA	NA	1
809	0.545698925	NA	NA	1
1834	0.521588402	NA	NA	1
1967	0.471328671	NA	NA	1
854	0.525914634	NA	NA	1
14710	0.515441377	NA	NA	1
4770	0.4965693	325	870	0
1352	0.510994764	NA	NA	1
1676	0.501872659	NA	NA	1
2734	0.480464625	NA	NA	1

1082	0.511784512	NA	NA		1
545	0.466244726	NA	NA		1
4339	0.548915449		605	939	1
2061	0.499433107	NA	NA		1
10357	0.548580104		644	1000	0
1853	0.521316784	NA	NA		1
1007	0.535211268	NA	NA		1
2113	0.489141675	NA	NA		1
730	0.43833581	NA	NA		1
809	0.466763006	NA	NA		1
1567	0.535220994	NA	NA		1
1327	0.549036044	NA	NA		1
894	0.404371585	NA	NA		1
676	0.442463533	NA	NA		1
6946	0.545990742		752	1000	1
1051	0.537583254	NA	NA		1
677	0.474153298	NA	NA		1
883	0.555420219	NA	NA		1
5859	0.461302211	NA	NA		1
12496	0.500226706	NA	NA		0
2319	0.471806047	NA	NA		1
4609	0.521706017		510	944	1
1186	0.477876106	NA	NA		1
1981	0.471983847	NA	NA		1
7166	0.535725165	NA	NA		0
3624	0.530674847		862	730	1
977	0.501762632	NA	NA		1
2658	0.467185221		541	1000	1
3044	0.515701017		521	812	1
3068	0.44968238		923	1000	1
15790	0.509897895		54	1000	0
1278	0.467057101	NA	NA		1
4697	0.515934539	NA	NA		1
6385	0.505373276	NA	NA		1
703	0.555408389	NA	NA		0
2515	0.493322027	NA	NA		0
1530	0.481288077	NA	NA		1
1571	0.499335989	NA	NA		1
10505	0.525055928		58	1000	1
800	0.472346786	NA	NA		1
883	0.467805519	NA	NA		1
1114	0.477809254	NA	NA		1
936	0.467824968	NA	NA		0
604	0.483443709	NA	NA		1
3071	0.475106137		745	940	1
825	0.463030303	NA	NA		1
1807	0.466835123	NA	NA		1
1562	0.487195903	NA	NA		1
2210	0.499555028	NA	NA		1
1800	0.496924969	NA	NA		1
5610	0.53187251	NA	NA		0
2911	0.555312424	NA	NA		0
8238	0.567015364	NA	NA		1
2776	0.506872137		805	873	1
2931	0.513177711		899	1000	1
1936	0.448538012	NA	NA		1
2521	0.536575229	NA	NA		1

5414	0.483449477	NA	NA		1
2949	0.537537538	NA	NA		1
2746	0.524763292		1000	1000	1
3910	0.494155644	NA	NA		1
2411	0.493810179		826	944	1
4888	0.503560529		747	885	0
3539	0.493573265		978	1000	0
39602	0.553672647	NA	NA		0
4203	0.490579843	NA	NA		0
776	0.423936554	NA	NA		1
1441	0.535045108	NA	NA		1
4436	0.470996529		914	983	1
1233	0.469610844	NA	NA		0
5167	0.62672136	NA	NA		1
3990	0.522794245		752	685	0
2014	0.521806054	NA	NA		1
1734	0.491372227	NA	NA		1
982	0.501915709	NA	NA		1
5231	0.475466637		916	901	1
1875	0.545768566	NA	NA		1
1110	0.502109705	NA	NA		0
1404	0.535147392	NA	NA		1
629	0.550442478	NA	NA		1
1955	0.488610478	NA	NA		1
1309	0.542604502	NA	NA		1
1019	0.498148148	NA	NA		0
787	0.488663018	NA	NA		1
2461	0.460279249		616	1000	1
1070	0.528816987	NA	NA		1
930	0.487554905	NA	NA		1
767	0.477929985	NA	NA		1
1124	0.466666667	NA	NA		1
2055	0.456587735	NA	NA		1
1929	0.483452304	NA	NA		1
1911	0.521682848	NA	NA		1
3133	0.474351903	NA	NA		1
6666	0.519015048	NA	NA		1
1366	0.477966102	NA	NA		1
3357	0.522377372		839	925	1
6755	0.493053559	NA	NA		1
6763	0.502867795	NA	NA		0
20547	0.51664035		692	1000	1
1651	0.525169046	NA	NA		0
2362	0.493911888		1000	1000	0
1747	0.50668037	NA	NA		0
2124	0.517383349	NA	NA		0
906	0.487661575	NA	NA		1
1518	0.534836066	NA	NA		1
2029	0.547346819	NA	NA		0
2389	0.564814815	NA	NA		1
403	0.508684864	NA	NA		1
604	0.532	NA	NA		1
3125	0.471751412		919	939	1
881	0.490351873	NA	NA		1
1278	0.48119891	NA	NA		0
1372	0.505084746	NA	NA		1
997	0.497858672	NA	NA		1

1163	0.51129178	NA	NA		1
3087	0.525983146	NA	NA		1
1134	0.537037037	NA	NA		1
1194	0.518987342	NA	NA		1
1237	0.515928516	NA	NA		0
1393	0.567587752	NA	NA		1
2560	0.568149038	NA	NA		0
7349	0.536602452	NA	NA		1
4390	0.554505006	NA	NA		0
721	0.495412844	NA	NA		1
3262	0.558592523	NA	NA		0
1057	0.490405117	NA	NA		1
1428	0.573498965	NA	NA		1
897	0.577430972	NA	NA		1
7155	0.552225413	NA	NA		0
23270	0.507829592	NA	NA		0
1329	0.561825726	NA	NA		1
1348	0.468651331	NA	NA		1
2302	0.516301921	NA	NA		0
19282	0.511597659	NA	NA		1
646	0.613003096	NA	NA		1
621	0.544283414	NA	NA		1
581	0.56454389	NA	NA		1
666	0.528830313	NA	NA		1
44679	0.520328236	NA	NA		1
577	0.488734835	NA	NA		1
1078	0.529684601	NA	NA		1
16971	0.585187124	NA	NA		1
1243	0.511073254	NA	NA		1
2133	0.528673835	NA	NA		1
980	0.55	NA	NA		1
1234	0.512385919	NA	NA		1
1324	0.577620173	NA	NA		1
2640	0.515254237	NA	NA		1
1922	0.521282477	NA	NA		1
1920	0.463978495	NA	NA		1
1339	0.511817441	NA	NA		1
1441	0.500429923	NA	NA		0
1656	0.519458545	NA	NA		1
857	0.53640416	NA	NA		1
1667	0.488501742	NA	NA		1
1009	0.471458774	NA	NA		1
1927	0.521054494	NA	NA		1
2287	0.509982886	NA	NA		1
1080	0.460470085	NA	NA		1
6475	0.522012579	NA	NA		1
4219	0.437854339	NA	NA		0
2300	0.476749648		829	1000	1
2511	0.510161663	NA	NA		0
4643	0.490923869		596	782	1
2243	0.533472803	NA	NA		1
4425	0.503280724		307	1000	0
6492	0.519934641	NA	NA		1
3464	0.540756157	NA	NA		1
772	0.487288136	NA	NA		1
8588	0.522410682		200	934	0
1486	0.522207268	NA	NA		1

6113	0.54054806	NA	NA		0
2770	0.536271451	1000	1000		1
7195	0.525847808	447	1000		0
1788	0.484094853	NA	NA		1
910	0.50994152	NA	NA		1
10594	0.519791638	798	1000		0
1041	0.552915767	NA	NA		1
1390	0.579136691	NA	NA		1
34140	0.5784	NA	NA		1
4185	0.466751646	937	1000		0
1656	0.507850242	NA	NA		1
1793	0.50424282	NA	NA		0
22863	0.518372703	NA	NA		0
602	0.49833887	NA	NA		1
18809	0.430503684	1000	721		0
3450	0.532993306	879	865		1
2524	0.488917306	NA	NA		1
3121	0.464805119	NA	NA		1
734	0.545941124	NA	NA		0
2402	0.537517053	925	1000		1
4340	0.49310138	NA	NA		0
1069	0.541247485	NA	NA		1
1555	0.564630225	NA	NA		1
1042	0.537981651	NA	NA		0
2141	0.512364181	NA	NA		0
16888	0.501310909	NA	NA		0
1944	0.55815554	NA	NA		1
3587	0.565333333	NA	NA		1
6952	0.548377294	835	1000		1
1971	0.50997151	NA	NA		1
765	0.546405229	NA	NA		1
3491	0.486846084	NA	NA		1
2395	0.521040189	1000	881		1
1228	0.587068966	NA	NA		1
2767	0.552368758	NA	NA		1
765	0.508353222	NA	NA		1
1785	0.47637457	NA	NA		0
1494	0.539236861	NA	NA		1
5835	0.455255141	812	1000		1
5245	0.47299435	512	1000		0
1941	0.517084282	NA	NA		1
4535	0.500923645	1000	941		1
7618	0.507265918	300	947		1
5209	0.444058501	447	945		1
1322	0.416462518	NA	NA		1
2604	0.479102026	881	841		1
2911	0.493723849	848	883		1
21634	0.521552019	NA	NA		0
1123	0.523597507	NA	NA		1
2324	0.468934495	809	934		1
11948	0.50455881	625	1000		1
1973	0.525014831	NA	NA		1
1394	0.428571429	NA	NA		1
1052	0.490026954	NA	NA		1
4205	0.47813028	NA	NA		0
597	0.463654224	NA	NA		1
5138	0.501082251	NA	NA		0

748	0.441199684	NA	NA	0
1151	0.464712269	NA	NA	1
2485	0.470422535	NA	NA	1
9600	0.473164923	100	560	0
1243	0.505229284	NA	NA	1
3533	0.543723339	NA	NA	0
4204	0.520334331	NA	NA	0
774	0.46124031	NA	NA	1
761	0.50065703	NA	NA	1
1297	0.556544969	NA	NA	1
795	0.544807966	NA	NA	1
32996	0.521742606	208	1000	0
1474	0.521800281	NA	NA	1
6449	0.516471838	310	1000	1
4908	0.493382017	NA	NA	1
2795	0.551889622	NA	NA	0
1342	0.462873674	NA	NA	1
1120	0.540178571	NA	NA	1
2444	0.444169817	931	1000	1
2007	0.545578231	NA	NA	1
1433	0.476635514	NA	NA	1
2257	0.477899116	NA	NA	1
1032	0.439716312	NA	NA	0
625	0.4192	NA	NA	1
848	0.525943396	NA	NA	1
1892	0.472994652	NA	NA	0
1382	0.504883546	NA	NA	1
5500	0.569728524	8	963	1
913	0.475355969	NA	NA	1
5263	0.512359837	NA	NA	1
1716	0.522398346	NA	NA	1
1602	0.565899069	NA	NA	0
883	0.504901961	NA	NA	1
2786	0.517668655	NA	NA	1
1477	0.576509512	NA	NA	1
6297	0.498821945	NA	NA	1
1181	0.529212532	NA	NA	1
1165	0.475536481	NA	NA	1
2635	0.537396122	NA	NA	0
1083	0.479224377	NA	NA	1
3975	0.542880391	NA	NA	1
3975	0.537560214	NA	NA	0
2756	0.518474843	861	1000	1
1659	0.493084371	NA	NA	0
4653	0.507628539	796	825	0
1011	0.541984733	NA	NA	1
596	0.495378928	NA	NA	1
2002	0.431352459	NA	NA	1
1703	0.500303582	NA	NA	1
2273	0.509458865	1000	1000	1
6422	0.491670559	342	749	0
23622	0.542290749	433	1000	1
1777	0.503262643	NA	NA	1
14402	0.524745399	NA	NA	1
14402	0.542389514	NA	NA	0
747	0.548338369	NA	NA	1
6120	0.505542359	NA	NA	0

2587	0.495168148	NA	NA	1
1293	0.512809564	NA	NA	0
517	0.473887814	NA	NA	1
1534	0.511734029	NA	NA	1
1487	0.538823529	NA	NA	1
1108	0.517435321	NA	NA	1
10814	0.552174935	593	825	0
1241	0.485865724	NA	NA	1
1184	0.524498886	NA	NA	1
1796	0.510440835	NA	NA	1
1052	0.564102564	NA	NA	1
40833	0.547307692	NA	NA	0
2538	0.508274232	NA	NA	1
1258	0.542925278	NA	NA	1
4763	0.562029619	NA	NA	1
6865	0.542605971	376	1000	0
5481	0.578347951	NA	NA	0
3050	0.536107483	NA	NA	1
7657	0.471554252	NA	NA	0
2531	0.499736148	NA	NA	0
4677	0.472318886	NA	NA	0
1881	0.504192286	NA	NA	1
2432	0.494623656	NA	NA	1
1546	0.573524394	NA	NA	0
6487	0.52720504	NA	NA	0
16790	0.458978222	298	942	0
1811	0.547985782	NA	NA	1
2699	0.498777506	939	893	1
1119	0.45844504	NA	NA	1
2624	0.490990991	NA	NA	1
10660	0.522696477	170	1000	1
1559	0.467242621	NA	NA	1
1338	0.467455621	NA	NA	1
1720	0.535737705	NA	NA	1
4666	0.487383573	451	892	0
697	0.523584906	NA	NA	1
1247	0.536170213	NA	NA	1
1489	0.557651992	NA	NA	1
18078	0.509416878	NA	NA	0
18078	0.509416878	NA	NA	0
2130	0.510328638	1000	1000	1
3947	0.522439282	1000	1000	1
678	0.506493506	NA	NA	1
835	0.467543139	NA	NA	0
2761	0.48338257	NA	NA	1
1073	0.480353635	NA	NA	1
29691	0.502324641	NA	NA	0
948	0.551412429	NA	NA	1
2235	0.524167562	NA	NA	1
4213	0.516801536	NA	NA	0
941	0.424528302	NA	NA	1
1170	0.421998562	NA	NA	1
10258	0.52681778	NA	NA	0
1241	0.543897216	NA	NA	1
1230	0.573446328	NA	NA	1
2156	0.529376136	NA	NA	0
2176	0.612732095	NA	NA	1

1397	0.50718065	NA	NA		1
1631	0.500919681	NA	NA		1
2255	0.465953018	NA	NA		1
5272	0.513858856		167	829	0
17366	0.524539736		180	794	0
15972	0.51075677	NA	NA		1
4121	0.460757156		729	950	0
15421	0.527943427		627	1000	0
1475	0.527457627	NA	NA		1
2954	0.552813067		825	1000	1
1089	0.548918641	NA	NA		1
1303	0.489639294	NA	NA		1
932	0.528969957	NA	NA		1
5215	0.552759382		469	1000	1
6354	0.488527447	NA	NA		1
544	0.420803783	NA	NA		1
630	0.499047619	NA	NA		1
1847	0.442880347	NA	NA		1
570	0.456140351	NA	NA		1
7929	0.480238224	NA	NA		1
1802	0.514568765	NA	NA		1
2050	0.510320285	NA	NA		0
1301	0.410714286	NA	NA		1
9573	0.528469842		451	1000	0
2716	0.522770398	NA	NA		0
1021	0.444150344	NA	NA		1
9691	0.414179104	NA	NA		1
5218	0.509708738	NA	NA		1
10650	0.499451153		472	772	1
7542	0.514527287		291	1000	0
336	0.49702381	NA	NA		1
1205	0.480507706	NA	NA		1
2933	0.465385506	NA	NA		0
6388	0.529820926	NA	NA		1
2336	0.524521739	NA	NA		0
3040	0.575737265	NA	NA		1
1860	0.423594132	NA	NA		1
931	0.441077441	NA	NA		0
1816	0.529339853	NA	NA		1
3316	0.463235294	NA	NA		1
3052	0.491289199	NA	NA		1
1301	0.452745275	NA	NA		1
26570	0.540765391	NA	NA		1
1367	0.489592007	NA	NA		1
1509	0.36929761	NA	NA		1
2625	0.436583811	NA	NA		1
2377	0.459435626		947	944	1
2690	0.497264022		669	937	1
1605	0.489115646	NA	NA		1
599	0.475138122	NA	NA		1
1713	0.487916395	NA	NA		1
2080	0.503042596	NA	NA		0
1345	0.48600311	NA	NA		1
1461	0.493230174	NA	NA		1
611	0.454991817	NA	NA		1
1231	0.498281787	NA	NA		1
3155	0.478661806		755	1000	0

1620	0.530463038	NA	NA		1
3036	0.513437719	NA	NA		0
3642	0.478906999	NA	NA		0
2896	0.485049834	936		993	1
5126	0.456776755	NA	NA		1
1138	0.485074627	NA	NA		1
8914	0.471017667	NA	NA		0
3550	0.510483136	1000		1000	1
8135	0.475836431	NA	NA		0
11413	0.516616766	257		1000	1
3412	0.492313324	NA	NA		1
8873	0.4815822	NA	NA		0
3514	0.506989044	NA	NA		1
1341	0.57495069	NA	NA		1
661	0.53442029	NA	NA		1
952	0.560674157	NA	NA		1
1028	0.581322141	NA	NA		1
1280	0.493975904	NA	NA		1
3162	0.516913721	NA	NA		1
765	0.380645161	NA	NA		0
411	0.467153285	NA	NA		1
3011	0.492335437	874		749	1
1182	0.453362256	NA	NA		0
936	0.474056604	NA	NA		1
1126	0.496447602	NA	NA		1
41505	0.488050711	367		1000	0
688	0.521285838	NA	NA		1
965	0.41865285	NA	NA		1
12739	0.431272933	633		1000	1
18740	0.517689207	446		889	0
1669	0.488731487	NA	NA		1
933	0.428174879	NA	NA		1
13319	0.546092881	313		762	0
4960	0.473396501	652		910	1
1490	0.528098537	NA	NA		1
872	0.569306931	NA	NA		1
719	0.534351145	NA	NA		1
2028	0.51928934	NA	NA		1
1471	0.63940256	NA	NA		1
1563	0.486884197	NA	NA		1
6099	0.486032591	354		928	0
11407	0.457773512	833		1000	0
1188	0.473905724	NA	NA		1
1142	0.471103327	NA	NA		1
619	0.479806139	NA	NA		1
1326	0.422376409	NA	NA		1
103466	0.53496983	NA	NA		1
4500	0.538214368	934		1000	1
2318	0.441325357	1000		885	0
34155	0.520541612	580		683	1
690	0.422047244	NA	NA		1
2363	0.52476489	NA	NA		1
1215	0.516513761	NA	NA		1
801	0.533083645	NA	NA		1
744	0.510752688	NA	NA		1
2296	0.511160714	NA	NA		1
545	0.487447699	NA	NA		1

1450	0.535862069	NA	NA	1
2149	0.463588767	NA	NA	1
2609	0.542836041	NA	NA	1
3342	0.426610348	NA	NA	0
668	0.476979742	NA	NA	1
3814	0.462312925	861	1000	1
1436	0.414037855	NA	NA	1
1792	0.4739819	NA	NA	1
12747	0.541374527	374	1000	0
6725	0.477365632	NA	NA	1
2532	0.535734706	NA	NA	1
2880	0.477161193	NA	NA	1
2403	0.417771232	NA	NA	1
1157	0.471655329	NA	NA	1
3480	0.517094017	NA	NA	1
1238	0.494652406	NA	NA	1
1778	0.545295149	NA	NA	1
4643	0.529588608	NA	NA	0
3161	0.567010309	NA	NA	1
2171	0.557234727	NA	NA	0
1256	0.543087971	NA	NA	1
949	0.47029703	NA	NA	1
2017	0.422513089	NA	NA	1
1926	0.441386901	NA	NA	1
1758	0.480710349	NA	NA	1
2493	0.474361725	948	1000	1
10146	0.456637168	NA	NA	1
3394	0.51981982	615	654	1
1576	0.416832339	NA	NA	1
932	0.580756014	NA	NA	1
895	0.543817527	NA	NA	1
843	0.572953737	NA	NA	1
1396	0.469197708	NA	NA	1
722	0.411965812	NA	NA	1
1126	0.474226804	NA	NA	1
1533	0.485322896	NA	NA	1
3105	0.518968386	NA	NA	0
2294	0.498211091	1000	942	1
557	0.449392713	NA	NA	1
1338	0.463114754	NA	NA	1
21645	0.526900382	NA	NA	1
1828	0.45418552	NA	NA	1
2535	0.468045977	883	846	1
4143	0.496212121	NA	NA	1
8856	0.555989805	517	548	0
1419	0.474806202	NA	NA	1
4135	0.449498843	NA	NA	1
5715	0.509172442	146	762	0
6414	0.485762144	520	1000	1
1290	0.490275527	NA	NA	1
1169	0.511827957	NA	NA	1
889	0.509068924	NA	NA	1
994	0.440604752	NA	NA	1
17557	0.494318182	NA	NA	1
1919	0.528697572	NA	NA	1
551	0.558762887	NA	NA	1
1984	0.578031539	NA	NA	1

348	0.488505747	NA	NA		1
478	0.482673267	NA	NA		1
1154	0.471317118	NA	NA		1
726	0.516666667	NA	NA		1
995	0.535752401	NA	NA		1
13200	0.508425959	NA	NA		1
16889	0.533541952	433		1000	1
1706	0.481519862	NA	NA		1
1426	0.536465638	NA	NA		1
3119	0.506782609	NA	NA		1
2989	0.484257292	852		1000	1
3804	0.541468389	469		918	1
4458	0.451118119	368		719	0
1752	0.519549763	NA	NA		1
659	0.491654021	NA	NA		1
2325	0.511923688	NA	NA		1
3300	0.409623939	NA	NA		0
956	0.550209205	NA	NA		1
678	0.526548673	NA	NA		1
6327	0.535860128	800		1000	1
3955	0.495400788	100		1000	1
6889	0.484831029	523		780	0
3795	0.484624146	938		946	1
2391	0.452112087	1000		1000	1
46476	0.524366216	340		741	0
849	0.479140329	NA	NA		1
696	0.482758621	NA	NA		1
2880	0.515560555	NA	NA		1
2112	0.555862069	NA	NA		1
4638	0.477717144	885		1000	1
4184	0.47418738	1000		1000	1
9436	0.428482587	718		625	1
1293	0.541376643	NA	NA		1
760	0.460526316	NA	NA		1
1130	0.423529412	NA	NA		1
1301	0.554189085	NA	NA		1
1455	0.555306428	NA	NA		1
884	0.433455433	NA	NA		1
720	0.456076135	NA	NA		1
1547	0.500715308	NA	NA		1
835	0.469461078	NA	NA		1
4178	0.480522946	934		873	1
5772	0.452526263	NA	NA		1
4138	0.442458899	NA	NA		1
2447	0.471094123	NA	NA		1
3290	0.506276151	NA	NA		0
3969	0.507400209	764		1000	1
5302	0.498279637	NA	NA		1
1595	0.522546419	NA	NA		1
1733	0.477784189	NA	NA		1
752	0.525473071	NA	NA		1
811	0.574239714	NA	NA		1
1696	0.420512821	NA	NA		1
1645	0.491792929	NA	NA		1
1836	0.451891447	NA	NA		1
5010	0.571556511	605		734	1
1521	0.551401869	NA	NA		1

1924	0.472527473	NA	NA		1
1224	0.453765491	NA	NA		1
2092	0.467225234	NA	NA		1
1910	0.543455497	NA	NA		1
5651	0.506004902	936		958	0
3942	0.550340829	938		1000	1
6296	0.518294222	186		1000	0
2966	0.470855069	NA	NA		0
9823	0.392067869	495		1000	0
5893	0.421742483	NA	NA		1
2297	0.516144814	902		845	1
1667	0.509609013	NA	NA		1
12418	0.47968885	258		1000	1
2240	0.520037718	1000		881	1
1852	0.471624266	NA	NA		1
14301	0.495826011	NA	NA		0
2573	0.526252505	NA	NA		1
1907	0.510791367	NA	NA		1
3867	0.525405787	NA	NA		0
4611	0.489130435	949		987	1
1179	0.488549618	NA	NA		1
397	0.530120482	NA	NA		1
623	0.477031802	NA	NA		1
1403	0.570216777	NA	NA		1
764	0.495726496	NA	NA		1
871	0.47875	NA	NA		1
8993	0.541072117	NA	NA		1
6755	0.485777514	NA	NA		0
1545	0.465139052	NA	NA		0
3223	0.510148675	827		891	1
1418	0.497829233	NA	NA		0
2936	0.491781723	NA	NA		0
1228	0.493783304	NA	NA		1
6555	0.518824136	NA	NA		1
3081	0.444588185	398		999	1
1340	0.467653936	NA	NA		1
1355	0.50265542	NA	NA		0
1473	0.466326531	NA	NA		1
5789	0.461538462	881		648	1
4005	0.483260553	422		818	0
9527	0.489330895	57		697	0
1879	0.453067258	NA	NA		1
8065	0.474855363	NA	NA		0
2917	0.502234518	NA	NA		0
864	0.498842593	NA	NA		1
12424	0.511164274	744		1000	1
1378	0.528712871	NA	NA		1
4653	0.483193723	910		1000	1
2577	0.467818832	NA	NA		1
3209	0.531149647	905		1000	1
1320	0.469588551	NA	NA		1
3017	0.464720195	NA	NA		0
1406	0.482553823	NA	NA		1
2421	0.457242117	1000		1000	1
2763	0.448275862	NA	NA		1
1760	0.451321728	NA	NA		1
1354	0.499261448	NA	NA		1

836	0.523923445	NA	NA		1
1026	0.472709552	NA	NA		1
11216	0.532784821	54		1000	0
1878	0.423641703	NA	NA		1
3120	0.497999111	NA	NA		0
910	0.505494505	NA	NA		1
868	0.584101382	NA	NA		1
898	0.580178174	NA	NA		1
1832	0.466761973	NA	NA		1
1080	0.472222222	NA	NA		1
13115	0.427378145	NA	NA		0
1359	0.478625954	NA	NA		1
1137	0.491146319	NA	NA		1
3280	0.527769159	1000		1000	1
2747	0.482641253	783		987	1
2570	0.509814613	NA	NA		1
22714	0.499189465	642		1000	0
9112	0.566616118	260		473	1
743	0.501492537	NA	NA		1
2302	0.507260891	1000		726	1
4143	0.489816337	840		938	1
1561	0.430562726	NA	NA		0
1561	0.45389049	NA	NA		1
3738	0.434022721	562		816	1
1094	0.430449069	NA	NA		1
900	0.488676996	NA	NA		1
1781	0.496770904	NA	NA		0
2654	0.451704545	541		1000	1
1215	0.469890511	NA	NA		1
1035	0.460512821	NA	NA		1
918	0.443925234	NA	NA		1
805	0.427525622	NA	NA		1
1479	0.447073474	NA	NA		1
366	0.537953795	NA	NA		1
2518	0.43989071	NA	NA		0
910	0.587912088	NA	NA		1
2721	0.510841602	1000		1000	1
704	0.539305302	NA	NA		1
774	0.546641791	NA	NA		1
521	0.50547046	NA	NA		1
4349	0.452543886	1000		1000	1
5623	0.472043543	NA	NA		0
1783	0.438011328	NA	NA		1
645	0.468217054	NA	NA		1
2817	0.48034188	904		945	0
670	0.423572744	NA	NA		1
1232	0.447519084	NA	NA		1
2456	0.495049505	NA	NA		1
6607	0.475365239	296		1000	0
1183	0.558909445	NA	NA		1
1236	0.514843087	NA	NA		1
345	0.533333333	NA	NA		1
1208	0.437092265	NA	NA		1
3022	0.489575138	NA	NA		1
2614	0.474989312	940		934	1
3379	0.447074122	NA	NA		1
20581	0.457692308	605		1000	0

970	0.435820896	NA	NA	1
853	0.48125	NA	NA	1
7881	0.484524264	NA	NA	1
2919	0.470921986	839	508	1
1651	0.455022981	NA	NA	1
1058	0.436672968	NA	NA	1
984	0.460365854	NA	NA	1
705	0.513475177	NA	NA	1
1395	0.493189964	NA	NA	1
1136	0.458626761	NA	NA	1
5200	0.518386108	NA	NA	1
2024	0.470095694	NA	NA	1
1703	0.428571429	NA	NA	1
1645	0.430971512	NA	NA	0
312	0.576923077	NA	NA	1
8173	0.49571871	NA	NA	0
4005	0.492023582	833	942	0
6172	0.479571333	NA	NA	1
1769	0.409412597	NA	NA	0
1111	0.401280683	NA	NA	1
3108	0.499856198	NA	NA	0
2138	0.524668435	NA	NA	1
866	0.490514905	NA	NA	1
32594	0.422485028	NA	NA	0
442	0.468325792	NA	NA	1
3413	0.508688784	NA	NA	1
3072	0.530428999	1000	1000	1
1232	0.453488372	NA	NA	1
5293	0.496358938	NA	NA	0
1948	0.484093212	NA	NA	0
1360	0.516403402	NA	NA	0
1815	0.468235294	NA	NA	1
2068	0.42027833	NA	NA	0
3414	0.447616329	NA	NA	0
1563	0.522954092	NA	NA	1
1017	0.425155925	NA	NA	1
2695	0.443251534	NA	NA	1
1058	0.453116531	NA	NA	0
906	0.493231313	NA	NA	1
4813	0.521170314	863	977	1
2494	0.525661588	1000	1000	1
5439	0.523867809	367	817	1
1265	0.466046512	NA	NA	1
1327	0.493091537	NA	NA	1
4516	0.499854904	346	599	0
1831	0.520903955	NA	NA	1
638	0.401253918	NA	NA	1
2933	0.455506929	NA	NA	1
593	0.420042644	NA	NA	1
4520	0.487381703	91	1000	1
2670	0.516738661	NA	NA	1
6686	0.445369917	236	1000	1
1903	0.443506494	NA	NA	1
1656	0.463446475	NA	NA	1
1240	0.490322581	NA	NA	1
1386	0.497835498	NA	NA	1
5066	0.532690247	NA	NA	1

561	0.494279176	NA	NA	1
12425	0.500618429	359	1000	1
2556	0.509027373	NA	NA	1
9948	0.530750799	154	825	1
3423	0.526682135	NA	NA	1
2908	0.436096479	NA	NA	1
1135	0.434995112	NA	NA	1
924	0.487012987	NA	NA	1
1885	0.501173709	NA	NA	1
35010	0.513377699	NA	NA	1
2274	0.485488127	1000	1000	1
3053	0.483610822	943	946	0
976	0.539959016	NA	NA	1
5864	0.479472825	717	868	0
3011	0.474173946	NA	NA	1
1115	0.323279925	NA	NA	1
2101	0.513744655	NA	NA	1
1200	0.448028674	NA	NA	1
2284	0.505691769	1000	1000	1
1058	0.45212766	NA	NA	1
1166	0.381315539	NA	NA	1
2283	0.405246495	NA	NA	1
2763	0.521187855	NA	NA	0
1675	0.519038076	NA	NA	1
4151	0.557687322	841	1000	1
903	0.429184549	NA	NA	1
1391	0.462257369	NA	NA	1
985	0.510845987	NA	NA	1
1135	0.519852262	NA	NA	1
1924	0.494499131	NA	NA	1
1690	0.536428111	NA	NA	1
1074	0.490689013	NA	NA	1
783	0.466966967	NA	NA	1
6391	0.508287293	NA	NA	1
1454	0.46883593	NA	NA	1
1054	0.530892449	NA	NA	1
1119	0.471513836	NA	NA	0
1224	0.532497149	NA	NA	1
6639	0.543419665	NA	NA	0
1177	0.515717927	NA	NA	1
1031	0.545751634	NA	NA	1
14770	0.521722075	NA	NA	1
7956	0.547470547	929	1000	1
2044	0.449414271	NA	NA	1
553	0.482820976	NA	NA	1
749	0.441922563	NA	NA	1
3212	0.488858748	NA	NA	0
1976	0.524291498	NA	NA	1
1259	0.524004924	NA	NA	0
652	0.490797546	NA	NA	1
2107	0.424800797	901	1000	1
4638	0.463811563	351	1000	1
3400	0.481657012	852	825	1
770	0.459497207	NA	NA	1
1141	0.47265987	NA	NA	1
1549	0.47254509	NA	NA	1
540	0.47037037	NA	NA	1

719	0.446453408	NA	NA		1
1951	0.533617021	NA	NA		1
2107	0.463917526	NA	NA		1
583	0.428816467	NA	NA		1
5365	0.44606221	NA	NA		0
584	0.462328767	NA	NA		1
929	0.473627557	NA	NA		1
1581	0.484503479	NA	NA		1
6803	0.504100418		596	832	1
9928	0.495588235		505	783	1
2873	0.541635125		874	895	1
76483	0.521271008		306	494	1
2706	0.368970013		595	1000	1
1639	0.514756349	NA	NA		1
5529	0.458876404		327	1000	1
1684	0.500440529	NA	NA		1
660	0.404545455	NA	NA		1
543	0.445672192	NA	NA		1
560	0.410714286	NA	NA		1
487	0.416837782	NA	NA		1
795	0.437735849	NA	NA		1
678	0.513274336	NA	NA		1
1245	0.469717362	NA	NA		1
3897	0.459004905		352	1000	1
11291	0.494903902	NA	NA		1
5441	0.527734497	NA	NA		1
3439	0.44749276	NA	NA		1
1985	0.459687124	NA	NA		1
1871	0.46162928	NA	NA		1
6827	0.467932203		579	1000	0
7648	0.47987092		1000	1000	0
4314	0.486860304	NA	NA		1
620	0.456451613	NA	NA		1
2830	0.449116905	NA	NA		1
2850	0.472045294	NA	NA		1
520	0.476923077	NA	NA		1
1034	0.47582205	NA	NA		1
2389	0.45440787	NA	NA		0
2338	0.4523914	NA	NA		1
2143	0.440749347		913	1000	1
10266	0.539724503		106	936	1
2109	0.497560976		941	1000	1
2532	0.479579458		941	1000	1
1705	0.518941123	NA	NA		1
2390	0.540180814	NA	NA		0
20163	0.487277062	NA	NA		1
1682	0.433412604	NA	NA		1
2198	0.494104436	NA	NA		1
1751	0.429078014	NA	NA		1
2724	0.471766849	NA	NA		1
7687	0.461061453		950	1000	0
1592	0.397507788	NA	NA		0
1726	0.420186113	NA	NA		0
47713	0.475538329	NA	NA		0
24297	0.436794228		165	1000	1
1078	0.500266099	NA	NA		1
1884	0.477963998	NA	NA		1

1767	0.508544087	NA	NA		1
1047	0.472727273	NA	NA		1
1412	0.528134255	NA	NA		1
1481	0.462862764	NA	NA		1
1593	0.465491924	NA	NA		1
1741	0.47965812	NA	NA		0
1272	0.486510009	NA	NA		1
1646	0.552666667	NA	NA		1
2761	0.40872136		945	1000	1
5401	0.475344607	NA	NA		0
1726	0.499673842	NA	NA		1
2255	0.542239686		781	1000	1
3203	0.471034483	NA	NA		1
2180	0.506304559	NA	NA		1
4031	0.475392392		885	878	0
4664	0.505387755	NA	NA		1
1396	0.407593123	NA	NA		1
25202	0.493420382	NA	NA		0
4253	0.534666667	NA	NA		1
2035	0.45826602	NA	NA		1
952	0.48136646	NA	NA		1
708	0.43404908	NA	NA		1
1352	0.522189349	NA	NA		1
284	0.576190476	NA	NA		1
700	0.444444444	NA	NA		1
1740	0.451149425	NA	NA		1
520	0.54	NA	NA		1
617	0.511029412	NA	NA		1
717	0.58685446	NA	NA		1
844	0.538363171	NA	NA		1
1095	0.47495183	NA	NA		1
558	0.484848485	NA	NA		1
687	0.591639871	NA	NA		1
642	0.57165109	NA	NA		1
606	0.544117647	NA	NA		1
635	0.549912434	NA	NA		1
865	0.514357054	NA	NA		1
568	0.545275591	NA	NA		1
595	0.534579439	NA	NA		1
637	0.56993007	NA	NA		1
1675	0.532723773	NA	NA		0
706	0.47592068	NA	NA		1
696	0.503289474	NA	NA		1
6250	0.525119617		150	1000	1
1326	0.476859504	NA	NA		1
4681	0.424886878	NA	NA		0
1114	0.489106754	NA	NA		1
4186	0.49182686		520	744	0
1886	0.487058824	NA	NA		1
1060	0.542372881	NA	NA		1
1131	0.585805085	NA	NA		1
3429	0.472623506	NA	NA		0
877	0.538198404	NA	NA		1
992	0.525026624	NA	NA		1
2248	0.527992278		906	944	1
2301	0.505715592		1000	886	1
1456	0.488155061	NA	NA		1

660	0.432387312	NA	NA	1
3214	0.441801459	NA	NA	1
2319	0.48297315	NA	NA	1
2234	0.503782938	NA	NA	0
19914	0.531092915	NA	NA	0
19417	0.460281138	NA	NA	0
2287	0.470319635	NA	NA	1
424	0.480337079	NA	NA	1
6965	0.492849285	NA	NA	1
1217	0.422158548	NA	NA	1
1826	0.546002191	NA	NA	1
743	0.456258412	NA	NA	1
3097	0.539620536	NA	NA	1
7153	0.521639378	340	935	0
623	0.494382022	NA	NA	1
3624	0.564182195	NA	NA	1
1456	0.498717949	NA	NA	1
17857	0.508746876	1000	939	0
2269	0.510957324	NA	NA	1
1192	0.571308725	NA	NA	1
1664	0.510996119	NA	NA	1
638	0.458610847	NA	NA	0
3513	0.535404992	844	1000	0
1311	0.450790861	NA	NA	1
1272	0.499213836	NA	NA	1
571	0.45709282	NA	NA	1
843	0.550415184	NA	NA	1
901	0.443951165	NA	NA	1
11778	0.502628918	518	940	1
2660	0.535554653	801	1000	1
5265	0.603283174	212	1000	1
1960	0.47755102	NA	NA	1
824	0.50656168	NA	NA	1
4092	0.493333333	NA	NA	1
1364	0.463572854	NA	NA	0
1132	0.482332155	NA	NA	1
1046	0.467653509	NA	NA	0
6102	0.416323948	NA	NA	0
24571	0.509920367	NA	NA	1
2057	0.535731648	1000	1000	1
1586	0.578184111	NA	NA	1
4544	0.442727496	NA	NA	1
1424	0.493633692	NA	NA	1
4427	0.522995283	NA	NA	1
6767	0.539461748	288	1000	1
5527	0.435540752	NA	NA	1
2001	0.506746627	NA	NA	1
1688	0.452606635	NA	NA	1
2224	0.475829384	947	939	1
2260	0.473015873	NA	NA	1
4141	0.505412557	119	931	0
4553	0.41353061	NA	NA	1
726	0.479514416	NA	NA	1
698	0.471346705	NA	NA	1
3700	0.482048574	NA	NA	1
4035	0.439835447	NA	NA	1
8924	0.464677737	581	984	1

1541	0.48956229	NA		NA	1
3075	0.460639395	NA		NA	1
2134	0.496321449	NA		NA	1
2144	0.391992091		879	1000	1
2321	0.501550731		1000	1000	1
2520	0.441297631	NA		NA	1
1201	0.413489736	NA		NA	1
1403	0.558802566	NA		NA	1
1991	0.427301587	NA		NA	1
1878	0.416020672	NA		NA	1
2051	0.381782946	NA		NA	1
751	0.521970706	NA		NA	1
1462	0.450171821	NA		NA	1
1346	0.462478185	NA		NA	1
1380	0.448301887	NA		NA	1
1727	0.466459627	NA		NA	1
1776	0.515833333	NA		NA	1
17629	0.464694937	NA		NA	0
1701	0.54468599	NA		NA	0
1328	0.443802925	NA		NA	1
1443	0.444765343	NA		NA	1
763	0.448586118	NA		NA	0
12233	0.472719477	NA		NA	0
896	0.421052632	NA		NA	1
1257	0.502968617	NA		NA	1
3601	0.500887574	NA		NA	1
2923	0.494377163	NA		NA	1
5891	0.463534019	NA		NA	1
1679	0.550943396	NA		NA	1
1079	0.479917611	NA		NA	1
1584	0.544084401	NA		NA	1
674	0.553412463	NA		NA	1
2734	0.480570975	NA		NA	1
2309	0.493410648	NA		NA	1
1337	0.459237098	NA		NA	1
1311	0.490673155	NA		NA	1
1066	0.528769841	NA		NA	1
1212	0.438415159	NA		NA	1
3212	0.652982456	NA		NA	0
3070	0.521036814	NA		NA	0
2437	0.520741825		612	1000	1
871	0.481056257	NA		NA	1
4821	0.48797018	NA		NA	1
4129	0.419274376		1000	616	0
3104	0.437520938	NA		NA	1
692	0.573954984	NA		NA	1
513	0.513100437	NA		NA	1
2673	0.482977927		1000	1000	1
4440	0.518475073	NA		NA	0
2411	0.501070664	NA		NA	1
2861	0.528450363	NA		NA	1
6008	0.534914361	NA		NA	1
5212	0.54580244		493	606	1
5748	0.499273256		1000	1000	1
2857	0.504617968	NA		NA	1
1444	0.494219653	NA		NA	1
2485	0.439348621		935	1000	0

2159	0.50680071	NA	NA		1
4346	0.480776959	NA	NA		1
1030	0.476699029	NA	NA		1
3808	0.500800854		938	1000	1
11377	0.4936784		361	1000	0
5047	0.5042		594	778	1
12862	0.572497824		385	728	1
2312	0.457073171	NA	NA		1
1147	0.453658537	NA	NA		1
1752	0.444590451	NA	NA		0
2921	0.541736695	NA	NA		1
1805	0.436825701	NA	NA		1
2202	0.527359618	NA	NA		0
1294	0.486245955	NA	NA		1
1000	0.446351931	NA	NA		1
2507	0.499338624		866	1000	1
3997	0.34347275	NA	NA		0
2973	0.445921173		582	1000	0
1221	0.461021505	NA	NA		1
3149	0.440265487	NA	NA		1
1220	0.469331496	NA	NA		1
1464	0.586972083	NA	NA		1
3051	0.544194107		899	947	1
583	0.49796748	NA	NA		1
1290	0.50487013	NA	NA		1
1237	0.531123686	NA	NA		1
1281	0.47332959	NA	NA		1
1196	0.523854069	NA	NA		1
1617	0.532526475	NA	NA		1
1426	0.556457565	NA	NA		1
1623	0.507225434	NA	NA		1
1725	0.553645117	NA	NA		1
1898	0.501557632	NA	NA		0
1151	0.546788991	NA	NA		1
1566	0.477423639	NA	NA		1
8731	0.542917623	NA	NA		0
4042	0.439409369	NA	NA		0
1138	0.475315729	NA	NA		1
10102	0.476872247	NA	NA		0
2715	0.505735141	NA	NA		1
1686	0.447916667	NA	NA		1
2743	0.495724212	NA	NA		1
1309	0.502371712	NA	NA		1
4882	0.471903136		784	869	0
1434	0.462835249	NA	NA		1
10706	0.512871569		63	1000	1
1690	0.531230284	NA	NA		1
1468	0.499816514	NA	NA		0
2523	0.419412063		666	1000	1
1703	0.498398006	NA	NA		0
8196	0.478841871	NA	NA		0
2330	0.525	NA	NA		1
9659	0.492802209	NA	NA		1
1576	0.520876112	NA	NA		1
2645	0.544274809	NA	NA		1
995	0.421221865	NA	NA		1
2538	0.477120536	NA	NA		0

1344	0.467261905	NA	NA	1
3755	0.498791687	NA	NA	1
1314	0.476856835	NA	NA	1
6361	0.442663996	NA	NA	1
1141	0.449119374	NA	NA	1
1365	0.507614213	NA	NA	0
1500	0.465972222	NA	NA	1
1584	0.489225858	NA	NA	1
1135	0.545374449	NA	NA	1
1435	0.569286143	NA	NA	0
6476	0.46415156	948	879	0
2377	0.526816609	935	1000	1
3662	0.493317493	1000	889	1
2723	0.503972098	902	1000	0
854	0.55443038	NA	NA	1
2340	0.468006795	NA	NA	1
996	0.479048698	NA	NA	1
3237	0.399135002	1000	1000	1
989	0.480832421	NA	NA	1
1019	0.492594624	NA	NA	1
4108	0.454260696	895	851	0
1258	0.452669903	NA	NA	1
2247	0.534825871	942	1000	1
1081	0.497687327	NA	NA	1
650	0.492333901	NA	NA	1
1865	0.507350641	NA	NA	0
3104	0.524357098	NA	NA	0
1937	0.520207852	NA	NA	1
5823	0.518135786	NA	NA	0
830	0.43627451	NA	NA	1
1656	0.508282476	NA	NA	0
2189	0.531081743	1000	879	1
3973	0.491307371	NA	NA	0
941	0.436073059	NA	NA	1
1301	0.457307061	NA	NA	1
589	0.522157996	NA	NA	1
2437	0.489086635	NA	NA	0
1500	0.474186704	NA	NA	1
3015	0.498416051	NA	NA	1
4521	0.458418442	NA	NA	0
1060	0.538285714	NA	NA	1
1164	0.481958763	NA	NA	1
1375	0.530425963	NA	NA	1
838	0.485477178	NA	NA	1
4898	0.432221952	NA	NA	0
4986	0.519628933	1000	980	0
482	0.507389163	NA	NA	1
11079	0.496973101	750	762	1
2849	0.490139688	1000	585	1
2360	0.43551797	NA	NA	1
854	0.492974239	NA	NA	1
2236	0.462724936	NA	NA	0
5648	0.353433353	177	1000	1
2051	0.483045425	NA	NA	1
6132	0.537706433	935	1000	0
947	0.483679525	NA	NA	1
4345	0.470011316	384	1000	1

775	0.543225806	NA	NA		1
823	0.512758202	NA	NA		1
679	0.540500736	NA	NA		1
690	0.592753623	NA	NA		1
826	0.49031477	NA	NA		1
831	0.574647887	NA	NA		1
903	0.558139535	NA	NA		1
1017	0.560471976	NA	NA		1
25176	0.493523316	NA	NA		1
890	0.613483146	NA	NA		1
1507	0.378014184	NA	NA		1
1107	0.460287081	NA	NA		1
3117	0.557927421	NA	NA		0
3941	0.517530357		798	1000	1
4553	0.480061645		134	947	0
579	0.513026052	NA	NA		1
12608	0.549603175		244	1000	1
2096	0.523854962		1000	1000	1
1026	0.555555556	NA	NA		1
1455	0.461168385	NA	NA		1
701	0.489300999	NA	NA		1
106677	0.549897571		694	445	1
872	0.506880734	NA	NA		1
2952	0.514361702	NA	NA		1
998	0.570140281	NA	NA		1
20682	0.543137255	NA	NA		1
744	0.536082474	NA	NA		1
927	0.548366432	NA	NA		1
494	0.536437247	NA	NA		1
3265	0.510590532		873	935	0
862	0.530162413	NA	NA		1
1541	0.479308005	NA	NA		0
5340	0.5	NA	NA		1
5266	0.546157813	NA	NA		1
809	0.47342398	NA	NA		1
1103	0.520398912	NA	NA		1
704	0.492897727	NA	NA		1
762	0.472307692	NA	NA		1
13042	0.540556542	NA	NA		1
6590	0.443877551		773	1000	0
77542	0.359802074		350	479	0
7364	0.503893026	NA	NA		0
1554	0.494208494	NA	NA		1
1742	0.548957828	NA	NA		1
1906	0.459031657	NA	NA		1
1491	0.46797671	NA	NA		0
8573	0.473524545	NA	NA		0
2548	0.552935737	NA	NA		1
1299	0.461330935	NA	NA		1
2837	0.484896038		943	946	1
2155	0.474911102	NA	NA		0
4687	0.491997133	NA	NA		0
6974	0.47235723		406	1000	1
1737	0.46193984	NA	NA		1
1724	0.508737864	NA	NA		1
1528	0.482470785	NA	NA		1
4144	0.457148275		282	1000	0

6847	0.449838932	NA	NA		1
4014	0.549894958	903		941	1
2183	0.502712477	NA	NA		1
1108	0.469314079	NA	NA		1
3901	0.513792143	1000		875	1
3410	0.519061584	1000		1000	1
1860	0.452661064	NA	NA		1
3586	0.524665897	287		830	0
1319	0.513673319	NA	NA		1
2663	0.47606383	NA	NA		1
3158	0.494832677	674		629	0
1315	0.519391635	NA	NA		1
2835	0.530932595	713		843	1
3603	0.549304882	811		846	1
2690	0.458932959	676		783	0
1443	0.522277228	NA	NA		1
603	0.535655058	NA	NA		1
449	0.418708241	NA	NA		1
1747	0.47151277	NA	NA		1
2234	0.4615	NA	NA		1
24532	0.505543237	NA	NA		1
19220	0.509906996	NA	NA		0
1696	0.556992724	NA	NA		1
1881	0.532222572	NA	NA		1
1844	0.552335896	NA	NA		0
1367	0.521753461	NA	NA		1
2056	0.538119087	NA	NA		1
1258	0.503968254	NA	NA		1
4357	0.524722503	NA	NA		1
430	0.481395349	NA	NA		1
1042	0.52866242	NA	NA		1
1031	0.561946903	NA	NA		1
1646	0.508603239	NA	NA		1
3709	0.456589492	610		1000	1
3536	0.534589041	NA	NA		1
5227	0.535073409	NA	NA		0
43048	0.453605201	NA	NA		1
1774	0.579260652	NA	NA		0
1198	0.527050611	NA	NA		1
2697	0.470770422	NA	NA		1
5963	0.511692501	NA	NA		0
694	0.515103339	NA	NA		1
2565	0.525729191	NA	NA		0
1109	0.544634806	NA	NA		1
9075	0.520734525	931		931	1
831	0.451263538	NA	NA		1
1755	0.478508217	NA	NA		1
1977	0.460470085	NA	NA		0
1017	0.529990167	NA	NA		1
1418	0.569816643	NA	NA		1
2059	0.46373057	NA	NA		1
1192	0.491620112	NA	NA		1
10218	0.483603948	860		1000	1
5373	0.465250111	29		1000	1
1462	0.525307798	NA	NA		1
1162	0.46627566	NA	NA		1
1699	0.490658499	NA	NA		1

1482	0.472058824	NA	NA		1
2000	0.462929476	NA	NA		1
5704	0.563319716	NA	NA		0
2048	0.470096463	NA	NA		0
1498	0.50200267	NA	NA		1
1024	0.499448732	NA	NA		1
10931	0.47415583		937	1000	1
2634	0.506527415	NA	NA		1
975	0.518438178	NA	NA		1
7155	0.485101311	NA	NA		0
5383	0.52969184		478	1000	0
958	0.5	NA	NA		1
736	0.445454545	NA	NA		0
4335	0.545176661		398	942	0
1360	0.543055556	NA	NA		1
855	0.516959064	NA	NA		1
2828	0.50470009	NA	NA		1
1814	0.439706396	NA	NA		1
8013	0.519172896		206	1000	1
4121	0.557719054	NA	NA		0
2362	0.516813451	NA	NA		1
1434	0.510669975	NA	NA		1
2692	0.475482912		1000	1000	1
742	0.527859238	NA	NA		1
1833	0.530957684	NA	NA		1
1328	0.525356577	NA	NA		1
906	0.505665722	NA	NA		1
798	0.475167785	NA	NA		1
1057	0.525773196	NA	NA		1
921	0.457433291	NA	NA		1
1410	0.497163121	NA	NA		1
3000	0.483808707	NA	NA		0
5394	0.480500487		611	1000	0
16730	0.494116958		514	814	1
4891	0.535189651		1000	1000	1
1695	0.52301106	NA	NA		0
16696	0.523324707		926	1000	0
1488	0.455817378	NA	NA		1
971	0.407228916	NA	NA		1
1122	0.430678466	NA	NA		1
1016	0.456256921	NA	NA		1
1153	0.387067395	NA	NA		1
2196	0.433835058	NA	NA		1
2947	0.488839286	NA	NA		1
4213	0.480500951	NA	NA		0
2847	0.509300147	NA	NA		0
29590	0.528735362	NA	NA		0
1954	0.49869281	NA	NA		1
1759	0.429174201	NA	NA		1
5765	0.517073171	NA	NA		1
498	0.47972973	NA	NA		1
2553	0.478351374		849	1000	1
2393	0.452082288	NA	NA		1
2745	0.467012987	NA	NA		1
16725	0.501894837		379	1000	1
2247	0.546195652	NA	NA		1
2764	0.485758276		948	886	1

900	0.513838748	NA	NA		1
1585	0.440509915	NA	NA		1
1952	0.464550265	NA	NA		1
1484	0.446376812	NA	NA		1
2293	0.451011715	NA	NA		1
1320	0.466372657	NA	NA		1
1426	0.487377279	NA	NA		1
1507	0.424242424	NA	NA		1
3641	0.488776978	436		788	1
897	0.422387137	NA	NA		1
4487	0.510383536	NA	NA		0
28546	0.518792628	866		560	0
2253	0.492348285	NA	NA		1
3701	0.457164291	781		1000	0
1048	0.463576159	NA	NA		1
4472	0.494388239	NA	NA		1
2196	0.476632047	NA	NA		1
2787	0.462538226	NA	NA		1
14574	0.508007687	NA	NA		0
1735	0.478127734	NA	NA		0
809	0.487021014	NA	NA		1
10960	0.456625865	NA	NA		1
2575	0.496043315	945		943	1
1757	0.431417188	NA	NA		1
2048	0.486176837	1000		1000	1
3079	0.438071339	872		1000	1
1193	0.375193798	NA	NA		1
7041	0.522977941	120		1000	0
1329	0.522167488	NA	NA		1
1326	0.499587799	NA	NA		1
1090	0.439449541	NA	NA		1
1150	0.549038462	NA	NA		1
1898	0.553523238	NA	NA		1
702	0.510887772	NA	NA		1
1837	0.464888405	NA	NA		1
631	0.40625	NA	NA		1
3064	0.522668264	604		1000	1
1718	0.521011162	NA	NA		0
2590	0.422627396	NA	NA		1
1069	0.515434986	NA	NA		1
3515	0.5	767		829	0
410	0.46097561	NA	NA		1
832	0.511191336	NA	NA		1
1080	0.520428016	NA	NA		1
1345	0.477294686	NA	NA		1
1317	0.53115016	NA	NA		1
813	0.40098401	NA	NA		1
14282	0.52991453	NA	NA		0
3257	0.496470588	NA	NA		0
5095	0.447263223	NA	NA		1
948	0.518417462	NA	NA		1
1681	0.545482673	NA	NA		1
1540	0.492610837	NA	NA		1
1071	0.483333333	NA	NA		1
1517	0.511295181	NA	NA		1
1327	0.433994823	NA	NA		1
1905	0.521452145	NA	NA		1

11772	0.490196078		307	1000	0
8111	0.526143227	NA	NA		1
18996	0.496972556	NA	NA		0
1757	0.515364917	NA	NA		1
1441	0.575039494	NA	NA		1
2160	0.494988067	NA	NA		1
1258	0.413278008	NA	NA		1
4855	0.458549223	NA	NA		0
5980	0.415636164		737	1000	0
1325	0.47108307	NA	NA		1
2870	0.476219637	NA	NA		1
4345	0.49018892	NA	NA		0
2062	0.524468085	NA	NA		1
10973	0.500766284		663	1000	1
1932	0.473977695	NA	NA		1
1576	0.458571429	NA	NA		1
982	0.455075846	NA	NA		1
1579	0.537682077	NA	NA		1
1840	0.466156563	NA	NA		1
4628	0.55450237		502	971	0
1377	0.494453249	NA	NA		1
4072	0.517027864	NA	NA		0
10728	0.519409203	NA	NA		1
1125	0.473777778	NA	NA		1
1389	0.454204204	NA	NA		1
5062	0.509572072		746	936	1
1139	0.4381036	NA	NA		1
1680	0.460875808	NA	NA		1
1028	0.463656388	NA	NA		1
775	0.478813559	NA	NA		1
1432	0.43006263	NA	NA		1
592	0.35472973	NA	NA		1
33538	0.424468482	NA	NA		1
1011	0.531143828	NA	NA		1
3483	0.540511343	NA	NA		1
1965	0.447596989	NA	NA		1
1205	0.503909644	NA	NA		1
11207	0.501167056	NA	NA		0
13653	0.545706823	NA	NA		0
1705	0.563556209	NA	NA		1
1694	0.461726883	NA	NA		1
2966	0.520887246	NA	NA		1
1141	0.491245136	NA	NA		1
2295	0.465011962	NA	NA		0
3268	0.507634777		1000	1000	1
5337	0.517487047	NA	NA		1
2643	0.51036036	NA	NA		1
1520	0.490136054	NA	NA		1
1356	0.481853282	NA	NA		1
2035	0.477532571	NA	NA		1
1173	0.508098892	NA	NA		1
23362	0.543440517	NA	NA		0
2256	0.509330906		1000	1000	1
1754	0.476938672	NA	NA		1
2608	0.522527473	NA	NA		1
1383	0.421677215	NA	NA		1
1524	0.42124845	NA	NA		1

1467	0.490486258	NA	NA	1
2821	0.478415842	819	1000	1
1609	0.504888268	NA	NA	1
2486	0.498679577	NA	NA	1
2177	0.471914008	NA	NA	1
1011	0.518025513	NA	NA	0
2626	0.548419218	668	942	0
2823	0.528280543	941	948	1
1034	0.474729242	NA	NA	1
2994	0.544039838	NA	NA	1
2370	0.402622632	NA	NA	1
3920	0.546554622	681	985	1
3283	0.515444618	922	1000	1
2176	0.52253999	887	1000	1
1193	0.493939394	NA	NA	1
998	0.428724544	NA	NA	1
1059	0.484295846	NA	NA	1
968	0.428412875	NA	NA	1
964	0.467777778	NA	NA	1
1124	0.546263345	NA	NA	1
2870	0.488501484	938	942	1
2776	0.547581903	NA	NA	1
1508	0.496564647	NA	NA	0
928	0.474034621	NA	NA	1
1685	0.488741722	NA	NA	1
1316	0.492227979	NA	NA	1
2361	0.517439083	NA	NA	1
1356	0.491143317	NA	NA	1
728	0.476550681	NA	NA	1
1553	0.438088829	NA	NA	1
1002	0.435129741	NA	NA	1
3614	0.485499829	NA	NA	1
2185	0.452321518	945	873	1
18438	0.472735466	94	1000	1
1033	0.507142857	NA	NA	1
1277	0.427212179	NA	NA	1
3750	0.514392991	665	939	1
6392	0.452736318	NA	NA	1
1115	0.476523477	NA	NA	1
4328	0.477866373	567	1000	1
1916	0.465761875	NA	NA	1
2915	0.547699214	NA	NA	1
2330	0.507631055	NA	NA	1
487	0.478439425	NA	NA	1
5447	0.444965185	NA	NA	0
1251	0.445445445	NA	NA	1
1744	0.497378768	NA	NA	1
9783	0.394973921	1000	929	1
3467	0.418292374	759	633	0
2202	0.470064325	933	886	1
921	0.434180139	NA	NA	1
3330	0.412358519	NA	NA	1
1914	0.468717232	NA	NA	0
1900	0.45926373	NA	NA	1
2458	0.495922528	NA	NA	1
5303	0.455495262	533	972	0
1836	0.496843434	NA	NA	1

2577	0.479280033	812	1000	1
1116	0.5 NA	NA		1
2616	0.518571429	625	1000	0
2569	0.531668697 NA	NA		1
3763	0.530888031 NA	NA		1
10872	0.479041916 NA	NA		1
1514	0.395596591 NA	NA		1
2569	0.485013624	1000	1000	1
1182	0.419799499 NA	NA		1
8220	0.523212045	153	935	0
716	0.541729894 NA	NA		1
1030	0.472815534 NA	NA		1
2210	0.512367491 NA	NA		1
1288	0.557155716 NA	NA		1
2147	0.524086818 NA	NA		1
1372	0.490524781 NA	NA		1
963	0.513186813 NA	NA		1
1464	0.569296375 NA	NA		1
2412	0.555216063 NA	NA		1
1240	0.49516129 NA	NA		1
628	0.425051335 NA	NA		1
2757	0.504750594 NA	NA		0
938	0.464457253 NA	NA		0
951	0.522271715 NA	NA		1
938	0.52233677 NA	NA		1
1771	0.445101907 NA	NA		1
860	0.540027137 NA	NA		1
1096	0.530271399 NA	NA		1
3621	0.503171247	918	943	1
2450	0.534439417 NA	NA		1
2318	0.519864088 NA	NA		1
7878	0.513328868	946	943	1
702	0.465648855 NA	NA		1
936	0.505773672 NA	NA		1
1806	0.526255708 NA	NA		1
2216	0.529628654 NA	NA		0
1674	0.535027898 NA	NA		1
7704	0.562650966 NA	NA		0
725	0.430344828 NA	NA		1
2149	0.487103175	895	1000	1
2845	0.487603306 NA	NA		1
2651	0.487889273	950	1000	1
3727	0.484172662	261	1000	0
1637	0.473214286 NA	NA		1
4179	0.472410033 NA	NA		0
1575	0.510124611 NA	NA		1
1370	0.450490633 NA	NA		1
705	0.484423676 NA	NA		1
3194	0.511043153 NA	NA		1
10676	0.496660595	549	608	1
2166	0.458153993	925	1000	1
5547	0.534339846	555	875	0
2218	0.467511419 NA	NA		0
2108	0.526902887 NA	NA		0
46760	0.46577159	667	1000	1
2834	0.487765744 NA	NA		1
3228	0.461973279 NA	NA		1

8682	0.489795918	NA	NA		1
2225	0.520718232	881	988		1
1212	0.48574057	NA	NA		1
2035	0.479007634	NA	NA		1
1817	0.523430592	NA	NA		1
1321	0.519386835	NA	NA		1
1444	0.52401011	NA	NA		1
4281	0.479047855	NA	NA		1
2089	0.501845018	NA	NA		1
1451	0.522398346	NA	NA		1
2384	0.50545302	1000	1000		1
2926	0.46147581	805	655		1
1875	0.4875	NA	NA		1
21371	0.504354428	375	879		0
6615	0.509423704	NA	NA		1
2309	0.500448029	1000	1000		1
1533	0.486334968	NA	NA		1
772	0.626666667	NA	NA		1
1583	0.561781609	NA	NA		1
1662	0.544198895	NA	NA		1
1029	0.506564551	NA	NA		1
1058	0.488770053	NA	NA		1
1925	0.416613824	NA	NA		1
2406	0.494596841	1000	1000		1
411	0.491484185	NA	NA		1
1659	0.477076125	NA	NA		0
1265	0.49789916	NA	NA		1
4160	0.516383735	1000	931		0
2266	0.514038877	NA	NA		0
2803	0.475174554	926	937		1
1194	0.57118928	NA	NA		1
839	0.562419562	NA	NA		1
1488	0.556776557	NA	NA		1
1654	0.464084947	NA	NA		1
733	0.403819918	NA	NA		1
1911	0.525490196	NA	NA		1
1874	0.527902502	NA	NA		0
2005	0.471927162	NA	NA		0
43237	0.507891991	331	759		0
2021	0.485875706	NA	NA		1
1367	0.493589744	NA	NA		1
1328	0.477477477	NA	NA		1
1467	0.474864656	NA	NA		1
2411	0.539946381	NA	NA		1
6290	0.501581722	280	877		1
11129	0.556394722	313	1000		0
1475	0.521255061	NA	NA		1
2097	0.471229558	NA	NA		1
615	0.434146341	NA	NA		1
2398	0.472243155	NA	NA		0
725	0.510815308	NA	NA		1
1756	0.469822485	NA	NA		1
1723	0.464307139	NA	NA		1
32117	0.495628415	NA	NA		1
7102	0.412698413	466	1000		1
1164	0.463068182	NA	NA		1
1173	0.461210571	NA	NA		1

783	0.456859972	NA	NA	1
9697	0.530640669	465	806	0
2577	0.518479958	NA	NA	1
1136	0.45334507	NA	NA	1
1279	0.522913257	NA	NA	1
10316	0.558241647	NA	NA	1
1192	0.509433962	NA	NA	1
2905	0.469135802	NA	NA	1
3626	0.489602008	NA	NA	0
1772	0.499618612	NA	NA	1
2334	0.534050179	NA	NA	1
851	0.449056604	NA	NA	1
985	0.431472081	NA	NA	1
1004	0.441647597	NA	NA	1
809	0.480263158	NA	NA	1
3539	0.518294623	1000	942	1
3488	0.522175732	779	919	0
5144	0.517928287	851	1000	1
678	0.438906752	NA	NA	1
3067	0.525475543	1000	1000	1
6132	0.423066702	NA	NA	0
749	0.432576769	NA	NA	1
3333	0.508944544	NA	NA	1
1446	0.55277145	NA	NA	1
1859	0.502597403	NA	NA	1
13595	0.513841864	NA	NA	1
585	0.533333333	NA	NA	1
729	0.466392318	NA	NA	1
494	0.546558704	NA	NA	1
2920	0.498648368	731	939	1
3511	0.531377343	NA	NA	1
1585	0.516789517	NA	NA	1
2882	0.540353775	NA	NA	1
2373	0.508923431	NA	NA	1
723	0.545454545	NA	NA	1
1215	0.476724138	NA	NA	1
856	0.398255814	NA	NA	1
1854	0.476163125	NA	NA	1
1159	0.515894641	NA	NA	1
2255	0.485051861	NA	NA	1
31011	0.46935107	688	1000	1
2903	0.500923077	NA	NA	0
4343	0.482718894	NA	NA	1
788	0.453781513	NA	NA	1
3291	0.494169096	1000	911	0
3284	0.456254272	705	1000	1
1016	0.427083333	NA	NA	1
5777	0.554471545	110	804	1
1817	0.516387473	NA	NA	1
3492	0.513279618	919	1000	1
945	0.467724868	NA	NA	1
917	0.475581395	NA	NA	1
2424	0.51780502	NA	NA	1
1064	0.484022556	NA	NA	1
668	0.4	NA	NA	1
13205	0.498443114	378	1000	0
2031	0.51275095	NA	NA	1

6515	0.48786353	NA	NA		1
789	0.49833887	NA	NA		0
970	0.432084309	NA	NA		1
2016	0.408502773	NA	NA		1
15892	0.467570755	NA	NA		1
891	0.508270677	NA	NA		1
540	0.562962963	NA	NA		1
15888	0.52008149	NA	NA		1
1772	0.550638298	NA	NA		1
1737	0.499182205	NA	NA		1
1380	0.476671851	NA	NA		1
1421	0.485484591	NA	NA		1
2168	0.502790462	NA	NA		1
2553	0.50097371	NA	NA		1
1695	0.5179941	NA	NA		1
3186	0.456787868	NA	NA		0
3814	0.486658581	NA	NA		1
2310	0.571755289	NA	NA		1
1562	0.51954937	NA	NA		1
903	0.457888493	NA	NA		1
2666	0.540713999		889	938	1
905	0.520441989	NA	NA		1
639	0.568075117	NA	NA		1
1157	0.552533333	NA	NA		1
15165	0.503643253		745	937	1
1843	0.489316239	NA	NA		1
9797	0.459173917		192	750	0
5192	0.513543192	NA	NA		1
5153	0.48570116	NA	NA		0
2154	0.542461832	NA	NA		1
2382	0.521662246	NA	NA		1
872	0.452314165	NA	NA		1
4113	0.502751789	NA	NA		1
1835	0.482806434	NA	NA		1
861	0.49188092	NA	NA		0
1891	0.562462996	NA	NA		1
1855	0.489490354	NA	NA		0
1850	0.509222661	NA	NA		1
1818	0.527117611	NA	NA		0
675	0.467874794	NA	NA		1
2700	0.475925926	NA	NA		1
1128	0.467361741	NA	NA		1
2119	0.529732679	NA	NA		1
2279	0.403906742	NA	NA		1
2141	0.463970812	NA	NA		0
13081	0.518194323		67	1000	0
999	0.569807037	NA	NA		1
667	0.506352087	NA	NA		1
1288	0.416149068	NA	NA		1
1379	0.504724409	NA	NA		1
1065	0.467124071	NA	NA		0
1326	0.497737557	NA	NA		1
1934	0.482908639	NA	NA		1
1035	0.479227053	NA	NA		1
1094	0.467425026	NA	NA		1
1683	0.458230958	NA	NA		1
895	0.50671785	NA	NA		0

2688	0.48735119	NA	NA	1
10029	0.546997098	407	881	0
1310	0.496086106	NA	NA	1
1225	0.452830189	NA	NA	1
1023	0.509259259	NA	NA	1
1718	0.507264687	NA	NA	1
1774	0.490566038	NA	NA	1
8470	0.516834281	511	875	1
4095	0.479632063	NA	NA	1
1714	0.492093612	NA	NA	1
1840	0.45748503	NA	NA	1
1773	0.510895884	NA	NA	1
3512	0.499084249	886	878	1
3092	0.413158628	NA	NA	1
1173	0.424603175	NA	NA	1
1163	0.459069021	NA	NA	1
1760	0.515132409	NA	NA	1
1142	0.521341463	NA	NA	1
549	0.474332649	NA	NA	1
1002	0.504219409	NA	NA	1
1561	0.491727333	NA	NA	1
1254	0.575826682	NA	NA	1
2180	0.434640523	NA	NA	1
2765	0.502554988	617	1000	0
3702	0.503762376	203	1000	1
561	0.489749431	NA	NA	1
1710	0.472611465	NA	NA	1
10854	0.518872017	216	818	1
1644	0.4513382	NA	NA	1
680	0.428078251	NA	NA	0
2828	0.546363636	NA	NA	1
1929	0.504088785	NA	NA	1
1874	0.48197923	NA	NA	1
2327	0.455529549	NA	NA	1
494	0.443319838	NA	NA	1
4260	0.539292523	199	857	0
2309	0.504114335	NA	NA	1
1996	0.547749726	NA	NA	1
2330	0.502396932	1000	821	1
3809	0.377169559	NA	NA	1
1212	0.45	NA	NA	1
1209	0.456201214	NA	NA	1
2265	0.483806677	NA	NA	1
1065	0.473972603	NA	NA	1
6005	0.484714172	521	1000	0
4549	0.508385744	124	1000	0
3672	0.460304288	NA	NA	1
851	0.500587544	NA	NA	1
1005	0.508457711	NA	NA	1
861	0.533834586	NA	NA	1
1085	0.484792627	NA	NA	1
959	0.486666667	NA	NA	1
1044	0.484231943	NA	NA	1
1242	0.471332209	NA	NA	1
15624	0.48733536	606	1000	1
16500	0.504113258	NA	NA	1
7313	0.439931934	NA	NA	0

1596	0.465538847	NA	NA		1
1222	0.424713584	NA	NA		1
729	0.498511905	NA	NA		1
513	0.479532164	NA	NA		1
1761	0.457497612	NA	NA		1
887	0.476888388	NA	NA		1
2251	0.476901798	NA	NA		1
2322	0.502174859	NA	NA		0
676	0.472924188	NA	NA		1
2440	0.496365004	NA	NA		1
1172	0.501818182	NA	NA		1
1096	0.453580902	NA	NA		1
511	0.5	NA	NA		1
587	0.517241379	NA	NA		1
819	0.498652291	NA	NA		1
1886	0.505422993	NA	NA		1
21274	0.445066335	NA	NA		1
1751	0.47396293	NA	NA		1
3504	0.500367918		706	829	1
782	0.491428571	NA	NA		1
6739	0.539379475		753	1000	1
12353	0.480062029		485	1000	1
1252	0.433224756	NA	NA		0
1952	0.540331492	NA	NA		1
1818	0.500910747	NA	NA		1
1726	0.530259366	NA	NA		1
1007	0.48119859	NA	NA		0
2085	0.416188289	NA	NA		1
771	0.456549935	NA	NA		1
2625	0.534690102		611	1000	1
1804	0.519522278	NA	NA		0
1804	0.492110454	NA	NA		1
3767	0.516138949	NA	NA		1
2060	0.467130215	NA	NA		0
2357	0.504132231	NA	NA		1
858	0.524475524	NA	NA		1
2201	0.525399129	NA	NA		1
724	0.527607362	NA	NA		1
948	0.511864407	NA	NA		1
2160	0.476476476	NA	NA		1
1216	0.519301471	NA	NA		1
1146	0.498498498	NA	NA		1
2328	0.555223881	NA	NA		1
2765	0.490500864	NA	NA		1
426	0.495844875	NA	NA		1
1231	0.558082859	NA	NA		1
1350	0.54962963	NA	NA		1
556	0.509018036	NA	NA		1
2963	0.529837875	NA	NA		1
2158	0.545588235	NA	NA		1
11132	0.558717501		217	931	0
27692	0.53718115		460	1000	1
2284	0.516413523	NA	NA		1
1933	0.415425356	NA	NA		1
1258	0.525565801	NA	NA		1
3873	0.485622145	NA	NA		1
1062	0.423748545	NA	NA		1

993	0.548275862	NA	NA	1
1485	0.428127428	NA	NA	1
17632	0.469489415	NA	NA	0
1779	0.495169082	NA	NA	1
28527	0.506588716	NA	NA	0
1342	0.467457181	NA	NA	0
758	0.434343434	NA	NA	1
12310	0.413737155	NA	NA	1
2408	0.516620499	NA	NA	1
2903	0.454384585	NA	NA	1
2439	0.522112495	1000	946	1
414	0.542613636	NA	NA	1
334	0.517241379	NA	NA	1
1979	0.529932327	NA	NA	1
8431	0.494242285	898	941	1
35919	0.513908423	144	850	0
43074	0.541084214	1000	1000	1
1499	0.423242468	NA	NA	1
1222	0.457364341	NA	NA	1
602	0.471760797	NA	NA	1
3739	0.453349774	1000	1000	1
1869	0.506153023	NA	NA	1
11882	0.515961396	958	945	0
1584	0.471627907	NA	NA	1
537	0.453674121	NA	NA	1
2345	0.508074995	NA	NA	0
1422	0.477394636	NA	NA	1
762	0.476377953	NA	NA	1
1089	0.528263104	NA	NA	1
881	0.515892421	NA	NA	1
10915	0.532950192	338	1000	1
507	0.481152993	NA	NA	1
34704	0.5367428	310	939	0
2253	0.459325397	815	948	1
1974	0.495862884	NA	NA	1
5049	0.520179372	NA	NA	1
5782	0.509072581	NA	NA	0
987	0.517730496	NA	NA	1
2238	0.51062753	NA	NA	1
1278	0.518779343	NA	NA	1
1060	0.499056604	NA	NA	1
1340	0.513557929	NA	NA	1
15367	0.531541948	NA	NA	0
1094	0.474059003	NA	NA	1
2019	0.459558824	NA	NA	1
2000	0.476949345	NA	NA	1
2840	0.449574362	945	1000	0
2091	0.441873278	NA	NA	1
1995	0.478798587	NA	NA	1
3101	0.501040462	575	740	1
2069	0.471188475	NA	NA	1
2404	0.490566038	732	890	1
16695	0.49697383	385	861	0
9327	0.460439654	NA	NA	0
1879	0.572057206	NA	NA	1
1573	0.54247392	NA	NA	1
5498	0.559607694	NA	NA	0

5732	0.507768362		502	790	1
2380	0.454424779	NA	NA		1
1822	0.480938416	NA	NA		1
1951	0.507614213	NA	NA		1
7081	0.462502145		313	856	1
6852	0.509952056		677	938	0
984	0.492886179	NA	NA		1
1904	0.495518566	NA	NA		0
2413	0.462722853	NA	NA		1
8080	0.532374101		301	1000	0
1469	0.469503546	NA	NA		1
7891	0.523258077		407	873	1
8592	0.515291023		479	1000	1
1019	0.468650372	NA	NA		0
1972	0.551904017	NA	NA		1
2053	0.471428571	NA	NA		1
3628	0.489061076		276	770	1
2323	0.521338506		794	945	1
2717	0.540669856		1000	1000	1
1729	0.524971623	NA	NA		0
4920	0.526422664	NA	NA		0
2739	0.498554176	NA	NA		1
2511	0.545155993	NA	NA		1
2175	0.549425287	NA	NA		1
3348	0.554183813	NA	NA		0
1542	0.460832745	NA	NA		1
1591	0.491514771	NA	NA		1
1321	0.54468599	NA	NA		1
3737	0.500567537		1000	1000	0
1560	0.494978479	NA	NA		1
4636	0.498618021	NA	NA		1
327	0.46641791	NA	NA		1
1232	0.462915601	NA	NA		1
973	0.504624872	NA	NA		1
1124	0.538028169	NA	NA		1
641	0.484662577	NA	NA		1
4045	0.487831067		564	971	1
2326	0.542657343	NA	NA		1
1648	0.526297814	NA	NA		1
5647	0.475318656		318	1000	1
7947	0.509948215		873	884	1
1129	0.61	NA	NA		1
1273	0.546875	NA	NA		1
49513	0.496545017	NA	NA		0
2480	0.420044215	NA	NA		1
2739	0.520701231	NA	NA		0
917	0.442424242	NA	NA		1
2869	0.426629488		1000	1000	1
6272	0.528397264	NA	NA		1
14913	0.508330611	NA	NA		1
1828	0.573309396	NA	NA		1
1172	0.494779116	NA	NA		1
813	0.482164822	NA	NA		1
1057	0.464362851	NA	NA		1
902	0.471867008	NA	NA		1
3771	0.597122302	NA	NA		1
993	0.497482377	NA	NA		1

2029	0.472750978	NA	NA	0
2502	0.496107868	1000	1000	0
1144	0.518375242	NA	NA	1
3953	0.498315789	878	944	0
1856	0.502155172	NA	NA	1
1167	0.559062218	NA	NA	1
3765	0.531259172	NA	NA	1
770	0.494350282	NA	NA	1
1272	0.509747979	NA	NA	1
2738	0.482563619	NA	NA	1
854	0.573737374	NA	NA	1
17335	0.489651897	NA	NA	1
28825	0.53640416	1000	596	1
1962	0.450980392	NA	NA	1
2956	0.498220641	935	911	0
4961	0.47490686	815	883	1
6757	0.496382289	212	681	0
4327	0.475401851	539	940	1
1039	0.492849285	NA	NA	1
1932	0.475198413	NA	NA	0
2496	0.463098986	NA	NA	1
531	0.435028249	NA	NA	1
823	0.411764706	NA	NA	1
1676	0.487980769	NA	NA	1
1776	0.438626126	NA	NA	1
1798	0.467741935	NA	NA	1
1516	0.526595745	NA	NA	1
2992	0.509771987	532	878	1
798	0.483651226	NA	NA	1
1246	0.514233242	NA	NA	1
1502	0.513313609	NA	NA	1
2462	0.507875895	895	877	1
2127	0.52997003	935	940	1
3092	0.494533221	NA	NA	1
24811	0.478698683	NA	NA	0
5592	0.481233376	NA	NA	1
1246	0.548003398	NA	NA	1
507	0.565420561	NA	NA	1
1063	0.475703325	NA	NA	1
3275	0.464237517	NA	NA	1
4569	0.476374156	NA	NA	0
4044	0.442870633	1000	924	1
1752	0.574200913	NA	NA	1
1688	0.509274874	NA	NA	0
1409	0.54772141	NA	NA	1
1294	0.495291902	NA	NA	0
1984	0.484601449	NA	NA	1
1916	0.49825784	NA	NA	1
2407	0.447619048	NA	NA	1
2353	0.485395645	NA	NA	1
10555	0.534987042	110	892	1
947	0.486455982	NA	NA	1
644	0.572981366	NA	NA	1
1223	0.485690924	NA	NA	1
15423	0.578884367	80	1000	1
912	0.498923187	NA	NA	1
738	0.451016636	NA	NA	0

20113	0.527538856	141	707	1
7744	0.495862884 NA	NA		1
4153	0.479814443	540	943	0
1424	0.513137558 NA	NA		1
1231	0.476754015 NA	NA		1
1364	0.533538936 NA	NA		1
15919	0.514293227	318	1000	0
559	0.429338104 NA	NA		1
2837	0.525989331	941	948	1
4245	0.49254122	885	937	1
2964	0.521021611	859	883	0
1983	0.4528403 NA	NA		1
1503	0.457165733 NA	NA		1
2977	0.496402878	942	1000	1
1499	0.559706471 NA	NA		1
5394	0.387069211 NA	NA		1
1496	0.463362069 NA	NA		1
978	0.535787321 NA	NA		1
1111	0.538825758 NA	NA		1
2262	0.515915119 NA	NA		1
2272	0.486795775	1000	1000	1
2210	0.504072398	1000	1000	1
25483	0.48082995 NA	NA		1
43139	0.509441102 NA	NA		0
1747	0.525472238 NA	NA		1
1174	0.521582734 NA	NA		1
633	0.541864139 NA	NA		1
3064	0.550618673 NA	NA		1
5492	0.523303097	1000	1000	1
2280	0.498141264 NA	NA		1
3525	0.535181237 NA	NA		1
4426	0.481362842 NA	NA		0
3137	0.498056995 NA	NA		1
4547	0.540047313 NA	NA		1
1733	0.492733564 NA	NA		0
1687	0.485526316 NA	NA		1
1490	0.507621951 NA	NA		1
1474	0.480399693 NA	NA		1
1448	0.459627329 NA	NA		1
42067	0.502248876 NA	NA		1
3938	0.484683653	794	875	1
5817	0.509947306 NA	NA		0
1300	0.476074615 NA	NA		1
862	0.445679012 NA	NA		1
2083	0.481481481 NA	NA		1
11770	0.493576222 NA	NA		0
21185	0.432379811	308	1000	0
2035	0.420250896 NA	NA		1
3528	0.480728051 NA	NA		1
3015	0.490696826 NA	NA		1
2234	0.48468849 NA	NA		0
2040	0.476190476 NA	NA		1
1959	0.483920368 NA	NA		1
1973	0.461281337 NA	NA		1
7567	0.530335628	278	816	1
1073	0.380392157 NA	NA		1
62295	0.499176277 NA	NA		0

1638	0.458485958	NA	NA		1
1680	0.443452381	NA	NA		1
5478	0.516602938		543	884	0
1438	0.478442281	NA	NA		1
1823	0.45861678	NA	NA		1
1811	0.427145709	NA	NA		1
2451	0.426512968		780	1000	1
22135	0.521047046		462	977	1
2649	0.443965517		825	876	1
2474	0.458697765		878	849	1
2981	0.540090772	NA	NA		1
964	0.502097902	NA	NA		1
1478	0.483950617	NA	NA		1
1891	0.551778656	NA	NA		1
1102	0.51465798	NA	NA		1
1785	0.476383266	NA	NA		1
1146	0.456395349	NA	NA		1
1894	0.526613817	NA	NA		1
1273	0.488180884	NA	NA		1
1143	0.537604457	NA	NA		1
7420	0.52106451		940	936	0
1700	0.484441733	NA	NA		1
2034	0.514184397	NA	NA		1
1263	0.497708524	NA	NA		1
1273	0.429852084	NA	NA		1
1086	0.458536585	NA	NA		1
802	0.459459459	NA	NA		1
2050	0.494756911	NA	NA		0
4792	0.534162644	NA	NA		0
3902	0.42777413	NA	NA		1
1357	0.448374761	NA	NA		1
2099	0.494976677	NA	NA		0
4111	0.489253759		790	940	0
2066	0.523760331	NA	NA		1
4486	0.497837924	NA	NA		0
4437	0.516636048	NA	NA		1
968	0.494834711	NA	NA		1
644	0.463554464	NA	NA		1
2459	0.534064929	NA	NA		1
2280	0.531330996	NA	NA		0
14599	0.559946516	NA	NA		1
2206	0.458263305	NA	NA		1
3042	0.460674157	NA	NA		1
6096	0.523331661	NA	NA		1
2583	0.494365507	NA	NA		1
6670	0.478881317	NA	NA		0
11400	0.495977011	NA	NA		1
4376	0.469376392		822	816	1
3015	0.475144092		938	939	1
3493	0.537646722	NA	NA		1
1781	0.432902864	NA	NA		1
1689	0.432062966	NA	NA		1
2199	0.535327488	NA	NA		1
3197	0.46177164	NA	NA		1
1034	0.518404908	NA	NA		1
4860	0.493722944	NA	NA		1
3003	0.505875554		934	976	0

7048	0.50800173	NA	NA		1
1854	0.501945525	NA	NA		1
2032	0.51145959	NA	NA		1
985	0.484358145	NA	NA		1
796	0.516666667	NA	NA		1
1164	0.483285578	NA	NA		1
2013	0.564309764	NA	NA		1
11577	0.529886016	NA	NA		0
11595	0.472781655	NA	NA		0
4265	0.519320843	NA	NA		1
1136	0.540160643	NA	NA		1
871	0.514056225	NA	NA		1
843	0.550415184	NA	NA		1
1740	0.555555556	NA	NA		1
786	0.448275862	NA	NA		1
5719	0.493617021	NA	NA		0
7704	0.490779588	NA	NA		0
11831	0.497135302	NA	NA		0
23576	0.472031696	143		1000	0
2067	0.529753266	1000		1000	1
1779	0.52260274	NA	NA		1
14675	0.538930689	829		1000	1
2694	0.502689487	608		851	1
1787	0.527377522	NA	NA		1
6542	0.444019383	325		884	0
767	0.5	NA	NA		1
3437	0.465956638	NA	NA		1
1177	0.463010864	NA	NA		1
467	0.456102784	NA	NA		1
504	0.476190476	NA	NA		1
1285	0.553307393	NA	NA		1
687	0.491994178	NA	NA		1
4413	0.519313827	NA	NA		0
2870	0.500491159	558		776	1
1933	0.487325401	NA	NA		1
4244	0.508369901	582		825	1
2715	0.474389283	936		887	1
1639	0.509456986	NA	NA		1
2164	0.515655965	NA	NA		1
38484	0.519415125	74		1000	0
4936	0.466150633	NA	NA		0
1085	0.449086162	NA	NA		1
3286	0.431573919	NA	NA		0
1178	0.449974346	NA	NA		1
914	0.481264637	NA	NA		1
988	0.456937799	NA	NA		1
1582	0.450345261	NA	NA		1
1879	0.421122995	NA	NA		1
1501	0.431976167	NA	NA		1
3291	0.43880597	864		813	1
1728	0.457746479	NA	NA		1
5420	0.438720981	367		1000	0
1827	0.521620142	NA	NA		1
7794	0.532055123	NA	NA		1
13832	0.519443566	NA	NA		1
864	0.406483791	NA	NA		1
2526	0.458668928	888		1000	1

8166	0.529657477	139	819	1
1883	0.471428571	NA	NA	1
1215	0.46884273	NA	NA	1
1560	0.461158192	NA	NA	0
1223	0.534545455	NA	NA	1
9079	0.508189785	480	929	1
1874	0.453419645	NA	NA	1
1547	0.473460722	NA	NA	1
550	0.49694501	NA	NA	1
1914	0.486007995	NA	NA	1
1305	0.467147436	NA	NA	1
3037	0.470063395	NA	NA	0
1402	0.482169391	NA	NA	1
1424	0.503311258	NA	NA	1
1396	0.482008996	NA	NA	1
1457	0.419790105	NA	NA	1
1478	0.404240283	NA	NA	1
5945	0.427694026	NA	NA	1
1981	0.490679095	NA	NA	1
1623	0.470383275	NA	NA	1
1651	0.42926491	NA	NA	1
1530	0.43197026	NA	NA	1
1699	0.445101907	NA	NA	1
1673	0.423740511	NA	NA	1
3922	0.49625	868	946	1
2152	0.518854415	NA	NA	1
1750	0.498235294	NA	NA	1
2241	0.485714286	NA	NA	1
1280	0.50078125	NA	NA	1
743	0.532974428	NA	NA	1
4960	0.517219113	1000	1000	1
2548	0.474547749	NA	NA	1
719	0.446130501	NA	NA	1
2005	0.481296758	1000	1000	1
17628	0.413907285	NA	NA	1
2266	0.527973601	1000	1000	1
13648	0.53208526	NA	NA	0
2117	0.539281102	NA	NA	1
12424	0.484699555	703	934	0
1610	0.460093897	NA	NA	1
818	0.465217391	NA	NA	1
1343	0.499111111	NA	NA	0
2824	0.504747811	932	727	0
1066	0.415572233	NA	NA	1
1653	0.432380952	NA	NA	1
919	0.418604651	NA	NA	1
765	0.464052288	NA	NA	1
680	0.421768707	NA	NA	1
1224	0.453431373	NA	NA	1
1096	0.528338136	NA	NA	1
10317	0.45959596	NA	NA	1
2693	0.45994832	NA	NA	1
5580	0.512547764	338	842	0
1530	0.541176471	NA	NA	1
1353	0.499210111	NA	NA	1
2269	0.480926431	1000	933	1
1456	0.468335788	NA	NA	1

32772	0.456463496	NA	NA		1
1304	0.491157556	NA	NA		1
846	0.519230769	NA	NA		1
724	0.522727273	NA	NA		1
877	0.502457002	NA	NA		1
848	0.545355191	NA	NA		1
1798	0.513564079	NA	NA		1
1744	0.532821229	NA	NA		1
22120	0.489615017		666	1000	0
1185	0.394092827	NA	NA		1
3995	0.47987043		109	887	1
1787	0.474408754	NA	NA		1
3078	0.528589994		1000	1000	1
14389	0.454998156		325	1000	0
1184	0.439189189	NA	NA		1
1748	0.494490358	NA	NA		1
2105	0.480153649	NA	NA		1
2052	0.439655172	NA	NA		1
1107	0.590136054	NA	NA		1
65140	0.451728248		494	1000	1
825	0.6	NA	NA		1
665	0.506766917	NA	NA		1
3827	0.53271028	NA	NA		1
1715	0.433512683	NA	NA		1
1815	0.437303088	NA	NA		1
8796	0.475768485		280	931	0
3102	0.448704389		351	912	0
9616	0.524921875		620	885	1
1607	0.567528736	NA	NA		1
2234	0.493182887		893	1000	1
1157	0.575618698	NA	NA		1
1113	0.488174078	NA	NA		1
5884	0.506415908	NA	NA		1
6939	0.546982429		376	1000	1
1082	0.438401776	NA	NA		1
1360	0.444043321	NA	NA		1
1512	0.468341709	NA	NA		1
3197	0.542477876	NA	NA		1
1795	0.467710372	NA	NA		1
2945	0.537485582		827	943	1
3071	0.524680704		934	1000	1
26311	0.545914069		676	1000	1
1836	0.567846608	NA	NA		1
1754	0.538831615	NA	NA		1
1064	0.45112782	NA	NA		1
968	0.523913043	NA	NA		1
860	0.469987229	NA	NA		1
5982	0.535738074	NA	NA		0
27781	0.546977167		711	769	1
2042	0.420010422	NA	NA		1
867	0.453658537	NA	NA		1
13069	0.547094852		150	1000	0
9599	0.515363881		591	1000	1
2651	0.517607973	NA	NA		1
26669	0.525565344	NA	NA		0
1433	0.464285714	NA	NA		1
3460	0.442194555		618	1000	0

14062	0.56204878	608	899	1
1046	0.495901639	NA	NA	1
3368	0.506898765	NA	NA	0
3756	0.52936533	942	866	1
890	0.471494607	NA	NA	1
1339	0.457611668	NA	NA	1
2751	0.53117506	NA	NA	1
1340	0.545522388	NA	NA	1
1246	0.524879615	NA	NA	1
1070	0.546360917	NA	NA	1
4534	0.532381352	880	699	1
2062	0.528705441	NA	NA	1
2410	0.541346974	1000	1000	1
1202	0.491727941	NA	NA	1
3543	0.522101217	814	935	1
1053	0.440645774	NA	NA	1
447	0.451901566	NA	NA	1
975	0.431794872	NA	NA	1
2750	0.556907378	1000	798	1
4832	0.551647332	401	1000	1
1442	0.454812638	NA	NA	1
2529	0.530357143	830	935	1
2388	0.465904092	885	1000	1
1307	0.522881356	NA	NA	1
1286	0.433164129	NA	NA	1
20585	0.504125692	331	956	0
7655	0.500271592	1000	538	0
1030	0.473786408	NA	NA	1
1236	0.473300971	NA	NA	1
5251	0.512192903	NA	NA	0
2348	0.545831414	838	1000	1
2127	0.522802069	NA	NA	1
2043	0.45437018	NA	NA	1
1189	0.4432	NA	NA	1
736	0.466895537	NA	NA	1
3692	0.477746654	NA	NA	1
1349	0.530763529	NA	NA	1
2009	0.518619934	NA	NA	1
1227	0.488646685	NA	NA	1
1212	0.525092937	NA	NA	1
1234	0.518783542	NA	NA	1
1316	0.519083969	NA	NA	1
3273	0.49164756	780	1000	1
42479	0.551512542	270	1000	1
3768	0.481458398	735	1000	1
3127	0.570564516	456	878	0
2000	0.526259378	NA	NA	1
2346	0.516024152	934	873	1
1216	0.499177632	NA	NA	1
1157	0.494020239	NA	NA	1
5357	0.557910674	580	504	1
21731	0.555727213	107	731	0
770	0.476635514	NA	NA	1
2786	0.564249821	NA	NA	1
1522	0.552311436	NA	NA	1
3388	0.475468165	565	884	1
1490	0.477982955	NA	NA	1

2292	0.558043876	996	947	1
1913	0.546408659	NA	NA	1
715	0.559440559	NA	NA	1
864	0.5	NA	NA	1
727	0.508940853	NA	NA	1
1566	0.553473849	NA	NA	1
1725	0.537484885	NA	NA	1
2102	0.502555366	NA	NA	1
675	0.542009885	NA	NA	1
4581	0.517894737	NA	NA	1
2038	0.495159059	NA	NA	0
1044	0.553043478	NA	NA	1
4435	0.541276596	630	1000	1
557	0.551166966	NA	NA	1
771	0.493706294	NA	NA	1
6837	0.552938767	102	1000	1
1137	0.53197405	NA	NA	1
2108	0.48495212	NA	NA	0
2160	0.532082922	921	945	1
1459	0.515421522	NA	NA	1
7821	0.508369846	618	657	0
1498	0.488472622	NA	NA	1
13043	0.549362223	NA	NA	1
2775	0.530177515	NA	NA	1
1265	0.498723404	NA	NA	1
1421	0.508888889	NA	NA	1
1987	0.523007856	NA	NA	1
5196	0.493676193	446	880	0
1515	0.519927536	NA	NA	1
1295	0.512588117	NA	NA	1
12230	0.484613151	1000	942	1
717	0.447328244	NA	NA	1
1959	0.542906876	NA	NA	1
3144	0.532415254	877	800	1
1181	0.454767726	NA	NA	1
3156	0.532630522	NA	NA	1
4889	0.547738693	NA	NA	1
2191	0.504489796	NA	NA	0
3478	0.544573643	NA	NA	1
2184	0.533469596	NA	NA	0
647	0.457597173	NA	NA	1
14332	0.548584299	446	1000	1
1210	0.550935551	NA	NA	1
7452	0.562250599	NA	NA	1
1446	0.492362983	NA	NA	1
5653	0.547874825	758	860	1
1439	0.535405872	NA	NA	1
6634	0.556712963	NA	NA	1
1903	0.47240752	NA	NA	1
916	0.477759473	NA	NA	1
812	0.376010782	NA	NA	1
583	0.501893939	NA	NA	1
664	0.472891566	NA	NA	1
2617	0.567073171	1000	1000	1
3076	0.525878004	780	941	1
8289	0.560027285	NA	NA	1
2428	0.529274005	NA	NA	1

3576	0.558434082	NA	NA		1
1991	0.436792453	NA	NA		1
2132	0.451178451	NA	NA		1
2096	0.486009732	NA	NA		1
2689	0.520707506	687		942	1
1500	0.469896194	NA	NA		1
8521	0.508229665	NA	NA		0
1927	0.478491066	NA	NA		0
1911	0.514316586	NA	NA		1
11452	0.507122507	NA	NA		1
1036	0.501930502	NA	NA		1
658	0.546474359	NA	NA		1
810	0.506566604	NA	NA		0
854	0.540689655	NA	NA		1
14185	0.494835455	NA	NA		1
1822	0.492864984	NA	NA		1
3792	0.538821954	144		914	1
1712	0.50780762	NA	NA		1
573	0.457242583	NA	NA		1
2302	0.437219731	NA	NA		1
2702	0.451006893	1000		1000	0
6811	0.554765455	218		462	0
1337	0.511092851	NA	NA		1
3486	0.486347087	NA	NA		0
10220	0.493340078	692		1000	0
652	0.424846626	NA	NA		1
2451	0.503178928	NA	NA		1
2752	0.511904762	348		1000	1
3893	0.522239729	NA	NA		0
1490	0.535191638	NA	NA		1
3187	0.555971419	NA	NA		0
780	0.526475038	NA	NA		1
7756	0.535323677	354		773	1
23134	0.505307	546		1000	0
1739	0.515923567	NA	NA		1
737	0.495251018	NA	NA		1
1534	0.514519732	NA	NA		1
1440	0.542568543	NA	NA		1
975	0.467300832	NA	NA		1
743	0.49127907	NA	NA		1
892	0.484337349	NA	NA		1
2867	0.49047619	487		937	1
2174	0.509027081	NA	NA		1
2561	0.479880137	NA	NA		1
2568	0.52869032	829		886	1
1567	0.4671875	NA	NA		1
3701	0.48651035	NA	NA		0
1302	0.462404871	NA	NA		0
32636	0.48022054	1000		1000	0
12780	0.507779349	NA	NA		0
1948	0.372686343	NA	NA		1
9640	0.372231451	466		516	0
2505	0.355525965	NA	NA		1
15174	0.392415393	369		724	0
27805	0.31062048	476		1000	0
2977	0.365120681	886		1000	0
15022	0.417738343	698		1000	0

6951	0.439566598	942	1000	1
12495	0.414781605	NA	NA	0
2499	0.471147749	NA	NA	1
2815	0.373958191	1000	1000	0
5660	0.386608696	287	943	1
10432	0.378879853	508	494	1
15741	0.397828447	NA	NA	0
2047	0.444260486	NA	NA	0
696	0.479885057	NA	NA	1
16082	0.417956656	NA	NA	0
11627	0.448081862	329	391	1
7523	0.434650456	NA	NA	1
12463	0.456743257	374	960	1
1838	0.434621711	NA	NA	0
1810	0.504371217	NA	NA	1
11005	0.404378264	301	1000	0
7672	0.431575785	417	749	0
2307	0.43435692	940	1000	1
13363	0.363477586	485	1000	0
45352	0.357842621	612	1000	0
1120	0.490168539	NA	NA	0
1242	0.360188088	NA	NA	1
2963	0.392484342	NA	NA	1
56866	0.404224034	NA	NA	0
1665	0.362186788	NA	NA	1
803	0.515566625	NA	NA	1
184	0.39673913	NA	NA	1
5974	0.406448337	NA	NA	0
13713	0.378519371	459	877	0
9812	0.38869064	138	940	1
10227	0.398978247	998	919	0
1549	0.363087248	NA	NA	1
7056	0.410888648	503	1000	0
395988	0.374703289	287	688	1
7587	0.379920932	37	944	0
2953	0.603965562	560	837	0
11034	0.489795918	109	757	0
176671	0.342905405	NA	NA	1
1613	0.377755511	NA	NA	1
1379	0.505249344	NA	NA	1
1352	0.480729167	NA	NA	0
2001	0.529494382	NA	NA	1
2241	0.517021865	NA	NA	1
11450	0.529705442	NA	NA	1
2372	0.503218884	NA	NA	1
2884	0.461283186	NA	NA	0
871	0.539408867	NA	NA	1
1810	0.504144687	NA	NA	1
1810	0.506685769	NA	NA	0
1788	0.523353293	NA	NA	1
2906	0.480934809	602	931	1
3572	0.507845504	NA	NA	1
2750	0.539636364	1000	1000	1
6644	0.535974418	881	1000	1
2008	0.511494253	NA	NA	1
3239	0.578653923	NA	NA	0
1236	0.587330317	NA	NA	1

669	0.473941368	NA	NA		1
779	0.42618742	NA	NA		1
792	0.400252525	NA	NA		1
1060	0.380188679	NA	NA		1
974	0.417864476	NA	NA		1
961	0.439735828	NA	NA		0
940	0.39787234	NA	NA		1
6377	0.507013442	NA	NA		0
4788	0.415224269		721	1000	1
4930	0.513219285	NA	NA		1
2173	0.487197232	NA	NA		1
3471	0.492249288	NA	NA		1
2273	0.54885344		878	924	1
869	0.477900552	NA	NA		1
8357	0.456347301		868	1000	1
2761	0.537780238		861	864	0
9022	0.48494535		294	847	1
1534	0.419144307	NA	NA		1
1968	0.423719056	NA	NA		1
1943	0.453731343	NA	NA		1
2123	0.456288776	NA	NA		1
1652	0.429292929	NA	NA		1
1801	0.433429395	NA	NA		1
1720	0.456298975	NA	NA		1
1679	0.454320988	NA	NA		1
1617	0.470624613	NA	NA		1
4090	0.531425652		882	1000	1
27997	0.470393375	NA	NA		1
4413	0.510847556		564	942	1
2766	0.496229261	NA	NA		1
3019	0.507926443		712	932	1
3042	0.467424242		627	1000	1
3711	0.45598665		413	1000	1
3591	0.533617337	NA	NA		0
506	0.513833992	NA	NA		1
1193	0.505607021	NA	NA		0
29657	0.488662023	NA	NA		0
23413	0.487122338	NA	NA		0
8368	0.494137451	NA	NA		0
1800	0.489361702	NA	NA		0
16757	0.487044754		642	883	0
1531	0.531025474	NA	NA		1
2507	0.538131962	NA	NA		1
1981	0.498700455	NA	NA		1
2341	0.522504892	NA	NA		1
1457	0.526661797	NA	NA		1
543	0.456709957	NA	NA		1
723	0.490878939	NA	NA		1
2591	0.548455804	NA	NA		0
881	0.536889898	NA	NA		1
1741	0.581275129	NA	NA		1
987	0.442501681	NA	NA		1
4428	0.512410912		938	947	1
1301	0.564950038	NA	NA		1
1632	0.524156188	NA	NA		1
2173	0.495167971	NA	NA		1
12720	0.460194963	NA	NA		1

1106	0.542495479	NA	NA	1
1168	0.567343173	NA	NA	1
3465	0.500514403	NA	NA	0
966	0.508281573	NA	NA	1
1883	0.508684035	NA	NA	0
783	0.554278416	NA	NA	1
2160	0.500925926	1000	1000	1
1913	0.522891566	NA	NA	0
491	0.470468432	NA	NA	1
1669	0.472021066	NA	NA	1
3791	0.455433455	NA	NA	1
8562	0.46265293	NA	NA	1
1955	0.502338009	NA	NA	1
2792	0.537373198	NA	NA	0
1054	0.551252847	NA	NA	1
2345	0.564748201	934	1000	0
2532	0.544570502	NA	NA	1
7662	0.459459459	656	659	0
9547	0.562926673	252	1000	0
25837	0.509174767	NA	NA	0
641	0.502340094	NA	NA	1
8652	0.552961083	595	1000	1
1814	0.553256239	NA	NA	1
1470	0.484381686	NA	NA	0
1105	0.504819277	NA	NA	1
60617	0.46269341	330	1000	0
1108	0.564981949	NA	NA	1
1747	0.515358362	NA	NA	1
946	0.426365796	NA	NA	1
3402	0.542950514	891	598	1
1031	0.506304559	NA	NA	1
17671	0.533290029	NA	NA	0
643	0.536547434	NA	NA	1
828	0.422425033	NA	NA	1
4213	0.46287703	736	879	1
6110	0.5513382	647	838	1
4245	0.455085585	834	829	1
4195	0.430981164	840	874	1
4364	0.432511295	743	882	0
1892	0.459290188	NA	NA	0
1938	0.521573948	NA	NA	1
865	0.47556391	NA	NA	1
622	0.407407407	NA	NA	1
1049	0.404398564	NA	NA	0
1887	0.438391699	NA	NA	1
1098	0.500421941	NA	NA	0
458	0.403930131	NA	NA	1
853	0.444900352	NA	NA	1
312	0.435897436	NA	NA	1
1871	0.45215311	NA	NA	1
440	0.518181818	NA	NA	1
10596	0.461965812	NA	NA	1
382	0.49235474	NA	NA	1
19673	0.412426489	NA	NA	1
5394	0.389638424	NA	NA	1
826	0.43001443	NA	NA	1
758	0.564643799	NA	NA	1

8317	0.523296355	NA	NA	1
408	0.426214482	NA	NA	0
384	0.604361371	NA	NA	1
2509	0.575492341	NA	NA	1
577	0.51187905	NA	NA	1
2713	0.487631184	NA	NA	0
1421	0.474903475	NA	NA	1
1367	0.479057592	NA	NA	1
495	0.357512953	NA	NA	1
564	0.425531915	NA	NA	1
409	0.439759036	NA	NA	1
569	0.501953125	NA	NA	1
548	0.516460905	NA	NA	1
1167	0.486111111	NA	NA	1
517	0.531960997	NA	NA	1
690	0.425760286	NA	NA	1
632	0.409810127	NA	NA	1
517	0.473887814	NA	NA	1
409	0.452322738	NA	NA	1
586	0.467505241	NA	NA	1
2019	0.558007566	NA	NA	1
676	0.520249221	NA	NA	1
544	0.490521327	NA	NA	1
729	0.431952663	NA	NA	1
507	0.529255319	NA	NA	1
431	0.48	NA	NA	1
443	0.503386005	NA	NA	1
51595	0.427776059	NA	NA	1
607	0.431630972	NA	NA	1
1024	0.439135381	NA	NA	1
371	0.456666667	NA	NA	1
399	0.446808511	NA	NA	1
430	0.472972973	NA	NA	1
80443	0.518095686	NA	NA	1
762	0.455399061	NA	NA	1
811	0.469333333	NA	NA	1
124023	0.497505543	NA	NA	1
1395	0.453976205	NA	NA	1
5821	0.488636364	NA	NA	0
3876	0.455876022	NA	NA	0
468	0.408119658	NA	NA	1
386	0.417098446	NA	NA	1
795	0.504926108	NA	NA	1
470	0.485074627	NA	NA	1
613	0.549382716	NA	NA	1
670	0.590233546	NA	NA	1
420	0.538095238	NA	NA	1
380	0.552631579	NA	NA	1
424	0.558659218	NA	NA	1
2586	0.396582056	NA	NA	0
3054	0.44332211	NA	NA	1
588	0.436911488	NA	NA	1
919	0.446808511	NA	NA	0
776	0.508406187	NA	NA	0
578	0.454545455	NA	NA	1
425	0.478142077	NA	NA	1
72289	0.391870766	NA	NA	1

541	0.473140496	NA	NA		1
1009	0.420057542	NA	NA		1
428	0.448598131	NA	NA		1
21675	0.517596567	NA	NA		1
559	0.553444181	NA	NA		1
594	0.490777339	NA	NA		0
319	0.548589342	NA	NA		1
768	0.541889483	NA	NA		1
508	0.486220472	NA	NA		1
415	0.438554217	NA	NA		1
436	0.516055046	NA	NA		1
438	0.398119122	NA	NA		1
9560	0.447126682	NA	NA		0
316	0.484177215	NA	NA		1
435	0.479224377	NA	NA		1
1062	0.577656676	NA	NA		1
483	0.455607477	NA	NA		1
388	0.53271028	NA	NA		1
904	0.52679109	NA	NA		1
1073	0.432172869	NA	NA		1
460	0.473913043	NA	NA		1
876	0.410958904	NA	NA		1
374	0.451871658	NA	NA		1
407	0.437346437	NA	NA		1
838	0.459197787	NA	NA		1
192	0.505208333	NA	NA		1
538	0.457249071	NA	NA		1
951	0.434516524	NA	NA		1
932	0.488145049	NA	NA		1
222	0.698198198	NA	NA		1
614	0.459923664	NA	NA		1
10316	0.37274781	622		355	1
3980	0.299884393	NA	NA		1
3503	0.508138352	NA	NA		1
80386	0.516075946	NA	NA		0
2934	0.441149635	651		1000	1
56635	0.546348553	NA	NA		0
38262	0.523020928	520		1000	1
2100	0.526592455	NA	NA		0
948	0.438818565	NA	NA		1
2561	0.554852321	1000		839	1
67126	0.51692503	627		989	0
23716	0.512276269	383		873	0
4989	0.475	489		944	0
8842	0.550642971	754		1000	0
1596	0.45882871	NA	NA		0
3724	0.466872111	937		829	1
7809	0.498755806	263		1000	0
1896	0.485080058	NA	NA		1
22450	0.529584639	190		1000	0
1179	0.413082437	NA	NA		1
1471	0.437765205	NA	NA		1
1280	0.468692449	NA	NA		1
2293	0.507422402	NA	NA		1
2337	0.480786416	NA	NA		0
2404	0.527027027	1000		816	1
861	0.51061008	NA	NA		1

963	0.496666667	NA	NA	1
943	0.487922705	NA	NA	0
17126	0.444333726	NA	NA	0
1895	0.566684812	NA	NA	1
24444	0.358099037	NA	NA	0
1856	0.511853448	NA	NA	1
2570	0.556214421	NA	NA	1
1962	0.493390716	NA	NA	0
2889	0.594494048	NA	NA	1
1753	0.491313412	NA	NA	1
5767	0.547858392	242	792	1
1676	0.439089692	NA	NA	1
5624	0.488036065	NA	NA	0
25644	0.526088982	NA	NA	0
2570	0.510060833	622	945	1
5843	0.547623556	936	1000	1
996	0.552230047	NA	NA	0
917	0.482295482	NA	NA	1
2023	0.483828671	NA	NA	0
1696	0.502242152	NA	NA	1
2279	0.504780115	NA	NA	1
2412	0.526650756	NA	NA	1
1754	0.457461646	NA	NA	1
2481	0.46473029	NA	NA	1
1415	0.450080515	NA	NA	1
454	0.531088083	NA	NA	1
1441	0.474025974	NA	NA	1
1948	0.517679558	NA	NA	1
1440	0.473646209	NA	NA	1
1586	0.409205549	NA	NA	1
1058	0.438563327	NA	NA	1
17341	0.457672575	243	1000	0
1120	0.44874477	NA	NA	1
1373	0.529259897	NA	NA	1
3467	0.491929134	NA	NA	1
2131	0.478304239	941	975	1
3797	0.459705882	NA	NA	1
41412	0.435855386	NA	NA	0
770	0.527272727	NA	NA	1
889	0.465270121	NA	NA	1
667	0.498141264	NA	NA	1
1548	0.466960352	NA	NA	1
945	0.459976105	NA	NA	1
1294	0.507692308	NA	NA	1
664	0.426666667	NA	NA	1
4253	0.483476356	NA	NA	1
30190	0.539998075	1000	1000	0
12321	0.489755512	NA	NA	0
8054	0.345287557	217	1000	1
1676	0.50440806	NA	NA	1
1507	0.500331785	NA	NA	1
2429	0.54713874	1000	1000	1
2646	0.514997693	919	805	1
869	0.458167331	NA	NA	1
9206	0.46706364	NA	NA	0
1613	0.508088235	NA	NA	1
3237	0.472662908	1000	1000	1

1379	0.455482662	NA	NA		0
5065	0.488353668	339		1000	1
1833	0.525032938	NA	NA		1
944	0.508474576	NA	NA		1
981	0.523955148	NA	NA		1
465	0.394472362	NA	NA		1
3998	0.431984074	NA	NA		0
563	0.355599214	NA	NA		1
568	0.388118812	NA	NA		1
594	0.397790055	NA	NA		1
1726	0.429644809	NA	NA		1
448	0.412658228	NA	NA		1
1661	0.423913043	NA	NA		1
748	0.49127907	NA	NA		1
720	0.498466258	NA	NA		1
2114	0.5359375	NA	NA		1
1921	0.544623656	NA	NA		1
8725	0.511676804	168		769	0
4413	0.51566307	NA	NA		1
1803	0.505135387	NA	NA		1
1828	0.484094053	NA	NA		1
20152	0.452535125	99		908	0
4873	0.53970193	286		1000	1
3537	0.458838148	821		875	1
19687	0.530750837	NA	NA		1
4856	0.544019564	NA	NA		1
26597	0.420479303	NA	NA		0
536	0.577731092	NA	NA		1
1225	0.505747126	NA	NA		1
3166	0.482917026	1000		634	0
549	0.4958159	NA	NA		1
823	0.464713715	NA	NA		1
8877	0.526172301	NA	NA		0
4254	0.502283105	711		994	1
64104	0.410202522	NA	NA		0
4641	0.531207289	NA	NA		1
6300	0.35267035	NA	NA		0
682	0.538053097	NA	NA		1
7436	0.476498237	NA	NA		1
8822	0.464870768	1000		1000	1
1566	0.512359551	NA	NA		1
1969	0.514664804	NA	NA		1
10025	0.453778517	752		822	1
6016	0.444698355	111		800	0
1798	0.412053718	NA	NA		1
1245	0.452020202	NA	NA		1
1862	0.483524355	NA	NA		1
3563	0.483908046	NA	NA		0
3428	0.499772727	NA	NA		0
3428	0.498250525	NA	NA		1
1832	0.467741935	NA	NA		1
7423	0.431352327	669		873	0
1221	0.441100602	NA	NA		1
6149	0.460362268	942		932	0
3294	0.478571429	226		1000	1
3397	0.463431151	NA	NA		0
890	0.496332518	NA	NA		1

7424	0.491001698	NA	NA		0
1671	0.528710726	NA	NA		1
24627	0.506776665	323		1000	0
3716	0.528333333	645		921	1
1228	0.529315961	NA	NA		1
340	0.367647059	NA	NA		1
2247	0.53617633	NA	NA		0
27854	0.553644554	NA	NA		0
2712	0.47238348	NA	NA		0
4233	0.466056205	NA	NA		1
854	0.463446475	NA	NA		1
1663	0.515828678	NA	NA		1
915	0.430219146	NA	NA		1
1662	0.527166882	NA	NA		1
2314	0.507550886	NA	NA		1
1184	0.444256757	NA	NA		1
42418	0.351216469	NA	NA		1
1772	0.516930023	NA	NA		1
6146	0.505908655	NA	NA		0
1775	0.483383686	NA	NA		1
18606	0.337152678	NA	NA		1
12147	0.398050366	NA	NA		1
35260	0.32881172	NA	NA		1
1451	0.467336683	NA	NA		1
1533	0.440313112	NA	NA		1
1463	0.465481887	NA	NA		1
18262	0.524255157	NA	NA		0
1339	0.52420307	NA	NA		1
896	0.493775934	NA	NA		1
4267	0.407051282	NA	NA		1
760	0.428571429	NA	NA		1
1182	0.418490397	NA	NA		0
896	0.485221675	NA	NA		1
2901	0.395023328	NA	NA		1
1316	0.377753304	NA	NA		1
913	0.476510067	NA	NA		1
1195	0.454271357	NA	NA		1
1867	0.439544808	NA	NA		1
1059	0.455467869	NA	NA		1
6523	0.428519627	839		970	1
1223	0.485930736	NA	NA		1
2512	0.254012175	937		1000	1
757	0.447443182	NA	NA		1
13185	0.466161779	1000		941	1
35623	0.520762712	871		1000	0
501	0.443946188	NA	NA		1
1651	0.37037037	NA	NA		1
6095	0.510894426	NA	NA		1
1620	0.434365782	NA	NA		1
856	0.445828144	NA	NA		1
540	0.411111111	NA	NA		1
809	0.593325093	NA	NA		1
1061	0.456797967	NA	NA		1
9029	0.504884122	64		873	0
1417	0.452276065	NA	NA		1
1393	0.500374532	NA	NA		1
957	0.566901408	NA	NA		1

5943	0.515108497	NA	NA		1
709	0.568518519	NA	NA		1
3621	0.491278035		823	881	1
3417	0.465307582		834	891	1
760	0.430555556	NA	NA		1
894	0.423462089	NA	NA		1
897	0.506410256	NA	NA		1
1144	0.487132353	NA	NA		1
1627	0.411930484	NA	NA		0
1580	0.42513465	NA	NA		1
571	0.495621716	NA	NA		1
2367	0.421424367	NA	NA		1
670	0.497014925	NA	NA		1
610	0.516393443	NA	NA		1
666	0.445544554	NA	NA		1
786	0.451751251	NA	NA		1
12407	0.480248963		1000	1000	1
1821	0.446601942	NA	NA		1
1821	0.446601942	NA	NA		1
757	0.413940256	NA	NA		1
747	0.477503628	NA	NA		1
2969	0.430026913	NA	NA		1
1079	0.481613286	NA	NA		1
1048	0.428571429	NA	NA		1
1024	0.514184397	NA	NA		1
34647	0.54925776		529	1000	1
1918	0.488595438	NA	NA		1
525	0.493333333	NA	NA		1
1094	0.468217054	NA	NA		1
1258	0.371057514	NA	NA		1
992	0.431396786	NA	NA		1
762	0.459317585	NA	NA		1
817	0.505249344	NA	NA		1
1943	0.536664504	NA	NA		1
691	0.429027113	NA	NA		1
6765	0.55	NA	NA		1
601	0.42047532	NA	NA		1
1344	0.447900467	NA	NA		1
1224	0.469771242	NA	NA		1
57637	0.534773577	NA	NA		1
9956	0.516908908		747	942	1
2851	0.465153325	NA	NA		1
2528	0.441931274	NA	NA		1
6941	0.527148462		799	869	0
23790	0.593323217	NA	NA		1
1541	0.511578947	NA	NA		1
1096	0.516441006	NA	NA		1
1621	0.531717263	NA	NA		0
980	0.553317536	NA	NA		1
826	0.447941889	NA	NA		1
3381	0.446965052		917	1000	1
1529	0.45323741	NA	NA		1
636	0.555031447	NA	NA		1
6885	0.549851559	NA	NA		1
752	0.563829787	NA	NA		1
1924	0.454487384	NA	NA		1
6056	0.533991228	NA	NA		0

626	0.483362522	NA	NA		1
587	0.527272727	NA	NA		1
3147	0.498610146		833	1000	1
1877	0.412855377	NA	NA		1
3004	0.509725906	NA	NA		1
8680	0.560393727		1000	753	1
2125	0.463579898	NA	NA		1
2831	0.578982597	NA	NA		1
4547	0.461579367	NA	NA		1
906	0.544502618	NA	NA		1
5301	0.558757485		113	668	0
785	0.534954407	NA	NA		1
4931	0.47461644	NA	NA		1
2433	0.486020503	NA	NA		1
982	0.498924731	NA	NA		1
1253	0.438423645	NA	NA		1
1193	0.456802383	NA	NA		1
1080	0.496458467	NA	NA		1
1122	0.476439791	NA	NA		1
1035	0.464075383	NA	NA		1
1175	0.492409867	NA	NA		1
1819	0.542372881	NA	NA		1
1809	0.479975728	NA	NA		1
8322	0.516778523		586	728	1
7134	0.47994101		247	886	0
921	0.504885993	NA	NA		1
648	0.515432099	NA	NA		1
688	0.534551231	NA	NA		1
5459	0.514946962	NA	NA		1
1471	0.497556207	NA	NA		1
1303	0.486985726	NA	NA		1
1193	0.535624476	NA	NA		1
1243	0.328029949	NA	NA		1
3264	0.475440222		656	844	1
3208	0.46285457		427	878	1
437	0.427821522	NA	NA		1
1525	0.46295082	NA	NA		1
2320	0.53215513		943	828	0
2027	0.52712766	NA	NA		1
57488	0.524508831		72	1000	0
1112	0.513489209	NA	NA		1
2075	0.489156627	NA	NA		1
1552	0.451030928	NA	NA		1
2075	0.491925065	NA	NA		0
474	0.434599156	NA	NA		1
1692	0.521462639	NA	NA		1
8567	0.518164436		596	1000	0
667	0.52640264	NA	NA		1
658	0.471666667	NA	NA		1
1603	0.522516083	NA	NA		1
941	0.539851222	NA	NA		1
24958	0.568370987	NA	NA		1
1154	0.467937608	NA	NA		1
1915	0.449324324	NA	NA		1
439	0.523917995	NA	NA		1
1359	0.489379779	NA	NA		1
1359	0.519346049	NA	NA		0

1199	0.417184265	NA	NA	1
12281	0.519360932	422	1000	0
1238	0.485875706	NA	NA	1
1504	0.470079787	NA	NA	1
1265	0.440246046	NA	NA	1
1216	0.472039474	NA	NA	1
3275	0.47050983	949	1000	1
4672	0.383203952	638	630	1
1000	0.518	NA	NA	1
1191	0.498239437	NA	NA	1
2160	0.435927443	NA	NA	1
3182	0.465095195	NA	NA	0
773	0.519431988	NA	NA	0
1259	0.556632878	NA	NA	1
1160	0.520017994	NA	NA	1
528	0.462121212	NA	NA	1
1813	0.534163987	NA	NA	1
1920	0.480477223	NA	NA	1
843	0.523131673	NA	NA	1
11832	0.53755785	NA	NA	1
17298	0.458607096	710	1000	1
11155	0.512817412	NA	NA	0
861	0.474440895	NA	NA	1
2012	0.441114774	NA	NA	0
1535	0.392857143	NA	NA	1
1120	0.421845574	NA	NA	1
2482	0.545603495	NA	NA	1
880	0.435227273	NA	NA	1
52885	0.387247912	NA	NA	0
410	0.312195122	NA	NA	1
2035	0.527302813	NA	NA	1
5218	0.563072894	77	891	1
860	0.374893617	NA	NA	1
4676	0.493202957	821	934	0
707	0.579901153	NA	NA	1
21272	0.51026264	NA	NA	0
2606	0.459337349	NA	NA	0
2602	0.47362607	NA	NA	1
57924	0.479899641	NA	NA	0
1785	0.424089636	NA	NA	1
1704	0.379746835	NA	NA	1
1802	0.519495413	NA	NA	1
1791	0.414293691	NA	NA	1
4263	0.513379992	68	883	1
905	0.356147022	NA	NA	1
744	0.414048059	NA	NA	1
589	0.499151104	NA	NA	1
3102	0.464539007	NA	NA	1
943	0.509580838	NA	NA	1
728	0.399725275	NA	NA	1
1157	0.479316051	NA	NA	1
1643	0.474226804	NA	NA	1
4221	0.4912847	NA	NA	1
1569	0.489841986	NA	NA	1
2375	0.508631579	NA	NA	1
44847	0.477675998	888	1000	0
1396	0.511800841	NA	NA	0

5363	0.510379384	NA	NA		1
1938	0.524108193	NA	NA		0
1717	0.470126735	NA	NA		1
17374	0.540052528	NA	NA		1
2114	0.447338618	NA	NA		1
1985	0.461084906	NA	NA		1
2446	0.487429451	NA	NA		1
4268	0.431302575		783	1000	1
1964	0.457897972	NA	NA		1
1836	0.480614973	NA	NA		1
3487	0.540532456		940	1000	1
4573	0.495497881		901	814	0
1739	0.506195225	NA	NA		1
1824	0.491776316	NA	NA		1
825	0.484415584	NA	NA		1
4784	0.547144557		300	753	1
2507	0.514529915		891	1000	1
837	0.565714286	NA	NA		1
5106	0.45072041		1000	1000	1
23652	0.531617413	NA	NA		1
398	0.479411765	NA	NA		1
968	0.54011976	NA	NA		1
820	0.493902439	NA	NA		1
707	0.49328215	NA	NA		1
9577	0.516205448	NA	NA		0
2008	0.508688784	NA	NA		1
2590	0.511836212	NA	NA		1
20660	0.503779543		318	818	1
2894	0.532352941	NA	NA		0
818	0.466666667	NA	NA		1
3028	0.42669363		939	1000	1
3047	0.493775934	NA	NA		1
1514	0.467684478	NA	NA		0
2807	0.47266436	NA	NA		1
1536	0.463778409	NA	NA		1
2270	0.443225453		945	934	0
41700	0.465946844		378	1000	0
653	0.516239316	NA	NA		1
1651	0.434664861	NA	NA		1
1602	0.498311951	NA	NA		1
1515	0.468950749	NA	NA		1
2133	0.464942529	NA	NA		1
2027	0.448389218	NA	NA		1
1502	0.426193922	NA	NA		1
2132	0.483151184	NA	NA		0
2410	0.452807646	NA	NA		1
1158	0.461809636	NA	NA		1
1102	0.426470588	NA	NA		1
1043	0.507614213	NA	NA		1
15964	0.505975114		213	768	0
1305	0.489754098	NA	NA		1
1748	0.48741581	NA	NA		1
4645	0.528754886	NA	NA		1
6237	0.509900152	NA	NA		1
1451	0.563059959	NA	NA		1
645	0.502546689	NA	NA		1
1224	0.414215686	NA	NA		1

1488	0.480510753	NA	NA		1
1367	0.514885496	NA	NA		1
20053	0.531912801	54		1000	1
1020	0.433922997	NA	NA		1
616	0.47763864	NA	NA		1
63906	0.527550711	70		1000	1
36626	0.500213803	420		1000	1
10268	0.547790339	1000		1000	1
879	0.425141243	NA	NA		1
4983	0.458137347	NA	NA		1
1785	0.444780635	NA	NA		1
21088	0.524384957	NA	NA		1
2679	0.50877193	826		827	1
1790	0.478991597	NA	NA		1
162292	0.49104985	NA	NA		0
834	0.364771151	NA	NA		1
756	0.454946996	NA	NA		0
914	0.46875	NA	NA		1
30835	0.551300668	75		626	0
36318	0.450147493	NA	NA		0
5097	0.487615712	NA	NA		1
1070	0.532082922	NA	NA		1
1105	0.526775956	NA	NA		1
4561	0.436083672	NA	NA		1
1686	0.472716489	NA	NA		1
1634	0.482864137	NA	NA		1
974	0.449291167	NA	NA		1
1066	0.396551724	NA	NA		1
996	0.431137725	NA	NA		1
3322	0.520467836	328		943	1
1557	0.465944272	NA	NA		0
2100	0.500736377	NA	NA		1
1264	0.508713693	NA	NA		1
1677	0.438282648	NA	NA		1
1211	0.514550265	NA	NA		1
416	0.370192308	NA	NA		1
2373	0.510932619	1000		868	1
7732	0.504483377	NA	NA		0
1142	0.451838879	NA	NA		1
1589	0.451381781	NA	NA		1
1373	0.453687822	NA	NA		1
1172	0.466856061	NA	NA		1
1224	0.414215686	NA	NA		1
5123	0.471143531	867		803	1
2177	0.50495566	NA	NA		1
5959	0.416524286	286		891	0
897	0.520624303	NA	NA		1
1035	0.529896907	NA	NA		1
1087	0.497285559	NA	NA		1
1699	0.423544067	NA	NA		1
1076	0.493062967	NA	NA		1
3096	0.481622912	NA	NA		1
929	0.501165501	NA	NA		1
2217	0.493440969	NA	NA		0
1559	0.437395099	NA	NA		1
2115	0.461286805	NA	NA		1
1598	0.472040668	NA	NA		1

7439	0.54754822	NA	NA		0
504	0.478651685	NA	NA		1
994	0.469318182	NA	NA		1
1782	0.490629183	NA	NA		1
31499	0.510547132		851	1000	1
4050	0.484296768	NA	NA		1
16459	0.449934555	NA	NA		0
23321	0.46362188	NA	NA		1
2070	0.513344691	NA	NA		1
2719	0.463773446	NA	NA		1
1419	0.486194478	NA	NA		1
5480	0.50534524	NA	NA		1
3735	0.512829057		670	930	1
5885	0.43597561	NA	NA		1
865	0.485549133	NA	NA		1
10722	0.558673682	NA	NA		0
1645	0.525227964	NA	NA		1
3688	0.468659315	NA	NA		1
706	0.355590062	NA	NA		1
16545	0.500176908		423	780	0
29521	0.470482056		203	922	0
9588	0.497132345	NA	NA		1
1523	0.400568182	NA	NA		1
2159	0.537820726	NA	NA		1
880	0.486860304	NA	NA		1
14245	0.551786307	NA	NA		0
1459	0.477153558	NA	NA		1
36194	0.413680782		65	1000	1
2300	0.501977587	NA	NA		1
962	0.504994451	NA	NA		1
1704	0.457746479	NA	NA		1
764	0.514397906	NA	NA		1
863	0.506903353	NA	NA		1
1783	0.421200224	NA	NA		1
3405	0.507631709		568	397	1
1148	0.552631579	NA	NA		1
717	0.481171548	NA	NA		1
717	0.507670851	NA	NA		1
1534	0.433161954	NA	NA		0
2957	0.4719078		459	930	0
1475	0.466226138	NA	NA		1
908	0.505980861	NA	NA		1
2858	0.487482117		938	1000	1
1339	0.462674323	NA	NA		1
3919	0.487306297		402	941	1
1563	0.469535415	NA	NA		1
1863	0.478110599	NA	NA		1
753	0.487839771	NA	NA		1
3181	0.445531637	NA	NA		1
1694	0.447461629	NA	NA		1
781	0.435562806	NA	NA		1
1350	0.480225989	NA	NA		0
1196	0.519877676	NA	NA		1
520	0.43558952	NA	NA		1
1136	0.430944964	NA	NA		1
1286	0.519417476	NA	NA		1
1004	0.452191235	NA	NA		1

3175	0.483779528	NA	NA	1
526	0.420042644	NA	NA	1
21469	0.464127148	NA	NA	0
2034	0.438995569	NA	NA	1
1171	0.39795047	NA	NA	1
646	0.419689119	NA	NA	1
2704	0.487426036	1000	1000	1
35021	0.501122705	NA	NA	1
2158	0.544949027	NA	NA	1
1875	0.616782676	NA	NA	1
999	0.562562563	NA	NA	1
945	0.541798942	NA	NA	1
1130	0.52300885	NA	NA	1
3958	0.540714786	946	812	0
7112	0.543358396	NA	NA	1
1090	0.360602799	NA	NA	1
3061	0.400820203	755	962	1
727	0.442622951	NA	NA	1
550	0.390243902	NA	NA	1
33132	0.454993283	NA	NA	0
44949	0.504125798	568	1000	0
1077	0.508147254	NA	NA	1
913	0.432905484	NA	NA	1
1556	0.457123098	NA	NA	1
14540	0.501183514	1000	862	1
1421	0.492272025	NA	NA	1
5865	0.4829896	NA	NA	1
24035	0.401325563	NA	NA	0
2011	0.507692308	NA	NA	1
504	0.41627907	NA	NA	1
32263	0.492345164	159	827	1
3696	0.437623762	NA	NA	1
893	0.501679731	NA	NA	1
946	0.432816537	NA	NA	1
860	0.453852021	NA	NA	0
22369	0.532287582	152	1000	1
22036	0.512791991	NA	NA	1
2106	0.467820443	NA	NA	1
21921	0.473640465	NA	NA	1
2256	0.491257755	NA	NA	1
1456	0.494232476	NA	NA	1
1598	0.455329154	NA	NA	1
1894	0.438642298	NA	NA	1
662	0.433534743	NA	NA	1
16814	0.514780726	NA	NA	0
1176	0.472172352	NA	NA	1
573	0.402390438	NA	NA	1
949	0.407797682	NA	NA	1
1180	0.470216606	NA	NA	1
716	0.423184358	NA	NA	1
2332	0.567858103	1000	1000	1
18405	0.545393239	NA	NA	0
44607	0.543440736	NA	NA	0
5892	0.480509745	NA	NA	1
544	0.482180294	NA	NA	1
654	0.45412844	NA	NA	1
1750	0.435038213	NA	NA	1

1776	0.433700058	NA	NA	1
322	0.422360248	NA	NA	1
1526	0.55193662	NA	NA	1
19532	0.425847458	331	1000	1
2981	0.462395543	NA	NA	1
3376	0.478985991	NA	NA	1
618	0.466071429	NA	NA	1
903	0.379370629	NA	NA	1
6172	0.468857335	320	816	0
858	0.465822785	NA	NA	1
3733	0.409917064	1000	853	1
82494	0.527150408	NA	NA	1
5918	0.531062874	875	809	1
11663	0.484287132	NA	NA	0
578	0.437716263	NA	NA	1
1512	0.430209569	NA	NA	1
907	0.428015564	NA	NA	1
1084	0.455719557	NA	NA	1
5805	0.433462116	NA	NA	1
2484	0.417626218	NA	NA	0
27434	0.490133089	NA	NA	1
1143	0.452117864	NA	NA	1
2493	0.477336542	NA	NA	1
88439	0.482173476	NA	NA	1
2187	0.455284553	NA	NA	1
1357	0.392439244	NA	NA	1
706	0.387129724	NA	NA	1
1713	0.472690492	NA	NA	1
8814	0.471967288	1000	804	0
2655	0.405361495	1000	807	1
1308	0.470691164	NA	NA	1
840	0.441666667	NA	NA	1
409	0.375358166	NA	NA	1
5266	0.482902515	491	941	1
4825	0.503212716	NA	NA	1
3367	0.451282051	948	1000	1
2031	0.480920021	NA	NA	1
823	0.455650061	NA	NA	1
985	0.454251884	NA	NA	1
856	0.387850467	NA	NA	1
2258	0.43691242	888	880	1
530	0.432075472	NA	NA	1
712	0.382022472	NA	NA	1
4835	0.448979592	NA	NA	1
1422	0.437134503	NA	NA	1
1333	0.423105776	NA	NA	1
718	0.408644401	NA	NA	1
491	0.436554133	NA	NA	1
1393	0.491044776	NA	NA	1
720	0.46749226	NA	NA	1
679	0.428571429	NA	NA	1
1225	0.506938776	NA	NA	1
930	0.488479263	NA	NA	1
881	0.509803922	NA	NA	1
860	0.387209302	NA	NA	1
1406	0.488023952	NA	NA	1
1142	0.415841584	NA	NA	1

718	0.391364903	NA	NA		1
2483	0.428068632	NA	NA		1
813	0.418699187	NA	NA		1
973	0.447969543	NA	NA		1
1155	0.512373969	NA	NA		1
1149	0.510599078	NA	NA		1
839	0.462455304	NA	NA		1
29070	0.498096102	NA	NA		0
3547	0.463911351		1000	875	1
1677	0.495378928	NA	NA		1
6886	0.427421265	NA	NA		0
834	0.450839329	NA	NA		1
3214	0.382389546		1000	1000	1
2983	0.467836257	NA	NA		1
1437	0.432283465	NA	NA		1
1534	0.407482993	NA	NA		1
694	0.347058824	NA	NA		1
863	0.534183082	NA	NA		1
2596	0.477606025	NA	NA		1
609	0.402414487	NA	NA		1
819	0.500633714	NA	NA		1
1351	0.478164323	NA	NA		1
2076	0.511078998		1000	1000	1
16304	0.509253139		1000	946	1
1287	0.435207824	NA	NA		0
786	0.41184868	NA	NA		1
482	0.541493776	NA	NA		1
1177	0.515717927	NA	NA		1
633	0.533965245	NA	NA		1
1025	0.521920668	NA	NA		1
709	0.409961686	NA	NA		1
2604	0.506772244		911	1000	0
979	0.455621302	NA	NA		1
676	0.406804734	NA	NA		1
40003	0.463395622		59	1000	0
1902	0.462028986	NA	NA		1
2609	0.484044055	NA	NA		1
2609	0.476881233	NA	NA		1
1208	0.453677173	NA	NA		1
11204	0.494963224	NA	NA		0
12328	0.458886598		32	1000	0
1386	0.372881356	NA	NA		1
9264	0.378917638		280	1000	1
10947	0.401884887		194	490	1
10905	0.368440577		102	929	0
4104	0.36722973		1000	291	1
12115	0.369468038		1000	501	1
5603	0.369580023		1000	496	1
6851	0.388871424		884	860	1
8696	0.319511875		135	1000	0
581	0.472380952	NA	NA		1
783	0.420027816	NA	NA		1
2382	0.479848866	NA	NA		1
1208	0.433270677	NA	NA		1
3111	0.538821328	NA	NA		1
997	0.511494253	NA	NA		1
931	0.454417952	NA	NA		1

1015	0.379732739	NA	NA		1
1425	0.462609971	NA	NA		1
11521	0.476353748		672	1000	0
2170	0.519553073	NA	NA		1
1971	0.48	NA	NA		1
820	0.385365854	NA	NA		1
111992	0.511002365	NA	NA		0
2074	0.486981678	NA	NA		1
1046	0.441048035	NA	NA		1
484	0.421348315	NA	NA		1
1156	0.45357524	NA	NA		1
369	0.550135501	NA	NA		1
462	0.504926108	NA	NA		1
2212	0.44354403	NA	NA		0
1646	0.522622951	NA	NA		1
1772	0.429403202	NA	NA		1
1867	0.439693682	NA	NA		1
27594	0.525554484	NA	NA		1
1664	0.444225722	NA	NA		1
1469	0.490810075	NA	NA		1
1467	0.525562372	NA	NA		1
1057	0.483443709	NA	NA		1
2259	0.463289963		893	1000	1
713	0.470588235	NA	NA		1
4766	0.398584384		893	1000	1
4027	0.361454913		932	1000	1
1421	0.365200765	NA	NA		1
1376	0.449854651	NA	NA		1
20345	0.431755225		472	1000	1
3350	0.460787923	NA	NA		0
13416	0.430442177	NA	NA		1
1620	0.357051282	NA	NA		1
787	0.465057179	NA	NA		1
1839	0.517086835	NA	NA		1
2692	0.57883924	NA	NA		1
5969	0.531445876		930	1000	0
16601	0.532692624		575	478	1
972	0.373493976	NA	NA		1
6289	0.43629622		515	1000	0
2860	0.406038935	NA	NA		1
29160	0.511454754	NA	NA		1
2379	0.420954163		759	1000	1
774	0.515503876	NA	NA		1
811	0.381011097	NA	NA		1
2054	0.546738072	NA	NA		1
4686	0.48812709	NA	NA		0
3208	0.422671353		700	799	1
421	0.534441805	NA	NA		1
1450	0.55505279	NA	NA		1
4194	0.546630728	NA	NA		1
2372	0.500238663		817	938	1
1422	0.321012658	NA	NA		1
1754	0.381179273	NA	NA		1
6190	0.402749282	NA	NA		1
1556	0.40682095	NA	NA		1
2243	0.399323181	NA	NA		1
1216	0.418624891	NA	NA		1

23618	0.428523602	NA	NA		1
1346	0.492165899	NA	NA		1
72572	0.469525648	NA	NA		1
1341	0.457486137	NA	NA		1
573	0.529182879	NA	NA		1
518	0.551198257	NA	NA		1
521	0.534632035	NA	NA		1
1175	0.399148936	NA	NA		1
1113	0.407221665	NA	NA		1
921	0.427440633	NA	NA		1
1724	0.49369483	NA	NA		1
1949	0.5	NA	NA		1
2031	0.434761201	NA	NA		1
766	0.455813953	NA	NA		1
1405	0.598086124	NA	NA		1
1937	0.396203307	NA	NA		1
1698	0.418138987	NA	NA		1
627	0.643884892	NA	NA		1
1012	0.550998948	NA	NA		1
2353	0.533259012	NA	NA		0
1031	0.491836735	NA	NA		1
1777	0.479253112	NA	NA		1
913	0.405960265	NA	NA		0
3489	0.571428571	NA	NA		0
1036	0.507722008	NA	NA		1
803	0.453300125	NA	NA		1
91164	0.509016712	NA	NA		0
1275	0.420392157	NA	NA		1
757	0.482889734	NA	NA		1
559	0.380368098	NA	NA		1
387	0.457364341	NA	NA		1
2034	0.534414946	NA	NA		1
2034	0.534414946	NA	NA		1
925	0.530810811	NA	NA		1
850	0.512941176	NA	NA		1
334	0.452095808	NA	NA		1
309	0.478964401	NA	NA		1
361	0.465373961	NA	NA		1
582	0.422319475	NA	NA		1
47066	0.553107545	NA	NA		0
1408	0.445273632	NA	NA		1
1408	0.441886581	NA	NA		0
3954	0.500371637		834	891	1
1077	0.516248839	NA	NA		1
661	0.485627837	NA	NA		1
4095	0.505007153		771	893	1
32624	0.533719881		349	158	1
817	0.515312916	NA	NA		1
1211	0.464079273	NA	NA		1
3028	0.395061728	NA	NA		1
18411	0.568284898		207	819	0
3003	0.505729565		859	875	0
2204	0.518394649	NA	NA		1
1095	0.407597536	NA	NA		1
410	0.390243902	NA	NA		1
32608	0.496122428		829	1000	0
108541	0.416352201	NA	NA		1

1855	0.467312349	NA	NA	1
1035	0.436923077	NA	NA	1
985	0.41420765	NA	NA	1
987	0.457638426	NA	NA	1
1701	0.506973924	NA	NA	1
26037	0.51240129	NA	NA	1
481	0.501196172	NA	NA	1
751	0.460218409	NA	NA	1
1513	0.474219317	NA	NA	1
1395	0.521774856	NA	NA	1
1616	0.520704846	NA	NA	1
1167	0.403209629	NA	NA	1
122124	0.433501975	NA	NA	0
16801	0.524955437	NA	NA	0
4028	0.483953422	NA	NA	0
11017	0.497873127	NA	NA	1
2185	0.453089245	1000	1000	1
4315	0.436197917	796	870	1
1744	0.482932289	NA	NA	0
1089	0.423119266	NA	NA	1
1352	0.565088757	NA	NA	1
707	0.47706422	NA	NA	0
880	0.436053593	NA	NA	1
37254	0.480200049	549	1000	0
2007	0.510437882	NA	NA	1
621	0.401360544	NA	NA	1
1477	0.432412791	NA	NA	1
882	0.425170068	NA	NA	1
1243	0.551890587	NA	NA	1
1081	0.497053045	NA	NA	1
10133	0.51948858	NA	NA	0
2811	0.384762817	NA	NA	1
1559	0.52894356	NA	NA	1
1107	0.467307692	NA	NA	1
1104	0.503846154	NA	NA	1
6324	0.564921513	659	847	0
856	0.529562982	NA	NA	1
2210	0.542435424	NA	NA	1
16877	0.552861093	620	932	1
582	0.4604811	NA	NA	1
1191	0.392947103	NA	NA	1
5150	0.504528986	NA	NA	1
75862	0.513949843	NA	NA	0
940	0.524627721	NA	NA	1
40542	0.547186844	NA	NA	0
3193	0.553191489	NA	NA	1
96514	0.522921693	NA	NA	1
35732	0.578234835	377	1000	1
397	0.508816121	NA	NA	1
2462	0.426976744	NA	NA	1
817	0.507955936	NA	NA	1
3166	0.589176471	416	1000	1
1447	0.586731168	NA	NA	1
1734	0.363872645	NA	NA	1
633	0.499210111	NA	NA	1
1030	0.521359223	NA	NA	1
1540	0.550512445	NA	NA	1

1900	0.55908799	NA	NA	0
1475	0.549618321	NA	NA	1
2569	0.414572864	NA	NA	1
2254	0.439552508	NA	NA	1
1323	0.41567696	NA	NA	1
870	0.417690418	NA	NA	1
1499	0.504336224	NA	NA	1
17627	0.470713525	NA	NA	1
670	0.43207856	NA	NA	1
1025	0.478887745	NA	NA	1
1021	0.478800414	NA	NA	1
1267	0.609106529	NA	NA	1
653	0.427350427	NA	NA	1
15121	0.499953104	510	805	0
22144	0.503748284	362	1000	0
3159	0.544556267	774	998	1
1395	0.503491078	NA	NA	1
1686	0.531158511	NA	NA	1
5919	0.441830896	NA	NA	1
642	0.456386293	NA	NA	1
497	0.461711712	NA	NA	1
496	0.465011287	NA	NA	1
1339	0.530059272	NA	NA	1
2038	0.449760766	NA	NA	1
8387	0.514681208	NA	NA	1
2309	0.550401427	1000	933	1
1167	0.566409597	NA	NA	1
3862	0.40333423	1000	929	1
13864	0.527483693	353	976	0
1316	0.408	NA	NA	1
485	0.354107649	NA	NA	1
61877	0.483748708	508	1000	1
502	0.524456522	NA	NA	1
2385	0.519496855	NA	NA	1
878	0.454441913	NA	NA	1
871	0.451205511	NA	NA	1
3458	0.428571429	NA	NA	1
1070	0.418691589	NA	NA	1
4189	0.526946819	152	945	0
83131	0.531137293	NA	NA	0
10345	0.541147254	939	411	0
3835	0.508773567	NA	NA	1
890	0.391515152	NA	NA	1
17188	0.477002631	NA	NA	1
60864	0.51152815	NA	NA	0
8439	0.470864153	NA	NA	1
12644	0.530002643	555	942	1
6323	0.51702462	712	1000	1
948	0.474285714	NA	NA	1
2937	0.505164499	867	1000	1
930	0.5	NA	NA	1
1403	0.399857448	NA	NA	1
726	0.530679934	NA	NA	1
704	0.463068182	NA	NA	1
1167	0.379023884	NA	NA	1
4252	0.402218782	770	914	1
1011	0.440158259	NA	NA	1

524	0.3930131	NA	NA		1
647	0.428571429	NA	NA		1
10682	0.5		990	947	1
474	0.556962025	NA	NA		1
776	0.524671053	NA	NA		0
12903	0.550368799	NA	NA		1
520	0.503184713	NA	NA		1
1931	0.483647799	NA	NA		1
1461	0.52264291	NA	NA		1
2170	0.43060686	NA	NA		1
17113	0.535242291	NA	NA		0
9733	0.479567535		155	1000	0
5814	0.494817787		650	1000	1
223590	0.441416894	NA	NA		1
1668	0.462111801	NA	NA		1
14189	0.476688103	NA	NA		0
1246	0.488073394	NA	NA		0
853	0.462017435	NA	NA		1
402	0.402985075	NA	NA		1
11936	0.546707715	NA	NA		0
5827	0.418502203	NA	NA		1
6299	0.532986111	NA	NA		1
154062	0.387615014	NA	NA		0
721	0.485212569	NA	NA		1
3216	0.579881657	NA	NA		0
3216	0.579881657		560	861	0
914	0.415754923	NA	NA		1
1856	0.357219828	NA	NA		1
868	0.411851852	NA	NA		1
1042	0.462571977	NA	NA		1
1671	0.484739677	NA	NA		1
2653	0.377227723	NA	NA		1
28128	0.513314968		653	1000	1
1182	0.422165821	NA	NA		1
1445	0.50055371	NA	NA		1
730	0.410958904	NA	NA		1
2019	0.519564141	NA	NA		1
674	0.39898563	NA	NA		1
2148	0.545279383	NA	NA		1
1383	0.421677215	NA	NA		1
8677	0.509979751		704	749	0
706	0.40368272	NA	NA		1
3208	0.387275833	NA	NA		0
37971	0.526508606		190	581	0
968	0.472107438	NA	NA		1
1037	0.476374156	NA	NA		1
829	0.48854041	NA	NA		1
623	0.522504892	NA	NA		1
2139	0.538698894	NA	NA		1
3013	0.474164134	NA	NA		1
5024	0.465027212		937	1000	1
845	0.49930459	NA	NA		1
1527	0.476911977	NA	NA		1
718	0.48189415	NA	NA		1
366	0.478142077	NA	NA		1
1370	0.502469136	NA	NA		1
4727	0.470619375	NA	NA		0

855	0.533919598	NA	NA		1
2761	0.504002287	NA	NA		1
1229	0.430331754	NA	NA		1
7334	0.483899043	89		925	1
3259	0.4	NA	NA		0
2144	0.474345974	NA	NA		1
1636	0.467912266	NA	NA		1
45364	0.483870968	NA	NA		0
5398	0.55583524	938		308	0
1461	0.470284238	NA	NA		1
1875	0.47581139	NA	NA		0
1026	0.426900585	NA	NA		1
16104	0.439128004	1000		876	0
1160	0.407300672	NA	NA		1
2251	0.498019242	NA	NA		1
3852	0.425369198	940		1000	1
1750	0.495623632	NA	NA		1
991	0.543895055	NA	NA		1
1367	0.534016094	NA	NA		1
5373	0.444518717	871		928	0
640	0.453608247	NA	NA		1
3394	0.555123217	197		1000	1
53952	0.559413315	NA	NA		0
36393	0.459765977	NA	NA		1
6017	0.497555576	NA	NA		0
825	0.524848485	NA	NA		1
633	0.470359572	NA	NA		1
2936	0.408743928	NA	NA		0
3322	0.438689548	883		1000	1
27745	0.513067266	NA	NA		1
20909	0.436016035	NA	NA		1
6490	0.438037075	NA	NA		1
26634	0.499705232	NA	NA		0
22271	0.501724462	NA	NA		0
677	0.488921713	NA	NA		1
1545	0.548867314	NA	NA		1
340	0.441605839	NA	NA		1
2661	0.579475485	898		927	1
112493	0.532784555	NA	NA		1
3424	0.436226415	NA	NA		1
693	0.382405745	NA	NA		1
18390	0.496079614	NA	NA		0
41268	0.525746811	NA	NA		0
1292	0.504643963	NA	NA		1
6179	0.329131022	603		943	1
668	0.403897254	NA	NA		1
1512	0.509259259	NA	NA		1
2867	0.481718464	NA	NA		0
18196	0.465888689	NA	NA		1
1199	0.450731707	NA	NA		1
920	0.442521632	NA	NA		1
1500	0.453333333	NA	NA		1
1863	0.468245021	NA	NA		1
2001	0.465550547	NA	NA		1
724	0.52553542	NA	NA		1
724	0.527182867	NA	NA		1
726	0.519736842	NA	NA		1

723	0.52640264	NA	NA	1
724	0.527182867	NA	NA	1
724	0.52553542	NA	NA	1
725	0.523178808	NA	NA	1
724	0.523887974	NA	NA	1
724	0.52553542	NA	NA	1
724	0.52553542	NA	NA	1
724	0.52553542	NA	NA	1
724	0.528830313	NA	NA	1
973	0.389499389	NA	NA	1
389	0.563636364	NA	NA	1
1088	0.408713693	NA	NA	1
1266	0.453143535	NA	NA	1
798	0.400318979	NA	NA	1
849	0.451118963	NA	NA	1
8116	0.476102941	NA	NA	1
783	0.394803018	NA	NA	1
388	0.440721649	NA	NA	1
490	0.426530612	NA	NA	1
636	0.375959079	NA	NA	1
1307	0.482019893	NA	NA	1
2404	0.510331872	NA	NA	1
1890	0.467466835	NA	NA	1
886	0.426829268	NA	NA	1
1132	0.449646643	NA	NA	1
767	0.471968709	NA	NA	1
3889	0.432301154	775	826	1
3806	0.44823459	784	817	1
5260	0.494690988	NA	NA	1
2202	0.432993445	NA	NA	1
654	0.45412844	NA	NA	1
722	0.472012103	NA	NA	1
9249	0.428859229	NA	NA	0
2577	0.410543131	NA	NA	1
748	0.507327285	NA	NA	1
5176	0.435351563	944	1000	1
1475	0.478813559	NA	NA	1
1605	0.512588767	NA	NA	1
855	0.49625	NA	NA	1
704	0.467826087	NA	NA	1
966	0.359562842	NA	NA	1
1798	0.382048331	NA	NA	1
391	0.518518519	NA	NA	1
1904	0.484052533	NA	NA	1
1082	0.397515528	NA	NA	1
2082	0.536585366	NA	NA	1
576	0.505208333	NA	NA	1
2629	0.431593794	824	881	1
1296	0.470530452	NA	NA	1
1152	0.526041667	NA	NA	1
1062	0.438689218	NA	NA	1
1220	0.446167883	NA	NA	1
936	0.421307506	NA	NA	1
1128	0.418526786	NA	NA	1
1130	0.448577681	NA	NA	1
1141	0.420718816	NA	NA	1
1093	0.467592593	NA	NA	1

1207	0.470935961	NA	NA		1
1726	0.59212051	NA	NA		1
1982	0.411062225	NA	NA		1
655	0.386054422	NA	NA		1
618	0.36407767	NA	NA		1
1347	0.510906447	NA	NA		1
6005	0.366841091	NA	NA		0
1444	0.385646688	NA	NA		0
2281	0.440890919	NA	NA		1
1867	0.439541547	NA	NA		1
2148	0.545279383	NA	NA		1
1901	0.513416816	NA	NA		1
1187	0.491557223	NA	NA		1
1902	0.43772242	NA	NA		1
1399	0.43715847	NA	NA		1
9467	0.480868666		883	1000	0
21584	0.578815718		380	366	0
1238	0.487701442	NA	NA		1
51020	0.512008005	NA	NA		1
18319	0.553478671		131	871	0
6066	0.507352941		359	1000	0
569	0.507366483	NA	NA		1
694	0.436950147	NA	NA		1
69129	0.510602045	NA	NA		1
713	0.460822343	NA	NA		1
693	0.490990991	NA	NA		1
7367	0.50044964		373	1000	1
9206	0.497293396	NA	NA		1
630	0.415384615	NA	NA		1
5066	0.475049116	NA	NA		0
43831	0.538446836	NA	NA		1
73048	0.366002863		320	622	1
49813	0.528395228	NA	NA		0
28480	0.554253858	NA	NA		1
45933	0.560021416	NA	NA		1
618	0.426587302	NA	NA		1
709	0.355432781	NA	NA		1
829	0.433029909	NA	NA		1
15265	0.491979146		980	1000	1
780	0.346740638	NA	NA		1
762	0.377492877	NA	NA		1
39784	0.379451106		213	944	1
618	0.373881932	NA	NA		1
683	0.358974359	NA	NA		1
618	0.368515206	NA	NA		1
683	0.365384615	NA	NA		1
618	0.372093023	NA	NA		1
646	0.367972743	NA	NA		1
824	0.453389831	NA	NA		1
818	0.39602649	NA	NA		1
1311	0.412887828	NA	NA		1
15325	0.43010355	NA	NA		1
11426	0.378136201	NA	NA		1
993	0.482710927	NA	NA		1
837	0.355244755	NA	NA		1
733	0.391941392	NA	NA		1
757	0.413522013	NA	NA		1

616	0.487455197	NA	NA		1
593	0.472998138	NA	NA		1
671	0.462365591	NA	NA		1
779	0.378512397	NA	NA		1
552	0.392982456	NA	NA		1
678	0.418943534	NA	NA		1
2285	0.413636364	NA	NA		0
3117	0.532051282	NA	NA		1
558	0.36643026	NA	NA		1
8456	0.492972519	NA	NA		1
44221	0.483108935	NA	NA		1
1171	0.402220325	NA	NA		1
1283	0.49004329	NA	NA		1
1864	0.460283902	NA	NA		1
491	0.394799054	NA	NA		1
18065	0.359069054		339	1000	0
979	0.484153005	NA	NA		1
917	0.45497076	NA	NA		1
892	0.497601918	NA	NA		1
17620	0.530804412		363	956	0
5098	0.467657113	NA	NA		0
652	0.412563667	NA	NA		1
3514	0.455026455	NA	NA		1
2009	0.443379791	NA	NA		1
503	0.523489933	NA	NA		1
801	0.408369408	NA	NA		1
1026	0.557377049	NA	NA		1
343	0.466666667	NA	NA		1
840	0.447552448	NA	NA		1
1033	0.391152503	NA	NA		1
1249	0.429012346	NA	NA		1
686	0.453720508	NA	NA		1
735	0.436026936	NA	NA		1
737	0.344311377	NA	NA		1
943	0.554046072	NA	NA		1
616	0.45046729	NA	NA		1
1328	0.385191348	NA	NA		1
488	0.416861827	NA	NA		1
5363	0.389912281		780	830	1
514	0.468131868	NA	NA		1
11225	0.375757576	NA	NA		1
1336	0.392561983	NA	NA		1
624	0.425795053	NA	NA		1
766	0.383045526	NA	NA		1
2197	0.392070485	NA	NA		1
2614	0.43490701	NA	NA		1
1007	0.441239316	NA	NA		1
2124	0.382731424	NA	NA		1
1896	0.363863447	NA	NA		1
1150	0.463054187	NA	NA		1
4300	0.499139415	NA	NA		1
514	0.484234234	NA	NA		1
1420	0.51026616	NA	NA		1
1091	0.40434333	NA	NA		1
962	0.444444444	NA	NA		1
507	0.478980892	NA	NA		1
629	0.43853211	NA	NA		1

1414	0.497313891	NA	NA	1
1393	0.47743755	NA	NA	1
1439	0.370823146	NA	NA	1
1141	0.422751323	NA	NA	1
7345	0.365049751	NA	NA	1
1432	0.369694397	NA	NA	1
414	0.572463768	NA	NA	1
466	0.449029126	NA	NA	1
91146	0.334890966	NA	NA	1
10200	0.40804829	NA	NA	0
210	0.438095238	NA	NA	1
215672	0.341728012	256	302	0
291	0.52233677	NA	NA	1
1930	0.351851852	NA	NA	1
2207	0.394064303	NA	NA	0
11502	0.339340569	NA	NA	0
725	0.459459459	NA	NA	1
396	0.398989899	NA	NA	1
610	0.421311475	NA	NA	1
546	0.34981685	NA	NA	1
70611	0.349282997	459	283	1
1400	0.445714286	NA	NA	1
147259	0.408014572	NA	NA	1
177	0.525423729	NA	NA	1
391	0.475703325	NA	NA	1
214	0.355140187	NA	NA	1
6329	0.526218951	NA	NA	1
587	0.470187394	NA	NA	1
852	0.443526171	NA	NA	1
11137	0.513763029	991	1000	1
4984	0.502598998	NA	NA	0
1340	0.556521739	NA	NA	1
1340	0.556521739	NA	NA	1
633	0.439964943	NA	NA	1
4159	0.518659766	833	938	1
1525	0.459467822	NA	NA	0
1880	0.503073071	NA	NA	0
1591	0.5	NA	NA	1
6612	0.448804957	257	1000	0
698	0.482808023	NA	NA	1
1211	0.50148368	NA	NA	1
11906	0.422449754	NA	NA	0
1962	0.550654206	NA	NA	0
732	0.461350692	NA	NA	0
35247	0.482691998	NA	NA	1
729	0.489711934	NA	NA	1
769	0.464239272	NA	NA	1
749	0.472630174	NA	NA	1
719	0.47566064	NA	NA	1
821	0.503045067	NA	NA	1
751	0.496671105	NA	NA	1
883	0.42015855	NA	NA	1
889	0.512935883	NA	NA	1
16449	0.491125402	NA	NA	0
1160	0.538104089	NA	NA	1
1818	0.312203791	NA	NA	1
1414	0.454377026	NA	NA	0

312	0.429487179	NA	NA		1
1686	0.50245098	NA	NA		1
5960	0.458201893	NA	NA		0
12012	0.524524848	NA	NA		0
3797	0.4798868	NA	NA		0
1887	0.469426752	NA	NA		0
3077	0.470454545	NA	NA		1
1618	0.49197861	NA	NA		1
3339	0.521937843		1000	1000	1
1184	0.429192007	NA	NA		1
1282	0.45467033	NA	NA		0
1354	0.390008058	NA	NA		1
382	0.515706806	NA	NA		1
454	0.544052863	NA	NA		1
513	0.431309904	NA	NA		1
18975	0.51279711		120	827	0
1617	0.478120978	NA	NA		1
794	0.483532934	NA	NA		1
897	0.445930881	NA	NA		1
3340	0.511676647		1000	1000	1
815	0.438848921	NA	NA		1
768	0.45203252	NA	NA		1
10452	0.373569199	NA	NA		1
1979	0.412903226	NA	NA		1
1264	0.508702532	NA	NA		1
2667	0.470633694	NA	NA		1
4670	0.437667878		899	943	0
1776	0.536275243	NA	NA		1
5293	0.533333333	NA	NA		0
4440	0.536661466		347	933	0
3376	0.54606073	NA	NA		1
860	0.50067659	NA	NA		1
1044	0.451649602	NA	NA		1
1063	0.544455544	NA	NA		1
1535	0.550654721	NA	NA		1
1948	0.302180685	NA	NA		1
623	0.39543058	NA	NA		1
25380	0.365955473		350	879	0
1201	0.527751646	NA	NA		1
1752	0.453616055	NA	NA		1
67339	0.377653631	NA	NA		1
46292	0.460950129	NA	NA		0
4059	0.450404858		596	1000	0
7424	0.534210918	NA	NA		0
78	0.307692308	NA	NA		1
162	0.462962963	NA	NA		1
147	0.503401361	NA	NA		1
142	0.366197183	NA	NA		1
135	0.437037037	NA	NA		1
80	0.325	NA	NA		1
82	0.243902439	NA	NA		1
85	0.282352941	NA	NA		1
85	0.388235294	NA	NA		1
87	0.32183908	NA	NA		1
91	0.384615385	NA	NA		1
83	0.373493976	NA	NA		1
83	0.361445783	NA	NA		1

83	0.361445783	NA	NA	1
84	0.380952381	NA	NA	1
75	0.333333333	NA	NA	1
75	0.333333333	NA	NA	1
75	0.373333333	NA	NA	1
80	0.3	NA	NA	1
80	0.275	NA	NA	1
71	0.281690141	NA	NA	1
77	0.337662338	NA	NA	1
77	0.311688312	NA	NA	1
86	0.337209302	NA	NA	1
81	0.37037037	NA	NA	1
73	0.328767123	NA	NA	1
79	0.341772152	NA	NA	1
79	0.316455696	NA	NA	1
79	0.316455696	NA	NA	1
76	0.263157895	NA	NA	1
100	0.38	NA	NA	1
76	0.289473684	NA	NA	1
145	0.35862069	NA	NA	1
144	0.395833333	NA	NA	1
140	0.457142857	NA	NA	1
140	0.464285714	NA	NA	1
140	0.457142857	NA	NA	1
140	0.442857143	NA	NA	1
140	0.435714286	NA	NA	1
150	0.4	NA	NA	1
150	0.426666667	NA	NA	1
140	0.45	NA	NA	1
140	0.421428571	NA	NA	1
132	0.371212121	NA	NA	1
132	0.393939394	NA	NA	1
133	0.353383459	NA	NA	1
141	0.446808511	NA	NA	1
132	0.454545455	NA	NA	1
153	0.477124183	NA	NA	1
152	0.453947368	NA	NA	1
150	0.38	NA	NA	1
150	0.4	NA	NA	1
133	0.398496241	NA	NA	1
135	0.377777778	NA	NA	1
136	0.411764706	NA	NA	1
155	0.419354839	NA	NA	1
142	0.535211268	NA	NA	1
135	0.42962963	NA	NA	1
130	0.484615385	NA	NA	1
144	0.319444444	NA	NA	1
140	0.328571429	NA	NA	1
145	0.317241379	NA	NA	1
149	0.241610738	NA	NA	1
139	0.45323741	NA	NA	1
137	0.437956204	NA	NA	1
141	0.361702128	NA	NA	1
135	0.355555556	NA	NA	1
134	0.373134328	NA	NA	1
138	0.376811594	NA	NA	1
140	0.464285714	NA	NA	1

136	0.352941176	NA	NA		1
141	0.340425532	NA	NA		1
136	0.382352941	NA	NA		1
148	0.351351351	NA	NA		1
151	0.370860927	NA	NA		1
151	0.364238411	NA	NA		1
142	0.38028169	NA	NA		1
143	0.412587413	NA	NA		1
145	0.372413793	NA	NA		1
148	0.486486486	NA	NA		1
150	0.493333333	NA	NA		1
151	0.483443709	NA	NA		1
141	0.397163121	NA	NA		1
138	0.398550725	NA	NA		1
133	0.338345865	NA	NA		1
138	0.347826087	NA	NA		1
129	0.348837209	NA	NA		1
126	0.317460317	NA	NA		1
124	0.306451613	NA	NA		1
131	0.358778626	NA	NA		1
128	0.2734375	NA	NA		1
137	0.357664234	NA	NA		1
132	0.318181818	NA	NA		1
152	0.473684211	NA	NA		1
140	0.407142857	NA	NA		1
136	0.338235294	NA	NA		1
136	0.308823529	NA	NA		1
135	0.348148148	NA	NA		1
134	0.388059701	NA	NA		1
135	0.437037037	NA	NA		1
130	0.361538462	NA	NA		1
137	0.372262774	NA	NA		1
134	0.402985075	NA	NA		1
135	0.348148148	NA	NA		1
134	0.358208955	NA	NA		1
138	0.391304348	NA	NA		1
140	0.407142857	NA	NA		1
139	0.388489209	NA	NA		1
139	0.410071942	NA	NA		1
141	0.510638298	NA	NA		1
155	0.406451613	NA	NA		1
159	0.427672956	NA	NA		1
137	0.437956204	NA	NA		1
136	0.455882353	NA	NA		1
137	0.445255474	NA	NA		1
138	0.449275362	NA	NA		1
143	0.405594406	NA	NA		1
137	0.416058394	NA	NA		1
140	0.457142857	NA	NA		1
139	0.446043165	NA	NA		1
148	0.418918919	NA	NA		1
38247	0.508717684	NA	NA		0
1403	0.389202256	NA	NA		1
565	0.493887531	NA	NA		1
16682	0.440462911		345	1000	1
54245	0.538724689		50	921	0
293	0.368600683	NA	NA		1

1452	0.468436155	NA	NA		1
62346	0.479320113	NA	NA		1
617	0.546191248	NA	NA		1
556	0.411870504	NA	NA		1
679	0.382978723	NA	NA		1
31649	0.544492356	NA	NA		1
24213	0.468881119	NA	NA		1
76659	0.527675853		160	1000	1
56321	0.497131682		123	1000	1
1435	0.45320197	NA	NA		1
796	0.463567839	NA	NA		1
480	0.516509434	NA	NA		1
16302	0.517120281		167	988	0
30078	0.519552319	NA	NA		0
7022	0.52629891		885	944	1
707	0.535493827	NA	NA		1
10791	0.518550928		427	876	1
67309	0.545681582		611	1000	1
972	0.502057613	NA	NA		1
974	0.484599589	NA	NA		1
1684	0.509066488	NA	NA		1
366	0.462585034	NA	NA		1
5790	0.492206828		629	878	1
485	0.494845361	NA	NA		1
18986	0.4708318	NA	NA		0
2011	0.444759207	NA	NA		1
1018	0.473565804	NA	NA		0
931	0.411764706	NA	NA		1
40665	0.529932617	NA	NA		1
1901	0.487283237	NA	NA		1
3694	0.508536725	NA	NA		1
7474	0.536067893	NA	NA		1
2676	0.485269594	NA	NA		1
5347	0.494117647	NA	NA		1
31612	0.589714715		701	989	1
345	0.496453901	NA	NA		1
817	0.414613424	NA	NA		1
1126	0.500475285	NA	NA		1
669	0.466666667	NA	NA		1
275	0.629090909	NA	NA		1
91	0.285714286	NA	NA		1
91	0.318681319	NA	NA		1
2139	0.388947184	NA	NA		1
5646	0.420007637	NA	NA		1
1351	0.439674315	NA	NA		1
398	0.530150754	NA	NA		1
351	0.41025641	NA	NA		1
1109	0.430117223	NA	NA		1
950	0.404210526	NA	NA		1
1801	0.470713525	NA	NA		1
697	0.40655106	NA	NA		1
1840	0.401741294	NA	NA		1
532	0.364113326	NA	NA		1
859	0.444703143	NA	NA		1
579	0.452868852	NA	NA		1
607	0.418454936	NA	NA		1
661	0.526475038	NA	NA		1

531	0.463362069	NA	NA	1
625	0.3872	NA	NA	1
314	0.414012739	NA	NA	1
1284	0.424294269	NA	NA	1
486	0.418212479	NA	NA	1
363	0.359223301	NA	NA	1
863	0.453744493	NA	NA	1
662	0.394259819	NA	NA	1
401	0.523690773	NA	NA	1
1150	0.444444444	NA	NA	1
1150	0.447310648	NA	NA	1
2053	0.54062149	NA	NA	1
431	0.423280423	NA	NA	1
795	0.437735849	NA	NA	1
275	0.461818182	NA	NA	1
727	0.450729927	NA	NA	1
1003	0.420979021	NA	NA	1
498	0.464566929	NA	NA	1
531	0.373493976	NA	NA	1
2155	0.560784314	NA	NA	1
933	0.3808933	NA	NA	1
369	0.471544715	NA	NA	1
309	0.40776699	NA	NA	1
685	0.434412266	NA	NA	1
398	0.466076696	NA	NA	1
855	0.356598985	NA	NA	1
613	0.502427184	NA	NA	1
1229	0.434447301	NA	NA	1
240	0.516666667	NA	NA	1
450	0.484444444	NA	NA	1
732	0.438518519	NA	NA	1
375	0.413793103	NA	NA	1
1177	0.499099099	NA	NA	1
1135	0.550373134	NA	NA	1
594	0.398130841	NA	NA	1
491	0.418604651	NA	NA	1
1099	0.538461538	NA	NA	1
828	0.414251208	NA	NA	1
306	0.490196078	NA	NA	1
380	0.523684211	NA	NA	1
561	0.496993988	NA	NA	1
569	0.437609842	NA	NA	1
380	0.471971067	NA	NA	0
593	0.427402863	NA	NA	1
594	0.500934579	NA	NA	1
495	0.444444444	NA	NA	1
1123	0.511949686	NA	NA	1
526	0.452471483	NA	NA	1
1262	0.465502909	NA	NA	1
2295	0.541313086	NA	NA	1
2144	0.46111869	NA	NA	1
345	0.512280702	NA	NA	1
453	0.441501104	NA	NA	1
315	0.523809524	NA	NA	1
487	0.593429158	NA	NA	1
463	0.417721519	NA	NA	1
706	0.412087912	NA	NA	1

975	0.441290323	NA	NA	1
811	0.480910918	NA	NA	1
660	0.446102819	NA	NA	1
2367	0.421424367	NA	NA	1
832	0.477272727	NA	NA	1
888	0.39657632	NA	NA	1
408	0.427362482	NA	NA	0
8789	0.550724638	NA	NA	1
538	0.456066946	NA	NA	1
683	0.401785714	NA	NA	1
1046	0.407922912	NA	NA	1
421	0.521246459	NA	NA	1
534	0.577752554	NA	NA	0
902	0.548286604	NA	NA	1
263	0.433460076	NA	NA	1
11545	0.516574586	NA	NA	0
3093	0.377089479	NA	NA	1
647	0.435950413	NA	NA	1
371	0.437106918	NA	NA	1
666	0.458633094	NA	NA	1
1080	0.477690289	NA	NA	1
734	0.382022472	NA	NA	1
1336	0.430913349	NA	NA	1
5754	0.471471471	NA	NA	1
899	0.421855146	NA	NA	1
738	0.553398058	NA	NA	1
314	0.441860465	NA	NA	1
706	0.480282519	NA	NA	0
500	0.385681293	NA	NA	1
267	0.546816479	NA	NA	1
401	0.48816568	NA	NA	1
408	0.47826087	NA	NA	1
658	0.503039514	NA	NA	1
556	0.401360544	NA	NA	1
446	0.486547085	NA	NA	1
949	0.384982122	NA	NA	1
1667	0.560573477	NA	NA	1
495	0.418823529	NA	NA	1
1875	0.608893957	NA	NA	1
276	0.547101449	NA	NA	1
927	0.459302326	NA	NA	1
447	0.430851064	NA	NA	1
391	0.523364486	NA	NA	1
525	0.530701754	NA	NA	1
519	0.489177489	NA	NA	1
596	0.448076923	NA	NA	1
1255	0.354101765	NA	NA	1
567	0.457925636	NA	NA	1
951	0.412197687	NA	NA	1
501	0.438073394	NA	NA	1
574	0.44964314	NA	NA	0
1266	0.459227468	NA	NA	1
459	0.5	NA	NA	1
846	0.380614657	NA	NA	1
507	0.441704036	NA	NA	1
911	0.450054885	NA	NA	1
867	0.435986159	NA	NA	1

472	0.473557692	NA	NA		1
872	0.438746439	NA	NA		1
609	0.474452555	NA	NA		1
978	0.408902692	NA	NA		1
833	0.410742496	NA	NA		0
376	0.446254072	NA	NA		1
825	0.402424242	NA	NA		1
545	0.39047619	NA	NA		1
5010	0.516233766	NA	NA		1
362	0.466666667	NA	NA		1
11721	0.497668796	NA	NA		1
838	0.367688022	NA	NA		1
785	0.477707006	NA	NA		1
563	0.4790174	NA	NA		1
479	0.482269504	NA	NA		1
2367	0.421424367	NA	NA		1
6807	0.50156041	NA	NA		1
1657	0.498687664	NA	NA		1
3780	0.505038168	NA	NA		1
1102	0.480705623	NA	NA		1
1279	0.507666099	NA	NA		1
12161	0.517980502	NA	NA		1
413	0.482200647	NA	NA		1
5376	0.539622642	NA	NA		1
7506	0.545525903	NA	NA		1
827	0.389896373	NA	NA		1
1079	0.550813008	NA	NA		1
6961	0.474658085	NA	NA		1
253	0.509881423	NA	NA		1
693	0.634920635	NA	NA		1
402	0.539800995	NA	NA		1
505	0.437623762	NA	NA		1
620	0.520967742	NA	NA		1
605	0.476033058	NA	NA		1
471	0.394230769	NA	NA		1
1738	0.570771001	NA	NA		1
2556	0.546557121	NA	NA		1
249	0.393574297	NA	NA		1
778	0.511627907	NA	NA		1
441	0.420485175	NA	NA		1
333	0.45045045	NA	NA		1
28993	0.547473201	NA	NA		1
38921	0.504115417	406		1000	0
7293	0.479964115	NA	NA		1
2239	0.410067873	NA	NA		1
119993	0.515265923	NA	NA		0
59603	0.537292465	758		541	1
4859	0.477267202	NA	NA		1
7259	0.439219039	373		784	0
13046	0.570301624	99		767	0
27748	0.418474892	NA	NA		1
137878	0.474095103	NA	NA		1
92407	0.489407211	497		1000	0
30230	0.449624866	NA	NA		1
22458	0.51956849	6		713	1
64664	0.530427214	NA	NA		1
165980	0.395519993	NA	NA		0

10666	0.525914022	396	930	0
49741	0.548062329	463	853	0
59151	0.549911772	259	358	0
42178	0.549474848	NA	NA	1
5781	0.526251151	NA	NA	0
74233	0.454545455	NA	NA	1
6492	0.524444444	NA	NA	1
52491	0.464579525	NA	NA	1
40960	0.518582841	NA	NA	1
27705	0.543705868	NA	NA	1
78800	0.476984078	360	1000	0
10424	0.531200721	196	1000	1
12711	0.526251277	NA	NA	1
57010	0.495251662	1000	794	0
43646	0.555671472	132	869	0
29964	0.553038991	186	857	1
31916	0.526744598	408	574	0
5511	0.547688328	NA	NA	1
47591	0.547325859	NA	NA	1
40425	0.514860845	1000	1000	0
55475	0.491254003	741	1000	1
30930	0.507383966	NA	NA	1
3273	0.54153074	NA	NA	1
90009	0.501703848	NA	NA	0
9383	0.49092022	709	1000	1
8412	0.444837341	NA	NA	0
39227	0.51537455	NA	NA	0
24509	0.510438413	357	287	1
14408	0.48444771	269	1000	0
40273	0.494217732	NA	NA	0
9219	0.549763928	NA	NA	0
24560	0.500937734	NA	NA	1
34579	0.551355425	233	800	0
6654	0.534675615	651	734	1
29333	0.576904015	NA	NA	1
15200	0.553114383	NA	NA	1
16657	0.52721519	NA	NA	0
44499	0.371134021	652	1000	0
3862	0.50931904	NA	NA	0
28635	0.485091049	NA	NA	0
2939	0.472472924	NA	NA	1
12335	0.508948412	NA	NA	0
9806	0.54823159	NA	NA	0
2610	0.527336861	NA	NA	1
27451	0.499831933	NA	NA	1
7064	0.499532127	NA	NA	0
95354	0.549722724	NA	NA	0
6969	0.463428068	NA	NA	0
48403	0.512328319	557	915	0
80220	0.552419225	NA	NA	0
9475	0.59345968	NA	NA	1
12803	0.546908316	1000	1000	1
91834	0.451264781	32	1000	0
12196	0.578112778	NA	NA	0
748	0.522772277	NA	NA	0
1055	0.578431373	NA	NA	1
1653	0.523290986	NA	NA	1

1137	0.453033268	NA	NA	1
1009	0.492121212	NA	NA	1
809	0.506493506	NA	NA	1
884	0.482666667	NA	NA	1
1034	0.470440252	NA	NA	1
2145	0.379487179	NA	NA	1
723	0.474412172	NA	NA	1
898	0.46220427	NA	NA	1
959	0.588888889	NA	NA	1
935	0.409035409	NA	NA	1
1007	0.388127854	NA	NA	1
1578	0.468801314	NA	NA	1
2956	0.495774648	NA	NA	1
2956	0.495774648	NA	NA	1
1043	0.430363864	NA	NA	1
1019	0.444444444	NA	NA	1
1078	0.425675676	NA	NA	1
582	0.5	NA	NA	1
186	0.596774194	NA	NA	1
1589	0.487406399	NA	NA	1
1157	0.479227053	NA	NA	1
28211	0.561070954	NA	NA	1
1306	0.549004594	NA	NA	1
3304	0.427529626	NA	NA	0
1281	0.435248296	NA	NA	1
872	0.412765957	NA	NA	1
670	0.394693201	NA	NA	1
1731	0.477415307	NA	NA	1
627	0.523125997	NA	NA	1
1511	0.424197162	NA	NA	1
774	0.53875969	NA	NA	1
1117	0.506137866	NA	NA	1
934	0.484293194	NA	NA	1
1437	0.478168264	NA	NA	1
1108	0.444338725	NA	NA	1
714	0.449541284	NA	NA	1
1081	0.436632747	NA	NA	1
4120	0.382619974	579	573	1
885	0.482485876	NA	NA	1
318	0.53030303	NA	NA	1
435	0.533333333	NA	NA	1
420	0.321428571	NA	NA	1
1057	0.460737938	NA	NA	1
2475	0.357979798	1000	1000	1
635	0.333913043	NA	NA	1
348	0.522988506	NA	NA	1
603	0.394693201	NA	NA	1
1072	0.362873134	NA	NA	1
1026	0.384990253	NA	NA	1
965	0.434734513	NA	NA	1
546	0.401656315	NA	NA	1
1312	0.40625	NA	NA	1
299	0.341137124	NA	NA	1
7103	0.383928571	NA	NA	1
142197	0.357852883	NA	NA	1
810	0.362433862	NA	NA	1
76287	0.365776369	NA	NA	0

244	0.364754098	NA	NA	1
238	0.453781513	NA	NA	1
327	0.370030581	NA	NA	1
1544	0.413860104	NA	NA	1
1166	0.343862816	NA	NA	1
634	0.402020202	NA	NA	1
581	0.389130435	NA	NA	1
179860	0.342151675	761	829	1
479	0.565762004	NA	NA	1
1516	0.486807388	NA	NA	1
1736	0.566037736	NA	NA	1
789	0.550063371	NA	NA	1
1281	0.426592798	NA	NA	1
1259	0.437079731	NA	NA	1
1259	0.437079731	NA	NA	1
1438	0.416184971	NA	NA	1
1091	0.327222731	NA	NA	1
237	0.421940928	NA	NA	1
99	0.494949495	NA	NA	1
109	0.504587156	NA	NA	1
106	0.358490566	NA	NA	1
109	0.422018349	NA	NA	1
107	0.373831776	NA	NA	1
74	0.378378378	NA	NA	1
106	0.471698113	NA	NA	1
67	0.313432836	NA	NA	1
116	0.465517241	NA	NA	1
118	0.330508475	NA	NA	1
128	0.4765625	NA	NA	1
116	0.353448276	NA	NA	1
116	0.5	NA	NA	1
79	0.556962025	NA	NA	1
78	0.256410256	NA	NA	1
91	0.461538462	NA	NA	1
135	0.37037037	NA	NA	1
100	0.39	NA	NA	1
73	0.397260274	NA	NA	1
101	0.326732673	NA	NA	1
88	0.295454545	NA	NA	1
71	0.38028169	NA	NA	1
99	0.464646465	NA	NA	1
118	0.440677966	NA	NA	1
99	0.424242424	NA	NA	1
79	0.405063291	NA	NA	1
108	0.287037037	NA	NA	1
81	0.296296296	NA	NA	1
79	0.303797468	NA	NA	1
77	0.337662338	NA	NA	1
75	0.373333333	NA	NA	1
96	0.354166667	NA	NA	1
75	0.333333333	NA	NA	1
75	0.333333333	NA	NA	1
85	0.376470588	NA	NA	1
84	0.428571429	NA	NA	1
93	0.258064516	NA	NA	1
72	0.513888889	NA	NA	1
95	0.547368421	NA	NA	1

115	0.434782609	NA	NA		1
92	0.369565217	NA	NA		1
70	0.257142857	NA	NA		1
72	0.222222222	NA	NA		1
84	0.30952381	NA	NA		1
80	0.35	NA	NA		1
71	0.422535211	NA	NA		1
75	0.306666667	NA	NA		1
78	0.346153846	NA	NA		1
77	0.220779221	NA	NA		1
67	0.343283582	NA	NA		1
67	0.343283582	NA	NA		1
67	0.343283582	NA	NA		1
132	0.484848485	NA	NA		1
78	0.435897436	NA	NA		1
88	0.329545455	NA	NA		1
115	0.304347826	NA	NA		1
81	0.469135802	NA	NA		1
73	0.301369863	NA	NA		1
73	0.301369863	NA	NA		1
121	0.41322314	NA	NA		1
101	0.465346535	NA	NA		1
89	0.325842697	NA	NA		1
5409	0.486839954		580	1000	0
1772	0.526069127	NA	NA		1
1632	0.489855072	NA	NA		1
1548	0.490975936	NA	NA		1
1236	0.572006472	NA	NA		1
627	0.532695375	NA	NA		1
15097	0.495738404		853	943	1
2568	0.517429651		937	945	1
3308	0.506856024		234	927	1
11457	0.534323382		293	1000	0
2663	0.538414634	NA	NA		1
25338	0.502655175	NA	NA		0
5092	0.460291734	NA	NA		0
13913	0.459904661		551	1000	0
4554	0.357051773		946	781	1
2905	0.552179128		814	1000	1
8134	0.558603981		1000	946	0
2099	0.539738431	NA	NA		1
2976	0.535509326		879	933	1
769	0.495726496	NA	NA		1
461	0.462871287	NA	NA		1
1993	0.363171355	NA	NA		1
1256	0.47678703	NA	NA		1
3426	0.495489691		942	922	1
2245	0.505764506	NA	NA		0
70	0.314285714	NA	NA		1
2296	0.504702194	NA	NA		1
54620	0.525335668		322	1000	1
3470	0.473984772	NA	NA		0
2549	0.439176869	NA	NA		1
841	0.525510204	NA	NA		1
117138	0.550971716	NA	NA		0
1765	0.461599367	NA	NA		1
29322	0.468731209		542	1000	0

32254	0.372540797	537		1000	0
2983	0.506907545	840		1000	1
4386	0.524437781	1000		1000	0
1816	0.439440049	NA	NA		1
1574	0.43571082	NA	NA		0
11046	0.53342246	288		771	0
28479	0.501738341	NA	NA		0
831	0.488679245	NA	NA		1
2040	0.568607069	NA	NA		1
1164	0.486386139	NA	NA		1
10948	0.545206359	NA	NA		0
1041	0.501122334	NA	NA		0
2142	0.553960396	1000		911	1
2455	0.547046843	1000		1000	1
2730	0.484806417	909		887	1
53423	0.459634929	453		1000	0
1489	0.535291087	NA	NA		1
19884	0.434699019	807		1000	1
16208	0.448791266	372		875	0
3953	0.408550468	NA	NA		1
22030	0.545734227	119		746	0
1462	0.524981357	NA	NA		1
2473	0.502369668	NA	NA		1
849	0.436984688	NA	NA		1
3951	0.501155327	NA	NA		1
11348	0.432807269	552		1000	0
68798	0.494703343	369		1000	0
454	0.531088083	NA	NA		1
40089	0.491411951	NA	NA		0
75433	0.476932397	NA	NA		1
3778	0.47816092	NA	NA		1
4381	0.522025316	1000		942	1
14127	0.48333831	764		1000	0
165318	0.532491685	NA	NA		0
1799	0.499143347	NA	NA		1
15417	0.535355317	1000		514	0
1150	0.445772059	NA	NA		1
31606	0.354781582	NA	NA		1
1224	0.49375	NA	NA		1
123452	0.506403963	NA	NA		0
127422	0.351740572	630		351	0
20430	0.478849321	476		1000	0
6936	0.489142282	NA	NA		0
2092	0.52988792	NA	NA		1
10175	0.460552933	NA	NA		1
987	0.424799082	NA	NA		1
3813	0.449863678	543		945	1
15811	0.515252855	NA	NA		0
1086	0.533658537	NA	NA		1
11367	0.458838662	708		1000	1
5523	0.49209622	NA	NA		1
12142	0.604113111	NA	NA		1
14017	0.489954527	NA	NA		0
7979	0.549832027	262		847	0
2944	0.436653772	277		1000	1
1988	0.588374852	NA	NA		1
135008	0.345024395	97		138	0

120684	0.471889016	NA	NA		0
6922	0.351733423	NA	NA		0
2096	0.494534712	NA	NA		0
657	0.503424658	NA	NA		1
30268	0.41991659		370	917	0
2225	0.505083179	NA	NA		1
458	0.405472637	NA	NA		1
516	0.433106576	NA	NA		1
860	0.501162791	NA	NA		1
2447	0.391571554		946	938	1
495	0.395027624	NA	NA		1
310	0.456790123	NA	NA		1
1253	0.574593796	NA	NA		1
632	0.520908005	NA	NA		0
769	0.456896552	NA	NA		0
4352	0.525418377	NA	NA		0
1017	0.450890585	NA	NA		1
821	0.478684531	NA	NA		1
1053	0.576862124	NA	NA		1
1151	0.514146341	NA	NA		1
1002	0.445929526	NA	NA		1
1114	0.429292929	NA	NA		1
86435	0.402128548		181	1000	0
650	0.375215146	NA	NA		1
1893	0.496038035	NA	NA		1
1023	0.573802542	NA	NA		1
9845	0.53948026		185	1000	0
14317	0.515380975		612	944	0
1817	0.486102898	NA	NA		1
33318	0.525081198	NA	NA		1
36660	0.49769107	NA	NA		1
10265	0.526439483	NA	NA		1
640	0.34083045	NA	NA		1
38672	0.558029048		744	884	0
76248	0.518669878	NA	NA		0
11887	0.551203134		33	791	1
7724	0.492051082		305	948	1
42299	0.511738811		1000	1000	0
12255	0.585837887		213	814	0
26045	0.496384075		462	1000	0
15650	0.547407542		319	456	1
1659	0.439054726	NA	NA		0
4215	0.498221741	NA	NA		0
12671	0.503683241		777	804	1
30603	0.542795157		651	1000	1
8805	0.470375	NA	NA		0
13408	0.44760985		521	1000	0
786	0.507101086	NA	NA		0
53238	0.577663357	NA	NA		0
8726	0.566790582		471	1000	1
3379	0.53875969	NA	NA		1
1253	0.34251607	NA	NA		1
3625	0.542894394		465	936	1
8535	0.592629046		1000	976	1
4336	0.468397677		1000	938	0
3654	0.439031863		923	942	0
32253	0.554159593		8	1000	1

26384	0.514887658	448	1000	0
5051	0.503257834	395	1000	1
5994	0.572800279	579	936	1
2717	0.512728208	NA	NA	0
24523	0.491123297	NA	NA	0
49903	0.464048854	325	1000	0
105693	0.542808511	NA	NA	1
35660	0.587205533	NA	NA	0
14088	0.509150189	NA	NA	1
16578	0.464740088	567	1000	0
75004	0.464592927	NA	NA	0
91091	0.460690563	NA	NA	0
4709	0.463975329	NA	NA	0
6199	0.528627838	NA	NA	1
650	0.414772727	NA	NA	1
784	0.436674437	NA	NA	1
1734	0.516724337	NA	NA	1
1329	0.534236268	NA	NA	1
1725	0.508405797	NA	NA	1
2088	0.519415028	NA	NA	1
1880	0.517241379	NA	NA	1
1874	0.485714286	NA	NA	1
792	0.464646465	NA	NA	1
3302	0.543383585	683	1000	1
2332	0.433782267	NA	NA	1
1894	0.455882353	NA	NA	1
737	0.466863034	NA	NA	1
649	0.496563574	NA	NA	1
19079	0.528508772	NA	NA	1
366	0.557377049	NA	NA	1
3322	0.496092529	NA	NA	1
3316	0.496085186	NA	NA	1
932	0.488145049	NA	NA	1
3134	0.497417471	NA	NA	0
4341	0.479335924	NA	NA	1
776	0.524671053	NA	NA	0
3211	0.522453889	437	941	1
12980	0.553921142	NA	NA	1
39291	0.510358334	603	1000	0
44756	0.523540951	NA	NA	1
64731	0.485144719	NA	NA	1
30085	0.398833379	180	912	0
8240	0.511500072	433	876	0
9559	0.376027773	1000	595	1
17427	0.490513328	NA	NA	0
6227	0.485291005	723	936	0
34696	0.492954619	514	1000	0
24068	0.494227148	NA	NA	0
16526	0.544730963	NA	NA	0
4567	0.584037876	NA	NA	1
22892	0.524952919	537	1000	1
113641	0.519213363	NA	NA	0
10280	0.566767157	148	821	0
17823	0.491874323	522	897	1
22131	0.528731478	440	1000	0
10485	0.486541599	653	933	1
42505	0.525436913	NA	NA	0

12382	0.560851583		95	1000	0
32045	0.515937621	NA	NA		0
50283	0.454522162	NA	NA		0
34554	0.540876164	NA	NA		0
10528	0.489051095		920	627	1
3001	0.49761838	NA	NA		0
9816	0.487820616		226	1000	1
186373	0.550277764	NA	NA		0
70093	0.499325691		337	1000	1
59113	0.500098328	NA	NA		1
19944	0.445016412	NA	NA		0
68560	0.554015953		371	1000	1
33147	0.48675878	NA	NA		1
37530	0.556800734		58	1000	0
41278	0.414916667		346	731	0
1232	0.403740374	NA	NA		1
1526	0.503934192	NA	NA		1
1741	0.539345204	NA	NA		1
17583	0.466564928	NA	NA		1
31643	0.499277587		1000	1000	0
21425	0.463483613		422	830	0
2148	0.492101484	NA	NA		1
3999	0.523240235	NA	NA		0
24740	0.471714922		861	392	1
19355	0.517310109		787	928	1
76789	0.341661825	NA	NA		1
11854	0.471632092	NA	NA		1
11854	0.451040148	NA	NA		0
813	0.413694722	NA	NA		1
1322	0.38503937	NA	NA		1
1405	0.394074074	NA	NA		1
2180	0.497289973	NA	NA		1
1017	0.504782147	NA	NA		1
1096	0.521916412	NA	NA		1
1676	0.358595194	NA	NA		1
1676	0.358595194	NA	NA		1
640	0.378125	NA	NA		1
848	0.435141509	NA	NA		1
3766	0.537254902		806	574	1
554	0.520325203	NA	NA		1
33540	0.448627951	NA	NA		1
1397	0.5547602	NA	NA		1
1968	0.521849593	NA	NA		1
729	0.431952663	NA	NA		1
817	0.488679245	NA	NA		1
895	0.512544803	NA	NA		1
3070	0.514093137	NA	NA		1
1215	0.476724138	NA	NA		1
3516	0.458309038	NA	NA		0
12898	0.552497717		509	964	1
2515	0.493322027	NA	NA		0
2515	0.493322027	NA	NA		0
2515	0.489157631	NA	NA		1
953	0.506145251	NA	NA		1
1177	0.571792693	NA	NA		1
1540	0.418181818	NA	NA		1
1540	0.418181818	NA	NA		1

1540	0.418181818	NA	NA		1
1540	0.418181818	NA	NA		1
23115	0.537943925	NA	NA		0
116980	0.524888594	NA	NA		0
13307	0.508253625	NA	NA		0
795	0.366037736	NA	NA		1
568	0.38028169	NA	NA		1
1482	0.338003503	NA	NA		1
1966	0.363102233	NA	NA		0
1079	0.5	NA	NA		1
3238	0.458254865	NA	NA		1
14566	0.489195077		470	1000	0
7988	0.480774475		531	1000	0
706	0.43625192	NA	NA		1
50400	0.51647001	NA	NA		0
787	0.457627119	NA	NA		1
16705	0.547091413		796	1000	0
1450	0.512413793	NA	NA		1
732	0.508943089	NA	NA		1
9058	0.518609484		1000	1000	1
64616	0.485546522		100	860	1
47293	0.49769789	NA	NA		0
7491	0.508114488		66	1000	1
697	0.396258503	NA	NA		1
557	0.394514768	NA	NA		1
1051	0.392007612	NA	NA		1
654	0.481651376	NA	NA		1
1459	0.403701165	NA	NA		1
1443	0.402633403	NA	NA		1
1327	0.397889977	NA	NA		1
1484	0.401617251	NA	NA		1
1190	0.43697479	NA	NA		1
1416	0.403248588	NA	NA		1
1450	0.412413793	NA	NA		1
1848	0.396645022	NA	NA		1
728	0.339285714	NA	NA		1
997	0.372116349	NA	NA		1
977	0.36642784	NA	NA		1
1265	0.354150198	NA	NA		1
1361	0.407788391	NA	NA		1
821	0.483556638	NA	NA		1
692	0.338150289	NA	NA		1
1339	0.413741598	NA	NA		1
817	0.345165239	NA	NA		1
1688	0.40936019	NA	NA		1
1664	0.406850962	NA	NA		1
652	0.40797546	NA	NA		1
500	0.346	NA	NA		1
852	0.483568075	NA	NA		1
1682	0.411414982	NA	NA		1
1383	0.402024584	NA	NA		1
1212	0.423267327	NA	NA		1
755	0.463576159	NA	NA		1
1356	0.402654867	NA	NA		1
1271	0.406766326	NA	NA		1
608	0.407894737	NA	NA		1
910	0.474725275	NA	NA		1

857	0.343057176	NA	NA		1
1023	0.464320626	NA	NA		1
1105	0.374660633	NA	NA		1
754	0.470822281	NA	NA		1
881	0.464245176	NA	NA		1
635	0.480314961	NA	NA		1
1023	0.380254154	NA	NA		1
1042	0.450095969	NA	NA		1
9702	0.507848569		854	720	1
2635	0.476938175		680	1000	0
4142	0.443523316	NA	NA		1
1965	0.413568773	NA	NA		1
8103	0.445229682	NA	NA		1
725	0.41641791	NA	NA		1
1217	0.59627592	NA	NA		1
16453	0.505885974		175	1000	1
2015	0.371244635	NA	NA		1
2903	0.327259475	NA	NA		0
1020	0.351543943	NA	NA		1
1439	0.348790323	NA	NA		1
1781	0.497596833	NA	NA		0
2921	0.465397924	NA	NA		1
71673	0.478729778	NA	NA		1
30793	0.483372756	NA	NA		1
11619	0.470460443	NA	NA		1
12493	0.472789116		1000	1000	1
352	0.376760563	NA	NA		1
599	0.309124767	NA	NA		1
575	0.383141762	NA	NA		1
515	0.399122807	NA	NA		1
444	0.421875	NA	NA		1
363	0.37	NA	NA		1
464	0.336658354	NA	NA		1
419	0.349862259	NA	NA		1
393	0.354651163	NA	NA		1
632	0.392294221	NA	NA		1
425	0.385113269	NA	NA		1
332	0.380434783	NA	NA		1
383	0.427272727	NA	NA		1
418	0.381615599	NA	NA		1
337	0.350148368	NA	NA		1
421	0.363420428	NA	NA		1
480	0.347826087	NA	NA		1
601	0.36802974	NA	NA		1
555	0.410810811	NA	NA		1
451	0.345864662	NA	NA		1
554	0.371841155	NA	NA		1
205	0.375609756	NA	NA		1
181	0.46961326	NA	NA		1
357	0.37	NA	NA		1
147	0.408163265	NA	NA		1
735	0.369343066	NA	NA		1
396	0.426767677	NA	NA		1
3386	0.529876977	NA	NA		1
3340	0.444729302		611	1000	0
2359	0.438006952	NA	NA		1
1083	0.452694611	NA	NA		0

88098	0.481067354	NA	NA	0
2209	0.449625468	NA	NA	1
7315	0.565602472	602	720	1
876	0.410958904	NA	NA	1
1058	0.469	NA	NA	1
367	0.487179487	NA	NA	1
965	0.436651584	NA	NA	0
1196	0.532797858	NA	NA	1
22812	0.485800901	NA	NA	0
395	0.533333333	NA	NA	1
2708	0.486403284	NA	NA	1
658	0.394052045	NA	NA	1
663	0.360559235	NA	NA	0
4513	0.511268513	NA	NA	1
1280	0.5390625	NA	NA	1
5747	0.465771952	877	937	1
2146	0.457783641	NA	NA	0
782	0.384266263	NA	NA	1
3631	0.520310766	343	877	0
1175	0.492409867	NA	NA	1
924	0.505797101	NA	NA	1
877	0.502457002	NA	NA	1
1197	0.568086884	NA	NA	1
344	0.50872093	NA	NA	1
951	0.434516524	NA	NA	1
1728	0.457746479	NA	NA	1
3688	0.468659315	NA	NA	1
3688	0.468659315	NA	NA	1
96514	0.522921693	NA	NA	1
10031	0.499575294	NA	NA	1
1019	0.468650372	NA	NA	0
34810	0.510365071	NA	NA	1
3483	0.540511343	NA	NA	1
1900	0.546015713	NA	NA	1
2148	0.492101484	NA	NA	1
10221	0.504901961	NA	NA	1
4371	0.48900305	NA	NA	0
1979	0.484387097	NA	NA	1
1979	0.484387097	NA	NA	1
1104	0.491166078	NA	NA	1
1154	0.493109646	NA	NA	0
1364	0.546522132	NA	NA	1
511	0.483588621	NA	NA	1
3989	0.494026477	485	930	1
10764	0.447915243	NA	NA	0
807	0.484804631	NA	NA	1
601	0.422628952	NA	NA	1
36209	0.477110583	NA	NA	1
1723	0.413415469	NA	NA	1
1714	0.442428035	NA	NA	1
63523	0.559800071	NA	NA	1
1229	0.550854353	NA	NA	1
3685	0.42039801	NA	NA	1
781	0.406196213	NA	NA	1
637	0.373280943	NA	NA	1
4074	0.51046798	386	544	0
2161	0.540345821	NA	NA	1

1407	0.52545156	NA	NA		1
29453	0.518402994	NA	NA		0
2384	0.55342196		918	785	1
17551	0.428122898	NA	NA		1
657	0.477929985	NA	NA		1
860	0.50067659	NA	NA		1
2173	0.500243072		1000	910	1
1101	0.504995459	NA	NA		1
2184	0.466443972	NA	NA		0
14096	0.537161548	NA	NA		1
5716	0.546998342	NA	NA		0
5960	0.451258659	NA	NA		0
1279	0.505902192	NA	NA		1
1646	0.55620155	NA	NA		1
1765	0.449547017	NA	NA		0
1846	0.515205725	NA	NA		1
990	0.48540146	NA	NA		1
1175	0.483404255	NA	NA		1
1835	0.547138965	NA	NA		1
2829	0.575020276	NA	NA		1
23114	0.460303413	NA	NA		1
31953	0.555931893	NA	NA		0
9079	0.405641637		587	1000	1
13531	0.491696323		493	905	0
902	0.369436202	NA	NA		1
987	0.346002621	NA	NA		1
437	0.457446809	NA	NA		1
330	0.5	NA	NA		1
841	0.539833532	NA	NA		1
4838	0.489242054		647	870	1
3257	0.526104418	NA	NA		1
36209	0.43235666	NA	NA		0
16729	0.554420081	NA	NA		1
6465	0.476691729	NA	NA		0
54586	0.52810654	NA	NA		0
363	0.61707989	NA	NA		1
330	0.466666667	NA	NA		1
810	0.418972332	NA	NA		1
2620	0.470970361	NA	NA		1
275	0.514767932	NA	NA		1
3703	0.388678717	NA	NA		0
659	0.411422528	NA	NA		1
8454	0.547446865		574	939	0
28396	0.487714303	NA	NA		0
6185	0.444766147		622	781	0
1399	0.49376947	NA	NA		1
22909	0.509771987		315	938	0
2975	0.567375887	NA	NA		1
1422	0.468	NA	NA		1
747	0.54398827	NA	NA		1
1332	0.453453453	NA	NA		1
1276	0.490654206	NA	NA		1
1276	0.490654206	NA	NA		1
1260	0.393735363	NA	NA		1
1265	0.4	NA	NA		0
831	0.419630156	NA	NA		1
568	0.426048565	NA	NA		1

4147	0.442011632	NA	NA	1
2874	0.538752363	NA	NA	1
907	0.485714286	NA	NA	1
1308	0.479279279	NA	NA	1
1631	0.505300353	NA	NA	1
1631	0.502403846	NA	NA	1
2148	0.506704981	NA	NA	1
2255	0.401112797	NA	NA	1
1001	0.424010217	NA	NA	1
7931	0.522222222	NA	NA	0
29554	0.500847099	NA	NA	1
25022	0.517705833	NA	NA	0
11303	0.452728255	NA	NA	1
148632	0.357601713	299	442	0
6778	0.490307868	481	1000	1
2444	0.497968397	886	940	1
2129	0.488571429	NA	NA	1
897	0.493961353	NA	NA	1
738	0.50761035	NA	NA	1
1430	0.448023426	NA	NA	1
279	0.47311828	NA	NA	1
279	0.47311828	NA	NA	1
300	0.453333333	NA	NA	1
300	0.453333333	NA	NA	1
313	0.434108527	NA	NA	1
714	0.492997199	NA	NA	1
318	0.459119497	NA	NA	1
4150	0.455580357	297	943	0
6569	0.531746032	NA	NA	0
29515	0.48378517	NA	NA	0
6847	0.53051248	932	825	0
10604	0.527293191	274	1000	1
60552	0.478723664	135	1000	0
179215	0.43803838	NA	NA	0
2084	0.453869698	NA	NA	0
2527	0.501893517	NA	NA	1
1225	0.506938776	NA	NA	1
670	0.431513903	NA	NA	1
314	0.519823789	NA	NA	1
24277	0.551267606	NA	NA	1
21274	0.479575624	NA	NA	0
10362	0.438778501	592	873	1
855	0.356725146	NA	NA	1
480	0.4375	NA	NA	1
630	0.449206349	NA	NA	1
16658	0.512871533	NA	NA	1
64919	0.555541204	NA	NA	1
1180	0.518617021	NA	NA	1
68548	0.494882795	NA	NA	1
44482	0.361387588	52	580	1
124735	0.391146149	NA	NA	0
588	0.450381679	NA	NA	1
2083	0.383928571	NA	NA	1
140	0.392857143	NA	NA	1
26891	0.518459186	NA	NA	0
11780	0.485500801	NA	NA	1
14098	0.485760066	1000	1000	1

21952	0.519249782		918	1000	1
451	0.362694301	NA	NA		1
483	0.435835351	NA	NA		1
153	0.37254902	NA	NA		1
289	0.268398268	NA	NA		1
706	0.384375	NA	NA		1
945	0.383033419	NA	NA		1
388	0.449848024	NA	NA		1
131	0.335877863	NA	NA		1
1961	0.502176279	NA	NA		1
786	0.466828479	NA	NA		0
11381	0.455903849	NA	NA		0
3016	0.466843501		1000	1000	1
1907	0.511339093	NA	NA		1
2167	0.478843064	NA	NA		1
20538	0.534634372	NA	NA		0
2488	0.505063849	NA	NA		1
41559	0.495762176	NA	NA		1
44577	0.514654403	NA	NA		0
140286	0.514166373		244	929	0
26706	0.520797175	NA	NA		0
48893	0.485279373		369	1000	0
421	0.467933492	NA	NA		1
6767	0.472279116		941	1000	0
1302	0.426386233	NA	NA		1
103441	0.313377679	NA	NA		1
91129	0.372611465	NA	NA		1
702	0.487179487	NA	NA		1
6912	0.525354043		773	763	1
636	0.420962199	NA	NA		1
903	0.382803298	NA	NA		1
156	0.429487179	NA	NA		1
114230	0.366589327	NA	NA		1
117	0.41025641	NA	NA		1
91137	0.357142857	NA	NA		1
973	0.356628983	NA	NA		1
5224	0.549958587		166	1000	1
1732	0.459563543	NA	NA		1
40135	0.528811087	NA	NA		1
1641	0.531685678	NA	NA		1
1641	0.531685678	NA	NA		1
1303	0.486678508	NA	NA		1
1028	0.486381323	NA	NA		1
1028	0.486381323	NA	NA		1
42603	0.487284747	NA	NA		0
74037	0.487096259	NA	NA		0
3886	0.467980296		604	1000	1
2760	0.508532423		874	784	1
2400	0.577948486		1000	938	1
1852	0.524288107	NA	NA		1
2342	0.478098291	NA	NA		1
2121	0.46809681	NA	NA		1
1529	0.487822878	NA	NA		1
8562	0.509053424		453	1000	0
484	0.455399061	NA	NA		1
3030	0.465219318		680	1000	1
1245	0.546546547	NA	NA		1

629	0.440381558	NA	NA		1
6794	0.360110803	NA	NA		1
4106	0.461033951	243		824	1
1628	0.535508637	NA	NA		1
832	0.438253012	NA	NA		1
4110	0.538519543	NA	NA		0
19553	0.532970027	191		1000	0
74931	0.534930756	306		735	0
136	0.441176471	NA	NA		1
141	0.489361702	NA	NA		1
157	0.324840764	NA	NA		1
101	0.386138614	NA	NA		1
2571	0.50318218	1000		1000	1
29104	0.516438058	1000		380	0
4570	0.480414374	1000		1000	1
468	0.489316239	NA	NA		1
776	0.414561664	NA	NA		1
2310	0.562962963	NA	NA		1
299	0.558528428	NA	NA		1
634	0.4	NA	NA		1
590	0.364508393	NA	NA		1
742	0.368015414	NA	NA		1
311	0.423529412	NA	NA		1
53275	0.546264281	NA	NA		1
5843	0.42973913	614		1000	1
1107	0.394144144	NA	NA		1
909	0.437908497	NA	NA		1
2795	0.479249138	NA	NA		1
484	0.4	NA	NA		1
3300	0.389174239	NA	NA		1
3300	0.389174239	NA	NA		1
2969	0.431956558	NA	NA		0
46372	0.487629886	NA	NA		1
23985	0.545211343	124		866	0
79215	0.524990546	NA	NA		0
118741	0.521995668	NA	NA		0
14656	0.529994702	1000		982	0
54266	0.54949896	216		1000	0
15136	0.54265011	75		523	0
14739	0.526585307	NA	NA		0
48020	0.507478189	258		543	0
48091	0.511890661	NA	NA		0
4674	0.389191498	NA	NA		0
5002	0.562195969	NA	NA		0
3701	0.540385041	834		584	1
65236	0.513491542	NA	NA		0
6955	0.497912896	NA	NA		0
57188	0.47509608	NA	NA		0
247	0.508196721	NA	NA		1
383	0.378787879	NA	NA		1
974	0.391143911	NA	NA		1
73621	0.475622497	NA	NA		1
41541	0.546279492	975		819	0
5344	0.439498433	1000		1000	1
25580	0.581663327	NA	NA		1
8968	0.534583248	613		1000	0
476	0.453012048	NA	NA		1

9626	0.535897436	NA	NA		1
732	0.363001745	NA	NA		1
1019	0.344061303	NA	NA		1
694	0.321252059	NA	NA		1
471	0.485294118	NA	NA		1
1942	0.434864695	NA	NA		1
455	0.398963731	NA	NA		1
1086	0.484107579	NA	NA		1
68781	0.566185254	NA	NA		0
1045	0.543176734	NA	NA		0
893	0.52248062	NA	NA		1
1188	0.517980107	NA	NA		0
25002	0.507253159	NA	NA		1
18475	0.5336985		726	807	0
16351	0.533261803		259	938	0
29983	0.50083212		262	1000	0
470	0.393203883	NA	NA		1
629	0.407185629	NA	NA		1
405	0.404558405	NA	NA		1
21407	0.542260161		88	1000	0
18258	0.488688579		534	1000	0
813	0.436654367	NA	NA		1
9107	0.508643101		382	1000	0
5892	0.468367497	NA	NA		0
598	0.424749164	NA	NA		1
632	0.353658537	NA	NA		1
633	0.521097046	NA	NA		1
692	0.4064	NA	NA		1
566	0.361419069	NA	NA		1
559	0.451417004	NA	NA		1
362	0.442176871	NA	NA		1
380	0.425925926	NA	NA		1
443	0.43019943	NA	NA		1
1020	0.409957627	NA	NA		1
285	0.484444444	NA	NA		1
12679	0.454472523	NA	NA		1
849	0.362779741	NA	NA		1
810	0.362962963	NA	NA		1
6628	0.513121623	NA	NA		0
162466	0.434253258	NA	NA		0
60964	0.509477665		261	1000	1
28563	0.499278721	NA	NA		1
2344	0.497592998	NA	NA		1
2344	0.497592998	NA	NA		1
577	0.447140381	NA	NA		1
558	0.489247312	NA	NA		1
2788	0.539127163	NA	NA		1
2788	0.539127163	NA	NA		1
630	0.41744186	NA	NA		1
62951	0.493979325		973	829	0
93339	0.552327164	NA	NA		0
312	0.567307692	NA	NA		1
310	0.495934959	NA	NA		1
809	0.409785933	NA	NA		1
21310	0.481908682		172	1000	0
4205	0.435240779	NA	NA		1
31862	0.469411424	NA	NA		1

1155	0.459286368	NA	NA		1
2582	0.404255319	NA	NA		1
17608	0.468366846	436		1000	1
1168	0.4643289	NA	NA		1
10855	0.591169022	NA	NA		0
6298	0.391402715	263		731	1
5422	0.516973415	NA	NA		1
685	0.518248175	NA	NA		1
9332	0.504644472	333		939	0
85373	0.470052539	NA	NA		1
30488	0.482578657	NA	NA		0
10208	0.529899135	NA	NA		0
115350	0.49901088	NA	NA		1
496	0.436018957	NA	NA		1
489	0.400817996	NA	NA		1
663	0.389140271	NA	NA		1
20445	0.520020158	1000		1000	0
257	0.49009901	NA	NA		1
840	0.477366255	NA	NA		1
623	0.438202247	NA	NA		1
966	0.381987578	NA	NA		1
694	0.470681458	NA	NA		1
303	0.463709677	NA	NA		1
240	0.420833333	NA	NA		1
342	0.415204678	NA	NA		1
540	0.372427984	NA	NA		1
698	0.360374415	NA	NA		1
1328	0.427699817	NA	NA		0
777	0.428822496	NA	NA		1
741	0.414807302	NA	NA		1
764	0.415954416	NA	NA		1
104814	0.502012982	NA	NA		0
1947	0.456198347	NA	NA		1
1947	0.456198347	NA	NA		1
2654	0.476010782	NA	NA		1
2654	0.506406345	NA	NA		1
2651	0.494129658	NA	NA		1
2652	0.493877551	NA	NA		1
2648	0.486178862	NA	NA		1
951	0.443743428	NA	NA		1
254	0.55511811	NA	NA		1
760	0.331491713	NA	NA		1
186	0.403225806	NA	NA		1
890	0.418635171	NA	NA		1
120	0.45	NA	NA		1
181	0.348066298	NA	NA		1
18489	0.523022064	NA	NA		0
84751	0.456717072	NA	NA		0
1404	0.437299035	NA	NA		1
576	0.464705882	NA	NA		1
249	0.449799197	NA	NA		1
35968	0.496520763	NA	NA		1
600	0.404109589	NA	NA		1
1416	0.448459086	NA	NA		1
46990	0.481621349	NA	NA		1
5713	0.507692308	NA	NA		1
60893	0.539098903	1000		1000	0

822	0.509702458	NA	NA		1
1236	0.464269663	NA	NA		0
77225	0.528394306	NA	NA		0
8550	0.579026217	524		868	1
402	0.442528736	NA	NA		1
332	0.528089888	NA	NA		1
468	0.560493827	NA	NA		1
24512	0.506576627	346		1000	0
48817	0.555390113	302		1000	1
14138	0.531345333	941		1000	0
551	0.33	NA	NA		1
12988	0.527194268	226		1000	0
13216	0.53522071	101		1000	0
5970	0.52462908	464		1000	1
41151	0.508107439	918		1000	1
98	0.326530612	NA	NA		1
91	0.318681319	NA	NA		1
707	0.486562942	NA	NA		1
3940	0.478175254	NA	NA		1
27952	0.53474118	NA	NA		0
1017	0.426023085	NA	NA		1
625	0.4624	NA	NA		1
438	0.563926941	NA	NA		1
418	0.433014354	NA	NA		1
694	0.523364486	NA	NA		1
805	0.438494935	NA	NA		1
1083	0.554858934	NA	NA		1
714	0.514005602	NA	NA		1
306	0.581699346	NA	NA		1
5830	0.463333333	936		803	1
9956	0.448209941	758		1000	1
3379	0.352977287	1000		879	1
577	0.499133449	NA	NA		1
2518	0.464450989	933		1000	1
530	0.460240964	NA	NA		1
468	0.463126844	NA	NA		1
636	0.443378119	NA	NA		1
506	0.448616601	NA	NA		1
2334	0.476798144	NA	NA		1
1525	0.508438819	NA	NA		1
677	0.368260427	NA	NA		0
543	0.348195329	NA	NA		1
720	0.381944444	NA	NA		1
87970	0.493319559	NA	NA		0
1266	0.413112164	NA	NA		1
198	0.429292929	NA	NA		1
264	0.401515152	NA	NA		1
369	0.528455285	NA	NA		1
327	0.464831804	NA	NA		1
775	0.474097331	NA	NA		1
2331	0.524705882	NA	NA		1
225	0.542222222	NA	NA		1
3753	0.398291925	1000		1000	1
21842	0.551348062	NA	NA		0
527	0.459203036	NA	NA		1
387	0.56969697	NA	NA		1
40748	0.55190017	NA	NA		1

571	0.343253968	NA	NA	1
634	0.455673759	NA	NA	1
829	0.339354839	NA	NA	1
784	0.446428571	NA	NA	1
1980	0.422736733	NA	NA	1
342	0.511695906	NA	NA	1
221	0.415584416	NA	NA	1
1058	0.382617383	NA	NA	1
1025	0.417661098	NA	NA	1
1270	0.426455026	NA	NA	1
17674	0.506991848	NA	NA	0
678	0.396135266	NA	NA	1
610	0.541176471	NA	NA	1
978	0.415803109	NA	NA	1
1461	0.371825162	NA	NA	0
549	0.436647173	NA	NA	1
6859	0.458192363	NA	NA	1
13873	0.528674784	NA	NA	1
48023	0.537926204	NA	NA	0
1063	0.570436508	NA	NA	1
6870	0.516802264	250	1000	1
3565	0.556580296	941	446	0
26110	0.534249596	685	1000	0
412	0.464589235	NA	NA	1
583	0.403071017	NA	NA	1
481	0.356796117	NA	NA	1
337	0.434628975	NA	NA	1
432	0.366847826	NA	NA	1
2202	0.497729337	NA	NA	1
426	0.427745665	NA	NA	1
1315	0.473472669	NA	NA	1
981	0.361904762	NA	NA	1
1162	0.40307971	NA	NA	1
38250	0.519627656	899	1000	1
610	0.431147541	NA	NA	1
392	0.408823529	NA	NA	1
143182	0.399822409	364	1000	1
1019	0.403544649	NA	NA	0
1474	0.404825737	NA	NA	1
577	0.405545927	NA	NA	1
464	0.451935081	NA	NA	1
652	0.435430464	NA	NA	1
531	0.331450094	NA	NA	1
668	0.40765391	NA	NA	1
415	0.399339934	NA	NA	1
476	0.516129032	NA	NA	1
303	0.475247525	NA	NA	1
427	0.437994723	NA	NA	1
14290	0.42629108	NA	NA	1
387	0.455927052	NA	NA	1
525	0.445714286	NA	NA	1
538	0.430327869	NA	NA	1
1529	0.423152387	NA	NA	1
11218	0.414007282	NA	NA	1
53148	0.477045294	NA	NA	0
11864	0.470602153	269	1000	0
3424	0.424845389	886	994	1

746	0.449061662	NA	NA		1
399	0.418546366	NA	NA		1
542	0.391076115	NA	NA		1
270	0.435483871	NA	NA		1
249	0.514056225	NA	NA		1
25150	0.61131422	NA	NA		0
694	0.400728597	NA	NA		1
674	0.43047619	NA	NA		1
842	0.409024745	NA	NA		1
1220	0.402	NA	NA		1
20876	0.544754059	NA	NA		0
39835	0.40878213		324	382	1
560	0.429141717	NA	NA		1
3933	0.465071151		651	1000	1
3587	0.434960865		694	1000	0
12656	0.478833155	NA	NA		1
8150	0.536902899		425	1000	0
413	0.370748299	NA	NA		1
518	0.384223919	NA	NA		1
436	0.382608696	NA	NA		1
856	0.412797992	NA	NA		1
1834	0.432512315	NA	NA		1
1747	0.450958286	NA	NA		1
1298	0.381956155	NA	NA		1
552	0.408817635	NA	NA		1
594	0.374538745	NA	NA		1
587	0.430210325	NA	NA		1
785	0.403047091	NA	NA		1
533	0.390756303	NA	NA		1
376	0.350482315	NA	NA		1
615	0.368231047	NA	NA		1
394	0.383248731	NA	NA		1
1164	0.428694158	NA	NA		1
627	0.425837321	NA	NA		1
285	0.540350877	NA	NA		1
459	0.433551198	NA	NA		1
401	0.345454545	NA	NA		1
26140	0.514387109		392	1000	0
475	0.446315789	NA	NA		1
1040	0.437336815	NA	NA		1
854	0.408010013	NA	NA		1
881	0.442728443	NA	NA		1
385	0.449350649	NA	NA		1
731	0.385670732	NA	NA		1
320	0.394636015	NA	NA		1
2031	0.413479053	NA	NA		1
602	0.405063291	NA	NA		1
340	0.458823529	NA	NA		1
22235	0.473792138		537	1000	0
310	0.513833992	NA	NA		1
555	0.488288288	NA	NA		1
1118	0.545150502	NA	NA		1
228	0.495614035	NA	NA		1
44963	0.518157909	NA	NA		0
1384	0.352012628	NA	NA		1
466	0.412017167	NA	NA		1
49703	0.532119914		195	1000	0

1091	0.522774327	NA	NA	1
886	0.388526728	NA	NA	1
888	0.423423423	NA	NA	1
872	0.389169675	NA	NA	1
64460	0.51995759	NA	NA	0
3272	0.576832151	NA	NA	0
4046	0.51959318	NA	NA	1
329	0.417582418	NA	NA	1
501	0.427937916	NA	NA	1
347	0.461538462	NA	NA	1
594	0.436229205	NA	NA	1
473	0.487671233	NA	NA	1
281	0.426605505	NA	NA	1
627	0.462519936	NA	NA	1
538	0.366108787	NA	NA	1
392	0.443877551	NA	NA	1
660	0.38317757	NA	NA	1
104810	0.512736587	NA	NA	0
554	0.487364621	NA	NA	1
43177	0.544496026	NA	NA	0
591	0.352362205	NA	NA	0
284	0.436936937	NA	NA	1
525	0.467239527	NA	NA	1
357	0.393939394	NA	NA	1
1512	0.324032403	NA	NA	1
20163	0.483358636	803	992	0
936	0.451923077	NA	NA	1
550	0.422916667	NA	NA	1
394	0.439086294	NA	NA	1
791	0.336971351	NA	NA	1
1012	0.436213992	NA	NA	1
20563	0.521973155	1000	1000	1
16387	0.518181818	NA	NA	1
362	0.330909091	NA	NA	1
535	0.340632603	NA	NA	1
564	0.397565923	NA	NA	1
1241	0.465648855	NA	NA	1
1636	0.451505017	NA	NA	1
9328	0.547401728	360	1000	1
54282	0.510480074	NA	NA	0
34340	0.556726401	110	1000	1
6289	0.523895401	604	1000	0
476	0.377990431	NA	NA	1
681	0.408163265	NA	NA	1
7520	0.517253552	781	1000	0
34095	0.413926183	393	1000	0
3920	0.3945	1000	1000	1
533	0.397748593	NA	NA	1
1201	0.466990291	NA	NA	1
489	0.40625	NA	NA	1
532	0.458646617	NA	NA	1
627	0.349565217	NA	NA	1
629	0.367556468	NA	NA	1
1565	0.442159383	NA	NA	1
12083	0.457875458	NA	NA	1
573	0.41723356	NA	NA	1
237	0.561181435	NA	NA	1

255	0.411764706	NA	NA	1
627	0.349565217	NA	NA	1
768	0.418367347	NA	NA	1
519	0.368015414	NA	NA	1
251	0.406374502	NA	NA	1
718	0.421052632	NA	NA	1
246	0.402439024	NA	NA	1
526	0.372844828	NA	NA	1
648	0.374367622	NA	NA	1
595	0.46803653	NA	NA	1
2715	0.405031447	NA	NA	1
177	0.497175141	NA	NA	1
403	0.486956522	NA	NA	1
1245	0.403543307	NA	NA	1
738	0.405975395	NA	NA	1
919	0.471676301	NA	NA	1
682	0.442815249	NA	NA	1
677	0.375639713	NA	NA	1
639	0.454880295	NA	NA	1
643	0.359698682	NA	NA	1
964	0.405436014	NA	NA	1
629	0.367556468	NA	NA	1
631	0.347150259	NA	NA	1
436	0.379679144	NA	NA	1
834	0.42765685	NA	NA	1
702	0.443057722	NA	NA	1
551	0.391203704	NA	NA	1
525	0.498777506	NA	NA	1
431	0.420765027	NA	NA	1
476	0.402702703	NA	NA	1
682	0.438748914	NA	NA	0
1891	0.424242424	NA	NA	1
1891	0.422558923	NA	NA	1
1891	0.424242424	NA	NA	1
1891	0.423400673	NA	NA	1
1891	0.423400673	NA	NA	1
1891	0.424242424	NA	NA	1
717	0.451612903	NA	NA	1
739	0.447214076	NA	NA	1
2719	0.479699248	NA	NA	1
183	0.481481481	NA	NA	1
215	0.4	NA	NA	1
462	0.470149254	NA	NA	1
14615	0.485579916	NA	NA	0
53675	0.516197048	NA	NA	0
105556	0.476422018	NA	NA	0
51855	0.520164872	NA	NA	1
10322	0.480744426	NA	NA	0
18013	0.519506226	369	1000	0
378	0.484126984	NA	NA	1
338	0.540384615	NA	NA	0
939	0.479349186	NA	NA	1
522	0.382608696	NA	NA	1
309	0.373493976	NA	NA	1
547	0.383838384	NA	NA	1
243	0.543209877	NA	NA	1
792	0.336283186	NA	NA	1

218	0.371559633	NA	NA		1
536	0.369294606	NA	NA		1
488	0.516393443	NA	NA		1
766	0.326676177	NA	NA		1
102	0.450980392	NA	NA		1
1215	0.372682927	NA	NA		1
1720	0.485321101	NA	NA		1
791	0.438718663	NA	NA		1
592	0.459876543	NA	NA		1
1408	0.455788514	NA	NA		1
639	0.350340136	NA	NA		1
531	0.480519481	NA	NA		1
887	0.37150466	NA	NA		1
381	0.388888889	NA	NA		1
558	0.442815249	NA	NA		1
310	0.34	NA	NA		1
1241	0.472760849	NA	NA		1
4190	0.378019324	NA	NA		1
599	0.407216495	NA	NA		1
408	0.387254902	NA	NA		1
2056	0.358531317	NA	NA		0
1966	0.449712644	NA	NA		1
1101	0.443064182	NA	NA		1
3921	0.422222222	NA	NA		1
566	0.465483235	NA	NA		1
92118	0.414687296	NA	NA		0
381	0.378125	NA	NA		1
932	0.438701923	NA	NA		1
318	0.399371069	NA	NA		1
741	0.525096525	NA	NA		1
769	0.519886364	NA	NA		1
298	0.445783133	NA	NA		1
160	0.5125	NA	NA		1
4229	0.477247191		331	1000	1
516	0.459508644	NA	NA		1
497	0.416572077	NA	NA		1
673	0.373182553	NA	NA		1
580	0.49132948	NA	NA		1
832	0.397683398	NA	NA		1
511	0.336263736	NA	NA		1
364	0.486486486	NA	NA		1
3999	0.402550091	NA	NA		1
954	0.363398693	NA	NA		1
1142	0.383624655	NA	NA		1
3336	0.479051383	NA	NA		1
1069	0.39505446	NA	NA		0
8021	0.394655704		905	1000	1
556	0.51618705	NA	NA		1
41129	0.480628147	NA	NA		0
3717	0.424242424	NA	NA		0
600	0.436149312	NA	NA		1
365	0.390322581	NA	NA		1
380	0.354304636	NA	NA		1
704	0.364726027	NA	NA		1
8144	0.3625	NA	NA		1
650	0.396226415	NA	NA		1
654	0.364107884	NA	NA		0

619	0.407407407	NA	NA		1
577	0.44047619	NA	NA		1
531	0.447191011	NA	NA		1
1228	0.466272189	NA	NA		1
398	0.506329114	NA	NA		1
3419	0.462048875	NA	NA		0
460	0.465373961	NA	NA		1
1770	0.397565923	NA	NA		1
392	0.442225392	NA	NA		1
515	0.42920354	NA	NA		1
530	0.424307036	NA	NA		1
1197	0.434782609	NA	NA		1
352	0.378378378	NA	NA		1
539	0.427104723	NA	NA		1
966	0.436819172	NA	NA		1
456	0.465373961	NA	NA		1
599	0.534223706	NA	NA		1
905	0.44198895	NA	NA		1
16351	0.528401585	NA	NA		1
18895	0.417816451		838	1000	1
542	0.466740576	NA	NA		1
728	0.538194444	NA	NA		1
964	0.501738123	NA	NA		1
643	0.444029851	NA	NA		1
224	0.419642857	NA	NA		1
1197	0.538011696	NA	NA		1
432	0.384259259	NA	NA		1
1342	0.479559748	NA	NA		1
6231	0.521028547		480	828	1
460	0.465217391	NA	NA		1
1446	0.556517036	NA	NA		1
1446	0.544483986	NA	NA		0
1441	0.471894518	NA	NA		1
1441	0.471894518	NA	NA		1
732	0.430327869	NA	NA		1
513	0.547758285	NA	NA		1
837	0.54958184	NA	NA		1
2042	0.4361167	NA	NA		1
17174	0.50429334		476	944	1
15466	0.432432432	NA	NA		1
1022	0.490543735	NA	NA		1
1014	0.494047619	NA	NA		1
1623	0.470020675	NA	NA		1
4296	0.544849867		156	940	0
1639	0.47545582	NA	NA		1
28544	0.509666299	NA	NA		0
1041	0.493756004	NA	NA		1
692	0.476265823	NA	NA		1
4514	0.466395923	NA	NA		0
17184	0.340550293		247	938	1
2299	0.497104677	NA	NA		1
2299	0.492917847	NA	NA		0
17381	0.586246765		56	1000	1
298	0.463087248	NA	NA		1
376	0.494680851	NA	NA		1
319	0.390804598	NA	NA		1
219	0.447488584	NA	NA		1

838	0.437869822	NA	NA		1
597	0.494047619	NA	NA		1
221	0.327272727	NA	NA		1
2243	0.450809465	NA	NA		0
549	0.387550201	NA	NA		1
1455	0.424332344	NA	NA		1
297	0.352459016	NA	NA		1
670	0.523880597	NA	NA		1
548	0.532846715	NA	NA		1
493	0.53346856	NA	NA		1
210	0.335526316	NA	NA		1
457	0.541284404	NA	NA		1
267	0.376237624	NA	NA		1
9759	0.548566501		146	1000	0
37440	0.496941299		458	1000	1
30011	0.493479408	NA	NA		0
53139	0.444047372		411	1000	0
8761	0.361799977		318	1000	0
5032	0.547356052	NA	NA		1
429	0.440559441	NA	NA		1
14614	0.486884491		600	1000	1
2000	0.458239278	NA	NA		1
51381	0.55541035	NA	NA		1
32774	0.504898454	NA	NA		1
2619	0.47520483	NA	NA		1
24401	0.534645457	NA	NA		1
137	0.423357664	NA	NA		1
278	0.463963964	NA	NA		1
914	0.420131291	NA	NA		1
1100	0.375454545	NA	NA		1
1106	0.397830018	NA	NA		1
810	0.40617284	NA	NA		1
918	0.41503268	NA	NA		1
1091	0.378551787	NA	NA		1
1101	0.375113533	NA	NA		1
1101	0.375113533	NA	NA		1
1473	0.43980344	NA	NA		1
10705	0.463773314	NA	NA		0
423	0.432624113	NA	NA		1
25242	0.541852721	NA	NA		0
886	0.534988713	NA	NA		1
1031	0.403679654	NA	NA		1
208	0.408653846	NA	NA		1
191	0.408376963	NA	NA		1
301	0.465116279	NA	NA		1
1833	0.439698492	NA	NA		1
9837	0.477379601	NA	NA		1
54298	0.493774732		702	1000	0
515	0.367965368	NA	NA		1
1112	0.465402358	NA	NA		1
67702	0.538138419		252	1000	0
806	0.431081081	NA	NA		1
397	0.451327434	NA	NA		1
472	0.415254237	NA	NA		1
591	0.350253807	NA	NA		1
596	0.469798658	NA	NA		1
695	0.471666667	NA	NA		1

481	0.401891253	NA	NA		1
1291	0.416253717	NA	NA		1
368	0.510869565	NA	NA		1
644	0.422360248	NA	NA		1
446	0.468609865	NA	NA		1
491	0.450101833	NA	NA		1
364	0.505494505	NA	NA		1
202	0.435643564	NA	NA		1
800	0.407303371	NA	NA		1
657	0.505573248	NA	NA		1
625	0.44549763	NA	NA		1
745	0.500671141	NA	NA		1
1722	0.434224991	NA	NA		1
704	0.498579545	NA	NA		1
1283	0.456742011	NA	NA		1
923	0.403033586	NA	NA		1
663	0.460030166	NA	NA		1
679	0.497790869	NA	NA		1
1083	0.495253165	NA	NA		0
1623	0.407868852	NA	NA		1
1723	0.371359223	NA	NA		1
3658	0.532651715	NA	NA		0
52611	0.494530488	NA	NA		0
2173	0.500746392		876	1000	0
1665	0.464264264	NA	NA		1
2365	0.54932638	NA	NA		1
1567	0.513720485	NA	NA		1
3227	0.473488372	NA	NA		1
855	0.416608997	NA	NA		1
680	0.438311688	NA	NA		1
584	0.518835616	NA	NA		1
836	0.454545455	NA	NA		1
667	0.452773613	NA	NA		1
535	0.442990654	NA	NA		1
700	0.4578125	NA	NA		1
1114	0.429292929	NA	NA		1
937	0.417289221	NA	NA		1
570	0.398245614	NA	NA		1
17070	0.533319407	NA	NA		0
27918	0.480398934	NA	NA		0
92953	0.514205055		626	1000	0
670	0.404682274	NA	NA		1
1433	0.357012083	NA	NA		1
534	0.462546816	NA	NA		1
641	0.412098299	NA	NA		1
937	0.359751895	NA	NA		1
281	0.473309609	NA	NA		1
940	0.453191489	NA	NA		1
897	0.491638796	NA	NA		1
792	0.51010101	NA	NA		1
1332	0.502252252	NA	NA		1
603	0.427860697	NA	NA		1
751	0.435483871	NA	NA		1
437	0.385269122	NA	NA		1
721	0.430597015	NA	NA		1
6454	0.430445477		1000	1000	1
2943	0.396731055	NA	NA		1

3201	0.447488584	NA	NA		1
113266	0.520363236	NA	NA		1
2019	0.576032226	NA	NA		1
3878	0.458520638	NA	NA		1
1164	0.528350515	NA	NA		1
688	0.414355628	NA	NA		1
2813	0.467902996	NA	NA		0
2115	0.434888241	NA	NA		1
2332	0.500682128	NA	NA		1
1959	0.50368899	NA	NA		1
668	0.443719413	NA	NA		1
807	0.500671141	NA	NA		1
1808	0.433628319	NA	NA		1
1097	0.56426618	NA	NA		1
503	0.413518887	NA	NA		1
3086	0.483837866	NA	NA		1
926	0.492485549	NA	NA		1
1640	0.480519481	NA	NA		1
7816	0.503151889		308	1000	1
3286	0.404266811		267	1000	1
147	0.414965986	NA	NA		1
151	0.410596026	NA	NA		1
147	0.380952381	NA	NA		1
226	0.455752212	NA	NA		1
166	0.373493976	NA	NA		1
161	0.50310559	NA	NA		1
136	0.426470588	NA	NA		1
88	0.352272727	NA	NA		1
166	0.409638554	NA	NA		1
101	0.415841584	NA	NA		1
153	0.405228758	NA	NA		1
156	0.352564103	NA	NA		1
311	0.453376206	NA	NA		1
131	0.503816794	NA	NA		1
151	0.370860927	NA	NA		1
147	0.394557823	NA	NA		1
151	0.364238411	NA	NA		1
149	0.516778523	NA	NA		1
221	0.515837104	NA	NA		1
161	0.440993789	NA	NA		1
141	0.29787234	NA	NA		1
146	0.417808219	NA	NA		1
150	0.393333333	NA	NA		1
161	0.385093168	NA	NA		1
171	0.304093567	NA	NA		1
136	0.308823529	NA	NA		1
161	0.416149068	NA	NA		1
179	0.424581006	NA	NA		1
176	0.357954545	NA	NA		1
139	0.395683453	NA	NA		1
186	0.580645161	NA	NA		1
201	0.412935323	NA	NA		1
1207	0.426246719	NA	NA		0
352	0.355113636	NA	NA		1
467	0.312633833	NA	NA		1
624	0.436083409	NA	NA		1
478	0.481171548	NA	NA		1

400	0.4725	NA	NA	1
727	0.422746781	NA	NA	1
689	0.471599792	NA	NA	1
997	0.348290598	NA	NA	1
1031	0.418040737	NA	NA	1
629	0.434022258	NA	NA	1
1002	0.511205977	NA	NA	1
3019	0.374042146	NA	NA	1
498	0.481927711	NA	NA	1
383	0.506527415	NA	NA	1
882	0.402165507	NA	NA	1
3675	0.321090047	330	1000	1
567	0.455026455	NA	NA	1
891	0.42364532	NA	NA	1
1619	0.430015552	NA	NA	1
1438	0.471354167	NA	NA	0
641	0.461778471	NA	NA	1
2286	0.453363229	NA	NA	1
405	0.35483871	NA	NA	1
1655	0.403896104	NA	NA	0
1244	0.492080253	NA	NA	0
719	0.52173913	NA	NA	1
4686	0.517761033	NA	NA	1
814	0.485257985	NA	NA	1
5452	0.44124424	NA	NA	1
290	0.448888889	NA	NA	1
169	0.414201183	NA	NA	1
5798	0.551951579	684	936	1
895	0.405092593	NA	NA	1
1375	0.415929204	NA	NA	1
581	0.452667814	NA	NA	1
1827	0.405817175	NA	NA	1
513	0.483430799	NA	NA	1
2096	0.399332061	1000	1000	1
1844	0.466739967	NA	NA	1
1522	0.489810771	NA	NA	1
1358	0.510513036	NA	NA	1
251	0.458167331	NA	NA	1
743	0.504710633	NA	NA	1
704	0.448863636	NA	NA	1
356	0.421348315	NA	NA	1
339	0.351032448	NA	NA	1
744	0.425253991	NA	NA	1
2738	0.45625	NA	NA	1
1178	0.509433962	NA	NA	1
745	0.390604027	NA	NA	1
617	0.364726027	NA	NA	1
2120	0.462365591	NA	NA	1
500	0.464	NA	NA	1
897	0.425863991	NA	NA	1
1283	0.51208106	NA	NA	1
598	0.535117057	NA	NA	1
188	0.423423423	NA	NA	1
631	0.378378378	NA	NA	1
614	0.358276644	NA	NA	1
531	0.450094162	NA	NA	1
795	0.465408805	NA	NA	1

6434	0.402376308	1000	1000	0
976	0.454785828	NA	NA	1
1354	0.454209749	NA	NA	1
2503	0.44371345	NA	NA	1
3899	0.393728844	546	1000	0
593	0.511363636	NA	NA	1
1892	0.432941176	NA	NA	1
33110	0.56392668	NA	NA	0
104625	0.528552628	353	1000	0
336	0.416666667	NA	NA	1
1326	0.436464088	NA	NA	1
544	0.408536585	NA	NA	1
2627	0.413352273	NA	NA	1
2627	0.487283038	NA	NA	1
3611	0.482514571	NA	NA	1
5536	0.502585315	NA	NA	0
855	0.535672515	NA	NA	1
624	0.483974359	NA	NA	1
715	0.445454545	NA	NA	1
905	0.4	NA	NA	1
2258	0.42120075	874	1000	1
1565	0.437699681	NA	NA	1
5533	0.486414709	809	923	1
917	0.515775034	NA	NA	1
22729	0.374948967	NA	NA	0
1726	0.509213759	NA	NA	1
32275	0.299457995	NA	NA	1
555	0.430630631	NA	NA	1
317	0.375394322	NA	NA	1
484	0.412855377	NA	NA	0
354	0.355932203	NA	NA	1
1363	0.52833638	NA	NA	1
1706	0.501428571	NA	NA	1
1087	0.574977001	NA	NA	1
416	0.346153846	NA	NA	1
1361	0.418806959	NA	NA	1
308	0.535714286	NA	NA	1
452	0.431415929	NA	NA	1
276	0.496376812	NA	NA	1
16080	0.542679519	353	1000	1
5795	0.434449761	NA	NA	0
3546	0.454912129	1000	925	1
63523	0.559712695	NA	NA	0
15012	0.498511166	179	887	0
428	0.432242991	NA	NA	1
699	0.489270386	NA	NA	1
2426	0.481863149	1000	1000	1
2403	0.491468997	1000	1000	1
770	0.54494382	NA	NA	1
93	0.580645161	NA	NA	1
389	0.4368	NA	NA	0
307	0.396078431	NA	NA	1
529	0.387523629	NA	NA	1
660	0.442906574	NA	NA	1
587	0.418386492	NA	NA	1
11980	0.499160085	NA	NA	0
159	0.51572327	NA	NA	1

465	0.470967742	NA	NA	1
1023	0.46627566	NA	NA	1
1239	0.491525424	NA	NA	1
69	0.275362319	NA	NA	1
785	0.468789809	NA	NA	1
672	0.464285714	NA	NA	1
604	0.432119205	NA	NA	1
424	0.519886364	NA	NA	1
566	0.448763251	NA	NA	1
452	0.497787611	NA	NA	1
812	0.434650456	NA	NA	1
161403	0.572059218	NA	NA	0
286	0.520979021	NA	NA	1

DMEL_OVERI	DMEL_MATEF	DMEL_ZYG	DE_RENZIS	DE_RENZIS	EMBRYONIC_EXP		
0	0	1	0	0			1

0	1	0	0	0	1
0	1	0	0	0	1
1	1	0	0	0	1
0	0	1	0	0	1
0	1	0	0	0	1
0	0	1	0	0	1
0	0	0	0	0	0
0	0	1	0	1	1
1	0	1	0	0	1
0	1	0	0	0	1
0	1	0	0	0	1
0	1	0	0	0	1
0	0	1	0	0	1
0	1	0	0	0	1
0	1	0	0	0	1
1	1	0	0	0	1
0	0	1	0	0	1
1	0	1	0	1	1
1	1	0	0	1	1
0	1	0	0	0	1
0	1	0	0	0	1
0	0	1	0	0	1
0	0	1	0	0	1
0	0	1	0	0	0
1	1	0	1	0	1
0	0	1	0	0	1
0	0	1	0	0	0
1	0	0	0	0	0
1	0	1	0	0	0
0	0	0	0	0	0
1	1	0	0	0	1
0	0	1	0	0	0
0	1	0	0	0	1
0	1	0	0	0	1
0	0	1	0	1	1
1	0	1	0	0	1
1	0	0	0	0	1
0	0	1	0	0	1
1	1	0	0	0	1
0	1	0	0	0	1
1	0	0	0	0	1
1	1	0	0	0	1
0	0	1	0	0	1
1	1	0	1	0	1
0	0	0	0	0	0
0	1	0	0	0	1
1	0	1	0	0	1
0	0	1	0	0	0
0	1	0	0	0	1
1	0	0	0	0	0
0	0	1	0	0	0
1	0	1	0	0	1
0	0	0	0	0	0
0	0	1	0	0	1
0	1	0	0	0	0
0	0	1	0	0	1
0	0	1	0	0	1

0	0	1	0	0	1
0	1	0	0	0	0
1	1	0	0	0	1
0	1	0	0	0	0
1	1	0	0	0	1
0	0	1	0	0	1
0	0	1	0	0	1
0	0	1	1	0	1
1	1	0	0	0	1
0	0	1	0	0	1
0	1	0	0	0	1
1	1	0	0	0	1
1	0	1	0	0	1
0	0	0	0	0	1
1	0	1	0	0	1
0	1	0	0	0	1
0	0	1	0	0	1
1	1	0	0	0	1
0	0	1	0	1	1
1	0	1	0	0	1
0	0	1	0	0	0
0	1	0	0	0	1
1	0	1	0	0	1
1	1	0	0	0	1
0	1	0	0	0	1
0	0	1	0	0	1
0	1	0	0	0	1
0	0	1	0	0	0
0	1	0	0	0	1
1	1	0	0	0	1
1	0	1	0	0	0
0	0	1	0	0	1
0	1	0	0	0	1
0	1	0	0	0	1
1	0	1	0	0	1
1	1	0	0	0	1
0	1	0	0	0	1
0	1	0	0	0	1
1	1	0	0	0	1
0	1	0	0	0	1
0	1	0	0	0	1
0	1	0	0	0	1
0	0	0	0	0	1
1	1	0	0	0	1
1	1	0	0	0	1
0	0	1	0	0	1
0	0	1	0	0	0
0	0	1	0	0	1
1	1	0	0	0	1
1	1	0	0	0	1
0	1	0	0	0	1
1	0	1	0	1	1
1	1	0	1	0	1
0	1	0	0	0	1
1	1	0	0	0	1
0	0	1	1	0	1
1	0	1	0	1	1

0	1	0	0	0	1
0	1	0	0	0	1
0	0	1	0	0	1
0	1	0	0	0	1
0	1	0	0	0	1
0	1	0	0	0	1
1	0	1	0	0	1
0	1	0	0	0	1
1	1	0	0	0	1
1	0	0	0	0	0
0	1	0	0	0	1
0	1	0	0	0	1
0	0	1	0	0	1
1	0	1	0	0	1
0	1	0	0	0	1
0	1	0	0	0	1
0	0	1	0	0	1
1	0	1	0	0	1
1	0	1	0	0	1
0	1	0	0	0	1
0	0	0	0	0	0
0	1	0	0	0	0
0	1	0	0	1	1
0	0	1	0	0	0
0	0	0	0	0	1
0	1	0	0	0	1
0	1	0	0	0	1
0	0	1	0	0	0
1	0	1	0	0	1
0	0	0	0	0	1
0	0	1	0	0	1
0	0	1	0	0	1
0	1	0	0	0	1
0	1	0	0	0	1
0	0	0	0	0	0
0	0	0	0	0	1
0	0	0	0	0	1
0	0	0	0	0	1
0	0	1	0	0	1
0	1	0	0	0	1
0	1	0	0	0	1
1	1	0	0	0	1
0	0	1	0	0	1
0	1	0	0	0	1
1	0	0	0	0	1
1	0	1	0	0	1
0	1	0	0	0	1
1	0	1	0	0	0
1	1	0	0	0	1
0	1	0	0	0	1
0	1	0	0	0	1
1	1	0	0	0	1
0	0	0	0	1	1
0	0	1	0	0	0
0	1	0	1	0	1
0	1	0	0	0	1
1	0	0	0	0	0

0	0	1	0	0	0
0	0	1	0	0	1
0	1	0	0	0	1
0	0	1	0	0	0
0	1	0	0	0	1
0	0	1	0	0	1
1	0	0	0	0	1
0	1	0	0	0	1
0	0	0	0	0	0
1	0	1	0	0	1
1	1	0	0	0	1
0	0	0	0	0	1
0	1	0	0	0	1
1	0	1	0	0	1
0	1	0	0	0	1
1	0	1	0	0	1
1	0	1	0	0	1
0	0	1	0	0	1
1	1	0	0	0	1
0	1	0	0	0	1
1	1	0	0	0	1
1	1	0	0	0	1
0	1	0	0	0	1
0	0	1	0	0	1
0	0	1	0	0	1
1	1	0	0	0	1
1	1	0	0	0	1
0	0	1	0	0	1
0	0	0	0	0	1
1	0	1	0	0	1
0	0	1	0	0	0
0	0	1	0	0	0
0	0	1	0	0	0
0	1	0	0	0	1
0	0	0	0	0	0
1	0	1	0	0	0
0	0	0	0	0	1
1	1	0	0	0	1
0	0	0	0	0	1
0	1	0	1	0	1
0	1	0	0	0	1
0	0	0	0	0	0
0	1	0	0	0	0
0	0	0	0	0	0
0	0	0	0	0	1
0	0	1	0	0	0
0	1	0	0	0	1
0	1	0	0	0	1
0	1	0	0	0	1
0	1	0	0	0	1
0	1	0	0	0	1
0	1	0	0	0	1
0	1	0	0	0	1
0	0	1	0	0	1
0	0	1	0	0	1
0	0	1	0	0	0
0	1	0	0	0	1

0	1	0	0	0		1
0	1	0	0	0		1
1	0	1	0	0		1
1	0	0	0	0		0
0	1	0	0	0		0
1	0	0	0	0		1
1	0	1	0	0		1
0	1	0	0	0		1
0	1	0	0	0		1
0	0	0	0	0		0
0	1	0	0	0		0
1	0	0	0	0		0
0	1	0	0	0		1
1	1	0	0	0		1
0	0	1	0	0		1
0	0	1	0	0		1
0	1	0	0	0		1
0	0	1	0	0		1
0	0	0	0	0		1
0	1	0	0	0		1
0	0	1	0	0		0
1	1	0	0	0		1
0	0	1	0	0		0
0	1	0	0	0		1
1	1	0	0	0		1
0	1	0	0	0		1
0	0	1	0	0		1
0	0	0	0	0		0
0	0	1	0	0		1
1	1	0	0	0		1
0	1	0	1	0		1
0	1	0	0	0		1
1	1	0	0	0		1
0	0	1	0	0		1
1	1	0	0	0		1
0	0	0	0	0		0
1	1	0	0	0		1
0	1	0	0	0		1
0	1	0	0	0		1
0	1	0	0	0		1
0	1	0	0	0		1
1	1	0	0	0		1
0	0	1	0	0		1
1	0	1	0	0		0
0	1	0	0	0		1
0	1	0	0	0		1
0	0	1	0	0		0
0	0	1	0	0		0
0	0	1	0	0		1
0	1	0	0	0		1
0	1	0	0	0		1
0	0	0	0	0		1
1	0	1	0	0		1
1	0	1	0	0		1
0	0	1	0	1		1
0	1	0	0	0		1
0	1	0	0	0		1

1	0	1	0	0	0
0	0	1	0	0	1
0	1	0	0	0	1
0	0	1	0	0	1
1	1	0	0	0	1
1	0	0	0	0	1
1	1	0	0	0	1
1	1	0	0	0	0
0	1	0	0	0	1
1	0	1	0	0	0
0	0	0	0	0	1
0	0	1	0	0	1
0	1	0	0	0	0
1	1	0	0	0	1
1	1	0	0	0	1
0	0	1	0	0	1
1	1	0	0	0	1
0	1	0	0	0	1
0	1	0	1	0	1
0	0	1	0	0	1
0	1	0	1	0	1
1	0	1	0	0	0
0	0	0	0	0	1
0	0	1	0	0	1
0	0	0	0	0	1
0	0	0	0	0	0
0	1	0	0	0	1
0	1	0	1	0	1
0	1	0	0	0	1
1	1	0	0	0	1
0	0	0	0	0	0
0	1	0	1	0	1
0	0	1	0	0	0
0	0	0	0	0	1
0	0	1	0	0	0
0	1	0	0	0	1
0	0	0	0	0	1
0	1	0	0	0	1
1	1	0	0	0	1
0	0	0	0	0	0
0	0	1	0	0	1
0	0	1	1	0	1
1	0	1	0	0	1
0	0	0	0	0	1
0	0	1	1	0	1
0	0	1	1	0	1
1	1	0	0	0	1
1	1	0	0	0	1
1	0	1	0	0	0
0	1	0	1	0	1
0	1	0	1	0	1
0	1	0	0	0	1
0	0	1	0	0	1
1	0	0	0	0	0
1	0	0	0	0	1
0	1	0	1	0	1
0	0	1	0	0	1

0	0	0	0	0	0
1	0	0	0	0	1
0	1	0	0	0	1
0	0	1	0	0	0
1	0	1	0	0	0
0	0	0	0	0	1
1	0	1	0	0	1
1	0	0	0	0	1
0	0	1	0	0	1
0	0	1	0	0	1
0	0	1	0	0	1
0	0	0	0	0	0
0	0	1	0	0	1
0	1	0	0	1	1
0	1	0	0	0	1
0	1	0	0	0	1
0	1	0	0	0	1
0	1	0	0	0	1
0	0	0	0	0	0
1	1	0	0	0	1
0	0	1	0	0	0
0	0	1	0	0	0
0	0	1	0	0	1
0	0	1	0	0	0
0	1	0	0	0	1
0	1	0	0	0	1
0	0	1	0	0	0
0	1	0	0	0	1
0	1	0	0	0	1
0	0	1	0	0	1
0	1	0	0	0	1
0	0	1	0	0	1
0	0	1	0	0	1
0	0	1	0	0	1
1	1	0	0	0	1
1	1	0	0	0	1
1	1	0	0	0	1
0	0	1	0	0	0
0	0	1	0	0	0
0	1	0	0	0	1
0	1	0	0	0	1
0	1	0	0	0	1
0	0	1	0	0	0
0	0	0	0	0	0
0	0	1	0	0	1
0	0	0	0	0	1
0	0	1	0	0	1
0	1	0	0	0	1
0	0	0	0	0	0
0	0	1	0	0	1
0	0	0	0	0	0
0	0	1	0	0	0
1	1	0	0	0	1
0	0	1	1	0	1
1	1	0	0	0	1
1	0	0	0	0	0
1	1	0	0	0	1

0	0	1	0	0	0
0	0	1	0	0	1
0	1	0	0	0	1
1	1	0	0	0	1
1	0	0	0	0	1
0	0	1	0	0	1
0	0	1	0	0	1
0	1	0	0	0	0
0	0	0	0	1	1
0	0	0	0	0	0
0	1	0	0	0	1
1	1	0	0	0	1
0	0	1	0	0	1
1	0	0	0	0	0
0	1	0	0	0	1
0	0	1	0	0	1
0	0	0	0	0	0
0	1	0	0	0	1
0	1	0	0	0	1
0	0	0	0	0	0
1	1	0	0	0	1
0	1	0	0	0	0
0	1	0	0	0	1
1	0	1	0	0	0
1	0	0	0	0	1
1	0	0	0	0	1
0	0	1	0	0	1
0	1	0	0	0	1
0	0	0	0	0	1
0	1	0	0	0	1
1	0	1	0	0	0
0	0	1	0	0	1
0	1	0	0	0	1
1	1	0	0	0	1
0	0	1	0	0	1
0	1	0	0	0	1
0	1	0	0	0	1
0	0	1	0	0	0
0	0	1	0	0	1
1	1	0	1	0	1
1	1	0	0	0	1
0	0	1	0	0	1
0	0	1	0	1	1
0	0	1	0	0	1
0	0	1	0	0	0
1	1	0	0	0	1
0	1	0	0	0	0
1	0	1	0	0	1
1	0	0	0	0	0
0	1	0	0	0	1
0	1	0	0	0	1
0	0	0	0	0	0
0	0	0	0	0	0
0	0	0	0	0	0
0	1	0	0	0	1
0	0	0	0	0	0
0	0	0	0	0	1

1	1	0	0	0	1
0	0	0	0	0	0
1	0	1	0	0	1
1	0	1	0	0	1
0	1	0	0	0	1
1	1	0	0	0	1
1	0	0	0	0	1
0	1	0	0	0	1
0	0	1	1	0	1
0	1	0	0	0	1
0	0	1	0	0	0
0	0	1	0	0	1
0	1	0	0	0	1
0	0	1	0	0	1
0	0	1	0	0	0
0	0	0	0	0	0
0	1	0	1	0	1
0	0	1	0	0	1
0	1	0	0	0	1
0	1	0	0	0	1
1	0	0	0	0	0
0	0	1	0	0	0
0	1	0	0	0	1
0	1	0	0	0	1
1	1	0	0	0	1
0	1	0	0	0	1
1	1	0	0	0	1
0	0	0	0	0	1
0	0	0	0	0	1
1	0	1	0	0	0
0	0	0	0	0	1
0	0	0	0	0	0
0	1	0	0	0	1
0	1	0	0	0	1
0	1	0	0	0	1
0	1	0	0	0	1
0	0	1	0	0	1
1	1	0	0	0	1
1	1	0	0	0	1
1	1	0	0	0	1
0	1	0	0	0	1
1	0	1	0	0	1
1	1	0	0	0	0
1	1	0	0	0	1
0	1	0	0	0	1
0	0	1	0	0	1
1	1	0	0	0	1
1	1	0	0	0	1
1	0	1	0	0	0
0	0	1	0	0	1
1	0	1	0	1	1
1	0	0	0	0	1
0	0	1	0	0	1
0	0	0	0	0	0
1	1	0	0	0	1
1	1	0	1	0	1
0	0	1	0	0	1

0	0	1	0	0	1
0	0	0	0	0	0
1	1	0	0	0	1
0	0	0	0	0	1
1	0	1	0	0	0
0	0	1	0	0	1
0	1	0	0	0	1
1	1	0	0	0	1
0	0	1	0	0	1
0	1	0	0	0	1
1	1	0	0	0	1
0	1	0	1	0	1
1	0	1	0	0	1
1	1	0	0	0	1
0	1	0	0	0	1
0	1	0	1	0	1
0	0	1	0	0	1
0	1	0	0	0	1
0	0	1	0	0	0
1	1	0	0	0	1
1	1	0	0	0	1
0	0	1	0	0	1
0	1	0	0	0	1
1	1	0	0	0	1
0	1	0	0	0	1
1	1	0	0	0	1
0	1	0	0	0	1
0	1	0	0	0	1
0	0	0	0	0	0
0	1	0	0	0	1
0	1	0	0	0	1
1	0	1	0	0	0
0	0	0	0	0	1
0	1	0	0	0	1
0	1	0	0	0	1
0	0	0	0	0	0
0	0	1	0	0	1
0	1	0	0	0	1
0	1	0	0	0	1
0	0	1	0	1	1
0	0	1	0	0	0
0	0	1	0	0	1
0	0	1	0	0	1
0	0	0	0	0	0
0	0	0	0	0	0
1	0	1	0	0	1
1	0	1	0	1	1
0	1	0	0	0	1
0	1	0	0	1	1
0	1	0	0	0	1
0	1	0	0	0	1
0	1	0	0	0	1
1	1	0	0	0	1
1	0	0	0	0	1
1	0	1	0	0	0
0	0	1	0	0	1
0	1	0	0	0	1

0	0	1	0	0	1
1	1	0	0	0	1
0	1	0	0	0	1
0	1	0	0	0	1
1	1	0	0	0	1
0	0	1	0	0	1
0	1	0	0	0	1
1	0	0	0	1	1
1	0	0	0	1	1
1	1	0	0	0	1
0	0	0	0	0	0
1	1	0	0	0	1
0	0	1	0	0	1
0	1	0	0	0	1
1	1	0	0	1	1
1	0	0	0	0	1
1	0	0	0	0	1
1	1	0	0	0	1
0	0	0	0	0	0
0	1	0	0	0	1
1	0	1	0	0	0
1	0	1	0	0	1
0	0	1	0	0	0
1	1	0	0	0	1
1	1	0	0	0	1
0	1	0	0	0	1
1	1	0	0	0	1
1	1	0	0	0	1
0	0	0	0	0	0
0	0	1	0	0	1
0	1	0	0	0	0
0	0	1	0	0	1
0	1	0	0	0	1
1	0	1	0	0	1
0	1	0	0	0	1
1	0	1	0	0	0
1	0	1	0	0	1
1	0	0	0	0	1
0	1	0	0	0	1
0	1	0	0	0	1
1	0	1	0	0	1
0	0	1	0	0	0
0	1	0	0	0	1
0	1	0	0	0	1
1	1	0	1	0	1
0	1	0	0	0	1
1	1	0	0	0	1
0	0	1	0	0	0
0	1	0	1	0	1
0	1	0	0	0	1
0	0	0	0	0	0
0	1	0	0	0	1
0	0	1	0	0	1
0	1	0	0	0	1
0	1	0	0	0	1
0	0	0	0	0	1
0	1	0	0	0	1

0	0	1	0	0	0
0	0	1	0	1	1
0	0	1	0	0	0
0	0	1	0	0	0
1	0	0	0	0	0
0	0	1	0	0	0
1	0	0	0	0	0
0	0	0	0	0	1
0	1	0	0	0	1
0	0	1	0	0	1
0	0	0	0	0	1
0	0	0	0	0	0
0	0	0	0	0	1
0	0	1	0	0	1
0	0	1	0	0	1
0	0	0	0	0	0
0	0	0	0	0	1
0	0	0	0	0	0
1	1	0	0	0	1
0	1	0	0	0	1
0	0	1	0	0	1
0	0	1	0	0	0
0	1	0	0	0	1
0	1	0	0	0	1
0	0	1	0	1	1
1	0	1	0	0	0
0	1	0	0	0	1
0	0	0	0	0	1
0	0	0	0	0	0
0	0	0	0	0	0
0	0	0	0	0	0
0	0	1	0	0	0
0	0	0	0	0	0
0	1	0	0	0	1
1	0	1	0	0	0
1	0	0	0	0	0
0	0	0	0	0	1
0	0	0	0	0	0
0	0	1	1	0	1
1	1	0	0	0	1
1	0	0	0	0	0
1	0	0	0	0	0
0	0	0	0	0	0
0	1	0	0	0	1
0	1	0	0	0	0
1	0	0	0	0	0
1	1	0	0	0	1
0	0	0	0	0	0
0	0	1	0	0	0
0	0	1	0	0	0
1	1	0	0	0	1
1	0	0	0	0	1
0	0	1	0	0	1
1	1	0	0	0	1
0	0	1	0	0	0
1	0	1	0	0	1
0	0	1	0	0	1

1	0	1	0	0	1
1	1	0	1	0	1
0	1	0	0	0	1
1	0	1	0	0	1
0	0	1	0	0	1
0	0	0	0	0	0
1	0	1	0	0	0
0	0	1	0	0	1
0	1	0	0	0	1
0	1	0	0	0	1
0	1	0	0	0	1
0	1	0	0	0	1
0	0	1	0	0	0
0	0	0	0	0	0
0	0	1	0	0	0
1	1	0	0	0	0
0	1	0	0	0	1
0	1	0	0	0	1
0	1	0	0	0	0
0	0	1	0	0	0
0	1	0	0	0	1
0	0	1	0	0	1
1	0	0	0	0	1
0	0	0	0	0	1
1	0	1	0	0	1
0	0	1	0	0	1
0	0	1	0	0	1
0	0	1	0	0	1
0	0	0	0	0	0
0	0	1	0	0	0
0	0	0	0	0	0
1	0	1	0	0	1
0	1	0	0	0	1
1	1	0	0	0	1
0	1	0	0	0	0
1	1	0	0	0	1
0	0	0	0	0	0
0	0	1	0	0	0
0	0	0	0	0	0
1	0	1	0	0	1
0	0	0	0	0	0
1	0	1	0	0	1
1	0	1	0	0	1
1	1	0	0	0	1
0	0	1	0	0	0
0	0	0	0	0	1
0	0	1	0	0	1
0	0	0	0	0	0
0	0	1	0	0	1
0	0	1	1	0	1
0	0	1	0	0	1
1	0	1	0	0	0
1	0	1	0	0	1
0	0	1	0	0	0
0	0	0	0	0	0
1	0	0	0	0	0
0	0	0	0	0	0

0	0	0	0	0	0
0	0	0	0	0	1
1	0	1	0	0	1
0	0	1	0	0	1
0	0	0	0	0	1
0	0	1	0	0	1
0	0	0	0	0	1
0	0	0	0	0	1
0	0	0	0	0	1
0	0	1	0	0	0
0	0	0	0	0	1
0	0	0	0	0	0
0	0	1	0	0	0
0	0	1	0	0	1
1	1	0	0	0	1
1	0	1	0	0	1
0	0	1	0	0	1
0	1	0	0	0	0
0	1	0	0	0	1
1	1	0	0	0	1
1	1	0	0	0	1
0	0	0	0	0	0
1	1	0	0	0	1
0	0	0	0	0	0
1	0	0	0	0	0
0	0	1	0	0	0
0	0	0	0	0	0
0	0	1	0	0	1
1	0	1	1	0	1
0	0	0	0	0	0
0	0	0	0	0	0
0	0	1	0	0	1
0	0	0	0	0	1
0	0	1	0	0	0
0	0	0	0	0	0
1	0	0	0	0	0
0	0	1	0	0	1
0	0	1	0	0	0
1	1	0	0	0	1
0	0	0	0	0	0
0	0	0	0	0	0
1	0	1	0	0	1
0	1	0	0	0	1
1	0	0	0	0	0
1	0	0	0	0	0
0	0	1	0	0	1
0	0	0	0	0	0
1	1	0	0	0	1
0	0	0	0	0	1
0	1	0	0	0	1
1	0	1	0	0	1
1	0	0	0	0	0
0	0	0	0	0	0
0	0	0	0	0	1
0	0	1	0	0	1
0	0	0	0	0	1
0	0	0	0	0	1

0	0	0	0	0	0
0	0	1	0	0	0
0	0	1	0	0	1
1	0	0	0	0	0
0	0	0	0	0	0
1	1	0	0	0	1
0	0	1	0	0	1
1	0	1	0	0	0
0	0	1	0	0	1
0	0	1	0	0	1
0	0	0	0	0	1
0	0	0	0	0	1
1	0	1	0	0	1
0	0	0	0	0	1
0	0	1	0	0	1
0	1	0	0	0	1
0	0	0	0	0	0
0	0	1	0	0	0
0	0	0	0	0	1
0	0	0	0	0	0
0	0	1	0	0	0
0	0	1	0	0	0
1	1	0	0	0	0
0	1	0	0	0	1
1	0	1	0	0	0
1	0	1	0	0	0
1	0	0	0	0	1
0	0	1	0	0	1
1	0	1	0	0	1
1	1	0	0	0	0
0	0	0	0	0	0
0	1	0	0	0	1
0	1	0	0	0	1
0	0	0	0	0	0
1	0	1	0	0	1
1	1	0	0	0	1
0	1	0	0	0	1
0	1	0	0	0	1
1	0	1	0	0	0
0	0	0	0	0	0
0	1	0	0	0	0
1	1	0	1	0	1
0	0	0	0	0	0
0	0	1	0	0	1
0	0	1	0	0	0
1	0	1	1	0	0
1	0	0	0	0	0
0	0	1	0	0	1
0	0	0	0	0	0
0	0	0	0	0	0
0	1	0	0	0	1
0	0	0	0	0	0
1	0	1	0	0	0
0	0	0	0	0	0
0	0	1	0	0	0
0	0	0	0	0	1
0	0	1	0	0	0

0	1	0	0	0	0
0	0	1	0	0	0
0	0	0	0	0	0
0	0	1	0	0	0
0	0	0	0	0	0
0	0	1	0	0	1
0	0	1	0	0	1
0	0	0	0	0	0
1	0	1	0	0	0
1	1	0	0	0	1
1	0	1	0	0	1
0	0	0	0	0	0
0	0	0	0	0	1
1	0	1	0	0	0
1	1	0	0	0	1
1	1	0	0	0	1
0	0	0	0	0	1
0	0	0	0	0	1
1	0	0	0	0	0
0	0	1	0	0	0
0	0	0	0	0	1
1	0	0	0	0	0
0	0	1	0	0	0
1	0	0	0	0	0
0	0	0	0	0	0
0	0	0	0	0	1
0	0	0	0	0	0
1	1	0	0	0	1
1	1	0	0	0	1
0	0	0	0	0	0
0	1	0	0	0	1
0	0	1	0	0	0
0	0	0	0	0	1
0	0	1	0	0	1
0	0	0	0	0	1
1	0	1	0	0	1
1	0	1	0	0	1
0	0	1	0	0	1
0	0	0	0	0	0
0	0	0	0	0	0
0	0	0	0	1	1
1	1	0	0	0	1
0	0	0	0	0	0
0	1	0	0	0	1
1	1	0	0	0	1
0	0	1	0	0	1
0	0	0	0	0	1
0	0	0	0	0	0
1	0	1	0	0	0
0	0	1	0	0	1
0	0	1	0	0	1
0	0	1	0	0	1
0	0	1	0	0	1
0	0	0	0	0	1
0	0	1	0	0	1
0	0	1	0	0	1
1	1	0	1	0	1

0	0	0	0	1	1
0	0	0	0	1	1
0	0	0	0	0	1
1	0	0	1	0	1
0	1	0	0	0	1
0	0	0	0	0	1
0	0	0	0	0	1
0	0	0	0	0	0
0	0	0	0	0	0
0	0	0	0	0	1
0	1	0	0	0	0
1	1	0	1	0	1
0	0	1	0	0	1
1	1	0	0	0	0
0	1	0	0	0	1
0	1	0	0	0	1
0	0	0	0	0	1
1	0	1	0	0	1
0	0	1	0	0	0
1	0	0	0	0	1
0	0	1	0	1	1
0	0	1	0	0	1
0	0	0	0	0	0
0	0	0	0	0	0
0	0	0	0	0	0
0	0	0	0	0	1
0	0	0	0	0	0
0	0	0	0	0	1
0	0	1	0	0	1
0	0	0	0	0	0
0	0	0	0	0	1
0	0	0	0	0	0
0	0	1	0	0	1
0	1	0	0	0	1
0	1	0	0	0	1
1	0	1	0	0	0
0	0	1	0	0	1
0	0	0	0	0	1
1	0	1	0	0	1
0	0	1	0	0	0
0	0	1	0	0	0
0	0	0	0	0	0
1	0	1	0	0	1
0	0	1	0	0	0
0	0	1	0	0	0
0	1	0	0	0	1
0	1	0	0	0	1
0	1	0	0	0	1
1	1	0	0	0	1
1	1	0	0	0	1
1	0	1	0	0	1
0	1	0	0	0	1
0	0	0	0	0	0
0	1	0	0	0	1
0	0	0	0	0	0
0	0	1	0	0	0
0	0	0	0	0	0

0	1	0	1	0	1
0	0	0	0	0	0
1	1	0	0	0	1
0	0	0	0	0	1
0	0	1	0	0	0
1	1	0	0	0	1
0	0	1	0	0	0
0	0	0	0	0	0
0	0	1	0	0	0
0	0	1	0	0	0
0	0	1	0	0	0
0	0	1	0	0	1
0	0	1	0	0	0
1	0	1	0	0	1
0	0	1	0	0	1
0	0	0	0	0	0
0	0	0	0	0	0
1	0	1	0	0	0
1	0	1	0	0	1
0	1	0	0	0	1
1	1	0	0	0	1
1	0	1	0	0	1
1	0	1	0	0	0
0	0	0	0	0	1
0	1	0	0	0	0
0	0	0	0	0	1
0	0	0	0	0	0
0	1	0	0	0	1
0	1	0	1	0	1
0	0	0	0	0	0
1	0	0	0	0	0
1	0	1	0	0	0
1	0	0	0	1	1
0	0	1	0	1	1
0	0	0	0	0	0
0	0	0	0	0	0
0	0	0	0	0	0
0	0	0	0	0	1
0	0	1	0	0	1
0	0	0	0	0	1
0	0	0	0	0	0
1	1	0	0	0	0
0	0	1	0	0	1
0	0	1	0	0	1
0	0	1	0	0	1
1	0	1	0	0	0
0	0	0	0	0	0
0	0	0	0	0	0
0	1	0	0	0	1
0	0	0	0	0	1
0	0	0	0	0	1
0	0	1	0	0	1
0	0	0	0	0	0
0	0	1	0	0	1
0	0	1	0	0	1
0	0	1	0	0	0
1	0	1	0	1	1

0	1	0	0	0	1
0	0	1	0	0	1
0	1	0	0	0	1
0	1	0	0	0	1
0	1	0	0	0	1
0	1	0	0	0	1
0	0	1	0	0	1
0	1	0	0	0	1
0	0	0	0	0	1
0	1	0	0	0	1
0	0	0	0	0	1
0	0	0	0	0	0
0	0	0	0	0	0
0	0	1	0	0	1
0	0	0	0	0	0
0	0	0	0	0	1
1	0	1	0	0	1
0	0	1	0	0	0
1	0	1	0	0	1
0	0	1	0	0	0
0	0	1	0	0	1
0	0	1	0	0	1
0	0	1	0	0	1
0	0	1	0	0	0
0	1	0	0	0	0
0	1	0	0	0	1
1	1	0	0	0	1
0	0	1	0	0	1
0	1	0	0	0	0
0	0	0	0	1	1
0	0	0	0	0	0
0	1	0	0	0	1
0	0	1	0	0	1
0	0	1	0	0	0
0	1	0	0	0	1
0	0	1	0	0	0
0	0	1	0	0	1
0	0	0	0	0	0
1	0	0	0	0	0
0	0	0	0	0	0
0	0	0	0	0	0
1	0	1	0	0	1
1	0	0	0	0	0
0	0	1	0	0	0
0	0	0	0	0	1
0	0	0	0	0	0
0	0	0	0	0	1
0	0	0	0	0	1
0	0	1	0	0	1
0	0	1	0	0	1
0	0	1	0	0	1
0	0	1	0	0	1
0	0	1	0	0	1
0	0	1	0	0	1
1	1	0	0	0	1
0	1	0	0	0	1
1	0	1	0	0	1

0	0	1	0	0	1
1	0	1	0	0	1
1	0	1	0	0	0
1	0	0	0	0	1
1	0	0	0	0	1
0	0	0	0	0	0
0	0	0	0	0	0
0	0	1	0	0	1
1	0	0	0	0	0
0	0	1	0	0	0
0	1	0	0	0	1
1	0	0	0	0	1
1	1	0	0	0	1
0	0	0	0	0	1
1	1	0	0	0	1
1	0	1	0	0	1
0	0	1	0	0	1
0	0	1	0	0	0
0	0	1	0	0	1
0	0	0	0	0	0
1	0	1	0	0	1
0	0	1	0	0	0
1	0	0	0	0	0
0	0	0	0	0	0
0	1	0	0	0	1
0	0	0	0	0	1
0	0	0	0	0	0
0	0	0	0	0	0
0	0	1	0	0	1
0	0	1	0	0	0
1	1	0	0	0	1
0	0	1	0	0	1
0	1	0	0	0	1
0	1	0	0	0	1
0	0	0	0	0	0
0	0	0	0	0	0
0	0	1	0	0	1
1	0	1	0	0	1
0	1	0	0	0	1
0	0	1	0	0	1
0	0	0	0	0	1
0	0	1	0	0	1
0	0	1	0	0	0
1	0	0	0	0	0
0	1	0	0	0	1
0	0	1	0	0	0
0	1	0	0	0	1
1	0	0	0	0	0
1	1	0	0	0	1
1	0	0	0	0	0
0	0	1	0	0	1
0	0	1	0	0	1
0	0	1	0	0	0
0	1	0	0	0	0
0	0	1	0	0	0
0	0	1	0	0	1
1	1	0	0	0	1

1	0	1	0	0	1
0	0	1	0	0	0
0	0	1	0	0	0
0	0	0	0	0	0
0	0	0	0	0	1
1	0	1	0	0	1
1	0	1	0	0	1
0	0	1	0	0	0
1	0	1	0	0	1
0	0	1	0	0	0
0	0	0	0	0	1
0	0	0	0	0	1
1	1	0	0	0	1
0	0	1	0	0	1
0	0	1	0	0	1
0	0	0	0	0	0
0	1	0	0	0	1
0	0	0	0	0	1
0	1	0	1	0	1
1	0	0	0	0	1
1	0	0	0	0	1
0	1	0	0	0	1
1	1	0	0	0	1
1	1	0	0	0	1
0	1	0	0	0	1
0	1	0	0	0	1
1	0	1	0	0	1
0	0	1	0	0	1
0	0	0	0	0	1
0	1	0	0	0	1
1	0	0	0	0	0
0	1	0	0	0	1
0	0	1	0	0	0
1	0	1	0	0	0
0	0	0	0	0	1
0	0	0	0	0	1
0	0	1	0	0	1
0	0	1	0	0	1
0	1	0	0	0	1
0	1	0	0	0	0
0	0	1	0	0	1
0	0	1	0	0	1
0	0	0	0	0	0
0	0	1	0	0	0
0	0	1	0	0	1
1	1	0	0	0	1
0	1	0	0	0	1
0	0	0	0	0	0
0	0	0	0	0	0
1	0	0	0	0	0
1	0	1	0	0	1
0	0	1	0	0	1
0	0	1	0	0	0
0	0	1	0	0	0
0	0	0	0	0	0
1	1	0	0	0	1
0	0	0	0	0	0

0	0	1	0	0	1
0	1	0	0	0	1
0	0	1	0	0	1
0	0	1	0	0	1
1	0	1	0	0	1
0	0	1	0	0	1
0	1	0	0	1	1
0	1	0	0	1	1
1	0	1	0	0	1
0	1	0	0	0	1
0	1	0	0	0	1
0	0	1	0	0	1
0	0	1	0	0	1
0	0	1	0	0	0
0	0	1	0	1	1
0	0	1	0	0	1
0	1	0	0	0	0
0	0	1	0	0	1
0	0	1	0	0	1
0	0	0	0	0	1
0	0	1	0	0	0
0	0	0	0	0	0
0	0	0	0	0	1
0	0	0	0	0	1
0	0	0	0	0	0
1	0	1	0	0	1
0	0	1	0	0	1
0	0	1	0	0	1
1	1	0	0	0	1
0	1	0	0	0	1
0	0	1	0	0	1
1	0	1	0	0	1
0	1	0	0	0	1
1	0	1	0	0	1
0	0	0	0	0	0
0	0	1	0	0	1
0	1	0	1	0	1
0	0	0	0	0	0
0	0	0	0	0	0
0	0	0	0	0	1
0	0	1	1	0	1
1	0	1	0	1	1
0	0	1	0	0	1
0	1	0	0	0	1
0	0	1	0	0	1
0	0	1	0	0	1
0	0	0	0	0	1
0	1	0	0	0	1
0	0	1	0	0	0
1	1	0	0	0	1
1	0	1	0	0	1
0	0	0	0	0	0
0	1	0	0	0	1
0	0	1	0	0	0
0	0	1	0	0	1
0	0	0	0	0	0
0	0	1	0	0	0

0	0	0	0	0	0
0	0	0	0	0	0
0	0	1	0	0	1
1	1	0	0	0	1
0	1	0	0	0	1
0	1	0	0	0	1
0	0	0	0	0	1
0	0	0	0	0	0
0	0	0	0	0	0
0	1	0	0	0	1
0	0	0	0	0	1
1	0	0	0	0	0
0	0	1	0	0	0
0	0	0	0	0	0
0	0	1	1	0	0
0	0	0	0	0	0
0	1	0	0	0	1
0	0	0	0	0	0
0	0	0	0	0	0
1	0	1	0	0	1
1	0	0	0	0	0
0	1	0	0	0	1
0	0	0	0	0	0
0	0	0	0	0	0
1	0	0	0	0	0
0	0	0	0	0	1
0	0	0	0	0	0
0	1	0	0	0	1
0	0	0	0	0	1
0	0	0	0	0	0
0	0	0	0	0	1
1	0	1	1	0	1
0	0	0	0	0	1
0	0	0	0	0	0
0	0	1	0	0	1
0	1	0	0	0	0
1	0	0	0	0	0
1	1	0	0	0	1
0	1	0	0	0	1
0	0	1	0	0	1
0	0	0	0	0	0
1	0	1	0	0	1
0	0	1	0	0	0
1	0	0	0	0	1
0	0	0	0	0	0
0	0	0	0	0	1
0	0	0	0	1	1
1	0	1	0	0	1
1	0	1	0	0	0
0	0	0	0	0	1
0	0	1	0	0	0
0	0	0	0	0	0
0	0	1	0	0	0
0	0	1	0	0	1
0	0	1	0	0	1
0	0	0	0	0	0
0	1	0	0	0	0

1	1	0	0	0	1
0	0	0	0	0	1
0	1	0	0	0	1
0	0	1	0	0	0
1	1	0	0	0	1
0	0	1	0	0	1
0	0	1	0	0	0
1	1	0	0	0	1
0	0	0	0	0	0
0	0	0	0	0	0
0	0	1	0	0	0
0	0	1	0	0	1
0	0	1	0	0	0
0	0	0	0	0	0
0	0	1	0	0	1
0	0	0	0	0	0
0	0	1	0	0	1
0	0	0	0	0	0
1	0	1	0	0	1
0	0	0	0	0	0
1	0	1	0	0	1
0	0	0	0	0	0
0	0	1	0	0	1
0	0	1	0	0	1
0	0	0	0	0	0
0	0	1	0	0	1
0	0	0	0	0	0
0	1	0	0	0	0
0	0	0	0	0	0
0	0	0	0	0	0
0	0	1	0	0	1
0	1	0	0	0	1
0	0	1	0	0	0
0	0	1	0	0	1
0	0	1	0	0	0
0	0	0	0	1	1
1	0	0	0	0	1
1	0	1	0	0	1
0	0	0	0	0	0
1	0	1	0	0	1
0	0	0	0	0	1
0	0	0	0	0	1
1	0	1	0	0	1
0	1	0	0	0	1
1	1	0	0	0	1
0	0	0	0	0	1
0	0	0	0	0	0
0	0	1	0	0	0
0	0	1	0	0	1
1	0	0	0	0	1
0	0	1	0	0	1
0	0	0	0	0	1
0	1	0	0	0	1
1	1	0	0	0	0
1	1	0	0	0	1
0	0	1	0	0	1
1	0	0	0	0	0

0	0	0	0	0	0
0	0	1	0	0	0
0	0	1	0	0	1
0	0	0	0	0	0
0	1	0	0	0	1
0	0	1	0	0	1
0	1	0	0	0	1
0	1	0	0	0	1
0	0	0	0	0	0
0	0	0	0	0	1
0	1	0	1	0	1
1	1	0	0	0	1
0	0	0	0	0	0
0	0	1	0	0	0
0	0	1	0	0	1
1	1	0	0	0	1
0	1	0	0	0	1
0	0	0	0	0	1
0	0	1	0	0	0
0	1	0	0	0	1
0	1	0	0	0	1
1	0	1	0	0	0
0	1	0	0	0	1
0	0	1	0	0	1
0	0	1	0	0	1
1	0	1	0	0	0
0	0	1	0	0	1
0	0	1	0	0	0
0	0	1	0	0	1
0	0	1	0	0	1
0	0	0	0	0	0
0	0	1	0	0	1
0	0	1	0	0	1
1	0	1	0	0	0
1	0	0	0	0	0
0	0	0	0	0	0
0	0	0	0	0	0
1	0	1	0	0	1
0	0	1	0	0	0
0	0	0	0	0	1
1	1	0	0	0	1
0	0	1	1	0	1
0	1	0	1	0	1
0	1	0	0	0	1
1	0	1	0	0	1
0	1	0	0	0	1
0	0	1	0	0	0
0	1	0	0	0	1
0	1	0	0	0	1
0	1	0	0	0	1
0	1	0	0	0	1
0	0	1	0	1	1
0	0	1	0	0	1
0	0	1	0	0	1
1	0	1	0	0	1
0	0	1	0	0	1
0	1	0	0	0	1

0	0	1	0	0	0
0	0	1	0	0	0
0	1	0	0	0	1
0	1	0	0	0	1
0	1	0	0	0	1
1	1	0	0	0	1
0	0	1	0	0	1
0	0	1	0	0	1
0	0	0	0	0	0
0	0	1	0	1	1
0	0	0	0	0	0
0	0	1	0	0	0
1	1	0	0	0	1
0	0	1	0	0	1
0	1	0	0	0	1
0	0	0	0	0	0
0	0	1	0	0	0
0	0	0	0	0	1
0	0	1	0	0	0
1	0	1	0	0	1
1	0	1	0	0	1
0	0	1	1	0	0
0	1	0	0	0	1
0	1	0	0	0	1
0	1	0	0	0	1
0	1	0	0	0	1
0	0	0	0	0	1
0	0	1	0	0	1
1	0	0	0	0	0
0	0	1	0	0	0
0	0	1	0	0	0
1	0	1	0	0	1
0	0	1	0	0	0
0	0	1	0	0	0
1	0	1	0	0	0
0	0	1	0	0	1
0	0	1	0	0	1
0	1	0	0	0	1
0	0	0	0	0	1
0	0	0	0	0	1
1	1	0	0	0	0
1	0	0	0	0	0
1	1	0	0	0	1
0	0	1	0	0	0
0	0	1	0	0	0
1	0	1	0	0	1
0	0	0	0	1	1
1	0	1	0	0	1
0	0	0	0	0	1
0	1	0	0	0	1
0	1	0	0	0	1
1	1	0	0	0	1
1	1	0	0	0	1
0	0	0	0	0	1
0	0	1	0	0	0
0	0	0	0	0	0
0	1	0	0	0	1

0	0	1	0	0	0
0	0	0	0	0	0
1	0	1	0	0	1
0	0	0	0	0	1
1	0	1	0	0	1
1	0	1	0	0	1
0	0	1	0	0	1
0	0	1	0	0	1
0	0	0	0	0	0
0	0	0	0	0	0
0	0	1	0	0	0
0	0	0	0	0	1
1	0	1	0	0	0
1	1	0	0	0	1
1	0	0	0	0	1
0	0	0	0	0	0
1	0	0	0	0	1
0	0	1	0	0	1
0	0	0	0	0	1
0	1	0	0	0	1
0	0	1	0	0	1
0	0	0	0	0	1
1	0	1	0	0	1
0	0	1	0	0	1
0	0	1	0	0	1
0	0	1	0	0	1
0	0	1	0	0	0
1	0	1	0	0	0
1	0	0	0	0	0
0	0	1	0	0	1
0	1	0	0	0	1
0	0	1	1	0	1
0	0	0	0	0	0
0	1	0	0	0	1
0	1	0	1	0	1
1	1	0	0	0	1
0	0	1	0	0	0
0	0	1	0	0	1
0	0	0	0	0	0
1	0	0	0	0	0
1	0	1	0	0	1
0	0	0	0	0	0
0	0	0	0	0	0
1	0	0	0	0	0
0	1	0	0	0	1
0	0	1	0	0	1
0	0	1	0	0	1
1	1	0	0	0	1
1	0	0	0	0	0
0	0	1	0	0	0
0	0	1	0	0	1
1	1	0	0	0	1
0	1	0	0	0	0
1	0	1	0	0	0
0	0	0	0	0	1
0	0	1	0	0	1
1	0	0	0	0	1

0	0	1	0	0	1
0	1	0	0	0	1
0	0	1	0	0	1
0	1	0	0	0	1
0	0	1	0	1	1
1	0	1	0	0	1
0	0	0	0	0	0
0	1	0	0	0	1
1	1	0	0	0	1
0	0	1	0	0	0
0	1	0	0	0	1
0	1	0	0	0	1
0	1	0	0	0	0
0	0	0	0	0	1
0	0	1	0	0	1
0	1	0	0	0	1
0	0	1	0	0	0
0	1	0	1	0	1
0	0	1	0	0	1
0	0	1	0	0	0
1	0	1	0	0	1
1	0	0	0	0	1
0	0	0	0	0	1
0	0	1	0	0	1
1	1	0	0	0	1
1	0	1	0	0	1
0	1	0	0	0	1
0	1	0	0	0	1
0	0	0	0	0	1
0	0	1	0	0	1
1	0	1	0	0	1
1	1	0	0	0	1
0	1	0	0	0	1
0	0	1	0	0	1
0	0	1	0	0	1
0	0	1	0	0	1
1	1	0	0	0	1
0	0	1	0	0	1
0	0	1	1	0	1
0	1	0	0	0	0
0	1	0	0	0	1
0	0	1	0	0	1
0	0	0	0	0	0
1	1	0	0	0	1
1	1	0	0	0	1
1	0	1	0	0	1
0	0	0	0	0	0
1	1	0	0	0	1
0	0	0	0	0	0
0	1	0	0	0	1
1	0	1	1	0	1
0	0	1	0	0	1
0	0	1	0	0	0
0	0	0	0	0	1
1	1	0	0	0	1
0	1	0	0	0	1
0	0	0	0	0	1

0	0	0	0	0	0
0	1	0	1	0	1
0	1	0	0	0	1
0	0	1	0	0	0
1	1	0	1	0	1
0	0	0	0	0	1
0	1	0	0	0	1
0	1	0	0	0	1
0	0	0	0	1	1
1	1	0	0	0	0
0	0	1	0	0	1
0	0	1	0	0	1
0	0	1	0	0	1
0	0	1	0	0	0
0	0	1	0	0	1
1	0	0	0	0	0
0	0	1	0	0	1
0	0	0	0	0	1
1	0	1	0	0	1
0	0	0	0	0	0
0	0	0	0	0	1
0	0	1	0	0	1
0	0	0	0	0	0
0	1	0	0	0	1
1	0	1	0	0	1
0	0	0	0	0	1
0	0	1	0	0	1
0	1	0	0	0	1
0	0	1	0	0	1
0	0	1	0	0	1
1	1	0	0	0	1
1	1	0	0	0	1
0	0	1	0	0	0
0	0	0	0	0	0
0	0	0	0	0	0
1	0	1	0	0	1
0	1	0	0	0	1
0	1	0	0	0	1
0	0	0	0	0	1
0	0	0	0	0	0
0	1	0	0	0	1
0	1	0	0	0	1
1	0	0	0	0	0
0	0	0	0	0	0
0	0	0	0	0	0
0	1	0	0	1	1
1	0	1	0	0	1
0	1	0	0	0	1
0	0	1	0	0	1
0	1	0	0	0	0
0	0	0	0	0	0
0	0	1	0	0	1
0	0	1	0	0	0
1	0	1	0	0	0
0	1	0	0	0	1
0	0	0	0	0	0
0	0	1	0	1	1

0	1	0	1	0	1
0	0	0	0	0	0
0	0	1	0	0	1
0	0	1	0	0	0
0	0	1	0	0	0
0	1	0	0	0	1
1	0	1	0	0	0
0	1	0	0	0	1
0	0	1	0	0	0
0	1	0	0	0	1
0	1	0	0	0	1
0	0	0	0	0	1
0	0	1	0	0	1
1	0	1	0	0	1
0	0	1	0	0	1
0	1	0	0	0	1
0	1	0	0	0	1
0	1	0	0	0	1
1	0	0	0	0	1
0	0	0	0	0	1
1	0	1	0	1	1
1	1	0	0	0	0
0	0	1	0	0	0
0	0	0	0	0	1
0	0	1	0	0	1
0	0	0	0	0	0
0	0	1	0	0	1
1	0	1	0	0	1
0	1	0	0	0	1
1	1	0	0	0	0
1	1	0	1	0	1
1	0	0	0	0	1
0	0	1	0	0	1
1	1	0	0	0	1
0	0	0	0	0	1
0	1	0	0	0	1
0	1	0	0	0	1
0	0	1	0	0	1
0	0	1	0	0	1
1	1	0	0	0	1
0	1	0	0	0	1
0	1	0	0	0	1
0	0	0	0	0	0
0	1	0	0	0	1
0	0	1	0	0	1
1	0	0	0	0	0
0	0	0	0	0	1
1	0	0	0	0	1
1	0	1	0	0	1
1	0	1	0	0	0
1	0	0	0	0	1
0	1	0	0	0	1
0	0	0	0	0	1
0	1	0	0	0	1
0	0	1	0	0	0
0	0	0	0	0	0
0	1	0	0	0	1

0	0	0	0	0	1
0	0	1	0	0	1
0	1	0	0	0	1
0	0	1	0	0	0
0	0	1	0	0	1
1	0	1	0	0	0
0	1	0	0	0	1
0	1	0	0	0	1
0	0	1	1	0	1
0	0	1	0	0	1
0	1	0	0	0	1
1	0	1	0	0	1
0	0	1	0	0	0
0	0	0	0	0	0
1	1	0	0	0	1
0	1	0	0	0	1
0	0	1	0	0	1
1	1	0	0	0	1
1	1	0	0	0	1
0	0	0	0	0	1
0	0	0	0	0	1
1	0	1	0	0	1
0	0	1	0	0	0
1	1	0	0	0	1
0	0	1	0	0	1
0	0	1	0	0	1
1	0	0	0	0	0
0	1	0	0	0	1
1	0	1	0	0	1
0	0	0	0	1	1
0	0	1	0	0	1
0	0	0	0	0	0
0	0	0	0	0	1
0	0	1	0	0	1
0	0	0	0	0	0
1	1	0	0	0	1
0	0	0	0	0	0
1	1	0	0	0	1
0	0	1	0	0	0
1	1	0	0	0	1
1	1	0	0	0	1
0	1	0	0	0	1
1	0	1	0	0	1
1	1	0	0	0	1
0	1	0	0	0	1
0	1	0	0	0	0
0	1	0	0	0	1
0	0	0	0	0	1
0	1	0	0	0	1
1	1	0	0	0	1
0	0	1	0	0	1
0	1	0	0	0	1
0	0	1	0	0	1
0	0	0	0	0	1
0	0	0	0	0	0
1	1	0	0	0	1
0	0	1	0	0	1

1	0	1	0	0	1
1	0	0	0	0	0
0	0	0	0	0	0
1	0	1	0	0	0
1	0	1	0	0	1
0	0	0	0	0	1
0	1	0	0	0	1
0	1	0	0	0	1
0	1	0	0	0	1
0	0	0	0	0	0
1	1	0	0	0	1
0	0	1	0	0	1
0	1	0	0	0	1
0	1	0	0	0	1
0	0	1	0	0	0
0	0	0	0	0	1
0	0	0	0	0	0
0	1	0	0	0	1
1	0	1	0	0	1
0	1	0	0	0	1
1	0	0	0	0	0
1	1	0	0	0	1
0	1	0	0	0	1
0	1	0	0	0	1
1	1	0	0	0	1
0	0	0	0	0	0
1	1	0	0	0	1
0	0	0	0	0	1
0	0	0	0	0	0
0	1	0	0	0	0
1	1	0	0	0	1
0	1	0	0	0	1
0	1	0	1	0	1
0	0	1	0	0	1
1	1	0	0	0	1
0	0	0	0	0	1
0	1	0	0	0	1
0	1	0	0	0	1
1	1	0	0	0	1
1	1	0	0	0	1
1	0	1	0	0	1
0	0	1	0	0	1
0	0	1	0	0	1
0	0	0	0	0	1
0	0	1	0	0	0
0	0	1	0	0	0
1	1	0	0	0	1
0	1	0	0	0	1
0	1	0	0	0	1
1	1	0	0	0	1
1	0	0	0	0	0
0	1	0	1	0	1
0	0	1	0	0	1
1	1	0	0	0	1
1	0	0	0	0	1
0	1	0	0	0	1
1	1	0	0	0	1

1	1	0	0	0	1
1	0	0	0	0	1
0	1	0	0	0	1
1	0	1	0	0	1
0	0	1	0	0	1
1	0	1	0	0	1
0	0	0	0	0	0
0	0	0	0	0	1
0	0	1	0	0	0
0	0	0	0	1	1
0	1	0	0	0	1
0	0	0	0	0	0
0	1	0	1	0	1
0	1	0	0	0	1
1	1	0	0	0	1
0	0	1	0	0	1
0	0	1	0	0	0
0	1	0	0	0	1
0	1	0	1	0	1
0	1	0	0	0	1
0	1	0	0	0	1
0	1	0	0	0	1
0	1	0	0	0	1
1	1	0	0	0	1
0	1	0	0	0	1
0	1	0	0	0	1
0	1	0	0	1	1
0	1	0	0	0	1
0	1	0	0	0	1
0	0	1	0	0	1
0	0	1	0	0	0
0	0	1	0	0	1
1	0	1	0	0	1
1	0	1	0	0	1
0	0	1	0	1	1
1	1	0	0	0	1
0	0	1	0	0	1
1	1	0	0	0	1
0	1	0	0	0	1
1	1	0	0	0	1
1	1	0	0	0	1
1	1	0	0	0	1
0	0	1	0	0	1
0	0	0	0	0	1
0	0	1	0	1	1
0	0	1	0	0	1
0	1	0	0	0	1
0	1	0	0	0	1
1	1	0	0	0	1
1	0	1	0	0	1
1	1	0	1	0	1
1	0	1	0	0	1
0	1	0	0	0	1
0	0	1	0	0	1
0	1	0	0	0	1
0	0	1	0	0	1
1	1	0	0	0	1

0	0	1	0	0	1
0	1	0	0	0	1
0	0	1	0	0	1
0	1	0	0	0	1
0	1	0	0	0	1
0	1	0	0	0	1
1	1	0	0	0	1
0	1	0	0	0	0
1	0	1	0	0	1
1	1	0	0	0	1
0	0	1	0	0	1
0	1	0	0	1	1
0	0	1	0	0	1
0	0	0	0	0	0
0	0	0	0	0	1
0	0	1	0	0	1
0	0	1	0	0	1
0	0	1	0	0	1
0	0	0	0	1	1
1	1	0	0	0	1
1	1	0	0	0	1
0	1	0	0	0	1
1	1	0	0	0	1
0	1	0	0	0	1
0	1	0	1	0	1
0	0	1	0	0	1
0	0	1	0	0	1
1	1	0	0	0	1
0	0	0	0	0	0
0	0	1	0	0	1
0	0	1	0	0	0
1	1	0	0	0	1
0	0	1	0	0	0
0	0	1	0	0	1
1	1	0	0	0	1
1	0	1	0	0	0
0	0	1	0	0	0
1	1	0	0	0	1
0	0	1	0	0	1
0	0	1	0	0	1
0	0	0	0	0	0
0	0	1	0	0	1
0	1	0	0	0	1
1	1	0	0	0	1
0	1	0	0	0	1
0	0	1	0	0	1
0	1	0	0	0	1
0	0	1	0	0	0
0	1	0	0	0	1
0	0	0	0	0	1
1	1	0	0	0	1
0	1	0	0	0	1
1	0	1	0	0	1
0	0	0	0	0	1
0	1	0	0	0	1
0	0	0	0	0	1
0	0	1	0	0	1

0	1	0	0	0	1
0	0	1	0	0	1
0	1	0	0	0	1
0	0	1	0	0	1
0	1	0	0	0	1
0	0	1	0	0	0
0	1	0	0	0	1
1	0	0	1	0	1
1	0	1	0	0	1
1	1	0	0	0	1
0	1	0	0	0	1
0	0	1	0	0	1
1	1	0	0	0	1
0	0	0	0	0	0
0	0	1	0	1	1
1	1	0	0	0	1
0	0	1	0	0	0
0	1	0	0	1	1
1	1	0	0	0	1
0	1	0	0	0	0
0	1	0	0	0	1
0	1	0	0	0	1
1	1	0	0	0	1
1	0	1	0	0	1
0	1	0	0	0	1
0	0	0	0	0	1
0	1	0	0	0	1
0	1	0	0	0	1
0	1	0	0	0	1
0	1	0	0	0	1
1	1	0	0	0	1
1	1	0	0	0	1
0	0	0	0	0	1
0	0	0	0	0	0
0	0	1	0	0	0
0	1	0	0	0	1
0	0	1	0	0	1
0	1	0	0	0	1
0	0	0	0	0	0
0	0	0	0	0	0
1	0	0	0	0	0
1	0	0	0	0	1
0	1	0	0	0	1
0	0	0	0	0	0
0	0	1	0	0	0
0	0	0	0	0	1
0	0	0	0	0	1
0	0	1	0	0	1
0	1	0	0	0	0
0	1	0	0	0	1
1	0	0	0	0	0
0	0	1	0	0	0
0	0	0	0	0	0
0	0	1	0	0	1
0	0	1	0	0	0
1	1	0	0	0	1
0	0	0	0	0	0

0	0	1	0	0	1
0	0	1	0	0	1
0	1	0	0	0	1
0	0	0	0	0	1
1	0	1	1	0	0
0	0	0	0	0	1
0	0	0	0	0	0
1	0	1	0	0	1
0	0	0	0	0	0
0	0	0	0	0	0
0	0	0	0	0	0
1	0	0	0	0	0
1	0	1	0	0	1
1	1	0	0	0	1
0	1	0	0	0	1
0	1	0	1	0	1
0	0	0	0	0	1
1	0	0	0	0	0
0	0	0	0	0	1
0	0	1	0	0	1
1	0	1	0	0	1
0	0	0	0	0	1
0	0	0	0	0	0
1	0	0	0	0	0
1	0	0	0	0	0
0	0	0	0	0	0
1	0	1	0	0	1
1	0	1	0	0	0
1	0	1	0	0	0
1	0	0	0	0	0
1	0	1	0	0	1
0	0	0	0	0	0
0	1	0	0	0	1
1	0	1	0	0	0
1	0	0	0	0	0
1	1	0	0	0	1
0	0	0	0	0	0
0	0	1	0	0	1
1	0	1	0	0	1
0	1	0	0	0	1
0	0	0	0	0	0
0	1	0	0	0	1
0	1	0	0	0	1
0	0	0	0	0	0
0	0	0	0	0	0
1	0	1	0	0	1
1	0	0	0	0	0
0	0	0	0	0	0
0	0	0	0	0	1
1	1	0	0	0	1
0	0	0	0	0	0
0	0	1	0	0	1
0	1	0	0	0	1
1	1	0	0	0	1
0	0	0	0	0	0
0	1	0	0	0	1
0	1	0	0	0	1

1	0	1	0	0	1
0	1	0	0	0	1
0	0	1	0	0	1
0	0	0	0	0	1
0	1	0	0	0	1
0	0	0	0	0	1
0	0	1	0	0	0
0	0	1	0	0	1
1	0	0	0	0	0
1	0	0	0	0	0
0	0	1	0	0	0
1	0	0	0	0	0
1	0	1	0	0	1
0	1	0	0	0	0
1	1	0	0	0	1
0	1	0	0	0	1
0	0	0	0	0	1
0	0	1	0	0	0
1	0	1	0	0	0
1	1	0	0	0	1
1	0	0	0	0	1
0	0	0	0	0	0
0	0	1	0	0	1
0	0	0	0	0	0
0	0	0	0	0	0
0	0	0	0	0	1
0	0	1	0	0	1
1	1	0	0	0	0
0	1	0	0	0	1
1	0	1	0	0	0
1	0	1	0	0	1
1	1	0	0	0	1
1	0	1	0	0	1
1	1	0	0	0	1
0	0	1	0	0	0
1	1	0	0	0	1
0	1	0	0	0	1
1	0	0	0	0	1
0	0	0	0	0	0
0	0	0	0	0	0
0	0	1	0	0	1
1	0	1	0	0	0
1	0	0	0	0	0
0	0	0	0	0	1
0	0	0	0	0	0
0	0	1	0	0	0
0	1	0	0	0	1
1	1	0	0	0	1
0	0	0	0	0	0
1	0	1	0	0	1
1	0	1	0	0	1
1	1	0	0	0	1
0	1	0	0	0	0
0	0	1	0	0	1
0	0	1	0	0	1
1	0	1	0	0	0
1	1	0	0	0	1

1	0	0	0	0	1
1	0	1	0	0	0
1	0	0	0	0	0
1	0	1	0	0	1
1	1	0	0	0	0
1	1	0	0	0	1
1	1	0	0	0	1
0	1	0	0	0	0
0	1	0	0	0	1
0	0	1	0	0	1
0	0	1	0	0	1
1	1	0	0	0	1
1	0	1	0	0	1
1	0	1	0	0	1
0	0	0	0	0	0
0	0	1	0	0	1
0	0	0	0	0	1
0	1	0	0	0	1
0	0	0	0	0	1
1	1	0	0	0	1
0	1	0	0	0	1
0	1	0	0	0	1
0	1	0	0	0	1
1	0	1	0	0	0
0	0	0	0	0	0
0	0	0	0	0	0
1	0	0	0	0	0
0	0	0	0	0	0
0	0	0	0	0	0
0	0	1	0	0	1
0	1	0	0	0	1
0	0	0	0	0	1
0	0	0	0	0	1
0	1	0	0	0	1
1	0	1	0	0	1
0	0	1	0	0	0
0	0	0	0	0	1
1	0	0	0	0	1
0	0	1	0	0	1
0	0	1	0	0	0
0	1	0	0	0	1
1	0	1	0	0	1
0	0	0	0	0	0
0	0	0	0	0	0
1	1	0	0	0	0
0	1	0	0	0	1
0	0	0	0	0	0
0	0	0	0	0	0
0	0	1	0	0	0
1	0	0	0	0	1
1	0	0	0	0	0
0	0	0	0	0	0
0	0	1	0	0	1
0	0	1	0	0	0
1	1	0	0	0	1
1	1	0	0	0	1
0	0	0	0	0	0

0	0	1	0	0	1
0	0	1	0	0	0
0	0	0	0	0	0
0	0	1	0	0	1
0	1	0	0	0	1
0	0	0	0	0	0
0	0	0	0	0	1
0	0	0	0	0	0
1	1	0	0	0	1
1	0	0	0	0	1
0	1	0	0	0	1
0	0	0	0	0	0
0	0	0	0	0	0
0	0	0	0	0	0
1	0	0	0	0	0
0	0	1	0	0	0
0	0	0	0	0	0
1	0	0	0	0	0
0	0	0	0	0	0
1	0	1	0	0	0
1	0	0	0	0	1
0	1	0	0	0	1
0	0	1	0	0	1
1	0	1	0	0	1
0	1	0	0	0	1
0	1	0	0	0	0
1	1	0	0	0	1
1	1	0	0	0	1
0	0	0	0	0	0
0	0	0	0	0	0
0	0	1	1	0	1
0	0	1	0	0	1
0	0	1	0	0	1
0	1	0	0	0	1
0	1	0	0	0	1
0	0	0	0	0	0
0	0	1	0	0	1
0	0	1	0	0	0
1	0	0	0	0	1
1	0	0	0	0	0
0	0	0	0	0	0
0	1	0	0	0	1
0	1	0	0	0	1
0	0	0	0	0	0
0	0	1	0	0	0
0	0	0	0	0	0
0	0	0	0	0	0
0	0	0	0	0	0
1	0	1	0	0	1
0	0	0	0	0	0
0	0	0	0	0	1
0	0	0	0	0	1
0	1	0	0	0	1
1	0	0	0	0	1
0	0	1	0	0	1
1	1	0	0	0	1
0	0	0	0	0	1

1	0	1	0	0	1
0	0	1	0	0	0
1	0	1	0	0	1
0	0	1	0	0	0
1	0	1	0	0	0
0	0	0	0	0	0
0	0	0	0	0	0
1	1	0	0	0	1
0	1	0	0	0	1
0	0	0	0	0	0
0	0	1	0	0	1
1	0	1	0	0	0
0	0	1	0	0	1
0	0	0	0	0	1
0	0	1	0	0	1
1	1	0	0	0	1
0	1	0	0	0	0
0	1	0	0	0	1
1	0	1	0	0	0
1	1	0	0	0	1
1	0	1	0	0	0
0	0	1	0	0	0
0	0	0	0	0	0
0	0	0	0	0	0
0	0	0	0	0	0
0	0	0	0	0	0
0	0	0	0	0	0
0	1	0	0	0	1
0	0	0	0	0	0
0	1	0	0	0	1
0	0	0	0	0	0
0	0	1	0	0	1
1	1	0	0	0	1
0	0	1	0	0	0
0	0	1	0	0	1
1	1	0	0	0	1
0	0	1	1	0	1
1	0	0	0	0	0
0	0	0	0	0	0
0	0	1	0	0	1
1	0	1	0	0	1
0	0	0	0	0	0
0	0	0	0	0	0
1	1	0	0	0	1
0	1	0	0	0	1
0	0	0	0	0	1
1	0	0	0	0	0
0	0	1	0	0	1
0	0	0	0	0	0
0	1	0	0	0	1
0	1	0	0	0	1
0	1	0	0	0	1
1	0	0	0	0	1
0	0	1	0	0	1
0	0	0	0	0	0
0	0	1	0	0	1
0	0	0	0	0	1

0	0	0	0	0	1
1	0	0	0	0	1
1	0	0	0	0	1
0	1	0	0	0	1
0	1	0	0	0	1
0	1	0	0	0	1
0	0	1	0	0	1
1	0	1	0	0	0
0	0	1	0	0	0
1	0	0	0	0	1
0	0	0	0	0	0
1	1	0	0	0	1
0	0	0	0	0	1
0	1	0	0	0	1
1	0	1	0	0	1
1	1	0	0	0	1
1	1	0	0	0	1
0	1	0	0	0	1
1	0	0	0	0	1
1	1	0	0	0	1
1	0	0	0	0	1
0	0	1	0	0	1
0	0	1	0	0	0
0	0	0	0	0	1
1	0	1	0	0	0
0	0	0	0	0	1
0	0	1	0	0	1
1	1	0	0	0	1
1	0	0	0	0	1
1	1	0	0	0	1
1	0	1	0	0	1
0	0	0	0	0	0
1	0	1	0	0	1
0	1	0	0	0	1
0	0	0	0	0	0
0	0	1	0	0	0
0	0	1	0	0	1
1	0	1	0	0	1
0	1	0	0	0	1
1	0	0	0	0	1
0	1	0	0	0	1
0	0	0	0	0	0
1	0	1	0	0	1
1	0	0	1	0	1
0	1	0	0	0	1
1	0	0	0	0	1
0	0	0	0	0	0
1	1	0	0	0	1
0	1	0	0	0	1
0	1	0	0	0	1
0	0	1	0	0	1
0	0	1	0	0	1
0	1	0	0	0	1
1	0	0	0	0	0
0	0	1	0	0	1
0	0	0	0	0	0
0	0	0	0	0	0

0	1	0	0	0	1
0	1	0	0	0	1
0	0	1	0	0	0
0	0	1	0	0	0
0	0	0	0	0	0
0	0	0	0	0	0
0	0	0	0	0	0
1	1	0	0	0	1
0	1	0	0	1	1
0	1	0	0	0	1
0	1	0	0	0	1
0	1	0	0	1	1
1	1	0	0	0	1
1	1	0	0	0	1
0	0	1	0	0	1
0	0	1	0	0	0
0	1	0	0	0	1
0	1	0	0	0	1
0	1	0	0	0	1
1	0	1	0	0	0
0	0	0	0	0	1
1	0	1	0	0	1
0	1	0	0	0	1
1	1	0	0	0	1
0	1	0	0	0	1
0	1	0	0	0	1
0	0	0	0	0	1
0	1	0	0	0	1
0	0	1	0	0	1
1	0	1	0	0	1
0	0	1	0	0	1
0	0	0	0	0	1
0	0	0	0	0	0
1	0	1	0	0	1
1	1	0	0	0	1
0	0	0	0	0	1
1	1	0	0	0	1
1	0	1	0	0	1
0	1	0	0	0	1
0	0	1	0	0	1
0	0	0	0	0	0
1	0	1	0	0	0
0	0	0	0	0	1
0	0	0	0	0	1
0	0	1	0	0	0
1	1	0	0	0	1
1	0	1	0	0	1
0	0	0	0	0	0
1	1	0	0	0	1
1	0	0	0	0	0
0	0	1	0	0	0
0	0	1	0	0	0
1	0	1	0	0	1
0	0	1	0	0	0
0	0	1	0	0	1
0	0	0	0	0	0
1	0	0	0	0	0

1	1	0	0	0	1
0	0	0	0	0	1
0	1	0	0	0	1
1	0	0	0	0	0
1	0	1	0	0	0
0	0	1	0	0	1
1	1	0	0	0	1
1	0	0	0	0	0
0	0	1	0	0	1
1	0	1	0	0	1
0	1	0	0	0	1
0	0	0	0	0	0
0	1	0	0	0	1
0	1	0	0	0	1
0	0	0	0	0	1
0	0	0	0	0	0
0	0	0	0	0	1
0	0	0	0	0	0
0	0	0	0	0	1
0	0	0	0	0	0
0	1	0	0	0	1
1	1	0	0	0	0
0	1	0	0	0	1
0	0	0	0	0	0
0	0	0	0	0	1
0	0	0	0	0	1
1	1	0	0	0	1
0	1	0	1	0	1
0	0	0	0	0	1
0	0	0	0	0	0
1	0	0	0	0	1
0	0	0	0	0	1
0	0	0	0	0	1
0	0	0	0	0	0
1	0	0	0	0	1
0	1	0	0	0	1
0	0	1	0	0	0
0	0	1	0	0	1
0	0	1	0	0	0
0	0	1	0	0	0
0	0	1	0	0	0
0	1	0	0	0	1
1	0	0	0	0	1
0	1	0	0	0	1
0	0	0	0	0	1
0	0	1	0	0	1
1	0	0	0	0	1
1	0	1	0	0	1
1	0	1	0	0	1
0	0	1	0	0	0
0	1	0	0	0	1
1	1	0	1	0	1
1	0	1	0	0	0
0	0	0	0	0	1
0	0	1	0	0	1
1	0	1	0	0	1
1	1	0	0	0	1

0	0	1	0	0	1
0	0	1	0	0	0
0	1	0	1	0	1
0	0	0	0	0	0
1	1	0	0	0	1
0	0	0	0	0	0
1	0	1	0	0	1
0	0	1	0	0	1
0	0	0	0	0	0
0	0	0	0	0	0
0	0	0	0	0	1
1	1	0	0	0	0
1	1	0	0	0	1
1	0	0	0	0	0
1	1	0	0	0	1
0	0	0	0	0	1
0	0	1	0	0	1
1	0	1	0	0	1
1	0	0	0	0	0
0	0	0	0	0	1
1	0	1	0	0	1
1	0	0	0	0	1
0	0	1	0	0	1
1	1	0	0	0	1
0	1	0	0	1	1
1	0	0	0	0	1
1	1	0	0	1	1
0	0	0	0	0	0
1	0	1	0	0	1
0	0	0	0	0	0
0	0	0	0	0	0
0	0	0	0	0	0
0	0	0	0	0	0
0	0	0	0	0	1
0	1	0	0	0	1
0	0	0	0	0	1
0	0	1	0	0	1
1	0	0	0	0	1
1	0	1	0	0	0
0	0	1	0	0	0
0	0	1	0	0	1
1	0	0	0	0	0
0	0	1	0	0	1
0	0	1	0	0	0
0	0	0	0	0	0
1	0	1	0	0	1
1	0	0	0	0	0
0	1	0	0	0	1
0	0	1	0	1	1
0	0	0	0	0	0
0	0	0	0	0	0
1	0	0	0	0	0
1	1	0	0	0	0
0	0	0	0	0	0
0	0	0	0	0	1
0	0	0	0	0	0
0	0	0	0	0	0

0	0	0	0	0	0
1	0	1	0	0	1
0	1	0	0	0	1
0	0	0	0	0	1
0	0	0	0	0	1
0	0	0	0	0	1
0	0	0	0	0	1
0	0	0	0	0	1
0	0	0	0	0	1
0	0	0	0	0	1
0	0	0	0	0	1
1	1	0	0	0	1
0	0	0	0	0	1
0	1	0	0	0	1
0	0	0	0	0	0
1	1	0	0	0	1
0	0	0	0	0	0
1	1	0	0	0	0
0	1	0	0	0	1
0	0	0	0	0	0
0	1	0	0	0	1
0	1	0	0	0	1
0	1	0	0	0	1
0	0	0	0	0	0
1	0	0	0	0	0
0	0	0	0	0	1
1	1	0	0	0	1
0	0	0	0	0	0
0	0	0	0	0	0
0	0	0	0	0	0
1	0	0	0	0	0
0	0	0	0	0	0
0	0	0	0	0	0
0	0	0	0	0	0
0	0	0	0	0	0
0	0	0	0	0	0
1	0	0	0	0	0
0	0	0	0	0	0
1	1	0	0	0	0
1	0	0	0	0	0
1	1	0	0	0	0
1	1	0	0	0	1
0	0	0	0	0	0
0	0	0	0	0	0
0	0	0	0	0	0
0	0	0	0	0	0
1	0	0	0	0	0
1	0	0	0	0	0
1	0	0	0	0	0
0	0	0	0	0	0
0	0	0	0	0	0
0	0	0	0	0	0
0	0	1	0	0	1
1	0	0	0	0	0
1	1	0	0	0	0
1	0	0	0	0	0
0	0	0	0	0	0

1	1	0	0	0	1
1	0	0	0	0	0
0	0	0	0	0	0
1	0	0	0	0	0
1	0	0	0	0	0
0	0	0	0	0	0
0	0	0	0	0	0
0	0	0	0	0	0
0	0	0	0	0	0
0	0	0	0	0	0
0	0	0	0	0	0
0	0	0	0	0	0
0	0	0	0	0	0
0	0	0	0	0	0
0	0	0	0	0	0
1	0	0	0	0	1
0	0	0	0	0	0
0	0	0	0	0	0
0	0	0	0	0	0
0	0	0	0	0	0
0	0	0	0	0	0
0	0	0	0	0	0
0	0	0	0	0	0
1	0	0	0	0	0
0	0	0	0	0	0
0	0	0	0	0	0
0	0	0	0	0	0
0	0	0	0	0	0
1	0	0	0	0	0
0	0	0	0	0	0
0	0	0	0	0	0
1	0	0	0	0	0
0	0	0	0	0	0
0	0	0	0	0	0
1	0	0	0	0	0
0	0	0	0	0	0
0	0	0	0	0	0
1	0	0	0	0	0
0	0	0	0	0	0
0	0	0	0	0	0
1	0	0	0	0	0
0	0	0	0	0	0
0	0	0	0	0	0
1	0	0	0	0	0
0	0	0	0	0	0
0	0	0	0	0	0
1	0	0	0	0	0
0	0	0	0	0	0
0	0	0	0	0	0
1	0	0	0	0	0
0	0	0	0	0	0
0	0	0	0	0	0
1	0	0	0	0	0
0	0	0	0	0	0
0	0	0	0	0	0
1	0	0	0	0	0
0	0	0	0	0	0

0	0	0	0	0	0
1	0	0	0	0	0
0	0	0	0	0	0
0	0	0	0	0	0
1	0	0	0	0	0
0	0	0	0	0	0
0	0	0	0	0	0
0	0	0	0	0	1
1	0	0	0	0	0
0	0	0	0	0	0
0	0	0	0	0	0
1	0	0	0	0	0
0	0	0	0	0	0
0	0	0	0	0	0
1	0	0	0	0	0
0	0	0	0	0	0
0	0	0	0	0	0
1	0	0	0	0	0
0	0	1	1	0	1
0	0	0	0	0	0
0	0	0	0	0	0
1	0	0	0	0	0
0	0	0	0	0	0
0	0	0	0	0	0
1	0	0	0	0	0
0	0	0	0	0	0
0	0	0	0	0	0
0	0	0	0	0	0
0	0	0	0	0	0
0	0	0	0	0	0
0	0	0	0	0	0
0	0	0	0	0	0
0	0	0	0	0	0
0	0	0	0	0	0
0	0	0	0	0	0
0	0	0	0	0	0
0	0	0	0	0	0
0	0	0	0	0	0
0	0	0	0	0	0
0	0	0	0	0	0
0	0	0	0	0	0
0	0	0	0	0	0
0	0	0	0	0	0
0	0	0	0	0	0
0	0	0	0	0	0
0	0	0	0	0	0
0	0	0	0	0	0
0	0	0	0	0	0
1	0	1	0	0	0
0	0	0	0	0	0
0	0	0	0	0	0
0	0	0	0	0	0
0	0	0	0	0	0
0	0	0	0	0	0
0	0	0	0	0	0
0	0	0	0	0	0
0	0	0	0	0	0

0	0	0	0	0	0
0	0	0	0	0	0
0	0	0	0	0	0
0	0	0	0	0	0
0	0	0	0	0	0
0	0	0	0	0	0
0	0	0	0	0	0
0	0	0	0	0	0
0	0	0	0	0	0
0	0	0	0	0	0
0	0	0	0	0	0
0	0	0	0	0	0
0	0	0	0	0	0
0	0	0	0	0	0
0	0	0	0	0	0
1	0	0	0	0	0
0	0	0	0	0	0
0	0	0	0	0	0
1	0	0	0	0	0
1	0	0	0	0	0
1	0	0	0	0	0
1	1	0	0	0	0
1	0	0	0	0	0
1	0	0	0	0	0
0	1	0	0	0	1
1	1	0	0	0	0
0	0	0	0	0	0
0	0	0	0	0	1
0	0	0	0	0	1
1	0	1	0	0	1
1	0	0	0	0	0
1	1	0	0	0	1
0	0	0	0	0	0
1	0	0	0	0	0
0	0	0	0	0	1
0	0	0	0	0	1
1	0	0	0	0	0
0	1	0	0	0	1
0	0	0	0	0	0
1	0	0	0	0	0
1	0	0	0	0	0
0	0	0	0	0	0
0	0	0	0	0	0
0	0	0	0	0	0
0	0	0	0	0	0
0	1	0	0	0	1
0	1	0	0	0	1
0	0	1	0	0	1
1	0	1	0	0	1
0	1	0	0	0	1
0	0	0	0	0	1
0	1	0	0	0	1
1	1	0	0	0	1
0	1	0	0	0	1
1	0	1	0	0	1
1	1	0	0	0	1
1	0	1	0	0	0

1	0	0	0	0	1
0	0	0	0	0	1
1	1	0	0	0	1
1	1	0	0	0	0
1	0	1	0	0	1
0	0	1	1	0	1
1	0	1	0	0	1
0	0	1	0	0	0
0	0	0	0	0	1
0	1	0	0	0	1
1	0	1	0	0	0
0	0	1	0	0	0
1	1	0	0	0	0
1	0	1	0	0	0
1	0	1	0	0	0
1	1	0	0	0	1
0	0	0	0	0	0
1	1	0	0	0	1
1	0	0	0	0	1
0	1	0	1	0	1
0	1	0	0	0	1
0	0	1	0	0	0
1	0	1	0	0	0
0	1	0	0	0	1
1	0	1	0	0	0
1	0	1	0	0	1
0	1	0	0	0	1
1	1	0	1	0	1
1	0	1	0	0	0
0	1	0	0	0	0
0	0	1	0	0	0
0	0	1	0	0	0
1	0	0	0	0	0
1	0	1	0	0	1
0	0	1	0	0	1
1	1	0	0	0	1
0	1	0	0	0	1
1	0	1	0	0	0
0	0	0	0	0	1
0	0	0	0	0	0
1	1	0	0	0	1
1	1	0	0	0	1
1	1	0	0	0	1
1	0	1	0	0	1
0	0	1	1	0	1
0	1	0	0	0	1
0	0	0	0	0	1
0	1	0	0	0	1
1	1	0	0	0	1
0	0	1	0	0	1
0	0	1	0	0	1
1	1	0	1	0	1
0	1	0	0	0	1
1	0	1	1	0	1
0	0	1	0	0	0
0	0	0	0	0	1
0	0	1	0	0	0

0	1	0	0	0	1
1	1	0	0	0	1
0	0	1	0	0	1
0	1	0	0	0	1
1	1	0	0	0	1
1	0	1	0	0	1
0	1	0	0	0	1
0	1	0	0	0	1
1	0	1	0	0	1
1	1	0	0	0	1
0	1	0	0	0	1
1	1	0	0	0	1
0	1	0	0	0	1
0	1	0	0	0	1
0	1	0	0	1	1
1	1	0	0	0	1
0	1	0	0	0	1
0	0	0	0	0	1
0	0	1	0	0	1
1	0	1	0	0	1
0	0	1	0	1	1
1	0	1	0	0	0
1	0	1	0	0	1
1	0	1	0	0	1
1	1	0	0	0	1
0	1	0	0	0	1
0	0	1	0	0	1
0	1	0	0	0	1
0	0	0	0	0	1
0	0	1	0	0	1
0	0	1	0	0	1
0	1	0	0	0	1
0	0	1	0	0	1
1	1	0	0	0	1
0	0	1	0	0	1
0	1	0	0	0	1
1	1	0	0	0	1
0	0	1	0	0	0
0	1	0	0	0	1
0	1	0	0	0	1
1	1	0	0	0	1
0	1	0	0	0	1
0	1	0	0	1	1
0	0	1	0	0	0
0	1	0	0	0	1
0	1	0	0	0	1
0	1	0	0	0	1
0	0	0	0	0	1
0	0	1	0	0	1
0	1	0	0	0	1
1	1	0	0	0	1
0	1	0	0	0	1
1	1	0	0	0	1
1	1	0	0	0	1
0	0	1	0	1	1
0	1	0	0	0	0
0	0	1	0	0	1

1	0	0	0	0	1
0	0	0	0	0	0
0	1	0	0	0	1
1	0	0	0	0	1
0	1	0	0	0	1
0	1	0	0	0	1
0	1	0	0	0	0
0	1	0	0	0	1
0	0	1	0	0	1
0	0	0	0	0	0
0	0	1	0	0	1
0	0	1	0	0	1
1	1	0	0	0	1
1	1	0	0	0	1
0	1	0	0	0	1
0	0	1	0	1	1
0	1	0	0	0	1
1	1	0	0	0	0
1	1	0	0	0	1
1	1	0	0	0	1
1	1	0	0	0	1
0	1	0	0	1	1
0	1	0	0	0	1
1	1	0	0	0	0
0	0	0	0	0	1
0	0	1	0	0	0
0	0	1	0	0	1
1	1	0	0	0	1
0	0	0	0	0	1
0	0	0	0	0	1
0	0	1	0	0	1
0	0	1	0	0	0
0	1	0	0	0	1
0	1	0	0	0	1
0	1	0	0	0	1
1	1	0	0	0	1
1	1	0	0	0	1
0	1	0	0	0	1
0	1	0	0	0	1
0	0	0	0	0	0
0	1	0	0	0	1
0	1	0	1	0	1
1	1	0	1	0	1
0	1	0	0	0	1
1	0	1	0	0	1
0	0	0	0	0	1
0	0	0	0	0	0
0	0	0	0	0	0
0	0	0	0	0	0
0	0	0	0	0	1
0	0	0	0	0	1
0	1	0	0	0	1
0	0	1	0	0	1
0	0	1	0	0	1
0	1	0	0	0	1
0	1	0	0	0	1
1	1	0	0	0	1

0	0	0	0	0	0
1	1	0	0	0	1
0	0	1	0	0	0
0	0	1	1	0	1
0	0	0	0	0	1
0	0	0	0	0	0
0	1	0	0	0	1
0	0	1	0	0	1
0	0	0	0	0	0
1	1	0	0	0	1
1	1	0	0	0	1
0	1	0	0	0	1
1	0	1	0	0	1
0	0	0	0	0	1
1	0	1	0	0	1
0	0	1	0	0	1
0	1	0	0	0	1
0	0	0	0	0	0
1	0	0	0	0	1
0	0	0	0	0	0
0	1	0	0	0	1
0	1	0	1	0	1
0	0	1	0	0	1
0	0	0	0	0	1
0	0	0	0	0	0
0	0	0	0	0	0
0	0	0	0	0	1
0	1	0	0	0	1
0	0	0	0	0	0
0	0	0	0	0	0
0	0	1	0	0	1
0	1	0	0	0	1
0	0	0	0	0	1
0	0	0	0	0	1
0	1	0	0	0	1
0	0	0	0	0	0
0	0	1	0	0	0
0	1	0	0	0	1
0	0	0	0	0	0
0	0	1	0	0	1
0	0	1	0	0	0
0	1	0	0	0	1
0	1	0	0	0	1
1	1	0	0	0	1
1	0	0	1	0	0
0	1	0	0	0	0
1	1	0	0	0	1
1	0	1	0	0	1
0	1	0	0	0	1
0	1	0	0	0	1
1	0	1	0	0	1
0	1	0	0	0	1
1	1	0	0	0	1
0	0	0	0	0	1
0	1	0	0	0	1
0	0	1	0	0	1
1	1	0	0	0	1

0	0	1	0	0	0
1	0	1	0	1	1
0	1	0	0	0	1
0	0	0	0	0	0
0	1	0	0	0	1
0	1	0	0	0	1
0	1	0	0	0	1
1	1	0	0	0	1
0	0	0	0	0	1
1	0	0	0	0	1
0	1	0	0	0	1
0	1	0	0	0	1
0	0	1	0	0	0
1	1	0	0	0	1
1	0	1	0	0	1
1	1	0	0	0	1
1	1	0	0	0	1
0	0	1	0	0	1
0	1	0	0	0	1
1	1	0	0	0	1
0	0	0	0	0	1
0	1	0	0	0	0
1	1	0	0	0	1
0	0	0	1	0	1
1	0	0	0	0	1
0	0	1	0	0	0
0	1	0	0	0	0
1	0	1	0	0	0
0	0	1	0	0	1
1	0	0	0	0	1
1	1	0	0	0	1
0	1	0	0	0	1
0	1	0	0	0	1
0	1	0	0	0	1
1	0	0	0	0	1
0	1	0	0	0	1
0	0	1	0	0	0
0	0	0	0	0	0
0	0	1	0	0	1
1	1	0	0	0	1
0	0	0	0	0	1
0	1	0	0	0	0
0	1	0	0	0	1
1	0	1	0	0	1
0	1	0	0	0	1
0	1	0	0	0	1
0	1	0	0	0	1
0	0	1	0	1	1
0	1	0	0	0	1
0	1	0	1	0	1
1	0	1	0	0	1
1	1	0	0	0	1
1	1	0	0	0	0
0	0	1	0	0	1
0	0	1	0	0	0
0	0	1	0	0	0
0	0	1	0	0	0

0	0	1	0	0	0
1	1	0	0	0	1
0	1	0	0	1	1
0	1	0	0	0	1
1	0	1	0	0	1
0	1	0	0	0	1
1	1	0	0	0	1
0	0	0	0	0	0
0	0	0	0	0	1
0	0	1	0	0	1
1	0	1	0	1	1
0	0	0	0	0	0
1	1	0	0	0	1
0	0	1	0	0	1
1	0	1	0	0	0
0	0	1	0	0	0
0	1	0	0	0	1
1	1	0	0	0	1
0	1	0	0	0	1
0	0	1	0	0	1
0	1	0	0	0	1
1	1	0	1	0	1
1	1	0	0	0	1
1	0	1	0	0	1
0	0	0	0	0	1
1	1	0	0	0	1
0	0	1	0	0	0
1	1	0	0	0	1
1	0	0	0	0	0
0	1	0	0	0	1
0	1	0	0	0	1
0	0	0	0	0	0
0	1	0	0	0	1
1	0	1	0	0	0
1	1	0	0	0	1
0	0	1	0	0	1
0	1	0	0	0	1
0	1	0	0	0	1
0	0	0	0	0	0
0	1	0	0	0	1
1	1	0	0	0	1
1	0	1	0	0	1
1	1	0	0	0	1
1	0	1	0	0	1
1	1	0	0	0	1
0	0	1	0	0	1
0	1	0	0	0	1
0	0	1	0	0	1
0	0	1	0	0	1
0	1	0	0	0	1
0	0	1	0	0	0
0	1	0	1	0	1
0	0	1	0	0	1
0	1	0	0	0	1
0	0	1	0	0	1
1	1	0	0	0	1
0	1	0	0	0	1

0	1	0	0	0	1
0	0	0	0	0	0
1	1	0	0	0	1
0	1	0	0	0	1
0	0	1	0	0	0
0	0	1	0	0	0
1	0	1	0	0	0
0	0	1	0	0	0
0	1	0	0	1	1
1	1	0	0	1	1
0	0	1	0	0	1
1	0	1	0	0	1
0	0	0	0	0	1
1	0	1	0	0	1
0	1	0	0	0	1
0	0	1	1	0	1
0	0	1	0	0	1
0	1	0	0	0	1
0	1	0	0	0	1
0	0	1	0	1	1
1	0	0	0	0	0
1	1	0	0	0	1
0	0	1	0	0	0
0	0	1	0	0	1
0	0	0	0	0	1
0	1	0	0	0	1
0	0	0	0	0	0
0	0	1	0	0	0
0	1	0	0	0	1
1	0	1	0	0	1
0	1	0	0	0	1
0	0	1	0	0	0
0	0	1	0	0	1
1	1	0	0	0	1
0	1	0	0	0	1
0	1	0	0	0	1
0	0	1	0	0	0
1	1	0	0	1	1
0	0	1	0	0	1
0	1	0	0	0	1
0	0	1	0	0	1
0	0	1	0	0	1
0	1	0	0	0	1
0	0	1	0	0	1
1	1	0	0	0	1
1	0	1	0	0	1
0	0	0	0	0	0
1	0	1	0	0	1
0	0	0	0	0	1
1	1	0	1	0	1
0	0	1	0	1	1
0	0	1	0	0	0
0	0	1	0	0	0
0	0	1	0	0	1
0	1	0	1	0	1
0	0	1	0	0	1
0	1	0	0	0	1

0	1	0	0	0		1
0	0	1	0	0		1
0	1	0	0	0		1
1	1	0	0	0		1
0	0	0	0	0		0
0	1	0	0	0		1
1	1	0	0	0		1
1	1	0	0	0		1
0	0	1	0	0		1
0	0	1	0	0		1
1	0	1	0	0		1
0	1	0	0	0		1
1	1	0	0	0		0
0	0	0	0	0		0
0	0	1	0	0		1
0	1	0	0	0		1
1	1	0	1	0		1
0	1	0	0	0		1
0	1	0	0	0		1
0	0	0	0	0		0
0	1	0	0	0		1
0	1	0	0	0		1
1	1	0	0	0		1
0	0	1	0	1		1
0	0	0	1	0		1
0	0	0	0	0		0
0	0	1	0	0		1
0	0	1	0	0		1
0	1	0	0	0		1
0	1	0	0	0		1
0	0	1	0	0		1
1	1	0	0	0		1
0	1	0	0	0		1
1	0	1	0	0		1
1	1	0	0	0		1
0	0	1	0	0		1
0	0	1	0	0		0
0	1	0	0	0		1
0	0	1	0	1		1
0	0	1	0	0		0
1	1	0	0	0		1
0	1	0	0	0		1
0	0	0	0	1		1
0	1	0	0	0		0
1	0	1	0	0		0
1	0	0	0	0		0
0	0	1	0	0		1
0	1	0	0	0		1
0	0	1	0	0		1
1	1	0	0	0		1
0	1	0	0	0		1
0	1	0	0	1		1
0	0	0	0	0		1
1	1	0	1	0		1
0	1	0	0	0		1
0	1	0	0	0		1
0	0	0	1	0		1

0	0	1	0	0	0
1	0	1	0	1	1
0	0	1	0	1	1
0	0	1	0	0	1
0	1	0	0	0	1
0	0	1	0	0	1
0	1	0	0	0	1
0	0	1	0	0	1
0	1	0	0	0	1
0	0	1	0	0	1
0	1	0	0	0	1
0	1	0	0	0	0
0	1	0	0	0	1
0	0	1	0	1	1
1	1	0	0	0	1
0	0	1	0	0	0
1	1	0	0	0	1
0	1	0	0	0	1
0	1	0	1	0	1
0	1	0	0	0	1
0	1	0	1	0	1
0	0	1	0	0	0
0	1	0	0	0	1
0	1	0	0	0	1
1	1	0	0	0	1
0	0	1	0	0	0
0	1	0	0	0	1
1	1	0	0	0	1
1	1	0	0	0	1
0	0	1	1	0	1
1	0	0	0	0	0
0	1	0	0	1	1
0	0	1	0	0	1
0	1	0	1	0	1
0	0	1	0	0	0
1	1	0	0	0	1
1	0	0	0	0	1
0	1	0	0	0	1
1	1	0	0	0	1
0	0	1	0	0	1
0	1	0	0	0	1
0	1	0	0	0	0
1	0	1	0	0	1
0	1	0	0	0	1
0	0	1	0	0	1
1	0	1	0	0	0
0	0	0	0	0	0
1	0	1	1	0	1
1	1	0	0	0	1
1	1	0	0	0	1
0	0	1	0	0	1
0	0	1	0	1	1
0	0	1	0	0	0
1	0	1	0	0	0
1	1	0	0	0	1
0	1	0	0	0	1
0	1	0	0	0	1

0	1	0	0	0	1
0	1	0	0	0	1
0	1	0	0	0	1
0	0	0	0	0	0
0	1	0	0	0	1
1	0	0	0	0	1
0	1	0	0	0	1
0	1	0	1	0	1
0	1	0	0	0	0
0	0	0	0	0	1
0	0	1	0	0	1
1	0	1	0	0	1
0	1	0	0	0	1
0	1	0	0	0	1
0	0	1	0	0	1
0	0	0	0	0	1
0	0	1	0	0	0
0	1	0	0	0	1
0	0	1	0	0	0
0	1	0	0	0	1
1	1	0	0	0	1
1	1	0	0	0	1
0	0	0	0	0	0
1	1	0	1	0	1
0	1	0	0	0	1
0	0	1	0	0	1
0	1	0	1	0	1
0	1	0	0	0	1
0	0	1	0	0	0
0	1	0	0	0	1
0	1	0	0	0	1
0	0	1	0	0	1
0	1	0	0	0	1
0	0	1	0	0	1
1	0	1	0	0	1
0	0	1	0	0	1
0	0	0	0	0	0
1	0	1	0	0	1
1	1	0	0	0	1
0	1	0	0	0	1
0	0	1	0	0	1
0	1	0	0	0	1
1	1	0	0	0	1
1	1	0	1	0	1
1	1	0	0	0	1
1	1	0	0	0	1
1	1	0	0	0	1
1	1	0	0	0	1
0	1	0	0	0	1
0	0	0	0	0	0
0	0	1	0	0	0
0	0	0	0	0	0
1	0	0	0	0	1
1	1	0	0	0	1
0	1	0	0	0	1
1	1	0	0	0	1
0	1	0	0	0	1

0	1	0	0	0	1
0	0	1	1	0	1
0	0	1	0	0	1
0	1	0	0	0	1
0	1	0	0	0	1
0	0	1	0	0	1
0	0	1	0	0	1
0	0	1	0	0	1
0	0	1	0	0	1
0	1	0	0	0	1
0	1	0	0	0	1
0	0	1	0	0	1
0	0	1	0	0	1
0	1	0	0	0	1
0	0	1	0	0	1
0	0	0	0	0	1
0	1	0	0	0	1
0	1	0	0	0	1
0	0	1	0	0	0
1	0	0	0	0	0
1	0	1	0	0	1
1	0	0	0	0	1
0	0	1	0	0	0
0	0	0	0	0	1
0	1	0	0	0	0
1	0	1	0	0	1
1	0	1	0	0	0
1	0	1	0	0	0
0	0	0	0	0	0
0	1	0	0	0	1
0	0	1	0	0	0
0	0	1	0	0	1
0	0	1	0	0	1
0	0	1	0	0	1
0	1	0	0	0	0
0	1	0	0	0	1
1	1	0	0	0	1
0	1	0	0	0	1
0	0	1	0	0	1
0	1	0	0	0	1
0	1	0	0	0	1
1	0	1	0	0	1
0	0	1	0	0	1
0	1	0	0	0	1
1	1	0	0	0	1
0	1	0	0	0	1
0	1	0	0	0	1
0	0	1	0	0	0
0	1	0	1	0	1
0	0	1	0	0	1
1	1	0	0	0	1
0	1	0	0	0	1
0	0	0	0	0	0
0	0	1	0	0	1
0	1	0	0	0	1
0	0	1	0	0	0
1	1	0	0	0	1

0	1	0	1	0	1
0	1	0	0	0	1
0	0	1	0	0	1
0	0	1	0	0	1
0	1	0	0	0	1
0	0	0	0	0	1
0	1	0	0	0	1
1	1	0	0	0	1
1	0	0	0	0	1
1	0	1	0	0	0
0	1	0	0	0	1
1	1	0	0	0	1
1	0	0	0	0	1
1	0	0	0	0	0
1	1	0	0	0	1
0	1	0	0	0	1
0	1	0	0	0	1
1	1	0	0	0	1
1	1	0	0	1	1
0	1	0	0	0	1
0	1	0	0	0	1
0	1	0	0	0	1
0	0	0	0	0	0
0	0	0	0	0	1
1	1	0	0	0	1
0	1	0	0	0	1
1	1	0	0	0	1
1	1	0	0	0	1
0	1	0	0	0	1
1	1	0	0	0	1
1	0	1	0	0	1
0	0	0	0	0	0
0	0	1	0	0	0
1	1	0	0	0	1
1	1	0	0	0	1
1	1	0	0	0	1
1	0	1	0	0	1
0	1	0	0	0	1
0	1	0	0	0	1
0	0	1	0	0	1
0	0	1	0	0	1
0	1	0	0	0	1
1	0	1	0	1	1
0	0	1	0	0	1
0	0	1	0	1	1
0	1	0	0	0	1
1	1	0	0	0	1
0	0	1	0	0	1
0	0	1	0	0	0
0	0	1	0	0	0
0	1	0	0	0	1
1	1	0	0	0	1
0	1	0	0	0	1
1	1	0	0	0	1
1	0	1	0	0	1
0	0	1	0	0	0
1	1	0	0	0	1

1	1	0	0	0	1
0	0	1	0	0	1
1	0	1	0	0	0
0	1	0	0	0	1
1	1	0	0	0	1
0	0	0	0	0	1
0	0	1	0	1	1
1	1	0	0	0	1
1	0	1	0	0	1
1	0	1	0	0	0
1	0	1	0	1	1
0	0	1	0	0	0
0	0	0	0	0	0
0	1	0	0	0	1
1	1	0	0	0	1
0	0	1	0	0	1
1	1	0	0	0	1
0	1	0	0	0	1
1	0	1	0	0	1
1	1	0	0	0	1
0	1	0	0	0	1
0	1	0	0	0	1
1	0	1	0	0	1
0	1	0	0	0	1
0	0	1	0	0	1
0	1	0	0	0	1
0	0	1	0	0	1
0	1	0	0	0	1
0	1	0	0	0	1
0	0	1	0	0	0
1	0	0	0	0	1
0	1	0	0	0	1
0	0	1	0	1	1
0	1	0	0	0	1
1	1	0	0	0	1
0	0	1	0	0	1
0	0	1	0	0	1
0	0	1	0	0	1
1	0	1	0	0	0
0	0	1	0	0	1
0	0	0	0	0	0
0	1	0	0	0	1
0	1	0	0	0	1
0	1	0	0	0	1
1	0	1	0	0	1
1	1	0	0	0	1
0	0	0	0	0	1
1	1	0	0	0	1
1	1	0	0	0	1
1	1	0	0	0	1
0	1	0	0	0	1
1	0	1	0	0	1
1	1	0	0	0	1
0	1	0	1	0	1
1	0	1	0	0	0
1	1	0	0	0	1
1	1	0	0	0	1

0	1	0	0	0	1
0	1	0	0	0	1
0	1	0	0	0	1
1	1	0	0	0	1
0	0	1	0	0	1
1	1	0	0	0	1
0	0	1	0	1	1
0	1	0	1	0	1
0	1	0	0	0	1
0	0	0	0	0	0
0	0	0	0	0	0
1	0	1	0	0	1
1	0	1	0	0	0
1	1	0	0	0	1
0	0	1	0	0	0
0	1	0	0	0	0
1	1	0	0	0	1
0	0	1	0	0	1
0	0	0	0	0	0
0	0	1	0	0	0
0	1	0	0	0	1
1	0	1	0	0	0
0	1	0	0	0	1
0	1	0	0	0	1
0	1	0	0	0	1
0	0	1	0	0	1
0	1	0	0	0	1
0	0	1	0	0	0
0	1	0	0	0	1
0	1	0	0	0	1
0	0	1	0	0	1
0	0	1	0	0	1
0	1	0	0	0	1
0	1	0	0	0	1
0	1	0	0	0	1
0	1	0	0	0	1
1	0	1	0	1	1
0	0	1	0	0	1
1	0	0	0	0	0
0	0	1	0	0	1
0	0	1	0	0	0
0	1	0	0	0	1
1	1	0	0	0	0
1	0	1	0	0	1
0	1	0	1	0	1
0	0	0	0	0	1
0	0	0	0	0	1
1	1	0	0	0	1
0	0	1	0	0	0
0	1	0	0	0	1
1	1	0	0	0	1
0	1	0	0	0	1
1	1	0	0	0	1
1	1	0	0	0	1
0	1	0	0	0	1
0	1	0	0	0	1
0	1	0	0	0	1

0	1	0	0	0	1
0	1	0	0	0	1
0	0	1	0	0	1
0	1	0	0	0	1
0	1	0	0	0	0
0	1	0	0	0	1
1	1	0	0	0	1
0	0	1	0	0	1
1	0	1	0	0	1
1	0	1	0	0	1
0	1	0	0	0	1
0	0	1	0	0	0
0	1	0	0	0	1
0	0	1	0	0	1
0	1	0	0	0	1
0	0	1	0	0	0
1	0	1	0	0	0
0	0	0	0	0	0
1	0	0	0	0	0
0	0	1	0	0	1
0	0	1	0	0	1
0	0	1	0	0	1
0	1	0	0	0	1
0	0	1	0	0	1
0	0	0	0	0	1
0	1	0	0	0	1
0	0	1	0	0	1
0	1	0	0	0	1
0	1	0	0	0	1
0	0	0	0	0	0
0	0	1	0	0	1
0	0	1	0	0	1
0	0	1	0	0	1
0	1	0	0	0	1
0	0	1	0	0	1
0	0	0	0	0	1
0	0	1	0	0	1
0	1	0	0	0	1
1	0	1	0	0	0
0	0	1	0	0	1
1	0	1	0	0	1
0	1	0	0	1	1
0	1	0	0	0	1
1	0	0	0	0	0
1	1	0	0	0	1
1	1	0	0	0	1
0	0	1	0	0	0
0	1	0	0	0	1
0	1	0	0	0	1
0	0	1	0	0	0
0	1	0	0	0	1
0	0	0	0	0	1
1	1	0	1	0	1
0	0	0	0	0	0
0	1	0	0	0	1
1	1	0	0	0	1
1	0	0	0	0	1

0	1	0	0	0	1
0	1	0	0	0	1
0	0	1	0	0	1
0	1	0	0	0	1
0	0	0	0	0	0
0	0	1	0	0	1
1	0	0	0	0	0
0	1	0	0	0	1
0	0	0	0	0	0
0	0	1	0	0	0
0	1	0	0	0	1
0	1	0	0	0	1
1	1	0	0	0	1
0	0	1	0	0	0
0	0	0	0	0	1
0	0	0	0	0	0
0	0	1	0	0	0
0	0	1	0	0	0
0	1	0	0	0	1
0	1	0	0	0	1
0	1	0	1	0	1
0	0	0	0	0	0
0	1	0	0	0	1
0	1	0	0	0	1
1	1	0	0	0	1
1	0	1	0	0	1
1	1	0	0	0	1
1	0	1	0	0	1
0	0	0	0	0	0
0	1	0	0	0	1
0	0	1	0	0	1
0	1	0	0	0	0
0	1	0	0	0	1
1	1	0	0	0	1
1	0	1	0	0	1
0	0	1	0	0	1
0	0	1	0	0	1
0	1	0	0	0	1
1	1	0	0	0	0
0	0	1	0	1	1
0	1	0	0	0	1
0	1	0	0	0	1
0	0	1	0	0	0
0	0	1	0	0	1
0	0	1	0	0	0
0	1	0	0	0	1
0	0	1	0	0	1
0	0	1	0	0	1
1	1	0	0	0	1
1	1	0	0	0	1
1	0	0	0	0	1
1	1	0	0	0	1
0	0	1	0	0	0
1	0	1	0	0	1
0	0	1	0	0	1
0	1	0	0	0	1
0	0	0	1	0	1

0	0	1	0	0	1
0	1	0	0	0	1
1	0	1	0	0	0
1	0	0	0	0	0
1	1	0	0	0	1
0	0	1	0	0	1
1	0	1	0	0	0
0	1	0	0	0	1
0	0	1	0	0	1
1	0	1	0	0	1
1	0	1	0	0	0
0	1	0	0	0	1
0	1	0	0	0	1
0	0	1	0	0	1
0	1	0	0	0	1
0	1	0	0	0	1
0	1	0	0	0	1
1	0	1	0	0	1
0	0	1	0	0	1
0	0	1	0	0	1
0	1	0	0	0	1
0	1	0	0	0	1
0	0	1	0	0	1
1	1	0	0	0	1
0	1	0	0	0	1
0	1	0	0	0	1
0	1	0	0	0	1
1	0	1	0	1	1
1	1	0	0	0	1
0	1	0	0	0	1
0	1	0	0	0	1
0	1	0	0	0	1
1	1	0	0	0	1
0	0	1	0	0	1
1	1	0	0	1	1
0	0	1	0	1	1
0	0	1	0	0	0
0	1	0	0	0	1
0	0	0	0	0	1
1	0	1	0	0	1
1	0	1	0	0	0
0	0	0	0	0	1
0	1	0	0	0	1
0	1	0	0	0	1
0	0	1	0	0	1
0	1	0	0	0	1
0	1	0	0	0	1
0	1	0	0	0	1
0	0	1	0	0	1
0	0	1	0	0	1
0	1	0	0	0	0
1	1	0	0	0	1
0	0	1	0	0	1
0	1	0	0	0	1
1	1	0	0	0	1
1	0	1	0	0	1
1	0	1	0	0	1

0	1	0	0	0	0
1	0	1	0	0	1
0	0	0	0	0	0
1	0	0	0	0	1
0	0	0	0	0	0
1	0	1	0	0	1
0	0	1	0	0	1
1	1	0	0	0	1
0	0	1	0	0	1
0	1	0	0	0	1
1	0	1	0	0	1
1	1	0	0	0	1
1	1	0	0	0	1
1	1	0	0	0	1
0	1	0	0	0	1
1	1	0	0	0	1
0	0	1	0	0	1
0	0	1	0	0	0
0	1	0	0	0	0
0	1	0	0	0	1
1	1	0	0	0	1
0	1	0	0	0	1
1	0	1	0	0	1
0	1	0	0	0	1
0	0	0	0	0	0
1	0	1	0	0	1
0	0	0	0	0	1
1	0	1	0	0	0
1	1	0	0	0	1
0	1	0	0	0	1
0	1	0	0	0	1
0	0	0	0	0	1
0	1	0	0	0	0
0	1	0	0	0	1
0	1	0	0	0	1
1	1	0	0	0	1
0	1	0	0	0	1
0	0	1	0	0	0
0	1	0	0	0	1
0	0	1	0	0	1
1	0	1	1	0	1
0	1	0	0	0	1
0	1	0	0	0	1
0	1	0	0	0	1
0	1	0	0	0	1
0	0	1	0	0	1
1	0	0	0	0	1
0	0	0	0	0	0
1	1	0	0	0	1
0	1	0	0	0	1
1	1	0	1	0	1
0	0	1	0	0	1
0	1	0	0	0	1
0	0	1	0	0	1
0	1	0	1	0	1
1	1	0	0	0	1
0	0	1	0	0	1

1	1	0	0	0	1
0	0	1	0	0	0
0	0	0	0	0	1
0	1	0	0	0	0
1	1	0	0	0	1
0	0	0	0	0	1
1	1	0	0	0	0
1	1	0	0	0	1
0	0	1	0	0	1
0	1	0	0	0	1
1	0	1	0	0	1
0	0	1	0	0	0
1	0	1	0	0	0
0	0	1	0	0	1
0	0	1	0	0	0
0	1	0	0	0	1
0	0	0	0	1	1
0	0	0	0	0	1
0	0	1	0	0	0
0	0	0	0	1	1
0	0	1	0	0	1
1	0	1	0	0	1
0	1	0	1	0	1
0	1	0	0	0	0
0	0	0	0	1	1
1	1	0	0	0	1
1	1	0	0	0	1
1	0	0	0	0	1
0	1	0	0	0	1
0	0	1	0	0	0
0	1	0	0	0	1
1	0	0	0	0	0
0	0	1	0	0	0
0	1	0	0	0	1
1	1	0	0	0	1
0	1	0	0	0	1
1	0	1	1	0	1
0	0	1	0	0	1
0	1	0	0	0	1
0	0	1	1	0	1
1	1	0	0	0	1
0	0	1	0	0	0
0	1	0	0	0	1
0	0	1	0	0	1
0	0	1	0	0	1
0	0	1	0	1	1
0	1	0	0	0	0
0	1	0	0	0	1
0	0	1	0	0	0
0	0	0	0	0	1
1	1	0	0	0	1
1	1	0	0	0	1
1	0	1	0	0	1
0	0	0	0	0	1
0	0	1	0	0	0
0	0	1	0	0	0
0	1	0	0	0	1

0	0	1	0	0	1
0	0	1	0	0	1
0	0	1	0	0	1
0	0	0	0	0	0
0	1	0	0	0	1
0	1	0	0	0	1
0	1	0	0	0	1
0	1	0	0	0	1
1	0	0	0	0	1
1	0	0	0	0	0
0	0	1	0	0	0
1	0	1	0	0	1
1	0	0	0	0	0
0	1	0	0	0	1
0	0	1	0	0	1
1	1	0	0	0	1
0	0	0	0	0	0
0	1	0	0	0	0
0	1	0	0	0	1
1	1	0	0	0	1
0	0	1	0	0	1
0	1	0	0	0	1
0	0	0	0	0	1
0	0	1	0	0	0
1	1	0	0	0	1
0	1	0	0	0	1
1	0	1	0	0	0
0	1	0	0	0	1
1	1	0	0	0	1
0	0	0	0	0	0
0	1	0	1	0	1
0	0	1	0	0	0
0	0	0	0	0	1
0	0	1	0	0	1
0	0	1	0	0	0
0	1	0	0	0	1
0	0	1	0	0	0
0	0	1	0	0	1
0	0	1	0	0	0
1	0	1	0	0	1
0	0	0	0	0	0
0	0	0	0	0	1
0	0	0	0	0	0
0	1	0	0	0	1
0	1	0	0	0	1
0	0	0	0	0	0
0	1	0	0	0	1
0	1	0	0	0	1
0	0	1	0	0	1
0	1	0	0	0	1
1	1	0	1	0	1
0	0	1	0	1	1
1	0	1	0	0	1
0	1	0	0	0	1
0	1	0	0	0	1
0	0	1	0	0	1
0	1	0	0	0	1

0	0	0	0	0	1
1	0	1	0	0	1
1	0	1	0	0	1
0	1	0	0	0	1
1	0	1	0	0	1
1	1	0	0	0	1
0	1	0	0	0	1
0	0	0	0	0	1
1	1	0	0	0	1
1	0	1	0	0	1
1	0	1	0	0	1
0	1	0	0	0	1
0	1	0	0	0	1
0	1	0	0	0	1
0	0	1	0	0	1
1	1	0	1	0	1
1	1	0	0	0	1
0	1	0	0	0	1
1	1	0	1	0	1
1	0	1	0	0	1
0	1	0	0	0	1
0	1	0	0	0	1
1	0	0	0	0	0
0	0	1	0	0	1
0	0	0	0	0	1
0	1	0	0	0	1
0	0	1	0	0	1
0	1	0	0	0	1
0	1	0	0	0	1
0	1	0	0	0	1
0	0	1	0	0	1
1	0	1	0	0	1
0	0	0	0	0	0
0	1	0	0	0	1
1	0	1	0	0	1
1	0	1	0	0	1
0	1	0	0	0	1
0	0	1	0	0	0
0	0	0	0	0	1
0	1	0	0	0	1
0	0	1	0	0	0
1	1	0	0	0	1
0	0	1	0	0	1
0	0	0	0	0	1
1	1	0	0	0	1
0	0	1	0	0	0
0	0	1	0	0	0
1	1	0	0	0	1
0	1	0	0	0	1
0	0	1	0	0	0
1	0	1	0	0	1
1	1	0	0	0	1
0	1	0	0	0	1
1	1	0	0	0	1
0	1	0	0	0	1
0	1	0	0	0	1
1	1	0	0	0	1

0	0	1	0	0	0
0	1	0	0	0	1
0	0	1	0	0	1
0	1	0	0	0	1
1	0	0	0	0	1
1	0	1	0	0	0
1	0	1	0	0	1
0	1	0	0	0	1
1	1	0	0	0	1
0	1	0	0	0	1
1	1	0	0	0	1
0	0	1	0	0	0
0	0	1	0	0	1
1	1	0	0	0	1
0	0	1	0	0	1
0	1	0	0	0	1
1	1	0	0	0	1
0	1	0	0	0	0
0	1	0	0	0	1
0	1	0	0	0	1
0	0	1	0	0	1
1	1	0	0	0	1
0	0	1	0	0	1
0	0	0	0	0	1
0	0	1	0	0	1
0	0	1	0	0	1
0	0	0	0	0	1
1	1	0	0	0	1
1	0	1	0	0	0
0	0	1	0	0	1
0	1	0	0	0	1
1	1	0	0	0	1
0	1	0	0	0	0
1	0	1	0	0	0
1	1	0	1	0	1
0	0	0	0	0	1
0	0	1	0	0	1
1	1	0	0	0	1
0	0	0	0	0	1
0	0	1	0	1	1
1	1	0	0	0	1
0	0	0	0	1	1
1	1	0	0	0	1
0	0	1	0	0	1
1	1	0	0	0	0
0	1	0	0	0	0
0	1	0	0	0	1
0	1	0	0	0	1
1	0	1	0	0	1
0	0	1	0	0	1
0	1	0	0	0	1
0	1	0	0	0	1
0	0	1	0	0	1
0	1	0	0	0	1
0	1	0	0	0	1
0	1	0	0	0	1
0	1	0	0	0	1

0	0	1	0	0	1
0	1	0	0	0	1
1	1	0	0	0	1
1	1	0	0	0	1
0	0	1	0	1	1
1	0	1	0	0	1
1	1	0	0	0	1
0	1	0	0	0	1
0	0	1	0	0	1
0	0	1	0	1	1
1	0	0	0	0	0
0	1	0	0	0	1
1	0	1	0	0	1
1	1	0	0	0	1
0	0	0	0	0	1
1	1	0	0	0	1
0	0	1	0	0	1
0	1	0	0	0	1
0	0	1	0	0	0
1	1	0	0	0	1
1	1	0	0	0	1
1	0	0	0	0	0
0	1	0	0	0	1
1	0	1	0	0	1
1	0	1	0	0	1
0	1	0	0	0	1
0	1	0	0	0	1
0	1	0	0	0	1
0	0	1	0	0	1
0	0	1	0	0	1
1	1	0	0	1	1
1	0	0	0	1	1
1	1	0	0	0	1
0	0	1	0	0	1
0	0	0	0	0	0
0	1	0	0	0	1
0	1	0	1	0	1
0	0	0	0	0	1
0	1	0	0	0	1
0	1	0	0	0	1
1	1	0	0	0	1
1	0	1	0	0	1
1	1	0	0	0	1
1	0	1	0	0	1
0	0	0	0	0	1
1	0	1	0	0	1
1	0	1	0	0	1
0	0	1	0	0	1
0	1	0	0	0	1
0	1	0	0	0	1
1	0	1	0	0	1
0	1	0	0	0	1
0	1	0	0	0	1
0	1	0	0	0	0
0	0	1	0	0	0
0	1	0	0	0	1
1	0	1	0	0	1

0	1	0	0	0	1
0	0	1	1	0	1
0	0	0	0	0	1
0	0	1	0	0	1
0	1	0	0	0	1
1	0	1	0	0	0
0	1	0	0	0	1
0	1	0	1	0	1
1	0	1	0	0	0
0	1	0	0	0	1
1	1	0	0	0	1
0	0	0	0	0	0
0	1	0	0	0	1
1	1	0	0	0	1
0	0	1	0	0	0
0	1	0	0	0	1
1	1	0	0	0	1
0	1	0	0	0	1
0	0	0	0	0	0
1	1	0	0	0	1
0	1	0	0	0	1
0	0	1	0	0	1
1	1	0	0	0	1
0	1	0	0	0	1
0	0	1	0	0	1
0	1	0	0	0	1
1	1	0	0	0	1
0	1	0	1	0	1
0	1	0	0	0	0
1	0	1	0	0	1
1	0	1	0	0	1
1	0	0	0	0	1
1	1	0	0	0	1
1	0	1	0	0	1
1	0	1	0	0	1
1	1	0	0	0	1
0	1	0	0	0	1
1	0	0	0	0	1
1	1	0	0	0	1
1	1	0	0	0	1
0	1	0	0	0	1
0	0	0	0	0	0
0	1	0	0	0	1
1	1	0	0	0	1
0	1	0	0	0	1
0	1	0	0	0	1
0	1	0	1	0	1
0	1	0	0	0	1
0	1	0	0	0	1
0	1	0	0	0	1
1	1	0	0	0	1
0	1	0	0	0	1
0	0	1	0	0	1
1	1	0	1	0	1
0	1	0	0	0	1
1	1	0	0	0	1
1	1	0	0	0	1

1	0	1	0	0	1
0	0	1	0	0	1
0	0	1	0	1	1
0	0	1	0	0	1
1	1	0	0	0	1
0	1	0	0	0	1
0	0	1	0	0	0
1	1	0	0	0	1
0	0	1	0	0	1
0	1	0	0	0	1
0	0	1	0	0	0
0	1	0	0	0	1
1	0	0	0	0	1
1	0	0	0	0	1
0	0	1	0	0	1
0	0	1	0	0	1
0	1	0	0	0	1
0	0	0	0	0	1
0	0	0	0	0	1
1	1	0	0	0	1
0	0	1	0	0	1
0	1	0	0	0	1
0	0	1	0	0	0
0	1	0	0	0	1
0	0	0	0	0	1
1	1	0	0	0	1
0	1	0	0	0	1
1	1	0	0	0	1
0	0	0	0	0	0
0	1	0	0	0	1
0	0	1	0	0	1
0	0	1	0	0	1
0	0	1	0	0	1
0	0	0	0	0	0
0	0	0	0	0	1
0	1	0	0	0	1
0	0	1	0	0	1
1	1	0	0	0	1
0	1	0	0	0	1
1	0	1	0	0	1
0	0	1	0	0	0
0	0	1	0	0	1
1	0	1	0	0	1
0	1	0	0	0	1
1	0	1	0	0	1
1	1	0	0	0	1
0	1	0	0	0	1
0	1	0	0	0	1
0	1	0	0	0	1
0	1	0	0	0	1
1	1	0	0	0	1
1	0	1	0	0	1
0	1	0	0	0	1
1	0	1	0	0	1
0	1	0	0	0	1
1	1	0	0	0	1
1	1	0	0	0	1

1	0	1	0	0	1
0	1	0	0	0	1
0	0	1	0	0	1
1	1	0	0	0	1
0	0	1	0	0	1
0	1	0	0	0	1
0	1	0	0	0	1
0	0	1	0	0	1
0	0	0	0	0	0
1	0	1	1	0	1
0	1	0	0	0	1
1	1	0	0	0	1
0	0	0	0	0	1
1	1	0	0	0	1
0	0	1	0	0	1
0	0	1	0	0	1
0	0	1	0	0	1
0	1	0	0	0	1
0	1	0	0	0	1
0	1	0	0	0	1
1	1	0	0	0	1
0	0	1	0	0	1
0	0	1	0	0	1
1	0	0	0	0	1
0	0	0	0	0	0
0	0	1	0	0	0
1	1	0	1	0	1
1	0	1	0	0	1
0	1	0	0	0	1
0	1	0	0	0	1
0	1	0	0	0	1
1	1	0	0	0	1
0	0	1	0	0	0
1	1	0	0	0	1
1	0	1	0	0	1
0	0	1	0	0	0
0	0	0	0	0	0
1	1	0	0	0	1
0	1	0	0	0	1
0	0	1	0	0	0
0	1	0	0	0	1
0	1	0	0	0	1
0	0	1	0	0	1
0	1	0	0	0	1
0	0	1	0	0	1
0	1	0	1	0	1
1	1	0	0	0	1
0	0	1	0	0	1
0	0	1	0	0	0
1	1	0	0	0	1
0	0	1	0	1	1
1	1	0	0	0	1
1	1	0	0	0	1
0	1	0	1	0	1
0	0	0	0	0	0
0	0	1	0	0	0
0	1	0	0	0	1

1	0	1	0	0	1
0	1	0	0	0	1
1	1	0	0	0	1
0	0	0	0	0	0
1	1	0	0	0	1
0	1	0	0	0	1
0	1	0	0	0	1
0	1	0	0	0	1
0	0	1	0	0	1
0	0	1	0	0	1
1	0	0	0	0	0
0	0	1	0	0	1
1	1	0	0	1	1
1	1	0	0	0	1
0	0	1	0	0	1
0	1	0	0	1	1
0	0	0	0	0	0
1	0	1	0	0	0
0	1	0	0	0	1
0	0	1	0	0	1
1	0	1	0	0	0
1	1	0	0	0	1
1	0	1	0	0	1
0	1	0	0	0	1
0	0	1	0	0	1
0	1	0	0	0	1
0	0	1	0	0	1
1	0	0	0	0	0
0	1	0	0	0	1
0	1	0	0	0	1
1	0	0	0	0	1
0	1	0	0	0	1
0	0	1	0	0	1
0	0	1	0	1	1
1	1	0	0	0	1
0	0	1	0	1	1
0	0	0	0	0	1
0	0	1	0	0	1
1	1	0	0	0	1
0	0	1	0	0	0
0	0	0	0	0	0
0	1	0	0	0	1
0	1	0	0	0	1
0	1	0	0	0	1
0	0	1	0	0	1
0	0	1	0	0	1
0	1	0	0	0	1
1	0	1	0	0	1
0	0	1	0	0	0
0	0	1	0	0	0
0	1	0	0	1	1
0	0	0	0	0	0
0	0	1	0	0	1
0	0	1	0	0	0
0	1	0	0	0	1
0	0	1	0	0	1
0	1	0	0	0	1

1	0	1	0	0		1
0	0	0	0	0		0
1	1	0	0	0		1
0	0	1	0	0		1
1	1	0	0	0		1
1	1	0	0	0		1
0	0	1	0	0		1
0	1	0	0	0		1
1	0	0	0	0		1
0	1	0	0	0		1
0	0	0	0	0		1
0	0	0	0	0		1
0	0	0	0	0		0
0	0	0	0	0		0
0	0	0	0	0		0
1	0	1	0	0		1
0	1	0	0	0		1
0	0	1	0	0		1
0	0	0	0	0		1
0	1	0	0	0		0
0	1	0	0	0		1
0	0	0	0	0		1
1	1	0	0	0		1
0	1	0	0	0		0
1	0	0	0	0		1
1	1	0	0	0		1
0	0	1	0	0		1
0	1	0	0	0		1
1	1	0	0	0		1
1	1	0	0	0		1
1	1	0	0	0		1
0	0	1	0	0		1
0	0	1	0	0		1
1	1	0	0	0		1
0	1	0	0	0		1
1	1	0	0	0		1
0	1	0	0	0		1
0	1	0	0	0		1
1	1	0	0	0		1
1	0	1	0	0		1
0	1	0	0	0		1
0	1	0	0	0		1
0	1	0	0	0		1
0	0	1	0	0		1
0	1	0	1	0		1
1	0	1	0	0		1
0	0	1	0	1		1
0	1	0	1	0		1
1	1	0	0	0		1
0	0	1	0	0		0
0	1	0	0	0		1
1	1	0	0	0		1
1	0	1	0	0		1
1	1	0	0	0		1
0	0	1	0	0		1
0	0	1	0	0		1
1	0	0	0	0		0

0	1	0	0	0	0
0	0	1	0	0	1
0	0	0	0	0	1
0	0	0	0	0	1
0	1	0	0	0	1
0	1	0	0	0	1
0	0	0	0	0	1
0	1	0	0	0	1
0	0	1	0	1	1
1	0	0	0	0	0
1	0	0	0	0	0
0	0	0	0	0	1
0	0	1	0	0	1
0	1	0	0	0	1
0	0	1	0	0	1
0	1	0	0	0	1
0	1	0	0	0	1
1	0	1	0	0	1
0	0	1	0	0	1
0	1	0	0	0	1
1	1	0	0	0	0
1	1	0	0	0	1
1	1	0	0	0	1
1	1	0	0	0	1
1	0	1	0	0	1
1	1	0	0	0	1
1	0	1	0	0	0
0	1	0	0	0	1
0	1	0	0	0	1
1	1	0	0	0	1
1	0	1	0	0	1
0	1	0	1	0	1
0	0	1	0	0	1
0	1	0	0	0	1
0	0	1	0	0	0
1	1	0	0	0	1
1	1	0	0	0	1
1	1	0	0	0	1
0	1	0	0	0	1
1	0	1	0	0	1
0	1	0	0	0	1
1	0	1	0	0	1
1	1	0	0	0	1
0	1	0	0	0	1
0	1	0	0	0	1
1	1	0	0	0	1
0	1	0	0	0	1
0	1	0	0	0	1
0	1	0	0	0	1
0	1	0	0	0	0
1	1	0	0	0	0
0	1	0	1	0	1
0	1	0	0	0	1
0	0	1	0	0	1
0	1	0	0	0	1
0	0	0	0	0	1
0	0	1	0	0	1

0	0	0	1	0	1
0	0	1	0	0	0
0	0	1	0	0	1
0	0	0	0	0	0
0	0	0	0	0	0
0	0	0	0	0	0
0	0	0	0	0	0
0	0	1	0	0	1
1	0	1	0	0	0
1	1	0	0	0	0
0	1	0	0	0	1
1	0	0	0	0	1
0	1	0	0	0	1
0	0	0	0	0	0
0	1	0	0	0	1
0	1	0	0	0	1
0	1	0	0	0	1
0	1	0	0	0	1
1	1	0	0	0	1
0	1	0	0	0	1
1	0	1	0	0	1
1	0	1	0	0	1
1	0	1	0	0	1
1	0	1	0	0	1
0	0	1	0	0	0
0	1	0	1	0	1
0	0	0	0	0	1
0	0	1	0	0	0
0	0	0	0	0	1
0	1	0	0	0	1
0	0	1	0	0	0
1	0	1	0	0	0
0	0	1	0	0	1
1	1	0	0	0	1
0	0	0	0	0	0
0	1	0	0	0	1
1	0	1	0	0	1
0	0	1	0	0	1
1	0	1	0	0	1
0	0	1	0	0	1
1	0	1	0	0	0
1	1	0	0	0	1
0	1	0	0	0	1
0	0	0	0	0	0
0	1	0	0	0	1
0	0	1	0	0	1
0	1	0	1	0	1
0	1	0	1	0	1
0	0	1	0	0	1
1	0	1	0	1	0
0	0	1	0	0	1
0	1	0	0	0	1
0	0	1	0	0	1
0	1	0	0	0	1
0	0	1	0	0	1
0	1	0	0	0	1
0	0	1	0	0	1

0	0	1	0	0	1
0	0	1	0	0	1
0	0	1	0	0	0
0	1	0	0	0	1
0	0	1	0	0	1
0	1	0	0	0	1
1	1	0	0	0	1
0	0	0	0	0	1
0	0	1	0	0	0
0	1	0	0	0	1
0	0	1	0	0	1
0	1	0	0	0	1
0	1	0	0	0	1
1	1	0	0	0	0
0	0	0	0	0	0
0	0	0	0	0	1
1	1	0	0	0	1
0	0	1	0	0	1
0	1	0	0	0	1
1	1	0	0	0	1
0	1	0	0	0	1
0	1	0	0	0	1
0	0	0	0	0	1
1	1	0	0	0	1
0	0	1	0	0	0
0	0	1	0	0	1
0	0	0	0	0	0
1	0	0	0	0	0
0	0	1	0	0	1
0	1	0	0	0	1
0	0	1	1	0	1
0	0	1	0	0	1
1	0	1	0	0	1
0	1	0	0	0	1
1	1	0	0	0	1
1	1	0	0	0	0
0	1	0	0	0	1
0	1	0	1	0	1
1	0	1	0	0	1
0	0	1	1	0	1
1	1	0	0	0	1
0	1	0	1	0	1
0	1	0	0	0	1
0	1	0	0	0	1
1	0	1	0	0	1
0	0	1	0	0	1
1	1	0	0	0	1
0	1	0	0	0	1
1	0	1	0	0	1
1	0	1	0	0	1
1	1	0	0	0	1
1	1	0	0	0	1
0	1	0	0	0	1
0	1	0	0	0	1
0	1	0	0	0	1
1	1	0	0	0	1
0	1	0	0	0	1

1	1	0	0	0	1
1	1	0	0	0	1
0	1	0	1	0	1
0	0	1	0	0	1
0	1	0	0	0	1
1	1	0	0	0	1
0	0	0	0	0	1
0	0	1	0	0	0
0	0	0	0	0	0
0	0	0	0	0	0
0	0	0	0	0	1
1	0	0	0	0	0
1	0	0	0	0	1
0	0	0	0	0	1
1	0	0	0	0	1
0	0	0	0	0	1
1	0	0	0	0	1
0	0	0	0	0	1
0	0	0	0	0	1
0	0	0	0	0	1
0	0	0	0	0	1
0	0	0	0	0	1
0	0	0	0	0	1
0	0	0	0	0	1
0	0	0	0	0	1
0	0	0	0	0	1
0	0	0	0	0	1
0	0	0	0	0	1
0	0	0	0	0	1
0	0	0	0	0	1
0	0	0	0	0	1
0	0	0	0	0	1
0	0	0	0	0	1
0	0	0	0	0	1
0	0	0	0	0	1
0	0	0	0	0	1
0	0	0	0	0	1
1	0	0	0	0	1
0	0	0	0	0	1
0	0	0	0	0	1
0	0	0	0	0	1
0	0	0	0	0	1
0	0	0	0	0	1
0	0	0	0	0	1
1	0	0	0	0	1
0	0	0	0	0	1
0	0	0	0	0	1
0	0	0	0	0	1
1	0	0	0	0	1
0	0	0	0	0	1
0	0	0	0	0	1
0	0	0	0	0	1
0	0	0	0	0	1
0	0	0	0	0	1
0	0	0	0	0	1
0	0	0	0	0	1
0	0	0	0	0	1

0	0	0	0	0	1
0	0	0	0	0	1
1	0	0	0	0	1
0	0	0	0	0	1
0	0	0	0	0	1
0	0	0	0	0	1
0	0	0	0	0	1
0	0	0	0	0	1
0	0	0	0	0	1
0	0	0	0	0	1
0	0	0	0	0	1
0	0	0	0	0	1
1	0	0	0	0	1
0	0	0	0	0	1
0	0	0	0	0	1
0	0	0	0	0	1
0	0	0	0	0	1
0	0	0	0	0	1
0	0	0	0	0	1
0	0	0	0	0	1
0	0	0	0	0	0
0	0	0	0	0	1
0	0	0	0	0	1
0	0	0	0	0	1
0	0	0	0	0	1
0	0	0	0	0	1
0	0	0	0	0	1
0	0	0	0	0	1
0	0	0	0	0	1
0	0	0	0	0	1
0	0	0	0	0	1
0	0	0	0	0	1
0	0	0	0	0	1
0	0	0	0	0	1
0	0	0	0	0	1
0	0	0	0	0	1
0	0	0	0	0	1
0	0	0	0	0	1
0	0	0	0	0	1
0	0	0	0	0	1
0	0	0	0	0	1
0	0	0	0	0	1
0	0	0	0	0	1
1	0	0	0	0	1
0	0	0	0	0	1
0	0	0	0	0	1
0	0	0	0	0	1
0	0	0	0	0	1
0	0	0	0	0	1
1	0	0	0	0	1
0	0	0	0	0	1
0	0	0	0	0	1
0	0	0	0	0	1
0	0	0	0	0	1
0	0	0	0	0	1
1	0	0	0	0	1
0	0	0	0	0	1

1	0	0	0	0	1
0	0	0	0	0	1
0	0	0	0	0	1
1	0	0	0	0	1
1	0	0	0	0	1
1	0	0	0	0	1
1	0	0	0	0	1
0	0	0	0	0	1
0	0	0	0	0	1
0	0	0	0	0	1
0	0	0	0	0	1
0	0	0	0	0	1
0	0	0	0	0	1
0	0	0	0	0	1
1	0	0	0	0	1
0	0	0	0	0	1
0	0	0	0	0	1
1	0	0	0	0	1
0	0	0	0	0	1
0	0	0	0	0	1
0	0	0	0	0	1
1	0	0	0	0	1
1	0	0	0	0	1
1	0	0	0	0	1
0	0	0	0	0	1
1	0	0	0	0	1
0	0	0	0	0	1
0	0	0	0	0	1
0	0	0	0	0	1
0	0	0	0	0	1
0	0	0	0	0	1
0	0	0	0	0	1
0	0	0	0	0	1
0	0	0	0	0	1
1	0	0	0	0	1
0	0	0	0	0	1
0	0	0	0	0	1
0	0	0	0	0	1
1	0	0	0	0	1
0	0	0	0	0	1
0	0	0	0	0	1
0	0	0	0	0	1
1	0	0	0	0	1
0	0	0	0	0	1
0	0	0	0	0	1
0	0	0	0	0	1
0	0	0	0	0	1
0	0	0	0	0	1
0	0	0	0	0	1
0	0	0	0	0	1
0	0	0	0	0	1
0	0	0	0	0	1
0	0	0	0	0	1
0	0	0	0	0	1
0	0	0	0	0	1
1	0	0	0	0	1
0	0	0	0	0	1

0	0	0	0	0	1
0	0	0	0	0	1
0	0	0	0	0	1
0	0	0	0	0	1
0	0	0	0	0	1
0	0	0	0	0	1
0	0	0	0	0	1
1	0	0	0	0	1
0	0	0	0	0	1
0	0	0	0	0	1
0	0	0	0	0	1
0	0	0	0	0	1
0	0	0	0	0	1
0	0	0	0	0	1
0	0	0	0	0	1
0	0	0	0	0	1
0	0	0	0	0	1
0	0	0	0	0	1
0	0	0	0	0	0
0	0	0	0	0	0
0	0	0	0	0	0
0	0	0	0	0	0
1	0	0	0	0	1
0	0	0	0	0	1
1	0	0	0	0	1
0	0	0	0	0	1
0	0	0	0	0	1
0	0	0	0	0	1
0	0	0	0	0	0
0	0	0	0	0	1
0	0	0	0	0	1
0	0	0	0	0	1
0	0	0	0	0	1
0	0	0	0	0	1
1	0	0	0	0	1
1	0	0	0	0	1
1	0	0	0	0	1
1	0	0	0	0	1
1	0	0	0	0	1
0	0	0	0	0	1
1	0	0	0	0	1
0	0	0	0	0	1
1	0	0	0	0	1
0	0	0	0	0	1
1	0	0	0	0	1
1	0	0	0	0	1
0	0	0	0	0	0
1	0	0	0	0	1
0	0	0	0	0	1
0	0	0	0	0	1
0	0	0	0	0	1
0	0	0	0	0	1
0	0	0	0	0	1
0	0	0	0	0	1
0	0	0	0	0	1
1	0	0	0	0	1
1	0	0	0	0	1

0	0	0	0	0	1
0	0	0	0	0	1
0	0	0	0	0	1
0	0	0	0	0	1
0	0	0	0	0	1
0	0	0	0	0	1
0	0	0	0	0	1
0	0	0	0	0	1
0	0	0	0	0	1
0	0	0	0	0	1
0	0	0	0	0	1
1	0	0	0	0	1
0	0	0	0	0	1
0	0	0	0	0	1
0	0	0	0	0	1
1	0	0	0	0	1
1	0	0	0	0	1
0	0	0	0	0	1
0	0	0	0	0	1
0	0	0	0	0	1
0	0	0	0	0	1
1	0	0	0	0	1
0	0	0	0	0	1
0	0	0	0	0	1
1	0	0	0	0	1
0	0	0	0	0	1
0	0	0	0	0	1
0	0	0	0	0	1
1	0	0	0	0	1
0	0	0	0	0	1
0	0	0	0	0	1
0	0	0	0	0	1
1	0	0	0	0	1
0	0	0	0	0	1
0	0	0	0	0	1
1	0	0	0	0	1
0	0	0	0	0	1
1	0	0	0	0	1
0	0	0	0	0	1
0	0	0	0	0	1
0	0	0	0	0	1
0	0	0	0	0	1
0	0	0	0	0	1
0	0	0	0	0	1
1	0	0	0	0	1
0	0	0	0	0	1
0	0	0	0	0	1
0	0	0	0	0	1
0	0	0	0	0	1
1	0	0	0	0	1
0	0	0	0	0	1
0	0	0	0	0	1
1	0	0	0	0	1
0	0	0	0	0	1
0	0	0	0	0	1
0	0	0	0	0	1
1	0	0	0	0	1

0	0	0	0	0	1
0	0	0	0	0	1
0	0	0	0	0	1
0	0	0	0	0	1
0	0	0	0	0	1
1	0	0	0	0	1
1	0	0	0	0	1
1	0	0	0	0	1
1	0	0	0	0	1
0	0	0	0	0	1
0	0	0	0	0	1
1	0	0	0	0	1
0	0	0	0	0	1
0	0	0	0	0	1
0	0	0	0	0	1
0	0	0	0	0	1
0	0	0	0	0	1
0	0	0	0	0	1
0	0	0	0	0	1
0	0	0	0	0	1
0	0	0	0	0	1
0	0	0	0	0	1
0	0	0	0	0	1
0	0	0	0	0	1
0	0	0	0	0	1
0	0	0	0	0	1
0	0	0	0	0	0
0	0	0	0	0	0
0	0	0	0	0	1
0	0	0	0	0	1
1	0	0	0	0	1
0	0	0	0	0	1
0	0	0	0	0	1
1	0	0	0	0	1
1	0	0	0	0	1
1	0	0	0	0	1
0	0	0	0	0	1
0	0	0	0	0	1
0	0	0	0	0	1
0	0	0	0	0	1
1	0	0	0	0	1
0	0	0	0	0	1
0	0	0	0	0	0
0	0	0	0	0	1
0	0	0	0	0	1
1	0	0	0	0	1
1	0	0	0	0	1
1	0	0	0	0	1
1	0	0	0	0	1
1	0	0	0	0	1
0	0	0	0	0	1
0	0	0	0	0	1
1	0	0	0	0	1
0	0	0	0	0	1
0	0	0	0	0	1
0	0	0	0	0	0
1	0	0	0	0	1

1	0	0	0	0	1
1	0	0	0	0	1
1	0	0	0	0	1
1	0	0	0	0	1
1	0	0	0	0	1
1	0	0	0	0	1
1	0	0	0	0	1
1	0	0	0	0	1
1	0	0	0	0	1
1	0	0	0	0	1
1	0	0	0	0	1
1	0	0	0	0	1
1	0	0	0	0	1
1	0	0	0	0	1
1	0	0	0	0	1
1	0	0	0	0	1
1	0	0	0	0	1
1	0	0	0	0	1
1	0	0	0	0	1
0	0	0	0	0	1
0	0	0	0	0	1
1	0	0	0	0	1
0	0	0	0	0	1
0	0	0	0	0	1
0	0	0	0	0	0
0	0	0	0	0	0
0	0	0	0	0	1
0	0	0	0	0	1
1	0	0	0	0	1
0	0	0	0	0	0
0	0	0	0	0	0
1	0	0	0	0	0
1	0	0	0	0	0
0	0	0	0	0	1
0	0	0	0	0	1
0	0	0	0	0	1
0	0	0	0	0	1
0	0	0	0	0	1
0	0	0	0	0	1
0	0	0	0	0	1
0	0	0	0	0	1
0	0	0	0	0	1
0	0	0	0	0	1
0	0	0	0	0	1
0	0	0	0	0	1
0	0	0	0	0	1
0	0	0	0	0	1
0	0	0	0	0	1
0	0	0	0	0	1
0	0	0	0	0	1
0	0	0	0	0	1
0	0	0	0	0	1
0	0	0	0	0	1
0	0	0	0	0	1
0	0	0	0	0	1
0	0	0	0	0	1
0	0	0	0	0	1

0	0	0	0	0		1
0	0	0	0	0		1
0	0	0	0	0		1
0	0	0	0	0		1
0	0	0	0	0		1
0	0	0	0	0		1
0	0	0	0	0		1
0	0	0	0	0		1
0	0	0	0	0		1
0	0	0	0	0		1
0	0	0	0	0		1
0	0	0	0	0		1
0	0	0	0	0		1
0	0	0	0	0		1
0	0	0	0	0		1
0	0	0	0	0		1
0	0	0	0	0		1
0	0	0	0	0		1
0	0	0	0	0		1
0	0	0	0	0		1
0	0	0	0	0		1
0	0	0	0	0		1
0	0	0	0	0		1
0	0	0	0	0		1
0	0	0	0	0		1
0	0	0	0	0		1
0	0	0	0	0		1
0	0	0	0	0		1
0	0	0	0	0		1
0	0	0	0	0		1
0	0	0	0	0		1
0	0	0	0	0		1
0	0	0	0	0		1
0	0	0	0	0		1
0	0	0	0	0		1
0	0	0	0	0		1
0	0	0	0	0		1
0	0	0	0	0		1
0	0	0	0	0		1
0	0	0	0	0		1
0	0	0	0	0		1
0	0	0	0	0		1
0	0	0	0	0		1
0	0	0	0	0		1
0	0	0	0	0		1
0	0	0	0	0		1
0	0	0	0	0		1
0	0	0	0	0		1
0	0	0	0	0		1
0	0	0	0	0		1
0	0	0	0	0		1
0	0	0	0	0		1
0	0	0	0	0		1
0	0	0	0	0		1
0	0	0	0	0		1
0	0	0	0	0		1
0	0	0	0	0		1

0	0	0	0	0	1
0	0	0	0	0	1
0	0	0	0	0	1
0	0	0	0	0	1
0	0	0	0	0	1
0	0	0	0	0	1
0	0	0	0	0	1
0	0	0	0	0	1
0	0	0	0	0	1
0	0	0	0	0	1
0	0	0	0	0	1
0	0	0	0	0	1
0	0	0	0	0	1
0	0	0	0	0	1
0	0	0	0	0	1
0	0	0	0	0	1
0	0	0	0	0	1
0	0	0	0	0	1
0	0	0	0	0	1
0	0	0	0	0	0
1	0	0	0	0	1
0	0	0	0	0	1
1	0	0	0	0	1
0	0	0	0	0	1
1	0	0	0	0	1
0	0	0	0	0	1
1	0	0	0	0	1
0	0	0	0	0	1
1	0	0	0	0	1
0	0	0	0	0	1
0	0	0	0	0	0
1	0	0	0	0	1
0	0	0	0	0	1
1	0	0	0	0	1
0	0	0	0	0	1
1	0	0	0	0	1
1	0	0	0	0	1
0	0	0	0	0	1
0	0	0	0	0	1
0	0	0	0	0	1
1	0	0	0	0	1
1	0	0	0	0	1
1	0	0	0	0	1
0	0	0	0	0	1
1	0	0	0	0	1
1	0	0	0	0	1
0	0	0	0	0	1
1	0	0	0	0	1
1	0	0	0	0	1
1	0	0	0	0	1
1	0	0	0	0	1
1	0	0	0	0	1
0	0	0	0	0	0
1	0	0	0	0	1
1	0	0	0	0	1
1	0	0	0	0	1
1	0	0	0	0	1

1	0	0	0	0	1
1	0	0	0	0	1
1	0	0	0	0	1
1	0	0	0	0	1
1	0	0	0	0	1
1	0	0	0	0	1
1	0	0	0	0	1
1	0	0	0	0	1
1	0	0	0	0	1
1	0	0	0	0	1
1	0	0	0	0	1
1	0	0	0	0	1
1	0	0	0	0	1
1	0	0	0	0	1
1	0	0	0	0	1
1	0	0	0	0	1
1	0	0	0	0	1
1	0	0	0	0	1
1	0	0	0	0	1
1	0	0	0	0	1
1	0	0	0	0	1
1	0	0	0	0	1
1	0	0	0	0	1
0	0	0	0	0	1
1	0	0	0	0	1
1	0	0	0	0	1
1	0	0	0	0	1
1	0	0	0	0	1
1	0	0	0	0	1
1	0	0	0	0	1
1	0	0	0	0	1
1	0	0	0	0	1
0	0	0	0	0	1
0	0	0	0	0	1
0	0	0	0	0	1
1	0	0	0	0	1
1	0	0	0	0	1
1	0	0	0	0	1
0	0	0	0	0	0
0	0	0	0	0	1
0	0	0	0	0	0
0	0	0	0	0	0
0	0	0	0	0	1
0	0	0	0	0	1
0	0	0	0	0	1
1	0	0	0	0	1
1	0	0	0	0	1
0	0	0	0	0	1
0	0	0	0	0	1
0	0	0	0	0	0
0	0	0	0	0	1
1	0	0	0	0	1
0	0	0	0	0	1
0	0	0	0	0	0
1	0	0	0	0	1
0	0	0	0	0	0
0	0	0	0	0	1

0	0	0	0	0	1
1	0	0	0	0	0
0	0	0	0	0	1
0	0	0	0	0	1
0	0	0	0	0	0
0	0	0	0	0	0
0	0	0	0	0	0
0	0	0	0	0	0
0	0	0	0	0	0
0	0	0	0	0	0
0	0	0	0	0	0
0	0	0	0	0	0
0	0	0	0	0	0
0	0	0	0	0	0
0	0	0	0	0	0
0	0	0	0	0	0
0	0	0	0	0	0
0	0	0	0	0	0
0	0	0	0	0	0
0	0	0	0	0	0
0	0	0	0	0	0
0	0	0	0	0	0
0	0	0	0	0	0
0	0	0	0	0	0
0	0	0	0	0	0
0	0	0	0	0	0
0	0	0	0	0	0
0	0	0	0	0	0
0	0	0	0	0	0
0	0	0	0	0	0
0	0	0	0	0	0
0	0	0	0	0	0
0	0	0	0	0	0
0	0	0	0	0	0
0	0	0	0	0	0
0	0	0	0	0	0
0	0	0	0	0	0
0	0	0	0	0	0
0	0	0	0	0	0
0	0	0	0	0	0
0	0	0	0	0	0
0	0	0	0	0	0
0	0	0	0	0	0
0	0	0	0	0	1
0	0	0	0	0	0
0	0	0	0	0	0
0	0	0	0	0	0
0	0	0	0	0	0
0	0	0	0	0	0
0	0	0	0	0	0
0	0	0	0	0	0
0	0	0	0	0	0
0	0	0	0	0	0
0	0	0	0	0	0
0	0	0	0	0	0
0	0	0	0	0	0
0	0	0	0	0	0

0	0	0	0	0	0
0	0	0	0	0	1
0	0	0	0	0	0
0	0	0	0	0	0
0	0	0	0	0	0
0	0	0	0	0	0
0	0	0	0	0	0
0	0	0	0	0	0
0	0	0	0	0	0
0	0	0	0	0	0
0	0	0	0	0	0
0	0	0	0	0	0
0	0	0	0	0	0
0	0	0	0	0	0
0	0	1	0	0	1
0	1	0	0	0	0
1	1	0	0	0	1
0	0	0	0	0	1
0	0	0	0	0	1
0	1	0	0	0	1
0	0	0	1	0	1
0	0	1	0	0	1
1	0	0	0	0	0
1	1	0	0	0	1
0	1	0	0	0	1
0	1	0	0	0	1
0	1	0	0	0	1
0	1	0	0	0	1
0	0	1	0	1	1
1	1	0	0	0	1
1	1	0	0	0	1
0	0	1	0	0	1
0	1	0	0	0	1
0	1	0	0	0	1
0	1	0	0	0	1
1	1	0	0	0	1
1	1	0	0	0	1
0	1	0	0	0	1
1	1	0	0	0	1
0	1	0	0	0	1
0	0	1	0	0	1
1	1	0	0	0	1
0	1	0	0	0	1
1	1	0	0	0	1
1	0	1	0	0	1
0	1	0	0	0	0
0	0	1	0	0	1
0	1	0	0	0	1
0	1	0	1	0	1
0	0	0	0	0	1
0	1	0	0	0	1
1	0	0	0	0	1
0	1	0	0	0	1
0	0	0	0	0	1
1	1	0	1	0	1
1	0	1	0	0	0
0	0	0	0	1	1

0	0	1	0	0	1
0	1	0	0	0	1
0	1	0	0	1	1
1	1	0	0	0	1
1	0	1	0	0	1
1	1	0	1	0	1
0	1	0	0	0	1
1	1	0	0	0	1
0	1	0	0	0	1
0	1	0	0	0	1
0	1	0	0	0	1
0	1	0	0	0	1
0	0	0	0	0	1
0	0	0	0	0	1
0	1	0	0	0	0
1	1	0	0	0	1
0	0	1	0	0	1
0	1	0	0	0	1
1	1	0	0	0	1
0	1	0	0	0	1
1	1	0	0	0	0
0	1	0	0	0	1
1	1	0	0	0	1
0	1	0	0	0	1
0	1	0	0	0	1
0	1	0	0	0	1
1	1	0	0	0	1
0	0	1	1	0	1
0	0	0	0	0	0
0	0	0	0	0	0
0	0	0	0	0	1
0	0	0	0	0	0
0	0	0	0	0	1
1	1	0	0	0	0
0	0	1	0	0	1
1	1	0	0	0	1
1	1	0	0	0	1
0	0	0	0	0	1
1	1	0	0	0	1
0	1	0	0	0	1
0	0	1	0	0	1
0	1	0	0	0	1
0	0	0	0	0	1
1	1	0	0	0	1
1	1	0	0	0	1
1	0	0	0	0	0
1	1	0	0	0	1
1	0	1	0	0	1
0	1	0	0	0	1
1	1	0	0	0	1
0	1	0	0	0	1
0	1	0	0	0	1
1	1	0	0	0	1
1	0	1	0	0	1
0	1	0	0	0	1
1	1	0	0	0	1
1	0	1	0	0	1

0	1	0	0	1	1
0	1	0	0	0	1
1	0	1	0	0	0
0	0	1	0	0	1
0	0	0	0	0	0
0	1	0	0	0	1
0	1	0	0	0	1
1	0	0	0	0	1
1	1	0	0	0	1
1	1	0	0	0	1
1	1	0	0	0	1
0	1	0	1	0	1
0	1	0	0	0	1
0	1	0	0	0	1
1	1	0	0	0	1
1	0	1	0	0	1
1	0	0	0	0	1
1	1	0	1	0	1
1	1	0	0	0	1
0	0	0	0	1	1
0	0	1	0	0	0
1	0	1	0	0	0
0	1	0	0	0	1
1	1	0	0	0	1
1	0	0	0	0	1
0	1	0	0	0	1
1	0	0	0	0	1
0	0	0	0	0	1
1	1	0	0	0	1
0	1	0	0	0	0
0	0	0	0	0	0
0	0	1	0	0	1
0	1	0	0	0	1
0	1	0	0	0	1
1	1	0	0	0	1
0	0	1	0	0	0
0	1	0	0	0	1
0	0	0	0	0	1
0	0	0	0	0	0
1	0	0	0	0	1
1	0	1	0	0	1
0	1	0	1	0	1
0	0	1	0	0	1
0	0	0	0	0	1
1	0	1	0	0	1
1	1	0	0	1	1
0	1	0	0	0	1
1	1	0	0	0	1
1	0	0	0	0	0
1	1	0	0	0	1
0	0	1	0	0	1
0	0	1	0	0	1
0	0	1	0	1	1
1	1	0	0	0	1
0	1	0	0	0	1
0	0	1	0	0	1
0	0	1	0	0	1

0	0	0	0	1	1
1	1	0	0	0	1
0	1	0	0	0	1
0	0	0	0	0	1
1	1	0	0	0	1
0	0	1	0	0	1
1	1	0	0	0	1
0	1	0	1	0	1
0	1	0	0	0	1
1	0	1	0	0	1
0	1	0	0	0	1
1	0	0	0	0	1
0	1	0	0	0	0
0	1	0	0	0	1
0	1	0	0	0	1
1	0	0	0	0	0
0	1	0	0	1	1
0	1	0	1	0	1
0	0	0	0	0	0
1	0	1	0	0	0
0	0	1	0	0	1
1	1	0	0	0	1
0	0	0	0	0	1
0	0	0	0	1	1
1	1	0	0	0	1
0	1	0	0	0	1
0	0	1	0	0	1
0	0	1	0	1	1
1	1	0	0	0	0
0	1	0	0	0	1
0	0	1	0	0	1
0	0	1	0	0	1
1	1	0	0	0	0
0	0	0	0	0	1
1	1	0	0	0	1
1	1	0	0	0	1
0	0	1	0	0	1
1	1	0	0	0	1
0	1	0	0	0	1
1	0	1	0	0	1
0	0	0	0	0	0
1	1	0	0	1	1
0	1	0	0	0	1
0	0	1	0	0	1
0	1	0	0	0	1
0	1	0	0	0	1
1	1	0	0	0	1
1	1	0	0	0	1
0	1	0	0	0	1
0	1	0	0	0	1
0	1	0	0	0	1
0	1	0	0	0	1
0	0	1	0	0	1
1	1	0	0	0	0
0	1	0	0	0	1
0	0	0	0	0	1
0	0	1	0	0	0

1	1	0	0	0	1
0	1	0	0	0	1
0	1	0	0	0	1
0	1	0	0	0	1
1	1	0	0	0	1
0	1	0	0	0	1
1	1	0	0	0	1
0	1	0	0	0	1
1	1	0	0	0	1
1	0	0	0	0	1
0	1	0	1	0	1
0	0	0	0	0	1
0	0	0	0	0	1
1	1	0	0	0	1
0	0	1	0	1	1
0	1	0	0	0	0
0	0	1	0	0	0
0	0	0	0	0	0
1	0	0	0	0	1
0	0	1	0	0	1
0	0	1	0	0	1
0	0	1	0	0	1
0	1	0	0	0	1
0	0	0	0	0	1
0	0	0	0	1	1
0	0	1	0	1	1
0	0	0	1	0	1
0	0	0	0	1	1
0	1	0	0	0	1
1	0	0	0	0	1
0	0	1	0	0	1
1	0	0	0	0	0
0	1	0	0	0	1
0	1	0	0	0	1
0	0	0	0	0	1
0	0	0	0	0	1
0	0	1	0	0	1
0	0	0	0	0	1
0	0	0	0	0	1
0	1	0	1	0	1
0	1	0	0	0	1
1	1	0	0	0	1
1	1	0	0	0	1
0	0	0	0	0	1
1	0	0	0	0	0
0	0	1	0	0	0
0	0	0	0	0	0
0	0	1	0	0	0
1	1	0	0	0	1
0	0	0	0	0	1
0	0	1	0	0	1
1	1	0	0	0	1
0	1	0	0	0	1
1	0	1	0	0	1
1	1	0	0	0	0
0	0	1	0	0	1
0	1	0	0	0	1

1	0	0	0	0	1
0	0	0	0	0	1
1	1	0	0	0	1
0	0	1	0	0	1
1	0	0	0	0	1
1	1	0	0	0	1
1	1	0	0	0	1
0	0	1	0	0	1
0	0	0	0	0	1
0	1	0	1	0	1
1	1	0	0	0	1
0	1	0	0	0	1
0	1	0	0	0	1
1	0	0	1	0	1
0	1	0	0	0	1
0	1	0	0	0	1
0	0	1	0	0	1
0	0	1	0	0	1
1	1	0	0	0	1
0	1	0	0	0	1
0	1	0	0	0	1
0	1	0	0	0	1
1	1	0	0	0	1
0	1	0	0	0	1
1	0	1	0	0	1
0	0	1	0	0	1
0	0	1	0	0	1
0	0	1	1	0	1
1	0	1	0	0	1
1	1	0	0	0	1
1	0	1	0	0	0
0	0	1	0	0	1
1	1	0	0	0	1
1	1	0	0	0	1
0	0	1	0	0	1
0	1	0	0	0	1
1	0	1	0	0	1
1	0	0	0	0	1
0	1	0	0	0	1
1	0	1	0	0	1
0	1	0	0	0	1
1	1	0	0	0	1
0	1	0	0	0	1
0	1	0	0	0	1
0	1	0	0	0	1
0	1	0	0	0	1
0	1	0	0	0	1
0	0	1	0	0	0
1	0	1	0	0	0
0	1	0	0	0	1
0	0	1	0	0	1
0	0	0	0	0	1
0	1	0	0	0	1
0	1	0	0	0	1
1	0	1	0	0	0
0	0	1	0	1	1
0	0	0	0	0	1

0	1	0	0	0	1
1	0	1	0	1	0
0	1	0	0	0	1
1	1	0	0	0	1
1	1	0	0	0	1
0	0	0	0	0	1
1	1	0	0	0	1
0	0	0	0	0	1
0	0	1	0	0	1
0	1	0	0	0	1
0	0	1	0	0	0
0	0	0	0	0	0
1	0	1	0	0	0
0	0	0	0	0	1
0	0	1	0	0	1
0	0	1	0	0	1
0	1	0	0	0	1
0	1	0	0	0	1
1	1	0	0	0	1
0	1	0	1	0	1
1	0	0	0	0	1
1	0	1	0	0	1
1	0	1	0	0	1
1	1	0	0	0	1
0	1	0	0	0	1
1	0	0	0	0	1
0	1	0	1	0	1
0	1	0	0	0	1
0	1	0	0	0	1
1	1	0	0	0	1
1	0	1	0	0	1
0	0	1	0	0	1
0	0	1	0	0	1
1	0	1	0	0	1
0	1	0	1	0	1
1	0	0	0	0	1
1	0	0	0	0	1
1	1	0	0	0	1
0	0	0	0	1	1
0	1	0	0	0	0
0	1	0	0	0	1
0	0	1	0	0	1
1	1	0	0	0	1
0	1	0	0	0	1
0	0	1	0	0	1
0	0	1	0	0	1
1	1	0	0	0	1
1	1	0	0	0	1
1	1	0	0	0	1
0	1	0	0	0	1
1	1	0	1	0	1
1	1	0	0	0	1
0	0	0	0	0	1
1	1	0	0	0	1
1	0	0	0	0	1
0	1	0	0	0	1
0	0	0	0	0	1

0	0	1	0	1	1
0	0	1	0	1	1
0	1	0	0	0	1
1	1	0	0	0	1
1	0	1	0	0	1
0	1	0	0	0	1
0	0	0	0	0	0
0	0	0	0	1	1
0	1	0	0	0	1
0	1	0	0	0	1
0	0	0	0	0	1
0	0	1	0	0	1
0	1	0	0	0	0
0	1	0	0	0	1
0	0	0	0	0	1
0	0	1	0	0	1
0	0	1	0	0	0
0	1	0	1	0	1
1	1	0	0	0	1
0	1	0	0	0	0
1	1	0	0	0	1
0	0	0	0	0	1
0	0	0	0	0	1
0	0	0	0	0	1
0	0	0	0	0	1
0	0	1	0	0	1
0	1	0	0	0	0
0	1	0	0	0	1
0	1	0	0	0	1
1	1	0	0	0	1
1	0	0	0	1	1
0	0	1	0	1	1
0	0	0	0	0	1
0	0	1	0	0	1
1	0	1	0	0	1
1	1	0	0	0	1
0	0	1	0	1	1
0	1	0	0	0	1
1	1	0	0	0	1
1	1	0	0	1	1
0	0	1	0	0	1
0	0	1	0	0	1
0	0	1	0	0	1
1	0	1	0	0	1
1	0	1	1	0	1
0	0	0	0	0	1
0	1	0	0	0	1
0	1	0	0	0	1
0	0	0	1	0	1
0	0	1	0	0	0
0	0	1	0	0	0
0	1	0	0	0	1
0	0	0	0	0	1
0	0	1	0	0	1
0	0	1	0	0	1
0	1	0	0	0	1
0	0	1	0	0	0

0	0	0	0	0	1
0	0	0	0	0	1
0	1	0	0	0	1
0	1	0	0	0	1
0	1	0	0	0	1
0	1	0	0	0	1
0	0	0	0	0	1
1	1	0	0	0	1
1	1	0	0	0	1
0	1	0	0	0	1
0	1	0	1	0	1
0	0	1	0	0	1
0	0	0	0	0	1
0	1	0	0	0	1
0	0	1	0	0	1
1	0	0	0	0	0
0	0	0	0	0	0
0	1	0	0	0	1
0	0	0	0	0	1
1	0	0	0	0	0
0	0	1	0	0	1
0	0	0	0	0	1
0	0	1	0	0	1
0	0	1	0	0	0
0	1	0	0	0	1
0	0	0	0	0	1
1	0	1	0	1	1
1	1	0	0	0	1
1	1	0	0	0	1
0	1	0	0	0	1
1	1	0	0	0	1
1	0	1	0	0	1
1	0	1	0	1	1
0	0	0	0	0	0
0	0	0	0	0	1
0	0	0	0	0	1
0	0	1	0	0	1
1	0	0	0	0	0
0	1	0	0	0	1
1	0	0	0	0	1
1	0	0	0	0	1
1	0	1	0	1	1
0	0	1	0	0	1
0	1	0	0	0	1
0	0	0	0	0	0
0	0	0	0	0	0
0	1	0	0	0	0
0	0	1	0	0	0
0	0	1	0	0	0
0	0	0	0	0	0
0	0	0	0	0	0
1	1	0	0	1	1
1	1	0	0	0	1
0	1	0	1	0	1
0	1	0	0	0	1
0	0	1	0	0	1
0	1	0	0	0	0

1	0	1	0	0	1
0	0	1	0	0	1
0	1	0	0	0	1
1	0	0	0	0	1
0	1	0	0	1	1
0	1	0	0	0	1
1	1	0	0	1	1
0	0	0	1	0	1
0	1	0	0	0	1
0	1	0	0	0	1
0	0	1	0	0	0
0	0	0	0	0	1
0	1	0	0	0	1
0	1	0	0	0	1
0	0	1	0	0	1
0	0	0	0	0	1
0	0	1	0	0	1
0	0	0	0	0	1
0	0	0	0	0	1
0	1	0	0	0	1
0	1	0	0	0	1
0	0	0	0	0	0
1	0	1	0	0	1
0	1	0	0	0	1
0	1	0	0	0	1
0	0	0	0	0	1
0	0	1	0	0	1
0	0	0	0	0	1
0	1	0	0	0	1
1	1	0	0	0	1
0	1	0	0	0	1
0	1	0	0	0	1
1	0	1	0	0	1
0	1	0	0	0	1
0	1	0	0	0	1
1	1	0	0	0	1
0	1	0	0	1	1
0	0	0	0	0	1
0	1	0	0	0	1
0	1	0	0	0	1
1	1	0	0	0	1
1	0	1	0	0	1
0	1	0	0	0	1
1	1	0	0	0	1
1	1	0	0	0	1
1	0	0	0	0	1
0	0	0	0	0	1
0	0	0	0	0	1
0	1	0	0	0	1
0	0	1	0	1	1
1	1	0	0	0	1
0	1	0	0	0	1
0	1	0	0	0	1
0	0	0	0	0	1
1	1	0	0	0	1
0	1	0	0	0	1
0	1	0	0	0	1

0	1	0	0	0	1
0	1	0	0	0	1
0	1	0	0	0	1
0	0	1	0	0	1
0	1	0	0	0	1
0	0	0	0	0	1
1	0	1	0	0	1
0	1	0	0	0	1
1	0	1	0	0	0
1	1	0	0	0	1
1	1	0	0	0	1
0	0	1	0	0	0
0	0	0	0	0	1
1	1	0	0	0	1
0	1	0	0	0	1
1	1	0	0	0	1
1	1	0	0	0	0
0	1	0	0	0	1
1	1	0	0	0	0
0	0	0	0	0	1
0	1	0	0	0	1
0	1	0	0	0	0
0	0	1	0	0	1
0	1	0	0	0	1
0	1	0	0	0	1
0	0	1	0	0	0
0	1	0	0	0	1
0	1	0	0	0	1
1	1	0	0	0	1
1	0	0	0	0	0
0	0	0	0	0	1
0	0	0	0	0	0
0	0	0	0	0	1
0	1	0	0	0	1
1	0	1	0	0	1
0	0	1	0	0	1
0	0	1	0	0	1
0	1	0	0	0	1
0	1	0	0	0	1
0	0	1	0	0	0
0	0	1	0	0	1
0	0	1	0	0	1
0	0	0	0	0	1
0	1	0	0	0	1
1	1	0	0	0	1
0	0	0	0	0	1
0	1	0	0	0	1
0	1	0	0	0	1
0	0	1	0	1	1
0	1	0	0	0	1
0	1	0	0	0	1
1	0	0	0	0	0
0	1	0	0	0	1
0	1	0	0	0	1
0	1	0	0	0	1
1	1	0	0	0	1
1	0	1	0	0	1

1	1	0	0	0	1
1	0	1	1	0	1
0	1	0	0	0	1
0	0	0	0	0	1
0	0	1	0	0	0
1	0	1	0	0	1
0	1	0	0	0	1
0	1	0	0	0	1
0	1	0	0	0	1
0	1	0	0	0	1
0	1	0	1	0	1
0	1	0	1	0	1
0	1	0	0	0	1
0	1	0	0	0	1
0	0	0	0	0	1
0	1	0	0	0	1
0	1	0	0	0	1
0	1	0	0	0	1
1	1	0	0	0	1
0	0	0	0	0	1
0	1	0	0	0	1
0	1	0	0	0	1
1	1	0	0	0	1
1	1	0	0	0	1
0	1	0	0	0	1
1	1	0	0	0	1
1	0	0	0	0	1
0	1	0	0	0	1
0	0	0	0	0	1
0	1	0	0	0	1
0	0	0	0	0	0
0	0	1	0	0	1
0	0	1	0	0	1
0	1	0	0	0	1
1	0	0	0	0	0
0	0	1	0	0	1
0	0	1	0	0	0
1	0	0	0	0	0
1	0	0	0	0	1
1	1	0	0	0	1
0	0	0	0	0	1
0	1	0	0	0	1
0	1	0	0	0	1
0	1	0	0	0	1
1	1	0	0	0	1
1	1	0	0	0	1
0	0	1	0	0	1
0	0	1	0	0	1
0	0	0	0	0	1
0	1	0	0	0	1
0	0	1	0	0	1
0	0	1	0	1	1
0	1	0	0	0	1
0	0	0	0	0	1
0	0	1	0	0	1
1	1	0	0	0	1
0	0	1	0	0	1

0	1	0	0	0	1
0	1	0	0	0	1
0	0	1	0	0	1
1	1	0	0	0	1
1	1	0	0	0	1
0	1	0	0	0	1
1	1	0	0	0	1
0	0	1	0	0	1
1	1	0	0	0	1
1	1	0	0	0	1
0	1	0	0	0	1
1	0	0	0	0	1
0	1	0	0	0	1
1	0	0	0	0	1
1	1	0	0	0	1
0	0	1	0	0	1
0	1	0	0	0	1
0	1	0	0	0	1
0	0	0	0	0	1
1	0	0	0	0	1
1	1	0	0	0	1
1	0	0	0	0	1
0	0	0	0	0	1
1	1	0	0	0	1
1	1	0	0	0	1
0	0	1	1	0	1
1	1	0	0	0	1
0	0	1	0	0	1
0	1	0	0	0	1
0	0	0	0	0	1
1	1	0	0	0	1
1	0	0	0	1	1
0	0	1	0	0	1
0	1	0	0	0	1
0	0	1	0	0	0
1	1	0	0	0	1
0	0	1	0	0	0
1	1	0	0	0	1
0	1	0	0	0	0
1	1	0	0	0	1
1	0	1	0	0	0
1	1	0	0	0	1
0	0	0	0	0	1
1	0	1	0	0	0
0	0	0	0	0	1
1	0	0	0	0	1
0	0	1	0	0	1
0	0	0	0	0	1
0	1	0	0	0	1
1	1	0	0	0	1
1	0	1	0	0	1
1	1	0	0	0	1
0	0	0	0	0	1
0	1	0	0	0	1
1	0	1	0	0	1
1	1	0	0	0	1
0	0	0	0	0	1

0	0	0	0	0	1
0	0	0	0	0	1
0	0	0	0	0	1
0	0	1	0	0	1
0	0	1	0	0	1
1	0	1	0	0	0
0	0	1	1	0	1
0	1	0	0	0	1
1	0	0	0	0	1
0	0	1	0	0	0
0	1	0	0	0	1
0	1	0	0	0	1
0	0	0	0	0	0
1	1	0	0	0	1
0	1	0	0	0	1
0	1	0	0	0	1
1	1	0	0	0	1
0	1	0	0	0	1
0	1	0	0	0	1
1	0	0	0	0	1
0	0	1	0	0	0
0	0	0	0	0	0
0	0	0	0	0	0
0	1	0	0	0	1
1	0	1	0	0	0
0	0	0	0	0	0
1	1	0	0	0	1
0	1	0	0	0	1
0	0	1	0	0	1
0	1	0	0	0	1
0	1	0	1	0	1
0	1	0	0	0	0
1	0	1	0	0	1
0	0	1	1	0	1
0	1	0	0	0	1
1	1	0	0	0	1
0	1	0	0	0	1
1	0	1	0	0	1
0	1	0	0	0	1
0	1	0	0	0	1
1	0	1	0	0	1
1	0	1	0	0	1
0	1	0	0	0	1
1	1	0	0	0	1
1	1	0	0	0	1
0	1	0	0	0	1
0	1	0	0	0	1
1	1	0	0	0	1
0	0	0	0	0	1
0	1	0	0	0	1
0	0	0	1	0	1
0	0	0	0	0	0
1	1	0	0	0	1
0	1	0	1	0	1
1	1	0	0	0	1
0	0	1	0	0	0
0	1	0	0	0	1

0	1	0	0	0	1
1	0	1	0	0	0
1	1	0	0	0	0
0	0	0	0	0	1
1	1	0	0	0	1
0	1	0	0	0	1
0	0	1	0	0	1
0	0	0	0	0	1
1	0	0	0	0	0
1	1	0	0	0	1
1	1	0	0	0	1
0	1	0	0	0	1
1	1	0	0	0	1
0	1	0	0	0	1
0	0	1	0	0	1
1	0	0	0	0	1
1	0	0	0	0	0
1	1	0	0	0	1
0	1	0	0	0	1
1	1	0	0	0	1
1	0	0	0	0	1
0	0	0	0	0	0
0	0	0	0	0	0
0	0	0	0	0	0
0	0	0	0	0	0
0	0	0	0	0	0
0	0	0	0	0	0
0	0	0	0	0	0
0	0	0	0	0	0
0	0	0	0	0	0
0	0	0	0	0	0
0	0	0	0	0	0
0	0	0	0	0	0
0	0	0	0	0	0
0	0	0	0	0	0
0	0	0	0	0	0
0	0	0	0	0	1
0	0	0	0	0	1
0	0	0	0	0	1
0	0	0	0	0	1
0	0	0	0	0	1
0	0	0	0	0	1
0	0	0	0	0	1
0	0	0	0	0	1
0	0	0	0	0	1
0	0	0	0	0	1
0	0	0	0	0	1
0	0	0	0	0	1
0	0	0	0	0	1
0	0	0	0	0	1
0	0	0	0	0	1
0	0	0	0	0	1
0	0	0	0	0	1
0	0	0	0	0	1
0	0	0	0	0	1
0	0	0	0	0	1
0	0	0	0	0	1

0	0	0	0	0	1
0	1	0	0	0	1
1	1	0	0	0	1
0	0	1	0	0	1
1	1	0	0	0	1
0	1	0	0	0	1
0	1	0	0	0	1
1	1	0	0	0	1
0	0	0	0	0	1
1	1	0	0	0	1
1	0	1	0	0	1
0	0	1	0	0	1
0	1	0	0	0	1
0	0	0	0	0	1
0	0	1	0	0	0
1	0	1	0	0	0
1	0	1	0	0	0
0	0	0	0	0	0
0	0	1	0	0	1
0	1	0	0	0	0
0	0	1	1	0	1
0	1	0	0	0	1
1	1	0	0	0	1
1	0	0	0	0	1
0	0	0	0	0	1
1	0	1	0	0	1
1	0	1	0	0	1
0	1	0	0	0	1
1	0	1	0	0	1
1	1	0	0	0	1
1	0	0	0	0	1
1	0	1	0	0	1
0	1	0	0	0	1
0	1	0	0	0	1
0	0	1	0	0	1
1	1	0	1	0	1
1	1	0	0	0	1
1	1	0	0	0	1
1	1	0	0	0	1
1	1	0	0	0	1
0	1	0	0	0	1
0	0	1	0	0	1
1	1	0	0	0	1
0	1	0	0	0	1
1	0	0	0	0	0
0	1	0	0	0	1
1	1	0	0	0	1
1	0	1	1	0	1
0	1	0	0	0	1
0	0	1	0	0	1
1	0	0	0	0	1
1	0	1	0	0	1
0	0	1	0	0	1
1	1	0	0	0	1
0	0	1	0	0	1
0	0	1	0	0	0
0	1	0	0	0	1

0	0	0	0	0	0
0	0	0	0	0	0
0	0	0	0	0	0
1	0	0	0	0	1
0	1	0	0	0	0
0	0	0	0	0	0
0	0	0	0	0	0
1	1	0	0	0	1
1	0	1	0	0	0
1	0	0	0	0	1
1	1	0	1	0	1
0	1	0	0	0	1
0	1	0	0	0	1
1	0	1	0	0	1
0	1	0	0	0	1
0	0	1	0	0	1
0	1	0	0	0	1
1	0	0	0	0	1
0	0	0	0	0	0
0	1	0	0	0	1
0	1	0	0	0	1
0	1	0	0	0	1
0	0	1	0	0	1
0	1	0	0	0	1
0	1	0	0	0	1
0	1	0	0	0	0
0	0	1	0	0	1
1	0	1	0	0	1
0	0	1	0	0	0
0	0	1	0	0	1
1	1	0	0	0	1
0	0	1	0	0	0
1	1	0	0	0	1
1	1	0	0	0	1
0	1	0	0	0	1
0	1	0	0	0	1
1	1	0	1	0	1
0	0	1	0	0	1
1	0	1	0	0	1
1	0	1	0	0	1
1	1	0	0	0	1
1	1	0	0	0	1
0	1	0	0	0	1
0	1	0	0	0	1
1	1	0	0	0	1
0	1	0	0	0	1
0	0	1	0	0	1
0	0	0	0	0	1
0	0	1	0	0	0
1	0	0	0	0	1
0	1	0	0	0	1
0	1	0	0	0	1
1	1	0	0	0	0
1	1	0	0	0	1
0	1	0	0	0	1
0	0	1	0	0	1
1	1	0	0	0	1

0	0	0	0	0	1
1	0	0	0	0	1
1	1	0	0	0	1
0	1	0	0	0	1
1	0	0	0	0	0
0	0	0	0	0	1
1	0	1	0	0	1
1	1	0	0	0	1
0	1	0	0	0	1
1	1	0	0	0	1
0	1	0	0	0	1
1	1	0	0	0	1
0	0	0	0	0	1
0	0	0	0	0	1
0	1	0	1	0	1
1	0	0	0	0	1
0	0	1	0	0	1
0	1	0	0	0	1
0	0	1	0	0	1
1	0	1	0	0	0
0	0	1	0	0	1
1	0	1	0	0	1
1	0	1	0	0	1
0	0	1	0	0	0
0	0	0	0	0	0
1	0	0	0	0	1
1	1	0	0	0	1
1	0	1	0	0	1
0	0	0	1	0	1
0	1	0	1	0	1
0	0	0	0	0	1
0	0	0	0	0	1
0	1	0	0	0	1
0	0	0	0	0	1
1	0	0	0	0	1
1	1	0	0	0	1
0	0	1	0	0	1
0	1	0	0	0	1
1	0	0	0	0	0
0	0	1	1	0	1
0	1	0	0	0	1
0	0	1	0	1	1
1	1	0	0	0	1
0	1	0	0	0	1
1	0	0	0	0	1
0	1	0	0	0	1
0	0	1	0	0	1
0	1	0	0	0	1
0	1	0	1	0	1
0	0	1	0	0	1
1	0	1	0	0	1
0	1	0	0	0	1
1	1	0	0	0	1
0	1	0	0	0	1
0	0	1	0	0	1
0	1	0	0	0	1
0	1	0	0	0	1

0	0	1	0	0	1
0	0	0	0	0	0
1	1	0	0	0	1
0	1	0	0	0	1
0	0	0	0	0	1
1	0	1	0	0	1
0	1	0	0	0	1
0	1	0	0	0	1
1	1	0	0	0	1
0	0	1	0	0	1
1	1	0	0	0	1
0	0	1	0	0	1
1	0	0	0	0	1
0	1	0	0	0	1
0	1	0	0	0	1
1	1	0	1	0	1
1	1	0	0	0	1
1	0	0	0	0	1
0	0	1	0	0	1
0	1	0	1	0	1
0	0	0	0	0	1
0	1	0	1	0	1
0	1	0	0	0	1
0	1	0	0	0	1
1	0	0	0	0	0
1	1	0	0	0	1
0	0	0	0	0	1
0	1	0	0	0	1
0	1	0	0	0	1
0	0	1	0	0	0
0	1	0	0	0	1
1	1	0	0	0	1
1	1	0	0	0	1
0	1	0	0	0	1
1	1	0	0	0	1
0	1	0	0	0	1
0	1	0	0	1	1
1	1	0	0	0	1
0	0	1	0	0	1
1	1	0	0	0	1
0	1	0	0	0	1
0	1	0	0	0	1
1	0	1	0	1	1
1	1	0	0	0	1
0	0	0	0	0	1
0	0	1	0	0	0
1	0	0	0	0	0
1	0	0	0	0	1
0	1	0	0	0	1
1	0	0	0	0	0
1	0	0	0	0	0
0	1	0	0	0	1
0	1	0	0	0	1
0	1	0	0	0	1
0	1	0	1	0	1
0	1	0	0	0	1
0	1	0	0	0	1

1	1	0	1	0	1
0	0	1	0	0	1
0	0	0	0	0	0
1	1	0	0	0	1
0	1	0	0	0	1
0	0	1	0	0	0
0	0	1	0	0	0
0	1	0	1	0	1
0	1	0	0	0	1
0	0	1	0	0	1
0	1	0	0	0	1
0	1	0	0	0	1
1	1	0	0	0	1
1	0	0	0	0	1
1	1	0	0	0	1
0	1	0	0	0	1
0	0	0	0	0	1
1	1	0	0	0	1
1	1	0	0	0	1
0	0	0	0	0	0
0	0	1	0	0	0
0	1	0	0	0	1
0	0	0	0	0	0
0	1	0	0	0	1
1	0	1	0	0	1
1	1	0	0	0	1
0	1	0	0	0	1
0	1	0	0	0	1
1	0	1	0	0	1
0	0	1	0	0	0
1	1	0	0	0	1
1	1	0	0	0	1
0	1	0	0	0	1
1	0	1	0	0	1
0	0	0	0	0	1
1	0	1	0	0	1
1	0	0	0	0	1
0	1	0	0	0	1
0	0	1	0	0	1
0	0	1	0	0	1
0	0	1	0	0	1
0	1	0	0	0	1
0	0	1	0	0	0
1	1	0	0	0	1
0	0	0	0	0	1
0	0	0	0	1	1
0	1	0	0	0	1
1	1	0	0	0	1
0	0	0	0	0	1
1	1	0	0	0	1
0	0	0	0	0	1
0	0	1	0	0	0
0	0	1	0	0	1
0	0	0	0	0	1
0	0	0	0	0	1
1	0	1	0	0	1
1	0	1	0	0	1

0	1	0	0	0	1
1	1	0	0	0	1
0	1	0	0	0	1
1	0	0	0	0	1
1	0	1	0	0	0
0	1	0	0	0	1
0	1	0	0	0	1
1	1	0	1	0	1
1	0	1	0	0	1
0	0	1	0	0	1
0	0	1	0	0	1
0	1	0	0	0	1
1	1	0	0	0	1
1	0	0	0	0	0
0	1	0	0	0	1
0	1	0	0	0	1
0	0	1	0	0	1
0	0	1	0	0	1
1	0	0	0	0	1
1	1	0	0	0	1
1	1	0	0	0	1
0	0	0	0	0	1
1	1	0	0	0	1
0	1	0	0	0	1
0	0	0	0	0	1
0	0	1	0	0	1
0	0	1	0	0	0
0	0	0	0	0	1
0	0	0	0	0	1
0	1	0	0	0	1
0	0	1	0	0	1
0	1	0	0	0	1
0	0	1	0	0	1
0	1	0	1	0	1
0	1	0	0	0	1
0	0	1	0	0	1
0	0	1	0	0	1
0	1	0	0	0	0
0	0	1	0	0	1
1	1	0	0	0	1
0	0	0	0	0	1
0	0	0	0	0	0
1	1	0	0	0	1
0	0	1	0	0	1
1	1	0	0	0	1
0	1	0	0	0	1
0	0	0	0	0	1
0	1	0	0	0	1
0	1	0	0	0	1
0	1	0	0	0	1
0	1	0	0	0	1
1	0	1	0	0	1
0	0	0	0	0	1
0	1	0	0	0	1
0	0	1	0	0	1
1	1	0	0	0	1
0	0	1	0	0	1

0	0	1	0	0	1
0	0	0	0	0	1
0	0	0	0	1	1
1	0	0	0	0	1
0	1	0	0	0	1
0	1	0	0	0	1
0	0	1	0	0	1
0	0	0	0	0	1
0	1	0	0	0	1
0	0	1	0	0	1
0	1	0	0	0	1
1	1	0	0	0	1
1	0	1	1	0	1
1	0	1	0	0	1
0	1	0	0	0	1
0	1	0	0	0	1
0	0	0	0	0	1
1	1	0	0	0	1
1	1	0	0	0	1
0	0	1	0	0	1
1	1	0	0	0	1
0	1	0	0	0	1
0	1	0	0	0	1
0	1	0	0	0	1
1	1	0	0	0	1
0	1	0	0	0	1
0	1	0	0	0	1
1	1	0	0	0	1
1	1	0	0	0	1
0	1	0	0	0	1
0	1	0	0	0	1
0	1	0	1	0	1
1	0	1	0	0	1
0	0	1	1	0	1
1	1	0	0	0	0
1	1	0	0	0	1
0	0	1	0	0	0
0	0	1	0	0	1
1	0	1	0	0	1
1	0	0	0	0	0
1	0	0	0	0	1
1	1	0	0	0	1
0	1	0	0	0	1
1	1	0	0	0	1
0	1	0	0	0	1
0	1	0	0	0	1
1	1	0	0	0	1
1	1	0	0	0	1
0	1	0	0	0	1
1	0	1	0	0	1
1	1	0	0	0	1
0	1	0	0	0	1
0	0	0	0	0	0
0	0	1	0	0	1
0	1	0	0	0	1
0	1	0	0	0	1
0	1	0	0	0	1

1	1	0	1	0	1
1	1	0	0	0	1
0	1	0	0	0	1
0	0	1	0	1	1
0	1	0	0	0	1
1	0	0	0	0	1
0	0	0	0	0	1
0	1	0	0	0	1
0	1	0	0	0	0
0	0	1	0	0	0
1	0	1	0	0	0
0	0	1	0	0	1
1	1	0	1	0	1
1	0	0	0	0	1
1	1	0	0	0	1
1	0	1	0	0	1
1	1	0	0	0	1
0	1	0	0	0	1
1	0	1	0	0	1
0	0	0	0	0	1
0	0	1	0	0	1
0	1	0	0	0	1
0	0	1	0	0	1
0	1	0	0	0	1
0	1	0	0	0	1
0	1	0	0	0	1
1	0	0	0	0	1
1	1	0	0	0	1
1	0	1	0	0	1
0	1	0	0	0	1
0	0	1	0	0	1
1	0	1	0	0	1
0	0	1	0	0	1
1	0	1	0	0	0
0	0	1	0	1	1
0	0	0	0	0	1
0	1	0	0	0	1
1	0	0	0	0	1
0	1	0	0	0	1
0	0	1	0	0	1
0	0	1	0	0	1
1	0	0	0	0	1
0	1	0	0	0	1
0	1	0	1	0	1
0	0	0	0	0	1
1	1	0	0	0	1
0	1	0	0	0	1
0	0	1	0	0	1
1	0	1	0	0	1
1	1	0	0	0	1
0	1	0	0	0	1
1	0	0	0	0	1
0	1	0	0	0	1
1	1	0	0	0	1
1	1	0	0	0	1
1	1	0	0	0	1
1	0	1	0	0	1

1	0	1	0	0	1
0	1	0	0	0	1
0	1	0	0	0	1
0	1	0	0	0	1
0	1	0	0	0	1
1	1	0	0	0	1
1	1	0	0	0	1
1	1	0	0	0	1
0	1	0	0	0	1
0	1	0	0	0	1
0	1	0	0	0	1
1	0	1	0	0	0
1	1	0	1	0	1
1	1	0	0	0	1
0	1	0	0	0	1
0	1	0	0	0	1
1	1	0	0	0	1
0	0	1	0	0	1
1	0	1	1	0	1
1	0	1	0	0	1
0	0	1	0	0	1
0	0	1	0	0	1
1	0	1	0	0	1
1	1	0	0	0	1
1	0	1	0	0	1
1	1	0	1	0	1
0	0	0	0	0	1
0	0	0	0	0	0
1	0	0	0	0	0
0	1	0	0	0	0
1	1	0	0	0	1
1	1	0	0	0	1
0	1	0	0	0	1
1	0	1	0	0	1
1	0	1	0	0	1
1	1	0	0	0	1
0	0	0	0	0	1
1	1	0	0	0	1
0	1	0	0	0	1
1	0	1	0	0	1
1	1	0	0	0	1
0	0	1	0	0	1
0	0	0	0	0	0
0	1	0	0	0	1
1	1	0	0	0	1
0	1	0	0	0	1
0	1	0	0	0	1
0	1	0	0	0	1
0	1	0	0	0	0
0	0	1	0	0	1
0	0	1	0	0	1
0	1	0	0	0	0
0	1	0	0	0	1
1	0	0	0	0	0
0	0	0	0	0	1
0	0	0	0	0	1
1	0	1	1	0	0

0	0	1	0	0	1
1	1	0	0	0	1
0	1	0	0	0	1
1	0	1	0	0	1
0	1	0	0	0	1
1	1	0	0	0	1
0	0	0	0	0	1
0	1	0	0	0	1
1	0	0	0	0	1
0	0	1	0	0	1
1	0	1	0	0	1
0	0	1	0	0	1
1	0	1	0	0	0
1	1	0	0	0	1
0	1	0	0	0	1
1	0	1	0	0	1
1	0	1	0	0	1
1	1	0	0	0	1
1	0	1	0	0	1
0	0	1	0	0	1
1	0	1	0	0	1
0	1	0	0	0	1
0	1	0	0	0	1
0	1	0	0	0	1
0	1	0	0	0	1
1	1	0	0	0	1
1	1	0	1	0	1
1	1	0	0	0	1
1	1	0	0	0	1
0	0	0	0	0	0
0	0	1	0	0	1
0	0	0	0	0	0
0	1	0	0	0	1
0	1	0	0	0	1
0	1	0	0	0	1
0	0	1	0	0	1
1	0	1	0	0	0
0	1	0	0	0	1
1	0	0	0	0	1
0	0	1	0	0	1
0	0	1	0	0	1
0	1	0	0	0	1
0	1	0	0	0	1
1	1	0	0	0	1
0	1	0	0	0	1
0	0	0	0	0	1
0	1	0	0	0	1
0	1	0	0	0	1
0	1	0	0	0	1
0	1	0	0	0	1
0	1	0	0	0	1
0	1	0	0	0	1
1	1	0	1	0	1
0	1	0	0	0	1
1	1	0	0	0	1
1	0	1	0	0	1
0	1	0	0	0	1

0	0	1	1	0	1
1	0	1	0	0	1
0	0	1	0	0	1
0	0	0	0	0	1
0	1	0	0	0	1
0	0	0	0	0	1
0	0	1	0	0	1
1	1	0	0	0	1
0	1	0	0	0	1
1	1	0	0	0	1
1	0	1	0	0	1
0	1	0	0	0	1
0	1	0	0	0	1
0	1	0	0	0	1
0	1	0	0	0	1
1	1	0	0	0	1
1	1	0	0	1	1
1	1	0	0	1	0
1	0	0	1	0	0
0	0	0	0	0	1
0	0	1	0	0	1
0	1	0	0	0	1
1	1	0	0	0	1
0	1	0	1	0	1
0	1	0	1	0	1
1	1	0	0	0	1
0	0	1	0	0	1
0	0	1	0	0	0
1	1	0	0	0	1
0	1	0	0	0	1
0	1	0	0	0	1
0	0	1	0	0	1
0	1	0	0	0	1
0	1	0	1	0	1
0	1	0	0	0	1
0	0	0	0	0	1
1	1	0	0	0	1
0	0	0	0	0	1
1	1	0	0	0	1
1	0	0	0	0	0
0	1	0	0	0	1
1	1	0	0	0	1
1	0	0	0	0	0
0	1	0	0	0	1
1	0	0	0	0	1
1	1	0	0	0	1
0	1	0	0	0	1
0	0	1	0	0	0
1	1	0	0	0	1
0	0	0	0	0	1
0	1	0	0	0	1
0	1	0	0	0	1
0	0	1	0	0	1
0	1	0	0	0	1
0	0	1	0	0	1
1	0	1	0	1	1
1	0	1	0	0	1

1	1	0	0	0	1
0	1	0	0	0	1
1	1	0	0	0	1
1	1	0	0	0	1
0	0	1	0	0	1
1	1	0	0	0	1
0	1	0	0	0	1
1	1	0	0	0	1
1	0	0	0	0	0
1	0	0	0	0	1
0	0	0	0	0	0
0	0	0	0	0	0
0	0	0	0	0	1
1	1	0	0	0	1
0	1	0	0	0	1
0	1	0	0	0	1
1	0	1	0	0	1
1	0	1	0	0	1
0	1	0	0	0	1
0	1	0	0	0	1
0	0	1	0	0	1
0	1	0	0	0	1
1	1	0	0	0	1
1	1	0	0	0	1
0	0	1	0	0	1
0	0	1	0	0	1
0	0	1	0	0	1
0	0	1	0	0	0
1	1	0	0	0	1
0	1	0	0	0	1
1	0	0	0	0	1
0	1	0	0	0	1
0	1	0	0	0	1
1	1	0	0	0	1
1	0	0	0	0	1
1	0	1	0	0	1
0	0	1	0	0	1
1	1	0	0	0	1
0	0	0	1	0	1
1	0	0	0	0	1
1	1	0	0	0	1
0	0	0	0	0	1
0	1	0	0	0	1
0	1	0	0	0	1
1	1	0	0	0	1
1	1	0	0	0	1
1	1	0	0	0	1
0	1	0	0	0	1
0	1	0	0	0	1
0	1	0	0	0	1
1	0	0	1	0	1
0	0	1	0	0	1
1	1	0	0	0	0
1	0	0	0	0	1
1	1	0	0	0	1
0	0	0	1	0	1
1	0	1	0	0	1

1	1	0	0	0	1
1	0	0	0	0	0
0	1	0	0	0	1
0	0	0	0	0	0
0	1	0	0	0	1
0	1	0	0	0	1
0	1	0	0	0	1
1	0	1	0	0	1
1	0	1	0	0	1
0	0	1	0	0	0
0	1	0	0	0	1
0	1	0	0	0	1
1	1	0	0	0	1
0	1	0	0	0	1
0	1	0	0	0	1
1	1	0	0	0	1
0	1	0	0	0	1
0	1	0	0	0	1
0	1	0	1	0	1
1	0	1	0	0	1
1	1	0	0	0	1
0	0	0	0	0	1
0	0	1	0	0	1
1	0	1	0	0	1
0	0	1	0	0	1
0	1	0	0	0	1
0	1	0	0	0	1
1	0	1	0	0	1
0	1	0	0	0	1
0	1	0	0	0	1
0	0	1	0	0	0
0	0	1	0	0	1
0	0	1	0	0	1
0	1	0	1	0	1
0	0	1	0	0	1
1	1	0	0	0	0
1	1	0	0	0	1
1	0	1	0	0	0
0	1	0	0	0	1
0	0	0	0	0	1
0	1	0	0	0	1
1	1	0	0	0	1
0	1	0	0	0	1
0	1	0	0	0	1
0	1	0	0	0	1
0	0	0	0	0	1
0	1	0	0	0	1
0	1	0	0	0	1
1	1	0	0	0	1
1	1	0	0	0	1
0	1	0	0	0	1
1	1	0	0	0	1
1	1	0	0	0	1
0	1	0	0	0	1
1	0	0	1	0	1
0	1	0	0	0	1
0	1	0	0	0	1

1	0	1	0	0	0
1	1	0	0	0	1
0	1	0	1	0	1
0	1	0	0	0	1
1	1	0	0	0	1
1	0	1	0	0	1
0	0	1	0	0	0
1	0	1	0	0	1
0	1	0	0	0	1
0	1	0	0	0	1
0	0	1	0	0	1
0	1	0	0	0	1
0	1	0	0	0	1
1	1	0	0	0	1
1	1	0	0	0	1
0	1	0	0	0	1
0	1	0	0	0	1
1	1	0	0	0	1
0	1	0	0	0	1
0	1	0	0	0	1
1	1	0	0	0	1
1	1	0	0	0	1
1	1	0	0	0	1
0	1	0	0	0	1
0	1	0	0	0	1
1	1	0	0	0	1
0	1	0	1	0	1
0	1	0	0	0	1
0	1	0	0	0	1
1	0	1	0	0	1
1	1	0	0	0	1
1	1	0	0	0	1
1	1	0	0	0	1
1	0	0	0	0	1
0	1	0	0	0	1
0	0	0	0	0	1
1	1	0	0	0	1
0	0	1	0	0	1
0	1	0	0	0	1
0	0	1	0	0	1
0	0	1	0	0	1
0	0	1	0	0	1
0	1	0	0	0	1
0	0	1	0	0	1
0	0	0	0	0	1
1	0	0	1	0	1
1	0	1	0	0	1
0	1	0	0	0	1
1	0	1	0	0	1
0	1	0	0	0	1
1	0	1	0	0	1
0	1	0	0	0	1
1	0	1	0	0	1
1	1	0	0	0	1
1	0	0	0	0	0
1	1	0	0	0	1
0	1	0	0	0	1

0	1	0	0	0	1
1	1	0	0	0	1
1	1	0	0	0	1
0	1	0	0	0	1
0	1	0	0	0	1
0	1	0	0	0	1
0	0	1	0	0	1
0	1	0	0	0	1
1	0	1	0	0	1
0	1	0	0	0	0
0	0	1	0	0	1
1	0	1	0	0	1
0	1	0	0	0	1
1	1	0	0	0	0
0	0	1	0	0	0
1	1	0	0	0	1
0	0	1	0	0	1
1	1	0	0	0	1
0	1	0	0	0	1
1	1	0	1	0	1
1	0	1	0	0	1
1	1	0	0	0	1
0	0	1	0	0	1
0	0	0	0	0	1
1	1	0	0	0	1
0	0	0	0	0	1
0	1	0	0	0	1
0	0	1	0	0	0
0	0	0	0	0	0
0	0	0	0	0	0
0	0	0	0	0	0
1	0	0	0	0	1
0	1	0	0	0	1
0	1	0	0	0	1
1	1	0	0	0	1
0	0	1	0	0	1
1	1	0	0	0	1
0	0	1	0	0	1
0	0	1	0	0	1
0	0	0	0	0	1
0	0	0	0	0	1
1	0	0	0	0	0
1	0	0	0	0	0
1	1	0	0	0	1
0	1	0	0	0	1
0	1	0	0	0	1
0	1	0	0	0	1
1	1	0	0	0	1
0	0	0	0	0	1
0	0	1	0	0	1
0	1	0	0	0	1
1	1	0	0	0	1
0	1	0	0	0	1
0	1	0	0	0	1
0	1	0	0	0	1
1	1	0	0	0	1
1	1	0	0	0	1

0	1	0	0	0	1
0	0	1	0	0	1
1	1	0	0	0	0
1	0	1	0	0	0
0	1	0	1	0	1
0	0	1	0	0	1
1	1	0	0	1	1
0	0	0	0	0	0
0	0	0	1	0	1
1	0	0	0	0	1
0	1	0	0	0	1
1	0	1	0	0	1
1	0	1	0	0	1
0	0	1	0	0	1
0	1	0	0	0	1
0	0	1	0	0	1
0	1	0	0	0	1
1	0	0	0	0	1
1	1	0	0	0	1
0	1	0	0	0	1
0	0	0	0	0	1
0	0	1	0	0	1
1	0	1	0	0	0
1	1	0	0	0	1
0	0	1	0	0	0
0	0	0	0	0	0
0	1	0	0	0	1
1	1	0	0	0	1
1	1	0	0	0	0
0	1	0	0	0	1
1	1	0	0	0	1
1	1	0	0	0	1
1	0	1	0	0	1
0	1	0	0	0	1
0	0	1	0	0	1
0	1	0	0	0	1
0	0	1	0	0	1
1	1	0	0	0	1
0	1	0	0	0	1
0	0	0	0	0	0
0	0	0	0	0	0
1	1	0	0	0	1
1	0	1	0	0	1
1	1	0	0	0	1
1	1	0	0	0	1
1	1	0	0	0	1
1	1	0	0	0	1
1	1	0	0	0	1
0	1	0	0	0	1
1	1	0	0	0	1
0	1	0	0	0	1
0	1	0	0	0	1
0	0	0	0	0	1
0	1	0	0	0	1
0	0	1	0	0	1
0	1	0	0	0	1
1	1	0	0	0	1

0	0	1	0	0	1
0	0	1	0	0	1
0	1	0	0	0	1
0	0	0	0	0	1
1	0	0	0	0	0
0	1	0	1	0	1
0	1	0	0	0	1
0	0	1	0	0	1
0	1	0	0	0	1
0	0	0	0	0	0
0	0	0	0	0	0
1	0	1	0	0	0
0	0	1	0	0	0
0	0	0	0	0	0
0	0	1	0	0	1
0	0	1	0	0	0
0	0	0	0	0	0
0	0	0	0	0	0
0	0	1	0	0	1
1	0	0	0	0	0
1	0	1	0	0	0
0	0	1	0	0	0
0	0	1	0	0	0
1	0	0	0	0	0
1	0	0	0	0	1
0	1	0	0	0	0
0	0	0	0	0	0
0	0	1	0	0	1
0	0	1	0	0	0
1	0	0	0	0	1
0	0	1	0	0	0
0	0	0	0	0	0
0	0	0	0	0	0
0	0	0	0	0	0
0	0	1	0	0	0
0	0	1	0	0	0
0	0	0	0	0	1
0	0	0	0	0	1
0	0	0	0	0	0
0	1	0	0	0	1
1	0	1	0	0	1
1	1	0	0	0	1
0	0	1	0	0	1
1	1	0	0	0	1
0	0	0	0	0	0
1	0	0	0	0	1
1	1	0	0	0	1
1	0	0	0	0	0
0	0	1	0	0	1
0	0	1	0	0	0
0	0	0	0	0	0
0	0	1	0	0	1
0	0	0	0	0	1
0	0	1	0	0	1
0	1	0	0	0	1
0	0	1	0	0	1
0	0	1	0	0	0

0	0	0	0	0	0
0	0	1	0	0	1
0	1	0	0	0	1
0	1	0	0	0	1
1	1	0	0	0	1
1	1	0	0	0	1
0	1	0	0	0	1
0	0	1	0	0	1
1	0	1	0	0	1
0	1	0	0	0	1
0	0	1	0	0	0
0	0	1	0	0	0
1	0	1	0	0	0
1	0	0	0	0	1
0	1	0	0	0	1
0	0	1	0	0	1
1	0	1	0	0	1
0	0	1	0	0	1
1	0	1	0	0	1
0	0	1	0	0	1
1	1	0	0	0	1
0	0	1	0	0	1
0	1	0	0	0	1
0	1	0	1	0	1
0	0	0	0	0	1
1	0	1	0	0	1
1	1	0	0	0	1
1	1	0	0	0	1
0	1	0	0	0	1
0	1	0	0	0	1
0	1	0	0	0	1
1	0	1	0	0	1
1	1	0	0	0	1
0	1	0	0	0	1
1	0	0	0	0	0
1	0	1	0	0	0
0	1	0	0	0	1
0	1	0	0	0	1
0	0	1	0	0	1
1	1	0	0	0	0
0	0	0	0	0	0
0	0	1	0	0	1
1	1	0	0	0	1
1	0	0	0	0	0
0	0	0	0	0	1
0	0	1	0	0	0
1	0	1	0	0	1
1	1	0	1	0	1
1	0	1	0	0	1
0	0	0	0	0	0
1	0	0	0	0	1
1	0	0	0	0	1
0	0	1	0	0	1
0	1	0	0	0	1
1	1	0	0	0	1
1	1	0	0	0	1
0	0	0	0	0	0

1	0	0	0	0	0
1	0	0	0	0	1
1	0	1	0	0	0
0	0	0	0	0	0
0	0	0	0	0	1
1	0	0	0	0	1
1	0	1	0	0	0
1	0	0	0	0	0
1	0	1	0	0	1
0	0	0	0	0	0
0	0	0	0	0	0
0	0	1	0	0	1
0	0	1	0	0	1
0	0	1	0	0	1
0	0	1	0	0	1
0	0	0	0	0	0
0	0	1	0	0	0
0	0	1	0	0	1
1	0	0	0	0	0
0	0	0	0	0	0
0	1	0	0	0	1
1	0	0	0	0	0
1	0	1	0	0	0
0	0	1	0	0	0
1	1	0	0	0	1
0	1	0	0	0	1
0	0	0	0	0	0
0	1	0	0	0	1
0	0	1	1	0	1
0	1	0	0	0	1
0	0	0	0	0	0
0	0	0	0	0	0
0	0	1	0	0	0
0	0	0	0	0	0
0	0	1	0	0	0
0	1	0	0	0	0
0	1	0	0	0	1
1	0	1	0	0	0
1	0	1	1	0	1
0	0	0	0	0	0
0	1	0	0	0	1
0	1	0	0	0	1
0	0	1	0	0	1
0	1	0	0	0	1
1	0	0	0	0	1
1	1	0	0	0	1
0	0	0	0	0	1
0	1	0	0	0	1
0	0	0	0	0	0
0	0	1	0	0	1
0	0	0	0	0	0
0	0	0	0	0	0
0	0	0	0	0	0
0	0	0	0	0	0
1	0	0	0	0	0
0	1	0	0	0	1
0	0	0	0	0	1

0	1	0	1	0		1
0	0	0	0	0		0
0	0	0	0	0		1
0	0	0	0	0		0
1	0	1	0	0		1
0	1	0	0	0		1
0	0	1	0	0		0
1	0	0	0	0		0
1	1	0	0	0		1
1	0	0	0	0		1
0	0	1	0	0		1
0	1	0	0	0		1
0	1	0	0	0		1
0	1	0	0	0		1
1	0	1	0	0		1
1	0	0	0	0		0
1	1	0	0	0		1
1	0	0	0	0		1
0	0	0	0	0		1
1	0	1	0	0		1
0	0	1	0	0		1
0	0	1	0	0		0
0	0	1	0	0		1
0	0	0	0	0		1
0	1	0	0	0		0
0	0	0	0	0		1
0	0	0	0	0		0
0	0	1	0	0		1
0	0	0	0	0		1
1	0	0	0	0		1
0	0	0	0	0		1
0	0	1	0	0		1
0	0	0	0	0		1
0	1	0	0	0		1
0	0	0	0	0		0
0	0	0	0	0		1
1	0	1	0	0		1
1	0	1	0	0		1
0	0	0	0	0		1
0	0	0	0	0		0
0	0	0	0	0		1
0	0	0	0	0		1
1	0	1	0	0		1
1	0	1	0	0		1
0	0	0	0	0		0
1	1	0	0	0		1
1	0	1	0	0		1
0	0	0	0	0		1
0	1	0	0	0		1
1	1	0	0	0		1
1	0	1	0	0		1
0	0	1	0	0		0
0	1	0	0	0		1
1	0	0	0	0		0
1	0	1	0	0		1
0	1	0	0	0		1
0	0	1	0	1		1

0	0	0	0	0	1
0	1	0	0	0	1
0	0	1	0	0	1
0	0	0	0	0	1
0	1	0	0	0	1
0	0	0	0	0	1
1	0	1	0	0	1
0	0	0	0	0	1
1	1	0	0	0	1
0	1	0	0	0	1
0	1	0	0	0	1
0	1	0	0	0	1
0	0	0	0	1	1
1	0	0	0	0	0
0	0	0	0	0	1
0	0	0	0	0	1
0	1	0	0	0	1
0	1	0	0	0	1
0	1	0	1	0	1
1	0	0	0	1	0
1	0	1	0	0	0
0	1	0	1	0	1
0	1	0	0	0	1
0	0	1	0	0	1
0	1	0	0	0	1
1	0	0	0	0	1
1	1	0	0	1	1
1	0	0	1	0	0
0	1	0	0	0	1
1	0	0	0	0	1
1	1	0	0	0	1
1	0	0	0	0	1
0	1	0	0	0	1
0	1	0	1	0	1
0	0	0	0	0	0
0	0	1	0	0	0
0	0	1	0	0	0
0	0	0	0	0	0
0	0	0	0	0	0
0	0	0	0	0	1
0	1	0	0	0	0
0	0	1	0	0	0
0	1	0	0	0	0
0	1	0	0	0	1
0	1	0	0	0	1
0	1	0	0	0	1
0	0	0	0	0	1
0	0	1	0	0	0
0	0	0	0	0	0
0	0	0	0	0	0
0	1	0	0	0	1
0	0	0	0	0	1
0	0	0	0	0	0
0	0	1	0	0	0
0	0	1	0	0	1
0	0	1	0	0	0
0	0	1	0	0	0

1	0	1	0	0	0
0	1	0	0	0	1
0	1	0	0	0	0
0	0	0	0	0	0
1	1	0	0	0	1
1	0	1	0	0	1
1	0	0	0	0	1
0	0	0	0	0	1
0	1	0	0	0	1
0	1	0	0	0	1
0	0	0	0	0	0
0	1	0	0	0	1
0	0	1	0	0	1
0	0	0	0	0	1
0	1	0	0	0	1
0	0	0	0	0	1
0	1	0	0	0	1
0	1	0	0	0	1
1	1	0	0	0	1
1	0	1	0	0	1
1	1	0	0	0	1
1	0	0	0	0	0
0	1	0	0	0	1
0	0	1	0	0	1
0	1	0	0	0	1
0	1	0	0	0	1
0	1	0	0	0	1
0	1	0	0	0	1
0	0	1	0	0	1
1	0	1	0	0	1
0	0	0	0	0	1
0	0	1	0	0	0
0	1	0	0	0	1
0	1	0	0	0	1
1	1	0	0	0	1
1	1	0	0	0	1
0	0	0	0	0	0
0	1	0	0	0	1
0	1	0	0	0	1
0	1	0	0	0	1
0	1	0	1	0	1
0	1	0	0	0	1
0	1	0	1	0	1
0	1	0	0	0	1
0	0	0	0	0	0
0	1	0	0	0	1
0	1	0	1	0	1
0	1	0	0	0	1
0	0	1	1	0	1
0	0	0	0	0	1
1	0	0	0	0	0
0	0	0	0	0	0
0	0	0	0	0	1
0	1	0	0	0	0
0	0	0	0	0	1
0	1	0	0	0	1
0	0	0	0	0	1

1	1	0	0	0	1
1	1	0	0	0	1
0	0	1	1	0	1
0	0	0	0	0	0
0	0	0	0	0	1
0	0	1	0	0	0
1	0	1	0	0	0
0	0	1	0	0	0
0	1	0	0	0	1
1	1	0	0	0	1
0	1	0	0	0	1
0	0	1	0	0	1
0	0	0	0	0	0
0	1	0	0	0	1
0	0	0	0	0	1
1	0	1	0	0	1
1	0	0	0	0	1
1	0	0	0	0	0
1	0	0	0	0	0
1	0	0	0	0	0
1	0	1	0	0	1
0	0	1	0	0	0
1	0	1	0	0	0
1	0	1	0	0	1
0	0	0	0	0	0
0	0	0	0	0	0
1	0	0	0	0	0
0	0	0	0	0	1
0	0	0	0	0	1
1	0	1	0	0	1
1	0	1	0	0	1
0	0	0	0	0	1
0	1	0	0	0	1
1	0	1	0	0	1
1	1	0	0	0	1
0	1	0	0	0	1
0	0	1	0	0	1
0	1	0	0	0	1
0	0	0	0	0	1
0	1	0	0	0	1
0	0	1	0	0	1
1	1	0	0	0	1
1	0	0	0	0	0
0	0	0	0	0	1
0	1	0	0	0	1
0	0	1	0	0	1
0	1	0	0	0	1
1	1	0	0	0	1
0	0	0	0	0	0
1	0	0	0	0	1
0	1	0	0	0	1
0	0	0	0	0	0
0	0	1	0	0	1
1	1	0	0	0	0
1	1	0	1	0	1
1	0	1	0	0	0
0	1	0	0	0	1

0	1	0	0	0	1
0	0	1	0	0	1
1	0	1	0	0	1
0	0	1	0	0	1
0	0	0	0	0	1
0	1	0	0	0	1
0	0	0	1	0	1
1	0	0	0	0	1
1	0	1	0	0	1
0	0	1	0	0	1
0	0	1	0	0	0
0	1	0	0	0	1
0	0	1	0	0	1
0	1	0	1	0	1
0	1	0	0	0	1
1	0	1	0	0	1
1	0	0	0	0	0
0	1	0	0	0	1
0	0	1	0	0	1
0	1	0	0	0	0
0	0	1	0	0	1
0	0	0	0	0	0
0	0	0	0	0	0
0	1	0	0	0	0
1	0	0	0	0	0
0	1	0	0	0	0
0	1	0	0	0	1
0	1	0	0	0	1
0	0	1	0	0	1
0	0	0	0	0	0
1	0	0	0	0	0
1	0	1	0	0	1
0	0	1	0	0	1
0	1	0	0	0	1
0	0	0	0	0	1
1	0	1	0	0	1
0	0	1	0	0	0
0	1	0	0	0	1
0	1	0	0	0	1
0	0	1	0	0	0
1	0	0	0	0	0
0	0	0	0	0	0
1	0	0	0	0	0
0	0	1	0	0	0
1	0	0	0	0	0
1	0	0	0	0	0
1	0	0	0	0	0
0	1	0	0	0	1
0	1	0	1	0	1
0	1	0	0	0	1
0	0	1	1	0	1
1	0	0	0	0	0
0	0	1	0	0	1
0	1	0	0	0	1
0	1	0	0	0	1
0	0	0	0	0	1
0	1	0	0	0	1

0	0	1	0	0	1
0	1	0	0	0	1
0	0	0	0	0	0
0	0	1	0	0	0
0	1	0	0	0	1
0	0	0	0	0	1
0	0	0	0	0	0
1	1	0	0	0	1
1	1	0	1	0	1
0	1	0	0	0	1
0	1	0	0	0	1
0	1	0	0	0	1
1	0	1	0	0	1
1	1	0	0	0	1
0	0	0	0	0	1
1	0	1	0	0	1
1	0	1	0	0	1
0	0	0	0	0	1
0	0	0	0	0	0
0	1	0	0	0	1
0	1	0	0	0	0
1	0	1	0	0	0
1	0	1	0	0	1
1	0	0	0	0	0
0	0	0	0	0	1
0	0	1	0	0	1
0	0	0	0	0	0
0	0	0	0	0	1
0	0	0	0	0	1
1	0	0	0	0	1
0	1	0	0	0	0
0	0	0	0	0	0
1	1	0	0	0	0
1	0	1	0	0	1
1	0	0	0	0	1
0	0	0	0	0	1
0	1	0	0	0	1
0	0	0	0	0	1
0	1	0	0	0	1
0	1	0	0	0	1
0	1	0	0	0	1
1	0	1	0	0	0
1	0	1	0	0	1
0	0	1	0	0	0
0	0	1	0	0	1
0	1	0	0	0	1
0	1	0	0	0	1
0	0	1	0	0	1
0	0	1	0	0	1
0	0	0	0	0	0
0	0	1	0	0	1
0	0	1	0	0	0
1	1	0	0	0	1
0	1	0	1	0	1
1	0	0	0	0	1
1	1	0	0	0	0
1	0	1	0	0	1

0	0	1	0	0	1
0	0	0	0	0	1
0	0	0	0	0	0
1	0	1	0	0	1
0	0	1	0	0	1
0	1	0	0	0	1
0	0	1	0	0	1
1	0	0	0	0	0
1	0	0	0	0	0
1	0	0	0	0	1
1	0	0	0	0	1
1	0	0	0	0	1
1	0	0	0	0	1
0	0	0	0	0	0
0	1	0	0	0	1
1	1	0	0	0	1
0	0	1	0	0	1
0	0	1	0	0	1
0	0	0	0	0	1
0	1	0	0	0	1
0	0	0	0	0	0
0	1	0	0	0	1
0	0	0	0	0	1
0	0	1	0	0	1
0	0	1	0	0	1
0	1	0	0	0	1
0	1	0	0	0	1
0	0	0	0	1	1
1	0	0	0	1	1
0	1	0	0	0	1
0	1	0	0	0	1
0	1	0	0	0	1
0	0	1	0	0	1
1	1	0	1	0	1
1	0	1	0	0	1
0	0	0	0	0	0
0	1	0	0	0	1
0	0	0	0	0	1
0	0	1	0	0	1
1	0	1	0	0	0
1	0	0	0	0	1
0	1	0	0	0	1
0	1	0	1	0	1
0	0	1	0	0	1
0	0	1	0	0	1
0	0	0	0	0	0
0	1	0	0	0	1
0	0	0	0	0	1
0	1	0	0	0	1
0	1	0	0	0	1
0	0	1	0	0	1
0	0	1	0	0	0
0	1	0	1	0	1
1	0	1	0	0	0
0	0	0	0	0	1
1	0	1	0	0	1
1	1	0	0	0	1

1	1	0	1	0	1
1	1	0	0	0	1
0	0	0	0	0	1
0	0	1	0	0	1
1	0	1	0	0	1
0	0	0	0	0	1
0	1	0	0	0	1
0	1	0	0	0	1
0	0	1	0	0	1
0	1	0	0	0	1
0	0	1	0	0	1
1	0	1	0	0	0
0	0	0	0	0	1
1	1	0	0	0	1
0	0	0	0	0	0
0	0	0	0	0	0
1	0	1	0	0	0
0	0	1	0	0	1
0	0	1	0	0	1
0	0	1	0	0	1
0	0	1	0	0	1
1	1	0	0	0	1
0	0	1	0	0	0
0	0	1	0	0	1
1	1	0	0	0	1
1	1	0	0	0	1
0	1	0	0	0	1
0	0	0	0	0	1
0	0	1	0	0	1
0	0	0	0	0	1
0	1	0	0	0	1
0	0	0	0	0	1
0	0	1	0	0	1
0	0	1	0	0	1
0	0	1	0	0	1
1	1	0	0	0	1
1	1	0	0	0	1
0	1	0	0	0	1
0	0	0	0	0	0
0	0	1	0	0	1
1	0	0	0	1	1
1	1	0	0	0	1
0	1	0	1	0	1
0	0	0	0	0	1
0	0	0	0	0	0
0	1	0	0	0	1
1	1	0	0	0	1
0	1	0	0	0	1
0	0	1	0	0	0
0	0	0	0	0	1
0	0	0	0	0	1
0	0	0	0	0	0
0	0	1	0	0	0
0	0	0	0	0	1
1	1	0	0	0	1
0	1	0	1	0	1
0	1	0	0	0	1

0	0	0	0	0	0
1	0	0	0	0	0
0	1	0	0	0	1
0	1	0	0	0	1
0	0	0	0	0	1
0	0	1	0	0	1
1	1	0	0	0	1
0	0	1	0	0	1
0	1	0	0	0	1
1	0	1	0	0	1
1	0	0	0	0	1
1	0	1	0	0	1
1	1	0	0	0	1
0	0	1	0	0	0
0	0	0	0	0	0
0	0	0	0	0	1
0	0	1	0	0	1
0	0	1	0	0	1
0	0	0	0	0	0
1	1	0	0	0	1
0	1	0	0	0	1
0	0	1	0	0	1
0	0	0	0	0	1
0	0	1	0	0	0
0	0	0	0	0	0
0	0	1	0	0	1
0	0	0	0	0	1
0	0	0	0	0	0
0	1	0	0	0	1
0	0	1	0	0	1
0	0	1	0	0	1
1	0	0	0	0	1
0	0	0	0	0	0
0	0	0	0	0	1
0	0	0	0	0	0
1	1	0	0	0	1
1	0	1	0	0	1
1	0	1	0	0	0
0	0	0	0	0	0
0	0	0	0	0	1
1	0	0	1	0	1
1	0	0	0	0	1
1	1	0	0	0	1
1	1	0	0	1	1
0	0	1	0	0	0
0	1	0	0	0	1
0	1	0	1	0	1
1	1	0	0	0	1
0	0	1	0	0	1
0	1	0	0	0	1
0	0	1	0	0	1
0	0	1	0	0	1
0	0	0	0	0	0
0	0	0	0	0	0
0	0	1	0	0	1
0	0	1	0	0	1
0	0	1	0	0	1

0	0	1	0	0	1
0	0	0	0	0	1
0	0	1	0	0	1
0	0	1	0	0	0
0	0	1	0	0	1
0	0	0	0	0	1
0	0	1	0	0	1
0	0	0	0	0	1
1	1	0	0	0	1
0	0	1	0	0	1
0	0	1	0	0	1
0	0	1	1	0	1
1	0	1	1	0	1
0	1	0	0	0	1
1	1	0	0	0	1
1	1	0	0	0	1
0	0	0	0	0	0
0	1	0	0	0	1
0	0	0	0	0	1
0	0	0	1	0	1
0	1	0	0	0	1
0	1	0	0	0	1
1	0	0	0	0	0
1	1	0	0	0	1
0	0	0	0	0	1
1	0	0	0	0	1
0	0	0	1	0	1
0	0	0	0	0	0
0	1	0	0	0	1
1	0	1	0	0	1
1	1	0	0	0	1
1	1	0	0	0	1
0	0	0	0	0	1
1	0	0	0	0	1
0	1	0	0	0	1
0	0	0	0	0	1
0	1	0	0	0	1
0	0	0	0	0	1
0	0	1	0	0	1
0	0	0	0	0	0
0	1	0	0	0	1
0	1	0	1	0	1
1	0	1	0	0	1
1	1	0	0	0	1
1	0	1	0	0	1
0	0	1	0	0	1
0	0	1	0	0	1
0	0	0	0	0	1
0	0	1	0	0	0
0	0	0	0	0	0
1	1	0	0	0	1
0	1	0	0	0	1
0	0	0	0	0	1
0	0	1	0	0	0
0	1	0	0	0	0
1	1	0	0	0	1
0	1	0	0	0	1

0	0	0	0	0	1
0	0	1	0	0	1
1	1	0	0	0	1
0	1	0	0	0	1
1	1	0	0	0	0
1	0	0	0	0	0
0	0	1	0	0	0
0	0	1	0	0	0
0	0	0	0	0	1
0	0	1	0	0	0
0	0	0	0	0	1
0	0	1	0	0	0
0	1	0	0	0	1
0	1	0	0	0	1
0	0	0	0	0	1
1	1	0	0	0	1
1	1	0	0	0	1
1	0	0	0	0	0
1	0	0	0	0	1
0	1	0	0	0	1
0	0	0	0	0	0
0	0	1	0	0	0
0	0	0	0	0	0
1	0	1	0	0	1
1	0	1	0	0	0
1	0	0	0	0	1
0	1	0	0	0	1
0	1	0	0	0	1
0	1	0	0	0	1
1	0	1	0	0	1
1	0	1	0	0	1
1	1	0	0	0	1
0	1	0	1	0	1
1	1	0	0	0	1
0	0	0	0	0	0
0	0	1	0	0	0
1	0	0	0	0	0
0	0	1	0	0	1
0	0	0	0	0	0
1	1	0	0	0	1
1	0	0	0	0	1
0	0	1	0	0	1
0	0	1	1	0	1
0	1	0	0	0	1
1	0	1	1	0	1
0	1	0	0	0	1
1	1	0	0	0	1
1	0	1	0	0	1
0	1	0	0	0	1
1	1	0	0	0	1
1	0	0	0	0	0
1	1	0	0	0	0
1	0	1	0	1	1
1	0	1	0	0	1
1	0	1	0	0	1
0	0	1	0	0	1
1	0	0	0	0	1

0	1	0	0	0	1
0	1	0	0	0	1
1	1	0	0	0	0
1	1	0	0	0	1
0	0	0	0	0	0
1	0	1	0	0	1
0	0	0	0	0	1
0	0	1	0	0	1
1	1	0	0	0	1
0	0	0	0	0	1
0	0	0	0	0	0
0	0	1	0	0	1
0	0	0	0	0	1
1	0	1	0	0	1
0	0	0	0	0	1
1	0	1	0	0	0
1	1	0	0	0	1
1	1	0	0	0	1
1	0	1	0	0	1
0	0	1	0	0	1
0	0	1	0	0	0
0	0	0	0	0	1
1	1	0	0	0	1
1	0	1	0	0	0
1	0	0	0	0	1
1	1	0	0	0	1
1	0	1	0	0	1
0	0	0	0	0	0
1	0	1	0	0	1
1	1	0	0	0	1
0	1	0	0	0	1
1	1	0	0	0	1
1	1	0	1	0	1
0	1	0	0	0	1
0	0	1	0	0	1
0	0	1	0	0	0
0	0	0	0	0	1
0	0	0	0	0	0
0	0	0	0	0	0
1	0	1	0	0	0
0	0	1	0	0	0
0	1	0	0	0	0
0	0	1	0	0	1
0	0	1	0	0	0
1	0	1	0	0	0
0	0	1	0	0	1
0	0	1	0	0	1
0	0	1	1	0	1
0	0	0	0	0	1
0	0	0	0	0	0
1	0	1	0	0	0
1	1	0	1	0	1
0	1	0	0	0	1
0	0	0	0	0	1
0	0	0	0	0	1
1	1	0	0	0	1
0	1	0	1	0	1

1	0	1	0	0	1
1	0	0	0	0	0
0	1	0	0	0	1
0	1	0	1	0	1
0	1	0	0	0	1
0	0	1	0	0	0
1	1	0	0	0	1
1	0	1	0	0	1
0	0	1	0	0	1
0	0	1	0	0	0
1	0	1	0	0	0
1	0	0	0	0	0
0	0	1	0	0	0
0	0	1	0	0	0
1	1	0	0	0	1
0	0	1	0	0	1
0	1	0	1	0	1
0	0	1	0	0	1
0	0	0	0	0	1
0	0	0	0	0	1
0	0	0	0	0	0
0	0	1	0	0	1
0	1	0	0	0	1
0	0	0	0	0	0
0	0	1	0	0	0
0	0	0	0	0	0
1	0	0	0	0	0
1	1	0	0	0	1
1	0	1	0	0	1
0	1	0	0	0	1
1	0	0	0	0	0
1	0	1	0	0	0
0	0	1	0	0	1
0	1	0	0	0	1
1	1	0	1	0	1
1	0	0	0	0	0
1	0	1	0	0	0
1	0	0	0	0	0
1	0	0	0	0	1
1	0	1	0	0	0
0	1	0	0	0	1
0	0	1	0	0	1
1	0	0	0	0	1
1	0	1	0	0	0
0	0	0	0	0	0
0	0	0	0	0	0
0	0	0	0	0	0
1	0	1	0	0	0
1	1	0	0	0	1
1	0	1	0	0	1
1	1	0	0	0	1
0	1	0	0	0	1
0	0	1	0	0	1
0	0	1	0	0	1
1	1	0	0	0	0
1	1	0	1	0	1
0	1	0	0	0	1

0	0	1	0	0	1
1	0	1	0	1	1
1	1	0	0	0	1
0	1	0	0	0	0
0	1	0	0	0	1
0	1	0	0	0	1
0	0	1	0	0	1
0	1	0	0	0	0
0	0	0	0	0	0
0	0	0	0	0	0
0	0	1	0	0	1
0	0	0	0	0	1
0	1	0	0	0	1
0	0	0	0	0	1
1	0	1	0	0	1
1	0	0	0	0	0
0	0	0	0	0	0
0	0	1	0	0	1
1	0	0	0	0	1
1	0	1	0	0	0
0	1	0	0	0	1
0	0	0	0	0	1
0	1	0	0	0	0
0	0	1	0	0	0
1	0	1	0	0	1
1	0	0	0	0	1
1	0	0	0	0	0
0	0	1	0	0	1
0	0	0	0	0	0
0	0	0	0	0	1
0	0	1	0	0	1
0	0	1	0	0	1
0	0	1	0	0	1
1	0	0	0	0	1
1	1	0	0	0	1
0	1	0	0	0	0
0	0	0	0	0	1
0	0	1	0	0	1
0	0	0	0	0	1
0	0	0	0	0	1
0	0	0	0	0	1
0	1	0	0	0	1
0	1	0	0	0	0
0	0	0	0	0	1
1	1	0	0	0	1
0	0	1	0	0	1
1	0	0	0	0	1
1	0	0	1	0	1
1	1	0	0	0	1
1	1	0	0	0	1
1	0	1	0	0	1
0	1	0	0	0	1
1	0	1	0	0	1
1	0	0	0	0	1
0	0	0	0	0	0
0	0	1	0	0	0
0	0	1	0	1	1

0	0	1	0	0	0
0	0	1	0	0	0
1	1	0	0	0	1
0	0	1	0	0	1
0	1	0	0	0	1
0	0	0	0	0	0
0	1	0	0	0	1
0	0	0	0	0	1
0	1	0	0	0	1
1	1	0	0	0	1
1	0	1	0	0	1
0	1	0	0	0	1
1	1	0	0	0	1
1	0	1	0	0	1
0	0	0	0	0	1
1	0	1	0	0	0
1	0	1	0	0	1
0	0	0	0	0	1
1	1	0	0	0	1
0	1	0	0	0	1
0	0	1	0	0	1
0	1	0	0	0	1
1	1	0	0	0	1
1	0	1	1	0	1
0	1	0	0	0	1
1	0	1	0	0	0
0	1	0	0	0	1
1	0	0	0	0	0
1	1	0	0	0	1
1	1	0	0	0	0
1	0	0	0	0	1
1	0	1	0	0	1
0	0	0	1	0	1
1	1	0	0	0	1
0	1	0	0	0	1
0	0	1	0	0	1
0	1	0	0	0	1
0	1	0	0	0	1
0	0	1	0	0	1
1	0	1	0	0	1
1	0	1	0	0	0
0	0	0	0	0	0
1	0	1	0	0	1
1	0	1	0	0	0
0	1	0	0	0	1
0	0	1	0	0	1
0	1	0	0	0	1
0	0	1	0	0	1
0	0	0	0	0	0
0	0	1	0	0	1
1	1	0	0	0	1
1	0	0	0	0	0
1	0	1	0	0	1
1	1	0	0	0	1
0	0	1	0	0	1
1	0	1	0	0	1
0	0	0	0	0	0

0	1	0	0	0	0
0	0	1	0	1	1
0	0	1	0	1	1
0	0	1	0	0	0
1	0	1	0	0	0
0	0	1	0	0	0
0	0	1	0	0	0
0	0	1	0	0	0
0	0	0	0	0	0
0	0	1	0	0	0
0	0	0	0	0	0
0	0	0	0	0	0
0	0	0	0	0	0
0	0	1	0	0	1
1	0	1	0	0	0
0	0	1	0	0	1
1	0	0	1	0	1
0	1	0	0	0	1
1	1	0	0	0	1
0	1	0	0	0	1
0	1	0	0	0	1
0	0	0	1	0	0
0	0	0	0	0	0
0	1	0	0	0	1
0	0	0	0	0	0
0	0	0	0	0	0
0	0	1	0	0	0
1	1	0	0	0	0
0	1	0	0	0	1
1	0	1	0	0	1
0	0	1	0	0	1
0	0	1	0	0	0
0	0	1	0	0	1
1	1	0	0	0	1
0	0	1	0	0	1
0	0	0	0	0	0
1	1	0	0	0	1
1	0	1	0	0	1
0	0	1	0	0	0
0	1	0	0	0	1
1	0	1	0	0	1
1	0	1	0	0	1
0	0	1	0	0	0
1	0	0	0	0	1
0	0	1	0	0	1
1	0	0	0	0	0
0	0	1	0	0	1
0	1	0	0	0	1
1	0	0	0	0	1
1	0	1	1	0	1
1	0	0	0	0	1
0	1	0	0	0	1
0	0	0	0	0	0
0	1	0	0	0	1
1	0	1	0	0	0
1	1	0	0	0	1
0	0	1	0	0	1

0	0	1	0	0	0
0	0	1	0	0	0
0	0	1	0	0	1
0	1	0	0	0	0
0	0	1	0	0	1
0	0	1	0	0	0
0	1	0	0	0	1
0	0	0	0	0	1
0	1	0	1	0	1
1	1	0	0	0	1
1	0	1	0	0	1
1	0	0	0	0	1
1	0	1	0	0	1
0	0	1	0	1	1
0	1	0	0	0	1
0	1	0	0	0	1
1	1	0	0	0	1
1	1	0	0	0	1
0	0	0	0	0	0
1	1	0	0	0	1
0	1	0	1	0	1
0	1	0	0	0	1
0	0	0	0	0	1
0	0	0	0	0	0
0	1	0	0	0	1
0	0	1	0	0	0
1	0	1	0	0	1
1	0	1	0	0	1
0	0	1	0	0	1
0	1	0	0	0	1
0	1	0	0	0	1
0	0	1	0	0	1
0	0	1	0	0	1
1	0	1	0	0	0
0	1	0	0	0	1
0	0	0	0	0	1
1	0	1	0	0	0
0	0	0	0	0	0
0	0	0	0	0	0
0	0	1	0	0	1
0	0	0	0	0	1
0	0	1	0	0	1
0	1	0	0	0	1
0	0	0	0	0	1
0	0	0	0	0	0
0	0	1	0	0	1
0	1	0	0	0	1
0	0	1	0	0	1
1	1	0	0	0	1
1	1	0	1	0	1
0	0	0	0	0	1
0	0	0	1	0	1
1	0	0	0	0	1
1	0	0	0	0	1
0	0	1	0	0	1
1	0	0	0	0	1
0	1	0	0	0	1

1	0	0	0	0	0
1	1	0	0	0	1
0	1	0	0	0	1
1	1	0	0	0	1
1	0	0	0	0	1
1	1	0	0	1	1
0	1	0	1	0	1
0	1	0	0	0	1
1	0	1	0	0	1
1	0	0	0	0	0
0	1	0	0	0	1
0	1	0	0	0	1
0	0	1	0	1	1
1	0	1	0	0	0
1	0	1	0	0	0
0	1	0	0	0	1
0	0	1	0	0	1
0	0	1	0	0	1
0	0	1	0	0	1
1	0	0	0	0	0
1	0	1	0	0	1
0	0	0	0	0	1
0	0	1	0	0	1
0	1	0	0	0	1
0	0	1	0	0	1
0	0	1	0	0	1
0	0	0	0	0	1
1	0	1	0	0	1
1	0	0	0	0	1
1	1	0	0	0	1
0	0	1	0	0	0
0	0	1	0	0	1
1	0	1	0	0	1
1	0	1	0	0	1
0	1	0	0	0	1
0	0	0	0	0	1
1	0	1	0	0	0
0	0	0	0	0	0
0	0	1	0	0	0
0	0	0	0	0	0
0	0	1	0	0	1
0	0	1	0	0	1
0	0	1	0	0	0
0	0	1	0	0	1
0	0	1	0	0	1
1	0	0	0	0	0
0	1	0	0	0	1
0	1	0	0	0	1
0	0	0	0	0	0
0	0	0	0	0	1
0	0	1	0	0	1
1	0	1	0	0	1
1	0	1	0	0	0
1	1	0	0	0	1
1	1	0	0	0	1
0	0	0	0	0	0
1	1	0	0	0	1

0	0	1	0	0	0
0	0	1	0	0	0
0	0	1	0	0	0
0	0	0	0	0	0
0	0	1	0	0	1
0	0	1	0	1	1
0	0	0	0	0	0
0	1	0	0	0	1
1	0	1	0	0	0
1	1	0	0	0	1
0	0	0	0	0	1
0	0	0	0	0	0
0	0	0	0	0	0
0	1	0	0	0	1
0	1	0	0	0	0
0	0	0	0	0	0
0	1	0	0	0	1
0	0	1	0	0	1
0	1	0	0	0	1
1	0	1	0	0	1
1	0	1	0	0	0
0	0	0	0	0	0
0	0	1	0	0	0
1	0	1	0	0	1
1	1	0	0	0	1
0	1	0	0	0	1
0	0	1	0	0	1
0	0	1	0	0	1
1	0	0	0	0	1
1	1	0	0	0	1
0	1	0	0	0	1
0	0	1	0	0	1
0	1	0	0	0	1
0	1	0	0	0	1
1	0	1	0	0	1
1	0	1	0	0	0
1	1	0	0	0	1
0	0	1	1	0	1
1	1	0	0	0	1
1	0	1	0	0	1
1	0	1	0	0	1
1	1	0	1	0	1
0	0	1	0	0	0
0	0	0	0	0	1
0	1	0	0	0	0
0	0	0	0	0	1
1	0	0	0	0	0
0	1	0	0	0	1
0	0	0	0	0	0
0	1	0	0	0	1
0	0	1	0	0	1
0	0	1	1	0	1
0	0	1	0	0	1
0	1	0	0	0	1
1	0	1	0	0	1
0	1	0	0	0	1
0	0	1	0	0	1

1	1	0	0	0	1
1	0	1	0	0	1
0	0	1	0	0	1
0	0	0	0	1	1
0	1	0	1	0	1
0	0	0	0	0	1
0	0	1	0	0	1
0	0	0	0	0	0
0	1	0	0	0	1
0	0	0	0	1	1
0	1	0	0	0	1
1	0	1	0	0	1
1	0	0	0	0	0
0	0	1	0	0	1
0	1	0	0	0	1
0	1	0	0	0	1
0	1	0	0	0	1
1	0	1	0	0	1
1	0	1	0	0	1
0	0	0	0	0	1
0	0	1	0	0	1
0	0	0	0	0	1
0	1	0	0	1	1
1	1	0	0	0	1
1	1	0	0	0	1
1	0	0	0	0	0
1	1	0	0	0	1
1	1	0	1	0	1
0	0	1	0	0	1
0	0	0	0	0	0
0	0	0	0	0	0
1	0	1	0	0	0
0	0	1	0	0	0
0	0	1	0	0	0
0	0	0	0	0	0
0	0	0	0	0	0
0	0	0	0	0	1
0	0	1	0	0	1
0	0	1	0	0	1
0	1	0	0	0	1
0	1	0	0	0	1
0	0	1	0	0	1
0	1	0	0	0	0
1	1	0	0	0	1
1	1	0	0	0	0
0	1	0	0	0	1
1	0	0	0	0	1
1	0	0	0	0	0
1	1	0	0	0	1
0	0	1	0	0	1
0	0	0	0	0	1
0	1	0	1	0	1
0	0	0	0	0	0
0	0	0	0	0	0
0	0	0	0	0	0
0	0	1	0	0	0
1	0	0	0	0	0

0	0	1	0	0	1
0	0	1	0	0	1
1	0	0	0	0	1
0	0	0	0	0	0
0	0	0	0	1	1
0	0	1	0	0	1
0	0	0	0	0	0
0	0	1	0	0	0
0	1	0	0	0	1
0	1	0	0	0	1
0	1	0	0	0	1
0	0	1	0	0	1
0	0	0	0	0	0
1	0	0	0	0	1
0	0	0	0	0	0
1	0	1	0	0	0
1	0	1	0	0	0
1	0	0	0	0	0
1	0	0	0	0	0
0	0	1	0	0	0
0	0	1	0	0	0
0	0	0	0	0	1
0	0	0	0	0	0
0	0	1	0	1	1
0	1	0	1	0	1
0	0	0	0	0	1
0	0	1	0	0	1
0	0	0	0	0	0
1	0	0	0	0	1
1	1	0	0	0	1
1	0	0	0	0	1
0	0	1	0	0	1
0	1	0	0	0	1
1	1	0	0	0	1
1	0	1	1	0	1
1	1	0	0	0	1
0	0	0	0	0	1
1	1	0	0	0	1
1	1	0	0	0	1
0	0	0	0	0	1
0	0	0	0	0	1
0	0	0	0	0	0
0	0	0	0	0	0
1	0	0	0	0	0
1	1	0	0	0	1
0	1	0	0	0	1
1	0	1	0	0	0
0	1	0	0	0	1
1	1	0	0	0	1
1	0	1	0	0	0
0	1	0	0	0	1
0	1	0	0	0	0
1	0	0	0	0	1
0	0	1	0	0	1
1	0	0	0	0	0
1	0	0	0	0	0
0	1	0	0	0	1

1	0	1	0	0	1
1	0	1	0	0	1
1	0	1	0	0	1
1	0	0	0	0	0
0	0	0	0	0	1
0	0	1	0	0	1
0	0	1	0	0	1
0	1	0	0	0	1
0	1	0	0	0	1
1	1	0	0	0	1
0	0	1	0	0	0
0	0	0	0	0	0
0	1	0	1	0	1
0	0	0	0	0	0
0	0	1	0	0	0
0	0	1	0	0	0
0	0	0	0	0	1
0	1	0	0	0	1
0	0	0	0	1	1
0	0	0	0	1	1
1	0	1	0	0	1
1	0	0	0	0	0
1	0	1	0	0	0
0	0	0	0	0	1
0	0	1	0	0	0
0	0	1	0	0	1
0	1	0	0	0	1
0	0	1	0	0	1
0	1	0	0	0	1
0	0	0	0	0	0
0	0	1	0	0	0
0	1	0	0	0	1
0	0	0	0	0	1
1	0	1	0	0	1
0	0	1	0	0	0
0	1	0	0	0	0
0	0	0	0	0	0
0	0	0	0	0	0
1	0	1	0	0	0
0	0	0	0	0	0
1	0	1	0	0	1
1	0	1	0	0	0
0	0	1	0	0	0
0	0	0	0	0	0
1	1	0	0	0	0
0	1	0	0	0	1
1	1	0	0	0	1
1	0	1	0	0	1
0	1	0	0	0	1
1	1	0	0	0	1
1	0	1	0	0	0
0	0	0	0	0	1
1	0	0	0	0	1
0	0	1	0	0	1
0	0	1	0	0	0
1	0	1	0	0	0
0	1	0	0	0	1

0	0	1	0	0	0
0	0	0	0	0	0
0	0	0	0	0	0
0	0	1	0	0	0
1	0	0	1	0	1
1	0	1	0	0	1
1	0	0	0	0	0
1	0	1	0	0	0
1	0	1	0	0	0
1	0	0	0	0	0
1	1	0	0	0	1
1	0	1	0	0	1
0	1	0	0	0	1
0	1	0	0	0	1
0	0	0	0	0	0
0	1	0	0	0	1
1	0	1	0	0	0
0	0	1	0	0	0
0	0	1	0	0	0
0	0	1	0	0	1
1	0	1	0	0	1
0	0	0	0	0	0
1	1	0	1	0	1
0	0	0	0	0	0
1	0	1	0	0	0
0	0	0	0	0	1
1	0	1	0	0	1
0	0	1	0	0	0
0	0	0	0	0	0
1	0	0	0	0	1
1	1	0	0	0	1
1	0	1	0	0	1
0	0	1	0	0	0
0	1	0	0	1	1
0	0	1	0	1	1
0	0	1	0	1	1
0	0	1	0	0	1
1	1	0	0	0	1
1	1	0	0	0	1
0	1	0	0	0	1
1	1	0	0	0	1
1	0	1	0	0	1
0	0	1	0	0	1
0	0	0	0	0	0
0	0	1	0	0	1
1	0	1	0	0	1
0	1	0	0	0	1
1	0	1	0	0	1
1	1	0	0	0	1
0	1	0	0	0	1
0	0	0	0	0	1
0	0	1	0	0	1
0	0	1	0	0	1
1	1	0	0	0	1
1	0	0	0	0	1
1	0	1	0	0	1
1	0	1	0	0	0

0	0	1	0	0	0
0	0	1	0	0	0
0	0	0	0	0	1
0	1	0	0	0	1
1	0	0	0	0	1
1	1	0	1	0	1
0	0	1	0	0	1
0	0	1	0	0	0
1	0	1	0	0	0
0	0	1	0	0	0
0	0	1	0	0	0
0	0	0	0	0	0
0	0	1	0	0	0
0	0	0	0	0	1
0	1	0	0	0	1
0	0	1	0	0	1
1	1	0	0	0	1
1	0	0	0	0	1
1	0	1	0	0	0
0	0	1	0	0	1
0	0	1	0	0	1
1	1	0	0	0	1
0	0	1	0	0	1
0	0	0	0	0	0
1	1	0	0	0	0
1	0	1	0	0	1
1	0	1	0	0	1
1	1	0	1	0	1
0	1	0	0	0	1
0	1	0	0	0	1
1	0	1	0	0	1
1	0	1	1	0	1
1	0	1	0	0	1
0	0	1	0	0	0
0	0	1	0	0	1
1	1	0	0	0	1
0	0	0	0	0	1
0	0	1	0	0	1
0	1	0	0	0	1
0	1	0	0	0	1
0	0	1	0	0	1
0	1	0	0	0	1
0	0	1	0	0	1
0	0	1	0	0	1
0	0	1	0	0	0
0	1	0	0	0	0
1	0	1	0	0	1
0	0	1	0	0	0
0	0	0	0	0	1
1	0	1	0	0	0
1	0	1	0	0	1
1	0	0	0	0	1
1	0	1	0	0	1
0	1	0	0	1	1
1	1	0	0	0	1
1	0	0	0	0	0
0	0	1	0	0	0

0	0	1	0	0	1
0	0	0	0	0	0
0	0	1	0	0	0
0	0	1	0	0	0
0	1	0	0	0	1
1	0	1	0	0	1
1	1	0	0	0	1
1	1	0	0	0	1
1	1	0	0	0	1
0	0	1	0	0	0
0	0	1	0	0	0
0	1	0	0	0	1
1	0	1	0	0	1
1	1	0	0	0	1
0	1	0	0	0	1
0	0	0	0	0	0
0	1	0	0	0	1
1	0	1	0	0	0
0	0	1	0	0	1
0	0	1	0	0	1
1	0	1	0	0	1
1	1	0	0	0	1
0	1	0	0	0	1
0	1	0	0	0	1
0	0	1	1	0	1
1	0	1	0	0	1
1	1	0	0	0	1
0	0	1	0	0	0
0	0	0	0	0	1
1	1	0	0	0	1
1	1	0	0	0	1
1	1	0	0	0	1
1	1	0	0	0	1
1	1	0	0	0	1
1	1	0	0	0	1
1	0	1	0	0	1
0	0	1	0	0	1
1	1	0	0	0	1
0	1	0	0	0	1
1	1	0	0	0	1
0	0	0	0	0	1
0	0	0	0	0	1
0	1	0	0	0	1
0	1	0	0	0	1
0	1	0	0	0	1
0	1	0	0	0	1
0	0	1	0	0	0
0	0	1	0	0	0
0	0	0	0	0	0
0	0	0	0	0	0
0	0	1	0	0	0
0	1	0	1	0	1
0	0	0	0	0	1
0	1	0	0	0	1
0	1	0	0	0	1
0	0	0	0	0	1
0	0	0	0	0	1

1	1	0	0	0	1
1	0	1	0	0	0
0	0	0	0	0	0
0	0	0	0	0	0
0	0	1	0	0	0
1	0	0	0	0	1
1	1	0	0	0	1
0	0	1	0	0	1
0	0	1	0	0	1
0	1	0	0	0	1
1	0	0	0	0	0
0	1	0	0	0	1
0	0	0	0	0	1
0	0	1	0	0	0
0	1	0	0	0	1
1	0	1	0	0	1
0	0	1	0	0	1
0	0	1	0	0	0
0	1	0	0	0	0
0	0	1	0	0	1
1	0	0	0	0	0
0	1	0	0	0	1
1	1	0	0	0	0
0	0	1	0	0	1
0	1	0	0	0	1
1	0	0	0	0	0
1	0	1	0	0	0
0	1	0	0	0	1
1	1	0	0	0	1
1	1	0	0	0	1
1	0	0	0	0	0
1	0	1	0	0	1
1	0	1	0	0	1
1	1	0	0	0	1
0	0	1	0	0	0
0	0	0	0	0	0
0	0	1	0	0	1
0	1	0	0	0	1
1	0	0	0	0	1
0	0	0	0	0	1
0	0	0	0	0	0
0	0	0	0	0	0
1	0	1	0	0	1
0	0	1	0	0	1
0	0	1	0	0	1
1	1	0	0	0	1
0	1	0	0	0	1
0	0	1	0	0	1
0	1	0	0	0	1
0	1	0	0	0	1
0	0	1	0	0	1
1	0	1	0	0	1
0	0	0	0	0	0
1	0	1	0	0	1
1	0	0	0	0	1
0	0	0	0	0	1
0	0	0	0	0	0

0	0	0	0	0	0
0	0	1	0	0	1
0	0	1	0	0	1
0	0	1	0	0	1
1	0	0	0	0	0
0	0	0	0	0	0
1	1	0	0	0	1
0	0	0	0	0	0
1	0	1	0	0	1
1	0	1	0	0	1
1	0	1	0	1	1
1	1	0	0	0	1
1	0	1	0	0	1
0	1	0	0	0	1
0	0	0	0	1	1
0	1	0	0	0	1
0	1	0	0	0	1
1	1	0	0	0	1
1	0	0	0	0	1
0	0	1	0	0	1
0	0	0	0	0	1
1	0	1	0	0	1
0	1	0	0	0	1
0	1	0	0	0	1
1	1	0	0	0	1
0	0	1	0	0	1
0	0	1	0	0	1
1	1	0	0	0	1
1	1	0	0	0	1
0	0	0	0	0	1
0	0	1	0	0	1
1	1	0	0	0	1
1	0	1	0	0	1
0	1	0	0	0	1
0	0	0	0	0	1
0	0	0	0	0	1
0	0	0	0	0	0
0	1	0	0	0	1
0	1	0	0	0	1
1	1	0	0	0	1
1	1	0	0	0	1
0	1	0	0	0	1
1	0	1	0	0	1
1	1	0	1	0	1
1	0	1	0	0	0
1	1	0	0	0	1
1	1	0	0	0	1
1	0	1	0	0	1
1	1	0	0	0	1
0	0	1	0	0	1
0	1	0	0	0	1
1	0	1	0	0	1
1	1	0	0	0	1
1	1	0	0	0	1
1	1	0	0	0	1
1	1	0	0	0	1
1	0	1	0	0	1

1	0	0	0	0	1
0	0	1	0	0	1
0	1	0	0	0	1
0	0	0	0	0	0
0	0	1	0	0	0
0	0	1	0	0	0
1	0	1	0	0	0
0	0	1	0	0	0
0	0	1	0	0	0
0	0	1	0	0	1
0	0	1	0	0	1
0	0	0	0	0	0
0	0	1	0	0	1
0	0	0	0	0	0
0	0	1	0	0	0
0	0	0	0	0	1
0	0	1	0	0	1
0	1	0	1	0	1
0	1	0	0	0	1
0	0	0	0	0	0
0	1	0	0	0	1
0	0	0	0	0	1
0	1	0	0	0	1
0	0	1	0	0	0
0	0	0	0	0	0
0	0	0	0	0	0
0	1	0	0	0	1
1	1	0	0	0	1
1	0	1	0	0	1
1	0	1	0	0	1
1	0	1	0	0	0
1	1	0	0	0	1
0	0	1	0	0	1
0	0	1	0	0	1
0	0	1	0	0	1
0	1	0	0	0	1
0	0	0	0	0	0
0	0	0	0	0	1
0	0	0	0	0	0
1	1	0	0	0	1
0	0	1	0	0	1
0	0	1	0	0	0
0	1	0	0	0	1
0	0	1	0	0	1
1	1	0	0	0	0
1	0	1	0	0	1
0	1	0	0	0	1
0	0	1	0	0	1
0	1	0	0	0	0
1	0	1	0	0	0
1	0	1	0	0	1
1	1	0	0	0	1
0	0	1	0	0	1
1	0	1	0	0	1
1	1	0	0	0	1
0	1	0	0	0	1
1	1	0	1	0	1

1	1	0	0	0		1
1	0	0	1	0		1
0	0	1	0	0		1
0	0	1	0	0		0
1	0	1	0	0		1
0	1	0	0	0		1
0	0	0	0	0		1
1	0	1	0	0		1
1	0	1	0	0		1
0	1	0	0	0		1
1	1	0	0	0		1
0	0	0	0	0		1
0	0	0	0	0		0
0	1	0	0	0		1
0	0	1	0	0		1
1	0	1	0	0		1
1	0	1	0	0		1
1	0	0	1	0		1
0	0	1	0	0		1
1	1	0	0	0		1
1	0	1	0	0		1
1	0	0	0	0		1
1	0	1	0	0		1
1	0	0	0	0		0
1	0	1	0	0		0
0	0	1	0	0		0
0	0	1	0	0		1
1	0	0	0	0		1
1	1	0	0	0		1
1	0	1	0	0		1
1	1	0	0	0		1
1	1	0	0	0		1
1	1	0	0	0		1
1	0	0	0	0		1
1	1	0	0	0		1
1	1	0	0	0		1
1	0	1	0	0		0
1	0	0	0	0		1
0	0	1	0	0		1
0	0	1	0	0		1
1	1	0	0	0		1
1	0	0	0	0		1
1	0	0	0	0		1
1	1	0	0	0		1
0	1	0	0	0		1
1	0	0	1	0		1
1	0	0	0	0		1
0	1	0	0	0		1
1	0	1	0	0		1
1	0	1	0	0		1
1	0	1	0	1		1
1	1	0	1	0		1
1	1	0	0	0		1
0	1	0	0	0		1
1	1	0	0	0		0
1	0	1	0	0		1
1	0	0	0	0		1

0	1	0	0	0	1
1	1	0	0	0	0
1	0	1	0	0	0
1	0	0	0	0	0
1	0	0	0	0	0
0	0	1	0	0	1
1	0	1	0	0	1
1	1	0	0	0	1
0	1	0	0	0	1
1	0	1	0	0	0
0	0	1	0	0	1
0	1	0	1	0	1
0	0	1	0	0	1
0	1	0	0	0	1
1	0	1	0	0	1
0	1	0	0	0	1
1	0	1	0	0	1
0	1	0	0	0	1
0	1	0	0	0	1
0	1	0	0	0	1
0	1	0	0	0	1
0	1	0	0	0	1
0	0	1	0	0	1
0	1	0	0	0	1
0	0	0	0	0	1
1	0	0	0	0	1
0	0	0	0	0	1
1	0	0	0	0	0
0	0	0	0	0	0
0	0	1	0	0	1
0	0	1	0	0	1
1	0	1	0	0	1
0	0	1	0	0	1
0	0	0	0	0	1
0	0	1	0	0	0
1	0	0	0	0	0
0	0	1	0	0	1
0	0	0	0	0	0
0	1	0	0	0	0
0	0	0	0	0	1
0	0	0	0	0	1
1	0	1	0	0	0
1	0	0	0	0	0
1	0	1	0	0	1
0	1	0	0	0	1
0	1	0	0	0	1
0	0	1	0	0	0
0	0	0	0	0	1
0	1	0	0	0	1
0	0	1	0	0	1
0	1	0	1	0	1
1	0	1	0	0	1
0	0	0	0	0	1
0	0	0	0	0	1
0	0	1	0	0	0
0	0	0	0	1	1
1	0	0	0	0	0

1	0	0	0	1	1
0	0	0	0	0	1
1	1	0	0	0	1
0	0	1	0	0	1
0	0	0	0	0	0
0	1	0	0	0	1
1	0	0	0	0	1
1	1	0	0	0	1
1	1	0	0	0	1
1	0	1	0	0	1
1	0	1	0	0	1
0	1	0	0	0	1
0	1	0	0	0	1
1	0	0	0	0	1
1	0	0	0	0	1
1	1	0	0	0	1
1	0	1	0	0	1
0	1	0	0	0	1
0	1	0	0	0	1
1	0	1	0	0	0
1	0	1	0	0	0
1	0	0	0	0	1
0	0	0	0	0	0
0	1	0	0	0	1
0	0	1	0	0	0
0	0	1	0	0	0
0	0	1	0	0	1
1	0	0	0	0	1
1	0	0	0	0	1
0	1	0	0	0	1
0	0	1	0	0	1
0	1	0	0	0	1
0	0	1	0	0	1
0	0	0	0	0	0
0	0	1	0	0	1
0	1	0	0	0	1
0	0	0	0	0	0
0	0	0	0	0	0
0	0	1	0	0	1
1	0	1	0	0	1
1	0	1	0	0	0
1	1	0	0	0	1
1	1	0	0	0	1
1	0	0	0	0	1
1	0	1	1	0	1
1	1	0	0	0	1
0	1	0	0	0	0
0	1	0	0	0	1
0	1	0	1	0	1
0	0	1	0	0	0
0	0	0	0	0	1
0	1	0	0	0	1
1	1	0	0	0	0
0	0	1	0	0	1
0	1	0	0	0	0
1	1	0	0	0	1
0	0	1	0	0	0

0	1	0	0	0	1
1	0	1	0	0	0
0	1	0	0	0	1
0	1	0	0	0	1
0	0	0	0	0	1
1	1	0	0	1	1
0	0	1	0	0	1
0	0	1	0	0	1
0	0	0	0	0	1
0	0	1	0	0	0
0	0	1	0	0	0
1	0	0	0	0	0
0	0	1	0	0	1
1	1	0	0	0	0
1	1	0	0	0	1
0	0	0	0	0	0
1	0	0	0	0	1
0	1	0	0	0	1
0	1	0	0	0	1
0	0	1	0	0	1
0	0	1	0	0	1
0	0	1	0	0	1
0	0	0	0	0	1
0	0	0	0	0	0
1	1	0	0	0	0
0	0	1	0	0	1
0	1	0	0	0	1
1	1	0	1	0	1
1	0	0	0	0	1
1	1	0	0	0	0
0	0	0	0	0	1
0	0	0	0	0	0
1	1	0	0	0	1
0	1	0	0	1	1
0	1	0	0	0	0
0	0	1	0	0	0
0	0	1	0	0	0
1	1	0	0	0	1
0	0	0	0	0	0
1	0	0	0	0	1
1	1	0	0	0	1
0	1	0	0	0	1
0	0	0	0	0	0
0	1	0	0	0	1
0	1	0	0	0	1
0	0	1	0	0	0
0	1	0	0	0	1
0	1	0	0	0	1
1	0	1	0	0	1
0	1	0	0	0	1
1	0	1	0	0	1
1	0	1	0	0	1
0	1	0	0	0	1
1	1	0	0	0	1
1	0	0	0	0	1
0	1	0	0	0	1
1	0	1	0	0	1

1	1	0	0	0	1
0	0	0	0	0	1
0	1	0	0	0	1
0	0	1	0	0	1
0	0	0	0	1	1
0	1	0	0	0	1
0	1	0	0	0	1
0	1	0	1	0	1
0	0	0	0	0	1
0	0	1	0	0	1
0	1	0	0	0	1
0	0	1	0	0	0
0	0	0	0	0	1
0	1	0	0	0	1
0	0	0	0	0	1
0	0	0	0	0	1
0	0	1	0	0	1
0	0	1	0	0	0
0	1	0	0	0	0
0	0	0	0	0	0
0	0	1	0	0	1
0	0	1	0	0	1
1	0	0	0	0	1
1	0	1	0	0	1
0	1	0	0	0	1
1	0	1	0	0	1
1	1	0	0	0	1
0	0	1	0	0	1
1	1	0	0	0	1
0	1	0	0	0	1
0	0	1	0	0	0
1	0	1	0	0	1
0	1	0	0	0	1
1	0	1	0	0	0
1	1	0	0	0	1
0	1	0	0	0	1
0	1	0	0	0	1
0	0	0	0	0	1
0	1	0	1	0	1
0	0	0	0	0	1
1	1	0	0	0	1
0	0	0	0	0	0
0	0	0	0	0	0
1	0	1	0	0	1
1	0	1	0	0	1
1	0	0	0	0	0
0	0	1	0	0	1
1	0	1	0	0	0
0	0	0	0	0	1
0	0	0	0	0	0
1	1	0	0	0	1
0	0	1	0	0	1
0	1	0	0	0	1
0	1	0	0	0	1
1	0	1	0	0	0
0	0	1	0	0	1
0	1	0	0	0	1

0	0	0	0	0	0
1	1	0	0	0	1
1	1	0	0	0	1
1	0	1	0	0	1
0	0	0	0	0	0
0	0	1	0	0	1
0	0	1	0	0	1
1	0	1	0	0	0
1	1	0	0	0	1
0	0	1	0	0	0
0	0	1	0	0	1
0	1	0	0	0	1
1	1	0	0	0	1
0	1	0	0	0	1
0	0	1	0	0	0
1	1	0	0	0	0
1	0	1	0	0	0
1	0	1	0	0	1
0	1	0	0	0	1
0	0	0	0	0	1
0	1	0	0	0	1
1	1	0	0	0	1
1	1	0	0	0	1
0	1	0	1	0	1
0	0	1	0	0	0
0	0	1	0	0	1
0	0	0	0	0	0
0	0	0	0	0	0
1	0	1	0	0	1
0	1	0	0	0	1
0	1	0	1	0	1
0	1	0	0	0	1
1	1	0	0	0	1
0	1	0	0	0	1
0	0	0	0	0	0
0	1	0	0	0	0
0	1	0	0	0	0
0	0	1	1	0	1
0	0	1	0	0	1
0	0	0	0	0	1
0	0	1	0	0	0
0	0	0	0	0	0
0	0	1	0	0	1
0	0	1	0	0	1
1	0	1	0	0	1
1	1	0	0	0	1
0	0	1	0	0	1
0	0	1	0	0	1
0	1	0	0	1	1
1	0	1	0	0	1
1	1	0	0	0	0
0	0	1	0	0	1
0	0	0	0	0	0
0	0	1	0	0	0
0	0	0	0	0	0
1	0	0	0	0	0
1	0	0	0	0	1

0	0	1	0	0	0
0	0	0	0	0	0
0	0	0	0	0	1
1	0	1	0	0	1
1	0	0	0	0	1
0	0	0	0	0	0
0	1	0	0	0	1
0	1	0	0	0	1
0	1	0	0	0	1
0	0	1	0	0	1
0	1	0	0	0	1
1	1	0	0	0	1
0	0	0	0	0	0
0	0	0	0	0	1
0	1	0	0	0	0
0	0	0	0	0	1
0	0	0	0	0	0
0	0	1	0	0	0
1	0	0	0	0	0
0	1	0	0	0	1
0	0	0	0	0	0
0	0	1	0	0	0
0	1	0	0	0	1
0	1	0	0	0	1
1	1	0	0	0	1
1	0	1	0	0	1
1	0	1	0	0	1
0	1	0	0	0	1
0	0	1	0	0	1
1	1	0	0	0	1
1	1	0	0	0	1
0	0	1	0	0	1
0	0	0	0	0	1
0	0	0	0	0	1
1	0	0	0	0	0
0	0	0	0	0	1
0	1	0	0	0	1
1	0	1	0	0	1
1	1	0	0	0	0
1	0	0	0	1	1
0	0	0	0	0	1
1	0	0	0	0	0
1	1	0	0	0	0
1	1	0	0	0	1
1	0	0	0	0	0
0	0	0	0	0	0
0	1	0	0	0	1
0	0	0	0	0	1
0	0	1	0	0	1
0	0	0	0	0	1
1	0	0	0	0	1
1	1	0	0	0	1
0	1	0	0	0	1
1	1	0	0	0	1
1	1	0	0	0	1
1	0	0	0	0	0
1	1	0	0	0	1

1	0	0	0	0	1
1	0	0	0	0	0
1	0	0	0	0	0
1	0	0	0	0	0
1	0	0	0	0	0
1	0	1	0	0	0
0	0	1	0	0	1
0	0	1	0	0	1
0	0	1	0	0	1
0	0	1	0	0	0
0	0	1	0	0	1
0	1	0	0	0	1
0	1	0	0	0	1
1	0	1	0	0	0
0	1	0	0	0	1
0	1	0	0	0	0
0	0	1	0	0	1
1	1	0	1	0	1
1	0	1	0	0	1
1	1	0	0	0	1
1	0	1	0	0	0
0	1	0	0	0	1
0	1	0	1	0	1
1	1	0	0	0	0
0	0	1	0	0	0
1	0	0	0	0	1
0	0	1	0	0	1
0	0	1	0	0	0
1	1	0	0	0	0
1	0	1	0	0	1
0	1	0	0	0	1
1	0	1	0	0	1
0	0	0	0	0	0
0	1	0	0	0	1
1	0	1	1	0	1
1	1	0	0	0	1
0	1	0	0	0	1
1	0	1	0	0	1
1	0	0	0	0	1
0	1	0	0	0	1
0	0	1	0	0	1
1	0	0	0	0	0
1	1	0	1	0	1
0	1	0	0	0	1
0	0	1	0	0	1
1	0	1	0	0	1
0	0	0	0	0	0
0	0	1	0	0	0
1	1	0	0	0	1
0	0	1	0	0	1
0	1	0	0	0	1
0	1	0	0	0	1
0	1	0	0	0	1
0	0	0	0	0	0
0	0	0	0	0	0
0	1	0	0	0	1
0	1	0	1	0	1

0	1	0	0	0	1
0	1	0	0	0	1
1	0	1	0	0	0
0	1	0	0	0	1
0	0	1	0	0	1
0	0	1	0	0	1
0	0	1	0	0	0
1	0	0	0	0	1
0	1	0	1	0	1
0	0	0	0	0	0
0	1	0	0	0	1
0	1	0	0	0	1
1	0	1	0	0	0
0	1	0	0	0	1
0	0	1	0	0	0
0	0	0	0	0	1
0	0	0	0	0	0
0	0	0	0	0	1
0	0	0	0	0	0
0	0	1	0	0	1
0	0	1	0	0	0
0	0	1	0	0	0
0	0	1	0	0	0
0	0	1	0	0	1
0	1	0	0	0	1
0	0	0	0	0	0
0	1	0	0	1	1
0	0	0	0	0	0
1	0	0	0	0	0
1	0	0	0	0	1
0	0	1	0	0	1
0	0	1	0	1	1
0	0	1	0	0	0
0	1	0	0	0	1
0	0	0	0	0	0
1	0	1	0	0	0
0	0	0	0	0	0
0	0	0	0	0	1
0	0	0	0	0	0
0	0	0	0	1	1
0	0	0	0	0	1
1	1	0	0	0	0
1	1	0	1	0	1
0	0	1	0	0	1
1	1	0	0	0	1
1	1	0	0	0	1
1	0	1	0	0	0
0	1	0	0	0	1
1	0	0	0	0	1
0	1	0	0	0	1
1	1	0	0	0	1
0	0	0	0	0	0
1	0	0	0	0	1
0	0	1	0	0	1
0	0	1	0	0	1
0	0	0	0	0	0
0	0	0	0	0	1

0	0	0	0	0	1
0	0	1	0	0	1
0	0	1	0	0	0
0	1	0	1	0	1
0	0	1	0	0	1
1	1	0	1	0	1
0	0	1	0	0	1
1	1	0	0	0	1
1	0	0	0	0	1
0	0	1	0	0	1
1	0	1	0	0	0
0	0	1	0	0	1
1	0	1	0	0	0
1	0	1	0	0	1
0	1	0	0	0	1
1	0	1	1	0	1
1	0	0	0	0	1
1	0	1	0	0	0
0	0	1	0	1	1
1	0	1	1	0	1
0	0	0	0	0	0
0	0	0	0	0	0
1	1	0	0	0	1
1	1	0	0	0	1
1	1	0	0	0	1
0	0	1	0	0	0
0	0	1	0	0	0
0	0	0	0	0	0
0	1	0	0	0	1
0	1	0	0	0	1
0	0	1	0	0	1
0	0	1	0	0	1
1	0	0	0	0	1
1	1	0	0	0	1
1	0	0	0	0	1
1	0	1	0	0	0
0	0	1	0	0	0
0	0	1	0	0	1
0	0	0	0	0	1
0	0	1	0	0	1
0	1	0	0	0	1
0	0	0	0	0	0
0	0	0	0	0	0
0	0	1	0	0	0
0	1	0	0	0	1
1	0	0	0	0	0
0	0	0	0	0	0
1	0	1	0	0	0
1	1	0	0	0	1
0	1	0	0	0	1
1	1	0	0	0	1
1	1	0	0	0	0
0	1	0	0	0	1
0	1	0	0	0	1
0	1	0	0	0	1
0	1	0	0	0	1
1	1	0	0	0	1

1	1	0	0	0	1
0	0	1	0	0	1
0	0	1	0	0	1
1	1	0	0	0	1
0	1	0	0	0	0
0	0	1	0	0	1
0	0	1	0	0	1
1	0	0	0	0	1
0	0	1	0	0	1
0	1	0	0	0	1
0	1	0	0	0	1
0	1	0	0	0	1
0	0	1	0	0	1
0	1	0	0	0	1
0	0	1	0	0	0
1	0	0	0	0	1
0	1	0	0	0	0
0	0	0	0	0	1
0	1	0	0	0	1
1	1	0	0	0	1
1	0	1	0	0	1
0	1	0	0	0	1
0	0	1	0	0	1
0	0	1	0	0	1
1	1	0	0	0	1
1	0	0	0	0	0
0	0	1	0	0	1
0	1	0	0	0	1
1	0	1	0	0	0
0	0	0	0	0	0
0	0	0	0	0	0
0	0	1	0	0	0
0	0	0	0	0	0
0	0	1	0	0	1
1	0	0	0	0	0
0	0	1	0	0	1
1	1	0	0	0	1
1	1	0	0	0	1
0	1	0	0	0	1
1	0	1	0	0	0
1	1	0	0	0	0
0	0	1	0	0	1
1	0	1	0	0	1
0	1	0	0	0	1
1	0	1	0	0	1
1	1	0	0	0	1
1	0	0	0	0	0
0	1	0	0	0	1
1	0	1	0	0	1
1	1	0	0	0	1
0	0	0	0	0	1
0	0	0	0	0	1
0	0	1	0	0	0
1	0	1	0	0	0
0	0	0	0	0	0
0	0	1	0	0	0
0	0	0	0	0	0

0	0	1	0	0	0
1	0	1	0	0	0
1	0	1	0	0	0
0	0	1	0	0	1
1	0	1	0	0	0
1	0	1	0	0	1
1	1	0	0	0	1
1	0	1	0	0	1
0	0	0	0	0	1
0	0	0	0	0	0
1	1	0	0	0	1
0	0	1	0	0	0
0	0	1	0	0	1
0	0	0	0	0	1
0	0	1	0	0	1
1	0	1	0	0	1
0	1	0	0	0	1
1	1	0	0	0	1
0	0	0	0	0	1
1	1	0	0	0	1
0	0	0	0	0	1
0	0	0	0	0	1
0	0	1	0	0	0
0	0	1	0	0	1
1	0	1	0	0	1
1	0	0	0	0	0
1	0	1	0	0	0
1	0	0	0	0	0
0	1	0	0	0	1
0	1	0	1	0	1
1	0	1	0	0	1
1	0	1	0	0	1
0	1	0	0	0	1
1	0	0	1	0	1
1	1	0	0	0	1
1	1	0	0	0	1
1	1	0	0	0	1
1	0	0	0	0	1
0	0	1	0	0	1
0	0	0	0	0	0
0	0	1	0	0	1
0	0	1	0	0	1
0	1	0	0	0	1
0	0	1	0	0	1
0	0	1	0	0	1
1	1	0	0	0	1
1	0	0	0	0	0
0	0	0	0	0	0
0	1	0	0	0	1
0	1	0	0	0	1
0	0	0	0	0	1
0	0	1	0	0	1
1	1	0	0	0	1
1	0	1	0	0	0
0	0	1	0	0	1
0	1	0	0	0	1
0	1	0	0	0	1

1	0	1	0	0	0
0	0	1	0	0	0
0	1	0	0	0	1
0	0	0	0	0	0
1	0	0	0	0	0
0	1	0	0	0	1
0	0	1	0	0	0
0	0	0	0	0	0
1	0	0	0	0	0
0	1	0	0	0	1
0	0	0	0	0	0
1	1	0	0	0	1
1	1	0	0	0	1
0	0	1	0	0	0
0	1	0	0	0	1
0	1	0	0	0	1
1	1	0	0	0	1
1	1	0	0	0	1
1	1	0	0	0	1
0	1	0	0	0	1
1	0	1	0	0	1
1	0	0	0	0	0
1	0	1	0	0	1
1	0	1	0	0	1
1	1	0	0	0	1
1	0	1	0	0	1
1	0	1	0	0	1
1	0	1	0	0	1
0	1	0	0	0	1
1	1	0	0	0	1
0	0	1	0	0	0
1	1	0	0	0	1
0	0	1	0	0	1
1	1	0	0	0	0
1	0	0	0	0	0
1	1	0	0	0	1
0	1	0	0	0	1
1	0	1	0	0	1
0	0	0	0	0	1
0	1	0	1	0	1
0	1	0	0	0	1
0	0	1	0	0	1
0	0	1	0	0	1
0	1	0	0	0	1
0	0	1	0	0	0
0	0	1	0	0	1
0	0	1	0	0	0
1	0	1	0	0	1
0	0	1	0	0	1
0	0	1	0	0	1
0	0	0	0	0	1
1	0	1	0	0	1
0	0	0	0	0	1
1	1	0	0	0	1
1	1	0	0	0	1
1	0	0	0	0	0
1	0	1	0	0	0

0	0	0	0	0	1
1	0	1	0	0	1
1	0	1	0	0	1
0	0	0	0	0	1
0	0	1	0	0	0
1	1	0	1	0	1
1	1	0	0	0	1
1	0	1	0	0	0
0	0	0	0	0	0
1	0	1	0	0	1
1	1	0	0	0	1
0	0	0	0	0	1
0	0	1	0	0	0
0	0	0	0	0	1
1	0	1	0	0	1
0	1	0	0	0	1
0	1	0	0	0	1
0	1	0	0	0	1
0	1	0	1	0	1
0	0	1	0	0	1
1	1	0	0	0	1
0	1	0	0	0	1
0	0	0	0	0	1
0	0	1	0	0	0
0	0	1	0	0	1
0	1	0	0	0	1
0	1	0	0	0	1
0	0	0	0	0	1
0	0	0	0	0	0
1	0	1	0	0	0
0	0	0	0	0	1
1	1	0	1	0	1
1	0	1	0	0	1
1	0	1	0	0	1
0	0	1	0	0	1
1	0	1	0	0	1
1	0	1	0	0	1
1	0	0	0	0	1
1	0	1	1	0	1
0	0	1	0	0	0
1	0	0	0	0	0
1	0	1	0	0	0
0	0	0	0	0	1
0	1	0	1	0	1
0	0	1	0	0	1
1	0	1	0	0	1
1	0	0	0	0	1
0	0	0	0	0	0
0	0	1	0	0	0
0	0	1	0	0	1
0	1	0	0	0	1
0	1	0	0	0	1
0	1	0	0	0	1
1	0	0	0	0	0
1	1	0	0	0	0
0	0	0	0	0	1
1	0	0	0	0	0

1	1	0	0	0	1
0	0	1	0	0	1
1	0	0	0	0	1
1	0	1	0	0	1
1	1	0	0	0	1
0	1	0	0	0	1
0	1	0	0	0	1
1	1	0	0	0	1
0	0	0	0	0	0
1	0	1	0	0	1
0	0	0	0	0	0
1	0	0	0	0	1
1	0	1	0	0	0
1	0	1	0	0	0
1	0	1	0	0	1
0	1	0	0	0	1
1	1	0	0	0	1
1	1	0	0	0	1
0	0	0	0	0	0
0	0	1	0	0	1
0	0	0	0	0	0
0	1	0	0	0	1
0	1	0	0	0	1
0	0	0	0	0	1
0	0	0	0	1	1
0	0	0	0	0	0
1	0	1	0	0	1
0	1	0	0	0	1
0	0	1	0	0	0
0	0	1	0	0	0
0	1	0	0	0	1
0	0	0	0	0	1
0	0	1	0	0	1
0	0	0	0	0	1
0	1	0	0	0	1
1	0	0	0	0	1
0	0	1	0	0	1
0	0	0	0	0	0
1	0	0	0	0	0
1	0	0	0	0	0
0	0	0	0	0	0
0	1	0	0	0	1
1	0	1	0	0	1
1	0	1	0	0	1
1	0	0	0	0	1
1	0	0	0	0	1
1	0	0	0	0	1
1	0	0	0	0	0
1	0	1	0	0	0
0	0	1	0	0	1
0	0	1	0	0	1
1	0	0	0	1	1
1	1	0	0	0	0
0	0	0	0	0	1
0	0	1	0	0	1
1	0	1	0	0	1
1	0	0	0	0	1

0	1	0	0	0	1
1	0	1	0	0	0
0	0	0	0	0	0
0	1	0	0	0	1
0	1	0	0	0	1
1	1	0	0	0	1
0	0	1	0	0	0
0	1	0	0	0	1
0	1	0	0	0	1
0	1	0	0	0	1
0	1	0	0	0	1
1	1	0	0	0	1
1	0	0	0	0	0
0	0	0	0	0	1
1	1	0	0	0	1
1	0	0	0	0	0
1	0	0	0	0	1
1	1	0	0	0	1
1	0	0	0	1	1
1	0	1	0	0	1
1	0	1	0	0	1
1	1	0	0	0	1
0	1	0	0	0	1
0	0	0	0	0	1
0	1	0	0	0	1
0	0	0	0	0	0
0	0	1	0	0	1
0	0	1	0	0	1
0	0	1	0	0	1
0	0	1	0	0	1
0	0	1	0	0	1
0	0	0	0	0	1
1	1	0	0	0	0
1	1	0	0	0	1
0	1	0	0	0	1
1	0	0	0	0	0
1	1	0	0	0	1
0	0	0	0	0	1
0	0	0	0	0	0
1	1	0	0	0	1
0	0	1	0	0	1
0	0	0	0	0	0
1	0	0	0	0	1
1	0	1	0	0	0
1	0	0	0	0	0
0	1	0	0	0	1
0	1	0	0	0	1
0	1	0	0	0	1
0	0	0	0	0	0
1	0	1	0	0	1
1	0	1	0	1	1
1	1	0	1	0	1
0	0	0	0	0	1
0	0	0	0	0	1
0	0	0	0	0	0
0	0	0	0	0	1
0	1	0	0	0	1

1	0	1	0	0	1
1	0	1	0	0	1
1	0	0	0	0	0
0	0	1	0	0	1
1	0	1	0	0	1
1	0	0	0	0	0
1	1	0	0	0	1
0	1	0	0	0	1
0	1	0	0	0	1
0	1	0	0	0	1
1	0	0	0	0	1
1	0	0	0	0	1
1	0	1	0	0	1
0	0	1	0	0	0
0	0	0	0	0	1
0	0	0	0	0	0
0	0	1	0	0	1
1	0	1	0	0	1
1	1	0	0	0	1
0	0	0	0	0	1
0	0	0	0	1	1
0	0	1	0	0	1
1	0	1	0	0	1
0	0	1	0	0	1
1	1	0	0	0	1
0	1	0	0	0	1
0	0	1	0	0	1
1	0	0	0	0	1
0	1	0	0	0	1
0	1	0	0	0	1
1	1	0	0	0	1
1	0	0	0	0	1
0	1	0	1	0	1
0	0	0	0	0	1
0	0	1	0	0	1
0	0	0	0	0	1
0	0	0	0	0	1
0	0	1	0	0	0
0	0	0	0	0	1
0	0	0	0	0	0
0	0	1	0	0	1
0	1	0	0	0	1
0	0	1	0	0	0
0	0	1	0	0	0
1	0	0	0	1	1
0	0	1	0	0	1
1	1	0	0	0	1
0	0	0	0	0	0
0	0	0	0	0	1
0	0	1	0	0	0
0	0	0	0	0	0
1	0	1	0	0	1
1	1	0	1	0	1
1	0	1	0	0	1
1	0	0	0	0	1
1	0	1	0	0	1
0	0	0	0	0	1

0	1	0	0	0	1
1	0	1	0	0	0
0	1	0	0	0	1
0	1	0	0	0	1
1	1	0	0	0	1
0	0	0	0	0	1
0	0	1	0	0	1
0	0	0	0	0	1
0	1	0	0	0	1
1	1	0	0	0	1
0	0	1	0	0	1
0	1	0	0	0	1
1	0	1	0	0	0
1	0	1	0	0	1
1	1	0	0	0	1
1	0	1	0	0	1
1	0	0	0	0	1
1	0	0	0	0	0
1	0	0	0	0	1
0	0	1	0	0	1
1	0	1	0	0	1
1	1	0	0	0	1
0	1	0	1	0	1
0	0	0	0	0	1
0	0	0	0	0	0
0	0	0	0	0	0
0	0	0	0	0	0
0	0	1	0	0	1
1	0	0	0	0	0
0	1	0	0	0	1
0	0	0	0	0	0
0	1	0	0	0	1
1	0	0	0	0	0
0	1	0	0	0	1
1	0	0	0	0	1
1	1	0	0	0	1
1	1	0	0	0	1
0	0	0	0	0	1
0	0	0	0	0	0
0	0	0	0	0	0
1	0	0	0	0	1
0	0	0	0	0	1
0	0	0	0	0	1
0	0	1	0	0	1
0	0	1	0	0	1
0	1	0	0	0	0
0	0	0	0	0	1
0	1	0	0	0	1
0	1	0	0	0	1
1	1	0	0	0	1
1	0	1	0	0	1
1	0	1	0	0	0
1	0	1	0	0	0
1	0	0	0	0	1
1	0	1	0	0	0
0	0	0	0	0	1
0	0	1	0	0	0

0	0	1	0	0	1
0	0	0	0	0	1
0	0	0	0	0	0
0	0	0	0	0	1
0	1	0	0	0	1
1	0	1	0	0	1
0	1	0	0	0	1
0	1	0	0	0	1
0	0	1	0	0	0
1	1	0	0	0	1
0	1	0	0	0	1
0	1	0	0	0	1
0	1	0	0	0	1
0	0	1	0	0	1
0	0	0	0	0	0
0	0	0	0	0	1
1	0	1	0	0	0
0	0	0	0	0	1
0	0	0	0	0	1
0	1	0	0	0	1
0	0	0	0	0	0
0	0	1	0	0	1
0	0	1	0	0	0
0	0	0	0	0	0
0	1	0	0	0	1
0	0	1	0	0	1
1	0	0	0	0	1
1	1	0	0	0	1
0	1	0	0	0	1
0	1	0	0	0	1
0	0	1	0	0	1
0	0	0	0	0	1
0	0	0	0	0	0
0	0	1	0	0	1
0	0	1	0	0	0
1	0	0	0	1	1
1	1	0	0	0	1
0	1	0	0	0	1
0	1	0	0	0	1
1	1	0	0	0	1
1	1	0	0	0	1
0	0	1	0	1	1
1	0	0	0	0	1
0	0	0	0	0	1
1	1	0	1	0	1
1	1	0	0	0	1
0	1	0	0	0	1
1	1	0	0	0	1
1	0	1	0	0	0
0	0	1	0	0	0
0	0	0	0	0	1
0	0	1	0	0	1
0	0	0	0	0	1
0	0	0	0	0	0
0	1	0	0	0	1
0	1	0	0	0	1
0	0	0	0	0	0

0	0	0	0	0	1
0	1	0	0	0	1
1	0	0	0	0	1
0	1	0	0	0	1
0	0	0	0	0	1
0	0	1	0	0	0
0	1	0	0	0	1
1	1	0	0	0	1
0	0	0	0	0	1
0	1	0	0	0	1
0	0	1	0	0	1
1	1	0	0	0	1
0	0	1	0	0	0
0	0	1	0	0	0
1	0	1	0	0	1
1	0	1	0	0	1
1	1	0	0	0	1
0	0	1	0	0	1
1	0	0	0	0	1
0	0	0	0	0	1
1	0	0	0	0	0
0	0	1	0	0	1
0	0	1	0	0	1
0	0	0	0	0	1
0	0	0	0	0	0
0	0	0	0	0	0
1	0	0	0	0	0
1	0	0	0	0	0
0	0	1	0	0	1
0	1	0	0	0	1
1	0	0	0	0	1
1	0	0	0	0	1
0	1	0	0	0	1
0	0	1	0	0	1
0	1	0	0	0	1
0	1	0	0	0	1
1	0	1	0	0	1
1	0	0	0	0	1
0	0	0	0	0	1
0	0	1	0	0	1
0	0	1	0	0	1
1	0	1	0	0	1
1	1	0	1	0	1
1	1	0	0	0	1
0	0	0	0	0	0
0	1	0	0	0	1
0	0	1	0	0	1
0	1	0	0	0	1
1	1	0	0	0	1
0	1	0	0	0	1
1	0	1	0	0	1
1	0	1	0	0	1
0	1	0	0	0	1
0	0	0	1	0	1
0	1	0	1	0	1
0	0	0	0	0	0
0	0	1	0	0	0

0	1	0	0	0	0
0	0	1	0	0	0
0	0	0	0	0	0
1	0	0	0	0	0
0	0	1	0	0	1
1	0	1	0	0	0
1	0	1	0	0	1
1	1	0	0	0	1
0	0	1	0	0	1
1	1	0	0	0	0
1	1	0	0	0	1
0	1	0	0	0	1
1	0	1	0	0	1
0	1	0	0	0	1
0	1	0	0	0	1
0	0	1	0	0	0
0	1	0	0	0	1
0	0	0	0	0	0
0	0	1	0	0	1
0	1	0	0	0	1
0	1	0	0	0	1
1	1	0	0	0	0
1	0	0	0	0	0
0	1	0	0	0	1
0	1	0	0	0	1
0	1	0	0	0	1
1	0	0	0	0	0
0	0	0	0	0	0
1	0	0	0	0	0
0	1	0	0	0	0
1	0	1	0	0	0
0	0	1	0	0	0
0	1	0	0	0	1
0	0	1	0	0	1
0	0	1	0	0	1
0	1	0	0	0	1
0	1	0	0	0	1
0	0	1	0	0	1
0	0	1	0	0	1
0	0	0	0	0	1
0	1	0	0	0	1
1	0	1	0	0	1
1	1	0	0	0	1
1	0	0	0	0	1
0	0	0	0	0	0
1	0	1	0	0	1
1	0	0	0	0	0
0	0	1	0	0	1
0	1	0	0	0	1
1	0	1	0	0	1
1	1	0	0	0	1
0	0	0	0	0	0
1	0	1	0	0	0
1	1	0	0	0	1
0	0	1	0	0	1
0	0	0	0	0	0
0	1	0	0	0	1

1	0	1	0	0	1
0	1	0	0	0	1
1	0	1	0	0	1
0	0	0	1	0	1
0	0	1	0	0	0
0	0	0	0	0	0
0	0	0	0	0	0
0	0	0	0	0	0
0	0	0	0	0	0
0	0	1	0	0	0
1	0	0	0	0	0
1	0	0	0	0	1
0	0	1	0	0	0
0	0	1	0	0	1
1	0	0	0	0	1
1	0	0	0	0	0
0	1	0	0	0	1
1	1	0	0	0	1
1	0	0	0	0	1
0	0	0	0	1	1
0	1	0	1	0	1
0	0	0	0	0	0
1	1	0	0	0	1
1	0	1	0	0	1
0	0	0	0	0	0
1	0	0	0	0	1
0	1	0	0	0	0
0	0	0	0	0	1
1	0	1	0	0	1
1	0	1	0	0	1
1	1	0	0	0	1
1	0	1	0	0	0
0	0	0	0	0	0
0	0	0	0	0	1
0	0	1	0	0	1
0	0	0	0	0	1
1	0	1	0	0	1
1	0	0	0	0	0
0	0	0	0	0	0
0	0	1	0	0	1
0	0	0	0	0	0
0	0	0	0	0	0
1	1	0	0	0	0
1	1	0	0	0	1
0	1	0	0	0	1
1	0	0	0	0	0
0	0	1	0	0	0
1	1	0	0	0	1
1	0	0	0	0	1
0	1	0	0	0	1
0	1	0	0	0	1
0	0	0	0	0	0
0	0	1	0	0	1
1	1	0	0	0	1
0	0	0	0	0	1
0	0	1	0	0	1
0	0	0	0	0	0

0	1	0	0	0	1
0	1	0	0	0	0
0	1	0	0	0	1
0	0	0	0	0	1
0	0	1	0	0	1
0	0	1	0	0	1
1	0	1	0	0	1
1	1	0	0	0	1
1	1	0	0	0	1
1	1	0	0	0	1
0	1	0	0	0	0
0	0	1	0	0	0
1	1	0	0	0	1
0	0	1	0	0	1
0	1	0	0	0	1
0	0	0	0	0	0
0	0	0	0	0	0
0	0	0	0	0	1
0	0	1	0	0	0
0	1	0	1	0	1
1	0	1	0	0	1
1	1	0	1	0	1
0	1	0	0	0	1
0	0	1	0	0	0
0	1	0	0	0	1
0	1	0	0	0	0
0	1	0	0	0	1
1	0	1	0	0	1
0	0	1	0	0	1
0	0	1	0	0	1
0	1	0	1	0	1
0	0	0	0	0	1
0	0	0	0	0	1
1	0	0	0	0	0
0	0	1	0	0	0
0	1	0	0	0	1
0	0	0	0	0	1
0	1	0	0	0	1
1	1	0	0	0	1
1	1	0	0	0	1
1	0	1	0	0	1
1	0	1	0	0	1
0	0	0	0	0	1
0	0	0	0	0	0
0	0	1	0	0	1
0	0	1	0	0	1
1	0	1	0	0	1
0	0	1	0	0	0
1	1	0	0	0	1
1	0	0	0	0	1
0	0	1	0	0	0
0	0	0	0	0	1
0	0	0	0	0	0
1	1	0	0	0	0
0	1	0	0	0	0
0	1	0	0	0	0
1	0	0	0	0	1

0	0	0	0	0	0
0	0	1	0	0	1
0	0	1	0	0	1
1	0	1	0	0	0
1	0	1	0	0	1
0	0	0	0	0	0
0	0	1	0	0	0
1	0	1	0	0	0
0	1	0	0	0	1
0	1	0	0	0	1
0	1	0	0	0	1
0	0	0	0	0	1
0	0	0	0	0	0
0	1	0	0	0	1
0	0	0	1	0	1
0	0	1	0	0	1
0	0	0	0	0	1
0	0	1	0	0	1
0	0	0	0	0	1
0	0	1	0	0	1
0	0	1	0	0	1
0	0	0	0	0	0
1	0	0	0	0	0
0	1	0	0	0	1
1	1	0	0	0	1
1	0	1	0	0	1
1	0	1	0	0	0
1	0	0	0	0	0
1	0	0	0	0	0
0	1	0	0	0	1
0	1	0	0	1	1
1	0	1	0	0	0
0	0	0	0	0	0
1	0	1	0	0	1
0	1	0	0	0	1
0	0	0	0	1	1
1	0	0	0	0	0
0	1	0	0	0	1
1	1	0	0	0	1
0	1	0	0	0	1
0	1	0	0	0	1
0	0	1	0	0	1
0	1	0	0	0	1
0	1	0	0	0	1
0	1	0	0	0	1
1	1	0	0	0	1
1	0	0	0	0	1
0	0	1	0	0	0
0	0	0	0	0	0
1	0	1	0	0	1
0	1	0	0	0	1
1	1	0	0	0	1
0	1	0	0	0	1
1	0	1	0	0	0
0	0	1	0	0	1
0	1	0	0	0	1
0	1	0	0	0	1

0	0	1	0	0	1
1	0	0	0	0	0
1	0	1	0	0	1
1	0	0	0	0	0
1	0	1	0	0	1
0	0	1	0	0	1
0	0	0	0	0	1
0	0	0	0	0	1
0	1	0	0	0	1
1	1	0	0	0	1
1	0	0	0	0	0
0	0	1	0	0	0
1	0	0	0	0	1
1	1	0	0	0	1
0	0	0	0	0	1
1	1	0	0	0	1
1	0	0	0	0	1
1	1	0	0	0	1
0	0	0	0	0	0
1	0	1	0	0	1
1	1	0	0	0	1
0	0	1	0	0	1
0	0	0	0	0	0
0	0	0	0	0	1
0	0	0	0	0	0
0	0	1	0	0	0
0	0	0	0	0	1
0	0	1	0	0	1
0	0	0	0	0	1
0	0	0	0	0	1
0	0	0	0	0	1
0	0	0	0	0	1
0	0	0	0	0	1
0	0	1	0	0	1
0	0	0	0	0	1
0	0	0	0	0	1
0	0	0	0	0	0
0	0	1	0	0	1
0	0	1	0	0	1
0	0	0	0	0	1
1	0	0	0	0	0
1	1	0	0	0	0
0	0	0	0	0	1
0	0	1	0	0	1
1	0	1	0	0	1
0	0	1	0	0	1
0	1	0	0	0	1
0	0	1	0	0	1
1	0	1	0	0	1
0	1	0	0	0	1
0	1	0	0	0	1
1	1	0	1	0	1
0	0	1	0	0	1
1	1	0	0	1	1
0	0	1	0	1	1
0	0	1	1	0	0
1	0	1	0	0	0

0	0	0	0	0	0
1	1	0	0	0	1
0	1	0	0	0	1
1	1	0	0	0	1
1	0	1	0	0	1
1	0	1	0	0	1
1	1	0	0	0	1
1	0	0	0	0	1
1	1	0	0	0	1
1	0	1	0	0	1
0	1	0	1	0	1
0	0	0	0	0	0
0	0	0	0	0	1
0	1	0	0	0	1
0	0	0	0	0	0
0	0	1	0	0	1
0	0	0	0	0	0
0	1	0	0	0	1
1	0	0	0	0	0
1	1	0	0	0	1
1	1	0	0	0	1
1	0	0	0	0	1
0	1	0	0	0	1
0	0	1	0	0	0
0	1	0	0	0	0
0	0	0	0	0	0
0	1	0	0	0	0
0	0	0	0	0	0
0	0	1	0	0	1
0	1	0	0	0	0
0	0	0	0	0	1
1	0	1	0	0	0
0	0	1	0	0	1
0	0	0	0	0	1
0	1	0	0	0	1
0	0	1	0	0	0
1	0	0	0	0	0
1	0	0	0	0	0
1	0	1	0	0	1
0	1	0	0	0	1
0	1	0	0	0	1
1	1	0	0	0	1
0	0	1	0	0	1
0	1	0	0	0	1
0	1	0	0	0	1
1	0	0	0	0	0
1	0	0	0	0	1
1	0	0	0	0	0
0	1	0	0	0	1
0	0	0	0	0	0
0	1	0	0	0	1
1	0	1	0	0	1
1	1	0	0	0	1
0	0	0	0	0	1
0	0	0	0	0	0
0	0	1	0	0	1
0	0	0	0	0	1

1	0	0	0	0	1
1	1	0	0	0	1
0	0	1	0	0	1
0	0	0	0	0	0
0	0	0	0	0	0
0	0	1	0	0	0
0	0	1	0	1	1
0	0	1	0	1	1
1	0	1	0	0	0
1	0	0	0	0	0
1	0	0	0	0	0
0	0	0	0	0	1
0	0	0	0	0	0
0	0	0	0	0	0
0	0	0	0	0	0
1	0	1	0	0	1
0	1	0	0	0	1
1	1	0	0	0	1
0	0	0	0	0	1
0	0	0	0	0	1
0	0	1	0	0	1
1	1	0	0	0	1
1	0	1	0	0	1
1	0	0	0	0	0
0	0	0	0	0	1
1	0	1	0	0	1
1	0	0	0	0	0
1	0	0	0	0	0
1	0	1	0	0	1
1	0	1	0	0	0
0	0	1	0	0	1
0	0	0	0	0	1
1	1	0	0	0	1
0	0	1	0	0	1
0	1	0	0	0	1
0	1	0	0	0	1
0	0	0	0	0	0
0	1	0	0	0	1
0	0	0	0	0	1
1	1	0	0	0	0
0	0	1	0	0	1
1	0	0	0	0	0
1	0	0	0	0	0
0	0	0	0	0	0
1	0	0	0	0	0
0	0	0	0	0	1
0	0	0	0	0	1
0	0	0	0	0	0
0	0	1	0	0	1
0	0	1	0	0	1
0	0	0	0	0	1
0	0	0	0	0	0
0	0	1	0	0	0
0	0	1	0	0	1
1	0	1	0	0	1
1	0	1	0	0	1
0	0	1	0	0	1

1	0	1	0	0	1
1	1	0	0	0	1
0	0	1	0	0	1
0	0	1	0	0	0
0	0	0	0	0	1
0	1	0	0	0	1
0	1	0	0	0	1
1	0	1	0	0	1
0	1	0	0	0	1
1	1	0	0	0	1
0	0	1	0	0	1
0	1	0	0	0	1
1	0	1	0	0	1
1	0	0	0	0	1
0	0	0	0	0	1
0	0	0	0	0	0
1	0	1	0	0	1
0	1	0	1	0	1
0	1	0	0	0	1
1	0	1	0	0	0
1	0	0	0	0	1
0	0	0	0	0	0
0	0	0	0	0	1
0	0	0	0	0	1
1	0	1	0	0	1
1	1	0	0	0	0
1	1	0	0	0	1
0	0	1	0	0	1
0	0	1	0	0	1
0	0	1	0	0	1
0	0	0	0	0	1
0	0	1	0	0	1
0	0	1	0	0	1
0	0	0	0	0	1
1	0	0	0	0	1
1	1	0	0	0	1
1	0	0	0	0	1
0	0	0	0	0	1
1	0	0	0	0	1
0	0	0	0	0	1
0	0	0	0	0	0
0	0	1	0	0	1
0	0	0	0	0	1
0	0	0	0	0	0
1	0	0	0	0	1
0	0	0	0	0	1
0	0	1	0	0	1
0	1	0	0	0	1
0	1	0	0	0	1
1	1	0	0	0	1
1	0	0	0	0	1
1	1	0	0	0	1
1	0	1	0	0	0
1	0	1	0	0	1
0	0	1	0	0	1
1	1	0	0	0	1
1	0	0	0	0	1

0	0	0	0	0	0
1	1	0	0	0	1
0	0	0	0	1	1
1	1	0	0	0	1
0	0	0	0	0	1
0	0	1	0	0	1
1	1	0	0	0	1
1	0	1	0	0	1
0	0	1	0	0	0
0	1	0	0	0	1
0	1	0	0	0	1
0	0	0	0	0	1
0	1	0	0	0	1
0	1	0	0	0	1
0	0	0	0	0	0
1	0	0	0	0	1
0	0	0	0	0	0
0	0	0	0	0	0
0	0	1	0	0	0
0	0	0	0	0	0
0	0	1	0	0	0
0	0	0	0	0	0
0	1	0	0	0	1
0	0	0	0	0	1
1	0	0	0	0	1
1	0	1	0	0	1
1	1	0	0	0	1
0	0	1	0	0	0
1	0	1	0	0	1
1	0	1	0	0	1
0	1	0	0	0	1
0	0	1	0	0	0
0	0	1	0	0	1
0	0	0	0	0	0
0	0	1	0	0	1
0	0	1	0	0	1
1	1	0	0	0	1
1	0	1	0	0	1
1	1	0	0	0	1
1	0	0	0	0	1
0	0	0	0	0	1
0	0	0	0	1	1
0	1	0	0	0	0
0	1	0	0	0	1
1	0	1	0	0	1
0	1	0	0	0	1
0	0	0	0	0	1
0	0	1	0	0	1
0	0	1	0	0	1
0	0	1	0	0	0
0	1	0	0	0	1
0	1	0	0	0	1
0	0	0	0	0	1
0	1	0	0	0	1
0	1	0	0	0	1
0	0	0	0	0	1
0	1	0	0	0	1

1	0	1	0	0		1
0	0	1	0	0		1
0	0	1	0	0		1
0	0	0	0	0		1
0	0	1	0	0		1
0	0	0	0	0		1
0	0	1	0	0		1
0	0	1	0	0		1
1	0	0	0	0		1
1	0	0	0	0		1
1	1	0	0	0		0
0	1	0	0	0		1
1	1	0	0	0		1
0	1	0	0	0		1
0	1	0	0	0		1
0	0	0	0	0		0
0	1	0	0	0		1
0	0	1	0	0		0
0	0	0	0	0		1
0	1	0	1	0		1
0	0	1	0	0		1
0	0	0	0	0		1
1	1	0	1	0		1
0	0	1	0	0		1
0	0	0	0	0		1
0	0	0	0	0		0
0	0	0	0	0		0
0	0	0	0	0		1
0	0	1	0	0		1
0	1	0	0	0		1
0	0	0	0	0		1
1	1	0	0	0		1
1	1	0	0	0		1
1	0	1	0	1		1
0	0	1	0	0		0
0	0	1	0	0		0
0	0	0	0	0		1
1	0	1	0	0		1
0	0	1	0	0		1
0	1	0	1	0		1
0	1	0	0	0		1
1	1	0	0	0		1
0	1	0	0	0		1
1	1	0	0	0		1
0	0	0	0	0		1
0	0	1	0	0		1
0	1	0	0	0		0
1	1	0	0	0		1
1	0	1	0	0		1
0	1	0	0	0		1
1	1	0	0	0		1
1	1	0	0	0		1
0	0	1	0	0		1
1	1	0	0	0		1
0	1	0	0	0		1
1	1	0	0	0		1
0	0	0	0	0		1

1	1	0	0	0	1
0	1	0	0	0	1
0	1	0	0	0	1
0	1	0	0	0	1
0	1	0	0	0	1
0	0	1	0	0	0
0	0	0	0	0	0
0	1	0	0	0	1
1	1	0	0	0	1
1	0	0	0	0	1
0	1	0	0	0	1
0	1	0	0	0	1
0	1	0	0	0	1
0	1	0	0	0	0
0	0	1	1	0	1
0	0	0	0	0	0
0	0	0	0	0	0
0	0	0	0	0	0
0	0	0	0	0	0
1	0	1	0	0	0
0	0	0	0	0	0
1	0	1	0	0	0
0	0	1	0	0	0
0	1	0	1	0	1
0	0	1	0	0	1
1	0	1	0	0	1
0	0	0	0	0	0
0	0	1	0	0	1
1	0	1	0	0	0
0	0	0	0	1	1
1	0	1	0	0	0
0	1	0	0	0	1
0	0	1	0	0	1
0	1	0	0	0	1
0	0	1	0	0	1
0	0	0	0	0	0
1	0	0	0	0	1
1	0	1	0	0	1
1	1	0	0	0	1
1	1	0	0	0	1
1	1	0	0	0	1
0	0	0	0	0	1
0	1	0	0	0	1
0	1	0	0	0	1
0	1	0	0	0	1
1	0	0	0	0	1
0	0	0	0	0	1
1	0	0	0	0	1
1	0	1	0	0	1
1	0	1	0	0	1
0	1	0	0	0	1
1	1	0	0	0	1
0	1	0	0	0	1
0	0	0	0	0	1
1	0	0	0	0	0
1	1	0	0	0	1
1	0	1	0	0	1

0	1	0	0	0	1
1	1	0	0	0	1
1	0	0	0	0	1
1	1	0	0	0	1
1	1	0	0	0	1
1	0	1	0	0	1
0	0	0	0	0	1
1	1	0	0	0	1
0	1	0	0	0	1
1	1	0	0	0	1
0	0	1	0	0	1
0	0	0	0	0	0
0	0	0	0	0	0
0	1	0	0	0	1
0	0	0	0	0	1
0	0	0	0	0	0
0	0	1	0	0	0
0	0	0	0	0	0
0	0	1	0	0	0
0	0	1	0	0	1
0	0	1	0	0	1
0	0	0	0	0	1
0	0	1	0	0	1
0	0	0	0	0	1
0	0	1	0	0	1
0	0	1	0	0	1
0	0	0	0	0	0
0	0	1	0	0	1
1	0	1	0	0	1
1	0	0	0	0	1
0	0	1	0	0	1
1	1	0	0	0	1
0	1	0	0	0	1
0	1	0	0	0	1
0	1	0	0	0	1
0	1	0	0	0	1
0	0	1	0	0	1
0	0	1	0	0	0
0	0	0	0	0	0
0	0	1	0	0	0
0	0	1	0	0	0
0	0	0	0	0	0
1	0	1	0	0	0
1	1	0	0	0	1
1	1	0	0	0	1
1	0	1	0	0	1
1	0	0	0	0	1
1	1	0	0	0	1
1	1	0	0	0	1
0	0	1	0	0	1
1	0	0	0	0	0
0	1	0	0	0	1
0	0	1	0	0	0
0	0	0	0	0	0
0	0	0	0	0	1
0	0	0	0	0	0
0	1	0	0	0	1

0	0	1	0	0	1
0	1	0	0	0	1
1	0	1	0	0	1
0	0	0	0	0	0
0	0	1	0	0	0
1	1	0	0	0	1
1	0	1	0	0	0
0	0	0	0	0	0
0	1	0	0	0	1
0	0	0	0	0	0
1	0	1	0	0	0
0	1	0	0	0	1
1	0	1	0	0	1
0	0	0	0	0	1
1	0	0	0	0	1
1	1	0	0	0	1
1	0	1	0	0	1
1	1	0	0	0	1
1	0	0	0	0	1
1	1	0	0	0	1
0	0	1	0	0	1
0	0	0	0	1	1
0	1	0	0	0	1
0	0	1	1	0	1
0	1	0	0	0	1
0	1	0	0	0	1
1	0	0	0	0	0
0	1	0	0	0	1
1	1	0	0	0	1
1	0	1	0	0	1
0	0	0	0	0	1
0	1	0	0	0	1
0	0	0	0	0	0
0	0	1	0	0	0
1	1	0	0	0	1
0	0	1	0	0	0
0	1	0	0	0	1
0	0	0	0	0	1
1	1	0	0	0	1
0	0	0	0	0	0
0	1	0	0	0	1
1	0	0	0	0	1
1	1	0	0	0	1
0	0	1	0	0	1
0	0	1	0	0	1
1	1	0	0	0	1
1	0	0	0	0	1
1	1	0	0	0	1
1	0	1	0	0	1
1	1	0	0	0	1
1	1	0	1	0	0
0	0	1	0	0	0
0	0	1	1	0	1
0	0	0	0	0	0
1	0	1	0	0	1
1	0	0	0	0	1
0	1	0	0	0	1

0	1	0	0	0	1
1	1	0	0	0	0
1	0	0	0	0	0
0	0	1	0	0	0
0	0	1	0	0	1
1	0	1	0	1	1
0	1	0	0	0	1
0	0	1	0	0	1
0	1	0	0	0	1
1	0	1	0	0	1
1	0	1	0	0	1
1	1	0	0	0	1
1	1	0	0	0	1
0	0	0	0	0	1
1	1	0	0	0	1
1	0	1	0	0	1
1	0	0	0	0	0
0	1	0	0	0	1
0	0	1	0	0	0
0	1	0	1	0	1
1	1	0	0	0	1
1	1	0	0	0	1
1	1	0	0	0	1
0	0	1	0	0	1
1	0	0	0	0	0
0	0	0	0	0	1
0	0	0	0	0	0
1	0	0	0	0	1
1	0	0	0	0	0
0	0	1	0	0	1
0	1	0	0	0	1
1	0	0	0	0	0
1	0	0	0	0	1
0	0	1	0	0	1
1	1	0	0	0	0
0	0	0	0	0	1
1	1	0	0	0	1
1	1	0	0	0	1
1	1	0	0	0	1
1	1	0	0	0	1
1	0	1	0	0	1
1	0	1	0	0	0
1	0	0	0	0	1
0	1	0	0	0	1
0	1	0	0	0	1
1	1	0	0	0	1
1	1	0	0	0	1
1	1	0	0	0	1
0	0	0	1	0	1
1	0	1	0	0	1
1	0	1	1	0	1
1	0	0	0	0	0
0	0	1	0	0	0
0	1	0	0	0	1
1	0	1	0	0	1
1	1	0	0	0	1
0	1	0	0	0	1

0	1	0	0	0	1
0	0	1	0	0	1
0	0	1	0	0	1
1	1	0	0	0	1
1	1	0	0	0	1
0	0	1	0	0	1
0	0	1	0	0	1
0	0	1	0	0	0
1	0	0	0	0	1
1	0	0	0	0	0
1	0	0	0	0	1
0	0	0	0	0	1
0	0	1	0	0	1
0	0	1	0	0	1
0	0	0	0	0	0
0	0	0	0	0	0
0	1	0	0	0	0
0	0	0	0	0	0
0	0	0	0	0	0
1	0	0	0	0	0
0	1	0	0	0	1
1	0	0	0	0	0
1	0	1	0	0	1
1	0	1	0	0	1
1	0	1	0	0	1
1	0	0	0	0	1
1	1	0	0	0	1
1	0	1	0	0	1
0	0	0	0	0	1
0	0	0	0	0	0
0	0	0	0	0	0
1	1	0	0	0	1
0	0	1	0	0	1
0	0	0	0	0	1
1	0	0	0	1	1
1	0	1	0	0	1
0	0	1	0	0	1
0	0	0	0	0	1
0	0	1	0	0	1
0	1	0	0	0	1
0	0	1	0	0	0
0	0	0	0	0	1
1	1	0	1	0	1
1	0	0	0	0	1
1	1	0	0	0	1
0	0	0	0	0	1
0	1	0	0	0	1
0	0	0	0	0	0
0	0	1	0	0	0
0	0	1	0	0	1
1	0	0	0	0	0
1	1	0	0	0	1
0	1	0	1	0	1
1	0	0	0	0	1
0	0	0	0	0	1
0	0	1	0	0	1
0	1	0	0	0	1

0	0	1	0	0	1
1	0	0	0	0	0
0	1	0	0	0	1
0	0	0	0	0	1
1	1	0	0	0	1
1	1	0	0	0	1
0	1	0	0	0	0
0	0	1	0	0	0
0	0	0	0	0	1
0	0	0	0	0	1
0	0	1	0	0	1
0	1	0	0	0	1
0	0	1	0	0	1
1	0	0	0	0	1
1	0	1	0	0	1
0	1	0	0	0	1
0	0	1	0	0	1
1	0	1	0	0	1
1	1	0	0	0	1
0	0	1	0	0	1
1	0	0	0	0	1
1	1	0	0	0	1
0	0	1	0	0	1
1	0	1	0	0	0
0	1	0	0	0	0
0	1	0	0	0	1
0	1	0	0	0	1
0	1	0	0	0	1
0	1	0	0	0	1
0	0	1	0	0	1
0	0	1	0	0	0
0	1	0	0	0	1
1	0	0	0	1	1
1	1	0	0	0	1
1	1	0	0	0	1
1	1	0	0	0	1
0	0	0	0	0	1
1	0	1	0	0	1
0	0	0	0	0	0
0	1	0	0	0	1
1	0	1	0	0	0
0	0	0	0	0	0
0	0	1	0	0	1
1	1	0	0	0	1
1	1	0	0	0	1
0	0	0	0	0	0
0	0	0	0	0	1
1	1	0	0	0	1
1	1	0	0	0	1
0	0	0	0	0	1
0	1	0	0	0	1
0	1	0	0	0	1
1	0	1	0	0	0
0	1	0	0	0	1
0	1	0	0	0	1
1	1	0	0	0	1
1	0	1	0	0	1

1	0	1	0	0	1
0	0	0	0	0	1
0	0	0	0	0	0
0	0	1	0	0	1
0	0	0	0	1	1
0	0	1	0	0	0
0	0	1	1	0	1
1	1	0	0	0	1
1	1	0	0	0	1
0	1	0	0	0	1
0	0	1	0	0	0
0	0	1	0	0	0
0	0	1	0	0	0
0	0	0	0	0	0
0	0	1	0	0	1
0	1	0	0	0	0
0	0	1	0	0	0
0	0	0	0	0	1
0	1	0	0	0	1
1	0	1	0	0	0
1	0	0	0	0	0
1	0	0	0	0	0
0	0	1	0	0	0
0	0	1	0	0	0
1	0	0	0	0	1
0	1	0	0	0	1
0	0	0	0	0	1
0	1	0	0	0	1
0	0	1	0	0	0
0	0	1	0	0	1
1	0	1	0	0	1
0	0	1	0	0	1
1	1	0	0	0	1
0	0	0	0	0	1
1	0	0	0	0	0
1	0	0	0	0	1
1	1	0	0	0	1
0	0	1	0	0	1
0	0	1	0	0	0
0	1	0	0	0	1
0	0	1	0	0	0
0	0	0	0	0	0
0	0	0	0	0	0
0	0	1	0	0	1
0	0	1	0	0	1
0	0	1	0	0	1
1	0	0	0	0	1
0	0	1	0	0	0
1	0	1	0	0	0
1	0	0	0	0	1
1	1	0	0	0	1
0	0	0	0	0	0
0	0	0	0	0	0
0	0	0	0	0	1
0	0	0	0	0	1
0	0	0	0	0	0
0	0	0	0	0	0

0	0	0	0	0		0
0	1	0	0	0		1
0	1	0	0	0		1
1	0	0	0	0		0
1	0	0	0	0		1
1	1	0	0	0		1
0	1	0	0	0		1
1	1	0	0	0		1
0	1	0	0	0		1
1	0	1	0	0		0
1	1	0	0	0		1
0	0	0	0	0		0
0	0	1	0	0		0
0	0	0	0	0		0
0	0	1	0	0		0
0	0	1	0	0		1
0	0	1	0	0		1
0	0	0	0	0		0
1	0	0	0	0		0
0	1	0	0	0		1
0	0	1	0	0		1
1	0	0	0	0		1
1	1	0	0	0		1
0	0	1	0	1		1
0	0	0	0	0		1
1	0	0	0	0		1
0	0	0	0	0		0
0	0	0	0	0		0
0	0	1	0	0		1
0	1	0	0	0		0
0	0	0	0	0		1
1	0	1	0	0		1
1	1	0	0	0		1
0	0	1	0	0		0
1	0	0	0	0		0
1	0	0	0	0		0
0	0	0	0	0		0
0	0	1	0	0		0
0	0	0	0	0		0
1	0	1	1	0		1
0	1	0	0	0		1
0	0	1	0	0		1
0	0	0	0	0		1
0	0	1	0	0		0
0	0	0	0	0		0
0	0	0	0	0		1
0	0	0	0	0		0
0	0	0	0	0		0
1	1	0	0	0		1
0	0	1	0	0		0
0	0	0	0	0		0
0	0	1	0	0		0
0	1	0	0	0		0
0	0	1	0	0		0
0	0	0	0	0		0
0	1	0	1	0		1
1	0	0	0	0		0

1	1	0	0	0	1
0	1	0	0	0	1
1	0	1	0	0	1
1	0	1	0	0	0
1	0	1	0	0	1
0	0	0	0	0	0
0	0	0	0	0	0
1	0	1	0	0	0
0	0	1	0	0	1
1	1	0	1	0	1
0	1	0	1	0	1
1	0	1	0	0	1
0	0	1	0	0	1
1	0	0	0	0	1
0	0	1	0	0	0
1	1	0	1	0	1
1	1	0	0	0	1
1	1	0	0	0	1
0	1	0	0	0	1
0	0	0	0	0	1
0	0	1	0	0	1
0	0	1	0	0	1
0	0	0	0	0	1
0	1	0	0	0	1
0	0	1	0	0	1
0	1	0	0	0	1
0	0	1	0	0	1
1	0	1	0	0	0
1	1	0	1	0	1
1	1	0	0	0	1
1	0	1	0	0	1
1	0	1	0	0	1
1	1	0	0	0	1
0	1	0	0	0	1
0	0	1	0	0	1
0	0	0	0	0	0
1	0	1	0	0	0
1	1	0	0	0	1
1	1	0	0	0	1
1	0	1	0	0	1
1	0	1	0	0	1
1	0	1	0	0	1
0	0	1	0	0	1
1	1	0	1	0	1
1	0	1	0	0	1
0	0	0	0	0	1
0	0	0	0	0	1
0	0	0	0	0	0
0	0	1	0	0	0
0	0	0	0	0	0
0	0	0	0	0	1
1	0	1	0	0	1
1	1	0	0	0	1
1	0	1	0	0	0
0	0	1	0	0	1
0	1	0	0	0	1
1	0	1	0	0	1

1	1	0	0	0	1
0	1	0	0	0	1
1	0	0	0	0	1
1	0	1	0	0	1
0	0	1	0	0	1
1	0	1	0	0	0
0	0	0	0	0	1
0	0	0	0	0	1
1	0	0	1	0	1
0	0	0	0	0	1
0	0	0	0	0	0
0	1	0	0	0	1
0	0	0	0	0	1
0	0	1	0	0	1
1	1	0	0	0	1
0	0	1	0	0	0
0	1	0	0	0	1
1	0	1	0	0	1
1	1	0	0	0	1
0	0	0	0	0	1
0	1	0	0	0	1
0	0	0	0	0	1
0	1	0	0	0	1
0	0	1	0	0	0
0	0	1	0	0	1
1	0	1	0	0	1
0	1	0	0	0	1
0	0	1	0	0	1
0	0	1	0	0	0
0	0	0	0	0	1
1	0	0	0	0	0
0	0	0	0	0	0
0	0	0	0	0	0
1	0	0	0	0	0
0	0	0	0	0	1
0	0	1	0	0	1
1	1	0	1	0	1
0	0	0	0	0	0
0	0	0	0	0	0
0	1	0	0	0	1
0	1	0	0	0	1
0	1	0	0	0	1
1	1	0	0	0	1
1	0	1	0	0	1
0	1	0	0	0	1
0	1	0	0	0	1
1	1	0	0	0	1
0	0	0	0	0	0
0	0	1	0	0	1
0	0	0	0	0	1
0	0	1	0	0	1
0	0	0	0	0	1
0	0	0	0	0	1
0	1	0	0	0	1
0	0	0	0	0	1
1	0	1	0	0	1
1	0	1	1	0	1

0	0	0	0	0	0
0	0	0	0	0	0
0	1	0	0	0	0
0	0	0	0	0	0
1	0	0	0	0	0
0	0	1	0	0	0
0	1	0	0	0	1
1	0	1	0	0	1
0	0	0	0	0	0
0	1	0	0	0	1
0	0	1	0	0	0
0	1	0	0	0	1
0	0	1	0	0	1
0	0	1	0	0	1
0	0	0	0	0	0
0	0	0	0	0	0
1	1	0	0	0	1
0	1	0	0	0	1
0	0	0	0	0	1
0	0	0	1	0	1
0	0	0	0	0	0
0	0	1	0	0	1
0	1	0	0	0	1
0	0	1	0	0	1
0	1	0	0	0	1
0	1	0	0	0	1
1	0	1	0	0	0
0	1	0	0	0	1
0	0	0	0	0	0
0	1	0	0	0	1
1	0	0	0	0	0
1	1	0	0	0	0
1	0	0	0	0	1
1	0	1	0	0	1
1	0	1	0	0	1
0	0	1	0	0	1
1	1	0	0	0	1
1	0	0	0	0	0
1	0	1	0	1	1
0	1	0	0	0	1
1	0	1	0	0	1
1	1	0	0	0	1
0	0	0	1	0	1
1	1	0	0	0	1
1	0	0	0	0	1
1	0	1	0	0	1
1	0	1	0	0	1
0	0	0	0	0	1
1	0	1	0	0	1
1	1	0	0	0	1
0	0	1	0	0	1
0	0	0	0	0	0
1	1	0	0	0	0
1	1	0	0	0	1
0	0	1	0	0	1
1	0	1	0	0	0
1	0	1	0	0	0

0	0	1	0	1	1
0	0	0	0	0	0
1	0	0	0	0	1
1	0	0	0	0	1
1	0	0	0	0	1
0	0	1	0	0	0
0	0	0	0	0	0
0	0	0	0	0	1
1	0	0	0	0	1
1	0	1	0	0	1
0	1	0	0	0	1
0	0	0	0	0	1
1	0	1	0	0	1
0	1	0	0	0	1
0	1	0	0	0	1
0	0	1	0	0	1
0	0	0	0	0	0
0	0	0	0	0	1
0	0	1	0	0	0
0	0	1	0	0	0
0	1	0	0	0	1
0	0	1	0	0	1
0	0	0	1	0	1
0	1	0	0	0	1
0	0	0	0	0	1
1	1	0	0	0	1
0	1	0	0	0	1
0	0	0	0	0	1
1	0	0	0	0	1
0	1	0	0	0	1
0	0	0	0	0	1
0	0	1	0	0	1
0	1	0	0	0	1
0	0	1	0	0	1
0	1	0	0	0	1
0	0	1	0	0	1
1	1	0	0	0	1
0	0	0	0	0	0
0	1	0	0	0	1
0	0	1	0	0	0
0	0	1	0	0	0
1	0	1	0	0	1
1	0	1	0	0	1
0	0	1	0	0	0
1	0	1	0	0	1
0	0	0	0	0	0
0	0	0	0	0	1
0	0	0	0	0	0
0	0	0	0	0	0
0	1	0	0	0	0
0	0	0	0	0	0
0	0	1	0	0	0
0	1	0	0	0	1
1	1	0	1	0	1
0	1	0	0	0	1
1	0	1	0	0	1
1	0	0	0	0	1

0	0	0	0	0	1
1	0	0	0	0	1
0	0	1	0	0	1
0	0	0	0	0	0
0	0	1	0	0	1
0	0	0	0	0	1
1	0	1	0	0	1
1	1	0	0	0	1
0	0	1	0	0	1
0	0	0	0	0	1
0	0	0	0	0	1
0	0	0	0	0	1
0	0	1	0	0	1
1	1	0	0	0	0
0	0	1	0	0	1
0	0	0	0	0	0
0	0	1	0	0	1
0	0	0	0	0	0
0	1	0	0	0	1
0	0	1	0	0	1
1	0	1	0	0	0
1	0	0	0	0	1
1	1	0	0	0	1
1	1	0	0	0	1
1	1	0	0	0	1
1	1	0	0	0	1
1	0	1	0	0	0
0	0	0	0	0	0
0	1	0	0	0	1
0	0	1	0	0	1
0	0	0	0	0	0
0	0	0	0	0	0
1	0	0	0	0	1
0	0	0	0	0	0
1	1	0	0	0	1
1	0	0	0	0	1
0	1	0	0	0	1
1	1	0	0	0	1
1	0	0	0	0	1
0	1	0	0	0	0
0	0	0	0	0	1
0	0	1	0	0	1
1	0	0	0	0	1
1	0	1	0	0	0
0	1	0	1	0	1
0	1	0	0	0	1
1	0	1	0	0	1
0	0	0	0	0	1
1	1	0	0	0	0
1	0	1	0	0	1
1	0	1	0	0	1
0	0	1	0	0	1
1	0	0	0	0	0
0	0	0	0	0	0
0	0	0	0	0	0
0	0	0	0	0	1
1	0	0	0	0	1

1	0	1	0	0	1
0	1	0	0	0	1
0	0	1	0	0	1
0	1	0	0	0	1
1	0	1	0	0	0
0	0	1	0	0	1
1	0	1	0	0	1
1	0	0	0	0	1
1	0	1	0	0	1
1	0	1	0	0	1
1	0	1	0	0	0
1	0	1	0	0	0
0	1	0	1	0	1
0	0	0	0	0	1
0	0	0	0	0	0
0	1	0	0	0	1
0	1	0	0	0	1
0	0	1	0	0	1
1	0	0	0	1	1
1	0	1	0	0	0
0	0	1	0	0	1
1	0	1	0	0	1
0	0	1	0	0	0
1	0	1	0	0	1
0	0	0	0	0	0
0	0	1	0	0	1
0	0	1	0	0	0
0	1	0	0	0	1
1	0	1	0	0	1
1	0	1	0	0	1
0	1	0	1	0	1
0	0	0	0	0	1
0	1	0	0	0	1
0	0	0	0	0	0
1	1	0	0	0	0
1	0	1	1	0	0
0	0	1	0	0	1
0	0	0	0	0	1
0	1	0	0	0	0
0	0	1	0	0	0
0	0	1	0	0	0
0	1	0	0	0	1
0	1	0	0	0	1
1	0	0	0	0	0
1	1	0	0	0	1
0	0	1	0	0	1
1	0	0	0	0	0
1	0	0	0	0	0
0	0	0	0	0	0
1	1	0	0	0	1
0	1	0	0	0	1
0	0	1	0	0	1
0	1	0	0	0	1
0	0	1	0	0	1
0	0	1	0	0	1
1	1	0	0	0	1
0	0	0	0	0	0

0	0	1	0	0	1
0	0	1	0	0	1
0	1	0	0	0	1
1	1	0	0	0	1
1	0	1	0	0	0
0	0	0	0	0	1
0	0	1	0	0	1
0	0	0	0	0	0
0	1	0	0	0	1
0	1	0	0	0	1
0	0	1	0	0	0
0	0	1	0	0	1
1	0	0	0	0	0
0	0	1	0	0	0
0	1	0	0	0	1
1	0	0	0	0	0
1	0	0	0	0	0
1	1	0	0	0	1
1	0	0	0	0	1
1	0	0	0	0	1
0	1	0	0	0	0
0	1	0	0	0	1
1	1	0	0	0	1
1	0	0	1	0	1
0	0	0	0	0	0
1	0	0	0	0	0
0	0	1	0	0	1
0	0	0	0	0	1
0	1	0	0	0	1
0	0	0	0	0	0
0	0	1	0	0	1
1	0	1	0	0	1
0	0	1	0	0	1
1	0	0	0	0	1
1	0	1	0	0	1
0	0	1	0	0	1
0	0	1	0	0	1
0	0	1	0	0	1
0	0	1	0	0	1
1	1	0	0	0	1
0	1	0	0	0	1
1	1	0	0	0	1
0	0	1	0	0	0
0	0	0	0	0	0
0	0	0	0	0	0
1	0	1	0	0	1
0	0	0	0	0	1
0	1	0	0	0	1
1	1	0	0	0	1
1	0	1	0	0	1
0	1	0	0	1	1
1	0	1	0	0	1
0	1	0	0	0	0
0	0	1	0	0	0
0	0	0	0	0	1
1	0	1	0	0	0
1	0	0	0	0	0

0	0	0	0	0	0
0	0	0	0	0	0
0	0	1	0	0	1
0	0	1	0	0	0
0	0	0	0	0	1
0	0	0	0	0	0
0	0	1	0	0	0
0	0	1	0	0	1
0	0	0	0	0	0
0	0	0	0	0	0
0	0	1	0	0	1
0	1	0	0	0	1
0	0	0	0	0	1
1	0	1	0	0	1
0	0	0	0	0	0
1	0	1	0	0	1
0	0	0	0	0	1
1	1	0	0	0	1
1	1	0	0	0	1
0	0	1	0	0	1
0	1	0	0	0	1
0	0	0	0	0	0
1	0	1	0	0	1
1	1	0	0	0	1
0	0	1	0	0	0
0	0	0	0	0	1
0	0	1	0	0	1
1	1	0	0	0	1
0	0	1	0	0	1
0	1	0	1	0	1
0	1	0	0	0	1
0	1	0	0	0	1
0	0	1	0	0	1
0	1	0	0	0	1
1	0	1	0	0	1
1	1	0	0	0	1
0	0	1	0	0	0
1	0	0	0	0	1
1	1	0	0	0	1
1	0	1	0	0	1
1	0	1	0	0	1
0	0	0	0	0	1
0	0	1	0	0	1
1	0	1	0	0	1
1	1	0	0	0	1
1	0	1	0	0	1
0	0	1	0	0	1
0	1	0	0	0	1
0	0	1	0	0	1
1	0	0	0	0	1
1	0	0	0	0	1
1	1	0	0	0	1
0	1	0	0	0	1
1	0	0	0	0	1
1	0	0	0	0	0
1	1	0	0	0	1
0	1	0	0	0	1

1	1	0	0	0	1
1	0	1	0	0	1
0	0	1	0	0	1
0	0	1	0	0	0
0	0	0	0	0	0
0	0	0	0	0	1
0	0	0	0	0	1
1	0	0	0	0	0
1	0	1	0	0	0
0	0	1	0	0	0
0	0	0	0	0	0
0	0	0	0	0	1
0	0	0	0	0	1
1	0	1	0	0	1
1	1	0	0	0	1
1	0	0	0	0	1
1	1	0	0	0	1
1	0	1	0	0	1
0	1	0	0	0	1
0	1	0	0	0	1
1	1	0	0	0	1
0	0	1	0	0	1
0	1	0	0	0	1
0	0	0	0	0	1
0	0	1	0	0	1
0	1	0	0	0	1
1	1	0	0	0	1
0	0	1	0	0	0
0	0	0	0	0	0
0	0	1	0	0	1
1	0	1	0	0	1
1	0	0	0	0	1
1	1	0	0	0	0
0	0	1	0	0	1
1	0	1	0	0	0
0	0	1	0	0	1
0	1	0	0	0	1
0	0	1	1	0	1
0	0	1	0	0	1
0	0	0	0	0	1
1	1	0	0	0	1
0	1	0	0	0	1
0	0	1	0	0	1
0	1	0	0	0	1
1	1	0	0	0	1
0	0	0	0	0	1
0	0	1	0	0	1
0	0	0	0	0	1
0	1	0	0	0	1
0	0	1	0	0	0
1	0	1	0	0	0
0	1	0	0	0	1
0	0	1	0	1	1
1	0	1	0	1	1
1	0	0	0	0	1
0	0	0	1	0	1
0	1	0	0	0	1

0	0	1	0	0	1
0	1	0	0	0	1
1	0	0	0	0	1
0	1	0	0	0	1
0	0	0	0	0	1
0	1	0	0	0	1
0	1	0	0	0	1
0	0	1	1	0	1
1	0	1	0	0	1
0	0	0	0	1	1
0	0	0	0	0	0
1	0	1	0	0	0
0	0	0	0	0	0
0	0	1	0	0	1
0	0	1	0	0	0
0	0	0	0	0	0
0	0	1	0	0	0
1	0	0	0	0	1
0	0	1	0	0	1
0	0	0	0	0	0
0	1	0	0	0	1
0	0	1	0	0	1
0	0	1	0	0	1
0	0	1	0	0	1
0	1	0	0	0	1
1	0	0	0	0	0
1	0	0	0	0	0
1	1	0	1	0	1
1	1	0	0	0	1
0	0	1	0	0	1
0	1	0	0	0	1
1	1	0	0	0	1
0	0	1	0	0	0
0	0	1	0	0	0
0	0	0	0	0	0
0	0	1	0	0	0
1	1	0	0	0	1
0	1	0	0	0	1
0	1	0	0	0	1
1	0	1	0	0	1
1	1	0	0	0	1
1	0	0	0	0	1
0	0	0	0	0	1
1	0	0	0	0	0
1	0	0	0	0	0
0	0	0	0	0	0
0	0	1	0	0	0
0	0	1	0	0	0
1	0	0	0	0	0
0	0	1	0	0	0
0	0	0	0	0	0
0	0	0	0	0	1
0	1	0	0	0	1
0	1	0	0	0	1
0	0	0	0	0	0
0	0	0	0	0	1
1	0	1	0	0	0

1	0	0	0	0	0
0	1	0	0	0	1
0	0	0	0	0	1
1	0	1	0	0	1
1	1	0	0	0	0
0	1	0	0	0	1
1	0	1	0	0	1
1	0	0	0	0	0
0	0	0	0	0	0
1	0	0	0	0	0
0	0	0	0	0	1
0	1	0	0	0	1
0	0	1	0	0	1
0	0	0	0	0	0
1	0	1	0	0	0
0	0	0	0	0	0
0	0	0	0	0	0
0	1	0	0	0	1
1	0	0	0	0	0
0	1	0	0	0	0
0	0	0	0	0	1
1	0	0	0	0	0
1	0	1	0	0	1
0	0	0	0	0	1
0	0	1	0	0	0
0	0	1	0	0	1
0	1	0	0	0	1
0	0	0	0	0	1
0	0	1	0	0	1
0	0	1	0	0	1
0	0	1	0	0	0
0	0	0	0	0	0
1	0	1	0	0	0
0	0	0	0	0	1
0	0	0	0	0	0
0	0	0	0	0	0
0	0	0	0	0	0
0	1	0	0	0	1
0	1	0	0	0	1
0	0	0	0	0	0
0	0	1	0	0	0
0	1	0	0	0	1
0	0	1	0	0	1
0	0	1	0	0	0
0	0	1	0	0	0
1	0	0	0	0	0
0	1	0	0	0	1
1	0	1	0	0	1
0	0	0	0	0	0
0	0	0	0	0	0
0	0	1	0	0	1
0	1	0	0	0	1
0	1	0	0	0	1
1	1	0	0	0	0
1	0	0	0	0	1
0	0	0	0	0	1
0	1	0	0	0	1

0	0	1	0	0	1
0	0	0	0	0	1
1	1	0	0	0	1
0	1	0	1	0	1
0	0	0	0	0	0
1	0	1	0	0	0
1	1	0	0	0	1
1	1	0	0	0	1
0	1	0	0	0	1
0	1	0	0	0	1
0	1	0	0	0	1
1	1	0	0	0	1
0	0	0	0	0	1
1	0	0	0	0	1
0	1	0	0	0	1
0	0	0	0	0	0
0	0	0	0	0	0
0	0	0	0	0	0
0	0	1	0	0	1
0	1	0	0	0	1
0	0	0	1	0	1
0	0	1	0	0	1
0	0	0	0	0	0
0	0	0	0	0	0
0	0	1	0	0	1
0	0	1	0	0	1
0	0	1	0	0	1
0	0	1	0	0	0
0	0	0	0	0	0
1	1	0	0	0	1
0	1	0	0	0	1
1	0	0	0	0	1
1	0	0	0	0	0
0	0	0	0	0	0
0	1	0	0	0	0
1	1	0	0	0	1
0	0	0	0	0	1
1	1	0	0	0	1
0	1	0	0	0	1
0	0	0	0	0	0
0	0	1	0	0	0
0	1	0	0	0	1
0	1	0	0	0	1
0	0	1	0	0	1
0	1	0	0	0	1
0	0	1	0	0	1
1	1	0	0	0	1
0	1	0	1	0	1
1	0	0	0	0	1
0	0	0	0	1	1
0	0	0	0	0	0
0	1	0	0	0	1
1	0	0	0	0	1
1	1	0	0	0	1
1	0	1	0	0	1
0	0	0	0	0	0
0	0	0	0	0	0

0	0	1	0	0	1
1	1	0	0	0	1
0	0	0	0	0	0
0	0	1	0	0	1
0	0	0	0	0	1
1	0	0	0	0	0
0	1	0	0	0	1
0	0	0	0	0	0
0	0	1	1	0	1
1	1	0	0	0	1
1	0	0	0	0	1
1	0	1	0	0	1
0	0	0	0	0	1
0	0	1	0	0	1
1	1	0	0	0	1
0	0	0	0	0	1
0	0	1	0	0	1
0	0	1	0	0	1
0	1	0	0	0	1
1	0	0	0	0	0
0	1	0	0	0	1
1	0	1	0	0	0
1	1	0	0	0	1
1	1	0	0	0	1
0	0	0	0	0	1
0	1	0	0	0	1
1	1	0	0	0	1
0	1	0	0	0	1
1	0	0	0	0	0
1	1	0	0	0	1
0	0	0	0	0	1
1	1	0	0	0	1
0	1	0	0	0	1
0	0	1	0	1	1
1	0	0	0	0	1
1	0	0	0	0	1
0	1	0	1	0	1
1	1	0	0	0	1
1	0	0	0	0	1
1	0	1	0	0	1
1	0	0	0	0	1
0	0	0	0	0	0
1	0	1	0	0	1
1	0	0	0	0	1
0	1	0	0	0	1
0	0	1	0	0	1
0	0	0	0	0	1
0	0	1	0	0	1
0	1	0	0	0	1
0	0	1	0	0	0
0	0	0	0	0	1
1	0	0	0	0	1
1	0	0	0	0	1
0	1	0	0	0	1
0	0	0	0	0	1
0	0	0	0	0	1
0	1	0	0	0	1

0	0	1	0	0	0
0	0	0	0	0	0
1	0	1	0	0	0
0	0	0	0	0	1
0	0	1	0	0	1
0	0	1	0	0	0
1	1	0	0	0	1
0	0	1	0	0	0
0	0	1	0	0	0
0	0	0	0	0	0
0	0	1	0	0	0
0	0	0	0	0	1
0	1	0	0	0	1
0	1	0	0	0	1
0	1	0	0	0	0
0	0	0	0	0	1
0	1	0	0	0	1
1	0	0	0	0	1
0	0	0	0	0	0
0	0	0	0	0	0
0	0	1	0	0	0
0	0	1	0	0	1
0	1	0	0	0	1
1	1	0	0	0	1
0	1	0	1	0	1
0	0	0	0	0	0
0	0	0	0	0	0
0	0	0	0	0	0
1	1	0	0	0	1
1	0	1	0	0	1
1	1	0	0	0	1
0	1	0	0	0	0
0	0	1	0	0	1
0	0	1	0	0	1
0	0	0	0	0	0
0	0	0	0	0	1
0	0	1	0	0	1
0	0	1	0	0	1
0	0	1	0	0	1
0	1	0	0	0	1
1	1	0	0	0	1
0	0	1	0	0	1
0	0	1	0	0	1
1	1	0	0	0	1
0	1	0	0	0	1
0	0	1	0	0	1
1	1	0	0	0	1
0	0	1	0	0	1
0	0	1	0	0	1
1	0	1	0	0	1
0	1	0	0	0	1
0	1	0	0	0	1
0	0	1	0	0	1
0	0	1	0	0	1
0	0	1	0	0	1
0	1	0	0	0	1
0	1	0	0	0	1

0	1	0	0	0	1
1	1	0	0	0	1
0	1	0	0	0	1
0	1	0	0	0	1
0	0	1	0	0	1
0	0	1	0	0	0
0	0	0	0	0	1
0	1	0	0	0	1
0	0	1	0	0	1
1	0	0	0	0	1
0	1	0	0	0	1
1	0	0	0	0	1
0	1	0	0	0	1
0	1	0	0	0	1
0	1	0	0	0	1
0	0	0	0	0	1
0	0	0	0	0	0
0	1	0	0	0	1
0	1	0	0	0	1
0	0	0	0	0	1
0	0	0	0	0	1
0	0	1	0	0	1
0	1	0	0	0	1
1	1	0	0	0	1
0	0	0	0	0	1
0	0	0	0	0	0
0	0	0	0	0	1
0	1	0	0	0	1
0	0	0	0	0	1
0	0	1	0	0	1
0	1	0	0	0	1
0	1	0	0	0	1
0	1	0	0	0	1
0	0	0	0	0	0
0	0	0	0	0	1
0	0	0	0	0	1
0	1	0	0	0	1
0	0	0	0	0	0
0	0	0	0	0	0
0	0	0	0	0	0
0	0	1	0	0	0
0	1	0	0	0	1
0	1	0	0	0	1
1	1	0	0	0	1
0	0	1	0	0	1
0	1	0	0	0	1
0	0	0	0	0	0
1	0	0	0	0	0
1	0	1	0	0	0
0	1	0	0	0	1
0	1	0	0	0	1
1	1	0	0	0	1
0	0	1	0	0	1
0	1	0	0	0	1
0	0	1	0	0	1
1	0	1	0	0	0
1	0	1	0	0	1

0	0	0	0	0	0
1	0	0	0	0	0
0	0	0	0	0	0
1	0	0	0	0	0
1	0	0	0	0	0
0	0	0	0	0	0
0	0	0	0	0	0
1	1	0	0	0	1
0	0	1	0	0	1
1	1	0	0	0	1
0	0	1	0	0	1
1	1	0	0	0	1
0	1	0	0	0	1
1	1	0	0	0	1
0	0	0	0	0	1
0	1	0	0	0	1
0	1	0	0	0	1
0	1	0	0	0	1
0	0	0	0	0	1
0	0	0	0	0	0
0	0	0	0	0	1
0	0	0	0	0	0
0	0	1	0	0	1
0	0	0	0	0	1
0	0	1	0	0	0
0	0	0	0	0	1
0	0	1	0	0	1
1	1	0	0	0	1
0	1	0	0	0	1
1	0	0	0	0	0
0	0	1	0	0	1
0	1	0	0	0	1
0	1	0	0	0	1
1	1	0	0	0	1
1	1	0	0	0	0
0	0	0	0	0	1
1	0	1	0	0	1
1	0	0	0	0	1
1	1	0	0	0	1
1	1	0	0	0	1
0	0	1	0	0	1
0	1	0	0	0	1
1	0	1	0	0	0
0	1	0	0	0	1
1	1	0	0	0	1
1	1	0	0	0	1
0	0	0	0	0	1
1	0	1	0	0	1
1	1	0	0	0	1
0	0	0	0	0	0
0	0	1	0	0	1
0	0	1	0	0	0
0	1	0	0	0	1
1	0	1	0	0	1
0	1	0	0	0	1
1	1	0	0	0	1
1	1	0	0	0	1

0	0	0	0	0	0
0	0	0	0	0	0
0	0	0	0	0	0
0	0	0	0	0	0
0	0	0	0	0	0
0	0	1	0	0	0
0	1	0	0	0	1
0	0	1	0	0	1
0	0	0	0	0	0
0	1	0	0	0	1
0	0	0	0	0	0
1	0	0	0	0	1
1	0	0	0	0	1
1	1	0	0	0	0
0	0	0	0	0	1
0	1	0	0	0	1
0	0	1	0	0	1
0	1	0	0	0	1
0	1	0	0	0	1
1	1	0	0	0	1
0	0	1	0	0	1
0	0	0	0	0	0
0	0	1	0	0	0
0	0	0	0	0	1
0	1	0	0	0	1
0	0	1	0	0	1
0	0	1	0	0	1
0	0	1	0	0	1
1	1	0	0	0	1
0	1	0	0	0	1
0	1	0	0	0	1
1	1	0	0	0	1
0	0	1	0	0	0
1	0	0	0	0	1
0	0	0	0	0	0
0	0	1	0	0	1
0	0	1	0	0	0
0	0	0	0	0	0
0	0	1	0	0	0
0	0	0	0	0	1
1	0	0	0	0	0
0	0	0	0	0	0
0	0	1	0	0	0
0	0	0	0	0	0
0	0	0	0	0	0
0	1	0	0	0	1
0	0	0	0	0	0
1	0	1	0	0	1
0	0	0	0	0	1
0	0	1	0	0	1
0	0	0	0	0	1
1	0	0	0	0	0
0	0	0	0	0	0
1	0	1	0	0	1
1	0	0	0	0	0
0	0	1	0	0	0
0	1	0	0	0	1

1	1	0	0	0		1
0	0	1	0	0		1
0	0	0	0	0		1
1	0	1	0	0		0
1	0	1	0	0		0
0	0	0	0	0		0
0	0	0	0	0		0
0	0	0	0	0		0
0	0	0	0	0		1
0	0	0	0	0		0
1	0	1	0	0		0
0	0	0	0	0		1
0	0	0	0	0		0
0	0	0	0	0		1
1	0	0	0	0		1
1	0	0	0	0		1
0	0	0	0	0		0
0	0	0	0	0		0
1	0	0	0	0		0
0	0	0	0	0		0
1	0	0	0	0		1
0	0	0	0	0		1
1	0	1	0	0		0
1	0	0	1	0		0
0	0	0	0	0		1
0	0	1	0	0		0
0	1	0	0	0		1
1	0	0	0	0		1
0	0	0	0	0		1
1	0	1	0	0		1
0	0	0	0	0		1
1	1	0	0	0		0
1	0	0	0	0		1
1	1	0	0	0		1
0	0	0	0	0		0
1	0	0	0	0		1
1	1	0	0	0		1
1	1	0	0	0		1
1	1	0	0	0		1
1	0	0	0	0		1
0	1	0	0	0		0
1	0	0	0	0		1
0	0	1	0	0		1
0	0	0	0	0		0
0	0	0	0	0		1
0	0	0	0	0		1
0	0	0	0	0		1
0	0	0	0	0		1
0	0	0	0	0		0
1	0	0	0	0		1
0	0	0	0	0		1
0	0	0	0	0		0
0	0	1	0	0		0
0	0	0	0	0		1
0	0	1	0	0		1
0	0	0	0	0		1
0	0	0	0	0		0

1	0	0	0	0	1
0	0	1	0	0	0
0	0	0	0	0	0
1	0	0	0	0	0
0	0	0	0	0	1
0	0	0	0	0	0
0	0	0	0	0	1
0	0	0	0	0	1
0	0	0	0	0	0
0	0	0	0	0	0
0	0	0	0	0	0
0	0	1	0	0	1
1	1	0	1	0	1
0	0	0	0	0	0
0	0	1	0	0	1
0	0	0	0	0	1
1	1	0	1	0	1
0	0	0	0	0	1
1	0	1	0	0	1
1	1	0	0	0	0
0	0	0	0	0	0
1	0	0	0	0	0
0	0	0	0	0	0
1	0	0	0	0	0
0	1	0	1	0	1
1	0	0	0	0	0
0	0	1	0	0	0
1	0	1	0	0	0
1	0	1	0	0	0
1	0	0	0	0	1
0	1	0	0	0	0
0	1	0	0	0	1
1	0	0	0	0	1
0	0	0	0	0	1
1	1	0	0	0	1
0	1	0	0	0	1
1	1	0	0	0	1
0	0	0	0	0	1
1	1	0	0	0	1
0	0	0	0	0	0
0	0	0	0	0	0
0	1	0	0	0	1
0	1	0	0	0	1
0	1	0	0	0	1
0	1	0	0	0	1
0	1	0	0	0	1
0	1	0	0	0	0
0	1	0	0	0	1
1	1	0	0	0	1
0	0	0	0	0	1
0	0	0	0	0	0
0	0	0	0	0	0
0	0	0	0	0	0
1	0	0	0	0	0
0	0	0	0	0	0
0	0	0	0	0	0
0	0	0	0	0	0

1	0	0	0	0		0
0	0	0	0	0		0
1	1	0	0	0		1
1	0	1	0	0		1
1	0	0	0	0		1
1	0	0	0	0		1
1	1	0	0	0		1
0	0	1	0	0		1
1	0	0	0	0		0
0	0	1	0	0		0
0	0	0	0	0		1
1	0	0	0	0		0
1	1	0	0	0		1
1	1	0	0	0		1
0	1	0	0	0		1
0	1	0	0	0		1
1	0	1	0	0		1
0	1	0	0	0		1
1	1	0	0	0		1
0	0	0	0	0		0
0	0	1	0	0		1
0	0	0	0	0		1
0	0	0	0	0		1
1	0	1	0	0		1
0	0	0	0	0		1
0	0	1	0	0		1
1	1	0	0	0		1
1	1	0	0	0		0
1	0	0	0	0		1
1	0	0	0	0		0
0	0	0	0	0		0
0	1	0	0	0		1
0	0	0	0	0		1
1	0	0	0	0		1
1	1	0	0	0		1
0	1	0	0	0		1
1	0	0	0	0		0
1	1	0	0	0		1
0	0	0	0	0		0
0	1	0	0	0		0
0	0	1	0	0		0
0	0	1	0	0		1
0	0	1	0	0		1
0	0	0	0	0		0
0	0	0	0	0		1
1	1	0	0	0		0
0	0	0	0	0		1
1	1	0	0	0		1
0	0	1	0	0		1
0	0	1	0	0		0
0	0	0	0	0		0
0	0	1	0	0		1
0	0	0	0	0		0
0	0	1	0	0		1
1	0	0	0	0		0
1	0	1	0	0		0
0	1	0	0	0		1

1	1	0	0	0	1
0	1	0	0	0	1
1	1	0	0	0	1
0	0	1	0	0	1
0	0	0	0	0	1
1	0	0	0	0	0
0	0	0	0	0	0
0	0	0	0	0	0
1	0	0	0	0	0
0	0	0	0	0	0
1	0	0	0	0	0
0	0	0	0	0	0
0	0	0	0	0	1
0	0	0	0	0	0
0	0	0	0	0	1
0	1	0	0	0	1
0	0	1	0	0	1
0	0	1	0	0	1
1	0	0	0	0	0
0	0	0	0	0	1
0	0	1	0	0	0
0	0	1	0	0	1
0	0	0	0	0	1
0	0	1	0	0	0
1	1	0	0	0	1
1	0	0	0	0	1
1	0	1	0	0	1
0	0	0	0	0	1
1	0	0	0	0	0
0	0	0	0	0	1
1	0	0	0	0	0
0	0	0	0	0	0
1	1	0	0	0	1
0	0	1	0	0	0
1	0	0	0	0	1
1	0	0	0	0	0
0	1	0	0	0	1
0	1	0	0	0	0
1	1	0	0	0	1
0	1	0	0	0	1
0	0	1	0	0	1
0	0	0	0	0	0
0	0	1	0	0	1
0	0	0	0	0	1
0	0	0	0	0	0
0	0	0	0	0	0
1	0	0	0	0	0
1	0	1	0	0	0
1	0	0	0	0	0
1	0	0	0	0	0
0	0	0	0	0	0
0	0	0	0	0	0
0	0	0	0	0	0
0	0	0	0	0	1
0	0	1	0	0	0
1	0	0	0	0	1
1	0	0	0	0	0

1	1	0	0	0	1
1	0	1	0	0	0
0	0	1	0	0	1
0	0	1	0	0	1
0	1	0	0	0	1
1	0	0	0	0	0
0	1	0	0	0	1
1	0	1	0	0	1
1	1	0	0	0	1
1	0	1	0	0	1
0	0	0	0	0	0
1	0	1	0	0	1
0	0	1	0	0	0
0	0	1	0	0	1
0	1	0	0	0	1
0	0	0	0	0	0
0	0	0	0	0	0
0	0	0	0	0	0
0	1	0	0	0	1
1	0	0	0	0	1
0	0	0	0	0	1
0	0	0	0	0	0
0	1	0	0	0	1
1	0	0	0	0	0
1	1	0	0	0	1
0	0	1	0	0	0
1	0	0	0	0	0
1	0	0	0	0	1
0	0	0	0	0	0
1	0	0	0	0	0
1	0	1	0	0	1
1	1	0	0	0	1
0	1	0	0	0	1
1	0	0	0	0	0
1	0	0	0	0	1
0	0	0	0	0	0
0	0	1	0	0	1
1	0	0	0	0	1
1	0	0	0	0	1
0	1	0	0	0	1
0	0	1	0	0	1
0	0	0	0	0	1
1	0	1	0	0	1
0	1	0	0	0	1
0	1	0	0	0	1
0	0	0	0	0	1
1	0	0	0	0	0
1	0	1	0	0	1
1	0	0	0	0	1
0	0	0	0	0	1
0	0	1	0	0	0
0	0	0	0	0	0
1	0	1	0	0	0
0	0	1	0	0	1
1	0	0	0	0	0
0	0	0	0	0	0
0	0	0	0	0	0

1	0	1	0	0		0
0	0	0	0	0		0
0	0	0	0	0		1
0	0	0	0	0		0
0	0	0	0	0		0
1	0	0	0	0		1
0	0	1	0	0		1
1	0	0	0	0		0
0	0	0	0	0		0
1	1	0	0	0		1
0	0	1	0	0		1
1	0	0	0	0		0
1	0	0	0	0		0
1	0	0	0	0		1
0	0	1	0	0		0
0	0	0	0	0		0
0	0	1	0	0		1
1	0	0	0	0		0
1	0	0	0	0		0
0	0	0	0	0		0
1	0	0	0	0		1
1	0	1	0	0		0
1	0	0	0	0		0
0	0	0	0	0		0
0	0	0	0	0		0
0	0	0	0	0		1
0	0	0	0	0		0
1	0	1	0	0		0
0	0	0	0	0		0
0	0	0	0	0		1
0	0	0	0	0		0
1	0	0	0	0		1
1	0	0	0	0		0
0	1	0	0	0		1
1	0	0	0	0		1
0	0	0	0	0		0
1	0	0	0	0		0
1	0	0	0	0		1
0	0	0	0	0		0
1	0	1	0	0		1
0	1	0	0	0		1
1	0	0	0	0		0
1	0	1	0	0		0
0	1	0	0	0		1
1	0	0	0	0		1
1	0	0	0	0		1
1	0	0	0	0		0
1	1	0	0	0		1
0	0	0	0	0		1
0	0	1	0	0		1
0	0	1	0	0		1
0	0	0	0	0		0
1	0	0	0	0		1
1	1	0	0	0		1
1	0	0	0	0		1
0	0	1	0	0		1
1	0	1	0	0		1

0	0	1	0	0	1
0	0	0	0	0	1
0	0	0	0	0	1
0	0	0	0	0	0
1	0	0	0	0	0
0	0	1	0	0	0
0	0	1	0	0	1
0	1	0	0	0	0
1	0	0	0	0	0
0	0	0	0	0	1
0	1	0	0	0	0
0	0	1	0	0	1
1	0	1	0	0	0
1	0	0	0	0	0
1	0	1	0	0	0
1	0	0	0	0	0
0	1	0	0	0	0
1	0	0	0	0	0
1	0	0	0	0	1
0	0	0	0	0	0
1	1	0	0	0	0
0	1	0	0	0	1
1	0	1	0	0	0
0	1	0	0	0	1
0	0	1	0	0	1
0	0	0	0	0	0
1	0	0	0	0	0
1	0	1	0	0	0
1	0	0	0	0	1
1	0	1	0	0	1
1	0	0	0	0	0
0	1	0	0	0	1
1	1	0	0	0	0
0	0	1	0	0	1
0	0	0	0	0	1
1	0	0	0	0	0
1	0	0	0	0	0
0	1	0	0	0	1
0	0	0	0	0	1
0	0	0	0	0	0
0	0	1	0	0	0
1	1	0	0	0	0
1	0	1	0	0	0
1	0	0	0	0	0
0	0	1	0	0	0
0	0	0	0	0	1
0	1	0	0	0	1
0	0	0	0	0	1
0	0	0	0	0	0
0	0	0	0	0	0
0	0	0	0	0	0
1	1	0	0	0	1
1	0	1	0	0	0
0	0	0	0	0	0
0	0	0	0	0	1
1	0	0	0	0	1
1	0	0	0	0	1

0	0	0	0	0	1
0	1	0	0	0	1
0	0	0	0	0	1
1	0	0	0	0	0
0	1	0	0	0	1
0	0	1	0	0	0
0	0	1	0	0	1
0	0	0	0	0	0
1	0	0	0	0	0
0	0	0	0	0	1
0	1	0	0	0	1
1	1	0	0	0	1
1	0	0	0	0	0
1	0	0	0	0	1
0	0	1	0	0	0
0	0	0	0	0	1
0	0	0	0	0	0
0	0	1	0	0	0
1	0	0	0	0	0
1	0	1	0	0	0
0	0	0	0	0	1
1	0	0	0	0	1
0	0	0	0	0	1
0	0	0	0	0	1
0	0	0	0	0	1
0	0	0	0	0	0
0	1	0	0	0	1
0	0	0	0	0	0
1	1	0	0	0	1
1	0	0	0	0	0
0	0	0	0	0	1
0	0	0	0	0	1
0	0	0	0	0	0
0	0	1	0	0	1
0	0	0	0	0	1
1	1	0	0	0	1
1	0	0	0	0	0
1	0	1	0	0	0
1	1	0	0	0	1
0	0	0	0	0	0
1	0	0	0	0	1
1	1	0	0	0	1
0	0	0	0	0	0
0	0	0	0	0	0
1	0	1	0	0	0
1	0	0	0	0	1
1	0	0	0	0	1
1	0	1	0	0	0
0	0	0	0	0	0
1	0	1	0	0	0
0	0	0	0	0	0
0	0	1	0	0	1
1	1	0	0	0	1
0	0	0	0	0	0
1	0	0	0	0	1
0	1	0	0	0	1
0	1	0	0	0	1

1	0	1	0	0	1
1	0	1	0	0	0
0	0	1	0	0	1
1	0	1	0	0	1
0	0	0	0	0	0
0	0	0	0	0	1
0	1	0	0	0	1
0	0	0	0	0	0
0	0	1	0	0	0
0	0	0	0	0	0
0	0	1	0	0	0
0	0	1	0	0	1
0	0	0	0	0	1
1	0	0	0	0	0
0	1	0	0	0	1
0	0	1	0	0	0
0	1	0	0	0	1
0	0	0	0	0	1
0	0	0	0	0	0
1	0	0	0	0	1
0	0	0	0	0	0
0	0	0	0	0	1
1	0	0	0	0	1
0	0	0	0	0	1
1	1	0	0	0	1
1	0	0	0	0	0
1	0	0	0	0	0
0	0	0	0	0	1
1	1	0	0	0	1
0	0	0	0	0	1
0	0	0	0	0	0
1	0	1	0	0	0
1	0	0	0	0	0
0	0	0	0	0	1
1	0	0	0	0	0
0	0	0	0	0	0
0	1	0	0	0	1
0	0	0	0	0	0
1	0	0	0	0	1
0	0	0	0	0	1
0	0	1	0	0	0
0	0	1	0	0	1
0	0	0	0	0	1
0	0	0	0	0	1
0	0	0	0	0	0
0	1	0	0	0	1
0	0	1	0	0	1
0	0	0	0	0	1
1	0	0	0	0	0
0	1	0	0	0	1
0	0	0	0	0	1
1	0	0	0	0	1
1	1	0	0	0	1
1	1	0	0	0	1
1	0	1	0	0	1
1	0	0	0	0	1
0	0	0	0	0	1

1	0	1	0	0	1
1	0	0	0	0	0
0	0	1	0	0	1
0	0	0	0	0	1
1	0	0	0	0	0
0	1	0	0	0	1
0	0	1	0	0	0
0	0	0	0	0	1
1	0	0	0	0	1
1	1	0	0	0	1
0	0	0	0	0	0
1	1	0	0	0	1
0	1	0	0	0	1
0	0	1	0	0	0
1	1	0	0	0	1
1	0	1	0	0	0
1	0	0	0	0	1
1	0	0	0	0	0
0	0	0	0	0	0
1	1	0	0	0	1
1	0	0	0	0	1
1	0	1	0	0	0
0	0	1	0	0	0
1	0	0	0	0	1
1	0	0	0	0	0
1	1	0	0	0	0
0	0	0	0	0	0
0	0	1	0	0	0
0	1	0	0	0	0
0	0	1	0	0	1
1	0	0	0	0	0
1	0	0	0	0	0
0	1	0	0	0	1
0	0	0	0	0	0
0	0	0	0	0	1
1	0	0	0	0	0
0	0	1	0	0	0
1	1	0	0	0	1
0	0	1	0	0	0
1	0	1	0	0	1
1	1	0	0	0	1
1	0	0	0	0	1
0	0	0	0	0	0
0	0	0	0	0	1
0	0	1	0	0	1
0	0	0	0	0	1
0	0	1	0	0	1
0	0	1	0	0	1
0	0	0	0	0	1
0	0	0	0	0	0
0	0	0	0	0	0
0	0	1	0	0	1
1	1	0	0	0	1
1	0	0	0	0	0
0	0	0	0	0	0
1	0	1	0	0	0
1	0	0	0	0	1

1	0	1	0	0	1
0	0	0	0	0	1
0	0	1	0	0	0
0	1	0	0	0	1
0	1	0	0	0	1
0	0	1	0	0	1
1	0	1	0	0	1
1	0	0	0	0	1
0	1	0	0	0	0
0	0	0	0	0	0
0	0	1	0	0	1
1	1	0	0	0	1
0	1	0	0	0	1
0	0	1	0	0	1
1	0	0	0	0	0
0	0	1	0	0	1
0	1	0	0	0	1
1	0	1	0	0	0
1	0	1	0	0	1
0	0	1	0	0	0
0	1	0	0	0	1
1	1	0	0	0	1
0	0	0	0	0	0
0	0	0	0	0	1
0	0	0	0	0	1
1	1	0	0	0	1
0	0	0	0	0	0
0	0	1	0	0	0
1	1	0	0	0	1
0	0	0	0	0	1
0	0	1	0	0	0
1	1	0	0	0	0
0	0	0	0	0	1
0	0	0	0	0	0
0	0	1	0	0	1
1	0	1	0	0	0
0	0	1	0	0	0
0	0	0	0	0	0
0	0	0	0	0	0
0	0	1	0	0	1
0	0	0	0	0	1
0	0	0	0	0	0
0	0	0	0	0	0
0	0	1	0	0	1
0	1	0	0	0	0
0	0	1	0	0	1
0	0	0	0	0	1
0	0	0	0	0	0
1	0	0	0	0	0
0	0	0	0	0	1
0	0	0	0	0	1
1	1	0	0	0	1
1	1	0	0	0	0
0	0	0	0	0	1
0	0	0	0	0	0
0	0	0	0	0	1
0	0	1	0	0	0

1	0	0	0	0	0
0	0	0	0	0	0
1	1	0	0	0	1
0	0	0	0	0	0
1	0	0	0	0	0
1	0	0	0	0	0
0	0	1	0	0	0
1	1	0	0	0	1
1	1	0	0	0	0
1	0	1	0	0	0
1	1	0	0	0	0
0	0	0	0	0	1
0	1	0	0	0	1
0	1	0	0	0	1
0	0	1	0	0	1
0	0	0	0	0	0
0	0	0	0	0	0
1	0	0	0	0	0
0	0	0	0	0	0
1	0	1	0	0	1
0	0	1	0	0	1
0	0	0	0	0	0
0	0	0	0	0	0
0	1	0	0	0	1
0	1	0	0	0	1
0	0	0	0	0	0
1	0	1	0	0	1
1	1	0	0	0	1
1	0	1	0	0	0
0	0	0	0	0	0
0	1	0	0	0	1
1	0	1	0	0	0
1	0	0	0	0	1
0	0	0	0	0	1
0	0	0	0	0	0
0	1	0	0	0	1
1	0	1	0	0	1
0	0	0	0	0	1
0	0	1	0	0	1
0	0	1	0	0	0
0	1	0	0	0	1
1	0	0	0	0	1
0	0	1	0	0	1
0	0	0	0	0	0
1	1	0	0	0	1
0	0	0	0	0	0
0	0	1	0	0	0
1	0	0	0	0	0
0	0	1	0	0	0
1	0	0	0	0	0
0	0	0	0	0	1
1	0	0	0	0	1
1	0	1	0	0	0
1	1	0	0	0	1
0	0	1	0	0	0
0	0	0	0	0	0
0	0	0	0	0	0

0	0	0	0	0	0
0	0	1	0	0	0
0	1	0	0	0	1
0	0	1	0	0	1
1	0	0	0	0	1
0	1	0	0	0	1
0	0	0	0	0	1
0	0	1	0	0	1
0	1	0	0	0	1
0	0	0	0	0	1
0	0	0	0	0	0
1	0	1	0	0	1
0	0	1	0	0	1
1	1	0	0	0	1
0	0	0	0	0	0
0	0	1	0	0	0
1	0	1	0	0	0
0	0	0	0	0	0
1	0	0	0	0	1
1	1	0	0	0	1
1	0	0	0	0	1
0	0	1	0	0	1
1	0	0	0	0	0
1	1	0	0	0	0
0	0	0	0	0	0
1	0	1	0	0	1
0	0	0	0	0	0
0	0	0	0	0	0
0	1	0	0	0	1
0	1	0	0	0	0
1	0	0	0	0	0
0	0	0	0	0	0
0	0	0	0	0	0
0	0	0	0	0	1
1	0	1	0	0	1
0	0	1	0	0	0
1	0	0	0	0	0
0	0	1	0	0	0
1	1	0	0	0	0
0	0	0	0	0	0
0	0	0	0	0	0
1	0	1	0	0	0
1	0	0	0	0	1
0	0	0	0	0	1
1	0	0	0	0	0
0	1	0	0	0	0
0	0	1	0	0	0
1	0	0	0	0	0
0	0	0	0	0	1
0	0	0	0	0	0
0	0	0	0	0	0
0	0	0	0	0	0
1	0	0	0	0	0
1	0	0	0	0	0
0	1	0	0	0	0
0	0	0	0	0	1
1	0	0	0	0	1

1	0	0	0	0	0
1	0	0	0	0	0
1	0	0	0	0	0
0	0	0	0	0	1
1	0	0	0	0	1
1	0	0	0	0	1
1	0	1	0	0	0
1	1	0	0	0	1
0	0	1	0	0	0
1	0	0	0	0	0
1	0	1	0	0	1
0	0	1	0	0	0
0	1	0	0	0	0
0	0	1	0	0	1
0	0	0	0	0	0
1	0	1	0	0	0
1	0	1	0	0	0
1	0	1	0	0	0
1	0	0	0	0	1
1	0	0	0	0	1
1	0	1	0	0	0
0	0	0	0	0	0
0	0	1	0	0	0
0	0	1	0	0	1
0	0	1	0	0	0
0	0	0	0	0	0
0	0	0	0	0	1
1	0	1	0	0	0
0	0	0	0	0	1
0	0	0	0	0	0
0	0	0	0	0	1
0	1	0	0	0	1
0	0	0	0	0	1
0	0	0	0	0	0
0	1	0	0	0	1
0	1	0	0	0	1
1	0	0	0	0	0
1	0	0	0	0	0
0	0	1	0	0	0
1	0	1	0	0	1
0	1	0	0	0	1
0	0	1	0	0	1
0	1	0	0	0	1
0	0	1	0	0	1
0	1	0	0	0	1
0	0	0	0	0	1
0	0	0	0	0	1
0	0	0	0	0	1
0	1	0	0	0	1
0	0	1	0	0	1
0	0	0	0	0	0
0	0	0	0	0	0
1	0	1	0	0	0
1	0	1	0	0	1
0	1	0	0	0	1
0	0	0	0	0	1
0	0	1	0	0	1

0	1	0	0	0	1
0	0	0	0	0	0
0	1	0	0	0	1
0	0	0	0	0	1
0	0	1	0	0	1
0	0	0	0	0	0
1	1	0	0	0	1
1	0	1	0	0	0
0	0	0	0	0	1
1	0	0	0	0	1
1	0	0	0	0	0
0	0	0	0	0	1
0	0	0	0	0	1
0	1	0	0	0	1
1	0	0	0	0	1
0	0	0	0	0	0
0	1	0	0	0	0
1	0	1	0	0	1
0	1	0	0	0	0
1	0	1	0	0	0
1	1	0	0	0	1
1	1	0	0	0	1
0	0	1	0	0	1
0	0	1	0	0	0
0	0	1	0	0	0
0	0	0	0	0	0
1	0	0	0	0	1
1	0	0	0	0	0
0	0	0	0	0	0
0	1	0	0	0	1
1	0	0	0	0	1
0	0	0	0	0	0
0	0	0	0	0	0
1	0	1	0	0	0
0	0	0	0	0	1
0	0	0	0	0	1
0	1	0	0	0	1
1	0	0	0	0	0
0	0	1	0	0	1
1	0	0	0	0	1
1	0	0	0	0	1
0	1	0	0	0	0
1	0	1	0	0	0
0	0	0	0	0	1
1	0	0	0	0	1
1	0	0	0	0	0
0	0	1	0	0	1
1	0	0	0	0	0
0	0	0	0	0	1
0	0	0	0	0	1
0	0	0	0	0	1
1	0	1	0	0	0
1	0	0	0	0	0
1	0	0	0	0	0
0	0	0	0	0	1
1	0	0	0	0	0
0	0	0	0	0	0

1	0	1	0	0	1
1	0	0	0	0	1
1	0	1	0	0	0
1	1	0	0	0	1
0	0	0	0	0	1
0	0	0	0	0	1
0	0	0	0	0	1
1	0	0	0	0	1
1	0	1	0	0	0
1	0	1	0	0	0
0	0	0	0	0	1
0	0	0	0	0	1
0	0	0	0	0	0
0	0	0	0	0	0
0	0	0	0	0	1
0	1	0	0	0	1
1	0	0	0	0	0
0	0	0	0	0	1
0	0	0	0	0	0
1	0	0	0	0	1
0	0	1	0	0	0
0	0	0	0	0	0
1	0	1	0	0	0
0	0	0	0	0	0
0	1	0	0	0	1
0	0	0	0	0	0
1	0	1	0	0	1
1	0	0	0	0	0
0	0	1	0	0	1
0	0	1	0	0	1
0	0	0	0	0	0
1	0	0	0	0	0
1	0	1	0	0	0
1	0	0	0	0	0
1	0	0	0	0	0
0	0	0	0	0	0
0	0	0	0	0	0
0	0	0	0	0	0
0	0	1	0	0	0
1	0	1	0	0	1
1	0	0	0	0	0
1	0	1	0	0	0
0	0	0	0	0	0
1	0	1	0	0	1
0	0	0	0	0	0
0	0	0	0	0	0
0	0	1	0	0	1
0	0	0	0	0	1
0	0	0	0	0	0
1	0	0	0	0	0
0	0	1	0	0	1
0	1	0	0	0	1
1	0	0	0	0	0
0	0	0	0	0	1
0	0	0	0	0	1
0	1	0	0	0	1
1	0	1	0	0	1

0	0	1	0	0	0
0	0	0	0	0	0
0	0	0	0	0	0
1	0	0	0	0	0
1	1	0	0	0	0
1	0	0	0	0	0
0	0	0	0	0	0
0	0	0	0	0	1
0	0	1	0	0	1
1	1	0	0	0	1
1	1	0	0	0	1
0	0	0	0	0	0
1	1	0	0	0	1
1	1	0	0	0	1
0	1	0	0	0	1
1	0	0	0	0	0
0	0	1	0	0	0
0	1	0	0	0	1
0	0	0	0	0	1
0	0	1	0	0	1
0	1	0	0	0	0
1	0	0	0	0	1
1	1	0	0	0	0
0	1	0	0	0	1
0	1	0	0	0	1
0	0	0	0	0	0
0	0	1	0	0	0
0	0	0	0	0	0
1	0	0	0	0	0
0	0	0	0	0	0
1	1	0	0	0	1
1	0	1	0	0	1
0	0	0	0	0	1
0	0	0	0	0	0
0	0	1	0	0	0
0	0	0	0	0	1
0	0	0	0	0	0
0	0	0	0	0	1
0	0	0	0	0	0
1	0	0	0	0	0
0	0	0	0	0	0
1	1	0	0	0	1
1	1	0	0	0	1
1	0	0	0	0	0
1	1	0	0	0	1
1	0	0	0	0	1
1	0	0	0	0	0
0	0	0	0	0	1
1	0	0	0	0	0
0	0	1	0	0	0
1	0	0	0	0	1
0	0	1	0	0	1
0	1	0	0	0	1
1	0	1	0	0	0
1	0	0	0	0	0
1	0	0	0	0	1
1	0	0	0	0	1

1	0	1	0	0	0
1	1	0	0	0	1
1	1	0	0	0	1
0	0	0	0	0	1
0	1	0	0	0	0
0	0	0	0	0	1
0	1	0	0	0	0
1	1	0	0	0	1
1	0	0	0	0	1
0	0	1	0	0	0
1	1	0	0	0	0
0	0	0	0	0	1
0	1	0	0	0	1
0	1	0	0	0	1
0	0	1	0	0	0
0	0	1	0	0	0
0	0	1	0	0	0
0	0	1	0	0	1
1	1	0	0	0	1
1	0	0	0	0	0
1	0	0	0	0	1
0	0	0	0	0	1
0	0	0	0	0	0
0	0	0	0	0	0
0	1	0	0	0	1
0	1	0	0	0	1
0	0	0	0	0	1
0	0	0	0	0	0
0	1	0	0	0	1
0	0	0	0	0	0
0	0	0	0	0	1
0	0	0	0	0	1
0	0	0	0	0	1
1	0	0	0	0	1
0	0	0	0	0	0
1	0	0	0	0	0
0	1	0	0	0	1
0	0	0	0	0	0
0	0	0	0	0	1
1	0	0	0	0	1
0	0	0	0	0	1
1	0	1	0	0	0
0	1	0	0	0	0
0	0	1	0	0	1
0	1	0	0	0	0
1	1	0	0	0	1
0	0	1	0	0	1
0	0	1	0	0	0
0	0	0	0	0	0
0	1	0	0	0	1
0	0	0	0	0	1
1	0	1	0	0	1
0	0	0	0	0	0
0	0	0	0	0	0
0	0	0	0	0	0
0	0	0	0	0	0
0	0	0	0	0	0

0	0	1	0	0	0
0	0	0	0	0	0
0	0	1	0	0	1
0	0	0	0	0	0
1	0	1	0	0	0
1	1	0	0	0	1
0	0	0	0	0	1
0	0	0	0	0	0
0	0	0	0	0	0
0	1	0	0	0	1
1	1	0	0	0	1
0	1	0	0	0	1
0	1	0	0	0	1
1	0	0	0	0	1
0	0	0	0	0	0
1	0	0	0	0	0
0	1	0	0	0	1
0	1	0	0	0	0
1	0	1	0	0	0
0	1	0	0	0	1
1	0	0	0	0	0
1	0	0	0	0	1
1	0	0	0	0	1
1	0	0	0	0	1
0	0	0	0	0	0
0	0	1	0	0	1
0	0	0	0	0	0
0	0	0	0	0	1
0	0	0	0	0	0
1	0	1	0	0	0
1	0	0	0	0	0
0	0	1	0	0	0
0	1	0	0	0	1
1	0	0	0	0	1
0	0	0	0	0	0
1	0	0	0	0	0
1	0	0	0	0	1
1	0	0	0	0	1
0	0	0	0	0	1
0	0	0	0	0	1
0	0	0	0	0	0
0	0	0	0	0	0
1	0	0	0	0	1
0	0	1	0	0	1
0	0	0	0	0	0
0	0	0	0	0	0
0	0	0	0	0	0
0	0	0	0	0	1
0	0	0	0	0	1
0	0	0	0	0	0
0	0	1	0	0	0
0	0	0	0	0	1
0	1	0	0	0	1
1	0	0	0	0	1
0	0	1	0	0	0
0	0	1	0	0	0
0	0	1	0	0	1

1	0	0	0	0	1
1	0	1	0	0	0
0	0	0	0	0	1
0	1	0	0	0	1
1	1	0	0	0	1
1	0	0	0	0	1
0	1	0	0	0	1
1	1	0	0	0	1
0	0	0	0	0	0
1	0	1	0	0	1
1	0	1	0	0	1
0	0	0	0	0	0
0	1	0	0	0	1
0	0	0	0	0	0
0	0	0	0	0	1
0	0	0	0	0	0
0	0	1	0	0	1
1	0	0	0	0	1
0	0	1	0	0	1
0	1	0	0	0	1
0	0	0	0	0	0
0	1	0	0	0	1
1	1	0	0	0	1
1	1	0	0	0	1
1	1	0	0	0	1
1	0	0	0	0	1
0	0	1	0	0	1
1	0	1	0	0	1
0	0	0	0	0	1
1	0	0	0	0	1
1	0	1	0	0	1
1	1	0	0	0	1
1	0	0	0	0	1
1	0	0	0	0	1
1	0	0	0	0	0
1	0	1	0	0	0
0	0	0	0	0	1
0	1	0	0	0	1
1	0	1	0	0	1
1	0	0	0	0	0
1	0	0	0	0	1
1	1	0	0	0	0
1	1	0	0	0	1
1	0	1	0	0	1
0	0	0	0	0	1
0	0	0	0	0	0
0	0	0	0	0	0
1	0	0	0	0	0
1	0	0	0	0	0
0	0	0	0	0	0
0	0	0	0	0	1
1	0	0	0	0	1
1	0	0	0	0	0
0	0	1	0	0	0
0	0	0	0	0	0
0	0	0	0	0	0
0	0	0	0	0	0

0	0	0	0	0	0
0	0	0	0	0	0
0	0	0	0	0	0
0	0	0	0	0	0
0	0	0	0	0	0
0	0	0	0	0	0
0	0	0	0	0	0
0	0	0	0	0	0
0	0	0	0	0	0
0	0	0	0	0	0
1	0	0	0	0	0
0	0	0	0	0	0
1	0	0	0	0	0
0	0	0	0	0	0
0	0	0	0	0	0
0	0	0	0	0	1
0	0	0	0	0	1
0	0	0	0	0	1
1	0	0	0	0	1
0	0	0	0	0	1
0	0	0	0	0	0
0	0	0	0	0	1
0	0	1	0	0	0
0	0	0	0	0	0
1	0	0	0	0	0
1	0	0	0	0	0
0	0	0	0	0	0
0	0	0	0	0	0
1	0	0	0	0	0
0	0	0	0	0	0
0	0	0	0	0	0
1	0	0	0	0	0
1	0	0	0	0	0
0	0	0	0	0	0
0	0	0	0	0	0
0	0	0	0	0	1
0	0	0	0	0	0
0	1	0	0	0	1
1	0	0	0	0	0
1	1	0	0	0	1
0	0	0	0	0	0
0	0	0	0	0	0
0	0	0	0	0	0
0	0	0	0	0	0
0	0	0	0	0	0
0	0	0	0	0	0
0	0	0	0	0	0
0	0	0	0	0	0
1	0	0	0	0	1
1	0	0	0	0	0
0	0	0	0	0	0
0	0	0	0	0	0
0	0	0	0	0	0
0	0	0	0	0	0
0	0	0	0	0	0
0	0	0	0	0	0
0	0	0	0	0	0

0	0	0	0	0	0
1	0	0	0	0	1
0	0	0	0	0	0
0	0	0	0	0	0
0	0	0	0	0	0
1	0	0	0	0	1
0	0	0	0	0	1
0	0	0	0	0	0
1	0	0	0	0	0
0	0	1	0	0	0
0	1	0	0	0	1
0	0	0	0	0	1
1	1	0	0	0	1
0	0	0	0	0	0
0	0	0	0	0	0
0	0	0	0	0	1
0	0	0	0	0	1
1	0	0	0	0	1
1	1	0	0	0	0
0	0	1	0	0	1
0	1	0	0	0	1
1	0	0	0	0	0
0	0	0	0	0	0
1	0	0	0	0	0
0	0	0	0	0	0
0	0	0	0	0	0
0	0	0	0	0	0
1	0	0	0	0	0
0	0	0	0	0	0
1	0	0	0	0	1
1	0	1	0	0	1
0	1	0	0	0	1
1	1	0	0	0	1
1	1	0	0	0	1
1	0	0	0	0	1
0	0	0	0	0	0
0	0	0	0	0	0
0	0	0	0	0	1
0	0	1	0	0	0
0	0	0	0	0	0
0	0	0	0	0	0
0	0	1	0	0	1
0	0	0	0	0	0
0	0	0	0	0	0
0	0	0	0	0	0
1	0	0	0	0	0
0	0	0	0	0	0
0	0	0	0	0	0
0	0	0	0	0	0
1	0	0	0	0	0
0	0	0	0	0	0
1	0	0	0	0	0
0	0	0	0	0	0
0	0	0	0	0	0
1	0	0	0	0	0
0	0	0	0	0	0
0	0	0	0	0	0

0	0	0	0	0		0
0	0	0	0	0		0
0	0	0	0	0		0
0	0	0	0	0		0
1	0	0	0	0		1
0	0	0	0	0		0
1	0	0	0	0		0
0	0	0	0	0		0
0	0	0	0	0		0
1	0	0	0	0		0
1	0	0	0	0		1
1	0	0	0	0		1
0	0	0	0	0		1
1	0	1	0	0		1
0	0	0	0	0		1
0	0	0	0	0		1
0	0	0	0	0		1
0	0	0	0	0		1
0	0	0	0	0		0
0	0	1	0	0		1
1	0	0	0	0		0
1	0	0	0	0		0
1	0	0	0	0		1
0	0	0	0	0		0
0	0	0	0	0		1
1	0	0	0	0		1
0	0	0	0	0		0
0	0	0	0	0		1
1	0	0	0	0		1
0	0	0	0	0		1
0	0	0	0	0		0
0	0	0	0	0		0
0	0	0	0	0		1
0	0	0	0	0		0
0	0	0	0	0		0
1	0	0	0	0		0
0	0	0	0	0		1
1	0	0	0	0		1
0	0	0	0	0		0
0	0	0	0	0		0
0	0	0	0	0		0
0	0	0	0	0		0
1	0	0	0	0		0
0	0	0	0	0		1
0	0	0	0	0		1
1	0	0	0	0		1
0	0	0	0	0		0
1	0	0	0	0		0
0	0	0	0	0		0
0	0	0	0	0		0
1	0	0	0	0		0
0	0	0	0	0		0
0	0	0	0	0		0
1	0	0	0	0		0
0	0	0	0	0		0
0	0	0	0	0		1
0	0	0	0	0		1

0	0	0	0	0	1
0	0	0	0	0	0
0	0	0	0	0	1
0	1	0	0	0	1
0	1	0	0	0	1
0	0	0	0	0	0
0	0	0	0	0	1
0	0	0	0	0	0
0	0	0	0	0	0
0	1	0	0	0	1
0	0	0	0	0	0
0	0	0	0	0	1
0	0	0	0	0	0
0	1	0	0	0	0
0	1	0	0	0	1
0	0	0	0	0	1
0	0	1	0	0	1
0	0	0	0	0	0
0	0	0	0	0	0
0	0	0	0	0	0
0	1	0	0	0	1
0	0	0	0	0	1
0	0	0	0	0	0
0	0	0	0	0	0
0	0	0	0	0	0
0	0	0	0	0	1
0	0	0	0	0	0
0	0	0	0	0	0
0	0	0	0	0	1
0	1	0	0	0	1
1	0	1	0	0	0
1	0	0	0	0	0
1	1	0	0	0	0
0	0	0	0	0	0
0	1	0	0	0	1
0	1	0	0	0	1
1	1	0	0	0	1
1	0	1	0	0	1
0	0	0	0	0	1
0	0	1	0	0	1
0	0	1	0	0	1
1	0	0	0	0	1
1	0	0	0	0	0
0	0	0	0	0	1
1	1	0	0	0	1
0	1	0	0	0	1
0	1	0	0	0	1
0	1	0	0	0	1
0	0	0	0	0	1
0	0	0	0	0	1
0	0	1	0	0	1
0	0	1	0	0	1
0	0	0	0	0	1
1	1	0	0	0	1
0	0	1	0	0	1
0	1	0	0	0	1
1	0	0	0	0	1

1	0	1	0	0	0
1	0	1	0	0	0
1	0	0	0	0	0
1	1	0	0	0	0
1	1	0	0	0	0
1	1	0	0	0	1
1	0	0	0	0	0
0	0	1	0	0	1
0	0	1	0	0	1
0	0	1	0	0	0
0	1	0	0	0	1
0	0	0	0	0	1
1	0	0	0	0	1
0	0	0	0	0	1
0	0	0	0	0	1
0	1	0	0	0	1
0	0	1	0	0	1
0	0	0	0	0	0
0	0	0	0	0	0
0	1	0	0	0	1
0	0	0	0	0	0
1	0	0	0	0	0
0	0	0	0	0	1
1	0	1	0	0	0
1	0	0	0	0	1
1	1	0	0	0	1
0	1	0	0	0	1
1	0	0	0	0	0
1	0	1	0	0	1
0	0	1	0	0	1
1	1	0	0	0	1
1	0	0	0	0	0
1	0	0	0	0	1
0	1	0	0	0	1
0	0	1	0	0	0
0	0	0	0	0	1
0	0	0	0	0	0
0	0	0	0	0	1
0	0	0	0	0	1
0	0	0	0	0	0
0	0	0	0	0	0
1	1	0	0	0	1
0	1	0	0	0	1
1	0	0	0	0	1
0	0	0	0	0	1
1	0	0	0	0	0
1	0	0	0	0	1
1	0	0	0	0	1
1	0	0	0	0	1
0	0	0	0	0	1
0	0	0	0	0	1
0	0	0	0	0	1
0	0	0	0	0	1
0	0	0	0	0	1
0	0	0	0	0	1
0	0	0	0	0	1
0	0	0	0	0	1

0	0	0	0	0	1
0	0	0	0	0	1
1	0	0	0	0	1
1	0	0	0	0	1
1	0	0	0	0	1
0	0	0	0	0	1
0	0	0	0	0	1
0	0	0	0	0	1
0	0	0	0	0	1
0	0	0	0	0	1
0	0	0	0	0	1
0	0	0	0	0	1
0	0	0	0	0	1
0	0	0	0	0	1
0	0	0	0	0	1
0	0	0	0	0	1
0	0	0	0	0	1
1	0	0	0	0	1
0	0	0	0	0	1
1	0	0	0	0	1
1	0	0	0	0	1
1	0	0	0	0	1
1	0	0	0	0	1
1	0	0	0	0	1
1	0	0	0	0	1
1	0	0	0	0	1
1	0	0	0	0	1
0	0	0	0	0	1
1	0	0	0	0	1
1	0	0	0	0	1
0	0	0	0	0	1
0	0	0	0	0	1
0	0	0	0	0	1
1	0	0	0	0	1
1	0	0	0	0	1
1	0	0	0	0	1
1	0	0	0	0	1
1	0	0	0	0	1
1	0	0	0	0	1
1	0	0	0	0	1
1	0	0	0	0	1
1	0	0	0	0	1
1	0	0	0	0	1
1	0	0	0	0	1
1	0	0	0	0	1
1	0	0	0	0	1
1	0	0	0	0	1
1	0	0	0	0	1
1	0	0	0	0	1
1	0	0	0	0	0
0	0	0	0	0	1
0	0	0	0	0	1
1	0	0	0	0	1
1	0	0	0	0	1
1	0	0	0	0	1
1	0	0	0	0	1
1	0	0	0	0	1

1	0	0	0	0	1
1	0	0	0	0	1
1	0	0	0	0	1
0	0	0	0	0	1
1	0	0	0	0	1
1	0	0	0	0	1
1	0	0	0	0	1
1	0	0	0	0	1
1	0	0	0	0	1
1	0	0	0	0	1
1	0	0	0	0	1
1	0	0	0	0	1
1	0	0	0	0	1
0	0	0	0	0	1
0	0	0	0	0	1
0	0	0	0	0	1
1	0	0	0	0	1
1	0	0	0	0	1
1	0	0	0	0	1
1	0	0	0	0	1
1	0	0	0	0	1
1	0	0	0	0	1
1	0	0	0	0	1
1	0	0	0	0	1
0	0	0	0	0	1
1	0	0	0	0	1
1	0	0	0	0	1
1	0	0	0	0	1
1	0	0	0	0	1
1	0	0	0	0	1
1	0	0	0	0	1
1	0	0	0	0	1
1	0	0	0	0	1
1	0	0	0	0	1
1	0	0	0	0	1
0	0	0	0	0	1
1	0	0	0	0	1
1	0	0	0	0	1
1	0	0	0	0	1
1	0	0	0	0	1
0	0	0	0	0	1
0	0	0	0	0	1
1	0	0	0	0	1
1	0	0	0	0	1
1	0	0	0	0	1
1	0	0	0	0	1
1	0	0	0	0	1
1	0	0	0	0	1
1	0	0	0	0	1
1	0	0	0	0	1
1	0	0	0	0	1
1	0	0	0	0	1
0	0	0	0	0	0
0	1	0	0	0	1
0	1	0	0	0	1
0	0	1	0	0	1
0	0	0	0	0	0

0	0	0	0	0	0
1	1	0	0	0	1
0	0	0	0	0	1
0	0	0	0	0	0
0	0	0	0	0	0
1	0	0	0	0	0
1	0	1	0	0	1
0	1	0	0	0	0
0	1	0	0	0	0
0	0	0	0	0	1
1	0	0	0	0	0
0	0	0	0	0	1
0	0	1	0	0	1
1	0	1	0	0	1
0	0	0	0	0	0
1	0	1	0	0	1
0	0	1	0	0	1
0	0	1	0	0	0
0	0	0	0	0	0
0	0	0	0	0	0
1	0	0	0	0	0
0	0	0	0	0	1
0	1	0	0	0	1
0	0	0	0	0	1
1	0	0	0	0	0
1	0	0	0	0	1
1	0	0	0	0	1
1	0	0	0	0	0
1	1	0	0	0	0
1	0	0	0	0	0
1	0	0	0	0	1
0	0	0	0	0	1
0	0	0	0	0	0
0	0	0	0	0	0
0	0	0	0	0	0
1	0	0	0	0	1
1	0	0	0	0	0
0	0	0	0	0	1
1	0	0	0	0	1
1	0	0	0	0	1
1	0	0	0	0	1
1	0	0	0	0	1
1	0	0	0	0	0
1	0	0	0	0	0
0	0	0	0	0	0
0	0	0	0	0	1
1	0	0	0	0	0
1	0	0	0	0	0
0	0	0	0	0	0
0	0	0	0	0	1
0	0	0	0	0	0
1	0	0	0	0	0
1	0	0	0	0	1
1	0	0	0	0	1
0	0	0	0	0	1
0	0	0	0	0	1
1	0	0	0	0	0

0	0	0	0	0	0
0	0	0	0	0	0
0	0	0	0	0	0
1	0	0	0	0	0
0	0	0	0	0	0
0	0	0	0	0	0
1	0	0	0	0	1
0	0	0	0	0	0
0	0	0	0	0	1
1	0	0	0	0	0
1	0	0	0	0	0
1	0	0	0	0	0
1	0	0	0	0	0
0	0	0	0	0	0
0	0	0	0	0	0
1	0	0	0	0	1
0	0	0	0	0	0
0	0	0	0	0	0
0	0	0	0	0	1
0	0	0	0	0	1
0	0	0	0	0	0
1	0	0	0	0	0
1	0	0	0	0	1
1	0	0	0	0	1
0	0	0	0	0	1
0	0	0	0	0	0
0	0	0	0	0	1
1	0	0	0	0	1
0	0	0	0	0	0
0	0	0	0	0	1
0	0	0	0	0	0
1	0	0	0	0	1
0	0	0	0	0	1
0	0	0	0	0	0
0	0	0	0	0	0
0	0	0	0	0	0
1	0	0	0	0	0
0	0	0	0	0	0
1	0	0	0	0	1
1	0	0	0	0	0
0	0	0	0	0	0
0	0	0	0	0	0
0	0	0	0	0	0
0	0	0	0	0	1
1	0	0	0	0	1
1	0	0	0	0	1
1	0	0	0	0	1
0	0	0	0	0	0
1	0	0	0	0	0
1	0	0	0	0	0
0	0	0	0	0	1
1	0	0	0	0	0
0	0	0	0	0	1
0	0	0	0	0	0
0	0	0	0	0	1
0	0	0	0	0	1
0	0	0	0	0	0

1	0	0	0	0	1
0	0	0	0	0	1
1	0	0	0	0	0
1	0	0	0	0	0
0	0	0	0	0	0
0	0	0	0	0	0
0	0	0	0	0	1
0	0	0	0	0	0
0	0	0	0	0	0
0	0	0	0	0	0
1	0	0	0	0	1
0	0	0	0	0	0
0	0	0	0	0	1
0	0	0	0	0	0
0	0	0	0	0	1
0	0	0	0	0	1
0	0	0	0	0	1
1	0	1	0	0	1
0	0	0	0	0	0
1	0	0	0	0	1
0	0	0	0	0	0
1	0	0	0	0	0
0	0	0	0	0	0
1	0	0	0	0	0
1	0	0	0	0	1
0	0	0	0	0	1
1	0	0	0	0	0
1	0	0	0	0	0
0	0	0	0	0	1
0	0	0	0	0	1
0	0	0	0	0	1
0	0	0	0	0	1
0	0	0	0	0	0
0	0	0	0	0	1
1	0	0	0	0	0
0	0	0	0	0	0
0	0	0	0	0	0
1	0	0	0	0	1
1	0	0	0	0	0
1	0	0	0	0	1
1	0	0	0	0	0
0	0	0	0	0	0
0	0	0	0	0	1
0	0	0	0	0	1
1	0	0	0	0	0
0	0	0	0	0	1
1	0	0	0	0	0
0	0	0	0	0	0
1	0	0	0	0	0
0	0	0	0	0	1
0	0	0	0	0	0
1	0	0	0	0	1
1	0	0	0	0	0
0	0	0	0	0	0
1	0	0	0	0	1
1	0	0	0	0	1
0	0	0	0	0	0

0	0	0	0	0	0
0	0	0	0	0	0
0	0	0	0	0	0
0	0	0	0	0	0
1	0	0	0	0	0
0	0	0	0	0	1
0	0	0	0	0	0
1	0	0	0	0	0
0	0	0	0	0	0
0	0	0	0	0	1
1	0	0	0	0	0
1	0	0	0	0	0
1	0	0	0	0	0
0	0	0	0	0	1
0	0	0	0	0	0
1	0	0	0	0	0
1	0	0	0	0	0
1	0	0	0	0	0
1	0	0	0	0	0
1	0	0	0	0	0
1	0	0	0	0	0
1	0	0	0	0	0
0	0	0	0	0	0
1	0	0	0	0	0
1	0	0	0	0	0
0	0	0	0	0	1
1	0	0	0	0	0
1	0	0	0	0	1
0	0	0	0	0	0
0	0	0	0	0	1
1	0	0	0	0	1
0	0	0	0	0	0
0	0	0	0	0	0
0	0	0	0	0	1
0	0	0	0	0	0
0	0	0	0	0	0
1	0	0	0	0	0
0	0	0	0	0	1
0	0	0	0	0	1
0	0	0	0	0	0
0	0	0	0	0	0
1	0	1	0	0	0
0	0	1	0	0	1
0	0	0	0	0	0
1	0	0	0	0	0
1	0	0	0	0	0
0	0	0	0	0	1
1	0	0	0	0	1
0	1	0	0	0	1
0	0	0	0	0	1
1	0	0	0	0	1
1	0	0	0	0	1
0	0	0	0	0	1
1	0	0	0	0	1
0	0	0	0	0	0
1	0	0	0	0	0
1	0	1	0	0	0

0	1	0	0	0	1
0	0	1	0	0	1
0	0	0	0	0	1
1	0	1	0	0	0
0	0	0	0	0	0
1	0	0	0	0	1
0	0	0	0	0	0
1	0	1	0	0	1
1	0	0	0	0	1
1	0	1	0	0	0
0	1	0	0	0	1
0	0	0	0	0	1
1	1	0	0	0	1
0	0	1	0	0	1
0	1	0	0	0	1
0	0	0	0	0	1
0	0	1	0	0	0
1	0	0	0	0	1
1	0	1	0	0	1
0	0	1	0	0	0
0	0	0	0	0	1
0	0	0	0	0	1
1	0	0	0	0	0
1	0	0	0	0	1
0	0	1	0	0	0
0	0	0	0	0	1
1	0	0	0	0	1
0	0	0	0	0	0
0	1	0	0	0	1
1	1	0	0	0	1
1	0	0	0	0	1
1	0	0	0	0	1
0	1	0	0	0	1
0	0	0	0	0	1
1	0	1	0	0	1
0	1	0	0	0	1
1	0	0	0	0	0
0	1	0	0	0	1
1	0	1	0	0	1
1	0	1	0	0	1
0	1	0	0	0	1
1	1	0	0	0	1
0	0	0	0	0	1
1	0	1	0	0	0
1	0	0	0	0	0
1	1	0	0	0	1
1	1	0	0	0	1
1	1	0	0	0	1
0	0	1	0	0	1
1	0	1	0	0	1
1	0	0	0	0	1
0	0	0	0	0	1
0	1	0	0	0	1
1	1	0	0	0	1
1	0	0	0	0	1
1	0	0	0	0	1
1	0	1	0	0	0

0	0	0	0	0	1
0	0	0	0	0	0
0	0	0	0	0	0
0	0	0	0	0	0
1	0	0	0	0	0
1	0	0	0	0	0
0	0	0	0	0	0
0	0	0	0	0	0
0	0	0	0	0	1
0	0	0	0	0	0
0	0	0	0	0	1
1	0	0	0	0	1
1	0	0	0	0	0
1	0	0	0	0	0
0	0	0	0	0	1
0	0	0	0	0	1
0	0	0	0	0	1
0	0	0	0	0	1
0	0	0	0	0	1
0	0	0	0	0	0
0	0	0	0	0	0
1	0	0	0	0	1
1	1	0	0	0	0
0	0	0	0	0	0
1	0	0	0	0	0
0	0	0	0	0	0
0	0	0	0	0	0
0	0	0	0	0	1
1	0	0	0	0	0
0	0	0	0	0	0
0	0	0	0	0	0
0	0	0	0	0	0
0	0	0	0	0	0
0	0	0	0	0	0
0	0	0	0	0	0
0	0	0	0	0	0
0	0	0	0	0	0
0	0	0	0	0	0
0	0	0	0	0	0
0	0	0	0	0	0
0	0	0	0	0	0
0	0	0	0	0	0
0	0	0	0	0	0
0	0	0	0	0	0
0	0	0	0	0	0
0	0	0	0	0	0
0	0	0	0	0	0
0	0	0	0	0	0
0	0	0	0	0	0
0	0	0	0	0	0
0	0	0	0	0	0
0	0	0	0	0	0
0	0	0	0	0	0
0	0	0	0	0	0
0	0	0	0	0	0
0	0	0	0	0	0
0	0	0	0	0	1

0	0	0	0	0	0
0	0	0	0	0	0
0	0	0	0	0	0
0	0	0	0	0	0
0	0	0	0	0	0
0	0	0	0	0	1
0	0	0	0	0	1
0	0	0	0	0	0
0	0	0	0	0	1
0	0	0	0	0	0
0	0	0	0	0	1
0	0	0	0	0	0
0	0	0	0	0	0
0	0	0	0	0	0
0	0	0	0	0	0
0	0	0	0	0	0
0	0	0	0	0	1
0	0	0	0	0	0
1	0	0	0	0	1
1	0	0	0	0	1
1	0	0	0	0	1
0	0	0	0	0	1
0	0	0	0	0	1
0	0	0	0	0	1
1	0	0	0	0	1
1	0	0	0	0	1
1	0	0	0	0	1
1	0	0	0	0	1
1	0	0	0	0	1
1	0	0	0	0	1
1	0	0	0	0	1
1	0	0	0	0	1
1	0	0	0	0	1
1	0	0	0	0	1
1	0	0	0	0	1
1	0	0	0	0	1
0	0	0	0	0	1
1	0	0	0	0	1
1	0	0	0	0	1
1	0	0	0	0	1
1	0	0	0	0	1
1	0	0	0	0	1
1	0	0	0	0	1
0	0	0	0	0	1
1	0	0	0	0	1
1	0	0	0	0	1
0	0	0	0	0	1
1	0	0	0	0	1
1	0	0	0	0	1
1	0	0	0	0	1
1	0	0	0	0	1
0	0	0	0	0	1
1	0	0	0	0	1
1	0	0	0	0	1
0	0	0	0	0	1
0	0	0	0	0	1
1	0	0	0	0	1

1	0	0	0	0	1
1	0	0	0	0	1
1	0	0	0	0	1
1	0	0	0	0	1
1	0	0	0	0	1
1	0	0	0	0	1
1	0	0	0	0	1
1	0	0	0	0	1
0	0	0	0	0	1
0	0	0	0	0	1
0	0	0	0	0	1
0	0	0	0	0	1
0	0	0	0	0	1
1	0	0	0	0	1
0	0	0	0	0	1
1	0	0	0	0	1
1	0	0	0	0	1
1	0	0	0	0	1
1	0	0	0	0	1
0	0	0	0	0	1
1	0	0	0	0	1
1	0	0	0	0	1
1	0	0	0	0	1
0	1	0	0	0	1
1	1	0	0	0	1
0	0	0	0	0	1
1	1	0	0	0	1
0	0	0	0	0	0
0	0	0	0	0	0
0	1	0	0	0	1
0	1	0	0	0	1
0	1	0	0	0	1
0	1	0	0	0	1
1	0	0	0	0	1
1	1	0	0	0	1
1	1	0	0	0	1
0	0	0	0	0	1
0	1	0	0	0	1
0	0	1	0	0	1
0	0	1	0	0	1
0	1	0	0	0	1
0	1	0	0	0	1
1	0	0	0	0	1
1	0	0	0	0	1
0	0	0	0	0	1
0	1	0	0	0	1
0	1	0	0	0	1
0	1	0	0	0	1
0	0	0	0	0	1
0	1	0	0	0	1
0	0	1	0	0	1
1	1	0	0	0	1
1	0	0	0	0	0
1	0	0	0	0	0
1	0	1	0	0	1
0	1	0	0	0	1
0	1	0	0	0	1

0	0	0	0	0	1
0	1	0	0	0	1
0	1	0	0	0	1
0	0	0	0	0	0
0	0	1	0	0	1
0	1	0	0	0	1
1	0	1	0	0	1
0	1	0	0	0	1
0	1	0	0	0	1
1	0	0	0	0	1
1	0	1	0	0	1
1	1	0	0	0	1
0	1	0	0	0	1
0	0	1	0	0	1
0	1	0	0	0	1
0	1	0	0	0	1
0	0	1	0	0	1
0	0	0	0	0	0
0	1	0	0	0	1
1	0	1	0	0	0
0	0	1	0	0	1
0	0	1	0	0	1
1	0	0	0	0	1
1	0	1	0	0	1
1	1	0	0	0	1
0	0	0	0	0	1
0	0	0	0	0	1
1	0	0	0	0	1
1	1	0	0	0	1
1	0	1	0	0	1
1	1	0	0	0	0
0	1	0	0	0	1
0	0	1	0	0	1
1	0	1	0	0	1
0	1	0	0	0	1
0	0	1	0	0	1
1	1	0	0	0	0
0	0	0	0	0	0
1	0	1	0	0	0
1	0	1	0	0	1
0	0	0	0	0	1
0	0	1	0	0	1
1	1	0	0	0	1
0	1	0	0	0	1
1	0	0	0	0	0
0	0	0	0	0	1
0	0	1	0	0	0
1	1	0	0	0	1
1	1	0	0	0	1
0	1	0	0	0	1
1	0	1	0	0	1
1	0	0	0	0	1
1	0	1	0	0	1
0	1	0	0	0	1
0	0	1	0	0	1
1	1	0	0	0	1
0	0	0	0	0	1

1	0	0	0	0	1
1	0	0	0	0	1
0	1	0	0	0	1
1	0	0	0	0	0
0	0	0	0	0	1
1	0	0	0	0	0
0	0	0	0	0	0
0	0	0	0	0	0
0	0	0	0	0	0
0	0	0	0	0	1
0	0	0	0	0	0
0	0	0	0	0	0
0	0	0	0	0	1
0	0	0	0	0	1
0	0	0	0	0	1
0	0	1	0	0	1
1	0	1	0	0	0
0	0	0	0	0	0
0	0	1	0	0	0
0	1	0	0	0	1
0	0	0	0	0	0
1	0	1	0	0	0
0	0	0	0	0	1
1	0	0	0	0	1
0	0	0	0	0	1
1	0	0	0	0	0
0	0	1	0	0	1
0	0	1	0	0	1
0	0	0	0	0	0
1	0	0	0	0	1
1	0	0	0	0	0
1	0	0	0	0	0
0	0	0	0	0	0
0	0	1	0	0	0
1	1	0	0	0	1
0	0	0	0	0	1
0	0	0	0	0	1
0	1	0	0	0	0
0	0	1	0	0	1
0	0	1	0	0	1
0	0	1	0	0	1
1	0	0	0	0	1
1	1	0	0	0	1
0	0	1	0	0	0
0	0	0	0	0	0
1	1	0	0	0	1
0	1	0	0	0	1
0	0	0	0	0	1
1	0	0	0	0	0
0	0	0	0	0	0
0	0	0	0	0	1
1	0	0	0	0	0
0	0	0	0	0	0
0	0	0	0	0	1
0	0	1	0	0	0
0	0	0	0	0	1
0	0	0	0	0	0

0	0	1	0	0	1
0	0	0	0	0	0
0	1	0	0	0	1
1	0	0	0	0	1
1	1	0	0	0	1
0	1	0	0	0	1
1	0	1	0	0	1
1	0	0	0	0	0
1	0	1	0	0	1
0	1	0	0	0	1
1	1	0	0	0	1
1	1	0	0	0	1
1	1	0	0	0	1
1	0	0	0	0	1
1	0	0	0	0	0
1	0	0	0	0	0
0	0	0	0	0	0
0	0	0	0	0	0
0	0	0	0	0	0
0	0	0	0	0	0
0	0	0	0	0	0
0	0	0	0	0	0
0	0	0	0	0	0
0	0	0	0	0	1
1	0	0	0	0	0
0	0	0	0	0	0
0	0	0	0	0	0
0	0	0	0	0	0
1	0	0	0	0	0
0	0	0	0	0	0
1	0	0	0	0	0
1	1	0	0	0	0
1	0	0	0	0	0
1	0	0	0	0	0
1	0	0	0	0	0
1	0	0	0	0	0
0	0	1	0	0	1
1	0	0	0	0	0
0	1	0	0	0	1
1	1	0	0	0	1
1	0	0	0	0	0
0	1	0	0	0	1
0	0	1	0	0	0
0	0	0	0	0	1
1	0	0	0	0	1
0	0	1	0	0	1
0	1	0	0	0	1
1	1	0	0	0	1
1	0	1	0	0	1
0	0	0	0	0	0
0	0	0	0	0	1
1	0	1	0	0	1
0	0	1	0	0	1
0	0	0	0	0	1
0	1	0	0	0	1
0	0	1	0	0	0
1	0	0	0	0	1

0	0	1	0	0	1
1	1	0	0	0	1
1	0	0	0	0	1
1	0	0	0	0	1
0	0	1	0	0	1
1	0	1	0	0	1
0	0	0	0	0	1
1	0	0	0	0	1
0	0	0	0	0	1
1	0	0	0	0	0
1	1	0	0	0	1
0	1	0	0	0	1
1	0	1	0	0	0
0	0	1	0	0	1
0	1	0	0	0	1
0	0	1	0	0	1
1	1	0	0	0	1
0	1	0	0	0	1
1	0	0	0	0	0
0	0	1	0	0	1
0	0	0	0	0	1
1	0	0	0	0	0
1	1	0	0	0	1
0	0	0	0	0	0
0	1	0	0	0	1
0	0	0	0	0	0
1	0	0	0	0	0
1	0	1	0	0	0
0	0	0	0	0	0
0	0	0	0	0	0
0	0	1	0	0	0
1	0	0	0	0	1
0	1	0	0	0	1
1	0	0	0	0	1
1	0	0	0	0	0
1	0	0	0	0	0
1	0	0	0	0	0
1	1	0	0	0	1
0	0	0	0	0	0
0	0	0	0	0	0
1	0	0	0	0	0
0	0	0	0	0	1
0	0	1	0	0	0
1	0	0	0	0	0
1	0	0	0	0	0
1	0	0	0	0	0
1	0	0	0	0	0
1	0	0	0	0	0
1	0	0	0	0	0
0	0	0	0	0	0
1	0	0	0	0	0
1	0	0	0	0	0
1	0	0	0	0	0
1	0	0	0	0	1
1	0	1	0	0	0
1	0	0	0	0	0
1	0	0	0	0	0

1	0	0	0	0	0
1	0	0	0	0	0
1	1	0	0	0	1
1	1	0	0	0	1
1	0	1	0	0	1
1	0	0	0	0	0
0	0	0	0	0	0
1	0	0	0	0	0
1	0	0	0	0	1
1	0	0	0	0	1
1	1	0	0	0	1
0	1	0	0	0	1
0	1	0	0	0	1
0	0	0	0	0	0
1	1	0	0	0	1
0	0	1	0	0	1
0	1	0	0	0	1
1	0	1	0	0	0
0	0	0	0	0	0
0	0	1	0	0	0
0	0	0	0	0	1
1	0	1	0	0	1
0	0	1	0	0	1
1	0	0	0	0	0
1	0	0	0	0	1
0	0	0	0	0	0
0	0	0	0	0	0
0	0	0	0	0	0
0	0	0	0	0	0
0	0	0	0	0	0
0	0	0	0	0	0
0	0	0	0	0	0
0	0	0	0	0	0
0	0	0	0	0	0
0	0	0	0	0	0
0	0	0	0	0	0
0	0	0	0	0	0
0	0	0	0	0	0
0	0	0	0	0	1
0	0	0	0	0	0
0	0	0	0	0	0
0	0	0	0	0	0
0	0	0	0	0	0
0	0	0	0	0	0
0	0	0	0	0	0
0	0	0	0	0	0
0	0	0	0	0	0
0	0	0	0	0	0
0	0	0	0	0	0
0	0	0	0	0	0
0	0	0	0	0	0
0	0	0	0	0	0
0	0	0	0	0	0
0	0	0	0	0	0
0	0	0	0	0	0
0	0	0	0	0	0
0	0	0	0	0	0

0	0	0	0	0	0
0	0	0	0	0	0
0	0	0	0	0	0
0	0	0	0	0	0
0	0	0	0	0	0
0	0	0	0	0	0
0	0	0	0	0	0
0	0	0	0	0	0
0	1	0	0	0	1
0	1	0	0	0	1
0	0	0	0	0	1
1	0	0	0	0	0
1	0	0	0	0	0
0	0	0	0	0	0
0	0	0	0	0	1
0	0	0	0	0	1
1	0	0	0	0	1
1	0	0	0	0	1
1	0	0	0	0	1
1	0	0	0	0	1
1	0	0	0	0	0
1	0	0	0	0	0
1	0	0	0	0	0
1	1	0	0	0	1
1	1	0	0	0	1
0	1	0	0	0	1
0	0	0	0	0	0
0	0	0	0	0	0
1	0	0	0	0	0
1	0	0	0	0	0
1	0	0	0	0	0
0	0	0	0	0	0
0	0	0	0	0	0
0	0	0	0	0	0
0	0	0	0	0	0
0	0	0	0	0	0
0	0	0	0	0	0
0	0	0	0	0	0
0	0	0	0	0	0
0	0	0	0	0	0
0	0	0	0	0	0
0	0	0	0	0	0
0	0	0	0	0	0
0	0	0	0	0	0
0	0	0	0	0	0
0	0	0	0	0	0
1	0	0	0	0	0
0	0	0	0	0	0
0	0	0	0	0	0
0	0	0	0	0	0
0	0	0	0	0	1
1	0	0	0	0	0
1	0	0	0	0	0
1	0	1	0	0	1
0	1	0	0	0	1
0	1	0	0	0	1
1	0	0	0	0	1

1	0	0	0	0	1
0	0	1	0	0	1
0	0	0	0	0	1
1	0	0	0	0	0
1	0	0	0	0	1
1	0	0	0	0	1
1	0	0	0	0	1
1	0	0	0	0	1
0	0	1	0	0	1
0	0	0	0	0	0
0	0	0	0	0	0
1	0	0	0	0	0
0	0	0	0	0	1
1	1	0	0	0	1
1	0	1	0	0	1
0	1	0	0	0	1
1	0	0	0	0	1
0	0	0	0	0	1
0	1	0	0	0	1
1	0	0	0	0	0
1	0	0	0	0	0
1	0	0	0	0	0
0	0	0	0	0	0
0	0	0	0	0	0
1	0	0	0	0	0
1	0	0	0	0	0
1	0	0	0	0	0
1	0	0	0	0	0
1	0	0	0	0	0
1	0	0	0	0	0
1	0	0	0	0	0
1	0	0	0	0	0
1	0	0	0	0	0
1	0	0	0	0	0
1	0	0	0	0	0
1	0	1	0	0	1
1	0	0	0	0	1
1	0	0	0	0	0
1	0	0	0	0	0
1	0	0	0	0	0
1	0	0	0	0	0
0	0	0	0	0	1
0	0	0	0	0	0
0	1	0	0	0	1
1	1	0	0	0	1
1	0	0	0	0	1
1	0	0	0	0	0
0	0	0	0	0	0
1	0	0	0	0	0
1	0	0	0	0	1
1	0	0	0	0	0
1	0	0	0	0	0
0	0	0	0	0	0
0	0	0	0	0	1
0	0	0	0	0	0
0	0	0	0	0	1
1	0	0	0	0	1

1	0	1	0	0	1
1	1	0	0	0	1
0	1	0	0	0	1
1	0	0	0	0	1
0	0	0	0	0	0
1	0	1	0	0	0
0	1	0	0	0	1
0	0	1	0	0	0
1	0	1	0	0	0
1	0	1	0	0	1
1	1	0	0	0	0
1	0	1	0	0	0
1	1	0	0	0	0
0	0	0	0	0	1
0	0	0	0	0	0
1	1	0	0	0	1
1	1	0	0	0	1
1	1	0	0	0	0
1	0	1	0	0	0
0	1	0	0	0	1
1	0	1	0	0	1
1	0	0	0	0	0
0	0	1	0	0	1
0	1	0	0	0	1
1	0	0	0	0	0
1	0	0	0	0	0
0	0	0	0	0	1
0	0	0	0	0	0
1	0	0	0	0	1
0	1	0	0	0	1
0	0	0	0	0	0
0	1	0	0	0	1
0	1	0	0	0	0
1	1	0	0	0	1
1	0	1	0	0	1
0	0	0	0	0	1
0	0	0	0	0	0
0	0	0	0	0	0
1	0	0	0	0	0
0	0	0	0	0	0
1	0	0	0	0	1
1	0	0	0	0	1
0	0	1	0	0	1
1	1	0	0	0	1
0	1	0	0	0	1
1	0	1	0	0	1
0	1	0	0	0	0
0	0	0	0	0	0
0	0	0	0	0	1
0	0	0	0	0	1
1	0	0	0	0	0
1	0	0	0	0	0
1	1	0	0	0	0
0	0	0	0	0	0
1	0	0	0	0	0
1	0	0	0	0	1
1	0	0	0	0	1

1	0	0	0	0	0
1	0	0	0	0	1
0	0	0	0	0	1
0	0	1	0	0	0
1	0	0	0	0	0
1	0	0	0	0	0
1	0	0	0	0	1
0	0	0	0	0	0
0	0	0	0	0	0
1	0	0	0	0	1
1	0	0	0	0	1
1	1	0	0	0	1
1	1	0	0	0	1
0	0	0	0	0	1
0	0	0	0	0	1
0	1	0	0	0	1
0	1	0	0	0	1
1	0	1	0	0	1
0	1	0	0	0	1
1	1	0	0	0	1
0	0	0	0	0	0
0	0	0	0	0	0
0	0	0	0	0	0
0	0	0	0	0	0
0	0	0	0	0	0
0	0	0	0	0	0
0	0	0	0	0	0
0	1	0	0	0	1
1	0	0	0	0	0
1	1	0	0	0	1
0	1	0	0	0	1
0	1	0	0	0	1
0	0	1	0	0	1
1	0	0	0	0	1
0	1	0	0	0	1
1	1	0	0	0	1
0	0	0	0	0	0
0	0	0	0	0	1
0	0	0	0	0	1
1	0	0	0	0	1
1	1	0	0	0	1
0	0	1	0	0	1
0	0	0	0	0	0
0	0	0	0	0	0
1	0	0	0	0	0
1	0	0	0	0	1
1	0	0	0	0	0
0	1	0	0	0	1
1	0	0	0	0	1
0	0	0	0	0	1
0	0	0	0	0	1
0	0	0	0	0	0
0	0	0	0	0	0
1	0	0	0	0	1
1	1	0	0	0	0
1	0	0	0	0	1
0	0	1	0	0	1

0	0	1	0	0	1
1	0	0	0	0	0
0	0	0	0	0	0
0	0	0	0	0	1
0	0	0	0	0	0
0	0	0	0	0	0
1	0	0	0	0	0
0	0	0	0	0	0
0	0	0	0	0	1
1	1	0	0	0	1
1	0	1	0	0	1
1	0	0	0	0	1
0	1	0	0	0	1
0	0	1	0	0	1
1	1	0	0	0	1
1	1	0	0	0	1
1	0	0	0	0	1
1	1	0	0	0	1
1	1	0	0	0	1
0	0	0	0	0	1
1	0	0	0	0	1
0	0	1	0	0	1
1	0	0	0	0	0
0	1	0	0	0	1
1	1	0	0	0	1
0	0	0	0	0	0
0	0	0	0	0	0
1	0	1	0	0	1
0	1	0	0	0	1
0	0	0	0	0	0
0	0	0	0	0	0
0	0	0	0	0	1
0	0	0	0	0	0
0	0	0	0	0	1
0	0	0	0	0	0
0	0	0	0	0	0
0	1	0	0	0	1
0	0	0	0	0	0
1	0	0	0	0	1
1	0	0	0	0	0
1	0	0	0	0	0
0	0	0	0	0	0
1	0	0	0	0	0
1	0	1	0	0	0
1	0	0	0	0	1
1	0	0	0	0	1
0	0	0	0	0	1
0	0	1	0	0	1
0	0	1	0	0	1
0	0	0	0	0	1
0	0	0	0	0	0
0	0	0	0	0	0
0	1	0	0	0	1
0	0	0	0	0	1
0	0	0	0	0	0
0	0	1	0	0	1
0	1	0	0	0	1

0	0	0	0	0	0
0	0	0	0	0	1
0	0	0	0	0	1
0	1	0	0	0	1
0	1	0	0	0	1
1	1	0	0	0	1
0	0	1	0	0	1
0	0	1	0	0	1
1	0	0	0	0	1
1	0	0	0	0	1
1	0	0	0	0	1
0	0	0	0	0	1
0	1	0	0	0	1
0	1	0	0	0	1
0	1	0	0	0	1
1	0	0	0	0	0
0	0	0	0	0	0
0	0	0	0	0	1
0	0	0	0	0	1
0	0	0	0	0	1
1	0	0	0	0	0
1	0	0	0	0	0
1	0	0	0	0	1
1	0	1	0	0	1
0	0	0	0	0	0
0	0	0	0	0	0
0	0	0	0	0	0
1	1	0	0	0	1
0	0	0	0	0	0
1	0	0	0	0	0
1	0	0	0	0	0
1	0	0	0	0	0
1	1	0	0	0	1
0	0	1	0	0	1
1	1	0	0	0	1
1	1	0	0	0	0
0	1	0	0	0	1
0	1	0	0	0	1
0	0	1	0	0	1
1	0	1	0	0	1
0	1	0	0	0	1
1	1	0	0	0	1
0	0	0	0	0	1
1	0	1	0	0	1
0	0	1	0	0	1
1	0	0	0	0	1
1	1	0	0	0	1
1	0	1	0	0	1
0	0	0	0	0	0
0	0	0	0	0	0
0	0	0	0	0	0
1	0	0	0	0	0
0	1	0	0	0	1
0	0	0	0	0	0
0	0	0	0	0	0
0	1	0	0	0	1
0	0	0	0	0	0

1	0	0	0	0	0
0	0	0	0	0	0
0	0	0	0	0	0
0	0	0	0	0	0
0	0	0	0	0	0
0	0	0	0	0	1
0	0	0	0	0	0
1	0	0	0	0	0
1	0	1	0	0	1
0	0	0	0	0	1
0	1	0	0	0	1
1	1	0	0	0	1
1	1	0	0	0	1
0	0	0	0	0	1
0	0	0	0	0	0
0	1	0	0	0	0
0	0	0	0	0	0
0	0	0	0	0	0
0	0	0	0	0	0
0	1	0	0	0	1
0	1	0	0	0	1
0	0	0	0	0	0
0	0	0	0	0	1
1	0	0	0	0	1
0	0	0	0	0	0
1	0	0	0	0	0
0	0	0	0	0	1
0	0	0	0	0	0
0	0	0	0	0	0
0	0	0	0	0	0
0	0	0	0	0	0
0	0	0	0	0	0
0	0	0	0	0	0
0	0	0	0	0	0
1	0	0	0	0	0
1	0	0	0	0	0
0	0	0	0	0	1
0	0	0	0	0	0
0	0	1	0	0	1
1	0	1	0	0	1
0	0	1	0	0	1
1	0	1	0	0	1
1	0	0	0	0	0
1	0	0	0	0	0
0	0	0	0	0	1
0	0	0	0	0	1
1	0	0	0	0	0
1	0	0	0	0	0
0	0	0	0	0	0
0	0	0	0	0	1
1	0	0	0	0	1
0	0	0	0	0	1
0	0	0	0	0	0
1	0	0	0	0	0
0	0	1	0	0	1
1	0	0	0	0	0
0	0	0	0	0	0

1	0	0	0	0	0
1	0	0	0	0	0
0	0	0	0	0	0
0	0	0	0	0	0
1	1	0	0	0	1
0	1	0	0	0	1
1	1	0	0	0	1
0	1	0	0	0	1
0	1	0	0	0	1
0	0	0	0	0	1
1	1	0	0	0	1
1	1	0	0	0	1
1	0	1	0	0	0
0	0	0	0	0	0
0	0	0	0	0	0
0	0	0	0	0	0
0	0	1	0	0	1
0	0	0	0	0	0
0	0	0	0	0	1
0	0	0	0	0	0
0	0	0	0	0	1
0	0	0	0	0	0
1	0	0	0	0	1
0	0	0	0	0	0
0	0	0	0	0	0
1	0	0	0	0	1
1	0	0	0	0	0
0	0	0	0	0	0
1	0	0	0	0	0
0	0	0	0	0	0
0	0	0	0	0	0
1	0	1	0	0	1
1	0	0	0	0	0
1	0	0	0	0	0
1	0	0	0	0	0
1	0	0	0	0	0
1	0	0	0	0	0
1	0	0	0	0	0
1	0	0	0	0	0
1	0	0	0	0	0
1	0	0	0	0	1
0	0	0	0	0	0
1	0	0	0	0	1
0	0	0	0	0	0
0	0	0	0	0	1
0	0	0	0	0	1
1	1	0	0	0	1
1	0	0	0	0	1
1	0	0	0	0	0
0	0	0	0	0	0
1	0	0	0	0	0
0	0	1	0	0	1
0	0	0	0	0	0
0	0	0	0	0	0
1	0	0	0	0	0
1	1	0	0	0	1
0	0	1	0	0	1

1	0	1	0	0	1
1	0	0	0	0	1
1	0	1	0	0	1
0	1	0	0	0	1
0	0	0	0	0	0
0	0	0	0	0	0
0	0	0	0	0	1
0	0	1	0	0	0
0	0	0	0	0	0
0	0	0	0	0	1
0	0	0	0	0	1
0	1	0	0	0	1
0	0	0	0	0	1
0	0	1	0	0	1
0	1	0	0	0	1
0	0	0	0	0	1
0	0	0	0	0	1
1	0	0	0	0	1
1	1	0	0	0	1
1	1	0	0	0	1
0	0	0	0	0	1
0	0	1	0	0	1
0	0	0	0	0	0
0	0	0	0	0	0
0	0	0	0	0	0
0	0	0	0	0	0
0	0	0	0	0	1
0	0	1	0	0	0
0	0	0	0	0	1
0	0	0	0	0	0
0	0	0	0	0	1
0	0	0	0	0	0
1	0	0	0	0	1
0	0	1	0	0	0
0	0	0	0	0	1
0	0	0	0	0	1
0	0	0	0	0	0
0	0	0	0	0	0
0	0	0	0	0	1
0	0	1	0	0	0
0	0	0	0	0	0
0	0	0	0	0	0
1	0	0	0	0	0
1	0	1	0	0	0
0	0	0	0	0	0
0	0	0	0	0	1
0	0	0	0	0	1
0	0	0	0	0	0
0	0	0	0	0	0
0	0	0	0	0	0
1	0	0	0	0	0
0	0	0	0	0	0
0	0	0	0	0	0
1	0	1	0	0	1
0	0	0	0	0	0
1	0	0	0	0	1
1	0	0	0	0	1

0	0	0	0	0	0
0	0	0	0	0	1
0	0	0	0	0	0
0	0	0	0	0	0
0	0	0	0	0	0
0	0	0	0	0	0
0	0	0	0	0	1
1	0	0	0	0	0
0	0	0	0	0	1
1	0	0	0	0	0
1	1	0	0	0	1
0	0	0	0	0	1
0	0	0	0	0	0
1	0	0	0	0	0
1	0	0	0	0	1
0	0	0	0	0	1
1	1	0	0	0	1
1	0	1	0	0	1
1	0	0	0	0	1
1	0	0	0	0	1
0	1	0	0	0	1
0	0	0	0	0	1
0	1	0	0	0	1
0	0	0	0	0	1
0	0	0	0	0	0
0	0	0	0	0	0
0	0	0	0	0	0
0	0	0	0	0	0
1	0	0	0	0	0
0	0	0	0	0	1
0	0	0	0	0	0
0	0	0	0	0	0
0	0	0	0	0	0
0	0	0	0	0	1
0	0	0	0	0	0
0	0	0	0	0	0
0	0	0	0	0	1
1	0	0	0	0	0
0	0	0	0	0	0
1	0	0	0	0	1
1	0	0	0	0	0
0	0	0	0	0	0
0	0	0	0	0	0
1	0	0	0	0	0
0	0	0	0	0	0
0	0	0	0	0	0
0	0	0	0	0	0
0	0	0	0	0	0
1	0	0	0	0	0
1	0	0	0	0	0
0	0	0	0	0	1
0	0	0	0	0	0
0	0	0	0	0	1
1	1	0	0	0	1
1	1	0	0	0	1
0	1	0	0	0	1
0	0	0	0	0	0

1	0	0	0	0	0
0	0	0	0	0	0
0	0	0	0	0	0
0	0	0	0	0	0
1	0	0	0	0	0
1	0	0	0	0	1
0	0	0	0	0	0
0	0	0	0	0	0
0	0	0	0	0	0
0	0	0	0	0	0
1	0	1	0	0	1
0	0	0	0	0	1
0	0	0	0	0	1
0	0	1	0	0	1
0	1	0	0	0	1
1	1	0	0	0	1
0	1	0	0	0	1
0	0	0	0	0	0
0	0	0	0	0	0
0	0	0	0	0	0
0	0	0	0	0	1
0	0	0	0	0	0
0	0	0	0	0	0
1	0	0	0	0	1
0	0	0	0	0	0
0	0	0	0	0	0
0	0	0	0	0	0
0	0	0	0	0	0
0	0	0	0	0	0
0	0	0	0	0	0
0	0	0	0	0	0
0	0	0	0	0	0
0	0	0	0	0	0
0	0	0	0	0	0
0	0	0	0	0	0
0	0	0	0	0	0
1	0	0	0	0	0
0	0	0	0	0	1
0	0	0	0	0	1
0	0	0	0	0	0
0	0	0	0	0	1
0	0	0	0	0	0
0	0	0	0	0	1
0	0	0	0	0	0
1	0	0	0	0	0
1	0	0	0	0	0
1	0	0	0	0	0
0	0	0	0	0	0
0	1	0	0	0	1
1	0	0	0	0	1
0	0	0	0	0	0
0	0	0	0	0	1
0	0	0	0	0	1
1	0	1	0	0	1
0	0	0	0	0	0
0	0	0	0	0	0
0	0	0	0	0	1

0	0	0	0	0	0
0	0	0	0	0	0
0	0	0	0	0	0
0	0	0	0	0	0
1	1	0	0	0	1
1	0	1	0	0	1
1	1	0	0	0	1
0	0	0	0	0	0
0	0	0	0	0	0
0	0	0	0	0	1
0	0	0	0	0	1
0	0	0	0	0	1
0	0	0	0	0	0
0	0	0	0	0	0
0	0	0	0	0	0
0	0	0	0	0	0
0	0	0	0	0	0
1	1	0	0	0	1
0	0	0	0	0	0
1	0	1	0	0	1
0	0	0	0	0	1
0	0	0	0	0	0
0	0	0	0	0	0
0	0	0	0	0	0
0	0	0	0	0	0
0	0	1	0	0	0
0	0	0	0	0	1
0	0	0	0	0	0
1	0	0	0	0	0
1	0	0	0	0	0
0	0	0	0	0	0
0	1	0	0	0	1
0	1	0	0	0	0
0	0	0	0	0	0
0	0	0	0	0	0
0	0	0	0	0	0
1	0	0	0	0	0
1	0	0	0	0	0
0	1	0	0	0	1
1	0	0	0	0	1
0	0	1	0	0	1
0	0	1	0	0	1
0	0	0	0	0	0
0	0	0	0	0	0
0	0	1	0	0	1
0	1	0	0	0	1
0	0	0	0	0	0
0	0	0	0	0	0
0	0	0	0	0	0
1	0	0	0	0	0
0	0	0	0	0	0
1	0	0	0	0	0
1	0	0	0	0	0
1	0	0	0	0	0
1	0	0	0	0	0
0	0	0	0	0	1
0	0	0	0	0	1

0	0	0	0	0	0
1	0	0	0	0	0
1	0	0	0	0	1
1	0	0	0	0	1
1	0	0	0	0	0
0	0	0	0	0	0
0	0	0	0	0	1
0	0	0	0	0	0
0	0	0	0	0	0
0	0	0	0	0	0
1	0	0	0	0	0
1	0	0	0	0	0
0	0	0	0	0	0
1	0	0	0	0	0
1	0	0	0	0	0
0	0	0	0	0	0
1	0	0	0	0	0
0	0	0	0	0	0
1	0	0	0	0	0
0	0	0	0	0	0
0	0	0	0	0	0
1	0	0	0	0	0
1	0	0	0	0	0
0	0	0	0	0	0
0	0	0	0	0	0
1	0	0	0	0	1
1	0	0	0	0	0
0	0	0	0	0	0
1	0	0	0	0	0
0	0	0	0	0	0
1	0	0	0	0	0
0	0	0	0	0	0
0	0	0	0	0	0
0	0	0	0	0	0
0	0	0	0	0	0
0	0	0	0	0	0
0	0	0	0	0	0
0	0	0	0	0	0
1	0	0	0	0	1
0	0	0	0	0	0
0	0	0	0	0	1
0	0	0	0	0	1
0	0	0	0	0	0
1	0	1	0	0	1
1	0	0	0	0	1
1	0	1	0	0	0
1	0	0	0	0	0
1	0	0	0	0	1
0	1	0	0	0	1
0	0	0	0	0	0
0	0	0	0	0	0
0	0	0	0	0	0
1	0	0	0	0	0
0	0	0	0	0	1
0	0	0	0	0	0
0	0	0	0	0	1
1	0	0	0	0	0

0	0	0	0	0	0
0	0	0	0	0	0
0	0	0	0	0	0
0	0	0	0	0	0
0	0	0	0	0	1
0	0	0	0	0	1
0	0	0	0	0	0
1	0	0	0	0	0
0	0	0	0	0	0
0	0	0	0	0	0
0	0	0	0	0	0
0	0	0	0	0	0
0	0	0	0	0	0
0	0	0	0	0	0
0	0	0	0	0	0
0	0	0	0	0	0
1	0	0	0	0	1
1	0	0	0	0	0
0	0	0	0	0	0
0	0	0	0	0	0
1	0	0	0	0	1
1	0	0	0	0	0
0	0	0	0	0	1
1	0	0	0	0	0
0	0	0	0	0	0
1	0	0	0	0	1
0	0	0	0	0	0
0	0	0	0	0	0
1	0	0	0	0	1
0	0	0	0	0	0
0	0	0	0	0	1
0	0	0	0	0	0
1	0	0	0	0	1
0	0	0	0	0	0
0	0	0	0	0	0
0	0	0	0	0	0
0	0	0	0	0	1
0	0	0	0	0	0
0	0	0	0	0	1
0	0	0	0	0	0
0	0	0	0	0	0
0	0	0	0	0	0
0	0	0	0	0	0
0	0	0	0	0	0
0	0	0	0	0	1
0	0	0	0	0	0
0	0	0	0	0	0
1	0	0	0	0	0
1	0	1	0	0	1
1	0	1	0	0	1
1	0	0	0	0	0
0	0	0	0	0	0
0	0	0	0	0	0
0	0	0	0	0	1
0	0	0	0	0	0
0	0	0	0	0	0
1	0	0	0	0	0

1	0	0	0	0	0
0	0	0	0	0	1
0	0	0	0	0	0
0	0	0	0	0	0
0	0	0	0	0	0
1	0	0	0	0	0
0	0	0	0	0	0
0	0	0	0	0	0
0	0	0	0	0	1
0	0	0	0	0	0
0	0	0	0	0	0
0	0	0	0	0	0
0	0	0	0	0	0
0	0	0	0	0	0
0	0	0	0	0	0
0	0	0	0	0	0
0	0	0	0	0	0
0	0	0	0	0	0
1	1	0	0	0	1
0	0	0	0	0	0
0	0	0	0	0	0
0	0	0	0	0	0
0	0	0	0	0	0
0	0	0	0	0	0
0	0	0	0	0	0
0	0	1	0	0	1
0	0	0	0	0	0
0	0	0	0	0	1
0	0	0	0	0	0
0	0	0	0	0	1
1	0	0	0	0	0
1	0	1	0	0	0
1	0	0	0	0	0
1	0	1	0	0	0
0	0	0	0	0	0
0	0	0	0	0	0
0	1	0	0	0	0
0	0	0	0	0	1
0	0	1	0	0	1
1	0	0	0	0	0
0	0	0	0	0	0
0	0	0	0	0	0
0	0	0	0	0	0
0	0	0	0	0	1
0	0	0	0	0	0
1	0	0	0	0	1
1	0	0	0	0	1
0	0	0	0	0	1
1	1	0	0	0	1
0	0	0	0	0	0
1	0	0	0	0	0
1	1	0	0	0	0
0	0	0	0	0	1
0	0	0	0	0	1
0	0	0	0	0	1
0	0	0	0	0	0
1	0	0	0	0	1

0	0	0	0	0	0
0	0	0	0	0	0
0	0	0	0	0	1
1	0	0	0	0	0
1	0	0	0	0	0
0	0	0	0	0	0
0	0	0	0	0	0
1	0	0	0	0	0
0	0	0	0	0	0
0	0	0	0	0	1
0	0	0	0	0	1
0	0	0	0	0	0
0	0	0	0	0	0
0	0	1	0	0	1
0	0	0	0	0	1
1	0	1	0	0	1
0	0	1	0	0	1
0	0	0	0	0	1
0	0	1	0	0	1
1	0	0	0	0	0
0	0	0	0	0	0
0	0	0	0	0	0
1	0	1	0	0	0
0	1	0	0	0	1
1	1	0	0	0	1
0	0	1	0	0	1
1	0	0	0	0	1
0	0	0	0	0	0
0	0	0	0	0	0
0	0	0	0	0	0
0	0	0	0	0	0
0	0	0	0	0	0
0	0	0	0	0	0
0	0	0	0	0	0
0	0	0	0	0	0
0	0	0	0	0	0
0	0	0	0	0	0
1	0	1	0	0	1
0	0	0	0	0	0
0	1	0	0	0	0
0	0	0	0	0	1
0	0	0	0	0	0
0	0	0	0	0	1
0	0	0	0	0	1
0	0	0	0	0	0
0	0	0	0	0	0
1	0	1	0	0	1
0	0	0	0	0	0
0	0	0	0	0	0
1	0	0	0	0	0
0	1	0	0	0	1
1	0	0	0	0	0
0	0	0	0	0	1
0	0	0	0	0	0
0	0	0	0	0	0
0	0	0	0	0	0
0	0	0	0	0	0

0	0	0	0	0	0
0	0	0	0	0	0
0	0	0	0	0	0
0	0	0	0	0	0
0	0	0	0	0	0
0	0	0	0	0	0
1	0	0	0	0	0
0	0	0	0	0	0
0	0	0	0	0	0
0	0	0	0	0	0
0	0	0	0	0	0
0	0	0	0	0	0
1	0	0	0	0	0
1	0	0	0	0	0
1	0	0	0	0	0
0	0	0	0	0	0
0	0	0	0	0	0
0	0	0	0	0	0
1	0	0	0	0	0
0	0	0	0	0	0
1	0	0	0	0	0
1	0	0	0	0	0
1	1	0	0	0	1
0	1	0	0	0	1
0	0	0	0	0	0
1	0	0	0	0	0
1	0	0	0	0	0
0	0	0	0	0	1
0	0	0	0	0	1
1	0	0	0	0	0
0	0	0	0	0	0
1	0	0	0	0	0
0	0	0	0	0	0
0	0	0	0	0	0
0	0	0	0	0	0
1	0	0	0	0	0
0	0	0	0	0	0
1	0	0	0	0	0
1	0	1	0	0	1
1	1	0	0	0	1
0	1	0	0	0	1
0	0	0	0	0	0
1	0	0	0	0	0
0	0	0	0	0	0
0	0	0	0	0	0
0	0	0	0	0	0
0	0	0	0	0	0
0	0	0	0	0	0
0	0	0	0	0	0
0	0	0	0	0	0
0	0	0	0	0	0
1	0	0	0	0	0
0	0	0	0	0	0
0	0	0	0	0	0
0	0	0	0	0	0
0	0	0	0	0	0
1	0	0	0	0	1

1	0	0	0	0	1
1	0	1	0	0	1
1	0	0	0	0	0
1	0	0	0	0	0
1	0	0	0	0	0
1	0	0	0	0	0
1	0	0	0	0	0
1	0	0	0	0	0
1	0	0	0	0	0
1	0	0	0	0	0
1	0	0	0	0	0
1	0	0	0	0	0
1	0	0	0	0	0
1	0	0	0	0	0
1	0	0	0	0	0
1	0	0	0	0	0
1	0	0	0	0	0
1	0	0	0	0	0
0	0	1	0	0	1
0	0	0	0	0	0
1	0	0	0	0	1
0	0	0	0	0	1
1	0	0	0	0	1
0	0	0	0	0	1
1	0	0	0	0	1
1	0	0	0	0	0
0	0	0	0	0	1
1	0	0	0	0	1
1	0	0	0	0	1
1	0	0	0	0	1
1	0	0	0	0	1
1	0	0	0	0	1
1	0	0	0	0	1
1	0	0	0	0	1
0	0	0	0	0	1
1	0	0	0	0	1
1	0	0	0	0	1
1	0	0	0	0	0
0	0	0	0	0	0
1	0	0	0	0	1
1	0	0	0	0	1
1	0	0	0	0	1
1	0	0	0	0	1
1	0	0	0	0	1
1	0	0	0	0	1
1	0	0	0	0	1
0	0	0	0	0	1
0	0	0	0	0	1
1	0	0	0	0	1
1	0	0	0	0	1
0	0	0	0	0	1
0	0	0	0	0	0
0	0	0	0	0	0
1	0	0	0	0	1
1	0	0	0	0	1
0	0	0	0	0	0
0	0	0	0	0	0

0	0	0	0	0		0
0	0	0	0	0		0
1	0	0	0	0		1
0	0	0	0	0		0
0	0	0	0	0		0
0	0	0	0	0		0
0	0	0	0	0		0
1	0	0	0	0		0
0	0	0	0	0		0
1	0	0	0	0		0
1	0	0	0	0		1
0	0	0	0	0		0
0	0	0	0	0		0
1	0	0	0	0		0
1	0	0	0	0		0
1	0	0	0	0		0
0	0	0	0	0		0
1	0	0	0	0		0
0	0	0	0	0		0
0	0	0	0	0		1
1	0	0	0	0		0
0	0	0	0	0		0
1	0	0	0	0		0
1	0	0	0	0		0
0	0	0	0	0		0
1	0	0	0	0		0
0	0	0	0	0		1
0	0	1	0	0		1
1	0	0	0	0		0
0	0	0	0	0		0
0	0	0	0	0		0
0	0	0	0	0		0
0	0	0	0	0		0
0	0	0	0	0		0
0	0	0	0	0		0
1	0	0	0	0		0
1	0	0	0	0		0
1	0	0	0	0		0
0	0	0	0	0		0
0	0	0	0	0		0
0	0	0	0	0		0
0	0	0	0	0		0
0	0	0	0	0		0
1	0	0	0	0		0
1	0	0	0	0		0
0	0	0	0	0		0
0	0	0	0	0		0
0	0	0	0	0		0
0	0	0	0	0		1
0	0	0	0	0		0
0	0	0	0	0		0
0	0	0	0	0		0
0	0	0	0	0		1
0	0	0	0	0		0
0	0	0	0	0		0
0	0	0	0	0		0
0	0	0	0	0		0

0	0	0	0	0	0
0	0	0	0	0	1
0	0	0	0	0	0
1	0	0	0	0	0
0	0	0	0	0	0
0	0	0	0	0	0
1	0	0	0	0	0
1	0	0	0	0	1
0	0	1	0	0	1
0	0	0	0	0	1
0	0	0	0	0	0
0	0	0	0	0	0
1	0	0	0	0	0
1	0	0	0	0	1
1	0	0	0	0	1
1	0	0	0	0	1
0	0	0	0	0	0
0	0	0	0	0	0
0	0	0	0	0	0
1	0	0	0	0	0
0	0	0	0	0	1
0	0	1	0	0	0
0	0	0	0	0	0
0	0	0	0	0	0
1	0	0	0	0	1
1	0	1	0	0	0
0	0	0	0	0	0
0	0	0	0	0	0
0	0	0	0	0	1
0	0	0	0	0	0
0	0	0	0	0	0
0	0	0	0	0	0
0	0	0	0	0	0
0	0	0	0	0	1
0	0	0	0	0	0
0	0	0	0	0	1
1	0	0	0	0	0
0	0	0	0	0	0
0	0	0	0	0	0
0	0	0	0	0	0
1	1	0	0	0	1
0	0	0	0	0	0
1	1	0	0	0	1
0	1	0	0	0	1
1	0	0	0	0	0
1	0	0	0	0	0
0	0	0	0	0	1
0	0	0	0	0	0
0	0	0	0	0	0
0	0	0	0	0	1
0	0	0	0	0	0
1	0	0	0	0	0
0	0	0	0	0	0
0	0	0	0	0	0
0	0	0	0	0	0
1	0	1	0	0	0
1	0	0	0	0	1

0	0	0	0	0	1
0	0	0	0	0	0
0	0	0	0	0	0
1	0	0	0	0	1
0	0	0	0	0	0
0	0	0	0	0	0
0	0	0	0	0	0
0	0	0	0	0	0
1	0	0	0	0	0
0	0	0	0	0	0
1	0	0	0	0	0
1	0	1	0	0	1
0	0	0	0	0	0

SYNCYTIAL_EXP	EMBRYONIC_01_COUNTS	EMBRYONIC_02_COUNTS
0	4	11

1	5125	1173
1	8368	7851
1	5106	6795
0	1196	1498
1	314	3961
0	14	39
0	0	0
1	4996	79464
0	6	21
0	46	213
1	3372	4511
1	6578	2025
0	70	1241
1	4494	8136
1	1951	9011
1	8539	6475
0	86	442
1	1285	17671
1	3559	6783
1	9673	11085
1	25495	15389
0	3	23
1	727	588
0	0	0
1	295	338
0	0	1
0	1	1
0	0	0
0	0	0
0	0	0
1	4654	4084
0	0	0
1	5037	4343
1	24003	22002
1	243	3148
0	138	94
1	6175	7939
0	19	53
1	2012	1037
0	86	231
1	8071	4649
1	12203	999
0	3	1
1	3519	1631
0	99	178
0	0	0
0	63	191
0	0	0
1	3195	4211
0	6	1
0	0	0
0	39	64
0	2	0
0	15	63
1	0	4
0	17	12
0	5	17

0	2	3
0	9	102
1	20854	5698
0	0	0
1	19471	30392
0	0	0
0	68	264
1	3442	2133
1	4145	1022
0	258	199
1	9782	14696
1	2920	4425
1	1394	653
1	10318	16840
1	3804	3029
1	48953	85539
0	3	6
1	2581	914
1	1399	11264
0	23	189
0	0	2
1	48512	3317
1	143	1841
1	2325	1765
1	8064	4367
0	5	36
1	7515	2254
0	0	1
1	97886	156458
1	13393	20355
1	16	203
0	25	23
1	5901	1648
1	14731	7874
1	1406	2144
1	7220	9785
1	9089	7636
1	4872	7269
0	80	78
1	3378	2329
1	2557	366
1	2090	2165
1	3580	3717
1	13844	22337
1	30172	30939
0	11	4
0	6	13
1	982	1365
1	1400	1177
1	974	508
1	12794	13891
1	65	184
1	3380	4721
0	28	71
1	4702	2910
1	22645	16025
1	86	1711

1	8791	13367
1	628	1909
0	7	21
1	8762	10353
1	1622	1659
1	12118	22363
1	3289	998
1	1713	566
1	6403	4455
0	0	1
1	13249	7718
1	6354	3713
0	10	64
1	6064	8590
1	1966	1432
1	4727	6488
1	811	1341
1	2232	1006
0	37	67
1	11969	20135
0	0	0
0	0	0
1	289	4121
0	0	3
0	14	89
1	4471	1149
1	7819	8030
0	36	36
1	2689	851
0	171	803
0	131	226
1	1042	2070
1	21122	38557
1	7253	607
0	0	0
0	61	62
0	2	6
0	105	111
1	6672	4955
1	22396	29032
1	20507	27031
1	1627	329
1	3000	2571
1	2078	1967
0	73	252
0	33	55
1	3712	1687
0	2	7
1	42168	34086
0	147	332
1	5914	12804
0	293	161
0	174	1876
0	0	5
0	65	566
1	1607	1392
0	0	0

0	6	19
1	96	1338
1	12833	7027
0	2	1
1	11545	13197
1	2956	2713
1	5911	1717
1	11061	2361
0	0	2
1	1390	1432
1	4582	1000
1	4322	1096
1	8937	3199
1	2368	1590
1	3937	4608
1	456	175
1	3185	1463
0	149	85
1	29617	18287
1	1483	1777
1	4125	1226
1	1972	2096
1	230766	98873
1	2445	2232
0	7	42
0	25	10
1	18757	27032
0	20	165
1	5816	2640
1	12551	18214
0	0	0
0	0	0
0	0	0
1	4601	3931
0	1	6
0	4	14
0	5	10
0	173	193
1	23477	5109
1	3438	1839
1	8125	13466
0	0	0
0	0	0
0	0	0
0	1	0
0	1	2
1	3841	1091
1	10483	5323
0	0	1
1	8515	1556
1	3166	2815
1	6063	2421
1	14319	17312
0	21	101
0	21	140
0	0	3
1	8276	12598

1	9586	11308
1	7558	6565
1	2541	937
0	0	5
0	0	0
0	8	62
1	1492	2077
1	26111	27246
1	13181	7769
0	22	23
1	0	0
0	0	0
1	1047	2093
1	8613	9591
0	3	4
0	7	5
0	0	0
1	1057	1134
1	10292	4025
1	569	488
0	0	0
1	10719	9447
0	0	0
1	7147	276
0	661	415
1	2828	3729
1	2124	1095
0	9	58
0	190	146
1	22056	15864
1	11716	11859
1	14458	866
1	2046	1088
0	1	2
1	3325	4619
0	4	56
1	1381	1401
1	9955	10865
1	12647	1841
1	9084	5511
1	4426	1283
1	5211	5813
1	12432	12754
0	0	1
1	5853	5714
1	26043	39639
0	8	31
0	0	1
1	7199	9765
1	1452	2307
1	5391	6608
0	2	8
0	8	8
1	61	793
1	193	878
1	14435	10136
1	1272	2845

0	0	0
0	14	21
0	930	2676
0	166	185
1	12445	15296
1	273	331
1	12025	9732
0	36	64
0	1265	118
0	0	2
0	83	215
1	2277	2195
0	0	0
1	10370	14743
1	1464	935
1	5217	3716
1	123	439
1	11091	1389
1	19368	765
0	22	5
1	3799	3476
0	14	9
1	621	597
0	104	131
0	18	12
0	0	0
1	1216	791
1	7150	7151
1	6731	724
1	3129	3669
0	1	1
0	68	490
0	3	2
0	20	46
0	0	0
1	13096	5661
0	32	204
1	31410	16706
1	14769	3560
0	0	30
0	0	3
0	5	77
0	6	3
0	48	16
1	1982	1001
0	25	69
1	2151	1892
1	4013	3063
0	1	10
1	1278	745
1	6778	2999
1	12895	6250
1	3079	292
0	0	3
1	133566	185086
1	6719	3071
1	725	272

0	1	1
1	13654	3732
1	12666	17340
0	0	0
0	1	9
0	94	827
0	25	126
0	1276	1147
0	3	2
0	0	7
0	17	182
0	2	33
1	2600	1304
1	583	16864
0	5489	230
1	4828	6258
0	22	33
1	11566	13651
0	35	175
0	10	5
0	0	4
0	0	0
0	21	42
0	0	0
1	25938	38325
1	4992	8667
0	0	0
0	45	46
0	0	7
1	1051	530
1	166	554
0	23	45
0	15	28
1	2596	425
1	19618	27604
1	19654	7351
1	2667	4807
0	0	0
0	0	0
0	6	19
1	10399	7694
1	8538	728
0	0	0
0	0	0
0	7	17
0	13	38
0	85	119
1	5741	1416
0	1	2
0	20	17
0	0	0
0	1	0
1	3766	1794
1	34	73
1	2556	499
0	0	0
1	3236	899

0	2	1
0	1275	765
1	111576	159119
1	51504	60998
0	320	393
0	14	16
0	190	92
0	4	2
1	28	710
0	63	19
1	1761	1750
1	6307	12696
1	1835	1341
0	0	0
1	29508	29237
0	300	163
0	2	8
1	2783	966
1	55428	111548
0	0	0
1	2287	3007
0	0	5
1	2245	1048
0	0	0
0	8	48
1	1661	1283
0	2	23
1	792	13511
0	10	8
1	3019	1442
0	2	11
0	98	1164
0	0	4
1	2428	89
0	998	1324
1	5960	4772
1	9329	4528
0	4	3
0	1	11
1	25155	7363
1	4640	6883
0	12	21
1	3607	6681
0	141	45
0	0	11
1	7362	5844
0	0	7
1	1672	553
0	3	1
1	9232	1862
0	4	7
0	0	2
0	0	2
0	0	0
1	3128	615
0	0	0
1	1937	1067

1	9113	8224
0	0	1
1	2837	418
1	1549	1820
1	6536	676
1	6338	4014
1	1619	841
1	2752	1792
1	42	238
1	7356	4927
0	0	1
0	22	150
1	10374	15567
0	9	7
0	0	0
0	0	0
1	12486	5745
1	1666	1637
1	9992	11295
1	45266	16732
0	0	0
0	0	1
1	8303	6992
1	55061	27072
1	4553	411
1	3697	1710
0	29	25
0	2	7
0	8	120
1	17	94
0	2541	3837
0	8	5
1	11626	13547
1	8666	10221
1	10759	5338
1	3350	3329
1	3897	6542
1	7206	7977
1	11789	23570
1	4556	2054
1	27240	35871
0	177	762
1	6	175
1	5005	5053
1	1984	1764
1	1923	783
1	4523	736
1	9895	8236
0	1	13
1	104	1848
1	156	2840
1	11923	11366
0	10	17
0	2	4
1	20436	31473
1	45268	6136
1	983	504

0	2	14
0	13	22
1	51769	67465
1	11392	5950
0	7	20
1	7773	12118
1	12561	7742
1	3764	3160
1	11094	4300
1	2486	1169
1	5982	5131
1	63611	5416
0	13	75
1	1095	750
1	26864	24990
1	2922	2452
1	9641	6277
1	6656	3757
0	1	9
1	3704	2490
1	1248	317
0	0	6
0	4	14
1	10254	13369
1	16332	6996
1	4687	3720
1	4991	2093
1	14400	3818
0	1	54
1	2576	2552
1	5565	8583
0	0	0
1	3831	1312
1	8529	1167
1	16469	13358
0	0	1
0	99	39
1	24973	29266
0	89	241
1	1165	4589
0	0	1
1	3908	1064
0	7	40
0	0	1
1	4	85
1	3204	2347
1	2553	26983
0	10	19
1	1174	2742
1	10366	6673
1	21721	5003
1	5998	3478
1	1050	1211
1	40699	68819
0	0	0
0	186	32
1	30616	15614

1	3358	2687
1	8924	1918
1	12136	8162
1	63018	45553
1	5342	1898
1	17	85
1	13815	14518
1	90	1066
1	2937	50874
1	7518	1585
0	2	0
1	12113	12710
1	21	53
1	4247	7357
1	270	3940
0	206	558
1	1983	1016
1	9407	11089
0	0	0
1	48153	50861
0	1	3
0	31	109
0	3	1
1	12243	3479
0	30	73
1	28044	31732
1	4257	6615
1	4977	3403
0	0	3
1	3914	984
0	0	9
0	231	174
1	11993	8976
1	1666	1394
1	3324	3105
0	5	72
1	748	524
0	0	0
1	5031	3953
1	19884	23029
1	4876	3735
0	0	0
1	9553	13385
1	5832	6060
1	1181	1432
1	9513	53309
1	2222	3216
0	1	23
1	14287	10405
1	14585	25644
0	3	14
1	17324	17967
1	2369	2923
1	349	735
1	3797	1107
1	596	1898
1	10046	10470

0	0	4
1	638	7104
0	0	0
0	20	7
0	1	1
0	0	0
0	0	0
1	235	3976
1	1568	1702
0	22	203
1	14104	6547
0	0	0
0	0	0
1	6	7
0	7	5
0	0	2
0	1	2
0	0	0
1	1230	245
1	21686	17624
0	5	1
0	0	0
1	3073	2839
1	18601	16860
1	914	12453
0	0	1
1	1302	414
0	26	14
0	74	75
0	0	0
0	0	0
0	0	0
0	0	2
1	159662	225896
0	19	131
0	0	1
0	30	73
0	0	1
1	222	3376
1	2002	2298
0	2	1
0	14	0
0	0	0
1	7175	6332
0	0	2
0	0	2
1	20464	35401
0	0	2
0	9	26
0	0	2
1	7816	8454
1	14535	8227
1	822	564
1	2712	2676
0	0	0
0	67	559
1	69	842

1	3049	3453
1	22571	3623
1	11198	20207
1	4254	6372
1	12383	10766
0	0	2
0	17	60
1	562	373
0	737	309
1	4837	1167
1	14290	15360
1	52306	84353
0	20	17
0	3	2
0	0	0
0	0	0
0	4	23
1	6761	5632
0	0	0
0	1	1
1	17674	3538
0	9	7
1	5	45
0	1	0
0	4	3
0	13	5
0	14	19
0	27	39
0	0	12
0	2	5
0	0	0
0	6	16
1	11749	10738
1	16116	5008
0	0	0
0	5	39
0	0	0
0	0	0
0	1	7
0	352	786
0	0	0
1	1238	534
1	1776	857
1	7597	1590
0	0	0
1	4384	1456
0	12	150
0	0	4
0	12	97
1	2045	2189
0	11	15
0	18	151
1	176	137
0	0	0
0	0	1
0	0	2
0	0	0

0	4	76
0	1	41
1	1556	726
0	8	1
0	17	7
0	86	37
0	0	0
0	2	1
0	12	29
0	0	2
1	334	4625
0	0	3
0	0	0
0	36	83
1	4014	3958
1	4806	1747
1	3621	3121
0	54	71
1	7031	8700
1	1693	2529
1	2770	878
0	0	0
1	6036	11429
0	0	0
0	0	0
0	0	0
0	1	9
1	30	373
1	780	873
0	0	6
0	0	0
0	1	0
1	11546	3848
0	0	1
0	0	0
0	0	0
0	3	11
0	0	0
0	53	65
0	0	0
0	0	0
0	9	17
1	2534	539
0	0	0
0	2	2
0	482	206
0	0	0
1	5666	2846
0	6	6
1	3954	3283
1	591	440
0	0	2
0	0	5
0	0	0
0	7	22
0	0	0
0	0	8

0	0	9
0	5	28
1	15	130
0	1	5
0	1	72
0	95	87
0	65	213
0	3	2
1	26376	7275
0	17	20
0	13	14
0	18	140
1	795	675
0	4	48
0	30	19
1	5335	8330
0	0	2
0	32	14
0	10	48
0	1	0
0	1	0
0	0	4
0	0	0
1	615	235
0	0	0
0	2	22
1	4431	7091
1	1110	1105
1	89	284
0	0	0
0	1	3
1	28840	42018
1	35631	64900
0	1	5
0	736	197
1	1606	955
1	5643	1258
1	3598	689
0	0	0
0	0	2
0	2	7
1	619	275
0	0	2
0	75	104
0	0	0
0	5	16
0	0	0
1	328	221
0	0	4
0	0	0
1	6304	6869
0	0	1
0	0	1
0	0	4
0	3	0
1	48	983
0	0	0

0	0	0
0	0	0
0	0	0
0	0	1
0	0	0
0	48	73
1	359	3589
0	0	4
0	0	0
1	5283	2887
1	3182	1482
0	0	10
0	29	14
0	0	4
1	11026	4064
1	10534	14230
0	0	0
0	3	2
0	1	37
0	0	0
1	5364	2896
0	0	0
0	0	7
0	9	16
0	0	0
1	862	1195
0	0	0
0	3	4
1	542	555
0	0	1
0	118	845
0	1	1
0	3	107
0	7	11
0	70	90
1	889	1456
1	4012	769
0	11	32
0	1	22
0	0	0
1	30	319
1	920	1316
0	0	1
1	2773	656
0	88	82
0	13	58
0	6	74
0	0	10
0	1	2
1	329	4059
1	2218	300
0	5	5
1	12	179
0	5	54
0	27	33
0	60	53
1	14403	10100

1	239	1836
1	331	983
1	388	1773
1	64	90
1	236962	21658
0	0	0
0	3	10
0	0	0
0	0	0
0	4	5
0	0	0
0	13	17
0	31	34
0	4	30
1	459	7745
1	9149	422
0	0	7
1	3423	317
0	21	4
1	491	426
1	454	5204
0	8	18
0	1	2
0	0	0
0	5	0
0	4	6
0	4	11
0	0	0
0	3209	2144
0	0	0
0	0	3
0	2	0
0	460	40
1	19243	19947
1	6238	2178
0	2	3
0	0	1
1	4266	1389
1	2146	1778
0	3	82
0	0	1
0	0	6
0	30	54
0	90	24
0	10	127
1	4251	783
1	88	841
1	3221	3461
1	32529	66495
1	11690	3188
0	55	259
1	2200	716
0	0	2
1	6979	1797
0	0	0
0	0	0
0	1	0

1	29316	14966
0	3	2
1	7646	948
1	1770	1063
0	2	23
1	464	1288
0	1	2
0	1	0
0	2	0
0	0	0
0	0	0
0	28	8
0	3	12
1	1061	1380
0	5	16
0	1	3
0	0	0
0	0	2
1	1373	1605
1	10557	6568
1	9821	12346
0	3	5
0	0	7
0	2	8
0	100	22
0	4	2
0	0	0
1	11332	5779
1	5735	4646
0	0	4
0	0	0
0	0	1
1	40	578
1	1029	17241
0	0	0
0	0	4
0	1	0
0	10	4
0	29	83
1	14	271
0	0	0
0	3	2
0	51	23
1	1199	1353
0	4	24
0	3	26
0	0	2
0	0	0
1	5999	2855
0	1	0
0	0	0
0	20	9
0	7	1
1	11670	16135
0	7	37
0	0	0
1	966	1145

1	5345	5563
1	233	484
1	17625	17863
1	4978	2293
1	24817	31582
1	4549	3386
0	2	38
1	4816	7073
1	282	370
1	33392	54636
0	8	27
0	0	1
0	0	2
0	375	184
0	2	2
0	12	9
0	1038	819
0	4	11
0	10	3
0	0	0
1	2499	3683
0	126	924
0	28	44
0	0	4
0	0	3
0	19	7
1	3338	2273
1	364	331
0	0	0
1	4101	45200
0	0	1
1	10180	14745
0	18	22
0	2	25
1	3018	1133
0	20	42
0	6	23
0	0	9
0	6	39
0	0	0
0	0	3
0	10	246
0	0	0
0	0	0
0	64	130
0	2	0
0	53	57
0	0	0
1	35	75
1	1992	1038
0	21	6
0	27	14
0	12	8
0	9	25
1	34771	18605
1	37019	32447
1	1930	35687

1	2381	686
0	9	10
0	0	1
1	11654	826
1	49	84
0	0	1
0	0	3
0	0	0
0	0	4
0	93	93
1	12686	5052
1	510	514
1	18562	3884
0	17	10
1	38260	3882
0	4	6
0	21	77
0	7	30
0	13	57
0	0	2
0	40	57
0	0	0
0	0	0
0	0	0
1	5233	105
1	6341	1977
0	6	10
0	1	0
0	3	2
0	0	0
0	7	34
1	913	400
0	6	45
1	6001	3352
0	0	3
0	0	4
0	8	15
0	32	21
0	376	173
0	172	287
0	42	50
0	120	45
0	4	3
0	0	1
1	3492	5488
0	1	3
1	1904	704
0	0	3
1	4295	527
0	0	1
0	3	1
0	15	103
0	3	1
0	0	0
0	0	3
0	60	572
1	14264	7316

1	2209	1756
0	0	0
0	0	2
0	0	0
0	1	2
0	791	95
1	102	1106
0	0	4
0	0	7
0	0	0
0	8	49
0	2	0
1	142188	182687
0	150	269
1	102	37
0	1	2
1	5009	5788
0	1	1
1	9045	3939
1	3733	36427
1	8542	9294
1	10467	10096
1	8583	9431
1	2351	1592
1	4882	4020
1	2011	1021
1	1136	656
0	5	0
0	194	57
1	16572	14300
0	0	0
1	14563	9195
0	0	0
0	0	3
1	0	0
1	26	111
0	2	5
0	18	68
1	8305	5393
0	0	0
0	52	527
0	11	15
0	1	0
0	15	12
0	15	21
1	24761	14360
1	26234	28136
0	0	0
0	9	26
0	1	102
1	1226	539
0	31	142
0	0	2
0	0	22
0	27	19
0	11	27
0	2	37

0	108	56
1	4438	1726
0	44	43
0	5	3
0	19	18
1	1357	1289
1	311	4420
1	654	10250
0	320	299
1	59008	81158
0	232	149
0	30	21
0	267	133
0	0	0
1	501	747
0	1	6
0	0	0
0	6	27
0	1	0
0	5	4
0	1	16
0	0	2
0	1	0
0	0	0
0	0	0
1	1064	430
0	134	128
1	149	3024
1	3426	3871
1	1557	1338
1	231	3245
0	14	31
1	2961	1943
1	5539	7909
0	0	0
0	47	28
1	9463	7139
0	0	0
0	0	0
1	0	2
1	33	411
1	43	703
1	6101	782
1	6473	7481
0	28	214
0	4	1
1	1320	8498
1	6684	4654
0	5	59
1	1552	1630
1	582	927
0	0	0
1	6411	2444
0	0	0
0	3	2
0	13	47
0	0	2

0	1	2
0	0	2
0	3	11
0	15	136
0	165	123
1	27894	50646
0	0	0
0	0	0
0	2	15
1	8734	21528
0	1	19
0	0	0
0	0	9
0	1	1
0	5	10
0	4	10
1	5962	2083
0	0	0
0	11	14
0	9	35
0	1	1
1	1003	943
0	0	0
0	0	0
0	0	2
1	774	303
0	0	1
0	0	0
0	11	8
0	0	10
0	1	45
1	894	7799
1	12	170
0	0	0
0	4	23
0	0	13
0	1	2
1	5504	1657
1	10837	6760
1	1699	4129
0	0	0
0	5	35
0	3	5
0	8	78
0	0	0
1	4720	8623
1	337	3847
1	1215	1413
0	13	113
1	19	122
0	0	0
0	0	0
0	0	2
0	23	33
0	5	11
0	0	0
0	1	1

1	1800	619
0	0	15
1	12354	3596
0	5	51
1	13626	15460
0	4	59
0	0	0
1	44242	54560
0	1	2
0	0	0
0	0	0
0	659	873
0	0	0
0	4	10
0	1	0
0	0	0
1	867	1259
0	2	6
1	1903	2082
0	0	0
0	9	26
0	1	1
0	12	8
1	10949	4158
0	0	0
0	97	41
0	1	0
0	3	1
0	0	0
0	0	0
0	22	28
1	11047	16754
0	0	0
0	15	6
1	20	154
1	983	19421
1	68	407
0	14	198
0	2	0
1	1705	604
1	58	250
1	3	180
0	15	24
1	9419	3023
1	9020	3047
0	11	9
0	2	19
0	1	1
0	11	9
1	12810	3259
0	5	15
1	18	190
1	39304	57761
0	124	258
1	1857	1807
1	4314	977
0	9	133

0	0	3
0	31	7
0	191	100
0	0	3
1	8784	5824
1	5600	8792
1	86460	56227
1	2622	1149
0	0	1
1	16013	22150
1	2221	2922
1	5758	784
0	0	0
0	0	0
0	22	71
0	14	17
1	31443	21149
1	17	42
0	0	0
1	7944	6284
1	9322	3102
0	2	8
1	5741	6745
0	12	23
1	370	600
0	1	3
1	3997	657
0	2	0
0	0	0
0	44	19
0	0	0
1	1288	569
0	9	26
0	0	0
0	0	0
0	0	0
0	0	1
1	270	237
0	0	0
0	3	0
1	17212	15194
1	204	1104
1	4312	3271
1	3486	932
0	27	44
1	10692	13294
0	0	4
1	49329	3602
1	1875	1370
1	1780	2930
1	12003	1321
1	422	5187
0	1284	37
1	4565	4856
0	54	72
0	13	126
1	6788	1087

0	0	5
0	1	0
1	109	90
1	8114	875
1	7074	595
0	5	15
1	4511	5300
1	902	2479
0	0	1
1	380	5348
0	0	0
0	0	0
1	26255	44243
0	2	3
1	1641	2195
0	18	53
0	0	9
0	8	29
0	0	0
0	6	89
1	1822	690
0	0	9
0	34	24
1	14378	11809
1	87	515
1	5150	7800
1	120	347
0	13	26
0	30	40
0	0	0
0	0	0
1	1096	1875
0	0	2
0	0	1
0	0	2
0	11	6
0	682	617
1	13999	12304
1	13	357
0	6	1
0	5	0
0	136	170
1	7348	7614
0	0	0
0	0	2
1	10233	11806
1	2371	1991
1	1412	616
0	0	12
1	27161	11252
1	5469	2424
1	7618	9082
1	112774	166841
0	16	52
0	0	0
0	0	0
1	5294	5875

0	0	0
0	0	2
1	10686	3387
1	23872	32436
0	15	19
1	278	405
0	12	48
1	6930	2551
0	0	0
0	0	12
0	0	0
1	3192	4719
0	0	0
1	653	1031
1	19087	24697
0	0	1
1	8741	6087
1	6967	8607
1	787	2877
1	10669	14949
0	15	12
0	0	0
1	19	352
0	23	95
0	514	756
0	3	6
0	5	80
0	0	0
0	0	0
1	729	647
1	8658	8962
1	6059	7712
0	2	0
1	4632	6957
1	9501	13689
1	31062	11069
0	0	0
0	2	0
0	2	0
0	0	7
1	4015	6224
0	0	0
0	0	0
0	0	2
1	328	2797
1	3659	1728
0	15	119
1	8502	8194
0	0	2
0	1	15
0	88	63
1	4240	5414
0	0	0
0	0	0
0	0	1
0	12	33
1	3888	1993

0	4	5
1	13858	10040
0	42	343
1	70845	83283
1	205	2853
1	2483	1537
0	0	0
1	4085	1432
1	2170	1268
0	2	10
1	9158	9403
1	34459	24415
0	0	14
1	3215	2685
0	94	563
1	4662	5596
0	11	154
1	15693	17587
0	301	416
0	3	8
0	13	16
1	2706	1023
1	9414	13002
1	6164	347
1	18344	15038
1	50	179
1	6215	8890
1	14040	6150
1	9618	12180
1	14187	16288
0	142	223
1	2394	3778
1	3603	7577
0	4	9
0	35	10
1	2623	595
1	11214	8053
0	1	4
1	1251	25466
0	0	1
1	10072	4077
0	16	54
0	11	9
1	4505	1735
1	1450	1990
1	237	321
0	0	0
1	14466	1058
0	0	1
1	1939	717
1	2647	2516
0	1055	195
0	0	0
0	222	76
1	4669	1380
1	7254	2547
1	4953	5412

0	0	0
1	4878	4519
1	5401	6640
0	1	56
1	6783	7242
1	3082	284
1	2862	780
1	10860	9663
1	1085	8891
0	0	0
0	15	17
0	1005	155
1	3923	5487
0	0	0
1	5716	2284
0	0	0
0	1	13
0	0	4
1	154	1723
0	0	0
0	95	10
1	2938	925
0	137	67
1	1147	738
1	1736	1927
0	1	1
0	2	3
1	3834	1332
1	1339	471
0	50	443
1	11431	8879
1	7199	5207
0	2	0
0	2	0
0	0	0
1	2182	287
1	21043	25933
0	2	2
1	2429	2197
0	0	0
1	7427	237
1	11938	18814
0	1	13
0	0	0
0	0	0
1	562	5315
0	9	43
1	10018	11950
0	41	392
0	0	0
0	0	0
0	188	1991
0	0	0
0	0	0
1	9954	16255
0	0	1
1	4445	67137

1	6311	7238
0	1	2
1	10069	14548
0	3	29
0	8	35
0	246	90
0	2	32
1	11312	9416
0	2	3
1	4770	6032
1	3288	2886
1	12281	16209
1	4437	5020
1	4631	5278
1	1822	1536
1	3245	4405
1	1915	720
0	20	0
0	10	16
1	320	254
1	123	1116
0	0	0
0	6	15
0	0	0
0	25	5
0	0	0
1	7726	11433
1	2422	396
1	7845	1278
0	1	0
1	9765	4569
0	0	0
0	466	587
1	4114	567
0	2	20
1	4532	3686
1	14236	16567
1	1002	414
0	4	11
1	86548	106819
1	27320	38349
1	69885	32794
0	0	2
1	12459	8193
0	46	260
0	0	0
1	625	1371
1	966	836
0	4	10
0	1	0
0	48	136
1	7052	11604
1	4	12
1	270	376
0	64	117
0	3	1
1	7302	9592

1	3063	3472
1	9352	3272
1	17265	18027
0	0	3
1	5	232
0	1	12
1	515	11048
1	2664	3142
1	4788	2517
1	7380	9436
1	630	620
1	286	383
0	1	0
0	0	12
1	7228	13335
1	2227	1934
0	18	4
1	13738	5275
1	331	3395
1	25626	13575
1	79	1085
1	462	608
0	0	6
1	655	738
0	27	209
0	17	85
0	0	0
1	10439	9047
0	17	5
1	321	425
0	35	106
0	1	5
0	11	27
1	1832	1821
0	1	0
1	27403	1649
0	1	4
1	5665	2006
0	18	227
0	10	15
1	7515	7244
1	2782	364
0	2	1
1	6580	8087
1	3227	905
0	6	25
1	3434	6188
0	1	7
1	8597	11933
1	33453	4825
1	465	804
1	5031	6451
0	13	4
0	2	35
0	0	2
1	2699	3083
0	28	213

0	18	4
0	1	2
0	0	0
0	1	4
1	1238	1670
0	24	1
1	5176	1647
1	33	656
0	12	12
0	0	2
1	2108	368
0	1	2
1	788	2585
1	1303	1288
0	2	7
0	90	83
0	0	3
1	1912	1264
1	2891	1065
1	9973	1963
0	0	0
1	2095	2916
1	57705	16135
1	6768	1606
0	163	121
0	0	0
1	96146	144728
1	1983	3239
0	0	0
0	5	2
1	5589	2408
0	67	1044
1	5721	2749
0	16	78
1	14402	14492
1	197532	364209
0	56	50
1	168	1826
1	5756	1636
1	17847	3579
1	1975	2015
0	7	14
0	2	80
1	24715	38247
0	0	0
0	0	10
1	25502	14731
1	4451	991
1	11659	2505
1	13821	28138
0	0	0
1	6754	6083
0	14	6
1	4020	2661
1	6883	9801
1	7046	9013
0	31	70

1	7191	9020
1	1379	1546
1	8320	3471
1	7029	2704
1	4444	5910
0	35	346
0	2	8
0	5	2
0	0	1
1	604	10737
1	50513	35030
0	0	0
0	2445	1378
1	5175	196
0	7	3
0	1	1
0	55	75
1	7306	4804
1	5918	2493
0	4	3
1	22869	23328
1	36078	44029
1	4782	5387
1	7651	3645
1	60035	91052
1	2821	1214
1	113	1664
1	6519	2328
1	4444	6060
0	7	85
0	0	0
0	2	1
1	753	1291
1	3545	1595
1	207	3182
1	47661	9458
0	12	16
1	7798	9658
1	32907	725
1	6508	11130
1	18432	9165
1	5411	960
0	968	648
1	19486	22463
1	54	1227
0	82	466
1	8543	7110
1	2508	1306
1	4219	5143
1	9602	13322
1	1550	690
1	1536	1635
1	33792	41405
1	4025	1942
0	36	2
0	24	7
1	392236	599377

0	32	43
1	9350	12628
0	43	91
1	23232	29155
1	6039	5196
1	7085	1022
1	5113	6961
0	0	0
1	4087	4253
1	13627	9606
1	7534	6888
1	470	7531
0	158	170
0	0	0
0	6	9
1	2742	1101
0	41	29
1	214	187
1	1509	27897
1	14803	16602
1	8235	11708
1	9574	14565
1	4171	2766
1	11322	9364
1	10251	991
0	471	227
0	0	0
1	4668	346
0	0	0
0	19	18
0	0	0
1	4585	2578
0	0	1
1	284	227
0	101	115
0	7	35
0	0	0
1	2637	2582
1	11366	3768
1	3201	726
0	0	0
0	12	71
1	12969	19106
1	17579	3834
1	21847	29103
0	19	144
1	9037	878
1	176	219
1	33570	968
0	69	11
1	2477	800
1	7241	1901
0	409	30
1	23180	3854
1	129743	169275
1	18	69
0	1	0

1	5670	244
0	107	78
1	22864	10037
1	399	309
1	7276	5580
0	0	0
1	10375	18448
1	9516	7914
0	9	8
1	28924	25811
1	36287	730
1	7838	10817
1	46623	62580
0	0	14
1	105	1838
1	20357	23679
0	0	0
1	1200	19918
1	5450	5969
0	4	9
1	66225	87427
0	469	141
1	4331	2790
1	5352	7427
1	26672	19484
1	199	2762
1	7599	5602
1	2366	2447
1	25897	5382
1	12638	6408
1	16144	19276
1	254	2879
0	3	1
0	7	55
0	0	0
0	17	210
0	11	46
1	383	528
0	0	2
0	0	0
0	0	7
0	10	141
1	34833	39120
0	0	3
0	4	36
1	757	1070
0	3	51
0	15	219
0	0	2
0	18	30
0	0	1
0	0	1
0	0	3
1	557	526
0	6	4
1	22443	9139
0	0	0

0	0	3
0	2	7
1	8077	2512
1	13	115
0	92	156
0	21	35
0	1	18
1	2445	582
0	1	7
0	0	0
0	0	0
0	0	0
0	1	5
1	5605	6099
1	7370	8130
1	178	1003
0	40	561
0	0	3
0	3	34
1	5544	1202
0	14	78
1	3	86
0	0	34
0	0	0
0	0	0
0	0	4
1	6488	4318
0	0	0
0	0	0
0	3	7
1	3740	10370
0	0	3
1	2006	2550
0	0	5
0	0	0
1	10275	8393
0	0	0
1	5770	1612
0	25	18
1	8097	2785
0	0	0
1	1992	2594
0	79	49
0	0	0
0	0	0
0	4	5
0	0	0
0	1	13
1	200	447
1	12755	6447
0	2	0
1	6760	7550
1	4657	3230
1	5737	4240
0	0	0
1	8617	4399
1	3815	2810

0	26	26
1	2508	791
0	11	33
0	3	2
1	14922	19006
1	126	1432
0	0	0
1	2158	602
0	0	0
0	0	0
0	0	0
0	7	75
1	146	1625
0	5	5
1	12311	2411
0	67	619
0	1301	71
0	7	59
0	0	2
0	15	10
0	0	0
0	0	0
0	43	40
0	0	1
0	0	1
1	7086	8290
0	5	2
0	0	1
1	4087	2994
1	237	348
0	25	14
1	8117	7780
0	50	199
1	2284	769
0	0	0
1	28511	11418
0	20	36
0	203	1027
0	0	0
0	0	0
0	25	7
0	4	4
0	0	0
0	45	33
0	0	0
0	1	6
1	4297	7000
1	7273	11395
0	0	2
1	412	439
0	5	16
1	25401	9472
0	0	0
0	46	31
0	21	26
0	0	5
1	20940	2344

1	219	215
0	1	30
0	6	33
0	90	217
0	1	3
1	13285	8806
1	1053	293
0	0	0
0	0	8
0	5	8
1	36	217
0	138	1302
1	1336	1415
1	3342	4354
0	0	1
1	2338	823
1	307	147
1	1426	1200
0	34	0
1	8537	10860
1	1919	381
0	29	34
1	8548	5646
0	0	2
0	0	7
0	0	2
0	0	0
0	0	0
0	0	0
0	2	0
1	201	292
1	66	92
1	2610	1107
1	8131	8491
1	3732	2187
0	0	0
1	1447	1234
0	27	293
0	8	4
0	0	2
0	13	13
1	2734	1703
0	6	81
0	0	0
0	0	0
1	1468	1167
0	0	0
0	0	0
0	0	0
1	3509	3581
0	2	23
0	4	7
1	8401	9461
0	3	7
0	7	22
1	11681	3861
0	0	0

0	19	124
0	8	52
0	0	0
0	47	18
1	3126	1258
0	0	0
0	3	2
0	0	0
1	7146	9269
1	11967	4198
1	6151	1492
0	0	0
0	0	0
0	0	0
0	0	0
0	1	6
0	0	1
0	2	10
0	1	0
0	1	1
1	3832	2385
1	1632	1222
0	16	67
0	7	90
1	5150	2639
0	0	0
1	3566	3056
1	11625	15715
0	0	1
0	0	0
1	13188	11100
0	24	446
0	19	9
1	996	1407
1	9755	723
0	0	1
1	1136	837
0	0	10
0	2	53
0	0	0
0	4	37
0	36	419
1	13852	17406
0	0	1
0	0	0
0	0	0
0	0	0
0	0	0
1	1191	582
0	0	0
0	56	52
0	0	4
1	26892	48596
1	597	1337
0	4	84
1	7950	3890
0	876	840

0	1	5
0	0	0
0	1118	349
0	0	6
0	1	3
0	0	0
0	0	0
1	2142	1875
1	56237	110400
0	0	0
0	726	276
0	0	0
0	19	258
0	1	0
1	4657	2612
1	464	377
0	0	3
1	7998	8055
0	0	0
1	3867	5279
0	0	2
0	0	4
0	0	1
0	0	0
0	0	0
0	0	2
0	0	0
1	51108	64963
0	0	0
1	3310	2445
0	1	0
1	4855	5377
1	956	514
0	0	6
1	1678	1868
1	109072	161109
1	26	267
0	3	0
0	4	39
1	5771	2010
0	9	12
0	0	0
0	6	30
1	7573	6418
1	13802	12956
0	0	1
0	1	2
0	19	18
1	3	11
0	10	12
1	14344	2560
1	9697	3923
1	2734	1545
0	7	35
0	5	20
0	29	348
0	3	3

1	17248	12169
0	107	1379
1	1983	297
0	40	329
0	12	69
1	11662	12872
1	1226	1616
0	0	0
0	0	0
0	757	98
0	3	27
1	10444	4632
1	993	352
1	773	470
1	3492	2906
1	3177	2230
1	1482	1281
1	3933	5391
0	8	71
0	3	2
0	0	0
0	0	0
0	0	5
0	1	20
0	1	2
1	821	1105
0	349	325
1	6222	3318
0	0	1
1	12550	4542
1	80	1130
0	0	0
1	4437	6051
1	2996	5594
0	0	0
0	0	0
0	19	10
1	1189	1729
1	10579	4541
1	4704	7015
1	10108	26349
0	0	0
1	1570	1304
1	5411	3610
1	88	1183
1	417	477
0	4	24
1	5443	6297
1	6984	7593
1	3360	3603
0	44	146
0	161	197
1	6773	3728
0	0	0
1	215	4726
0	0	0
0	0	0

1	8063	11262
1	8018	1364
0	2	15
0	2	19
0	0	0
0	0	0
0	0	0
0	4	8
1	629	5937
1	5450	971
1	1660	2139
1	76	1321
0	4	11
1	18112	15740
0	398	276
0	2	0
1	5395	2241
1	4693	3489
0	32	299
0	1	0
0	22	23
0	73	359
1	112	1506
1	30966	35373
1	4616	1351
1	10674	5650
1	1433	538
1	1287	4856
0	135	76
1	16214	23860
0	5	26
1	8975	5162
0	8	37
1	1453	2584
1	480	157
0	10	4
0	50	21
1	6686	3693
1	18883	25922
1	1284	916
0	0	0
0	0	0
1	3586	1200
1	24	28
0	0	0
1	799	747
0	2	11
0	0	3
1	12971	22025
0	4	4
0	0	0
0	0	0
0	77	61
0	0	0
0	140	95
0	1	0
0	10	17

1	31926	7279
0	9	118
1	7411	8010
0	1	43
0	3	5
0	12	31
1	8293	13193
0	0	0
0	0	2
0	144	87
1	14482	8387
0	2	7
1	5507	5322
1	10560	8477
1	15726	2836
0	1	5
0	0	2
0	2	2
1	303	462
1	314	409
1	2872	3812
0	146	180
0	15	20
0	2	3
1	7	92
1	11	205
1	4240	3777
1	8391	10406
1	362	425
0	2	0
1	28	333
0	2	8
0	524	451
0	0	0
1	1181	1026
1	3048	1662
0	0	0
0	0	3
0	0	7
0	1	0
0	0	4
1	12448	3884
0	33	170
0	25	25
0	1	0
1	1454	50
1	243	218
0	610	27
0	6	28
0	1	3
1	14186	14585
1	13372	4428
0	0	0
1	4182	2293
0	3	5
1	1283	1272
1	5860	4205

0	2	4
0	0	14
1	13573	1717
0	1	3
1	5563	1133
0	1	0
0	17	13
1	4363	3640
1	0	34
0	2	39
1	93	1445
0	0	0
1	2927	475
0	5	13
1	5705	221
0	24	22
0	31	57
0	267	80
0	0	2
1	623	1018
0	8	5
0	179	142
1	246	4621
1	17930	15376
1	985	10380
1	1598	1119
1	4906	17721
0	2	0
0	9	28
0	1	6
0	0	0
0	0	0
0	0	0
0	0	0
1	13751	1722
0	1	0
0	109	119
0	64	507
0	1	0
0	0	0
1	39	141
0	0	0
0	6	10
0	0	10
0	4	3
1	4304	5885
0	3	2
1	4377	2783
1	651	12915
0	0	0
0	0	0
0	0	3
0	22	358
0	0	0
0	9	44
0	3	0
0	0	0

0	0	0
0	10	45
0	0	7
1	263	41
1	56	3
1	45	141
0	4	39
1	40	19
1	281	351
1	0	5
1	669	606
1	1770	1504
0	4	5
1	216	531
0	0	0
1	1581	1982
0	1	5
0	6	7
1	692	6350
0	0	0
1	12824	5774
0	1076	510
0	13	7
0	0	0
0	5	14
0	3	10
1	11574	2145
0	0	0
0	0	0
0	0	0
0	1	19
0	0	0
0	0	0
0	0	0
0	0	1
0	0	0
0	0	2
0	0	0
0	0	0
0	0	0
0	0	0
0	5560	851
0	0	0
0	0	0
0	0	0
0	0	0
0	0	0
0	0	0
0	1	6
0	0	0
0	0	0
0	0	0
1	3636	427
0	0	0
0	0	0
0	0	4
0	0	1

1	24459	15858
0	0	0
0	0	2
0	0	5
0	0	0
0	0	0
0	0	1
0	0	1
0	0	0
0	0	1
0	0	0
0	0	0
0	6	14
0	0	0
0	0	0
0	8	3
0	0	0
0	0	0
0	0	0
0	0	0
0	0	0
0	0	0
0	0	1
0	0	0
0	0	0
0	0	0
0	0	0
0	0	0
0	0	0
0	0	0
0	0	0
0	0	0
0	0	0
0	0	0
0	0	0
0	0	0
0	0	0
0	0	0
0	0	0
0	0	0
0	0	0
0	0	0
0	0	0
0	0	0
0	0	0
0	0	0
0	0	0
0	0	0
0	0	0
0	16	6
0	0	0
0	0	0
0	0	0
0	0	0
0	0	0
0	0	0
0	0	0
0	0	0

0	0	0
0	0	0
0	0	0
0	0	0
0	0	0
0	0	0
0	0	0
1	75	661
0	0	0
0	0	0
0	0	0
0	0	0
0	0	0
0	0	0
0	0	0
0	0	0
0	0	0
0	0	0
1	366	957
0	0	0
0	0	0
0	0	0
0	0	0
0	0	0
0	0	0
0	0	0
0	0	0
0	0	0
0	0	0
0	0	0
0	0	0
0	0	0
0	0	0
0	0	0
0	0	0
0	0	0
0	0	0
0	0	0
0	0	0
0	0	0
0	0	0
0	0	0
0	0	0
0	0	0
0	0	0
0	0	0
0	0	0
0	0	0
1	40	719
0	0	0
0	0	0
0	0	0
0	0	0
0	0	0
0	0	0
0	0	0
0	0	0

0	0	0
0	0	0
0	0	0
0	0	0
0	0	0
0	0	0
0	0	0
0	0	0
0	0	0
0	0	1
0	0	0
0	0	0
0	0	0
0	0	0
0	0	4
0	0	1
0	0	0
0	0	0
0	0	0
0	0	0
0	0	0
0	0	0
0	0	0
0	12	17
1	3714	541
0	0	0
0	2	1
1	8425	5498
0	127	113
0	30	9
0	4	83
1	23713	29921
0	2	4
0	0	0
0	21	81
1	2	31
0	0	1
1	916	2892
0	0	0
0	0	1
0	0	0
0	0	0
0	0	0
0	1	0
0	0	2
1	3836	2339
1	23021	9837
0	52	67
1	2633	2877
1	23186	5219
1	3931	1289
1	10717	12639
1	5794	1771
1	20708	7441
0	57	121
1	3726	4335
0	6	49

1	13575	10656
1	5068	2940
1	379	411
0	1	29
1	1589	331
1	4969	6508
1	4150	1040
0	0	0
0	4	65
1	1125	1182
0	15	4
0	0	6
0	0	0
0	0	0
0	0	2
1	8471	4893
0	0	0
1	2836	1566
1	8984	17801
1	6818	9616
1	5321	6980
0	0	0
0	0	0
1	1246	1630
0	10	57
1	3002	404
1	1984	3028
1	2592	1232
0	17	9
0	2	9
0	0	0
0	0	6
0	0	1
1	2140	612
1	2553	4003
1	13855	8379
1	11504	2433
0	0	8
1	9021	15191
0	0	0
1	9065	11615
1	1669	1825
1	2811	500
0	69	174
0	117	631
1	28609	26256
0	0	2
1	9669	4577
1	31957	24434
0	64	81
1	1156	299
1	10141	8495
0	1214	2817
0	37	122
0	4	7
1	1205	1894
0	0	0

0	2	0
1	4551	1650
0	76	239
1	14889	6228
0	94	44
1	6281	5761
1	44959	26959
1	5496	2950
1	2094	2241
1	2707	2509
1	21721	53405
1	14857	763
1	41472	21888
1	4451	3668
1	9017	11223
1	5435	7856
1	4233	4337
1	17257	14602
1	3875	2045
0	7	140
1	351	2227
0	2	10
1	3628	5168
0	126	163
1	10619	11678
1	8258	8089
1	1580	883
1	2587	402
1	10844	3857
1	18082	19980
0	27	29
0	5	6
1	8114	9881
1	2621	1308
1	612	651
1	1023	1457
1	4791	4113
0	0	1
1	58801	69410
1	16454	21275
1	14320	13043
0	41	45
1	432	9249
0	0	15
1	3011	1012
1	13259	21454
1	20033	9652
0	51	599
1	6538	7884
1	7516	11291
1	5739	5586
1	1499	1721
1	15924	9229
1	5440	133
1	1329	5853
0	0	2
1	502	1196

1	2509	2474
0	0	0
1	2491	926
1	55060	96562
1	12986	16421
1	14670	16567
0	0	10
1	21807	6764
1	2745	2517
0	0	0
0	19	46
1	1487	399
1	13936	14088
1	12445	7152
0	5	20
1	663	10933
1	927	1788
0	1	1
1	3141	714
1	9200	3168
1	6792	3667
1	230	3084
1	5947	1446
0	16	20
0	15	53
0	1	23
0	15	15
1	41856	27263
1	1508	1655
1	2469	1384
1	1814	3385
0	1	14
1	10421	5779
1	9116	6034
1	1469	1535
1	58798	59327
1	1201	445
1	276187	401674
1	19501	12642
0	0	2
1	7301	1161
1	40492	9995
1	5534	7936
1	25296	17166
1	535	908
1	384	428
0	0	0
0	0	1
0	5	8
1	2818	3765
1	9763	5602
1	10196	6071
1	9955	5767
0	10	11
0	138	73
1	13048	6817
1	3587	1163

0	0	0
1	11553	9203
0	0	0
1	9104	4358
1	4	7
0	0	0
1	5508	4750
1	62978	99588
0	22	194
1	7276	4064
1	16221	1405
1	25237	30378
1	911	246
0	30	321
0	107	858
1	3028	996
1	13621	11654
0	1	2
0	117	76
0	1	1
1	15366	16645
0	3	2
0	168	1489
0	0	6
0	71	65
0	6	4
0	26	91
1	10490	6192
0	0	8
0	0	0
1	209	327
0	820	750
1	134	271
1	94515	41350
1	3758	4265
0	0	0
0	2	3
1	11889	18974
0	0	3
0	74	779
0	0	0
1	11273	4903
1	32050	29650
1	1596	3788
0	51	33
0	4	2
1	2720	2501
0	1	4
1	87168	3628
1	223750	6821
0	210	47
1	7984	10475
1	2904	1859
1	9147	16192
1	7721	4113
1	1966	28285
1	7923	8117

0	0	0
1	210	5205
1	13943	2386
0	3	31
1	11366	10560
0	701	59
1	19965	11757
0	932	110
1	6615	1769
1	23669	35730
1	22857	25948
1	4317	5194
0	3	6
1	12040	13883
1	18791	31005
1	1696	2137
1	1608	1617
0	2	9
1	21199	27174
1	7982	1323
0	2	0
0	20	25
1	11945	6913
1	29	604
1	21664	11465
0	1	4
0	0	0
0	3	27
1	1342	614
1	4632	981
1	2703	985
0	12	220
1	15913	7960
0	151	102
0	8	21
0	70	354
0	0	12
0	0	1
1	16071	11205
1	31975	19164
1	8631	5459
0	2	2
1	7613	11645
1	3666	2446
1	11578	5156
1	770	765
1	3451	1448
1	1199	18066
1	3717	5910
1	5427	3917
1	2011	1637
1	3971	4014
0	7	17
1	13799	245
0	0	0
0	0	0
0	0	0

0	0	0
1	9534	8122
1	617	7678
1	27199	49022
0	337	10
1	9316	9245
1	6122	9073
0	0	0
1	7811	4503
0	1	11
1	347	1929
0	6	55
1	10293	12672
0	4	15
0	5	19
0	1	4
1	5425	9197
1	6563	2266
1	5021	4470
0	12	5
1	4871	2344
1	16361	13223
1	10007	4314
0	58	76
0	10	20
1	20626	14171
0	2	61
1	20939	5792
0	0	5
1	24024	38816
1	10934	9172
0	0	0
1	24147	34980
0	0	0
1	14206	12630
0	5	3
1	20472	60280
1	3340	783
0	1	2
1	3780	1420
1	10956	1094
1	46	560
1	3339	664
1	97	1230
1	6319	3185
1	6924	3434
1	3109	1624
1	1685	809
1	2321	1649
1	19198	19873
0	0	2
1	5424	1738
0	47	31
0	2	65
0	188	105
0	62	499
1	14238	27298

1	6602	1727
0	0	0
1	3562	2285
1	15570	14242
0	0	0
0	1	0
0	0	2
0	1	0
1	521	9335
1	1644	31314
0	71	190
1	9880	16263
0	3	8
1	1626	1665
1	7406	9663
1	3926	3443
1	3833	617
0	0	1
1	60202	92597
1	74	1967
0	0	0
1	4034	4468
0	5	10
1	2019	933
1	8481	1902
1	230	234
0	0	0
0	100	97
1	4049	4951
0	7	61
1	10617	3846
0	0	0
0	51	62
1	5622	5593
1	5636	7253
1	21059	7557
0	4	2
1	94	1740
0	13	17
1	11841	2718
0	9	46
0	6	11
1	4462	1576
1	665	1457
1	1092	769
1	1702	776
0	0	4
1	447	322
1	9162	9949
1	4238	1691
1	168	2984
0	0	0
0	0	0
0	30	298
1	4526	5842
1	1065	878
1	9898	1490

1	9757	10943
0	20	27
1	6035	5804
1	3296	1301
0	3	6
0	23	29
1	3995	527
1	13191	21346
1	1194	719
1	2384	324
1	20386	30177
1	59394	87952
0	0	12
0	0	0
0	16	18
1	9257	1776
1	10345	7201
1	4641	3657
1	34371	9961
0	0	0
1	8347	8857
1	8916	4585
1	7213	2262
1	96	2017
0	13	181
0	0	0
1	2503	1679
1	7193	554
1	14397	13175
1	4308	1493
0	33	23
1	2891	97
1	5176	4939
1	2808	2756
1	2913	473
1	572	353
0	1	0
1	8600	8529
1	1246	16001
0	4	4
1	12030	2740
1	4695	2465
1	145	2518
0	0	0
0	8	20
0	0	15
1	472	814
1	6247	4434
1	7700	4465
1	1047	1312
1	11586	6756
1	134	1702
1	21773	12738
1	1477	1581
1	3375	3638
1	7292	3177
1	391	1146

0	1	26
1	1168	2673
1	211	2384
1	817	888
1	4379	679
0	15	187
1	478	315
0	8	5
1	8463	3837
1	2865	1978
1	36305	58809
1	0	7
1	3327	4245
1	1985	32165
1	4369	4989
0	0	9
1	5630	9346
1	4032	1508
1	2315	3634
0	1755	794
1	3759	3679
0	4	10
1	9274	4741
1	17157	22962
1	22763	33888
0	0	0
1	15710	10352
1	16531	10980
1	3169	1886
1	1879	1899
0	3	2
1	104	3853
1	3607	4989
1	9261	3643
0	0	0
1	20461	18488
1	60188	89270
1	12794	1411
1	14218	22704
0	16	24
0	6	6
0	12	14
1	1805	1679
1	11332	432
0	4	1
0	0	0
0	0	0
1	1014	393
1	6309	8805
1	691	730
1	4094	1547
1	860	2155
0	0	0
0	0	1
1	14849	11227
1	27144	10332
1	8910	14073

1	15269	11296
1	5969	12328
1	35752	9962
0	9	159
1	28791	5386
0	4	11
1	18912	6197
1	1379	1143
0	0	0
0	31	103
1	338	176
0	204	202
1	15629	4775
1	6959	5252
0	12	13
0	43	194
0	0	0
1	49589	85856
0	1	0
1	4792	24173
1	42787	50248
1	1938	1635
0	0	1
1	178	413
1	5878	6314
0	14	25
1	7086	7050
1	2319	1591
0	18	81
1	17631	16938
1	4754	2460
0	468	438
1	7288	3543
0	187	159
1	1424	506
1	6295	2727
0	12	50
1	2734	2986
1	42021	51186
1	3586	1252
0	39	32
1	38565	63417
1	179652	259131
1	3256	1678
1	5679	8462
1	4932	5118
1	10723	15830
1	40668	60844
1	7939	11133
0	7	46
0	1	12
0	0	0
1	10736	10778
1	4734	3931
1	10474	4428
1	10276	9089
1	3158	3888

1	3326	375
1	4398	2785
1	630	8706
1	3479	817
1	6572	1853
0	72	17
0	8	5
1	8997	2425
1	6823	5937
1	3532	4386
1	6178	8183
0	267	177
0	15	31
1	7215	1140
1	875	1224
1	46486	37045
1	21468	20571
1	3669	3744
0	0	0
0	7	1
1	1454	728
0	286	36
0	0	3
1	937	1206
0	4	1
0	61	57
0	3	26
0	137	89
0	0	3
1	26655	31648
0	0	0
1	2657	4853
0	37	548
1	2788	222
0	0	0
1	3061	2486
1	8461	3828
1	18456	10096
0	8	5
1	3294	2021
1	5538	4448
0	89	74
1	21488	19392
1	5296	3795
1	2609	1255
1	2598	2645
1	10533	6837
0	2	51
1	16627	9050
1	5855	5331
1	8379	8982
1	6727	4785
0	0	4
0	4	4
1	7789	6088
0	2	0
0	152	153

1	4166	2555
1	8192	7186
0	9	1
0	2	1
1	22026	23606
0	6	43
1	6749	898
1	15170	15255
1	7015	5063
0	1	0
1	7568	8031
1	8171	5021
0	5	4
0	0	0
0	40	187
1	44839	66847
1	2948	3289
1	4479	4190
1	2257	32534
1	14489	14524
1	5886	6121
1	3088	2854
1	8	7
1	15928	22061
1	8862	6145
1	5873	746
1	36795	45695
1	112	549
1	3034	4089
1	5020	3108
1	3906	2505
0	1	4
0	7	36
1	1381	2959
1	10746	29692
1	3014	722
0	56	73
1	16503	8685
1	4577	3837
0	2	4
1	6370	3332
1	20585	3997
1	760	10141
0	4	0
1	1266	17751
1	6513	2306
1	1673	1306
0	65	99
0	0	0
0	6	3
1	5603	1065
1	14500	18946
1	17660	32564
1	28462	40488
1	29429	47725
0	0	22
1	9275	919

1	17888	15064
0	66	255
0	0	31
1	6872	1425
1	5282	3566
0	4	14
1	47	828
1	10100	5161
1	356	1034
0	0	0
1	880	510
0	0	2
0	5	16
1	13071	9930
1	11559	13603
0	610	24
1	6130	11803
1	7319	5529
1	3065	5388
1	4494	2521
1	10085	3725
1	3193	4485
0	1	14
1	4635	4855
1	123	421
1	8396	5955
0	0	4
1	24211	1370
1	2596	1018
0	0	1
0	6	23
1	4017	1972
1	44	829
1	3252	3949
1	14420	1701
0	9	49
0	1560	130
0	45	172
0	0	0
0	11	4
0	0	8
1	89875	131381
0	1328	70
1	5469	2334
1	4051	952
1	1447	1647
0	7	6
1	7923	11457
1	13164	14341
1	6367	2099
1	6428	8363
1	1823	1334
1	896	1282
1	5803	13204
0	9	14
1	13774	11154
1	960	832

1	10029	13570
1	16625	6830
1	10290	8907
1	34284	48861
1	14009	19431
1	11136	8802
1	1361	23575
1	26579	9155
1	8870	3048
0	0	2
0	0	3
1	1711	2042
0	2	0
1	7304	6627
0	207	76
0	6	0
1	6319	1007
0	7	2
0	0	13
0	1	0
1	11705	582
0	0	0
1	18484	11114
1	13108	944
1	15601	14308
1	7364	9233
1	21787	8284
0	0	0
0	17	9
1	262	395
0	21	268
0	57	421
1	15324	8550
0	31	10
1	59792	85182
1	13610	3427
1	185	679
0	56	26
0	6	8
1	10732	7491
0	43	15
1	868	545
0	0	0
0	17	117
1	835	1115
1	2246	259
1	20	256
1	5182	7341
0	6	9
1	7345	7868
1	14104	24213
1	20601	14835
1	29537	11687
1	30046	51927
0	18	226
1	14572	18313
1	12931	11198

1	9577	20312
0	3099	1222
0	12	1
1	7303	1689
0	5	76
0	42	14
1	7225	6956
0	103	28
0	38	140
0	22	14
1	12716	4990
0	0	0
1	5452	4481
0	9	9
1	4309	371
0	0	0
0	0	11
1	12	25
0	0	0
0	9	3
0	0	0
0	91	81
1	18142	6655
1	3035	4049
0	2	5
0	4	5
0	24	9
0	0	5
1	34290	2525
0	0	0
1	2349	1229
1	286	6944
0	9	131
0	31	7
0	10	9
0	2	0
0	137	130
1	10627	9132
0	0	1
1	1289	800
1	8510	6963
1	368	7801
0	178	182
0	0	0
1	12146	8691
1	86359	121864
0	0	0
1	7845	8876
1	4560	6074
0	9	123
1	86	813
0	10	29
1	17617	13301
0	0	8
1	6945	11655
1	920	1566
1	8823	10223

1	14316	14775
1	5304	1408
0	1	14
1	5733	2099
0	0	0
1	208	4283
0	0	1
1	10630	10508
0	6	19
0	0	0
0	10	69
1	6008	9315
1	6331	6499
0	0	0
1	5610	4365
0	1	1
0	1	16
0	22	63
1	5388	2366
1	11779	11455
1	3073	802
0	0	0
1	9062	2121
1	2283	2632
1	10842	11255
0	28	84
1	5478	7952
1	1389	1013
0	0	0
1	1297	1203
0	5	47
0	0	0
1	6637	8071
0	3	43
0	7	8
0	15	84
0	36	335
1	11895	6422
0	3	5
1	288	7681
1	13148	17696
1	15091	13558
0	1	1
1	3127	1498
0	0	0
1	5018	3744
1	623	8342
0	13	6
1	3470	361
1	8540	3262
1	5178	7431
1	3694	1099
0	4	4
0	21	59
1	4937	6212
1	10852	3609
1	34596	1094

0	431	9
1	11599	2217
0	0	9
0	26	13
1	6332	573
0	156	219
0	0	2
1	11672	10665
1	544	736
1	449	365
0	3	19
1	2877	3108
1	3045	511
1	77	515
1	16360	8976
1	4074	4678
1	8334	11611
0	14	42
0	10	57
0	4	4
0	13	1
1	99172	133610
0	44	38
1	14343	9326
1	13274	12419
1	7479	8574
1	6238	5065
1	189	3431
0	17	104
1	5140	6479
1	9979	2555
1	28858	13899
1	4955	7255
1	1206	699
1	1771	3986
1	539	3801
0	0	2
1	1162	1780
1	4196	7611
0	239	733
0	1	0
1	208	383
1	15394	14821
1	8121	11807
0	17	18
1	8057	1379
1	32200	39733
1	3999	1331
0	2	2
0	2	4
0	0	0
1	8835	1710
0	22	13
1	3181	279
1	7674	8702
1	5441	2285
0	111	161

0	0	45
0	29	30
0	0	3
1	9307	11812
0	0	0
0	1	21
1	923	219
1	1726	1279
1	3177	2587
1	16282	21426
0	2	43
1	18003	3259
1	19729	18618
1	48304	55092
1	21760	4767
1	2460	3109
0	393	321
0	35	66
0	1	0
1	6518	1820
1	16547	22160
1	17229	16536
1	2154	1331
1	5240	4979
0	0	7
1	123	281
1	26749	38277
0	0	13
1	1575	607
1	4219	7655
1	2197	2293
0	19	18
0	7	6
1	64163	6249
1	4188	7041
1	2668	1908
1	6808	8576
0	0	0
1	9243	3993
1	6014	7268
1	1028	1621
1	23	224
0	4	2
1	39174	52432
1	135912	187379
0	4	5
0	3	1
0	0	2
1	2547	745
1	18158	15305
1	3522	666
0	82	749
1	4407	4441
0	201	70
1	13361	12139
1	10026	2154
0	2	9

1	4487	826
0	0	6
1	1617	728
0	0	0
1	3191	3466
0	17	972
0	2	15
1	5963	5536
0	2	3
1	5564	3190
1	8771	18548
0	0	3
0	8	33
0	5	2
0	0	0
1	4524	6581
1	131	3031
0	16	108
0	2	15
1	1155	15424
0	63	107
1	3207	4121
1	47415	6611
0	3	1
1	219	4057
1	6236	4217
1	12333	3079
0	31	84
1	3640	5930
0	0	19
1	7467	595
0	0	0
0	0	0
1	581	550
1	14098	11795
1	8507	1966
1	943	530
0	28	13
1	10212	7349
1	1786	995
1	1100	1021
1	15	205
1	3336	4527
1	3006	1708
0	18	324
1	14228	38208
0	1	22
0	2	10
0	4	56
1	6453	2289
1	14721	26885
1	5666	8878
1	2336	2004
0	0	0
0	0	19
0	2	0
1	24256	26459

0	246	45
1	3989	2209
0	36	20
0	0	1
1	26133	5498
1	15445	11495
1	19658	6356
1	3932	476
0	20	67
0	2	7
0	22	8
0	142	1075
0	0	2
1	4604	1251
1	58	68
1	6458	6764
0	1	10
1	1	1
1	4722	1663
1	14187	24676
1	1805	1280
1	12791	3118
1	67	340
0	2	8
1	8715	4594
1	20699	24457
0	1	26
1	8466	5657
1	8180	8661
0	3	46
1	27241	13619
0	0	13
0	922	202
1	209	3296
0	0	2
1	9266	12303
0	2	17
1	2336	2693
0	0	1
0	41	73
0	3	33
1	7750	4687
0	0	5
1	31856	50290
1	19447	8903
0	0	0
1	20806	21978
1	6502	4282
1	622	235
1	53197	84385
1	587	962
1	167	3865
1	950	1209
0	43	257
1	13720	24556
0	12	93
1	4085	4681

0	6	41
0	184	120
0	14	8
1	4966	7635
1	3653	4950
1	114	556
0	9	30
0	117	41
1	3345	4430
0	54	29
0	28	10
0	57	117
1	5925	6888
1	12681	18372
1	5855	2300
1	7292	3144
1	6637	9681
1	7245	3122
1	3052	4013
1	9807	16038
1	8360	15257
1	30902	48056
0	97	40
0	6	9
0	4	23
1	45207	13443
1	21903	31437
1	3538	1839
1	60	984
1	37225	37562
0	4	5
0	25	45
0	0	0
1	8041	13443
1	3245	1804
1	1569	1290
1	12728	7660
0	3	40
0	1	0
0	133	344
0	0	0
1	8614	4428
0	9	72
1	7694	5900
1	963	631
0	0	11
0	0	0
1	2929	3142
1	7314	9543
0	0	2
1	3627	2782
1	15337	17422
1	9226	9383
1	4985	1759
1	10750	15888
1	10643	14010
1	16367	11974

0	7	3
1	3418	1768
0	8	105
1	14710	6382
1	5670	4325
0	0	2
1	675	959
1	629	433
1	1873	928
1	3953	5624
1	9802	12993
0	0	0
0	17	10
1	1488	4256
0	2	3
1	520	12015
1	3387	931
0	4	38
1	3318	4628
1	150	2106
0	77	88
1	17336	27270
1	8019	4985
0	33	303
1	3929	2430
0	2	29
0	447	112
1	25783	33613
0	3	17
1	214	1308
1	9748	13976
1	4303	5117
0	1	27
0	1	26
1	57342	30374
0	38	389
1	3990	4380
1	1427	2346
1	1326	569
1	595	8665
1	5327	1674
1	1437	7989
1	5701	2372
1	1413	88
0	0	0
0	1	0
1	2518	1289
1	18444	4219
0	19	121
1	17478	5227
1	2940	1134
1	3463	4152
1	965	1538
1	16443	9902
1	17104	17678
0	266	326
1	16450	9614

1	4677	5116
1	4840	943
1	49979	6270
1	4238	4380
1	1061	14331
0	14	12
1	19474	13723
1	8715	3973
0	35	43
1	544	477
0	5	2
1	11443	15544
1	530	971
1	2144	1376
1	22	74
1	12642	19007
1	753	960
1	2068	699
0	0	2
0	3	61
1	10703	8712
0	0	0
1	3427	2601
0	25	313
1	1409	1157
1	1464	568
1	6541	5879
1	6333	5694
0	34	18
0	11	3
1	1097	1877
1	345	4949
1	4717	1893
0	0	0
0	0	0
1	42625	1645
1	8361	5701
1	12662	13813
1	33639	20543
1	9575	7083
1	2366	902
1	355	493
0	50	78
1	5073	7300
0	5	18
1	1131	390
1	3344	1333
1	2173	3070
1	4220	1619
1	2280	828
1	610	676
1	5131	6114
1	10154	6527
1	2	15
0	2	2
1	2405	576
0	8	42

1	3333	872
0	9	19
0	10	29
0	53	905
1	20552	3274
0	3	23
0	25	272
1	38379	6519
0	2	47
1	6794	11002
1	3993	768
0	21	131
1	39061	60492
1	33543	15521
0	2	5
1	5453	3526
1	12030	10654
1	5765	10421
0	0	0
1	3035	1730
1	8625	6499
1	852	1026
1	2625	281
1	2299	3170
1	12704	2415
1	2930	3229
1	32977	10237
1	7750	6933
0	1	0
1	840	617
1	6051	6489
1	12516	17862
1	11541	14879
1	1362	1331
1	1121	1537
1	2399	1762
1	20587	23290
1	596	1016
1	5150	6110
1	6177	491
1	12692	9124
0	7	15
1	8601	10923
1	23844	8120
1	94651	130769
1	13055	23450
1	4884	3461
1	15168	10883
1	14283	7830
1	13645	11287
1	5559	784
1	3390	4054
0	3	2
1	15408	8965
1	11254	6554
1	2540	1806
1	3285	3367

1	1892	298
1	3349	1874
1	172	1524
1	2206	2938
0	290	1746
1	2623	1630
0	0	0
1	12663	8374
0	9	36
1	10577	7536
0	0	0
1	9892	2667
1	130	640
0	24	44
1	3975	2237
0	8	3
1	13359	7616
0	3	0
0	22	18
1	16014	870
0	2	18
0	7	6
0	0	1
1	22663	4708
0	0	0
1	17084	26991
1	14342	8192
1	2002	813
0	12	131
1	31991	37425
0	31	15
0	23	16
0	25	159
0	3	2
0	6	2
1	3724	3983
0	0	1
1	3953	3484
0	19	0
1	3577	12436
0	0	4
0	8	21
0	26	13
1	15135	5497
0	122	965
1	21364	31149
1	18972	7577
1	10918	16201
1	5159	1043
0	2136	512
1	3911	7168
1	1230	490
1	8306	8209
1	2406	1984
1	3233	477
1	4703	5299
1	71261	97499

1	740	1213
1	16769	33797
0	35	12
1	7291	4589
0	8	5
1	9793	3114
1	17826	27485
0	47	23
0	0	1
1	3229	476
1	11844	3040
1	10844	8216
0	2	2
1	3797	3131
0	1	2
0	12	25
0	23	92
1	11691	20405
1	2789	3088
1	4787	530
1	20731	18266
0	26	45
0	31	34
0	27	17
0	0	0
0	1	1
1	2536	568
1	1845	693
1	5163	3688
1	7429	1243
1	7591	9416
1	15706	1995
0	6	13
1	8157	3443
0	102	560
0	0	0
0	0	5
1	45475	7043
1	5991	3446
0	5	4
0	35	133
1	7142	1550
0	22	34
1	11171	6067
0	3	2
1	25288	11129
1	4452	3245
1	913	520
1	55	85
1	9938	5821
1	530	5139
1	5958	3026
1	10721	6348
1	3196	1717
0	0	0
0	13	18
1	5748	1990

1	3687	1263
1	6704	8482
1	2025	1463
0	2	25
1	8404	648
1	64658	21582
1	5033	5708
1	2409	1102
0	33	1
0	35	0
0	1	5
0	69	18
1	162	2499
1	14705	14881
0	83	59
1	806	15010
0	88	751
0	1	1
1	7132	13341
0	56	163
0	1	3
1	30517	46310
0	34	31
1	10385	18130
0	57	40
1	16781	22019
1	9252	16074
0	17	28
1	8584	1153
1	130583	16132
1	8334	4903
1	4876	16975
0	41	474
1	1648	7631
1	4459	3489
1	70	1425
1	150	2317
1	1641	785
1	1973	2693
0	0	0
0	0	0
1	57336	61310
0	31	80
1	13459	18452
1	6825	7222
1	44	296
1	32878	30200
1	180	273
0	0	1
0	0	7
0	173	2081
0	4	4
1	4651	6417
0	0	6
1	5825	1228
0	2	62
1	13470	21047

0	11	4
0	0	0
1	25827	15064
1	1682	2108
0	86	56
1	13032	14549
1	6954	2050
1	4713	3825
1	13587	10846
1	9315	7350
0	1489	126
1	507	775
0	0	34
0	0	0
0	0	1
0	2	13
1	6834	2429
1	23	41
1	55203	86855
0	56	80
0	182	139
0	163	1489
1	4754	1314
0	0	0
0	0	0
1	10137	3112
0	32	45
1	25137	14048
1	11137	4247
1	5506	6861
1	2861	2272
1	2405	3979
1	4746	408
0	1072	112
1	10023	4301
1	13092	1152
1	22760	33316
0	42	65
1	13836	9018
1	5581	4153
1	17444	4354
1	1185	2161
1	9875	8946
1	1162	1290
1	6269	4122
0	52	573
1	818	18941
1	2874	725
1	98	697
0	0	2
1	13406	13023
1	14251	12681
1	884	692
1	11262	584
1	5217	1414
1	226	1737
0	0	1

0	66	20
1	1432	1993
0	6	2
0	0	8
1	255394	397177
1	46391	25939
0	1	1
1	22986	2135
1	117	772
0	3	5
0	0	28
0	4	2
1	1786	3547
0	49	61
1	3548	5003
1	8757	12369
1	3848	1262
1	3968	4062
0	27	43
0	3	1
0	2	146
1	5266	4282
1	13129	13322
1	1215	2178
0	2	11
1	3121	4075
0	1	45
1	34826	57954
0	23	31
1	3682	4591
0	95	45
1	951	1674
0	2	5
1	5957	1378
0	0	0
1	2451	1143
1	6724	1870
1	10702	9958
1	5943	4530
0	15	105
0	0	9
1	93	1614
1	6679	9720
0	384	197
1	54082	33389
0	35	123
1	21951	26846
1	8817	1596
0	2	30
0	2	8
0	0	0
1	21158	24536
1	7586	3471
1	14347	4572
1	14984	3061
0	149	76
0	8	8

1	13387	11347
0	0	0
0	9	4
0	1	2
0	1	12
0	1	0
0	4	3
1	57	680
0	1	10
0	0	0
1	5944	1095
0	13	25
1	6983	1634
0	0	0
1	5964	1693
1	6891	2422
0	704	126
1	8301	689
1	15234	6994
0	13	3
0	52	60
1	5501	4923
0	2235	94
1	3906	4904
0	0	6
1	4067	1214
1	9312	5349
0	0	0
1	98	384
1	10087	1298
0	0	0
0	7	85
1	2618	1424
1	8607	4127
0	6	63
1	6663	453
1	2916	99
0	49	279
0	8	59
1	1372	909
0	0	0
1	3638	8235
1	19045	9976
0	0	2
1	6052	6494
1	5713	3321
1	13668	8333
1	1752	1548
1	1210	2155
0	3	29
0	1	3
0	10	17
0	30	16
1	3723	565
0	8	7
1	5041	986
0	6	11

0	5	36
1	2389	863
0	1	0
1	4855	1161
0	3	10
1	12570	14878
1	20297	17048
1	11869	17738
0	26	53
1	28543	29382
1	2	101
1	6757	5040
1	13138	5169
0	6	21
0	0	0
0	3	56
1	4778	1251
1	279	236
1	10537	5889
1	6489	5964
1	5629	7122
1	2151	2776
0	411	26
1	5906	3508
0	1	4
0	7	9
0	0	0
0	0	0
1	3836	4007
1	14475	36402
1	12086	6406
1	311	460
0	22	42
1	3416	93
1	16812	2707
1	1	0
1	69318	16755
1	17668	2700
0	22	108
1	2435	956
1	3072	1097
1	11997	4831
0	2040	51
1	5255	2375
1	5236	1481
1	4577	2181
1	15003	12733
1	2214	33461
1	17829	19506
0	72	209
1	2248	1553
1	2793	3175
0	13	17
1	7797	5081
0	3	2
1	38125	70706
1	6940	11812

1	15733	5711
1	5489	774
1	3673	1626
0	1	10
1	4388	3932
1	13076	21260
1	512	552
0	0	1
0	0	0
0	51	2
0	189	101
0	0	0
0	0	0
0	0	0
1	0	0
1	0	0
1	0	0
1	0	0
1	0	0
0	0	0
0	0	0
1	0	0
1	0	0
1	0	0
1	0	0
1	0	0
1	0	0
0	0	0
1	0	0
1	0	0
1	0	0
0	0	0
1	0	0
0	0	0
1	0	0
1	0	0
1	0	0
1	0	0
0	0	0
1	0	0
1	0	0
1	0	0
1	0	0
1	0	0
0	0	3
0	0	0
0	0	0
0	0	0
1	0	1
1	0	0
1	0	1
1	1	0
1	0	0
1	0	0
1	0	0
1	0	0
0	0	0

0	0	0
0	0	0
0	0	1
1	0	0
0	0	0
1	0	0
0	0	0
0	0	0
1	0	0
1	0	0
1	0	0
1	0	0
1	0	0
1	0	0
1	0	0
1	0	0
1	0	0
1	0	0
0	0	0
0	0	0
0	0	6
0	0	0
0	0	1
0	0	0
0	0	0
1	0	0
0	0	0
0	0	0
0	0	0
1	0	0
0	0	0
1	0	0
0	0	0
1	1	0
1	0	0
1	0	0
1	0	0
1	0	0
1	0	0
1	0	0
1	0	0
1	0	0
1	0	0
1	0	0
1	0	0
1	0	0
1	0	0
1	0	0
1	0	0
0	0	1
0	0	0
1	0	0
1	0	0
1	0	0
1	0	0
1	7	4
0	0	0

0	0	0
1	0	0
1	0	1
1	0	0
1	22	477
1	65	1582
0	0	0
0	0	0
0	0	0
0	0	0
0	0	0
1	38	6
0	0	0
1	0	0
0	0	0
1	0	0
0	0	0
1	0	0
0	0	0
0	0	0
0	0	0
0	0	0
0	0	0
0	0	0
1	0	0
0	0	0
1	0	0
0	0	0
1	0	0
0	0	0
0	0	0
0	0	0
0	0	0
1	0	0
1	0	0
0	0	0
0	0	0
0	0	0
1	0	0
0	0	0
1	0	0
1	0	0
1	0	0
0	0	0
1	0	0
1	0	0
1	0	0
1	0	0
1	0	0
1	0	0
0	0	0
1	0	0
1	0	0
1	0	0
1	1	0
0	0	0
1	0	0

1	0	0
1	0	1
1	0	0
1	0	0
1	0	0
1	0	0
1	0	0
0	0	0
1	0	0
0	0	0
0	0	0
0	0	0
1	0	0
0	0	0
1	0	0
0	0	1
0	0	0
0	0	0
0	1	3
0	0	0
0	0	0
0	0	3
0	4	1
0	0	0
1	0	0
0	0	0
0	22	24
1	0	1
0	0	0
1	0	0
0	0	0
1	5	8
1	2	0
1	17	8
1	2	9
0	0	0
0	0	1
0	0	0
0	0	0
0	0	0
0	0	0
0	0	0
0	0	0
1	0	0
0	0	0
0	0	0
0	0	0
1	0	0
0	0	0
0	0	0
0	0	0
0	0	0
1	0	0
0	0	0
1	0	0
1	0	0
0	0	0

1	0	0
1	0	0
1	0	0
1	0	0
1	0	0
1	0	0
1	0	0
1	0	0
1	0	0
0	0	0
0	0	0
0	0	0
1	0	0
0	0	0
1	0	1
0	0	0
0	0	0
1	0	0
0	0	0
1	0	0
1	0	0
1	0	0
1	0	0
0	0	0
1	876	391
1	0	0
1	0	0
1	0	0
0	0	0
1	0	0
0	0	0
0	0	0
0	0	0
0	0	0
1	0	0
1	0	0
0	0	0
0	0	0
0	0	0
0	0	0
0	0	0
0	0	0
0	0	0
0	0	0
0	0	0
1	0	1
0	0	0
0	0	0
0	0	0
0	0	0
0	0	0
0	0	0
1	0	0
1	0	0
0	0	0
0	0	0
1	0	0

0	0	0
0	0	0
0	0	0
1	0	0
1	0	0
0	0	0
0	0	0
1	0	1
0	0	0
0	1	0
1	0	0
1	0	0
1	0	0
0	0	0
1	0	0
0	0	0
1	0	0
0	0	0
1	0	0
1	0	0
1	0	0
1	0	0
0	0	0
1	0	0
0	0	0
1	0	0
0	2	0
0	0	0
0	0	0
0	0	0
0	0	0
1	993	16737
1	0	0
0	0	0
1	0	2
1	0	0
1	0	0
0	0	0
1	0	0
0	0	0
1	2	57
1	902	1700
0	2	6
1	18	0
1	1	10
0	18	133
1	155	571
1	0	9
1	0	3
0	0	1
1	0	0
1	2	4
0	0	0
1	0	0
1	0	0
1	0	0
1	1	25

1	61	564
1	0	0
1	1	4
1	0	0
1	0	0
1	0	1
1	0	0
1	0	0
1	1	1
1	0	0
1	0	0
1	0	0
1	0	2
1	0	1
1	0	2
1	0	1
1	0	8
1	17	38
1	0	0
1	0	0
1	0	2
1	1	8
1	23	4
1	3	3
0	0	0
0	0	0
0	1	1
0	0	0
1	1	1
0	0	0
0	0	6
0	1	5
0	0	0
0	0	0
0	0	0
0	0	0
0	0	0
0	0	0
0	0	0
0	0	0
0	0	0
0	0	0
0	0	0
0	0	0
0	0	0
0	0	0
0	0	0
0	0	0
0	0	0
0	0	0
0	0	0
0	0	0
0	0	0
0	0	0
0	0	0
0	0	0
0	0	0

0	0	0
0	0	0
0	0	0
0	0	0
0	0	0
0	0	0
0	0	0
0	0	0
0	0	0
0	0	0
0	0	0
0	0	0
0	0	0
0	0	0
0	0	0
0	0	0
0	0	0
0	0	0
0	0	0
0	0	0
0	0	0
0	0	0
0	0	0
0	0	0
0	0	0
0	0	0
0	0	0
0	0	0
0	0	0
0	0	0
0	0	0
0	0	0
0	0	0
0	0	0
0	0	0
0	0	0
0	0	0
0	0	0
0	0	0
0	0	0
0	0	0
0	0	0
0	0	0
0	0	0
0	0	0
0	0	0
0	0	0
0	0	0
0	0	0
0	0	0
0	0	0
0	0	0
0	0	0
0	0	0
0	0	0
0	0	0
0	0	0

0	0	0
0	0	0
0	0	0
0	0	0
0	0	0
0	0	0
0	0	0
0	0	0
0	0	0
0	0	0
0	0	0
0	0	1
0	0	0
0	0	0
0	0	0
0	0	0
0	0	0
0	0	0
0	0	0
0	0	5
1	0	2
1	0	0
0	0	0
1	0	0
1	0	0
0	0	0
1	0	0
1	0	0
1	0	7
1	28	8
0	0	0
0	0	2
0	0	0
0	0	0
1	0	10
1	0	2
1	2	26
1	0	2
0	2	0
0	0	0
1	0	0
0	0	0
0	0	3
1	2	3
1	1	2
1	0	28
1	167	115
1	3	24
0	0	0
0	2	1
1	2	14
0	0	0
0	0	0
1	0	2
0	0	0
1	1	10
0	0	0

0	0	0
0	0	2
0	0	1
0	0	0
0	0	0
0	0	0
0	0	0
0	0	0
0	0	0
0	0	0
0	0	0
0	0	0
0	0	0
0	0	0
0	0	0
0	0	0
0	0	0
0	0	0
0	0	0
0	0	0
0	0	0
0	0	0
0	0	0
1	13	125
0	1	7
1	2	4
1	2	4
1	34	98
1	2	7
1	1	2
1	0	5
0	0	0
1	0	1
1	10	10
0	0	0
0	0	0
1	0	6
1	1	10
0	0	0
0	29	3
0	0	0
0	0	1
0	4	0
0	0	0
0	0	0
0	0	0
1	5	13
1	29	35
1	9	13
0	0	8
0	5	3
1	1309	198
0	0	0
0	0	1
1	260	262
0	0	0
1	0	0

1	0	0
0	6	12
1	140	125
0	0	0
0	4	0
0	0	0
0	0	0
0	0	0
0	0	0
0	0	0
0	0	0
1	0	0
0	0	0
0	0	0
0	0	0
0	0	0
0	0	0
0	0	0
0	0	0
0	0	0
0	0	0
0	0	0
0	0	0
0	0	0
0	0	0
0	0	0
0	0	0
0	1	1
0	0	0
0	2	0
0	0	1
0	0	0
0	0	0
0	0	0
0	0	0
0	1	1
0	0	0
0	0	0
0	0	0
0	1	3
0	1	0
0	2	3
0	0	0
0	0	0
0	0	0
0	0	0
1	1	1
0	0	0
0	1	0
0	0	0
0	0	0
0	0	0
0	0	0
0	0	0
0	3	1
0	0	0
0	2	0

0	12	6
1	316	313
0	0	0
0	0	0
0	0	0
0	0	0
0	0	0
0	0	0
0	2	2
0	0	0
0	0	0
0	0	0
0	0	0
0	26	3
0	6	70
0	0	0
1	7568	2549
1	165	1760
1	19576	18599
1	1847	286
1	105	848
0	7	12
0	7	46
1	3176	3055
1	569	1710
1	5568	2693
1	4775	1207
1	13721	15499
1	1933	25064
1	3270	1623
1	3104	449
0	115	185
1	101343	142305
0	343	229
1	1395	2022
1	76155	8610
1	5223	5246
1	23083	1162
1	8407	12562
0	3	4
0	28	308
1	4892	7529
1	6636	6076
1	73514	49371
1	1107	514
0	104	120
1	31	645
1	12290	14825
1	10640	5198
1	32139	11658
1	15301	19303
0	146	158
1	62063	53248
1	110302	3146
1	21135	11761
0	98	222
1	361	4452

1	2109	1458
1	20443	90324
1	1050	1429
1	22090	21918
0	80	487
1	5124	434
1	741	412
1	4024	4274
1	24214	10481
1	39641	63006
1	4089	578
1	6670	3766
1	10246	4637
1	10429	13507
1	48	44
1	8547	1226
0	21	50
1	34775	36226
1	14684	18856
1	22680	36471
0	2	6
1	13801	15273
1	5782	6053
1	41707	54868
0	148	2022
1	1744	2278
1	3270	2740
1	1621	731
0	0	3
0	0	0
0	1	30
0	0	0
0	2	0
0	13	35
1	6012	9620
1	58910	14765
1	1369	1426
1	257	3963
1	108554	80223
1	249012	130732
0	68	786
1	21331	40088
1	11244	13934
0	16	19
1	4214	6543
0	1	11
1	4474	4778
0	108	241
0	330	238
1	4219	3292
1	135	1510
1	13861	28459
1	7188	8047
0	1	52
1	121	1470
1	6275	3213
1	4193	4100

1	242	4017
1	13004	14459
0	8	15
0	4	7
0	7	62
1	4722	2954
1	35771	26275
1	935	14980
1	5348	2968
1	7151	6875
1	877472	1224303
1	17408	5100
1	1516	2117
1	10384	12746
0	26	47
0	2284	504
1	8371	15678
1	91875	19339
1	9071	4218
1	70	515
0	1	22
0	37	90
1	44117	1275
1	4172	6413
1	1451	1889
1	7455	3932
0	34	463
1	15379	21699
0	320	1001
0	2	11
0	5	3
1	339	4272
1	310	5475
1	11915	15506
1	20542	12726
0	1	10
1	30417	28933
1	840	213
0	6	10
1	55099	21390
1	30686	26530
1	21169	15007
0	40	279
1	12132	6930
0	64	405
1	6503	124479
1	93569	2715
1	27031	28788
0	0	0
1	22003	34221
0	9	68
0	263	221
1	220	2611
1	25017	519
1	10837	3826
1	4876	4456
1	1534	817

1	118	1034
1	34346	47410
1	24162	7457
1	132	2238
1	38923	923
1	1967	24505
1	4311	5014
1	1812	1941
1	13927	4818
0	2	24
1	3116	3013
1	6237	1941
1	2	9
1	411208	547853
1	16921	8781
0	0	0
1	1557	4562
1	57255	11341
0	59	61
0	0	0
0	19	37
1	267765	44675
1	145	2119
1	568	11005
1	5356	1455
1	34414	3750
1	210	2321
1	356	2488
1	120	225
1	175	311
1	199	1213
0	9	163
0	13	93
1	2291	2554
1	3585	5975
1	4236	1265
1	174	886
0	1907	1723
1	28045	14790
0	160	660
0	0	0
1	23141	21694
0	68	399
0	52	64
1	7801	6911
1	9710	3814
1	14105	17521
1	8537	3624
1	13599	24546
1	11456	8764
1	124183	201595
1	10650	10784
0	2	8
0	3	6
1	4857	7104
1	4053	5489
0	6	38

0	13	22
1	21624	6125
1	14701	2768
1	20932	32183
1	7737	3194
1	28365	9320
1	8757	2615
1	240	1743
1	26930	36714
1	3884	2356
0	23	238
1	28978	51380
1	1614	30157
1	19337	8446
1	476	3361
0	12	116
0	10	21
0	19	39
0	170	222
0	36	86
0	61	417
0	39	172
1	113598	160234
1	81240	115451
1	500	3877
1	150	1544
0	4	26
1	178	1778
1	41241	62179
1	2606	2107
1	3811	1763
0	7	18
1	2986	3149
1	12387	1720
1	1077	899
1	128	1070
0	51	292
0	70	533
0	185	1597
1	5965	3948
1	32907	53000
1	80648	102446
1	33310	53752
0	2	36
0	1	0
0	0	0
0	0	0
0	0	0
1	8068	8104
1	25722	32761
1	13968	1801
1	5335	382
1	3988	2481
1	2265	2337
0	0	0
0	21	192
1	46689	14175

0	2	23
1	65	1684
1	2671	10229
0	8	29
1	31248	2784
1	2225	5405
1	3870	3967
1	1854	1979
1	62008	69957
1	55106	3297
1	5351	2515
1	16437	9337
1	43118	5506
1	90552	101156
1	15939	20759
1	20072	8541
0	49	43
0	28	3
1	14617	23155
0	56	59
1	14659	4137
1	9057	7491
1	3270	1182
1	4450	3751
0	68	266
0	4	4
1	2619	604
1	3471	1389
1	2355	1519
0	17	148
0	0	27
0	728	2248
1	26581	24411
1	3192	5249
0	443	439
1	9417	1165
1	188	136
1	35843	22799
1	15526	16019
1	12862	6878
1	11944	1807
1	18132	1257
1	12075	18603
1	3964	2924
1	5118	1450
1	12880	13509
1	10489	1104
0	12	9
0	3	49
1	75589	106232
1	81209	120107
1	3141	6423
1	2175	2250
1	5604	1326
0	5	9
1	652	7921
1	1048	1175

1	1309	566
0	0	0
1	9585	9015
1	7873	5403
1	17281	6060
0	40	453
1	901	2522
0	34	0
0	7	13
1	14431	21840
0	0	0
0	0	0
0	2	16
0	26	51
0	8	5
0	1	28
1	10860	10857
1	4728	241
1	34946	18498
1	11200	2206
1	1574	22402
0	23	127
0	329	905
1	16200	19053
1	6711	9110
1	979	627
1	8640	8488
1	21839	22012
1	16131	4263
1	7218	2825
1	3487	1565
1	1458	352
1	425	290
1	405	8179
1	6144	882
0	35	120
1	19476	26909
1	18691	25340
1	1401	12387
0	14	71
1	7730	11346
0	5	12
1	155699	244788
1	18073	23117
1	1178	50
1	3118	5102
1	12687	19557
1	29513	29590
1	11281	22692
1	32693	19826
1	12084	10135
0	42	259
1	33089	7791
1	27944	20123
1	265	1470
1	5327	7957
1	3321	1825

1	274	4646
1	720	9622
1	21797	29389
1	10869	7645
1	702	1250
1	10780	1974
0	9	58
1	556	2478
1	11860	4009
1	8415	4753
1	16307	36637
1	60	74
1	3	19
1	95	77
1	10	21
0	70	611
0	0	54
1	2362	4276
1	14983	2367
0	5	109
1	3575	4910
0	6	11
0	8	12
1	154	1120
1	141	2603
0	197	127
0	6	14
0	20	41
1	12337	13721
1	7296	6913
1	657	12096
1	603	4474
1	104	551
1	29061	28523
0	1079	46
1	4223	1192
1	251	3133
1	20855	28588
1	10256	5013
1	13310	125793
0	375	285
1	17	377
1	3412	4166
0	49	207
1	863	2930
1	58137	47842
1	7827	3865
0	570	391
1	378	1666
0	0	0
0	0	0
1	12178	16669
1	42	13
0	70	489
0	20	40
1	40052	52379
0	4	6

0	3	25
0	22	188
1	49421	85066
1	70411	65065
1	14108	13240
1	7728	3317
1	15765	12609
1	7631	9147
1	13285	17118
1	10044	4364
1	9433	12244
0	269	1085
1	40813	50771
1	45624	83349
1	7912	6361
0	0	0
0	0	0
1	125920	59205
0	2	2
0	0	0
0	2	4
0	0	2
0	2	3
0	2	0
1	5585	4430
1	4789	5260
1	6207	18319
1	308	3925
1	8043	7639
1	6026	2870
1	10803	11085
1	4121	519
1	839	22950
0	3	15
0	1	11
0	5	5
1	3056	3378
0	0	5
1	9255	5694
0	17	27
0	21	30
1	1048	2945
1	7736	4191
1	33067	63309
0	0	0
0	0	0
0	0	0
0	0	0
0	0	3
0	0	0
0	0	0
1	551	8368
1	3042	4124
1	49007	19934
1	4222	3058
1	258	1997
0	8	17

0	15	191
0	6	3
1	4090	3847
1	8207	2131
1	167	3195
1	14916	28160
1	1182	11814
1	29817	32070
1	15685	14053
1	22474	914
0	4	14
1	37184	42144
1	6338	10220
1	19193	24813
0	131	101
0	1	3
0	27	47
0	42	29
0	61	22
1	9525	12053
1	77220	99626
0	4	59
0	14	11
1	4222	6174
1	14201	21832
0	30	124
1	752	12828
1	3089	4683
1	974	6500
0	118	18
1	189848	276441
1	26933	5607
1	34242	14626
1	17529	5206
1	22350	1590
1	20155	14911
1	194	2449
0	8	136
1	7959	1734
1	5107	2436
1	704	487
0	15	18
1	14988	1932
1	7214	464
0	16	67
1	116140	186427
1	62217	73978
1	16409	9266
1	11366	5162
1	413	5152
1	4693	3929
1	29160	37803
1	6663	8956
1	15702	8710
1	10450	272
0	15	14
1	9081	6664

1	13067	22013
1	3141	1618
1	16953	7733
0	152	161
0	182	171
1	39855	34298
1	1124	3512
1	112325	161557
0	2	2
1	131215	205372
1	36183	28119
0	0	3
1	19535	40632
1	7501	8059
1	19557	12034
1	4395	7123
0	0	0
1	36335	28572
0	8	23
0	8	3
0	2	65
0	0	0
0	249	326
1	55666	12329
1	2462	9562
0	45	750
1	4953	1620
1	9498	3330
1	80	191
0	0	0
0	9	132
0	3	13
0	602	175
0	37	32
0	5	18
0	48	121
0	38	8
1	484	27
0	10	9
0	6	4
0	1	8
0	2	20
1	29	560
1	485	434
1	60596	95243
1	7476	10392
1	12237	785
1	34407	30988
1	293	5049
1	23154	5240
1	4043	5623
0	4	13
1	52893	86971
1	7908	5585
1	65928	55800
1	1324	2299
1	756	1640

1	9185	2539
0	7	47
1	3897	6197
1	21110	32509
0	14	101
1	243	256
1	71640	110065
1	7816	2976
1	30098	25220
1	12962	18793
1	15705	1132
1	5640	4133
1	51819	28798
1	9396	1753
1	13885	18566
1	5750	2911
1	12798	8532
1	9450	1485
1	2528	212
0	145	343
1	57381	42440
1	7741	2456
1	783	300
1	13992	21227
1	19127	29755
1	21874	34633
1	34578	4628
1	16953	22572
1	18302	26661
1	8069	8846
0	0	0
0	0	0
0	2	23
1	49602	29057
0	0	2
0	29	17
0	0	0
0	1	0
0	31	106
1	121655	180550
1	134676	180570
1	85019	115917
1	7675	16229
1	6321	9935
1	6276	7883
1	8410	10067
0	88	68
0	194	121
1	4136	3780
0	564	1613
0	71	202
1	906	15104
1	1887	2496
1	357	7479
0	60	93
1	2599	2336
1	2258	2289

0	653	94
1	8302	8048
0	58	47
1	25549	4525
1	40724	44470
1	18476	31152
1	2699	2771
1	7250	12007
1	56673	17162
1	2677	3069
1	6772	3596
1	13258	9306
1	11635	17290
1	4390	6659
1	4401	6569
1	3229	635
1	34365	40909
1	6536	1335
1	1135	2309
1	29986	23384
1	7813	12145
1	8136	3638
1	6968	5643
1	28186	15186
1	6857	6886
1	34	161
1	7640	2320
1	111984	54704
1	33071	44973
1	11855	16688
1	16535	21944
1	9523	11506
1	2106	2218
1	7863	2274
0	0	1
1	1327	1468
0	1	0
1	3152	3075
0	0	0
1	65884	88926
0	0	0
0	19	27
0	3	8
0	7	9
0	4	5
1	4983	2654
0	154	1321
0	1	5
1	6400	8306
1	1861	2042
0	65	40
1	3853	1030
1	9282	2525
1	12691	14634
1	5738	7837
1	12454	10541
1	685	548

1	21819	28856
1	3370	2182
0	4	2
0	3	0
0	12	2
0	0	5
1	17356	23596
1	139	502
0	121	1118
0	2	3
1	4428	3005
1	13657	18917
0	21	17
0	22	20
1	13913	2731
1	2091	4196
1	9393	9372
1	8362	5861
1	29057	12559
1	8791	6665
0	0	0
0	0	0
0	0	0
1	9265	2578
0	30	81
0	0	0
0	30	81
1	37392	16266
1	15659	26115
1	12427	18189
1	16401	9143
0	0	31
1	8264	2844
0	33	377
1	14238	19471
1	10614	3117
1	28084	41465
1	2882	2897
1	7928	15966
1	24723	6477
1	607	98
1	4845	2502
1	5828	4890
1	7503	1090
1	28897	37614
1	7492	2545
1	7260	4457
1	13407	19836
0	4	3
1	109894	47113
1	14573	4566
0	0	0
1	9844	13617
1	8537	1694
1	18984	501
0	0	2
1	1774	4698

1	13318	6686
0	0	28
1	119	213
1	250	200
1	1774	1775
1	4499	3807
0	3	2
0	2	0
0	1	9
1	66064	91700
1	4180	5916
0	48	73
1	2361	4302
1	5970	10889
0	4	12
1	826	851
0	3	50
0	7	8
1	23889	7611
1	32401	10997
1	57513	96067
1	0	0
1	0	0
1	0	0
1	0	0
1	0	0
1	0	0
1	0	0
0	0	0
1	0	0
1	0	0
1	0	0
1	0	0
0	0	0
1	0	0
0	0	0
1	0	0
1	0	0
1	0	0
1	0	0
1	0	0
1	0	0
1	0	0
1	0	0
1	0	0
1	0	0
1	0	0
1	0	0
1	0	0
1	0	0
1	0	0
1	0	0
1	0	0
1	0	0
1	0	0
1	0	0
1	0	0

1	0	0
1	8619	9998
1	12794	11070
1	10550	12488
1	5611	8597
1	7314	9927
1	12988	4567
1	16743	20749
0	3	120
1	49114	42314
0	47	3
0	7	49
1	6263	3553
1	11110	7119
0	7	51
0	330	301
0	25	207
0	1	27
0	4942	134
0	11	16
1	158	2331
1	34058	51927
0	2	2
0	1	14
1	16568	26814
1	1142	661
1	3002	3371
1	14077	11820
1	141	877
1	19222	1055
1	5291	3818
0	1	29
1	12651	15359
1	59137	84867
1	9627	2600
1	1618	2472
1	1049	1449
1	10476	1590
1	138775	203042
1	9849	8777
1	12845	18794
0	83	113
1	35686	35234
0	18	24
0	0	0
1	10976	6333
0	75	927
1	16334	4824
1	45917	68902
1	108	499
1	18071	5960
1	5943	8853
1	42	256
1	5890	4884
1	348	424
0	4	13
1	19536	1603

0	13	3
0	12	3
0	2	2
1	6995	2670
0	0	0
0	0	0
0	0	0
1	11517	25696
0	7	41
1	157	427
1	23749	7488
1	6171	1352
1	15049	27388
0	11	114
1	10547	5639
1	1524	634
1	1828	1768
0	1	47
0	0	2
0	4	4
1	38311	4586
1	21154	33996
1	965	573
1	6672	9766
0	8	14
0	0	0
1	358	477
0	8	8
0	0	5
0	35	344
1	5713	2019
0	1	5
1	18141	26681
1	29058	41592
1	6296	836
1	7333	5502
1	49526	11253
1	6417	4919
1	3947	2450
1	2527	2034
1	13854	10186
1	27547	22871
1	20645	5025
1	7383	3153
1	29885	49937
1	13237	3353
1	2071	1466
1	2360	648
0	0	0
0	27	52
1	17644	14452
1	2810	3915
0	0	0
1	10718	8603
1	22883	14198
1	3665	780
1	4580	5067

1	11476	1967
0	11	131
1	44975	54760
1	12218	12056
0	3	21
1	44332	60989
0	22	161
1	10013	15290
0	42	147
1	27542	21651
1	9613	5574
1	8589	2234
1	1641	3432
1	18121	3205
1	3269	2545
1	724	17
0	86	458
1	1493	215
0	32	35
0	14	103
0	4	48
0	5	5
1	2613	291
0	0	0
0	0	0
1	18943	8266
1	9988	10497
0	360	4406
1	25465	36141
1	8609	1381
1	6441	3592
1	3752	2606
1	19369	12001
1	17686	8147
0	70	69
1	13095	18977
0	179	45
1	15951	10510
0	0	0
1	1951	274
1	10884	5629
1	244	4435
0	104	1009
1	13340	3149
1	2113	139
1	4754	4378
1	2283	414
1	4783	3425
1	7397	3606
1	271	100
1	8187	10348
1	28908	52337
1	9797	6054
1	2724	869
1	3893	1683
1	6134	7348
1	10718	4961

1	1560	2353
0	12	47
1	27275	40690
1	4301	4834
1	8816	4699
1	19207	18454
0	9	58
1	9695	24903
1	12799	2414
1	6879	7321
1	20570	16583
1	23165	10996
1	16030	1918
1	8823	47461
0	13	24
1	7683	6068
0	37	32
1	10167	1451
1	5220	1809
1	17377	12573
0	33	273
1	123925	71403
0	57	145
1	2744	2303
1	245	305
1	10040	13113
1	9940	12642
1	15319	15159
1	2381	2408
0	0	0
1	17247	5558
1	13512	18172
1	12582	7339
1	434	303
1	24282	13240
1	21746	24482
1	9925	10226
1	3199	1790
0	22	45
1	24987	35482
0	4425	1949
1	23663	38548
1	594	943
1	12904	4032
1	1236	8607
0	10	4
0	4	23
1	1207	138
1	236	50
0	0	0
0	0	0
1	22385	34331
1	7253	9343
1	22976	30063
1	6578	11332
1	11455	12553
1	2779	1107

1	2481	2833
0	5618	1692
0	0	4
1	113135	182843
1	3977	1030
0	0	0
0	0	0
1	8719	7491
1	11329	292
1	18858	13678
1	4688	4263
1	25851	11257
1	1024	2149
1	84135	110492
1	17746	21980
1	16850	20449
0	5	42
1	3572	3155
1	30321	9758
0	10	78
0	0	0
1	11905	4893
0	6	10
1	1966	1736
1	34015	54405
1	7574	13392
1	11890	15708
1	11437	8850
1	3994	328
0	3	36
1	7819	7415
1	3701	1846
1	20957	13177
1	26421	9358
1	67295	122692
1	6128	3812
1	2774	7915
1	78762	110685
0	1	18
1	19000	10362
1	6431	5866
1	4348	763
0	0	1
1	8978	10790
1	81373	119160
1	429	7034
1	10910	17296
1	71640	83860
0	56	445
1	20320	3615
1	45728	8976
0	0	6
1	671	12477
0	14	200
1	3517	1414
1	944	643
1	4407	5878

1	21499	16016
1	8740	9739
1	4896	1618
0	102	271
0	1	20
1	3430	3595
0	73	61
1	654	656
0	10	5
0	1	27
0	66	56
1	5712	3187
1	5326	1370
0	0	2
1	9573	8938
1	6185	628
0	68	726
1	7146	4099
1	10006	4295
1	9787	10907
1	13412	11456
0	512	90
1	14921	6365
1	29213	48488
0	1	6
0	36	23
0	0	0
0	0	5
0	0	0
1	10100	3825
0	61	440
1	3409	753
0	1	0
1	2511	1178
1	7804	2907
0	8	105
0	7	13
0	23	34
1	3312	3695
1	149617	230915
0	50	345
0	0	6
1	15943	24701
1	27163	9065
1	4976	953
1	8473	2562
1	2485	2181
1	6504	7949
1	7206	2440
1	16396	5785
1	18575	11567
1	1019	1574
1	9915	10504
1	7524	2469
1	1968	916
1	20010	27042
1	2427	1726

1	12839	17221
1	2721	4613
1	759	1562
1	13752	13656
1	9813	2372
1	11377	1961
1	332	972
1	1032	1912
1	7532	13809
1	464	608
1	9900	10218
1	22983	18137
1	7814	1558
1	2480	3041
1	750	749
1	4309	695
1	5864	6166
0	7801	350
1	18514	21498
1	4087	2115
1	47792	41389
0	103	80
1	5000	5351
1	76161	137882
1	6162	328
1	2453	2936
1	19893	20027
1	14518	16757
1	2608	4054
1	3349	7458
1	4977	6315
1	1307	1524
1	103	1762
0	229	409
0	4	4
1	14714	1394
0	4	29
1	8447	588
0	94	196
0	20	91
1	19225	9925
1	4844	3611
1	62904	15977
1	7948	8078
1	23951	52015
1	2405	2940
1	20450	5918
1	20212	23590
1	19288	19868
0	1	0
1	2623	1011
1	4734	810
0	6	27
1	5435	7041
1	8974	10048
1	21772	10959
1	14691	5883

1	6110	4265
1	7184	3234
1	9244	2329
1	44	429
1	10225	4080
1	20918	22917
1	33007	7146
1	13943	1905
0	31	70
0	2	15
0	8	1
0	28	25
1	4800	2121
1	6685	9748
1	9562	2712
1	5336	6440
1	6837	3758
1	43133	3795
1	3775	546
1	1235	566
1	2876	2149
1	10327	12647
0	85	114
1	9892	6343
1	25778	26797
1	16242	7351
1	2371	545
1	5884	4613
0	47	88
1	3166	243
0	34	107
1	4218	1911
1	4044	2464
0	2	11
1	594	8944
1	7839	4077
1	7462	9970
0	91	721
1	2807	3467
1	2788	3766
1	3680	1735
1	12162	11431
1	2636	3007
1	310	6267
1	2723	5093
1	15088	19095
1	21070	32879
0	16	165
0	3	4
1	13061	5914
1	1756	2850
0	64	137
1	7866	3603
1	22604	12042
1	3796	3855
1	23090	10360
0	4225	656

0	45	119
1	10663	7315
1	543	527
1	8645	3854
1	13965	5345
1	22526	33793
1	15559	6577
1	8492	10286
1	12575	14882
1	6164	9387
1	84739	126101
0	5	9
1	3136	3260
1	11077	8782
1	5401	10160
1	2568	3564
1	10540	14316
0	90	511
1	4901	368
1	4957	778
1	3151	4582
1	4512	1136
0	23	26
1	3657	3375
1	596	638
1	7003	1363
1	90496	31666
0	2	56
0	11	71
0	4	27
1	3363	3895
1	11074	17084
1	3705	909
0	6	25
1	6656	4506
1	5008	4018
1	16297	24594
1	11980	1957
1	5091	1110
0	5	36
1	3670	2662
1	6006	5489
0	0	6
1	20320	8355
1	9696	15052
1	12561	3090
1	8758	9128
1	4672	8824
0	0	0
1	1754	579
0	4	21
1	246	408
1	6370	5724
1	120	169
0	10	17
0	12	47
0	15	27

1	1161	2374
1	9875	17148
1	18886	25864
1	1927	1070
1	3340	3644
1	5606	6290
1	594	869
1	9223	7967
1	24284	35672
0	0	0
1	2130	1839
0	2	12
0	5	107
0	36	184
1	16343	16596
0	37	333
1	5464	568
1	1637	1866
1	1046	603
1	134	324
1	353	325
1	38322	7389
1	3083	1009
1	8926	13211
1	18969	29514
1	9493	13856
1	38677	23256
1	1727	2552
1	20715	18539
0	6	5
0	108	66
0	2	1
1	9069	9294
1	14465	28337
1	14756	9181
1	2654	653
0	0	0
1	6636	2071
1	195	1935
1	379	473
0	24	341
1	12300	9389
1	15273	6855
1	6998	6978
1	3075	2747
1	2229	4147
1	18331	24762
1	5257	1154
1	22002	26006
1	10965	9914
1	3609	7473
1	7375	2708
1	23228	22814
1	2516	2754
1	1331	440
1	2678	1631
1	6590	9561

1	7745	4773
1	1941	1229
1	5870	7345
0	85	109
1	16706	15057
0	5	5
1	2122	903
1	28156	30916
1	7484	8799
1	10373	10338
0	3	17
1	8499	1432
1	7691	8862
1	16012	10038
1	3362	1607
1	8925	5897
1	304	3369
0	0	0
0	0	0
1	5477	2064
0	323	295
1	4546	6210
1	5544	4809
1	53653	18589
1	20566	2413
1	2628	2467
1	1545	1836
0	0	0
1	15773	1817
1	8051	7208
1	11076	1882
1	1724	1060
1	5342	5497
1	7557	10777
1	416	7410
0	66	75
1	11343	17405
0	270	1580
1	6397	8135
0	0	1
1	9026	8731
1	18436	22168
0	13	61
1	7072	11101
1	7786	1760
1	16603	14274
1	7295	6905
0	5	22
1	3373	4322
1	16075	6209
1	3618	157
1	11672	6617
1	1497	869
1	13153	15101
1	4417	4123
1	891	12879
1	2677	4443

1	7938	11543
1	4536	32184
1	25247	13543
1	3344	4135
1	119172	172671
1	4887	7094
1	3062	1829
1	19118	19222
0	0	0
1	1139	1287
0	0	0
0	0	0
1	8606	10343
1	3170	1268
1	7150	6708
1	51102	57186
1	26238	34762
1	529	801
1	39724	8965
1	13094	11709
1	19645	6758
1	20525	2813
1	2290	3382
1	14736	2030
0	6	2
0	29	68
0	204	312
0	2	38
0	0	0
1	9426	12267
1	6907	6423
1	2406	1815
1	51560	59986
1	3239	3013
1	5434	8550
0	32	10
1	1720	566
1	14455	20190
0	1825	142
1	24641	34088
1	3518	2022
1	13714	5624
1	4065	1474
1	2672	495
1	3704	2805
1	3091	335
1	1630	800
1	2657	3009
1	6035	5691
1	14939	3549
1	16773	22956
1	7277	2330
0	0	0
1	6866	8380
1	3589	1055
1	8679	4727
0	109	154

1	2329	1614
0	51	150
1	6355	1077
0	3	14
1	5456	7040
1	8903	10110
1	12178	21688
1	4821	3402
0	14	129
0	0	1
1	9499	11701
1	20692	21139
1	736	839
1	23153	18884
1	16050	21212
1	12640	4721
1	19990	3918
1	4453	4596
1	2985	2398
1	48	309
1	15650	54560
0	0	2
0	31	221
1	8234	8383
1	7434	3292
1	15939	8952
1	45370	9653
1	3876	3419
1	15052	4132
1	38695	57314
0	28	10
0	83	117
1	2330	434
1	15569	888
1	4776	1196
0	0	0
1	3904	1311
0	10	18
1	5278	2254
1	22903	12058
1	30246	42918
0	306	332
1	29517	7211
1	3934	7125
1	10586	12151
1	6359	7257
1	10670	4570
1	5613	1100
1	16236	11344
1	2843	3447
1	10041	15682
1	8363	9748
1	17668	2299
1	11924	15313
1	12095	9428
1	17654	21295
1	1996	158

0	0	0
1	5338	4599
1	7042	2127
1	3724	1082
1	15105	5167
0	4	22
0	1	1
1	2766	2053
1	3772	705
1	40568	50021
0	190	170
1	3010	1745
0	0	39
1	17349	9626
1	20777	1006
1	6926	4075
1	4320	3687
0	42	27
1	6707	9616
1	39362	51688
1	264	237
1	7064	10756
1	8401	3352
1	871	1923
1	8731	5067
1	4194	3473
1	10209	3496
1	10135	8209
1	7168	2262
0	10	19
1	3168	846
1	1188	778
1	2410	3324
1	3876	5497
1	4434	4968
1	2771	2178
1	2903	4094
1	5854	7246
1	5467	2656
0	17	30
1	4218	1386
1	70	465
1	3301	2389
1	114	216
0	1	26
1	24310	21611
0	1	2
1	876	722
1	13338	9865
1	3262	1861
1	6034	7019
1	4876	13572
0	7	3
1	194	435
0	1	14
1	14397	9029
1	1899	782

1	26279	8529
1	9427	16816
1	23592	30696
1	8181	2643
1	6746	5689
1	30672	24919
1	10824	13550
1	30695	18198
0	1780	1268
0	2	9
1	1058	15041
0	0	1
1	6126	7177
0	0	0
0	1	42
1	6958	4026
0	10	9
1	1723	2015
1	5576	3566
1	2388	1521
1	1439	1885
1	6016	11726
0	25	28
1	6743	5870
1	2854	2073
1	1324	857
1	26961	28455
0	0	0
0	0	0
0	0	0
0	0	0
0	9	46
1	7272	1685
0	113	271
1	11009	675
1	225	378
1	7578	8293
1	2034	1366
1	5695	3458
0	100	211
1	5346	5876
0	0	0
0	0	0
0	20	12
1	8273	1174
1	13138	16407
1	42022	70271
1	10322	8058
1	19080	12861
0	18	38
1	9309	9405
1	2486	2756
1	6518	9397
1	1084	2329
1	6980	2190
1	5323	3764
1	410	953

1	2927	7464
1	2789	1188
0	0	12
0	0	0
1	2980	505
0	11	11
1	792	13298
0	0	5
1	10384	7060
1	4124	5830
1	1778	2645
0	4	5
0	5	1
0	22	288
1	10743	15219
1	6689	2776
0	38	22
0	30	16
0	122	100
1	5257	6628
0	374	189
1	1061	728
0	1	20
1	12595	1259
0	0	0
0	0	3
0	219	313
1	12403	19838
0	0	0
1	8403	4639
0	28	234
0	14	20
0	24	37
1	8901	506
1	4940	1551
1	15823	10349
1	4626	2260
1	1493	1547
1	18780	8689
0	0	13
0	0	1
1	11380	12676
0	16	33
1	18567	3654
1	22971	28199
1	29520	18695
1	18141	4196
1	29487	30021
1	25345	9089
1	15196	6339
1	22627	27080
1	18570	4077
1	41791	67121
1	64845	119117
0	6	0
1	8644	2366
0	26	130

1	139	266
1	4493	5270
1	25380	5426
1	6245	1340
0	0	0
1	1801	1326
1	10329	17474
1	1058	689
1	4546	3210
0	0	0
0	0	0
0	0	0
0	0	0
0	55	21
0	38	22
0	0	2
0	0	0
0	0	0
0	247	118
0	7	1
0	0	0
0	0	0
0	0	0
0	5	14
0	7	27
1	10	11
0	0	0
0	2	5
0	4	0
1	1617	940
0	2	10
0	1	39
0	0	0
0	0	0
0	1	0
0	0	0
0	2	8
0	52	41
0	2	19
1	2276	2235
0	1	9
1	1121	2273
1	678	584
1	1034	829
0	0	2
0	3	6
0	156	248
0	0	2
0	3	13
0	0	0
0	1	0
0	3	2
0	0	1
1	1181	1155
1	10807	14615
1	3320	2677
0	4	32

0	2	0
1	4879	2767
1	13078	5413
1	10192	3091
1	1748	2390
1	13091	6908
1	3577	3342
1	353	899
1	8512	6798
1	2609	1160
0	0	0
0	0	1
0	0	22
1	2640	5595
1	4214	2980
0	36	233
1	2055	729
0	98	892
1	343	1500
0	41	207
1	5315	5290
0	21	199
1	21698	12628
1	11377	5812
0	6	28
1	1795	321
1	2656	4392
1	1839	1607
1	796	17085
1	6976	659
1	12902	13672
0	689	343
0	31	134
1	9786	11459
0	0	0
0	0	0
1	11007	786
1	24973	10251
1	2896	1312
0	0	1
0	0	2
1	6654	3475
1	13814	1635
0	0	0
1	143978	223164
0	0	2
1	3188	822
1	3741	780
1	779	709
0	4	32
1	2149	865
0	21	73
1	68	62
1	6366	6897
1	43	231
0	38	93
0	0	1

0	0	3
1	974	758
0	1	1
0	0	0
0	8	14
0	12	160
0	0	3
0	2	18
0	348	160
0	4	0
0	10	15
0	124	48
0	7	4
0	25	12
0	4	2
0	2	3
0	28	20
0	343	32
0	23	52
0	6	9
0	0	3
0	0	2
0	1	5
0	3	11
1	3074	2402
1	2853	3636
0	0	9
1	13810	11045
1	2607	1807
1	4746	2598
0	0	0
0	0	2
0	0	0
0	0	0
0	0	4
0	1	3
1	21895	3259
0	0	5
1	5185	628
0	30	17
0	11	14
1	7249	7627
1	505	707
1	4606	2532
1	284	149
1	3658	2040
1	3534	2992
1	7344	10548
0	0	1
0	23	21
0	0	2
0	0	4
0	19	6
0	0	1
0	0	3
1	17565	400
1	5208	2155

1	14972	6266
0	0	0
0	11	4
0	0	2
1	62	380
0	40	136
0	41	65
0	46	54
1	6173	9026
1	6932	6053
0	25	35
1	28310	33579
1	3928	4430
1	41900	73438
0	39	466
0	1	26
1	31	210
1	2695	1158
1	12213	783
0	83	920
0	263	129
0	0	3
0	10	40
0	2	2
0	0	0
1	323	180
0	0	1
1	7235	2009
1	375	439
1	12485	1427
1	1169	988
0	18	157
0	1	3
1	7226	7428
0	50	238
0	13	16
0	5	9
0	8	53
0	26	268
0	2	6
1	3710	545
0	6	2
1	1342	697
0	2536	187
1	53	60
1	3299	1383
1	2436	1080
0	12	25
1	4495	1390
1	6876	9880
1	2909	1745
0	11	21
1	13219	1030
0	11	3
1	2228	2414
1	13837	6989
1	4061	2444

1	97	39
1	15873	12740
1	574	248
1	26939	10752
1	3111	506
1	1943	2040
0	33	86
0	29	172
1	24962	26624
1	16804	30142
1	14415	4986
1	2020	1413
1	196	3202
0	2	17
1	7313	1890
1	7670	3426
1	1924	987
1	4854	1790
1	3427	3395
1	3	26
0	9	39
1	8588	3198
1	2147	666
0	431	99
1	4231	712
0	1	45
1	631	8974
0	0	0
1	4981	5431
0	8558	1957
1	42307	56183
1	1899	575
1	2287	2265
1	6454	5469
0	11	76
0	1	0
0	4	3
0	0	1
0	1	0
0	1	2
0	2	1
0	0	0
0	0	3
1	10134	3128
1	4370	1172
1	2065	1716
1	5	13
0	0	0
0	0	1
0	6	9
1	6105	5289
1	5683	4327
0	0	5
0	40	30
1	7085	2663
0	12	35
0	0	10

0	0	0
1	5575	7828
0	2	63
0	2	11
1	9070	7053
0	101	405
0	10	38
1	830	972
1	8769	1691
1	4222	89
0	0	0
0	3103	253
0	1119	134
1	2291	132
1	4380	808
0	75	155
1	20377	8974
1	2418	439
1	2539	1140
0	25	84
1	2248	1742
0	21	22
1	4913	5861
1	14965	22134
1	9169	14619
1	10447	3103
1	26015	32957
0	1772	27
0	110	114
0	27	20
1	35244	34072
0	0	1
0	2	2
0	1969	183
1	6616	7465
1	1318	1936
0	0	14
1	2635	3377
1	142	138
1	1941	655
1	6810	6809
0	43	24
1	4688	3170
1	5890	9542
0	2	12
1	68301	8414
1	22477	5714
1	2618	1526
1	25445	13740
0	2	2
0	1	3
0	1	10
1	156	167
1	11	64
1	7004	3708
1	31619	55658
0	765	169

1	12072	10787
1	3627	3070
1	17619	20400
0	3	9
0	7	5
0	0	2
0	0	0
0	0	1
1	5037	800
1	6384	1993
1	6073	2711
1	1156	181
0	129	69
1	1116	384
0	880	468
1	998	587
0	5	7
0	3	15
0	5	18
0	0	0
0	1965	356
0	10	31
0	1	2
0	217	91
0	0	0
0	0	0
0	0	2
0	0	19
0	179	2872
0	44	76
0	14	30
1	1816	706
1	9891	2184
0	19	30
1	12420	19818
0	22	78
0	2	0
0	0	0
0	4	27
0	11	24
0	9	82
1	5719	2469
0	0	0
1	16334	6697
1	8501	2601
0	11	94
1	1563	1029
1	3056	1485
0	0	7
1	8984	1084
0	52	29
0	0	30
1	1843	570
0	9	119
1	6351	9644
0	3	18
1	11101	5348

1	15413	8355
1	3608	3388
0	0	2
1	6377	2552
1	41669	73129
1	11041	7100
1	14028	7030
0	5	6
0	11	13
0	38	16
0	0	0
0	247	95
1	16033	6340
1	3335	1038
1	4366	677
1	386	468
0	0	3
1	3281	2017
0	177	9
0	6	39
1	869	794
0	0	2
0	0	7
0	42	45
0	0	1
0	0	0
1	8363	1989
1	5842	3060
1	2749	4192
0	0	1
0	0	9
1	1906	1468
1	1473	349
0	6112	625
1	3718	491
0	1	1
0	0	0
1	4412	565
1	9574	2665
0	1	3
0	0	0
0	0	0
0	0	0
0	0	0
0	0	0
0	0	7
0	3	2
1	11016	11388
1	140	57
0	45	49
1	2351	937
0	0	2
1	4978	1527
1	13796	12422
0	85	154
0	16	147
1	1302	4901

1	1583	1924
1	3321	1097
0	0	2
1	79	63
1	25887	26662
0	1	1
0	0	0
1	4205	5518
1	11443	6868
1	2005	2805
1	9515	10097
1	2690	572
0	104	84
1	12578	5921
1	12091	4624
1	2422	2695
1	4758	2209
1	30588	48265
0	1	5
1	10268	3950
0	16	26
0	0	0
0	19	6
0	0	0
0	63	48
0	109	75
0	0	3
0	2	4
0	0	6
0	4	15
0	0	4
0	0	0
0	0	1
1	1736	2369
1	1829	769
1	862	173
0	23	25
0	4	22
1	5423	1076
1	13652	3397
1	2699	1616
0	2	2
0	7	7
0	0	4
0	25	28
0	20	10
0	22	146
0	127	485
0	17	261
0	0	1
0	662	110
0	0	0
1	588	1124
1	69907	10549
0	24	271
0	1	12
0	4	6

0	161	165
0	3	5
0	0	0
1	3191	3163
1	1391	1788
1	2266	3115
0	3	5
0	19	20
0	12	18
0	74	212
0	43	30
0	468	47
1	2916	504
0	15	19
1	8697	4647
1	4737	4507
1	1048	1475
1	12028	14643
1	3586	1180
1	3629	768
0	0	6
1	3038	553
1	1470	1786
1	1679	1192
1	1629	2540
1	3978	2179
1	4090	839
1	1153	941
0	25	54
1	1907	1304
1	4498	862
1	14450	13332
1	3865	783
0	4179	613
0	0	8
0	24	261
1	8539	6812
1	1280	226
0	2154	141
0	5	5
1	598	483
1	9519	5177
1	12009	10416
1	2363	252
0	9	42
0	0	0
1	13131	12120
1	2313	2656
1	935	2586
1	5197	4287
1	7195	8372
0	44	19
1	7461	2305
0	2	5
0	7	5
1	5073	7507
1	4547	500

1	9016	4802
1	994	526
1	11505	15489
0	18	19
0	8	15
0	3	10
0	967	79
1	15772	1208
0	1242	237
1	2691	1301
0	93	109
0	4	13
1	6548	6909
1	7930	10618
0	1	3
0	0	14
0	0	0
0	3	4
0	13	13
0	1	9
0	3	28
0	16	9
0	0	0
1	3591	1887
1	6755	8879
1	2588	1081
1	12036	11491
1	5104	3538
1	3267	511
1	1858	571
1	3104	253
0	46	208
0	79	597
1	2381	258
0	12	36
1	1000	996
1	3282	3341
1	7331	8918
0	0	0
1	2367	2411
1	503	1668
1	8985	4286
1	22772	20527
0	10	226
0	0	0
1	32093	34294
1	3851	3072
1	23906	610
0	0	13
0	3	7
0	0	0
0	0	1
0	0	0
0	1	3
1	8593	4385
1	3484	2510
0	4	2

0	0	0
0	9	4
1	11227	2978
1	13418	14422
1	17	132
1	13220	6121
1	4893	1295
1	3467	810
1	7417	5972
1	3441	4332
0	600	52
1	7660	5922
1	3421	1739
0	2	3
0	1	12
1	321	149
1	2489	995
0	10	38
0	1	2
1	8305	9434
1	3842	1757
0	173	438
1	7130	5242
0	0	9
0	0	0
0	43	356
0	28	454
0	5	5
1	3698	5188
0	261	73
0	630	91
0	1	0
0	0	2
0	2892	94
0	1	5
1	6952	10584
1	4669	4586
0	0	1
0	0	0
0	7	87
1	3143	4197
0	8	19
1	4468	6011
1	1743	3670
0	16	49
1	5054	1681
1	3446	1258
1	3648	1325
1	1096	41
1	7577	10449
0	8	5
0	19	15
0	1	2
0	0	0
0	6	8
0	3	6
1	6750	1814

1	8556	2986
0	34	85
0	7	15
0	2	5
0	44	80
0	14	243
0	17	11
0	13	156
1	4737	4882
0	21	234
1	2799	1236
1	9401	11578
0	1586	2068
1	35275	6937
1	2001	1129
1	18350	2113
0	0	2
1	53267	49148
0	234	79
0	138	773
1	3265	2426
1	5249	5767
0	0	0
0	130	54
1	89	395
0	19	188
1	31139	28811
0	11	110
1	2209	1917
1	3172	1356
1	5137	1750
1	7087	3754
0	5	56
1	10297	11087
1	3590	1003
1	1752	1078
1	34084	39221
0	2	3
0	10	31
0	0	9
0	18	12
1	13877	15461
1	2353	253
1	5491	4796
1	3018	303
0	208	142
1	1663	559
0	4	79
0	0	0
0	1	18
1	3910	729
1	19519	13912
0	2	1
0	1	1
0	8	6
1	5828	7998
1	3484	520

0	18	6
1	2698	1856
1	15722	15053
1	13659	7035
0	4	4
0	0	10
0	0	1
0	1	1
1	0	0
0	0	1
0	2	0
0	13	100
1	4112	1733
1	7517	12940
1	162	385
1	23210	30123
1	3710	917
0	3	4
1	1514	2119
1	2102	1932
0	0	1
0	0	0
0	0	1
1	4973	5127
0	287	141
0	10	7
1	11196	16172
1	7099	7736
1	5019	974
1	2103	355
1	10530	2872
1	2825	1301
1	7380	2715
1	1918	929
0	2	2
0	10	91
0	1	0
1	3181	719
0	0	0
1	4713	1182
0	4	7
0	14	61
1	1325	743
1	779	724
1	3390	3786
1	4657	4653
1	4472	1835
0	14	23
1	6252	4566
1	3197	2447
0	13	204
0	3	52
1	963	3018
0	0	0
0	8	7
0	6	125
1	4170	4597

1	5600	8425
1	132	1778
0	158	130
0	802	65
0	9	58
1	938	804
1	11792	19775
1	4167	418
1	3401	2422
1	201	478
0	2	3
0	2362	282
0	2	0
1	1833	2462
0	464	197
0	73	70
1	1290	1132
1	10714	17751
1	1235	740
1	1339	931
0	8	2
0	0	4
0	22	48
0	0	1
0	718	1085
1	5262	6532
0	16	145
0	1	12
1	1204	1767
1	1547	366
1	7248	9413
1	3848	1616
1	3248	2443
1	2358	699
0	28	35
0	0	1
0	4	1
0	0	0
0	0	0
0	0	1
0	0	0
0	231	112
1	1782	1260
0	0	8
0	3	7
1	1596	871
1	2355	558
1	4717	5050
1	768	1047
0	0	0
0	1	0
1	12721	7717
1	3768	1478
1	11026	2338
1	868	81
1	4039	977
1	6406	5642

1	8127	1405
0	1	0
1	13944	21667
1	3267	3065
1	4666	3459
0	18	13
1	3308	4672
1	829	493
1	1618	201
0	0	2
0	0	0
0	0	0
0	0	1
0	0	0
0	25	53
0	21	214
1	6607	7885
1	447	577
0	8	4
0	7	3
0	0	0
1	14	38
1	2693	3073
0	0	1
0	0	0
0	0	0
0	0	0
1	11407	9726
1	5400	7977
1	10054	45104
0	0	0
0	0	0
1	6965	9002
1	4340	6382
1	6786	6586
0	2	13
0	0	1
0	2	30
1	24212	1410
0	0	0
0	0	5
0	11	8
0	1	1
0	0	0
0	0	0
0	0	0
0	1	0
0	5	70
1	6859	2028
1	1081	1209
1	11437	718
1	2716	1366
1	1447	1804
0	81	263
0	6	134
1	4358	2913
1	7027	8710

1	10681	16741
1	204	580
1	3888	3280
0	1	21
1	14303	3375
1	1964	872
1	1305	556
0	14	45
0	0	1
0	0	0
0	8	71
0	82	118
1	1271	693
0	7	0
0	6	4
0	0	1
0	6	88
1	2801	3423
0	50	97
0	0	0
1	11810	1321
0	2	5
0	3	4
0	0	1
0	21	53
1	3311	1651
0	0	0
0	1	2
0	0	0
0	2	2
0	12	13
0	11	2
0	3	2
0	0	0
1	4290	3725
0	3	19
0	58	152
1	3909	592
0	2	1
0	4	1
0	0	1
1	2724	943
0	0	1
1	36	721
0	1967	367
1	1676	2087
0	5	77
1	657	1124
1	5479	2007
1	5185	1605
1	2555	2774
1	1936	1309
1	1769	605
0	0	12
0	0	0
0	0	3
1	2423	21712

0	2	7
0	0	0
0	7	13
1	261	787
1	5993	5716
0	6	122
1	8495	5473
1	321	3539
1	6174	386
1	3122	4092
1	2744	1129
1	4887	5371
1	5771	4905
0	15	22
0	0	4
0	1	18
1	1227	542
1	2906	621
1	1688	582
1	5903	1543
1	4323	4228
1	4809	3206
1	3763	881
1	2230	2178
1	5675	2355
0	0	0
1	8273	11687
0	0	0
1	7335	12858
0	0	0
0	39	141
1	2189	507
1	6738	483
1	1133	1306
1	11703	2989
1	5507	2217
1	1516	2150
1	3039	3819
0	4	6
0	6	10
0	5	12
0	0	0
0	1	6
0	0	5
1	11202	8550
0	1	7
1	24752	16032
0	2	62
0	0	20
0	80	91
1	1115	454
1	22	147
1	2012	1001
1	2857	2360
1	9791	13400
1	5327	941
0	0	0

0	92	38
1	100	921
1	2547	16580
0	2	30
0	0	28
0	0	0
0	0	3
0	4	22
0	0	6
0	0	0
0	0	0
0	6	0
0	0	0
0	6	25
0	0	7
0	22	23
1	5258	6409
0	78	36
1	4345	2156
1	4937	4761
1	3487	2661
1	43	63
0	0	3
1	3949	1953
0	0	0
0	4	28
0	0	10
0	0	0
1	37332	44899
0	124	233
1	2799	5044
0	0	10
1	820	681
1	3903	5087
1	1353	1345
0	19	107
1	3584	3132
1	243	327
0	1	0
1	4232	2006
1	5140	8111
1	2983	2539
0	6	62
0	1	5
0	2	8
0	68	16
1	645	932
1	7384	3007
0	25	42
1	1392	985
1	1755	329
1	11545	17997
0	2	15
1	13701	8168
0	13	40
0	88	484
1	4401	4259

0	0	10
0	0	0
0	28	29
0	1	4
0	4	5
0	0	2
0	49	30
0	58	212
1	6411	6321
1	8641	2906
1	1084	1716
1	647	609
1	7576	12725
1	2009	31278
1	2570	1875
1	12516	17648
1	7477	3400
1	2520	3787
0	2	0
1	4238	4358
1	7232	5697
1	7237	11631
0	16	171
0	2	9
1	4913	4118
0	0	4
0	2	25
1	92	951
0	151	105
1	2078	3097
0	5	13
0	64	125
0	30	30
0	0	16
0	16	12
0	1	2
0	0	0
0	1	10
0	0	0
0	5	35
0	4	22
0	14	11
1	3989	4521
0	3316	206
0	9	120
1	1207	46
1	15729	1864
0	64	177
1	2120	1503
1	2108	2352
0	12	36
1	801	434
0	45	100
1	6924	9608
1	586	205
1	46010	65908
1	7424	5048

0	1	24
1	2674	536
1	9864	2956
1	11970	23958
1	2061	731
1	4158	14016
1	11822	3383
1	4538	5425
1	2424	763
0	52	102
1	3031	3851
0	588	104
1	111	679
0	2	2
0	1	12
1	9619	7906
0	688	1462
0	8	5
0	23	167
0	205	59
1	2186	825
0	1	2
0	1	2
1	36310	3461
1	3211	279
1	496	260
1	1148	482
1	1862	2040
1	3011	4419
1	4750	7556
0	1	1
1	1115	300
0	2	52
0	6	8
1	14738	35011
1	1169	640
0	0	0
0	0	0
0	1	0
0	0	1
0	14	8
0	22	5
0	0	8
0	20	16
0	58	45
0	0	0
1	2280	1043
0	25	150
0	3	51
0	8	72
0	23	11
0	3	4
0	0	0
0	3	5
1	3093	3801
0	1	4
1	16690	25091

0	1	0
0	0	0
0	0	0
0	2	15
0	21	226
1	695	851
0	10	39
1	12801	8487
0	0	0
1	9667	13001
0	1	4
0	0	0
0	0	0
0	49	47
0	0	3
0	0	1
1	4083	1073
0	171	1158
1	19826	31259
0	3	9
0	0	2
0	3	5
0	0	2
0	22	16
1	1328	109
1	129	421
1	423	529
0	7	10
1	238	353
1	2604	618
0	41	292
1	12007	2624
1	3999	1209
1	1277	188
0	1038	126
0	6	4
1	1591	981
1	2691	1062
1	2057	2248
1	7892	2224
0	1167	262
1	5278	3434
0	0	0
1	11	76
0	7	130
0	0	4
0	0	0
0	8	18
0	0	4
1	10230	2723
0	9	50
1	599	694
1	4735	774
1	14639	22707
1	3162	4934
1	3328	1694
1	473	144

1	6908	1592
1	116	43
0	6	4
0	24	145
1	5451	3051
1	2319	2106
1	31	493
0	3	60
1	4115	6596
1	61	868
1	5320	1331
1	51	268
0	0	0
1	15812	20019
1	2245	3347
1	3127	1397
1	7135	2169
1	563	600
1	2695	3566
1	2047	1915
0	37	55
0	19	147
1	1209	1701
1	4596	4107
1	2348	1701
0	3	2
1	1136	335
1	11381	4524
0	43	356
0	0	0
0	0	1
0	0	3
0	1	17
0	2	1
0	0	6
0	0	2
0	4	0
0	40	30
0	21	27
0	29	53
0	3	12
0	5	7
0	1	0
1	1814	2973
1	5	90
1	6761	5246
1	3334	4095
0	6	54
1	12680	12740
1	1985	3509
0	81	33
1	4794	6771
0	0	0
0	0	0
0	0	0
0	0	0
0	0	0

1	2943	4027
0	9	16
0	1650	213
0	4	1
1	815	13169
0	3	42
0	0	2
0	1	4
1	5224	1836
1	16126	11545
1	11917	6801
1	4134	5212
0	2	42
0	28	17
0	4	4
0	1	0
0	0	0
0	0	0
0	0	0
0	0	0
0	0	0
1	3790	96
0	8	18
1	307	5462
1	9387	12782
0	4	0
1	2116	2355
0	0	0
0	3	34
1	8451	16440
1	2013	2646
1	2566	965
1	1460	253
1	3615	1126
1	2741	1276
1	6976	10717
0	16	195
1	3869	4542
1	4127	5578
0	3	13
0	25	41
0	0	0
0	0	1
0	0	7
1	2756	2388
0	132	70
0	0	8
1	4038	4973
0	739	445
0	0	6
0	636	651
0	1	1
0	1	10
0	2	8
0	0	11
0	1	2
1	33061	13328

1	1387	358
1	613	477
0	77	85
0	0	6
1	1324	274
1	5342	1226
1	133	171
1	8756	408
1	14925	25774
1	7590	7429
0	3	1
0	0	2
1	17699	11848
0	0	8
0	0	0
0	0	1
1	26446	33042
1	22978	31311
1	227	1703
1	108	1774
0	20	91
0	0	0
0	0	2
0	4	9
0	0	0
0	8	9
1	3415	4061
1	2438	2537
1	58534	77417
0	5	3
0	7	31
1	1294	1072
0	105	108
0	119	87
0	0	2
0	0	0
0	0	2
0	0	0
0	0	0
0	2	8
1	3560	2385
0	0	0
0	0	0
0	4	7
0	0	0
1	15897	17761
1	1924	2896
0	37	258
1	4869	10354
0	23	27
0	1	9
0	18	29
0	7	10
0	15	60
0	0	0
0	0	0
1	2709	2123

0	0	0
0	0	1
0	0	3
0	0	0
1	1264	2050
1	9322	13984
0	0	0
0	3	0
0	0	0
0	0	1
1	10269	14789
1	1423	1105
1	12378	10085
1	2205	1452
0	17	10
1	8004	2717
0	0	5
0	1	4
0	0	2
0	16	110
0	0	3
0	0	0
1	4219	3445
0	0	0
0	3	5
0	6	18
0	21	30
0	3	106
0	0	33
0	8	44
1	1968	2475
0	10	14
0	0	0
1	194	3897
1	60	1777
0	61	746
0	3	27
1	5759	3013
1	2619	4175
1	6049	2365
1	11579	2663
0	29	308
0	12	17
0	0	0
0	92	140
1	1685	1329
1	12069	5619
1	1431	1673
1	16858	8628
1	3523	3342
1	2451	3993
0	90	601
0	73	457
1	1423	2440
1	968	865
1	2260	1607
0	5	22

0	0	8
0	2	0
1	26694	25063
1	1903	455
1	480	516
1	6539	7337
0	2	4
0	0	0
0	0	0
0	0	11
0	0	2
0	5	19
0	0	3
0	4	1
0	46	34
0	14	41
0	3879	306
0	747	202
0	132	65
1	6381	1307
1	1311	85
1	18339	2516
1	739	460
0	0	17
0	9	26
1	1200	242
1	419	281
1	212	598
1	14531	8132
0	4349	5936
0	29	120
1	1479	721
1	523	571
0	0	12
0	2	8
1	1395	1308
1	4609	5401
0	67	66
1	4480	1250
1	4126	2105
1	1047	1690
1	13767	13853
0	0	1
1	15995	18383
0	1	87
0	0	3
0	9	20
0	0	2
0	0	2
0	19	154
1	605	739
1	1292	1372
1	1842	2952
1	2261	2614
1	1797	1413
0	8	63
0	9	1

0	3	3
0	2	20
0	0	0
1	0	1
1	4460	2969
1	932	1405
1	3875	849
1	15243	13804
1	2475	3463
0	0	1
0	1	37
1	10471	8061
1	1128	1218
1	4763	1034
1	4973	5991
0	2	7
0	2640	898
0	3	13
0	85	95
0	9	14
0	2	14
1	12844	2482
1	4458	2127
1	7303	4178
1	1909	1547
0	6	48
0	4518	239
0	0	1
0	1	0
1	8291	7715
1	7061	11203
1	684	851
1	4496	4305
1	10972	1173
1	11652	17062
1	8621	6065
1	4935	4128
1	1440	842
1	8065	7264
1	11781	15518
1	8	25
0	8	7
1	15499	22857
1	7250	6245
1	1080	1093
1	10744	10342
0	0	3
0	0	2
0	0	5
0	0	3
0	0	3
1	18690	18814
0	1	8
1	1520	1341
1	7970	3786
1	2950	3355
1	1730	2302

1	1285	1285
0	3	4
0	0	4
0	0	2
0	3	4
1	32	356
1	5836	4755
1	6991	2491
0	4	28
1	4284	5019
0	0	6
1	2948	4722
1	1106	1469
0	11	51
1	20314	32528
0	2	47
0	3	1
0	4	16
0	8	63
1	15	135
0	11	54
1	11555	264
0	4	1
1	306	462
1	21484	23941
0	3	0
0	5	4
1	3356	1571
1	2048	736
1	1333	919
0	11	18
1	1405	513
1	62	325
1	8052	6800
0	31	16
0	0	2
1	2123	2323
1	11313	5233
1	1289	1583
1	3580	2305
0	7	13
0	3	17
0	23	147
0	15	25
1	1862	1158
1	5433	1551
1	6617	7203
0	9	48
1	3452	73
0	17	11
0	1	2
0	17	4
0	2	7
0	4	12
0	0	0
0	5	2
0	0	0

0	0	0
0	10	9
0	13	167
1	584	9307
0	0	9
0	0	7
1	11612	12098
0	0	0
1	4370	6183
0	37	84
1	1175	2237
1	6936	6078
1	3212	4484
1	11733	2001
1	1047	3322
1	13160	4176
1	1688	1186
1	7662	5543
0	3	50
1	4636	654
0	6	127
1	4574	4109
1	1530	890
1	2963	450
1	1347	1332
0	5	6
0	2	6
0	286	314
1	4902	756
0	6	42
1	2170	1631
1	4077	878
1	2562	1184
1	9910	7069
0	4	25
0	4	6
0	1	0
1	1730	1821
1	6211	3870
0	98	103
1	2018	444
1	6498	8223
0	648	262
1	2894	2582
0	0	1
0	506	262
1	9554	23530
0	8	149
1	5081	3406
1	15957	1260
0	13	15
1	5716	930
1	7546	5105
1	13240	14820
1	1355	2239
1	3419	1323
1	65	175

1	6221	1399
0	19	48
1	3781	1106
0	0	0
0	0	0
0	0	0
0	0	4
0	0	0
0	0	1
0	7	4
0	11	3
0	0	0
0	2	0
0	0	0
0	13	13
0	32	27
0	7	12
1	7152	2697
1	5961	1366
0	20	33
1	17046	22348
1	2169	1078
1	8799	9493
0	0	2
0	0	0
0	0	1
1	3638	418
1	13594	7490
1	622	1007
1	2170	2635
0	55	73
1	1676	595
1	12525	5547
1	2292	2300
1	3253	806
1	9232	14158
0	9	57
1	2081	1748
0	2	1
1	1190	1792
1	2429	3034
0	0	1
0	110	225
0	19	30
0	1	2
0	66	51
1	101	283
0	3	42
0	1	6
0	4	49
1	719	1032
0	2369	285
1	1716	1740
1	4634	7149
1	6729	8024
1	4293	2078
1	2650	3786

1	5604	4483
1	7239	3563
0	4	43
0	0	20
1	85	128
1	12383	1584
1	16319	9287
0	4	40
0	0	1
1	3366	4842
1	3946	1251
0	4	19
0	3	18
1	3895	812
1	2074	2471
1	883	530
1	6670	2108
1	167	182
1	465	224
1	3064	3879
0	12	39
0	32	88
0	10	17
0	1	9
0	0	0
0	9	24
1	44	52
0	1619	175
1	436	374
1	1603	109
1	6299	4783
1	3332	4094
1	4315	3214
1	2227	223
1	611	858
1	16190	7274
0	0	0
1	8057	9438
0	55	44
1	7818	2835
1	9676	14144
1	340	449
1	2449	1239
1	3952	1081
1	4344	4082
1	2423	813
1	7930	4127
1	1737	1763
1	1294	1285
0	15	139
1	370	6481
0	20	195
1	2942	4512
1	9060	5356
0	0	3
1	1873	1472
0	43	102

1	6197	957
0	1	0
0	0	5
0	0	3
0	3	11
0	26	83
0	27	119
1	602	386
1	2409	3369
0	0	0
1	81	216
1	2001	3823
1	222	789
1	984	1562
0	14	69
1	13690	10933
1	1184	1751
1	2604	1455
1	4679	6335
0	2564	73
1	5371	5788
1	432	615
0	24	22
1	3841	3333
1	1128	942
1	1880	2871
1	2576	3268
0	0	0
0	0	0
0	1	0
0	3	2
1	25	112
0	6	57
0	0	2
0	0	0
0	0	0
0	2	0
0	0	0
0	0	0
0	2	1
0	111	89
0	4	16
0	0	0
1	1986	2434
1	4671	408
1	4604	588
0	0	18
0	0	2
1	16006	3577
0	7	24
1	11999	1225
1	20420	26664
1	32058	21215
1	5985	1445
0	14	20
1	77	1755
0	0	6

1	207	3540
1	3846	2753
1	5973	5754
0	45	28
0	3	16
1	8253	6007
1	476	256
1	63693	73026
1	1933	3197
1	5949	4791
1	2829	1585
1	5894	1013
1	735	1046
0	233	167
0	18	113
1	43233	42041
1	1825	567
1	3031	4059
1	4058	343
0	4	28
0	3	17
1	96148	140526
0	1	2
0	30	101
0	3	1
0	3	3
0	9	2
1	1646	1947
1	12295	5859
1	11064	3797
1	3449	2425
1	2234	1352
0	461	416
0	0	0
0	6	8
0	0	13
0	0	0
0	2	29
1	4114	4680
1	1202	1625
0	7	17
1	8254	6801
1	3806	3138
1	74	235
1	400	221
1	4714	2977
0	1	1
1	2981	555
1	5700	5179
0	5	24
0	97	207
1	10101	6114
0	37	65
1	950	1128
0	0	0
1	4723	129
0	1	0

1	4546	2830
0	0	0
1	2483	1995
1	9594	2713
1	4571	1718
1	1009	1539
0	21	85
0	94	102
0	7	0
0	6	6
0	0	16
0	0	8
1	15	480
0	1	0
1	2460	3666
0	10	139
1	1164	323
1	8503	12088
1	553	453
1	3927	1757
1	12	257
0	3	5
0	20	19
0	0	0
0	0	3
1	3536	5012
1	15485	9930
1	19517	15920
1	11304	6156
0	0	0
0	0	12
0	0	3
1	6290	6192
1	3625	67110
0	20	124
0	0	0
0	1	6
1	14332	9614
0	27	40
1	5276	1468
1	1913	377
1	8248	2701
0	0	9
1	20256	8450
0	8	66
0	2	77
1	15842	17392
1	15413	3866
1	1757	1440
1	16857	16720
1	3080	1699
1	615	336
1	22617	30879
0	250	57
0	13	26
1	369	498
1	5861	837

1	1813	1075
0	6	3
1	8269	2282
0	226	140
1	106	836
1	3775	4930
1	4183	4058
1	5262	5426
1	900	1146
1	737	19
1	9275	5707
0	0	3
1	6869	8061
1	3963	888
0	7	14
1	306	75
1	1202	915
0	0	0
0	0	0
0	0	0
0	16	13
1	740	543
1	2183	1749
1	475	443
0	19	17
1	3299	4123
0	1047	172
0	90	67
1	21564	270
0	35	14
0	2	0
0	12	62
1	8400	6463
0	0	7
1	11292	6185
1	2898	970
1	2581	2059
1	779	208
1	1244	486
1	2600	2187
1	5196	1359
0	3	6
0	4	4
1	992	1557
1	826	645
0	0	13
0	4	6
0	0	0
0	114	182
0	0	0
1	929	1080
0	7	93
1	2866	1694
1	2685	4008
0	16	49
0	10	16
1	6766	6664

0	0	2
1	1361	1524
1	15305	20857
0	9	6
0	0	0
1	6584	6875
1	2288	384
0	0	0
1	12176	3242
0	0	6
0	31	61
1	7805	11545
1	6725	2649
1	530	5675
0	0	22
0	0	0
0	0	1
1	3113	3894
1	4049	5505
0	21	55
1	1897	3267
1	1808	2243
1	6348	9499
1	10625	2117
0	0	1
0	1	6
0	0	0
0	5	11
1	1897	3091
1	4428	6514
1	1326	703
1	5305	4095
1	3956	4512
1	6865	10644
0	0	1
0	0	4
0	0	0
1	337	229
0	7	4
0	6	1
0	2	6
0	1	2
0	11	22
0	7	25
0	16	59
1	79503	117637
0	37	69
1	5944	2945
1	805	2253
0	53	28
0	0	0
0	214	247
0	5	2
0	0	0
0	0	0
0	5	5
1	3783	1241

0	0	0
0	1	35
0	57	34
0	1	6
0	2	25
0	0	0
1	2997	3656
1	4531	8117
1	25641	25518
0	17	217
1	3986	2026
1	11051	1016
0	0	0
0	1	1
0	0	1
0	4	4
0	3	1
0	0	0
0	0	0
1	3958	1144
0	6	70
0	4	75
1	7874	4858
0	22	298
1	3271	1135
1	1309	584
1	1147	1754
1	50719	69866
1	20851	16224
1	1988	2833
1	4940	6113
0	4	81
1	992	1220
1	10570	6636
0	0	28
1	1200	431
1	8683	1421
1	3782	1618
0	0	0
1	57	992
1	3606	711
0	21	25
0	4	95
1	10592	3509
0	10	30
0	0	4
1	22307	35315
0	6	6
0	9	5
0	6	0
0	0	1
1	10581	6034
1	2848	1588
1	3431	654
1	4534	1223
0	0	0
1	511	612

0	116	115
0	3	5
0	1	15
0	3	2
0	1	7
0	3	11
0	4	0
0	1	2
0	1	0
0	1	0
1	2703	3596
1	8863	10308
1	5120	4009
0	0	0
1	1709	1492
0	0	10
0	59	603
1	593	432
1	1482	1443
1	3792	2376
0	0	0
1	5290	4737
1	4281	2830
0	0	0
0	1	47
0	3	0
0	4	5
0	0	0
0	9	2
1	2879	513
1	5000	154
0	37	79
0	1	22
1	10924	12418
1	2460	1897
1	11615	4046
1	10805	796
1	1013	864
1	10449	1183
1	4773	251
0	1173	106
0	0	4
0	103	452
1	12935	9155
0	249	87
0	85	64
0	0	0
0	0	0
1	6586	2792
1	3830	4019
1	3830	687
1	2843	2806
1	6285	5394
0	0	2
0	3	20
1	2952	4268
1	11962	13671

1	4930	2671
0	22	33
0	1	1
1	466	1228
0	2	10
0	3	3
0	2	0
1	853	584
1	571	983
0	4	10
1	12642	2908
1	5331	5537
0	0	0
1	5180	1126
0	0	6
0	0	0
0	0	5
0	6	72
0	1	25
1	31	315
0	0	4
0	0	2
0	0	0
0	2	1
0	186	1781
0	0	0
1	78	1851
0	0	0
0	1	8
1	18	126
1	652	719
1	1031	9829
0	1	22
1	8319	29896
0	0	1
0	0	0
0	0	0
0	0	2
0	0	28
1	4720	84290
0	3	77
0	0	0
1	5044	1945
1	278	657
1	2883	845
0	72	103
0	2	29
1	2570	3861
1	1637	328
1	6727	7618
0	3	6
0	0	4
1	150	1944
1	6983	1482
1	2614	1358
0	0	0
0	9	100

0	4	5
1	2126	662
0	14	168
1	2557	853
1	12660	9541
1	6639	3844
1	4432	6139
1	12572	9111
0	707	137
1	1061	350
0	3	84
0	2	7
0	0	0
0	79	58
1	19052	24629
1	2182	1383
1	1128	214
0	3	2
1	72	1009
1	5222	6277
0	0	10
0	10	24
1	2037	838
1	3238	4849
1	10174	3889
0	5	5
0	0	2
0	0	2
1	4088	487
1	3189	4013
0	5	36
0	28	9
0	2	23
1	17641	6868
1	2734	2757
0	0	0
0	0	1
0	19	4
0	58	78
0	2	0
0	0	0
0	0	0
0	0	3
0	0	1
1	5402	4117
0	0	0
0	2	0
0	104	20
1	3951	1513
1	3715	2268
1	9518	1003
0	1	3
0	35	21
1	3002	152
1	6897	7159
1	1736	1382
1	6605	2562

1	893	522
1	611	287
1	4456	5840
1	11308	12344
0	1	18
0	118	1213
0	24	20
0	292	265
0	13	22
1	1320	691
1	5298	1587
1	5915	1597
1	1296	59
1	7	54
0	2	3
0	4	56
0	3	11
0	4	8
1	2822	3636
1	61522	92197
1	3394	1391
1	54671	16517
0	33	46
0	102	347
0	26	29
0	2	1
0	12	3
1	7214	2934
0	4	53
0	2	10
0	1	1
0	0	2
0	0	1
0	1	11
0	6	33
0	5	6
0	65	118
1	4674	4803
1	12112	7526
0	0	0
0	0	1
1	201	846
1	1085	783
1	79187	54397
1	2607	2227
1	4965	780
0	0	0
1	2961	2930
1	2518	1892
1	2153	964
0	20	15
0	17	2
0	0	5
0	0	2
0	104	92
0	0	2
0	0	0

0	0	0
0	0	15
0	3	66
1	1850	779
0	1	15
0	8	8
1	8137	1930
0	4	27
0	5	3
0	0	0
1	9920	10565
0	0	0
0	10	30
0	0	0
0	62	49
0	48	247
0	27	13
0	22	13
0	1	3
1	10174	1794
0	0	0
0	2	0
0	3	0
1	2852	4191
0	30	31
0	0	0
0	1	16
0	7	2
1	3800	2896
1	13142	19852
1	5538	1278
1	2095	476
1	4207	3359
1	8304	1912
1	2856	3004
0	1565	74
1	5150	2285
0	77	11
0	6	105
0	0	1
0	74	24
1	1803	1492
1	5407	2107
0	14	45
1	1334	457
1	13127	5171
0	66	97
0	20	33
1	6334	2592
1	6147	5408
0	1	15
0	48	48
0	9	36
0	1	0
1	2400	2638
1	8154	11872
1	2411	3554

0	0	6
0	0	4
1	5546	4855
0	0	0
0	1	0
1	23413	28293
0	0	1
0	0	0
0	0	2
1	10416	2838
0	0	3
1	1147	1256
1	3879	3847
0	0	0
0	21	286
1	8784	3657
1	4655	4069
1	5250	7591
1	9692	11183
1	6532	561
0	16	11
0	0	0
0	17	5
1	3118	2313
1	4052	1794
0	18	40
0	19	65
1	28	247
1	12379	5475
1	2749	616
0	0	14
1	3227	6158
0	18	14
0	0	0
0	5	30
1	1197	1393
1	7637	2772
1	1017	1373
0	25	26
1	8596	3348
0	11	26
0	90	520
0	11	33
1	178	282
0	0	2
0	85	455
0	2	28
0	102	1228
1	1996	2230
0	58	312
1	18282	7091
1	7364	7189
1	869	746
1	3421	4728
1	10133	5123
0	0	0
0	367	360

1	2646	1849
1	2821	1431
0	20	29
0	136	183
0	1	8
1	7763	11848
1	3886	1975
0	7	46
0	1	0
1	657	489
1	2940	4276
1	9982	16470
0	1	7
0	21	167
0	47	118
1	2620	1924
1	3156	1848
1	2286	2349
1	3842	3973
0	16	35
1	1325	984
1	957	1121
1	654	480
0	0	0
1	1549	1254
1	10397	6565
1	1982	985
0	13	60
0	0	0
0	14	15
0	9	15
1	4906	3385
0	68	221
0	0	12
1	705	775
0	25	68
0	4	48
1	3309	2077
1	12277	5080
1	9	86
0	0	0
0	0	0
1	3904	996
1	2982	1510
1	9528	9413
0	477	834
1	4574	3522
0	3	9
0	2	34
0	16	107
1	7329	7241
1	21081	6986
1	3969	3263
0	0	0
0	0	0
1	1306	693
0	4	12

1	76	268
1	2210	3206
1	180	179
1	1342	2454
1	4898	3688
1	5140	6245
1	16905	24550
1	1941	2442
0	0	1
0	386	3071
0	0	0
0	25	24
0	17	83
0	2	3
1	719	1554
1	10760	1193
1	7125	8519
1	5566	546
0	0	0
0	10	7
0	1	6
1	5644	646
1	3851	3275
0	3	38
0	12	20
0	2	8
1	4762	8044
1	2919	1401
0	2	11
0	1	41
0	58	127
0	117	26
1	1196	24
0	10	37
1	6227	6609
1	1262	2190
0	21	30
0	0	0
0	0	0
0	0	0
0	0	0
1	7723	5715
1	1576	399
1	1080	620
1	4735	4636
1	1995	1081
1	2068	4008
0	3	9
0	3	19
1	174	2334
1	296	489
1	516	517
0	119	176
0	7	91
0	12	56
1	40	311
0	5	47

1	19750	35198
0	0	0
0	0	1
1	14548	8130
1	24069	28159
0	146	131
0	0	0
1	6769	3020
1	2043	2732
1	17942	1877
1	6455	6652
1	1269	555
0	6	37
0	66	132
1	16407	9218
0	0	0
0	0	0
1	2882	3572
1	839	1104
1	9705	12116
1	8404	10402
1	28512	50887
1	8980	11085
1	30210	5118
1	12840	10055
0	0	0
0	1	5
0	14	33
0	47	42
0	75	89
0	0	11
1	1446	667
0	0	1
1	11997	8855
1	11606	2876
0	20	53
1	2762	1166
1	1769	670
0	5	180
1	5447	1415
0	9	8
0	1	3
0	20	45
0	0	0
0	0	0
1	2283	1811
1	3142	2080
1	11309	19891
0	2	31
1	1710	2334
1	760	1500
1	7409	1448
1	2883	568
0	0	3
0	0	0
0	2	0
1	1520	2581

1	2350	1865
1	448	479
0	0	0
1	8637	3008
0	27	37
0	0	2
1	2469	2845
1	10593	13452
1	6042	6434
1	24669	25524
0	25	88
0	18	178
0	26	31
0	0	1
0	51	16
0	0	1
0	101	103
1	1091	705
1	4386	745
1	15	150
1	253	1318
1	4230	8045
1	2266	4124
1	4131	1010
1	3425	742
1	9977	10796
1	1261	1738
1	513	218
0	14	39
0	43	194
1	2014	463
1	1784	1637
1	189	2560
0	4	48
0	25	27
1	12	14
0	41	24
0	0	2
0	0	2
0	0	0
0	27	19
1	11878	12744
0	0	3
0	0	0
1	1103	7779
1	44	198
1	154	256
0	2	98
0	4	3
0	2	8
0	0	0
0	21	134
1	1751	365
1	2087	1266
1	633	684
1	1340	2019
1	1880	797

1	6078	1412
0	0	3
1	14816	19632
1	21805	26556
1	5076	2316
0	2	0
1	18	231
0	16	3
0	82	45
1	12067	13038
0	22	165
1	9672	13783
0	1	81
1	2157	2510
1	32624	6476
1	9849	3788
1	4244	1112
0	16	14
0	9	4
0	7	43
1	6437	7174
1	7226	6578
0	7336	4127
1	3494	295
0	1	9
0	0	2
0	1	3
0	3	15
0	0	0
0	19	8
0	1	0
0	16	2
1	8	84
1	1386	1726
1	35	184
1	1243	1080
1	5791	1643
0	455	24
0	0	1
0	0	0
0	22	221
1	1182	413
1	2391	663
1	5255	3086
0	23	260
0	0	0
0	3	13
0	7	49
1	29909	25905
1	993	1255
1	825	409
0	30	26
0	0	0
0	1	4
0	2	0
0	305	175
0	3	15

0	8	6
0	1	5
0	1	10
0	3	49
1	2424	2679
0	2	7
0	6	22
1	7999	10570
0	9	40
1	12291	4289
1	10602	6474
1	20327	13354
1	3803	3912
0	5	94
0	0	2
0	0	2
0	0	0
0	1	5
0	19	3
1	18679	21732
0	85	78
0	86	65
0	0	0
0	0	0
1	3757	1427
1	1019	1358
0	7	0
1	8125	1997
1	29296	23676
1	5450	1847
0	4	6
0	4	4
0	1	1
0	3	1
0	3	33
1	745	929
1	3971	1733
1	2604	3312
1	4388	7594
1	5365	4874
1	2285	1403
1	2400	713
1	9189	3391
0	4	18
1	3218	2270
1	3647	1215
1	6266	15869
1	8328	10080
0	8	87
0	0	0
0	10	16
0	16	26
1	81	146
0	19	21
1	5362	7490
1	32826	31005
0	1	7

0	24	31
1	1808	2372
1	2182	1897
1	18935	16143
1	6076	3168
0	91	81
0	1310	564
1	4768	2194
1	24901	24965
1	2692	1391
1	2191	2546
1	3924	127
0	3	7
0	0	8
1	2290	2479
0	48	23
1	14388	18822
0	5	9
0	14	48
1	3803	1060
0	0	2
0	10	7
0	4	0
0	1	0
0	0	0
0	0	0
0	5	9
0	0	1
1	3653	362
1	11727	11542
0	11	14
1	2393	660
1	4300	155
1	94	1436
1	12916	12885
1	1016	801
1	3292	499
1	1121	515
0	238	1163
0	5	2
0	21	44
0	1	1
1	12830	3637
1	7305	13773
0	2	3
1	8808	7045
0	21	25
1	5399	5524
1	6960	3909
1	18001	4328
1	2136	969
1	1117	609
1	974	2355
1	34	817
1	3032	651
0	0	0
0	0	0

0	0	1
0	0	0
0	9	6
0	0	0
1	3589	3220
0	1	2
0	5	5
0	18	3
1	2628	644
0	0	4
1	8847	11334
1	4600	6403
1	463	557
1	4750	3423
1	5890	4671
0	42	58
1	15688	8869
0	0	0
0	1	3
1	3857	3092
1	13863	1972
0	0	0
0	0	0
1	7983	7352
1	1629	1975
1	1338	1096
0	0	1
0	0	0
0	0	35
0	0	10
0	0	1
0	0	2
1	2017	1998
0	3	55
0	5	5
0	22	12
1	7271	11360
0	14	8
0	16	11
1	342	426
1	30725	48456
0	15	12
1	1333	1306
1	883	1083
0	14	27
1	388	228
0	1	4
1	9591	6985
1	11295	16293
1	2437	2981
1	4516	954
0	0	1
0	0	3
1	12268	20439
1	1712	1276
0	0	1
1	9930	2134

0	10	25
0	2	14
1	3542	5801
1	46	755
0	6	5
0	0	1
0	0	0
0	0	0
0	0	1
0	0	0
0	8	16
0	6	88
0	0	3
1	1764	1180
0	0	0
0	2	4
1	9570	4120
1	3859	3307
1	558	609
1	544	618
1	7066	11761
0	1	19
1	1978	1299
0	41	55
0	0	0
0	63	43
0	26	193
1	548	340
1	6166	4304
1	183	432
1	68257	115406
0	13	69
0	0	0
0	0	1
0	2	3
0	1	0
0	0	0
0	0	0
0	0	0
0	43	30
0	0	3
0	2	11
0	0	0
1	8459	4460
1	16429	10251
0	0	1
0	0	0
1	2565	1099
1	628	127
1	7485	749
1	27811	40588
0	1	7
0	3	5
1	3674	3866
0	4	0
1	1847	734
0	2	2

1	2101	2803
0	4	24
1	5829	2202
1	35	705
0	23	8
1	4065	1804
1	684	765
1	1781	455
1	4421	4859
1	4238	3844
0	0	2
0	0	2
1	3543	6018
1	2859	1479
1	2040	2194
0	0	0
0	1	5
0	130	46
0	0	0
1	857	984
1	1106	467
1	2017	1316
1	6314	4314
0	48	84
1	18824	24757
0	1	17
0	1	63
0	41	65
1	116	250
1	2882	2396
1	7825	10938
1	2388	1064
0	1	7
0	4	5
0	0	2
0	8	1
0	2	6
0	7	27
1	7408	7664
1	1956	333
0	5	12
1	1891	502
1	19140	18589
0	0	14
0	0	0
0	3	0
1	5175	5106
0	3	1
1	4199	2093
1	1142	1213
0	1	16
0	27	230
0	12	172
0	1	8
0	3	14
0	0	0
0	5	6

0	0	1
1	15	66
1	898	766
0	0	0
0	84	170
0	1	0
0	0	0
0	0	5
1	15229	13406
1	6508	8747
1	8180	5749
0	19	46
0	0	15
1	2697	1609
1	14	205
1	5203	4873
0	3	1
0	15	1
0	3	3
0	40	18
0	87	84
0	0	1
0	1	31
1	2910	3193
1	4514	7172
0	16	209
0	0	24
0	3	9
0	0	3
1	1692	886
1	538	8221
0	0	0
0	0	0
1	117	129
1	1975	445
1	148	3065
0	0	0
1	5527	6083
1	3516	5423
1	5566	5281
1	16016	16361
1	3492	1897
1	3561	4667
1	5632	2932
1	6028	4436
1	12607	17349
1	5019	2594
0	0	1
0	0	0
0	1	2
1	14996	14124
1	2476	4421
1	3212	5292
0	0	0
0	22	32
1	2356	1789
1	6333	5950

0	33	204
0	0	8
1	88	160
0	1	16
0	1	15
0	1	40
0	8	8
1	5534	3083
1	6736	8797
1	12527	12677
0	1	26
0	4	37
0	1408	87
1	2935	3552
1	2157	586
1	17197	9430
0	0	3
1	7422	976
0	2	2
1	2773	3901
1	3565	4117
1	1828	2769
0	1	3
0	0	0
0	0	0
0	0	0
0	6	6
0	31	45
0	58	43
0	16	11
0	5	0
0	7	10
0	39	25
0	57	67
0	33	35
0	1	2
0	0	2
0	71	53
0	59	54
0	87	76
0	0	0
0	0	0
0	0	0
0	205	117
1	1598	513
0	22	26
1	1854	20
1	4701	5219
1	942	787
0	32	68
0	25	24
1	5990	7260
1	7568	10316
1	1693	960
1	1131	744
0	1	5
0	2	2

0	0	0
1	7006	1503
1	13868	17927
1	2893	560
1	7798	1383
0	9	13
1	4406	4842
1	1147	1183
1	16232	10047
1	203	571
1	5394	2978
0	1	0
0	3	20
0	46	185
0	0	17
0	13	129
0	0	0
1	9159	4223
0	0	0
1	2362	1221
1	3092	2729
1	1256	1706
1	13012	8669
0	0	2
0	0	0
0	0	0
0	0	0
0	0	0
1	338	4790
0	3	25
0	260	243
0	14	49
1	4432	5050
0	10	161
1	9834	9030
0	0	4
0	12	17
0	1	6
1	107	2102
1	7068	4832
1	5356	10172
1	13523	12830
1	3317	2259
0	24	44
1	7063	4987
0	0	0
1	536	256
0	1	0
1	2306	2884
0	0	4
1	5457	3555
1	354	174
0	2	2
0	0	0
0	3	2
1	3200	126
1	7631	1624

1	685	385
1	2194	761
1	46	490
0	0	0
0	0	11
0	0	6
0	83	139
1	1897	28482
0	4	7
0	6	1
0	0	1
1	2090	1987
0	0	0
0	0	5
0	7	16
1	1799	1588
1	5150	7353
1	11118	9880
0	7	125
1	1161	788
1	770	141
1	1636	830
1	117	216
0	0	0
1	3595	4681
1	5469	3672
0	0	0
0	0	0
0	1	0
0	0	0
0	1	0
0	2	0
1	3559	730
0	1	3
1	3015	3996
1	2518	3062
0	14	86
1	2911	3412
0	1	1
0	0	0
1	463	1447
0	0	1
0	2	24
0	1	0
0	0	0
0	22	15
1	13	4
0	2	9
0	41	124
0	2	5
0	8	2
0	6	0
0	0	4
1	60	1245
1	9103	3888
1	2599	1528
0	2	14

0	8	8
1	6849	7771
1	2259	290
0	26	170
1	4652	5568
1	3782	1731
0	27	108
1	1322	555
1	2594	2331
1	1234	636
0	81	197
1	2022	193
1	7037	9883
1	1262	2044
1	1303	841
0	1	5
0	101	158
1	11015	12235
1	7360	8800
0	11	123
1	2761	2615
0	2	3
0	11	110
0	1	55
0	11	6
0	0	0
0	43	45
0	2	1
0	55	50
0	11	1
0	0	0
0	49	96
0	31	14
0	6	2
0	295	35
1	13719	7011
1	910	1241
1	1076	1470
1	8313	10882
0	7	17
0	1	12
0	57	261
1	61	113
0	2	46
0	8	16
1	57	490
0	5	22
1	1906	943
1	2193	813
0	37	170
0	13	35
1	8586	3382
0	8	23
0	4	3
0	9	14
1	2371	1573
1	2678	1321

0	0	0
1	8795	10244
1	832	1235
0	1	58
1	634	464
1	2263	870
1	2666	2425
1	3827	3394
0	0	0
1	16255	11250
1	5999	2495
1	6595	3676
1	15966	7734
1	4351	5207
0	1	5
1	1928	856
0	0	0
0	0	0
0	0	0
0	0	0
0	0	4
0	19	14
1	3272	2022
1	1950	1259
1	54	89
1	68	527
1	22019	33669
0	1	4
0	12	58
1	707	366
1	4654	602
0	22	15
0	23	174
0	0	2
0	12	42
0	22	62
1	2054	315
1	3241	2012
1	9744	11125
1	1553	698
0	0	4
1	675	4008
1	15	89
1	2404	2430
1	423	796
1	16033	26730
1	5596	6735
1	14890	2890
0	59	34
0	2	3
1	3188	3770
1	3814	4991
0	2	15
1	3828	2344
1	17016	13176
1	1821	241
1	3683	1851

1	18	149
0	12	23
0	18	10
0	12	22
0	1	1
0	5	5
0	10	7
0	21	16
0	27	45
0	1	2
0	0	0
1	1784	1422
1	29833	46528
1	28362	44451
1	7707	893
0	0	0
0	73	122
0	0	2
1	3478	9231
1	2534	3020
1	1311	451
0	65	718
1	4352	4753
0	9	17
0	0	0
0	0	1
0	0	2
0	3	2
0	28	21
1	10486	15386
0	42	138
1	2483	1527
1	3720	3052
1	3178	4112
0	0	0
0	0	1
0	9	11
1	5081	5930
0	30	108
1	5968	2947
0	757	863
1	5518	2227
1	9775	16555
0	4	15
0	4	16
0	14	12
0	2	3
0	28	77
1	3236	1783
1	2513	2251
1	3318	3875
1	8130	10058
0	13	153
1	2343	2556
1	5052	869
1	3384	907
1	66903	25818

1	22797	2570
1	56602	84304
1	5504	4077
1	1451	1132
1	12868	17583
0	18	115
0	6	2
1	14015	25826
1	4196	3868
1	659	640
1	880	372
1	24335	32765
0	83	78
0	5	21
1	2902	2108
0	1	11
0	3	6
0	0	2
0	0	5
0	0	8
0	2	28
0	6	36
0	7	52
1	2309	993
1	7682	4370
1	2407	2682
0	76	39
1	1791	272
0	0	14
1	24	340
0	3	8
1	6256	4888
1	4304	5158
1	10171	8647
0	53	61
0	0	4
0	12	5
1	4816	3611
1	2554	2088
1	4722	1211
1	8990	5599
1	1084	1188
1	2677	2132
1	3304	1736
1	28314	33056
0	275	36
1	1100	1090
1	537	449
1	6115	3599
1	2546	818
1	4956	3112
1	1705	2658
1	16800	24529
1	9243	1663
0	1	1
1	2517	619
1	3237	2722

1	2922	4396
1	5886	6348
0	22	205
1	9610	6698
1	4472	4649
1	4400	817
0	78	68
1	4302	5809
1	2264	967
0	2	1
0	9	10
0	0	1
0	1	0
1	1338	1197
0	5	2
0	0	0
0	0	0
0	0	2
0	3	5
0	49	19
0	3	0
0	4	0
0	878	585
0	5	1
0	127	67
0	248	111
0	0	26
0	5	34
0	20	50
1	6294	9844
0	600	82
1	2040	2648
1	6031	4868
1	10348	11159
1	5593	2820
1	6895	8843
0	9	12
0	0	0
0	0	0
0	0	1
0	0	0
0	0	0
0	0	0
1	3080	2318
1	4034	3848
1	1794	2968
1	21509	16120
1	4861	2028
1	1690	730
0	1	14
0	0	0
1	9670	3506
0	0	8
0	0	5
0	14	25
0	1	2
1	4070	6569

1	550	903
1	2640	2800
1	791	210
0	0	0
0	0	0
1	1398	2717
0	0	0
0	0	0
1	1512	1010
0	0	0
0	1	2
0	1134	1014
1	1615	519
1	2865	399
1	1293	781
1	7440	7079
1	2121	2106
1	3064	2346
1	12346	9405
1	1135	1577
1	934	737
1	15	77
1	8989	1786
1	737	699
0	16	36
1	8080	3465
0	9	4
0	58	87
1	4572	3654
1	767	761
1	3923	3750
1	2639	5513
0	6	22
0	0	2
1	2004	1529
0	2	25
1	24077	2321
1	717	763
1	6446	1961
0	0	9
1	5291	2285
0	135	83
1	1695	1113
1	2921	3317
1	2786	2053
1	3168	1003
1	638	564
1	5918	969
1	1254	613
1	14430	14090
0	5	6
0	64	69
1	1354	1237
0	0	34
0	977	26
1	850	441
1	7454	11599

1	2200	407
1	313	331
0	0	0
0	0	0
0	5	2
1	1256	3709
1	1449	1030
0	9	71
1	42016	33351
1	1765	826
1	7048	8431
1	14799	11194
1	4492	3054
1	61	865
1	817	97
1	2868	1663
0	0	0
1	7619	7534
0	137	80
1	29214	16878
1	1776	2005
1	8320	11424
1	5642	4000
1	863	1467
0	3	7
1	3410	2931
0	0	0
1	1194	849
0	0	26
1	2516	4221
1	1021	2115
0	0	0
0	0	1
0	14	109
0	0	0
0	6	35
1	5062	9100
1	16550	13221
1	3996	4152
1	9192	13592
1	356	298
0	9	101
1	520	652
1	3100	3151
1	8877	2582
1	4323	4759
1	8296	1899
1	3682	3176
1	2906	564
1	4211	2030
0	39	37
0	8	32
0	2	16
1	25686	7375
0	16	53
1	1002	1058
1	3159	2565

1	960	949
1	1064	1073
1	473	479
1	7989	8838
1	2199	493
1	3755	3543
0	28	55
0	12	24
1	48925	87648
0	7	51
1	5433	685
1	13741	6377
1	1595	1179
1	772	795
0	0	0
0	0	2
0	2	1
0	0	0
0	3	0
0	6	92
1	16211	6360
0	4	55
1	1654	1428
1	724	411
1	1278	1916
1	1456	112
1	4602	5137
1	1549	514
0	0	1
0	0	0
0	8	13
1	4669	7139
1	1495	2319
0	15	28
1	157	3093
0	2	31
0	64	487
1	62	237
1	859	839
1	6837	3262
0	4	0
1	1374	2462
1	6697	9021
0	7	71
1	1228	1493
0	3	0
1	25937	21705
0	1	14
0	0	0
1	2106	1583
0	0	0
0	1672	299
1	14236	4870
1	106	310
1	701	407
1	7116	5009
1	4867	4537

0	27	71
0	1	5
1	3736	700
0	18	35
1	5546	4982
1	2471	1098
0	0	1
0	0	0
0	0	0
1	2036	155
1	2648	1909
1	107147	172964
1	8456	3629
1	1125	1126
1	2598	2482
1	10126	14561
1	136	134
1	1966	466
1	7412	8690
1	1412	999
1	256	198
1	6193	1618
1	1411	326
1	14	15
1	37	26
1	7340	777
1	1371	756
1	5492	1191
1	18784	18138
0	6	29
0	3	19
1	7156	1760
0	155	167
1	11369	16114
1	2940	2247
1	3722	1555
1	406	1928
0	6	17
0	0	1
1	2241	775
0	11	2
0	6	0
1	3530	210
1	1856	1841
1	277	338
0	20	1
0	189	116
1	4032	5642
0	2592	1550
0	4	5
1	5677	10612
1	11052	2926
0	5	109
1	4654	490
0	30	66
1	5872	8914
1	1046	391

1	11043	20077
1	4787	6335
0	33	35
0	91	190
1	693	936
1	0	5
1	533	531
1	6170	4424
1	6662	3571
1	2250	3704
0	0	0
0	0	0
0	0	0
0	11	11
1	295	2490
0	0	0
0	0	0
0	0	5
1	1128	383
0	0	2
0	0	0
0	0	0
0	0	0
0	0	2
0	10	3
1	2351	3312
0	3	129
1	10917	4746
0	4	106
1	1666	1166
0	15	103
1	7293	535
1	7275	3907
1	772	354
0	2	6
1	3484	935
1	2359	696
1	785	1683
0	5	0
1	8139	13781
0	6	31
0	0	23
0	0	29
1	9	90
0	10	23
0	39	76
0	6	0
0	0	5
0	0	1
0	2	20
1	740	209
0	0	4
0	0	0
0	4	3
0	0	0
0	0	0
0	0	0

0	0	8
1	82	128
1	6341	258
0	10	30
1	2348	1044
1	25032	12567
0	11	113
1	3282	2696
1	8397	6925
0	4	92
0	85	83
0	0	0
0	0	0
0	0	0
0	0	0
0	19	75
0	30	12
0	2	1
0	0	0
1	5101	4436
0	0	6
0	2	0
1	26267	17149
1	198	3143
0	30	290
0	44	171
0	0	0
0	0	0
0	1	1
0	3	9
0	3	22
1	2463	898
1	7603	3750
0	2	28
0	2	9
0	0	0
0	0	0
0	0	1
0	0	3
1	59	305
1	6426	1691
0	20	12
0	0	0
0	0	0
0	0	1
0	15	93
0	0	6
0	7	11
1	5313	617
0	0	3
0	0	0
0	0	0
0	0	2
0	1	0
0	0	0
1	7688	3346
0	6	7

1	6573	3132
1	7104	7494
1	676	1280
0	0	0
0	14	22
0	2	9
0	0	0
0	13	131
1	44	105
1	7124	4355
1	16577	5788
0	8	29
0	7	37
0	2	30
0	3	17
1	2046	1821
1	7952	9432
1	29558	12459
1	1427	4255
1	275	232
1	5977	10576
1	1545	2240
0	5	52
1	58759	62259
1	19809	6968
0	37	93
0	116	62
0	5	6
1	3455	1834
1	6111	7727
1	3890	2955
1	589	395
1	4538	4873
1	4729	6040
1	3260	3069
0	0	5
0	20	106
1	1503	494
0	6	13
1	249	254
1	1124	1266
1	2024	2445
1	249	502
1	1943	989
1	770	675
0	154	199
0	0	12
0	0	3
0	0	2
0	18	59
1	2298	1250
1	523	731
1	1784	2581
0	0	28
1	1937	856
1	4731	3817
1	1934	2160

1	8684	5160
0	89	369
0	9	48
1	33	159
1	561	1315
0	0	4
0	65	28
0	2	1
1	6824	8766
0	2	12
0	2	8
0	4	13
1	5442	1172
1	500	430
1	692	751
0	0	0
1	5194	2414
1	1384	765
1	1172	1085
1	4815	5166
1	2489	122
1	40	310
1	4164	1648
0	9	60
0	11	121
1	1036	727
1	5269	2465
0	31	37
0	2	0
1	955	498
0	3	3
0	57	20
0	0	0
0	0	0
0	0	5
0	15	35
1	8462	2216
0	0	3
0	0	8
1	2682	3307
1	1892	2374
1	1635	1995
1	5551	7553
1	1484	1671
1	940	5364
1	41990	147959
1	16396	6468
0	1	0
0	7	0
0	5	6
0	1	0
0	0	5
1	481	70
1	1945	1469
0	8	22
1	5052	5048
1	5064	7709

0	0	4
0	0	0
0	1	1
0	0	0
0	0	1
0	0	0
1	6655	1744
1	158	692
0	0	2
1	4227	1635
0	0	2
0	12	9
0	0	0
0	0	0
0	0	1
0	0	0
1	4881	1651
1	2315	2926
1	25831	3862
1	42	705
0	1	1
0	5	84
1	1777	2056
1	8758	4491
1	6937	8370
1	6204	5201
0	0	0
1	2773	2735
0	0	0
1	9824	4992
0	0	0
0	0	0
1	12615	14503
1	9186	979
1	2138	3041
1	6189	903
1	3995	2720
0	9	28
1	173	352
1	2853	1113
1	4275	5840
1	1605	1036
1	18846	952
1	6291	4156
1	92	127
1	621	738
0	4	42
0	8	9
1	3257	4260
0	55	249
0	51	115
0	2	2
0	1	18
1	3301	835
1	3732	1219
0	0	0
0	1	0

1	1595	640
0	0	1
1	327	293
1	353	243
0	15	23
0	0	0
0	0	0
0	0	1
1	5550	2419
1	5278	6169
1	4125	3606
0	6	0
0	12	3
1	5887	1346
0	6672	1203
0	427	478
0	2	1
0	19	34
0	14	4
0	0	0
1	5624	2977
0	51	507
0	318	461
1	4515	2613
1	19	151
1	1880	3806
1	2609	2923
1	1888	2468
0	27	34
1	2673	2787
0	105	115
1	1046	728
0	87	143
0	1	4
0	2	1
1	81	74
1	6393	835
0	0	0
1	5299	1269
0	0	12
0	1	9
1	2108	437
0	64	431
0	0	1
0	14	16
0	0	8
1	2323	3221
0	1	14
0	0	7
0	0	0
0	0	0
0	0	3
0	27	133
1	4729	2746
1	8411	6505
1	4043	1275
0	23	33

0	27	14
0	6	22
0	2	10
0	0	0
1	2242	2244
1	13376	6488
1	1145	655
1	13646	12837
1	4500	2417
0	531	134
1	1683	784
1	196	107
1	12149	1614
0	0	0
0	2	3
0	0	2
0	1	8
0	3	54
1	3827	3040
0	1	13
0	35	49
1	6389	1573
1	8170	8509
1	3532	1167
1	7035	7419
1	4571	3221
0	0	0
0	3	15
1	15439	12366
1	894	349
0	0	0
0	1	0
0	3	15
0	0	0
1	2673	756
1	2194	1962
1	11847	9331
1	5924	1485
1	2305	2279
0	3	14
1	5328	4013
0	1	1
1	82	381
0	6	9
1	249	71
1	6079	7835
1	1689	1016
0	15	6
0	1	1
0	5	7
0	14	11
0	2	0
0	0	2
0	0	5
0	0	1
0	2	90
1	2836	2203

1	1087	253
1	3510	1831
1	330	419
1	7329	2067
0	3	2
1	2114	2339
1	7174	1947
1	546	412
1	2290	2250
1	1474	1714
0	0	0
0	2	73
0	156	1142
0	14	154
0	1	2
1	3616	417
1	3935	4557
0	13	39
1	1335	1216
0	35	102
1	176	245
0	0	0
0	0	0
0	211	213
0	0	1
0	12	31
0	0	4
1	14396	9111
1	1561	1278
1	3228	1147
1	10341	4715
0	42	269
1	7300	9319
0	2	2
0	0	0
1	21	213
0	70	33
0	0	1
0	2	0
0	0	0
0	1	0
1	8435	3444
1	10327	17303
0	0	0
1	2187	581
1	916	546
0	0	1
0	5	4
0	1	10
1	4106	358
0	34	40
1	2269	3270
0	35	99
1	930	449
0	34	375
1	4273	4386
0	0	1

0	1	3
0	78	71
1	9511	6978
1	7895	2053
0	3	35
1	4996	4568
1	1995	9692
0	1	7
1	4174	2058
1	7993	7696
0	19	298
0	298	188
0	0	2
0	1	3
1	3425	1597
0	0	0
0	3	10
1	3627	5258
1	772	725
0	7	12
0	1	13
0	17	30
1	3223	3737
1	17678	12907
0	0	0
0	1	2
0	0	1
0	2	3
0	57	626
0	0	5
0	19	23
1	2973	2409
0	16	257
0	12	53
1	2405	2064
0	2	49
0	12	20
0	5	32
0	4	18
0	44	103
1	11940	10559
1	13482	7366
0	1	18
0	0	2
0	1	2
1	4005	452
0	27	157
1	3233	1427
1	12476	11600
1	4218	2515
1	657	544
1	34	199
0	6	66
0	1	6
0	18	36
0	0	0
0	3	5

0	0	0
0	0	0
0	3	2
0	0	0
0	5	0
0	0	2
0	0	0
0	4	7
0	0	4
0	0	2
1	676	102
1	3776	4241
0	12	228
0	11	39
0	0	2
0	7	24
0	0	3
1	18683	12149
1	638	1329
1	725	1183
1	29637	21245
0	39	42
1	9698	5783
1	3915	1457
0	2	8
1	1822	3242
0	21	43
1	4689	7336
0	4	14
1	3350	2933
1	3061	1043
1	6351	11342
1	1328	1160
1	2139	2313
1	7870	8382
1	7191	2177
0	3	18
1	471	778
1	7949	4464
0	4	2
0	64	136
1	1754	414
0	3	75
0	23	73
1	8143	773
1	1192	1376
0	6	12
1	5428	1443
1	664	235
1	8658	2917
1	493	267
1	5756	6372
1	7237	1239
1	3014	2405
0	8	22
1	13062	17554
1	26437	15165

1	9859	4226
1	2985	3401
1	1977	1215
0	3	7
0	0	2
0	1	0
0	3	7
0	0	0
0	0	3
0	0	0
0	1	2
0	1	4
0	3	17
1	2028	1378
0	310	334
1	7338	8817
1	18323	17182
0	25	32
1	5780	1758
1	5533	4384
1	6480	2740
1	16492	14277
1	5537	827
0	4	5
0	2	12
1	2270	3180
1	11104	15201
0	0	6
0	1	1
0	0	3
0	6	10
0	3	3
0	0	0
0	21	67
0	0	2
1	532	454
1	4647	6222
1	649	249
1	836	353
0	56	199
1	12788	12539
0	22	15
1	1464	3516
1	2777	1231
1	1867	2722
1	1016	780
1	1599	618
1	1851	1378
1	2150	3363
0	13	15
0	0	0
0	17	187
1	239	5770
1	1153	25957
0	33	466
1	1033	755
1	5018	1205

0	20	56
1	3232	803
0	1	0
1	29807	44428
1	4141	1846
1	54989	72702
1	9119	8191
1	2312	1330
1	1284	1621
1	3145	2093
0	0	0
0	0	0
0	0	0
0	0	0
0	2	0
0	0	0
0	4	1
0	0	1
0	18	175
0	4	12
0	2	77
0	1	1
0	3	2
0	4	6
1	2176	928
0	5	19
0	0	7
1	1581	1140
1	1286	666
1	1752	1019
1	19685	30653
1	3184	1379
0	0	0
1	11	113
0	0	83
0	2	43
1	17414	2692
1	3366	1419
1	22678	19936
1	1553	1841
1	454	384
1	708	483
1	10129	4285
0	0	0
0	0	0
0	0	0
0	0	0
0	0	2
0	0	0
0	0	0
0	0	30
0	1	4
1	6265	2310
1	5791	1710
0	4	12
0	5	3
0	0	0

0	1	2
1	2313	1173
1	1428	1439
1	724	285
0	0	0
1	10036	11254
1	829	755
0	0	2
0	2	16
0	0	0
0	3	6
1	2269	420
1	484	345
0	1	22
0	0	0
0	0	0
0	0	0
1	3783	5284
0	0	1
0	361	263
0	1	9
0	1	0
1	2089	611
1	112	647
0	1	1
1	2538	2270
1	7150	10374
1	10506	2646
0	93	74
0	4	13
0	4	3
0	0	0
0	4	2
0	67	30
0	0	0
0	0	0
0	0	2
0	0	8
1	1795	2184
0	1	2
0	4	4
1	21887	15631
0	11	26
0	3	16
0	1	5
0	8	23
1	2210	2475
1	4941	2822
0	1	4
0	0	0
0	2	5
0	37	379
1	15630	7585
0	1	3
0	66	79
0	823	136
1	7678	7098

0	25	9
1	2761	227
1	5544	6003
1	10190	10249
0	0	0
0	0	4
1	9473	10452
1	4764	5135
1	2221	2811
1	3862	5606
1	10956	14877
1	6863	1937
1	77	234
1	2876	899
1	7859	15099
0	0	0
0	0	0
0	0	0
1	6684	4679
1	14171	22426
1	449	579
1	1203	860
0	34	56
0	0	0
0	4	30
0	98	61
0	0	12
0	0	14
0	0	3
1	7202	20447
1	6001	7378
0	7	14
0	0	0
0	0	0
0	1	7
1	8473	4419
1	4509	2718
1	3318	5189
1	2221	1312
0	0	15
0	5	21
0	43	183
1	4443	5007
1	19634	19607
1	9903	2774
1	10289	2994
1	3235	3656
1	6254	6597
1	432	357
1	1395	599
0	0	0
0	24	58
1	377	202
1	5525	8460
0	18	192
0	2	0
0	0	2

1	2891	506
1	662	299
0	0	3
0	10	1
0	3	5
0	4	11
1	5131	3544
0	0	20
1	758	7034
1	13707	18088
0	3	6
0	44	30
0	8	86
0	2	12
1	1315	1640
1	99	163
1	1959	786
1	686	765
1	8790	11159
0	2	8
1	5805	5532
0	0	0
0	81	492
1	9696	7905
1	3160	3363
1	7061	5825
1	125008	174706
1	3688	1176
0	0	13
1	1401	1430
1	3256	1948
1	854	1434
1	8584	6643
1	67	1078
1	15923	9395
0	19	218
1	13005	1080
1	8214	4258
0	9	162
1	6273	11168
0	4	23
0	0	4
0	1	2
0	22	283
1	1573	1049
0	225	141
0	2	2
0	82	200
0	5	6
0	0	0
1	927	817
1	894	838
1	515	792
1	17666	17873
1	8	90
0	3	11
1	3125	3753

0	0	3
0	0	0
0	0	0
0	2	19
0	2	9
0	37	22
1	7802	2296
0	2	5
0	1	11
0	0	4
0	0	1
0	36	171
0	0	3
1	3650	2033
0	3	17
0	7	4
0	9	81
0	15	223
0	0	0
0	0	0
0	1	43
0	56	399
1	6621	9358
1	86652	51788
1	15886	9081
0	0	2
0	0	2
0	4	29
1	3774	1053
0	18	169
1	7228	5088
0	1	0
0	123	173
0	51	94
0	36	67
0	518	190
1	2700	619
1	3182	4484
1	2473	3287
1	4383	273
1	1645	1740
0	14	42
0	79	901
1	1617	2221
1	5637	5488
1	2230	739
1	6585	966
1	602	509
0	186	235
1	485	146
1	1812	638
1	6102	4772
0	5	22
0	32	46
0	10	53
1	3325	4744
1	11015	16925

1	11414	13656
1	2152	2691
1	6726	9245
1	3321	5038
0	32	36
0	3	14
1	1540	748
1	7540	3036
1	1466	1809
0	325	375
1	23623	16725
1	226	528
0	1064	204
1	7155	2567
1	7446	8277
0	392	300
0	0	0
1	1715	1228
1	9802	7082
1	11874	12488
1	1957	248
1	1995	2159
1	5643	4383
1	38982	15921
1	2074	2917
1	0	14
0	1	0
1	5078	5269
1	7059	5726
0	60	124
1	2719	2819
1	3895	5899
1	14056	15591
0	55	25
1	3153	2432
0	10	12
1	5316	738
0	0	3
0	0	0
1	0	12
0	0	0
1	15433	3570
1	8017	11418
1	6983	2536
0	0	0
1	14638	939
0	0	0
0	0	0
0	0	0
1	11867	16194
1	4243	1819
1	12870	17655
1	5979	8743
0	492	1090
0	55	64
0	0	0
0	1	0

0	9	26
0	0	0
0	0	0
0	0	5
0	2	0
0	0	9
0	1	0
0	875	132
1	8978	8357
1	10128	7539
0	3	9
1	5153	1265
1	40510	49835
1	1205	1587
1	9515	4776
1	9282	4456
1	7064	3967
1	4638	3971
0	1	0
0	14	20
0	0	6
0	0	0
0	0	3
0	0	4
0	0	0
0	6	93
1	6779	1266
1	5064	6366
1	17187	26060
0	0	0
0	11	20
1	19303	32003
1	6473	7490
1	6127	13022
0	0	0
1	303	4033
1	861	5376
0	4	162
1	3654	1303
1	2427	1247
0	61	1048
1	156	78
0	20	11
1	2027	2624
1	4326	1132
1	2712	2342
0	29	11
1	3531	3115
1	5451	6698
0	0	0
1	18	232
0	0	0
1	4009	1204
0	20	156
1	5964	6291
1	5905	2550
1	154	229

0	1	0
0	1	0
0	6	7
0	0	5
0	0	5
0	0	3
0	4	5
0	32	98
0	3	1
1	2049	557
0	0	4
0	142	129
1	3311	1070
0	1	3
0	11	10
1	4567	1387
1	107	67
1	2570	252
1	123	2117
1	8201	2492
1	1924	1935
0	15	56
0	2	35
1	22	305
1	2108	256
1	10214	7536
0	145	321
1	1717	2135
1	2238	2517
1	8924	2318
1	4799	1127
1	20600	2597
0	0	0
0	24	165
0	0	12
0	15	115
0	0	2
0	0	0
0	0	5
1	3748	2202
0	0	0
0	2	3
0	0	2
0	1	3
0	4	33
1	7355	3357
0	1	0
1	14	31
1	2146	3579
1	215	166
0	0	0
0	0	1
0	0	0
0	5	1
0	0	0
0	0	2
1	106	70

0	14	26
0	0	0
0	0	1
0	0	0
0	0	0
0	1	1
0	0	0
0	0	0
1	146	26
0	0	0
0	1	2
1	566	324
0	2	1
0	2	4
1	270	593
0	20	83
0	1	27
0	0	0
0	0	0
0	0	0
0	12	78
0	1	2
0	0	3
0	0	0
0	10	0
0	0	0
0	6	6
1	8	13
0	1	4
0	3	8
0	6	2
0	0	2
1	320	181
1	21561	14066
0	0	7
1	41	104
0	35	347
1	3641	4764
1	9895	6381
0	133	190
0	0	6
1	223	211
1	8377	10373
0	0	0
0	1	0
0	21	11
0	17	22
0	32	41
0	0	0
0	17	1
0	3	3
0	0	0
0	0	0
0	1	3
0	28	34
0	27	73
0	2	1

0	2	11
0	4	4
0	0	1
0	0	2
1	1713	364
0	0	0
1	1917	282
0	33	48
0	0	0
0	0	1
0	3	24
1	918	934
1	8955	2335
0	0	0
0	3	0
0	0	0
1	4863	925
0	0	1
1	676	330
0	1	8
0	0	0
0	0	0
0	0	0
0	0	0
1	2876	2544
0	0	5
0	0	0
0	0	0
0	0	0
0	0	0
0	1	2
1	4341	4404
1	770	652
1	8742	4208
1	3368	2587
1	19578	23028
0	66	309
0	37	28
0	967	250
0	0	1
0	1	1
1	13632	13655
1	17475	7337
1	9892	1213
1	19765	6221
1	5002	3759
0	0	0
1	8206	6598
1	4680	6842
1	12791	2362
0	0	0
0	0	1
0	0	0
0	5	28
0	0	0
0	0	0
0	0	0

0	1	1
0	3	32
1	8113	3929
1	4613	4537
0	34	32
1	6185	1698
1	3563	4182
1	59	927
0	0	0
0	3	6
1	361	641
0	0	33
1	10246	1901
1	9307	11179
1	4646	405
1	33198	38362
0	10	11
1	2038	926
0	391	180
0	2	11
0	4	13
0	3	15
0	1	2
0	145	1049
0	1	0
0	51	57
1	1013	1178
0	6	127
1	395	570
0	0	0
0	1	15
1	9863	5963
0	3	1
1	1537	1012
1	1380	466
1	9989	2917
0	0	0
1	6158	2832
0	0	0
0	0	0
0	0	0
1	1052	868
0	4	6
1	33	146
1	431	237
0	0	0
0	64	194
1	13867	7502
0	14	25
0	0	0
0	0	0
1	8546	11521
0	8	21
1	3413	2256
0	1	17
0	0	19
1	2822	207

1	3277	4531
1	35154	48518
1	5711	3683
0	9	12
0	5	1
0	0	0
0	0	0
0	0	3
0	0	0
0	0	0
0	0	0
0	0	0
0	2	8
0	0	3
1	71	267
0	16	57
1	350	2922
0	66	98
0	0	0
0	23	12
0	0	0
0	385	400
1	28505	175658
0	1	32
1	54950	49281
1	18089	19393
1	1214	1723
1	2488	1355
0	0	3
0	69	210
0	0	2
0	7	18
1	14356	15802
1	26	196
1	55295	16374
0	0	0
1	2400	1782
0	0	0
1	3570	3566
1	10697	13901
1	3787	2654
0	29	32
1	5172	1707
1	14229	18124
0	0	1
0	0	0
0	0	0
0	0	0
0	0	0
0	0	0
0	0	0
0	0	0
0	0	0
0	10	1
0	0	0
1	13901	11611
0	0	4

1	230	365
0	0	0
0	57	742
0	20	17
1	16073	7933
0	0	0
1	25709	4005
1	2346	779
1	5648	3049
1	368	7064
0	0	2
1	2230	672
0	0	0
1	1538	1600
0	43	41
0	16	70
0	0	0
0	0	0
1	4226	2016
0	1	0
1	2066	1860
0	0	0
1	1583	2628
0	4	12
1	6299	8670
0	0	0
0	0	0
0	0	1
0	2	6
0	6	5
1	343	266
1	6335	9695
1	2750	2232
0	63	80
1	182	160
0	0	0
1	2780	3300
1	641	635
1	1109	143
1	4031	3730
0	9	19
0	3	20
1	338	66
1	5859	6984
1	770	9035
0	5	45
0	0	0
0	66	336
1	1179	1386
0	0	5
0	0	0
0	0	3
0	4	15
0	155	378
0	7	93
0	0	29
0	0	0

0	7	38
0	0	2
0	44	39
0	1	23
0	0	0
1	549	124
0	1	8
0	1	4
1	17	42
1	2448	936
0	6	30
0	0	0
0	0	2
0	14	118
0	2	1
0	0	0
1	52	87
0	0	0
0	0	0
0	0	0
1	914	682
0	0	0
0	5	17
0	0	0
0	1	1
1	243	4304
0	16	98
0	0	0
0	0	3
1	610	597
0	0	0
0	3	51
0	0	0
0	188	143
1	770	996
0	0	2
0	0	0
1	1316	241
0	0	2
1	136	1244
1	11102	2379
0	0	0
0	0	0
1	26044	43471
0	3	11
0	62	107
0	87	125
1	3450	363
0	0	1
1	7	105
1	2653	321
0	0	0
0	1	13
1	12413	2132
0	65	20
0	28	82
0	13	60

0	1	10
0	6	0
0	0	7
0	0	2
0	4	27
0	12	13
0	4	62
0	0	0
0	4	36
0	11	7
0	1	46
0	0	1
0	0	4
0	2	50
0	1	0
0	1	2
0	1	3
0	1	24
1	13	179
0	2	12
0	0	0
1	4533	2643
0	1	24
1	1781	2332
0	12	108
0	1	36
0	4	33
0	4	4
0	178	80
0	8	21
0	6	27
1	2725	4683
0	19	18
1	2987	3752
0	4	21
0	0	0
0	1	34
1	13457	13921
0	6	2
0	9	21
0	9	40
0	0	0
0	0	3
0	0	0
0	0	0
0	7	8
1	14437	9387
1	1376	1088
0	3	4
0	0	0
0	0	0
1	14554	2383
0	0	2
0	0	6
0	2	27
1	1269	585
1	6502	7033

1	46	246
1	14084	5089
1	2230	2519
0	0	0
1	2594	538
0	0	0
1	3183	3361
0	0	2
0	18	72
0	208	26
1	1033	689
1	7778	10697
0	10	63
1	10652	3031
0	0	0
0	102	74
0	129	143
0	0	1
0	0	1
0	3	9
0	11	11
1	2163	1285
1	3709	5189
0	164	166
1	49	290
0	1	1
1	92	975
0	0	34
0	312	512
0	2	5
1	1978	642
0	7	30
0	0	1
0	20	10
0	57	37
0	73	268
0	0	0
0	0	0
1	4975	1121
0	0	0
0	159	98
1	4176	3114
0	0	2
0	0	9
0	0	0
1	1183	1506
0	2	2
0	0	6
0	0	4
0	0	7
0	0	11
0	460	117
1	1224	647
0	9	59
1	2112	2541
1	94038	80895
1	6273	7763

1	40	230
0	0	0
0	70	522
1	288	736
0	6	8
0	7	47
0	67	18
0	2	4
0	1	6
0	2	7
0	0	0
0	10	44
0	28	103
0	0	1
0	93	284
0	11	6
1	2194	1026
0	22	32
0	258	250
1	7239	1080
0	0	0
0	3	9
1	637	547
1	111	331
1	3556	2747
0	19	19
1	5	236
1	5509	4179
0	5711	95
0	3	0
0	0	0
0	0	0
0	0	0
0	0	0
0	0	0
0	0	0
1	12712	6530
0	0	0
0	0	5
1	951	460
0	1	1
0	4	8
0	2	7
0	0	0
0	3	10
0	3	43
1	2401	1861
1	571	967
0	10	20
0	25	37
1	165	629
0	254	396
1	7199	2046
1	8347	4798
1	105	522
1	1267	749
1	122	3012

1	1155	678
0	9	81
0	35	68
1	2	120
0	0	0
1	372	236
0	6	10
1	21833	13011
1	2768	3558
1	3148	1628
0	1	0
1	18586	11092
1	1557	1541
0	39	161
1	18460	16249
0	6	15
1	2352	3035
0	0	0
0	4	2
0	3	4
1	13511	15478
0	0	1
0	0	2
0	74	42
0	8	65
0	0	1
0	0	2
0	1	6
0	0	0
1	967	1692
0	0	9
0	1	3
1	1079	1986
0	0	0
1	1216	871
0	1	3
0	6	30
1	7964	9499
0	5	14
0	2	7
1	2751	222
1	675	229
1	1	4
1	11363	4196
0	7	8
1	314	63
0	6	0
0	5	4
0	3	2
0	2	26
0	0	0
0	6	46
1	278	315
0	154	95
0	0	0
0	0	0
0	2	0

0	17	34
1	20	42
0	2	0
1	6763	10462
1	6965	4543
0	10	101
0	4	7
0	3	6
0	0	0
0	0	0
0	10	7
1	22337	10677
0	36	28
0	1	1
0	7	25
1	376	1160
1	5540	7366
0	0	0
1	327	223
0	8	154
1	23358	35533
1	5107	7472
0	5	2
0	1	8
1	387	418
0	43	76
0	0	2
0	0	3
1	778	1252
1	2257	2889
0	2	38
0	0	0
0	3	5
0	0	2
0	5	84
0	0	8
0	0	0
0	0	0
0	0	0
1	1046	499
0	1	3
0	0	5
0	6	22
1	1325	1242
0	6	7
0	18	69
0	2	28
0	0	3
0	2	17
1	18	249
0	4	61
1	3089	1546
0	8	70
0	774	864
0	1	0
0	2	2
0	0	3

0	31	142
0	0	0
1	2905	1892
0	0	0
0	1	15
0	1	1
0	0	0
1	342	3002
0	3	27
0	0	0
0	13	8
1	2719	350
1	7621	8749
1	4936	6703
0	51	58
0	0	0
0	5	1
0	0	9
0	0	0
1	458	638
0	57	205
0	0	12
0	2	12
1	1351	1201
1	15475	6874
0	0	2
1	3301	2564
1	16980	2855
0	2	58
0	0	0
0	3	4
0	0	3
1	1320	359
1	702	529
0	0	2
0	15	62
0	6	12
0	4	3
0	14	11
0	6	14
1	1125	1532
1	40	61
0	4	4
0	0	0
1	8488	5434
0	1	7
0	0	0
0	0	0
0	3	5
0	21	14
1	1422	1148
1	7027	2311
0	15	16
1	18855	7937
0	1	3
0	0	0
0	2	0

0	0	0
0	0	0
0	1	0
0	4	3
1	14	192
1	2187	2752
1	304	552
1	1303	1799
1	18195	16741
1	1524	1150
0	19	74
1	97	1412
0	5	54
1	15626	15222
0	0	0
0	0	0
0	0	0
0	0	0
1	5041	4203
1	947	931
0	15	318
0	0	7
0	0	0
0	1	8
0	2	9
0	3	1
0	0	0
0	0	0
0	50	27
0	0	0
0	0	2
0	0	0
0	0	0
0	6	114
1	3089	2106
0	0	1
0	3	4
0	0	0
0	8	42
0	0	0
0	0	6
0	0	0
1	46	39
0	1	101
1	60	78
0	0	0
0	1	0
0	0	0
0	0	2
0	0	0
0	0	0
0	0	2
0	0	2
0	0	0
0	0	0
1	4039	2462
1	2127	1956

0	0	1
0	0	11
0	0	1
1	440	513
1	524	944
1	450	782
0	0	0
0	8	11
0	0	0
0	0	4
1	1175	1191
0	0	0
0	159	82
0	8	23
0	0	1
0	0	4
0	0	4
0	4	14
1	2128	1040
1	54	75
0	0	0
0	0	0
0	0	0
1	3148	943
0	0	0
0	0	0
1	901	1133
0	0	0
1	630	229
0	0	0
0	0	10
0	19	58
1	1421	1148
0	0	0
1	95	395
0	1	38
0	0	0
0	0	0
0	2	32
1	2472	1825
1	44360	60625
0	39	28
1	10617	5276
0	260	502
1	6236	7618
1	155	391
1	480	1227
1	362	687
1	9941	8268
0	10	14
0	0	0
0	0	1
0	2	14
1	1398	1059
0	61	40
0	4	19
1	2385	2947

1	819	439
0	0	8
1	26268	45093
0	75	558
0	6	45
0	0	0
1	2616	4017
0	0	0
1	1792	1427
1	446	427
0	1	4
0	16	9
0	0	7
1	2081	2771
1	2917	112
0	0	0
0	1	0
0	121	85
0	1	1
0	0	0
1	4051	3749
1	365	450
1	2623	2357
0	0	0
0	0	1
0	0	0
1	624	341
0	2	59
0	9	24
1	1525	458
1	2423	1783
0	2	2
0	0	0
0	2	0
0	2	0
0	64	66
1	9592	4552
0	0	19
0	15	128
1	415	442
0	63	53
0	0	0
0	2	2
1	655	775
0	12	9
0	0	0
0	3	17
0	1	6
0	12	18
1	12874	23492
0	11	5
0	0	0
0	0	0
0	286	298
0	102	89
0	0	0
0	0	0

0	5	6
1	1418	595
0	2	9
1	1831	1245
1	10	8
1	0	5
1	2	4
1	271	653
0	0	0
0	0	0
1	819	249
0	24	60
0	4	20
0	1	0
0	2	2
1	2804	1905
0	0	7
0	2	1
0	0	0
1	5375	1465
0	0	0
0	6	6
0	32	16
0	0	5
1	1501	1055
0	1	20
1	9006	9293
0	2	0
0	41	36
1	1065	1212
0	0	0
0	0	0
0	0	0
0	2	16
0	0	22
0	0	8
0	0	0
0	0	0
0	0	3
1	8840	6627
0	0	0
0	0	0
0	5	7
0	17	2
0	0	1
0	0	0
0	166	150
1	1403	1686
0	4	2
0	0	3
0	4	60
1	3638	1161
0	2	13
0	3026	669
0	1	15
0	53	134
1	2838	2653

0	0	0
0	0	0
0	1	0
0	0	0
0	0	9
0	66	44
0	0	1
0	22	22
0	8	61
1	6564	10135
1	7393	306
0	0	4
1	9792	3065
1	8410	6216
1	5738	2071
0	16	157
0	3	2
1	6265	2560
1	4757	4359
1	869	1031
0	1	16
1	365	389
0	0	27
1	20323	15700
1	4799	2704
0	0	0
0	1	7
0	0	0
0	0	0
0	0	5
1	19574	11873
0	7	52
0	3	24
0	0	0
0	0	0
1	1709	824
0	0	0
1	624	130
0	19	143
0	0	0
0	0	0
1	6477	4979
1	4903	5039
0	1	0
1	7071	2574
1	3683	1277
0	0	0
0	51	50
0	0	0
0	0	0
0	125	174
0	3	74
0	81	31
0	3	27
0	3	24
0	133	116
0	45	39

0	0	0
0	7	37
1	393	414
1	2692	3820
0	13	5
1	638	750
0	0	1
1	3145	5055
0	132	176
0	0	4
0	2	4
0	5	1
1	2810	3992
1	10374	1081
0	65	51
0	9	24
0	0	0
0	26	13
1	3480	3015
0	0	3
1	216	278
1	677	1310
0	0	0
0	0	0
0	2	1
1	3583	1715
0	81	48
0	0	0
1	31366	14838
0	1	5
0	32	34
1	11477	6896
0	14	27
0	10	48
0	0	0
0	0	0
1	6130	11624
0	0	2
1	5521	3532
0	43	91
1	9233	12362
0	0	43
0	0	1
0	49	38
0	31	19
1	9598	3565
1	342	773
0	8	83
0	3	14
1	5502	2946
1	1350	1833
0	3	5
0	21	30
0	0	5
0	0	0
0	7	13
0	0	0

0	0	0
0	0	1
1	6441	929
0	1	2
0	0	0
1	8317	11275
1	4	58
0	0	0
0	4	28
1	1392	1179
1	7490	3050
1	27619	21252
1	8071	1263
0	223	232
0	0	0
0	53	45
1	2375	2455
0	0	1
0	0	0
0	640	332
0	22	17
1	37	430
0	334	246
1	153	125
0	13	5
0	23	14
0	0	0
1	3429	1444
0	0	0
0	0	0
0	0	0
0	0	0
0	10	3
0	5	62
0	0	2
0	2	10
0	22	24
1	461	383
1	961	203
1	345	332
0	13	25
0	0	1
0	139	59
1	380	6679
0	0	3
0	1	1
0	0	2
1	1976	1467
0	7	24
0	0	3
0	2	5
1	246	523
1	2278	792
0	74	152
0	0	0
0	0	0
1	433	362

0	8	48
0	0	8
0	4	15
1	10507	8888
1	5909	10272
1	7524	11008
1	61	902
1	5784	2617
0	0	0
1	2028	1311
1	258	554
0	0	0
1	17090	32507
0	0	0
0	4	2
0	0	1
0	0	6
0	7	1
0	16	7
1	21146	6852
0	1	6
1	18937	3064
1	4568	1503
0	4	31
1	21326	6346
0	5	20
1	521	213
1	829	1108
1	2971	642
1	8912	3373
0	3	2
1	686	1683
1	11308	6359
1	6279	4854
0	0	3
0	2	5
0	1	2
1	7826	1814
1	269	1730
0	0	4
1	47	924
0	84	129
1	12597	9402
1	2245	1478
1	724	1159
0	1	16
1	2	0
0	0	8
0	44	26
0	0	2
1	685	302
1	5755	4775
0	0	2
0	1	1
0	0	0
0	0	0
0	0	0

0	0	0
0	0	0
0	0	0
0	0	0
0	0	1
0	0	0
0	0	0
0	1	0
0	0	0
0	0	0
0	0	0
0	0	3
0	49	25
0	0	1
0	0	0
0	2	52
0	4	5
0	2	2
0	3	1
1	183	295
0	0	2
0	1	4
0	0	0
0	0	1
0	0	10
0	0	0
0	0	0
0	0	1
0	2	54
0	0	12
0	0	1
0	1	8
0	41	60
0	0	1
0	0	1
0	8	96
0	11	18
1	12176	6514
0	1	3
0	1	0
0	0	3
0	0	0
0	1	2
0	0	0
0	0	7
0	0	3
0	0	0
0	0	3
0	0	6
0	0	8
0	0	0
0	0	0
0	0	3
0	0	6
0	1	2
0	0	9
0	1	4

0	0	4
1	543	8199
0	0	3
0	4	20
0	3	1
0	4	3
0	11	56
0	0	0
0	2	1
0	0	2
1	384	88
1	3831	1964
1	1858	367
0	0	0
0	0	2
0	8	20
0	12	9
0	24	33
0	1	16
0	57	74
1	6916	3403
0	2	32
0	1	0
0	1	34
0	0	1
0	0	2
0	1	9
0	0	0
0	0	2
1	13674	18981
0	16	22
1	5925	8764
1	3760	48095
0	66	105
1	22880	3988
0	0	0
0	0	4
1	484	359
0	1	5
0	0	10
0	2	2
0	99	544
0	0	0
0	0	0
0	0	0
0	0	0
0	0	0
0	0	0
0	0	0
0	8	5
0	4	1
0	0	1
0	0	0
0	0	0
0	0	0
0	0	0
0	0	1

0	0	0
0	0	0
0	0	0
0	0	0
1	429	807
0	0	1
0	0	0
0	0	32
0	0	0
0	4	1
0	10	89
0	17	47
0	6	3
1	2468	808
1	459	433
0	53	76
0	0	2
0	6	2
0	1	2
0	29	512
1	128	80
0	0	0
0	461	58
0	0	0
0	13	12
0	0	0
0	0	3
0	3	23
1	261	342
0	4	48
0	0	0
0	0	14
1	985	1094
0	1	3
0	1	5
0	0	0
0	23	324
0	0	1
0	1	0
0	0	2
0	2	31
0	0	0
0	0	1
0	0	6
0	7	9
1	263	161
0	0	3
0	0	0
0	1	11
0	2	15
0	0	0
0	0	2
0	1	2
0	0	0
0	6	24
0	1	11
0	1	3

0	15	15
0	0	5
1	666	766
1	8319	1612
1	8039	2623
0	0	0
1	38	30
1	0	2
0	0	0
1	2392	1937
0	0	0
1	676	792
0	0	1
0	0	0
1	5039	2229
1	1060	1669
0	0	1
0	0	0
0	0	0
0	0	0
1	2435	623
1	290	524
0	46	49
0	0	0
0	0	0
1	1	3
0	3	7
0	0	0
1	839	766
1	32907	45184
0	0	0
0	0	0
0	0	0
0	0	3
1	12133	11128
1	3828	4237
1	15939	3192
0	2	17
1	3145	559
1	3185	4389
1	4601	5781
0	131	126
0	0	4
0	26	92
1	12163	3996
1	1663	520
1	1659	2230
1	6340	289
0	21	30
0	4	1
1	80	104
0	2	2
1	4	2
0	3	20
0	5	34
1	823	997
0	28	35

0	0	0
0	0	0
0	0	0
1	671	955
0	0	0
1	137105	216169
0	2	1
0	1	4
1	6590	2915
0	2	9
0	4	16
1	793	1009
0	4	23
0	74	81
1	105989	152486
1	7620	3353
1	4024	2781
0	8	14
0	0	0
1	10464	2093
0	0	0
0	3	15
1	4856	6537
0	0	0
0	870	189
1	1723	2259
1	4570	3640
0	0	0
0	528	155
1	227	129
1	9107	13659
0	0	0
1	6	114
1	3381	4987
0	2	79
1	650	882
0	1	0
0	49	49
1	10	78
0	3	11
0	0	0
1	3424	5039
1	23292	42727
1	18154	10946
1	0	4
0	0	0
0	2	5
0	0	0
1	1	12
1	0	0
1	1	3
1	0	2
1	0	5
1	0	0
1	0	7
1	0	0
1	0	0

1	0	0
1	0	0
1	0	0
1	0	0
1	0	0
1	0	0
1	0	0
1	0	3
1	1	3
1	0	2
1	2	11
1	0	0
1	0	0
1	0	0
1	0	0
1	1	1
1	0	0
1	0	0
1	1	1
1	5	14
0	0	7
0	1	6
0	1	5
1	1	1
0	1	1
1	2	10
1	0	3
0	0	2
0	0	10
0	0	1
0	0	1
0	0	2
1	0	0
1	0	5
1	1	2
0	0	1
1	2	58
0	2	1
0	1	1
1	1	13
0	0	5
0	1	5
0	6	12
0	0	0
1	1	9
1	1	24
1	0	0
1	0	3
1	0	0
1	0	0
0	1	3
0	1	1
1	0	14
1	1	2
1	0	6
1	3	9
1	1	17

1	0	5
1	1	4
1	2	4
1	1	12
0	0	0
0	1	3
0	1	1
0	1	1
0	0	0
0	1	3
0	0	6
0	0	3
1	2	6
0	0	0
1	0	1
1	0	0
1	0	0
1	1	0
1	1	0
1	2	1
1	0	0
0	2	1
1	0	0
1	53	133
0	0	1
1	0	0
1	0	0
1	1	1
0	0	0
0	0	2
1	0	0
0	0	0
0	1	0
1	0	1
0	0	1
1	4	33
1	0	1
0	0	1
0	1	1
0	0	3
0	0	0
0	0	1
1	0	6
1	1	8
1	0	10
0	0	3
0	0	3
0	1	3
1	0	5
0	0	3
0	0	2
1	24821	9723
0	0	1
1	4538	5807
0	2	5
1	160	1730
0	0	0

0	0	0
1	1938	1297
0	6	0
0	0	0
0	0	13
0	0	2
1	13	372
0	4	15
0	0	2
0	11	6
0	0	0
1	1879	1310
1	1184	613
1	6790	2760
0	16	110
1	2377	2668
1	11183	1996
0	6	24
0	0	0
0	0	0
0	0	0
1	1220	184
1	11865	4577
1	228	258
0	0	0
0	12	38
1	2087	17704
0	0	0
0	4	10
0	0	0
0	39	177
1	119	527
0	0	1
0	0	1
0	2	2
1	593	561
0	19	9
1	441	274
0	262	60
1	225	85
1	2	7
1	0	1
1	12	16
1	20	341
0	0	10
0	1	25
0	1	4
0	1	9
0	1	24
1	4817	8087
0	0	3
0	0	0
1	524	770
1	91	436
0	2	5
0	12	1
0	0	0

0	0	0
0	0	0
0	0	0
0	0	0
0	0	1
0	0	0
1	484	596
0	0	0
0	5	1
0	0	0
0	0	0
0	0	0
0	0	1
0	0	0
0	0	3
1	458	340
0	1	23
0	0	0
1	783	1308
1	139	54
0	0	0
0	0	8
0	4	3
1	1353	836
0	4	34
0	0	0
0	5	78
1	1852	454
0	0	0
0	7	1
0	0	0
1	1424	1535
0	0	2
0	0	3
0	2	1
0	0	0
0	2	9
0	0	0
0	8	9
0	4	8
0	0	14
0	1	1
0	0	2
1	177	247
1	1556	20838
0	2	6
1	928	706
0	3	10
0	25	14
1	8	150
0	2	2
0	0	1
1	177	3419
0	0	0
0	0	0
1	236	168
0	0	16

1	170	155
0	5	28
0	0	0
0	0	0
0	0	4
0	0	0
1	311	392
0	1	0
0	0	0
0	0	0
1	465	344
0	0	1
0	0	1
0	1	2
1	145	92
0	0	0
1	3120	1782
0	0	0
0	4	10
1	174	223
0	2	3
0	0	0
0	0	0
0	2	0
1	651	790
1	185	52
0	0	0
0	1	12
0	0	0
1	284	843
0	12	6
0	3	4
0	0	10
0	2	0
0	0	0
0	0	0
0	0	12
0	7	13
0	0	0
0	0	0
0	0	1
0	0	0
0	1	0
0	0	0
0	0	1
0	10	2
0	0	0
0	0	0
0	0	3
0	10	5
0	0	0
0	2	0
0	0	0
0	0	0
1	323	59
0	2	10
0	0	0

0	1	0
0	0	2
0	0	2
0	0	0
0	0	0
0	0	0
0	0	0
0	0	1
0	2	2
0	2	8
1	23	515
0	0	3
0	2	9
1	260	313
0	0	1
0	0	0
0	0	0
0	0	0
0	0	0
0	25	28
0	0	0
0	0	1
0	0	0
0	0	0
0	0	0
1	48	46
0	0	0
0	24	5
0	0	0
0	3	1
0	3	13
0	0	0
0	2	2
0	4	2
0	0	0
0	3	12
1	53	66
1	1821	1601
0	43	10
0	0	0
0	0	0
0	2	7
1	10181	3650
0	0	2
0	0	0
0	39	42
0	270	501
1	3903	1305
1	739	1812
0	60	90
0	2	3
0	6	33
0	0	1
0	55	213
0	1	2
0	31	16
0	9	22

0	414	104
0	23	21
0	8	8
0	0	11
0	0	10
0	64	253
0	0	0
0	10	1
1	142	2208
0	10	3
1	7655	7597
0	5	6
1	781	1282
0	50	458
1	616	7405
0	239	1802
0	2	7
0	45	203
1	394	7799
0	32	61
0	6	6
0	14	59
0	0	1
0	226	2269
0	3	32
0	3	2
0	475	354
0	0	13
1	10719	5178
1	321	5014
0	36	381
0	21	17
0	39	62
0	35	50
0	4	4
1	26984	17154
0	2	3
1	576	2027
1	530	882
0	471	1012
1	1169	1427
1	50184	56802
1	9991	4095
0	8	207
0	1	23
1	10388	4932
1	16254	10136
1	14230	4148
1	268	283
0	25	28
0	28	512
0	7	11
0	481	109
1	14089	20693
0	50	99
1	1265	1348
0	3	1

0	2	0
0	0	0
0	0	3
0	0	1
0	2	15
0	0	15
0	2	1
0	0	3
0	7	5
0	0	0
0	0	0
1	497	397
0	0	0
0	0	0
0	0	5
0	0	0
0	0	0
0	33	12
0	7	2
0	1	1
0	0	0
1	10494	5003
0	2	22
0	0	1
0	1	1
0	1	3
0	0	3
1	2939	4918
0	0	11
0	1	1
0	0	0
0	1	2
0	2	1
0	0	0
0	0	0
0	0	0
0	0	0
0	2	0
0	0	1
0	0	0
0	0	0
0	0	0
0	0	0
0	0	0
0	0	0
0	0	0
0	0	0
0	0	0
0	0	0
0	0	0
0	0	1
1	0	0
0	0	1
0	2	6
0	1	0
0	0	0
1	532	432

0	0	3
1	0	0
0	0	1
0	0	0
0	0	0
1	82	71
1	300	371
0	0	1
0	16	3
1	89	43
1	2490	627
0	0	0
1	0	0
1	4	4
1	0	0
1	75	20
1	516	460
1	0	2
1	0	0
0	0	0
1	0	0
0	0	0
1	0	0
1	0	0
1	60	90
1	2	3
0	0	0
1	0	0
0	0	1
1	2	9
0	0	0
0	0	0
1	0	0
1	0	0
0	0	1
1	0	0
1	0	9
1	0	0
1	0	0
1	0	0
0	0	0
0	0	1
1	0	2
0	0	0
1	0	0
1	0	1
1	0	0
1	0	4
1	2	35
1	0	0
1	0	0
1	0	4
1	0	0
0	0	0
1	0	8
0	0	0
0	0	2

0	0	0
1	0	2
1	0	1
1	0	1
1	0	0
1	0	3
0	0	0
1	0	6
1	6	16
1	0	1
1	0	0
1	0	0
1	0	0
0	0	0
0	0	0
1	0	1
1	0	0
0	0	0
1	2	4
1	0	0
0	0	0
0	0	0
1	0	0
1	8783	9572
1	2407	2885
0	5	7
1	5550	4789
0	0	9
0	0	2
1	8129	3453
1	26352	17027
1	28487	26258
1	17964	6504
1	1802	1224
1	14168	21990
1	5313	4936
1	9579	1422
1	2271	1952
1	11446	9225
0	39	609
1	14546	5678
1	3455	2927
1	1558	2315
1	245	333
1	640	865
1	76047	113176
1	3395	1716
1	5832	4499
1	0	3
1	9853	3734
0	5	12
1	4660	3898
0	29	11
0	0	0
1	10400	20134
1	4025	3153
1	8709	2660

0	46	141
1	6093	1783
0	138	734
0	18	34
1	5310	4823
1	16683	14381
1	7775	12416
1	2969	1132
1	7651	1388
1	351	507
0	143	200
1	39882	54857
1	3023	2203
1	4974	3686
1	3512	137
0	250	274
1	2892	4267
0	5	44
1	6904	2972
0	1	11
0	1832	1518
1	1273	857
1	9056	3511
1	2302	2381
1	10543	16125
1	52370	93520
1	46705	49635
0	29	12
1	51841	69603
1	295	1068
0	1	5
1	12540	18868
1	1409	15538
1	3665	2852
1	5614	6889
0	2	18
0	0	2
0	0	0
0	0	10
1	1854	3358
0	3	22
1	23	687
1	12169	17499
1	41598	1185
0	0	0
1	976	239
0	0	0
1	8183	5676
0	48	11
1	16132	12095
1	1838	420
0	5	42
0	7	9
1	36555	18669
1	6113	8025
1	15768	4719
1	5207	6584

1	294	3083
0	12	26
1	6332	6962
0	0	0
1	15579	17269
0	2	3
0	0	0
0	0	1
0	0	0
1	4743	6129
0	0	0
0	0	0
0	15	26
0	2	5
1	9088	10166
0	32	119
0	0	0
0	0	0
0	0	1
1	27339	33384
0	0	0
0	0	0
1	865	1597
1	242	196
1	13616	12057
0	3	3
0	121	157
1	1068	4390
0	6	7
0	0	1
0	21	25
0	0	0
0	1	1
0	7	12
1	7203	5865
0	5	10
1	16288	5393
0	51	247
1	1460	529
0	46	31
1	1419	6012
1	355	239
1	17175	30474
0	24	157
0	2	51
1	11853	12924
1	5252	960
1	529	474
0	12	70
0	1	0
0	0	0
0	0	0
0	2	17
0	9	52
0	9	41
1	4219	958
0	0	0

0	53	89
0	0	12
1	10072	1038
0	14	143
1	21293	20370
1	11218	8781
1	707	342
0	0	4
1	1361	11440
1	23123	9210
0	31	15
1	26993	14134
1	2423	401
0	6	22
[truncated: 316,272 more chars]
